# Accessing elusive *σ*-type cyclopropenium cation equivalents through redox gold catalysis

**DOI:** 10.1038/s41557-024-01535-8

**Published:** 2024-05-23

**Authors:** Xiangdong Li, Matthew D. Wodrich, Jérôme Waser

**Affiliations:** 1 Laboratory of Catalysis and Organic Synthesis, Institute of Chemical Sciences and Engineering, Ecole Polytechnique Fédérale de Lausanne, Lausanne, Switzerland; 2 Laboratory for Computational Molecular Design, Institute of Chemical Sciences and Engineering, Ecole Polytechnique Fédérale de Lausanne, Lausanne, Switzerland

**Keywords:** Synthetic chemistry methodology, Homogeneous catalysis, Synthetic chemistry methodology

## Abstract

Cyclopropenes are the smallest unsaturated carbocycles. Removing one substituent from cyclopropenes leads to cyclopropenium cations (C_3_^+^ systems, CPCs). Stable aromatic *π*-type CPCs were discovered by Breslow in 1957 by removing a substituent on the aliphatic position. In contrast, *σ*-type CPCs—formally accessed by removing one substituent on the alkene—are unstable and relatively unexplored. Here we introduce electrophilic cyclopropenyl-gold(III) species as equivalents of *σ*-type CPCs, which can then react with terminal alkynes and vinylboronic acids. With catalyst loadings as low as 2 mol%, the synthesis of highly functionalized alkynyl- or alkenyl-cyclopropenes proceeded under mild conditions. A class of hypervalent iodine reagents—the cyclopropenyl benziodoxoles (CpBXs)—enabled the direct oxidation of gold(I) to gold(III) with concomitant transfer of a cyclopropenyl group. This protocol was general, tolerant to numerous functional groups and could be used for the late-stage modification of complex natural products, bioactive molecules and pharmaceuticals.

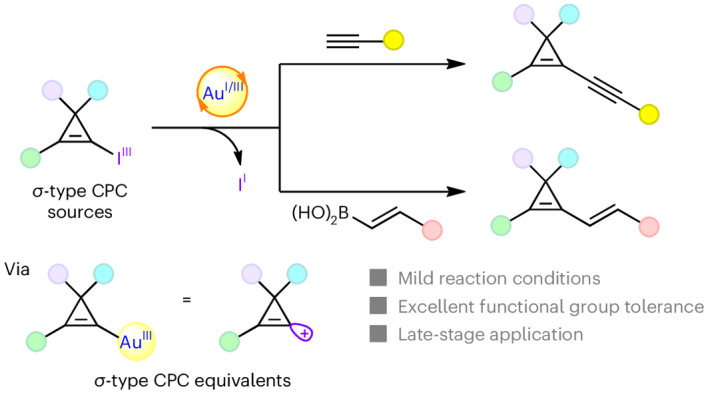

## Abstract (web summary)

The *σ*-type cyclopropenium cations (CPCs) are unstable species and currently underdeveloped. Now, an iodine(III)-based cyclopropenyl transfer reagent has been developed, which can generate electrophilic cyclopropenyl-gold(III) species as equivalents of *σ*-type CPCs. The synthetic potential has been demonstrated by the transfer of *σ*-type CPCs to terminal alkynes and vinylboronic acids.

## Main

The search for reactive functional groups and synthons has long been one of the most productive wellsprings of discovery in chemistry, enabling the design and development of novel transformations^1^ and opening opportunities for drug discovery^2^. As recent striking examples, Suero and colleagues unveiled the dual radical and carbene character of carbyne equivalents generated in situ from a hypervalent iodine reagent bearing a diazo ester^3^, and Garg and colleagues demonstrated that 1,2,3-cyclohexatriene is a powerful and versatile reagent in synthetic chemistry^4^. The search for synthetic equivalents for not-yet-existing synthons thus continues to drive progress in synthetic chemistry by enabling unprecedented bond disconnections.

**Fig. 1:**
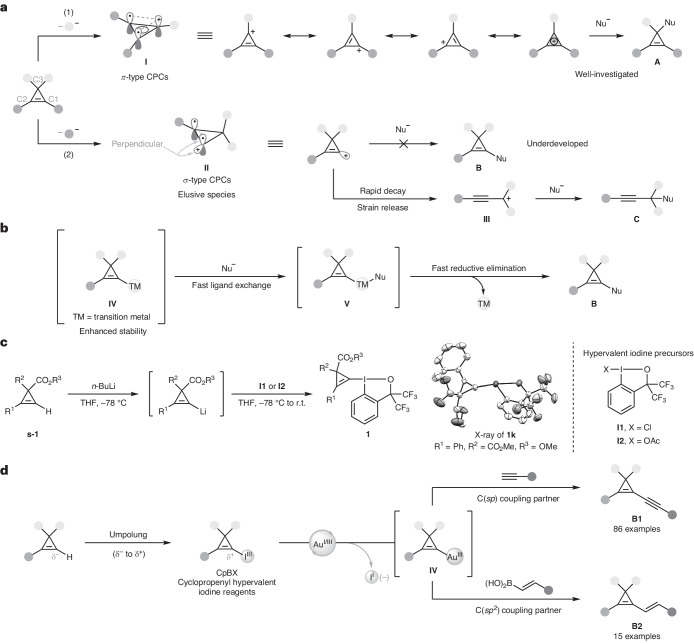
Structure of *π*-type and *σ*-type CPCs and our design. **a**, Bonding analysis and reactivity of *π*-type and *σ*-type CPCs **I** and **II**. **b**, Design of transition metal-based *σ*-type CPC equivalents. **c**, Synthesis of CpBXs via a cyclopropenyl lithium intermediate. **d**, Development of iodine(III)-based electrophilic *σ*-type CPC precursors by an umpolung strategy and *σ*-type CPC transfer reactions to terminal alkynes and vinylboronic acids via redox gold catalysis. Nu, nucleophile.

**Fig. 2:**
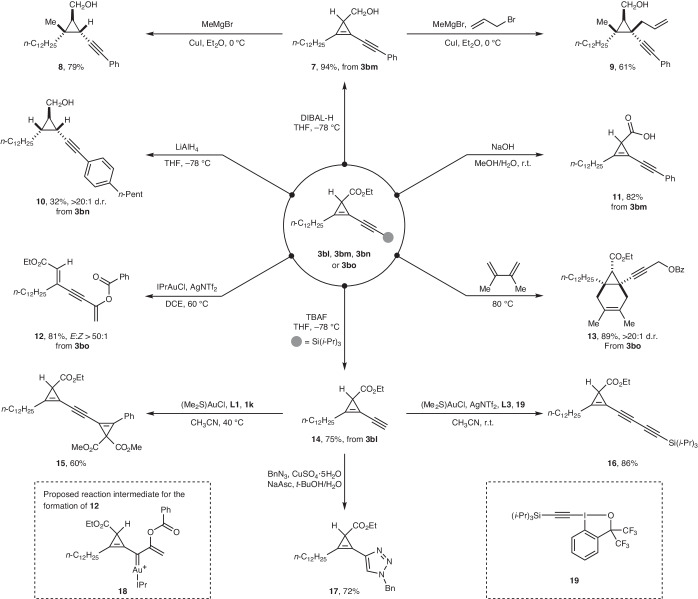
Synthetic modifications of the alkynylcyclopropenes. The obtained alkynylcyclopropenes can be used as precursors for accessing different types of functionalized cyclopropenes, cyclopropanes or ring-opening products. Reduction of alkynyl–cyclopropene **3bm** with DIBAL-H gave cyclopropene **7**, which was converted into alkynyl cyclopropane carbinol **8** and **9** by copper-catalysed carbomagnesiation quenched by methanol and allyl bromide, respectively. Reduction of **3bn** by LiAlH_4_ afforded cyclopropane **10**. Saponification of **3bm** using sodium hydroxide afforded cyclopropene **11**. Gold(I)-catalysed ring-opening of **3bo** via gold carbene **18** furnished **12**. Diels–Alder reaction of **3bo** with 2,3-dimethylbutadiene produced fused bicycle **13**. Desilylation of **3bl** using TBAF led to the formation of **14**, which could be converted into cyclopropenes **15** and **16** by gold-catalysed cross-coupling with **1k** and **19**, respectively. Copper(I)-catalysed alkyne–azide cycloaddition of **14** and benzyl azide gave triazole **17**. NaAsc, (+)-sodium l-ascorbate; IPr, 2,6-bis(diisopropylphenyl)imidazole-2-ylidene; DCE, 1,2-dichloroethane; Tf, trifluoromethanesulfonyl. Supplementary Section 7 provides all the experimental details.

Cyclopropenes, the smallest cyclic alkenes, possess substantial strain energy (54.6 kcal mol^−1^)^5^, which leads to a unique reactivity in ring-opening transformations^6–8^ and C=C bond functionalization^9–11^. The *π*-type cyclopropenium cations (CPCs, **I**)^12,13^, a C_3_^+^ system generated by removing one substituent from the aliphatic C1 site of cyclopropenes, possess extraordinary stability (Fig. 1a, (1)) due to the aromatic character of this system^14^. Neutral cyclopropene precursors are easily ionized, because the aromaticity helps to offset the cost of generating the positive charge^15,16^. Since their discovery, *π*-type CPCs **I** have led to important advances in aromaticity theory^14^, catalysis^17^ and material science^18^. The *π*-type CPCs **I** also provide a good platform for the synthesis of functionalized cyclopropenes of type **A** by the addition of nucleophiles on the C3 position^19^. In contrast, *σ*-type CPCs **II**, formally generated from cyclopropenes by removing one substituent from the C1 or the C2 position, have remained unexplored in synthetic chemistry (Fig. 1a, (2)). In *σ*-type CPCs **II**, the empty *σ*-orbital is perpendicular to the C1–C2 *π*-orbital, thereby making the positive charge localized on a single carbon atom without aromatic stabilization. As such, free *σ*-type CPCs **II** decay rapidly into propargylic cations **III** and give open-chain products of type **C**^20,21^. Therefore, *σ*-type CPCs **II** can usually not be used to access C1/C2-substituted cyclopropenes of type **B**, making this class of products more difficult to access. Chemists have therefore developed synthetic equivalents of *σ*-type CPCs, but only with limited success. Cyclopropenyl bromides^22^ or iodides^23,24^ can act as *σ*-type CPC precursors in the presence of a palladium catalyst, but this approach has been limited to 3-difluoromethylated or 3,3′-difluoro cyclopropenes in cross-coupling with terminal alkynes, alkenes or aryl boronic acids. Considering the versatile role of cyclopropenes in synthetic chemistry^25^, chemical biology^26^, and medicinal^27^ and material^28^ chemistry, the availability of broadly applicable synthetic equivalents of *σ*-type CPCs would facilitate the synthesis of functionalized cyclopropenes and accelerate progress in these areas.

Our strategy was based on the generation of a transient electrophilic cyclopropenyl-metal species **IV**, which could act as a *σ*-type CPC equivalent through ligand exchange with a nucleophile to give **V**, followed by fast reductive elimination (Fig. 1b). Key design elements are good stability of the transient organometallic intermediate **IV**, fast ligand exchange and reductive elimination, and especially catalytic generation of **IV**, as the use of stoichiometric transition-metal reagents would be not sustainable. Based on the limitations of the reported approaches using cyclopropenyl bromides and iodides^22–24^, more reactive yet stable precursors will be needed for our strategy. Over the past three decades, hypervalent iodine reagents (HIRs) have been broadly applied for the umpolung of nucleophiles owing to their unique combination of extreme leaving-group ability, stability and availability^29–33^. Building on our previous studies on hypervalent iodine-based reagents^34^, we attempted the synthesis of the proposed *σ*-type CPC precursors by mixing iodine(III) compounds and nucleophilic cyclopropenyl partners (Fig. 1c). The cyclopropenyl organolithium reagents could be generated through deprotonation of cyclopropenes **s-1** with *n*-butyllithium at −78 °C. Addition of hypervalent iodine precursors **I1** or **I2** then gave various cyclopropenyl benziodoxoles (CpBXs) **1** in good yields (see Supplementary Section 2.3 for details). The structure of CpBX **1k** was confirmed by X-ray crystallography. The CpBX compounds are stable and easy to manipulate.

In this Article we describe the application of these CpBX reagents as *σ*-type CPC synthetic equivalents together with gold catalysis^35^ (Fig. 1d). We demonstrate that the oxidative addition of CpBXs to the gold catalyst occurs under mild conditions^36^, thus giving access to the *σ*-type CPC synthetic equivalent **IV** with high functional-group tolerance. Using terminal alkynes or vinylboronic acids as coupling partners, alkynyl- or alkenyl-cyclopropenes **B1** and **B2** were accessed, often in close to quantitative yields. Concerning cyclopropenyl-gold species, Hashmi and colleagues disclosed that stoichiometric Au(I)–cyclopropenyl complexes can be used as aurated carbenoids or quasi-carbene precursors^37^. In contrast, the transient Au(III)–cyclopropenyl species in our study reacted as *σ*-type CPC equivalents. Our findings reveal that the reactivity of *σ*-type CPC equivalents can be harnessed based on a gold redox process^38–40^ that enables the divergent synthesis of functionalized cyclopropenes. In addition, the obtained alkynyl–cyclopropenes can serve as versatile building blocks to access valuable functionalized cyclopropenes, cyclopropanes and conjugated enynes. The power of the transformation is further highlighted in the late-stage modification of complex natural products, bioactive molecules and drugs.

## Results and discussion

### Reaction development

The success of the proposed *σ*-type CPC transfer reaction hinged on our hypothesis that transition-metal catalysts would prefer to cleave the C–I(III) bond of CpBXs (oxidative addition) rather than the C–C bond of cyclopropenes (ring-opening reactions). Furthermore, coordination of the nucleophilic reaction partner and subsequent reductive elimination will need to be efficient to overcome the expected limited stability of the formed cyclopropenyl intermediate. Terminal alkynes are well-established partners for cross-coupling reactions and are widely represented in commercially available compounds and pharmaceuticals^41^. The cross-coupling between CpBXs and terminal alkynes would give access to useful alkynyl–cyclopropene building blocks. The synthesis of such compounds has been reported by Hashmi and colleagues using a reverse polarity approach (cyclopropenes as nucleophiles and ethynylbenziodoxole (EBX) reagents as electrophiles)^42^. However, terminal cyclopropenes with two electron-withdrawing groups are required for efficient C–H activation, resulting in a narrow scope. Monocyclopropenation of 1,3-diynes by transition-metal-catalysed reaction of diazo compounds or their surrogates is also a viable process affording alkynyl cyclopropenes^43–46^, but is limited to symmetrical 1,3-diynes. Therefore, the coupling of CpBX **1a** bearing one ester group on the C3 position and phenylacetylene (**2a**) was selected as our prototypical system. A wide range of transition-metal catalysts and ligands were investigated, and the selected conditions are presented in Table 1 (a complete list of the conditions screened is provided in Supplementary Section 4). We identified the commercial complex (Me_2_S)AuCl as an effective transition-metal catalyst, together with ligand **L1**, to deliver the cross-coupled product **3a** in 96% NMR yield in CH_3_CN at room temperature (r.t., entry 1). Without (Me_2_S)AuCl, no desired product was obtained (entry 2), and without **L1**, a drop in yield and a longer reaction time were observed (entry 3). Palladium or nickel, commonly used catalysts for C–C bond formation^47^, did not promote the desired transformation, even when a higher reaction temperature was applied, thus underscoring the specific role of gold in catalysing the *σ*-type CPC transfer reaction (entry 4). This result is noteworthy, given that gold(I) species are difficult to oxidize (Au(III)/Au(I) = 1.41 V) when compared to palladium(0) (Pd(II)/Pd(0) = 0.91 V) or nickel (0) (Ni(II)/Ni(0) = −0.24 V)^48,49^. Our findings further highlight the unique properties of hypervalent iodine reagents to promote redox-gold catalysis^42,50–52^. Increasing the amount of **L1** to 25 mol% afforded almost the same yield of **3a** as with 10 mol% (entry 5). PPh_3_-ligated gold catalysts, previously shown to be the optimal catalysts for alkynylation using EBXs^53,54^, were ineffective (entry 6). Other gold catalysts coordinated by strong *σ*-donating ligands all failed to give **3a** (Supplementary Table 1). Electron-deficient **L1** was found to be superior in accelerating the reaction than its more electron-rich analogues **L2** or **L3** (entries 7 and 8)^55^. The screening of solvents revealed that CH_3_CN was the optimal solvent compared with less polar ones, such as CH_2_Cl_2_ or tetrahydrofuran (THF; entries 9 and 10). A similar catalytic performance was also observed with AuCl as the catalyst, albeit a longer reaction time was necessary for the full conversion of **1a** (entry 11). The slightly lower reaction rate observed when using AuCl as the catalyst might be attributed to a necessary dissociative process of the polymeric gold(I) source compared to monomeric (Me_2_S)AuCl. Replacing (Me_2_S)AuCl with AuCl_3_ resulted in a drop in the yield of **3a**, which could be improved when increasing the temperature to 40 °C (entries 12 and 13). The sluggish performance for the reaction using AuCl_3_ as catalyst could be attributed to an additional induction period needed to generate the catalytically active Au(I) species^56^. As a control experiment, the use of cyclopropenyl iodide **1a-1** as the coupling partner did not yield any desired product under the standard conditions, highlighting the importance of the hypervalent iodine reagent for successful oxidative addition (entry 14). Although alkynyl silane **2a-1**^57^ (entry 15) and alkynyl pinacol boronate **2a-2** (entry 16) also gave good yields of **3a**, other acetylide surrogates, such as potassium alkynyltrifluoroborate **2a-3** (entry 17) and alkynylgermane **2a-4**^58^ (entry 18) were less efficient. It is noteworthy that the iodoarene **4** obtained during the reaction can be recovered and recycled for the synthesis of hypervalent iodine precursors **I1** or **I2** (Supplementary Sections 5 and 6 provide details).

**Table 1:**
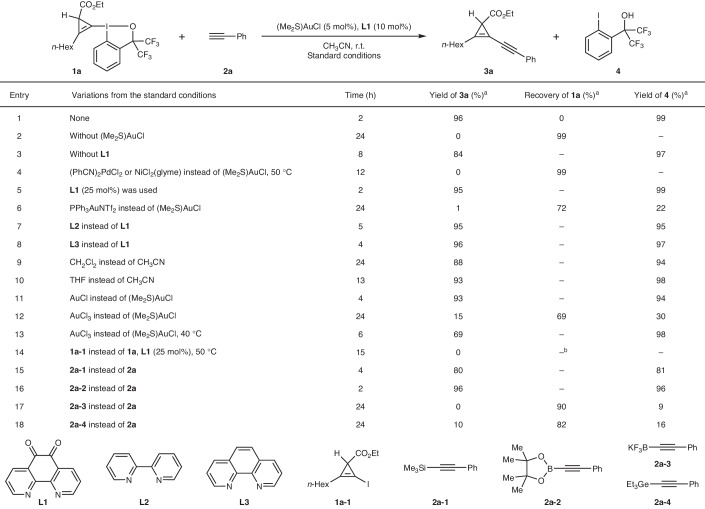
Optimization of the gold-catalysed *σ*-type CPC transfer reaction

Reactions performed on a 0.1 mmol scale.

^a^Yields or recoveries were determined by ^1^H NMR spectroscopy using dibromomethane as the internal standard.

^b^98% NMR recovery of **1a-1**.

### Scope of *σ*-type CPC transfer to terminal alkynes

With the optimal conditions in hand, we explored the scope and limitations of this *σ*-type CPC transfer reaction in terms of both functional-group compatibility and structural diversity. We first examined variation of the terminal alkyne component (Table 2). Our process worked well for terminal alkynes with aryl rings substituted with alkyl (**3b**), alkenyl (**3c**), phenyl (**3d**), CF_3_ (**3e**–**3f**), nitro (**3g**), ester (**3h**), aldehyde (**3i**) and carboxylic acid (**3j**–**3k**) groups in the *para* and *meta* positions. Electron-donating functionalities on the aryl substituent of the terminal alkyne, such as methoxy (**3l**–**3m**), trifluoromethoxy (**3n**) and carbamate (**3o**), were well tolerated. Functionalities, such as halogens (-Cl, -Br and -I, **3p**–**3r**), which can react in the presence of many transition-metal catalysts, remained intact using our protocol, thus highlighting the orthogonal reactivity of gold over palladium or nickel catalysis and allowing the installation of halogen handles for further diversification. Intriguingly, terminal alkynes bearing functionalities such as aryl silane^59^ (**3s**), aryl germane^60^ (**3t**) and aryl boronate^61^ (**3u**), previously reported to be suitable coupling partners in redox gold catalysis, remained untouched, thus underscoring the chemoselectivity of the *σ*-type CPC transfer reaction for alkynes. Additionally, this gold-catalysed cross-coupling system was shown to tolerate polyaromatic or heteroaryl-substituted alkynes, as exemplified by the synthesis of alkynylcyclopropenes substituted with naphthalene (**3v**), phenanthrene (**3w**), thiophene (**3x**) and pyridine (**3y**) moieties. A conjugated enyne was also tolerated (**3z**). The use of aliphatic terminal alkyl alkynes as coupling partners was also successful and further illustrated the compatibility of the process with a broad range of functionalities, including alkyls (**3aa–3ab**), cyclic alkanes (**3ac**–**3ad**), a chloride (**3ae**), a bromide (**3af**), an iodide (**3ag**), free alcohols (**3ah**, **3al**), a silyl ether (**3ai**), a cyclic carbamate (**3aj**), an imide (**3ak**), a benzylic ether (**3am**), a phenyl ether (**3an**), an ester (**3ao**, **3ap**) and a cyano (**3aq**) group. In particular, a propargyl benzoate, which is known to easily undergo a 1,2-migration using gold catalysis^62^, could also be accommodated, furnishing **3ao** in 93% yield. Ethyl propiolate was also suitable for this reaction, giving **3ar** in 84% yield. Acetylene gas itself, which is of particular interest owing to its availability in bulk quantity^63^, led to **3as** in moderate yield, although a higher ligand loading and higher reaction temperature were required. Notably, 1,*n*-diynes tethered by an alkyl chain or an aromatic framework underwent smooth double cross-coupling in excellent yields (**3at**–**3av**). The structure of **3au** with two alkynylcyclopropene units attached to the *para* positions of benzene was confirmed by X-ray crystallography. Next, we examined the scope of CpBXs using silyl-substituted terminal alkynes as the representative coupling partners. CpBXs bearing different alkyl substituents (R^1^) attached to the cyclopropene were suitable reaction partners and furnished products **3aw** and **3ax** in >90% yield. CpBXs featuring various ester substituents provided products **3ay** to **3bc** in excellent yields. CpBX **1i** bearing an additional alkyl substituent at the C3 position of the cyclopropenyl moiety underwent coupling with ethynyltriisopropylsilane to give tetrasubstituted alkynylcyclopropene **3bd** in 89% yield. Remarkably, trifluoromethyl-substituted CpBX **1j** could also be used in our gold-catalysed *σ*-type CPC transfer protocol to give **3be** in 78% yield. Furthermore, CpBXs derived from cyclopropenes bearing two methyl ester substituents^42,64^ at the C3 position were also excellent substrates, as showcased by the formation of alkyl- (**3bf**), phenyl- (**3bg**), fluoroaryl- (**3bh**–**3bi**) and bromoaryl- (**3bj**) substituted alkynylcyclopropenes. Other esters at the C3 position were also tolerated (**3bk**). For some substrates, the reaction temperature was increased to 40 °C to increase the reaction rate. To demonstrate the practical utility of our method, the synthesis of representative alkynylcyclopropenes **3bl**, **3bm**, **3bn** and **3bo** was performed with decreased catalyst loading (2 mol% (Me_2_S)AuCl and 4 mol% **L1**) on 2.0, 1.2, 0.6 and 1.8 mmol scales, respectively. The products were obtained in comparable yields, albeit with extended reaction times.

**Table 2:**
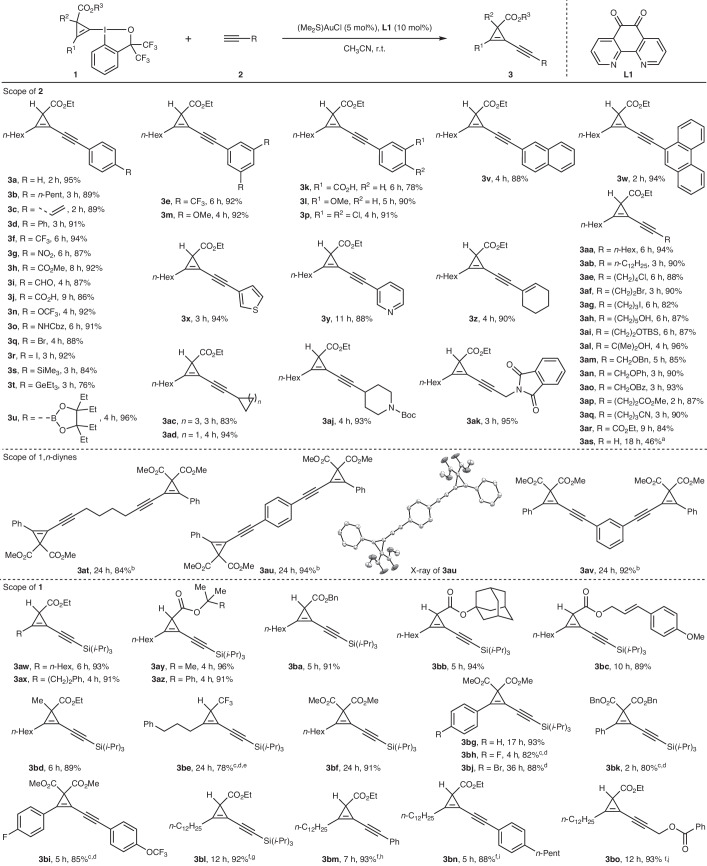
Scope of the gold-catalysed *σ*-type CPC transfer to terminal alkynes

Reaction conditions: CpBX **1** (0.2 mmol, 1.0 equiv.), terminal alkyne **2** (0.2 mmol, 1.0 equiv.), (Me_2_S)AuCl (5 mol%) and **L1** (10 mol%) were stirred in CH_3_CN (2.0 ml) at room temperature for the indicated time, unless noted otherwise. Isolated yields are given. Bz, benzoyl; Cbz, carbobenzyloxy; TBS, *tert*-butyldimethylsilyl.

^a^An acetylene balloon (1 atm) and **L1** (25 mol%) were used at 40 °C.

^b^CpBX **1k** (0.4 mmol, 2.0 equiv.), diyne **2** (0.2 mmol, 1.0 equiv.), (Me_2_S)AuCl (10 mol%) and **L1** (20 mol%) were used.

^c^Reactions carried out at 40 °C.

^d^0.1 mmol scale.

^e^CpBX **1j** (1.5 equiv.) and **L1** (25 mol%) were used instead.

^f^(Me_2_S)AuCl (2 mol%) and **L1** (4 mol%) were used instead.

^g^2 mmol scale.

^h^1.2 mmol scale.

^i^0.6 mmol scale.

^j^1.8 mmol scale.

Late-stage functionalization^65^ has emerged as an appealing strategy for the identification of bioactive compounds and requires further extension of the boundaries of modern synthesis in its ability to build and tolerate molecular complexity. Pleasingly, late-stage modification of complex natural products modified with a propargylic alkyne handle, such as (−)-camphanic acid (**3bp**) and (−)-borneol (**3bq**), as well as biologically relevant molecules such as (l)-propargylglycine (**3br**), (l)-phenylalanine (**3bs**), α-(d)-allofuranose (**3bt**) and (d)-biotin (**3bu**) could be achieved efficiently, thus confirming the generality of our method (Table 3). We next evaluated a selection of drug derivatives, including sulbactam (**3bv**), fenofibric acid (**3bw**), ciprofibrate (**3bx**), naproxen (**3by**), oxaprozin (**3bz**), isoxepac (**3ca**), febuxostat (**3cb**), indomethacin (**3cc**), mestranol (**3cd**) and norethindrone (**3ce**), resulting in the formation of the corresponding modified drug molecules in 83–99% yield. Remarkably, the sensitive core heterocyclic fragments in ezetimibe (**3cf**), artesunate (**3cg**) and gibberellic acid (**3ch**) were also well tolerated in the cross-coupling.

**Table 3:**
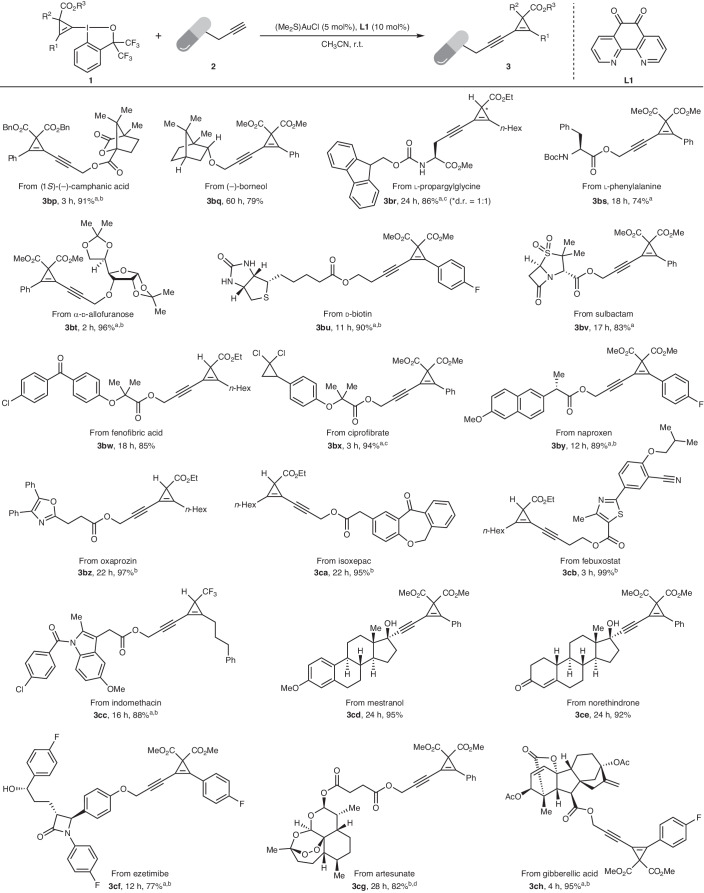
Late-stage functionalization of bioactive natural and synthetic molecules

Reaction conditions: **1** (0.1 mmol, 1.0 equiv.), **2** (0.1 mmol, 1.0 equiv.), (Me_2_S)AuCl (5 mol%) and **L1** (10 mol%) were stirred in CH_3_CN (2.0 ml) at room temperature for the indicated time, unless noted otherwise. Isolated yields are given.

^a^Reactions carried out at 40 °C.

^b^**1** (0.12 mmol, 1.2 equiv.) was used instead.

^c^**1** (0.13 mmol, 1.3 equiv.) was used instead.

^d^Reactions carried out at 30 °C.

### Scope of *σ*-type CPC transfer to vinylboronic acids

To expand the generality of our method, other potential acceptors for *σ*-type CPCs were also examined. Following extensive screening of various *sp*^2^-hybridized coupling partners, we were pleased to find that vinylboronic acids (**5**) also participate in the *σ*-type CPCs transfer reaction. With only minor modification of the standard conditions (optimization details are provided in Supplementary Table 6), the scope of this *σ*-type CPC transfer reaction to vinylboronic acids was explored. As shown in Table 4, vinylboronic acids bearing aromatic rings with different electronic properties (**6a**–**6c**) or a halogen substituent (**6d**) gave the desired products in 55–86% yield. Vinylboronic acids with an alkyl group (**6e**) or a benzyl group (**6f**) on the alkene were tolerated. Cyclic disubstituted-vinylboronic acids were also suitable substrates, furnishing the corresponding coupled products **6g**–**6j** in 53–62% yields. In addition, the scope of CpBXs was investigated with (*E*)-(4-methylstyryl)boronic acid **5b**. To our delight, CpBXs **1** bearing various alkyl substituents on the ester underwent coupling smoothly with vinylboronic acid **5b** to give vinyl–cyclopropenes **6k**–**6o** in 63–88% yield. The use of other nucleophilic partners such as allenamides or indoles was not successful (details are provided in Supplementary Section 6.3).

**Table 4:**
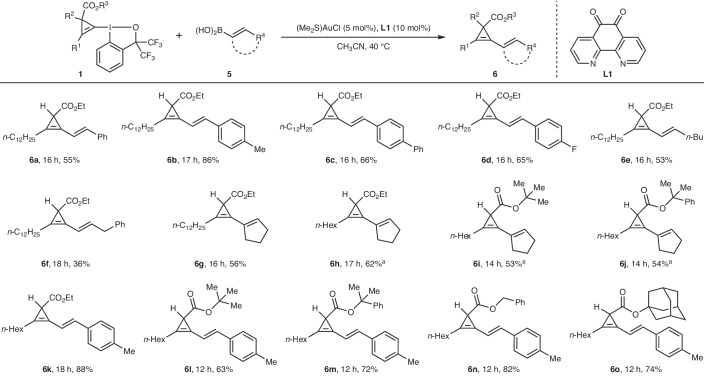
Scope of the gold-catalysed *σ*-type CPC transfer to vinylboronic acids

Reaction conditions: **1** (0.13 mmol, 1.3 equiv.), **5** (0.1 mmol, 1.0 equiv.), (Me_2_S)AuCl (5 mol%) and **L1** (10 mol%) were stirred in CH_3_CN (2.0 ml) at 40 °C for the indicated time, unless noted otherwise. Isolated yields are given.

^a^**1** (1.0 equiv.) was used instead.

### Synthetic transformations

The alkynyl–cyclopropene products are versatile building blocks for the synthesis of substituted cyclopropenes, functionalized cyclopropanes or ring-opening products (Fig. 2). Selective reduction of the ester functionality in **3bm** with diisobutylaluminium hydride (DIBAL-H)^66^ afforded hydroxymethylcyclopropene **7** in 94% yield. Alternatively, the alkene unit and the ester functionality in **3bn** were both reduced when treated with LiAlH_4_ (ref. ^67^) to provide cyclopropane **10** in 32% yield with excellent diastereoselectivity. Moreover, **7** could serve as starting material for a copper-catalysed carbomagnesiation reaction proceeding in a regio- and diastereoselective manner^68^. The in situ-formed cyclopropyl metal species could be quenched by methanol and allyl bromide, affording polysubstituted cyclopropanes **8** and **9**, respectively. Saponification of **3bm** using sodium hydroxide furnished cyclopropene carboxylic acid **11** in 82% yield. Interestingly, **3bo** could be readily converted into conjugated enyne **12** with excellent stereoselectivity in the presence of a cationic gold(I)–carbene complex^69^. Gold carbene **18**, presumably generated via 1,2-benzoyloxy migration of **3bo**, can be proposed as the key reactive intermediate, which then underwent ring-opening of the cyclopropene (a complete speculative mechanism is provided in Supplementary Fig. 1). A Diels–Alder reaction of **3bo** with 2,3-dimethylbutadiene gave fused bicycle **13** in 89% yield and >20:1 diastereoselectivity (d.r.). Additionally, desilylation of **3bl** using tetrabutylammonium fluoride (TBAF)^70^ allowed access to cyclopropene **14** bearing a terminal alkyne, which can itself serve as a suitable partner in the gold-catalysed *σ*-type CPC transfer reaction, affording non-symmetrical 1,2-bis-cyclopropenyl substituted alkyne **15**. A gold-catalysed cross-coupling of **14** with hypervalent iodine reagent **19** (ref. ^53^) gave cyclopropenyl 1,3-diyne **16** in 86% yield. Finally, copper(I)-catalysed alkyne–azide cycloaddition^71^ of **14** and benzyl azide provided cyclopropenyl triazole **17** in 72% yield.

### Mechanistic investigations

**Fig. 3:**
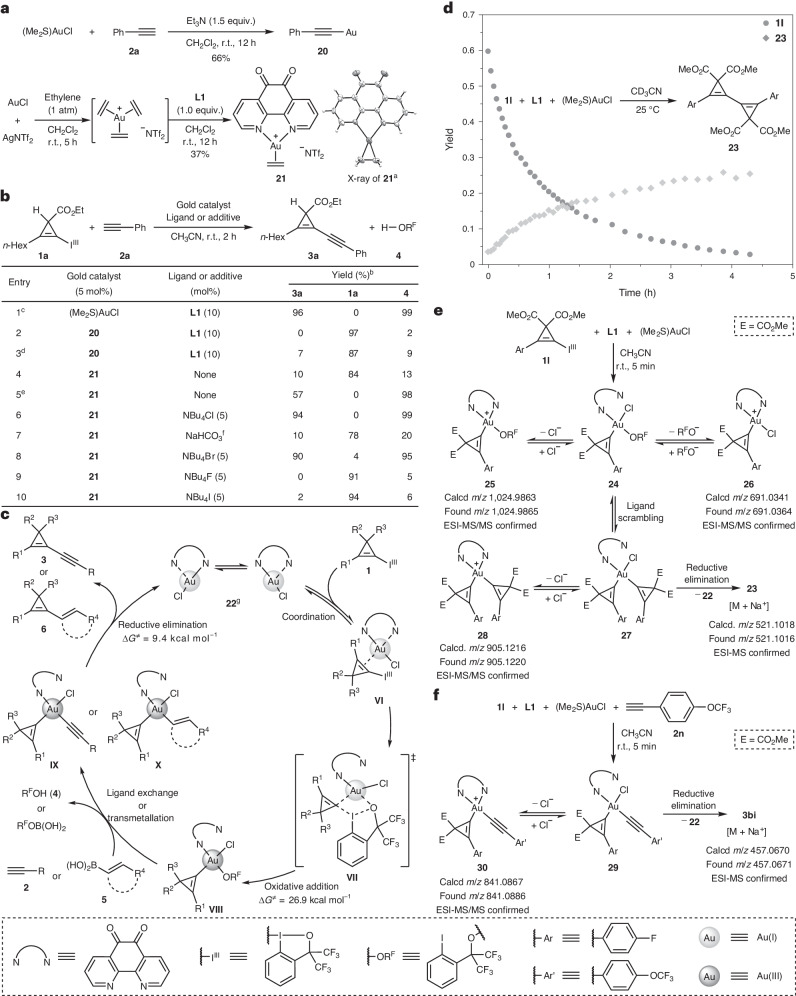
Mechanistic studies on the gold-catalysed *σ*-type CPC transfer reaction. **a**, Preparation of gold(I)–phenylacetylide **20** and cationic gold(I)–ethylene complex **21**. **b**, Control experiments for determining the catalytically active species. Gold(I)–acetylide complex **20** was not a competent catalyst. Chloride plays a crucial role in accelerating the coupling reaction. **c**, Proposed reaction mechanism. **d**, Reaction profile of the stoichiometric reaction of CpBX **1l** and (Me_2_S)AuCl, with **L1** as the ligand. **e**, ESI-MS/MS analysis of the stoichiometric reaction of CpBX **1l**, (Me_2_S)AuCl and **L1** supporting the formation of intermediate **VIII**. **f**, ESI-MS/MS analysis of the stoichiometric reaction of CpBX **1l**, (Me_2_S)AuCl, **L1** and alkyne **2n** supporting the formation of intermediate **IX**. ^a^Solid-state structure of 21, with thermal displacement ellipsoids given at 50% probability. The counterion is omitted for clarity. ^b^Determined by ^1^H NMR. ^c^Standard condition (Table 1, entry 1). ^d^Reaction carried out at 40 °C for 24 h. ^e^Reaction carried out at 40 °C for 10 h. ^f^NaHCO3 (3.0 equiv.) was used. ^g^**22** is proposed to be a fluxional species with fast exchange of the coordination sites of gold(I) to the two nitrogen atoms of L1. ESI, electrospray ionization. Source data

To gain some insights into the reaction mechanism^72,73^, we first attempted to identify the active gold species at the start of the catalytic cycle. We prepared the ligand-free polymeric gold(I)–phenylacetylide **20**^74^ and the cationic gold(I)–ethylene complex **21**^75^ as potential gold sources (Fig. 3a). We first investigated the use of 5 mol% **20** in the cross-coupling of CpBX **1a** and terminal alkyne **2a** under the standard conditions. No cross-coupled product was observed (Fig. 3b, entry 2). Running the reaction at 40 °C for 24 h, only 7% yield of product **3a** was observed with most **1a** (87%) recovered (Fig. 3b, entry 3). These results do not support a catalytic cycle involving the direct oxidation of a gold(I)–acetylide complex by CpBX **1**. When the cationic gold(I)–ethylene complex **21** was used, a 10% yield of **3a** was observed at room temperature after 2 h (Fig. 3b, entry 4). The yield could be improved to 57% by running the reaction at 40 °C for 10 h with full conversion of **1a** (Fig. 3b, entry 5), indicating that the cationic gold(I) species can be oxidized by CpBX **1**. The poor catalytic performance of cationic gold(I) complex **21** under the standard conditions could be attributed to the formation of a catalytically inert gold(I)–acetylide in the presence of an excess of terminal alkyne (Supplementary Section 8.3 presents more details). In contrast, when 5 mol% of **21** and 5 mol% of NBu_4_Cl were used, **3a** was obtained in 94% yield (Fig. 3b, entry 6), with an efficiency similar to the one observed under standard conditions (Fig. 3b, entry 1). A control experiment using NaHCO_3_ (3.0 equiv.) as an additive did not show any improvement (Fig. 3b, entry 7), supporting the important role of chloride. To support this hypothesis, other halogenide additives were examined. Bromide exhibited a similar effect on the reaction outcome (Fig. 3b, entry 8). In contrast, the use of fluoride and iodide nearly completely suppressed the formation of **3a** (Fig. 3b, entries 9 and 10). A catalytically active tricoordinated gold(I) chloride species^55^, **22**, undergoing oxidative addition onto CpBX **1** would be in accordance with these observations (Fig. 3c). The NMR spectra of **22** prepared independently by mixing an equimolar amount of **21** and NBu_4_Cl in CD_2_Cl_2_ showed symmetric ligand backbone signals, which were distinct from those of **21** or **L1** (spectra details are provided in Supplementary Fig. 3). Species **22** is therefore proposed to be a fluxional species with fast exchange of the coordination sites of gold(I) to the two nitrogen atoms of **L1**^76^. A weak coordination of the gold centre to CpBX **1** would afford transient *π*-type Au(I) species **VI**. Subsequently, oxidation of gold(I) to gold(III) with the concomitant transfer of the cyclopropenyl moiety from iodine to gold via a concerted four-membered ring transition state **VII** would generate the square-planar Au(III)–cyclopropenyl species **VIII**^77^. The highly electrophilic and reactive species **VIII** would capture terminal alkyne **2** via ligand exchange or undergo transmetallation with vinylboronic acid **5** to generate gold(III) species **IX** or **X**, which, upon reductive elimination, would yield cross-coupled products **3** or **6**, respectively, and regenerate the gold(I) catalyst **22**.

We next performed further computations and mechanistic experiments to support the proposed catalytic cycle and the putative Au(III)–cyclopropenyl species. First, we used density functional theory (DFT) at the B3PW91-D3(BJ)/def2-TZVP//PBE0-D3(BJ)/def2-SVP level (Supplementary Section 8.8 and Supplementary Fig. 19) to further assess the feasibility of the key oxidative addition and reductive elimination steps. Activation energies of 26.9 kcal mol^−1^ and 9.4 kcal mol^−1^ were obtained for the oxidative addition and reductive elimination processes, respectively. This further supported that both steps were feasible, even if the energy for the oxidative addition step remains a little high when considering the reaction rate. We then used ^19^F NMR spectroscopy to monitor the stoichiometric reaction of an equimolar amount of CpBX **1l**, (Me_2_S)AuCl and **L1** in CD_3_CN at ambient temperature. As shown in Fig. 3d, CpBX **1l** readily reacted with gold(I) and was completely consumed within 5 h, thus confirming the reactivity of CpBX **1** with gold(I) species. Intriguingly, the homo-coupled product **23** was formed, which may originate from the labile Au(III)–cyclopropenyl species **24** (vide infra). To further probe the intermediacy of the proposed Au(III)–cyclopropenyl species **VIII**, we then used electrospray ionization mass spectrometry (ESI-MS) techniques to monitor the reaction mixture. As shown in Fig. 3e, a reaction mixture of equimolar **1l**, (Me_2_S)AuCl and **L1** in CH_3_CN (at room temperature for 5 min) was subjected to high-resolution mass analysis. Although **24** was not observed directly by mass spectrometry due to its electroneutral nature, cationic Au(III) species **25** and **26** derived from **24** by losing one anionic fragment were both observed by ESI-MS and structurally confirmed by tandem mass spectrometry (MS/MS) (Supplementary Section 8.6). Interestingly, the cationic Au(III)-bis(cyclopropenyl) species **28**, derived from **27** by losing chloride, was also observed, indicating the mechanism for the formation of **23**, that is, ligand scrambling^78^ of **24** to **27** followed by reduction elimination to furnish **23**. Finally, we sought to gain support for the putative Au(III) species **IX**, a key organogold species in the catalytic cycle to connect the transmetallation and reductive elimination step. As shown in Fig. 3f, a reaction mixture of equimolar **1l**, (Me_2_S)AuCl, **L1** and terminal alkyne **2n** in CH_3_CN was subjected to high-resolution mass analysis. Gratifyingly, the expected cationic Au(III) species **30**, derived from **29** by losing chloride, was observed by ESI-MS and further structurally determined by MS/MS analysis, thus providing direct evidence for the participation of Au(III)–cyclopropenyl species **IX** in the catalytic cycle. Overall, these experiments support the mechanism proposed in Fig. 3c well, even if it is not completely certain which of the chloride or cationic species is on or off the catalytic cycle.

## Conclusion

In summary, we have developed broadly applicable synthetic equivalents of the elusive and untapped *σ*-type CPCs. The required CpBXs, newly designed iodine(III)-based precursors of *σ*-type CPCs, can be prepared from readily available reagents. Gold(I) complexes were used as catalysts for the intermolecular *σ*-type CPC transfer reaction of CpBXs **1** to terminal alkynes or vinylboronic acids under mild conditions, providing straightforward access to alkynyl–cyclopropenes and vinyl–cyclopropenes. The gold-catalysed protocol exhibited a broad substrate scope and tolerated numerous functional groups. This protocol can be further applied to the late-stage elaboration of complex organic compounds and drug molecules containing an alkyne handle. The alkynyl–cyclopropene products have been shown to be versatile synthetic intermediates for downstream diversification. Mechanistic studies support the intermediacy of a highly electrophilic cyclopropenyl–Au(III) species as a *σ*-type CPC equivalent and provide evidence for the crucial role of chloride as a supporting ligand for efficient coupling. Our work therefore substantially extends the chemical diversity of easily accessible cyclopropene building blocks, with applications in synthetic and medicinal chemistry, and will inspire other researchers in the design of new synthons based on the merger of hypervalent iodine reagents and redox gold catalysis.

## Methods

### General procedure for the synthesis of cyclopropenyl benziodoxoles 1 (CpBXs)

An oven-dried Schlenk tube was charged with a magnetic stirring bar and the terminal cyclopropene **s-1** (4.00 mmol, 1.00 equiv.). The Schlenk tube was then evacuated and backfilled with nitrogen three times. THF (40 ml) was added by syringe and the Schlenk tube was placed at −78 °C in a dry ice/acetone bath. We then added *n*-butyllithium (2.5 M in hexane; typically, 4.2 mmol, 1.7 ml, 1.05 equiv.) dropwise via a syringe pump over 5 min, and the reaction mixture was stirred at −78 °C for an additional 10 min. Hypervalent iodine precursor **I1** (typically, 4.40 mmol, 1.78 g, 1.10 equiv.) was added in one portion under nitrogen. The reaction mixture was stirred at −78 °C for 15 min, then the cooling bath was removed. The reaction mixture was allowed to warm to room temperature gradually (typically for ~15 min) while stirring. The reaction mixture was then quenched by adding saturated aqueous NaHCO_3_ (40 ml). The organic layer was removed, and the remaining aqueous portion was extracted with EtOAc (3 × 10 ml). The combined organic portions were dried over Na_2_SO_4_, filtered, and the volatiles removed under reduced pressure. The crude product was purified by flash chromatography on silica gel, and the fractions that contained the product were collected and concentrated by rotary evaporation to afford the purified compound.

### General procedure for the synthesis of alkynyl–cyclopropenes 3

An oven-dried 10-ml Schlenk tube with a magnetic stirring bar was sequentially charged with **L1** (4.20 mg, 20.0 μmol, 10.0 mol%), (Me_2_S)AuCl (2.95 mg, 10.0 μmol, 5.0 mol%), terminal alkyne **2** (200 μmol, 1.00 equiv.) and CpBX **1** (200 μmol, 1.00 equiv.). The Schlenk tube was then evacuated and backfilled with nitrogen three times. Subsequently, CH_3_CN (2.0 ml) was added by syringe. If **2** was a liquid, it was added last. The reaction mixture was stirred at room temperature (~21 °C) for the specified time. The reaction mixture was then filtered through a silica gel pad and washed with CH_2_Cl_2_ (3 × 5 ml). Excess solvent was removed under reduced pressure and the desired product **3** was obtained by column chromatography on silica gel.

### General procedure for the synthesis of vinyl–cyclopropenes 6

An oven-dried 10-ml Schlenk tube with a magnetic stirring bar was sequentially charged with **L1** (2.10 mg, 10.0 μmol, 10.0 mol%), (Me_2_S)AuCl (1.47 mg, 5.00 μmol, 5.0 mol%), vinylboronic acid **5** (100 μmol, 1.00 equiv.) and CpBX **1** (typically, 130 μmol, 1.30 equiv.). The Schlenk tube was then evacuated and backfilled with nitrogen three times. Subsequently, CH_3_CN (2.0 ml) was added by syringe. The reaction mixture was stirred at 40 °C for the specified time. The reaction mixture was then filtered through a silica gel pad and eluted with CH_2_Cl_2_ (3 × 5 ml). The solvent was removed under reduced pressure, and the resulting crude residue was subjected to a short column chromatography stage (silica). The fractions that contained the products were collected and analysed by ^1^H NMR spectroscopy. The recovered sample was purified by flash column chromatography (C18 reverse phase) to give cross-coupled product **6**.

## Online content

Any methods, additional references, Nature Portfolio reporting summaries, source data, extended data, supplementary information, acknowledgements, peer review information; details of author contributions and competing interests; and statements of data and code availability are available at 10.1038/s41557-024-01535-8.

### Supplementary information

Supplementary Information The Supplementary Information file contains 11 sections, covering the experimental procedure, synthesis and characterization data, NMR spectra, X-ray crystallographic data, DFT calculation and references, Figs. 1–19 and Tables 1–9.

Supplementary Data 1 Crystallographic data for compound **1k**; CCDC reference 2260609.

Supplementary Data 2 Crystallographic data for compound **3au**; CCDC reference 2260610.

Supplementary Data 3 Crystallographic data for compound **21**; CCDC reference 2260611.

Supplementary Data 4 Cartesian coordinates for the computation performed in Fig. 3.

### Source data

Source Data Fig. 3 Statistical source data of Fig. 3d.

## Data Availability

Materials and methods, experimental procedures, computational details, mechanistic studies, ^1^H NMR spectra, ^13^C NMR spectra, ^11^B NMR spectra, ^19^F NMR spectra and mass spectrometry data, as well as all other supporting data for the article, are available in the Supplementary Information. Raw data for compound characterization are available with free access on zenodo.org: 10.5281/zenodo.10674147 (ref. ^79^). Crystallographic data for the structures reported in this article have been deposited at the Cambridge Crystallographic Data Centre, under deposition nos. CCDC 2260609 (**1k**), 2260610 (**3au**) and 2260611 (**21**). Copies of the data can be obtained free of charge via https://www.ccdc.cam.ac.uk/structures/. Source data are provided with this paper.
