# Supplementary material for: Accessing elusive σ-type cyclopropenium cation equivalents through redox gold catalysis
Source: Nat Chem. 2024 May 23;16(6):901–12. doi: 10.1038/s41557-024-01535-8 (PMC11164686; doi:10.1038/s41557-024-01535-8)
Supplement: Supplementary file 1 — The Supplementary Information file contains 11 sections, covering the experimental procedure, synthesis and characterization data, NMR spectra, X-ray crystallographic data, DFT calculation and references, Figs. 1–19 and Tables 1–9. [file 41557_2024_1535_MOESM1_ESM.pdf]

nature chemistry

Supplementary information https://doi.org/10.1038/s41557-024-01535-8

Accessing elusive σ-type cyclopropenium cation equivalents through redox gold catalysis

In the format provided by the authors and unedited

Table of Contents

1. General Information 3

2. Development of σ-type cyclopropenium cations transfer reagents 4

2.1. Synthesis of terminal cyclopropenes by rhodium catalysis 4

2.2. Synthesis of hypervalent iodine precursor 10

2.3. Synthesis of σ-type cyclopropenium cations transfer reagents CpBXs 11

3. Synthesis and characterisation of terminal alkynes and alkyne surrogates 21

3.1. Terminal alkynes and alkyne surrogates 21

3.2. Terminal alkynes tethered with natural products, bioactive molecules and pharmaceuticals 27

3.3. Synthesis and characterization of *N*-allenamides 37

4. Optimization of σ-type cyclopropenium cation transfer reaction with terminal alkynes 38

4.1. Evaluation of gold catalysts 38

4.2. Evaluation of bidentate ligands 39

4.3. Evaluation of transition metal catalysts 40

4.4. Variations from the standard condition 41

5. Substrate scope of σ-type cyclopropenium cation transfer to terminal alkyne 42

5.1. Substrate scope of terminal alkyne 43

5.2. Substrate scope of cyclopropenylbenziodoxoles CpBXs 66

5.3. Scale up experiment of σ-type cyclopropenium cation transfer to terminal alkyne 73

5.4. Late-stage functionalization of bioactive and drug molecules 75

6. Substrate scope of σ-type cyclopropenium cation transfer to vinylboronic acid 86

6.1. Optimization of σ-type cyclopropenium cation transfer to vinylboronic acid 86

6.2. Survey of gold-catalysed σ-type cyclopropenium cation transfer to vinylboronic acid 87

6.3. Attempts of gold-catalysed σ-type cyclopropenium cation transfer to other substrates 94

7. Transformations of products and applications 96

7.1. Selective reduction of alkynyl-cyclopropenes using DIBAL-H 96

7.2. Hydroalkylation of alkynyl-cyclopropene **7** via copper-catalyzed carbomagnesiation 97

7.3. Difunctionalization of alkynyl-cyclopropene **7** via copper-catalyzed carbomagnesiation[33] 97

7.4. Selective reduction of alkynyl-cyclopropenes using $LiAlH_4$ 98

7.5. Saponification of alkynyl-cyclopropene **3bm** 99

7.6. Gold(I)-catalysed rearrangement of propargylic benzoate and ring-opening cascade 99

7.7. Diels–Alder reaction of **3bo** with 2,3-dimethylbutadiene 100

7.8. Desilylation of **3bl** using TBAF to terminal cyclopropenyl alkyne **14** 101

7.9. Gold-catalysed σ-type CPCs transfer reaction for the synthesis of non-symmetrical 1,2-bis-cyclopropenyl substituted alkyne 101

7.10. Au-Ag bimetallic catalysis providing unsymmetrical cyclopropenyl 1,3-diyne 102

7.11. Copper(I)-catalysed alkyne-azide cycloaddition of **14** and benzyl azide 102

8. Mechanistic investigations **103**
8.1. Preparation of potential gold catalysts 103
8.2. Control experiments for determining the catalytically active species 104
8.3. Stoichiometric reaction of cationic gold(I)-ethylene complex **21** and terminal alkyne **2n** 105
8.4. Generation of **L1**-ligated AuCl **22** from cationic gold(I)-ethylene complex **21** and Bu_4NCl 107
8.5. Stoichiometric reaction of CpBX **1l** and chloride-supported gold(I) catalyst 108
8.6. Tandem mass spectrometry (ESI-MS/MS) analysis for the capture of active intermediates 110
8.7. Stoichiometric reaction monitored by NMR 117
8.8. DFT Calculations 120
9. Single crystal X-ray diffraction analysis **121**
9.1. Crystal data and structure refinement for **1k** 121
9.2. Crystal data and structure refinement for **3au** 122
9.3. Crystal data and structure refinement for **21** 123
10. NMR spectra **124**
11. References **314**

1. General Information

All reactions were carried out using standard Schlenk technique under nitrogen unless otherwise stated. All reagents were purchased from major commercial suppliers (Sigma-Aldrich, Merck, Fluorochem, Combi-blocks, Fluka, Apollo Scientific, Fischer Scientific, Tokyo Chemical Industry, Acros Organics) and used as such unless otherwise noted. Dry solvents (DCM, THF, MeCN, toluene and Et_2O) were obtained fresh from an Innovative Technology solvent purification system having been passed through anhydrous alumina columns. 1,2-Dichloroethane (99.5%, extra dry over molecular sieve, AcrosSeal®) was purchased from Thermo Scientific Chemicals. Unless otherwise stated, solvents were used without further drying or degassing. (Me_2S)AuCl and 1,10-phenanthroline-5,6-dione (**L1**) used in the σ-type CPC transfer reactions were purchased from Sigma-Aldrich and Combi-Blocks, respectively. The AuCl (97%, 99.99%-Au) was purchased from abcr GmbH. Gold(III) chloride (99%) was purchased from Sigma-Aldrich. Ethyl diazoacetate (contains ≥13 wt. % dichloromethane) was purchased from Sigma-Aldrich (E22201-100G) and used without further purification. Reactions were monitored by thin layer chromatography (TLC) on Merck silica gel 60 F_{254} TLC glass plates and visualized with ultraviolet irradiation (254 nm) and/or potassium permanganate stain. Flash column chromatography (FCC) was carried out using Biotage Isolera One with pre-packaged silica cartridges (EcoFlex Silica 4 g, 12 g, 25 g, 40g, 80g, 120g) purchased from Büchi or C18 reverse phase chromatography (Aquarius C18AQ 20 g; 100 Å, Spherical, 30 µm, Flow rate: 10-25 mL/min). ^1H NMR spectra were recorded on a Bruker Ascend™ 400 400 MHz spectrometer and reported as chemical shifts (δ) in parts per million (ppm) relative to the residual non-deuterated solvent signal as internal reference (chloroform-*d*: 7.26 ppm; DMSO-d_6: 2.50 ppm; acetoned-d_6: 2.06 ppm; CD_3CN: 1.94 ppm). ^{13}C NMR spectra were recorded with {^1H} decoupling on a Bruker Ascend™ 400 101 MHz spectrometer and reported in ppm using the residual solvent signal as internal reference (chloroform-*d*: 77.16 ppm; DMSO-d_6: 39.52 ppm; acetoned-d_6: 29.84 ppm; CD_3CN: 1.32 ppm). ^{19}F-NMR spectra were recorded with {^1H} decoupling on a Bruker Ascend™ 400 376 MHz spectrometer. Data are reported as follows: chemical shift, multiplicity (s = singlet, d = doublet, t = triplet, q = quartet, pent = pentet, sept = septet, br = broad, m = multiplet), coupling constants (Hz) and integration. NMR spectra were processed with MestReNova (version 14.2.1). High resolution mass spectrometric measurements were performed by the mass spectrometry service of ISIC at the EPFL. Electrospray-ionisation HRMS data were acquired on a Q-Tof Ultima mass spectrometer (Waters) or a Q-Tof 6530 Accurate mass spectrometer (Agilent) operated in the positive ionization mode and fitted with a standard Z-spray ion source equipped with the Lock-Spray interface. Data from the Lock-Spray were used to calculate a correction factor for the mass scale and provide accurate mass information of the analyte. Data were processed using the MassLynx 4.1 software. Atmospheric pressure photo-ionisation (APPI) HRMS measurements were done on an LTQ Orbitrap Elite instrument (Thermofisher) operated in the positive ionization mode. The raw data obtained from the Q-TOF Waters instrument does not consider the mass of the electron for the ion, the obtained raw data has been corrected by removing (positive ionization) or adding (negative ionization) the mass of the electron (0.5 mDa). Infrared spectra were recorded using a JASCO FT/IR-4100 Fourier Transform Infrared Spectrometer at room temperature, and the stretching frequencies are reported in wavenumbers (cm^{-1}) (s = strong, m = medium, w = weak). Elemental Analyses were performed using an UNICUBE analyzer (Elementar, France) operated in the CHNS mode. Melting points were measured using a Büchi Melting Point B-540 and were uncorrected. The specific rotation was measured with a Jasco P-2000 polarimeter at 20 °C. The given specific rotation is the mean value from 10 measurements. The concentration for the specific rotation measurements is given in 10 mg/mL.

2. Development of σ-type cyclopropenium cations transfer reagents

2.1. Synthesis of terminal cyclopropenes by rhodium catalysis

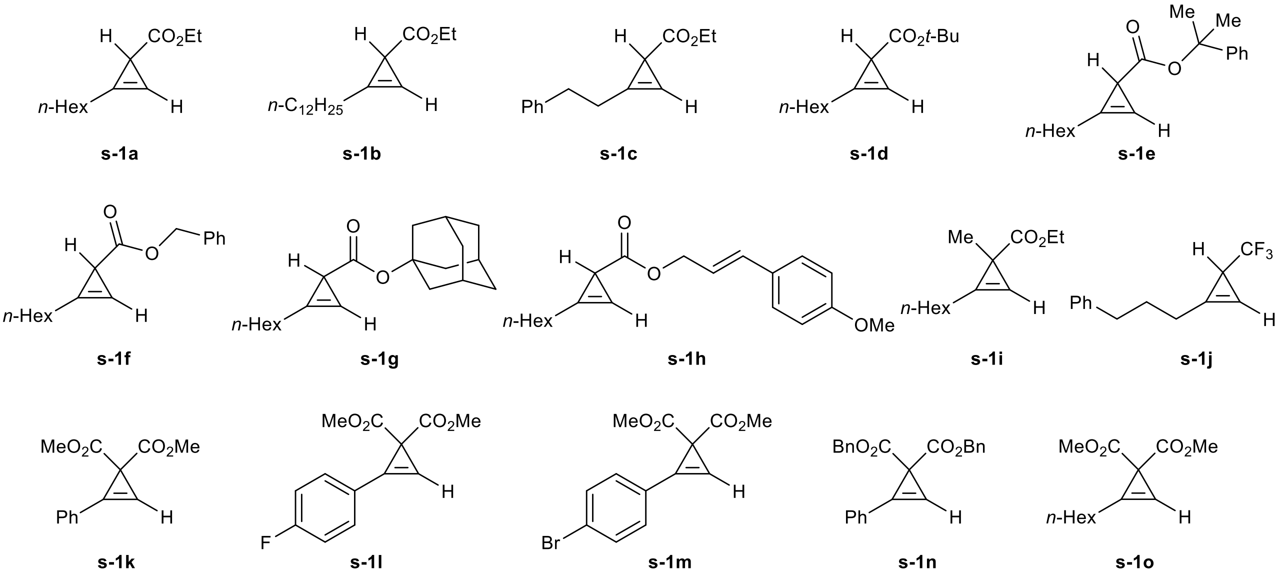

General Procedure A (**GPA**)[1]:

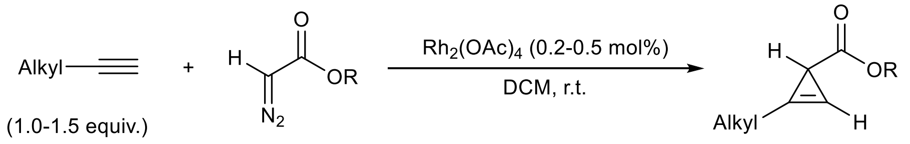

s-1

GPA: To a stirred solution of $Rh_2(OAc)_4$ (0.2-0.5 mol%) and terminal alkyne (1.0-1.5 equiv.) in CH_2Cl_2 (1.00-1.50 M) at room temperature was added a solution of diazo ester (1.0 equiv.) in CH_2Cl_2 (1.00 M) via syringe pump over 8 h under nitrogen. After the addition was complete, the mixture was stirred for additional 8 h, filtered through a short pad of Celite® eluting with CH_2Cl_2, and concentrated under reduced pressure. Purification of the residue by column chromatography (pentane/ethyl acetate) to afford **s-1**.

2.1.1. Synthesis and characterization of ethyl 2-hexylcycloprop-2-ene-1-carboxylate (s-1a)

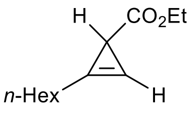

s-1a

Following **GPA**, oct-1-yne (1.65 g, 15.0 mmol, 1.50 equiv.), ethyl diazoacetate (1.21 mL, 10.0 mmol, 1.00 equiv.), $Rh_2(OAc)_4$ (22.1 mg, 50.0 μmol, 0.5 mol%) and CH_2Cl_2 (20 mL) were used. Column chromatography on silica gel (eluent: pentane/ethyl acetate = 50:1) afforded **s-1a** in 79% yield (1.55 g, 7.88 mmol) as a colourless oil. **TLC**: R_f (*n*-hexane: EtOAc = 40:1) = 0.23; **^{1}H NMR** (400 MHz, $CDCl_3$) δ 6.32 (q, *J* = 1.5 Hz, 1H, C=C*H*), 4.21 – 4.05 (m, 2H, OCH_2CH$_3$), 2.49 (td, *J* = 7.3, 1.4 Hz, 2H, CH$_2$CH_2C), 2.12 (d, *J* = 1.5 Hz, 1H, C*H*CO$_2$Et), 1.64 – 1.51 (m, 2H, CH_2CH$_2$C), 1.44 – 1.20 (m, 9H, CH_2 & OCH$_2$CH_3), 0.96 – 0.82 (m, 3H, CH_3); **^{13}C NMR** (101 MHz, $CDCl_3$) δ 176.8, 115.8, 94.0, 60.3, 31.6, 28.9, 26.8, 25.1, 22.7, 19.9, 14.5, 14.2. The NMR spectroscopic data is consistent with previous report[2].

2.1.2. Synthesis and characterization of ethyl 2-dodecylcycloprop-2-ene-1-carboxylate (s-1b)

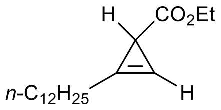

s-1b

Following **GPA**, tetradec-1-yne (1.94 g, 10.0 mmol, 1.0 equiv.), ethyl diazoacetate (1.21 mL, 10.0 mmol, 1.0 equiv.), $Rh_2(OAc)_4$ (22.1 mg, 50.0 µmol, 0.5 mol%) and CH_2Cl_2 (20 mL) were used. Column chromatography on silica gel (eluent: pentane/ethyl acetate = 50:1) afforded **s-1b** in 70% yield (1.97 g, 7.03 mmol) as a colourless oil. **TLC**: R_f (*n*-hexane: EtOAc = 20:1) = 0.44; ^{1}**H NMR** (400 MHz, $CDCl_3$) δ 6.31 (q, *J* = 1.5 Hz, 1H, C=C*H*), 4.18 – 4.06 (m, 2H, OCH_2CH_3), 2.48 (td, *J* = 7.3, 1.4 Hz, 2H, CH_2CH_2C), 2.12 (d, *J* = 1.5 Hz, 1H, C*H*CO_2Et), 1.61 – 1.54 (m, 2H, CH_2CH_2C), 1.39 – 1.19 (m, 21H, CH_2 & OCH_2CH_3), 0.88 (t, *J* = 6.8 Hz, 3H, CH_3); ^{13}**C NMR** (101 MHz, $CDCl_3$) δ 176.8, 115.8, 94.0, 60.3, 32.1, 29.80, 29.78, 29.76, 29.7, 29.5, 29.4, 29.3, 26.8, 25.1, 22.8, 19.9, 14.5, 14.3; **IR** (ν_{max}, cm^{-1}) 2955 (m), 2925 (s), 2855 (s), 1725 (s), 1465 (m), 1370 (w), 1339 (w), 1253 (m), 1183 (s), 1038 (m), 960 (w), 745 (w); **HRMS** (ESI/QTOF) m/z: $[M + Na]^+$ Calcd for $C_{18}H_{32}NaO_2^+$ 303.2295; Found 303.2295.

2.1.3. Synthesis and characterization of ethyl 2-phenethylcycloprop-2-ene-1-carboxylate (s-1c)

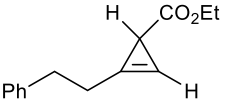

s-1c

Following **GPA**, but-3-yn-1-ylbenzene (1.95 g, 15.0 mmol, 1.50 equiv.), ethyl diazoacetate (1.21 mL, 10.0 mmol, 1.00 equiv.), $Rh_2(OAc)_4$ (8.84 mg, 20.0 µmol, 0.2 mol%) and CH_2Cl_2 (20 mL) were used. Column chromatography on silica gel (eluent: pentane/ethyl acetate = 50:1) afforded **s-1c** in 67% yield (1.46 g, 6.74 mmol) as a colourless oil. **TLC**: R_f (*n*-hexane: EtOAc = 20:1) = 0.29; ^{1}**H NMR** (400 MHz, $CDCl_3$) δ 7.33 – 7.24 (m, 2H, Ar*H*), 7.24 – 7.16 (m, 3H, Ar*H*), 6.36 (q, *J* = 1.4 Hz, 1H, C=C*H*), 4.20 – 4.04 (m, 2H, OCH_2CH_3), 2.95 – 2.88 (m, 2H, CH_2CH_2), 2.86 – 2.78 (m, 2H, CH_2CH_2), 2.14 (d, *J* = 1.5 Hz, 1H, C*H*CO_2Et), 1.25 (t, *J* = 7.1 Hz, 3H, OCH_2CH_3). The NMR spectroscopic data is consistent with previous report[1].

2.1.4. Synthesis and characterization of *tert*-butyl 2-hexylcycloprop-2-ene-1-carboxylate (s-1d)

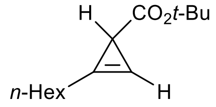

s-1d

Following **GPA**, oct-1-yne (482 mg, 4.37 mmol, 1.5 equiv.), *tert*-butyl 2-diazoacetate (414 mg, 2.92 mmol, 1.0 equiv.), $Rh_2(OAc)_4$ (6.44 mg, 14.6 µmol, 0.5 mol%) and CH_2Cl_2 (10 mL) were used. Column chromatography on silica gel (eluent: pentane/ethyl acetate = 50:1) afforded **s-1d** in 53% yield (350 mg, 1.56 mmol) as a colourless oil. **TLC**: R_f (*n*-hexane: EtOAc = 20:1) = 0.41; ^{1}**H NMR** (400 MHz, $CDCl_3$) δ 6.29 (q, *J* = 1.5 Hz, 1H, C=C*H*), 2.47 (tt, *J* = 7.2, 1.6 Hz, 2H, CH_2CH_2C), 2.02 (d, *J* = 1.6 Hz, 1H, C*H*CO_2), 1.61 – 1.53 (m, 2H, CH_2CH_2C), 1.43 (s, 9H, $C(CH_3)_3$), 1.40 – 1.25 (m, 6H, CH_2), 0.88 (t, *J* = 6.8 Hz, 3H, CH_3); ^{13}**C NMR** (101 MHz, $CDCl_3$) δ 176.2, 116.1, 94.3, 79.7, 31.7, 29.0, 28.3, 26.9, 25.1, 22.7, 20.8, 14.2; **IR** (ν_{max}, cm^{-1}) 2958 (m), 2930 (m), 2860 (w), 1801 (w), 1720 (s), 1458 (w), 1391 (w), 1367 (m), 1346 (m), 1273 (w), 1254 (m), 1213 (m), 1153 (s), 963 (m), 858 (w), 741 (m); **HRMS** (ESI/QTOF) m/z: $[M + Na]^+$ Calcd for $C_{14}H_{24}NaO_2^+$ 247.1669; Found 247.1677.

2.1.5. Synthesis and characterization of 2-phenylpropan-2-yl 2-hexylcycloprop-2-ene-1-carboxylate (s-1e)

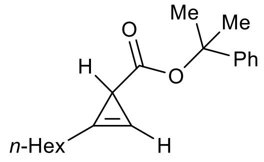

s-1e

Following **GPA**, oct-1-yne (491 mg, 4.46 mmol, 1.50 equiv.), 2-phenylpropan-2-yl 2-diazoacetate[3] (606 mg, 2.97 mmol, 1.00 equiv.), $Rh_2(OAc)_4$ (6.60 mg, 14.9 µmol, 0.5 mol%) and CH_2Cl_2 (10 mL) were used. Column chromatography on silica gel (eluent: pentane/ethyl acetate = 50:1) afforded **s-1e** in 63% yield (537 mg, 1.88 mmol) as a colourless oil. **TLC**: R_f (*n*-hexane: EtOAc = 20:1) = 0.36; **^{1}H NMR** (400 MHz, $CDCl_3$) δ 7.37 – 7.29 (m, 4H, Ar*H*), 7.24 – 7.20 (m, 1H, Ar*H*), 6.32 (q, *J* = 1.5 Hz, 1H, C=C*H*), 2.48 (tt, *J* = 7.2, 1.3 Hz, 2H, CH_2CH_2C), 2.14 (d, *J* = 1.5 Hz, 1H, C*H*CO_2), 1.78 (s, 3H, C(CH_3)), 1.75 (s, 3H, C(CH_3)), 1.61 – 1.53 (m, 2H, CH_2CH_2C), 1.40 – 1.23 (m, 6H, CH_2), 0.89 (t, *J* = 6.8 Hz, 3H, CH_3); **^{13}C NMR** (101 MHz, $CDCl_3$) δ 175.4, 146.6, 128.3, 126.9, 124.4, 116.0, 94.2, 81.1, 31.7, 29.1, 29.0, 28.6, 26.9, 25.2, 22.7, 20.8, 14.2; **IR** (ν_{max}, cm^{-1}) 2979 (m), 2957 (m), 2929 (m), 2863 (m), 2858 (m), 1802 (w), 1726 (s), 1496 (m), 1449 (m), 1382 (m), 1270 (m), 1191 (s), 1136 (s), 1102 (s), 1076 (m), 1031 (m), 967 (m), 763 (s); **HRMS** (ESI/QTOF) m/z: $[M + Na]^+$ Calcd for $C_{19}H_{26}NaO_2^+$ 309.1825; Found 309.1829.

2.1.6. Synthesis and characterization of benzyl 2-hexylcycloprop-2-ene-1-carboxylate (s-1f)

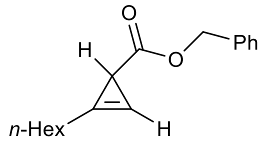

s-1f

Following **GPA**, oct-1-yne (3.31 g, 30.0 mmol, 1.50 equiv.), benzyl 2-diazoacetate (90% w/w in CH_2Cl_2, 3.92 g, 20.0 mmol, 1.00 equiv.), $Rh_2(OAc)_4$ (44.2 mg, 100 µmol, 0.5 mol%) and CH_2Cl_2 (30 mL) were used. Column chromatography on silica gel (eluent: pentane/ethyl acetate = 50:1) afforded **s-1f** in 62% yield (3.21 g, 12.4 mmol) as a colourless oil. **TLC**: R_f (*n*-hexane: EtOAc = 40:1) = 0.19; **^{1}H NMR** (400 MHz, $CDCl_3$) δ 7.41 – 7.28 (m, 5H, Ar*H*), 6.34 (q, *J* = 1.5 Hz, 1H, C=C*H*), 5.20 – 5.05 (m, 2H, OCH_2Ph), 2.49 (td, *J* = 7.3, 1.4 Hz, 2H, CH_2CH_2C), 2.19 (d, *J* = 1.6 Hz, 1H, C*H*CO_2), 1.66 – 1.46 (m, 2H, CH_2CH_2C), 1.42 – 1.18 (m, 6H, CH_2), 0.88 (t, *J* = 6.8 Hz, 3H, CH_3); **^{13}C NMR** (101 MHz, $CDCl_3$) δ 176.6, 136.6, 128.6, 128.2, 128.1, 115.7, 94.0, 66.1, 31.6, 28.9, 26.7, 25.1, 22.7, 19.9, 14.2. The NMR spectroscopic data is consistent with previous report[4].

2.1.7. Synthesis and characterization of adamantan-1-yl 2-hexylcycloprop-2-ene-1-carboxylate (s-1g)

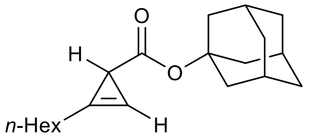

s-1g

Following **GPA**, oct-1-yne (331 mg, 3.00 mmol, 1.50 equiv.), adamantan-1-yl 2-diazoacetate[7] (459 mg, 2.00 mmol, 1.00 equiv.), $Rh_2(OAc)_4$ (4.40 mg, 10.0 µmol, 0.5 mol%) and CH_2Cl_2 (10 mL) were used. Column chromatography on silica gel (eluent: pentane/ethyl acetate = 50:1) afforded **s-1g** in 70% yield (421 mg, 1.39 mmol) as a colourless oil. **TLC**: R_f (*n*-hexane: EtOAc = 40:1) = 0.22; **^{1}H NMR** (400 MHz, $CDCl_3$) δ 6.28 (q, *J* = 1.4 Hz, 1H, C=C*H*), 2.46 (tt, *J* = 7.2, 1.6 Hz, 2H, CH_2CH_2C), 2.17 – 2.11 (m, 3H,

C*H*(adamantyl)), 2.11 – 2.07 (m, 6H, C*H*$_2$(adamantyl)), 2.01 (d, *J* = 1.6 Hz, 1H, C*H*CO$_2$), 1.69 – 1.60 (m, 6H, C*H*$_2$(adamantyl)), 1.59 – 1.52 (m, 2H, C*H*$_2$CH$_2$C), 1.40 – 1.23 (m, 6H, C*H*$_2$), 0.88 (t, *J* = 6.8 Hz, 3H, C*H*$_3$); **^{13}C NMR** (101 MHz, CDCl$_3$) δ 175.9, 116.1, 94.3, 79.8, 41.6, 36.4, 31.6, 31.0, 28.9, 26.9, 25.1, 22.7, 20.9, 14.2; **IR** (ν_{max}, cm^{-1}) 2954 (w), 2912 (m), 2855 (m), 1800 (w), 1712 (m), 1456 (m), 1346 (m), 1256 (m), 1181 (s), 1103 (w), 1056 (s), 971 (m), 734 (s); **HRMS** (ESI/QTOF) m/z: [M + Na]$^+$ Calcd for $C_{20}H_{30}NaO_2^+$ 325.2138; Found 325.2136.

2.1.8. Synthesis and characterization of (*E*)-3-(4-methoxyphenyl)allyl 2-hexylcycloprop-2-ene-1-carboxylate (s-1h)

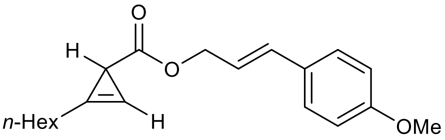

s-1h

Following **GPA**, oct-1-yne (260 mg, 2.36 mmol, 1.50 equiv.), (*E*)-3-(4-methoxyphenyl)allyl 2-diazoacetate[5] (366 mg, 1.57 mmol, 1.00 equiv.), Rh$_2$(OAc)$_4$ (3.50 mg, 7.85 μmol, 0.5 mol%) and CH$_2$Cl$_2$ (10 mL) were used. Column chromatography on silica gel (eluent: pentane/ethyl acetate = 10:1) afforded **s-1h** in 65% yield (321 mg, 1.02 mmol) as a colourless oil. **TLC**: R$_f$ (*n*-hexane: EtOAc = 5:1) = 0.49; **^{1}H NMR** (400 MHz, CDCl$_3$) δ 7.37 – 7.29 (m, 2H, Ar*H*), 6.92 – 6.78 (m, 2H, Ar*H*), 6.68 – 6.50 (m, 1H, C=C*H*), 6.34 (q, *J* = 1.4 Hz, 1H, CH$_2$C*H*=CH), 6.16 (dt, *J* = 15.8, 6.6 Hz, 1H, CH$_2$CH=C*H*), 4.71 (ddd, *J* = 6.7, 3.4, 1.3 Hz, 2H, C*H*$_2$CH=CH), 3.81 (s, 3H, OC*H*$_3$), 2.50 (td, *J* = 7.4, 1.4 Hz, 2H, CH$_2$C*H*$_2$C), 2.17 (d, *J* = 1.6 Hz, 1H, C*H*CO$_2$), 1.63 – 1.53 (m, 2H, C*H*$_2$CH$_2$C), 1.43 – 1.19 (m, 6H, C*H*$_2$), 0.87 (t, *J* = 6.8 Hz, 3H, C*H*$_3$); **^{13}C NMR** (101 MHz, CDCl$_3$) δ 176.6, 159.6, 133.7, 129.3, 128.0, 121.5, 115.7, 114.1, 94.0, 65.3, 55.4, 31.6, 28.9, 26.8, 25.1, 22.7, 19.9, 14.2; **IR** (ν_{max}, cm^{-1}) 3144 (w), 2957 (m), 2930 (m), 2859 (m), 1721 (m), 1607 (m), 1512 (s), 1464 (m), 1378 (w), 1337 (w), 1305 (w), 1248 (s), 1169 (s), 1032 (m), 966 (m), 836 (m), 808 (w), 719 (w); **HRMS** (ESI/QTOF) m/z: [M + Na]$^+$ Calcd for $C_{20}H_{26}NaO_3^+$ 337.1774; Found 337.1788.

2.1.9. Synthesis and characterization of ethyl 2-hexyl-1-methylcycloprop-2-ene-1-carboxylate (s-1i)

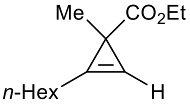

s-1i

Following **GPA**, oct-1-yne (579 mg, 5.25 mmol, 1.50 equiv.), ethyl diazoalaninate (448 mg, 3.50 mmol, 1.00 equiv.), Rh$_2$(OAc)$_4$ (7.74 mg, 17.5 μmol, 0.5 mol%) and CH$_2$Cl$_2$ (15 mL) were used. Column chromatography on silica gel (eluent: pentane/ethyl acetate = 50:1) afforded **s-1i** in 42% yield (313 mg, 1.49 mmol) as a colourless oil. **TLC**: R$_f$ (*n*-hexane: EtOAc = 20:1) = 0.34; **^{1}H NMR** (400 MHz, CDCl$_3$) δ 6.38 (td, *J* = 1.4, 0.7 Hz, 1H, C=C*H*), 4.15 – 3.97 (m, 2H, OC*H*$_2$CH$_3$), 2.44 (td, *J* = 7.3, 1.4 Hz, 2H, CH$_2$C*H*$_2$C), 1.59 – 1.47 (m, 2H, C*H*$_2$CH$_2$C), 1.42 – 1.23 (m, 9H, C*H*$_2$ & CC*H*$_3$), 1.20 (t, *J* = 7.1 Hz, 3H, OCH$_2$C*H*$_3$), 0.97 – 0.77 (m, 3H, C*H*$_3$); **^{13}C NMR** (101 MHz, CDCl$_3$) δ 177.8, 121.5, 100.0, 60.3, 31.6, 29.0, 27.0, 24.5, 24.3, 22.6, 20.8, 14.5, 14.1. The NMR spectroscopic data is consistent with previous report[6].

2.1.10. Synthesis and characterization of (3-(3-(trifluoromethyl)cycloprop-1-en-1-yl)propyl)benzene (s-1j)

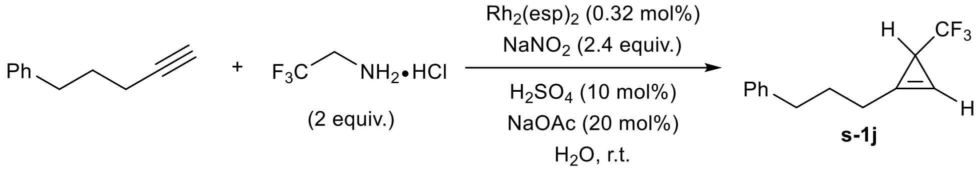

Following a reported procedure[7]: An oven-dried 100 mL Schlenk tube was sequentially charged with a magnetic stir-bar, $Rh_2(esp)_2$ (24.2 mg, 31.7 μmol, 0.32 mol%) and NaOAc (164 mg, 2.00 mmol, 20 mol%). The Schlenk tube was then evacuated and backfilled with nitrogen three times. Subsequently, distilled water (36 mL) was added under nitrogen. To this stirring mixture was added trifluoroethylamine hydrochloride (2.71 g, 20.0 mmol, 2.00 equiv.), H_2SO_4 (53.6 μL, 1.00 mmol, 10 mol%) and pent-4-yn-1-ylbenzene (1.44 g, 10.0 mmol, 1.0 equiv.) sequentially. Then, aqueous $NaNO_2$ (1.66 g, 24.0 mmol, 2.40 equiv.; dissolved in 20 mL of water) was added by syringe pump over 10 hours. After additional 4 hours, CH_2Cl_2 and water were added, and the water layer was extracted with CH_2Cl_2 (3 × 20 mL). The combined organic portions were dried with Na_2SO_4 and evaporated under reduced pressure. The resulting crude residue was purified by column chromatography on silica gel (eluent: pentane) to afford **s-1j** in 34% yield (774 mg, 3.42 mmol) as a colourless oil. **TLC**: R_f (*n*-hexane: EtOAc = 40:1) = 0.47; **^{1}H NMR** (400 MHz, $CDCl_3$) δ 7.35 – 7.27 (m, 2H, Ar*H*), 7.25 – 7.15 (m, 3H, Ar*H*), 6.43 (hept, *J* = 1.6 Hz, 1H, C=C*H*), 2.70 (dd, *J* = 8.4, 6.8 Hz, 2H, CH_2CH_2C), 2.53 (td, *J* = 7.3, 1.3 Hz, 2H, CH_2CH_2C), 2.01 – 1.92 (m, 3H, C*H*CF_3 & CH_2); **^{13}C NMR** (101 MHz, $CDCl_3$) δ 141.6, 128.6, 128.6, 126.6 (q, *J* = 275.6 Hz), 126.2, 116.7 (q, *J* = 2.7 Hz), 95.0 (q, *J* = 3.3 Hz), 35.3, 28.4, 24.5, 19.2 (q, *J* = 39.3 Hz); **^{19}F NMR** (377 MHz, $CDCl_3$) δ -67.0; **IR** (ν_{max}, cm^{-1}) 3151 (w), 3029 (w), 2944 (w), 2864 (w), 1497 (w), 1455 (w), 1366 (w), 1275 (s), 1120 (s), 953 (w), 829 (m), 745 (m); **HRMS** (APPI/LTQ-Orbitrap) m/z: $[M]^+$ Calcd for $C_{13}H_{13}F_3^+$ 226.0964; Found 226.0967.

2.1.11. Synthesis and characterization of dimethyl 2-phenylcycloprop-2-ene-1,1-dicarboxylate (s-1k)

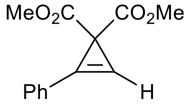

s-1k

Following **GPA**, phenylacetylene (0.98 mL, 8.91 mmol, 1.50 equiv.), dimethyl diazomalonate (940 mg, 5.94 mmol, 1.00 equiv.), $Rh_2(OAc)_4$ (13.1 mg, 29.7 μmol, 0.5 mol%) and CH_2Cl_2 (10 mL) were used. Column chromatography on silica gel (eluent: pentane/ethyl acetate = 5:1) afforded **s-1k** in 62% yield (850 mg, 3.66 mmol) as a colourless solid. **TLC**: R_f (*n*-hexane: EtOAc = 5:1) = 0.24; **^{1}H NMR** (400 MHz, $CDCl_3$) δ 7.64 – 7.61 (m, 2H, Ar*H*), 7.54 – 7.30 (m, 3H, Ar*H*), 6.89 (s, 1H, C=C*H*), 3.73 (s, 6H, OCH_3); **^{13}C NMR** (101 MHz, $CDCl_3$) δ 171.3, 130.7, 130.5, 129.0, 124.1, 112.4, 95.4, 52.5, 32.9. The NMR spectroscopic data is consistent with previous report[8].

2.1.12. Synthesis and characterization of dimethyl 2-(4-fluorophenyl)cycloprop-2-ene-1,1-dicarboxylate (s-1l)

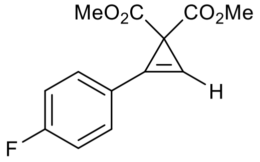

s-1l

Following **GPA**, 1-ethynyl-4-fluorobenzene (961 mg, 8.00 mmol, 1.00 equiv.), dimethyl diazomalonate (1.27 g, 8.00 mmol, 1.00 equiv.), $Rh_2(OAc)_4$ (17.7 mg, 40.0 µmol, 0.5 mol%) and CH_2Cl_2 (8.0 mL) were used. Column chromatography on silica gel (eluent: pentane/ethyl acetate = 4:1) afforded **s-1l** in 48% yield (961 mg, 3.84 mmol) as a colourless solid. **TLC**: R_f (*n*-hexane: EtOAc = 4:1) = 0.25; **^{1}H NMR** (400 MHz, $CDCl_3$) δ 7.68 – 7.57 (m, 2H, Ar*H*), 7.21 – 7.07 (m, 2H, Ar*H*), 6.86 (s, 1H, C=C*H*), 3.73 (s, 6H, OC*H*$_3$); **^{13}C NMR** (101 MHz, $CDCl_3$) δ 171.2, 165.3, 162.8, 132.6 (d, *J* = 8.8 Hz), 120.4 (d, *J* = 3.3 Hz), 116.4 (d, *J* = 22.3 Hz), 111.5, 95.0 (d, *J* = 2.6 Hz), 52.6, 33.0; **^{19}F NMR** (376 MHz, $CDCl_3$) δ -108.1. The NMR spectroscopic data is consistent with previous report[9].

2.1.13. Synthesis and characterization of dimethyl 2-(4-bromophenyl)cycloprop-2-ene-1,1-dicarboxylate (s-1m)

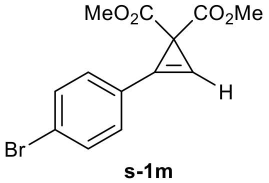

s-1m

Following **GPA**, starting from dimethyl 2-diazomalonate (395 mg, 2.50 mmol, 1.00 equiv.) and 1-bromo-4-ethynylbenzene (1.36 g, 7.50 mmol, 3.00 equiv.), the product **s-1m** was obtained after purification by column chromatography (SiO_2, pentane: EtOAc = 95:5 to 85:15) as a pale-yellow oil (554 mg, 1.78 mmol, 71% yield). **TLC**: R_f (*n*-hexane: EtOAc = 4:1) = 0.28; **^{1}H NMR** (400 MHz, $CDCl_3$) δ 7.62 – 7.56 (m, 2H, Ar*H*), 7.51 – 7.46 (m, 2H, Ar*H*), 6.94 (s, 1H, C=C*H*), 3.73 (s, 6H, CO_2C*H*$_3$); **^{13}C NMR** (101 MHz, $CDCl_3$) δ 171.0, 132.4, 131.9, 125.4, 123.1, 111.7, 96.4, 52.7, 33.0. The NMR spectroscopic data is consistent with previous report[7].

2.1.14. Synthesis and characterization of dibenzyl 2-phenylcycloprop-2-ene-1,1-dicarboxylate (s-1n)

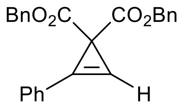

s-1n

Following **GPA**, phenylacetylene (613 mg, 6.00 mmol, 1.50 equiv.), dibenzyl 2-diazomalonate (1.24 g, 4.00 mmol, 1.00 equiv.), $Rh_2(OAc)_4$ (8.80 mg, 20.0 µmol, 0.5 mol%) and CH_2Cl_2 (20 mL) were used. Column chromatography on silica gel (eluent: pentane/ethyl acetate = 5:1) afforded **s-1n** in 48% yield (734 mg, 1.91 mmol) as a colourless oil. **TLC**: R_f (*n*-hexane: EtOAc = 5:1) = 0.32; **^{1}H NMR** (400 MHz, $CDCl_3$) δ 7.64 – 7.55 (m, 2H, Ar*H*), 7.40 (dd, *J* = 5.0, 2.0 Hz, 3H, Ar*H*), 7.31 – 7.19 (m, 10H, Ar*H*), 6.89 (s, 1H, C=C*H*), 5.16 (s, 4H, OC*H*$_2$Ph); **^{13}C NMR** (101 MHz, $CDCl_3$) δ 170.7, 135.9, 130.7, 130.5, 129.0, 128.6, 128.2, 128.0, 124.0, 112.5, 95.3, 67.0, 33.5. The NMR spectroscopic data is consistent with previous report[7].

2.1.15. Synthesis and characterization of dimethyl 2-hexylcycloprop-2-ene-1,1-dicarboxylate (s-1o)

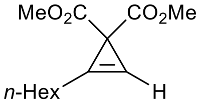

s-1o

Following **GPA**, oct-1-yne (1.1 mL, 7.50 mmol, 1.50 equiv.), dimethyl 2-diazomalonate (791 mg, 5.00 mmol, 1.00 equiv.), $Rh_2(OAc)_4$ (4.40 mg, 10.0 µmol, 0.2 mol%) and CH_2Cl_2 (10 mL) were used. Column chromatography on silica gel (eluent: pentane/ethyl acetate = 4:1) afforded **s-1o** in 69% yield (832 mg,

3.46 mmol) as a colourless oil. **TLC**: R_f (*n*-hexane: EtOAc = 4:1) = 0.28; **^{1}H NMR** (400 MHz, $CDCl_3$) δ 6.35 (t, *J* = 1.5 Hz, 1H, C=C*H*), 3.71 (s, 6H, CO_2CH_3), 2.54 (td, *J* = 7.4, 1.5 Hz, 2H, CH_2CH_2C), 1.66 – 1.51 (m, 2H, CH_2CH_2C), 1.46 – 1.18 (m, 6H, CH_2), 1.02 – 0.75 (m, 3H, CH_3). The NMR spectroscopic data is consistent with previous report[10].

2.2. Synthesis of hypervalent iodine precursor

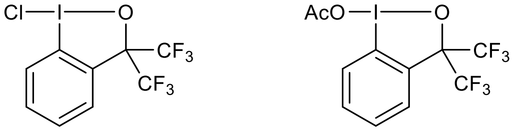

I1 **I2**

2.2.1. Synthesis and characterization of 1-chloro-3,3-bis(trifluoromethyl)-1,3-dihydro-1λ^3-benzo[*d*][1,2]iodaoxole (I1)[11]

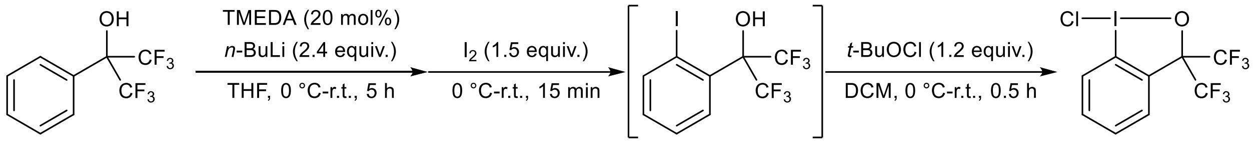

Under nitrogen, TMEDA (1.2 mL, 8.00 mmol, 20.0 mol%) was added to a solution of *n*-BuLi (2.5 M in hexane, 38.4 mL, 96 mmol, 2.40 equiv.). After 15 min, the cloudy solution was cooled to 0 °C and 1,1,1,3,3,3-hexafluoro-2-phenylpropan-2-ol (9.77 g, 40.0 mmol, 1.00 equiv.) in THF (10 mL) was added dropwise. The reaction was stirred at 0 °C for 30 min and then at room temperature for 5 hours. I_2 (15.2 g, 60.0 mmol, 1.50 equiv.) was then added in several portions at 0 °C and the mixture was stirred at 0 °C for 5 min and room temperature for 10 min. The reaction was quenched with saturated NH_4Cl (sat. aq., 10 mL). Et_2O (20 mL) was added and the layers were separated. The aqueous layer was then extracted with Et_2O (10 mL × 2). The organic layers were combined, washed twice with sodium bisulfite solution ($NaHSO_3$, ⩾37% in water; 10 mL), dried over Na_2SO_4, and filtered. The resulting solvent was evaporated under the reduced pressure to afford 1,1,1,3,3,3-hexafluoro-2-(2-iodophenyl)propan-2-ol (**4**) as an brown oil which was used without further purification. The crude product was dissolved in CH_2Cl_2 (10 mL) under air. *t*-BuOCl (5.21 g, 48.0 mmol, 1.20 equiv.) was then added dropwise at 0 °C. The resulting suspension was stirred under room temperature for 30 min. Then, the reaction mixture was filtered and washed with CH_2Cl_2 (10 mL) and pentane (10 mL) to afford 1-chloro-3,3-bis(trifluoromethyl)-1,3-dihydro-1λ^3-benzo[*d*][1,2]iodaoxole (**I1**) in 58% yield (9.38 g, 23.2 mmol) as a yellow solid. **^{1}H NMR** (400 MHz, Acetone-d_6) δ 8.17 (dd, *J* = 8.4, 1.1 Hz, 1H), 8.08 – 8.04 (m, 1H), 7.96 – 7.92 (m, 1H), 7.86 – 7.83 (m, 1H); **^{13}C NMR** (101 MHz, Acetone-d_6) δ 135.2, 133.1, 132.9, 130.5 (m), 130.0, 124.1 (q, *J* = 288.7 Hz), 114.1, 86.2 (m); **^{19}F NMR** (377 MHz, Acetone-d_6) δ -76.5. The NMR spectroscopic data is consistent with previous report[12].

2.2.2. Synthesis and characterization of 3,3-bis(trifluoromethyl)-1λ^3-benzo[*d*][1,2]iodaoxol-1(3*H*)-yl acetate (I2)[13]

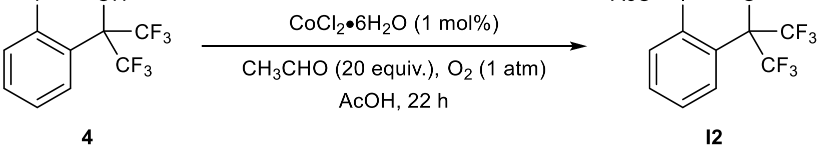

An oven-dried Schlenk tube was charged with a magnetic stir-bar and $CoCl_2{\cdot}6H_2O$ (32.5 mg, 137 μmol, 1.00 mol%). The Schlenk tube was then evacuated and backfilled with oxygen for three times. After that, 1,1,1,3,3,3-hexafluoro-2-(2-iodophenyl)propan-2-ol (**4**) (5.05 g, 13.7 mmol, 1.00 equiv. dissolved

in 68 mL glacial AcOH) and acetaldehyde (137 mmol, 7.7 mL, 10.0 equiv.) were added by syringe. The reaction mixture was stirred under 1 atm O_2, delivered by inflated balloon at 21 °C for 12 hours. After that, additional acetaldehyde (137 mmol, 7.7 mL, 10.0 equiv.) was added by syringe. The reaction mixture was stirred under 1 atm O_2 and 21 °C for additional 10 hours. The solvent was removed in vacuo and the residue was dissolved in CH_2Cl_2 (20 mL). The organic layer was washed with distilled water (20 mL) and extracted with CH_2Cl_2 (3 × 10 mL). The organic layer was dried over $MgSO_4$ and solvent was removed in vacuo to afford the oily product. Pentane (150 mL) was added gradually to the flask containing the product, which caused a precipitation. Filtration of the resulting suspension afforded 3,3-bis(trifluoromethyl)-1λ^3-benzo[*d*][1,2]iodaoxol-1(3*H*)-yl acetate (**I2**) as a white solid in 56% yield (3.28 g, 7.67 mmol). **^{1}H NMR** (400 MHz, $CDCl_3$) δ 7.94 – 7.92 (m, 1H), 7.86 – 7.45 (m, 3H), 2.18 (s, 3H); **^{13}C NMR** (101 MHz, $CDCl_3$) δ 176.7, 133.6, 131.7, 131.2, 130.4, 129.8 (m), 123.2 (q, *J* = 289.3 Hz), 115.9, 85.8 (m), 20.6; **^{19}F NMR** (377 MHz, $CDCl_3$) δ -75.9. The NMR spectroscopic data is consistent with previous report[14].

2.3. Synthesis of σ-type cyclopropenium cations transfer reagents CpBXs

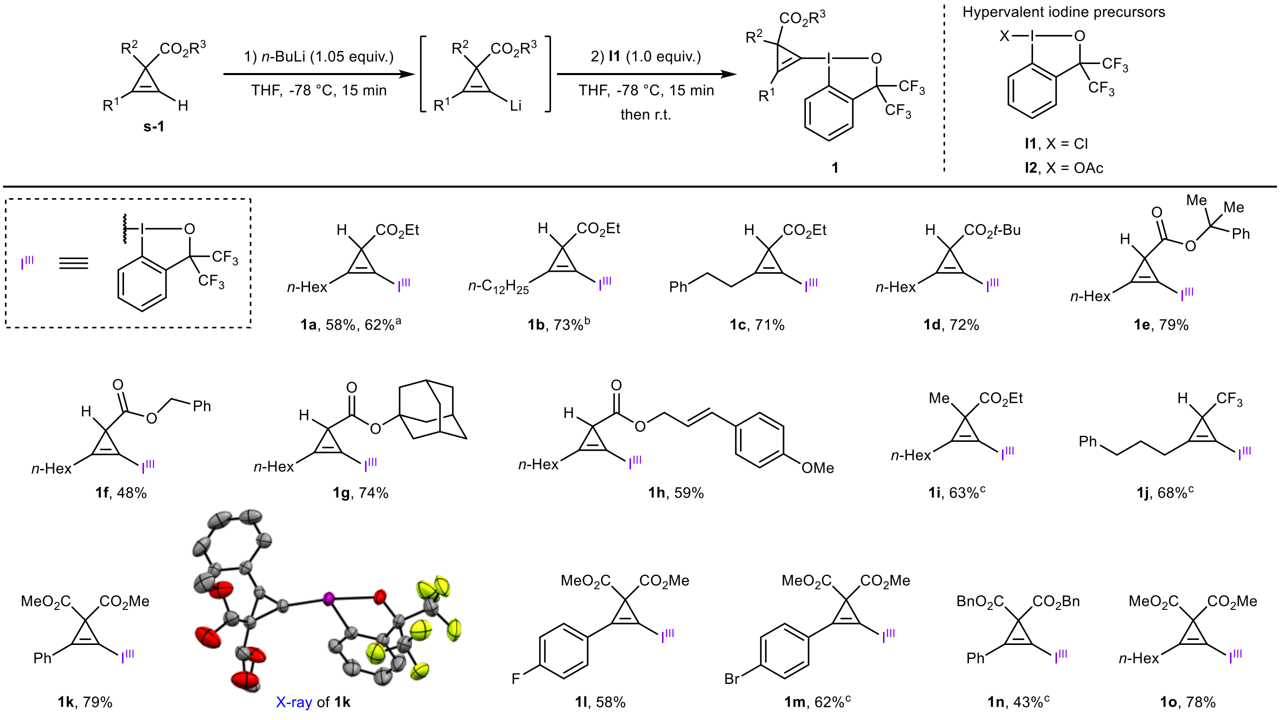

Reaction conditions: (1) Terminal cyclopropenes (1.0 equiv.), *n*-butyllithium (1.05 equiv.), THF (0.1 M), -78 °C for 15 min; (2) **I1** (1.0 equiv.), −78 °C for 15 min, then warm up to room temperature. [a]**I2** was used instead of **I1**. [b]**I1** (1.1 equiv.) was used. [c]N_2(l)/ethanol bath (−116 °C) and THF/Et_2O = 1:1 as the mixed solvents were used instead of THF.

General Procedure B (**GPB**): Synthesis of σ-type cyclopropenium cation transfer reagents (CpBXs). **GPB** was applied for the synthesis of **1a**, **1b**, **1c**, **1d**, **1e**, **1f**, **1g**, **1h**, **1k**, **1l** and **1o**.

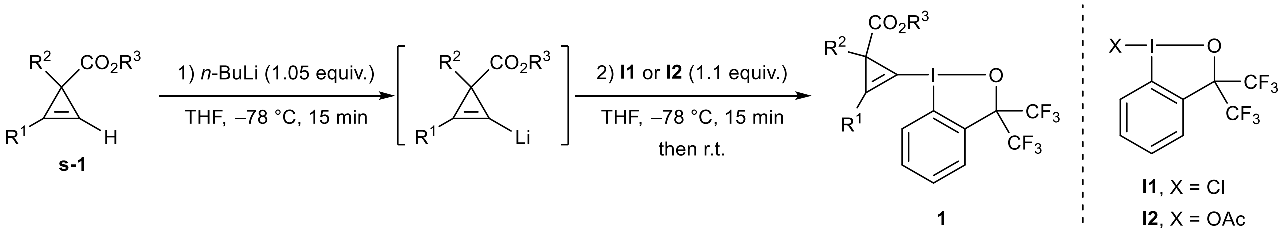

GPB: An oven-dried Schlenk tube was charged with a magnetic stir-bar and terminal cyclopropene **s-1** (1.00 equiv.). The Schlenk tube was then evacuated and backfilled with nitrogen three times. After that, THF (typically, 0.10 M) was added by syringe and the Schlenk tube was placed at −78 °C in a dry ice/acetone bath. *n*-Butyllithium (2.5 M in hexane; 1.05 equiv.) was added dropwise by a syringe pump

over 5 min and the reaction mixture was stirred at −78 °C for additional 10 min. Then, hypervalent iodine precursor **I1** or **I2** (1.10 equiv.) was added in one portion under nitrogen. The reaction mixture was stirred at −78 °C for 15 min, then the cooling bath was removed. The reaction mixture was allowed to warm to room temperature gradually (typically, ca. 15 min) while keeping stirring. The reaction mixture was then quenched by adding saturated aqueous $NaHCO_3$ (10 ml/mmol). The organic phase was removed, and the remaining aqueous portion was extracted with EtOAc. The combined organic portions were dried over Na_2SO_4, filtered and the volatiles removed under reduced pressure. The crude product was purified via flash chromatography on silica gel, and the fractions that contained the product were collected and concentrated by rotary evaporation to afford the purified compound **1**.

General Procedure C (**GPC**): Synthesis of σ-type cyclopropenium cation transfer reagents (CpBXs). **GPC** was applied for the synthesis of **1i**, **1j**, **1m** and **1n**.

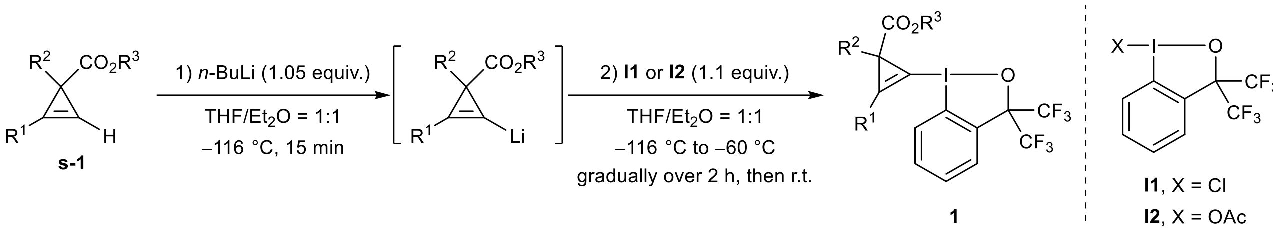

GPC: An oven-dried Schlenk tube was charged with a magnetic stir-bar and terminal cyclopropene **s-1** (1.00 equiv.). The Schlenk tube was then evacuated and backfilled with nitrogen three times. After that, THF/Et_2O = 1:1 was added by syringe and the Schlenk tube was placed at −116 °C in a liquid nitrogen/ethanol bath. *n*-Butyllithium (2.5 M in hexane; 1.05 equiv.) was added dropwise by a syringe pump over 5 min and the reaction mixture was stirred at −116 °C for additional 10 min. Then, hypervalent iodine precursor **I1** or **I2** (1.1 equiv.) was added in one portion under nitrogen. The reaction mixture was stirred in the cooling bath without adding more liquid nitrogen for additional 2 hours, thus resulting a gradual warm-up of the stirring mixture to ca. −60 °C. Then, the cooling bath was removed. The reaction mixture was allowed to warm further to room temperature naturally (typically, ca. 15 min) while keeping stirring. The reaction mixture was then quenched by adding saturated aqueous $NaHCO_3$ (10 ml/mmol). The organic phase was removed, and the remaining aqueous portion was extracted with EtOAc (3 × 10 mL). The combined organic portions were dried over Na_2SO_4, filtered and the volatiles removed under reduced pressure. The crude product was purified via flash chromatography on silica gel, and the fractions that contained the product were collected and concentrated by rotary evaporation to afford the purified compound **1**.

2.3.1. Synthesis and characterization of ethyl 2-(3,3-bis(trifluoromethyl)-1λ^3-benzo[*d*][1,2]iodaoxol-1(3*H*)-yl)-3-hexylcycloprop-2-ene-1-carboxylate (1a)

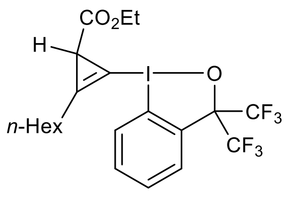

1a

Following **GPB**, **s-1a** (1.96 g, 10.0 mmol, 1.00 equiv.), *n*-butyllithium (2.5 M in hexane; 4.0 mL, 10.0 mmol, 1.00 equiv.), **I1** (4.05 g, 10.0 mmol, 1.00 equiv.) and THF (30 mL) were used. Column chromatography on silica gel (eluent: pentane/ethyl acetate = 8:1) afforded **1a** in 58% yield (3.30 g, 5.85 mmol) as a colourless oil, which turned to be solidified when stored in the freezer.

In a second reaction, **s-1a** (1.96 g, 10.0 mmol, 1.00 equiv.), *n*-butyllithium (2.5 M in hexane; 4.2 mL, 10.5 mmol, 1.05 equiv.), **I2** (4.28 g, 10.0 mmol, 1.00 equiv.) and THF (30 mL) were used, which afforded **1a** in 62% yield (3.49 g, 6.18 mmol).

M.p. 54 – 55 °C. **TLC**: R_f (*n*-hexane: EtOAc = 5:1) = 0.28; **^{1}H NMR** (400 MHz, $CDCl_3$) δ 7.84 – 7.81 (m, 1H, Ar*H*), 7.71 (dd, *J* = 8.2, 1.2 Hz, 1H, Ar*H*), 7.64 (td, *J* = 7.4, 1.2 Hz, 1H, Ar*H*), 7.57 (ddd, *J* = 8.6, 7.1, 1.6 Hz, 1H, Ar*H*), 4.17 (q, *J* = 7.1 Hz, 2H, OCH_2CH_3), 2.70 (t, *J* = 7.3 Hz, 2H, CH_2CH_2C), 2.64 (s, 1H, $CHCO_2$), 1.73 – 1.57 (m, 2H, CH_2CH_2C), 1.44 – 1.32 (m, 2H, CH_2), 1.29 – 1.23 (m, 7H, CH_2 & OCH_2CH_3), 0.94 – 0.76 (m, 3H, CH_3); **^{13}C NMR** (101 MHz, $CDCl_3$) δ 174.6, 133.8, 132.8, 131.1, 130.9, 130.3 (hept, *J* = 2.5 Hz), 129.4, 123.8 (q, *J* = 291.6 Hz), 111.7, 81.1 (hept, *J* = 29.2 Hz), 80.4, 61.1, 31.5, 29.0, 27.0, 26.3, 26.1, 22.6, 14.4, 14.0; **^{19}F NMR** (376 MHz, $CDCl_3$) δ -76.1 (m); **IR** (ν_{max}, cm^{-1}) 2960 (w), 2932 (w), 2861 (w), 1809 (w), 1717 (m), 1565 (w), 1465 (w), 1441 (w), 1370 (w), 1337 (w), 1264 (m), 1178 (s), 1150 (s), 1021 (w), 965 (m), 949 (s), 761 (m), 754 (m), 730 (s); **HRMS** (ESI/QTOF) m/z: $[M + H]^+$ Calcd for $C_{21}H_{24}F_6IO_3^+$ 565.0669; Found 565.0686.

2.3.2. Synthesis and characterization of ethyl 2-(3,3-bis(trifluoromethyl)-1λ³-benzo[*d*][1,2]iodaoxol-1(3*H*)-yl)-3-dodecylcycloprop-2-ene-1-carboxylate (1b)

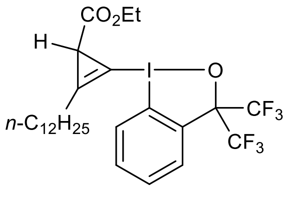

1b

Following **GPB**, **s-1b** (1.35 g, 4.83 mmol, 1.00 equiv.), *n*-butyllithium (2.5 M in hexane; 2.0 mL, 5.07 mmol, 1.05 equiv.), **I1** (2.15 g, 5.31 mmol, 1.10 equiv.) and THF (20 mL) were used. Column chromatography on silica gel (eluent: pentane/ethyl acetate = 8:1) afforded **1b** in 73% yield (2.27 g, 3.51 mmol) as a colourless oil, which turned to be solidified when stored in the freezer. **M.p.** 39 – 40 °C. **TLC**: R_f (*n*-hexane: EtOAc = 3:1) = 0.55; **^{1}H NMR** (400 MHz, $CDCl_3$) δ 7.84 (dq, *J* = 7.8, 1.5 Hz, 1H, Ar*H*), 7.72 (dd, *J* = 8.2, 1.2 Hz, 1H, Ar*H*), 7.65 (td, *J* = 7.4, 1.2 Hz, 1H, Ar*H*), 7.58 (ddd, *J* = 8.5, 7.1, 1.6 Hz, 1H, Ar*H*), 4.18 (q, *J* = 7.1 Hz, 2H, OCH_2CH_3), 2.71 (t, *J* = 7.3 Hz, 2H, CH_2CH_2C), 2.64 (s, 1H, $CHCO_2$), 1.72 – 1.58 (m, 2H, CH_2CH_2C), 1.43 – 1.33 (m, 2H, CH_2), 1.33 – 1.14 (m, 19H, CH_2 & OCH_2CH_3), 0.87 (t, *J* = 6.8 Hz, 3H, CH_3); **^{13}C NMR** (101 MHz, $CDCl_3$) δ 174.6, 133.8, 132.9, 131.1, 130.9, 130.4 (m), 129.4, 123.8 (q, *J* = 290.8 Hz), 111.7, 81.2 (hept, *J* = 29.3 Hz), 80.5, 61.2, 32.0, 29.73 (2C), 29.69, 29.6, 29.5, 29.4, 29.3, 27.0, 26.4, 26.2, 22.8, 14.5, 14.2; **^{19}F NMR** (376 MHz, $CDCl_3$) δ -76.1 (m); **IR** (ν_{max}, cm^{-1}) 2928 (m), 2855 (m), 1811 (w), 1721 (m), 1565 (w), 1465 (w), 1442 (w), 1371 (w), 1335 (w), 1264 (s), 1180 (s), 1149 (s), 1020 (w), 964 (m), 950 (s), 867 (w), 757 (m), 727 (m); **HRMS** (ESI/QTOF) m/z: $[M + Na]^+$ Calcd for $C_{27}H_{35}F_6INaO_3^+$ 671.1427; Found 671.1435.

2.3.3. Synthesis and characterization of ethyl 2-(3,3-bis(trifluoromethyl)-1λ³-benzo[*d*][1,2]iodaoxol-1(3*H*)-yl)-3-phenethylcycloprop-2-ene-1-carboxylate (1c)

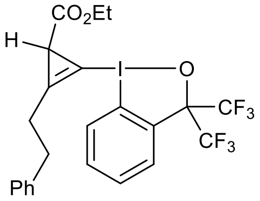

1c

Following **GPB**, **s-1c** (865 mg, 4.00 mmol, 1.00 equiv.), *n*-butyllithium (2.5 M in hexane; 1.6 mL, 4.00 mmol, 1.00 equiv.), **I1** (1.62 g, 4.00 mmol, 1.00 equiv.) and THF (24 mL) were used. Column

chromatography on silica gel (eluent: pentane/ethyl acetate = 8:1) afforded **1c** in 71% yield (1.66 g, 2.84 mmol) as a colourless oil. **TLC**: R_f (*n*-hexane: EtOAc = 4:1) = 0.26; **^{1}H NMR** (400 MHz, $CDCl_3$) δ 7.83 (d, *J* = 7.6 Hz, 1H, Ar*H*), 7.71 – 7.58 (m, 1H, Ar*H*), 7.58 – 7.44 (m, 2H, Ar*H*), 7.36 – 7.12 (m, 5H, Ar*H*), 4.19 (q, *J* = 7.1 Hz, 2H, OCH_2CH_3), 3.19 – 3.08 (m, 2H, CH_2), 3.08 – 2.95 (m, 2H, CH_2), 2.62 (s, 1H, $CHCO_2$), 1.30 (t, *J* = 7.2 Hz, 3H, OCH_2CH_3); **^{13}C NMR** (101 MHz, $CDCl_3$) δ 174.5, 139.5, 132.8, 132.5, 130.9, 130.7, 130.2 (m), 129.3, 128.9, 128.3, 126.9, 123.8 (q, *J* = 290.8 Hz), 111.6, 81.6, 81.1 (hept, *J* = 29.4 Hz), 61.2, 32.1, 27.3, 26.9, 14.4; **^{19}F NMR** (376 MHz, $CDCl_3$) δ -76.0; **IR** (ν_{max}, cm^{-1}) 3066 (w), 3030 (w), 2982 (w), 2933 (w), 1810 (w), 1715 (m), 1440 (w), 1264 (m), 1178 (s), 1149 (s), 1133 (s), 1021 (m), 964 (m), 949 (s), 866 (w), 795 (w), 753 (m), 730 (s); **HRMS** (ESI/QTOF) m/z: $[M + H]^+$ Calcd for $C_{23}H_{20}F_6IO_3^+$ 585.0356; Found 585.0351.

2.3.4. Synthesis and characterization of *tert*-butyl 2-(3,3-bis(trifluoromethyl)-1λ^3-benzo[*d*][1,2]iodaoxol-1(3*H*)-yl)-3-hexylcycloprop-2-ene-1-carboxylate (1d)

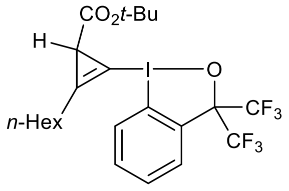

1d

Following **GPB**, **s-1d** (329 mg, 1.47 mmol, 1.00 equiv.), *n*-butyllithium (2.5 M in hexane; 0.62 mL, 1.54 mmol, 1.05 equiv.), **I1** (593 mg, 1.47 mmol, 1.00 equiv.) and THF (15 mL) were used. Column chromatography on silica gel (eluent: pentane/ethyl acetate = 8:1) afforded **1d** in 72% yield (627 mg, 1.06 mmol) as a colourless oil. **TLC**: R_f (*n*-hexane: EtOAc = 4:1) = 0.43; **^{1}H NMR** (400 MHz, $CDCl_3$) δ 7.84 (dq, *J* = 7.7, 1.4 Hz, 1H, Ar*H*), 7.71 – 7.60 (m, 2H, Ar*H*), 7.56 (ddd, *J* = 8.5, 7.1, 1.6 Hz, 1H, Ar*H*), 2.70 (td, *J* = 7.2, 1.4 Hz, 2H, CH_2CH_2C), 2.56 (s, 1H, $CHCO_2$), 1.68 – 1.61 (m, 2H, CH_2CH_2C), 1.46 (s, 9H, $C(CH_3)_3$), 1.43 – 1.34 (m, 2H, CH_2), 1.33 – 1.24 (m, 4H, CH_2), 0.93 – 0.79 (m, 3H, CH_3); **^{13}C NMR** (101 MHz, $CDCl_3$) δ 173.8, 134.5, 132.8, 131.1, 130.9, 130.4 (m), 129.5, 123.9 (q, *J* = 290.4 Hz), 111.7, 81.3, 81.1 (hept, *J* = 29.2 Hz), 81.0, 31.5, 29.1, 28.28, 28.25, 26.5, 26.2, 22.6, 14.1; **^{19}F NMR** (376 MHz, $CDCl_3$) δ -76.1 (m); **IR** (ν_{max}, cm^{-1}) 2961 (w), 2934 (w), 2861 (w), 1807 (w), 1713 (m), 1462 (w), 1369 (w), 1265 (m), 1179 (s), 1148 (s), 965 (m), 950 (s), 762 (m); **HRMS** (ESI/QTOF) m/z: $[M + Na]^+$ Calcd for $C_{23}H_{27}F_6INaO_3^+$ 615.0801; Found 615.0810.

2.3.5. Synthesis and characterization of 2-phenylpropan-2-yl 2-(3,3-bis(trifluoromethyl)-1λ^3-benzo[*d*][1,2]iodaoxol-1(3*H*)-yl)-3-hexylcycloprop-2-ene-1-carboxylate (1e)

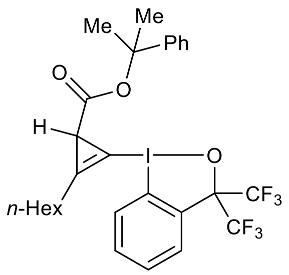

1e

Following **GPB**, **s-1e** (411 mg, 1.44 mmol, 1.00 equiv.), *n*-butyllithium (2.5 M in hexane; 0.60 mL, 1.51 mmol, 1.05 equiv.), **I1** (581 mg, 1.44 mmol, 1.00 equiv.) and THF (15 mL) were used. Column chromatography on silica gel (eluent: pentane/ethyl acetate = 8:1) afforded **1d** in 79% yield (744 mg, 1.14 mmol) as a colourless oil. **TLC**: R_f (*n*-hexane: EtOAc = 10:1) = 0.13; **^{1}H NMR** (400 MHz, $CDCl_3$) δ 7.86 (dq, *J* = 7.8, 1.4 Hz, 1H, Ar*H*), 7.69 – 7.55 (m, 2H, Ar*H*), 7.43 (ddd, *J* = 8.5, 7.1, 1.5 Hz, 1H, Ar*H*), 7.39 – 7.31 (m, 2H, Ar*H*), 7.33 – 7.25 (m, 2H, Ar*H*), 7.27 – 7.16 (m, 1H, Ar*H*), 2.73 – 2.69 (m, 2H, CH_2CH_2C),

2.68 (s, 1H, C*H*CO_2), 1.82 (s, 3H, C(C*H*$_3$)), 1.81 (s, 3H, C(C*H*$_3$)), 1.73 – 1.58 (m, 2H, C*H*$_2$CH_2C), 1.47 – 1.35 (m, 2H, C*H*$_2$), 1.32 – 1.38 (m, 4H, C*H*$_2$), 0.95 – 0.80 (m, 3H, C*H*$_3$); ^{13}**C NMR** (101 MHz, $CDCl_3$) δ 173.2, 145.7, 134.1, 133.0, 131.0, 130.8, 130.3 (m), 129.5, 128.4, 127.2, 124.3, 123.9 (q, *J* = 290.8 Hz), 111.5, 82.5, 81.1 (hept, *J* = 29.3 Hz), 80.7, 31.5, 29.1, 29.0, 28.6, 28.0, 26.5, 26.2, 22.6, 14.1; ^{19}**F NMR** (376 MHz, $CDCl_3$) δ -76.1 (m); **IR** (ν_{max}, cm^{-1}) 3069 (w), 2933 (w), 2863 (w), 1808 (w), 1718 (m), 1465 (w), 1265 (s), 1179 (s), 1151 (s), 1133 (s), 1102 (m), 965 (s), 946 (s), 762 (m), 730 (s); **HRMS** (ESI/QTOF) m/z: [M + Na]$^+$ Calcd for $C_{28}H_{29}F_6INaO_3^+$ 677.0958; Found 677.0975.

2.3.6. Synthesis and characterization of benzyl 2-(3,3-bis(trifluoromethyl)-1λ³-benzo[*d*][1,2]iodaoxol-1(3*H*)-yl)-3-hexylcycloprop-2-ene-1-carboxylate (1f)

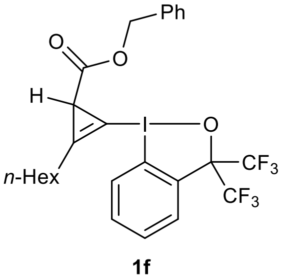

1f

Following **GPB**, **s-1f** (517 mg, 2.00 mmol, 1.00 equiv.), *n*-butyllithium (2.5 M in hexane; 0.84 mL, 2.10 mmol, 1.05 equiv.), **I1** (809 mg, 2.00 mmol, 1.00 equiv.) and THF (20 mL) were used. Column chromatography on silica gel (eluent: pentane/ethyl acetate = 8:1) afforded **1f** in 48% yield (599 mg, 956 μmol) as a colourless oil. **TLC**: R_f (*n*-hexane: EtOAc = 4:1) = 0.38; ^{1}**H NMR** (400 MHz, $CDCl_3$) δ 7.84 (dq, *J* = 7.8, 1.4 Hz, 1H, Ar*H*), 7.66 (dd, *J* = 8.3, 1.0 Hz, 1H, Ar*H*), 7.63 (td, *J* = 7.5, 1.1 Hz, 1H, Ar*H*), 7.45 (ddd, *J* = 8.5, 7.2, 1.5 Hz, 1H, Ar*H*), 7.39 – 7.30 (m, 5H, Ar*H*), 5.16 (d, *J* = 2.5 Hz, 2H, OC*H*$_2$Ph), 2.71 (t, *J* = 7.3 Hz, 2H, CH_2C*H*$_2$C), 2.70 (s, 1H, C*H*CO_2), 1.69 – 1.57 (m, 2H, C*H*$_2$CH_2C), 1.42 – 1.32 (m, 2H, C*H*$_2$), 1.31 – 1.20 (m, 4H, C*H*$_2$), 0.95 – 0.78 (m, 3H, C*H*$_3$); ^{13}**C NMR** (101 MHz, $CDCl_3$) δ 174.4, 135.8, 133.6, 132.9, 131.1, 130.8, 130.4 (m), 129.3, 128.8, 128.6, 128.5, 123.8 (q, *J* = 290.7 Hz), 111.7, 81.2 (hept, *J* = 29.1 Hz), 80.4, 67.1, 31.5, 29.1, 27.0, 26.4, 26.2, 22.6, 14.1; ^{19}**F NMR** (376 MHz, $CDCl_3$) δ -76.1 (m); **IR** (ν_{max}, cm^{-1}) 2957 (w), 2932 (w), 2859 (w), 1811 (w), 1720 (m), 1565 (w), 1464 (w), 1440 (w), 1379 (w), 1340 (w), 1265 (m), 1215 (m), 1179 (s), 1150 (s), 1003 (w), 965 (m), 949 (s), 755 (m), 730 (s); **HRMS** (ESI/QTOF) m/z: [M + Na]$^+$ Calcd for $C_{26}H_{25}F_6INaO_3^+$ 649.0645; Found 649.0657.

2.3.7. Synthesis and characterization of adamantan-1-yl 2-(3,3-bis(trifluoromethyl)-1λ³-benzo[*d*][1,2]iodaoxol-1(3*H*)-yl)-3-hexylcycloprop-2-ene-1-carboxylate (1g)

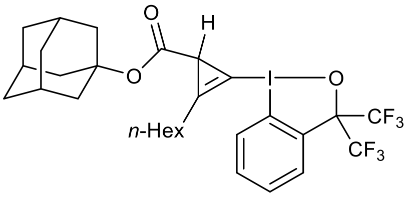

1g

Following **GPB**, **s-1g** (367 mg, 1.21 mmol, 1.0 equiv.), *n*-butyllithium (2.5 M in hexane; 1.3 mL, 1.27 mmol, 1.05 equiv.), **I1** (489 mg, 1.21 mmol, 1.00 equiv.) and THF (20 mL) were used. Column chromatography on silica gel (eluent: pentane/ethyl acetate = 8:1) afforded **1g** in 74% yield (598 mg, 892 μmol) as a colourless oil, which turned to be solidified when stored in the freezer. **M.p.** 73 – 75 °C. **TLC**: R_f (*n*-hexane: EtOAc = 4:1) = 0.50; ^{1}**H NMR** (400 MHz, $CDCl_3$) δ 7.86 – 7.76 (m, 1H, Ar*H*), 7.66 (dd, *J* = 8.0, 1.2 Hz, 1H, Ar*H*), 7.61 (td, *J* = 7.4, 1.2 Hz, 1H, Ar*H*), 7.54 (ddd, *J* = 8.4, 7.1, 1.5 Hz, 1H, Ar*H*), 2.67 (td, *J* = 7.2, 2.3 Hz, 2H, CH_2C*H*$_2$C), 2.53 (s, 1H, C*H*CO_2), 2.17 – 2.10 (m, 3H, C*H*(adamantyl)), 2.10 – 2.04 (m, 6H, C*H*$_2$(adamantyl)), 1.73 – 1.52 (m, 8H, C*H*$_2$ & C*H*$_2$(adamantyl)), 1.46 – 1.31 (m, 2H, C*H*$_2$), 1.26 (dt,

J = 7.5, 3.8 Hz, 4H, CH_2), 0.89 – 0.76 (m, 3H, CH_3); **^{13}C NMR** (101 MHz, $CDCl_3$) δ 173.4, 134.5, 132.8, 131.1, 130.9, 130.4, 129.5, 123.9 (q, J = 290.8 Hz), 111.7, 81.4, 81.06, 81.14 (hept, J = 29.3 Hz), 41.6, 36.3, 31.5, 31.0, 29.1, 28.3, 26.5, 26.2, 22.6, 14.1; **^{19}F NMR** (376 MHz, $CDCl_3$) δ -76.1 (m); **IR** (ν_{max}, cm^{-1}) 2917 (m), 2857 (w), 1810 (w), 1712 (m), 1457 (w), 1347 (w), 1264 (m), 1179 (s), 1151 (s), 1051 (m), 965 (s), 950 (s), 760 (m), 730 (s); **HRMS** (ESI/QTOF) m/z: [M + Na]$^+$ Calcd for $C_{29}H_{33}F_6INaO_3^+$ 693.1271; Found 693.1271.

2.3.8. Synthesis and characterization of (*E*)-3-(4-methoxyphenyl)allyl 2-(3,3-bis(trifluoromethyl)-1λ^3-benzo[*d*][1,2]iodaoxol-1(3*H*)-yl)-3-hexylcycloprop-2-ene-1-carboxylate (1h)

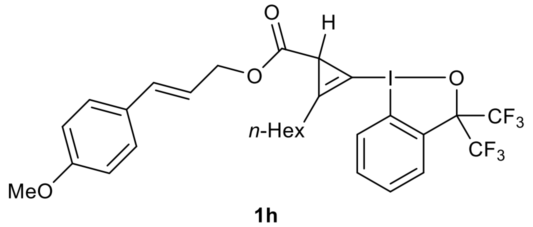

1h

Following **GPB**, **s-1h** (297 mg, 946 μmol, 1.00 equiv.), *n*-butyllithium (2.5 M in hexane; 0.40 mL, 993 μmol, 1.05 equiv.), **I1** (383 mg, 946 μmol, 1.00 equiv.) and THF (20 mL) were used. Column chromatography on silica gel (eluent: pentane/ethyl acetate = 5:1) afforded **1h** in 59% yield (382 mg, 559 μmol) as a colourless oil. **TLC**: R_f (*n*-hexane: EtOAc = 4:1) = 0.30; **^{1}H NMR** (400 MHz, $CDCl_3$) δ 7.87 – 7.79 (m, 1H, Ar*H*), 7.72 (dd, J = 8.1, 1.2 Hz, 1H, Ar*H*), 7.60 (td, J = 7.4, 1.2 Hz, 1H, Ar*H*), 7.53 (ddd, J = 8.5, 7.1, 1.6 Hz, 1H, Ar*H*), 7.36 – 7.27 (m, 2H, Ar*H*), 6.90 – 6.80 (m, 2H, Ar*H*), 6.60 (d, J = 15.8 Hz, 1H, CH_2CH=C*H*), 6.14 (dt, J = 15.8, 6.7 Hz, 1H, CH_2C*H*=CH), 4.76 (dd, J = 6.7, 1.3 Hz, 2H, CH_2CH=CH), 3.79 (s, 3H, OCH_3), 2.72 (t, J = 7.3 Hz, 2H, CH_2CH_2C), 2.69 (s, 1H, C*H*CO_2), 1.81 – 1.56 (m, 2H, C$H_2$$CH_2$C), 1.43 – 1.32 (m, 2H, CH_2), 1.32 – 1.20 (m, 4H, CH_2), 0.93 – 0.72 (m, 3H, CH_3); **^{13}C NMR** (101 MHz, $CDCl_3$) δ 174.4, 159.8, 134.5, 133.7, 132.9, 131.0, 130.8, 130.3, 129.4, 128.8, 128.0, 123.8 (q, J = 290.9 Hz), 120.6, 114.1, 111.6, 81.1 (hept, J = 29.4 Hz), 80.3, 66.0, 55.3, 31.4, 29.0, 27.0, 26.3, 26.1, 22.5, 14.0; **^{19}F NMR** (376 MHz, $CDCl_3$) δ -76.1 (m); **IR** (ν_{max}, cm^{-1}) 2956 (w), 2933 (w), 2860 (w), 1811 (w), 1717 (m), 1608 (w), 1512 (m), 1464 (w), 1441 (w), 1264 (m), 1179 (s), 1150 (s), 1035 (w), 965 (m), 943 (m), 739 (s); **HRMS** (ESI/QTOF) m/z: [M + Na]$^+$ Calcd for $C_{29}H_{33}F_6INaO_3^+$ 693.1271; Found 693.1271.

2.3.9. Synthesis and characterization of ethyl 2-(3,3-bis(trifluoromethyl)-1λ^3-benzo[*d*][1,2]iodaoxol-1(3*H*)-yl)-3-hexyl-1-methylcycloprop-2-ene-1-carboxylate (1i)

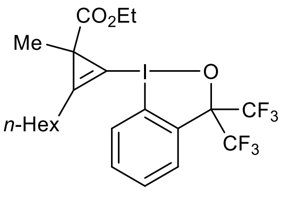

1i

Following **GPC**, **s-1i** (377 mg, 1.79 mmol, 1.00 equiv.), *n*-butyllithium (2.5 M in hexane; 0.75 mL, 1.88 mmol, 1.05 equiv.), **I1** (724 mg, 1.79 mmol, 1.00 equiv.), THF (10 mL) and Et_2O (10 mL) were used. Flash column chromatography on silica gel (eluent: pentane/ethyl acetate = 8:1) afforded **1i** in 63% yield (657 mg, 1.14 mmol) as a colourless oil. **TLC**: R_f (*n*-hexane: EtOAc = 5:1) = 0.35; **^{1}H NMR** (400 MHz, $CDCl_3$) δ 7.85 (dq, J = 7.7, 1.4 Hz, 1H, Ar*H*), 7.74 (dd, J = 8.2, 1.1 Hz, 1H, Ar*H*), 7.65 (ddd, J = 7.8, 7.2, 1.1 Hz, 1H, Ar*H*), 7.55 (ddd, J = 8.5, 7.2, 1.5 Hz, 1H, Ar*H*), 4.15 (q, J = 7.1 Hz, 2H, OC$H_2$$CH_3$), 2.67 (t, J = 7.3 Hz, 2H, CH_2CH_2C), 1.68 – 1.58 (m, 2H, C$H_2$$CH_2$C), 1.50 (s, 3H, C(C$H_3$)), 1.45 – 1.33 (m, 2H, CH_2), 1.33 – 1.20 (m, 7H, CH_2 & OCH_2CH_3), 0.95 – 0.79 (m, 3H, CH_3); **^{13}C NMR** (101 MHz, $CDCl_3$) δ 175.4, 140.9, 132.7, 131.13, 131.06, 130.4 (m), 129.5, 123.9 (q, J = 291.3 Hz), 111.4, 87.8, 81.0 (hept, J = 29.4 Hz),

61.4, 32.7, 31.5, 29.1, 26.6, 25.6, 22.6, 20.7, 14.5, 14.1; **^{19}F NMR** (376 MHz, $CDCl_3$) δ -76.1 (m); **IR** (ν_{max}, cm^{-1}) 3073 (w), 2959 (w), 2931 (w), 2861 (w), 1800 (w), 1714 (m), 1565 (w), 1465 (w), 1441 (w), 1380 (w), 1260 (s), 1215 (m), 1178 (s), 1151 (s), 1132 (m), 1116 (s), 1025 (w), 965 (m), 950 (s), 795 (w), 759 (m), 730 (s); **HRMS** (ESI/QTOF) m/z: $[M + H]^+$ Calcd for $C_{22}H_{26}F_6IO_3^+$ 579.0825; Found 579.0827.

2.3.10. Synthesis and characterization of 1-(2-(3-phenylpropyl)-3-(trifluoromethyl)cycloprop-1-en-1-yl)-3,3-bis(trifluoromethyl)-1,3-dihydro-1λ³-benzo[*d*][1,2]iodaoxole (1j)

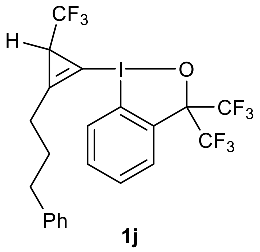

1j

Following **GPC**, **s-1j** (385 mg, 1.70 mmol, 1.00 equiv.), *n*-butyllithium (2.5 M in hexane; 0.71 mL, 1.79 mmol, 1.05 equiv.), **I1** (688mg, 1.70 mmol, 1.00 equiv.), THF (10 mL) and Et_2O (10 mL) were used. Flash column chromatography on silica gel (eluent: pentane/ethyl acetate = 10:1) afforded **1j** in 68% yield (682 mg, 1.15 mmol) as a colourless oil, which turned to be solidified when stored in the freezer. **M.p.** 102 – 104 °C. **TLC**: R_f (*n*-hexane: EtOAc = 5:1) = 0.37; **^{1}H NMR** (400 MHz, $CDCl_3$) δ 7.88 (dq, *J* = 7.8, 1.4 Hz, 1H, Ar*H*), 7.68 (td, *J* = 7.5, 1.1 Hz, 1H, Ar*H*), 7.57 (ddd, *J* = 8.5, 7.1, 1.5 Hz, 1H, Ar*H*), 7.39 (dd, *J* = 8.3, 1.0 Hz, 1H, Ar*H*), 7.35 – 7.27 (m, 2H, Ar*H*), 7.25 – 7.19 (m, 1H, Ar*H*), 7.19 – 7.12 (m, 2H, Ar*H*), 2.78 – 2.71 (m, 4H, CH_2), 2.46 (q, *J* = 4.5 Hz, 1H, C*H*(CF_3)), 2.03 (pent, *J* = 7.4 Hz, 2H, CH_2); **^{13}C NMR** (101 MHz, $CDCl_3$) δ 140.7, 132.94, 132.91, 131.3, 130.8, 130.7 (m), 128.8, 128.6, 128.4, 126.5, 125.7 (q, *J* = 275.5 Hz), 123.8 (q, *J* = 290.8 Hz), 111.5, 81.4 (d, *J* = 2.6 Hz), 81.3 (hept, *J* = 29.4 Hz), 35.4, 27.9, 26.4 (q, *J* = 39.6 Hz), 25.6; **^{19}F NMR** (376 MHz, $CDCl_3$) δ -66.5 (CHCF_3), -76.0 (m, C(CF_3)$_2$); **IR** (ν_{max}, cm^{-1}) 3030 (w), 2943 (w), 2864 (w), 1797 (w), 1605 (w), 1497 (w), 1364 (w), 1266 (s), 1217 (m), 1183 (s), 1150 (s), 1131 (s), 965 (s), 952 (s), 829 (w), 754 (m), 730 (s); **HRMS** (ESI/QTOF) m/z: $[M + H]^+$ Calcd for $C_{22}H_{17}F_9IO^+$ 595.0175; Found 595.0184.

2.3.11. Synthesis and characterization of dimethyl 2-(3,3-bis(trifluoromethyl)-1λ³-benzo[*d*][1,2]iodaoxol-1(3*H*)-yl)-3-phenylcycloprop-2-ene-1,1-dicarboxylate (1k)

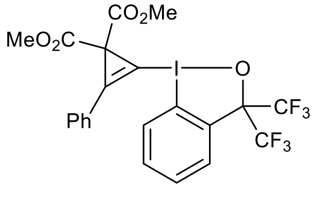

1k

Following **GPB**, **s-1k** (994 mg, 4.28 mmol, 1.00 equiv.), *n*-butyllithium (2.5 M in hexane; 1.8 mL, 4.49 mmol, 1.05 equiv.), **I1** (1.73 g, 4.28 mmol, 1.00 equiv.) and THF (40 mL) were used. Column chromatography on silica gel (eluent: pentane/ethyl acetate = 4:1) afforded **1k** in 79% yield (2.02 g, 3.37 mmol) as a colourless solid. **M.p.** 121 – 124 °C. **TLC**: R_f (*n*-hexane: EtOAc = 4:1) = 0.23; **^{1}H NMR** (400 MHz, $CDCl_3$) δ 7.92 – 7.86 (m, 1H, Ar*H*), 7.84 (dd, *J* = 8.4, 1.0 Hz, 1H, Ar*H*), 7.71 – 7.66 (m, 1H, Ar*H*), 7.66 – 7.61 (m, 2H, Ar*H*), 7.60 – 7.46 (m, 4H, Ar*H*), 3.79 (s, 6H, OCH_3); **^{13}C NMR** (101 MHz, $CDCl_3$) δ 170.2, 133.2, 132.3, 131.4, 130.9, 130.8, 130.5 (m), 129.5, 129.4, 126.8, 123.7 (q, *J* = 290.5 Hz), 123.4, 111.8, 81.4 (hept, *J* = 29.6 Hz), 80.1, 53.0, 37.7; **^{19}F NMR** (376 MHz, $CDCl_3$) δ -76.0; **IR** (ν_{max}, cm^{-1}) 3069 (w), 2956 (w), 2847 (w), 1923 (w), 1793 (w), 1729 (m), 1437 (m), 1265 (s), 1181 (s), 1147 (s), 1062

(m), 950 (s), 756 (s), 730 (s); **HRMS** (ESI/QTOF) m/z: [M + Na]$^+$ Calcd for $C_{22}H_{15}F_6INaO_5^+$ 622.9761; Found 622.9778.

2.3.12. Synthesis and characterization of dimethyl 2-(3,3-bis(trifluoromethyl)-1λ^3-benzo[*d*][1,2]iodaoxol-1(3*H*)-yl)-3-(4-fluorophenyl)cycloprop-2-ene-1,1-dicarboxylate (1l)

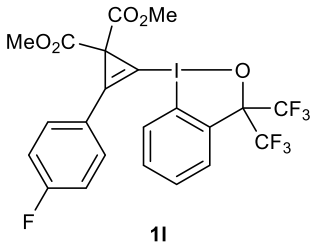

1l

Following **GPB**, **s-1l** (751 mg, 3.00 mmol, 1.00 equiv.), *n*-butyllithium (2.5 M in hexane; 1.3 mL, 3.15 mmol, 1.05 equiv.), **I1** (1.21 g, 3.00 mmol, 1.00 equiv.) and THF (15 mL) were used. Column chromatography on silica gel (eluent: pentane/ethyl acetate = 4:1) afforded **1l** in 58% yield (1.08 g, 1.75 mmol) as a colourless oil, which turned to be an amorphous solid when stored in the freezer. **TLC**: R_f (*n*-hexane: EtOAc = 4:1) = 0.17; **^{1}H NMR** (400 MHz, $CDCl_3$) δ 7.90 – 7.85 (m, 1H, Ar*H*), 7.80 (dd, *J* = 8.4, 1.0 Hz, 1H, Ar*H*), 7.72 – 7.60 (m, 3H, Ar*H*), 7.59 – 7.54 (m, 1H, Ar*H*), 7.23 – 7.15 (m, 2H, Ar*H*), 3.79 (s, 6H, OC*H*$_3$); **^{13}C NMR** (101 MHz, $CDCl_3$) δ 170.1, 165.0 (d, *J* = 255.7 Hz), 133.2, 133.1 (d, *J* = 9.2 Hz), 131.4, 130.9, 130.6 (m), 129.3, 125.9, 123.7 (q, *J* = 290.5 Hz), 119.8 (d, *J* = 3.3 Hz), 117.0 (d, *J* = 22.4 Hz), 111.7, 81.4 (hept, *J* = 29.7 Hz), 79.7 (d, *J* = 3.2 Hz), 53.1, 37.7; **^{19}F NMR** (376 MHz, $CDCl_3$) δ -75.9 (C(C*F*$_3$)$_2$), -104.7 (Ar*F*); **IR** (ν_{max}, cm^{-1}) 3076 (w), 2957 (w), 2849 (w), 1926 (w), 1794 (w), 1732 (s), 1602 (m), 1504 (m), 1465 (w), 1438 (m), 1263 (s), 1237 (s), 1183 (s), 1148 (s), 1065 (m), 1013 (w), 965 (m), 950 (s), 842 (m), 798 (w), 759 (m); **HRMS** (ESI/QTOF) m/z: [M + Na]$^+$ Calcd for $C_{22}H_{14}F_7INaO_5^+$ 640.9666; Found 640.9677.

2.3.13. Synthesis and characterization of dimethyl 2-(3,3-bis(trifluoromethyl)-1λ^3-benzo[*d*][1,2]iodaoxol-1(3*H*)-yl)-3-(4-bromophenyl)cycloprop-2-ene-1,1-dicarboxylate (1m)

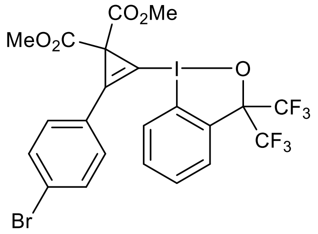

1m

Following **GPC**, **s-1m** (350 mg, 1.12 mmol, 1.00 equiv.), *n*-butyllithium (2.5 M in hexane; 0.47 mL, 1.18 mmol, 1.05 equiv.), **I1** (455mg, 1.12 mmol, 1.00 equiv.), THF (10 mL) and Et_2O (10 mL) were used. Flash column chromatography on silica gel (eluent: pentane/ethyl acetate = 5:1) afforded **1m** in 62% yield (475 mg, 699 µmol) as a colourless solid. **M.p.** 150 – 152 °C. **TLC**: R_f (*n*-hexane: EtOAc = 4:1) = 0.20; **^{1}H NMR** (400 MHz, $CDCl_3$) δ 7.88 (d, *J* = 7.7 Hz, 1H, Ar*H*), 7.78 (dd, *J* = 8.3, 1.1 Hz, 1H, Ar*H*), 7.74 – 7.61 (m, 3H, Ar*H*), 7.56 (ddd, *J* = 8.5, 7.1, 1.5 Hz, 1H, Ar*H*), 7.52 – 7.43 (m, 2H, Ar*H*), 3.79 (s, 6H, OC*H*$_3$); **^{13}C NMR** (101 MHz, $CDCl_3$) δ 170.0, 133.3, 132.9, 132.0, 131.5, 130.8, 130.6 (m), 129.3, 127.2, 125.9, 123.7 (q, *J* = 290.5 Hz), 122.3, 111.8, 81.4 (hept, *J* = 29.7 Hz), 81.3, 53.1, 37.6; **^{19}F NMR** (376 MHz, $CDCl_3$) δ -75.9; **IR** (ν_{max}, cm^{-1}) 2955 (w), 1797 (w), 1730 (m), 1585 (w), 1482 (m), 1464 (w), 1438 (m), 1398 (w), 1288 (s), 1267 (s), 1184 (s), 1150 (s), 1134 (m), 1068 (m), 1012 (m), 967 (m), 953 (m), 829 (m), 755 (m), 731 (m); **HRMS** (ESI/QTOF) m/z: [M + Na]$^+$ Calcd for $C_{22}H_{14}BrF_6INaO_5^+$ 700.8866; Found 700.8885.

2.3.14. Synthesis and characterization of dibenzyl 2-(3,3-bis(trifluoromethyl)-1λ³-benzo[*d*][1,2]iodaoxol-1(3*H*)-yl)-3-phenylcycloprop-2-ene-1,1-dicarboxylate (1n)

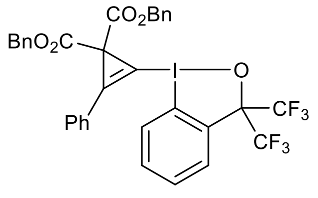

1n

Following **GPC**, **s-1n** (550 mg, 1.43 mmol, 1.00 equiv.), *n*-butyllithium (2.5 M in hexane; 0.60 mL, 1.50 mmol, 1.05 equiv.), **I1** (578 mg, 1.43 mmol, 1.00 equiv.), THF (20 mL) and Et_2O (20 mL) were used. Flash column chromatography on silica gel (eluent: pentane/ethyl acetate = 5:1) afforded **1n** in 43% yield (467 mg, 620 μmol) as a colourless oil. **TLC**: R_f (*n*-hexane: EtOAc = 4:1) = 0.33; **^{1}H NMR** (400 MHz, $CDCl_3$) δ 7.86 – 7.77 (m, 1H, Ar*H*), 7.68 (dd, *J* = 8.3, 0.9 Hz, 1H, Ar*H*), 7.59 – 7.52 (m, 3H, Ar*H*), 7.51 – 7.40 (m, 3H, Ar*H*), 7.30 – 7.12 (m, 11H, Ar*H*), 5.26 – 5.09 (m, 4H, OCH_2Ph); **^{13}C NMR** (101 MHz, $CDCl_3$) δ 169.6, 135.4, 133.2, 132.3, 131.3, 130.8, 130.6, 130.4 (m), 129.4, 129.3, 128.7, 128.5, 128.1, 126.9, 123.7 (q, *J* = 290.6 Hz), 123.3, 111.7, 81.4 (hept, *J* = 29.4 Hz), 80.0, 67.7, 38.1; **^{19}F NMR** (376 MHz, $CDCl_3$) δ -75.9; **IR** (ν_{max}, cm^{-1}) 3068 (w), 3034 (w), 2957 (w), 2891 (w), 2237 (w), 2139 (w), 1959 (w), 1736 (m), 1584 (w), 1498 (w), 1455 (w), 1257 (s), 1213 (s), 1193 (s), 1180 (s), 1147 (m), 1109 (m), 1015 (w), 963 (m), 946 (m), 926 (m), 755 (s), 729 (s); **HRMS** (ESI/QTOF) m/z: [M + Na]$^+$ Calcd for $C_{34}H_{23}F_6INaO_5^+$ 775.0387; Found 775.0394.

2.3.15. Synthesis and characterization of dimethyl 2-(3,3-bis(trifluoromethyl)-1λ³-benzo[*d*][1,2]iodaoxol-1(3*H*)-yl)-3-hexylcycloprop-2-ene-1,1-dicarboxylate (1o)

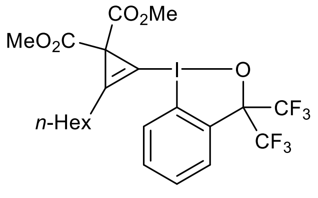

1o

Following **GPB**, **s-1o** (240 mg, 1.00 mmol, 1.00 equiv.), *n*-butyllithium (2.5 M in hexane; 0.42 mL, 1.05 mmol, 1.05 equiv.), **I1** (405 g, 1.00 mmol, 1.00 equiv.) and THF (10 mL) were used. Column chromatography on silica gel (eluent: pentane/ethyl acetate = 4:1) afforded **1o** in 78% yield (475 mg, 781 μmol) as a colourless oil. **TLC**: R_f (*n*-hexane: EtOAc = 4:1) = 0.27; **^{1}H NMR** (400 MHz, $CDCl_3$) δ 7.82 – 7.77 (m, 1H, Ar*H*), 7.75 (dd, *J* = 8.2, 1.1 Hz, 1H, Ar*H*), 7.62 (td, *J* = 7.4, 1.1 Hz, 1H, Ar*H*), 7.54 (ddd, *J* = 8.5, 7.2, 1.5 Hz, 1H, Ar*H*), 3.72 (s, 6H, OCH_3), 2.70 (t, *J* = 7.3 Hz, 2H, CH_2CH_2C), 1.61 (pent, *J* = 7.3 Hz, 2H, C$H_2$$CH_2$C), 1.44 – 1.28 (m, 2H, CH_2), 1.28 – 1.13 (m, 4H, CH_2), 0.83 – 0.77 (m, 3H, CH_3); **^{13}C NMR** (101 MHz, $CDCl_3$) δ 170.6, 132.9, 131.2, 131.0, 130.8, 130.2 (m), 129.5, 123.6 (q, *J* = 290.6 Hz), 111.4, 81.1 (hept, *J* = 29.5 Hz), 79.1, 52.7, 37.5, 31.3, 28.9, 26.0, 25.1, 22.4, 13.9; **^{19}F NMR** (376 MHz, $CDCl_3$) δ -76.0; **IR** (ν_{max}, cm^{-1}) 2956 (w), 2931 (w), 2859 (w), 1813 (w), 1726 (m), 1464 (w), 1437 (m), 1281 (m), 1265 (s), 1216 (m), 1181 (s), 1149 (s), 1134 (m), 1065 (m), 966 (m), 950 (s), 837 (w), 754 (m), 730 (s); **HRMS** (ESI/QTOF) m/z: [M + H]$^+$ Calcd for $C_{22}H_{24}F_6IO_5^+$ 609.0567; Found 609.0576.

2.4. Synthesis and characterization of ethyl 2-hexyl-3-iodocycloprop-2-ene-1-carboxylate (1a-1)

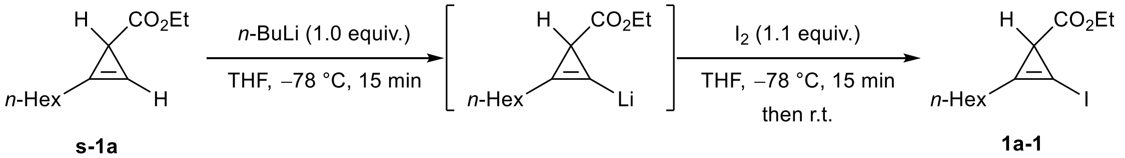

s-1a **1a-1**

An oven-dried Schlenk tube was charged with a magnetic stir-bar and terminal cyclopropene **s-1a** (589 mg, 3.00 mmol, 1.00 equiv.). The Schlenk tube was then evacuated and backfilled with nitrogen three times. After that, THF (30 mL) was added by syringe and the Schlenk tube was placed at −78 °C in a dry ice/acetone bath. *n*-Butyllithium (2.5 M in hexane; 1.2 mL, 3.00 mmol, 1.00 equiv.) was added dropwise by a syringe pump over 5 min and the reaction mixture was stirred at −78 °C for additional 10 min. Then, elemental iodine (838 mg, 3.30 mmol, 1.10 equiv.) was added in one portion under nitrogen. The reaction mixture was stirred at −78 °C for 15 min, then the cooling bath was removed. The reaction mixture was allowed to warm to room temperature gradually (ca. 15 min) while keeping stirring. The reaction mixture was then quenched by adding sodium bisulfite solution ($NaHSO_3$, ⩾37% in water; 10 mL). The organic phase was separated, and the remaining aqueous portion was extracted with EtOAc (3 × 10 mL). The combined organic portions were dried over Na_2SO_4, filtered and the volatiles removed under reduced pressure. The crude product was purified via flash chromatography on silica gel (eluent: pentane/ethyl acetate = 20:1), and the fractions that contained the product were collected and concentrated by rotary evaporation to afford the purified compound **1a-1** in 59% yield (575 mg, 1.78 mmol) as a colourless oil. **TLC**: R_f (*n*-hexane: EtOAc = 20:1) = 0.33; **^{1}H NMR** (400 MHz, $CDCl_3$) δ 4.14 (q, *J* = 7.1 Hz, 2H, OCH_2CH_3), 2.49 (t, *J* = 7.2 Hz, 2H, CH_2CH_2C), 2.45 (s, 1H, $CHCO_2$), 1.68 – 1.52 (m, 2H, CH_2CH_2C), 1.44 – 1.20 (m, 9H, CH_2 & OCH_2CH_3), 0.96 – 0.83 (m, 3H, CH_3); **^{13}C NMR** (101 MHz, $CDCl_3$) δ 174.7, 125.3, 60.7, 46.7, 31.6, 29.0, 26.7, 26.0, 25.1, 22.7, 14.5, 14.2; **IR** (ν_{max}, cm^{-1}) 2955 (m), 2929 (m), 2858 (w), 1720 (s), 1643 (w), 1607 (w), 1463 (m), 1391 (w), 1371 (m), 1320 (w), 1302 (w), 1255 (m), 1180 (s), 1131 (w), 1095 (w), 1026 (m), 891 (w), 865 (w), 799 (w), 724 (w); **HRMS** (ESI/QTOF) m/z: $[M + Na]^+$ Calcd for $C_{12}H_{19}INaO_2^+$ 345.0322; Found 345.0329.

3. Synthesis and characterisation of terminal alkynes and alkyne surrogates

3.1. Terminal alkynes and alkyne surrogates

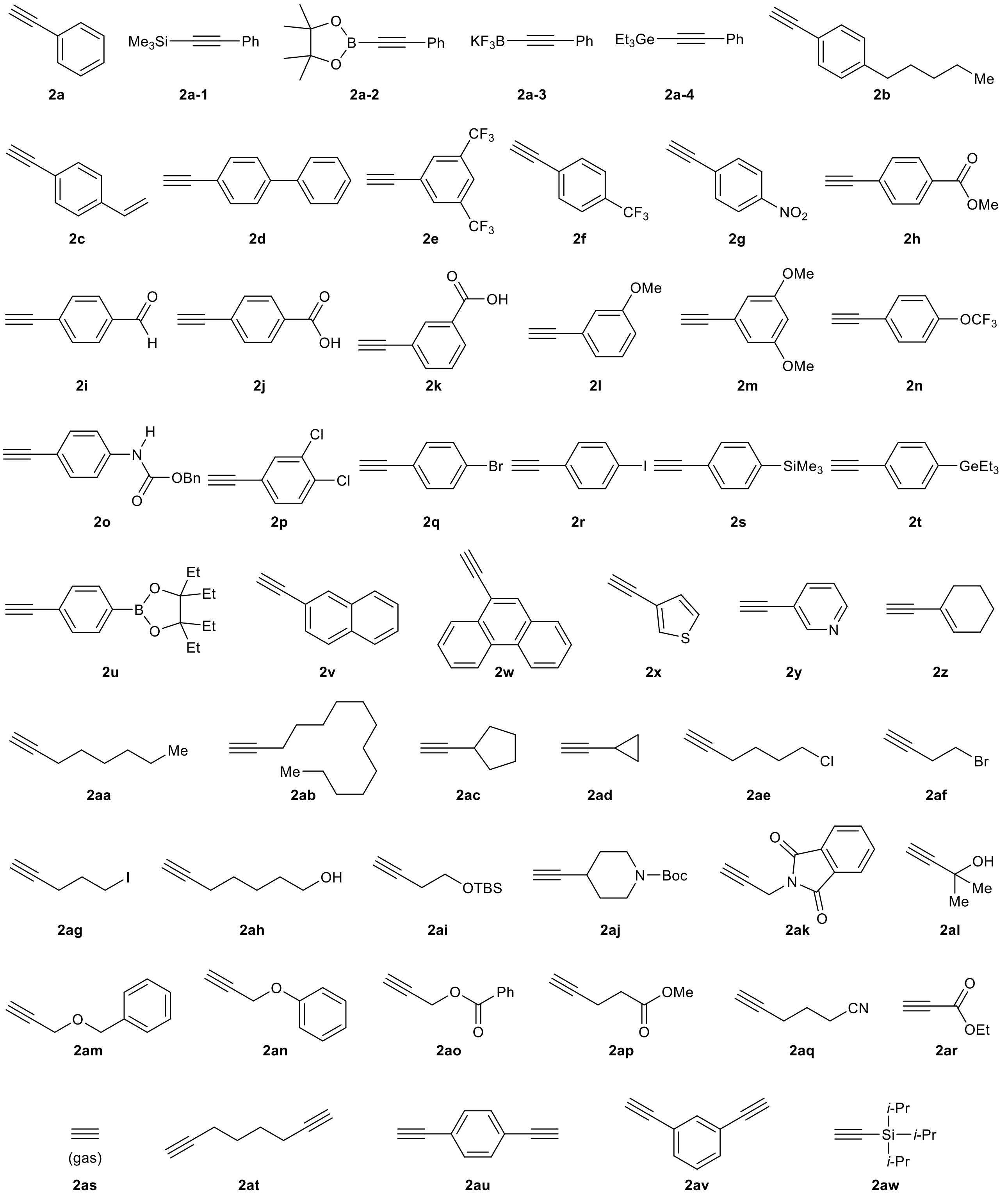

Terminal alkyne **2a**, **2b**, **2d**, **2e**, **2f**, **2g**, **2h**, **2i**, **2j**, **2k**, **2l**, **2m**, **2n**, **2p**, **2q**, **2v**, **2w**, **2x**, **2y**, **2z**, **2aa**, **2ab**, **2ac**, **2ad**, **2ae**, **2af**, **2ag**, **2ah**, **2ai**, **2aj**, **2ak**, **2al**, **2am**, **2an**, **2ao**, **2ap**, **2aq**, **2ar**, **2as**, **2at**, **2au**, **2av**, **2aw** and terminal alkyne surrogates **2a-1**, **2a-2** were commercially available and used as received.

3.1.1. Synthesis and characterization of potassium trifluoro(phenylethynyl)borate (2a-3)[15]

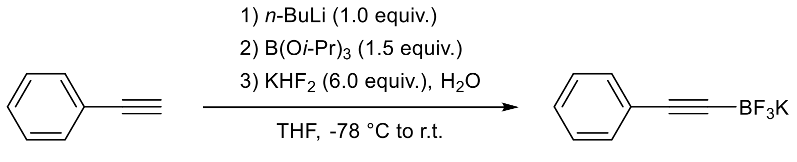

An oven-dried round-bottom flask, charged with a magnetic stir-bar was evacuated and backfilled with N_2 (3 times). Then, phenylacetylene **2a** (1.53 g, 15.0 mmol, 1.00 equiv.) and dry THF (50 mL) were added. The mixture was cooled to -78 °C and a solution of *n*-BuLi (2.5 M, 6.0 mL, 15.0 mmol, 1.00 equiv.) in hexane was added dropwise under N_2. The reaction was stirred at -78 °C for 1 hour and B(O*i*-Pr)$_3$ (4.23 g, 22.5 mmol, 1.50 equiv.) was added quickly. The reaction was stirred 10 min at -78 °C then 2 hours at room temperature. The mixture was cooled to 0 °C and a saturated solution of KHF_2 (7.03 g, 90.0 mmol, 6.00 equiv.) in water (20 mL + 20 mL to rinse the remaining solid) was added. The reaction was stirred at room temperature open to air for 2 hours then concentrated in vacuo. The wet solid obtained was further dried by co-evaporation with acetone. To the dry solid was added acetone (~50 mL) and the resulting mixture was placed on a rotary evaporator and rotated rapidly at atmospheric pressure with the bath set at 45 °C for 15 minutes. The flask was removed and the mixture carefully filtered taking care to leave the insoluble material in the reaction flask. Acetone was once again added and the process (heating for 15 min then collection of the liquid) was repeated 2 more times. The combined acetone filtrates were concentrated in vacuo to approximately 1/3 of the initial volume. Et_2O (~60 mL) was added causing a white solid to precipitate. The mixture was cooled to 0 °C for 10 min then filtered. The solid obtained was washed with Et_2O and dried in vacuo to afford the desired **2a-3** in 83% yield (2.60 g, 12.5 mmol) as a white solid. **^{1}H NMR** (400 MHz, acetone-d$_6$) δ 7.35 – 7.29 (m, 2H, Ar*H*), 7.27 – 7.17 (m, 3H, Ar*H*); **^{13}C NMR** (101 MHz, acetone-d$_6$) δ 132.1, 128.8, 127.4, 127.2; **^{19}F NMR** (376 MHz, acetone-d6) δ -135.0. The NMR spectroscopic data is consistent with previous report[16].

3.1.2. Synthesis and characterization of triethyl(phenylethynyl)germane (2a-4)[17]

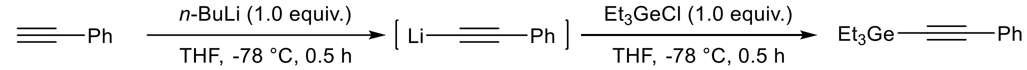

An oven-dried Schlenk tube was charged with a magnetic stir-bar. The Schlenk tube was then evacuated and backfilled with nitrogen three times. After that, ethynylbenzene **2a** (0.22 mL, 2.00 mmol, 1.00 equiv.) and THF (10 mL) was added by syringe and the Schlenk tube was placed at −78 °C in a dry ice/acetone bath. *n*-Butyllithium (2.5 M in hexane; 2.00 mmol, 0.80 mL, 1.00 equiv.) was added dropwise by a syringe pump over 5 min and the reaction mixture was stirred at −78 °C for additional 25 min. Then, Et_3GeCl (2.00 mmol, 391 mg, 1.00 equiv.) was added dropwise under nitrogen. The reaction mixture was stirred at −78 °C for 30 min, then the cooling bath was removed. The reaction mixture was allowed to warm to room temperature gradually while keeping stirring for additional 15 min. The reaction mixture was then quenched by adding saturated aqueous NH_4Cl (10 mL). The organic phase was removed, and the remaining aqueous portion was extracted with EtOAc (3 × 5 mL). The combined organic portions were dried over Na_2SO_4, filtered and the volatiles removed under reduced pressure. The crude product was purified via flash chromatography on silica gel (eluent : pentane). Terminal alkyne surrogate **2a-4** was obtained in 98% yield (509 mg, 1.95 mmol) as a colourless oil. **TLC**: R_f (*n*-hexane) = 0.54; **^{1}H NMR** (400 MHz, $CDCl_3$) δ 7.52 – 7.38 (m, 2H, Ar*H*), 7.34 – 7.17 (m, 3H, Ar*H*), 1.14 (t, *J* = 7.8 Hz, 9H, Ge(CH$_2$C*H*$_3$)$_3$), 0.93 (qd, *J* = 7.7, 1.1 Hz, 6H, Ge(C*H*$_2$CH$_3$)$_3$); **^{13}C NMR** (101 MHz, $CDCl_3$) δ 132.1, 128.3, 128.1, 123.9, 106.1, 92.2, 9.2, 6.0; **HRMS** (ESI/QTOF) m/z: [M +

Ag]$^+$ Calcd for $C_{14}H_{20}AgGe^+$ 368.9822; Found 368.9820. The spectroscopic data is consistent with previous report[18].

3.1.3. Synthesis and characterization of 1-ethynyl-4-vinylbenzene (2c)[19]

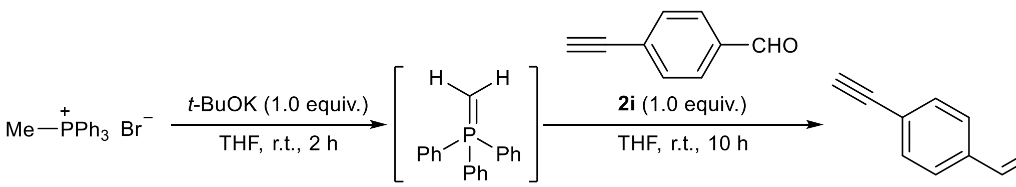

2c

In a nitrogen-filled glovebox, a 50 ml Schlenk tube was sequentially charged with a magnetic stir bar and *t*-BuOK (224 mg, 2.00 mmol, 1.00 equiv.). The tube was then tightly sealed with a rubber cap. The tube was brought out of the glovebox. After that, THF (10 mL) and methyltriphenylphosphonium bromide (714 mg, 2.00 mmol, 1.00 equiv.) was added sequentially. The reaction mixture was stirred under room temperature for 2 hours. Then 4-ethynylbenzaldehyde **2i** (260 mg, 2.00 mmol, 1.00 equiv.) was added in one portion. The reaction mixture was stirred under room temperature for additional 10 hours. The reaction mixture was then filtered through a silica gel pad and washed with EtOAc. The organic solution of crude product was washed with brine, dried over Na_2SO_4, filtered and concentrated under reduced pressure. The crude product was purified via flash chromatography on silica gel (eluent: pentane). Terminal alkyne **2c** was obtained in 65% yield (167 mg, 1.30 mmol) as a colorless oil. **TLC**: R_f (*n*-hexane) = 0.56; **^{1}H NMR** (400 MHz, $CDCl_3$) δ 7.49 – 7.42 (m, 2H, Ar*H*), 7.41 – 7.32 (m, 2H, Ar*H*), 6.70 (dd, *J* = 17.6, 10.9 Hz, 1H, C*H*=CH_2), 5.77 (dd, *J* = 17.6, 0.8 Hz, 1H, CH=CH_2), 5.30 (dd, *J* = 10.9, 0.8 Hz, 1H, CH=CH_2), 3.11 (s, 1H, C≡C*H*); **^{13}C NMR** (101 MHz, $CDCl_3$) δ 138.1, 136.3, 132.5, 126.3, 121.5, 115.2, 83.8, 77.9; **HRMS** (APPI/LTQ-Orbitrap) m/z: [M]$^+$ Calcd for $C_{10}H_8^+$ 128.0621; Found 128.0622. The spectroscopic data is consistent with previous report[20].

3.1.4. Synthesis and characterization of benzyl (4-ethynylphenyl)carbamate (2o)[21]

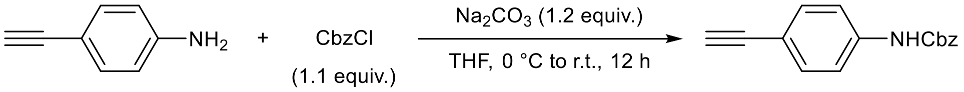

2o

An oven-dried Schlenk tube was charged with a magnetic stir-bar. The Schlenk tube was then evacuated and backfilled with nitrogen three times. After that, 4-ethynylaniline (234 mg, 2.00 mmol, 1.00 equiv.), Na_2CO_3 (254 mg, 2.40 mmol, 1.20 equiv.) and THF (10 mL) were added and the resulting mixture was stirred at 0 °C. Then, benzyl chloroformate (CbzCl; 2.20 mmol, 0.31 mL, 1.10 equiv.) was added dropwise by a syringe pump and the reaction mixture was stirred at room temperature for 12 hours. The reaction mixture was then quenched by adding saturated aqueous NH_4Cl (10 mL). The organic phase was collected, and the remaining aqueous portion was extracted with EtOAc (3 × 5 mL). The combined organic portions were dried over Na_2SO_4, filtered and the volatiles removed under reduced pressure. The crude product was purified via flash chromatography on silica gel (eluent: pentane/EtOAc = 10:1). Terminal alkyne **2o** was obtained in 99% yield (497 mg, 1.98 mmol) as a colourless oil. **TLC**: R_f (*n*-hexane: EtOAc = 10:1) = 0.24; **^{1}H NMR** (400 MHz, $CDCl_3$) δ 7.47 – 7.30 (m, 9H, Ar*H*), 6.82 (s, 1H, N*H*), 5.20 (s, 2H, CH_2Ph), 3.04 (s, 1H, C≡C*H*); **^{13}C NMR** (101 MHz, $CDCl_3$) δ 153.1, 138.4, 135.9, 133.2, 128.8, 128.6, 128.5, 118.3, 117.0, 83.5, 76.7, 67.3. The spectroscopic data is consistent with previous report[22].

3.1.5. Synthesis and characterization of 1-ethynyl-4-iodobenzene (2r)[23]

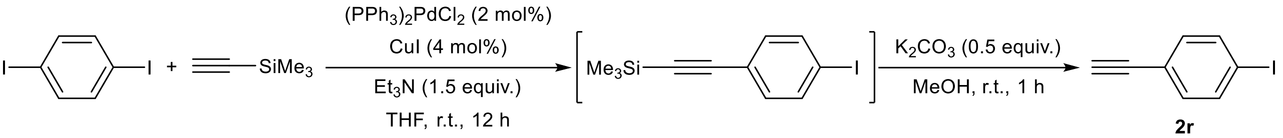

2r

An oven-dried Schlenk tube was charged with $(PPh_3)_2PdCl_2$ (56.2 mg, 80.0 µmol, 2.00 mol%), CuI (30.5 mg, 160 µmol, 4.00 mol%), 1,4-diiodobenzene (1.32 g, 4.00 mmol, 1.00 equiv.) and a magnetic stir-bar. The Schlenk tube was then evacuated and backfilled with nitrogen three times. After that, THF (10 mL) was added by syringe and the reaction mixture was stirred under room temperature. Then, ethynyltrimethylsilane (393 mg, 4.00 mmol, 1.00 equiv.) was added dropwise by a syringe and the reaction mixture was stirred at room temperature for additional 12 hours. The reaction mixture was then quenched by adding saturated aqueous NH_4Cl (10 mL). The organic phase was collected, and the remaining aqueous portion was extracted with EtOAc (3 × 5 mL). The combined organic portions were dried over Na_2SO_4, filtered and the volatiles removed under reduced pressure. The crude residue was dissolved by MeOH (10 mL) under air. Then, K_2CO_3 (276 mg, 2.00 mmol, 0.500 equiv.) was added. The reaction mixture was stirred under room temperature for 1 hour. The reaction mixture was then diluted by adding deionized water (20 mL) and EtOAc (20 mL). The organic phase was collected, and the remaining aqueous portion was extracted with EtOAc (3 × 5 mL). The combined organic portions were dried over Na_2SO_4, filtered and the volatiles removed under reduced pressure. The crude product was purified via flash chromatography on silica gel (eluent : pentane). Terminal alkyne **2r** was obtained in 31% yield (283 mg, 1.24 mmol) as a white solid. **TLC**: R_f (*n*-hexane) = 0.60;**^{1}H NMR** (400 MHz, $CDCl_3$) δ 7.76 – 7.57 (m, 2H, Ar*H*), 7.25 – 7.09 (m, 2H, Ar*H*), 3.13 (s, 1H, C≡C*H*); **^{13}C NMR** (101 MHz, $CDCl_3$) δ 137.6, 133.7, 121.7, 95.0, 82.8, 78.8. The spectroscopic data is consistent with previous report[24].

3.1.6. Synthesis and characterization of (4-ethynylphenyl)trimethylsilane (2s)[25]

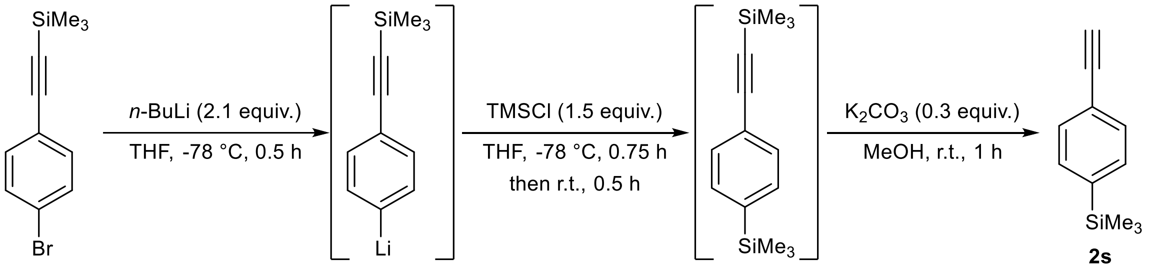

2s

An oven-dried Schlenk tube was charged with ((4-bromophenyl)ethynyl)trimethylsilane (506 mg, 2.00 mmol, 1.00 equiv.) and a magnetic stir-bar. The Schlenk tube was then evacuated and backfilled with nitrogen three times. After that, THF (10 mL) was added by syringe and the Schlenk tube was placed at −78 °C in a dry ice/acetone bath. *n*-Butyllithium (2.5 M in hexane; 4.20 mmol, 1.68 mL, 2.10 equiv.) was added dropwise by a syringe pump over 5 min and the reaction mixture was stirred at −78 °C for additional 25 min. Then, TMSCl (3.00 mmol, 381 µL, 1.50 equiv.) was added dropwise under nitrogen. The reaction mixture was stirred at −78 °C for 45 min, then the cooling bath was removed. The reaction mixture was allowed to warm to room temperature gradually while keeping stirring for 30 min. The reaction mixture was then quenched by adding saturated aqueous NH_4Cl (10 mL). The organic phase was collected, and the remaining aqueous portion was extracted with EtOAc (3 × 5 mL). The combined organic portions were dried over Na_2SO_4, filtered and the volatiles removed under reduced pressure. The crude residue was dissolved by MeOH (10 mL) under air. Then, K_2CO_3 (82.9 mg, 600 µmol, 0.300 equiv.) was added. The reaction mixture was stirred under room temperature for 1 hour. The reaction mixture was then diluted by adding deionized water (20 mL) and EtOAc (20 mL). The organic phase was collected, and the remaining aqueous portion was extracted with EtOAc

(3 × 5 mL). The combined organic portions were dried over Na_2SO_4, filtered and the volatiles removed under reduced pressure. The crude product was purified via flash chromatography on silica gel (eluent : pentane). Terminal alkyne **2s** was obtained in 77% yield (268 mg, 1.54 mmol) as a colourless oil. **TLC**: R_f (*n*-hexane) = 0.38; **^{1}H NMR** (400 MHz, $CDCl_3$) δ 7.50 – 7.45 (m, 4H, Ar*H*), 3.09 (s, 1H, C≡C*H*), 0.27 (s, 9H, Si(C*H*$_3$)$_3$); **^{13}C NMR** (101 MHz, $CDCl_3$) δ 141.9, 133.3, 131.3, 122.5, 83.9, 77.6, -1.1. The spectroscopic data is consistent with previous report[26].

3.1.7. Synthesis and characterization of triethyl(4-ethynylphenyl)germane (2t)[27]

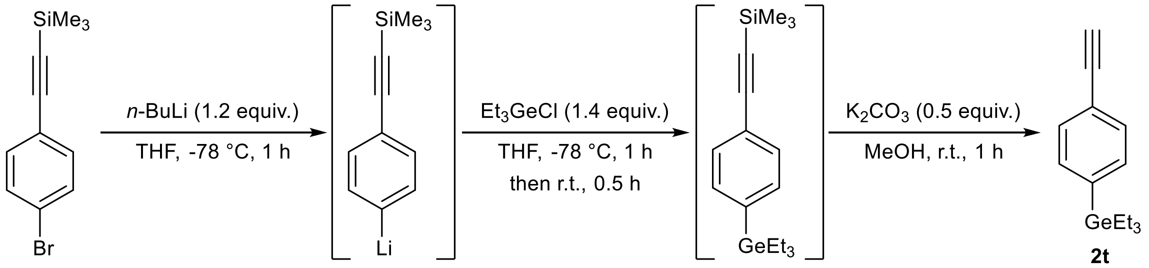

An oven-dried Schlenk tube was charged with ((4-bromophenyl)ethynyl)trimethylsilane (506 mg, 2.00 mmol, 1.00 equiv.) and a magnetic stir-bar. The Schlenk tube was then evacuated and backfilled with nitrogen three times. After that, THF (10 mL) was added by syringe and the Schlenk tube was placed at −78 °C in a dry ice/acetone bath. *n*-Butyllithium (2.5 M in hexane; 2.40 mmol, 0.96 mL, 1.20 equiv.) was added dropwise by a syringe pump over 5 min and the reaction mixture was stirred at −78 °C for additional 55 min. Then, Et_3GeCl (2.80 mmol, 547 mg, 1.40 equiv.) was added dropwise under nitrogen. The reaction mixture was stirred at −78 °C for 1 hour, then the cooling bath was removed. The reaction mixture was allowed to warm to room temperature gradually while keeping stirring for 30 min. The reaction mixture was then quenched by adding saturated aqueous NH_4Cl (10 mL). The organic phase was removed, and the remaining aqueous portion was extracted with EtOAc (3 × 5 mL). The combined organic portions were dried over Na_2SO_4, filtered and the volatiles removed under reduced pressure. The crude residue was dissolved by MeOH (10 mL) under air. Then, K_2CO_3 (138 mg, 1.00 mmol, 0.500 equiv.) was added. The reaction mixture was stirred under room temperature for 1 hour. The reaction mixture was then diluted by adding deionized water (20 mL) and EtOAc (20 mL). The organic phase was removed, and the remaining aqueous portion was extracted with EtOAc (3 × 5 mL). The combined organic portions were dried over Na_2SO_4, filtered and the volatiles removed under reduced pressure. The crude product was purified via flash chromatography on silica gel (eluent: pentane). Terminal alkyne **2t** was obtained in 89% yield (463 mg, 1.78 mmol) as a colourless oil. **TLC**: R_f (*n*-hexane) = 0.52; **^{1}H NMR** (400 MHz, $CDCl_3$) δ 7.45 (d, *J* = 8.1 Hz, 2H, Ar*H*), 7.42 – 7.35 (m, 2H, Ar*H*), 3.07 (s, 1H, C≡C*H*), 1.11 – 0.93 (m, 15H, Ge(C*H*$_2$C*H*$_3$)$_3$); **^{13}C NMR** (101 MHz, $CDCl_3$) δ 141.7, 134.0, 131.4, 121.8, 84.0, 77.3, 9.0, 4.3; **IR** (ν_{max}, cm^{-1}) 3300 (m), 3065 (w), 2952 (m), 2930 (m), 2906 (m), 2871 (m), 2391 (w), 2186 (w), 2108 (w), 1912 (w), 1805 (w), 1458 (m), 1231 (w), 1083 (m), 1015 (m), 970 (w), 819 (s); **HRMS** (APCI/QTOF) m/z: $[M + H]^+$ Calcd for $C_{14}H_{21}Ge^+$ 263.0850; Found 263.0857.

3.1.8. Synthesis and characterization of 4,4,5,5-tetraethyl-2-(4-ethynylphenyl)-1,3,2-dioxaborolane (2u)[28]

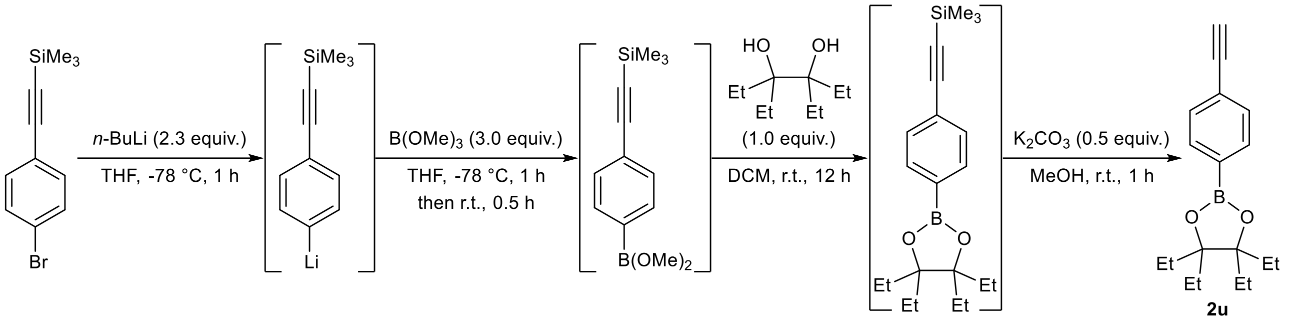

An oven-dried Schlenk tube was charged with ((4-bromophenyl)ethynyl)trimethylsilane (506 mg, 2.00 mmol, 1.00 equiv.) and a magnetic stir-bar. The Schlenk tube was then evacuated and backfilled with nitrogen three times. After that, THF (10 mL) was added by syringe and the Schlenk tube was placed at −78 °C in a dry ice/acetone bath. *n*-Butyllithium (2.5 M in hexane; 4.60 mmol, 1.8 mL, 2.30 equiv.) was added dropwise by a syringe pump over 5 min and the reaction mixture was stirred at −78 °C for additional 55 min. Then, $B(OMe)_3$ (6.00 mmol, 0.67 mL, 3.00 equiv.) was added dropwise under nitrogen. The reaction mixture was stirred at −78 °C for 1 hour, then the cooling bath was removed. The reaction mixture was allowed to warm to room temperature gradually while keeping stirring for 30 min. The reaction mixture was then quenched by adding saturated aqueous NH_4Cl (10 mL). The organic phase was removed, and the remaining aqueous portion was extracted with EtOAc (3 × 5 mL). The combined organic portions were dried over Na_2SO_4, filtered and the volatiles removed under reduced pressure. The crude residue was dissolved in DCM (10 mL) under air. Then, 3,4-diethylhexane-3,4-diol (349 mg, 2.00 mmol, 1.00 equiv.) was added. The reaction mixture was stirred under room temperature for 12 hours. The reaction mixture was then diluted by adding deionized water (10 mL) and DCM (10 mL). The organic phase was removed, and the remaining aqueous portion was extracted with DCM (3 × 5 mL). The combined organic portions were dried over Na_2SO_4, filtered and the volatiles removed under reduced pressure. The crude residue was dissolved by MeOH (10 mL) under air. Then, K_2CO_3 (138 mg, 1.00 mmol, 0.500 equiv.) was added. The reaction mixture was stirred under room temperature for 1 hour. The reaction mixture was then diluted by adding deionized water (20 mL) and EtOAc (20 mL). The organic phase was removed, and the remaining aqueous portion was extracted with EtOAc (3 × 5 mL). The combined organic portions were dried over Na_2SO_4, filtered and the volatiles removed under reduced pressure. The crude product was purified via flash chromatography on silica gel (eluent: pentane). Terminal alkyne **2u** was obtained in 83% yield (474mg, 1.67 mmol) as a colourless oil. **TLC**: R_f (*n*-hexane/EtOAc = 40:1) = 0.24; **^{1}H NMR** (400 MHz, $CDCl_3$) δ 7.83 – 7.71 (m, 2H, Ar*H*), 7.52 – 7.43 (m, 2H, Ar*H*), 3.13 (s, 1H, C≡C*H*), 1.86 – 1.59 (m, 8H, CCH_2CH_3), 0.96 (t, *J* = 7.5 Hz, 12H, CCH_2CH_3); **^{13}C NMR** (101 MHz, $CDCl_3$) δ 134.7, 131.4, 124.7, 89.2, 84.0, 78.3, 26.6, 9.0; **^{11}B NMR** (128 MHz, $CDCl_3$) δ 28.9; **IR** (ν_{max}, cm^{-1}) 3301 (w), 2979 (m), 2945 (m), 2885 (w), 2108 (w), 1932 (w), 1722 (w), 1607 (m), 1547 (w), 1511 (w), 1460 (w), 1400 (s), 1364 (s), 1350 (s), 1292 (m), 1260 (m), 1180 (w), 1090 (s), 1021 (w), 958 (w), 921 (s), 838 (m), 772 (w), 741 (m); **HRMS** (APPI/LTQ-Orbitrap) m/z: $[M + H]^+$ Calcd for $C_{18}H_{26}BO_2^+$ 285.2020; Found 285.2027.

3.2. Terminal alkynes tethered with natural products, bioactive molecules and pharmaceuticals

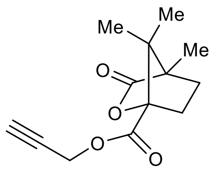

2ax
From (1*S*)-(-)-camphanic acid

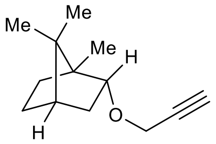

2ay
From (-)-borneol

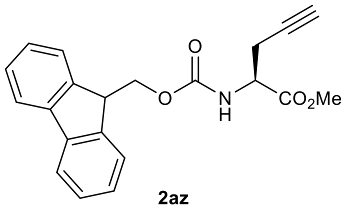

2az
From L-propargylglycine

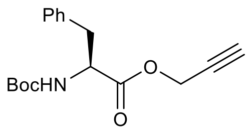

2ba
From L-phenylalanine

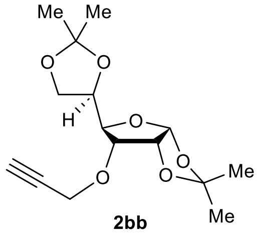

2bb
From α-D-allofuranose

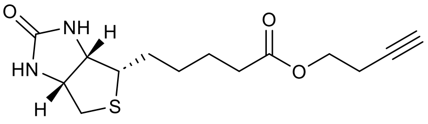

2bc
From D-biotin

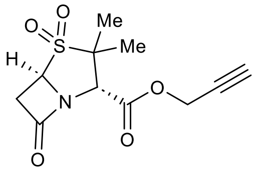

2bd
From Sulbactam

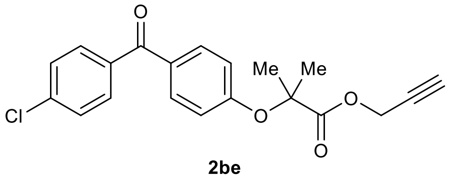

2be
From fenofibric acid

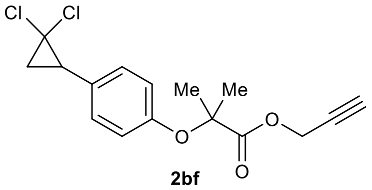

2bf
From ciprofibrate

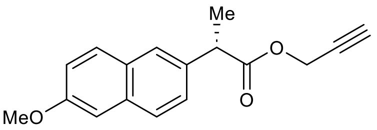

2bg
From naproxen

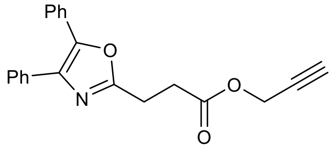

2bh
From oxaprozin

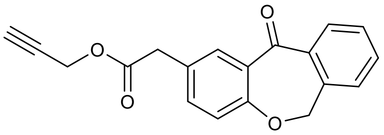

2bi
From isoxepac

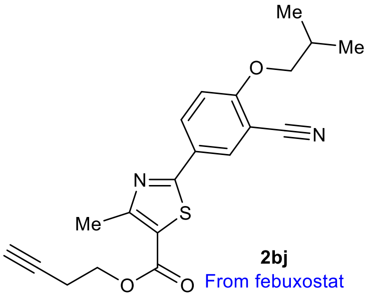

2bj
From febuxostat

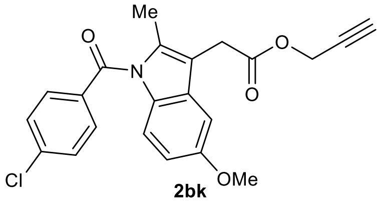

2bk
From indomethacin

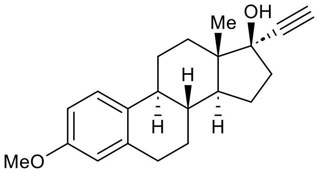

2bl
From mestranol

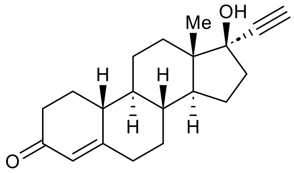

2bm
From norethindrone

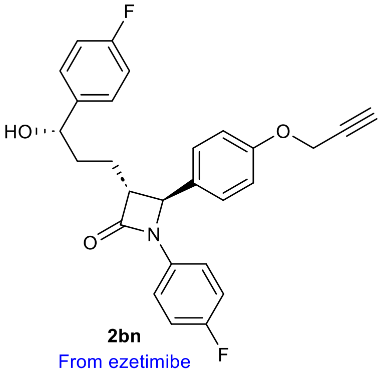

2bn
From ezetimibe

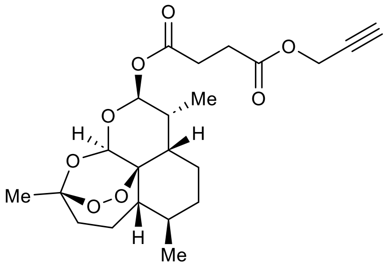

2bo
From artesunate

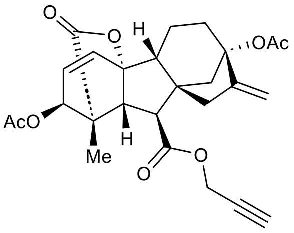

2bp
From gibberellic acid

Alkyne derivative **2bl** and **2bm** were commercially available and used as received. Alkyne derivative **2ax**, **2ay**, **2az**, **2ba**, **2bb**, **2bc**, **2bd**, **2be**, **2bf**, **2bg**, **2bh**, **2bi**, **2bj**, **2bk**, **2bn**, **2bo** and **2bp** were prepared according to the following procedures:

3.2.1. Synthesis and characterization of prop-2-yn-1-yl (1*S*,4*R*)-4,7,7-trimethyl-3-oxo-2-oxabicyclo[2.2.1]heptane-1-carboxylate (2ax)[29]

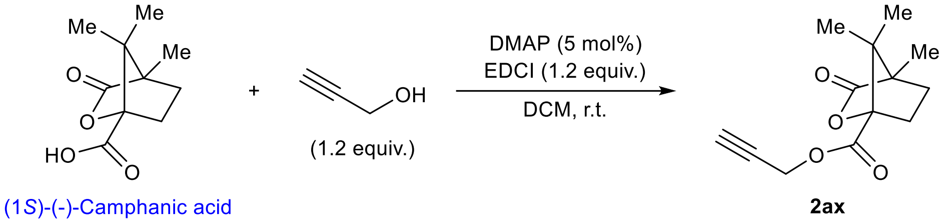

A mixture of 4-(dimethylamino)pyridine (DMAP; 6.10 mg, 50.0 µmol, 5.00 mol%), *N*-Ethyl-*N'*-(3-dimethylaminopropyl)carbodiimide hydrochloride (EDCI; 230 mg, 1.20 mmol, 1.20 equiv.), (1*S*)-(−)-camphanic acid (198 mg, 1.00 mmol, 1.00 equiv.), prop-2-yn-1-ol (67.3 mg, 1.20 mmol, 1.20 equiv.) and DCM (10 mL) was stirred under room temperature for 12 hours. The reaction mixture was then filtered through a silica gel pad and washed with DCM (3 × 5 mL). The organic solution of the crude product was washed with brine, dried over Na_2SO_4, filtered and concentrated under reduced pressure. The crude product was purified via flash chromatography on silica gel (eluent: pentane/ethyl acetate = 10:1). Alkyne derivative **2ax** was obtained in 65% yield (155 mg, 654 µmol) as a colourless solid. **M.p.** 52 – 53 °C. **ORD**: $[\alpha]_D^{20}$ = -22.4 (c = 1.00, $CHCl_3$). **TLC**: R_f (*n*-hexane/EtOAc = 4:1) = 0.31; **^{1}H NMR** (400 MHz, $CDCl_3$) δ 4.82 (dd, *J* = 2.5, 1.1 Hz, 2H, OC*H*$_2$), 2.51 (t, *J* = 2.4 Hz, 1H, C≡C*H*), 2.45 (ddd, *J* = 13.4, 10.7, 4.2 Hz, 1H, C*H*$_2$), 2.05 (ddd, *J* = 13.6, 9.3, 4.5 Hz, 1H, C*H*$_2$), 1.93 (ddd, *J* = 13.2, 10.7, 4.6 Hz, 1H, C*H*$_2$), 1.70 (ddd, *J* = 13.4, 9.4, 4.3 Hz, 1H, C*H*$_2$), 1.12 (s, 3H, C*H*$_3$), 1.08 (s, 3H, C*H*$_3$), 0.99 (s, 3H, C*H*$_3$); **^{13}C NMR** (101 MHz, $CDCl_3$) δ 178.0, 167.0, 90.9, 77.0, 75.8, 54.9, 54.7, 52.9, 30.7, 29.1, 16.9, 16.8, 9.8; **IR** (ν_{max}, cm^{-1}) 3278 (w), 2971 (w), 2940 (w), 2881 (w), 2130 (w), 1787 (s), 1757 (s), 1744 (s), 1474 (w), 1447 (w), 1399 (w), 1381 (w), 1335 (w), 1312 (m), 1269 (s), 1226 (w), 1168 (m), 1102 (s), 1057 (s), 1020 (m), 959 (m), 932 (m), 795 (w); **HRMS** (ESI/QTOF) m/z: [M + Na]$^+$ Calcd for $C_{13}H_{16}NaO_4^+$ 259.0941; Found 259.0949.

3.2.2. Synthesis and characterization of (1*S*,2*R*,4*S*)-1,7,7-trimethyl-2-(prop-2-yn-1-yloxy)bicyclo[2.2.1]heptane (2ay)[30]

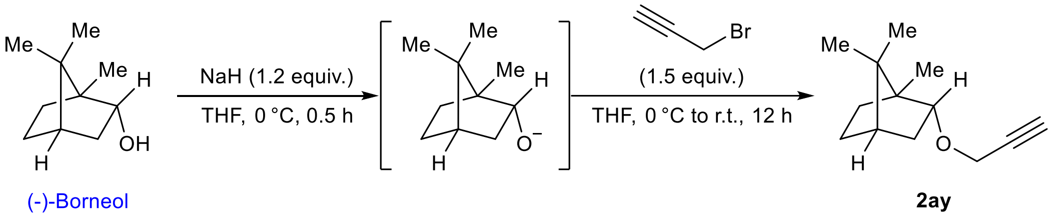

An oven-dried Schlenk tube was charged with (-)-borneol (674 mg, 4.37 mmol, 1.00 equiv.) and a magnetic stir-bar. The Schlenk tube was then evacuated and backfilled with nitrogen three times. After that, THF (10 mL) was added by syringe and the Schlenk tube was placed at 0 °C in an ice/water bath. Subsequently, NaH (60% dispersion in mineral oil; 5.24 mmol, 210 mg, 1.20 equiv.) was added in several portions and the reaction mixture was stirred at 0 °C for 30 min. Then, 3-bromoprop-1-yne (6.56 mmol, 780 mg, 1.50 equiv.) was added dropwise under nitrogen. Then the cooling bath was removed. The reaction mixture was stirred under room temperature for 12 hours. The reaction mixture was then quenched under 0 °C by adding saturated aqueous NH_4Cl (10 mL) and diluted with EtOAc (10 mL). The organic phase was removed, and the remaining aqueous portion was extracted with EtOAc (3 × 5 mL). The combined organic portions were washed with brine, dried over Na_2SO_4, filtered and the volatiles removed under reduced pressure. The crude product was purified via flash chromatography on silica gel (eluent: pentane). Alkyne derivative **2ay** was obtained in 58% yield (488 mg, 2.54 mmol) as a colourless oil. **ORD**: $[\alpha]_D^{20}$ = -74.8 (c = 0.26, $CHCl_3$). **TLC**: R_f (*n*-hexane/EtOAc = 10:1) = 0.17; **^{1}H NMR** (400 MHz, $CDCl_3$) δ 4.24 – 4.03 (m, 2H, OC*H*$_2$), 3.78 (ddd, *J* = 9.4, 3.4, 1.8 Hz, 1H,

OC*H*), 2.37 (t, *J* = 2.4 Hz, 1H, C≡C*H*), 2.14 (dddd, *J* = 12.9, 9.4, 4.8, 3.2 Hz, 1H, CH_2), 1.95 (ddd, *J* = 11.9, 9.6, 4.4 Hz, 1H, CH_2), 1.77 – 1.58 (m, 2H, CH_2), 1.33 – 1.16 (m, 2H, CH_2), 1.06 (dd, *J* = 13.1, 3.4 Hz, 1H, C*H*), 0.88 (s, 3H, CH_3), 0.86 (s, 3H, CH_3), 0.85 (s, 3H, CH_3); **^{13}C NMR** (101 MHz, $CDCl_3$) δ 84.5, 81.0, 73.6, 57.4, 49.3, 48.0, 45.1, 36.0, 28.3, 26.7, 19.9, 19.0, 13.9; **HRMS** (APPI/LTQ-Orbitrap) m/z: $[M]^+$ Calcd for $C_{13}H_{20}O^+$ 192.1509; Found 192.1512. The spectroscopic data is consistent with previous report[31].

3.2.3. Synthesis and characterization of methyl (*S*)-2-((((9*H*-fluoren-9-yl)methoxy)carbonyl)amino)pent-4-ynoate (2az)[32]

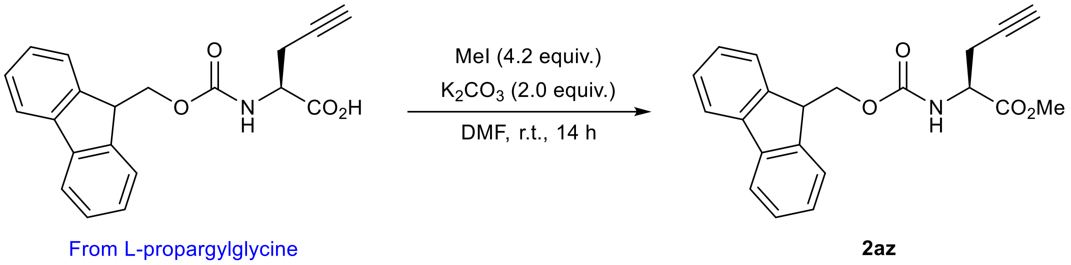

2az

An oven-dried Schlenk tube was charged with Fmoc-L-propargylglycine (335 mg, 1.00 mmol, 1.00 equiv.), K_2CO_3 (276 mg, 2.00 mmol, 2.00 equiv.) and a magnetic stir-bar. The Schlenk tube was then evacuated and backfilled with nitrogen three times. After that, dimethylformamide (DMF; 10 mL) was added by syringe and the resulting mixture was stirring under room temperature. Subsequently, MeI (4.20 mmol, 0.26 mL, 4.20 equiv.) was added dropwise and the reaction mixture was stirred at room temperature for 14 hours. The reaction mixture was then filtered through a silica gel pad and washed with EtOAc (3 × 5 mL). The organic solution of the crude product was washed with brine (3 × 10 mL), deionized water (3 × 10 mL), dried over Na_2SO_4, filtered and concentrated under reduced pressure. The crude product was purified via flash chromatography on silica gel (eluent: pentane/ethyl acetate = 3:1). Alkyne derivative **2az** was obtained in 94% yield (327 mg, 936 μmol) as a colorless solid. ^{13}C NMR showed the formation of rotamers (1:1 based on ^{13}C NMR) even if under 60 °C. **ORD**: $[\alpha]_D^{20}$ = +45.9 (c = 0.21, $CHCl_3$). **TLC**: R_f (*n*-hexane/EtOAc = 4:1) = 0.23; **^{1}H NMR** (400 MHz, $CDCl_3$, 60 °C) δ 7.77 (d, *J* = 7.5 Hz, 2H, Ar*H*), 7.68 – 7.56 (m, 2H, Ar*H*), 7.40 (t, *J* = 7.6 Hz, 2H, Ar*H*), 7.32 (tt, *J* = 7.4, 1.2 Hz, 2H, Ar*H*), 5.61 (bs, 1H, N*H*), 4.54 (bs, 1H, NC*H*), 4.44 (d, *J* = 7.1 Hz, 2H, CH_2(fluorenyl)), 4.26 (t, *J* = 7.0 Hz, 1H, C*H*(fluorenyl)), 3.80 (s, 3H, OCH_3), 2.78 (bs, 2H, CH_2C≡CH), 2.07 (t, *J* = 2.7 Hz, 1H, C≡C*H*); **^{13}C NMR** (101 MHz, $CDCl_3$, 60 °C) δ 170.8, 155.7 (bs), 144.1*, 144.0*, 141.5, 127.9, 127.23#, 127.22#, 125.2, 120.1, 78.5 (bs), 71.9, 67.5, 52.8, 52.7 (bs), 47.5, 22.9. *These two signals are assigned to one certain aryl carbon atom on the 9-fluorenyl group, which has also been reported by a previous study[33]; #These two signals are assigned to another one certain aryl carbon atom on the 9-fluorenyl group. **HRMS** (ESI/QTOF) m/z: $[M + Na]^+$ Calcd for $C_{21}H_{19}NNaO_4^+$ 372.1206; Found 372.1209. The NMR spectroscopic data is consistent with the previous reports[20,34,35].

3.2.4. Synthesis and characterization of methyl prop-2-yn-1-yl (*tert*-butoxycarbonyl)-*L*-phenylalaninate (2ba)[36]

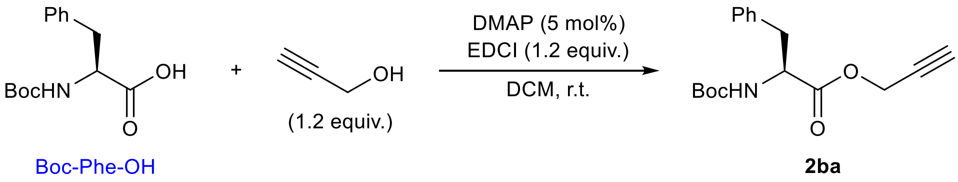

2ba

A mixture of 4-(dimethylamino)pyridine (DMAP; 6.10 mg, 50.0 μmol, 5.00 mol%), *N*-Ethyl-*N′*-(3-dimethylaminopropyl)carbodiimide hydrochloride (EDCI; 230 mg, 1.20 mmol, 1.20 equiv.), Boc-L-phenylalanine (265 mg, 1.00 mmol, 1.00 equiv.), prop-2-yn-1-ol (67.3 mg, 1.20 mmol, 1.20 equiv.) and DCM (10 mL) was stirred under room temperature for 12 hours. The reaction mixture was then

filtered through a silica gel pad and washed with DCM (3 × 5 mL). The organic solution of the crude product was washed with brine, dried over Na_2SO_4, filtered and concentrated under reduced pressure. The crude product was purified via flash chromatography on silica gel (eluent: pentane/ethyl acetate = 5:1). Alkyne derivative **2ba** was obtained in 84% yield (256 mg, 844 μmol) as a colourless oil. **ORD**: $[\alpha]_D^{20}$ = +12.1 (c = 0.81, $CHCl_3$). **TLC**: R_f (*n*-hexane/EtOAc = 10:1) = 0.20; **^{1}H NMR** (400 MHz, $CDCl_3$, 60 °C) δ 7.36 – 7.23 (m, 3H, Ar*H*), 7.22 – 7.15 (m, 2H, Ar*H*), 4.91 (bs, 1H, N*H*), 4.74 (qd, *J* = 15.5, 2.4 Hz, 2H, OC*H*$_2$), 4.63 (bs, 1H, NC*H*), 3.34 – 2.91 (m, 2H, PhC*H*$_2$), 2.51-2.50 (m, 1H, C≡C*H*), 1.45 (s, 9H, OC(C*H*$_3$)$_3$); **^{13}C NMR** (101 MHz, $CDCl_3$, 60 °C) δ 171.3, 155.1 (bs), 136.0, 129.6, 128.8, 127.3, 80.3 (bs), 77.3, 75.5, 54.8 (bs), 52.7, 38.5, 28.5; **HRMS** (ESI/QTOF) m/z: $[M + Na]^+$ Calcd for $C_{17}H_{21}NNaO_4^+$ 326.1363; Found 326.1366. The NMR spectroscopic data is consistent with the previous report[37].

3.2.5. Synthesis and characterization of (3a*R*,5*S*,6*R*,6a*R*)-5-((*R*)-2,2-dimethyl-1,3-dioxolan-4-yl)-2,2-dimethyl-6-(prop-2-yn-1-yloxy)tetrahydrofuro[2,3-*d*][1,3]dioxole (2bb)[38]

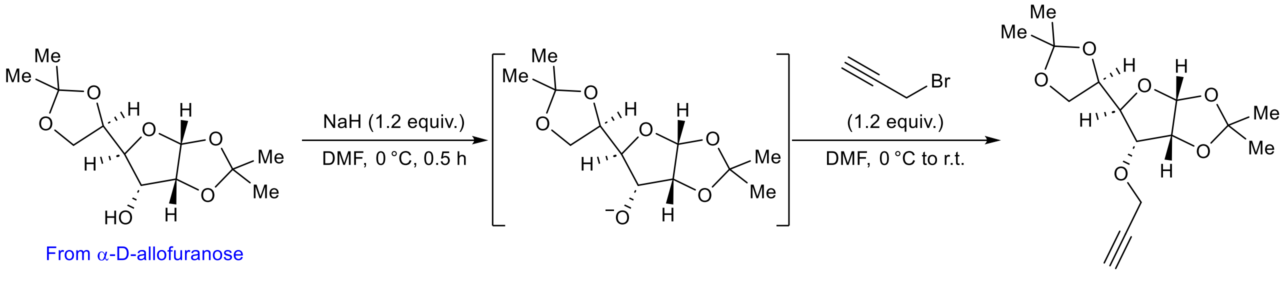

An oven-dried Schlenk tube was charged with 1,2:5,6-di-*O*-isopropylidene-α-D-allofuranose (521 mg, 2.00 mmol, 1.00 equiv.) and a magnetic stir-bar. The Schlenk tube was then evacuated and backfilled with nitrogen three times. After that, DMF (5 mL) was added by syringe and the Schlenk tube was placed at 0 °C in an ice/water bath. Subsequently, NaH (60% dispersion in mineral oil; 2.40 mmol, 96.0 mg, 1.20 equiv.) was added in several portions and the reaction mixture was stirred at 0 °C for 30 min. Then, 3-bromoprop-1-yne (2.40 mmol, 286 mg, 1.20 equiv.) was added dropwise under nitrogen. Then the cooling bath was removed. The reaction mixture was stirred under room temperature for 10 hours. The reaction mixture was then quenched under 0 °C by adding saturated aqueous NH_4Cl (5 mL) and diluted with EtOAc (10 mL). The organic phase was removed, and the remaining aqueous portion was extracted with EtOAc (3 × 5 mL). The combined organic portions were washed with brine (3 × 15 mL), deionized water (3 × 15 mL), dried over Na_2SO_4, filtered and the volatiles removed under reduced pressure. The crude product was purified via flash chromatography on silica gel (eluent: pentane). Alkyne derivative **2bb** was obtained in 95% yield (569 mg, 1.91 mmol) as a colourless solid. **M.p.** 118 – 120 °C. **ORD**: $[\alpha]_D^{20}$ = +106.8 (c = 0.35, $CHCl_3$). **TLC**: R_f (*n*-hexane/EtOAc = 5:1) = 0.29; **^{1}H NMR** (400 MHz, $CDCl_3$) δ 5.78 (d, *J* = 3.7 Hz, 1H, C*H*), 4.71 (t, *J* = 4.0 Hz, 1H, C*H*), 4.46 – 4.23 (m, 3H, OC*H*$_2$ & C*H*), 4.18 – 4.04 (m, 2H, C*H*), 4.05 – 3.93 (m, 2H, OC*H*$_2$), 2.47 (t, *J* = 2.4 Hz, 1H, C≡C*H*), 1.56 (s, 3H, C*H*$_3$), 1.46 (s, 3H, C*H*$_3$), 1.36 (s, 3H, C*H*$_3$), 1.34 (s, 3H, C*H*$_3$); **^{13}C NMR** (101 MHz, $CDCl_3$) δ 113.2, 109.9, 103.9, 79.3, 78.0, 77.9, 77.0, 75.6, 74.9, 65.2, 57.5, 26.9, 26.6, 26.4, 25.3; **HRMS** (ESI/QTOF) m/z: $[M + Na]^+$ Calcd for $C_{15}H_{22}NaO_6^+$ 321.1309; Found 321.1310. The spectroscopic data is consistent with previous report[39].

3.2.6. Synthesis and characterization of but-3-yn-1-yl 5-((3a*S*,4*S*,6a*R*)-2-oxohexahydro-1*H*-thieno[3,4-*d*]imidazol-4-yl)pentanoate (2bc)[40]

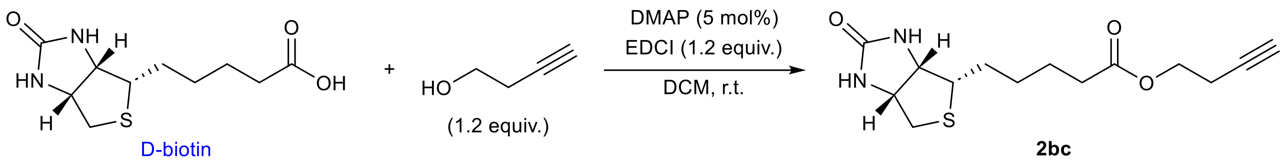

A mixture of 4-(dimethylamino)pyridine (DMAP; 12.2 mg, 100 μmol, 5.00 mol%), *N*-Ethyl-*N'*-(3-dimethylaminopropyl)carbodiimide hydrochloride (EDCI; 460 mg, 2.40 mmol, 1.20 equiv.), D-biotin (489 mg, 2.00 mmol, 1.00 equiv.), but-3-yn-1-ol (168 mg, 2.40 mmol, 1.20 equiv.) and DCM (10 mL) was stirred under room temperature for 3 days. The reaction mixture was then filtered through a silica gel pad and washed with DCM (3 × 5 mL). The organic solution of the crude product was washed with brine, dried over Na_2SO_4, filtered and concentrated under reduced pressure. The crude product was purified via flash chromatography on silica gel (eluent: DCM/MeOH = 20:1). Alkyne derivative **2bc** was obtained in 81% yield (480 mg, 1.62 mmol) as a colourless solid. **M.p.** 118 – 121 °C. **ORD**: $[\alpha]_D^{20}$ = +54.1 (c = 0.20, $CHCl_3$). **TLC**: R_f (DCM/MeOH = 20:1) = 0.26; **^{1}H NMR** (400 MHz, $CDCl_3$) δ 5.92 (bs, 1H, N*H*), 5.42 (bs, 1H, N*H*), 4.50 (ddd, *J* = 7.7, 5.0, 1.2 Hz, 1H, C*H*), 4.31 (dd, *J* = 7.8, 4.6 Hz, 1H, C*H*), 4.18 (td, *J* = 6.8, 0.8 Hz, 2H, CH_2), 3.15 (ddd, *J* = 8.1, 6.5, 4.6 Hz, 1H, C*H*), 2.91 (ddd, *J* = 12.9, 5.0, 1.2 Hz, 1H, CH_2), 2.74 (d, *J* = 12.8 Hz, 1H, CH_2), 2.52 (td, *J* = 6.7, 2.6 Hz, 2H, CH_2), 2.36 (t, *J* = 7.5 Hz, 2H, CH_2), 2.01 (t, *J* = 2.7 Hz, 1H, C≡C*H*), 1.69 (ttd, *J* = 10.6, 5.8, 2.6 Hz, 4H, CH_2), 1.57 – 1.36 (m, 2H, CH_2); **^{13}C NMR** (101 MHz, $CDCl_3$) δ 173.6, 163.7, 80.3, 70.1, 62.2, 62.1, 60.3, 55.6, 40.7, 33.9, 28.44, 28.37, 24.9, 19.1; **IR** (ν_{max}, cm^{-1}) 3343 (w), 3252 (m), 3201 (m), 3117 (w), 3065 (w), 2914 (w), 2849 (w), 1736 (m), 1706 (s), 1473 (m), 1420 (m), 1356 (w), 1317 (w), 1267 (m), 1212 (w), 1173 (s), 1105 (m), 1075 (w), 993 (w), 878 (w), 860 (w), 730 (m); **HRMS** (ESI/QTOF) m/z: $[M + Na]^+$ Calcd for $C_{14}H_{20}N_2NaO_3S^+$ 319.1087; Found 319.1092.

3.2.7. Synthesis and characterization of prop-2-yn-1-yl (2*S*,5*R*)-3,3-dimethyl-7-oxo-4-thia-1-azabicyclo[3.2.0]heptane-2-carboxylate 4,4-dioxide (2bd)[40]

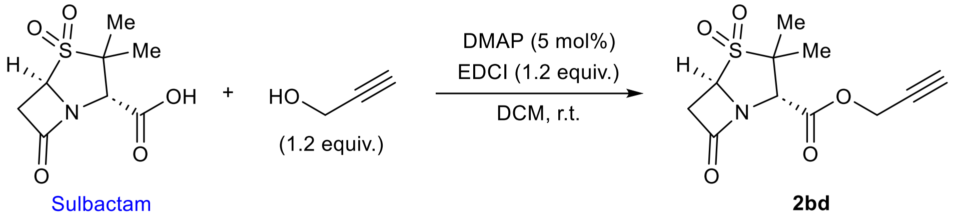

A mixture of 4-(dimethylamino)pyridine (DMAP; 6.10 mg, 50.0 μmol, 5.00 mol%), *N*-Ethyl-*N'*-(3-dimethylaminopropyl)carbodiimide hydrochloride (EDCI; 230 mg, 1.20 mmol, 1.20 equiv.), Sulbactam (233 mg, 1.00 mmol, 1.00 equiv.), prop-2-yn-1-ol (67.3 mg, 1.20 mmol, 1.20 equiv.) and DCM (10 mL) was stirred under room temperature for 12 hours. The reaction mixture was then filtered through a silica gel pad and washed with DCM (3 × 5 mL). The organic solution of the crude product was washed with brine, dried over Na_2SO_4, filtered and concentrated under reduced pressure. The crude product was purified via flash chromatography on silica gel (eluent: pentane/ethyl acetate = 4:1). Alkyne derivative **2bd** was obtained in 87% yield (237 mg, 873 μmol) as a colourless oil. **ORD**: $[\alpha]_D^{20}$ = +220.7 (c = 0.25, $CHCl_3$). **TLC**: R_f (*n*-hexane/EtOAc = 3:1) = 0.15; **^{1}H NMR** (400 MHz, $CDCl_3$) δ 4.90 (dd, *J* = 15.5, 2.5 Hz, 1H, CH_2), 4.72 (dd, *J* = 15.5, 2.5 Hz, 1H, CH_2), 4.62 (dd, *J* = 4.1, 2.2 Hz, 1H, C*H*), 4.42 (s, 1H, C*H*), 3.57 – 3.38 (m, 2H, CH_2), 2.55 (t, *J* = 2.5 Hz, 1H, C≡C*H*), 1.64 (s, 3H, CH_3), 1.45 (s, 3H, CH_3); **^{13}C NMR** (101 MHz, $CDCl_3$) δ 170.8, 166.4, 76.4, 76.4, 63.1, 63.0, 61.2, 53.5, 38.5, 20.4, 18.7; **IR** (ν_{max}, cm^{-1}) 3644 (w), 3283 (w), 2989 (w), 2130 (w), 1793 (s), 1762 (s), 1466 (w), 1442 (w), 1398 (w), 1376 (w), 1319 (s), 1274 (m), 1184 (s), 1157 (s), 1118 (s), 1085 (m), 1023 (m), 996 (m), 944 (s), 902 (w), 861 (w),

828 (w), 775 (w), 736 (m); **HRMS** (ESI/QTOF) m/z: [M + Na]$^+$ Calcd for $C_{11}H_{13}NNaO_5S^+$ 294.0407; Found 294.0408.

3.2.8. Synthesis and characterization of prop-2-yn-1-yl 2-(4-(4-chlorobenzoyl)phenoxy)-2-methylpropanoate (2be)[40]

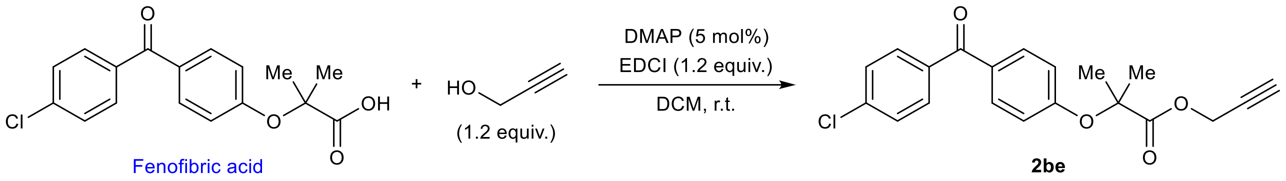

A mixture of 4-(dimethylamino)pyridine (DMAP; 6.10 mg, 50.0 μmol, 5.00 mol%), *N*-Ethyl-*N*′-(3-dimethylaminopropyl)carbodiimide hydrochloride (EDCI; 230 mg, 1.20 mmol, 1.20 equiv.), Fenofibric acid (319 mg, 1.00 mmol, 1.00 equiv.), prop-2-yn-1-ol (67.3 mg, 1.20 mmol, 1.20 equiv.) and DCM (5 mL) was stirred under room temperature for 12 hours. The reaction mixture was then filtered through a silica gel pad and washed with DCM (3 × 5 mL). The organic solution of the crude product was washed with brine, dried over Na_2SO_4, filtered and concentrated under reduced pressure. The crude product was purified via flash chromatography on silica gel (eluent: pentane/ethyl acetate = 10:1). Alkyne derivative **2be** was obtained in 72% yield (257 mg, 721 μmol) as a colourless oil. **TLC**: R_f (*n*-hexane/EtOAc = 10:1) = 0.20; **^{1}H NMR** (400 MHz, $CDCl_3$) δ 7.78 – 7.65 (m, 4H, Ar*H*), 7.52 – 7.39 (m, 2H, Ar*H*), 6.94 – 6.83 (m, 2H, Ar*H*), 4.77 (d, *J* = 2.4 Hz, 2H, OC*H*$_2$), 2.48 (t, *J* = 2.5 Hz, 1H, C≡C*H*), 1.69 (s, 6H, C*H*$_3$); **^{13}C NMR** (101 MHz, $CDCl_3$) δ 194.4, 173.1, 159.5, 138.6, 136.5, 132.1, 131.3, 130.8, 128.7, 117.8, 79.4, 76.9, 75.6, 53.1, 25.5; **IR** (ν_{max}, cm^{-1}) 3296 (w), 3072 (w), 2997 (w), 2942 (w), 2129 (w), 1922 (w), 1742 (m), 1653 (m), 1598 (s), 1505 (m), 1386 (w), 1305 (m), 1278 (s), 1247 (s), 1170 (s), 1130 (s), 1090 (s), 1013 (m), 989 (m), 959 (m), 927 (s), 852 (m), 839 (m), 762 (s), 738 (m); **HRMS** (ESI/QTOF) m/z: [M + Na]$^+$ Calcd for $C_{20}H_{17}ClNaO_4^+$ 379.0708; Found 379.0712.

3.2.9. Synthesis and characterization of prop-2-yn-1-yl 2-(4-(2,2-dichlorocyclopropyl)phenoxy)-2-methylpropanoate (2bf)[40]

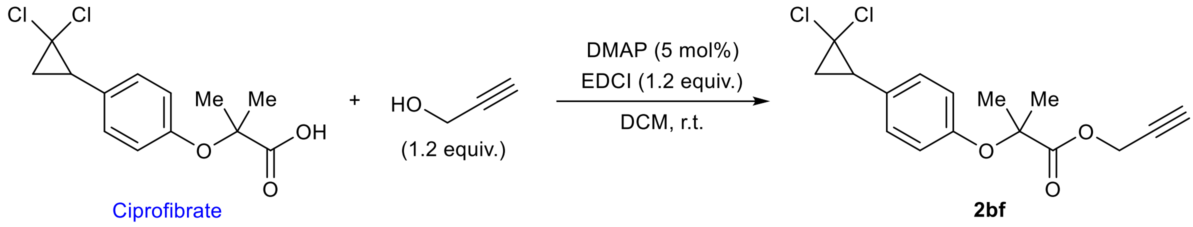

A mixture of 4-(dimethylamino)pyridine (DMAP; 6.10 mg, 50.0 μmol, 5.00 mol%), *N*-Ethyl-*N*′-(3-dimethylaminopropyl)carbodiimide hydrochloride (EDCI; 230 mg, 1.20 mmol, 1.20 equiv.), Ciprofibrate (289 mg, 1.00 mmol, 1.00 equiv.), prop-2-yn-1-ol (67.3 mg, 1.20 mmol, 1.20 equiv.) and DCM (10 mL) was stirred under room temperature for 12 hours. The reaction mixture was then filtered through a silica gel pad and washed with DCM (3 × 5 mL). The organic solution of the crude product was washed with brine, dried over Na_2SO_4, filtered and concentrated under reduced pressure. The crude product was purified via flash chromatography on silica gel (eluent: pentane/ethyl acetate = 20:1). Alkyne derivative **2bf** was obtained in 74% yield (241 mg, 736 μmol) as a colourless oil. **TLC**: R_f (*n*-hexane/EtOAc = 10:1) = 0.35; **^{1}H NMR** (400 MHz, $CDCl_3$) δ 7.17 – 7.03 (m, 2H, Ar*H*), 6.91 – 6.77 (m, 2H, Ar*H*), 4.76 (d, *J* = 2.5 Hz, 2H, OC*H*$_2$), 2.83 (dd, *J* = 10.7, 8.3 Hz, 1H, C*H*), 2.47 (t, *J* = 2.5 Hz, 1H, C≡C*H*), 1.94 (dd, *J* = 10.7, 7.4 Hz, 1H, C*H*$_2$), 1.77 (dd, *J* = 8.4, 7.4 Hz, 1H, C*H*$_2$), 1.61 (s, 6H, C*H*$_3$); **^{13}C NMR** (101 MHz, $CDCl_3$) δ 173.6, 154.8, 129.8, 128.7, 119.3, 79.3, 75.4, 61.0, 52.9, 35.0, 26.0, 25.44, 25.43; **IR** (ν_{max}, cm^{-1}) 3296 (w), 2996 (w), 2130 (w), 1742 (m), 1611 (w), 1510 (m), 1465 (w), 1436 (w), 1385 (w), 1367 (w), 1274 (m), 1239 (m), 1173 (m), 1127 (s), 1051 (w), 1012 (w), 989 (w), 966 (w), 932 (w),

889 (w), 835 (m), 761 (m), 734 (w); **HRMS** (ESI/QTOF) m/z: [M + Na]$^+$ Calcd for $C_{16}H_{16}Cl_2NaO_3^+$ 349.0369; Found 349.0376.

3.2.10. Synthesis and characterization of prop-2-yn-1-yl (*S*)-2-(6-methoxynaphthalen-2-yl)propanoate (2bg)[40]

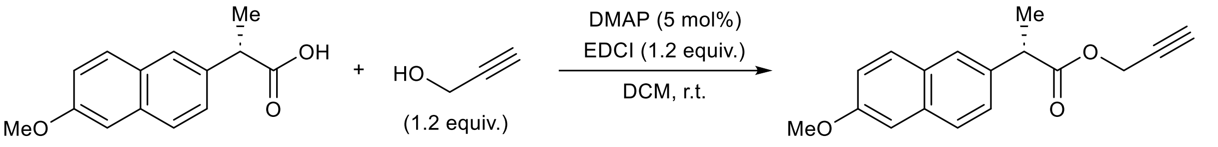

Naproxen **2bg**

A mixture of 4-(dimethylamino)pyridine (DMAP; 6.10 mg, 50.0 mmol, 5.00 mol%), *N*-Ethyl-*N'*-(3-dimethylaminopropyl)carbodiimide hydrochloride (EDCI; 230 mg, 1.20 mmol, 1.20 equiv.), Naproxen (230 mg, 1.00 mmol, 1.00 equiv.), prop-2-yn-1-ol (67.3 mg, 1.20 mmol, 1.20 equiv.) and DCM (10 mL) was stirred under room temperature for 12 hours. The reaction mixture was then filtered through a silica gel pad and washed with DCM (3 × 5 mL). The organic solution of the crude product was washed with brine, dried over Na_2SO_4, filtered and concentrated under reduced pressure. The crude product was purified via flash chromatography on silica gel (eluent: pentane/ethyl acetate = 10:1). Alkyne derivative **2bg** was obtained in 94% yield (254 mg, 945 μmol) as a colourless solid. **M.p.** 69 – 70 °C. **ORD**: $[\alpha]_D^{20}$ = +30.9 (c = 0.16, $CHCl_3$). **TLC**: R_f (*n*-hexane/EtOAc = 10:1) = 0.28; **^{1}H NMR** (400 MHz, $CDCl_3$) δ 7.74 – 7.69 (m, 2H, Ar*H*), 7.69 – 7.65 (m, 1H, Ar*H*), 7.41 (dd, *J* = 8.5, 1.9 Hz, 1H, Ar*H*), 7.19 – 7.07 (m, 2H, Ar*H*), 4.81 – 4.49 (m, 2H, OC*H*$_2$), 3.92 (s, 3H, OC*H*$_3$), 3.91 (q, *J* = 7.1 Hz, 1H, C*H*), 2.43 (t, *J* = 2.5 Hz, 1H, C≡C*H*), 1.60 (d, *J* = 7.2 Hz, 3H, C*H*$_3$); **^{13}C NMR** (101 MHz, $CDCl_3$) δ 174.0, 157.8, 135.2, 133.9, 129.4, 129.0, 127.4, 126.3, 126.2, 119.2, 105.7, 77.7, 75.0, 55.5, 52.4, 45.3, 18.7; **HRMS** (ESI/QTOF) m/z: [M + Na]$^+$ Calcd for $C_{17}H_{16}NaO_3^+$ 291.0992; Found 291.0982. The NMR spectroscopic data is consistent with previous report[41].

3.2.11. Synthesis and characterization of prop-2-yn-1-yl 3-(4,5-diphenyloxazol-2-yl)propanoate (2bh)[40]

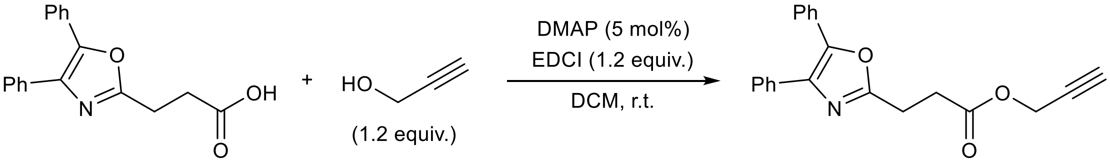

Oxaprozin **2bh**

A mixture of 4-(dimethylamino)pyridine (DMAP; 6.10 mg, 50.0 mmol, 5.00 mol%), *N*-Ethyl-*N'*-(3-dimethylaminopropyl)carbodiimide hydrochloride (EDCI; 230 mg, 1.20 mmol, 1.20 equiv.), Oxaprozin (293 mg, 1.00 mmol, 1.00 equiv.), prop-2-yn-1-ol (67.3 mg, 1.20 mmol, 1.20 equiv.) and DCM (10 mL) was stirred under room temperature for 12 hours. The reaction mixture was then filtered through a silica gel pad and washed with DCM (3 × 5 mL). The organic solution of the crude product was washed with brine, dried over Na_2SO_4, filtered and concentrated under reduced pressure. The crude product was purified via flash chromatography on silica gel (eluent: pentane/ethyl acetate = 10:1). Alkyne derivative **2bh** was obtained in 97% yield (320 mg, 966 μmol) as a colourless solid. **TLC**: R_f (*n*-hexane/EtOAc = 3:1) = 0.52; **^{1}H NMR** (400 MHz, $CDCl_3$) δ 7.67 – 7.61 (m, 2H, Ar*H*), 7.60 – 7.54 (m, 2H, Ar*H*), 7.41 – 7.28 (m, 6H, Ar*H*), 4.74 (d, *J* = 2.4 Hz, 2H, OC*H*$_2$), 3.32 – 3.14 (m, 2H, C*H*$_2$), 2.99 – 2.96 (m, 2H, C*H*$_2$), 2.46 (t, *J* = 2.5 Hz, 1H, C≡C*H*); **^{13}C NMR** (101 MHz, $CDCl_3$) δ 171.4, 161.6, 145.6, 135.3, 132.5, 129.1, 128.8, 128.7, 128.6, 128.2, 128.0, 126.6, 77.6, 75.2, 52.4, 31.0, 23.5; **HRMS** (ESI/QTOF) m/z: [M + H]$^+$ Calcd for $C_{21}H_{18}NO_3^+$ 332.1281; Found 332.1279. The NMR spectroscopic data is consistent with previous report[42].

3.2.12. Synthesis and characterization of prop-2-yn-1-yl 2-(11-oxo-6,11-dihydrodibenzo[*b*,*e*]oxepin-2-yl)acetate (2bi)[40]

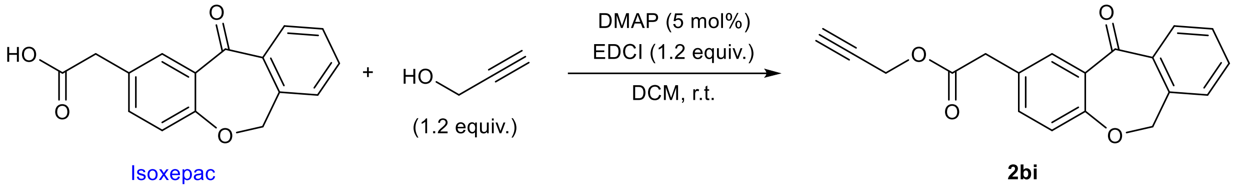

A mixture of 4-(dimethylamino)pyridine (DMAP; 6.10 mg, 50.0 µmol, 5.00 mol%), *N*-Ethyl-*N′*-(3-dimethylaminopropyl)carbodiimide hydrochloride (EDCI; 230 mg, 1.20 mmol, 1.20 equiv.), Isoxepac (268 mg, 1.00 mmol, 1.00 equiv.), prop-2-yn-1-ol (67.3 mg, 1.20 mmol, 1.20 equiv.) and DCM (10 mL) was stirred under room temperature for 12 hours. The reaction mixture was then filtered through a silica gel pad and washed with DCM (3 × 5 mL). The organic solution of the crude product was washed with brine, dried over Na_2SO_4, filtered and concentrated under reduced pressure. The crude product was purified via flash chromatography on silica gel (eluent: pentane/ethyl acetate = 10:1). Alkyne derivative **2bi** was obtained in 96% yield (295 mg, 962 µmol) as a colourless solid. **M.p.** 96 – 98 °C. **TLC**: R_f (*n*-hexane/EtOAc = 3:1) = 0.40; **^{1}H NMR** (400 MHz, $CDCl_3$) δ 8.12 (d, *J* = 2.4 Hz, 1H, Ar*H*), 7.89 (dd, *J* = 7.7, 1.4 Hz, 1H, Ar*H*), 7.56 (td, *J* = 7.4, 1.4 Hz, 1H, Ar*H*), 7.51 – 7.40 (m, 2H, Ar*H*), 7.36 (dd, *J* = 7.4, 1.3 Hz, 1H, Ar*H*), 7.03 (d, *J* = 8.4 Hz, 1H, Ar*H*), 5.19 (s, 2H, CH_2), 4.71 (d, *J* = 2.4 Hz, 2H, CH_2), 3.70 (s, 2H, CH_2), 2.48 (t, *J* = 2.5 Hz, 1H, C≡C*H*); **^{13}C NMR** (101 MHz, $CDCl_3$) δ 190.9, 170.7, 160.7, 140.6, 136.4, 135.6, 132.9, 132.7, 129.6, 129.4, 128.0, 127.3, 125.3, 121.3, 77.5, 75.3, 73.8, 52.6, 39.9; **IR** (ν_{max}, cm^{-1}) 3284 (w), 3065 (w), 3033 (w), 2974 (w), 2950 (w), 2921 (w), 2872 (w), 2129 (w), 1799 (w), 1739 (s), 1646 (m), 1611 (m), 1601 (m), 1570 (w), 1489 (s), 1453 (w), 1414 (m), 1376 (w), 1300 (s), 1285 (m), 1242 (m), 1221 (m), 1202 (m), 1139 (s), 1121 (s), 1013 (s), 964 (w), 938 (w), 860 (w), 830 (m), 800 (w), 762 (s); **HRMS** (ESI/QTOF) m/z: $[M + Na]^+$ Calcd for $C_{19}H_{14}NaO_4^+$ 329.0784; Found 329.0784.

3.2.13. Synthesis and characterization of but-3-yn-1-yl 2-(3-cyano-4-isobutoxyphenyl)-4-methylthiazole-5-carboxylate (2bj)[40]

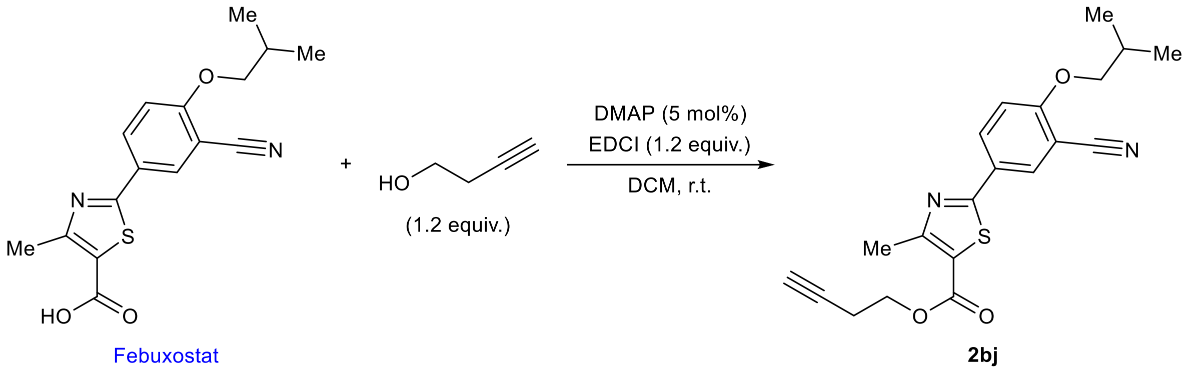

A mixture of 4-(dimethylamino)pyridine (DMAP; 6.10 mg, 50.0 mmol, 5.00 mol%), *N*-Ethyl-*N′*-(3-dimethylaminopropyl)carbodiimide hydrochloride (EDCI; 230 mg, 1.20 mmol, 1.20 equiv.), Febuxostat (316 mg, 1.00 mmol, 1.00 equiv.), but-3-yn-1-ol (84.1 mg, 1.20 mmol, 1.20 equiv.) and DCM (10 mL) was stirred under room temperature for 12 hours. The reaction mixture was then filtered through a silica gel pad and washed with DCM (3 × 5 mL). The organic solution of the crude product was washed with brine, dried over Na_2SO_4, filtered and concentrated under reduced pressure. The crude product was purified via flash chromatography on silica gel (eluent: pentane/ethyl acetate = 5:1). Alkyne derivative **2bj** was obtained in 84% yield (308 mg, 835 µmol) as a colourless solid. **TLC**: R_f (*n*-hexane/EtOAc = 3:1) = 0.48; **^{1}H NMR** (400 MHz, $CDCl_3$) δ 8.18 (d, *J* = 2.3 Hz, 1H, Ar*H*), 8.09 (dd, *J* = 8.8, 2.3 Hz, 1H, Ar*H*), 7.01 (d, *J* = 8.9 Hz, 1H, Ar*H*), 4.41 (t, *J* = 6.7 Hz, 2H, CH_2), 3.90 (d, *J* = 6.5 Hz, 2H, CH_2), 2.77 (s, 3H, CH_3), 2.66 (td, *J* = 6.7, 2.7 Hz, 2H, CH_2), 2.20 (dt, *J* = 13.3, 6.7 Hz, 1H, C*H*), 2.04 (t, *J* = 2.7 Hz,

1H, C≡C*H*), 1.09 (d, *J* = 6.7 Hz, 6H, C*H*$_3$); **^{13}C NMR** (101 MHz, $CDCl_3$) δ 167.7, 162.7, 161.9, 161.8, 132.7, 132.3, 126.1, 121.6, 115.5, 112.8, 103.2, 79.9, 75.9, 70.4, 63.0, 28.3, 19.3, 19.2, 17.7; **HRMS** (ESI/QTOF) m/z: [M + H]$^+$ Calcd for $C_{20}H_{21}N_2O_3S^+$ 369.1267; Found 369.1254. The spectroscopic data is consistent with previous report[43].

3.2.14. Synthesis and characterization of prop-2-yn-1-yl 2-(1-(4-chlorobenzoyl)-5-methoxy-2-methyl-1*H*-indol-3-yl)acetate (2bk)[44]

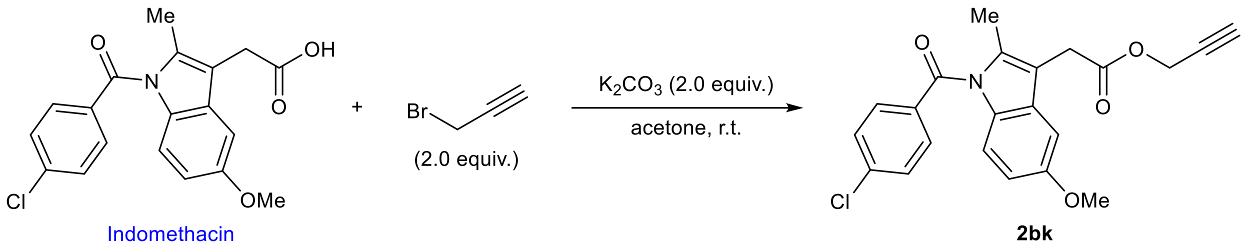

An oven-dried Schlenk tube was charged with Indomethacin (358 mg, 1.00 mmol, 1.00 equiv.), K_2CO_3 (276 mg, 2.00 mmol, 2.00 equiv.) and a magnetic stir-bar. The Schlenk tube was then evacuated and backfilled with nitrogen three times. After that, acetone (10 mL) was added by syringe and the resulting mixture was stirring under room temperature. Subsequently, 3-bromoprop-1-yne (2.00 mmol, 238 mg, 2.00 equiv.) was added dropwise and the reaction mixture was stirred at room temperature for 12 hours. The reaction mixture was then filtered through a silica gel pad and washed with EtOAc (3 × 5 mL). The solvent was removed under reduced pressure. The resulting residue was dissolved in EtOAc (15 mL) and the organic solution of the crude product was washed with brine (15 mL), deionized water (15 mL), dried over Na_2SO_4, filtered and concentrated under reduced pressure. The crude product was purified via flash chromatography on silica gel (eluent: pentane/ethyl acetate = 5:1). Alkyne derivative **2bk** was obtained in 98% yield (388 mg, 980 μmol) as a colourless oil, which turned to be solidified when stored in the freezer. **TLC**: R_f (*n*-hexane/EtOAc = 4:1) = 0.35; **^{1}H NMR** (400 MHz, $CDCl_3$) δ 7.70 – 7.62 (m, 2H, Ar*H*), 7.50 – 7.42 (m, 2H, Ar*H*), 6.96 (d, *J* = 2.5 Hz, 1H, Ar*H*), 6.87 (d, *J* = 9.1 Hz, 1H, Ar*H*), 6.67 (dd, *J* = 9.0, 2.5 Hz, 1H, Ar*H*), 4.71 (d, *J* = 2.5 Hz, 2H, C*H*$_2$), 3.84 (s, 3H, C*H*$_3$), 3.72 (s, 2H, C*H*$_2$), 2.48 (t, *J* = 2.5 Hz, 1H, C≡C*H*), 2.39 (s, 3H, C*H*$_3$); **^{13}C NMR** (101 MHz, $CDCl_3$) δ 170.2, 168.4, 156.2, 139.4, 136.3, 134.0, 131.3, 130.9, 130.6, 129.3, 115.1, 112.1, 112.0, 101.3, 77.6, 75.3, 55.8, 52.6, 30.2, 13.5; **HRMS** (ESI/QTOF) m/z: [M + H]$^+$ Calcd for $C_{22}H_{19}ClNO_4^+$ 396.0997; Found 396.0982. The NMR spectroscopic data is consistent with the previous reports[45].

3.2.15. Synthesis and characterization of (3*R*,4*S*)-1-(4-fluorophenyl)-3-((*S*)-3-(4-fluorophenyl)-3-hydroxypropyl)-4-(4-(prop-2-yn-1-yloxy)phenyl)azetidin-2-one (2bn)[46]

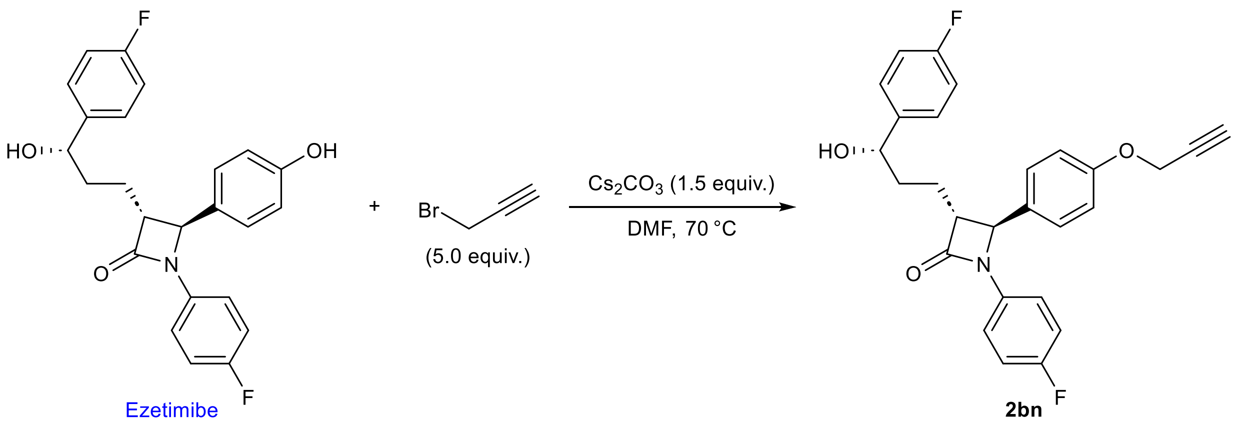

An oven-dried Schlenk tube was charged with Ezetimibe (409 mg, 1.00 mmol, 1.00 equiv.), Cs_2CO_3 (489 mg, 1.50 mmol, 1.50 equiv.) and a magnetic stir-bar. The Schlenk tube was then evacuated and backfilled with nitrogen three times. After that, dimethylformamide (DMF; 10 mL) was added by syringe and the resulting mixture was stirring under room temperature. Subsequently, 3-bromoprop-1-yne (5.00 mmol, 595 mg, 5.00 equiv.) was added dropwise and the reaction mixture was stirred at 70 °C for 12 hours. The reaction mixture was then filtered through a silica gel pad and washed with EtOAc (3 × 5 mL). The organic solution of the crude product was washed with brine (3 × 15 mL), deionized water (3 × 15 mL), dried over Na_2SO_4, filtered and concentrated under reduced pressure. The crude product was purified via flash chromatography on silica gel (eluent: pentane/ethyl acetate = 5:1). Alkyne derivative **2bn** was obtained in 71% yield (319 mg, 712 µmol) as a colourless oil. **ORD**: $[\alpha]_D^{20}$ = -42.8 (c = 0.33, $CHCl_3$). **TLC**: R_f (*n*-hexane/EtOAc = 1:1) = 0.61; **^{1}H NMR** (400 MHz, $CDCl_3$) δ 7.26 – 7.10 (m, 6H, Ar*H*), 7.00 – 6.77 (m, 6H, Ar*H*), 4.66 – 4.59 (m, 1H, O*H*), 4.61 (d, *J* = 2.4 Hz, 2H, C*H*$_2$), 4.50 (d, *J* = 2.3 Hz, 1H, C*H*), 2.99 (td, *J* = 7.4, 2.5 Hz, 1H, C*H*), 2.45 (t, *J* = 2.4 Hz, 1H, C≡C*H*), 2.25 (d, *J* = 3.7 Hz, 1H, C*H*), 2.01 – 1.71 (m, 4H, C*H*$_2$); **^{13}C NMR** (101 MHz, $CDCl_3$) δ 167.7, 162.3 (d, *J* = 245.5 Hz), 159.1 (d, *J* = 243.8 Hz), 157.9, 140.2 (d, *J* = 3.2 Hz), 134.0 (d, *J* = 2.8 Hz), 130.5, 127.5 (d, *J* = 8.1 Hz), 127.3, 118.5 (d, *J* = 7.9 Hz), 116.0 (d, *J* = 22.7 Hz), 115.7, 115.5 (d, *J* = 21.4 Hz), 78.4, 76.0, 73.2, 61.2, 60.5, 56.0, 36.7, 25.2; **^{19}F NMR** (376 MHz, $CDCl_3$) δ -114.9 (Ar*F*), -118.0 (Ar*F*); **IR** (ν_{max}, cm^{-1}) 3294 (w), 3066 (w), 2928 (w), 2862 (w), 2122 (w), 1894 (w), 1736 (s), 1607 (w), 1510 (s), 1387 (m), 1219 (s), 1156 (w), 1140 (w), 1025 (m), 833 (s), 735 (m); **HRMS** (ESI/QTOF) m/z: [M + Na]$^+$ Calcd for $C_{27}H_{23}F_2NNaO_3^+$ 470.1538; Found 470.1540.

3.2.16. Synthesis and characterization of prop-2-yn-1-yl ((3*R*,5a*S*,6*R*,8a*S*,9*R*,10*S*,12*R*,12a*R*)-3,6,9-trimethyldecahydro-12*H*-3,12-epoxy[1,2]dioxepino[4,3-*i*]isochromen-10-yl) succinate (2bo)[40]

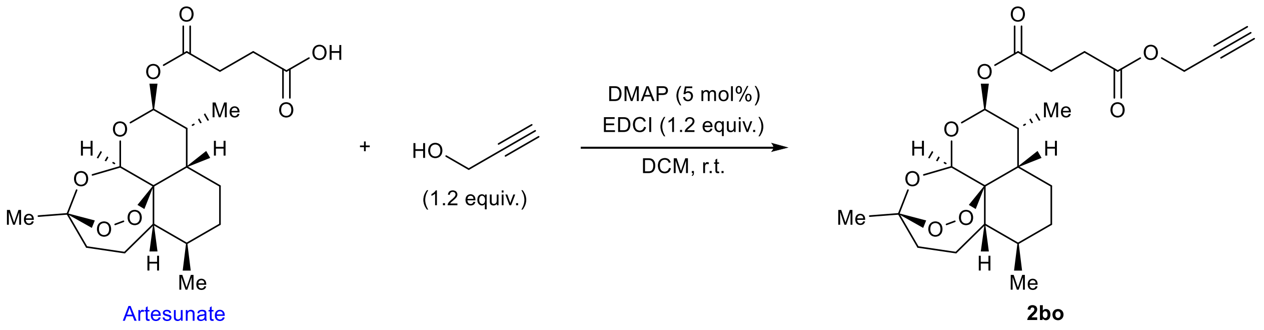

A mixture of 4-(dimethylamino)pyridine (DMAP; 6.10 mg, 50.0 mmol, 5.00 mol%), *N*-ethyl-*N'*-(3-dimethylaminopropyl)carbodiimide hydrochloride (EDCI; 230 mg, 1.20 mmol, 1.20 equiv.), Artesunate (384 mg, 1.00 mmol, 1.00 equiv.), prop-2-yn-1-ol (67.3 mg, 1.20 mmol, 1.20 equiv.) and DCM (10 mL) was stirred under room temperature for 12 hours. The reaction mixture was then filtered through a silica gel pad and washed with DCM (3 × 5 mL). The organic solution of the crude product was washed with brine, dried over Na_2SO_4, filtered and concentrated under reduced pressure. The crude product was purified via flash chromatography on silica gel (eluent: pentane/ethyl acetate = 10:1). Alkyne derivative **2bo** was obtained in 82% yield (345 mg, 816 µmol) as a colourless oil. **ORD**: $[\alpha]_D^{20}$ = +6.7 (c = 0.20, $CHCl_3$). **TLC**: R_f (*n*-hexane/EtOAc = 3:1) = 0.37; **^{1}H NMR** (400 MHz, $CDCl_3$) δ 5.79 (d, *J* = 9.8 Hz, 1H, C*H*), 5.43 (s, 1H, C*H*), 4.69 (td, *J* = 2.5, 0.6 Hz, 2H, C*H*$_2$), 2.81 – 2.61 (m, 4H, C*H*$_2$), 2.57 (dtd, *J* = 9.9, 7.3, 4.6 Hz, 1H, C*H*), 2.47 (t, *J* = 2.5 Hz, 1H, C≡C*H*), 2.43 – 2.30 (m, 1H, C*H*), 2.03 (ddd, *J* = 14.6, 4.9, 3.0 Hz, 1H, C*H*), 1.89 (ddt, *J* = 13.5, 6.6, 3.6 Hz, 1H, C*H*), 1.74 (ddq, *J* = 19.7, 13.4, 3.6 Hz, 2H, C*H*$_2$), 1.65 – 1.58 (m, 1H, C*H*), 1.55 – 1.20 (m, 7H, C*H* & C*H*$_3$), 1.07 – 0.95 (m, 1H, C*H*), 0.96 (d, *J* = 5.9 Hz, 3H, C*H*$_3$), 0.85 (d, *J* = 7.1 Hz, 3H, C*H*$_3$); **^{13}C NMR** (101 MHz, $CDCl_3$) δ 171.5, 171.1, 104.6, 92.4, 91.7, 80.2, 77.6, 75.2, 52.4, 51.7, 45.4, 37.4, 36.3, 34.2, 31.9, 29.2, 28.8, 26.1, 24.7, 22.1, 20.4, 12.2; **HRMS** (ESI/QTOF)

m/z: $[M + Na]^+$ Calcd for $C_{22}H_{30}NaO_8^+$ 445.1833; Found 445.1828. The spectroscopic data is consistent with previous report[47].

3.2.17. Synthesis and characterization of (1*S*,2*S*,4a*R*,4b*R*,7*S*,9a*S*,10*S*,10a*R*)-1-methyl-8-methylene-13-oxo-10-((prop-2-yn-1-yloxy)carbonyl)-1,2,5,6,8,9,10,10a-octahydro-4a,1-(epoxymethano)-7,9a-methanobenzo[*a*]azulene-2,7(4b*H*)-diyl diacetate (2bp)[40]

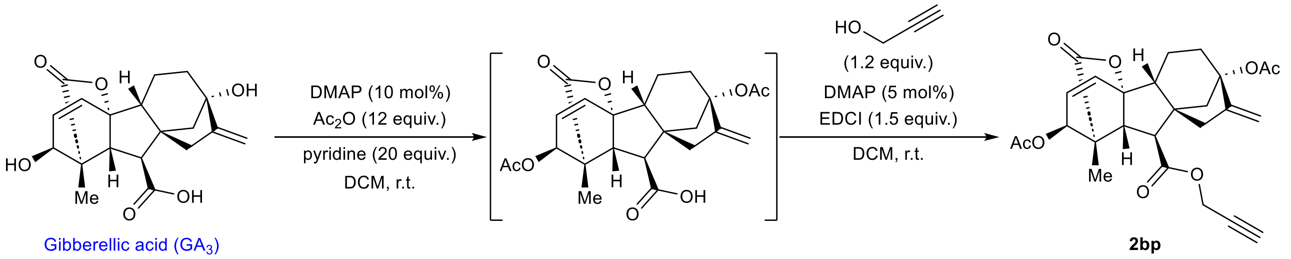

To a solution of gibberellic acid (GA_3; 693 mg, 2.00 mmol, 1.00 equiv.), acetic anhydride (Ac_2O; 2.3 mL, 24.0 mmol, 12.0 equiv.) and DMAP (24.4 mg, 200 µmol, 10.0 mol%) in anhydrous DCM (20 mL) at room temperature was added pyridine (3.2 mL, 40.0 mmol, 20.0 equiv.). After the addition, the mixture was allowed to stir for 48 hours at room temperature before addition of 10 mL of deionized water. After extraction with DCM (10 mL × 2), the organic phase was dried over Na_2SO_4, filtered and concentrated under reduced pressure to give a colourless oil. The crude was then dissolved in DCM (15 mL). To the solution was sequentially added *N*-ethyl-*N'*-(3-dimethylaminopropyl)carbodiimide hydrochloride (EDCI; 460 mg, 2.40 mmol, 1.20 equiv.), prop-2-yn-1-ol (135 mg, 2.40 mmol, 1.20 equiv.) and DMAP (12.2 mg, 100 µmol, 5.00 mol%). After the addition, the mixture was allowed to stir for 12 hours at room temperature before addition of 15 mL of deionized water. The organic phase was removed, and the remaining aqueous portion was extracted with DCM (3 × 5.0 mL). The combined organic portions were washed with brine, dried over Na_2SO_4, filtered and the volatiles removed under reduced pressure. The crude product was purified via flash chromatography on silica gel (eluent: pentane/ethyl acetate = 3:1). Alkyne derivative **2bp** was obtained in 29% yield (272 mg, 580 µmol) as a colourless solid. **ORD**: $[\alpha]_D^{20}$ = +152.5 (c = 0.22, $CHCl_3$). **TLC**: R_f (*n*-hexane/EtOAc = 4:1) = 0.16; **^{1}H NMR** (400 MHz, $CDCl_3$) δ 6.35 (dd, *J* = 9.3, 0.8 Hz, 1H, C*H*=CH), 5.85 (dd, *J* = 9.3, 3.8 Hz, 1H, CH=C*H*), 5.31 (dd, *J* = 3.8, 0.8 Hz, 1H, CH_2=C), 5.16 (dd, *J* = 3.2, 1.5 Hz, 1H, CH_2=C), 4.98 – 4.97 (m, 1H, C*H*), 4.72 (dd, *J* = 2.5, 0.6 Hz, 2H, CH_2), 3.31 (d, *J* = 11.0 Hz, 1H, C*H*), 2.78 (d, *J* = 11.0 Hz, 1H, C*H*), 2.49 (t, *J* = 2.4 Hz, 1H, C≡C*H*), 2.42 – 2.21 (m, 4H, CH_2), 2.18 – 2.12 (m, 1H, C*H*), 2.09 (s, 3H, CH_3), 2.00 (s, 3H, CH_3), 2.04 – 1.87 (m, 2H, CH_2), 1.81 – 1.60 (m, 2H, CH_2), 1.13 (s, 3H, CH_3); **^{13}C NMR** (101 MHz, $CDCl_3$) δ 177.0, 171.1, 170.1, 169.9, 153.4, 134.3, 129.3, 108.4, 89.9, 84.1, 77.1, 75.6, 70.2, 53.5, 52.5, 52.2, 51.3, 51.1, 50.2, 42.5, 39.9, 36.3, 22.1, 20.9, 16.9, 14.4; **HRMS** (ESI/QTOF) m/z: $[M + Na]^+$ Calcd for $C_{26}H_{28}NaO_8^+$ 491.1676; Found 491.1668. The spectroscopic data is consistent with previous report[48].

3.3. Synthesis and characterization of *N*-allenamides

3.3.1 Synthesis of 4-methyl-*N*-phenyl-*N*-(prop-2-yn-1-yl)benzenesulfonamide

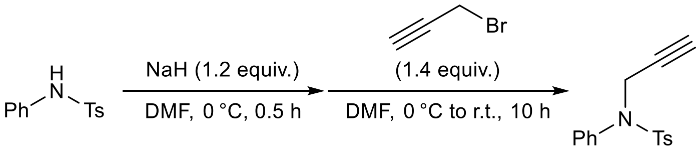

The product 4-methyl-*N*-phenyl-*N*-(prop-2-yn-1-yl)benzenesulfonamide was prepared following the procedure described in the literature[49]. Under argon, to a cooled (0 °C) solution of 4-methyl-*N*-phenylbenzenesulfonamide (742 mg, 3.00 mmol, 1.00 equiv.) in dry DMF (10 mL), NaH (60 wt.% in mineral oil, 144 mg, 3.6 mmol, 1.20 equiv.) was added in one portion. After stirring for 30 min at 0 °C, propargyl bromide (500 mg, 4.2 mmol, 1.40 equiv.) was added and the mixture was stirred at room

temperature for additional 10 h. The resulting mixture was quenched with a saturated aqueous solution of NH_4Cl (10 mL) and extracted with ethyl acetate (3 × 10 mL). The combined organic portions were washed with brine, dried over Na_2SO_4, filtered and the volatiles removed under reduced pressure. The crude product was purified via flash chromatography on silica gel (eluent: pentane/ethyl acetate = 20:1). 4-Methyl-*N*-phenyl-*N*-(prop-2-yn-1-yl)benzenesulfonamide was obtained in 97% yield (832 mg, 2.91 mmol) as a colourless oil. **TLC**: R_f (*n*-hexane/EtOAc = 20:1) = 0.34; **^{1}H NMR** (400 MHz, $CDCl_3$) δ 7.59 – 7.48 (m, 2H, Ar*H*), 7.35 – 7.27 (m, 3H, Ar*H*), 7.24 – 7.20 (m, 4H, Ar*H*), 4.43 (d, *J* = 2.5 Hz, 2H, NC*H*$_2$), 2.40 (s, 3H, C*H*$_3$), 2.17 (t, *J* = 2.5 Hz, 1H, C≡C*H*); **^{13}C NMR** (101 MHz, $CDCl_3$) δ 143.8, 139.3, 135.5, 129.3, 129.1, 128.4, 128.2, 128.0, 78.1, 73.9, 41.1, 21.6. The spectroscopic data is consistent with previous report[49].

3.3.2 Synthesis of 4-methyl-*N*-phenyl-*N*-(propa-1,2-dien-1-yl)benzenesulfonamide

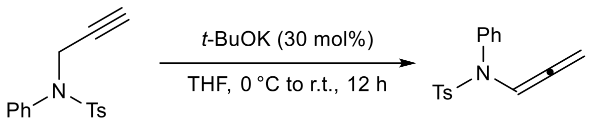

The product 4-methyl-*N*-phenyl-*N*-(propa-1,2-dien-1-yl)benzenesulfonamide was prepared following the procedure described in the literature[50]. To a solution of 4-methyl-*N*-phenyl-*N*-(prop-2-yn-1-yl)benzenesulfonamide (641 mg, 2.25 mmol, 1.00 equiv.) in 10 ml of anhydrous THF under argon atmosphere at 0 °C was added in one portion of *t*-BuOK (75.6 mg, 674 μmol, 0.300 equiv.). The reaction was allowed to stir at room temperature. After 12 h the mixture was diluted with Et_2O (10 mL), and then filtrated over celite. The residue was washed with diethyl ether (3 × 10 mL). The collected filtrate was concentrated in vacuo and the residue was purified via flash chromatography on silica gel (eluent: pentane/ethyl acetate = 30:1). 4-Methyl-*N*-phenyl-*N*-(propa-1,2-dien-1-yl)benzenesulfonamide was obtained in 70% yield (446 mg, 1.56 mmol) as a colourless solid. **TLC**: R_f (*n*-hexane/EtOAc = 30:1) = 0.48; **^{1}H NMR** (400 MHz, $CDCl_3$) δ 7.60 – 7.49 (m, 2H, Ar*H*), 7.33 – 7.22 (m, 5H, Ar*H*), 7.10 (t, *J* = 6.3 Hz, 1H, C*H*=C=CH$_2$), 7.05 – 6.94 (m, 2H, Ar*H*), 5.01 (s, 1H, CH=C=C*H*$_2$), 5.00 (s, 1H, CH=C=C*H*$_2$), 2.42 (s, 3H, C*H*$_3$); **^{13}C NMR** (101 MHz, $CDCl_3$) δ 201.1, 144.0, 137.3, 135.3, 129.6, 128.8, 128.7, 127.8, 102.5, 87.6, 21.7. The spectroscopic data is consistent with previous report[50].

4. Optimization of σ-type cyclopropenium cation transfer reaction with terminal alkynes

4.1. Evaluation of gold catalysts

An oven-dried 10 mL Schlenk tube was sequentially charged with a magnetic stir-bar, **L3** (4.50 mg, 25.0 μmol, 25.0 mol%), gold catalyst (5.00 μmol, 5.00 mol%) and CpBX **1a** (56.4 mg, 0.100 mmol, 1.00 equiv.). The Schlenk tube was then evacuated and backfilled with nitrogen three times. Subsequently, CH_3CN (0.10 M; 1.0 mL) and terminal alkyne **2a** (11 μL, 0.100 mmol, 1.00 equiv.) were added by syringe. The reaction mixture was stirred at 50 °C for the specified time. The resulting reaction mixture was diluted with CH_2Cl_2 (5.0 mL) and filtered through a short pad of silica gel by eluting with CH_2Cl_2 (3 × 5.0 mL). The filtrate was then concentrated to dryness and the residue was subjected to flash column chromatography on silica gel (eluent: pentane/EtOAc = 20:1). The fractions that contained the product **3a** and **4** were collected and concentrated by rotary evaporation. The yields of **3a** and **4** were obtained by quantitative ^{1}H NMR analysis using CH_2Br_2 (^{1}H NMR δ 4.92) as the internal standard.

Table 1 | Evaluation of gold catalysts

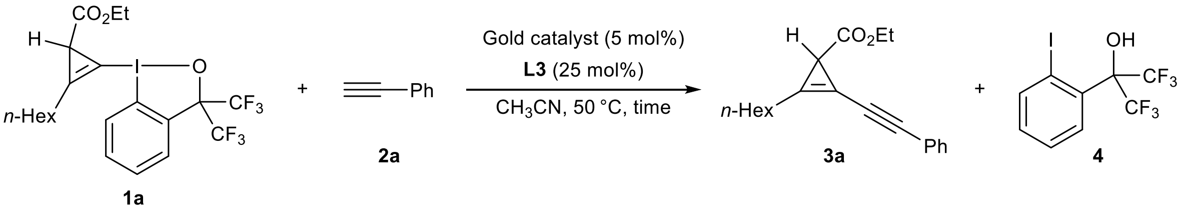

Entry	Gold catalyst	Time (h)	Yield (%)	
			**3a**	**4**
1	PPh_3AuCl	10	trace	-
2	AuCl	10	94	96
3	$AuCl_3$	10	90	99
4	$AuBr_3$	10	85	93
5	$PPhMe_2AuCl$	10	trace	-
6	$Au(OAc)_3$	10	18	22
7	PPh_3AuMe	10	trace	-
8	Au_2O_3[a]	10	35	42
9	$(ArO)_3PAuCl$	10	trace	-
10	IMesAuCl	10	0	-
11	$(Me_2S)AuCl$	2	97	99
12	PPh_3AuNTf_2	9	trace	92
13	$IPrAuNTf_2$	9	0	96
14	$HAuCl_4$•xH_2O	9	77	97
15[b]	none	9	0	-

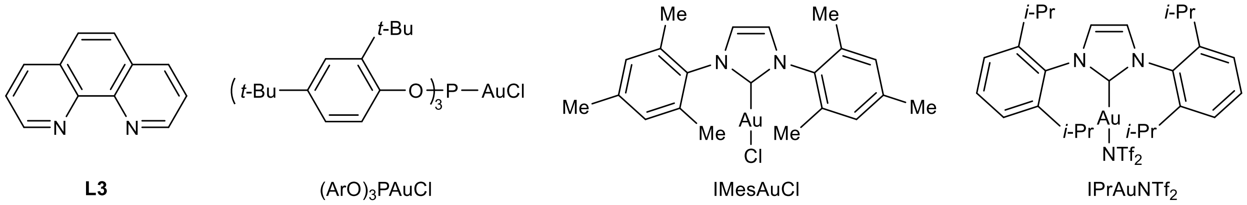

Reactions performed on a 0.100 mmol scale. Yields were determined by ^1H NMR spectroscopy using dibromomethane as the internal standard. [a]Au_2O_3 (2.50 mol%) was used instead. [b]**1a** was recovered in 99% NMR yield.

4.2. Evaluation of bidentate ligands

An oven-dried 10 mL Schlenk tube was sequentially charged with a magnetic stir-bar, bidentate ligand (25.0 μmol, 25.0 mol%), $(Me_2S)AuCl$ (1.47 mg, 5.00 μmol, 5.00 mol%) and CpBX **1a** (56.4 mg, 0.100 mmol, 1.00 equiv.). The Schlenk tube was then evacuated and backfilled with nitrogen three times. Subsequently, CH_3CN (0.10 M; 1.0 mL) and terminal alkyne **2a** (11 μL, 0.100 mmol, 1.00 equiv.) were added by syringe. The reaction mixture was stirred at 50 °C for the specified time. The resulting reaction mixture was diluted with CH_2Cl_2 (5.0 mL) and filtered through a short pad of silica gel by eluting with CH_2Cl_2 (3 × 5.0 mL). The filtrate was then concentrated to dryness and the residue was subjected to flash column chromatography on silica gel (eluent: pentane/EtOAc = 20:1). The fractions that contained the product **3a** and **4** were collected and concentrated by rotary evaporation. The yields of **3a** and **4** were obtained by quantitative ^1H NMR analysis using CH_2Br_2 (^1H NMR δ 4.92) as the internal standard.

Table 2 | Evaluation of bidentate ligands

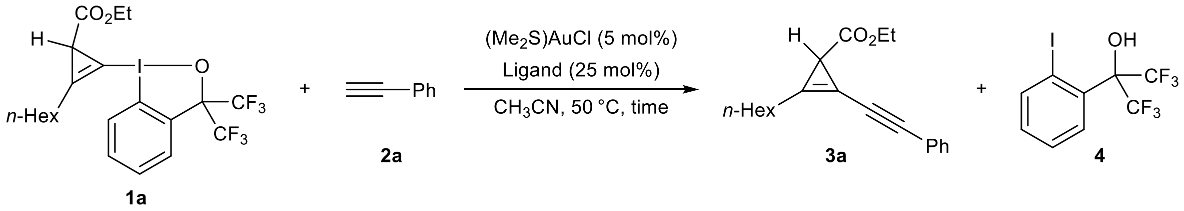

Entry	Ligand	Time (h)	Yield (%)	
			**3a**	**4**
1	**L1**	2	97	99
2	**L2**	11	92	97
3	**L3**	3	93	91
4	**L4**	11	90	93
5	**L5**	11	84	87
6	**L6**	11	80	85
7	**L7**	11	10	16
8	**L8**	11	29	32
9	**L9**	11	90	94
10	**L10**	11	27	34
11	none	9	55	66

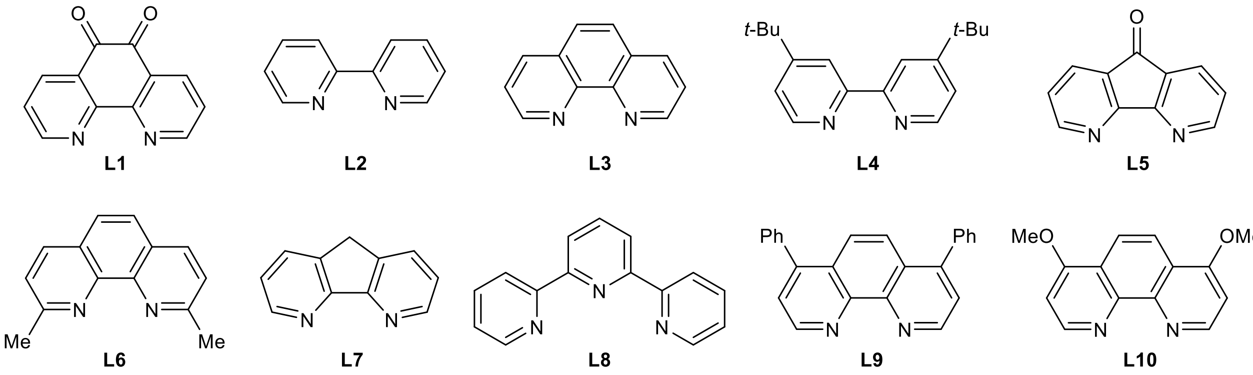

Reactions performed on a 0.100 mmol scale. Yields were determined by ^{1}H NMR spectroscopy using dibromomethane as the internal standard.

4.3. Evaluation of transition metal catalysts

An oven-dried 10 mL Schlenk tube was sequentially charged with a magnetic stir-bar, **L1** (5.25 mg, 25.0 μmol, 25.0 mol%), transition metal catalyst (TM catalyst; 5.00 μmol, 5.00 mol%) and CpBX **1a** (56.4 mg, 0.100 mmol, 1.00 equiv.). The Schlenk tube was then evacuated and backfilled with nitrogen three times. Subsequently, CH_3CN (0.10 M; 1.0 mL) and terminal alkyne **2a** (11 μL, 0.100 mmol, 1.00 equiv.) were added by syringe. The reaction mixture was stirred at 50 °C for the specified time. The resulting reaction mixture was diluted with CH_2Cl_2 (5.0 mL) and filtered through a short pad of silica gel by eluting with CH_2Cl_2 (3 × 5.0 mL). The filtrate was then concentrated to dryness and the residue was subjected to flash column chromatography on silica gel (eluent: pentane/EtOAc = 20:1). The fractions that contained the product **3a** and the remaining CpBX **1a** were collected separately and concentrated by rotary evaporation. The yields of **3a** and the recoveries of **1a** were obtained by quantitative ^{1}H NMR analysis using CH_2Br_2 (^{1}H NMR δ 4.92) as the internal standard.

Table 3 | Evaluation of transition metal catalysts

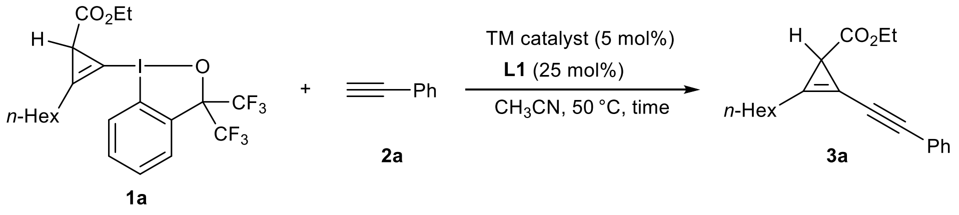

Entry	Transition metal catalyst	Time (h)	Yield (%)	
			3a	1a
1	$[Rh(nbd)Cl]_2$[a]	12	0	95
2	$FeCl_2$	12	0	99
3	$PtCl_2$	12	0	98
4	$RuCl_3$	12	0	99
5	AgCl	12	0	99
6	$(PhCN)_2PdCl_2$	12	0	99
7	$NiCl_2$(glyme)	12	0	99
8	CuCl	12	trace	78
9	$(Me_2S)AuCl$	2	97	0

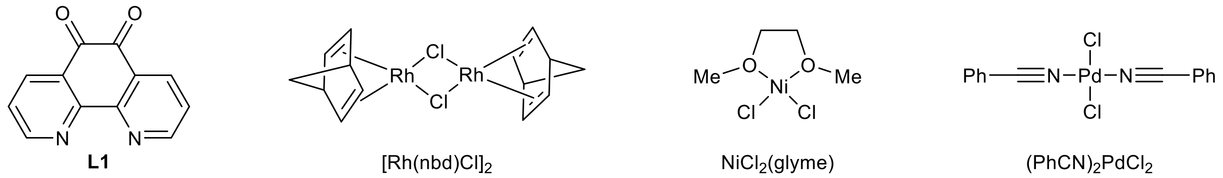

Reactions performed on a 0.100 mmol scale. Yields were determined by ^{1}H NMR spectroscopy using dibromomethane as the internal standard. [a]$[Rh(nbd)Cl]_2$ (2.50 mol%) was used instead.

4.4. Variations from the standard condition

An oven-dried 10 mL Schlenk tube was sequentially charged with a magnetic stir-bar, ligand, catalyst and CpBX **1a** (56.4 mg, 0.100 mmol, 1.00 equiv.) or **1a-1** (32.2 mg, 0.100 mmol, 1.00 equiv.). The Schlenk tube was then evacuated and backfilled with nitrogen three times. Subsequently, solvent (0.10 M; 1.0 mL) and terminal alkyne **2a** (11 µL, 0.100 mmol, 1.00 equiv.) or its surrogates were added. The reaction mixture was stirred at the indicated temperature for the specified time. The resulting reaction mixture was diluted with CH_2Cl_2 (5.0 mL)and filtered through a short pad of silica gel by eluting with CH_2Cl_2 (3 × 5.0 mL). The filtrate was then concentrated to dryness and the residue was subjected to flash column chromatography on silica gel (eluent: pentane/EtOAc = 20:1 to 5:1). The fractions that contained the product **3a**, **4** and the remaining CpBX **1a** or **1a-1** were collected separately and concentrated by rotary evaporation. The yields of **3a**, **4** and the recoveries of **1a** or **1a-1** were obtained by quantitative ^{1}H NMR analysis using CH_2Br_2 (^{1}H NMR δ 4.92) as the internal standard.

Table 4 | Variations from the standard conditions

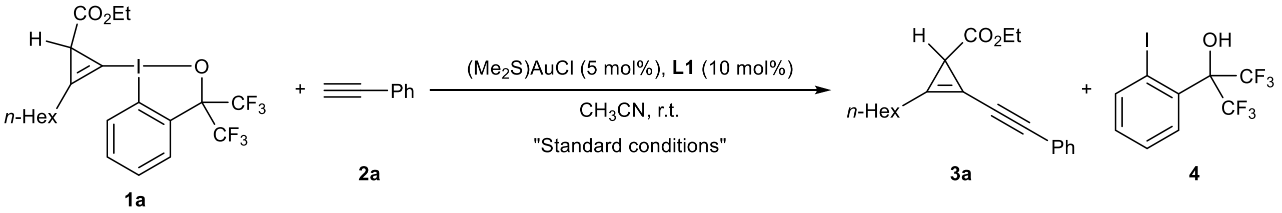

Entry	Variations from the "standard conditions"	Time (h)	Yield (%)[a]		
			3a	1a	4
1	none	2	96	0	99
2	without $(Me_2S)AuCl$	24	0	99	-
3	without **L1**	8	84	-	97
4	$(PhCN)_2PdCl_2$ or $NiCl_2$(glyme) instead of $(Me_2S)AuCl$, 50 °C	12	0	99	-
5	**L1** (25 mol%) was used	2	95	-	99
6	PPh_3AuNTf_2 instead of $(Me_2S)AuCl$	24	1	72	22
7	**L2** instead of **L1**	5	95	-	95
8	**L3** instead of **L1**	4	96	-	97
9	DCM instead of CH_3CN	24	88	-	94
10	THF instead of CH_3CN	13	93	-	98
11	AuCl instead of $(Me_2S)AuCl$	4	93	-	94
12	$AuCl_3$ instead of $(Me_2S)AuCl$	24	15	69	30
13	$AuCl_3$ instead of $(Me_2S)AuCl$, 40 °C	6	69	-	98
14	**1a-1** instead of **1a**, **L1** (25 mol%), 50 °C	15	0	-[b]	-
15	**2a-1** instead of **2a**	4	80	-	81
16	**2a-2** instead of **2a**	2	96	-	96
17	**2a-3** instead of **2a**	24	0	90	9
18	**2a-4** instead of **2a**	24	10	82	16

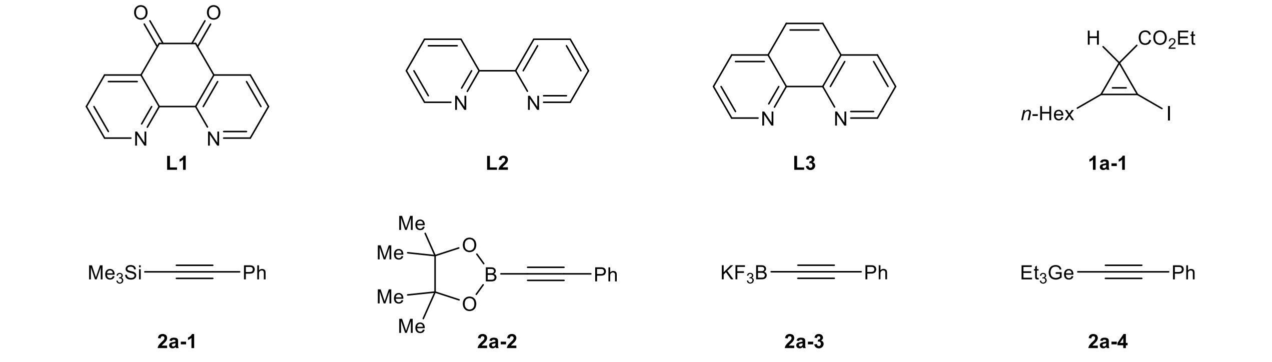

Reactions performed on a 100 µmol scale. [a]Yields or recovery were determined by ^1H NMR spectroscopy using dibromomethane as the internal standard. [b]98% NMR recovery of **1a-1**.

5. Substrate scope of σ-type cyclopropenium cation transfer to terminal alkyne

General Procedure D (**GPD**) for the gold-catalysed σ-type cyclopropenium cation transfer to terminal alkyne:

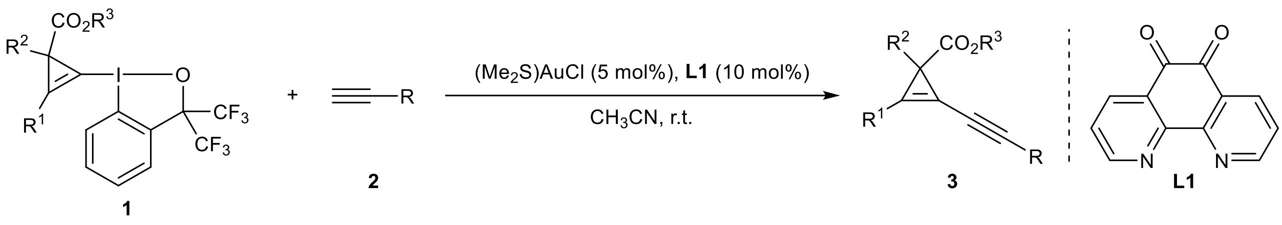

GPD: An oven-dried 10 mL Schlenk tube was sequentially charged with a magnetic stir-bar, **L1** (4.20 mg, 20.0 µmol, 10.0 mol%), (Me_2S)AuCl (2.95 mg, 10.0 µmol, 5.00 mol%), terminal alkyne **2** (0.200 mmol, 1.00 equiv.) and CpBX **1** (0.200 mmol, 1.00 equiv.). The Schlenk tube was then evacuated and backfilled with nitrogen three times. Subsequently, CH_3CN (0.10 M; 2.0 mL) was added by syringe; if **2** was a liquid, it was added last. The reaction mixture was stirred at room temperature (ca. 21 °C) for the specified time. The reaction mixture was then filtered through a silica gel pad and washed with CH_2Cl_2 (3 × 5.0 mL). Excess solvent was removed under reduced pressure and the desired product **3** was obtained by column chromatography on silica gel. The by-product **4** is a volatile colourless liquid. Thus, the fractions that contained **4** were collected and concentrated by rotary evaporation (vacuum pressure higher than 100 mBar, 40 °C water bath) to afford a concentrated solution of **4** in EtOAc. The yield of **4**[51] was determined by quantitative ^{1}H NMR analysis of the collected residue using CH_2Br_2 (^{1}H NMR δ 4.92) as the internal standard.

5.1. Substrate scope of terminal alkyne

5.1.1. Synthesis and characterization of ethyl 2-hexyl-3-(phenylethynyl)cycloprop-2-ene-1-carboxylate (3a)

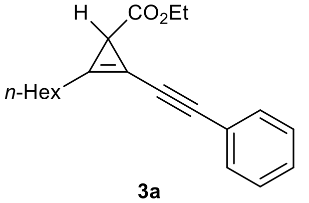

3a

Following **GPD**, a mixture of (Me_2S)AuCl (2.95 mg, 10.0 µmol, 5.00 mol%), **L1** (4.20 mg, 20.0 µmol, 10.0 mol%), CpBX **1a** (113 mg, 0.200 mmol, 1.00 equiv.), terminal alkyne **2a** (22 µL, 0.200 mmol, 1.00 equiv.) and CH_3CN (2.0 mL) was stirred at room temperature for 2 hours. Flash column chromatography on silica gel (eluent: pentane/ethyl acetate = 50:1) afforded **3a** in 95% yield (56.5 mg, 191 µmol) as a colourless oil and **4** in 98% NMR yield. **TLC**: R_f (*n*-hexane/EtOAc = 10:1) = 0.48; **^{1}H NMR** (400 MHz, $CDCl_3$) δ 7.56 – 7.42 (m, 2H, Ar*H*), 7.37 – 7.30 (m, 3H, Ar*H*), 4.23 – 4.11 (m, 2H, $CO_2CH_2CH_3$), 2.58 (t, *J* = 7.2 Hz, 2H, CH_2CH_2C), 2.50 (s, 1H, $CHCO_2$), 1.74 – 1.57 (m, 2H, CH_2CH_2C), 1.47 – 1.36 (m, 2H, CH_2), 1.36 – 1.21 (m, 7H, CH_2 & $CO_2CH_2CH_3$), 0.98 – 0.81 (m, 3H, CH_3); **^{13}C NMR** (101 MHz, $CDCl_3$) δ 174.3, 131.9, 129.1, 128.5, 122.5, 116.9, 99.0, 90.6, 76.7, 60.6, 31.6, 28.9, 26.7, 26.1, 25.3, 22.6, 14.5, 14.2; **IR** (ν_{max}, cm^{-1}) 2971 (m), 2928 (m), 2858 (m), 2121 (w), 1858 (w), 1753 (w), 1721 (s), 1490 (m), 1369 (m), 1335 (m), 1249 (m), 1188 (s), 1026 (m), 756 (s); **HRMS** (ESI/QTOF) m/z: [M + Na]$^+$ Calcd for $C_{20}H_{24}NaO_2^+$ 319.1669; Found 319.1667.

5.1.2. Synthesis and characterization of ethyl 2-hexyl-3-((4-pentylphenyl)ethynyl)cycloprop-2-ene-1-carboxylate (3b)

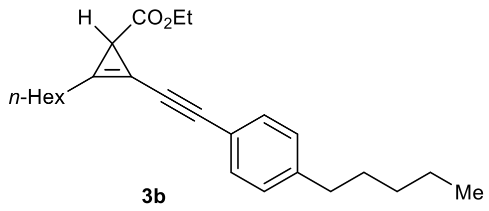

3b

Following **GPD**, a mixture of (Me_2S)AuCl (2.95 mg, 10.0 µmol, 5.00 mol%), **L1** (4.20 mg, 20.0 µmol, 10.0 mol%), CpBX **1a** (113 mg, 0.200 mmol, 1.00 equiv.), terminal alkyne **2b** (34.5 mg, 0.200 mmol, 1.00 equiv.) and CH_3CN (2.0 mL) was stirred at room temperature for 3 hours. Flash column chromatography on silica gel (eluent: pentane/ethyl acetate = 50:1) afforded **3b** in 89% yield (65.0 mg, 177 µmol) as a colourless oil and **4** in 99% NMR yield. **TLC**: R_f (*n*-hexane/EtOAc = 10:1) = 0.56; **^{1}H NMR** (400 MHz, $CDCl_3$) δ 7.43 – 7.36 (m, 2H, Ar*H*), 7.18 – 7.10 (m, 2H, Ar*H*), 4.23 – 4.11 (m, 2H, $CO_2CH_2CH_3$), 2.62 – 2.56 (m, 4H, CH_2), 2.49 (s, 1H, C*H*CO_2), 1.68 – 1.53 (m, 4H, CH_2), 1.48 – 1.37 (m, 2H, CH_2), 1.37 – 1.21 (m, 11H, CH_2 & $CO_2CH_2CH_3$), 0.89 (td, *J* = 7.0, 4.9 Hz, 6H, CH_3); **^{13}C NMR** (101 MHz, $CDCl_3$) δ 174.4, 144.4, 131.8, 128.6, 119.6, 116.3, 99.3, 90.7, 76.1, 60.6, 36.0, 31.6, 31.5, 31.0, 28.9, 26.7, 26.1, 25.3, 22.7, 22.6, 14.5, 14.2, 14.1; **IR** (ν_{max}, cm^{-1}) 2956 (s), 2930 (s), 2871 (m), 2858 (m), 2197 (m), 1725 (s), 1653 (m), 1606 (s), 1509 (w), 1466 (m), 1413 (w), 1373 (w), 1334 (w), 1179 (m), 1095 (m), 1022 (m), 839 (m); **HRMS** (ESI/QTOF) m/z: $[M + Na]^+$ Calcd for $C_{22}H_{22}NaO_5^+$ 389.1359; Found 389.1362.

5.1.3. Synthesis and characterization of ethyl 2-hexyl-3-((4-vinylphenyl)ethynyl)cycloprop-2-ene-1-carboxylate (3c)

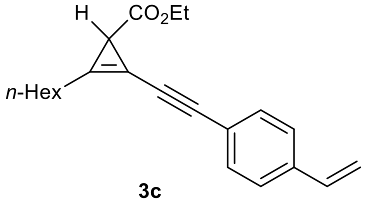

3c

Following **GPD**, a mixture of (Me_2S)AuCl (2.95 mg, 10.0 µmol, 5.00 mol%), **L1** (4.20 mg, 20.0 µmol, 10.0 mol%), CpBX **1a** (113 mg, 0.200 mmol, 1.00 equiv.), terminal alkyne **2c** (25.6 mg, 0.200 mmol, 1.00 equiv.) and CH_3CN (2.0 mL) was stirred at room temperature for 2 hours. Flash column chromatography on silica gel (eluent: pentane/ethyl acetate = 50:1) afforded **3c** in 89% yield (57.3 mg, 178 µmol) as a colourless oil and **4** in 92% NMR yield. **TLC**: R_f (*n*-hexane/EtOAc = 10:1) = 0.61; **^{1}H NMR** (400 MHz, $CDCl_3$) δ 7.48 – 7.42 (m, 2H, Ar*H*), 7.40 – 7.33 (m, 2H, Ar*H*), 6.70 (dd, *J* = 17.6, 10.9 Hz, 1H, C*H*=CH_2), 5.78 (dd, *J* = 17.6, 0.8 Hz, 1H, CH=CH_2), 5.31 (dd, *J* = 10.9, 0.8 Hz, 1H, CH=CH_2), 4.27 – 4.08 (m, 2H, $CO_2CH_2CH_3$), 2.59 (t, *J* = 7.2 Hz, 2H, CH_2CH_2C), 2.50 (s, 1H, C*H*CO_2), 1.72 – 1.60 (m, 2H, CH_2CH_2C), 1.49 – 1.37 (m, 2H, CH_2), 1.37 – 1.16 (m, 7H, CH_2 & $CO_2CH_2CH_3$), 0.96 – 0.81 (m, 3H, CH_3); **^{13}C NMR** (101 MHz, $CDCl_3$) δ 174.4, 138.3, 136.3, 132.1, 126.3, 121.7, 117.0, 115.4, 99.0, 90.6, 77.4, 60.7, 31.6, 29.0, 26.7, 26.1, 25.3, 22.7, 14.5, 14.2; **IR** (ν_{max}, cm^{-1}) 2955 (m), 2928 (m), 2859 (m), 2202 (w), 1714 (s), 1649 (m), 1602 (m), 1509 (w), 1466 (m), 1407 (m), 1376 (m), 1265 (s), 1202 (s), 1181 (s), 1092 (s), 1018 (s), 835 (m), 736 (s); **HRMS** (ESI/QTOF) m/z: $[M + H]^+$ Calcd for $C_{22}H_{27}O_2^+$ 323.2006; Found 323.2013.

5.1.4. Synthesis and characterization of ethyl 2-([1,1'-biphenyl]-4-ylethynyl)-3-hexylcycloprop-2-ene-1-carboxylate (3d)

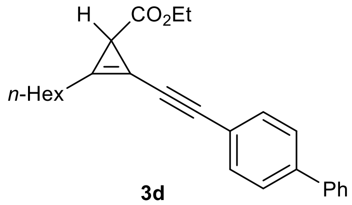

3d

Following **GPD**, a mixture of $(Me_2S)AuCl$ (2.95 mg, 10.0 μmol, 5.00 mol%), **L1** (4.20 mg, 20.0 μmol, 10.0 mol%), CpBX **1a** (113 mg, 0.200 mmol, 1.00 equiv.), terminal alkyne **2d** (35.7 mg, 0.200 mmol, 1.00 equiv.) and CH_3CN (2.0 mL) was stirred at room temperature for 3 hours. Flash column chromatography on silica gel (eluent: pentane/ethyl acetate = 50:1) afforded **3d** in 91% yield (67.6 mg, 181 μmol) as a colourless oil and **4** in 98% NMR yield. **TLC**: R_f (*n*-hexane/EtOAc = 10:1) = 0.46; ^{1}**H NMR** (400 MHz, $CDCl_3$) δ 7.63 – 7.52 (m, 6H, Ar*H*), 7.47 – 7.43 (m, 2H, Ar*H*), 7.39 – 7.34 (s, 1H, Ar*H*), 4.25 – 4.13 (m, 2H, $CO_2CH_2CH_3$), 2.61 (t, *J* = 7.2 Hz, 2H, CH_2CH_2C), 2.52 (s, 1H, $CHCO_2$), 1.72 – 1.62 (m, 2H, CH_2CH_2C), 1.47 – 1.38 (m, 2H, CH_2), 1.38 – 1.23 (m, 7H, CH_2 & $CO_2CH_2CH_3$), 1.03 – 0.81 (m, 3H, CH_3); ^{13}**C NMR** (101 MHz, $CDCl_3$) δ 174.4, 141.8, 140.3, 132.3, 129.0, 127.9, 127.2, 121.4, 117.0, 98.9, 90.6, 77.4, 60.7, 31.6, 29.0, 26.7, 26.1, 25.4, 22.7, 14.5, 14.2; **IR** (ν_{max}, cm^{-1}) 2979 (w), 2956 (m), 2928 (m), 2858 (m), 2196 (w), 1777 (w), 1711 (s), 1601 (m), 1486 (m), 1407 (w), 1267 (m), 1180 (m), 1094 (w), 1026 (m), 1007 (m), 842 (m), 764 (s); **HRMS** (ESI/QTOF) m/z: $[M + H]^+$ Calcd for $C_{26}H_{29}O_2^+$ 373.2162; Found 373.2160.

5.1.5. Synthesis and characterization of ethyl 2-((3,5-bis(trifluoromethyl)phenyl)ethynyl)-3-hexylcycloprop-2-ene-1-carboxylate (3e)

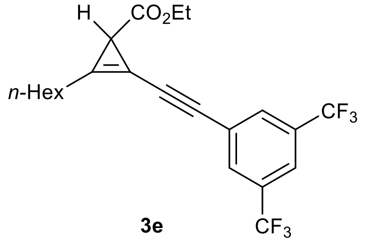

3e

Following **GPD**, a mixture of $(Me_2S)AuCl$ (2.95 mg, 10.0 μmol, 5.00 mol%), **L1** (4.20 mg, 20.0 μmol, 10.0 mol%), CpBX **1a** (113 mg, 0.200 mmol, 1.00 equiv.), terminal alkyne **2e** (47.6 mg, 0.200 mmol, 1.00 equiv.) and CH_3CN (2.0 mL) was stirred at room temperature for 6 hours. Flash column chromatography on silica gel (eluent: pentane/ethyl acetate = 50:1) afforded **3e** in 92% yield (79.7 mg, 184 μmol) as a colourless oil and **4** in 99% NMR yield. **TLC**: R_f (*n*-hexane/EtOAc = 10:1) = 0.68; ^{1}**H NMR** (400 MHz, $CDCl_3$) δ 7.94 – 7.87 (m, 2H, Ar*H*), 7.83 – 7.81 (m, 1H, Ar*H*), 4.18 (qd, *J* = 7.1, 4.1 Hz, 2H, $CO_2CH_2CH_3$), 2.62 (t, *J* = 7.3 Hz, 2H, CH_2CH_2C), 2.54 (s, 1H, $CHCO_2$), 1.75 – 1.58 (m, 2H, CH_2CH_2C), 1.52 – 1.37 (m, 2H, CH_2), 1.37 – 1.19 (m, 7H, CH_2 & $CO_2CH_2CH_3$), 0.96 – 0.79 (m, 3H, CH_3); ^{13}**C NMR** (101 MHz, $CDCl_3$) δ 173.8, 132.2 (q, *J* = 33.8 Hz), 131.7 (m), 124.9, 123.0 (q, *J* = 272.9 Hz), 122.4 (hept, *J* = 3.8 Hz), 120.5, 95.5, 89.9, 80.3, 60.8, 31.6, 28.9, 26.6, 26.3, 25.5, 22.7, 14.5, 14.1; ^{19}**F NMR** (376 MHz, $CDCl_3$) δ -63.3; **IR** (ν_{max}, cm^{-1}) 2961 (w), 2934 (w), 2863 (w), 2218 (w), 1725 (m), 1617 (w), 1465 (w), 1386 (m), 1277 (s), 1175 (s), 1133 (s), 1027 (w), 899 (m), 848 (w); **HRMS** (ESI/QTOF) m/z: $[M + H]^+$ Calcd for $C_{22}H_{23}F_6O_2^+$ 433.1597; Found 433.1597.

5.1.6. Synthesis and characterization of ethyl 2-hexyl-3-((4-(trifluoromethyl)phenyl)ethynyl)cycloprop-2-ene-1-carboxylate (3f)

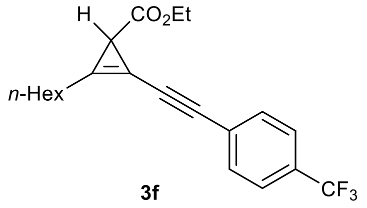

3f

Following **GPD**, a mixture of (Me_2S)AuCl (2.95 mg, 10.0 µmol, 5.00 mol%), **L1** (4.20 mg, 20.0 µmol, 10.0 mol%), CpBX **1a** (113 mg, 0.200 mmol, 1.00 equiv.), terminal alkyne **2f** (34.0 mg, 0.200 mmol, 1.00 equiv.) and CH_3CN (2.0 mL) was stirred at room temperature for 6 hours. Flash column chromatography on silica gel (eluent: pentane/ethyl acetate = 50:1) afforded **3f** in 94% yield (68.8 mg, 189 µmol) as a colorless oil and **4** in 98% NMR yield. **TLC**: R_f (*n*-hexane/EtOAc = 10:1) = 0.55; **^{1}H NMR** (400 MHz, $CDCl_3$) δ 7.59 (s, 4H, Ar*H*), 4.17 (qt, *J* = 6.9, 3.5 Hz, 2H, $CO_2CH_2CH_3$), 2.60 (t, *J* = 7.2 Hz, 2H, CH_2CH_2C), 2.53 (s, 1H, $CHCO_2$), 1.70 – 1.59 (m, 2H, CH_2CH_2C), 1.46 – 1.36 (m, 2H, CH_2), 1.36 – 1.20 (m, 7H, CH_2 & $CO_2CH_2CH_3$), 0.93 – 0.84 (m, 3H, CH_3); **^{13}C NMR** (101 MHz, $CDCl_3$) δ 174.1, 132.1, 130.7 (q, *J* = 32.7 Hz), 126.3, 125.5 (q, *J* = 3.8 Hz), 123.9 (q, *J* = 272.2 Hz), 118.9, 97.3, 90.2, 79.1, 60.7, 31.6, 28.9, 26.6, 26.2, 25.4, 22.6, 14.5, 14.1; **^{19}F NMR** (376 MHz, $CDCl_3$) δ -62.9; **IR** (ν_{max}, cm^{-1}) 2957 (w), 2932 (w), 2867 (w), 2208 (w), 1725 (m), 1617 (w), 1469 (w), 1407 (w), 1323 (s), 1167 (m), 1129 (s), 1106 (m), 1065 (s), 1017 (m), 841 (m); **HRMS** (ESI/QTOF) m/z: $[M + H]^+$ Calcd for $C_{21}H_{24}F_3O_2^+$ 365.1723; Found 365.1722.

5.1.7. Synthesis and characterization of ethyl 2-hexyl-3-((4-nitrophenyl)ethynyl)cycloprop-2-ene-1-carboxylate (3g)

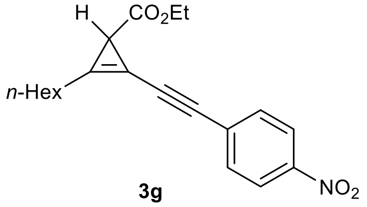

3g

Following **GPD**, a mixture of (Me_2S)AuCl (2.95 mg, 10.0 µmol, 5.00 mol%), **L1** (4.20 mg, 20.0 µmol, 10.0 mol%), CpBX **1a** (113 mg, 0.200 mmol, 1.00 equiv.), terminal alkyne **2g** (29.4 mg, 0.200 mmol, 1.00 equiv.) and CH_3CN (2.0 mL) was stirred at room temperature for 6 hours. Flash column chromatography on silica gel (eluent: pentane/ethyl acetate = 10:1) afforded **3g** in 87% yield (59.3 mg, 174 µmol) as a light-yellow oil and **4** in 88% NMR yield. **TLC**: R_f (*n*-hexane/EtOAc = 10:1) = 0.35; **^{1}H NMR** (400 MHz, $CDCl_3$) δ 8.24 – 8.15 (m, 2H, Ar*H*), 7.66 – 7.57 (m, 2H, Ar*H*), 4.23 – 4.12 (m, 2H, $CO_2CH_2CH_3$), 2.61 (t, *J* = 7.2 Hz, 2H, CH_2CH_2C), 2.54 (s, 1H, $CHCO_2$), 1.70 – 1.57 (m, 2H, CH_2CH_2C), 1.44 – 1.36 (m, 2H, CH_2), 1.36 – 1.20 (m, 7H, CH_2 & $CO_2CH_2CH_3$), 0.95 – 0.82 (m, 3H, CH_3); **^{13}C NMR** (101 MHz, $CDCl_3$) δ 173.8, 147.5, 132.6, 129.3, 123.8, 120.4, 96.7, 90.0, 81.8, 60.8, 31.5, 28.9, 26.6, 26.3, 25.5, 22.6, 14.4, 14.1; **IR** (ν_{max}, cm^{-1}) 2961 (m), 2928 (m), 2856 (w), 2211 (w), 1858 (w), 1724 (m), 1593 (m), 1520 (s), 1466 (w), 1341 (s), 1182 (m), 1108 (w), 1023 (w), 854 (s), 749 (s); **HRMS** (ESI/QTOF) m/z: $[M + H]^+$ Calcd for $C_{20}H_{24}NO_4^+$ 342.1700; Found 342.1698.

5.1.8. Synthesis and characterization of methyl 4-((3-(ethoxycarbonyl)-2-hexylcycloprop-1-en-1-yl)ethynyl)benzoate (3h)

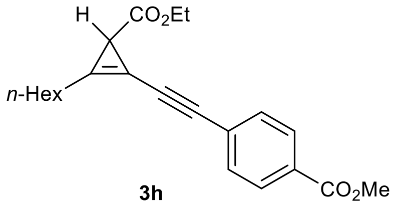

3h

Following **GPD**, a mixture of $(Me_2S)AuCl$ (2.95 mg, 10.0 μmol, 5.00 mol%), **L1** (4.20 mg, 20.0 μmol, 10.0 mol%), CpBX **1a** (113 mg, 0.200 mmol, 1.00 equiv.), terminal alkyne **2h** (32.0 mg, 0.200 mmol, 1.00 equiv.) and CH_3CN (2.0 mL) was stirred at room temperature for 8 hours. Flash column chromatography on silica gel (eluent: pentane/ethyl acetate = 10:1) afforded **3h** in 92% yield (65.5 mg, 185 μmol) as a colourless oil and **4** in 99% NMR yield. **TLC**: R_f (*n*-hexane/EtOAc = 10:1) = 0.36; **^{1}H NMR** (400 MHz, $CDCl_3$) δ 8.03 – 7.92 (m, 2H, Ar*H*), 7.58 – 7.46 (m, 2H, Ar*H*), 4.22 – 4.10 (m, 2H, $CO_2CH_2CH_3$), 3.90 (s, 3H, CO_2CH_3), 2.59 (t, *J* = 7.2 Hz, 2H, CH_2CH_2C), 2.51 (s, 1H, C*H*CO_2), 1.70 – 1.56 (m, 2H, CH_2CH_2C), 1.45 – 1.35 (m, 2H, CH_2), 1.35 – 1.19 (m, 7H, CH_2 & $CO_2CH_2CH_3$), 0.95 – 0.77 (m, 3H, CH_3); **^{13}C NMR** (101 MHz, $CDCl_3$) δ 174.0, 166.5, 131.7, 130.2, 129.6, 127.1, 118.7, 98.0, 90.3, 79.5, 60.7, 52.4, 31.5, 28.9, 26.6, 26.2, 25.4, 22.6, 14.4, 14.1; **IR** (ν_{max}, cm^{-1}) 2954 (m), 2932 (m), 2859 (w), 2204 (w), 1724 (s), 1605 (m), 1464 (w), 1436 (m), 1405 (w), 1371 (w), 1276 (s), 1177 (m), 1107 (m), 1018 (m), 861 (m), 769 (m); **HRMS** (ESI/QTOF) m/z: $[M + H]^+$ Calcd for $C_{22}H_{27}O_4^+$ 355.1904; Found 355.1902.

5.1.9. Synthesis and characterization of ethyl 2-((4-formylphenyl)ethynyl)-3-hexylcycloprop-2-ene-1-carboxylate (3i)

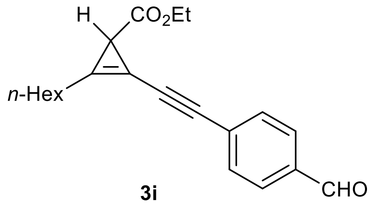

3i

Following **GPD**, a mixture of $(Me_2S)AuCl$ (2.95 mg, 10.0 μmol, 5.00 mol%), **L1** (4.20 mg, 20.0 μmol, 10.0 mol%), CpBX **1a** (113 mg, 0.200 mmol, 1.00 equiv.), terminal alkyne **2i** (26.0 mg, 0.200 mmol, 1.00 equiv.) and CH_3CN (2.0 mL) was stirred at room temperature for 4 hours. Flash column chromatography on silica gel (eluent: pentane/ethyl acetate = 20:1) afforded **3i** in 87% yield (56.5 mg, 174 μmol) as a colorless oil and **4** in 97% NMR yield. **TLC**: R_f (*n*-hexane/EtOAc = 10:1) = 0.26; **^{1}H NMR** (400 MHz, $CDCl_3$) δ 10.00 (s, 1H, C*H*O), 7.89 – 7.78 (m, 2H, Ar*H*), 7.67 – 7.54 (m, 2H, Ar*H*), 4.23 – 4.11 (m, 2H, $CO_2CH_2CH_3$), 2.60 (t, *J* = 7.3 Hz, 2H, CH_2CH_2C), 2.52 (s, 1H, C*H*CO_2), 1.71 – 1.57 (m, 2H, CH_2CH_2C), 1.44 – 1.36 (m, 2H, CH_2), 1.35 – 1.20 (m, 7H, CH_2 & $CO_2CH_2CH_3$), 0.95 – 0.76 (m, 3H, CH_3); **^{13}C NMR** (101 MHz, $CDCl_3$) δ 191.4, 174.0, 136.0, 132.4, 129.6, 128.6, 119.3, 97.8, 90.2, 80.5, 60.7, 31.5, 28.9, 26.6, 26.2, 25.4, 22.6, 14.4, 14.1; **IR** (ν_{max}, cm^{-1}) 2957 (w), 2930 (m), 2858 (w), 2203 (w), 1717 (m), 1702 (s), 1602 (m), 1562 (w), 1466 (w), 1411 (w), 1302 (m), 1266 (m), 1204 (s), 1095 (w), 1016 (m), 831 (m), 735 (s); **HRMS** (ESI/QTOF) m/z: $[M + H]^+$ Calcd for $C_{21}H_{25}O_3^+$ 325.1798; Found 325.1798.

5.1.10. Synthesis and characterization of 4-((3-(ethoxycarbonyl)-2-hexylcycloprop-1-en-1-yl)ethynyl)benzoic acid (3j)

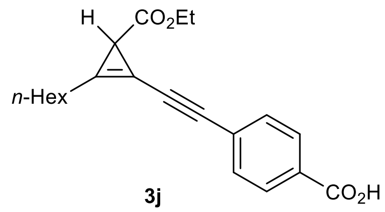

3j

Following **GPD**, a mixture of $(Me_2S)AuCl$ (2.95 mg, 10.0 μmol, 5.00 mol%), **L1** (4.20 mg, 20.0 μmol, 10.0 mol%), CpBX **1a** (113 mg, 0.200 mmol, 1.00 equiv.), terminal alkyne **2j** (29.2 mg, 0.200 mmol, 1.00 equiv.) and CH_3CN (2.0 mL) was stirred at room temperature for 9 hours. Flash column chromatography on silica gel (eluent: DCM/MeOH = 20:1) afforded **3j** in 86% yield (58.7 mg, 172 μmol) as a colourless solid and **4** in 95% NMR yield. **M.p.** 87 – 89 °C. **TLC**: R_f (DCM/MeOH = 20:1) = 0.27; **^{1}H NMR** (400 MHz, $CDCl_3$) δ 10.30 (bs, 1H, CO_2H), 8.07 (d, *J* = 8.0 Hz, 2H, Ar*H*), 7.56 (d, *J* = 7.9 Hz, 2H, Ar*H*), 4.25 – 4.13 (m, 2H, $CO_2CH_2CH_3$), 2.61 (t, *J* = 7.3 Hz, 2H, CH_2CH_2C), 2.54 (s, 1H, $CHCO_2$), 1.67 – 1.61 (m, 2H, CH_2CH_2C), 1.45 – 1.37 (m, 2H, CH_2), 1.33 – 1.25 (m, 7H, CH_2 & $CO_2CH_2CH_3$), 0.91 – 0.85 (m, 3H, CH_3); **^{13}C NMR** (101 MHz, $CDCl_3$) δ 174.2, 171.3, 131.8, 130.2, 129.5, 127.9, 118.9, 97.9, 90.3, 79.9, 60.8, 31.6, 28.9, 26.6, 26.2, 25.4, 22.6, 14.5, 14.1; **IR** (ν_{max}, cm^{-1}) 2957 (m), 2932 (m), 2853 (w), 1729 (s), 1689 (s), 1682 (s), 1604 (m), 1558 (w), 1465 (w), 1426 (m), 1316 (m), 1296 (s), 1280 (m), 1185 (s), 1017 (m), 952 (w), 859 (m), 775 (m); **HRMS** (ESI/QTOF) m/z: $[M + H]^+$ Calcd for $C_{21}H_{25}O_4^+$ 341.1747; Found 341.1748.

5.1.11. Synthesis and characterization of 3-((3-(ethoxycarbonyl)-2-hexylcycloprop-1-en-1-yl)ethynyl)benzoic acid (3k)

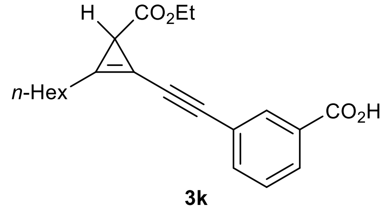

3k

Following **GPD**, a mixture of $(Me_2S)AuCl$ (2.95 mg, 10.0 μmol, 5.00 mol%), **L1** (4.20 mg, 20.0 μmol, 10.0 mol%), CpBX **1a** (113 mg, 0.200 mmol, 1.00 equiv.), terminal alkyne **2k** (29.2 mg, 0.200 mmol, 1.00 equiv.) and CH_3CN (2.0 mL) was stirred at room temperature for 6 hours. Flash column chromatography on silica gel (eluent: DCM/MeOH = 20:1) afforded **3k** in 78% yield (52.9 mg, 155 μmol) as a colourless oil and **4** in 90% NMR yield. **TLC**: R_f (DCM/MeOH = 20:1) = 0.31; **^{1}H NMR** (400 MHz, $CDCl_3$) δ 8.45 (bs, 1H, CO_2H), 8.23 (t, *J* = 1.7 Hz, 1H, Ar*H*), 8.07 (dt, *J* = 7.9, 1.4 Hz, 1H, Ar*H*), 7.69 (dt, *J* = 7.8, 1.4 Hz, 1H, Ar*H*), 7.45 (t, *J* = 7.8 Hz, 1H, Ar*H*), 4.25 – 4.13 (m, 2H, $CO_2CH_2CH_3$), 2.60 (t, *J* = 7.2 Hz, 2H, CH_2CH_2C), 2.53 (s, 1H, $CHCO_2$), 1.72 – 1.54 (m, 2H, CH_2CH_2C), 1.45 – 1.37 (m, 2H, CH_2), 1.36 – 1.25 (m, 7H, CH_2 & $CO_2CH_2CH_3$), 1.00 – 0.74 (m, 3H, CH_3); **^{13}C NMR** (101 MHz, $CDCl_3$) δ 174.4, 171.1, 136.6, 133.6, 130.6, 130.0, 128.8, 123.2, 118.1, 97.6, 90.4, 77.8, 60.8, 31.6, 28.9, 26.7, 26.2, 25.4, 22.6, 14.5, 14.2; **IR** (ν_{max}, cm^{-1}) 2958 (w), 2929 (m), 2859 (w), 2200 (w), 1722 (s), 1697 (s), 1603 (w), 1581 (w), 1444 (m), 1412 (m), 1372 (w), 1336 (w), 1300 (w), 1266 (w), 1186 (m), 1168 (m), 1096 (w), 1023 (w), 916 (w), 755 (m), 737 (s); **HRMS** (ESI/QTOF) m/z: $[M + H]^+$ Calcd for $C_{21}H_{25}O_4^+$ 341.1747; Found 341.1743.

5.1.12. Synthesis and characterization of ethyl 2-hexyl-3-((3-methoxyphenyl)ethynyl)cycloprop-2-ene-1-carboxylate (3l)

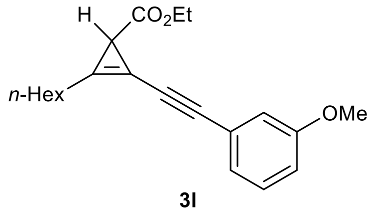

3l

Following **GPD**, a mixture of (Me_2S)AuCl (2.95 mg, 10.0 µmol, 5.00 mol%), **L1** (4.20 mg, 20.0 µmol, 10.0 mol%), CpBX **1a** (113 mg, 0.200 mmol, 1.00 equiv.), terminal alkyne **2l** (26.4 mg, 0.200 mmol, 1.00 equiv.) and CH_3CN (2.0 mL) was stirred at room temperature for 5 hours. Flash column chromatography on silica gel (eluent: pentane/ethyl acetate = 10:1) afforded **3l** in 90% yield (58.8 mg, 180 µmol) as a colorless oil and **4** in 98% NMR yield. **TLC**: R_f (*n*-hexane/EtOAc = 10:1) = 0.48; **^{1}H NMR** (400 MHz, $CDCl_3$) δ 7.27 – 7.18 (m, 1H, Ar*H*), 7.08 (dt, *J* = 7.6, 1.2 Hz, 1H, Ar*H*), 7.01 (dd, *J* = 2.7, 1.4 Hz, 1H, Ar*H*), 6.90 (ddd, *J* = 8.4, 2.6, 1.0 Hz, 1H, Ar*H*), 4.23 – 4.11 (m, 2H, $CO_2CH_2CH_3$), 3.79 (s, 3H, OCH_3), 2.58 (t, *J* = 7.2 Hz, 2H, CH_2CH_2C), 2.50 (s, 1H, $CHCO_2$), 1.70 – 1.55 (m, 2H, CH_2CH_2C), 1.40 (dq, *J* = 8.5, 6.7 Hz, 2H, CH_2), 1.36 – 1.22 (m, 7H, CH_2 & $CO_2CH_2CH_3$), 0.95 – 0.80 (m, 3H, CH_3); **^{13}C NMR** (101 MHz, $CDCl_3$) δ 174.3, 159.4, 129.5, 124.4, 123.4, 117.1, 116.4, 115.9, 98.9, 90.5, 76.5, 60.6, 55.4, 31.5, 28.9, 26.7, 26.1, 25.2, 22.6, 14.5, 14.1; **IR** (ν_{max}, cm^{-1}) 2956 (m), 2930 (m), 2858 (m), 2193 (w), 1858 (w), 1723 (s), 1597 (m), 1575 (m), 1489 (m), 1465 (m), 1321 (m), 1291 (m), 1213 (m), 1178 (s), 1094 (w), 1039 (s), 870 (m), 853 (m), 785 (s); **HRMS** (ESI/QTOF) m/z: $[M + H]^+$ Calcd for $C_{21}H_{27}O_3^+$ 327.1955; Found 327.1949.

5.1.13. Synthesis and characterization of ethyl 2-((3,5-dimethoxyphenyl)ethynyl)-3-hexylcycloprop-2-ene-1-carboxylate (3m)

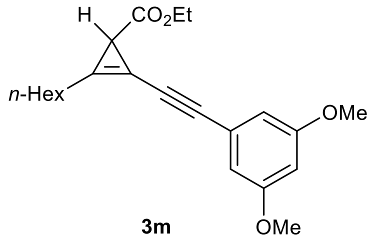

3m

Following **GPD**, a mixture of (Me_2S)AuCl (2.95 mg, 10.0 µmol, 5.00 mol%), **L1** (4.20 mg, 20.0 µmol, 10.0 mol%), CpBX **1a** (113 mg, 0.200 mmol, 1.00 equiv.), terminal alkyne **2m** (32.4 mg, 0.200 mmol, 1.00 equiv.) and CH_3CN (2.0 mL) was stirred at room temperature for 4 hours. Flash column chromatography on silica gel (eluent: pentane/ethyl acetate = 8:1) afforded **3m** in 92% yield (65.7 mg, 184 µmol) as a colorless oil and **4** in 99% NMR yield. **TLC**: R_f (*n*-hexane/EtOAc = 10:1) = 0.35; **^{1}H NMR** (400 MHz, $CDCl_3$) δ 6.64 (d, *J* = 2.3 Hz, 2H, Ar*H*), 6.46 (t, *J* = 2.3 Hz, 1H, Ar*H*), 4.22 – 4.11 (m, 2H, $CO_2CH_2CH_3$), 3.77 (s, 6H, OCH_3), 2.58 (t, *J* = 7.2 Hz, 2H, CH_2CH_2C), 2.49 (s, 1H, $CHCO_2$), 1.68 – 1.56 (m, 2H, CH_2CH_2C), 1.44 – 1.37 (m, 2H, CH_2), 1.35 – 1.24 (m, 7H, CH_2 & $CO_2CH_2CH_3$), 0.91 – 0.87 (m, 3H, CH_3); **^{13}C NMR** (101 MHz, $CDCl_3$) δ 174.3, 160.6, 123.7, 117.2, 109.5, 102.7, 99.0, 90.5, 76.2, 60.6, 55.5, 31.6, 28.9, 26.7, 26.1, 25.3, 22.6, 14.5, 14.1; **IR** (ν_{max}, cm^{-1}) 2957 (w), 2932 (m), 2858 (w), 2193 (w), 1855 (w), 1800 (w), 1721 (m), 1591 (s), 1458 (m), 1418 (m), 1345 (w), 1205 (s), 1182 (s), 1154 (s), 1063 (m), 1024 (w), 931 (w), 836 (m); **HRMS** (ESI/QTOF) m/z: $[M + H]^+$ Calcd for $C_{22}H_{29}O_4^+$ 357.2060; Found 357.2057.

5.1.14. Synthesis and characterization of ethyl 2-hexyl-3-((4-(trifluoromethoxy)phenyl)ethynyl)cycloprop-2-ene-1-carboxylate (3n)

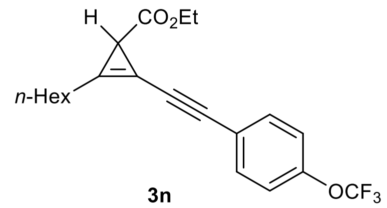

3n

Following **GPD**, a mixture of $(Me_2S)AuCl$ (2.95 mg, 10.0 µmol, 5.00 mol%), **L1** (4.20 mg, 20.0 µmol, 10.0 mol%), CpBX **1a** (113 mg, 0.200 mmol, 1.00 equiv.), terminal alkyne **2n** (37.2 mg, 0.200 mmol, 1.00 equiv.) and CH_3CN (2.0 mL) was stirred at room temperature for 4 hours. Flash column chromatography on silica gel (eluent: pentane/ethyl acetate = 20:1) afforded **3n** in 92% yield (69.7 mg, 183 µmol) as a colourless oil and **4** in 98% NMR yield. **TLC**: R_f (*n*-hexane/EtOAc = 10:1) = 0.58; **^{1}H NMR** (400 MHz, $CDCl_3$) δ 7.56 – 7.45 (m, 2H, Ar*H*), 7.22 – 7.10 (m, 2H, Ar*H*), 4.23 – 4.11 (m, 2H, $CO_2CH_2CH_3$), 2.59 (t, *J* = 7.3 Hz, 2H, CH_2CH_2C), 2.50 (s, 1H, $CHCO_2$), 1.68 – 1.57 (m, 2H, CH_2CH_2C), 1.44 – 1.36 (m, 2H, CH_2), 1.36 – 1.20 (m, 7H, CH_2 & $CO_2CH_2CH_3$), 0.97 – 0.77 (m, 3H, CH_3); **^{13}C NMR** (101 MHz, $CDCl_3$) δ 174.2, 149.5 (d, *J* = 2.0 Hz), 133.4, 121.3, 121.0, 120.5 (q, *J* = 258.0 Hz), 117.9, 97.4, 90.4, 77.6, 60.7, 31.6, 28.9, 26.7, 26.2, 25.3, 22.7, 14.5, 14.1; **^{19}F NMR** (376 MHz, $CDCl_3$) δ -57.8; **IR** (ν_{max}, cm^{-1}) 2960 (w), 2932 (w), 2863 (w), 2205 (w), 1725 (m), 1603 (w), 1507 (m), 1373 (w), 1253 (s), 1206 (s), 1163 (s), 1095 (w), 1018 (m), 923 (w), 853 (m); **HRMS** (ESI/QTOF) m/z: $[M + H]^+$ Calcd for $C_{21}H_{24}F_3O_3^+$ 381.1672; Found 381.1672.

5.1.15. Synthesis and characterization of ethyl 2-((4-(((benzyloxy)carbonyl)amino)phenyl)ethynyl)-3-hexylcycloprop-2-ene-1-carboxylate (3o)

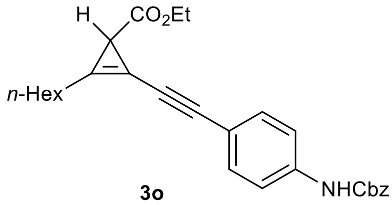

3o

Following **GPD**, a mixture of $(Me_2S)AuCl$ (2.95 mg, 10.0 µmol, 5.00 mol%), **L1** (4.20 mg, 20.0 µmol, 10.0 mol%), CpBX **1a** (113 mg, 0.200 mmol, 1.00 equiv.), terminal alkyne **2o** (50.3 mg, 0.200 mmol, 1.00 equiv.) and CH_3CN (2.0 mL) was stirred at room temperature for 6 hours. Flash column chromatography on silica gel (eluent: pentane/ethyl acetate = 20:1) afforded **3o** in 91% yield (81.0 mg, 182 µmol) as a colourless oil and **4** in 96% NMR yield. **TLC**: R_f (*n*-hexane/EtOAc = 10:1) = 0.14; **^{1}H NMR** (400 MHz, $CDCl_3$) δ 7.45 – 7.30 (m, 9H, Ar*H*), 7.21 (s, 1H, N*H*), 5.19 (s, 2H, OCH_2Ph), 4.22 – 4.09 (m, 2H, $CO_2CH_2CH_3$), 2.56 (t, *J* = 7.2 Hz, 2H, CH_2CH_2C), 2.50 (s, 1H, $CHCO_2$), 1.66 – 1.58 (m, 2H, CH_2CH_2C), 1.48 – 1.12 (m, 9H, CH_2 & $CO_2CH_2CH_3$), 1.02 – 0.75 (m, 3H, CH_3); **^{13}C NMR** (101 MHz, $CDCl_3$) δ 174.6, 153.2, 138.9, 136.0, 132.8, 128.7, 128.5, 128.4, 118.3, 117.0, 116.3, 99.0, 90.5, 76.1, 67.2, 60.7, 31.5, 28.9, 26.7, 26.0, 25.2, 22.6, 14.4, 14.1; **IR** (ν_{max}, cm^{-1}) 3336 (w), 2957 (w), 2929 (w), 2869 (w), 2858 (w), 2193 (w), 1734 (m), 1704 (s), 1590 (m), 1524 (s), 1456 (w), 1410 (m), 1315 (m), 1210 (s), 1179 (s), 1047 (s), 837 (m), 742 (m); **HRMS** (ESI/QTOF) m/z: $[M + H]^+$ Calcd for $C_{28}H_{32}NO_4^+$ 446.2326; Found 446.2327.

5.1.16. Synthesis and characterization of ethyl 2-((3,4-dichlorophenyl)ethynyl)-3-hexylcycloprop-2-ene-1-carboxylate (3p)

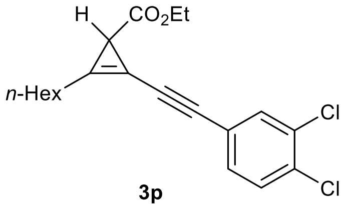

3p

Following **GPD**, a mixture of (Me_2S)AuCl (2.95 mg, 10.0 μmol, 5.00 mol%), **L1** (4.20 mg, 20.0 μmol, 10.0 mol%), CpBX **1a** (113 mg, 0.200 mmol, 1.00 equiv.), terminal alkyne **2p** (34.2 mg, 0.200 mmol, 1.00 equiv.) and CH_3CN (2.0 mL) was stirred at room temperature for 4 hours. Flash column chromatography on silica gel (eluent: pentane/ethyl acetate = 40:1) afforded **3p** in 91% yield (66.4 mg, 182 μmol) as a colourless oil and **4** in 98% NMR yield. **TLC**: R_f (*n*-hexane/EtOAc = 10:1) = 0.59; **^{1}H NMR** (400 MHz, $CDCl_3$) δ 7.56 (d, *J* = 1.9 Hz, 1H, Ar*H*), 7.40 (d, *J* = 8.3 Hz, 1H, Ar*H*), 7.29 (dd, *J* = 8.3, 1.9 Hz, 1H, Ar*H*), 4.23 – 4.11 (m, 2H, $CO_2CH_2CH_3$), 2.59 (t, *J* = 7.3 Hz, 2H, CH_2CH_2C), 2.50 (s, 1H, C*H*CO_2), 1.67 – 1.59 (m, 2H, CH_2CH_2C), 1.44 – 1.36 (m, 2H, CH_2), 1.35 – 1.25 (m, 7H, CH_2 & $CO_2CH_2CH_3$), 0.94 – 0.81 (m, 3H, CH_3); **^{13}C NMR** (101 MHz, $CDCl_3$) δ 174.0, 133.6, 133.4, 132.8, 130.9, 130.6, 122.5, 118.8, 96.4, 90.1, 78.7, 60.7, 31.5, 28.9, 26.6, 26.2, 25.3, 22.6, 14.5, 14.1; **IR** (ν_{max}, cm^{-1}) 2958 (m), 2929 (m), 2858 (m), 2206 (w), 1725 (s), 1587 (w), 1466 (s), 1375 (m), 1248 (m), 1182 (s), 1132 (s), 1031 (s), 880 (m), 822 (m); **HRMS** (ESI/QTOF) m/z: $[M + H]^+$ Calcd for $C_{20}H_{23}Cl_2O_2^+$ 365.1070; Found 365.1066.

5.1.17. Synthesis and characterization of ethyl 2-((4-bromophenyl)ethynyl)-3-hexylcycloprop-2-ene-1-carboxylate (3q)

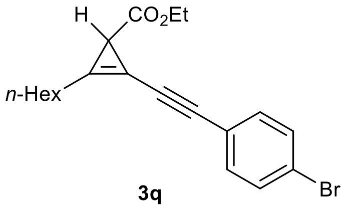

3q

Following **GPD**, a mixture of (Me_2S)AuCl (2.95 mg, 10.0 μmol, 5.00 mol%), **L1** (4.20 mg, 20.0 μmol, 10.0 mol%), CpBX **1a** (113 mg, 0.200 mmol, 1.00 equiv.), terminal alkyne **2q** (36.2 mg, 0.200 mmol, 1.00 equiv.) and CH_3CN (2.0 mL) was stirred at room temperature for 4 hours. Flash column chromatography on silica gel (eluent: pentane/ethyl acetate = 50:1) afforded **3q** in 88% yield (65.7 mg, 175 μmol) as a colourless oil and **4** in 89% NMR yield. **TLC**: R_f (*n*-hexane/EtOAc = 10:1) = 0.47; **^{1}H NMR** (400 MHz, $CDCl_3$) δ 7.50 – 7.42 (m, 2H, Ar*H*), 7.40 – 7.30 (m, 2H, Ar*H*), 4.23 – 4.11 (m, 2H, $CO_2CH_2CH_3$), 2.58 (t, *J* = 7.2 Hz, 2H, CH_2CH_2C), 2.49 (s, 1H, C*H*CO_2), 1.69 – 1.55 (m, 2H, CH_2CH_2C), 1.44 – 1.36 (m, 2H, CH_2), 1.35 – 1.25 (m, 7H, CH_2 & $CO_2CH_2CH_3$), 0.94 – 0.79 (m, 3H, CH_3); **^{13}C NMR** (101 MHz, $CDCl_3$) δ 174.2, 133.2, 131.8, 123.5, 121.5, 117.8, 97.8, 90.4, 77.9, 60.7, 31.5, 28.9, 26.6, 26.1, 25.3, 22.6, 14.5, 14.1; **IR** (ν_{max}, cm^{-1}) 2957 (m), 2932 (m), 2859 (m), 2201 (w), 1723 (s), 1584 (m), 1486 (m), 1467 (m), 1395 (m), 1369 (m), 1248 (m), 1184 (s), 1094 (m), 1070 (s), 1028 (m), 1010 (s), 823 (s), 737 (w); **HRMS** (ESI/QTOF) m/z: $[M + H]^+$ Calcd for $C_{20}H_{24}BrO_2^+$ 375.0954; Found 375.0953.

5.1.18. Synthesis and characterization of ethyl 2-hexyl-3-((4-iodophenyl)ethynyl)cycloprop-2-ene-1-carboxylate (3r)

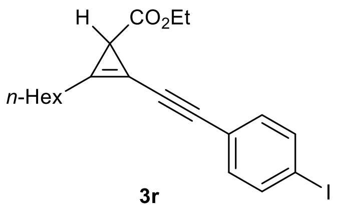

3r

Following **GPD**, a mixture of (Me_2S)AuCl (2.95 mg, 10.0 μmol, 5.00 mol%), **L1** (4.20 mg, 20.0 μmol, 10.0 mol%), CpBX **1a** (113 mg, 0.200 mmol, 1.00 equiv.), terminal alkyne **2r** (45.6 mg, 0.200 mmol, 1.00 equiv.) and CH_3CN (2.0 mL) was stirred at room temperature for 3 hours. Flash column chromatography on silica gel (eluent: pentane/ethyl acetate = 50:1) afforded **3r** in 92% yield (77.5 mg, 184 μmol) as a colourless oil and **4** in 98% NMR yield. **TLC**: R_f (*n*-hexane/EtOAc = 10:1) = 0.46; **^{1}H NMR** (400 MHz, $CDCl_3$) δ 7.72 – 7.60 (m, 2H, Ar*H*), 7.23 – 7.13 (m, 2H, Ar*H*), 4.22 – 4.11 (m, 2H, $CO_2CH_2CH_3$), 2.58 (t, *J* = 7.2 Hz, 2H, CH_2CH_2C), 2.49 (s, 1H, C*H*CO_2), 1.71 – 1.54 (m, 2H, CH_2CH_2C), 1.44 – 1.36 (m, 2H, CH_2), 1.35 – 1.23 (m, 7H, CH_2 & $CO_2CH_2CH_3$), 0.95 – 0.78 (m, 3H, CH_3); **^{13}C NMR** (101 MHz, $CDCl_3$) δ 174.1, 137.7, 133.2, 122.0, 117.8, 98.0, 95.3, 90.4, 78.1, 60.6, 31.5, 28.9, 26.6, 26.2, 25.3, 22.6, 14.5, 14.1; **IR** (ν_{max}, cm^{-1}) 2954 (m), 2929 (m), 2857 (m), 2200 (w), 1722 (s), 1601 (w), 1580 (m), 1483 (m), 1466 (m), 1391 (m), 1369 (w), 1264 (m), 1181 (m), 1095 (m), 1057 (m), 1032 (m), 1006 (s), 819 (s), 737 (m); **HRMS** (ESI/QTOF) m/z: $[M + H]^+$ Calcd for $C_{20}H_{24}IO_2^+$ 423.0816; Found 423.0809.

5.1.19. Synthesis and characterization of ethyl 2-hexyl-3-((4-(trimethylsilyl)phenyl)ethynyl)cycloprop-2-ene-1-carboxylate (3s)

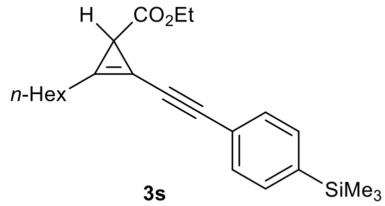

3s

Following **GPD**, a mixture of (Me_2S)AuCl (2.95 mg, 10.0 μmol, 5.00 mol%), **L1** (4.20 mg, 20.0 μmol, 10.0 mol%), CpBX **1a** (113 mg, 0.200 mmol, 1.00 equiv.), terminal alkyne **2s** (34.9 mg, 0.200 mmol, 1.00 equiv.) and CH_3CN (2.0 mL) was stirred at room temperature for 3 hours. Flash column chromatography on silica gel (eluent: pentane/ethyl acetate = 50:1) afforded **3s** in 84% yield (61.8 mg, 168 μmol) as a colourless oil and **4** in 92% NMR yield. **TLC**: R_f (*n*-hexane/EtOAc = 10:1) = 0.50; **^{1}H NMR** (400 MHz, $CDCl_3$) δ 7.53 – 7.41 (m, 4H, Ar*H*), 4.24 – 4.12 (m, 2H, $CO_2CH_2CH_3$), 2.59 (t, *J* = 7.2 Hz, 2H, CH_2CH_2C), 2.50 (s, 1H, C*H*CO_2), 1.70 – 1.57 (m, 2H, CH_2CH_2C), 1.45 – 1.37 (m, 2H, CH_2), 1.36 – 1.21 (m, 7H, CH_2 & $CO_2CH_2CH_3$), 0.98 – 0.84 (m, 3H, CH_3), 0.27 (s, 9H, $Si(CH_3)_3$); **^{13}C NMR** (101 MHz, $CDCl_3$) δ 174.4, 142.2, 133.3, 130.9, 122.7, 116.9, 99.2, 90.7, 77.0, 60.6, 31.6, 28.9, 26.7, 26.1, 25.3, 22.7, 14.5, 14.2, -1.1; **IR** (ν_{max}, cm^{-1}) 2956 (m), 2933 (m), 2860 (w), 2201 (w), 1724 (m), 1601 (w), 1467 (w), 1392 (w), 1250 (m), 1185 (m), 1109 (w), 1095 (w), 1030 (w), 842 (s), 822 (s), 757 (m); **HRMS** (ESI/QTOF) m/z: $[M + H]^+$ Calcd for $C_{23}H_{33}O_2Si^+$ 369.2244; Found 369.2247.

5.1.20. Synthesis and characterization of ethyl 2-hexyl-3-((4-(triethylgermyl)phenyl)ethynyl)cycloprop-2-ene-1-carboxylate (3t)

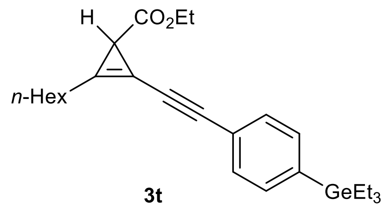

3t

Following **GPD**, a mixture of $(Me_2S)AuCl$ (2.95 mg, 10.0 μmol, 5.00 mol%), **L1** (4.20 mg, 20.0 μmol, 10.0 mol%), CpBX **1a** (113 mg, 0.200 mmol, 1.00 equiv.), terminal alkyne **2t** (52.2 mg, 0.200 mmol, 1.00 equiv.) and CH_3CN (2.0 mL) was stirred at room temperature for 3 hours. Flash column chromatography on silica gel (eluent: pentane/ethyl acetate = 50:1) afforded **3t** in 76% yield (68.9 mg, 151 μmol) as a colourless oil and **4** in 97% NMR yield. **TLC**: R_f (*n*-hexane/EtOAc = 10:1) = 0.50; ^{1}**H NMR** (400 MHz, $CDCl_3$) δ 7.47 – 7.36 (m, 4H, Ar*H*), 4.17 (qq, *J* = 7.0, 3.7 Hz, 2H, $CO_2CH_2CH_3$), 2.59 (t, *J* = 7.2 Hz, 2H, CH_2CH_2C), 2.50 (s, 1H, C*H*CO_2), 1.74 – 1.57 (m, 2H, CH_2CH_2C), 1.47 – 1.37 (m, 2H, CH_2), 1.37 – 1.22 (m, 7H, CH_2 & $CO_2CH_2CH_3$), 1.09 – 0.93 (m, 15H, $Ge(CH_2CH_3)_3$), 0.93 – 0.85 (m, 3H, CH_3); ^{13}**C NMR** (101 MHz, $CDCl_3$) δ 174.4, 142.1, 134.0, 130.9, 122.1, 116.7, 99.3, 90.7, 76.8, 60.6, 31.6, 28.9, 26.7, 26.1, 25.3, 22.7, 14.5, 14.2, 9.0, 4.3; **IR** (ν_{max}, cm^{-1}) 2954 (s), 2930 (s), 2908 (s), 2871 (s), 2198 (m), 1725 (s), 1602 (m), 1463 (m), 1427 (m), 1183 (s), 1084 (m), 1018 (s), 972 (w), 819 (s); **HRMS** (ESI/QTOF) m/z: $[M + H]^+$ Calcd for $C_{26}H_{39}GeO_2^+$ 457.2156; Found 457.2159.

5.1.21. Synthesis and characterization of ethyl 2-hexyl-3-((4-(4,4,5,5-tetraethyl-1,3,2-dioxaborolan-2-yl)phenyl)ethynyl)cycloprop-2-ene-1-carboxylate (3u)

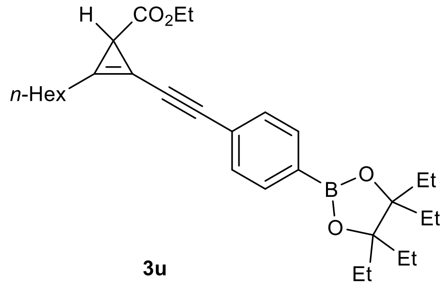

3u

Following **GPD**, a mixture of $(Me_2S)AuCl$ (2.95 mg, 10.0 μmol, 5.00 mol%), **L1** (4.20 mg, 20.0 μmol, 10.0 mol%), CpBX **1a** (113 mg, 0.200 mmol, 1.00 equiv.), terminal alkyne **2u** (56.8 mg, 0.200 mmol, 1.00 equiv.) and CH_3CN (2.0 mL) was stirred at room temperature for 4 hours. Flash column chromatography on silica gel (eluent: pentane/ethyl acetate = 40:1) afforded **3u** in 96% yield (92.2 mg, 193 μmol) as a colourless oil and **4** in 98% NMR yield. **TLC**: R_f (*n*-hexane/EtOAc = 10:1) = 0.45; ^{1}**H NMR** (400 MHz, $CDCl_3$) δ 7.78 (d, *J* = 8.1 Hz, 2H, Ar*H*), 7.47 (d, *J* = 8.2 Hz, 2H, Ar*H*), 4.23 – 4.12 (m, 2H, $CO_2CH_2CH_3$), 2.59 (t, *J* = 7.2 Hz, 2H, CH_2CH_2C), 2.50 (s, 1H, C*H*CO_2), 1.85 – 1.59 (m, 10H, CH_2), 1.47 – 1.36 (m, 2H, CH_2), 1.36 – 1.22 (m, 7H, CH_2 & $CO_2CH_2CH_3$), 0.96 (t, *J* = 7.4 Hz, 12H, CH_3), 0.93 – 0.85 (m, 3H, CH_3); ^{13}**C NMR** (101 MHz, $CDCl_3$) δ 174.3, 134.8, 131.0, 124.9, 117.4, 99.2, 90.6, 89.2, 77.8, 60.6, 31.6, 29.0, 26.7, 26.6, 26.2, 25.4, 22.7, 14.5, 14.2, 9.0; The carbon attached to boron was not observed due to quadrupolar relaxation; ^{11}**B NMR** (128 MHz, $CDCl_3$) δ 29.3; **IR** (ν_{max}, cm^{-1}) 2979 (m), 2937 (m), 2885 (m), 2200 (w), 1727 (m), 1647 (w), 1606 (m), 1512 (w), 1460 (m), 1400 (s), 1366 (s), 1352 (s), 1312 (m), 1291 (m), 1263 (m), 1181 (m), 1091 (s), 1021 (m), 921 (s), 836 (m); **HRMS** (ESI/QTOF) m/z: $[M + H]^+$ Calcd for $C_{30}H_{44}BO_4^+$ 479.3327; Found 479.3328.

5.1.22. Synthesis and characterization of ethyl 2-hexyl-3-(naphthalen-2-ylethynyl)cycloprop-2-ene-1-carboxylate (3v)

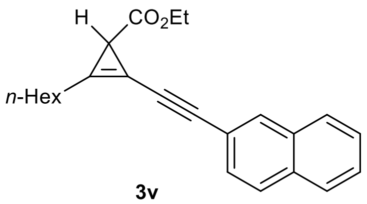

3v

Following **GPD**, a mixture of $(Me_2S)AuCl$ (2.95 mg, 10.0 μmol, 5.00 mol%), **L1** (4.20 mg, 20.0 μmol, 10.0 mol%), CpBX **1a** (113 mg, 0.200 mmol, 1.00 equiv.), terminal alkyne **2v** (30.4 mg, 0.200 mmol, 1.00 equiv.) and CH_3CN (2.0 mL) was stirred at room temperature for 4 hours. Flash column chromatography on silica gel (eluent: pentane/ethyl acetate = 50:1) afforded **3v** in 88% yield (60.9 mg, 176 μmol) as a colourless oil and **4** in 99% NMR yield. **TLC**: R_f (*n*-hexane/EtOAc = 10:1) = 0.51; **^{1}H NMR** (400 MHz, $CDCl_3$) δ 8.03 (d, *J* = 1.6 Hz, 1H, Ar*H*), 7.84 – 7.77 (m, 3H, Ar*H*), 7.57 – 7.46 (m, 3H, Ar*H*), 4.26 – 4.14 (m, 2H, $CO_2CH_2CH_3$), 2.62 (t, *J* = 7.2 Hz, 2H, CH_2CH_2C), 2.55 (s, 1H, $CHCO_2$), 1.73 – 1.60 (m, 2H, CH_2CH_2C), 1.49 – 1.39 (m, 2H, CH_2), 1.39 – 1.21 (m, 7H, CH_2 & $CO_2CH_2CH_3$), 0.96 – 0.84 (m, 3H, CH_3); **^{13}C NMR** (101 MHz, $CDCl_3$) δ 174.3, 133.2, 133.0, 132.1, 128.23, 128.20, 128.0, 127.9, 127.2, 126.8, 119.8, 117.1, 99.4, 90.7, 77.0, 60.6, 31.6, 29.0, 26.7, 26.2, 25.4, 22.7, 14.5, 14.2; **IR** (ν_{max}, cm^{-1}) 3059 (w), 2955 (m), 2928 (s), 2857 (m), 2188 (w), 1854 (w), 1792 (w), 1723 (s), 1597 (m), 1502 (w), 1465 (m), 1368 (m), 1332 (w), 1246 (m), 1180 (s), 1095 (w), 1022 (m), 954 (w), 894 (m), 859 (s), 817 (s), 747 (s); **HRMS** (ESI/QTOF) m/z: $[M + H]^+$ Calcd for $C_{24}H_{27}O_2^+$ 347.2006; Found 347.2006.

5.1.23. Synthesis and characterization of ethyl 2-hexyl-3-(phenanthren-9-ylethynyl)cycloprop-2-ene-1-carboxylate (3w)

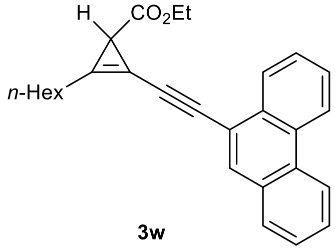

3w

Following **GPD**, a mixture of $(Me_2S)AuCl$ (2.95 mg, 10.0 μmol, 5.00 mol%), **L1** (4.20 mg, 20.0 μmol, 10.0 mol%), CpBX **1a** (113 mg, 0.200 mmol, 1.00 equiv.), terminal alkyne **2w** (40.5 mg, 0.200 mmol, 1.00 equiv.) and CH_3CN (2.0 mL) was stirred at room temperature for 2 hours. Flash column chromatography on silica gel (eluent: pentane/ethyl acetate = 50:1) afforded **3w** in 94% yield (74.3 mg, 187 μmol) as a colourless oil and **4** in 96% NMR yield. **TLC**: R_f (*n*-hexane/EtOAc = 20:1) = 0.48; **^{1}H NMR** (400 MHz, $CDCl_3$) δ 8.74 – 8.57 (m, 2H, Ar*H*), 8.47 – 8.37 (m, 1H, Ar*H*), 8.07 (s, 1H, Ar*H*), 7.86 (dd, *J* = 7.9, 1.5 Hz, 1H, Ar*H*), 7.74 – 7.63 (m, 3H, Ar*H*), 7.62 – 7.58 (m, 1H, Ar*H*), 4.29 – 4.18 (m, 2H, $CO_2CH_2CH_3$), 2.72 – 2.65 (m, 2H, CH_2CH_2C), 2.64 (s, 1H, $CHCO_2$), 1.79 – 1.68 (m, 2H, CH_2CH_2C), 1.59 – 1.43 (m, 2H, CH_2), 1.43 – 1.23 (m, 7H, CH_2 & $CO_2CH_2CH_3$), 1.02 – 0.85 (m, 3H, CH_3); **^{13}C NMR** (101 MHz, $CDCl_3$) δ 174.3, 132.8, 131.1, 130.9, 130.7, 130.1, 128.9, 128.0, 127.34, 127.27, 127.1, 127.0, 122.9, 122.8, 119.0, 117.5, 97.4, 90.7, 81.0, 60.7, 31.6, 29.0, 26.8, 26.3, 25.6, 22.7, 14.5, 14.2; **IR** (ν_{max}, cm^{-1}) 3062 (w), 2955 (m), 2928 (m), 2857 (m), 2189 (w), 1854 (w), 1720 (m), 1604 (w), 1451 (m), 1369 (w), 1242 (w), 1177 (m), 1029 (m), 892 (w), 763 (m), 748 (s), 724 (s); **HRMS** (ESI/QTOF) m/z: $[M + H]^+$ Calcd for $C_{28}H_{29}O_2^+$ 397.2162; Found 397.2165.

5.1.24. Synthesis and characterization of ethyl 2-hexyl-3-(thiophen-3-ylethynyl)cycloprop-2-ene-1-carboxylate (3x)

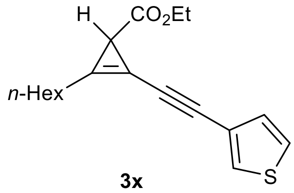

3x

Following **GPD**, a mixture of (Me_2S)AuCl (2.95 mg, 10.0 μmol, 5.00 mol%), **L1** (4.20 mg, 20.0 μmol, 10.0 mol%), CpBX **1a** (113 mg, 0.200 mmol, 1.00 equiv.), terminal alkyne **2x** (21.6 mg, 0.200 mmol, 1.00 equiv.) and CH_3CN (2.0 mL) was stirred at room temperature for 3 hours. Flash column chromatography on silica gel (eluent: pentane/ethyl acetate = 50:1) afforded **3x** in 94% yield (56.7 mg, 188 μmol) as a colourless oil and **4** in 98% NMR yield. **TLC**: R_f (*n*-hexane/EtOAc = 20:1) = 0.34; **^{1}H NMR** (400 MHz, $CDCl_3$) δ 7.53 (dd, *J* = 3.0, 1.1 Hz, 1H, Ar*H*), 7.28 (dd, *J* = 5.0, 3.0 Hz, 1H, Ar*H*), 7.15 (dd, *J* = 5.0, 1.1 Hz, 1H, Ar*H*), 4.23 – 4.11 (m, 2H, $CO_2CH_2CH_3$), 2.57 (t, *J* = 7.3 Hz, 2H, CH_2CH_2C), 2.49 (s, 1H, C*H*CO_2), 1.67 – 1.59 (m, 2H, CH_2CH_2C), 1.44 – 1.37 (m, 2H, CH_2), 1.35 – 1.21 (m, 7H, CH_2 & $CO_2CH_2CH_3$), 0.95 – 0.83 (m, 3H, CH_3); **^{13}C NMR** (101 MHz, $CDCl_3$) δ 174.4, 130.0, 129.9, 125.7, 121.7, 116.7, 94.2, 90.5, 76.4, 60.6, 31.6, 28.9, 26.7, 26.1, 25.3, 22.7, 14.5, 14.2; **IR** (ν_{max}, cm^{-1}) 3110 (w), 2957 (m), 2930 (s), 2870 (m), 2858 (m), 2196 (w), 1722 (s), 1599 (w), 1514 (w), 1466 (m), 1370 (w), 1248 (m), 1211 (m), 1182 (s), 1096 (w), 1026 (m), 870 (m), 783 (s); **HRMS** (ESI/QTOF) m/z: [M + H]$^+$ Calcd for $C_{18}H_{23}O_2S^+$ 303.1413; Found 303.1406.

5.1.25. Synthesis and characterization of ethyl 2-hexyl-3-(pyridin-3-ylethynyl)cycloprop-2-ene-1-carboxylate (3y)

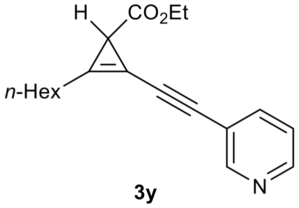

3y

Following **GPD**, a mixture of (Me_2S)AuCl (2.95 mg, 10.0 μmol, 5.00 mol%), **L1** (4.20 mg, 20.0 μmol, 10.0 mol%), CpBX **1a** (113 mg, 0.200 mmol, 1.00 equiv.), terminal alkyne **2y** (20.6 mg, 0.200 mmol, 1.00 equiv.) and CH_3CN (2.0 mL) was stirred at room temperature for 11 hours. Flash column chromatography on silica gel (eluent: pentane/ethyl acetate = 10:1) afforded **3y** in 88% yield (52.3 mg, 176 μmol) as a colourless oil and **4** in 94% NMR yield. **TLC**: R_f (*n*-hexane/EtOAc = 4:1) = 0.34; **^{1}H NMR** (400 MHz, $CDCl_3$) δ 8.70 (dd, *J* = 2.2, 0.9 Hz, 1H, Ar*H*), 8.53 (dd, *J* = 4.9, 1.7 Hz, 1H, Ar*H*), 7.75 (dt, *J* = 7.9, 1.9 Hz, 1H, Ar*H*), 7.29 – 7.23 (m, 1H, Ar*H*), 4.22 – 4.10 (m, 2H, $CO_2CH_2CH_3$), 2.59 (t, *J* = 7.2 Hz, 2H, CH_2CH_2C), 2.51 (s, 1H, C*H*CO_2), 1.70 – 1.53 (m, 2H, CH_2CH_2C), 1.43 – 1.35 (m, 2H, CH_2), 1.34 – 1.20 (m, 7H, CH_2 & $CO_2CH_2CH_3$), 0.93 – 0.80 (m, 3H, CH_3); **^{13}C NMR** (101 MHz, $CDCl_3$) δ 174.0, 152.4, 149.2, 138.7, 123.2, 119.8, 118.8, 95.4, 90.1, 80.0, 60.7, 31.5, 28.9, 26.6, 26.2, 25.3, 22.6, 14.4, 14.1; **IR** (ν_{max}, cm^{-1}) 2957 (m), 2928 (m), 2856 (m), 2213 (w), 1856 (w), 1724 (s), 1476 (m), 1407 (m), 1368 (w), 1334 (w), 1247 (m), 1180 (s), 1096 (w), 1022 (m), 805 (m), 703 (s); **HRMS** (ESI/QTOF) m/z: [M + H]$^+$ Calcd for $C_{19}H_{24}NO_2^+$ 298.1802; Found 298.1802.

5.1.26. Synthesis and characterization of ethyl 2-(cyclohex-1-en-1-ylethynyl)-3-hexylcycloprop-2-ene-1-carboxylate (3z)

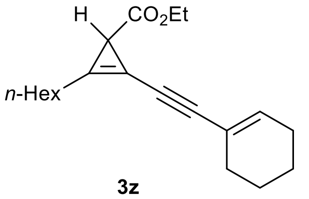

3z

Following **GPD**, a mixture of $(Me_2S)AuCl$ (2.95 mg, 10.0 μmol, 5.00 mol%), **L1** (4.20 mg, 20.0 μmol, 10.0 mol%), CpBX **1a** (113 mg, 0.200 mmol, 1.00 equiv.), terminal alkyne **2z** (21.2 mg, 0.200 mmol, 1.00 equiv.) and CH_3CN (2.0 mL) was stirred at room temperature for 4 hours. Flash column chromatography on silica gel (eluent: pentane/ethyl acetate = 50:1) afforded **3z** in 90% yield (54.3 mg, 181 μmol) as a colourless oil and **4** in 98% NMR yield. **TLC**: R_f (*n*-hexane/EtOAc = 20:1) = 0.33; **^{1}H NMR** (400 MHz, $CDCl_3$) δ 6.21 (tt, *J* = 3.8, 1.7 Hz, 1H, C=C*H*), 4.19 – 4.07 (m, 2H, $CO_2CH_2CH_3$), 2.51 (t, *J* = 7.2 Hz, 2H, CH_2CH_2C), 2.39 (s, 1H, C*H*CO_2), 2.17 – 2.09 (m, 4H, C*H*$_2$), 1.66 – 1.54 (m, 6H, C*H*$_2$), 1.44 – 1.16 (m, 9H, C*H*$_2$ & $CO_2CH_2CH_3$), 0.95 – 0.74 (m, 3H, C*H*$_3$); **^{13}C NMR** (101 MHz, $CDCl_3$) δ 174.5, 137.3, 120.5, 115.1, 101.0, 90.7, 74.0, 60.5, 31.6, 28.9, 28.8, 26.7, 25.9, 25.1, 22.6, 22.3, 21.5, 14.5, 14.1; **IR** (ν_{max}, cm^{-1}) 2953 (m), 2935 (m), 2860 (m), 2193 (w), 1719 (s), 1462 (m), 1371 (m), 1242 (m), 1182 (s), 1095 (m), 1031 (m), 862 (w), 736 (m); **HRMS** (ESI/QTOF) m/z: $[M + H]^+$ Calcd for $C_{20}H_{29}O_2^+$ 301.2162; Found 301.2161.

5.1.27. Synthesis and characterization of ethyl 2-hexyl-3-(oct-1-yn-1-yl)cycloprop-2-ene-1-carboxylate (3aa)

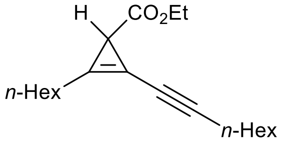

3aa

Following **GPD**, a mixture of $(Me_2S)AuCl$ (2.95 mg, 10.0 μmol, 5.00 mol%), **L1** (4.20 mg, 20.0 μmol, 10.0 mol%), CpBX **1a** (113 mg, 0.200 mmol, 1.00 equiv.), terminal alkyne **2aa** (22.0 mg, 0.200 mmol, 1.00 equiv.) and CH_3CN (2.0 mL) was stirred at room temperature for 6 hours. Flash column chromatography on silica gel (eluent: pentane/ethyl acetate = 50:1) afforded **3aa** in 94% yield (57.4 mg, 189 μmol) as a colourless oil and **4** in 95% NMR yield. **TLC**: R_f (*n*-hexane/EtOAc = 10:1) = 0.63; **^{1}H NMR** (400 MHz, $CDCl_3$) δ 4.19 – 4.07 (m, 2H, $CO_2CH_2CH_3$), 2.48 (t, *J* = 7.3 Hz, 2H, C*H*$_2$), 2.39 (t, *J* = 7.1 Hz, 2H, C*H*$_2$), 2.34 (s, 1H, C*H*CO_2), 1.64 – 1.49 (m, 4H, C*H*$_2$), 1.45 – 1.20 (m, 15H, C*H*$_2$ & $CO_2CH_2CH_3$), 0.90 – 0.85 (m, 6H, C*H*$_3$); **^{13}C NMR** (101 MHz, $CDCl_3$) δ 174.7, 113.6, 101.4, 91.1, 67.9, 60.4, 31.6, 31.4, 28.9, 28.7, 28.4, 26.7, 25.8, 24.8, 22.6 (2C), 20.1, 14.5, 14.1 (2C); **IR** (ν_{max}, cm^{-1}) 2957 (s), 2931 (s), 2871 (m), 2859 (m), 2215 (w), 1720 (s), 1660 (m), 1604 (w), 1465 (m), 1376 (m), 1265 (m), 1248 (m), 1175 (m), 1160 (m), 1027 (m), 863 (w), 738 (s); **HRMS** (ESI/QTOF) m/z: $[M + H]^+$ Calcd for $C_{20}H_{33}O_2^+$ 305.2475; Found 305.2466.

5.1.28. Synthesis and characterization of ethyl 2-hexyl-3-(tetradec-1-yn-1-yl)cycloprop-2-ene-1-carboxylate (3ab)

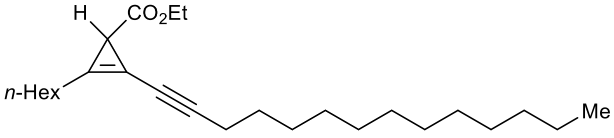

3ab

Following **GPD**, a mixture of (Me_2S)AuCl (2.95 mg, 10.0 μmol, 5.00 mol%), **L1** (4.20 mg, 20.0 μmol, 10.0 mol%), CpBX **1a** (113 mg, 0.200 mmol, 1.00 equiv.), terminal alkyne **2ab** (38.9 mg, 0.200 mmol, 1.00 equiv.) and CH_3CN (2.0 mL) was stirred at room temperature for 3 hours. Flash column chromatography on silica gel (eluent: pentane/ethyl acetate = 50:1) afforded **3ab** in 90% yield (70.1 mg, 180 μmol) as a colourless oil and **4** in 92% NMR yield. **TLC**: R_f (*n*-hexane/EtOAc = 20:1) = 0.45; **^{1}H NMR** (400 MHz, $CDCl_3$) δ 4.20 – 4.08 (m, 2H, $CO_2CH_2CH_3$), 2.48 (t, J = 7.2 Hz, 2H, CH_2), 2.40 (t, J = 7.2 Hz, 2H, CH_2), 2.35 (s, 1H, $CHCO_2$), 1.62 – 1.52 (m, 4H, CH_2), 1.46 – 1.19 (m, 27H, CH_2 & $CO_2CH_2CH_3$), 0.90 – 0.86 (m, 6H, CH_3); **^{13}C NMR** (101 MHz, $CDCl_3$) δ 174.7, 113.6, 101.5, 91.1, 67.9, 60.5, 32.1, 31.6, 29.81, 29.77 (2C), 29.6, 29.5, 29.3, 29.1, 28.9, 28.5, 26.7, 25.8, 24.8, 22.8, 22.7, 20.2, 14.5, 14.3, 14.2; **IR** (ν_{max}, cm^{-1}) 2955 (m), 2925 (s), 2855 (s), 2214 (w), 1716 (s), 1658 (m), 1606 (m), 1465 (m), 1376 (w), 1249 (m), 1177 (m), 1160 (m), 1034 (m), 725 (w); **HRMS** (ESI/QTOF) m/z: $[M + Na]^+$ Calcd for $C_{26}H_{44}NaO_2^+$ 411.3234; Found 411.3233.

5.1.29. Synthesis and characterization of ethyl 2-(cyclopentylethynyl)-3-hexylcycloprop-2-ene-1-carboxylate (3ac)

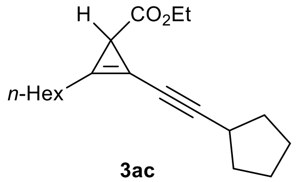

3ac

Following **GPD**, a mixture of (Me_2S)AuCl (2.95 mg, 10.0 μmol, 5.00 mol%), **L1** (4.20 mg, 20.0 μmol, 10.0 mol%), CpBX **1a** (113 mg, 0.200 mmol, 1.00 equiv.), terminal alkyne **2ac** (18.8 mg, 0.200 mmol, 1.00 equiv.) and CH_3CN (2.0 mL) was stirred at room temperature for 3 hours. Flash column chromatography on silica gel (eluent: pentane/ethyl acetate = 50:1) afforded **3ac** in 83% yield (47.6 mg, 165 μmol) as a colourless oil and **4** in 97% NMR yield. **TLC**: R_f (*n*-hexane/EtOAc = 10:1) = 0.51; **^{1}H NMR** (400 MHz, $CDCl_3$) δ 4.20 – 4.08 (m, 2H, $CO_2CH_2CH_3$), 2.81 (pent, J = 7.5 Hz, 1H, C≡CC*H*), 2.48 (t, J = 7.2 Hz, 2H, CH_2CH_2C), 2.34 (s, 1H, $CHCO_2$), 2.03 – 1.88 (m, 2H, CH_2CH_2C), 1.80 – 1.49 (m, 8H, CH_2), 1.46 – 1.16 (m, 9H, CH_2 & $CO_2CH_2CH_3$), 0.95 – 0.80 (m, 3H, CH_3); **^{13}C NMR** (101 MHz, $CDCl_3$) δ 174.7, 113.5, 105.4, 91.1, 67.4, 60.4, 33.7, 31.6, 31.3, 28.9, 26.7, 25.8, 25.2, 24.8, 22.7, 14.5, 14.2; **IR** (ν_{max}, cm^{-1}) 2955 (m), 2934 (m), 2871 (m), 2209 (w), 1733 (s), 1714 (s), 1654 (m), 1604 (m), 1456 (m), 1410 (w), 1373 (w), 1298 (w), 1250 (m), 1178 (m), 1097 (w), 1032 (m), 858 (w); **HRMS** (ESI/QTOF) m/z: $[M + Na]^+$ Calcd for $C_{19}H_{28}NaO_2^+$ 311.1982; Found 311.1987.

5.1.30. Synthesis and characterization of ethyl 2-(cyclopropylethynyl)-3-hexylcycloprop-2-ene-1-carboxylate (3ad)

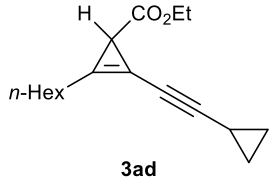

3ad

Following **GPD**, a mixture of (Me_2S)AuCl (2.95 mg, 10.0 μmol, 5.00 mol%), **L1** (4.20 mg, 20.0 μmol, 10.0 mol%), CpBX **1a** (113 mg, 0.200 mmol, 1.00 equiv.), terminal alkyne **2ad** (13.2 mg, 0.200 mmol, 1.00 equiv.) and CH_3CN (2.0 mL) was stirred at room temperature for 4 hours. Flash column chromatography on silica gel (eluent: pentane/ethyl acetate = 50:1) afforded **3ad** in 94% yield (49.1 mg, 188 μmol) as a colourless oil and **4** in 98% NMR yield. **TLC**: R_f (*n*-hexane/EtOAc = 20:1) = 0.32; **^{1}H NMR** (400 MHz, $CDCl_3$) δ 4.19 – 4.08 (m, 2H, $CO_2CH_2CH_3$), 2.47 (t, J = 7.3 Hz, 2H, CH_2CH_2C), 2.34 (s, 1H, $CHCO_2$), 1.61 – 1.51 (m, 2H, CH_2CH_2C), 1.48 – 1.41 (m, 1H, C≡CC*H*), 1.39 – 1.19 (m, 9H, CH_2 & $CO_2CH_2CH_3$), 0.91 – 0.76 (m, 7H, CH_2 & CH_3); **^{13}C NMR** (101 MHz, $CDCl_3$) δ 174.6, 113.5, 104.4, 91.0,

63.2, 60.5, 31.6, 28.9, 26.7, 25.8, 24.9, 22.6, 14.5, 14.2, 9.1, 0.8; **IR** (ν_{max}, cm^{-1}) 2957 (m), 2932 (m), 2861 (m), 2209 (m), 1713 (s), 1601 (m), 1465 (m), 1378 (m), 1256 (m), 1166 (m), 1094 (m), 1027 (m); **HRMS** (ESI/QTOF) m/z: [M + H]+ Calcd for $C_{17}H_{25}O_2^+$ 261.1849; Found 261.1854.

5.1.31. Synthesis and characterization of ethyl 2-(6-chlorohex-1-yn-1-yl)-3-hexylcycloprop-2-ene-1-carboxylate (3ae)

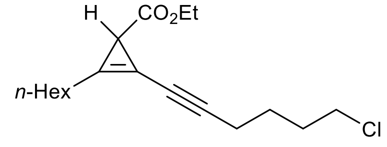

3ae

Following **GPD**, a mixture of (Me_2S)AuCl (2.95 mg, 10.0 μmol, 5.00 mol%), **L1** (4.20 mg, 20.0 μmol, 10.0 mol%), CpBX **1a** (113 mg, 0.200 mmol, 1.00 equiv.), terminal alkyne **2ae** (23.3 mg, 0.200 mmol, 1.00 equiv.) and CH_3CN (2.0 mL) was stirred at room temperature for 6 hours. Flash column chromatography on silica gel (eluent: pentane/ethyl acetate = 40:1) afforded **3ae** in 88% yield (54.8 mg, 176 μmol) as a colourless oil and **4** in 97% NMR yield. **TLC**: R_f (*n*-hexane/EtOAc = 10:1) = 0.48; **^{1}H NMR** (400 MHz, $CDCl_3$) δ 4.19 – 4.07 (m, 2H, $CO_2CH_2CH_3$), 3.55 (t, *J* = 6.5 Hz, 2H, CH_2), 2.50 – 2.44 (m, 4H, CH_2), 2.34 (s, 1H, $CHCO_2$), 1.96 – 1.84 (m, 2H, CH_2CH_2C), 1.80 – 1.66 (m, 2H, CH_2), 1.63 – 1.50 (m, 2H, CH_2), 1.43 – 1.18 (m, 9H, CH_2 & $CO_2CH_2CH_3$), 0.87 (t, *J* = 6.8 Hz, 3H, CH_3); **^{13}C NMR** (101 MHz, $CDCl_3$) δ 174.5, 114.3, 100.2, 90.9, 68.6, 60.5, 44.5, 31.6, 31.5, 28.9, 26.6, 25.8, 25.6, 24.7, 22.6, 19.4, 14.5, 14.1; **IR** (ν_{max}, cm^{-1}) 2956 (s), 2934 (s), 2861 (m), 2218 (w), 1725 (s), 1656 (m), 1604 (m), 1462 (m), 1265 (m), 1181 (s), 1029 (m); **HRMS** (ESI/QTOF) m/z: [M + H]+ Calcd for $C_{18}H_{28}ClO_2^+$ 311.1772; Found 311.1771.

5.1.32. Synthesis and characterization of ethyl 2-(4-bromobut-1-yn-1-yl)-3-hexylcycloprop-2-ene-1-carboxylate (3af)

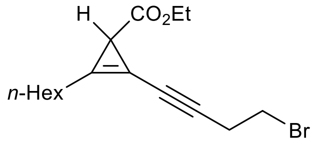

3af

Following **GPD**, a mixture of (Me_2S)AuCl (2.95 mg, 10.0 μmol, 5.00 mol%), **L1** (4.20 mg, 20.0 μmol, 10.0 mol%), CpBX **1a** (113 mg, 0.200 mmol, 1.00 equiv.), terminal alkyne **2af** (26.6 mg, 0.200 mmol, 1.00 equiv.) and CH_3CN (2.0 mL) was stirred at room temperature for 3 hours. Flash column chromatography on silica gel (eluent: pentane/ethyl acetate = 50:1) afforded **3af** in 90% yield (59.1 mg, 181 μmol) as a colourless oil and **4** in 98% NMR yield. **TLC**: R_f (*n*-hexane/EtOAc = 10:1) = 0.49; **^{1}H NMR** (400 MHz, $CDCl_3$) δ 4.20 – 4.08 (m, 2H, $CO_2CH_2CH_3$), 3.46 (t, *J* = 7.4 Hz, 2H, CH_2), 2.98 (t, *J* = 7.3 Hz, 2H, CH_2), 2.50 (t, *J* = 7.2 Hz, 2H, CH_2CH_2C), 2.38 (s, 1H, $CHCO_2$), 1.63 – 1.52 (m, 2H, CH_2CH_2C), 1.42 – 1.20 (m, 9H, CH_2 & $CO_2CH_2CH_3$), 0.91 – 0.84 (m, 3H, CH_3); **^{13}C NMR** (101 MHz, $CDCl_3$) δ 174.3, 115.9, 97.2, 90.5, 69.9, 60.6, 31.5, 28.9, 28.7, 26.6, 25.9, 24.8, 24.5, 22.6, 14.5, 14.2; **IR** (ν_{max}, cm^{-1}) 2957 (m), 2930 (m), 2858 (m), 2240 (w), 1723 (s), 1654 (m), 1596 (m), 1466 (m), 1372 (w), 1268 (m), 1181 (s), 1096 (m), 1026 (m), 861 (w), 740 (w); **HRMS** (ESI/QTOF) m/z: [M + H]+ Calcd for $C_{16}H_{24}BrO_2^+$ 327.0954; Found 327.0952.

5.1.33. Synthesis and characterization of ethyl 2-hexyl-3-(5-iodopent-1-yn-1-yl)cycloprop-2-ene-1-carboxylate (3ag)

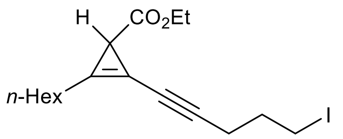

3ag

Following **GPD**, a mixture of $(Me_2S)AuCl$ (2.95 mg, 10.0 µmol, 5.00 mol%), **L1** (4.20 mg, 20.0 µmol, 10.0 mol%), CpBX **1a** (113 mg, 0.200 mmol, 1.00 equiv.), terminal alkyne **2ag** (38.8 mg, 0.200 mmol, 1.00 equiv.) and CH_3CN (2.0 mL) was stirred at room temperature for 6 hours. Flash column chromatography on silica gel (eluent: pentane/ethyl acetate = 50:1) afforded **3ag** in 82% yield (63.5 mg, 164 µmol) as a colourless oil and **4** in 92% NMR yield. **TLC**: R_f (*n*-hexane/EtOAc = 10:1) = 0.53; **^{1}H NMR** (400 MHz, $CDCl_3$) δ 4.17 – 4.10 (m, 2H, $CO_2CH_2CH_3$), 3.28 (t, *J* = 6.8 Hz, 2H, CH_2), 2.56 (t, *J* = 6.8 Hz, 2H, CH_2), 2.49 (t, *J* = 7.2 Hz, 2H, CH_2CH_2C), 2.35 (s, 1H, $CHCO_2$), 2.04 (pent, *J* = 6.8 Hz, 2H, CH_2CH_2C), 1.64 – 1.51 (m, 2H, CH_2), 1.43 – 1.17 (m, 9H, CH_2 & $CO_2CH_2CH_3$), 0.94 – 0.79 (m, 3H, CH_3); **^{13}C NMR** (101 MHz, $CDCl_3$) δ 174.5, 114.7, 98.7, 90.7, 69.1, 60.5, 31.9, 31.5, 28.9, 26.6, 25.8, 24.8, 22.6, 21.2, 14.5, 14.2, 5.1; **IR** (ν_{max}, cm^{-1}) 2955 (m), 2932 (m), 2856 (m), 2219 (w), 1722 (s), 1660 (m), 1604 (m), 1466 (m), 1371 (m), 1220 (m), 1178 (s), 1025 (m), 735 (w); **HRMS** (ESI/QTOF) m/z: $[M + H]^+$ Calcd for $C_{17}H_{26}IO_2^+$ 389.0972; Found 389.0971.

5.1.34. Synthesis and characterization of ethyl 2-hexyl-3-(7-hydroxyhept-1-yn-1-yl)cycloprop-2-ene-1-carboxylate (3ah)

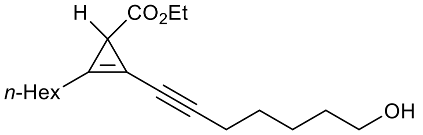

3ah

Following **GPD**, a mixture of $(Me_2S)AuCl$ (2.95 mg, 10.0 µmol, 5.00 mol%), **L1** (4.20 mg, 20.0 µmol, 10.0 mol%), CpBX **1a** (113 mg, 0.200 mmol, 1.00 equiv.), terminal alkyne **2ah** (22.4 mg, 0.200 mmol, 1.00 equiv.) and CH_3CN (2.0 mL) was stirred at room temperature for 6 hours. Flash column chromatography on silica gel (eluent: pentane/ethyl acetate = 10:1) afforded **3ah** in 87% yield (53.1 mg, 173 µmol) as a colourless oil and **4** in 96% NMR yield. **TLC**: R_f (*n*-hexane/EtOAc = 4:1) = 0.17; **^{1}H NMR** (400 MHz, $CDCl_3$) δ 4.18 – 4.06 (m, 2H, $CO_2CH_2CH_3$), 3.62 (t, *J* = 6.5 Hz, 2H, CH_2), 2.47 (t, *J* = 7.3 Hz, 2H, CH_2), 2.41 (t, *J* = 7.0 Hz, 2H, CH_2CH_2C), 2.33 (s, 1H, $CHCO_2$), 1.78 (bs, 1H, O*H*), 1.64 – 1.51 (m, 6H, CH_2), 1.51 – 1.41 (m, 2H, CH_2), 1.41 – 1.14 (m, 9H, CH_2 & $CO_2CH_2CH_3$), 0.93 – 0.80 (m, 3H, CH_3); **^{13}C NMR** (101 MHz, $CDCl_3$) δ 174.7, 113.7, 101.0, 90.9, 68.0, 62.8, 60.5, 32.3, 31.5, 28.9, 28.2, 26.6, 25.8, 25.2, 24.7, 22.6, 20.1, 14.4, 14.1; **IR** (ν_{max}, cm^{-1}) 2932 (s), 2859 (m), 2348 (w), 2214 (w), 1721 (s), 1660 (m), 1607 (m), 1462 (m), 1371 (m), 1248 (m), 1178 (s), 1071 (m), 1031 (m), 866 (w), 732 (w); **HRMS** (ESI/QTOF) m/z: $[M + Na]^+$ Calcd for $C_{19}H_{30}NaO_3^+$ 329.2087; Found 329.2088.

5.1.35. Synthesis and characterization of ethyl 2-(4-((*tert*-butyldimethylsilyl)oxy)but-1-yn-1-yl)-3-hexylcycloprop-2-ene-1-carboxylate (3ai)

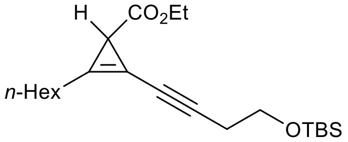

3ai

Following **GPD**, a mixture of $(Me_2S)AuCl$ (2.95 mg, 10.0 µmol, 5.00 mol%), **L1** (4.20 mg, 20.0 µmol, 10.0 mol%), CpBX **1a** (113 mg, 0.200 mmol, 1.00 equiv.), terminal alkyne **2ai** (36.9 mg, 0.200 mmol, 1.00 equiv.) and CH_3CN (2.0 mL) was stirred at room temperature for 6 hours. Flash column chromatography on silica gel (eluent: pentane/ethyl acetate = 50:1) afforded **3ai** in 87% yield (65.8 mg, 174 µmol) as a colourless oil and **4** in 99% NMR yield. **TLC**: R_f (*n*-hexane/EtOAc = 10:1) = 0.59; **^{1}H NMR** (400 MHz, $CDCl_3$) δ 4.20 – 4.05 (m, 2H, $CO_2CH_2CH_3$), 3.76 (t, *J* = 7.0 Hz, 2H, CH_2), 2.62 (t, *J* = 7.0 Hz, 2H, CH_2), 2.48 (t, *J* = 7.3 Hz, 2H, CH_2CH_2C), 2.35 (s, 1H, C*H*CO_2), 1.63 – 1.50 (m, 2H, CH_2CH_2C), 1.42 – 1.19 (m, 9H, CH_2 & $CO_2CH_2CH_3$), 0.92 – 0.81 (m, 12H, $C(CH_3)_3$ & CH_3), 0.06 (s, 6H, $Si(CH_3)_2$); **^{13}C NMR** (101 MHz, $CDCl_3$) δ 174.5, 114.3, 98.2, 90.9, 69.0, 61.6, 60.5, 31.6, 28.9, 26.6, 26.0, 25.8, 24.8, 24.5, 22.6, 18.5, 14.5, 14.2, -5.2; **IR** (ν_{max}, cm^{-1}) 2957 (m), 2932 (m), 2858 (m), 2217 (w), 1725 (s), 1661 (m), 1608 (w), 1469 (m), 1372 (w), 1253 (m), 1177 (m), 1098 (m), 1035 (m), 836 (s), 777 (s), 736 (m); **HRMS** (ESI/QTOF) m/z: [M + Na]$^+$ Calcd for $C_{22}H_{38}NaO_3Si^+$ 401.2482; Found 401.2483.

5.1.36. Synthesis and characterization of *tert*-butyl 4-((3-(ethoxycarbonyl)-2-hexylcycloprop-1-en-1-yl)ethynyl)piperidine-1-carboxylate (3aj)

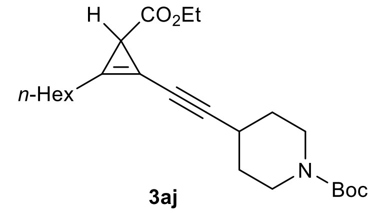

3aj

Following **GPD**, a mixture of $(Me_2S)AuCl$ (2.95 mg, 10.0 µmol, 5.00 mol%), **L1** (4.20 mg, 20.0 µmol, 10.0 mol%), CpBX **1a** (113 mg, 0.200 mmol, 1.00 equiv.), terminal alkyne **2aj** (41.9 mg, 0.200 mmol, 1.00 equiv.) and CH_3CN (2.0 mL) was stirred at room temperature for 4 hours. Flash column chromatography on silica gel (eluent: pentane/ethyl acetate = 10:1) afforded **3aj** in 93% yield (75.4 mg, 187 µmol) as a colourless oil and **4** in 99% NMR yield. **TLC**: R_f (*n*-hexane/EtOAc = 10:1) = 0.18; **^{1}H NMR** (400 MHz, $CDCl_3$) δ 4.12 (qd, *J* = 7.1, 0.9 Hz, 2H, $CO_2CH_2CH_3$), 3.75 – 3.59 (m, 2H, CH_2), 3.19 – 3.12 (m, 2H, CH_2), 2.79 – 2.73 (m, 1H, C≡CC*H*), 2.47 (t, *J* = 7.2 Hz, 2H, CH_2CH_2C), 2.34 (s, 1H, C*H*CO_2), 1.90 – 1.71 (m, 2H, CH_2CH_2C), 1.70 – 1.51 (m, 4H, CH_2), 1.42 (s, 9H, $C(CH_3)_3$), 1.39 – 1.13 (m, 9H, CH_2 & $CO_2CH_2CH_3$), 0.96 – 0.73 (m, 3H, CH_3); **^{13}C NMR** (101 MHz, $CDCl_3$) δ 174.4, 154.8, 114.8, 102.4, 90.7, 79.6, 69.2, 60.4, 42.3 (bs), 31.5, 31.1, 28.8, 28.5, 28.3, 26.6, 25.8, 24.8, 22.6, 14.4, 14.1; **IR** (ν_{max}, cm^{-1}) 2971 (m), 2956 (m), 2931 (m), 2863 (w), 2212 (w), 1725 (m), 1693 (s), 1663 (s), 1467 (m), 1422 (m), 1367 (m), 1273 (m), 1234 (m), 1162 (s), 1126 (m), 1026 (m), 865 (m), 770 (w); **HRMS** (ESI/QTOF) m/z: [M + Na]$^+$ Calcd for $C_{24}H_{37}NNaO_4^+$ 426.2615; Found 426.2610.

5.1.37. Synthesis and characterization of ethyl 2-(3-(1,3-dioxoisoindolin-2-yl)prop-1-yn-1-yl)-3-hexylcycloprop-2-ene-1-carboxylate (3ak)

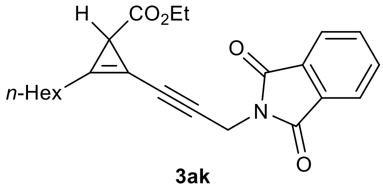

3ak

Following **GPD**, a mixture of $(Me_2S)AuCl$ (2.95 mg, 10.0 µmol, 5.00 mol%), **L1** (4.20 mg, 20.0 µmol, 10.0 mol%), CpBX **1a** (113 mg, 0.200 mmol, 1.00 equiv.), terminal alkyne **2ak** (37.0 mg, 0.200 mmol, 1.00 equiv.) and CH_3CN (2.0 mL) was stirred at room temperature for 3 hours. Flash column chromatography on silica gel (eluent: pentane/ethyl acetate = 4:1) afforded **3ak** in 95% yield (72.3 mg, 191 µmol) as a colourless oil and **4** in 97% NMR yield. **TLC**: R_f (*n*-hexane/EtOAc = 4:1) = 0.43; **^{1}H NMR** (400 MHz, $CDCl_3$) δ 7.88 – 7.84 (m, 2H, Ar*H*), 7.79 – 7.68 (m, 2H, Ar*H*), 4.65 (s, 2H, $CO_2CH_2CH_3$), 4.16 –

4.04 (m, 2H, NCH_2), 2.47 (t, J = 7.3 Hz, 2H, CH_2CH_2C), 2.35 (s, 1H, C$H$$CO_2$), 1.59 – 1.45 (m, 2H, CH_2CH_2C), 1.37 – 1.15 (m, 9H, CH_2 & $CO_2CH_2CH_3$), 0.88 – 0.77 (m, 3H, CH_3); **^{13}C NMR** (101 MHz, $CDCl_3$) δ 174.0, 167.0, 134.3, 132.1, 123.7, 117.7, 92.9, 90.1, 70.7, 60.5, 31.4, 28.8, 28.1, 26.5, 25.9, 24.8, 22.5, 14.4, 14.1; **IR** (ν_{max}, cm^{-1}) 2957 (w), 2931 (w), 2859 (w), 2226 (w), 1774 (m), 1717 (s), 1610 (w), 1468 (w), 1417 (m), 1390 (m), 1343 (m), 1315 (w), 1258 (w), 1188 (m), 1115 (m), 1087 (w), 1026 (w), 940 (m), 794 (w), 726 (s), 716 (s); **HRMS** (ESI/QTOF) m/z: [M + Na]$^+$ Calcd for $C_{23}H_{25}NNaO_4^+$ 402.1676; Found 402.1681.

5.1.38. Synthesis and characterization of ethyl 2-hexyl-3-(3-hydroxy-3-methylbut-1-yn-1-yl)cycloprop-2-ene-1-carboxylate (3al)

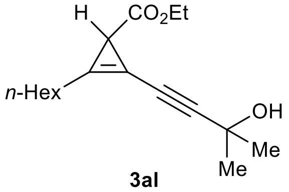

3al

Following **GPD**, a mixture of (Me_2S)AuCl (2.95 mg, 10.0 μmol, 5.00 mol%), **L1** (4.20 mg, 20.0 μmol, 10.0 mol%), CpBX **1a** (113 mg, 0.200 mmol, 1.00 equiv.), terminal alkyne **2al** (16.8 mg, 0.200 mmol, 1.00 equiv.) and CH_3CN (2.0 mL) was stirred at room temperature for 4 hours. Flash column chromatography on silica gel (eluent: pentane/ethyl acetate = 8:1) afforded **3al** in 96% yield (53.5 mg, 192 μmol) as a colourless oil and **4** in 99% NMR yield. **TLC**: R_f (*n*-hexane/EtOAc = 5:1) = 0.27; **^{1}H NMR** (400 MHz, $CDCl_3$) δ 4.19 – 4.08 (m, 2H, $CO_2CH_2CH_3$), 2.50 (t, J = 7.3 Hz, 2H, CH_2CH_2C), 2.37 (s, 1H, C$H$$CO_2$), 2.29 (bs, 1H, O$H$), 1.61 – 1.51 (m, 8H, C$H_2$ & C(CH_3)$_2$), 1.45 – 1.17 (m, 9H, CH_2 & $CO_2CH_2CH_3$), 0.87 (t, J = 6.8 Hz, 3H, CH_3); **^{13}C NMR** (101 MHz, $CDCl_3$) δ 174.4, 116.5, 103.9, 90.2, 69.8, 65.8, 60.6, 31.5, 31.2, 28.9, 26.6, 25.8, 24.8, 22.6, 14.4, 14.1; **IR** (ν_{max}, cm^{-1}) 2979 (m), 2960 (m), 2932 (m), 2859 (m), 2221 (w), 1715 (s), 1660 (m), 1609 (m), 1464 (m), 1371 (s), 1335 (m), 1246 (s), 1200 (s), 1176 (s), 1025 (m), 959 (m), 869 (w); **HRMS** (ESI/QTOF) m/z: [M + Na]$^+$ Calcd for $C_{17}H_{26}NaO_3^+$ 301.1774; Found 301.1778.

5.1.39. Synthesis and characterization of ethyl 2-(3-(benzyloxy)prop-1-yn-1-yl)-3-hexylcycloprop-2-ene-1-carboxylate (3am)

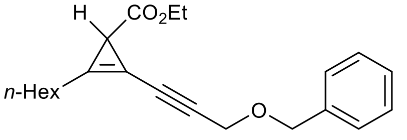

3am

Following **GPD**, a mixture of (Me_2S)AuCl (2.95 mg, 10.0 μmol, 5.00 mol%), **L1** (4.20 mg, 20.0 μmol, 10.0 mol%), CpBX **1a** (113 mg, 0.200 mmol, 1.00 equiv.), terminal alkyne **2am** (29.2 mg, 0.200 mmol, 1.00 equiv.) and CH_3CN (2.0 mL) was stirred at room temperature for 5 hours. Flash column chromatography on silica gel (eluent: pentane/ethyl acetate = 40:1) afforded **3am** in 85% yield (57.9 mg, 170 μmol) as a colourless oil and **4** in 91% NMR yield. **TLC**: R_f (*n*-hexane/EtOAc = 20:1) = 0.20; **^{1}H NMR** (400 MHz, $CDCl_3$) δ 7.40 – 7.27 (m, 5H, ArH), 4.62 (s, 2H, OCH_2), 4.38 (s, 2H, OCH_2), 4.21 – 4.10 (m, 2H, $CO_2CH_2CH_3$), 2.54 (t, J = 7.3 Hz, 2H, CH_2CH_2C), 2.43 (s, 1H, C$H$$CO_2$), 1.68 – 1.55 (m, 2H, CH_2CH_2C), 1.45 – 1.18 (m, 9H, CH_2 & $CO_2CH_2CH_3$), 0.95 – 0.76 (m, 3H, CH_3); **^{13}C NMR** (101 MHz, $CDCl_3$) δ 174.2, 137.3, 128.6, 128.2, 128.1, 117.1, 95.6, 90.3, 74.1, 71.9, 60.6, 58.1, 31.5, 28.9, 26.6, 26.0, 25.0, 22.6, 14.4, 14.1; **IR** (ν_{max}, cm^{-1}) 2957 (m), 2931 (m), 2859 (m), 2221 (w), 1721 (s), 1605 (m), 1455 (m), 1372 (m), 1258 (m), 1182 (m), 1094 (s), 1072 (m), 1027 (m), 744 (s); **HRMS** (ESI/QTOF) m/z: [M + Na]$^+$ Calcd for $C_{22}H_{28}NaO_3^+$ 363.1931; Found 363.1930.

5.1.40. Synthesis and characterization of ethyl 2-hexyl-3-(3-phenoxyprop-1-yn-1-yl)cycloprop-2-ene-1-carboxylate (3an)

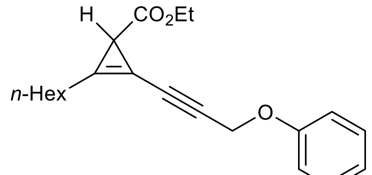

3an

Following **GPD**, a mixture of $(Me_2S)AuCl$ (2.95 mg, 10.0 μmol, 5.00 mol%), **L1** (4.20 mg, 20.0 μmol, 10.0 mol%), CpBX **1a** (113 mg, 0.200 mmol, 1.00 equiv.), terminal alkyne **2an** (26.4 mg, 0.200 mmol, 1.00 equiv.) and CH_3CN (2.0 mL) was stirred at room temperature for 3 hours. Flash column chromatography on silica gel (eluent: pentane/ethyl acetate = 40:1) afforded **3an** in 90% yield (59.0 mg, 181 μmol) as a colourless oil and **4** in 92% NMR yield. **TLC**: R_f (*n*-hexane/EtOAc = 10:1) = 0.43; ^{1}**H NMR** (400 MHz, $CDCl_3$) δ 7.35 – 7.27 (m, 2H, Ar*H*), 7.03 – 6.93 (m, 3H, Ar*H*), 4.89 (s, 2H, OCH_2), 4.20 – 4.09 (m, 2H, $CO_2CH_2CH_3$), 2.52 (t, *J* = 7.3 Hz, 2H, CH_2CH_2C), 2.42 (s, 1H, $CHCO_2$), 1.63 – 1.51 (m, 2H, CH_2CH_2C), 1.43 – 1.15 (m, 9H, CH_2 & $CO_2CH_2CH_3$), 0.96 – 0.76 (m, 3H, CH_3); ^{13}**C NMR** (101 MHz, $CDCl_3$) δ 174.0, 157.7, 129.6, 121.7, 118.0, 115.0, 94.3, 90.1, 74.7, 60.6, 56.7, 31.5, 28.9, 26.5, 26.0, 25.0, 22.6, 14.4, 14.1; **IR** (ν_{max}, cm^{-1}) 2955 (m), 2929 (m), 2858 (w), 2229 (w), 1860 (w), 1723 (m), 1599 (m), 1589 (m), 1495 (s), 1458 (w), 1370 (w), 1335 (w), 1303 (w), 1260 (m), 1213 (s), 1174 (s), 1033 (s), 1018 (m), 753 (s); **HRMS** (ESI/QTOF) m/z: $[M + H]^+$ Calcd for $C_{21}H_{27}O_3^+$ 327.1955; Found 327.1955.

5.1.41. Synthesis and characterization of 3-(3-(ethoxycarbonyl)-2-hexylcycloprop-1-en-1-yl)prop-2-yn-1-yl benzoate (3ao)

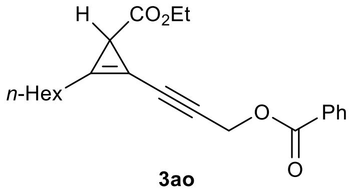

3ao

Following **GPD**, a mixture of $(Me_2S)AuCl$ (2.95 mg, 10.0 μmol, 5.00 mol%), **L1** (4.20 mg, 20.0 μmol, 10.0 mol%), CpBX **1a** (113 mg, 0.200 mmol, 1.00 equiv.), terminal alkyne **2ao** (32.0 mg, 0.200 mmol, 1.00 equiv.) and CH_3CN (2.0 mL) was stirred at room temperature for 3 hours. Flash column chromatography on silica gel (eluent: pentane/ethyl acetate = 10:1) afforded **3ao** in 93% yield (65.8 mg, 186 μmol) as a colourless oil and **4** in 94% NMR yield. **TLC**: R_f (*n*-hexane/EtOAc = 10:1) = 0.33; ^{1}**H NMR** (400 MHz, $CDCl_3$) δ 8.12 – 7.97 (m, 2H, Ar*H*), 7.60 – 7.55 (m, 1H, Ar*H*), 7.51 – 7.37 (m, 2H, Ar*H*), 5.13 (s, 2H, OCH_2), 4.20 – 4.09 (m, 2H, $CO_2CH_2CH_3$), 2.53 (t, *J* = 7.2 Hz, 2H, CH_2CH_2C), 2.43 (s, 1H, $CHCO_2$), 1.65 – 1.50 (m, 2H, CH_2CH_2C), 1.43 – 1.19 (m, 9H, CH_2 & $CO_2CH_2CH_3$), 0.94 – 0.69 (m, 3H, CH_3); ^{13}**C NMR** (101 MHz, $CDCl_3$) δ 174.0, 165.9, 133.5, 130.0, 129.5, 128.6, 118.3, 93.3, 90.1, 74.3, 60.7, 53.3, 31.5, 28.9, 26.6, 26.0, 25.0, 22.6, 14.4, 14.1; **IR** (ν_{max}, cm^{-1}) 2957 (w), 2930 (w), 2860 (w), 2233 (w), 1725 (s), 1602 (w), 1585 (w), 1452 (m), 1372 (w), 1315 (w), 1265 (s), 1177 (m), 1094 (m), 1069 (m), 1026 (m), 711 (s); **HRMS** (ESI/QTOF) m/z: $[M + H]^+$ Calcd for $C_{22}H_{27}O_4^+$ 355.1904; Found 355.1903.

5.1.42. Synthesis and characterization of ethyl 2-hexyl-3-(5-methoxy-5-oxopent-1-yn-1-yl)cycloprop-2-ene-1-carboxylate (3ap)

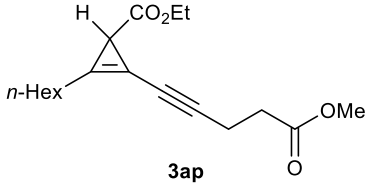

3ap

Following **GPD**, a mixture of $(Me_2S)AuCl$ (2.95 mg, 10.0 μmol, 5.00 mol%), **L1** (4.20 mg, 20.0 μmol, 10.0 mol%), CpBX **1a** (113 mg, 0.200 mmol, 1.00 equiv.), terminal alkyne **2ap** (22.4 mg, 0.200 mmol, 1.00 equiv.) and CH_3CN (2.0 mL) was stirred at room temperature for 2 hours. Flash column chromatography on silica gel (eluent: pentane/ethyl acetate = 10:1) afforded **3ap** in 87% yield (53.2 mg, 174 μmol) as a colourless oil and **4** in 98% NMR yield. **TLC**: R_f (*n*-hexane/EtOAc = 10:1) = 0.25; **^{1}H NMR** (400 MHz, $CDCl_3$) δ 4.18 – 4.06 (m, 2H, $CO_2CH_2CH_3$), 3.68 (s, 3H, OCH_3), 2.76 – 2.66 (m, 2H, CH_2), 2.62 – 2.52 (m, 2H, CH_2), 2.47 (t, *J* = 7.3 Hz, 2H, CH_2CH_2C), 2.34 (s, 1H, $CHCO_2$), 1.62 – 1.47 (m, 2H, CH_2CH_2C), 1.42 – 1.16 (m, 9H, CH_2 & $CO_2CH_2CH_3$), 0.93 – 0.79 (m, 3H, CH_3); **^{13}C NMR** (101 MHz, $CDCl_3$) δ 174.4, 172.2, 114.8, 98.7, 90.7, 68.6, 60.5, 52.0, 32.9, 31.5, 28.9, 26.6, 25.8, 24.7, 22.6, 15.9, 14.4, 14.1; **IR** (ν_{max}, cm^{-1}) 2957 (m), 2932 (m), 2858 (w), 2221 (w), 1738 (s), 1660 (m), 1606 (w), 1462 (w), 1439 (m), 1369 (m), 1249 (m), 1200 (m), 1167 (s), 1029 (m); **HRMS** (ESI/QTOF) m/z: $[M + Na]^+$ Calcd for $C_{18}H_{26}NaO_4^+$ 329.1723; Found 329.1727.

5.1.43. Synthesis and characterization of ethyl 2-(5-cyanopent-1-yn-1-yl)-3-hexylcycloprop-2-ene-1-carboxylate (3aq)

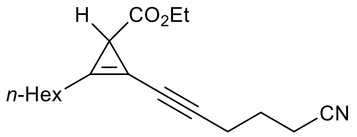

3aq

Following **GPD**, a mixture of $(Me_2S)AuCl$ (2.95 mg, 10.0 μmol, 5.00 mol%), **L1** (4.20 mg, 20.0 μmol, 10.0 mol%), CpBX **1a** (113 mg, 0.200 mmol, 1.00 equiv.), terminal alkyne **2aq** (18.6 mg, 0.200 mmol, 1.00 equiv.) and CH_3CN (2.0 mL) was stirred at room temperature for 3 hours. Flash column chromatography on silica gel (eluent: pentane/ethyl acetate = 10:1) afforded **3aq** in 90% yield (51.6 mg, 180 μmol) as a colourless oil and **4** in 99% NMR yield. **TLC**: R_f (*n*-hexane/EtOAc = 5:1) = 0.29; **^{1}H NMR** (400 MHz, $CDCl_3$) δ 4.19 – 4.07 (m, 2H, $CO_2CH_2CH_3$), 2.60 (t, *J* = 6.8 Hz, 2H, CH_2), 2.49 (td, *J* = 7.2, 1.2 Hz, 4H, CH_2), 2.36 (s, 1H, $CHCO_2$), 1.91 (pent, *J* = 7.0 Hz, 2H, CH_2CH_2C), 1.64 – 1.49 (m, 2H, CH_2), 1.43 – 1.17 (m, 9H, CH_2 & $CO_2CH_2CH_3$), 0.96 – 0.72 (m, 3H, CH_3); **^{13}C NMR** (101 MHz, $CDCl_3$) δ 174.3, 119.0, 115.4, 97.7, 90.5, 69.8, 60.5, 31.5, 28.8, 26.6, 25.8, 24.7, 24.4, 22.6, 19.2, 16.3, 14.4, 14.1; **IR** (ν_{max}, cm^{-1}) 2956 (m), 2933 (m), 2859 (m), 2248 (w), 2218 (w), 1719 (s), 1660 (m), 1607 (m), 1457 (m), 1428 (m), 1371 (m), 1330 (w), 1250 (m), 1180 (m), 1071 (w), 1029 (m), 937 (w), 865 (w), 735 (w); **HRMS** (ESI/QTOF) m/z: $[M + H]^+$ Calcd for $C_{18}H_{26}NO_2^+$ 288.1958; Found 288.1957.

5.1.44. Synthesis and characterization of ethyl 2-(3-ethoxy-3-oxoprop-1-yn-1-yl)-3-hexylcycloprop-2-ene-1-carboxylate (3ar)

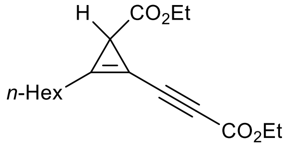

3ar

Following **GPD**, a mixture of $(Me_2S)AuCl$ (2.95 mg, 10.0 μmol, 5.00 mol%), **L1** (4.20 mg, 20.0 μmol, 10.0 mol%), CpBX **1a** (113 mg, 0.200 mmol, 1.00 equiv.), terminal alkyne **2ar** (19.6 mg, 0.200 mmol, 1.00 equiv.) and CH_3CN (2.0 mL) was stirred at room temperature for 9 hours. Flash column chromatography on silica gel (eluent: pentane/ethyl acetate = 10:1) afforded **3ar** in 84% yield (49.0 mg, 168 μmol) as a colourless oil and **4** in 98% NMR yield. **TLC**: R_f (*n*-hexane/EtOAc = 10:1) = 0.50; **^{1}H NMR** (400 MHz, $CDCl_3$) δ 4.26 (q, *J* = 7.1 Hz, 2H, $CO_2CH_2CH_3$), 4.22 – 4.05 (m, 2H, $CO_2CH_2CH_3$), 2.58 (t, *J* = 7.2 Hz, 2H, CH_2CH_2C), 2.51 (s, 1H, $CHCO_2$), 1.66 – 1.50 (m, 2H, CH_2CH_2C), 1.43 – 1.16 (m, 12H, CH_2 & $CO_2CH_2CH_3$), 0.95 – 0.82 (m, 3H, CH_3); **^{13}C NMR** (101 MHz, $CDCl_3$) δ 173.0, 153.4, 125.3, 89.6, 89.0, 73.7,

62.5, 60.9, 31.4, 28.8, 26.6, 26.4, 25.6, 22.6, 14.4, 14.11, 14.09; **IR** (ν_{max}, cm^{-1}) 2981 (w), 2957 (m), 2928 (m), 2858 (w), 2234 (w), 1712 (s), 1613 (w), 1466 (w), 1369 (m), 1298 (w), 1258 (m), 1176 (m), 1160 (m), 1097 (m), 1023 (s), 858 (w); **HRMS** (ESI/QTOF) m/z: [M + H]$^+$ Calcd for $C_{17}H_{25}O_4^+$ 293.1747; Found 293.1748.

5.1.45. Synthesis and characterization of ethyl 2-ethynyl-3-hexylcycloprop-2-ene-1-carboxylate (3as)

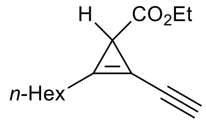

3as

Following **GPD**, a mixture of (Me_2S)AuCl (2.95 mg, 10.0 μmol, 5.00 mol%), **L1** (10.5 mg, 50.0 μmol, 25.0 mol%), CpBX **1a** (113 mg, 0.200 mmol, 1.00 equiv.) and CH_3CN (2.0 mL) was stirred under the atmosphere of acetylene (**2as**, ca. 1.0 atm.) at 40 °C for 18 hours. Flash column chromatography on silica gel (eluent: pentane/ethyl acetate = 40:1) afforded **3as** in 46% yield (20.4 mg, 92.6 μmol) as a colourless oil and **4** in 71% NMR yield. **TLC**: R_f (*n*-hexane/EtOAc = 20:1) = 0.33; **^{1}H NMR** (400 MHz, $CDCl_3$) δ 4.20 – 4.09 (m, 2H, $CO_2CH_2CH_3$), 3.49 (s, 1H, C≡C*H*), 2.53 (t, *J* = 7.2 Hz, 2H, CH_2CH_2C), 2.43 (s, 1H, C*H*CO_2), 1.68 – 1.52 (m, 2H, CH_2CH_2C), 1.44 – 1.19 (m, 9H, CH_2 & $CO_2CH_2CH_3$), 0.88 (t, *J* = 6.9 Hz, 3H, CH_3); **^{13}C NMR** (101 MHz, $CDCl_3$) δ 173.9, 118.4, 90.3, 87.7, 71.3, 60.6, 31.5, 28.9, 26.5, 26.1, 24.9, 22.6, 14.5, 14.1; **IR** (ν_{max}, cm^{-1}) 3274 (w), 2957 (m), 2930 (m), 2859 (m), 2113 (w), 2096 (w), 1722 (s), 1611 (w), 1464 (m), 1373 (m), 1331 (m), 1298 (m), 1239 (m), 1182 (s), 1097 (m), 1029 (s), 861 (w); **HRMS** (ESI/QTOF) m/z: [M + H]$^+$ Calcd for $C_{14}H_{21}O_2^+$ 221.1536; Found 221.1543.

5.1.46. Synthesis and characterization of tetramethyl 3,3'-(octa-1,7-diyne-1,8-diyl)bis(2-phenylcycloprop-2-ene-1,1-dicarboxylate) (3at)

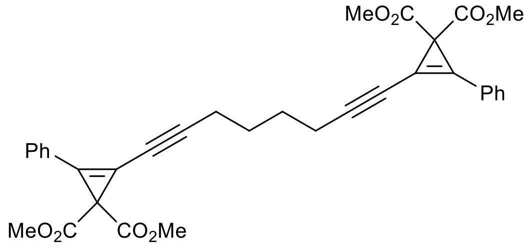

3at

Following **GPD**, a mixture of (Me_2S)AuCl (2.95 mg, 10.0 μmol, 10.0 mol%), **L1** (4.20 mg, 20.0 μmol, 20.0 mol%), CpBX **1k** (120 mg, 0.200 mmol, 2.00 equiv.), diyne **2at** (10.6 mg, 0.100 mmol, 1.00 equiv.) and CH_3CN (2.0 mL) was stirred at room temperature for 24 hours. Flash column chromatography on silica gel (eluent: pentane/ethyl acetate = 4:1) afforded **3at** in 84% yield (47.8 mg, 84.4 μmol) as a colorless oil and **4** in 99% NMR yield. **TLC**: R_f (*n*-hexane/EtOAc = 3:1) = 0.14; **^{1}H NMR** (400 MHz, $CDCl_3$) δ 7.66 – 7.53 (m, 4H, Ar*H*), 7.47 – 7.34 (m, 6H, Ar*H*), 3.74 (s, 12H, CO_2CH_3), 2.68 – 2.47 (m, 4H, CH_2), 1.81 (q, *J* = 3.0 Hz, 4H, CH_2); **^{13}C NMR** (101 MHz, $CDCl_3$) δ 169.8, 130.6, 130.4, 129.0, 124.5, 108.1, 104.8, 92.2, 67.3, 52.6, 36.5, 27.3, 20.0; **IR** (ν_{max}, cm^{-1}) 3059 (w), 3026 (w), 3004 (w), 2953 (w), 2899 (w), 2844 (w), 2226 (w), 2204 (w), 1726 (s), 1491 (w), 1447 (m), 1434 (m), 1333 (w), 1279 (s), 1241 (s), 1188 (w), 1059 (s), 972 (m), 919 (w), 834 (w), 763 (s), 736 (m); **HRMS** (ESI/QTOF) m/z: [M + Na]$^+$ Calcd for $C_{34}H_{30}NaO_8^+$ 589.1833; Found 589.1849.

5.1.47. Synthesis and characterization of tetramethyl 3,3'-(1,4-phenylenebis(ethyne-2,1-diyl))bis(2-phenylcycloprop-2-ene-1,1-dicarboxylate) (3au)

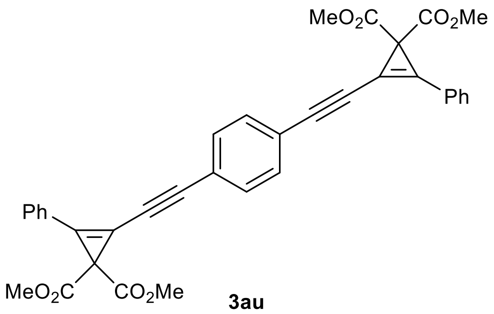

3au

Following **GPD**, a mixture of $(Me_2S)AuCl$ (2.95 mg, 10.0 μmol, 10.0 mol%), **L1** (4.20 mg, 20.0 μmol, 20.0 mol%), CpBX **1k** (120 mg, 0.200 mmol, 2.00 equiv.), diyne **2au** (12.6 mg, 0.100 mmol, 1.00 equiv.) and CH_3CN (2.0 mL) was stirred at room temperature for 24 hours. Flash column chromatography on silica gel (eluent: pentane/ethyl acetate = 4:1) afforded **3au** in 94% yield (55.2 mg, 94.1 μmol) as a yellow solid and **4** in 98% NMR yield. **M.p.** 210 – 213 °C (decomp.). **TLC**: R_f (*n*-hexane/EtOAc = 3:1) = 0.18; **^{1}H NMR** (400 MHz, $CDCl_3$) δ 7.72 – 7.64 (m, 4H, Ar*H*), 7.61 – 7.56 (m, 4H, Ar*H*), 7.51 – 7.42 (m, 6H, Ar*H*), 3.78 (s, 12H, CO_2CH_3); **^{13}C NMR** (101 MHz, $CDCl_3$) δ 169.5, 132.2, 131.1, 130.6, 129.2, 124.4, 123.0, 111.3, 101.7, 91.1, 78.0, 52.8, 36.9; **IR** (ν_{max}, cm^{-1}) 3026 (w), 2954 (w), 2843 (w), 2198 (m), 1744 (s), 1434 (s), 1278 (s), 1240 (m), 1187 (m), 1060 (s), 984 (w), 838 (s), 755 (s), 737 (m); **HRMS** (ESI/QTOF) m/z: $[M + Na]^+$ Calcd for $C_{36}H_{26}NaO_8^+$ 609.1520; Found 609.1526.

5.1.48. Synthesis and characterization of tetramethyl 3,3'-(1,3-phenylenebis(ethyne-2,1-diyl))bis(2-phenylcycloprop-2-ene-1,1-dicarboxylate) (3av)

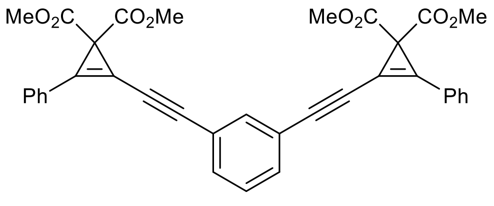

3av

Following **GPD**, a mixture of $(Me_2S)AuCl$ (2.95 mg, 10.0 μmol, 10.0 mol%), **L1** (4.20 mg, 20.0 μmol, 20.0 mol%), CpBX **1k** (120 mg, 0.200 mmol, 2.00 equiv.), diyne **2av** (12.6 mg, 0.100 mmol, 1.00 equiv.) and CH_3CN (2.0 mL) was stirred at room temperature for 24 hours. Flash column chromatography on silica gel (eluent: pentane/ethyl acetate = 3:1) afforded **3av** in 92% yield (54.2 mg, 92.4 μmol) as a light-yellow solid and **4** in 99% NMR yield. **M.p.** 175 – 176 °C. **TLC**: R_f (*n*-hexane/EtOAc = 3:1) = 0.15; **^{1}H NMR** (400 MHz, $CDCl_3$) δ 7.82 (t, *J* = 1.6 Hz, 1H, Ar*H*), 7.72 – 7.66 (m, 4H, Ar*H*), 7.60 (dd, *J* = 7.8, 1.7 Hz, 2H, Ar*H*), 7.51 – 7.43 (m, 6H, Ar*H*), 7.44 – 7.35 (m, 1H, Ar*H*), 3.78 (s, 12H, CO_2CH_3); **^{13}C NMR** (101 MHz, $CDCl_3$) δ 169.5, 135.7, 133.0, 131.1, 130.6, 129.1, 128.9, 124.4, 122.5, 111.2, 101.0, 91.2, 76.5, 52.8, 36.9; **IR** (ν_{max}, cm^{-1}) 3061 (w), 3003 (w), 2952 (w), 2845 (w), 2200 (w), 1727 (s), 1591 (w), 1475 (m), 1434 (m), 1278 (s), 1242 (s), 1139 (w), 1060 (s), 973 (m), 761 (s), 736 (m); **HRMS** (ESI/QTOF) m/z: $[M + Na]^+$ Calcd for $C_{36}H_{26}NaO_8^+$ 609.1520; Found 609.1535.

5.2. Substrate scope of cyclopropenylbenziodoxoles CpBXs

5.2.1. Synthesis and characterization of ethyl 2-hexyl-3-((triisopropylsilyl)ethynyl)cycloprop-2-ene-1-carboxylate (3aw)

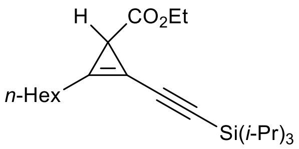

3aw

Following **GPD**, a mixture of $(Me_2S)AuCl$ (2.95 mg, 10.0 μmol, 5.00 mol%), **L1** (4.20 mg, 20.0 μmol, 10.0 mol%), CpBX **1a** (113 mg, 0.200 mmol, 1.00 equiv.), terminal alkyne **2aw** (36.5 mg, 0.200 mmol, 1.00 equiv.) and CH_3CN (2.0 mL) was stirred at room temperature for 6 hours. Flash column chromatography on silica gel (eluent: pentane/ethyl acetate = 50:1) afforded **3aw** in 93% yield (70.3 mg, 187 μmol) as a colourless oil and **4** in 98% NMR yield. **TLC**: R_f (*n*-hexane/EtOAc = 10:1) = 0.73; **^{1}H NMR** (400 MHz, $CDCl_3$) δ 4.22 – 4.05 (m, 2H, $CO_2CH_2CH_3$), 2.51 (td, *J* = 7.1, 1.8 Hz, 2H, CH_2CH_2C), 2.42 (s, 1H, $CHCO_2$), 1.66 – 1.52 (m, 2H, CH_2CH_2C), 1.45 – 1.35 (m, 2H, CH_2), 1.35 – 1.18 (m, 7H, CH_2 & $CO_2CH_2CH_3$), 1.18 – 0.99 (m, 21H, Si(*i*-Pr)$_3$), 0.92 – 0.81 (m, 3H, CH_3); **^{13}C NMR** (101 MHz, $CDCl_3$) δ 174.1, 116.5, 103.3, 93.1, 91.1, 60.5, 31.6, 28.9, 26.6, 26.2, 25.6, 22.7, 18.7, 14.5, 14.2, 11.3; **IR** (ν_{max}, cm^{-1}) 2956 (s), 2942 (s), 2931 (s), 2897 (m), 2866 (s), 2150 (m), 1790 (w), 1728 (s), 1463 (s), 1384 (w), 1368 (m), 1332 (w), 1245 (m), 1179 (s), 1095 (w), 1073 (w), 1019 (m), 997 (m), 920 (w), 882 (s), 781 (w); **HRMS** (ESI/QTOF) m/z: $[M + H]^+$ Calcd for $C_{23}H_{41}O_2Si^+$ 377.2870; Found 377.2872.

5.2.2. Synthesis and characterization of ethyl 2-phenethyl-3-((triisopropylsilyl)ethynyl)cycloprop-2-ene-1-carboxylate (3ax)

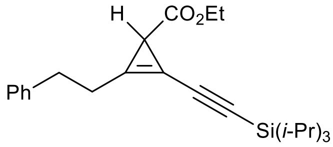

3ax

Following **GPD**, a mixture of $(Me_2S)AuCl$ (2.95 mg, 10.0 μmol, 5.00 mol%), **L1** (4.20 mg, 20.0 μmol, 10.0 mol%), CpBX **1c** (117 mg, 0.200 mmol, 1.00 equiv.), terminal alkyne **2aw** (36.5 mg, 0.200 mmol, 1.00 equiv.) and CH_3CN (2.0 mL) was stirred at room temperature for 4 hours. Flash column chromatography on silica gel (eluent: pentane/ethyl acetate = 50:1) afforded **3ax** in 91% yield (72.3 mg, 182 μmol) as a colorless oil and **4** in 93% NMR yield. **TLC**: R_f (*n*-hexane/EtOAc = 20:1) = 0.35; **^{1}H NMR** (400 MHz, $CDCl_3$) δ 7.34 – 7.15 (m, 5H, Ar*H*), 4.24 – 4.02 (m, 2H, $CO_2CH_2CH_3$), 3.00 – 2.90 (m, 2H, CH_2), 2.90 – 2.76 (m, 2H, CH_2), 2.43 (s, 1H, $CHCO_2$), 1.24 (t, *J* = 7.1 Hz, 3H, $CO_2CH_2CH_3$), 1.14 – 1.06 (m, 21H, Si(*i*-Pr)$_3$); **^{13}C NMR** (101 MHz, $CDCl_3$) δ 174.0, 140.6, 128.6, 128.5, 126.4, 115.2, 104.0, 92.9, 91.9, 60.6, 32.8, 28.1, 25.8, 18.7, 14.5, 11.3; **IR** (ν_{max}, cm^{-1}) 2944 (s), 2892 (m), 2866 (s), 2149 (m), 1789 (w), 1725 (s), 1604 (w), 1497 (m), 1465 (m), 1388 (w), 1333 (m), 1245 (m), 1181 (s), 1072 (m), 1022 (m), 997 (m), 883 (s), 783 (w), 746 (m); **HRMS** (Sicrit plasma/LTQ-Orbitrap) m/z: $[M + H]^+$ Calcd for $C_{25}H_{37}O_2Si^+$ 397.2557; Found 397.2544.

5.2.3. Synthesis and characterization of *tert*-butyl 2-hexyl-3-((triisopropylsilyl)ethynyl)cycloprop-2-ene-1-carboxylate (3ay)

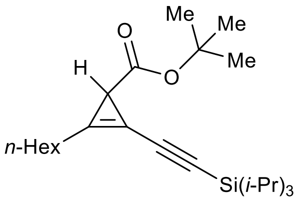

3ay

Following **GPD**, a mixture of (Me_2S)AuCl (2.95 mg, 10.0 µmol, 5.00 mol%), **L1** (4.20 mg, 20.0 µmol, 10.0 mol%), CpBX **1d** (119 mg, 0.200 mmol, 1.00 equiv.), terminal alkyne **2aw** (36.5 mg, 0.200 mmol, 1.00 equiv.) and CH_3CN (2.0 mL) was stirred at room temperature for 4 hours. Flash column chromatography on silica gel (eluent: pentane/ethyl acetate = 50:1) afforded **3ay** in 96% yield (77.5 mg, 191 µmol) as a colourless oil and **4** in 99% NMR yield. **TLC**: R_f (*n*-hexane/EtOAc = 20:1) = 0.40; **^{1}H NMR** (400 MHz, $CDCl_3$) δ 2.50 (td, *J* = 7.1, 1.7 Hz, 2H, CH_2CH_2C), 2.32 (s, 1H, C*H*CO_2), 1.68 – 1.52 (m, 2H, CH_2CH_2C), 1.42 (s, 9H, $C(CH_3)_3$), 1.44 – 1.36 (m, 2H, CH_2), 1.35 – 1.20 (m, 4H, CH_2), 1.14 – 1.02 (m, 21H, Si(*i*-Pr)$_3$), 0.97 – 0.79 (m, 3H, CH_3); **^{13}C NMR** (101 MHz, $CDCl_3$) δ 173.4, 116.9, 102.9, 93.5, 91.6, 80.2, 31.7, 28.9, 28.3, 26.7, 26.7, 26.3, 22.7, 18.7, 14.2, 11.3; **IR** (ν_{max}, cm^{-1}) 2934 (s), 2866 (s), 2149 (w), 1773 (w), 1717 (m), 1596 (w), 1463 (m), 1392 (w), 1368 (m), 1334 (w), 1252 (m), 1153 (s), 1072 (w), 997 (m), 883 (s); **HRMS** (Sicrit plasma/LTQ-Orbitrap) m/z: $[M + H]^+$ Calcd for $C_{25}H_{45}O_2Si^+$ 405.3183; Found 405.3170.

5.2.4. Synthesis and characterization of 2-phenylpropan-2-yl 2-hexyl-3-((triisopropylsilyl)ethynyl)cycloprop-2-ene-1-carboxylate (3az)

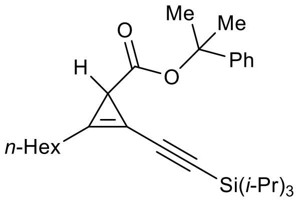

3az

Following **GPD**, a mixture of (Me_2S)AuCl (2.95 mg, 10.0 µmol, 5.00 mol%), **L1** (4.20 mg, 20.0 µmol, 10.0 mol%), CpBX **1e** (119 mg, 0.200 mmol, 1.00 equiv.), terminal alkyne **2aw** (36.5 mg, 0.200 mmol, 1.00 equiv.) and CH_3CN (2.0 mL) was stirred at room temperature for 4 hours. Flash column chromatography on silica gel (eluent: pentane/ethyl acetate = 50:1) afforded **3az** in 91% yield (84.5 mg, 181 µmol) as a colourless oil and **4** in 99% NMR yield. **TLC**: R_f (*n*-hexane/EtOAc = 20:1) = 0.44; **^{1}H NMR** (400 MHz, $CDCl_3$) δ 7.38 – 7.34 (m, 2H, Ar*H*), 7.33 – 7.28 (m, 2H, Ar*H*), 7.25 – 7.19 (m, 1H, Ar*H*), 2.55 (td, *J* = 7.1, 1.6 Hz, 2H, CH_2CH_2C), 2.42 (s, 1H, C*H*CO_2), 1.79 (s, 3H, CH_3), 1.73 (s, 3H, CH_3), 1.63 (pent, *J* = 7.1 Hz, 2H, CH_2CH_2C), 1.47 – 1.39 (m, 2H, CH_2), 1.37 – 1.23 (m, 4H, CH_2), 1.19 – 1.06 (m, 21H, Si(*i*-Pr)$_3$), 0.94 – 0.85 (m, 3H, CH_3); **^{13}C NMR** (101 MHz, $CDCl_3$) δ 172.6, 146.3, 128.3, 126.9, 124.3, 116.7, 103.1, 93.4, 91.6, 81.5, 31.7, 29.3, 28.9, 28.5, 26.8, 26.7, 26.3, 22.7, 18.7, 14.2, 11.4; **IR** (ν_{max}, cm^{-1}) 2942 (m), 2865 (m), 2150 (m), 1773 (w), 1699 (m), 1463 (m), 1266 (m), 1139 (s), 1102 (m), 1073 (m), 1000 (m), 882 (s), 763 (s); **HRMS** (Sicrit plasma/LTQ-Orbitrap) m/z: $[M + H]^+$ Calcd for $C_{30}H_{47}O_2Si^+$ 467.3340; Found 467.3338.

5.2.5. Synthesis and characterization of benzyl 2-hexyl-3-((triisopropylsilyl)ethynyl)cycloprop-2-ene-1-carboxylate (3ba)

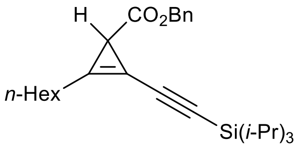

3ba

Following **GPD**, a mixture of $(Me_2S)AuCl$ (2.95 mg, 10.0 μmol, 5.00 mol%), **L1** (4.20 mg, 20.0 μmol, 10.0 mol%), CpBX **1f** (125 mg, 0.200 mmol, 1.00 equiv.), terminal alkyne **2aw** (36.5 mg, 0.200 mmol, 1.00 equiv.) and CH_3CN (2.0 mL) was stirred at room temperature for 5 hours. Flash column chromatography on silica gel (eluent: pentane/ethyl acetate = 40:1) afforded **3ba** in 91% yield (79.9 mg, 182 μmol) as a colourless oil and **4** in 94% NMR yield. **TLC**: R_f (*n*-hexane/EtOAc = 20:1) = 0.40; **^{1}H NMR** (400 MHz, $CDCl_3$) δ 7.40 – 7.27 (m, 5H, Ar*H*), 5.23 – 5.07 (m, 2H, OCH_2Ph), 2.61 – 2.44 (m, 3H, $CHCO_2$ & CH_2CH_2C), 1.66 – 1.53 (m, 2H, CH_2CH_2C), 1.47 – 1.34 (m, 2H, CH_2), 1.33 – 1.22 (m, 4H, CH_2), 1.16 – 1.03 (m, 21H, Si(*i*-Pr)$_3$), 0.95 – 0.82 (m, 3H, CH_3); **^{13}C NMR** (101 MHz, $CDCl_3$) δ 173.9, 136.5, 128.6, 128.1, 128.0, 116.4, 103.5, 93.0, 91.0, 66.2, 31.6, 28.9, 26.6, 26.2, 25.6, 22.6, 18.7, 14.2, 11.3; **IR** (ν_{max}, cm^{-1}) 2956 (s), 2942 (s), 2892 (m), 2865 (s), 2150 (m), 1730 (s), 1498 (w), 1458 (m), 1381 (w), 1336 (w), 1239 (m), 1171 (s), 1072 (w), 995 (m), 883 (s), 740 (m); **HRMS** (Sicrit plasma/LTQ-Orbitrap) m/z: $[M + H]^+$ Calcd for $C_{28}H_{43}O_2Si^+$ 439.3027; Found 439.3013.

5.2.6. Synthesis and characterization of adamantan-1-yl 2-hexyl-3-((triisopropylsilyl)ethynyl)cycloprop-2-ene-1-carboxylate (3bb)

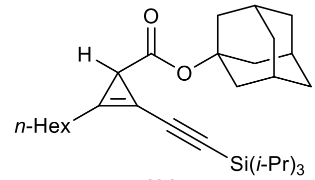

3bb

Following **GPD**, a mixture of $(Me_2S)AuCl$ (2.95 mg, 10.0 μmol, 5.00 mol%), **L1** (4.20 mg, 20.0 μmol, 10.0 mol%), CpBX **1g** (134 mg, 0.200 mmol, 1.00 equiv.), terminal alkyne **2aw** (36.5 mg, 0.200 mmol, 1.00 equiv.) and CH_3CN (2.0 mL) was stirred at room temperature for 5 hours. Flash column chromatography on silica gel (eluent: pentane/ethyl acetate = 40:1) afforded **3bb** in 94% yield (90.6 mg, 188 μmol) as a colourless oil and **4** in 96% NMR yield. **TLC**: R_f (*n*-hexane/EtOAc = 20:1) = 0.47; **^{1}H NMR** (400 MHz, $CDCl_3$) δ 2.56 – 2.44 (m, 2H, CH_2CH_2C), 2.31 (s, 1H, $CHCO_2$), 2.17 – 2.10 (m, 3H, C*H*(adamantyl)), 2.10 – 2.05 (m, 6H, CH_2(adamantyl)), 1.71 – 1.53 (m, 8H, CH_2), 1.45 – 1.35 (m, 2H, CH_2), 1.35 – 1.20 (m, 4H, CH_2), 1.14 – 1.03 (m, 21H, Si(*i*-Pr)$_3$), 0.93 – 0.80 (m, 3H, CH_3); **^{13}C NMR** (101 MHz, $CDCl_3$) δ 173.1, 117.0, 102.8, 93.6, 91.6, 80.2, 41.5, 36.4, 31.7, 31.0, 28.9, 26.7, 26.2, 22.7, 18.7, 14.2, 11.3; **IR** (ν_{max}, cm^{-1}) 2940 (s), 2915 (s), 2865 (s), 2149 (w), 1718 (s), 1459 (m), 1352 (m), 1248 (m), 1183 (s), 1103 (m), 1053 (s), 997 (m), 883 (s); **HRMS** (Sicrit plasma/LTQ-Orbitrap) m/z: $[M + H]^+$ Calcd for $C_{31}H_{51}O_2Si^+$ 483.3653; Found 483.3644.

5.2.7. Synthesis and characterization of (*E*)-3-(4-methoxyphenyl)allyl 2-hexyl-3-((triisopropylsilyl)ethynyl)cycloprop-2-ene-1-carboxylate (3bc)

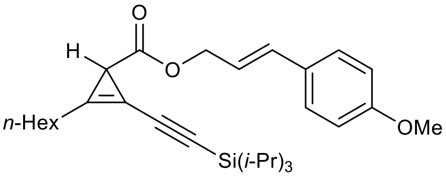

3bc

Following **GPD**, a mixture of (Me_2S)AuCl (2.95 mg, 10.0 μmol, 5.00 mol%), **L1** (4.20 mg, 20.0 μmol, 10.0 mol%), CpBX **1h** (137 mg, 0.200 mmol, 1.00 equiv.), terminal alkyne **2aw** (36.5 mg, 0.200 mmol, 1.00 equiv.) and CH_3CN (2.0 mL) was stirred at room temperature for 10 hours. Flash column chromatography on silica gel (eluent: pentane/ethyl acetate = 10:1) afforded **3bc** in 89% yield (88.0 mg, 178 μmol) as a colourless oil and **4** in 97% NMR yield. **TLC**: R_f (*n*-hexane/EtOAc = 20:1) = 0.31; **^{1}H NMR** (400 MHz, $CDCl_3$) δ 7.38 – 7.28 (m, 2H, Ar*H*), 6.91 – 6.79 (m, 2H, Ar*H*), 6.59 (dt, *J* = 15.6, 1.4 Hz, 1H, $CH_2CH=CH$), 6.15 (dt, *J* = 15.8, 6.5 Hz, 1H, CH_2CH=CH), 4.82 – 4.64 (m, 2H, CH_2CH=CH), 3.81 (s, 3H, OCH_3), 2.54 (td, *J* = 7.1, 2.8 Hz, 2H, CH_2CH_2C), 2.49 (s, 1H, $CHCO_2$), 1.71 – 1.52 (m, 2H, CH_2CH_2C), 1.43 – 1.36 (m, 2H, CH_2), 1.34 – 1.22 (m, 4H, CH_2), 1.14 – 1.02 (m, 21H, Si(*i*-Pr)$_3$), 0.93 – 0.82 (m, 3H, CH_3); **^{13}C NMR** (101 MHz, $CDCl_3$) δ 173.9, 159.6, 133.7, 129.3, 128.0, 121.4, 116.5, 114.1, 103.5, 93.0, 91.1, 65.4, 55.4, 31.6, 28.9, 26.6, 26.2, 25.6, 22.7, 18.7, 14.2, 11.3; **IR** (ν_{max}, cm^{-1}) 2955 (m), 2940 (m), 2896 (m), 2864 (m), 2148 (w), 1727 (m), 1608 (m), 1512 (s), 1463 (m), 1444 (w), 1248 (s), 1168 (s), 1037 (m), 964 (m), 883 (m); **HRMS** (Sicrit plasma/LTQ-Orbitrap) m/z: [M + H]$^+$ Calcd for $C_{31}H_{47}O_3Si^+$ 495.3289; Found 495.3275.

5.2.8. Synthesis and characterization of ethyl 2-hexyl-1-methyl-3-((triisopropylsilyl)ethynyl)cycloprop-2-ene-1-carboxylate (3bd)

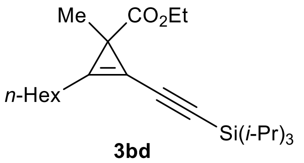

3bd

Following **GPD**, a mixture of (Me_2S)AuCl (2.95 mg, 10.0 μmol, 5.00 mol%), **L1** (4.20 mg, 20.0 μmol, 10.0 mol%), CpBX **1i** (116 mg, 0.200 mmol, 1.00 equiv.), terminal alkyne **2aw** (36.5 mg, 0.200 mmol, 1.00 equiv.) and CH_3CN (2.0 mL) was stirred at room temperature for 6 hours. Flash column chromatography on silica gel (eluent: pentane/ethyl acetate = 40:1) afforded **3bd** in 89% yield (69.2 mg, 177 μmol) as a colourless oil and **4** in 99% NMR yield. **TLC**: R_f (*n*-hexane/EtOAc = 20:1) = 0.31; **^{1}H NMR** (400 MHz, $CDCl_3$) δ 4.17 – 3.99 (m, 2H, $CO_2CH_2CH_3$), 2.48 (t, *J* = 7.2 Hz, 2H, CH_2CH_2C), 1.65 – 1.51 (m, 2H, CH_2CH_2C), 1.43 – 1.35 (m, 5H, CH_2 & CH_3), 1.34 – 1.23 (m, 4H, CH_2), 1.20 (t, *J* = 7.1 Hz, 3H, $CO_2CH_2CH_3$), 1.13 – 1.03 (m, 21H, Si(*i*-Pr)$_3$), 0.95 – 0.80 (m, 3H, CH_3); **^{13}C NMR** (101 MHz, $CDCl_3$) δ 175.4, 122.2, 103.5, 96.9, 93.0, 60.5, 31.6, 30.2, 28.9, 26.8, 25.6, 22.7, 19.1, 18.7, 14.5, 14.2, 11.3; **IR** (ν_{max}, cm^{-1}) 2958 (s), 2932 (s), 2894 (m), 2866 (s), 2145 (m), 1776 (w), 1721 (s), 1464 (m), 1388 (w), 1267 (s), 1252 (s), 1171 (m), 1112 (s), 1074 (w), 1019 (m), 996 (m), 946 (w), 883 (s), 766 (m); **HRMS** (Sicrit plasma/LTQ-Orbitrap) m/z: [M + H]$^+$ Calcd for $C_{24}H_{43}O_2Si^+$ 391.3027; Found 391.3012.

5.2.9. Synthesis and characterization of triisopropyl((2-(3-phenylpropyl)-3-(trifluoromethyl)cycloprop-1-en-1-yl)ethynyl)silane (3be)

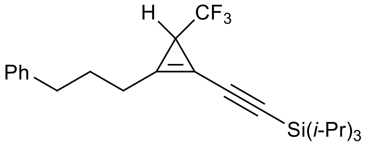

3be

Following **GPD**, a mixture of (Me_2S)AuCl (1.47 mg, 5.00 μmol, 5.00 mol%), **L1** (5.25 mg, 25.0 μmol, 25.0 mol%), CpBX **1j** (59.4 mg, 0.100 mmol, 1.00 equiv.), terminal alkyne **2aw** (27.4 mg, 0.150 mmol, 1.50 equiv.) and CH_3CN (2.0 mL) was stirred at 40 °C for 24 hours. Flash column chromatography on silica gel (eluent: pentane/ethyl acetate = 50:1) afforded **3be** in 78% yield (31.8 mg, 78.2 μmol) as a colourless oil and **4** in 81% NMR yield. **TLC**: R_f (*n*-hexane/EtOAc = 10:1) = 0.75; **^{1}H NMR** (400 MHz, $CDCl_3$) δ 7.34 – 7.27 (m, 2H, Ar*H*), 7.25 – 7.16 (m, 3H, Ar*H*), 2.73 (t, *J* = 7.6 Hz, 2H, CH_2), 2.55 (t, *J* = 7.0 Hz, 2H, CH_2), 2.29 (q, *J* = 4.4 Hz, 1H, $CH(CF_3)$), 2.01 – 1.93 (m, 2H, CH_2), 1.18 – 1.06 (m, 21H, Si(*i*-Pr)$_3$);

^{13}**C NMR** (101 MHz, $CDCl_3$) δ 141.4, 128.7, 128.6, 126.2, 125.7 (q, *J* = 275.8 Hz), 116.3 (q, *J* = 3.0 Hz), 104.8, 92.5, 91.7 (q, *J* = 3.9 Hz), 35.1, 28.3, 25.4, 24.3 (q, *J* = 39.2 Hz), 18.7, 11.3; ^{19}**F NMR** (376 MHz, $CDCl_3$) δ -66.8; **IR** (ν_{max}, cm^{-1}) 2945 (m), 2894 (w), 2867 (m), 2154 (w), 1497 (w), 1462 (w), 1359 (w), 1270 (m), 1185 (w), 1134 (s), 1075 (w), 997 (w), 920 (w), 883 (m), 805 (w), 745 (w); **HRMS** (APPI/LTQ-Orbitrap) m/z: $[M]^+$ Calcd for $C_{24}H_{33}F_3Si^+$ 406.2298; Found 406.2302.

5.2.10. Synthesis and characterization of dimethyl 2-hexyl-3-((triisopropylsilyl)ethynyl)cycloprop-2-ene-1,1-dicarboxylate (3bf)

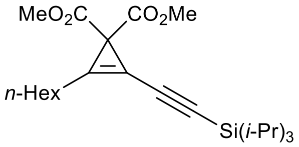

3bf

Following **GPD**, a mixture of $(Me_2S)AuCl$ (1.47 mg, 5.00 μmol, 5.00 mol%), **L1** (2.10 mg, 10.0 μmol, 10.0 mol%), CpBX **1o** (60.8 mg, 0.100 mmol, 1.00 equiv.), terminal alkyne **2aw** (18.2 mg, 0.100 mmol, 1.00 equiv.) and CH_3CN (2.0 mL) was stirred at room temperature for 24 hours. Flash column chromatography on silica gel (eluent: pentane/ethyl acetate = 5:1) afforded **3bf** in 91% yield (38.2 mg, 90.8 μmol) as a colourless oil and **4** in 95% NMR yield. **TLC**: R_f (*n*-hexane/EtOAc = 10:1) = 0.39; ^{1}**H NMR** (400 MHz, $CDCl_3$) δ 3.72 (s, 6H, CO_2CH_3), 2.56 (t, *J* = 7.2 Hz, 2H, CH_2CH_2C), 1.65 – 1.58 (m, 2H, CH_2CH_2C), 1.42 – 1.35 (m, 2H, CH_2), 1.34 – 1.18 (m, 4H, CH_2), 1.14 – 1.03 (m, 21H, Si(*i*-Pr)$_3$), 0.95 – 0.80 (m, 3H, CH_3); ^{13}**C NMR** (101 MHz, $CDCl_3$) δ 170.1, 113.6, 104.6, 91.1, 90.6, 52.4, 37.0, 31.5, 28.8, 26.4, 25.2, 22.6, 18.6, 14.2, 11.2; **IR** (ν_{max}, cm^{-1}) 2946 (m), 2894 (m), 2866 (m), 2155 (w), 1733 (s), 1463 (m), 1434 (m), 1385 (w), 1279 (s), 1245 (s), 1173 (m), 1105 (w), 1063 (s), 997 (m), 883 (s); **HRMS** (ESI/QTOF) m/z: $[M + H]^+$ Calcd for $C_{24}H_{41}O_4Si^+$ 421.2769; Found 421.2775.

5.2.11. Synthesis and characterization of dimethyl 2-phenyl-3-((triisopropylsilyl)ethynyl)cycloprop-2-ene-1,1-dicarboxylate (3bg)

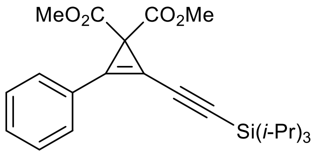

3bg

Following **GPD**, a mixture of $(Me_2S)AuCl$ (2.95 mg, 10.0 μmol, 5.00 mol%), **L1** (4.20 mg, 20.0 μmol, 10.0 mol%), CpBX **1k** (120 mg, 0.200 mmol, 1.00 equiv.), terminal alkyne **2aw** (36.5 mg, 0.200 mmol, 1.00 equiv.) and CH_3CN (2.0 mL) was stirred at room temperature for 17 hours. Flash column chromatography on silica gel (eluent: pentane/ethyl acetate = 5:1) afforded **3bg** in 93% yield (76.8 mg, 186 μmol) as a colourless solid and **4** in 98% NMR yield. **TLC**: R_f (*n*-hexane/EtOAc = 20:1) = 0.16; ^{1}**H NMR** (400 MHz, $CDCl_3$) δ 7.67 – 7.57 (m, 2H, Ar*H*), 7.48 – 7.37 (m, 3H, Ar*H*), 3.74 (s, 6H, CO_2CH_3), 1.24 – 1.04 (m, 21H, Si(*i*-Pr)$_3$); ^{13}**C NMR** (101 MHz, $CDCl_3$) δ 169.5, 130.8, 130.6, 129.1, 124.6, 109.6, 108.5, 92.0, 91.4, 52.6, 37.1, 18.7, 11.3; **IR** (ν_{max}, cm^{-1}) 2948 (m), 2892 (m), 2866 (m), 2147 (w), 1734 (s), 1464 (m), 1439 (m), 1281 (s), 1245 (s), 1142 (w), 1063 (s), 1016 (m), 883 (m), 761 (m); **HRMS** (ESI/QTOF) m/z: $[M + Na]^+$ Calcd for $C_{24}H_{32}NaO_4Si^+$ 435.1962; Found 435.1973. The NMR spectroscopic data is consistent with previous report[52].

5.2.12. Synthesis and characterization of dimethyl 2-(4-fluorophenyl)-3-((triisopropylsilyl)ethynyl)cycloprop-2-ene-1,1-dicarboxylate (3bh)

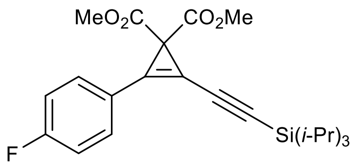

3bh

Following **GPD**, a mixture of (Me_2S)AuCl (1.47 mg, 5.00 µmol, 5.00 mol%), **L1** (2.10 mg, 10.0 µmol, 10.0 mol%), CpBX **1l** (61.8 mg, 0.100 mmol, 1.00 equiv.), terminal alkyne **2aw** (18.2 mg, 0.100 mmol, 1.00 equiv.) and CH_3CN (2.0 mL) was stirred at 40 °C for 4 hours. Flash column chromatography on silica gel (eluent: pentane/ethyl acetate = 5:1) afforded **3bh** in 82% yield (35.4 mg, 82.2 µmol) as a colourless solid and **4** in 96% NMR yield. **M.p.** 105 – 107 °C. **TLC**: R_f (*n*-hexane/EtOAc = 10:1) = 0.30; **^{1}H NMR** (400 MHz, $CDCl_3$) δ 7.70 – 7.54 (m, 2H, Ar*H*), 7.20 – 7.06 (m, 2H, Ar*H*), 3.75 (s, 6H, CO_2CH_3), 1.24 – 1.02 (m, 21H, Si(*i*-Pr)$_3$); **^{13}C NMR** (101 MHz, $CDCl_3$) δ 169.4, 164.1 (d, *J* = 253.5 Hz), 132.7 (d, *J* = 8.9 Hz), 121.0 (d, *J* = 3.3 Hz), 116.5 (d, *J* = 22.4 Hz), 108.7, 108.6, 91.6 (d, *J* = 2.8 Hz), 91.2, 52.7, 37.1, 18.7, 11.3; **^{19}F NMR** (376 MHz, $CDCl_3$) δ -107.2; **IR** (ν_{max}, cm^{-1}) 2948 (m), 2896 (m), 2867 (m), 2723 (w), 2147 (w), 1743 (s), 1599 (m), 1504 (m), 1463 (m), 1438 (m), 1411 (w), 1277 (s), 1236 (s), 1192 (w), 1153 (m), 1063 (s), 1022 (m), 996 (m), 882 (m), 838 (m), 788 (w), 741 (w); **HRMS** (ESI/QTOF) m/z: $[M + Na]^+$ Calcd for $C_{24}H_{31}FNaO_4Si^+$ 453.1868; Found 453.1868.

5.2.13. Synthesis and characterization of dimethyl 2-(4-fluorophenyl)-3-((4-(trifluoromethoxy)phenyl)ethynyl)cycloprop-2-ene-1,1-dicarboxylate (3bi)

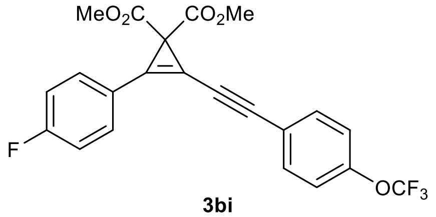

3bi

Following **GPD**, a mixture of (Me_2S)AuCl (1.47 mg, 5.00 µmol, 5.00 mol%), **L1** (2.10 mg, 10.0 µmol, 10.0 mol%), CpBX **1l** (61.8 mg, 0.100 mmol, 1.00 equiv.), terminal alkyne **2n** (18.6 mg, 0.100 mmol, 1.00 equiv.) and CH_3CN (2.0 mL) was stirred at 40 °C for 5 hours. Flash column chromatography on silica gel (eluent: pentane/ethyl acetate = 5:1) afforded **3bi** in 85% yield (37.1 mg, 85.4 µmol) as a colourless oil and **4** in 98% NMR yield. **TLC**: R_f (*n*-hexane/EtOAc = 4:1) = 0.43; **^{1}H NMR** (400 MHz, CD_3CN) δ 7.78 – 7.66 (m, 4H, Ar*H*), 7.38 – 7.34 (m, 2H, Ar*H*), 7.32 – 7.23 (m, 2H, Ar*H*), 3.72 (s, 6H, CO_2CH_3); **^{13}C NMR** (101 MHz, CD_3CN) δ 167.0, 165.2 (d, *J* = 251.8 Hz), 150.9 (q, *J* = 2.0 Hz), 135.0, 133.8 (d, *J* = 9.1 Hz), 122.4, 121.8 (d, *J* = 3.3 Hz), 121.4 (q, *J* = 256.8 Hz), 121.3, 117.6 (d, *J* = 22.8 Hz), 111.8, 101.6, 91.6 (d, *J* = 2.8 Hz), 76.6, 53.3, 37.8; **^{19}F NMR** (376 MHz, CD_3CN) δ -58.5 (OC*F*$_3$), -108.4 (Ar*F*); **IR** (ν_{max}, cm^{-1}) 3106 (w), 3076 (w), 3005 (w), 2956 (w), 2852 (w), 2208 (w), 1733 (s), 1602 (m), 1502 (m), 1437 (w), 1247 (s), 1217 (s), 1159 (s), 1061 (s), 1017 (w), 974 (w), 924 (w), 840 (s), 815 (w), 743 (w); **HRMS** (ESI/QTOF) m/z: $[M + Na]^+$ Calcd for $C_{22}H_{14}F_4NaO_5^+$ 457.0670; Found 457.0662.

5.2.14. Synthesis and characterization of dimethyl 2-(4-bromophenyl)-3-((triisopropylsilyl)ethynyl)cycloprop-2-ene-1,1-dicarboxylate (3bj)

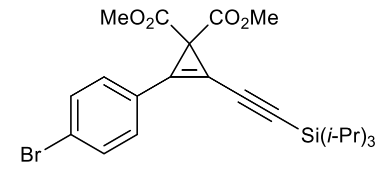

3bj

Following **GPD**, a mixture of $(Me_2S)AuCl$ (1.47 mg, 5.00 μmol, 5.00 mol%), **L1** (2.10 mg, 10.0 μmol, 10.0 mol%), CpBX **1m** (67.9 mg, 0.100 mmol, 1.00 equiv.), terminal alkyne **2aw** (18.2 mg, 0.100 mmol, 1.00 equiv.) and CH_3CN (2.0 mL) was stirred at room temperature for 36 hours. Flash column chromatography on silica gel (eluent: pentane/ethyl acetate = 5:1) afforded **3bj** in 88% yield (43.3 mg, 88.1 μmol) as a colorless oil and **4** in 99% NMR yield. **TLC**: R_f (*n*-hexane/EtOAc = 10:1) = 0.36; **^{1}H NMR** (400 MHz, $CDCl_3$) δ 7.62 – 7.53 (m, 2H, Ar*H*), 7.52 – 7.42 (m, 2H, Ar*H*), 3.74 (s, 6H, CO_2CH_3), 1.24 – 1.03 (m, 21H, Si(*i*-Pr)$_3$); **^{13}C NMR** (101 MHz, $CDCl_3$) δ 169.3, 132.4, 131.8, 125.5, 123.5, 109.6, 108.6, 92.8, 91.1, 52.7, 37.0, 18.7, 11.3; **IR** (ν_{max}, cm^{-1}) 2948 (m), 2892 (m), 2866 (m), 2145 (w), 1734 (s), 1585 (m), 1484 (m), 1463 (m), 1435 (m), 1397 (w), 1277 (s), 1243 (s), 1142 (w), 1065 (s), 1009 (s), 976 (w), 882 (m), 826 (m); **HRMS** (ESI/QTOF) m/z: $[M + Na]^+$ Calcd for $C_{24}H_{31}BrNaO_4Si^+$ 513.1067; Found 513.1074.

5.2.15. Synthesis and characterization of dibenzyl 2-phenyl-3-((triisopropylsilyl)ethynyl)cycloprop-2-ene-1,1-dicarboxylate (3bk)

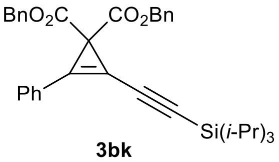

3bk

Following **GPD**, a mixture of $(Me_2S)AuCl$ (1.47 mg, 5.00 μmol, 5.00 mol%), **L1** (2.10 mg, 10.0 μmol, 10.0 mol%), CpBX **1n** (75.2 mg, 0.100 mmol, 1.00 equiv.), terminal alkyne **2aw** (18.2 mg, 0.100 mmol, 1.00 equiv.) and CH_3CN (2.0 mL) was stirred at 40 °C for 2 hours. Flash column chromatography on silica gel (eluent: pentane/ethyl acetate = 5:1) afforded **3bk** in 80% yield (45.2 mg, 80.0 μmol) as a colorless oil and **4** in 95% NMR yield. **TLC**: R_f (*n*-hexane/EtOAc = 10:1) = 0.37; **^{1}H NMR** (400 MHz, $CDCl_3$) δ 7.66 – 7.56 (m, 2H, Ar*H*), 7.42 (tt, *J* = 3.9, 2.4 Hz, 3H, Ar*H*), 7.29 (s, 10H, Ar*H*), 5.36 – 5.09 (m, 4H, OC*H*$_2$Ph), 1.22 – 1.05 (m, 21H, Si(*i*-Pr)$_3$); **^{13}C NMR** (101 MHz, $CDCl_3$) δ 168.8, 135.9, 130.8, 130.7, 129.0, 128.5, 128.0, 127.7, 124.5, 109.7, 108.5, 91.8, 91.4, 67.0, 37.4, 18.7, 11.3; **IR** (ν_{max}, cm^{-1}) 3065 (w), 3034 (w), 2944 (m), 2865 (m), 2146 (w), 1732 (s), 1498 (m), 1456 (m), 1376 (w), 1271 (s), 1226 (s), 1140 (w), 1055 (s), 1015 (m), 883 (m), 759 (m); **HRMS** (ESI/QTOF) m/z: $[M + Na]^+$ Calcd for $C_{36}H_{40}NaO_4Si^+$ 587.2588; Found 587.2582.

5.3. Scale up experiment of σ-type cyclopropenium cation transfer to terminal alkyne

Table 5 | Scale up experiment

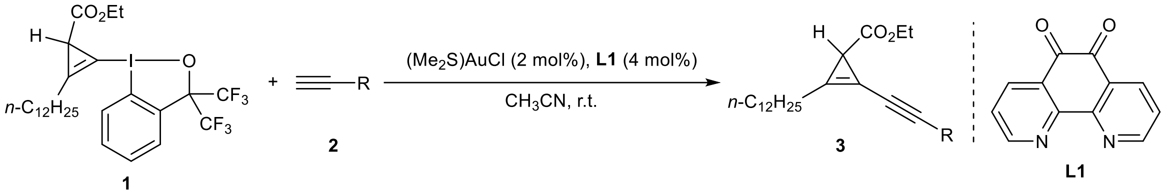

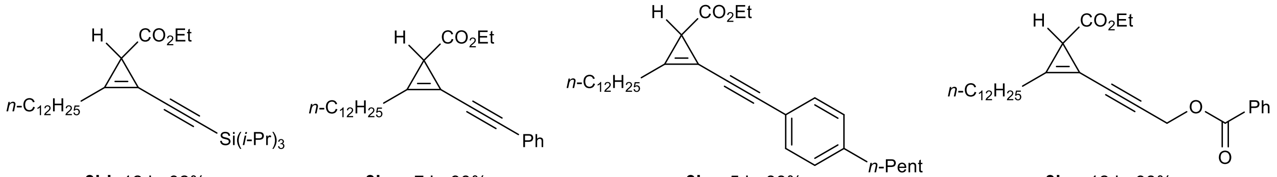

3bl, 12 h, 92%	3bm, 7 h, 93%	3bn, 5 h, 88%	3bo, 12 h, 93%
2 mmol scale	1.2 mmol scale	0.6 mmol scale	1.8 mmol scale

Reaction conditions: CpBX **1** (1.00 equiv.), terminal alkyne **2** (1.00 equiv.), $(Me_2S)AuCl$ (2.00 mol%) and **L1** (4.00 mol%) in CH_3CN were stirred at room temperature for the indicated time.

5.3.1. Synthesis and characterization of ethyl 2-dodecyl-3-((triisopropylsilyl)ethynyl)cycloprop-2-ene-1-carboxylate (3bl)

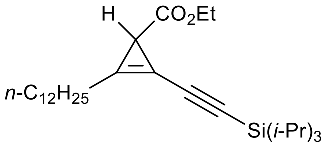

3bl

Following **GPD**, a mixture of $(Me_2S)AuCl$ (11.8 mg, 40.0 µmol, 2.00 mol%), **L1** (16.8 mg, 80.0 µmol, 4.00 mol%), CpBX **1b** (1.29 g, 2.00 mmol, 1.00 equiv.), terminal alkyne **2aw** (365mg, 2.00 mmol, 1.00 equiv.) and CH_3CN (20 mL) was stirred at room temperature for 12 hours. Flash column chromatography on silica gel (eluent: pentane/ethyl acetate = 50:1) afforded **3bl** in 92% yield (851.6 mg, 1.85 mmol) as a colourless oil and **4** in 99% NMR yield. **TLC**: R_f (*n*-hexane/EtOAc = 20:1) = 0.33; **^{1}H NMR** (400 MHz, $CDCl_3$) δ 4.22 – 4.07 (m, 2H, $CO_2CH_2CH_3$), 2.52 (td, *J* = 7.1, 2.0 Hz, 2H, CH_2CH_2C), 2.43 (s, 1H, $CHCO_2$), 1.68 – 1.53 (m, 2H, CH_2CH_2C), 1.45 – 1.35 (m, 2H, CH_2), 1.34 – 1.19 (m, 19H, CH_2 & $CO_2CH_2CH_3$), 1.16 – 1.03 (m, 21H, Si(*i*-Pr)$_3$), 0.96 – 0.81 (m, 3H, CH_3); **^{13}C NMR** (101 MHz, $CDCl_3$) δ 174.1, 116.6, 103.3, 93.1, 91.1, 60.5, 32.1, 29.82, 29.81, 29.79, 29.7, 29.51, 29.49, 29.3, 26.7, 26.2, 25.7, 22.8, 18.7, 14.5, 14.3, 11.4; **IR** (ν_{max}, cm^{-1}) 2956 (m), 2925 (s), 2861 (s), 2149 (w), 1728 (s), 1464 (m), 1370 (w), 1333 (w), 1245 (m), 1179 (s), 1073 (w), 1020 (m), 997 (m), 921 (w), 885 (m), 724 (w); **HRMS** (ESI/QTOF) m/z: $[M + Na]^+$ Calcd for $C_{29}H_{52}NaO_2Si^+$ 483.3629; Found 483.3622.

5.3.2. Synthesis and characterization of ethyl 2-dodecyl-3-(phenylethynyl)cycloprop-2-ene-1-carboxylate (3bm)

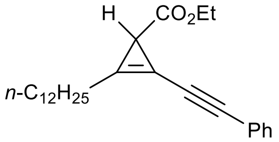

3bm

Following **GPD**, a mixture of $(Me_2S)AuCl$ (7.07 mg, 24.0 µmol, 2.00 mol%), **L1** (10.1 mg, 48.0 µmol, 4.00 mol%), CpBX **1b** (778 mg, 1.20 mmol, 1.00 equiv.), terminal alkyne **2a** (0.13 mL, 1.20 mmol, 1.00 equiv.) and CH_3CN (12 mL) was stirred at room temperature for 7 hours. Flash column chromatography on silica gel (eluent: pentane/ethyl acetate = 50:1) afforded **3bm** in 93% yield (427

mg, 1.12 mmol) as a colourless oil and **4** in 98% NMR yield. **TLC**: R_f (*n*-hexane/EtOAc = 20:1) = 0.39; **^{1}H NMR** (400 MHz, $CDCl_3$) δ 7.54 – 7.43 (m, 2H, Ar*H*), 7.39 – 7.28 (m, 3H, Ar*H*), 4.24 – 4.12 (m, 2H, $CO_2CH_2CH_3$), 2.59 (t, *J* = 7.2 Hz, 2H, CH_2CH_2C), 2.50 (s, 1H, C*H*CO_2), 1.71 – 1.59 (m, 2H, CH_2CH_2C), 1.44 – 1.37 (m, 2H, CH_2), 1.35 – 1.21 (m, 19H, CH_2 & $CO_2CH_2CH_3$), 0.88 (t, *J* = 6.8 Hz, 3H, CH_3); **^{13}C NMR** (101 MHz, $CDCl_3$) δ 174.4, 131.9, 129.1, 128.5, 122.5, 117.0, 99.0, 90.6, 76.7, 60.6, 32.1, 29.82, 29.78, 29.77, 29.7, 29.5, 29.4, 29.3, 26.7, 26.1, 25.3, 22.8, 14.5, 14.3; **IR** (ν_{max}, cm^{-1}) 3058 (w), 2926 (m), 2854 (w), 2200 (w), 1720 (m), 1606 (w), 1490 (w), 1464 (w), 1372 (w), 1332 (w), 1260 (w), 1185 (m), 1094 (w), 1026 (w), 737 (s); **HRMS** (ESI/QTOF) m/z: $[M + H]^+$ Calcd for $C_{26}H_{37}O_2^+$ 381.2788; Found 381.2779.

5.3.3. Synthesis and characterization of ethyl 2-dodecyl-3-((4-pentylphenyl)ethynyl)cycloprop-2-ene-1-carboxylate (3bn)

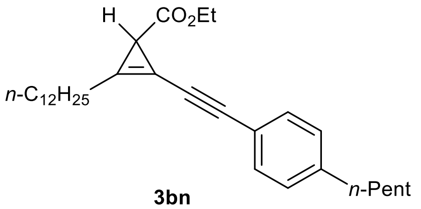

3bn

Following **GPD**, a mixture of (Me_2S)AuCl (3.53 mg, 12.0 μmol, 2.00 mol%), **L1** (5.04 mg, 24.0 μmol, 4.00 mol%), CpBX **1b** (389 mg, 0.600 mmol, 1.00 equiv.), terminal alkyne **2b** (103 mg, 0.600 mmol, 1.00 equiv.) and CH_3CN (6.0 mL) was stirred at room temperature for 5 hours. Flash column chromatography on silica gel (eluent: pentane/ethyl acetate = 50:1) afforded **3bn** in 88% yield (239 mg, 530 μmol) as a colourless oil and **4** in 95% NMR yield. **TLC**: R_f (*n*-hexane/EtOAc = 20:1) = 0.40; **^{1}H NMR** (400 MHz, $CDCl_3$) δ 7.48 – 7.34 (m, 2H, Ar*H*), 7.18 – 7.08 (m, 2H, Ar*H*), 4.23 – 4.11 (m, 2H, $CO_2CH_2CH_3$), 2.62 – 2.56 (m, 4H, CH_2), 2.49 (s, 1H, C*H*CO_2), 1.68 – 1.57 (m, 4H, CH_2), 1.51 – 1.13 (m, 25H, CH_2 & $CO_2CH_2CH_3$), 0.90 – 0.86 (m, 6H, CH_3); **^{13}C NMR** (101 MHz, $CDCl_3$) δ 174.4, 144.4, 131.8, 128.6, 119.7, 116.3, 99.3, 90.7, 76.1, 60.6, 36.1, 32.1, 31.6, 31.0, 29.82, 29.79, 29.77, 29.7, 29.5, 29.4, 29.3, 26.8, 26.1, 25.3, 22.8, 22.6, 14.5, 14.3, 14.1; **IR** (ν_{max}, cm^{-1}) 2955 (m), 2925 (s), 2855 (s), 2201 (w), 1857 (w), 1725 (s), 1607 (w), 1509 (w), 1464 (m), 1370 (w), 1334 (w), 1248 (m), 1180 (s), 1093 (w), 1024 (m), 942 (w), 910 (w), 836 (w), 798 (w), 733 (s); **HRMS** (ESI/QTOF) m/z: $[M + H]^+$ Calcd for $C_{31}H_{47}O_2^+$ 451.3571; Found 451.3577.

5.3.4. Synthesis and characterization of 3-(2-dodecyl-3-(ethoxycarbonyl)cycloprop-1-en-1-yl)prop-2-yn-1-yl benzoate (3bo)

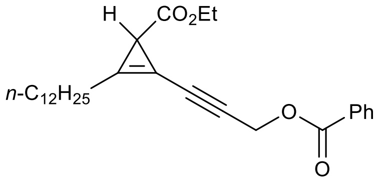

3bo

Following **GPD**, a mixture of (Me_2S)AuCl (10.6 mg, 36.0 μmol, 2.00 mol%), **L1** (15.1 mg, 72.0 μmol, 4.00 mol%), CpBX **1b** (1.17 g, 1.80 mmol, 1.00 equiv.), terminal alkyne **2ao** (288 mg, 1.80 mmol, 1.00 equiv.) and CH_3CN (18 mL) was stirred at room temperature for 12 hours. Flash column chromatography on silica gel (eluent: pentane/ethyl acetate = 10:1) afforded **3bo** in 93% yield (736 mg, 1.68 mmol) as a colourless oil and **4** in 97% NMR yield. **TLC**: R_f (*n*-hexane/EtOAc = 20:1) = 0.20; **^{1}H NMR** (400 MHz, $CDCl_3$) δ 8.23 – 7.97 (m, 2H, Ar*H*), 7.63 – 7.53 (m, 1H, Ar*H*), 7.48 – 7.44 (m, 2H, Ar*H*), 5.13 (s, 2H, OCH_2), 4.21 – 4.09 (m, 2H, $CO_2CH_2CH_3$), 2.53 (t, *J* = 7.3 Hz, 2H, CH_2CH_2C), 2.44 (s, 1H, C*H*CO_2), 1.68 – 1.46 (m, 2H, CH_2CH_2C), 1.47 – 1.11 (m, 21H, CH_2 & $CO_2CH_2CH_3$), 0.88 (t, *J* = 6.8 Hz, 3H, CH_3); **^{13}C NMR** (101 MHz, $CDCl_3$) δ 174.0, 165.9, 133.5, 130.0, 129.5, 128.6, 118.3, 93.3, 90.1, 74.3, 60.7, 53.3, 32.1, 29.79, 29.78, 29.7, 29.6, 29.5, 29.4, 29.3, 26.6, 26.1, 25.1, 22.8, 14.5, 14.3; **IR** (ν_{max}, cm^{-1})

2926 (m), 2854 (m), 2232 (w), 1725 (s), 1603 (w), 1452 (w), 1370 (w), 1264 (s), 1179 (m), 1094 (s), 1069 (m), 1027 (m), 912 (w), 734 (m), 711 (s); **HRMS** (ESI/QTOF) m/z: [M + Na]$^+$ Calcd for $C_{28}H_{38}NaO_4^+$ 461.2662; Found 461.2659.

5.4. Late-stage functionalization of bioactive and drug molecules

5.4.1. Synthesis and characterization of dibenzyl 2-phenyl-3-(3-(((1*S*,4*R*)-4,7,7-trimethyl-3-oxo-2-oxabicyclo[2.2.1]heptane-1-carbonyl)oxy)prop-1-yn-1-yl)cycloprop-2-ene-1,1-dicarboxylate (3bp)

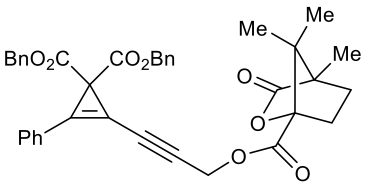

3bp

Following **GPD**, a mixture of (Me_2S)AuCl (1.47 mg, 5.00 µmol, 5.00 mol%), **L1** (2.10 mg, 10.0 µmol, 10.0 mol%), CpBX **1n** (90.3 mg, 0.120 mmol, 1.20 equiv.), alkyne derivative **2ax** (23.6 mg, 0.100 mmol, 1.00 equiv.) and CH_3CN (2.0 mL) was stirred at 40 °C for 3 hours. Flash column chromatography on silica gel (eluent: pentane/ethyl acetate = 5:1) afforded **3bp** in 91% yield (56.3 mg, 91.0 µmol) as a colourless oil and **4** in 87% NMR yield based on **1n**. **ORD**: $[\alpha]_D^{20}$ = -3.0 (c = 0.17, $CHCl_3$); **TLC**: R_f (*n*-hexane/EtOAc = 4:1) = 0.11; **¹H NMR** (400 MHz, $CDCl_3$) δ 7.68 – 7.58 (m, 2H, Ar*H*), 7.49 – 7.41 (m, 3H, Ar*H*), 7.35 – 7.25 (m, 10H, Ar*H*), 5.22 (s, 4H, OC*H*₂Ph), 5.13 (d, *J* = 1.7 Hz, 2H, OC*H*₂), 2.48 (ddd, *J* = 13.5, 10.7, 4.3 Hz, 1H, C*H*₂), 2.09 (ddd, *J* = 13.7, 9.3, 4.6 Hz, 1H, C*H*₂), 1.96 (ddd, *J* = 13.2, 10.8, 4.6 Hz, 1H, C*H*₂), 1.73 (ddd, *J* = 13.4, 9.3, 4.3 Hz, 1H, C*H*₂), 1.14 (s, 3H, C*H*₃), 1.08 (s, 3H, C*H*₃), 0.99 (s, 3H, C*H*₃); **¹³C NMR** (101 MHz, $CDCl_3$) δ 177.9, 168.6, 166.9, 135.7, 131.3, 130.8, 129.1, 128.6, 128.2, 127.8, 124.0, 112.2, 95.7, 90.9, 90.3, 73.5, 67.2, 54.9, 54.7, 53.5, 37.1, 30.7, 29.0, 16.85, 16.77, 9.8; **IR** (ν_{max}, cm^{-1}) 3061 (w), 2976 (w), 2193 (w), 1789 (w), 1732 (m), 1588 (w), 1568 (w), 1496 (w), 1452 (w), 1377 (w), 1335 (w), 1310 (w), 1265 (m), 1233 (m), 1181 (w), 1102 (w), 1058 (m), 1019 (w), 993 (w), 910 (w), 732 (s); **HRMS** (ESI/QTOF) m/z: [M + Na]$^+$ Calcd for $C_{38}H_{34}NaO_8^+$ 641.2146; Found 641.2154.

5.4.2. Synthesis and characterization of dimethyl 2-phenyl-3-(3-(((1*S*,2*R*,4*S*)-1,7,7-trimethylbicyclo[2.2.1]heptan-2-yl)oxy)prop-1-yn-1-yl)cycloprop-2-ene-1,1-dicarboxylate (3bq)

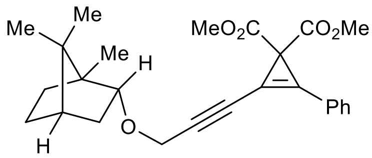

3bq

Following **GPD**, a mixture of (Me_2S)AuCl (1.47 mg, 5.00 µmol, 5.00 mol%), **L1** (2.10 mg, 10.0 µmol, 10.0 mol%), CpBX **1k** (60.0 mg, 0.100 mmol, 1.00 equiv.), alkyne derivative **2ay** (19.2 mg, 0.100 mmol, 1.00 equiv.) and CH_3CN (2.0 mL) was stirred at room temperature for 60 hours. Flash column chromatography on silica gel (eluent: pentane/ethyl acetate = 5:1) afforded **3bq** in 79% yield (33.3 mg, 78.8 µmol) as a colourless oil and **4** in 87% NMR yield. **ORD**: $[\alpha]_D^{20}$ = -27.1 (c = 0.19, $CHCl_3$); **TLC**: R_f (*n*-hexane/EtOAc = 10:1) = 0.19; **¹H NMR** (400 MHz, $CDCl_3$) δ 7.67 – 7.57 (m, 2H, Ar*H*), 7.52 – 7.39 (m, 3H, Ar*H*), 4.52 – 4.26 (m, 2H, OC*H*₂), 3.86 – 3.77 (m, 1H, OC*H*), 3.74 (s, 6H, CO_2C*H*₃), 2.22 – 2.15 (m, 1H, C*H*₂), 2.00 – 1.94 (m, 1H, C*H*₂), 1.75 – 1.65 (m, 2H, C*H*₂), 1.35 – 1.17 (m, 2H, C*H*₂), 1.11 (dd, *J* = 13.2, 3.3 Hz, 1H, C*H*), 0.91 (s, 3H, C*H*₃), 0.87 (s, 3H, C*H*₃), 0.86 (s, 3H, C*H*₃); **¹³C NMR** (101 MHz, $CDCl_3$) δ 169.6, 130.9, 130.6, 129.1, 124.4, 110.1, 100.8, 91.4, 85.3, 71.8, 58.5, 52.7, 49.4, 48.0, 45.1, 36.6, 36.2, 28.3, 26.7, 19.9, 19.0, 13.9; **IR** (ν_{max}, cm^{-1}) 2952 (m), 2877 (m), 2190 (w), 1731 (s), 1589 (w), 1566 (w), 1448 (m), 1436

(m), 1351 (w), 1282 (m), 1240 (s), 1189 (w), 1112 (m), 1089 (m), 1066 (s), 977 (w), 763 (m), 740 (m); **HRMS** (ESI/QTOF) m/z: [M + Na]+ Calcd for $C_{26}H_{30}NaO_5^+$ 445.1985; Found 445.1988.

5.4.3. Synthesis and characterization of ethyl 2-((*S*)-4-((((9*H*-fluoren-9-yl)methoxy)carbonyl)amino)-5-methoxy-5-oxopent-1-yn-1-yl)-3-hexylcycloprop-2-ene-1-carboxylate (3br)

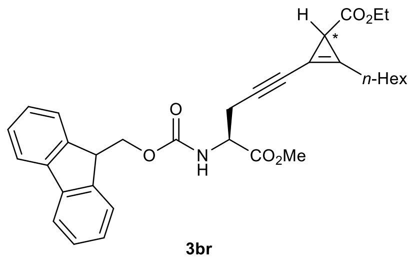

3br

Following **GPD**, a mixture of $(Me_2S)AuCl$ (1.47 mg, 5.00 μmol, 5.00 mol%), **L1** (2.10 mg, 10.0 μmol, 10.0 mol%), CpBX **1a** (73.4 mg, 0.130 mmol, 1.30 equiv.), alkyne derivative **2az** (34.9 mg, 0.100 mmol, 1.00 equiv.) and CH_3CN (2.0 mL) was stirred at 40 °C for 24 hours. Flash column chromatography on silica gel (eluent: pentane/ethyl acetate = 3:1) afforded **3br** in 86% yield (46.8 mg, 86.1 μmol, diastereomer ratio, 1:1) as a colourless oil and **4** in 93% NMR yield based on **1a**. ^{13}C NMR showed the formation of rotamers even if under 60 °C. **TLC**: R_f (*n*-hexane/EtOAc = 4:1) = 0.35; **^{1}H NMR** (400 MHz, $CDCl_3$, 60 °C) δ 7.76 (d, *J* = 7.5 Hz, 2H, Ar*H*), 7.60 (d, *J* = 7.6 Hz, 2H, Ar*H*), 7.39 (t, *J* = 7.5 Hz, 2H, Ar*H*), 7.31 (t, *J* = 7.4 Hz, 2H, Ar*H*), 5.59 (bs, 1H, N*H*), 4.57 (bs, 1H, C*H*CO_2Me), 4.43 (d, *J* = 7.1 Hz, 2H, CH_2(fluorenyl)), 4.25 (t, *J* = 7.0 Hz, 1H, C*H*(fluorenyl)), 4.15 (q, *J* = 7.2 Hz, 2H, $CO_2CH_2CH_3$), 3.79 (s, 3H, CO_2CH_3), 3.01 (bs, 2H, CH_2C≡C), 2.51 (t, *J* = 7.2 Hz, 2H, CH_2CH_2C), 2.39 (s, 1H, C*H*CO_2), 1.71 – 1.52 (m, 2H, CH_2CH_2C), 1.51 – 1.12 (m, 9H, CH_2 & $CO_2CH_2CH_3$), 0.89 (t, *J* = 6.7 Hz, 3H, CH_3); **^{13}C NMR** (101 MHz, $CDCl_3$, 60 °C) δ 174.0, 170.7, 155.7 (bs), 144.1#, 144.0#, 141.6, 127.9, 127.27£, 127.25£, 125.2, 120.1, 116.3, 94.5, 90.9, 71.43$, 71.40$, 67.54*, 67.52*, 60.5, 52.9 (bs), 52.8, 47.5, 31.5, 28.9, 26.7, 26.0, 25.1, 24.5, 22.6, 14.5, 14.0; #These two signals are assigned to one certain quaternary aryl carbon atom on the 9-fluorenyl group; £These two signals are assigned to one certain aryl (C-H) carbon atom on the 9-fluorenyl group; $These two signals are assigned to one certain quaternary carbon atom; *These two signals are assigned to one certain carbon atom; **IR** (ν_{max}, cm^{-1}) 3054 (w), 2958 (w), 2931 (w), 2859 (w), 2308 (w), 1750 (w), 1721 (m), 1617 (w), 1515 (w), 1449 (w), 1373 (w), 1335 (w), 1265 (m), 1207 (m), 1185 (m), 1080 (w), 1030 (w), 870 (w), 734 (s); **HRMS** (ESI/QTOF) m/z: [M + Na]+ Calcd for $C_{33}H_{37}NNaO_6^+$ 566.2513; Found 566.2522. The ratio of the two diastereomer (see below) was determined by chiral supercritical fluid chromatography (SFC).

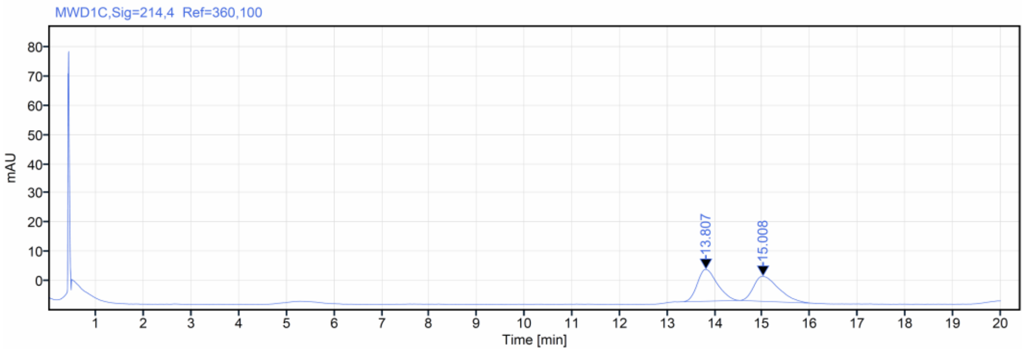

Signal: MWD1C,Sig=214,4 Ref=360,100

RT [min]	Type	Width [min]	Area	Height	Area%	Name
13.807	MM m	1.23	317.08	10.90	50.21	
15.008	MM m	1.78	314.38	8.56	49.79	
		**Sum**	**631.46**			

5.4.4. Synthesis and characterization of dimethyl 2-(3-(((*tert*-butoxycarbonyl)-*L*-phenylalanyl)oxy)prop-1-yn-1-yl)-3-phenylcycloprop-2-ene-1,1-dicarboxylate (3bs)

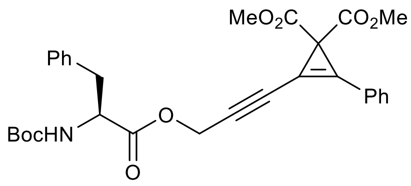

3bs

Following **GPD**, a mixture of (Me_2S)AuCl (1.47 mg, 5.00 µmol, 5.00 mol%), **L1** (2.10 mg, 10.0 µmol, 10.0 mol%), CpBX **1k** (60.0 mg, 0.100 mmol, 1.00 equiv.), alkyne derivative **2ba** (34.9 mg, 0.100 mmol, 1.00 equiv.) and CH_3CN (2.0 mL) was stirred at 40 °C for 18 hours. Flash column chromatography on silica gel (eluent: pentane/ethyl acetate = 4:1) afforded **3bs** in 74% yield (39.4 mg, 73.8 µmol) as a colourless oil and **4** in 99% NMR yield. **ORD**: $[\alpha]_D^{20}$ = -23.5 (c = 0.20, $CHCl_3$). **TLC**: R_f (*n*-hexane/EtOAc = 3:1) = 0.23; **^{1}H NMR** (400 MHz, $CDCl_3$, 60 °C) δ 7.69 – 7.64 (m, 2H, Ar*H*), 7.49 – 7.45 (m, 3H, Ar*H*), 7.35 – 7.27 (m, 2H, Ar*H*), 7.27 – 7.18 (m, 3H, Ar*H*), 5.12 – 4.96 (m, 2H, OCH_2), 4.94 (bs, 1H, N*H*), 4.66 (bs, 1H, NC*H*), 3.78 (s, 6H, CO_2CH_3), 3.21 – 3.10 (m, 2H, CH_2Ph), 1.45 (s, 9H, C(CH_3)$_3$); **^{13}C NMR** (101 MHz, $CDCl_3$, 60 °C) δ 171.2, 169.3, 155.1, 135.9, 131.2, 130.8, 129.5, 129.2, 128.8, 127.3, 124.4, 112.5, 96.1, 91.0, 80.3, 73.4, 54.8, 53.3, 52.6, 38.5, 37.0, 28.5; **IR** (ν_{max}, cm^{-1}) 3381 (w), 2959 (w), 1726 (s), 1497 (m), 1436 (m), 1392 (m), 1370 (m), 1345 (m), 1278 (s), 1245 (s), 1163 (s), 1106 (m), 1061 (s), 1023 (m), 975 (m), 763 (s), 735 (s); **HRMS** (ESI/QTOF) m/z: [M + Na]$^+$ Calcd for $C_{30}H_{31}NNaO_8^+$ 556.1942; Found 556.1946.

5.4.5. Synthesis and characterization of dimethyl 2-(3-(((3a*R*,5*S*,6*R*,6a*R*)-5-((*R*)-2,2-dimethyl-1,3-dioxolan-4-yl)-2,2-dimethyltetrahydrofuro[2,3-*d*][1,3]dioxol-6-yl)oxy)prop-1-yn-1-yl)-3-phenylcycloprop-2-ene-1,1-dicarboxylate (3bt)

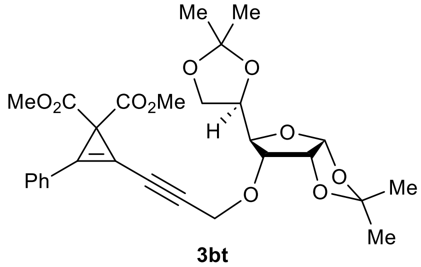

3bt

Following **GPD**, a mixture of (Me_2S)AuCl (1.47 mg, 5.00 µmol, 5.00 mol%), **L1** (2.10 mg, 10.0 µmol, 10.0 mol%), CpBX **1k** (72.0 mg, 0.120 mmol, 1.20 equiv.), alkyne derivative **2bb** (29.8 mg, 0.100 mmol, 1.00 equiv.) and CH_3CN (2.0 mL) was stirred at 40 °C for 2 hours. Flash column chromatography on silica gel (eluent: pentane/ethyl acetate = 10:1) afforded **3bt** in 96% yield (50.5 mg, 95.5 µmol) as a colourless oil and **4** in 95% NMR yield based on **1k**. **ORD**: $[\alpha]_D^{20}$ = +72.5 (c = 0.22, $CHCl_3$). **TLC**: R_f (*n*-hexane/EtOAc = 2:1) = 0.21; **^{1}H NMR** (400 MHz, $CDCl_3$) δ 7.67 – 7.56 (m, 2H, Ar*H*), 7.45 – 7.41 (m, 3H, Ar*H*), 5.80 (d, *J* = 3.7 Hz, 1H, C*H*), 4.77 (t, *J* = 3.9 Hz, 1H, C*H*), 4.72 – 4.53 (m, 2H, OCH_2), 4.37 (td, *J* = 6.9, 3.1 Hz, 1H, C*H*), 4.17 – 4.07 (m, 2H, OCH_2), 4.07 – 3.95 (m, 2H, C*H*), 3.73 (s, 6H, CO_2CH_3), 1.58 (s, 3H, CH_3), 1.45 (s, 3H, CH_3), 1.36 (s, 3H, CH_3), 1.35 (s, 3H, CH_3); **^{13}C NMR** (101 MHz, $CDCl_3$) δ 169.4, 131.1, 130.6, 129.1, 124.1, 113.2, 111.2, 109.9, 103.9, 98.6, 90.8, 78.1, 78.0, 77.8, 74.9, 73.4, 65.3, 58.6, 52.7, 36.6, 26.9, 26.6, 26.4, 25.3; **IR** (ν_{max}, cm^{-1}) 3061 (w), 2988 (w), 2953 (w), 2903 (w), 2191 (w), 1732 (s), 1440 (w), 1375 (m), 1247 (s), 1217 (m), 1164 (m), 1132 (m), 1101 (m), 1062 (s), 1024 (s), 974 (m), 918 (w), 870 (m), 852 (m), 764 (m), 735 (s); **HRMS** (ESI/QTOF) m/z: [M + Na]$^+$ Calcd for $C_{28}H_{32}NaO_{10}^+$ 551.1888; Found 551.1887.

5.4.6. Synthesis and characterization of dimethyl 2-(4-fluorophenyl)-3-(4-((5-((3a*S*,4*S*,6a*R*)-2-oxohexahydro-1*H*-thieno[3,4-*d*]imidazol-4-yl)pentanoyl)oxy)but-1-yn-1-yl)cycloprop-2-ene-1,1-dicarboxylate (3bu)

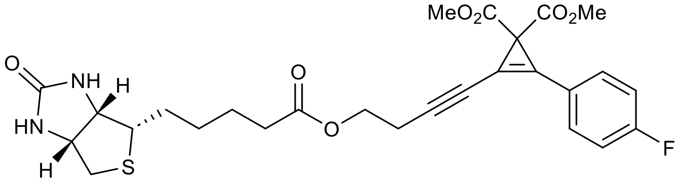

3bu

Following **GPD**, a mixture of $(Me_2S)AuCl$ (1.47 mg, 5.00 μmol, 5.00 mol%), **L1** (2.10 mg, 10.0 μmol, 10.0 mol%), CpBX **1l** (74.2 mg, 0.120 mmol, 1.20 equiv.), alkyne derivative **2bc** (29.6 mg, 0.100 mmol, 1.00 equiv.) and CH_3CN (2.0 mL) was stirred at 40 °C for 11 hours. Flash column chromatography on silica gel (eluent: DCM/MeOH = 20:1) afforded **3bu** in 90% yield (49.1 mg, 90.2 μmol) as a colourless oil and **4** in 98% NMR yield based on **1l**. **ORD**: $[\alpha]_D^{20}$ = +28.6 (c = 0.11, $CHCl_3$). **TLC**: R_f (DCM/MeOH = 20:1) = 0.23; **^{1}H NMR** (400 MHz, $CDCl_3$) δ 7.71 – 7.54 (m, 2H, Ar*H*), 7.19 – 7.07 (m, 2H, Ar*H*), 5.89 (bs, 1H, N*H*), 5.41 (bs, 1H, N*H*), 4.49 – 4.45 (m, 1H, C*H*), 4.29 – 4.25 (m, 3H, C*H* & OCH_2), 3.74 (s, 6H, CO_2CH_3), 3.16 – 3.11 (m, 1H, C*H*), 2.98 – 2.64 (m, 4H, CH_2), 2.37 (t, *J* = 7.5 Hz, 2H, CH_2), 1.76 – 1.60 (m, 4H, CH_2), 1.57 – 1.32 (m, 2H, CH_2); **^{13}C NMR** (101 MHz, $CDCl_3$) δ 173.5, 169.6, 164.0 (d, *J* = 253.4 Hz), 163.7, 132.6 (d, *J* = 8.8 Hz), 120.8 (d, *J* = 3.3 Hz), 116.5 (d, *J* = 22.4 Hz), 108.2, 100.7, 91.4 (d, *J* = 2.8 Hz), 68.2, 62.1, 61.5, 60.2, 55.5, 52.8, 40.7, 36.6, 33.9, 28.4, 28.3, 24.9, 21.0; **^{19}F NMR** (376 MHz, $CDCl_3$) δ -107.3; **IR** (ν_{max}, cm^{-1}) 3057 (w), 2953 (w), 2234 (w), 1730 (m), 1703 (m), 1600 (w), 1506 (w), 1454 (w), 1436 (w), 1266 (m), 1239 (m), 1156 (w), 1064 (m), 841 (w), 733 (s); **HRMS** (ESI/QTOF) m/z: $[M + Na]^+$ Calcd for $C_{27}H_{29}FN_2NaO_7S^+$ 567.1572; Found 567.1575.

5.4.7. Synthesis and characterization of dimethyl 2-(3-(((2*S*,5*R*)-3,3-dimethyl-4,4-dioxido-7-oxo-4-thia-1-azabicyclo[3.2.0]heptane-2-carbonyl)oxy)prop-1-yn-1-yl)-3-phenylcycloprop-2-ene-1,1-dicarboxylate (3bv)

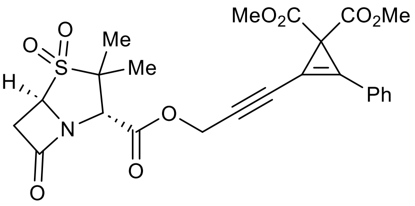

3bv

Following **GPD**, a mixture of $(Me_2S)AuCl$ (1.47 mg, 5.00 μmol, 5.00 mol%), **L1** (2.10 mg, 10.0 μmol, 10.0 mol%), CpBX **1k** (60.0 mg, 0.100 mmol, 1.00 equiv.), alkyne derivative **2bd** (27.1 mg, 0.100 mmol, 1.00 equiv.) and CH_3CN (2.0 mL) was stirred at 40 °C for 17 hours. Flash column chromatography on silica gel (eluent: pentane/ethyl acetate = 2:1) afforded **3bv** in 83% yield (41.8 mg, 83.3 μmol) as a colourless oil and **4** in 99% NMR yield. **ORD**: $[\alpha]_D^{20}$ = +150.2 (c = 0.27, $CHCl_3$). **TLC**: R_f (*n*-hexane/EtOAc = 1:1) = 0.49; **^{1}H NMR** (400 MHz, $CDCl_3$) δ 7.68 – 7.55 (m, 2H, Ar*H*), 7.51 – 7.42 (m, 3H, Ar*H*), 5.22 – 4.94 (m, 2H, OCH_2), 4.64 (dd, *J* = 4.3, 2.1 Hz, 1H, NC*H*), 4.45 (s, 1H, C*H*CO_2), 3.74 (s, 6H, CO_2CH_3), 3.55 – 3.36 (m, 2H, CH_2), 1.65 (s, 3H, CH_3), 1.46 (s, 3H, CH_3); **^{13}C NMR** (101 MHz, $CDCl_3$) δ 170.8, 169.2, 166.4, 131.5, 130.8, 129.2, 123.9, 112.6, 94.9, 90.1, 74.1, 63.1, 63.0, 61.2, 54.2, 52.8, 38.5, 36.7, 20.4, 18.7; **IR** (ν_{max}, cm^{-1}) 3058 (w), 2989 (w), 2950 (w), 1802 (w), 1766 (w), 1732 (w), 1437 (w), 1325 (w), 1266 (m), 1184 (w), 1159 (w), 1119 (w), 1065 (w), 964 (w), 910 (w), 732 (s); **HRMS** (ESI/QTOF) m/z: $[M + Na]^+$ Calcd for $C_{24}H_{23}NNaO_9S^+$ 524.0986; Found 524.0997.

5.4.8. Synthesis and characterization of ethyl 2-(3-((2-(4-(4-chlorobenzoyl)phenoxy)-2-methylpropanoyl)oxy)prop-1-yn-1-yl)-3-hexylcycloprop-2-ene-1-carboxylate (3bw)

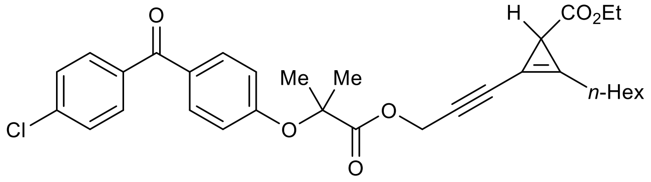

3bw

Following **GPD**, a mixture of (Me_2S)AuCl (1.47 mg, 5.00 µmol, 5.00 mol%), **L1** (2.10 mg, 10.0 µmol, 10.0 mol%), CpBX **1a** (56.4 mg, 0.100 mmol, 1.00 equiv.), alkyne derivative **2be** (35.7 mg, 0.100 mmol, 1.00 equiv.) and CH_3CN (2.0 mL) was stirred at room temperature for 18 hours. Flash column chromatography on silica gel (eluent: pentane/ethyl acetate = 5:1) afforded **3bw** in 85% yield (46.8 mg, 84.9 µmol) as a colourless oil and **4** in 95% NMR yield. **TLC**: R_f (*n*-hexane/EtOAc = 10:1) = 0.15; **^{1}H NMR** (400 MHz, $CDCl_3$) δ 7.71 (t, *J* = 8.3 Hz, 4H, Ar*H*), 7.53 – 7.36 (m, 2H, Ar*H*), 6.98 – 6.79 (m, 2H, Ar*H*), 4.97 (s, 2H, $CO_2CH_2CH_3$), 4.11 (q, *J* = 7.0 Hz, 2H, OCH_2), 2.49 (t, *J* = 7.3 Hz, 2H, CH_2CH_2C), 2.39 (s, 1H, $CHCO_2$), 1.68 (s, 6H, $OCCH_3$), 1.61 – 1.48 (m, 2H, CH_2CH_2C), 1.38 – 1.13 (m, 9H, CH_2 & $CO_2CH_2CH_3$), 0.86 (t, *J* = 6.8 Hz, 3H, CH_3); **^{13}C NMR** (101 MHz, $CDCl_3$) δ 194.2, 173.8, 173.0, 159.4, 138.5, 136.4, 132.1, 131.3, 130.8, 128.6, 118.8, 117.8, 92.3, 89.8, 79.4, 74.8, 60.7, 53.9, 31.5, 28.8, 26.5, 26.0, 25.5, 25.4, 24.9, 22.6, 14.4, 14.1; **IR** (ν_{max}, cm^{-1}) 2957 (w), 2934 (w), 2871 (w), 2232 (w), 1925 (w), 1744 (m), 1723 (m), 1655 (m), 1597 (s), 1505 (w), 1466 (w), 1390 (w), 1369 (w), 1304 (m), 1279 (s), 1248 (s), 1172 (s), 1129 (s), 1090 (m), 1014 (m), 953 (w), 928 (s), 853 (m), 844 (m), 763 (s), 737 (m); **HRMS** (ESI/QTOF) m/z: $[M + H]^+$ Calcd for $C_{32}H_{36}ClO_6^+$ 551.2195; Found 551.2188.

5.4.9. Synthesis and characterization of dimethyl 2-(3-((2-(4-(2,2-dichlorocyclopropyl)phenoxy)-2-methylpropanoyl)oxy)prop-1-yn-1-yl)-3-phenylcycloprop-2-ene-1,1-dicarboxylate (3bx)

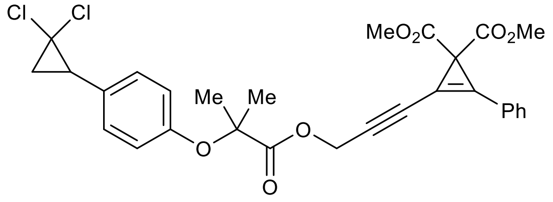

3bx

Following **GPD**, a mixture of (Me_2S)AuCl (1.47 mg, 5.00 µmol, 5.00 mol%), **L1** (2.10 mg, 10.0 µmol, 10.0 mol%), CpBX **1k** (78.0 mg, 0.130 mmol, 1.30 equiv.), alkyne derivative **2bf** (32.7 mg, 0.100 mmol, 1.00 equiv.) and CH_3CN (2.0 mL) was stirred at 40 °C for 3 hours. Flash column chromatography on silica gel (eluent: pentane/ethyl acetate = 5:1) afforded **3bx** in 94% yield (52.6 mg, 94.4 µmol) as a colourless oil and **4** in 95% NMR yield based on **1k**. **TLC**: R_f (*n*-hexane/EtOAc = 3:1) = 0.37; **^{1}H NMR** (400 MHz, $CDCl_3$) δ 7.68 – 7.57 (m, 2H, Ar*H*), 7.51 – 7.41 (m, 3H, Ar*H*), 7.16 – 7.07 (m, 2H, Ar*H*), 6.90 – 6.81 (m, 2H, Ar*H*), 5.06 (s, 2H, OCH_2), 3.75 (s, 6H, CO_2CH_3), 2.75 (dd, *J* = 10.7, 8.3 Hz, 1H, C*H*), 1.83 (dd, *J* = 10.7, 7.4 Hz, 1H, CH_2), 1.71 (dd, *J* = 8.4, 7.4 Hz, 1H, CH_2), 1.63 (s, 6H, CH_3); **^{13}C NMR** (101 MHz, $CDCl_3$) δ 173.5, 169.3, 154.7, 131.3, 130.7, 129.8, 129.2, 128.8, 124.1, 119.3, 112.1, 96.1, 90.6, 79.3, 73.1, 60.9, 53.5, 52.8, 36.7, 34.9, 25.8, 25.5, 25.4; **IR** (ν_{max}, cm^{-1}) 3060 (w), 2995 (w), 2953 (w), 2844 (w), 2194 (w), 1732 (s), 1610 (w), 1583 (w), 1571 (w), 1511 (m), 1435 (m), 1386 (w), 1368 (w), 1342 (w), 1270 (s), 1242 (s), 1195 (w), 1175 (m), 1125 (s), 1061 (m), 958 (w), 834 (w), 765 (s), 736 (s); **HRMS** (ESI/QTOF) m/z: $[M + Na]^+$ Calcd for $C_{29}H_{26}Cl_2NaO_7^+$ 579.0948; Found 579.0955.

5.4.10. Synthesis and characterization of dimethyl (*S*)-2-(4-fluorophenyl)-3-(3-((2-(6-methoxynaphthalen-2-yl)propanoyl)oxy)prop-1-yn-1-yl)cycloprop-2-ene-1,1-dicarboxylate (3by)

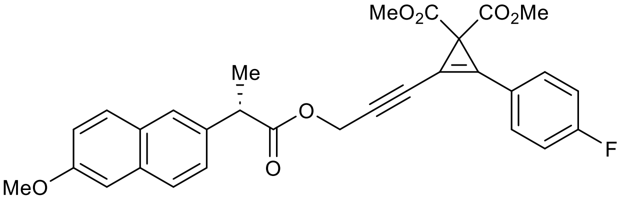

3by

Following **GPD**, a mixture of (Me_2S)AuCl (1.47 mg, 5.00 µmol, 5.00 mol%), **L1** (2.10 mg, 10.0 µmol, 10.0 mol%), CpBX **1l** (74.2 mg, 0.120 mmol, 1.20 equiv.), alkyne derivative **2bg** (26.8 mg, 0.100 mmol, 1.00 equiv.) and CH_3CN (2.0 mL) was stirred at 40 °C for 12 hours. Flash column chromatography on silica gel (eluent: pentane/ethyl acetate = 5:1) afforded **3by** in 89% yield (46.1 mg, 89.3 µmol) as a colourless oil and **4** in 98% NMR yield based on **1l**. **ORD**: $[\alpha]_D^{20}$ = -28.2 (c = 0.43, $CHCl_3$). **TLC**: R_f (*n*-hexane/EtOAc = 4:1) = 0.19; **^{1}H NMR** (400 MHz, $CDCl_3$) δ 7.77 – 7.66 (m, 3H, Ar*H*), 7.64 – 7.54 (m, 2H, Ar*H*), 7.42 (dd, *J* = 8.5, 1.9 Hz, 1H, Ar*H*), 7.18 – 7.04 (m, 4H, Ar*H*), 5.13 – 4.78 (m, 2H, OCH_2), 3.94 (q, *J* = 7.2 Hz, 1H, C*H*), 3.91 (s, 3H, OCH_3), 3.74 (s, 3H, OCH_3), 3.73 (s, 3H, OCH_3), 1.62 (d, *J* = 7.2 Hz, 3H, CH_3); **^{13}C NMR** (101 MHz, $CDCl_3$) δ 173.9, 169.3, 164.3 (d, *J* = 253.9 Hz), 157.9, 135.1, 133.9, 132.9 (d, *J* = 8.9 Hz), 129.4, 129.0, 127.4, 126.22, 126.19, 120.6 (d, *J* = 3.3 Hz), 119.2, 116.6 (d, *J* = 22.4 Hz), 110.7, 105.7, 96.8, 90.4 (d, *J* = 2.8 Hz), 72.6, 55.4, 53.0, 52.8, 45.3, 36.7, 18.7; **^{19}F NMR** (376 MHz, $CDCl_3$) δ -106.6; **IR** (ν_{max}, cm^{-1}) 2954 (m), 2926 (m), 2848 (w), 2197 (w), 1734 (s), 1633 (w), 1603 (m), 1506 (m), 1484 (w), 1460 (w), 1437 (m), 1392 (w), 1367 (w), 1235 (s), 1177 (s), 1152 (s), 1062 (m), 927 (m), 841 (m), 730 (m); **HRMS** (ESI/QTOF) m/z: [M + Na]$^+$ Calcd for $C_{30}H_{25}FNaO_7^+$ 539.1477; Found 539.1475.

5.4.11. Synthesis and characterization of ethyl 2-(3-((3-(4,5-diphenyloxazol-2-yl)propanoyl)oxy)prop-1-yn-1-yl)-3-hexylcycloprop-2-ene-1-carboxylate (3bz)

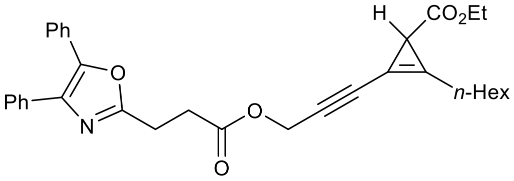

3bz

Following **GPD**, a mixture of (Me_2S)AuCl (1.47 mg, 5.00 µmol, 5.00 mol%), **L1** (2.10 mg, 10.0 µmol, 10.0 mol%), CpBX **1a** (67.7 mg, 0.120 mmol, 1.20 equiv.), alkyne derivative **2bh** (33.1 mg, 0.100 mmol, 1.00 equiv.) and CH_3CN (2.0 mL) was stirred at room temperature for 22 hours. Flash column chromatography on silica gel (eluent: pentane/ethyl acetate = 10:1) afforded **3bz** in 97% yield (51.2 mg, 97.4 µmol) as a colourless oil and **4** in 98% NMR yield based on **1a**. **TLC**: R_f (*n*-hexane/EtOAc = 5:1) = 0.26; **^{1}H NMR** (400 MHz, $CDCl_3$) δ 7.68 – 7.60 (m, 2H, Ar*H*), 7.60 – 7.53 (m, 2H, Ar*H*), 7.40 – 7.28 (m, 6H, Ar*H*), 4.95 (s, 2H, OCH_2), 4.21 – 4.09 (m, 2H, $CO_2CH_2CH_3$), 3.32 – 3.12 (m, 2H, CH_2), 3.00 – 2.96 (m, 2H, CH_2), 2.52 (t, *J* = 7.3 Hz, 2H, CH_2CH_2C), 2.42 (s, 1H, $CHCO_2$), 1.65 – 1.53 (m, 2H, CH_2CH_2C), 1.44 – 1.18 (m, 9H, CH_2 & $CO_2CH_2CH_3$), 0.95 – 0.76 (m, 3H, CH_3); **^{13}C NMR** (101 MHz, $CDCl_3$) δ 174.0, 171.3, 161.5, 145.6, 135.2, 132.5, 129.0, 128.8, 128.7, 128.6, 128.2, 128.0, 126.6, 118.4, 93.0, 90.0, 74.3, 60.7, 53.1, 31.5, 31.0, 28.9, 26.5, 26.0, 25.0, 23.5, 22.6, 14.4, 14.1; **IR** (ν_{max}, cm^{-1}) 3058 (w), 2956 (m), 2931 (m), 2860 (w), 2229 (w), 1738 (s), 1683 (m), 1597 (m), 1581 (m), 1503 (w), 1447 (m), 1373 (w), 1328 (w), 1244 (m), 1210 (m), 1156 (s), 1054 (m), 1022 (m), 964 (m), 871 (w), 766 (m); **HRMS** (ESI/QTOF) m/z: [M + H]$^+$ Calcd for $C_{33}H_{36}NO_5^+$ 526.2588; Found 526.2601.

5.4.12. Synthesis and characterization of ethyl 2-hexyl-3-(3-(2-(11-oxo-6,11-dihydrodibenzo[*b*,*e*]oxepin-2-yl)acetoxy)prop-1-yn-1-yl)cycloprop-2-ene-1-carboxylate (3ca)

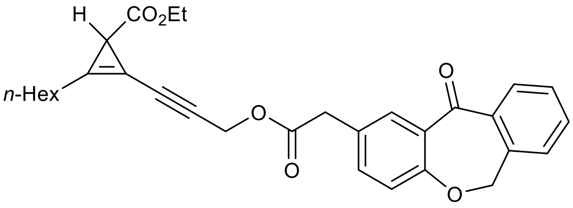

3ca

Following **GPD**, a mixture of (Me_2S)AuCl (1.47 mg, 5.00 µmol, 5.00 mol%), **L1** (2.10 mg, 10.0 µmol, 10.0 mol%), CpBX **1a** (67.7 mg, 0.120 mmol, 1.20 equiv.), alkyne derivative **2bi** (30.6 mg, 0.100 mmol, 1.00 equiv.) and CH_3CN (2.0 mL) was stirred at room temperature for 22 hours. Flash column chromatography on silica gel (eluent: pentane/ethyl acetate = 8:1) afforded **3ca** in 95% yield (47.5 mg, 94.9 µmol) as a colourless oil and **4** in 99% NMR yield based on **1a**. **TLC**: R_f (*n*-hexane/EtOAc = 5:1) = 0.24; **^{1}H NMR** (400 MHz, $CDCl_3$) δ 8.11 (d, *J* = 2.4 Hz, 1H, Ar*H*), 7.88 (dd, *J* = 7.7, 1.4 Hz, 1H, Ar*H*), 7.55 (td, *J* = 7.4, 1.4 Hz, 1H, Ar*H*), 7.50 – 7.39 (m, 2H, Ar*H*), 7.35 (dd, *J* = 7.5, 1.3 Hz, 1H, Ar*H*), 7.03 (d, *J* = 8.4 Hz, 1H, Ar*H*), 5.18 (s, 2H, OCH_2), 4.91 (s, 2H, OCH_2), 4.20 – 4.08 (m, 2H, $CO_2CH_2CH_3$), 3.70 (s, 2H, CH_2), 2.53 (t, *J* = 7.3 Hz, 2H, CH_2CH_2C), 2.42 (s, 1H, $CHCO_2$), 1.64 – 1.51 (m, 2H, CH_2CH_2C), 1.44 – 1.15 (m, 9H, CH_2 & $CO_2CH_2CH_3$), 0.97 – 0.78 (m, 3H, CH_3); **^{13}C NMR** (101 MHz, $CDCl_3$) δ 190.9, 174.0, 170.7, 160.7, 140.5, 136.4, 135.6, 132.9, 132.7, 129.6, 129.4, 127.9, 127.3, 125.3, 121.3, 118.4, 93.0, 90.0, 74.4, 73.7, 60.7, 53.3, 39.9, 31.5, 28.9, 26.5, 26.0, 25.0, 22.6, 14.4, 14.1; **IR** (ν_{max}, cm^{-1}) 2957 (m), 2930 (m), 2863 (w), 2233 (w), 1738 (s), 1721 (s), 1649 (m), 1611 (m), 1494 (m), 1455 (m), 1414 (m), 1372 (m), 1300 (s), 1243 (s), 1186 (s), 1138 (s), 1122 (s), 1016 (s), 936 (w), 831 (w), 761 (m), 737 (m); **HRMS** (ESI/QTOF) m/z: $[M + Na]^+$ Calcd for $C_{31}H_{32}NaO_6^+$ 523.2091; Found 523.2090.

5.4.13. Synthesis and characterization of 4-(3-(ethoxycarbonyl)-2-hexylcycloprop-1-en-1-yl)but-3-yn-1-yl 2-(3-cyano-4-isobutoxyphenyl)-4-methylthiazole-5-carboxylate (3cb)

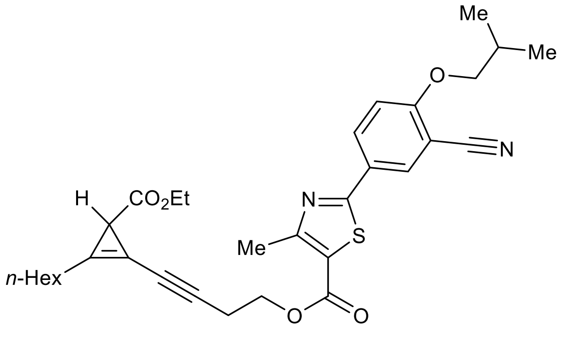

3cb

Following **GPD**, a mixture of (Me_2S)AuCl (1.47 mg, 5.00 µmol, 5.00 mol%), **L1** (2.10 mg, 10.0 µmol, 10.0 mol%), CpBX **1a** (67.7 mg, 0.120 mmol, 1.20 equiv.), alkyne derivative **2bj** (36.9 mg, 0.100 mmol, 1.00 equiv.) and CH_3CN (2.0 mL) was stirred at room temperature for 3 hours. Flash column chromatography on silica gel (eluent: pentane/ethyl acetate = 3:1) afforded **3cb** in 99% yield (55.8 mg, 99.2 µmol) as a colourless oil and **4** in 98% NMR yield based on **1a**. **TLC**: R_f (*n*-hexane/EtOAc = 4:1) = 0.34; **^{1}H NMR** (400 MHz, $CDCl_3$) δ 8.17 (d, *J* = 2.3 Hz, 1H, Ar*H*), 8.08 (dd, *J* = 8.9, 2.3 Hz, 1H, Ar*H*), 7.00 (d, *J* = 8.9 Hz, 1H, Ar*H*), 4.42 (t, *J* = 6.7 Hz, 2H, OCH_2), 4.18 – 4.06 (m, 2H, $CO_2CH_2CH_3$), 3.88 (d, *J* = 6.5 Hz, 2H, OCH_2), 2.88 (t, *J* = 6.7 Hz, 2H, CH_2), 2.75 (s, 3H, CH_3), 2.49 (t, *J* = 7.3 Hz, 2H, CH_2CH_2C), 2.37 (s, 1H, $CHCO_2$), 2.24 – 2.14 (m, 1H, $CH_2CH(CH_3)_2$), 1.66 – 1.48 (m, 2H, CH_2CH_2C), 1.47 – 1.16 (m, 9H, CH_2 & $CO_2CH_2CH_3$), 1.07 (d, *J* = 6.8 Hz, 6H, $CH_2CH(CH_3)_2$), 0.92 – 0.75 (m, 3H, CH_3); **^{13}C NMR** (101 MHz, $CDCl_3$) δ 174.3, 167.6, 162.6, 161.8, 161.7, 132.7, 132.2, 126.0, 121.5, 115.5, 115.4, 112.7, 103.1, 96.0, 90.6,

75.8, 69.8, 62.7, 60.5, 31.5, 28.8, 28.2, 26.6, 25.9, 24.8, 22.6, 20.7, 19.1, 17.7, 14.4, 14.1; **IR** (ν_{max}, cm^{-1}) 2959 (m), 2932 (m), 2878 (w), 2229 (w), 1861 (w), 1716 (s), 1604 (m), 1509 (m), 1464 (w), 1450 (m), 1432 (m), 1373 (m), 1328 (m), 1295 (m), 1259 (s), 1183 (s), 1095 (s), 1041 (m), 1012 (s), 820 (w), 759 (m); **HRMS** (ESI/QTOF) m/z: $[M + H]^+$ Calcd for $C_{32}H_{39}N_2O_5S^+$ 563.2574; Found 563.2562.

5.4.14. Synthesis and characterization of 3-(2-(3-phenylpropyl)-3-(trifluoromethyl)cycloprop-1-en-1-yl)prop-2-yn-1-yl 2-(1-(4-chlorobenzoyl)-5-methoxy-2-methyl-1*H*-indol-3-yl)acetate (3cc)

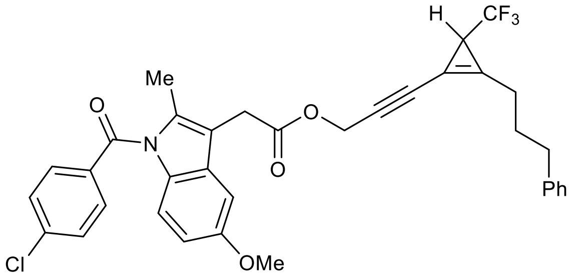

3cc

Following **GPD**, a mixture of $(Me_2S)AuCl$ (1.47 mg, 5.00 μmol, 5.00 mol%), **L1** (2.10 mg, 10.0 μmol, 10.0 mol%), CpBX **1j** (71.3 mg, 0.120 mmol, 1.20 equiv.), alkyne derivative **2bk** (39.6 mg, 0.100 mmol, 1.00 equiv.) and CH_3CN (2.0 mL) was stirred at 40 °C for 16 hours. Flash column chromatography on silica gel (eluent: pentane/ethyl acetate = 8:1) afforded **3cc** in 88% yield (54.7 mg, 88.2 μmol) as a colourless oil and **4** in 87% NMR yield based on **1j**. **TLC**: R_f (*n*-hexane/EtOAc = 4:1) = 0.44; **^{1}H NMR** (400 MHz, $CDCl_3$) δ 7.72 – 7.62 (m, 2H, Ar*H*), 7.54 – 7.41 (m, 2H, Ar*H*), 7.35 – 7.27 (m, 2H, Ar*H*), 7.25 – 7.12 (m, 3H, Ar*H*), 6.98 (d, *J* = 2.5 Hz, 1H, Ar*H*), 6.88 (d, *J* = 9.0 Hz, 1H, Ar*H*), 6.68 (dd, *J* = 9.0, 2.5 Hz, 1H, Ar*H*), 4.94 (s, 2H, OCH_2), 3.83 (s, 3H, OCH_3), 3.74 (s, 2H, CH_2), 2.71 – 2.68 (m, 2H, CH_2), 2.56 (t, *J* = 7.2 Hz, 2H, CH_2), 2.40 (s, 3H, CH_3), 2.29 (q, *J* = 4.4 Hz, 1H, C*H*(CF_3)), 2.07 – 1.88 (m, 2H, CH_2); **^{13}C NMR** (101 MHz, $CDCl_3$) δ 170.1, 168.4, 156.2, 141.2, 139.4, 136.3, 133.9, 131.3, 130.9, 130.6, 129.2, 128.6 (2C), 126.2, 125.4 (q, *J* = 275.5 Hz), 118.5 (q, *J* = 3.1 Hz), 115.1, 112.0, 112.0, 101.2, 94.1, 90.6 (q, *J* = 4.0 Hz), 73.8, 55.8, 53.2, 35.2, 30.2, 28.2, 25.4, 24.0 (q, *J* = 39.3 Hz), 13.5; **^{19}F NMR** (376 MHz, $CDCl_3$) δ -66.8; **IR** (ν_{max}, cm^{-1}) 3063 (w), 3029 (w), 2939 (w), 2860 (w), 2835 (w), 2232 (w), 1744 (m), 1683 (m), 1595 (w), 1478 (m), 1456 (m), 1400 (w), 1357 (m), 1321 (m), 1267 (m), 1223 (m), 1131 (s), 1087 (m), 1065 (m), 1037 (m), 1017 (m), 986 (w), 925 (w), 913 (w), 832 (m), 805 (w), 751 (m), 741 (m); **HRMS** (ESI/QTOF) m/z: $[M + Na]^+$ Calcd for $C_{35}H_{29}ClF_3NNaO_4^+$ 642.1629; Found 642.1644.

5.4.15. Synthesis and characterization of dimethyl 2-(((8*R*,9*S*,13*S*,14*S*,17*S*)-17-hydroxy-3-methoxy-13-methyl-7,8,9,11,12,13,14,15,16,17-decahydro-6*H*-cyclopenta[*a*]phenanthren-17-yl)ethynyl)-3-phenylcycloprop-2-ene-1,1-dicarboxylate (3cd)

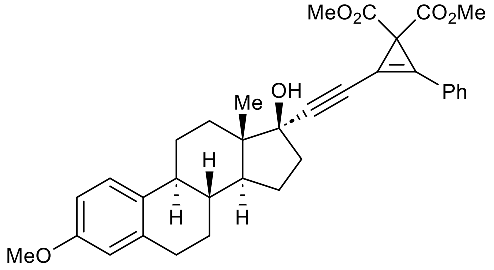

3cd

Following **GPD**, a mixture of $(Me_2S)AuCl$ (2.95 mg, 10.0 μmol, 5.00 mol%), **L1** (4.20 mg, 20.0 μmol, 10.0 mol%), CpBX **1k** (120 mg, 0.200 mmol, 1.00 equiv.), alkyne derivative **2bl** (62.1 mg, 0.200 mmol, 1.00 equiv.) and CH_3CN (2.0 mL) was stirred at room temperature for 24 hours. Flash column

chromatography on silica gel (eluent: pentane/ethyl acetate = 4:1) afforded **3cd** in 95% yield (103 mg, 190 μmol) as a colourless solid and **4** in 95% NMR yield. **M.p.** 135 – 138 °C. **ORD**: $[\alpha]_D^{20}$ = -34.1 (c = 0.32, $CHCl_3$). **TLC**: R_f (*n*-hexane/EtOAc = 4:1) = 0.14; **^{1}H NMR** (400 MHz, $CDCl_3$) δ 7.70 – 7.58 (m, 2H, Ar*H*), 7.48 – 7.41 (m, 3H, Ar*H*), 7.25 – 7.20 (m, 1H, Ar*H*), 6.73 (dd, *J* = 8.6, 2.8 Hz, 1H, Ar*H*), 6.65 (d, *J* = 2.7 Hz, 1H, Ar*H*), 3.78 (s, 3H, OCH_3), 3.75 (s, 6H, CO_2CH_3), 3.01 – 2.74 (m, 2H, CH_2), 2.49 – 2.37 (m, 3H, O*H* & CH_2), 2.29 – 2.22 (m, 1H, C*H*), 2.15 – 2.08 (m, 1H, C*H*), 1.94 – 1.70 (m, 5H, C*H* & CH_2), 1.56 – 1.36 (m, 4H, CH_2), 0.95 (s, 3H, CH_3); **^{13}C NMR** (101 MHz, $CDCl_3$) δ 169.7, 157.6, 138.1, 132.5, 130.9, 130.5, 129.1, 126.5, 124.3, 113.9, 111.6, 109.8, 106.9, 91.4, 81.0, 72.3, 55.3, 52.7, 50.1, 48.2, 43.7, 39.6, 39.0, 36.7, 33.3, 29.9, 27.4, 26.5, 23.1, 13.0; **IR** (ν_{max}, cm^{-1}) 3464 (w), 2935 (m), 2870 (m), 2210 (w), 1730 (m), 1609 (m), 1576 (w), 1499 (m), 1434 (m), 1281 (s), 1248 (s), 1187 (w), 1066 (s), 841 (w), 762 (m), 735 (s); **HRMS** (ESI/QTOF) m/z: $[M + Na]^+$ Calcd for $C_{34}H_{36}NaO_6^+$ 563.2404; Found 563.2408.

5.4.16. Synthesis and characterization of dimethyl 2-(((8*R*,9*S*,10*R*,13*S*,14*S*,17*S*)-17-hydroxy-13-methyl-3-oxo-2,3,6,7,8,9,10,11,12,13,14,15,16,17-tetradecahydro-1*H*-cyclopenta[*a*]phenanthren-17-yl)ethynyl)-3-phenylcycloprop-2-ene-1,1-dicarboxylate (3ce)

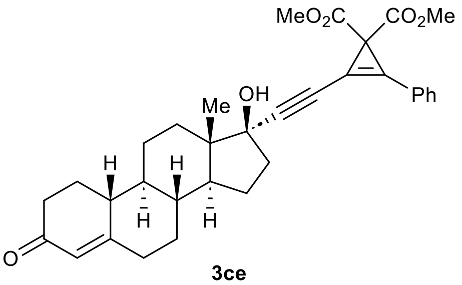

3ce

Following **GPD**, a mixture of $(Me_2S)AuCl$ (2.95 mg, 10.0 μmol, 5.00 mol%), **L1** (4.20 mg, 20.0 μmol, 10.0 mol%), CpBX **1k** (120 mg, 0.200 mmol, 1.00 equiv.), alkyne derivative **2bm** (59.7 mg, 0.200 mmol, 1.00 equiv.) and CH_3CN (2.0 mL) was stirred at room temperature for 24 hours. Flash column chromatography on silica gel (eluent: pentane/ethyl acetate = 4:1) afforded **3ce** in 92% yield (97.6 mg, 185 μmol) as a colourless oil and **4** in 99% NMR yield. **ORD**: $[\alpha]_D^{20}$ = -54.6 (c = 0.30, $CHCl_3$). **TLC**: R_f (*n*-hexane/EtOAc = 2:1) = 0.12; **^{1}H NMR** (400 MHz, $CDCl_3$) δ 7.66 – 7.52 (m, 2H, Ar*H*), 7.49 – 7.32 (m, 3H, Ar*H*), 5.82 (s, 1H, C*H*=C), 3.71 (s, 6H, CO_2CH_3), 2.90 (s, 1H, O*H*), 2.52 – 2.32 (m, 3H, C*H* & CH_2), 2.32 – 2.14 (m, 3H, CH_2), 2.14 – 1.98 (m, 2H, CH_2), 1.98 – 1.60 (m, 5H, C*H* & CH_2), 1.43 – 1.47 (m, 2H, CH_2), 1.43 – 1.20 (m, 3H, CH_2), 1.16 – 1.00 (m, 1H, C*H*), 0.94 (s, 3H, CH_3), 0.93 –0.83 (m, 1H, C*H*); **^{13}C NMR** (101 MHz, $CDCl_3$) δ 200.1, 169.6, 166.7, 130.9, 130.4, 129.0, 124.6, 124.2, 109.7, 106.8, 91.2, 80.6, 72.2, 52.6, 49.7, 49.0, 47.8, 42.6, 41.1, 38.8, 36.6, 36.5, 35.5, 32.8, 30.6, 26.6, 26.3, 23.1, 12.9; **IR** (ν_{max}, cm^{-1}) 3414 (w), 2951 (m), 2869 (m), 2210 (w), 1730 (s), 1658 (s), 1619 (m), 1448 (m), 1434 (m), 1246 (s), 1132 (w), 1062 (s), 969 (w), 885 (w), 763 (s), 733 (s); **HRMS** (ESI/QTOF) m/z: $[M + Na]^+$ Calcd for $C_{33}H_{36}NaO_6^+$ 551.2404; Found 551.2414.

5.4.17. Synthesis and characterization of dimethyl 2-(4-fluorophenyl)-3-(3-(4-((2S,3R)-1-(4-fluorophenyl)-3-((S)-3-(4-fluorophenyl)-3-hydroxypropyl)-4-oxoazetidin-2-yl)phenoxy)prop-1-yn-1-yl)cycloprop-2-ene-1,1-dicarboxylate (3cf)

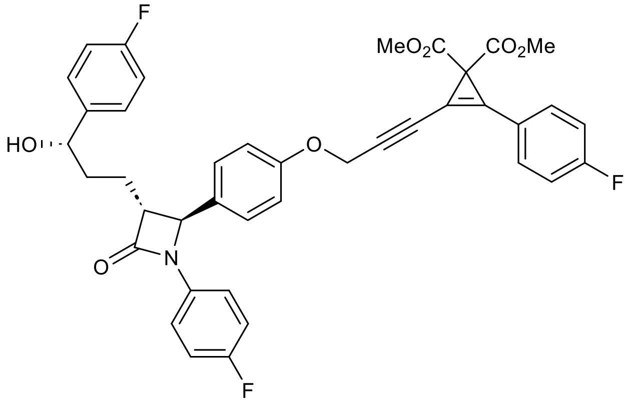

3cf

Following **GPD**, a mixture of $(Me_2S)AuCl$ (1.47 mg, 5.00 μmol, 5.00 mol%), **L1** (2.10 mg, 10.0 μmol, 10.0 mol%), CpBX **1l** (74.2 mg, 0.120 mmol, 1.20 equiv.), alkyne derivative **2bn** (44.8 mg, 0.100 mmol, 1.00 equiv.) and CH_3CN (2.0 mL) was stirred at 40 °C for 12 hours. Flash column chromatography on silica gel (eluent: pentane/ethyl acetate = 4:1) afforded **3cf** in 77% yield (53.5 mg, 76.9 μmol) as a colourless oil and **4** in 96% NMR yield based on **1l**. **ORD**: $[\alpha]_D^{20}$ = -22.4 (c = 0.30, $CHCl_3$). **TLC**: R_f (*n*-hexane/EtOAc = 2:1) = 0.11; **^{1}H NMR** (400 MHz, $CDCl_3$) δ 7.66 – 7.55 (m, 2H, Ar*H*), 7.31 – 7.25 (m, 4H, Ar*H*), 7.25 – 7.19 (m, 2H, Ar*H*), 7.17 – 7.06 (m, 2H, Ar*H*), 7.02 – 6.96 (m, 4H, Ar*H*), 6.96 – 6.86 (m, 2H, Ar*H*), 4.96 (s, 2H, OCH_2), 4.70 (t, *J* = 6.0 Hz, 1H, C*H*), 4.59 (d, *J* = 2.3 Hz, 1H, C*H*), 3.721 (s, 3H, CO_2CH_3), 3.718 (s, 3H, CO_2CH_3), 3.10 – 3.05 (m, 1H, C*H*), 2.50 (s, 1H, O*H*), 2.09 – 1.80 (m, 4H, CH_2); **^{13}C NMR** (101 MHz, $CDCl_3$) δ 169.3, 167.7, 164.3 (d, *J* = 254.2 Hz), 162.3 (d, *J* = 245.7 Hz), 159.1 (d, *J* = 243.3 Hz), 157.9, 140.2 (d, *J* = 3.1 Hz), 133.9 (d, *J* = 2.7 Hz), 132.9 (d, *J* = 9.0 Hz), 130.8, 127.5 (d, *J* = 8.1 Hz), 127.3, 120.5 (d, *J* = 3.3 Hz), 118.5 (d, *J* = 7.8 Hz), 116.6 (d, *J* = 22.3 Hz), 116.0 (d, *J* = 22.7 Hz), 115.8, 115.4 (d, *J* = 21.3 Hz), 110.7, 97.4, 90.3 (d, *J* = 2.9 Hz), 73.4, 73.2, 61.1, 60.5, 57.0, 52.8, 36.7 (2C), 25.1; **^{19}F NMR** (376 MHz, $CDCl_3$) δ -106.3 (Ar*F*), -114.9 (Ar*F*), -117.9 (Ar*F*); **IR** (ν_{max}, cm^{-1}) 3465 (w), 2953 (w), 2926 (w), 2856 (w), 2197 (w), 1731 (s), 1602 (m), 1508 (s), 1435 (w), 1389 (m), 1285 (m), 1220 (s), 1157 (m), 1102 (w), 1064 (m), 1016 (m), 836 (s), 737 (w); **HRMS** (ESI/QTOF) m/z: $[M + Na]^+$ Calcd for $C_{40}H_{32}F_3NNaO_7^+$ 718.2023; Found 718.2029.

5.4.18. Synthesis and characterization of dimethyl 2-(3-((4-oxo-4-(((3*R*,5a*S*,6*R*,8a*S*,9*R*,10*S*,12*R*,12a*R*)-3,6,9-trimethyldecahydro-12*H*-3,12-epoxy[1,2]dioxepino[4,3-*i*]isochromen-10-yl)oxy)butanoyl)oxy)prop-1-yn-1-yl)-3-phenylcycloprop-2-ene-1,1-dicarboxylate (3cg)

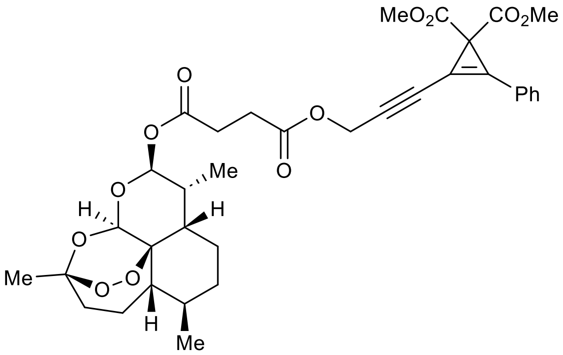

3cg

Following **GPD**, a mixture of $(Me_2S)AuCl$ (1.47 mg, 5.00 µmol, 5.00 mol%), **L1** (2.10 mg, 10.0 µmol, 10.0 mol%), CpBX **1k** (72.0 mg, 0.120 mmol, 1.20 equiv.), alkyne derivative **2bo** (42.3 mg, 0.100 mmol, 1.00 equiv.) and CH_3CN (2.0 mL) was stirred at 30 °C for 28 hours. Flash column chromatography on silica gel (eluent: pentane/ethyl acetate = 4:1) afforded **3cg** in 82% yield (53.8 mg, 82.4 µmol) as a colourless oil and **4** in 99% NMR yield based on **1k**. **ORD**: $[\alpha]_D^{20}$ = +8.2 (c = 0.51, $CHCl_3$). **TLC**: R_f (*n*-hexane/EtOAc = 3:1) = 0.11; ^{1}**H NMR** (400 MHz, $CDCl_3$) δ 7.71 – 7.58 (m, 2H, Ar*H*), 7.52 – 7.37 (m, 3H, Ar*H*), 5.79 (d, *J* = 9.8 Hz, 1H, C*H*), 5.42 (s, 1H, C*H*), 4.98 (d, *J* = 2.7 Hz, 2H, OC*H*$_2$), 3.74 (s, 6H, CO_2CH_3), 2.85 – 2.63 (m, 4H, C*H*$_2$), 2.60 – 2.51 (m, 1H, C*H*), 2.40 – 2.32 (m, 1H, C*H*), 2.05 – 1.99 (m, 1H, C*H*), 1.96 – 1.82 (m, 1H, C*H*), 1.78 – 1.65 (m, 2H, C*H*$_2$), 1.60 (dt, *J* = 13.7, 4.4 Hz, 1H, C*H*), 1.51 – 1.38 (m, 4H, C*H*$_2$ & C*H*$_3$), 1.36 – 1.22 (m, 3H, C*H*$_2$), 1.08 – 0.90 (m, 4H, C*H* & C*H*$_3$), 0.85 (d, *J* = 7.1 Hz, 3H, C*H*$_3$); ^{13}**C NMR** (101 MHz, $CDCl_3$) δ 171.4, 171.0, 169.3, 131.2, 130.7, 129.1, 124.1, 111.7, 104.6, 96.5, 92.4, 91.6, 90.7, 80.2, 72.9, 53.0, 52.7, 51.6, 45.3, 37.3, 36.6, 36.3, 34.2, 31.9, 29.2, 28.8, 26.1, 24.7, 22.1, 20.3, 12.2; **IR** (ν_{max}, cm^{-1}) 3061 (w), 2952 (m), 2928 (m), 2878 (w), 1736 (s), 1436 (m), 1377 (w), 1348 (w), 1281 (m), 1248 (s), 1201 (m), 1148 (s), 1101 (m), 1036 (s), 1014 (s), 974 (m), 945 (w), 925 (w), 877 (m), 845 (w), 827 (w), 765 (m), 735 (m); **HRMS** (ESI/QTOF) m/z: [M + Na]$^+$ Calcd for $C_{35}H_{40}NaO_{12}^+$ 675.2412; Found 675.2393.

5.4.19. Synthesis and characterization of dimethyl 2-(3-((((1*S*,2*S*,4a*R*,4b*R*,7*S*,9a*S*,10*S*,10a*R*)-2,7-diacetoxy-1-methyl-8-methylene-13-oxo-1,2,4b,5,6,7,8,9,10,10a-decahydro-4a,1-(epoxymethano)-7,9a-methanobenzo[a]azulene-10-carbonyl)oxy)prop-1-yn-1-yl)-3-(4-fluorophenyl)cycloprop-2-ene-1,1-dicarboxylate (3ch)

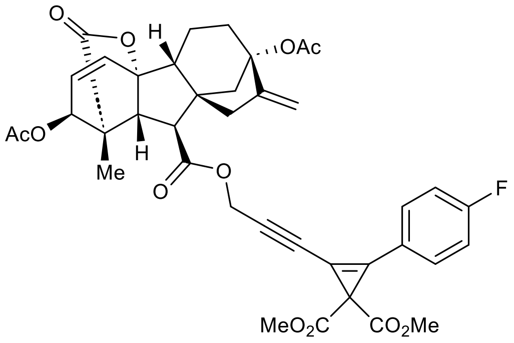

3ch

Following **GPD**, a mixture of $(Me_2S)AuCl$ (1.47 mg, 5.00 µmol, 5.00 mol%), **L1** (2.10 mg, 10.0 µmol, 10.0 mol%), CpBX **1k** (72.0 mg, 0.120 mmol, 1.20 equiv.), alkyne derivative **2bo** (42.3 mg, 0.100 mmol, 1.00 equiv.) and CH_3CN (4.0 mL) was stirred at 40 °C for 4 hours. Flash column chromatography on silica gel (eluent: pentane/ethyl acetate = 2:1) afforded **3ch** in 95% yield (67.9 mg, 94.7 µmol) as a colourless solid and **4** in 95% NMR yield based on **1k**. **M.p.** 172 – 174 °C. **ORD**: $[\alpha]_D^{20}$ = +128.7 (c = 0.23, $CHCl_3$). **TLC**: R_f (*n*-hexane/EtOAc = 2:1) = 0.19; ^{1}**H NMR** (400 MHz, $CDCl_3$) δ 7.73 – 7.53 (m, 2H, Ar*H*), 7.20 – 7.03 (m, 2H, Ar*H*), 6.36 (d, *J* = 9.3 Hz, 1H, CHCH=C*H*), 5.92 – 5.81 (m, 1H, CHC*H*=CH), 5.32 (d, *J* = 3.7 Hz, 1H, C*H*CH=CH), 5.22 – 5.12 (m, 1H, C=C*H*$_2$), 5.11 – 4.92 (m, 3H, C=C*H*$_2$ & OC*H*$_2$), 3.73 (s, 6H, CO_2CH_3), 3.34 (d, *J* = 10.9 Hz, 1H, C*H*), 2.83 (d, *J* = 11.0 Hz, 1H, C*H*), 2.46 – 2.25 (m, 4H, C*H*$_2$), 2.21 – 2.16 (m, 1H, C*H*), 2.09 (s, 3H, C*H*$_3$), 2.06 – 1.88 (m, 5H, C*H*$_2$ & C*H*$_3$), 1.84 – 1.64 (m, 2H, C*H*$_2$), 1.15 (s, 3H, C*H*$_3$); ^{13}**C NMR** (101 MHz, $CDCl_3$) δ 176.9, 171.0, 170.1, 169.9, 169.1, 164.3 (d, *J* = 254.1 Hz), 153.3, 134.2, 132.9 (d, *J* = 8.9 Hz), 129.3, 120.5 (d, *J* = 3.3 Hz), 116.6 (d, *J* = 22.4 Hz), 111.1, 108.6, 96.0, 90.0 (d, *J* = 2.8 Hz), 89.9, 84.1, 73.1, 70.2, 53.6, 53.2, 52.8, 52.2, 51.3, 51.1, 50.2, 42.6, 40.0, 36.7, 36.3, 22.1, 20.9, 16.9, 14.5; ^{19}**F NMR** (376 MHz, $CDCl_3$) δ -106.4; **IR** (ν_{max}, cm^{-1}) 2989 (w), 2955 (w), 2882 (w), 2849 (w), 1781 (m), 1734 (s), 1599 (w), 1506 (w), 1436 (w), 1372 (m), 1281 (m), 1231 (s), 1155 (m), 1094 (w), 1058 (m), 1026 (m), 975 (m), 897 (w), 842 (m), 736 (m); **HRMS** (ESI/QTOF) m/z: [M + Na]$^+$ Calcd for $C_{39}H_{37}FNaO_{12}^+$ 739.2161; Found 739.2163.

5.4.20. Unsuccessful substrates

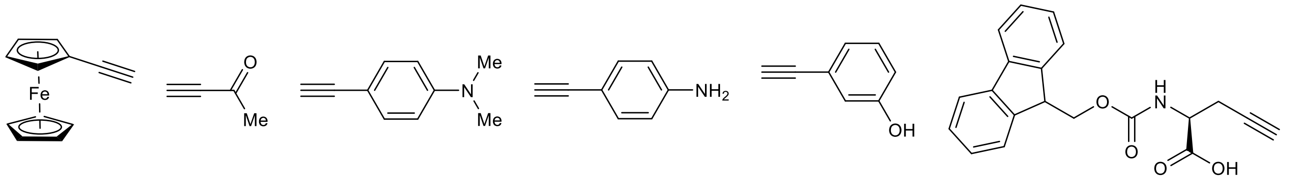

6. Substrate scope of σ-type cyclopropenium cation transfer to vinylboronic acid

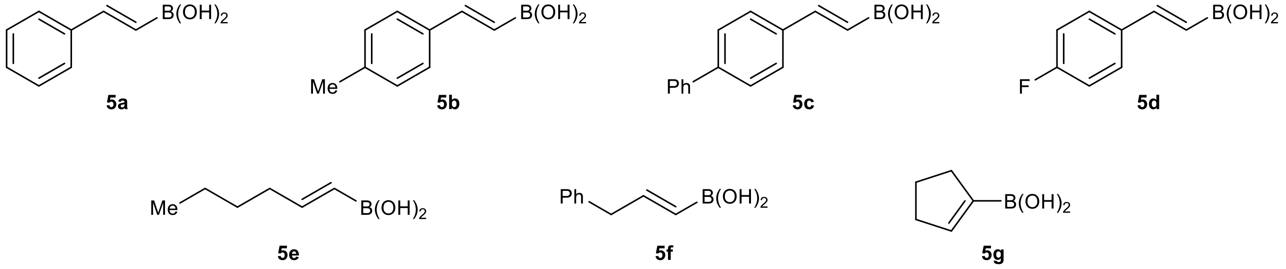

Vinylboronic acid **5a**, **5b**, **5c**, **5d**, **5e**, **5f**, **5g** were commercially available and used as received.

6.1. Optimization of σ-type cyclopropenium cation transfer to vinylboronic acid

An oven-dried 10 mL Schlenk tube was sequentially charged with a magnetic stir-bar, **L1** (2.10 mg, 10.0 µmol, 10.0 mol%), $(Me_2S)AuCl$ (1.47 mg, 5.00 µmol, 5.00 mol%), vinylboronic acid **5b** (16.2 mg, 0.100 mmol, 1.00 equiv.) and CpBX **1a** (1.00 – 2.00 equiv.); if the additive was used, it was added last. The Schlenk tube was then evacuated and backfilled with nitrogen three times. Subsequently, CH_3CN (0.050 M; 2.0 mL) was added by syringe. The reaction mixture was stirred at 40 °C for the specified time. The resulting reaction mixture was diluted with CH_2Cl_2 (5.0 mL) and filtered through a short pad of silica gel by eluting with CH_2Cl_2 (3 × 5.0 mL). The filtrate was then concentrated to dryness and the residue was subjected to flash column chromatography on silica gel (eluent: pentane/EtOAc = 20:1). The fractions that contained the product **6k**, **4** and the remaining CpBX **1a** were collected separately and concentrated by rotary evaporation. The yields of **6k**, **4** and the recovery of **1a** were obtained by quantitative ^1H NMR analysis using CH_2Br_2 (^1H NMR δ 4.92) as the internal standard.

Table 6 | Optimization of σ-type cyclopropenium cation transfer to vinylboronic acid

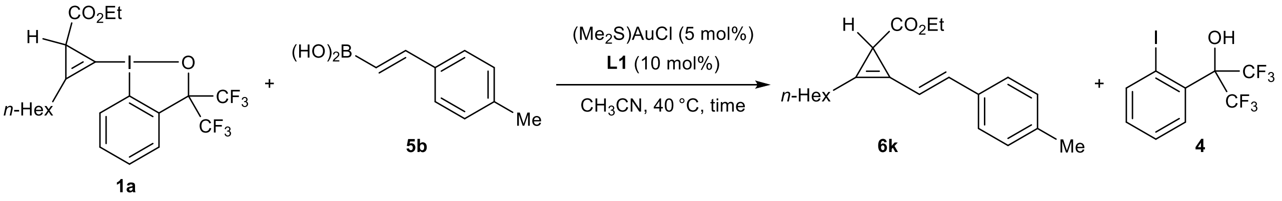

Entry	**1a** (equiv.)	**5b** (equiv.)	Additive (equiv.)	Time (h)	Yield of **6k** (%)[a]	Yield of **4** (%)[b]	Recovery of **1a** (%)[b]
1	1.0	1.0	none	10	71	99	0
2	1.0	1.0	CsF (1.0)	3	trace	60	0
3	1.3	1.0	none	5	85	99	0
4	1.5	1.0	none	5	64	89	10
5	2.0	1.0	none	12	59	96	0

Reactions performed on a 100 µmol scale. Yields and recovery were determined by ^1H NMR spectroscopy using dibromomethane as the internal standard. [a]Yield was determined based on **5b**. [b]Yield and recovery were determined based on **1a**.

6.2. Survey of gold-catalysed σ-type cyclopropenium cation transfer to vinylboronic acid

General procedure E (**GPE**) for gold-catalysed σ-type cyclopropenium cation transfer to vinylboronic acid:

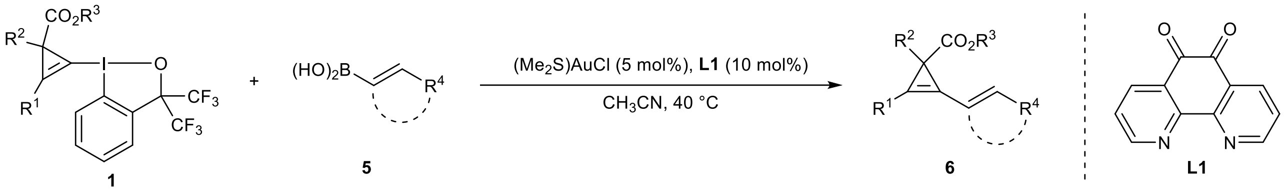

GPE: An oven-dried 10 mL Schlenk tube was sequentially charged with a magnetic stir-bar, **L1** (2.10 mg, 10.0 µmol, 10.0 mol%), $(Me_2S)AuCl$ (1.47 mg, 5.00 µmol, 5.00 mol%), vinylboronic acid **5** (0.100 mmol, 1.00 equiv.) and CpBX **1** (0.130 mmol, 1.30 equiv.). The Schlenk tube was then evacuated and backfilled with nitrogen three times. Subsequently, CH_3CN (0.10 M; 2.0 mL) was added by syringe. The reaction mixture was stirred at 40 °C for the specified time. The reaction mixture was then filtered through a silica gel pad and washed with CH_2Cl_2 (3 × 5.0 mL). The solvent was removed under reduced pressure, and the resulting crude residue was subjected to a short column chromatography (silica). The fractions that contained the products were collected and analysed by ^1H NMR spectroscopy. The recovered sample was purified by flash column chromatography (C18 reverse phase) to give the cross-coupled product **6**.

6.2.1. Synthesis and characterization of ethyl (*E*)-2-dodecyl-3-styrylcycloprop-2-ene-1-carboxylate (6a)

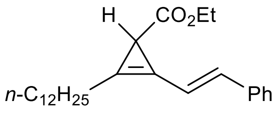

6a

Following **GPE**, a mixture of $(Me_2S)AuCl$ (1.47 mg, 5.00 µmol, 5.00 mol%), **L1** (2.10 mg, 10.0 µmol, 10.0 mol%), CpBX **1b** (84.3 mg, 0.130 mmol, 1.30 equiv.), vinylboronic acid **5a** (14.8 mg, 0.100 mmol, 1.00 equiv.) and CH_3CN (2.0 mL) was stirred at 40 °C for 16 hours. Analysis of the crude product gave 57% NMR yield of **6a** and the by-product **4** was observed in 96% NMR yield based on **1b**. Flash column chromatography (C18 reverse phase; eluent: CH_3CN/H_2O = 1:9 to 9:1) afforded **6a** in 55% yield (21.1 mg, 55.2 µmol) as a colourless oil. **TLC**: R_f (*n*-hexane/EtOAc = 20:1) = 0.28; **^{1}H NMR** (400 MHz, $CDCl_3$) δ 7.49 – 7.42 (m, 2H, Ar*H*), 7.39 – 7.30 (m, 2H, Ar*H*), 7.30 – 7.23 (m, 1H, Ar*H*), 6.90 (d, *J* = 15.6 Hz, 1H, C*H*=CH), 6.71 (d, *J* = 15.6 Hz, 1H, CH=C*H*), 4.22 – 4.10 (m, 2H, $CO_2CH_2CH_3$), 2.58 (t, *J* = 7.3 Hz, 2H, CH_2CH_2C), 2.33 (s, 1H, C*H*CO_2), 1.71 – 1.56 (m, 2H, CH_2CH_2C), 1.49 – 1.14 (m, 21H, CH_2 & $CO_2CH_2CH_3$), 0.97 – 0.77 (m, 3H, CH_3); **^{13}C NMR** (101 MHz, $CDCl_3$) δ 175.9, 138.1, 136.5, 128.8, 128.6, 127.1, 113.0, 111.7, 103.5, 60.2, 32.1, 29.82, 29.79 (2C), 29.7, 29.51, 29.48, 29.4, 27.2, 25.7, 22.8, 22.0, 14.6, 14.3; **IR** (ν_{max}, cm^{-1}) 2954 (m), 2925 (s), 2854 (s), 1710 (s), 1630 (m), 1593 (m), 1465 (m), 1450 (m), 1404 (w), 1376 (m), 1304 (w), 1245 (m), 1203 (m), 1178 (m), 1150 (m), 1094 (w), 1073 (m), 1030 (m), 971 (w), 863 (w), 753 (m); **HRMS** (Sicrit plasma/LTQ-Orbitrap) m/z: $[M + H]^+$ Calcd for $C_{26}H_{39}O_2^+$ 383.2945; Found 383.2945.

6.2.2. Synthesis and characterization of ethyl (*E*)-2-dodecyl-3-(4-methylstyryl)cycloprop-2-ene-1-carboxylate (6b)

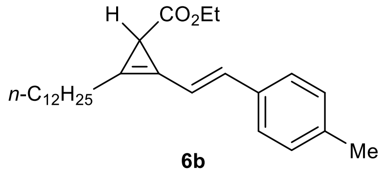

6b

Following **GPE**, a mixture of $(Me_2S)AuCl$ (1.47 mg, 5.00 µmol, 5.00 mol%), **L1** (2.10 mg, 10.0 µmol, 10.0 mol%), CpBX **1b** (84.3 mg, 0.130 mmol, 1.30 equiv.), vinylboronic acid **5b** (16.2 mg, 0.100 mmol, 1.00 equiv.) and CH_3CN (2.0 mL) was stirred at 40 °C for 17 hours. Analysis of the crude product gave 88% NMR yield of **6b** and the by-product **4** was observed in 98% NMR yield based on **1b**. Flash column chromatography (C18 reverse phase; eluent: CH_3CN/H_2O = 1:9 to 9:1) afforded **6b** in 86% yield (34.2 mg, 86.2 µmol) as a colourless oil. **TLC**: R_f (*n*-hexane/EtOAc = 20:1) = 0.30; **^{1}H NMR** (400 MHz, $CDCl_3$) δ 7.37 – 7.31 (m, 2H, Ar*H*), 7.17 – 7.12 (m, 2H, Ar*H*), 6.84 (d, *J* = 15.6 Hz, 1H, C*H*=CH), 6.68 (d, *J* = 15.6 Hz, 1H, CH=C*H*), 4.21 – 4.10 (m, 2H, $CO_2CH_2CH_3$), 2.57 (t, *J* = 7.3 Hz, 2H, CH_2CH_2C), 2.35 (s, 3H, CH_3), 2.31 (s, 1H, $CHCO_2$), 1.74 – 1.52 (m, 2H, CH_2CH_2C), 1.45 – 1.13 (m, 21H, CH_2 & $CO_2CH_2CH_3$), 0.88 (t, *J* = 6.8 Hz, 3H, CH_3); **^{13}C NMR** (101 MHz, $CDCl_3$) δ 175.9, 138.6, 138.1, 133.7, 129.5, 127.0, 112.0, 111.0, 103.6, 60.2, 32.1, 29.82, 29.79 (2C), 29.7, 29.51, 29.48, 29.4, 27.2, 25.7, 22.8, 22.0, 21.4, 14.6, 14.3; **IR** (ν_{max}, cm^{-1}) 3026 (w), 2950 (m), 2854 (s), 1867 (w), 1721 (s), 1604 (w), 1512 (w), 1463 (m), 1369 (w), 1331 (w), 1302 (w), 1243 (m), 1173 (s), 1095 (w), 1036 (m), 997 (w), 959 (m), 943 (w), 854 (w), 803 (m), 723 (w); **HRMS** (ESI/QTOF) m/z: $[M + H]^+$ Calcd for $C_{27}H_{41}O_2^+$ 397.3101; Found 397.3096.

6.2.3. Synthesis and characterization of ethyl (*E*)-2-(2-([1,1'-biphenyl]-4-yl)vinyl)-3-dodecylcycloprop-2-ene-1-carboxylate (6c)

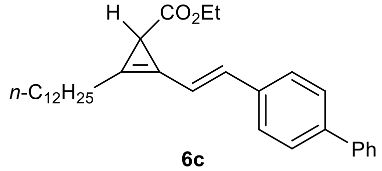

6c

Following **GPE**, a mixture of $(Me_2S)AuCl$ (1.47 mg, 5.00 µmol, 5.00 mol%), **L1** (2.10 mg, 10.0 µmol, 10.0 mol%), CpBX **1b** (84.3 mg, 0.130 mmol, 1.30 equiv.), vinylboronic acid **5c** (22.4 mg, 0.100 mmol, 1.00 equiv.) and CH_3CN (2.0 mL) was stirred at 40 °C for 16 hours. Analysis of the crude product gave 67% NMR yield of **6c** and the by-product **4** was observed in 98% NMR yield based on **1b**. Flash column chromatography (C18 reverse phase; eluent: CH_3CN/H_2O = 1:9 to 9:1) afforded **6c** in 66% yield (30.2 mg, 65.8 µmol) as a colourless oil. **TLC**: R_f (*n*-hexane/EtOAc = 20:1) = 0.24; **^{1}H NMR** (400 MHz, $CDCl_3$) δ 7.68 – 7.55 (m, 4H, Ar*H*), 7.52 (d, *J* = 8.4 Hz, 2H, Ar*H*), 7.49 – 7.41 (m, 2H, Ar*H*), 7.41 – 7.31 (m, 1H, Ar*H*), 6.94 (d, *J* = 15.6 Hz, 1H, C*H*=CH), 6.75 (d, *J* = 15.6 Hz, 1H, CH=C*H*), 4.23 – 4.11 (m, 2H, $CO_2CH_2CH_3$), 2.60 (t, *J* = 7.3 Hz, 2H, CH_2CH_2C), 2.35 (s, 1H, $CHCO_2$), 1.81 – 1.60 (m, 2H, CH_2CH_2C), 1.50 – 1.13 (m, 21H, CH_2 & $CO_2CH_2CH_3$), 1.08 – 0.77 (m, 3H, CH_3); **^{13}C NMR** (101 MHz, $CDCl_3$) δ 175.9, 141.3, 140.6, 137.6, 135.5, 129.0, 127.6, 127.53, 127.48, 127.1, 113.0, 111.8, 103.6, 60.3, 32.1, 29.83, 29.80 (2C), 29.7, 29.51, 29.48, 29.4, 27.2, 25.8, 22.8, 22.0, 14.6, 14.3; **IR** (ν_{max}, cm^{-1}) 3418 (w), 3031 (w), 2924 (s), 2853 (s), 1709 (s), 1602 (m), 1486 (w), 1464 (m), 1407 (w), 1375 (w), 1245 (m), 1176 (m), 1077 (w), 1033 (m), 1007 (w), 964 (w), 835 (m), 763 (s), 727 (m); **HRMS** (Sicrit plasma/LTQ-Orbitrap) m/z: $[M + H]^+$ Calcd for $C_{32}H_{43}O_2^+$ 459.3258; Found 459.3258.

6.2.4. Synthesis and characterization of ethyl (*E*)-2-dodecyl-3-(4-fluorostyryl)cycloprop-2-ene-1-carboxylate (6d)

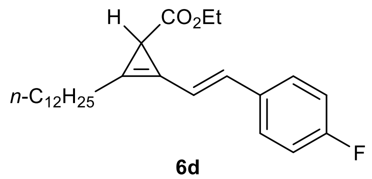

6d

Following **GPE**, a mixture of $(Me_2S)AuCl$ (1.47 mg, 5.00 µmol, 5.00 mol%), **L1** (2.10 mg, 10.0 µmol, 10.0 mol%), CpBX **1b** (84.3 mg, 0.130 mmol, 1.30 equiv.), vinylboronic acid **5d** (16.6 mg, 0.100 mmol, 1.00 equiv.) and CH_3CN (2.0 mL) was stirred at 40 °C for 16 hours. Analysis of the crude product gave 66% NMR yield of **6d** and the by-product **4** was observed in 98% NMR yield based on **1b**. Flash column chromatography (C18 reverse phase; eluent: CH_3CN/H_2O = 1:9 to 9:1) afforded **6d** in 65% yield (26.2 mg, 65.4 µmol) as a colourless oil. **TLC**: R_f (*n*-hexane/EtOAc = 20:1) = 0.27; **^{1}H NMR** (400 MHz, $CDCl_3$) δ 7.48 – 7.33 (m, 2H, Ar*H*), 7.09 – 6.95 (m, 2H, Ar*H*), 6.81 (d, *J* = 15.6 Hz, 1H, C*H*=CH), 6.66 (d, *J* = 15.6 Hz, 1H, CH=C*H*), 4.21 – 4.10 (m, 2H, $CO_2CH_2CH_3$), 2.58 (t, *J* = 7.3 Hz, 2H, CH_2CH_2C), 2.32 (s, 1H, C*H*CO_2), 1.70 – 1.58 (m, 2H, CH_2CH_2C), 1.46 – 1.18 (m, 21H, CH_2 & $CO_2CH_2CH_3$), 0.93 – 0.80 (m, 3H, CH_3); **^{13}C NMR** (101 MHz, $CDCl_3$) δ 175.8, 162.9 (d, *J* = 248.8 Hz), 136.8, 132.7 (d, *J* = 3.4 Hz), 128.7 (d, *J* = 8.1 Hz), 115.9 (d, *J* = 21.8 Hz), 112.9 (d, *J* = 2.5 Hz), 111.8, 103.3, 60.3, 32.1, 29.82, 29.79 (2C), 29.7, 29.51, 29.47, 29.4, 27.2, 25.7, 22.8, 21.9, 14.6, 14.3; **^{19}F NMR** (376 MHz, $CDCl_3$) δ -112.8; **IR** (ν_{max}, cm^{-1}) 2926 (s), 2855 (s), 1865 (w), 1720 (s), 1599 (m), 1509 (s), 1464 (m), 1369 (w), 1332 (w), 1235 (s), 1176 (s), 1161 (s), 1094 (w), 1036 (m), 1000 (w), 958 (m), 856 (w), 820 (m); **HRMS** (Sicrit plasma/LTQ-Orbitrap) m/z: $[M + H]^+$ Calcd for $C_{26}H_{38}FO_2^+$ 401.2850; Found 401.2850.

6.2.5. Synthesis and characterization of ethyl (*E*)-2-dodecyl-3-(hex-1-en-1-yl)cycloprop-2-ene-1-carboxylate (6e)

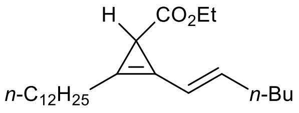

6e

Following **GPE**, a mixture of $(Me_2S)AuCl$ (1.47 mg, 5.00 µmol, 5.00 mol%), **L1** (2.10 mg, 10.0 µmol, 10.0 mol%), CpBX **1b** (84.3 mg, 0.130 mmol, 1.30 equiv.), vinylboronic acid **5e** (12.8 mg, 0.100 mmol, 1.00 equiv.) and CH_3CN (2.0 mL) was stirred at 40 °C for 16 hours. Analysis of the crude product gave 55% NMR yield of **6e** and the by-product **4** was observed in 88% NMR yield based on **1b**. Flash column chromatography (C18 reverse phase; eluent: CH_3CN/H_2O = 1:9 to 9:1) afforded **6e** in 53% yield (19.3 mg, 53.2 µmol) as a colourless oil. **TLC**: R_f (*n*-hexane/EtOAc = 20:1) = 0.34; **^{1}H NMR** (400 MHz, $CDCl_3$) δ 6.18 (dt, *J* = 15.2, 1.5 Hz, 1H, C*H*=CH), 5.92 (dt, *J* = 14.7, 7.0 Hz, 1H, CH=C*H*), 4.12 (q, *J* = 7.1 Hz, 2H, $CO_2CH_2CH_3$), 2.48 (t, *J* = 7.3 Hz, 2H, CH_2CH_2C), 2.27 – 2.12 (m, 3H, C*H*CO_2 & CH_2), 1.65 – 1.52 (m, 2H, CH_2CH_2C), 1.47 – 1.14 (m, 25H, CH_2 & $CO_2CH_2CH_3$), 0.91 – 0.86 (m, 6H, CH_3); **^{13}C NMR** (101 MHz, $CDCl_3$) δ 176.2, 142.1, 114.8, 108.0, 103.2, 60.1, 32.5, 32.1, 31.1, 29.81, 29.79 (2C), 29.7, 29.51, 29.47, 29.4, 27.2, 25.4, 22.8, 22.4, 21.8, 14.6, 14.3, 14.0; **IR** (ν_{max}, cm^{-1}) 2957 (m), 2926 (s), 2855 (m), 1724 (m), 1464 (w), 1369 (w), 1333 (w), 1244 (w), 1174 (m), 1094 (w), 1038 (w), 961 (w); **HRMS** (Sicrit plasma/LTQ-Orbitrap) m/z: $[M + H]^+$ Calcd for $C_{24}H_{43}O_2^+$ 363.3258; Found 363.3207.

6.2.6. Synthesis and characterization of ethyl (*E*)-2-dodecyl-3-(3-phenylprop-1-en-1-yl)cycloprop-2-ene-1-carboxylate (6f)

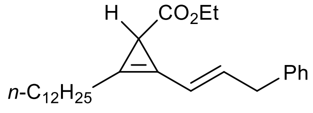

6f

Following **GPE**, a mixture of (Me_2S)AuCl (1.47 mg, 5.00 µmol, 5.00 mol%), **L1** (2.10 mg, 10.0 µmol, 10.0 mol%), CpBX **1b** (84.3 mg, 0.130 mmol, 1.30 equiv.), vinylboronic acid **5f** (16.2 mg, 0.100 mmol, 1.00 equiv.) and CH_3CN (2.0 mL) was stirred at 40 °C for 18 hours. Analysis of the crude product gave 37% NMR yield of **6f** and the by-product **4** was observed in 84% NMR yield based on **1b**. Flash column chromatography (C18 reverse phase; eluent: CH_3CN/H_2O = 1:9 to 9:1) afforded **6f** in 36% yield (14.4 mg, 36.3 µmol) as a colourless oil. **TLC**: R_f (*n*-hexane/EtOAc = 20:1) = 0.29; **^{1}H NMR** (400 MHz, $CDCl_3$) δ 7.34 – 7.27 (m, 2H, Ar*H*), 7.25 – 7.14 (m, 3H, Ar*H*), 6.22 (dt, *J* = 15.1, 1.5 Hz, 1H, C*H*=CH), 6.07 (dt, *J* = 15.1, 6.8 Hz, 1H, CH=C*H*), 4.12 (q, *J* = 7.1 Hz, 2H, $CO_2CH_2CH_3$), 3.53 (d, *J* = 6.7 Hz, 2H, CH_2Ph), 2.49 (t, *J* = 7.3 Hz, 2H, CH_2CH_2C), 2.19 (s, 1H, C*H*CO_2), 1.66 – 1.50 (m, 2H, CH_2CH_2C), 1.45 – 1.15 (m, 21H, CH_2 & $CO_2CH_2CH_3$), 1.00 – 0.78 (m, 3H, CH_3); **^{13}C NMR** (101 MHz, $CDCl_3$) δ 176.0, 139.8, 139.3, 128.9, 128.7, 126.5, 116.2, 109.3, 103.0, 60.1, 39.1, 32.1, 29.81, 29.78 (2C), 29.7, 29.50, 29.46, 29.4, 27.2, 25.4, 22.8, 21.8, 14.5, 14.3; **IR** (ν_{max}, cm^{-1}) 3028 (w), 2956 (m), 2925 (s), 2854 (s), 1875 (w), 1724 (s), 1457 (m), 1368 (w), 1333 (w), 1245 (m), 1173 (s), 1036 (w), 961 (m), 734 (w); **HRMS** (ESI/QTOF) m/z: $[M + H]^+$ Calcd for $C_{27}H_{41}O_2^+$ 397.3101; Found 397.3102.

6.2.7. Synthesis and characterization of ethyl 2-(cyclopent-1-en-1-yl)-3-dodecylcycloprop-2-ene-1-carboxylate (6g)

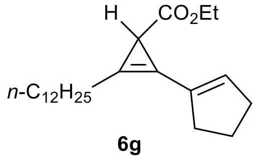

6g

Following **GPE**, a mixture of (Me_2S)AuCl (1.47 mg, 5.00 µmol, 5.00 mol%), **L1** (2.10 mg, 10.0 µmol, 10.0 mol%), CpBX **1b** (84.3 mg, 0.130 mmol, 1.30 equiv.), vinylboronic acid **5g** (11.2 mg, 0.100 mmol, 1.00 equiv.) and CH_3CN (2.0 mL) was stirred at 40 °C for 16 hours. Analysis of the crude product gave 59% NMR yield of **6g** and the by-product **4** was observed in 95% NMR yield based on **1b**. Flash column chromatography (C18 reverse phase; eluent: CH_3CN/H_2O = 1:9 to 9:1) afforded **6g** in 56% yield (19.5 mg, 56.3 µmol) as a colourless oil. **TLC**: R_f (*n*-hexane/EtOAc = 20:1) = 0.33; **^{1}H NMR** (400 MHz, $CDCl_3$) δ 5.86 – 5.84 (m, 1H, C=C*H*), 4.20 – 4.05 (m, 2H, $CO_2CH_2CH_3$), 2.69 – 2.41 (m, 6H, CH_2), 2.22 (s, 1H, C*H*CO_2), 2.05 – 1.92 (m, 2H, CH_2), 1.67 – 1.54 (m, 2H, CH_2), 1.45 – 1.19 (m, 21H, CH_2 & $CO_2CH_2CH_3$), 0.93 – 0.79 (m, 3H, CH_3); **^{13}C NMR** (101 MHz, $CDCl_3$) δ 176.2, 135.0, 130.5, 108.6, 101.1, 60.1, 34.1, 33.0, 32.1, 29.80, 29.78 (2C), 29.7, 29.5 (2C), 29.4, 27.5, 25.5, 23.9, 22.8, 22.4, 14.6, 14.3; **IR** (ν_{max}, cm^{-1}) 3429 (w), 2925 (s), 2853 (s), 1874 (w), 1724 (s), 1607 (w), 1464 (m), 1369 (w), 1323 (w), 1244 (m), 1174 (s), 1095 (w), 1037 (m), 1003 (w), 950 (w), 811 (w), 723 (w); **HRMS** (Sicrit plasma/LTQ-Orbitrap) m/z: $[M + H]^+$ Calcd for $C_{23}H_{39}O_2^+$ 347.2945; Found 347.2945.

6.2.8. Synthesis and characterization of ethyl 2-(cyclopent-1-en-1-yl)-3-hexylcycloprop-2-ene-1-carboxylate (6h)

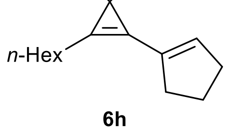

6h

Following **GPE**, a mixture of $(Me_2S)AuCl$ (1.47 mg, 5.00 μmol, 5.00 mol%), **L1** (2.10 mg, 10.0 μmol, 10.0 mol%), CpBX **1a** (56.4 mg, 0.100 mmol, 1.00 equiv.), vinylboronic acid **5g** (11.2 mg, 0.100 mmol, 1.00 equiv.) and CH_3CN (2.0 mL) was stirred at 40 °C for 17 hours. Analysis of the crude product gave 63% NMR yield of **6h** and the by-product **4** was observed in 99% NMR yield. Flash column chromatography (C18 reverse phase; eluent: CH_3CN/H_2O = 1:9 to 9:1) afforded **6h** in 62% yield (16.3 mg, 62.1 μmol) as a colourless oil. **TLC**: R_f (*n*-hexane/EtOAc = 20:1) = 0.30; **^{1}H NMR** (400 MHz, $CDCl_3$) δ 5.86 – 5.84 (m, 1H, C=C*H*), 4.21 – 4.05 (m, 2H, $CO_2CH_2CH_3$), 2.70 – 2.32 (m, 6H, CH_2), 2.22 (s, 1H, $CHCO_2$), 2.08 – 1.84 (m, 2H, CH_2), 1.64 – 1.57 (m, 2H, CH_2), 1.46 – 1.13 (m, 9H, CH_2 & $CO_2CH_2CH_3$), 1.02 – 0.73 (m, 3H, CH_3); **^{13}C NMR** (101 MHz, $CDCl_3$) δ 176.2, 135.0, 130.6, 108.6, 101.1, 60.1, 34.1, 33.0, 31.7, 29.1, 27.5, 25.5, 23.9, 22.7, 22.5, 14.6, 14.2; **IR** (ν_{max}, cm^{-1}) 2956 (s), 2931 (s), 2858 (m), 1722 (s), 1587 (w), 1465 (m), 1373 (w), 1335 (w), 1303 (w), 1249 (m), 1185 (s), 1096 (w), 1034 (m), 865 (w), 744 (w); **HRMS** (ESI/QTOF) m/z: $[M + H]^+$ Calcd for $C_{17}H_{27}O_2^+$ 263.2006; Found 263.2004.

6.2.9. Synthesis and characterization of *tert*-butyl 2-(cyclopent-1-en-1-yl)-3-hexylcycloprop-2-ene-1-carboxylate (6i)

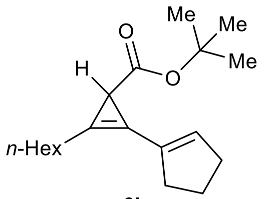

6i

Following **GPE**, a mixture of $(Me_2S)AuCl$ (1.47 mg, 5.00 μmol, 5.00 mol%), **L1** (2.10 mg, 10.0 μmol, 10.0 mol%), CpBX **1d** (59.2 mg, 0.100 mmol, 1.00 equiv.), vinylboronic acid **5g** (11.2 mg, 0.100 mmol, 1.00 equiv.) and CH_3CN (2.0 mL) was stirred at 40 °C for 14 hours. Analysis of the crude product gave 53% NMR yield of **6i** and the by-product **4** was observed in 98% NMR yield. Flash column chromatography (C18 reverse phase; eluent: CH_3CN/H_2O = 1:9 to 9:1) afforded **6i** in 53% yield (15.4 mg, 53.0 μmol) as a colourless oil. **TLC**: R_f (*n*-hexane/EtOAc = 20:1) = 0.38; **^{1}H NMR** (400 MHz, $CDCl_3$) δ 5.84 – 5.81 (m, 1H, C=C*H*), 2.73 – 2.34 (m, 6H, CH_2), 2.13 (s, 1H, $CHCO_2$), 2.06 – 1.90 (m, 2H, CH_2), 1.64 – 1.56 (m, 2H, CH_2), 1.54 – 1.21 (m, 15H, CH_2 & $CO_2C(CH_3)_3$), 1.02 – 0.69 (m, 3H, CH_3); **^{13}C NMR** (101 MHz, $CDCl_3$) δ 175.6, 134.5, 130.8, 109.0, 101.4, 79.6, 34.1, 33.0, 31.8, 29.1, 28.4, 27.6, 25.5, 23.9, 23.4, 22.7, 14.2; **IR** (ν_{max}, cm^{-1}) 2958 (m), 2931 (m), 2856 (m), 1720 (s), 1458 (w), 1367 (m), 1345 (w), 1324 (w), 1253 (w), 1211 (w), 1154 (s), 1004 (w), 957 (w), 853 (w), 813 (w), 734 (w); **HRMS** (ESI/QTOF) m/z: $[M + Na]^+$ Calcd for $C_{19}H_{30}NaO_2^+$ 313.2138; Found 313.2153.

6.2.10. Synthesis and characterization of 2-phenylpropan-2-yl 2-(cyclopent-1-en-1-yl)-3-hexylcycloprop-2-ene-1-carboxylate (6j)

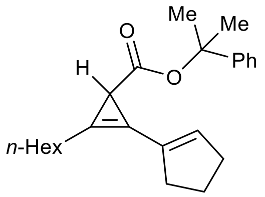

6j

Following **GPE**, a mixture of $(Me_2S)AuCl$ (1.47 mg, 5.00 μmol, 5.00 mol%), **L1** (2.10 mg, 10.0 μmol, 10.0 mol%), CpBX **1e** (65.4 mg, 0.100 mmol, 1.00 equiv.), vinylboronic acid **5g** (11.2 mg, 0.100 mmol, 1.00 equiv.) and CH_3CN (2.0 mL) was stirred at 40 °C for 14 hours. Analysis of the crude product gave 55% NMR yield of **6j** and the by-product **4** was observed in 99% NMR yield. Flash column chromatography (C18 reverse phase; eluent: CH_3CN/H_2O = 1:9 to 9:1) afforded **6j** in 54% yield (19.1 mg, 54.2 μmol) as a colourless oil. **TLC**: R_f (*n*-hexane/EtOAc = 20:1) = 0.32; **^{1}H NMR** (400 MHz, $CDCl_3$) δ 7.46 – 7.08 (m,

5H, Ar*H*), 5.95 – 5.77 (m, 1H, C=C*H*), 2.66 – 2.34 (m, 6H, CH_2), 2.23 (s, 1H, C*H*CO_2), 2.03 – 1.95 (m, 2H, CH_2), 1.80 – 1.73 (m, 6H, CH_3), 1.69 – 1.50 (m, 2H, CH_2), 1.47 – 1.16 (m, 6H, CH_2), 0.89 (t, *J* = 6.6 Hz, 3H, CH_3); **^{13}C NMR** (101 MHz, $CDCl_3$) δ 174.8, 146.8, 134.7, 130.8, 128.2, 126.8, 124.4, 108.9, 101.4, 80.9, 34.1, 33.1, 31.7, 29.2, 29.0, 28.9, 27.6, 25.6, 23.9, 23.5, 22.7, 14.2; **IR** (ν_{max}, cm^{-1}) 2955 (s), 2930 (s), 2857 (m), 1724 (s), 1602 (w), 1496 (w), 1465 (w), 1450 (m), 1382 (w), 1365 (w), 1344 (w), 1324 (w), 1270 (m), 1249 (m), 1187 (m), 1139 (s), 1101 (m), 1077 (w), 957 (w), 845 (w), 765 (m); **HRMS** (ESI/QTOF) m/z: [M + Na]$^+$ Calcd for $C_{24}H_{32}NaO_2^+$ 375.2295; Found 375.2291.

6.2.11. Synthesis and characterization of ethyl (*E*)-2-hexyl-3-(4-methylstyryl)cycloprop-2-ene-1-carboxylate (6k)

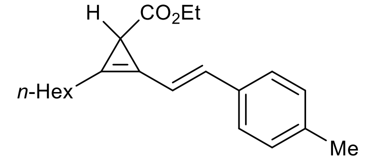

6k

Following **GPE**, a mixture of (Me_2S)AuCl (1.47 mg, 5.00 μmol, 5.00 mol%), **L1** (2.10 mg, 10.0 μmol, 10.0 mol%), CpBX **1a** (73.4 mg, 0.130 mmol, 1.30 equiv.), vinylboronic acid **5b** (16.2 mg, 0.100 mmol, 1.00 equiv.) and CH_3CN (2.0 mL) was stirred at 40 °C for 18 hours. Analysis of the crude product gave 90% NMR yield of **6k** and the by-product **4** was observed in 99% NMR yield based on **1a**. Flash column chromatography (C18 reverse phase; eluent: CH_3CN/H_2O = 1:9 to 9:1) afforded **6k** in 88% yield (27.6 mg, 88.3 μmol) as a colourless oil. **TLC**: R_f (*n*-hexane/EtOAc = 20:1) = 0.27; **^{1}H NMR** (400 MHz, $CDCl_3$) δ 7.36 – 7.31 (m, 2H, Ar*H*), 7.16 – 7.13 (m, 2H, Ar*H*), 6.85 (d, *J* = 15.6 Hz, 1H, C*H*=CH), 6.68 (d, *J* = 15.6 Hz, 1H, CH=C*H*), 4.21 – 4.10 (m, 2H, $CO_2CH_2CH_3$), 2.57 (t, *J* = 7.3 Hz, 2H, CH_2CH_2C), 2.34 (s, 3H, CH_3), 2.32 (s, 1H, C*H*CO_2), 1.68 – 1.60 (m, 2H, CH_2CH_2C), 1.46 – 1.17 (m, 9H, CH_2 & $CO_2CH_2CH_3$), 1.03 – 0.76 (m, 3H, CH_3); **^{13}C NMR** (101 MHz, $CDCl_3$) δ 175.9, 138.6, 138.1, 133.7, 129.5, 127.0, 112.0, 111.0, 103.6, 60.2, 31.7, 29.1, 27.2, 25.7, 22.7, 21.9, 21.4, 14.5, 14.2; **IR** (ν_{max}, cm^{-1}) 3026 (w), 2957 (m), 2928 (s), 2858 (m), 1866 (w), 1720 (s), 1602 (w), 1512 (w), 1465 (w), 1369 (w), 1332 (w), 1244 (m), 1175 (s), 1097 (w), 1036 (m), 960 (m), 856 (w), 805 (m), 730 (w); **HRMS** (ESI/QTOF) m/z: [M + H]$^+$ Calcd for $C_{21}H_{29}O_2^+$ 313.2162; Found 313.2168.

6.2.12. Synthesis and characterization of *tert*-butyl (*E*)-2-hexyl-3-(4-methylstyryl)cycloprop-2-ene-1-carboxylate (6l)

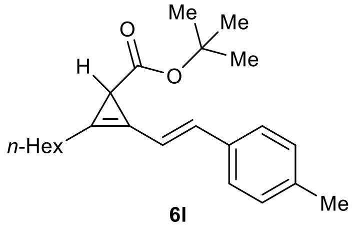

6l

Following **GPE**, a mixture of (Me_2S)AuCl (1.47 mg, 5.00 μmol, 5.00 mol%), **L1** (2.10 mg, 10.0 μmol, 10.0 mol%), CpBX **1d** (77.0 mg, 0.130 mmol, 1.30 equiv.), vinylboronic acid **5b** (16.2 mg, 0.100 mmol, 1.00 equiv.) and CH_3CN (2.0 mL) was stirred at 40 °C for 12 hours. Analysis of the crude product gave 64% NMR yield of **6l** and the by-product **4** was observed in 96% NMR yield based on **1d**. Flash column chromatography (C18 reverse phase; eluent: CH_3CN/H_2O = 1:9 to 9:1) afforded **6l** in 63% yield (21.5 mg, 63.1 μmol) as a colourless oil. **TLC**: R_f (*n*-hexane/EtOAc = 20:1) = 0.29; **^{1}H NMR** (400 MHz, $CDCl_3$) δ 7.38 – 7.30 (m, 2H, Ar*H*), 7.16 – 7.12 (m, 2H, Ar*H*), 6.84 (d, *J* = 15.5 Hz, 1H, C*H*=CH), 6.67 (d, *J* = 15.5 Hz, 1H, CH=C*H*), 2.56 (t, *J* = 7.2 Hz, 2H, CH_2CH_2C), 2.35 (s, 3H, CH_3), 2.21 (s, 1H, C*H*CO_2), 1.72 – 1.55 (m, 2H, CH_2CH_2C), 1.50 – 1.22 (m, 15H, CH_2 & $CO_2C(CH_3)_3$), 0.98 – 0.83 (m, 3H, CH_3); **^{13}C NMR** (101 MHz, $CDCl_3$) δ 175.3, 138.5, 137.7, 133.9, 129.5, 127.0, 112.4, 111.4, 104.0, 79.7, 31.7, 29.1, 28.4, 27.3, 25.8,

23.0, 22.7, 21.4, 14.2; **IR** (ν_{max}, cm^{-1}) 2957 (m), 2929 (m), 2859 (m), 1714 (s), 1599 (w), 1509 (w), 1455 (w), 1367 (m), 1335 (w), 1253 (m), 1148 (s), 997 (w), 961 (m), 851 (w), 802 (m); **HRMS** (ESI/QTOF) m/z: $[M + Na]^+$ Calcd for $C_{23}H_{32}NaO_2^+$ 363.2295; Found 363.2295.

6.2.13. Synthesis and characterization of 2-phenylpropan-2-yl (*E*)-2-hexyl-3-(4-methylstyryl)cycloprop-2-ene-1-carboxylate (6m)

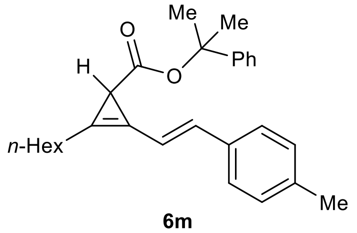

6m

Following **GPE**, a mixture of $(Me_2S)AuCl$ (1.47 mg, 5.00 μmol, 5.00 mol%), **L1** (2.10 mg, 10.0 μmol, 10.0 mol%), CpBX **1e** (85.1 mg, 0.130 mmol, 1.30 equiv.), vinylboronic acid **5b** (16.2 mg, 0.100 mmol, 1.00 equiv.) and CH_3CN (2.0 mL) was stirred at 40 °C for 12 hours. Analysis of the crude product gave 73% NMR yield of **6m** and the by-product **4** was observed in 95% NMR yield based on **1e**. Flash column chromatography (C18 reverse phase; eluent: CH_3CN/H_2O = 1:9 to 9:1) afforded **6m** in 72% yield (28.9 mg, 71.8 μmol) as a colourless oil. **TLC**: R_f (*n*-hexane/EtOAc = 20:1) = 0.24; **^{1}H NMR** (400 MHz, $CDCl_3$) δ 7.44 – 7.34 (m, 4H, Ar*H*), 7.34 – 7.26 (m, 2H, Ar*H*), 7.26 – 7.13 (m, 3H, Ar*H*), 6.91 (d, *J* = 15.5 Hz, 1H, C*H*=CH), 6.75 (d, *J* = 15.6 Hz, 1H, CH=C*H*), 2.62 (t, *J* = 7.2 Hz, 2H, CH_2CH_2C), 2.40 (s, 3H, CH_3), 2.33 (s, 1H, C*H*CO_2), 1.82 (s, 3H, CH_3), 1.76 (s, 3H, CH_3), 1.73 – 1.65 (m, 2H, CH_2CH_2C), 1.51 – 1.22 (m, 6H, CH_2), 1.02 – 0.82 (m, 3H, CH_3); **^{13}C NMR** (101 MHz, $CDCl_3$) δ 174.6, 146.6, 138.6, 138.1, 133.8, 129.6, 128.2, 127.0, 126.8, 124.4, 112.3, 111.2, 104.1, 80.9, 31.7, 29.3, 29.1, 28.6, 27.3, 25.8, 23.0, 22.7, 21.5, 14.2; **IR** (ν_{max}, cm^{-1}) 3029 (w), 2949 (m), 2932 (s), 2859 (m), 1723 (s), 1605 (w), 1512 (w), 1455 (w), 1379 (w), 1249 (m), 1184 (m), 1138 (s), 1102 (m), 1076 (w), 1000 (w), 959 (m), 849 (w), 802 (m), 766 (m); **HRMS** (ESI/QTOF) m/z: $[M + Na]^+$ Calcd for $C_{28}H_{34}NaO_2^+$ 425.2451; Found 425.2435.

6.2.14. Synthesis and characterization of benzyl (*E*)-2-hexyl-3-(4-methylstyryl)cycloprop-2-ene-1-carboxylate (6n)

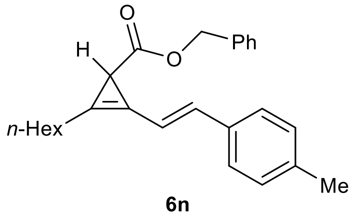

6n

Following **GPE**, a mixture of $(Me_2S)AuCl$ (1.47 mg, 5.00 μmol, 5.00 mol%), **L1** (2.10 mg, 10.0 μmol, 10.0 mol%), CpBX **1f** (81.4 mg, 0.130 mmol, 1.30 equiv.), vinylboronic acid **5b** (16.2 mg, 0.100 mmol, 1.00 equiv.) and CH_3CN (2.0 mL) was stirred at 40 °C for 12 hours. Analysis of the crude product gave 85% NMR yield of **6n** and the by-product **4** was observed in 93% NMR yield based on **1f**. Flash column chromatography (C18 reverse phase; eluent: CH_3CN/H_2O = 1:9 to 9:1) afforded **6n** in 82% yield (30.8 mg, 82.2 μmol) as a colourless oil. **TLC**: R_f (*n*-hexane/EtOAc = 20:1) = 0.22; **^{1}H NMR** (400 MHz, $CDCl_3$) δ 7.45 – 7.27 (m, 7H, Ar*H*), 7.16 (d, *J* = 7.9 Hz, 2H, Ar*H*), 6.86 (d, *J* = 15.6 Hz, 1H, C*H*=CH), 6.68 (d, *J* = 15.6 Hz, 1H, CH=C*H*), 5.24 – 5.11 (m, 2H, OCH_2Ph), 2.58 (t, *J* = 7.3 Hz, 2H, CH_2CH_2C), 2.39 (s, 1H, C*H*CO_2), 2.36 (s, 3H, CH_3), 1.78 – 1.53 (m, 2H, CH_2CH_2C), 1.48 – 1.16 (m, 6H, CH_2), 1.02 – 0.75 (m, 3H, CH_3); **^{13}C NMR** (101 MHz, $CDCl_3$) δ 175.8, 138.7, 138.4, 136.8, 133.6, 129.5, 128.6, 128.0 (2C), 127.0, 111.9, 110.8, 103.6, 65.9, 31.6, 29.1, 27.2, 25.7, 22.7, 22.0, 21.4, 14.2; **IR** (ν_{max}, cm^{-1}) 3433 (w), 3029 (w), 2954 (m), 2928 (s), 2858 (m), 1717 (s), 1599 (m), 1512 (w), 1456 (m), 1379 (w), 1331 (w), 1239 (m), 1159 (s),

999 (m), 961 (m), 805 (m), 737 (m); **HRMS** (ESI/QTOF) m/z: [M + H]$^+$ Calcd for $C_{26}H_{31}O_2^+$ 375.2319; Found 375.2304.

6.2.15. Synthesis and characterization of adamantan-1-yl 2-hexyl-3-((*E*)-4-methylstyryl)cycloprop-2-ene-1-carboxylate (6o)

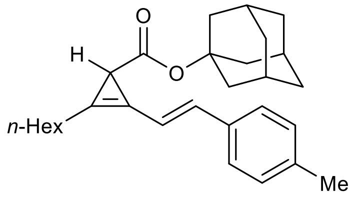

6o

Following **GPE**, a mixture of (Me_2S)AuCl (1.47 mg, 5.00 µmol, 5.00 mol%), **L1** (2.10 mg, 10.0 µmol, 10.0 mol%), CpBX **1g** (87.2 mg, 0.130 mmol, 1.30 equiv.), vinylboronic acid **5b** (16.2 mg, 0.100 mmol, 1.00 equiv.) and CH_3CN (2.0 mL) was stirred at 40 °C for 12 hours. Analysis of the crude product gave 77% NMR yield of **6o** and the by-product **4** was observed in 95% NMR yield based on **1g**. Flash column chromatography (C18 reverse phase; eluent: CH_3CN/H_2O = 1:9 to 9:1) afforded **6o** in 74% yield (31.1 mg, 74.3 µmol) as a colourless oil. **TLC**: R_f (*n*-hexane/EtOAc = 20:1) = 0.30; **^{1}H NMR** (400 MHz, $CDCl_3$) δ 7.36 – 7.32 (m, 2H, Ar*H*), 7.17 – 7.12 (m, 2H, Ar*H*), 6.83 (d, *J* = 15.6 Hz, 1H, C*H*=CH), 6.68 (d, *J* = 15.5 Hz, 1H, CH=C*H*), 2.56 (t, *J* = 7.2 Hz, 2H, CH_2CH_2C), 2.35 (s, 3H, CH_3), 2.20 (s, 1H, , C*H*CO_2), 2.16 – 2.05 (m, 9H, C*H*(adamantyl) & CH_2(adamantyl)), 1.74 – 1.56 (m, 8H, CH_2), 1.49 – 1.21 (m, 6H, CH_2), 0.96 – 0.84 (m, 3H, CH_3); **^{13}C NMR** (101 MHz, $CDCl_3$) δ 175.0, 138.4, 137.7, 133.9, 129.5, 127.0, 112.4, 111.5, 104.1, 79.7, 41.6, 36.4, 31.7, 31.0, 29.1, 27.3, 25.7, 23.1, 22.7, 21.4, 14.2; **IR** (ν_{max}, cm^{-1}) 3028 (w), 2913 (s), 2856 (s), 1712 (s), 1602 (m), 1514 (w), 1456 (m), 1348 (w), 1251 (m), 1179 (s), 1105 (w), 1061 (s), 967 (m), 805 (m), 737 (w); **HRMS** (ESI/QTOF) m/z: [M + Na]$^+$ Calcd for $C_{29}H_{38}NaO_2^+$ 441.2764; Found 441.2782.

6.3. Attempts of gold-catalysed σ-type cyclopropenium cation transfer to other substrates

6.3.1 Gold-catalysed σ-type cyclopropenium cation transfer reaction of CpBXs with allenamides

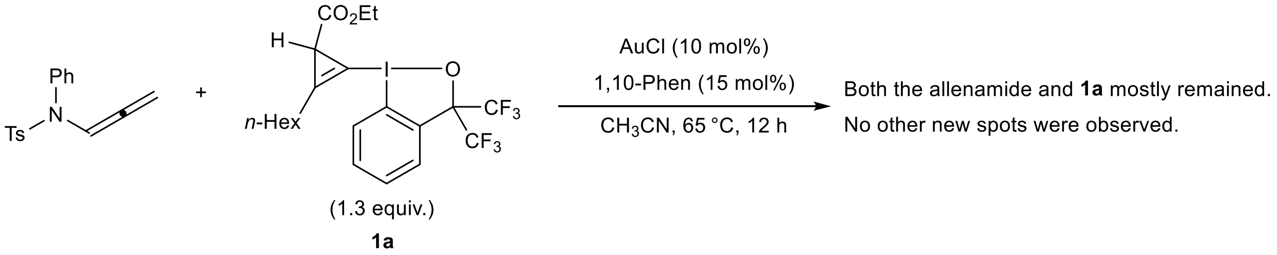

The reaction was run according to a previously reported procedure[53]. An oven-dried 10 mL Schlenk tube was sequentially charged with a magnetic stir-bar, 1,10-phenanthroline (2.70 mg, 15.0 µmol, 15.0 mol%), 4-methyl-*N*-phenyl-*N*-(propa-1,2-dien-1-yl)benzenesulfonamide (28.5 mg, 100 µmol, 1.00 equiv.) and CpBX **1a** (73.4 mg, 130 µmol, 1.30 equiv.). The Schlenk tube was then introduced into a glovebox and AuCl (2.32 mg, 10.0 µmol, 10.0 mol%) was added. The Schlenk tube was then taken out of the glovebox. Subsequently, CH_3CN (0.05 M; 2.0 mL) was added by syringe. The reaction mixture was stirred at 65 °C for 12 hours. Thin layer chromatography indicated that both the allenamide and CpBX **1a** mostly remained, and no other new spots were observed.

6.3.2 Gold-catalysed σ-type cyclopropenium cation transfer reaction of CpBXs with allenamides in the presence of 2-iodobenzoic acid

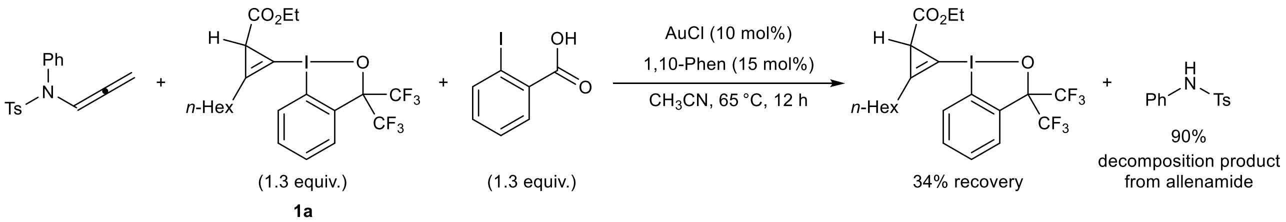

The reaction was run according to a previously reported procedure[53]. An oven-dried 10 mL Schlenk tube was sequentially charged with a magnetic stir-bar, 1,10-phenanthroline (2.70 mg, 15.0 µmol, 15.0 mol%), 4-methyl-*N*-phenyl-*N*-(propa-1,2-dien-1-yl)benzenesulfonamide (28.5 mg, 100 µmol, 1.00 equiv.), 2-iodobenzoic acid (32.2 mg, 130 µmol, 1.30 equiv.) and CpBX **1a** (73.4 mg, 130 µmol, 1.30 equiv.). The Schlenk tube was then introduced into a glovebox and AuCl (2.32 mg, 10.0 µmol, 10.0 mol%) was added. The Schlenk tube was then taken out of the glovebox. Subsequently, CH_3CN (0.05 M; 2.0 mL) was added by syringe. The reaction mixture was stirred at 65 °C for 12 hours. The reaction mixture was then filtered through a silica gel pad and washed with CH_2Cl_2 (3 × 5.0 mL). The solvent was removed under reduced pressure, and the resulting crude residue was subjected to preparative thin-layer chromatography (eluent: hexane/ethyl acetate = 10:1). The major new spot was isolated and indentified to be 4-methyl-*N*-phenylbenzenesulfonamide (22.3 mg, 90.2 µmol, 90% yield), which comes from the decomposition of the substrate 4-methyl-*N*-phenyl-*N*-(propa-1,2-dien-1-yl)benzenesulfonamide. The CpBX **1a** was recovered in 34% yield (24.7 mg). Apart from those, no other prominent new spots were observed.

6.3.3 Gold-catalysed σ-type cyclopropenium cation transfer reaction of CpBXs with indole

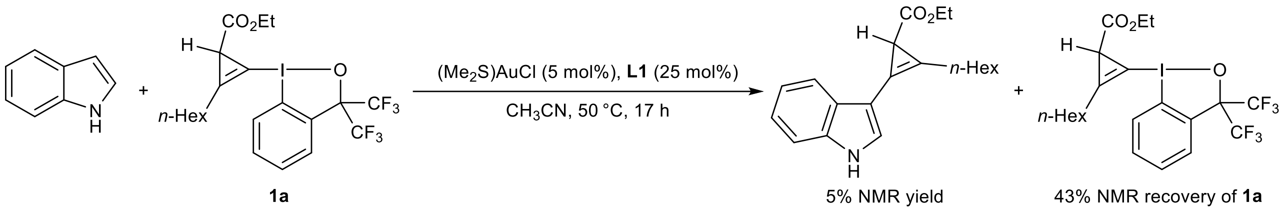

An oven-dried 10 mL Schlenk tube was sequentially charged with a magnetic stir-bar, **L1** (5.25 mg, 25.0 µmol, 25.0 mol%), $(Me_2S)AuCl$ (1.47 mg, 5.00 µmol, 5.00 mol%), indole (11.7 mg, 100 µmol, 1.00 equiv.) and CpBX **1a** (56.43 mg, 100 µmol, 1.00 equiv.). The Schlenk tube was then evacuated and backfilled with nitrogen three times. Subsequently, CH_3CN (0.05 M; 2.0 mL) was added by syringe. The reaction mixture was stirred at 50 °C for 17 hours. The reaction mixture was then filtered through a silica gel pad and washed with CH_2Cl_2 (3 × 5.0 mL). The solvent was removed under reduced pressure, and the resulting crude residue was subjected to a flash column chromatography on silica gel (eluent: pentane/ethyl acetate = 4:1). The fractions that contained the products were collected and analysed by ^1H NMR spectroscopy. Analysis of the product fraction gave 5% NMR yield of the desired product and CpBX **1a** was recovered in 43% NMR yield.

6.3.4 Gold-catalysed σ-type cyclopropenium cation transfer reaction of CpBXs with 1-methyl-1*H*-indole

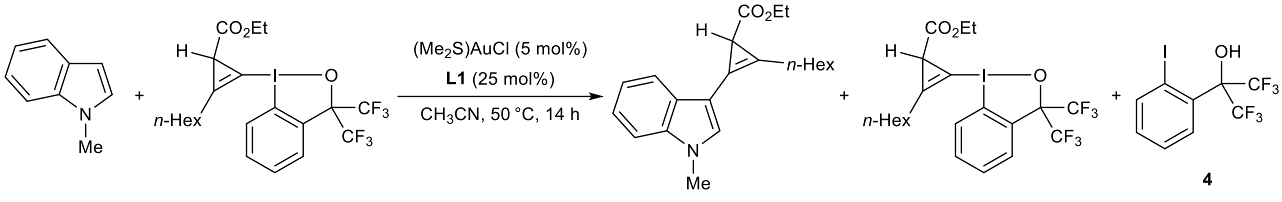

An oven-dried 10 mL Schlenk tube was sequentially charged with a magnetic stir-bar, **L1** (5.25 mg, 25.0 µmol, 25.0 mol%), (Me_2S)AuCl (1.47 mg, 5.00 µmol, 5.00 mol%), 1-methyl-1*H*-indole (13.1 mg, 100 µmol, 1.00 equiv.) and CpBX **1a** (56.43 mg, 100 µmol, 1.00 equiv.). The Schlenk tube was then evacuated and backfilled with nitrogen three times. Subsequently, CH_3CN (0.05 M; 2.0 mL) was added by syringe. The reaction mixture was stirred at 50 °C for 14 hours. The reaction mixture was then filtered through a silica gel pad and washed with CH_2Cl_2 (3 × 5.0 mL). The solvent was removed under reduced pressure, and the resulting crude residue was subjected to a flash column chromatography on silica gel (eluent: pentane/ethyl acetate = 5:1). The fractions that contained the products were collected and analysed by ^{1}H NMR spectroscopy. Analysis of the product fraction gave 21% NMR yield of the desired product and CpBX **1a** was recovered in 27% NMR yield. The by-product **4** was observed in 69% NMR yield.

7. Transformations of products and applications

7.1. Selective reduction of alkynyl-cyclopropenes using DIBAL-H[54]

7.1.1. Synthesis and characterization of (2-dodecyl-3-(phenylethynyl)cycloprop-2-en-1-yl)methanol (7)

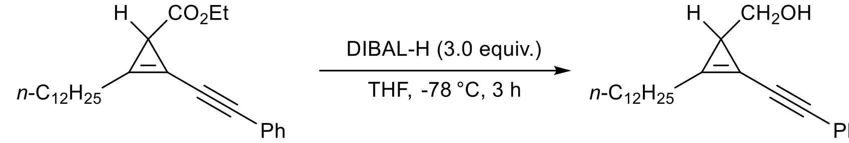

A 25 mL Schlenk tube was charged with **3bm** (190 mg, 0.500 mmol, 1.00 equiv.) and a magnetic stir-bar. The Schlenk tube was then evacuated and backfilled with nitrogen three times. Subsequently, THF (0.1 M; 5.0 mL) was added by syringe and the Schlenk tube was placed at −78 °C in a dry ice/acetone bath. After that, diisobutylaluminum hydride (DIBAL-H, 1.0 M in toluene; 1.50 mmol, 1.5 mL, 3.00 equiv.) was added dropwise by a syringe pump over 5 min and the reaction mixture was stirred at −78 °C for additional 3 hours. Then the cooling bath was removed. The reaction mixture was allowed to warm to room temperature gradually (ca. 20 min) while keeping stirring. The reaction mixture was then quenched by adding saturated aqueous NH_4Cl (10 mL) and extracted with EtOAc (10 ml × 3). The combined organic layers were successively washed with brine and H_2O, then dried over anhydrous Na_2SO_4, filtered, and concentrated under reduced pressure. The residue was purified by column chromatography on silica gel (eluent: pentane/EtOAc = 10:1) to give the desired product **7** in 94% yield (159 mg, 469 µmol) as a colorless oil. **TLC**: R_f (*n*-hexane/EtOAc = 4:1) = 0.45; **^{1}H NMR** (400 MHz, $CDCl_3$) δ 7.51 – 7.43 (m, 2H, Ar*H*), 7.39 – 7.30 (m, 3H, Ar*H*), 3.72 – 3.63 (m, 2H, C*H*$_2$OH), 2.59 (t, *J* = 7.2 Hz, 2H, CH_2C*H*$_2$C), 2.11 (t, *J* = 4.4 Hz, 1H, C*H*CH_2OH), 1.68 – 1.60 (m, 2H, C*H*$_2CH_2$C), 1.45 – 1.21 (m, 19H, C*H*$_2$ & O*H*), 0.96 – 0.81 (m, 3H, C*H*$_3$); **^{13}C NMR** (101 MHz, $CDCl_3$) δ 131.7, 128.8, 128.5, 128.3, 123.1, 100.2, 96.9, 79.0, 67.5, 32.1, 29.83, 29.79 (2C), 29.7, 29.51 (2C), 29.45, 27.5, 27.2, 26.4, 22.8, 14.3; **IR** (ν_{max}, cm^{-1}) 3359 (w), 2924 (s), 2852 (s), 2203 (w), 1821 (w), 1595 (w), 1490 (w), 1465 (m), 1443 (w),

1054 (w), 1018 (m), 755 (s); **HRMS** (ESI/QTOF) m/z: [M + Na]$^+$ Calcd for $C_{24}H_{34}NaO^+$ 361.2502; Found 361.2506.

7.2. Hydroalkylation of alkynyl-cyclopropene 7 via copper-catalyzed carbomagnesiation[55]

7.2.1. Synthesis and characterization of (2-dodecyl-2-methyl-3-(phenylethynyl)cyclopropyl)methanol (8)

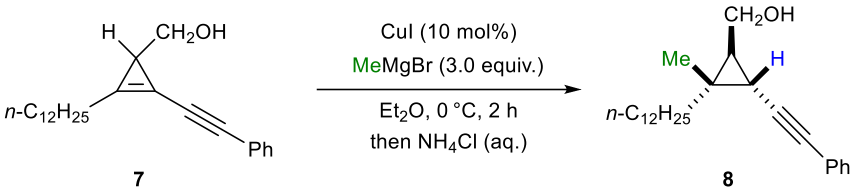

A 10 mL Schlenk tube was charged with **7** (33.9 mg, 0.100 mmol, 1.00 equiv.), copper iodide (CuI; 1.90 mg, 10.0 µmol, 10.0 mol%) and a magnetic stir-bar. The Schlenk tube was then evacuated and backfilled with nitrogen three times. Subsequently, Et_2O (0.05 M; 2.0 mL) was added by syringe and the mixture was stirred at room temperature. Then methylmagnesium bromide (MeMgBr, 3.0 M in Et_2O; 0.10 mL, 0.300 mmol, 3.00 equiv.) was added dropwise. The resulting reaction mixture was stirred at room temperature for 2 hours. Then the mixture was quenched by adding saturated aqueous NH_4Cl (1.0 mL) and extracted with EtOAc (5.0 ml × 3). The combined organic layers were successively washed with brine and H_2O, then dried over anhydrous Na_2SO_4, filtered, and concentrated under reduced pressure. The residue was purified by column chromatography on silica gel (eluent: pentane/EtOAc = 10:1) to give the hydroalkylation product **8** in 79% yield (28.0 mg, 79.0 µmol) as a colorless oil. **TLC**: R_f (*n*-hexane/EtOAc = 10:1) = 0.07; **^{1}H NMR** (400 MHz, $CDCl_3$) δ 7.26 – 7.21 (m, 2H, Ar*H*), 7.19 – 7.08 (m, 3H, Ar*H*), 3.74 – 3.64 (m, 1H, C*H*$_2$OH), 3.51 – 3.41 (m, 1H, C*H*$_2$OH), 1.48 – 1.06 (m, 25H, C*H* & C*H*$_2$ & O*H*), 1.03 (s, 3H, C*H*$_3$), 0.76 (t, *J* = 6.7 Hz, 3H, C*H*$_3$); **^{13}C NMR** (101 MHz, $CDCl_3$) δ 131.6, 128.3, 127.6, 124.2, 90.3, 79.1, 62.7, 37.5, 36.1, 32.1, 30.1, 29.9, 29.82 (2C), 29.79 (2C), 29.5, 28.2, 26.7, 22.8, 19.5, 17.5, 14.3; **IR** (ν_{max}, cm^{-1}) 3366 (w), 2950 (m), 2925 (s), 2853 (s), 2222 (m), 1599 (w), 1490 (w), 1463 (m), 1445 (m), 1379 (w), 1253 (w), 1069 (m), 1023 (m), 907 (w), 754 (s); **HRMS** (ESI/QTOF) m/z: [M + Na]$^+$ Calcd for $C_{25}H_{38}NaO^+$ 377.2815; Found 377.2813.

7.3. Difunctionalization of alkynyl-cyclopropene 7 via copper-catalyzed carbomagnesiation[33]

7.3.1. Synthesis and characterization of (2-allyl-3-dodecyl-3-methyl-2-(phenylethynyl)cyclopropyl)methanol (9)

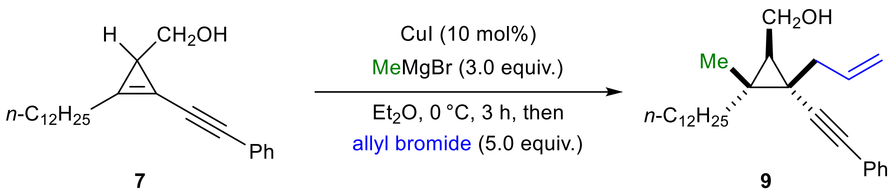

A 10 mL Schlenk tube was charged with **7** (33.9 mg, 0.100 mmol, 1.00 equiv.), copper iodide (CuI; 1.90 mg, 10.0 µmol, 10.0 mol%) and a magnetic stir-bar. The Schlenk tube was then evacuated and backfilled with nitrogen three times. Subsequently, Et_2O (0.05 M; 2.0 mL) was added by syringe and the mixture was stirred at room temperature. Then methylmagnesium bromide (MeMgBr, 3.0 M in Et_2O; 0.10 mL, 0.300 mmol, 3.00 equiv.) was added dropwise. The resulting reaction mixture was stirred at room temperature for 3 hours. Allyl bromide (43.3 µL, 0.500 mmol, 5.00 equiv.) was added dropwise to the reaction mixture. The resulting reaction mixture was stirred at room temperature for additional 5 hours. Then the mixture was quenched by adding saturated aqueous NH_4Cl (1.0 mL) and extracted with EtOAc (5.0 ml × 3). The combined organic layers were successively washed with brine and H_2O, then dried over anhydrous Na_2SO_4, filtered, and concentrated under reduced pressure. The

residue was purified by column chromatography on silica gel (eluent: pentane/EtOAc = 10:1) to give the difunctionalization product **9** in 61% yield (24.2 mg, 61.3 µmol) as a colorless oil. **TLC**: R_f (*n*-hexane/EtOAc = 5:1) = 0.35; **^{1}H NMR** (400 MHz, $CDCl_3$) δ 7.33 – 7.24 (m, 2H, Ar*H*), 7.24 – 7.13 (m, 3H, Ar*H*), 6.07 – 5.97 (m, 1H, $CH_2CH{=}CH_2$), 5.12 (dq, *J* = 17.2, 1.7 Hz, 1H, $CH_2CH{=}CH_2$), 5.08 – 5.04 (m, 1H, $CH_2CH{=}CH_2$), 3.65 (d, *J* = 7.7 Hz, 2H, $CH_2CH{=}CH_2$), 2.37 – 2.20 (m, 2H, CH_2OH), 1.66 – 1.39 (m, 3H, C*H* & CH_2), 1.39 – 1.10 (m, 21H, CH_2 & O*H*), 1.04 (s, 3H, CH_3), 0.90 – 0.70 (m, 3H, CH_3); **^{13}C NMR** (101 MHz, $CDCl_3$) δ 136.8, 131.6, 128.3, 127.5, 124.3, 116.4, 94.1, 79.4, 59.7, 39.9, 37.7, 33.5, 32.1, 30.12, 30.06, 29.9, 29.84, 29.82 (2C), 29.80, 29.5, 26.7, 25.1, 22.8, 14.3, 13.1; **IR** (ν_{max}, cm^{-1}) 3359 (w), 2926 (s), 2854 (s), 2221 (w), 1642 (w), 1598 (w), 1493 (w), 1463 (w), 1068 (w), 1004 (m), 912 (m), 755 (m); **HRMS** (ESI/QTOF) m/z: $[M + Na]^+$ Calcd for $C_{28}H_{42}NaO^+$ 417.3128; Found 417.3129.

7.4. Selective reduction of alkynyl-cyclopropenes using $LiAlH_4$[56]

7.4.1. Synthesis and characterization of (2-dodecyl-3-((4-pentylphenyl)ethynyl)cyclopropyl)methanol (10)

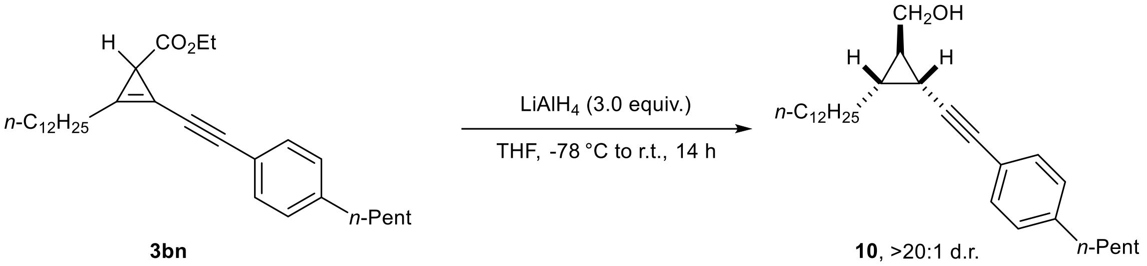

A 25 mL Schlenk tube was charged with **3bn** (45.1 mg, 0.100 mmol, 1.00 equiv.) and a magnetic stir-bar. The Schlenk tube was then evacuated and backfilled with nitrogen three times. Subsequently, THF (0.05 M; 2.0 mL) was added by syringe and the Schlenk tube was placed at −78 °C in a dry ice/acetone bath. After that, lithium aluminum hydride ($LiAlH_4$, 2.4 M in THF; 0.300 mmol, 0.13 mL, 3.00 equiv.) was added dropwise and then the reaction mixture was stirred with the dry ice/acetone bath. The reaction mixture was allowed to warm to room temperature naturally over 14 hours while keeping stirring. The reaction mixture was then quenched by adding saturated aqueous NH_4Cl (2.0 mL) and extracted with EtOAc (10 ml × 3). The combined organic layers were successively washed with brine and H_2O, then dried over anhydrous Na_2SO_4, filtered, and concentrated under reduced pressure. The residue was purified by column chromatography on silica gel (eluent: pentane/EtOAc = 8:1) to give the reduced product **10** in 32% yield (13.2 mg, 32.1 µmol, >20:1 dr. based on ^{1}H NMR) as a colorless oil. **TLC**: R_f (*n*-hexane/EtOAc = 4:1) = 0.40; **^{1}H NMR** (400 MHz, $CDCl_3$) δ 7.29 – 7.25 (m, 2H, Ar*H*), 7.13 – 7.00 (m, 2H, Ar*H*), 3.74 (dd, *J* = 11.5, 7.1 Hz, 1H, CH_2OH), 3.64 (dd, *J* = 11.5, 7.7 Hz, 1H, CH_2OH), 2.63 – 2.49 (m, 2H, CH_2Ph), 1.74 – 1.52 (m, 3H, C*H* & CH_2), 1.52 – 1.16 (m, 28H, C*H* & CH_2 & O*H*), 1.10 (t, *J* = 4.8 Hz, 1H, C*H*), 0.90 – 0.86 (m, 6H, CH_3); **^{13}C NMR** (101 MHz, $CDCl_3$) δ 142.7, 131.6, 128.4, 121.0, 91.4, 77.0, 62.0, 35.9, 32.1, 31.6, 31.1, 29.84 (2C), 29.81 (2C), 29.78, 29.7, 29.6, 29.5, 29.4, 28.0, 27.7, 22.8, 22.7, 14.3, 14.2, 12.6; **IR** (ν_{max}, cm^{-1}) 2954 (m), 2926 (s), 2855 (m), 2225 (w), 1800 (w), 1739 (m), 1512 (w), 1462 (w), 1443 (w), 1323 (w), 1287 (w), 1267 (w), 1253 (w), 1185 (w), 1160 (w), 1121 (w), 1064 (w), 1024 (w), 837 (w), 761 (w); **HRMS** (APCI/QTOF) m/z: $[M + H]^+$ Calcd for $C_{29}H_{47}O^+$ 411.3621; Found 411.3620.

7.5. Saponification of alkynyl-cyclopropene 3bm[57]

7.5.1. Synthesis and characterization of 2-dodecyl-3-(phenylethynyl)cycloprop-2-ene-1-carboxylic acid (11)

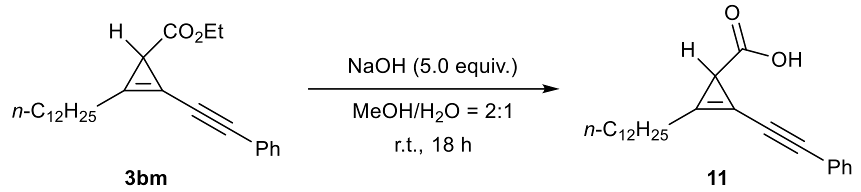

NaOH (20.0 mg, 0.500 mmol, 5.00 equiv.) was added to a 10 mL vial that contained the **3bm** (38.1 mg, 0.100 mmol, 1.00 equiv.), MeOH (0.25 mL) and deionized water (0.13 mL). The mixture was stirred under room temperature for 18 hours. The mixture was acidified by adding aqueous HCl (1.0 M, 0.50 mL) and extracted with DCM (5.0 ml × 4). The combined organic layers were successively washed with brine and H_2O, then dried over anhydrous Na_2SO_4, filtered, and concentrated under reduced pressure. The residue was purified by column chromatography (C18 reverse phase) to give the saponification product **11** in 82% yield (29.0 mg, 82.3 μmol) as a colorless oil. **TLC**: R_f (*n*-hexane/EtOAc = 4:1) = 0.10; **^{1}H NMR** (400 MHz, $CDCl_3$) δ 11.56 (bs, 1H, CO_2*H*), 7.60 – 7.45 (m, 2H, Ar*H*), 7.40 – 7.29 (m, 3H, Ar*H*), 2.66 – 2.54 (m, 2H, CH_2C*H*$_2$C), 2.51 (s, 1H, C*H*CO_2), 1.72 – 1.59 (m, 2H, C*H*$_2CH_2$C), 1.51 – 1.11 (m, 18H, C*H*$_2$), 0.88 (t, *J* = 6.8 Hz, 3H, C*H*$_3$); **^{13}C NMR** (101 MHz, $CDCl_3$) δ 181.1, 132.0, 129.2, 128.5, 122.4, 116.0, 99.2, 90.3, 76.2, 32.1, 29.80, 29.78, 29.74, 29.65, 29.5, 29.4, 29.3, 26.7, 26.1, 25.0, 22.8, 14.3; **IR** (ν_{max}, cm^{-1}) 2924 (s), 2853 (m), 2535 (w), 2204 (w), 1861 (w), 1692 (s), 1490 (w), 1462 (w), 1423 (m), 1271 (m), 1232 (m), 1072 (w), 999 (w), 944 (w), 919 (w), 755 (s); **HRMS** (ESI/QTOF) m/z: $[M + H_{-1}]^-$ Calcd for $C_{24}H_{31}O_2^-$ 351.2330; Found 351.2323.

7.6. Gold(I)-catalysed rearrangement of propargylic benzoate and ring-opening cascade

7.6.1. Synthesis and characterization of (*E*)-5-(2-ethoxy-2-oxoethylidene)heptadec-1-en-3-yn-2-yl benzoate (12)

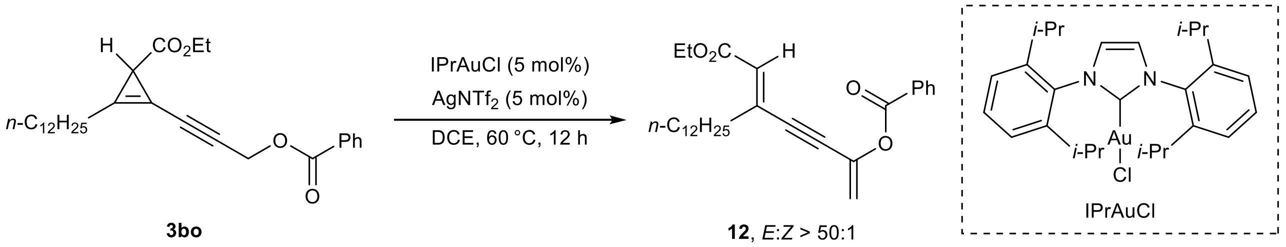

In a nitrogen-filled glovebox, a 10 ml Schlenk tube was sequentially charged with IPrAuCl (6.20 mg, 5.00 μmol, 5.00 mol%) and $AgNTf_2$ (1.90 mg, 5.00 μmol, 5.00 mol%). A magnetic stir bar was added to the Schlenk tube. The tube was then sealed with a rubber cap. The Schlenk tube was brought out of the glovebox and 1,2-dichloroethane (DCE; 1.0 mL) was added. The mixture was stirred under room temperature for 5 min. Then, **3bo** (43.9 mg, 0.100 mmol, 1.00 equiv., dissolved in 2.0 mL DCE) was added. The resulting mixture was stirred under 60 °C for 12 hours. The reaction mixture was then filtered through a silica gel pad and eluted with CH_2Cl_2 (3 × 5.0 mL). The solvent was removed under reduced pressure, and the resulting crude residue was purified by flash column chromatography on silica gel (eluent: pentane/EtOAc = 10:1) to give the ring-opening product **12** in 81% yield (35.4 mg, 80.7 μmol, *E*: *Z* > 50:1 base on ^{1}H NMR) as a colorless oil. **TLC**: R_f (*n*-hexane/EtOAc = 20:1) = 0.33; **^{1}H NMR** (400 MHz, $CDCl_3$) δ 8.15 – 8.05 (m, 2H, Ar*H*), 7.66 – 7.57 (m, 1H, Ar*H*), 7.51 – 7.45 (m, 2H, Ar*H*), 6.10 – 6.09 (m, 1H, C=C*H*), 5.46 (d, *J* = 1.8 Hz, 1H, C=C*H*$_2$), 5.42 (d, *J* = 1.8 Hz, 1H, C=C*H*$_2$), 4.15 (q, *J* = 7.1 Hz, 2H, CO_2C*H*$_2CH_3$), 2.76 – 2.72 (m, 2H, C*H*$_2$C=CH), 1.59 – 1.48 (m, 2H, C*H*$_2$), 1.36 – 1.16 (m, 21H, C*H*$_2$ & CO_2CH_2C*H*$_3$), 0.88 (t, *J* = 6.8 Hz, 3H, C*H*$_3$); **^{13}C NMR** (101 MHz, $CDCl_3$) δ 165.6, 164.1, 141.5, 136.2, 133.8,

130.3, 129.1, 128.7, 125.6, 113.5, 90.8, 87.4, 60.3, 32.1, 31.9, 29.79 (2C), 29.77, 29.7, 29.52, 29.49, 29.4, 28.5, 22.8, 14.33, 14.26; **IR** (ν_{max}, cm^{-1}) 2925 (s), 2855 (m), 1746 (s), 1717 (s), 1629 (m), 1603 (m), 1458 (w), 1455 (m), 1370 (w), 1245 (s), 1192 (s), 1174 (s), 1084 (s), 1065 (s), 1027 (m), 881 (m), 707 (s); **HRMS** (ESI/QTOF) m/z: $[M + H]^+$ Calcd for $C_{28}H_{39}O_4^+$ 439.2843; Found 439.2849.

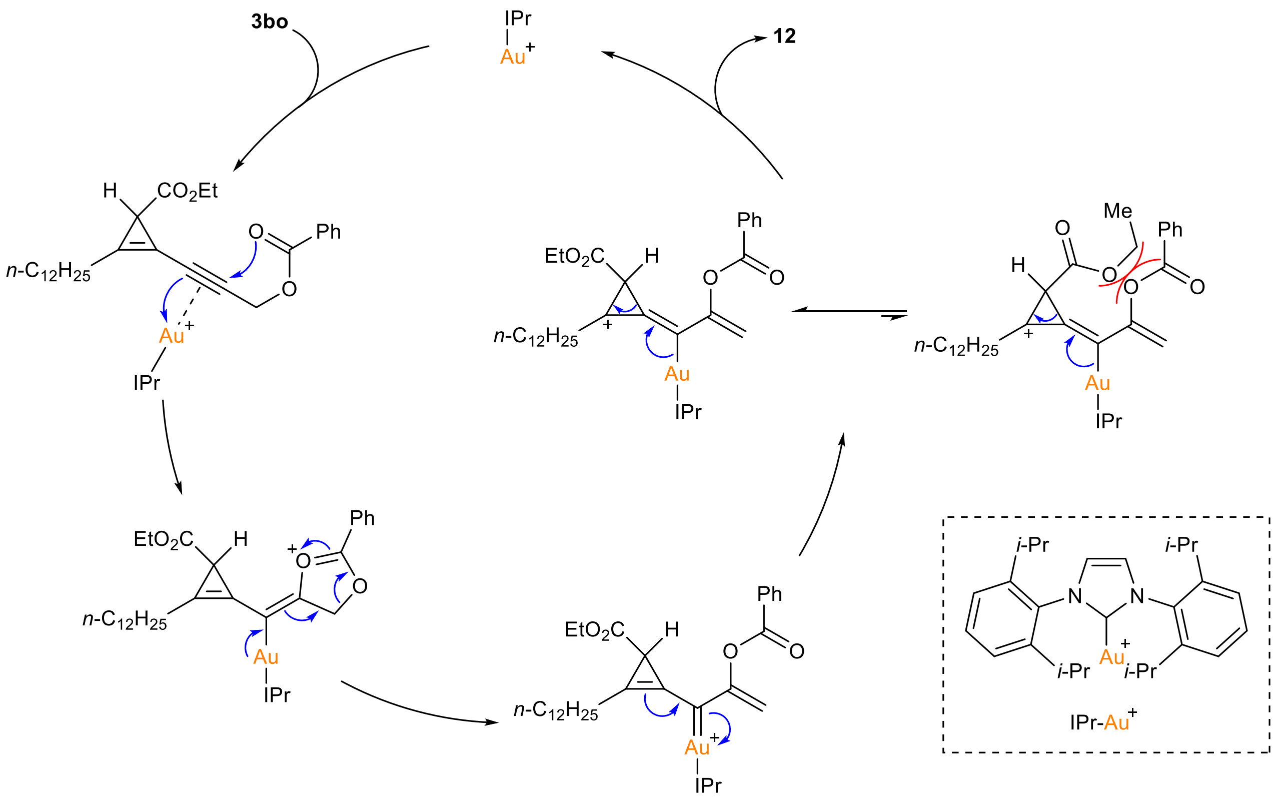

Fig. 1 | Proposed mechanism of gold(I)-catalysed 1,2-benzoyloxy migration of propargylic benzoate and ring-opening cascade.

7.7. Diels–Alder reaction of 3bo with 2,3-dimethylbutadiene[58]

7.7.1. Synthesis and characterization of ethyl 1-(3-(benzoyloxy)prop-1-yn-1-yl)-6-dodecyl-3,4-dimethylbicyclo[4.1.0]hept-3-ene-7-carboxylate (13)

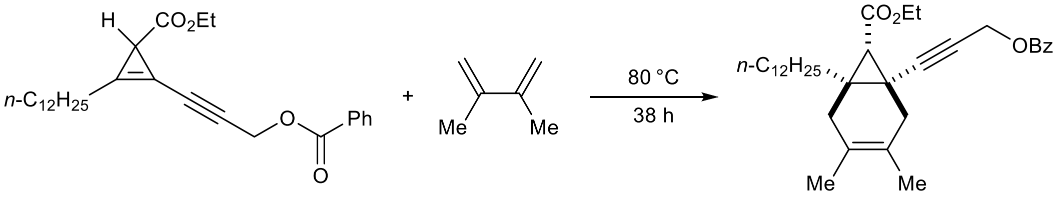

3bo **13**, >20:1 d.r.

A solution of **3bo** (43.9 mg, 0.100 mmol, 1.00 equiv.) in 2,3-dimethylbutadiene (0.50 mL) was heated at 80 °C in a sealed tube for 38 hours. After being cooled to room temperature, the mixture was concentrated and purified by column chromatography on silica gel (eluent: pentane/EtOAc = 10:1) to give the cycloaddition product **13** in 89% yield (46.4 mg, 89.1 μmol, dr. >20:1 based on ^1H NMR) as a colorless oil. **TLC**: R_f (*n*-hexane/EtOAc = 20:1) = 0.27; **^{1}H NMR** (400 MHz, $CDCl_3$) δ 8.13 – 8.00 (m, 2H, Ar*H*), 7.62 – 7.50 (m, 1H, Ar*H*), 7.42 (t, *J* = 7.7 Hz, 2H, Ar*H*), 4.98 (s, 2H, C*H*$_2$OBz), 4.17 – 4.05 (m, 2H, $CO_2CH_2CH_3$), 2.57 – 2.43 (m, 2H, C*H*$_2$), 2.23 (s, 2H, C*H*$_2$), 2.10 – 1.90 (m, 2H, C*H*$_2$), 1.88 (s, 1H, C*H*CO_2), 1.83 – 1.73 (m, 1H, C*H*$_2$), 1.72 – 1.61 (m, 2H, C*H*$_2$), 1.60 – 1.51 (m, 6H, C*H*$_3$), 1.51 – 1.11 (m, 20H, C*H*$_2$ & $CO_2CH_2CH_3$), 0.87 (t, *J* = 6.8 Hz, 3H, C*H*$_3$); **^{13}C NMR** (101 MHz, $CDCl_3$) δ 170.2, 166.0, 133.1, 130.0, 129.9, 128.4, 122.1, 121.5, 87.0, 76.2, 60.2, 53.6, 37.7, 36.0, 34.9, 32.0, 30.6, 30.2, 30.1, 29.9, 29.84, 29.80,

29.78 (2C), 29.5, 26.6, 26.5, 22.8, 19.1, 18.7, 14.5, 14.2; **IR** (ν_{max}, cm^{-1}) 2925 (s), 2855 (m), 2247 (w), 1728 (s), 1602 (w), 1451 (m), 1416 (w), 1372 (m), 1314 (w), 1266 (s), 1148 (s), 1098 (s), 1068 (m), 1027 (w), 957 (w), 711 (s); **HRMS** (ESI/QTOF) m/z: $[M + Na]^+$ Calcd for $C_{34}H_{48}NaO_4^+$ 543.3445; Found 543.3436.

7.8. Desilylation of 3bl using TBAF to terminal cyclopropenyl alkyne 14[59]

7.8.1. Synthesis and characterization of ethyl 2-dodecyl-3-ethynylcycloprop-2-ene-1-carboxylate (14)

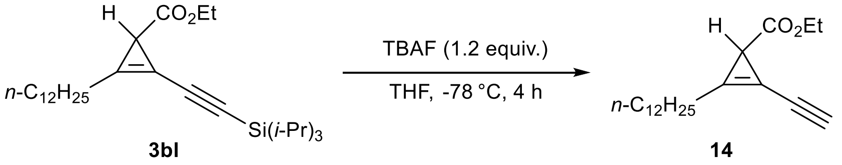

A 25 mL Schlenk tube was charged with **3bl** (286 mg, 0.622 mmol, 1.00 equiv.) and a magnetic stir-bar. The Schlenk tube was then evacuated and backfilled with nitrogen three times. Subsequently, THF (5.0 mL) was added by syringe and the Schlenk tube was placed at −78 °C in a dry ice/acetone bath. After that, tetrabutylammonium fluoride (TBAF, 1.0 M in THF; 0.746 mmol, 0.75 mL, 1.20 equiv.) was added dropwise by a syringe pump over 5 min and the reaction mixture was stirred at −78 °C for additional 4 hours. Then the cooling bath was removed. The reaction mixture was allowed to warm to room temperature gradually (ca. 20 min) while keeping stirring. The reaction mixture was then quenched by adding saturated aqueous NH_4Cl (5.0 mL) and extracted with EtOAc (10 ml × 3). The combined organic layers were successively washed with brine and H_2O, then dried over anhydrous Na_2SO_4, filtered, and concentrated under reduced pressure. The residue was purified by column chromatography on silica gel (eluent: pentane/EtOAc = 20:1) to give the desilylation product **14** in 75% yield (141 mg, 464 μmol) as a colorless oil. **TLC**: R_f (*n*-hexane/EtOAc = 20:1) = 0.38; **^{1}H NMR** (400 MHz, $CDCl_3$) δ 4.21 – 4.10 (m, 2H, $CO_2CH_2CH_3$), 3.49 (s, 1H, C≡C*H*), 2.53 (t, *J* = 7.2 Hz, 2H, CH_2CH_2C), 2.43 (s, 1H, C*H*CO_2), 1.68 – 1.53 (m, 2H, CH_2CH_2C), 1.44 – 1.16 (m, 21H, CH_2 & $CO_2CH_2CH_3$), 0.88 (t, *J* = 6.7 Hz, 3H, CH_3); **^{13}C NMR** (101 MHz, $CDCl_3$) δ 173.9, 118.5, 90.3, 87.7, 71.3, 60.7, 32.1, 29.793, 29.787, 29.7, 29.6, 29.5, 29.4, 29.2, 26.6, 26.1, 25.0, 22.8, 14.5, 14.3; **IR** (ν_{max}, cm^{-1}) 3306 (w), 2950 (m), 2926 (s), 2855 (s), 2114 (w), 1846 (w), 1727 (s), 1465 (w), 1371 (w), 1334 (w), 1252 (w), 1186 (s), 1095 (w), 1028 (w), 722 (w); **HRMS** (ESI/QTOF) m/z: $[M + Na]^+$ Calcd for $C_{20}H_{32}NaO_2^+$ 327.2295; Found 327.2308.

7.9. Gold-catalysed σ-type CPCs transfer reaction for the synthesis of non-symmetrical 1,2-bis-cyclopropenyl substituted alkyne

7.9.1. Synthesis and characterization of dimethyl 2-((2-dodecyl-3-(ethoxycarbonyl)cycloprop-1-en-1-yl)ethynyl)-3-phenylcycloprop-2-ene-1,1-dicarboxylate (15)

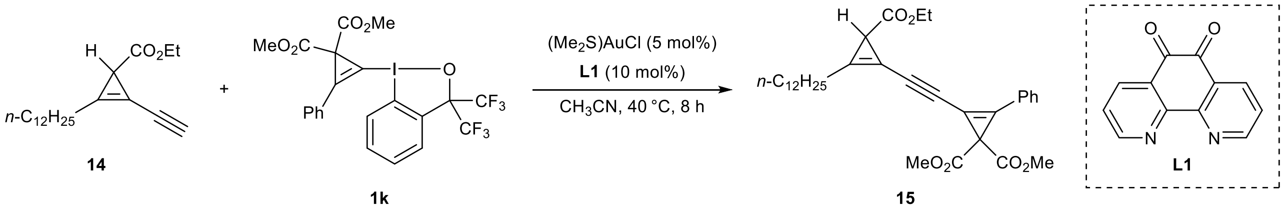

An oven-dried 10 mL Schlenk tube was sequentially charged with **14** (30.5 mg, 0.100 mmol, 1.00 equiv.), **L1** (2.10 mg, 10.0 μmol, 10.0 mol%), $(Me_2S)AuCl$ (1.47 mg, 5.00 μmol, 5.00 mol%), CpBX **1k** (72.0 mg, 0.120 mmol, 1.20 equiv.) and a magnetic stir-bar. The Schlenk tube was then evacuated and backfilled with nitrogen three times. Subsequently, CH_3CN (0.05 M; 2.0 mL) was added by syringe. The reaction mixture was stirred at 40 °C for 8 hours. The resulting reaction mixture was then filtered

through a silica gel pad and eluted with CH_2Cl_2 (3 × 5.0 mL). The solvent was removed under reduced pressure, and the resulting crude residue was purified by flash column chromatography on silica gel (eluent: pentane/EtOAc = 5:1) to give the cross-coupled product **15** in 60% yield (32.2 mg, 60.2 μmol) as a colorless oil. The by-product **4** was recovered in 99% NMR yield based on **1k**. **TLC**: R_f (*n*-hexane/EtOAc = 4:1) = 0.40; **^{1}H NMR** (400 MHz, $CDCl_3$) δ 7.72 – 7.60 (m, 2H, Ar*H*), 7.47 – 7.42 (m, 3H, Ar*H*), 4.27 – 4.08 (m, 2H, $CO_2CH_2CH_3$), 3.75 (s, 6H, CO_2CH_3), 2.62 (t, *J* = 7.3 Hz, 2H, CH_2CH_2C), 2.57 (s, 1H, $CHCO_2$), 1.70 – 1.56 (m, 2H, CH_2CH_2C), 1.48 – 1.16 (m, 21H, CH_2 & $CO_2CH_2CH_3$), 0.87 (t, *J* = 6.8 Hz, 3H, CH_3); **^{13}C NMR** (101 MHz, $CDCl_3$) δ 173.5, 169.3, 131.3, 130.8, 129.2, 124.4, 122.3, 112.5, 90.8, 90.0, 89.5, 84.6, 60.9, 52.8, 37.2, 32.0, 29.78, 29.76 (2C), 29.6, 29.5, 29.4, 29.3, 26.6, 26.5, 26.1, 22.8, 14.5, 14.2; **IR** (ν_{max}, cm^{-1}) 2946 (m), 2928 (s), 2856 (m), 2189 (w), 1873 (w), 1732 (s), 1602 (w), 1436 (m), 1369 (w), 1281 (m), 1245 (s), 1184 (s), 1063 (m), 1031 (w), 763 (w); **HRMS** (ESI/QTOF) m/z: $[M + Na]^+$ Calcd for $C_{33}H_{42}NaO_6^+$ 557.2874; Found 557.2876.

7.10. Au-Ag bimetallic catalysis providing unsymmetrical cyclopropenyl 1,3-diyne[60]

7.10.1. Synthesis and characterization of ethyl 2-dodecyl-3-((triisopropylsilyl)buta-1,3-diyn-1-yl)cycloprop-2-ene-1-carboxylate (16)

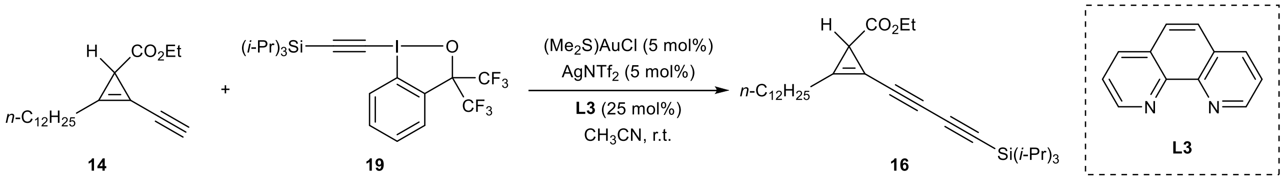

An oven-dried 10 mL Schlenk tube was sequentially charged with **14** (30.5 mg, 0.100 mmol, 1.00 equiv.), **L3** (4.51 mg, 25.0 μmol, 25.0 mol%), $(Me_2S)AuCl$ (1.47 mg, 5.00 μmol, 5.00 mol%), **19** (55.0 mg, 0.100 mmol, 1.00 equiv.) and a magnetic stir-bar. The Schlenk tube was then evacuated and backfilled with nitrogen three times. Subsequently, $AgNTf_2$ (1.90 mg, 5.00 μmol, 5.00 mol%) and CH_3CN (0.05 M; 2.0 mL) was added. The reaction mixture was stirred at room temperature for 1 hour. The resulting reaction mixture was then filtered through a silica gel pad and eluted with CH_2Cl_2 (3 × 5 mL). The solvent was removed under reduced pressure, and the resulting crude residue was purified by flash column chromatography on silica gel (eluent: pentane/EtOAc = 20:1) to give the cross-coupled product **16** in 86% yield (41.9 mg, 86.4 μmol) as a colorless oil. **TLC**: R_f (*n*-hexane/EtOAc = 20:1) = 0.45; **^{1}H NMR** (400 MHz, $CDCl_3$) δ 4.22 – 4.06 (m, 2H, $CO_2CH_2CH_3$), 2.54 (t, *J* = 7.3 Hz, 2H, CH_2CH_2C), 2.48 (s, 1H, $CHCO_2$), 1.66 – 1.53 (m, 2H, CH_2CH_2C), 1.40 – 1.19 (m, 21H, CH_2 & $CO_2CH_2CH_3$), 1.14 – 0.97 (m, 21H, Si(*i*-Pr)$_3$), 0.88 (t, *J* = 6.8 Hz, 3H, CH_3); **^{13}C NMR** (101 MHz, $CDCl_3$) δ 173.4, 120.7, 92.1, 90.2, 89.1, 84.4, 62.7, 60.8, 32.1, 29.79, 29.77, 29.7, 29.6, 29.5, 29.3, 29.2, 26.6 (2C), 25.9, 22.8, 18.7, 14.5, 14.3, 11.4; **IR** (ν_{max}, cm^{-1}) 2952 (s), 2927 (s), 2859 (s), 2189 (w), 2102 (w), 1847 (w), 1730 (s), 1465 (m), 1387 (w), 1368 (w), 1333 (m), 1249 (m), 1181 (s), 1095 (w), 1076 (w), 1018 (m), 998 (m), 892 (w); **HRMS** (ESI/QTOF) m/z: $[M + H]^+$ Calcd for $C_{31}H_{53}O_2Si^+$ 485.3809; Found 485.3820.

7.11. Copper(I)-catalysed alkyne-azide cycloaddition of 14 and benzyl azide[61]

7.11.1. Synthesis and characterization of ethyl 2-(1-benzyl-1*H*-1,2,3-triazol-4-yl)-3-dodecylcycloprop-2-ene-1-carboxylate (17)

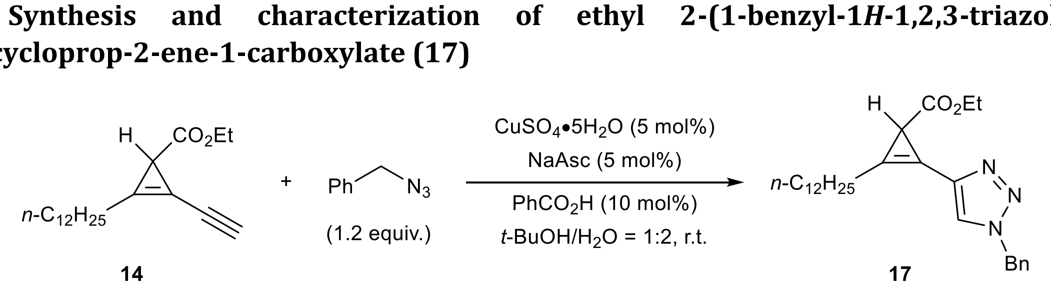

An oven-dried 10 mL Schlenk tube was sequentially charged with **14** (30.5 mg, 0.100 mmol, 1.00 equiv.), $CuSO_4 \cdot 5H_2O$ (1.25 mg, 5.00 μmol, 5.00 mol%), sodium ascorbate (NaAsc; 0.99 mg, 5.00 μmol, 5.00 mol%), $PhCO_2H$ (1.22 mg, 0.100 mmol, 10.0 mol%) and a magnetic stir-bar. The Schlenk tube was then evacuated and backfilled with nitrogen three times. Subsequently, *t*-BuOH (1.0 mL), deionized water (2.0 mL) and benzyl azide (16.0 mg, 0.120 mmol, 1.20 equiv.) was added. The reaction mixture was stirred at room temperature for 2 hours. The resulting reaction mixture was then diluted by adding CH_2Cl_2 (2.0 mL) and further extracted with CH_2Cl_2 (5.0 ml × 3). The combined organic layers were successively washed with brine and H_2O, then dried over anhydrous Na_2SO_4, filtered, and concentrated under reduced pressure. The residue was purified by column chromatography on silica gel (eluent: pentane/EtOAc = 3:1) to give the cycloaddition product **17** in 72% yield (31.5 mg, 72.0 μmol) as a colorless oil. **TLC**: R_f (*n*-hexane/EtOAc = 4:1) = 0.22; **^{1}H NMR** (400 MHz, $CDCl_3$) δ 7.44 (s, 1H, triazole), 7.33 – 7.27 (m, 3H, Ar*H*), 7.25 – 7.14 (m, 2H, Ar*H*), 5.54 – 5.37 (m, 2H, NCH_2Ph), 4.08 – 4.00 (m, 2H, $CO_2CH_2CH_3$), 2.57 (t, *J* = 7.4 Hz, 2H, CH_2CH_2C), 2.38 (s, 1H, $CHCO_2$), 1.65 – 1.57 (m, 2H, CH_2CH_2C), 1.41 – 1.06 (m, 21H, CH_2 & $CO_2CH_2CH_3$), 0.80 (t, *J* = 6.8 Hz, 3H, CH_3); **^{13}C NMR** (101 MHz, $CDCl_3$) δ 175.4, 137.2, 134.3, 129.3, 129.0, 128.3, 123.5, 111.3, 95.2, 60.4, 54.4, 32.0, 29.8, 29.7 (2C), 29.6, 29.5, 29.4 (2C), 27.1, 25.4, 22.8, 22.3, 14.4, 14.2; **IR** (ν_{max}, cm^{-1}) 3133 (w), 2925 (s), 2852 (m), 1897 (w), 1721 (s), 1459 (m), 1369 (w), 1335 (w), 1249 (m), 1184 (s), 1039 (m), 1004 (w), 802 (w), 723 (s); **HRMS** (ESI/QTOF) m/z: $[M + H]^+$ Calcd for $C_{27}H_{40}N_3O_2^+$ 438.3115; Found 438.3120.

8. Mechanistic investigations

8.1. Preparation of potential gold catalysts

8.1.1. Preparation of the ligand-free polymeric gold(I)-phenylacetylide (20)[62]

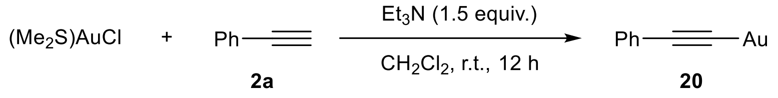

A 10 mL Schlenk tube containing a stirring bar was charged with $(Me_2S)AuCl$ (147 mg, 0.500 mmol, 1.00 equiv.), dichloromethane (3.0 mL) and phenylacetylene **2a** (55 μL, 0.500 mmol, 1.00 equiv.) under air, sequentially. While stirring, Et_3N (104 μL, 0.750 mmol, 1.50 equiv.) was added dropwise, which resulted in the immediate formation of a yellow precipitate. After stirring for 12 hours at room temperature, the precipitate was filtered, washed with dichloromethane and dried under vacuum to give **20** in 66% yield (99.0 mg, 332 μmol) as a grey solid. **Anal**. Calcd for C_8H_5Au: C, 32.23; H, 1.69; N, 0.00; S, 0.00. Found: C, 31.69; H, 1.65; N, 0.03; S, 0.00.

8.1.2. Preparation of cationic gold(I)-ethylene complex (21)[63]

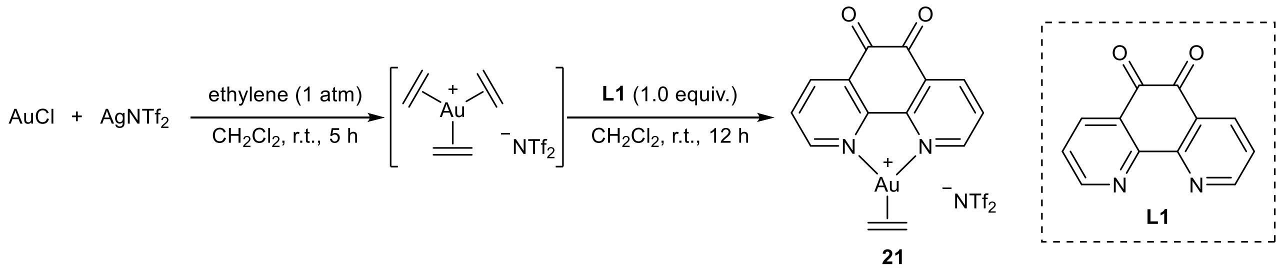

In a nitrogen-filled glovebox, a 100 ml Schlenk tube was sequentially charged with AuCl (93.0 mg, 0.400 mmol, 1.00 equiv.) and a magnetic stir bar then wrapped with foil to protect the contents from light. The Schlenk tube was then sealed with a rubber cap and brought out of the glovebox. Subsequently, the Schlenk tube was evacuated and backfilled with ethylene gas for three times by a balloon filled with ethylene (ca. 1 atm.). To the Schlenk tube was added dry CH_2Cl_2 (2.0 mL). The resulting solution was stirred for 5 min at room temperature. After that, a solution of $AgNTf_2$ (155 mg,

0.400 mmol, 1.00 equiv.) in CH_2Cl_2 (25 mL) was added via a syringe pump over 30 min. After the addition was complete, the mixture was stirred for additional 4.5 hours at room temperature. The resulting suspension[64] was filtered through a syringe filter (membrane-⌀: 25 mm; pore size: 0.2 μm). The filtrate was directly introduced into another 100 ml Schlenk tube (wrapped with aluminium foil and filled with nitrogen) charged with **L1** (84.1 mg, 0.400 mmol, 1.00 equiv.) and a magnetic stir bar. The resulting mixture was stirred at room temperature for 12 hours and then filtered through a syringe filter (membrane-⌀: 25 mm; pore size: 0.2 μm). The filtrate was directly introduced into a third 100 ml Schlenk tube (wrapped with aluminium foil and filled with nitrogen). Dry Et_2O (25 mL) was added slowly as a layer. The Schlenk tube was kept standing at room temperature with a balloon filled with nitrogen for 24 h, which gave a crystalline solid. After decanting the solvent, the solid was washed with Et_2O (3 × 10 mL) and dried in vacuo to afford the gold(I)-ethylene complex **21** (107 mg, 149 μmol, 37% yield) as a light-yellow crystal. **^{1}H NMR** (400 MHz, CD_2Cl_2) δ 9.09 (d, *J* = 4.9 Hz, 2H, Ar*H*), 8.83 (dd, *J* = 8.0, 1.6 Hz, 2H, Ar*H*), 8.06 (dd, *J* = 8.0, 5.2 Hz, 2H, Ar*H*), 4.01 (s, 4H, C*H*$_2$=C*H*$_2$); **^{13}C NMR** (101 MHz, CD_2Cl_2) δ 175.2, 156.8, 151.2, 141.1, 130.2, 129.8, 120.0 (q, *J* = 321.6 Hz), 65.0; **^{19}F NMR** (376 MHz, CD_2Cl_2) δ -79.3; **IR** (ν_{max}, cm^{-1}) 3100 (w), 2982 (w), 2939 (w), 2888 (w), 1701 (m), 1577 (m), 1478 (w), 1432 (w), 1350 (s), 1335 (m), 1300 (w), 1194 (s), 1136 (m), 1053 (m), 934 (w), 818 (w), 791 (w), 734 (m), 710 (w); **HRMS** (ESI$^+$) m/z Calcd. for $C_{14}H_{10}AuN_2O_2$ $[M-NTf_2]^+$ 435.0402, found 435.0413; **Anal**. Calcd for $C_{12}H_6N_2O_2$ • $C_2AuF_6NO_4S_2$ • C_2H_4: C, 26.86; H, 1.41; N, 5.87; S, 8.96. Found: C, 28.09; H, 1.47; N, 6.14; S, 8.64.

8.2. Control experiments for determining the catalytically active species

An oven-dried 10 mL Schlenk tube was sequentially charged with gold catalyst [$(Me_2S)AuCl$, **20** or **21**; 5.00 μmol, 5.00 mol%], **L1** (if used; 2.10 mg, 10.0 μmol, 10.0 mol%) or additive (if used; NBu_4X, X = Cl, F, Br, I; 5.00 μmol, 5.00 mol%), $NaHCO_3$ (if used; 25.2 mg, 0.300 mmol, 3.00 equiv.), CpBX **1a** (56.4 mg, 0.100 mmol, 1.00 equiv.) and a magnetic stir-bar. The Schlenk tube was then evacuated and backfilled with nitrogen three times. Subsequently, CH_3CN (0.10 M; 1.0 mL) and terminal alkyne **2a** (11 μL, 0.100 mmol, 1.00 equiv.) were added by syringe. The reaction mixture was stirred at room temperature for 2 hours (unless otherwise noted). The resulting reaction mixture was diluted with CH_2Cl_2 and filtered through a short pad of silica gel by eluting with CH_2Cl_2 (3 × 5.0 mL). The filtrate was then concentrated to dryness and the residue was subjected to flash column chromatography on silica gel (eluent: pentane/EtOAc = 20:1 to 5:1). The fractions that contained the product **3a**, **4** and the remained CpBX **1a** were collected and concentrated by rotary evaporation. The yields of **3a**, **4** and the recoveries of **1a** were obtained by quantitative ^{1}H NMR analysis using CH_2Br_2 (^{1}H NMR δ 4.92) as the internal standard.

Table 7 | Control experiments for determining the catalytically active species

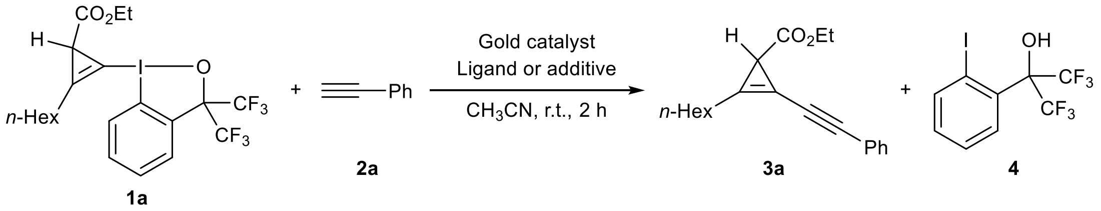

Entry	Gold catalyst (5 mol%)	Ligand or additive (mol%)	Yield of **3a** (%)[a]	Recovery of **1a** (%)[a]	Yield of **4** (%)[a]
1[b]	$(Me_2S)AuCl$	**L1** (10)	96	0	99
2	**20**	**L1** (10)	0	97	2
3[c]	**20**	**L1** (10)	7	87	9
4	**21**	none	10	84	13
5[d]	**21**	none	57	0	98
6	**21**	NBu_4Cl (5)	94	0	99
7	**21**	$NaHCO_3$[e]	10	78	20
8	**21**	NBu_4Br (5)	90	4	95
9	**21**	NBu_4F (5)	0	91	5
10	**21**	NBu_4I (5)	2	94	6

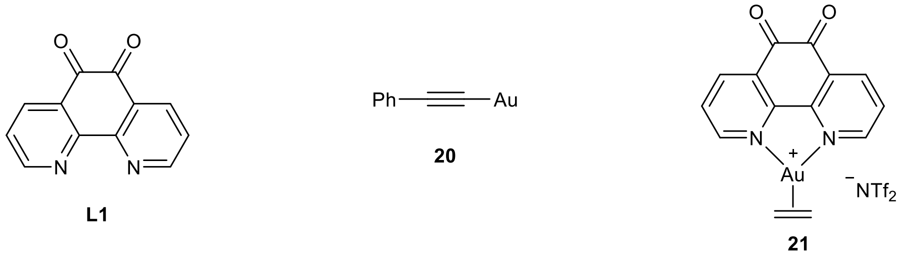

Reactions performed on a 100 μmol scale. [a]Yields or recovery were determined by ^1H NMR spectroscopy using dibromomethane as the internal standard. [b]Standard condition. [c]Reaction carried out at 40 °C for 24 h. [d]Reaction carried out at 40 °C for 10 h. [e]$NaHCO_3$ (3.00 equiv.) was used.

These results disfavour a catalytic cycle involving a direct oxidation of gold(I)-acetylide by CpBX, and cationic gold(I) species are competent in catalysing the reaction.

8.3. Stoichiometric reaction of cationic gold(I)-ethylene complex 21 and terminal alkyne 2n

To a 1.5 mL dry vial were sequentially added 1,3,5-tris(trifluoromethyl)benzene (internal standard) and 1-ethynyl-4-(trifluoromethoxy)benzene **2n** (1.00 or 20.0 equiv.). The vial was introduced into a nitrogen-filled glovebox. Then, the cationic gold(I)-ethylene complex **21** (1.00 equiv.) and CD_3CN (0.75 mL) was added. The vial was capped and shaken violently for 30 seconds. The resulting solution was transferred into a J. Young NMR tube and subjected to NMR spectroscopy analysis immediately. The yields of the proposed **21-alkyne** (δ –58.5 ppm) were determined by ^{19}F NMR analysis.

Table 8 | Generation of L1-ligated gold(I)-acetylide from cationic gold(I) species and alkyne

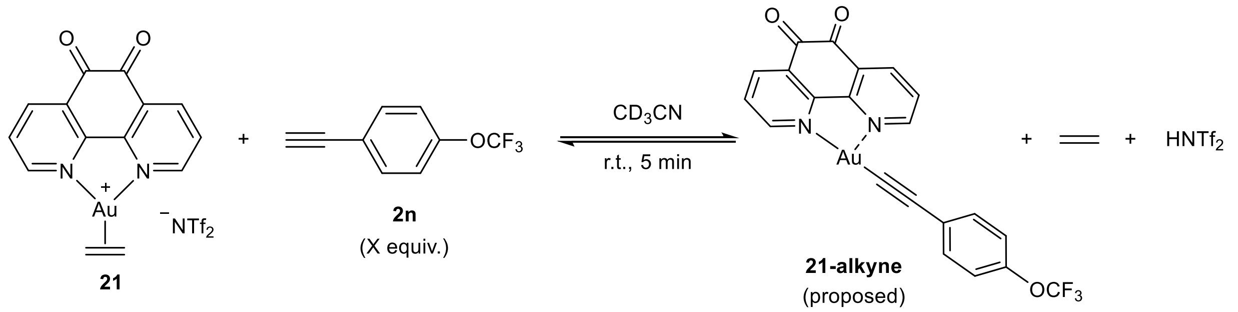

Entry	Reaction scale (μmol)	Initial concentration of **21** (mM)	Equiv. of **2n**	Yield of **21-alkyne** (proposed) (%)[a]
1	10	13.33	1.0	15
2	10	13.33	20.0	50
3	3.75	5	20.0	39
4	1.875	2.5	20.0	35

[a]Yields were determined by ^{19}F NMR spectroscopy.

The above experimental results showed that increasing the equivalent of terminal alkyne **2n** or the initial concentration of cationic gold(I)-ethylene complex **21** can push the equilibrium forward, thus affording higher yield of the proposed **L1**-ligated gold(I)-acetylide **21-alkyne**. Especially, the experimental result showed in entry 3 (Table 8) mimics the reaction condition in Table 7, entry 4. Both reaction conditions (Table 7, entry 4 and Table 8, entry 3) exhibited the same stoichiometric ratio of **21** and the terminal alkyne, as well as the same initial concentration of **21**. Therefore, it is reasonable to postulate that near 40% of the cationic gold(I)-ethylene complex **21** turned into the **L1**-ligated gold(I)-acetylide **21-alkyne** during the first 5 minutes for the reaction showed in Table 7, entry 4. However, extremely low yield of the desired cross-coupled product **3a** was observed and most of the oxidant **1a** was recovered in this case, which indicated that the **L1**-ligated gold(I)-acetylide **21-alkyne** is a catalytically inert species towards the oxidation of **1a**. Thus, gold(I)-acetylide is less likely involved in the productive catalytic cycle. The in-situ formation of the catalytically inactive species, i.e. gold(I)-acetylide, explained the low catalytic activity of cationic gold(I)-ethylene complex **21** towards the coupling reaction.

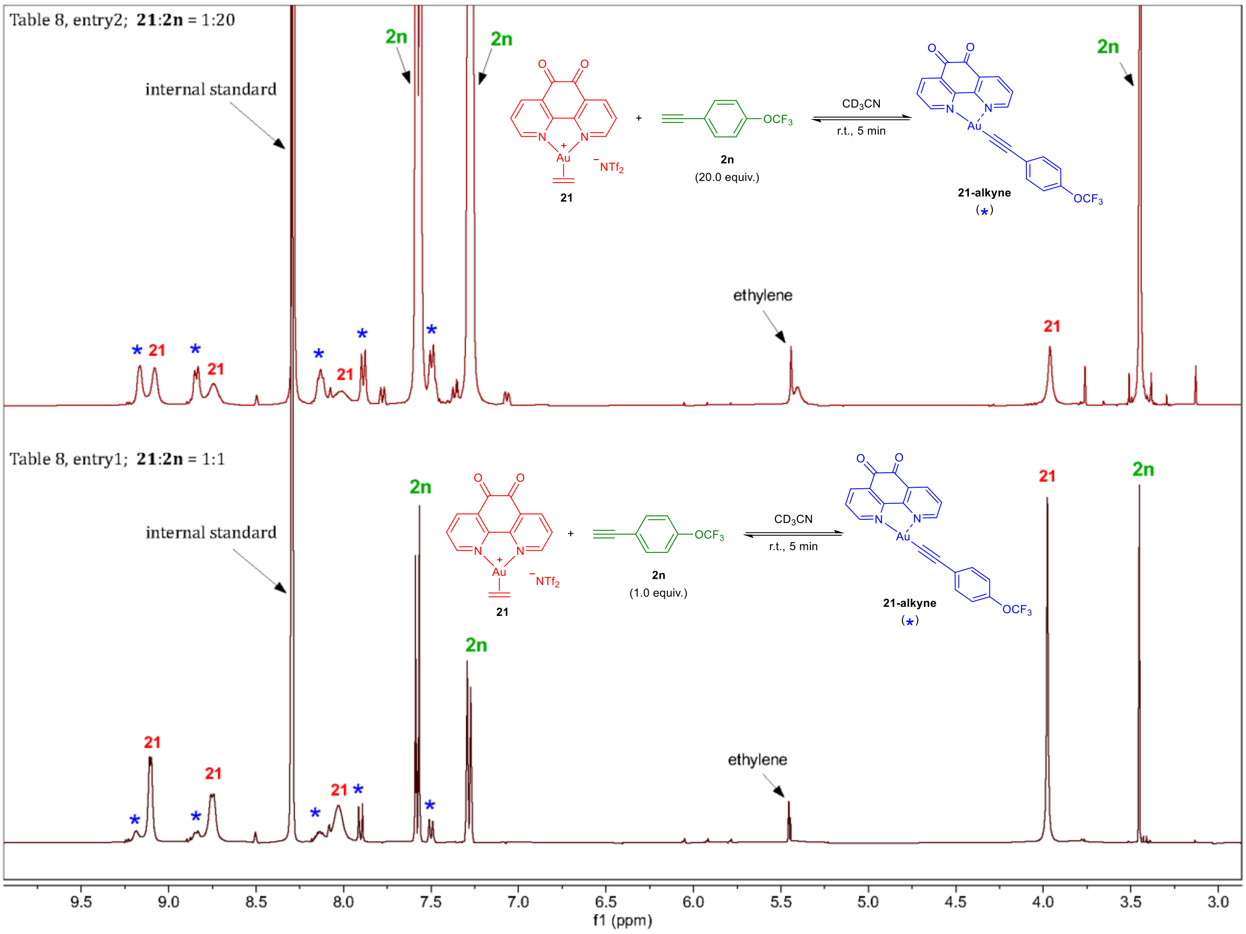

Fig. 2 | Stacked ^{1}H NMR spectra (CD_3CN, 25 °C) of stoichiometry study. The bottom ^{1}H NMR spectrum revealed the experiment showed in Table 8, entry 1; the top ^{1}H NMR spectrum revealed the experiment showed in Table 8, entry 2. The stacked spectra showed clearly that increasing the equivalent of **2n** led an increased consumption of cationic gold(I)-ethylene complex **21**, and gave an increased yield of the proposed **L1**-ligated gold(I)-acetylide **21-alkyne**. The ^{1}H NMR signals assigned to **21-alkyne** has been labelled with asterisk (*) in blue.

8.4. Generation of L1-ligated AuCl 22 from cationic gold(I)-ethylene complex 21 and Bu_4NCl

To a 1.5 mL dry vial were added tetrabutylammonium chloride (Bu_4NCl; 1.39 mg, 5.00 μmol, 1.00 equiv.). The vial was introduced into a nitrogen-filled glovebox. Then, the cationic gold(I)-ethylene complex **21** (3.6 mg, 5.00 μmol, 1.00 equiv.) and CD_2Cl_2 (0.75 mL) were sequentially added. The vial was capped and shaken violently for 20 seconds. The resulting solution was transferred into a J. Young NMR tube and subjected to NMR spectroscopy analysis immediately.

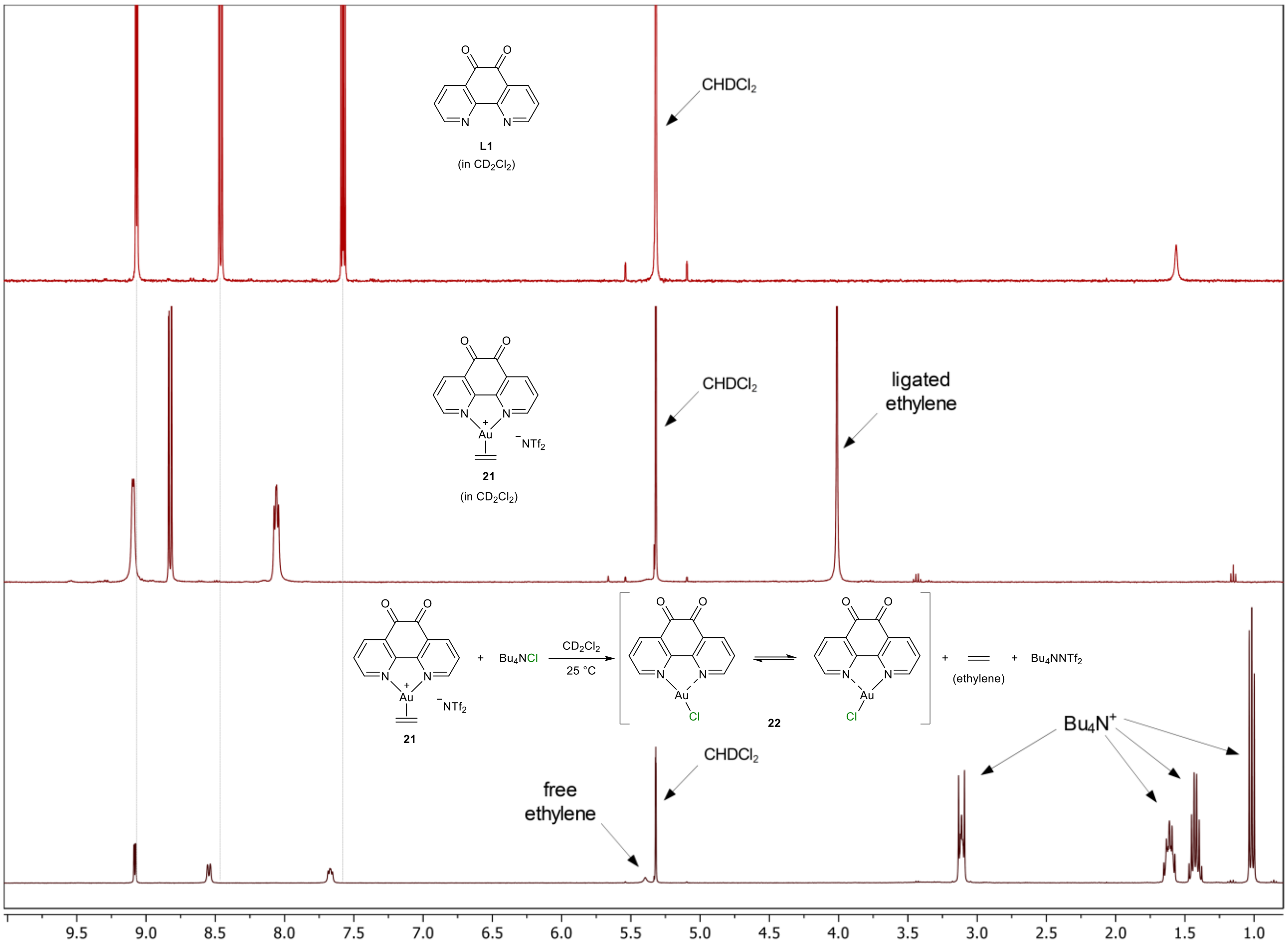

Fig. 3 | Stacked ^{1}H NMR spectra of equimolar mixture of 21 and NBu_4Cl in CD_2Cl_2 at 25 °C (bottom), ^{1}H NMR spectra of the authentic sample of L1 (top) and the cationic gold(I)-ethylene complex 21 (middle) in CD_2Cl_2 at 25 °C for reference. The bottom ^{1}H NMR spectrum showed that upon mixing cationic gold(I)-ethylene complex **21** and NBu_4Cl in CD_2Cl_2, a new set of proton signals derived from the ligand **L1** arose and the signals assigned to the free ethylene were also observed. Such results suggest that the ethylene ligand can be readily replaced by the σ-donor ligand, i.e. chloride irreversibly. The new proton signals (δ 9.2 – 7.5 ppm) can be putatively assigned to the **L1**-ligated AuCl **22**, which was envisaged to be a fluxional species in which the coordination site of gold(I) rapidly exchanges between the two nitrogen atoms of **L1**. **^{1}H NMR** of **22** (400 MHz, CD_2Cl_2) δ 9.08 (dd, *J* = 4.8, 1.8 Hz, 2H, Ar*H*), 8.55 (d, *J* = 7.9 Hz, 2H, Ar*H*), 7.67 (dd, *J* = 7.9, 4.8 Hz, 2H, Ar*H*).

8.5. Stoichiometric reaction of CpBX 1l and chloride-supported gold(I) catalyst

To a 1.5 mL dry vial were sequentially added 1,3,5-tris(trifluoromethyl)benzene (internal standard; 28.2 mg, 100 μmol, 5.00 equiv.), **L1** (4.20 mg, 20.0 μmol, 1.00 equiv.), $(Me_2S)AuCl$ (5.89 mg, 20.0 μmol, 1.00 equiv.) and **1l** (12.4 mg, 20.0 μmol, 1.00 equiv.). The vial was introduced into a nitrogen-filled glovebox and CD_3CN (0.75 mL) was then added. The vial was capped and shaken violently for 20 seconds. The resulting solution was transferred into a J. Young NMR tube and subjected to NMR spectroscopy analysis immediately. The reaction was then monitored by NMR at 25 °C with a duration of ca. 5 hours. The conversion of **1a** and the yield of the homo-coupled product **23** were determined by ^{19}F NMR analysis.

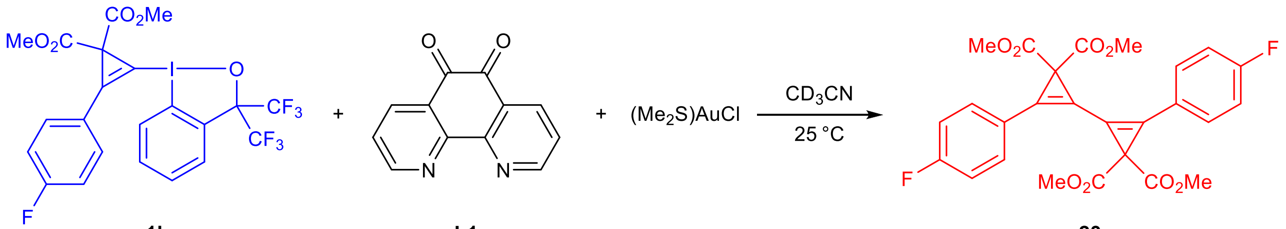

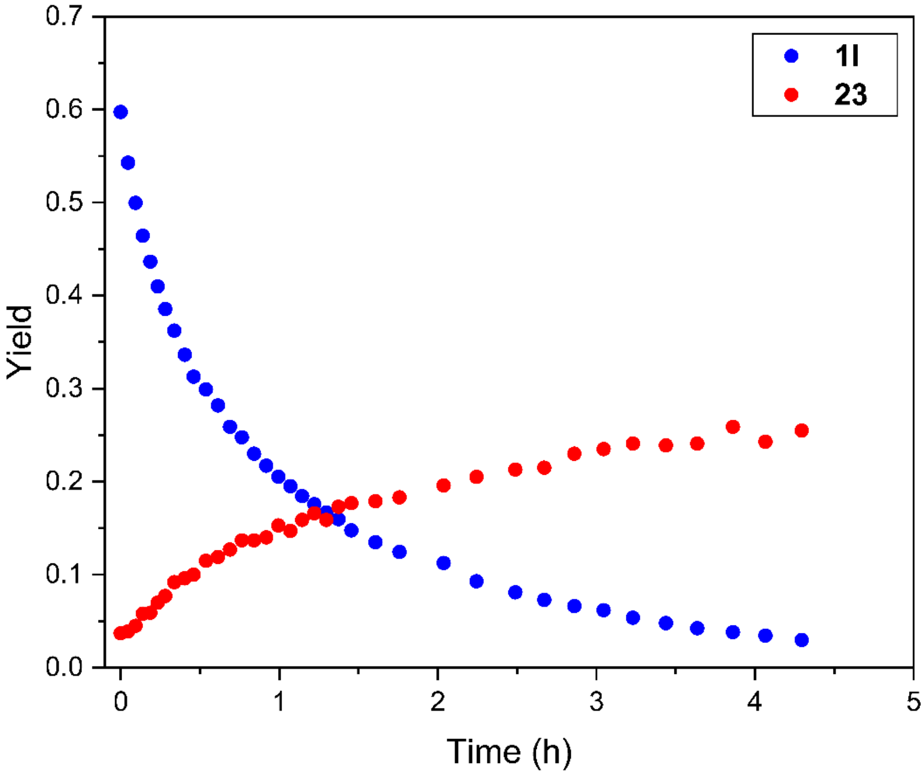

Fig. 4 | Reaction profile of the stoichiometric reaction of CpBX 1l and (Me_2S)AuCl with L1 as the ligand in CD_3CN monitored by ^{19}F NMR at 25 °C. This stoichiometric reaction of CpBX **1l** and chloride-supported gold(I) catalyst clearly showed that the CpBX reagent can oxidize the gold(I) complex efficiently as proved by the full conversion of the reagent **1l** with the presence of the gold(I) species. The formation of the homo-coupled product **23** also indicated the involvement of an oxidation event (*vide infra*).

Upon the NMR monitoring experiment was done, the NMR sample was recovered and eluted with DCM. The resulting solution was filtered through a silica gel pad and eluted with CH_2Cl_2 (3 × 5.0 mL). The solvent was removed under reduced pressure, and the resulting crude residue was purified by flash column chromatography on silica gel (eluent: pentane/EtOAc = 3:1) to give the homo-coupled product **23** in 18% yield (0.90 mg, 1.81 μmol) as a colorless oil. **TLC**: R_f (*n*-hexane/EtOAc = 4:1) = 0.15; **^{1}H NMR** (400 MHz, CD_3CN) δ 7.81 – 7.78 (m, 4H, Ar*H*), 7.34 – 7.30 (m, 4H, Ar*H*), 3.71 (s, 12H, CO_2CH_3); **^{13}C NMR** (101 MHz, CD_3CN) δ 170.3, 165.5 (d, *J* = 252.5 Hz), 134.3 (d, *J* = 9.2 Hz), 121.7 (d, *J* = 3.3 Hz), 117.8 (d, *J* = 22.8 Hz), 112.5, 93.8 (d, *J* = 2.9 Hz), 53.4, 36.6; **^{19}F NMR** (376 MHz, CD_3CN) δ -108.0; **IR** (ν_{max}, cm^{-1}) 2955 (m), 2921 (s), 2851 (s), 1800 (w), 1740 (s), 1598 (m), 1502 (m), 1461 (m), 1436 (w), 1377 (w), 1287 (s), 1236 (s), 1187 (w), 1155 (m), 1067 (s), 925 (w), 842 (m); **HRMS** (ESI/QTOF) m/z: $[M + Na]^+$ Calcd for $C_{26}H_{20}F_2NaO_8^+$ 521.1018; Found 521.1022.

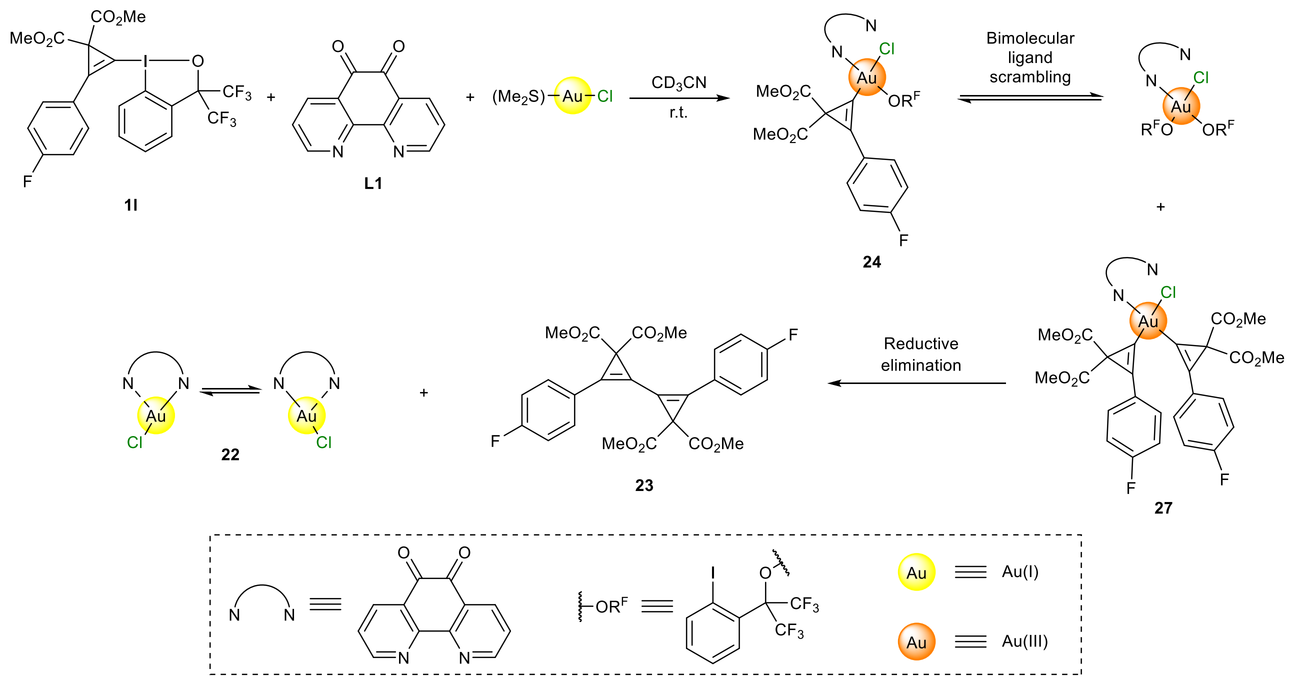

Fig. 5 | Proposed mechanism for the formation of homo-coupled product 23 by direct oxidation of gold(I) complex using CpBX. The homo-coupled product **23** can be generated by the reduction elimination of Au(III) species **27**, which can be derived from the bimolecular ligand scrambling of Au(III) species **24**.

8.6. Tandem mass spectrometry (ESI-MS/MS) analysis for the capture of active intermediates

8.6.1. General remarks

Samples were analyzed using a Waters Acquity-I-UPLC Class system (Waters Corporation, Milford, MA, USA), coupled with a Waters Vion IMS-QTof Mass Spectrometer equipped with LockSpray (Leucine-enkephalin, 200 pg/μL). The instrument was controlled by Waters UNIFI 1.9.4 (3.1.0, Waters Corporation, Milford, MA, USA). The injection volume was 5 μL, and the instrument was operated in positive polarity sensitivity mode (with a resolution of 33,000 FWHM at 556.2766 m/z). Data was acquired in HDMSe mode with a scan time of 0.036 seconds, and the recorded mass range was from 50 to 1200 m/z for both low and high energy spectra. The collision energy was ramped from 20 to 40 V, while the cone voltage was set to 30 V, capillary voltage was set to 3 kV, and the source offset was set to 50 V. The source temperature was set to 120°C, and the desolvation temperature was set to 500°C. The cone gas flow rate was set to 50 L/h, and the desolvation gas flow rate was set to 1000 L/h.

MS/MS experiments were performed on an LTQ Orbitrap FTMS instrument (LTQ Orbitrap Elite FTMS, Thermo Scientific, Bremen, Germany) operated in positive mode, coupled with a robotic chip-based nano-ESI source (TriVersa Nanomate, Advion Biosciences, Ithaca, NY, U.S.A.). A standard data acquisition and instrument control system were utilized (Thermo Scientific), while the ion source was controlled by Chipsoft 8.3.1 software (Advion BioScience). Samples were loaded onto a 96-well plate (Eppendorf, Hamburg, Germany) with an injection volume of 5 μL. The experimental conditions for the ionization voltage were +1.4 kV, and the gas pressure was set at 0.30 psi. The temperature of the ion transfer capillary was 120°C. FTMS spectra were obtained in the 80-1000 m/z range in the reduced profile mode, with a resolution set to 120,000. In all spectra, one microscan was acquired with a maximum injection time value of 1000 ms. The isolation window was set at 5 Da, and the NCE was typically between 20-24.

8.6.2. Tandem mass spectrometry for determining the intermediacy of Au(III) species 24

To a 1.5 mL dry vial were sequentially added **L1** (0.42 mg, 2.00 μmol, 1.00 equiv.), (Me_2S)AuCl (0.59 mg, 2.00 μmol, 1.00 equiv.) and **1l** (1.24 mg, 2.00 μmol, 1.00 equiv.). The vial was introduced into a nitrogen-filled glovebox and CH_3CN (1.0 mL) was then added. The vial was capped and shaken violently for 30 seconds. The resulting solution was subjected to tandem mass spectrometry (ESI-MS/MS) analysis in 5 min.

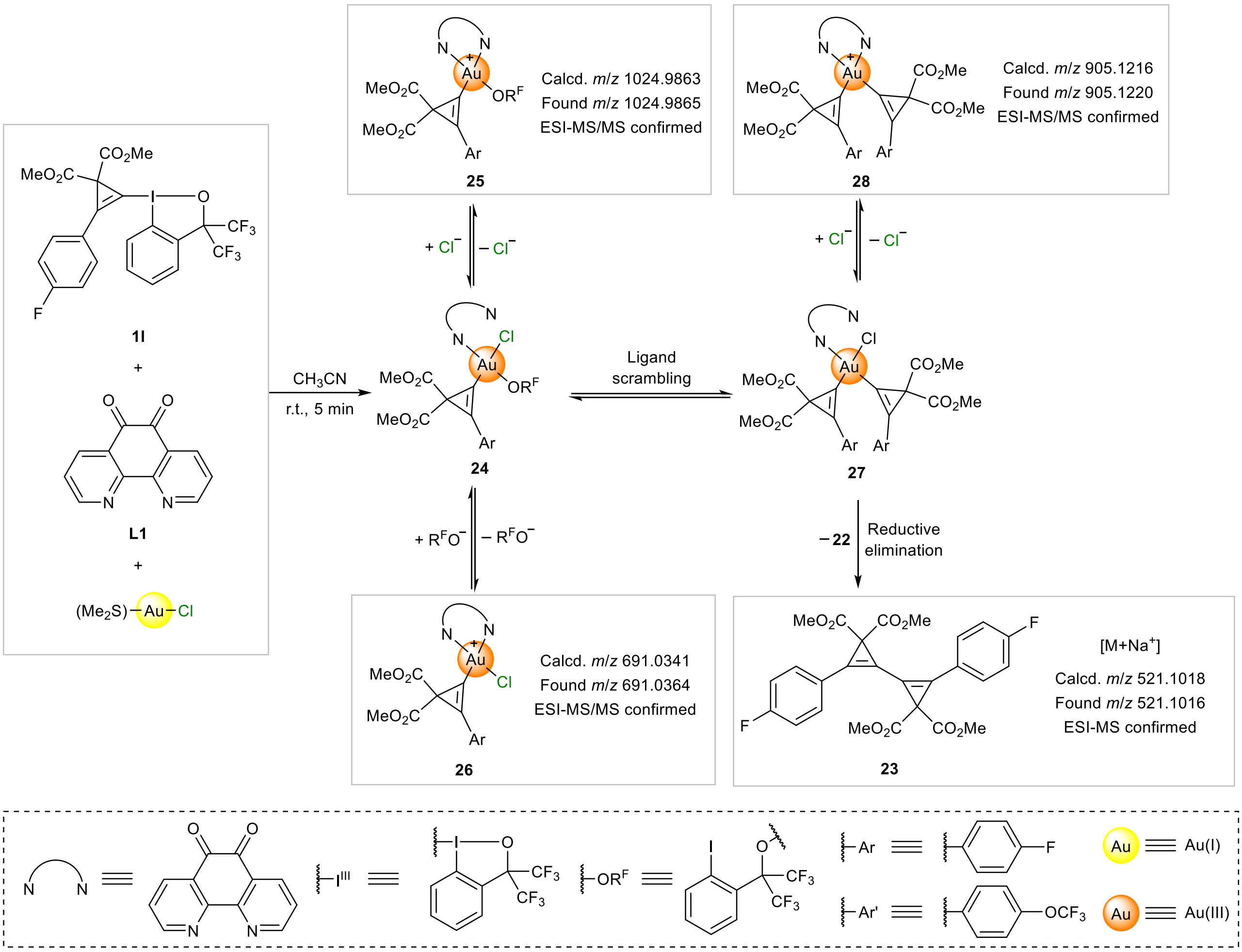

Fig. 6 | Tandem mass spectrometry (ESI-MS/MS) analysis of the stoichiometric reaction of CpBX 1l, (Me_2S)AuCl and L1 determined the formation of intermediate 24. Although **24** was not observed directly by mass spectrometry due to its electroneutral nature, cationic Au(III) species **25** and **26** derived from **24** by losing one anionic fragment were both observed by ESI-MS and structurally confirmed by tandem mass spectrometry (MS/MS) (*vide infra*). Moreover, the cationic Au(III)-bis(cyclopropenyl) species **28** derived from **27** by losing chloride was also observed, thus indicating the mechanism for the formation of **23**, i.e., ligand scrambling of **24** to **27** followed by reduction elimination to furnish **23**.

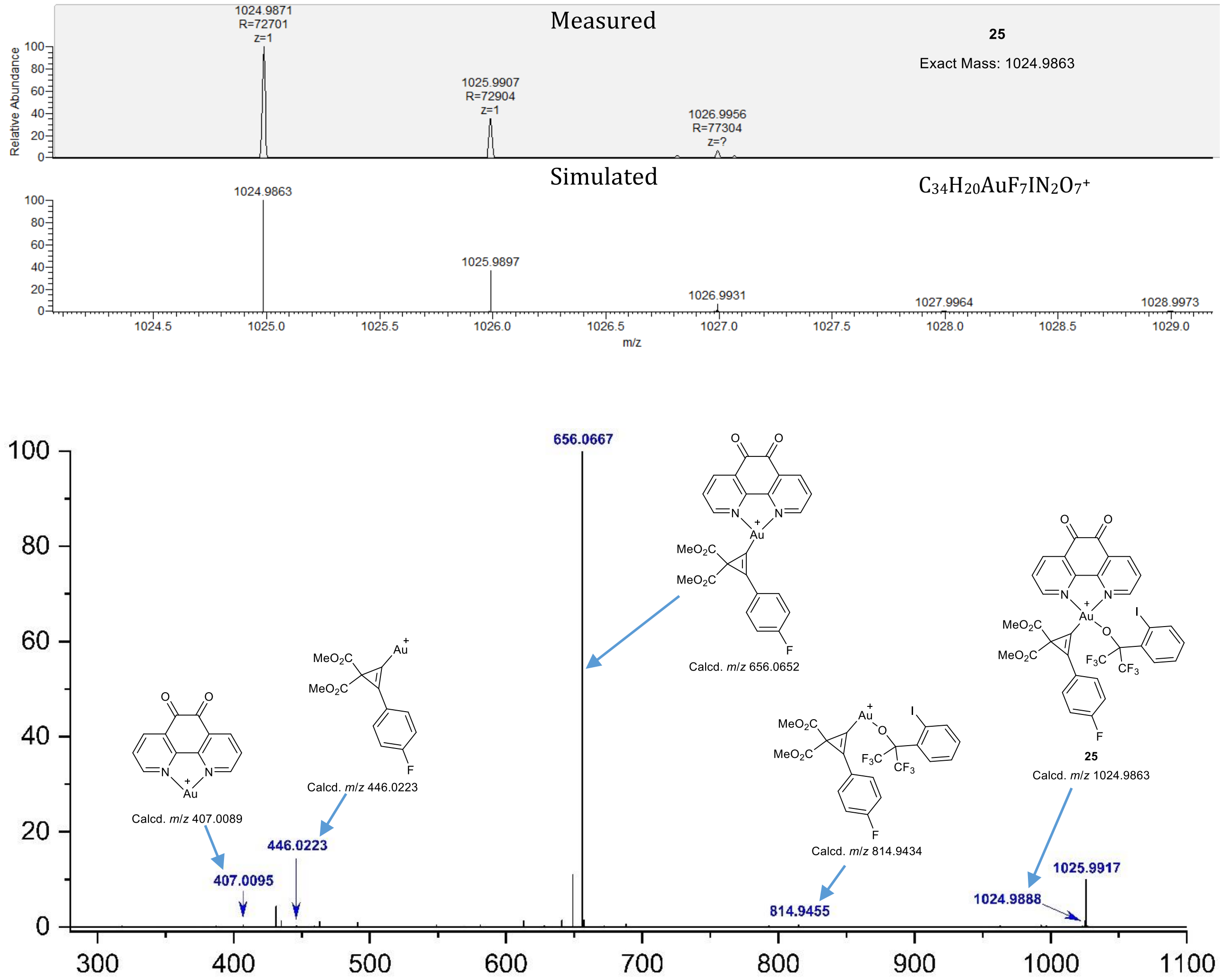

Fig. 7 | Tandem mass spectra of stoichiometric reaction of CpBX 1l, (Me_2S)AuCl and L1, isolated precursor ion *m/z* 1024.90. Cationic Au(III) species **25** derived from **24** by losing chloride was observed by ESI-MS (top) and structurally confirmed by tandem mass spectrometry (MS/MS; bottom).

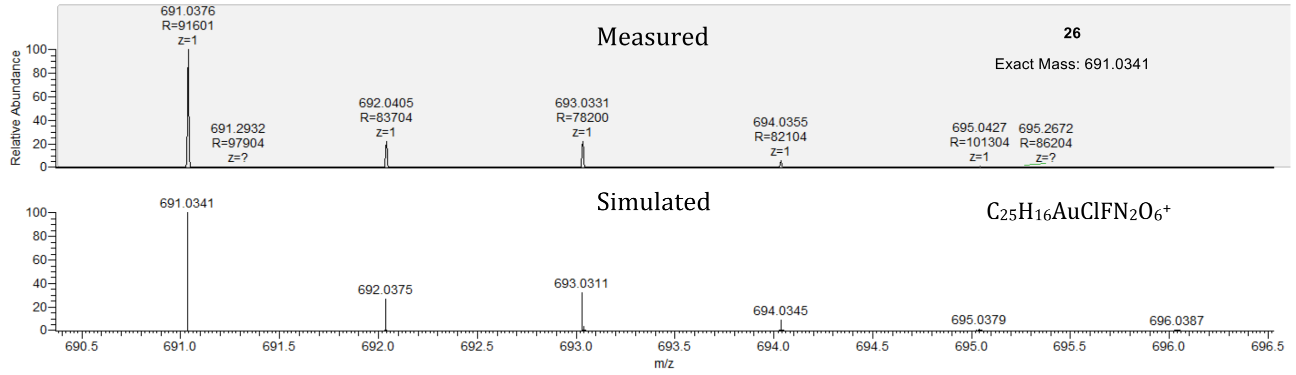

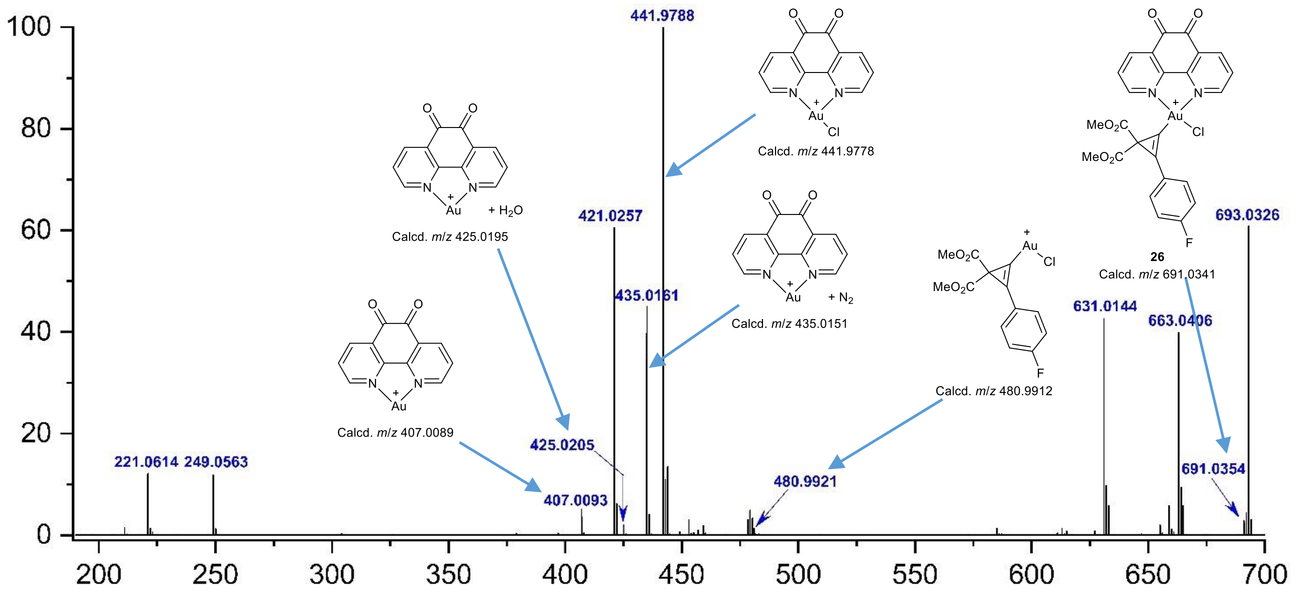

Fig. 8 | Tandem mass spectra of stoichiometric reaction of CpBX 1l, $(Me_2S)AuCl$ and L1, isolated precursor ion *m/z* 691.03. Cationic Au(III) species **26** derived from **24** by losing R^FO^- was observed by ESI-MS (top) and structurally confirmed by tandem mass spectrometry (MS/MS; bottom).

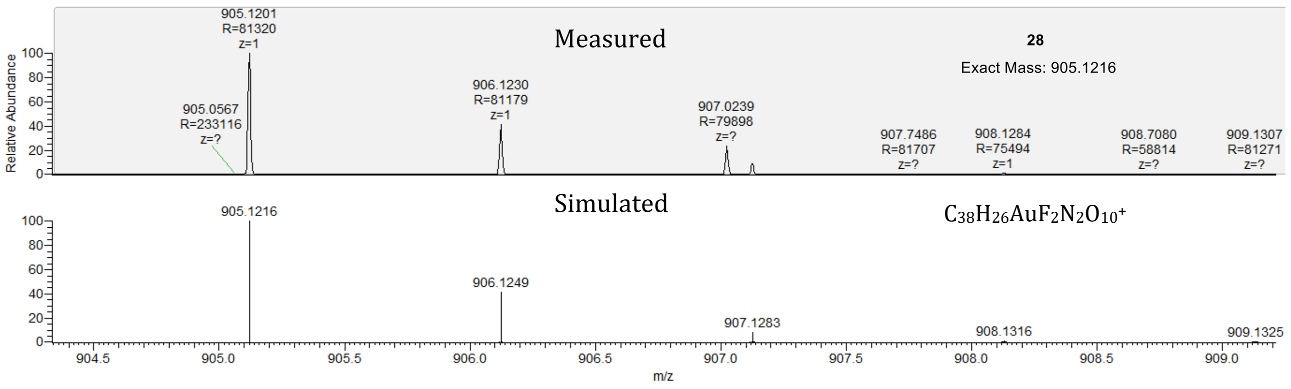

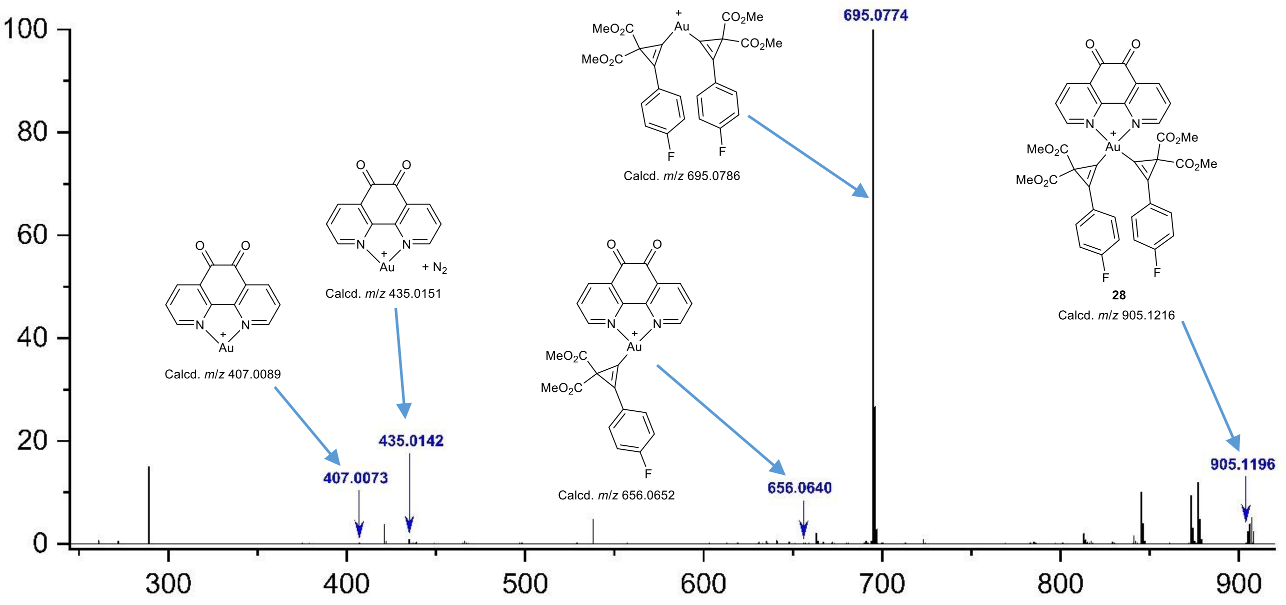

Fig. 9 | Tandem mass spectra of stoichiometric reaction of CpBX 1l, $(Me_2S)AuCl$ and L1, isolated precursor ion *m/z* 906.00. Cationic Au(III) species **28** derived from **27** by losing chloride was observed by ESI-MS (top) and structurally confirmed by tandem mass spectrometry (MS/MS; bottom).

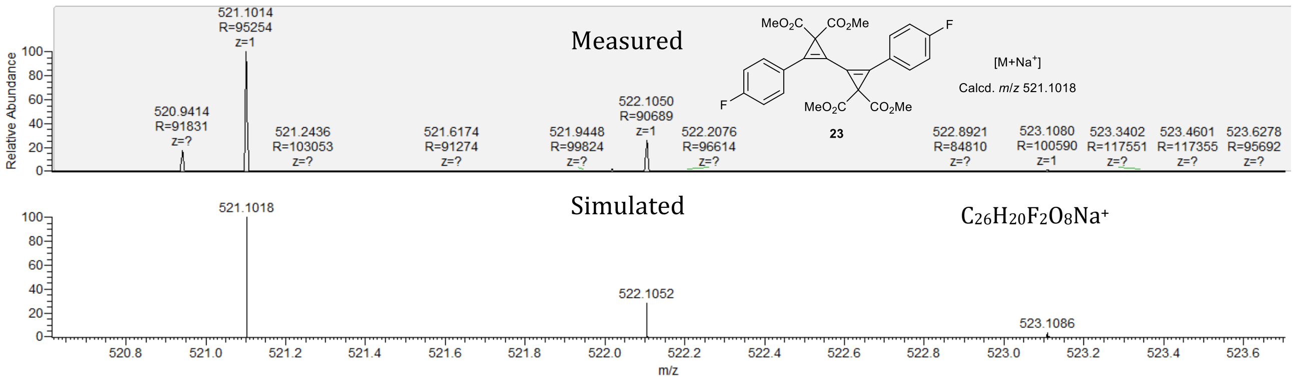

Fig. 10 | Mass spectra of stoichiometric reaction of CpBX 1l, $(Me_2S)AuCl$ and L1. Homo-coupling product **23** derived from **27** by reduction elimination was confirmed by ESI-MS.

8.6.3. Tandem mass spectrometry for determining the intermediacy of Au(III) species 29

To a 1.5 mL dry vial were sequentially added terminal alkyne **2n** (0.37 mg, 2.00 μmol, 1.00 equiv.), **L1** (0.42 mg, 2.00 μmol, 1.00 equiv.), $(Me_2S)AuCl$ (0.59 mg, 2.00 μmol, 1.00 equiv.) and **1l** (1.24 mg, 2.00 μmol, 1.00 equiv.). The vial was introduced into a nitrogen-filled glovebox and CH_3CN (1.0 mL) was then added. The vial was capped and shaken violently for 30 seconds. The resulting solution was subjected to tandem mass spectrometry (ESI-MS/MS) analysis in 5 min.

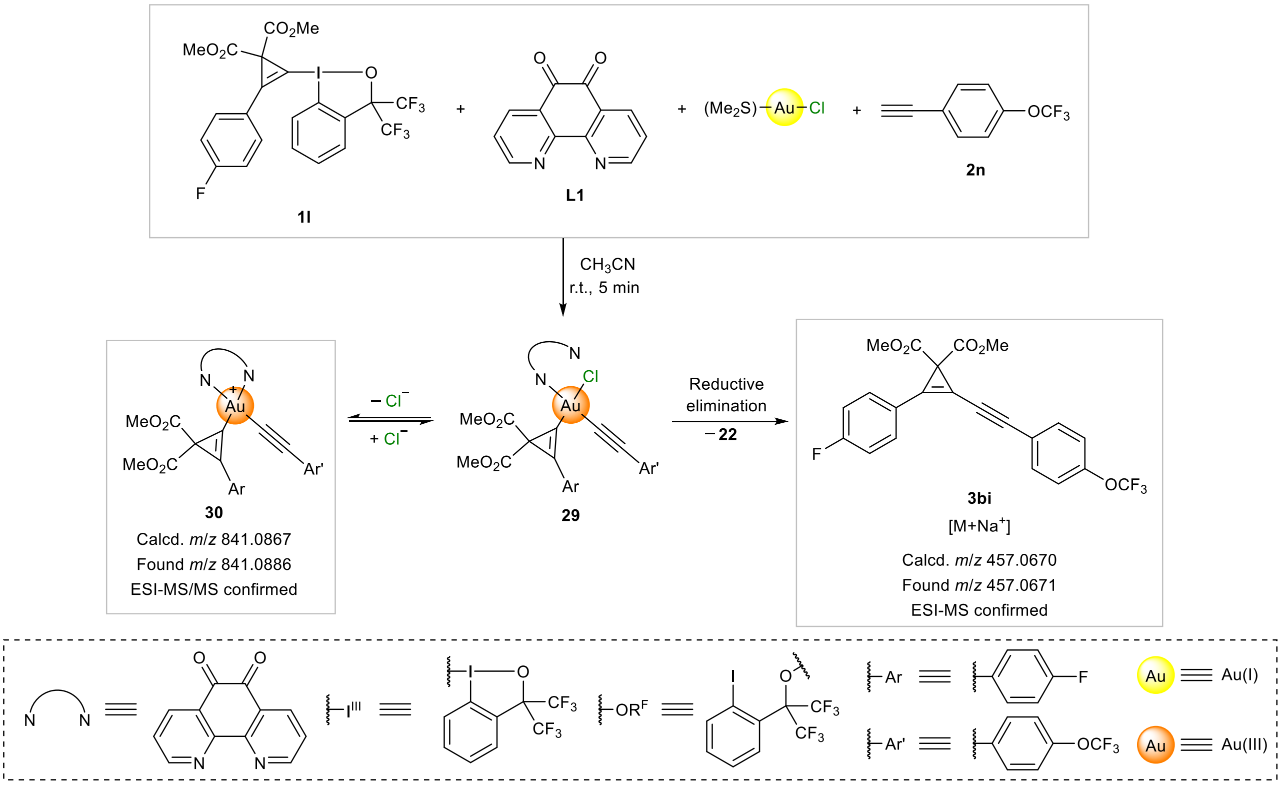

Fig. 11 | Tandem mass spectrometry (ESI-MS/MS) analysis of the stoichiometric reaction of CpBX 1l, $(Me_2S)AuCl$, L1 and alkyne 2n determined the formation of Au(III) intermediate 29. The cationic Au(III) species **30** derived from **29** by losing chloride was observed by ESI-MS and was further structurally determined by MS/MS analysis, thus providing direct evidence for the

participation of the Au(III)-cyclopropenyl species in the catalytic cycle. The Au(III) intermediate **29** is the key organogold species to connect the transmetallation and reductive elimination step.

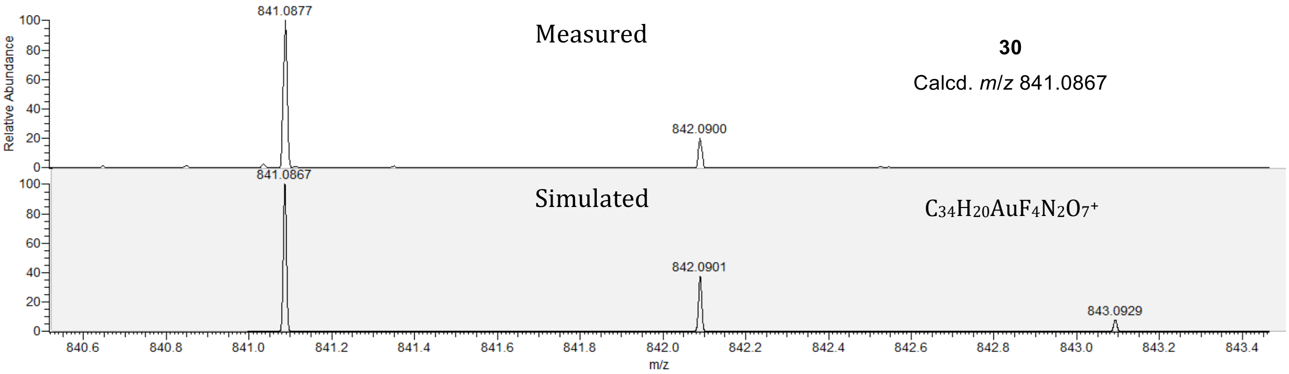

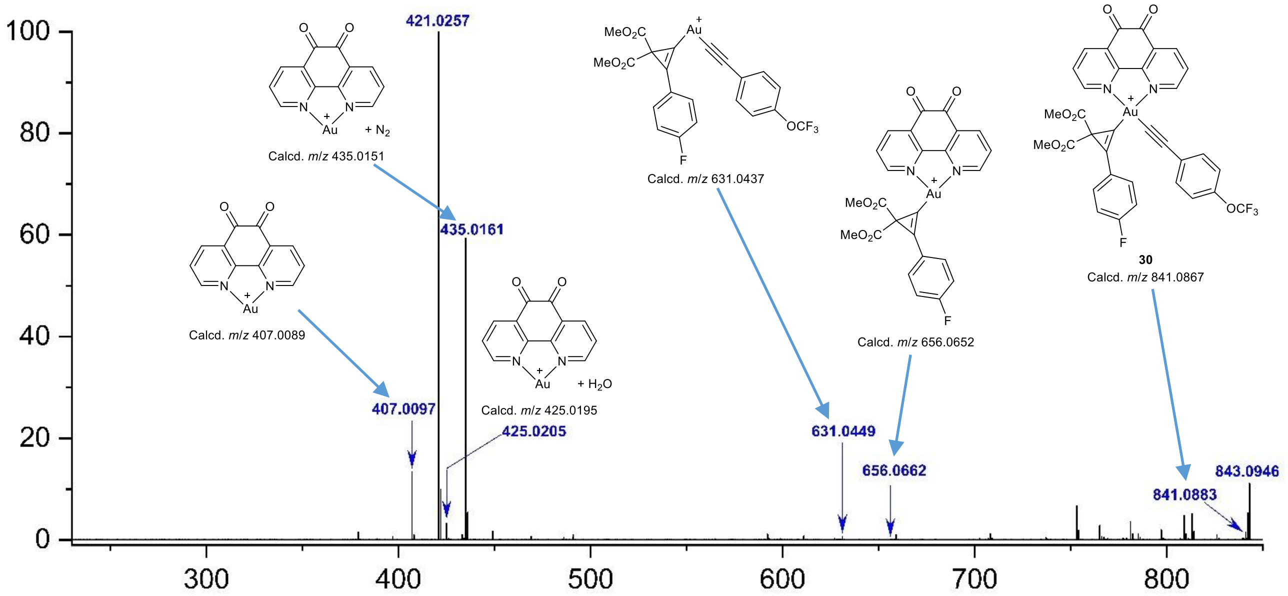

Fig. 12 | Tandem mass spectra of stoichiometric reaction of CpBX 1l, $(Me_2S)AuCl$, L1 and alkyne 2n, isolated precursor ion *m/z* 841.10. Cationic Au(III) species **30** derived from **29** by losing chloride was observed by ESI-MS (top) and structurally confirmed by tandem mass spectrometry (MS/MS; bottom).

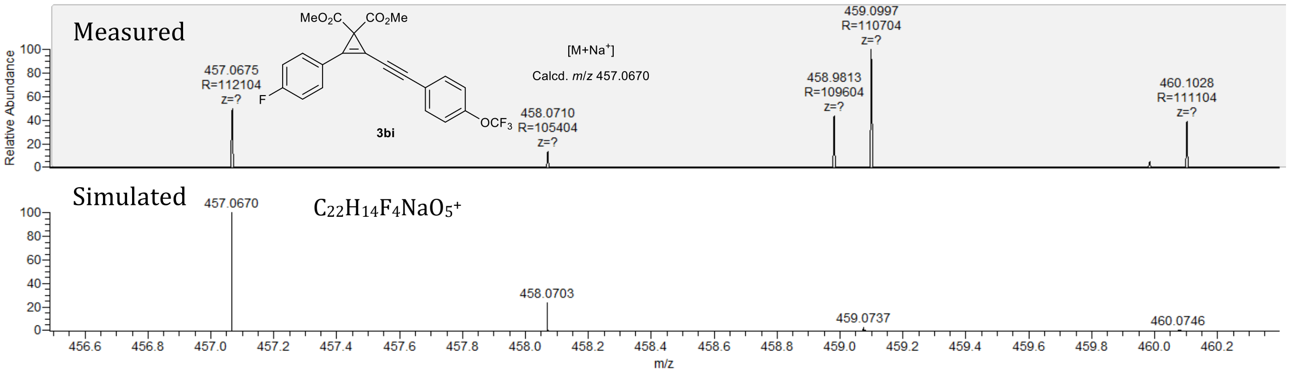

Fig. 13 | Mass spectra of stoichiometric reaction of CpBX 1l, $(Me_2S)AuCl$, L1 and alkyne 2n. Cross-coupling product **3bi** derived from **29** by reduction elimination was confirmed by ESI-MS.

8.6.4. Tandem mass spectrometry evidence for the oxidation event of cationic Au(I) and CpBXs

To a 1.5 mL dry vial was added CpBX **1l** (1.24 mg, 2.00 µmol, 1.00 equiv.). The vial was introduced into a nitrogen-filled glovebox. Then, gold(I)-ethylene complex **21** (1.4 mg, 2.00 µmol, 1.00 equiv.) and CH_3CN (1.0 mL) was then added. The vial was capped and shaken violently for 30 seconds. The resulting solution was subjected to tandem mass spectrometry (ESI-MS/MS) analysis in 5 min.

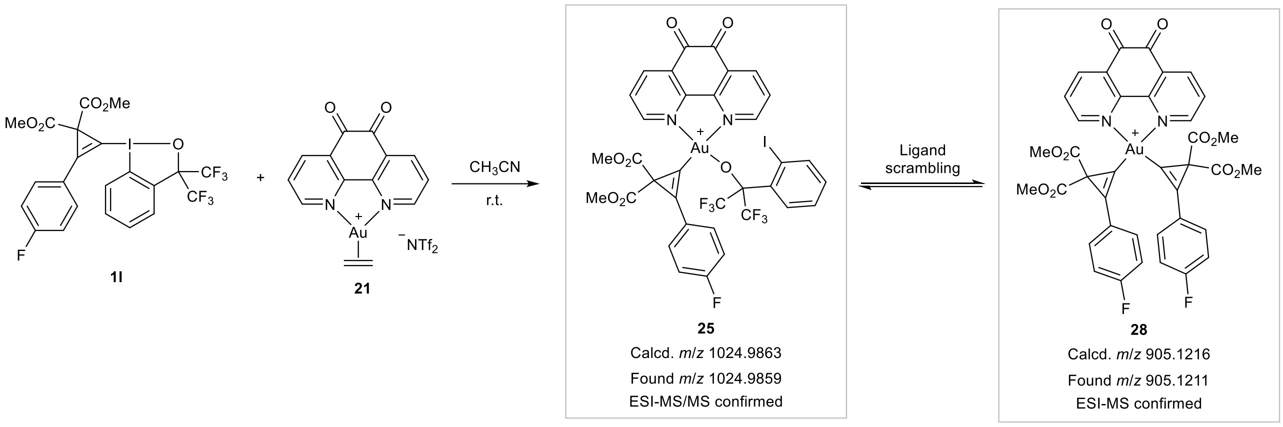

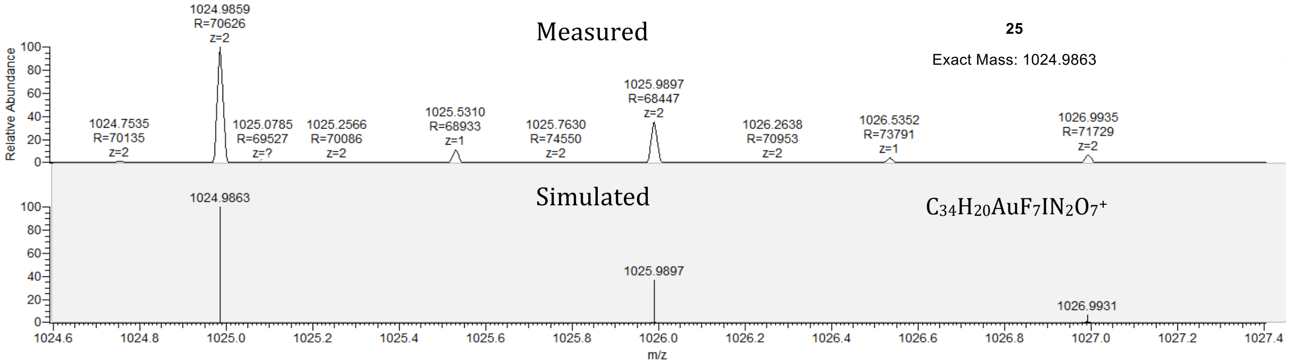

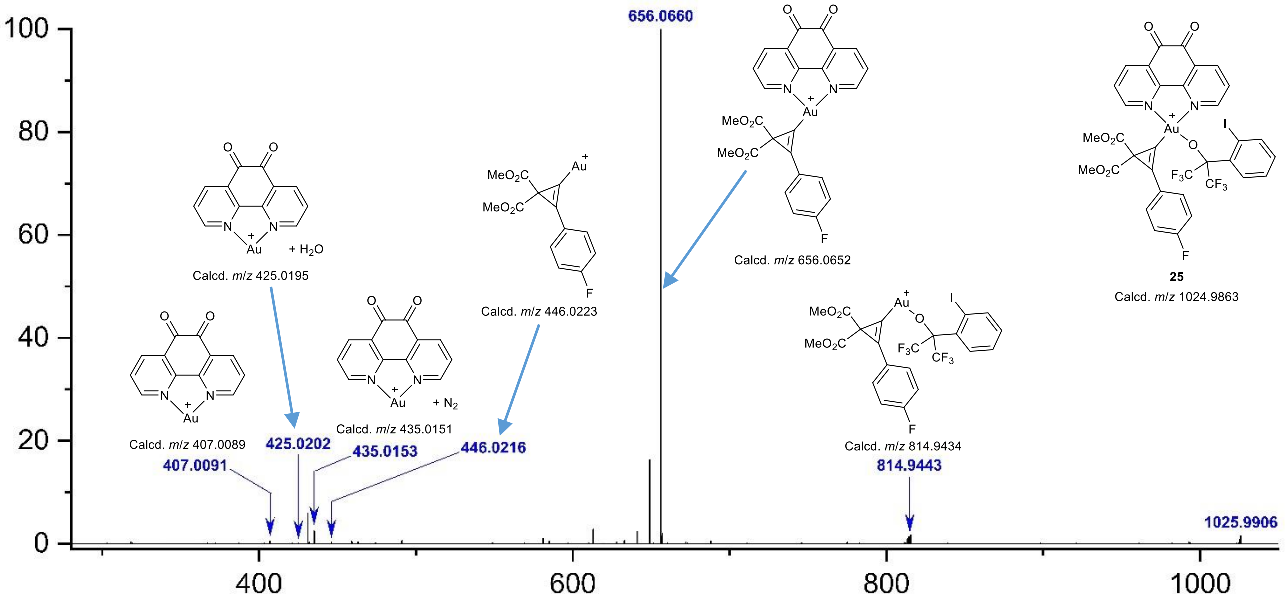

Fig. 14 | Tandem mass spectra of stoichiometric reaction of CpBX 1l and cationic gold(I)-ethylene complex 21, isolated precursor ion *m/z* 1025.00. Cationic Au(III) species **25** was observed by ESI-MS (top) and structurally confirmed by tandem mass spectrometry (MS/MS; bottom), which revealed that the CpBXs can oxidize cationic gold(I) species directly.

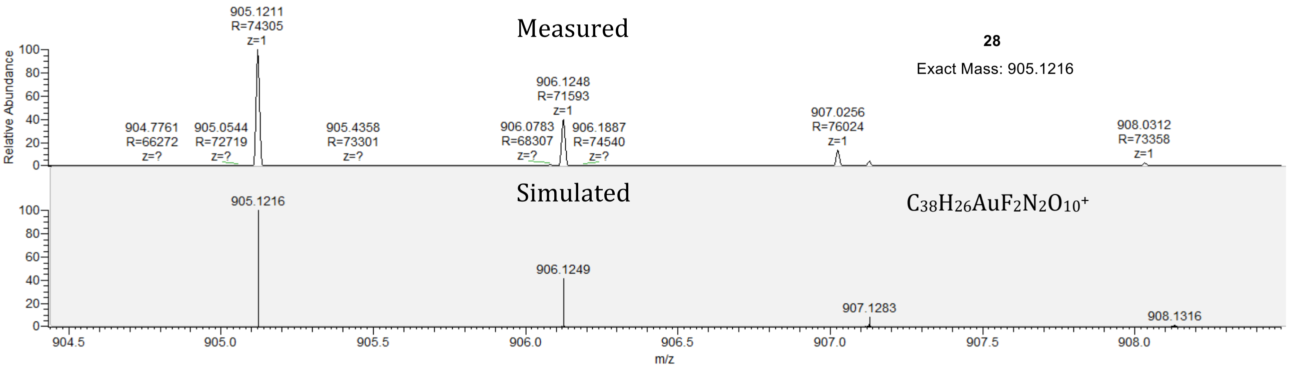

Fig. 15 | Mass spectra of stoichiometric reaction of CpBX 1l and cationic gold(I)-ethylene complex 21. Cationic Au(III) species **28** derived from **25** by ligand scrambling was observed by ESI-MS.

8.7. Stoichiometric reaction monitored by NMR

8.7.1. Stoichiometric reaction of CpBX 1l, (Me_2S)AuCl and alkyne 2n with L1 as the ligand

To a 1.5 mL dry vial were sequentially added 1,3,5-tris(trifluoromethyl)benzene (internal standard; 28.2 mg, 100 μmol, 5.00 equiv.), 1-ethynyl-4-(trifluoromethoxy)benzene **2n** (3.72 mg, 20.0 μmol, 1.00 equiv.), **L1** (4.20 mg, 20.0 μmol, 1.00 equiv.), (Me_2S)AuCl (5.89 mg, 20.0 μmol, 1.00 equiv.) and CpBX **1l** (12.4 mg, 20.0 μmol, 1.00 equiv.). The vial was introduced into a nitrogen-filled glovebox. Then, CD_3CN (0.75 mL) was added. The vial was capped and shaken violently for 20 seconds. The resulting solution was transferred into a J. Young NMR tube and subjected to NMR spectroscopy analysis immediately at 25 °C.

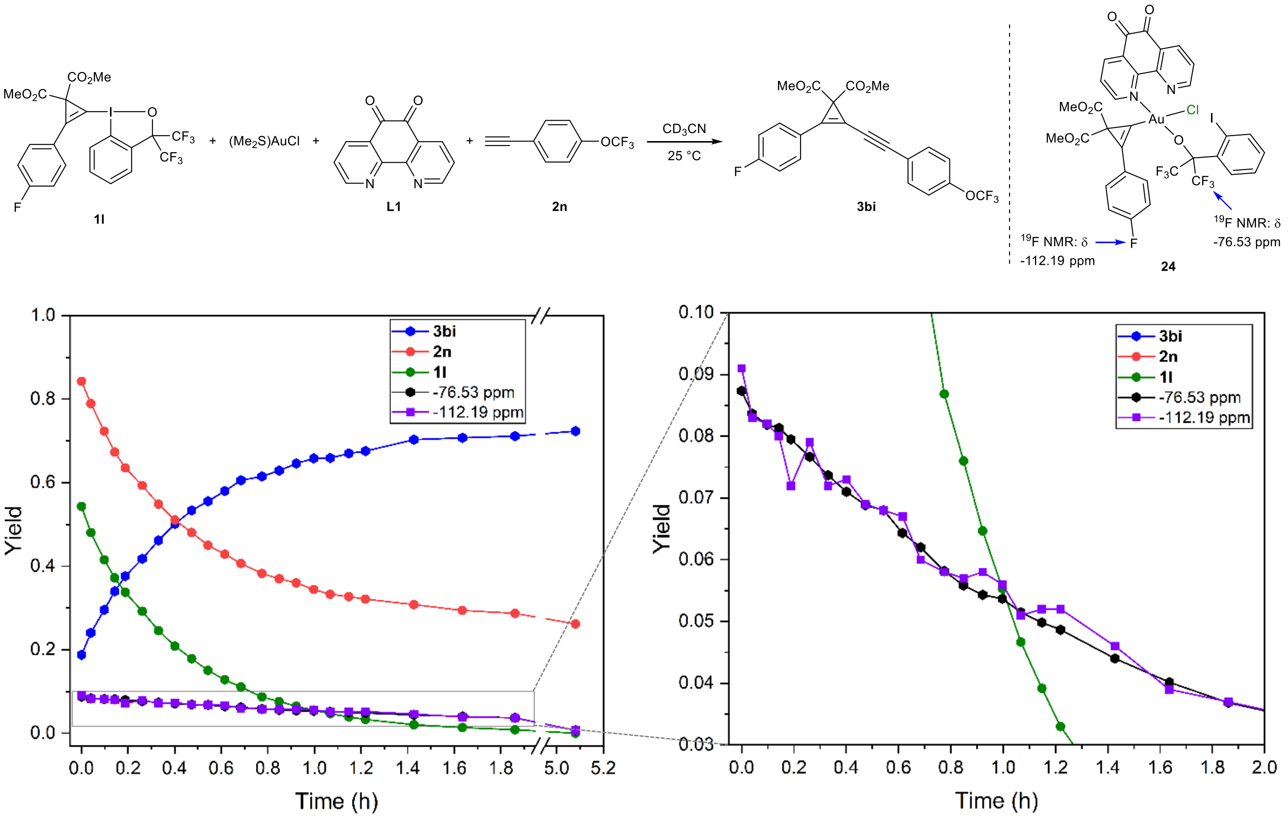

Fig. 16 | Reaction profile of the stoichiometric reaction of CpBX 1l, (Me_2S)AuCl and alkyne 2n with L1 as the ligand in CD_3CN monitored by ^{19}F NMR at 25 °C. The yield of **3bi** (δ -58.58 ppm) and

the conversion of **2n** (δ -58.68 ppm), **1l** (δ -76.53 ppm) were determined by ^{19}F NMR. The NMR monitoring experiment showed that the consuming rate of CpBX **1l** is faster than that of terminal alkyne **2n**. Interestingly, two ^{19}F NMR signals (δ -76.53 ppm and -112.19 ppm) showed high correlation during the whole reaction course, thus allowing a tentative assignment of the two ^{19}F NMR signals to the proposed cyclopropenyl-Au(III) intermediate **24**.

8.7.2. Stoichiometric reaction of CpBX 1l, gold(I)-ethylene complex 21 and alkyne 2n

To a 1.5 mL dry vial were sequentially added 1,3,5-tris(trifluoromethyl)benzene (internal standard; 28.2 mg, 100 μmol, 5.00 equiv.), 1-ethynyl-4-(trifluoromethoxy)benzene **2n** (3.72 mg, 20.0 μmol, 1.00 equiv.) and CpBX **1l** (12.4 mg, 20.0 μmol, 1.00 equiv.). The vial was introduced into a nitrogen-filled glovebox. Then, gold(I)-ethylene complex **21** (14.3 mg, 20.0 μmol, 1.00 equiv.) and CD_3CN (0.75 mL) was added. The vial was capped and shaken violently for 20 seconds. The resulting solution was transferred into a J. Young NMR tube and subjected to NMR spectroscopy analysis immediately at 25 °C.

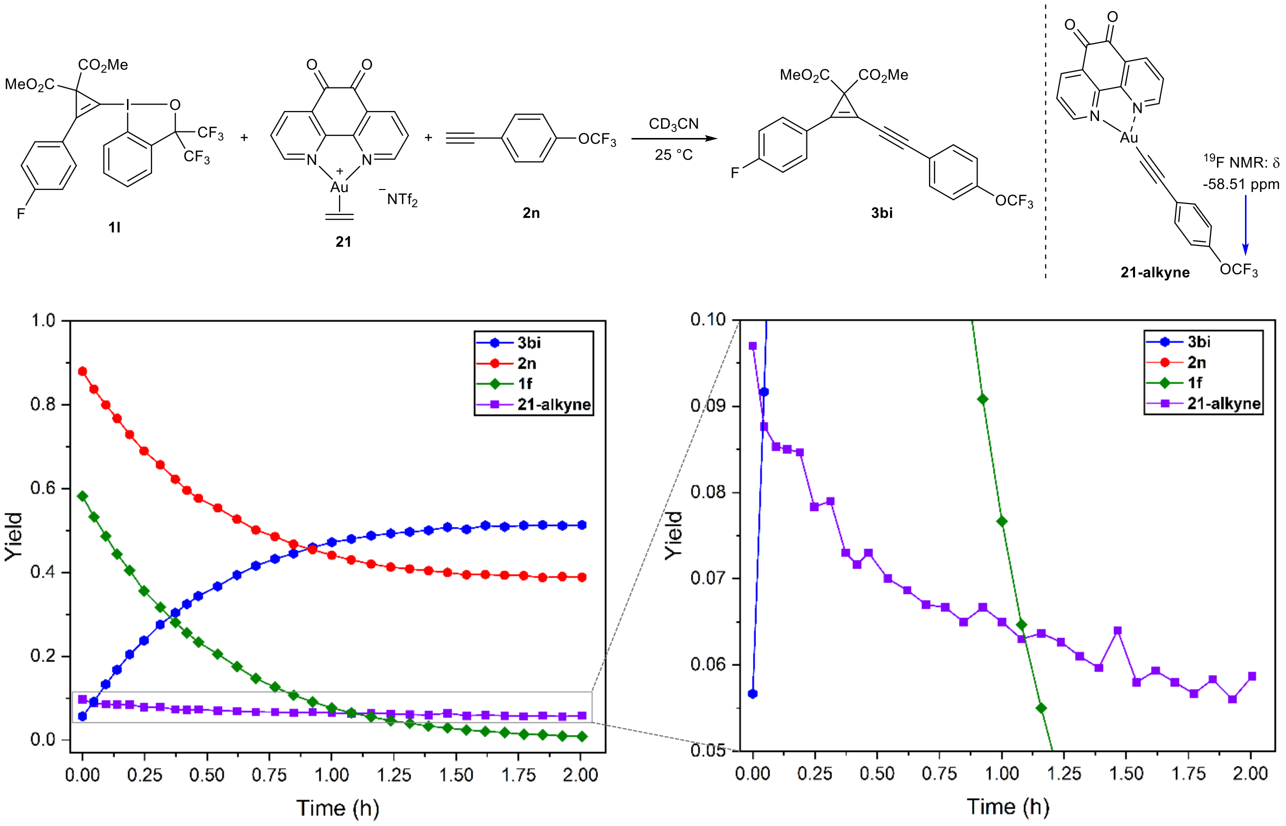

Fig. 17 | Reaction profile of the stoichiometric reaction of CpBX 1l, gold(I)-ethylene complex 21 and alkyne 2n in CD_3CN monitored by ^{19}F NMR at 25 °C. Although the gold(I)-acetylide **21-alkyne** was observed in the stoichiometric reaction, however, unlike the case in Table 7, entry 4 (**2a**:**21** = 20:1), the stoichiometric reaction of CpBX **1l**, gold complex **21** and alkyne **2n** (**2n**:**21** = 1:1) took place smoothly and the product **3bi** was observed in around 60% yield in the end, which was close to the yield observed in Table 7, entry 5. These results indicate that using excess amount of gold(I)-ethylene complex **21** can restore the catalytic activity of **21** even if partial gold(I)-ethylene complex **21** was consumed by the formation of the catalytically inert gold(I)-acetylide. However, the yield of the desired product **3bi** was comparatively lower than the one using chloride-supported Au(I) catalyst (as revealed by the case showed in Fig. 8), thus suggesting the significant role of chloride in maintaining high efficiency and turn-over number of the gold catalyst. The gradual consumption of gold(I)-acetylide **21-alkyne** in the stoichiometric reaction can be explained by the reversible release of free

terminal alkyne **2n** in view of the decreasing concentration of **2n** as the reaction going on. Alternatively, gold(I)-acetylide **21-alkyne** underwent smooth transmetallation with the cyclopropenyl-Au(III) intermediate **25** (*vide infra*), thus resulting its decreasing concentration during the reaction course.

8.7.3. Stoichiometric reaction of CpBX 1l and gold(I)-ethylene complex 21

To a 1.5 mL dry vial were sequentially added 1,3,5-tris(trifluoromethyl)benzene (internal standard; 28.2 mg, 100 μmol, 5.00 equiv.) and CpBX **1l** (12.4 mg, 20.0 μmol, 1.00 equiv.). The vial was introduced into a nitrogen-filled glovebox. Then, gold(I)-ethylene complex **21** (14.3 mg, 20.0 μmol, 1.00 equiv.) and CD_3CN (0.75 mL) was added. The vial was capped and shaken violently for 20 seconds. The resulting solution was transferred into a J. Young NMR tube and subjected to NMR spectroscopy analysis immediately at 25 °C.

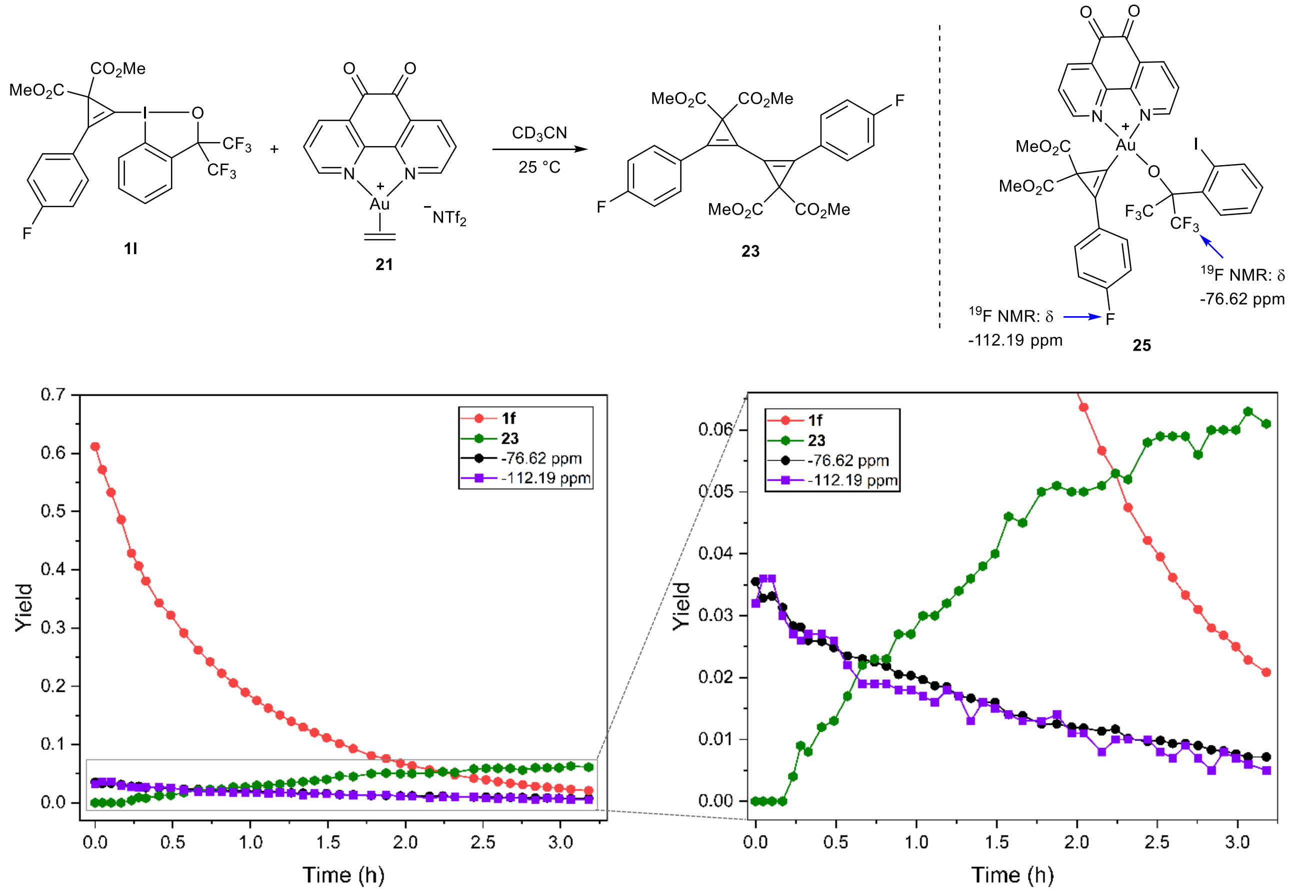

Fig. 18 | Reaction profile of the stoichiometric reaction of CpBX 1l and gold(I)-ethylene complex 21 in CD_3CN monitored by ^{19}F NMR at 25 °C. The yield of **23** (δ -107.86 ppm) and the conversion of **1l** (δ -76.71 ppm) were determined by ^{19}F NMR. The NMR monitoring experiment showed clearly that the CpBX reagents, such as **1l** can directly oxidize cationic gold(I)-ethylene complex **21**, which was also proved by the formation of the homo-coupled product **23** by ligand scrambling (the same mechanism showed in Fig. 5.) of the putative cyclopropenyl-Au(III) intermediate **25**. The relative lower yield of homo-coupled product **23** in the end of the reaction compared with the one showed in Fig. 4 (with chloride as a supporting ligand) was attributed to its relatively poorer stability of cyclopropenyl-Au(III) intermediate **25** compared with its chloride-supported counterpart **24**. Indeed, two ^{19}F NMR signals (δ -76.62 ppm and -112.19 ppm) showed high correlation during the whole reaction course, thus allowing a tentative assignment of the two ^{19}F NMR signals to the proposed cyclopropenyl-Au(III) intermediate **25**.

8.8. DFT Calculations

To gain further understanding of the catalytic cycle, we computed key energetic barriers associated with oxidative addition and reductive elimination on a model substrate (**1a**) reacting with phenylacetylene (**2a**). The geometries of all structures were first optimized at the PBE0[65,66]-D3(BJ)[67,68,69,70] /def2-SVP[71] level using the SMD implicit solvent model[72] for acetonitrile in Gaussian16.[73] Refined energy estimates were obtained by singlet point computations at the B3PW91[74,75,76]-D3(BJ)/def2-TZVP[71] level on the PBE0-D3(BJ) optimized geometries. Each species was characterized as either a minimum (zero imaginary frequencies) or a transition state (one imaginary frequency) via examination of the vibrational frequencies of the optimized structures. Free energies reported include B3PW91-D3(BJ)/def2-TZVP//PBE0-D3(BJ)/def2-SVP electronic energies along with free energy corrections (at the PBE0-D3(BJ)/def2-SVP level) using the quasi rigid-rotor harmonic oscillator model and a pressure correction for acetonitrile (19.15 mol/L) to treat translational entropy in solution using the approach of Martin, Hay, and Pratt.[77] Optimized Cartesian coordinates of all species can be found as additional supporting information files (.zip).

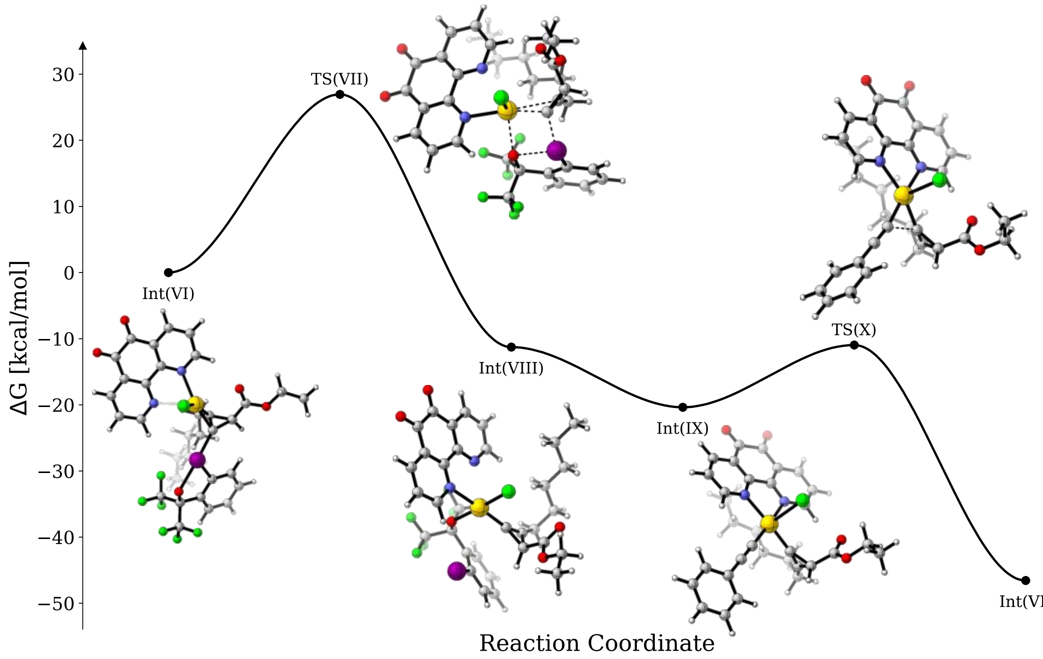

Fig. 19 | Computed free energy profile (at the B3PW91-D3(BJ)/def2-TZVP//PBE0-D3(BJ)/def2-SVP level) of the catalytic cycle. Values in kcal/mol.

Table 9 | Computed values of key species.

	PBE0-D3(BJ)/def2-SVP Electronic Energy [hartree]	PBE0-D3(BJ)/def2-SVP Free Energy Correction [hartree]	B3PW91-D3(BJ)/def2-TZVP// PBE0-D3(BJ)/def2-SVP Electronic Energy [hartree]	Total Free Energy [hartree]	ΔG (Relative to Int(VI)) [kcal/mol]
**1a**	-1933.530468	0.348311	-1936.710065	-1936.361754	--
**2a**	-307.822759	0.085690	-308.415394	-308.329704	--
**3a** (Product)	-925.244523	0.340375	-927.0201408	-926.6797658	--
**4** (Alcohol)	-1316.201691	0.095126	-1318.181023	-1318.085897	--
Int(**VI**)	-3248.824032	0.492439	-3253.714552	-3253.222113	0.00
TS(**VII**)	-3248.785956	0.490436	-3253.669623	-3253.179187	26.94
Int(**VIII**)	-3248.849422	0.492924	-3253.732997	-3253.240073	-11.27
Int(**IX**)	-2240.476560	0.484620	-2243.982999	-2243.498379	-20.37
TS(**X**)	-2240.467656	0.483783	-2243.967175	-2243.483392	-10.96

9. Single crystal X-ray diffraction analysis

9.1. Crystal data and structure refinement for 1k

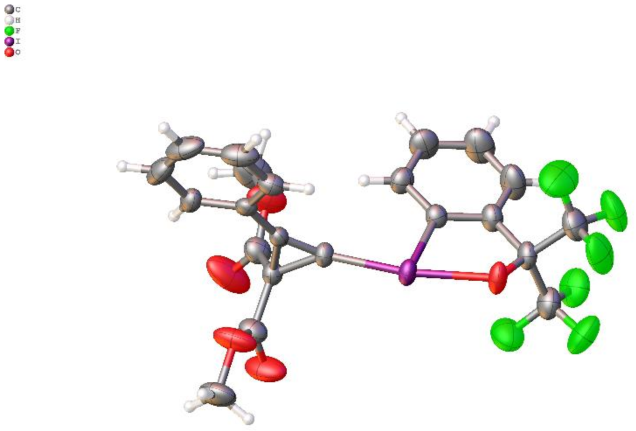

Compound	1k
CCDC code	2260609
Formula	$C_{22}H_{15}F_6IO_5$
Dcalc	1.746
μ/mm^{-1}	1.482
Formula Weight	600.24
Colour	clear pale colourless
Shape	plate-shaped
Size/mm^3	0.17×0.15×0.08
T/K	229.99(10)
Crystal System	monoclinic
Space Group	$P2_1/n$
a/Å	16.6125(5)
b/Å	7.7673(2)
c/Å	18.9493(6)
α/°	90
β/°	110.931(3)
γ/°	90
V/Å^3	2283.74(12)
Z	4
Z'	1
Wavelength/Å	0.71073
Radiation type	Mo K$_\alpha$
Θ_{min}/°	2.806
Θ_{max}/°	29.537
Measured Refl's.	18406
Indep't Refl's	5533
Refl's I≥2 σ(I)	4588
R_{int}	0.0247
Parameters	328
Restraints	30
Largest Peak	0.537
Deepest Hole	-0.376
GooF	1.036
wR_2 (all data)	0.0749
wR_2	0.0689
R_1 (all data)	0.0438
R_1	0.0322

Preparation. Monocrystal suitable for X-ray diffraction analysis was grown by slow diffusion of hexane into saturated CH_2Cl_2 solution of **1k**.

Experimental. Single clear pale colourless plate-shaped crystals of **1k** were used as supplied. A suitable crystal with dimensions 0.17 × 0.15 × 0.08 mm^3 was selected and mounted on a SuperNova, Dual, Cu at home/near, AtlasS2 diffractometer. The crystal was kept at a steady T = 229.99(10) K during data collection. The structure was solved with the **ShelXT** (Sheldrick, 2015) solution program using dual methods and by using **Olex2** 1.5 (Dolomanov et al., 2009) as the graphical interface. The model was refined with **ShelXL** 2018/3 (Sheldrick, 2015) using full matrix least squares minimisation on $\boldsymbol{F}^2$.

Crystal Data. $C_{22}H_{15}F_6IO_5$, M_r = 600.24, monoclinic, $P2_1/n$ (No. 14), a = 16.6125(5) Å, b = 7.7673(2) Å, c = 18.9493(6) Å, β = 110.931(3)°, α = γ = 90°, V = 2283.74(12) Å^3, T = 229.99(10) K, Z = 4, Z' = 1, μ(Mo K$_\alpha$) = 1.482, 18406 reflections measured, 5533 unique (R_{int} = 0.0247) which were used in all calculations. The final wR_2 was 0.0749 (all data) and R_1 was 0.0322 (I≥2 σ(I)).

9.2. Crystal data and structure refinement for 3au

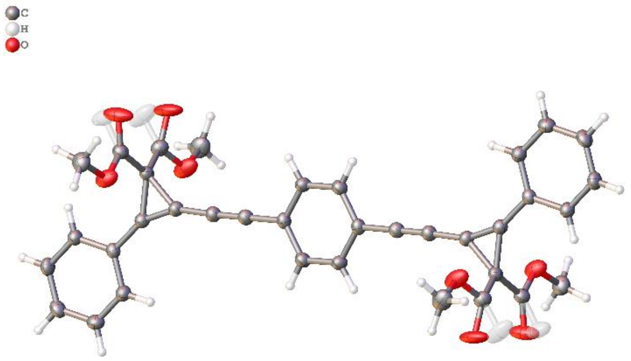

Compound	3au
CCDC code	2260610
Formula	$C_{36}H_{26}O_8$
$D_{calc.}$/ g cm^{-3}	1.309
μ/mm^{-1}	0.764
Formula Weight	586.57
Colour	clear pale yellow
Shape	prism-shaped
Size/mm^3	0.29×0.15×0.09
T/K	139.99(10)
Crystal System	triclinic
Space Group	P-1
a/Å	7.79978(19)
b/Å	10.2444(3)
c/Å	10.4570(3)
α/°	64.821(3)
β/°	79.661(2)
γ/°	85.675(2)
V/Å^3	743.89(4)
Z	1
Z'	0.5
Wavelength/Å	1.54184
Radiation type	Cu K_α
Θ_{min}/°	4.736
Θ_{max}/°	74.473
Measured Refl's.	14396
Indep't Refl's	2921
Refl's I≥2 σ(I)	2526
R_{int}	0.0367
Parameters	271
Restraints	36
Largest Peak	0.293
Deepest Hole	-0.331
GooF	1.077
wR_2 (all data)	0.1379
wR_2	0.1326
R_1 (all data)	0.0533
R_1	0.0473

Preparation. Monocrystal suitable for X-ray diffraction analysis was grown by slow diffusion of pentane into saturated CH_2Cl_2 solution of **3au**.

Experimental. Single clear pale-yellow prism-shaped crystals of **3au** were used as supplied. A suitable crystal with dimensions 0.29 × 0.15 × 0.09 mm^3 was selected and mounted on a XtaLAB Synergy R, DW system, HyPix-Arc 150 diffractometer. The crystal was kept at a steady T = 139.99(10) K during data collection. The structure was solved with the **ShelXT** (Sheldrick, 2015) solution program using dual methods and by using **Olex2** 1.5 (Dolomanov et al., 2009) as the graphical interface. The model was refined with **ShelXL** 2018/3 (Sheldrick, 2015) using full matrix least squares minimisation on $\boldsymbol{F^2}$.

Crystal Data. $C_{36}H_{26}O_8$, M_r = 586.57, triclinic, P-1 (No. 2), a = 7.79978(19) Å, b = 10.2444(3) Å, c = 10.4570(3) Å, α = 64.821(3)°, β = 79.661(2)°, γ = 85.675(2)°, V = 743.89(4) Å^3, T = 139.99(10) K, Z = 1, Z' = 0.5, μ(Cu K_α) = 0.764, 14396 reflections measured, 2921 unique (R_{int} = 0.0367) which were used in all calculations. The final wR_2 was 0.1379 (all data) and R_1 was 0.0473 (I≥2 σ(I)).

9.3. Crystal data and structure refinement for 21

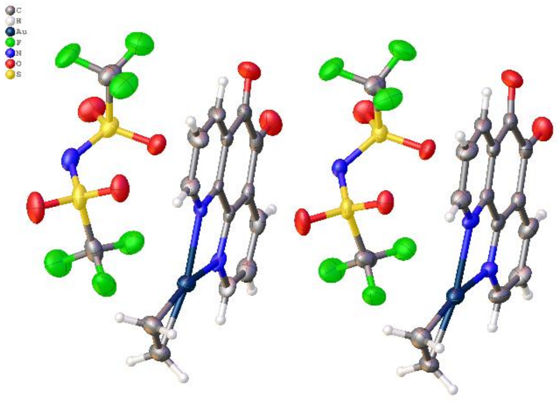

Preparation. Monocrystal suitable for X-ray diffraction analysis was grown by slow diffusion of Et_2O (top layer) into saturated CH_2Cl_2 solution of **21** (bottom layer).

Experimental. Single clear pale colourless plate-shaped crystals of **21** were used as supplied. A suitable crystal with dimensions 0.22 × 0.05 × 0.03 mm^3 was selected and mounted on a XtaLAB Synergy R, DW system, HyPix-Arc 150 diffractometer. The crystal was kept at a steady T = 140.00(10) K during data collection. The structure was solved with the **ShelXT** (Sheldrick, 2015) solution program using dual methods and by using **Olex2** 1.5 (Dolomanov et al., 2009) as the graphical interface. The model was refined with **olex2.refine** 1.5 (Bourhis et al., 2015) using full matrix least squares minimisation on $\boldsymbol{F}^2$.

Crystal Data. $C_{16}H_{10}AuF_6N_3O_6S_2$, M_r = 715.363, monoclinic, $P2_1/n$ (No. 14), a = 16.2041(4) Å, b = 15.2787(3) Å, c = 17.5817(4) Å, β = 106.170(2)°, $\alpha = \gamma$ = 90°, V = 4180.64(16) $Å^3$, T = 140.00(10) K, Z = 8, Z' = 2, μ(Mo K_α) = 7.333, 109486 reflections measured, 8536 unique (R_{int} = 0.0974) which were used in all calculations. The final wR_2 was 0.1072 (all data) and R_1 was 0.0427 (I≥2 σ(I))

**Compound**	**21**
CCDC code	2260611
Formula	$C_{16}H_{10}AuF_6N_3O_6S_2$
$D_{calc.}$/ g cm^{-3}	2.273
μ/mm^{-1}	7.333
Formula Weight	715.363
Colour	clear pale colourless
Shape	plate-shaped
Size/mm^3	0.22×0.05×0.03
T/K	140.00(10)
Crystal System	monoclinic
Space Group	$P2_1/n$
a/Å	16.2041(4)
b/Å	15.2787(3)
c/Å	17.5817(4)
α/°	90
β/°	106.170(2)
γ/°	90
V/$Å^3$	4180.64(16)
Z	8
Z'	2
Wavelength/Å	0.71073
Radiation type	Mo K_α
Θ_{min}/°	2.01
Θ_{max}/°	26.37
Measured Refl's.	109486
Indep't Refl's	8536
Refl's I≥2 σ(I)	7291
R_{int}	0.0974
Parameters	663
Restraints	90
Largest Peak	2.9128
Deepest Hole	-2.5025
GooF	1.0469
wR_2 (all data)	0.1072
wR_2	0.1032
R_1 (all data)	0.0526
R_1	0.0427

10. NMR spectra

^{1}H NMR (400 MHz, $CDCl_3$) of s-1a

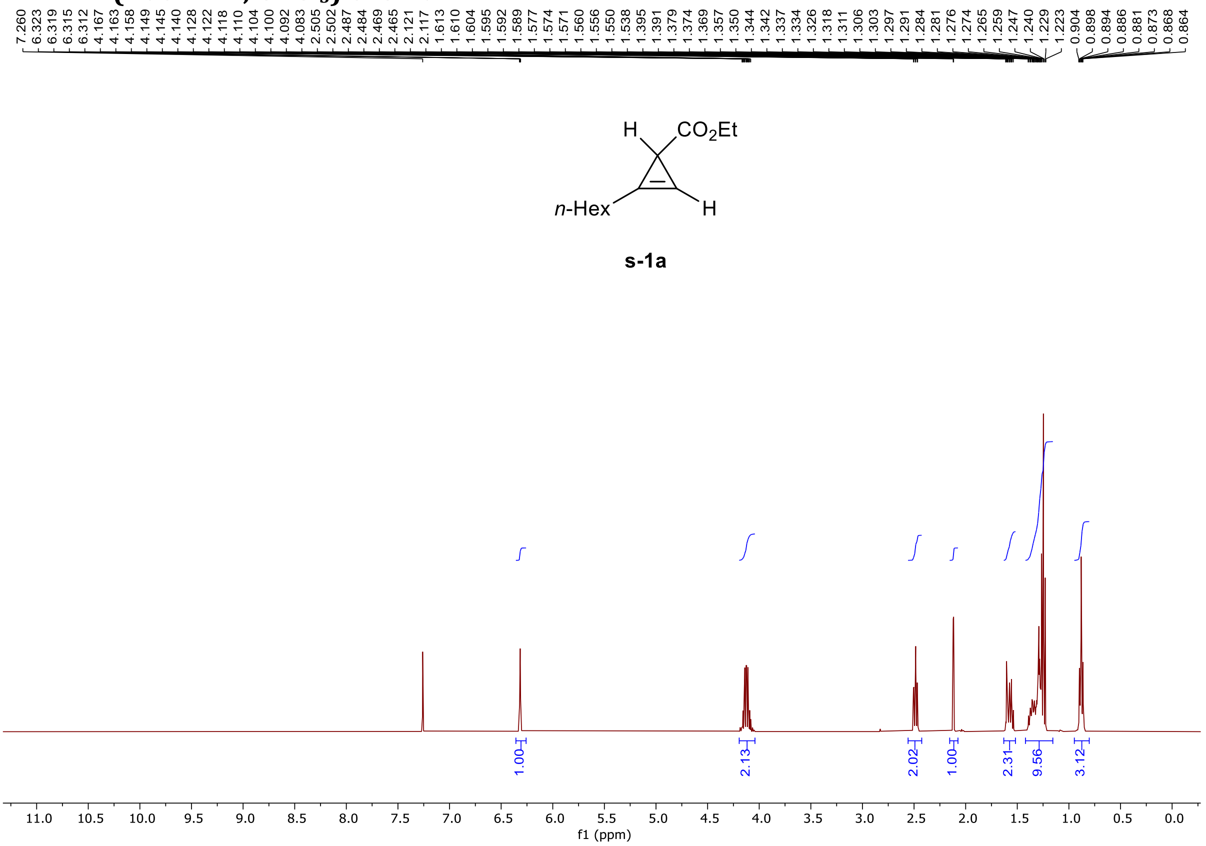

^{13}C NMR (101 MHz, $CDCl_3$) of s-1a

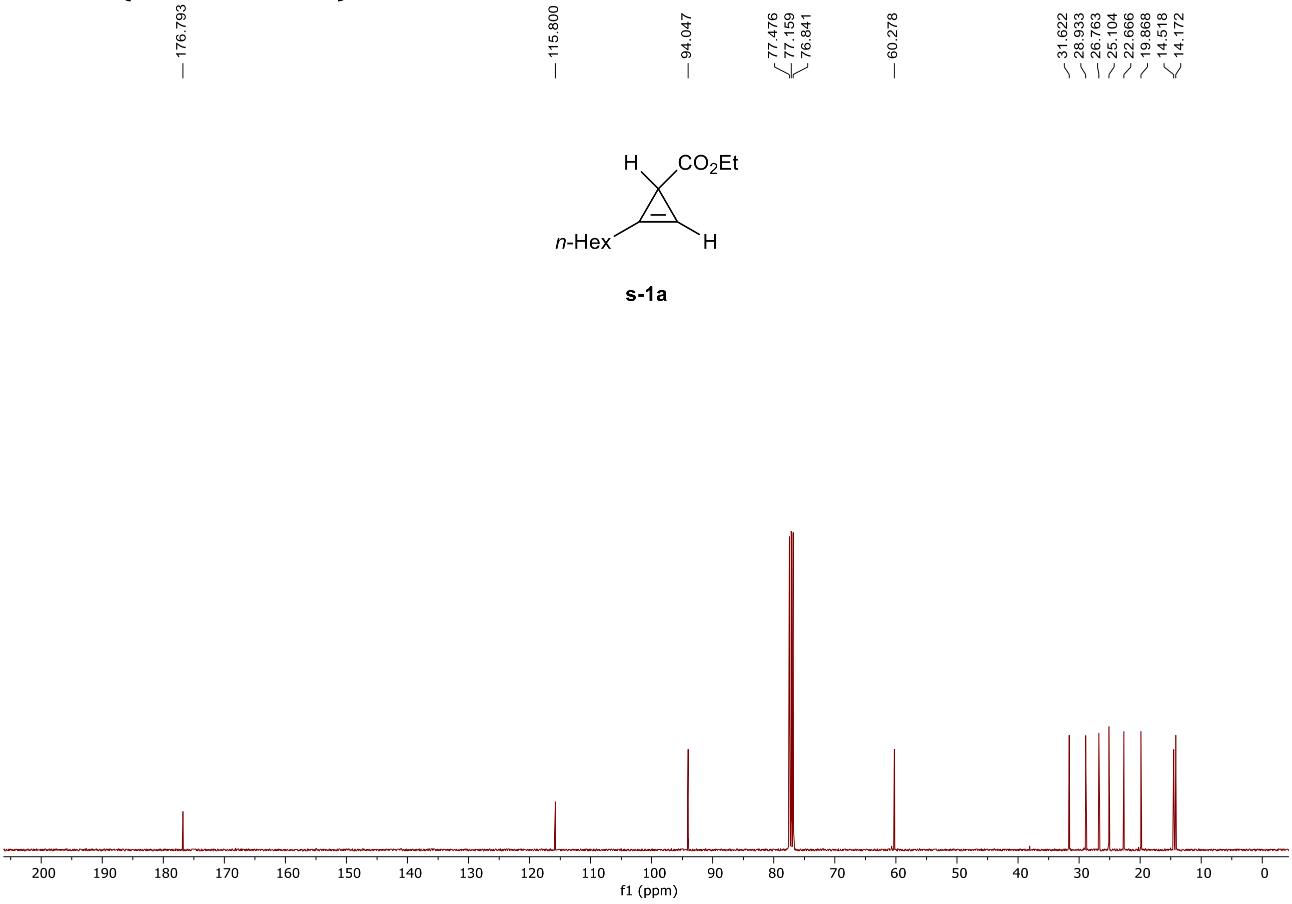

^{1}H NMR (400 MHz, $CDCl_3$) of s-1b

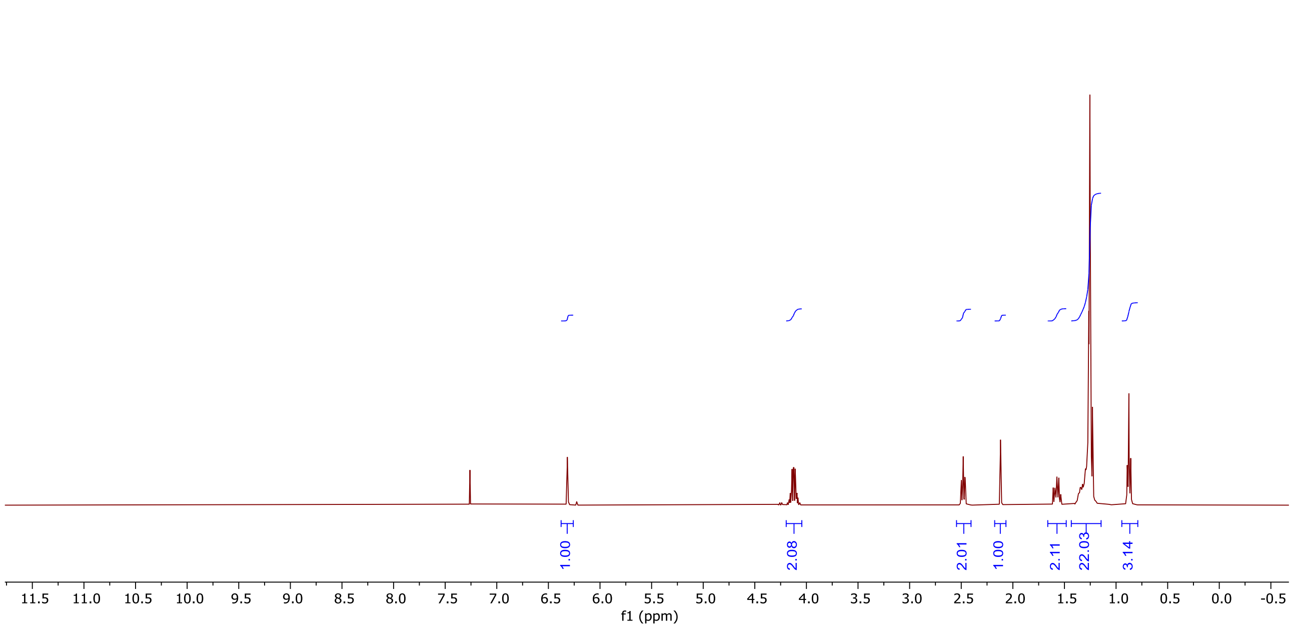

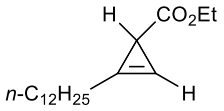

s-1b

^{13}C NMR (101 MHz, $CDCl_3$) of s-1b

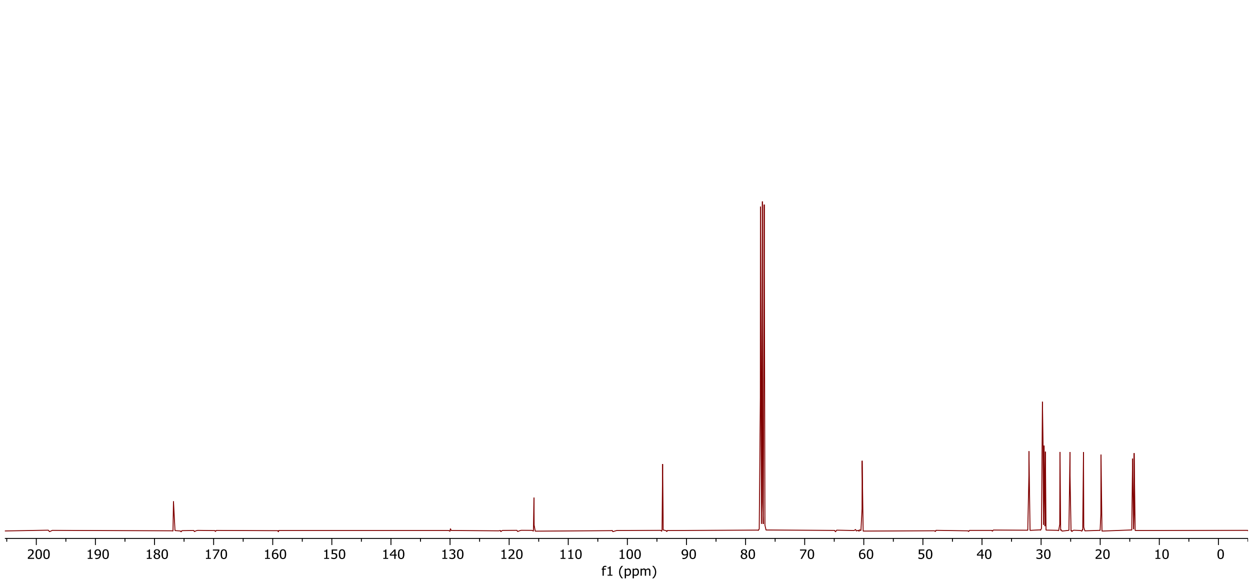

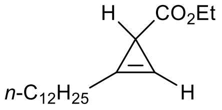

s-1b

^{1}H NMR (400 MHz, $CDCl_3$) of *s*-1c

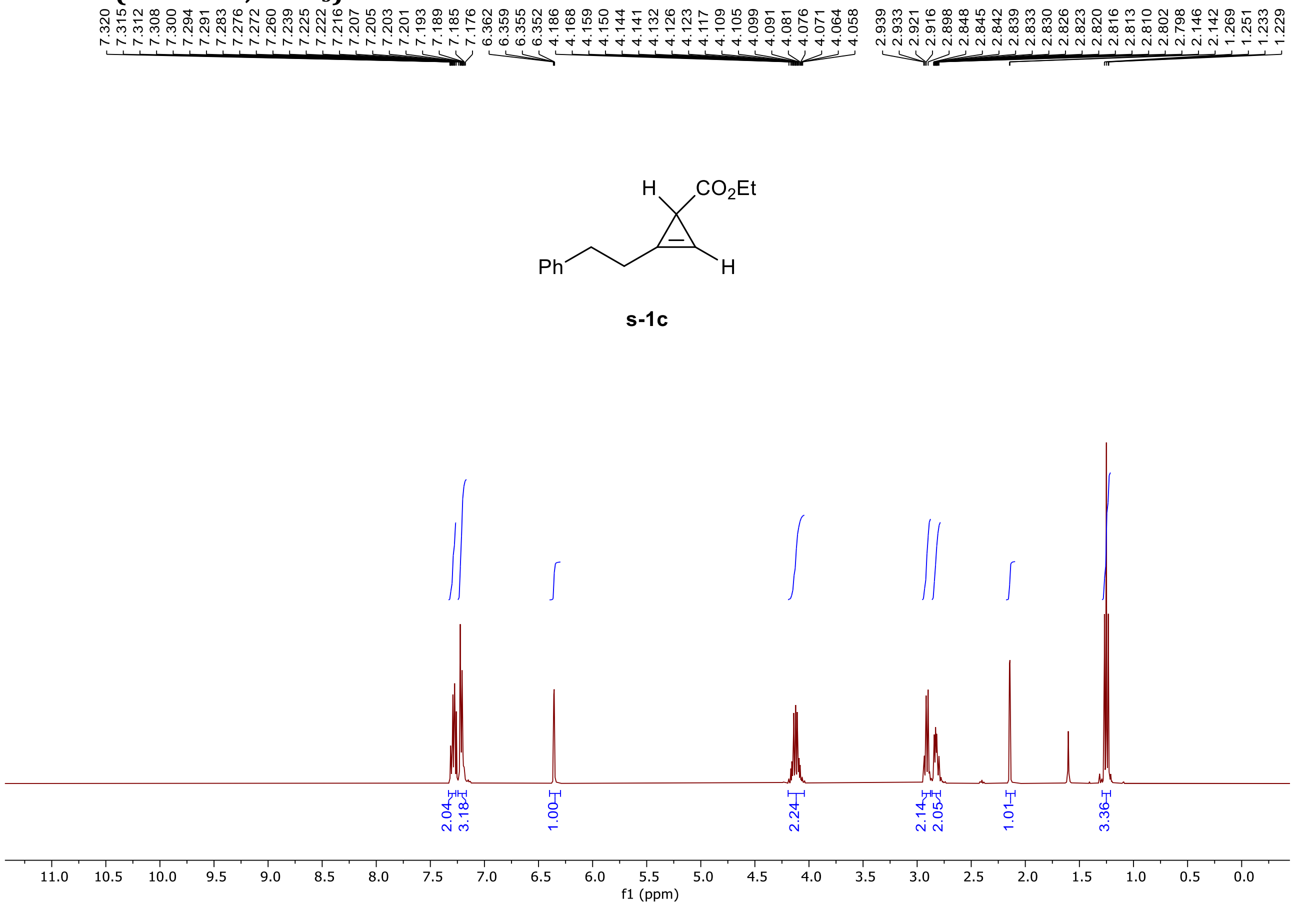

s-1c

^{1}H NMR (400 MHz, $CDCl_3$) of *s*-1d

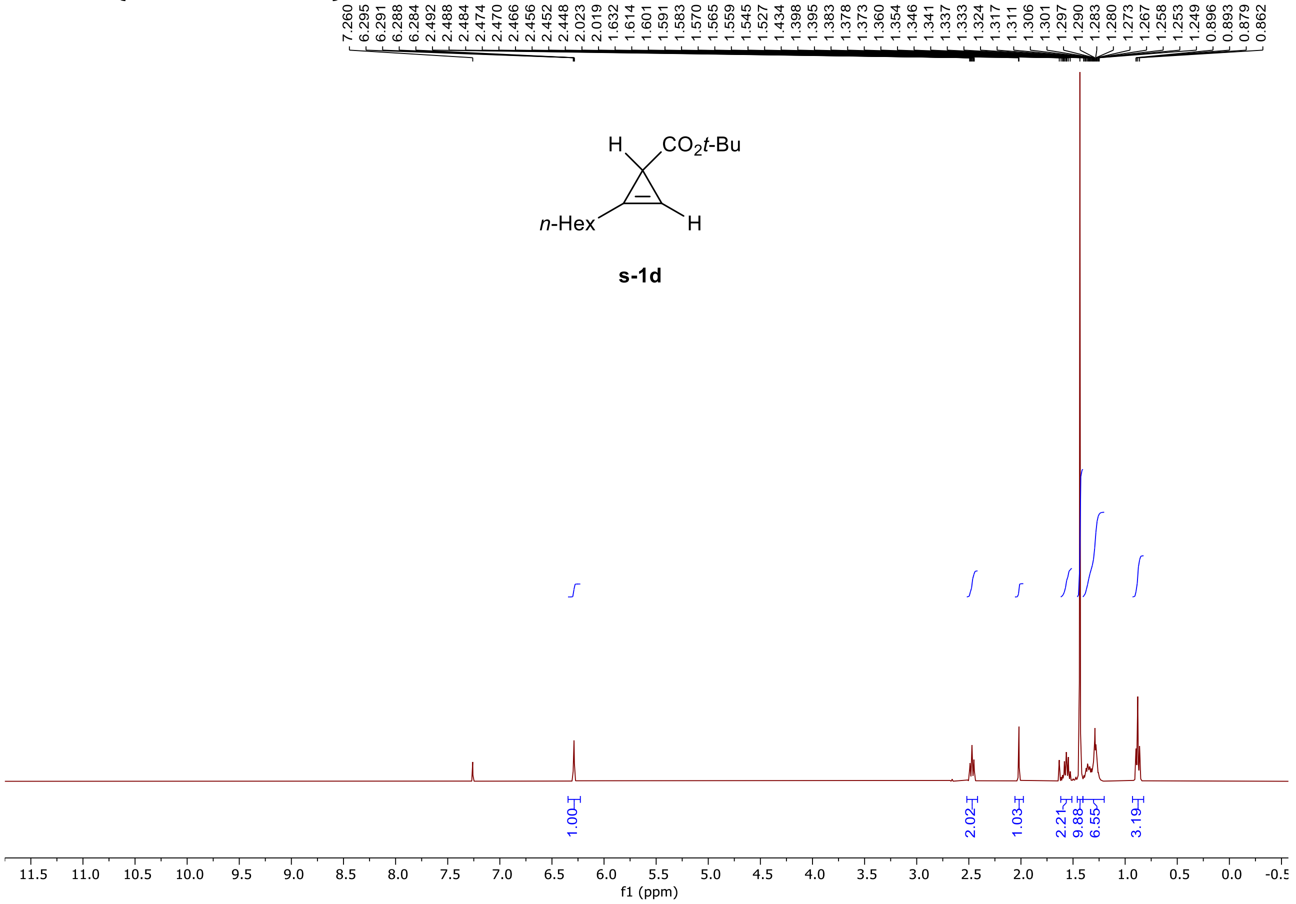

s-1d

^{13}C NMR (101 MHz, $CDCl_3$) of s-1d

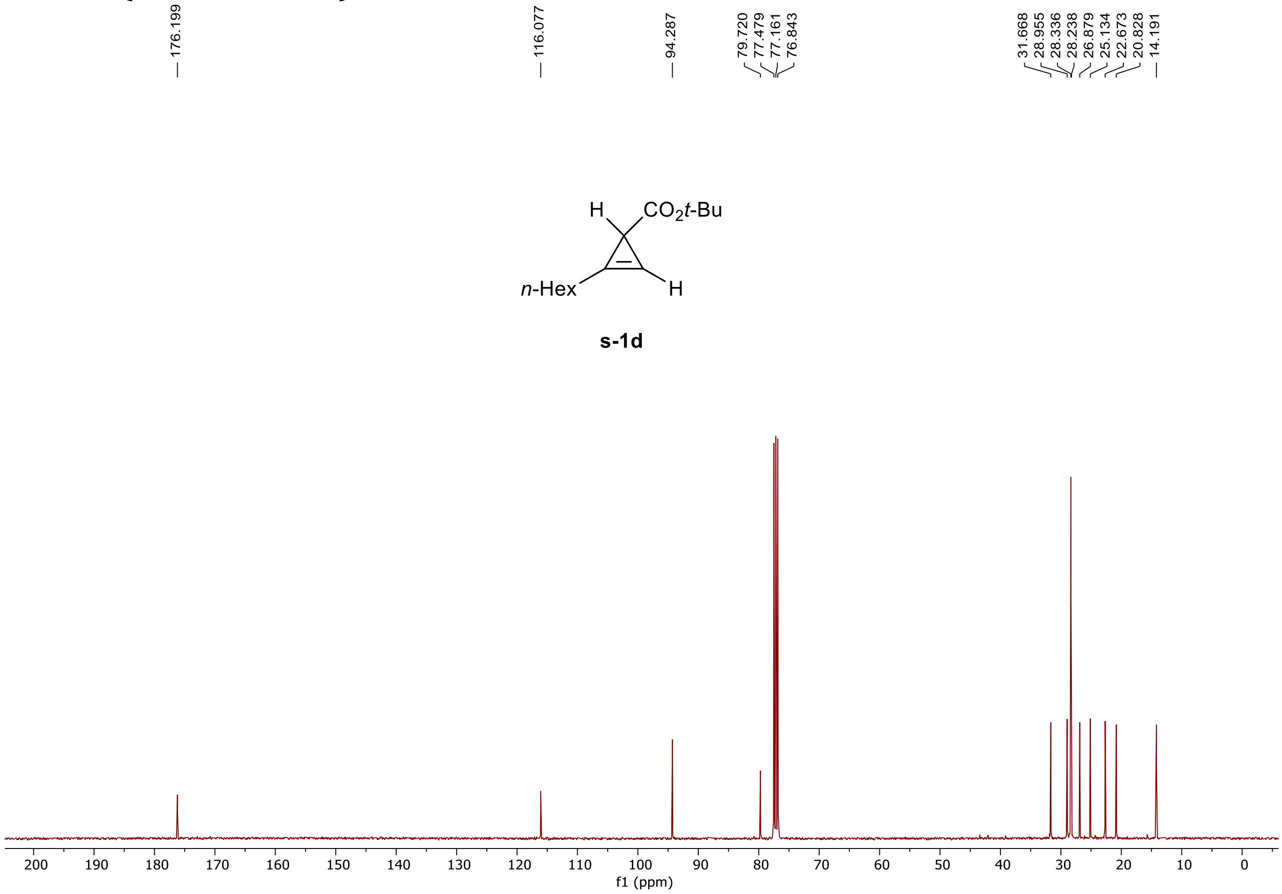

^{1}H NMR (400 MHz, $CDCl_3$) of s-1e

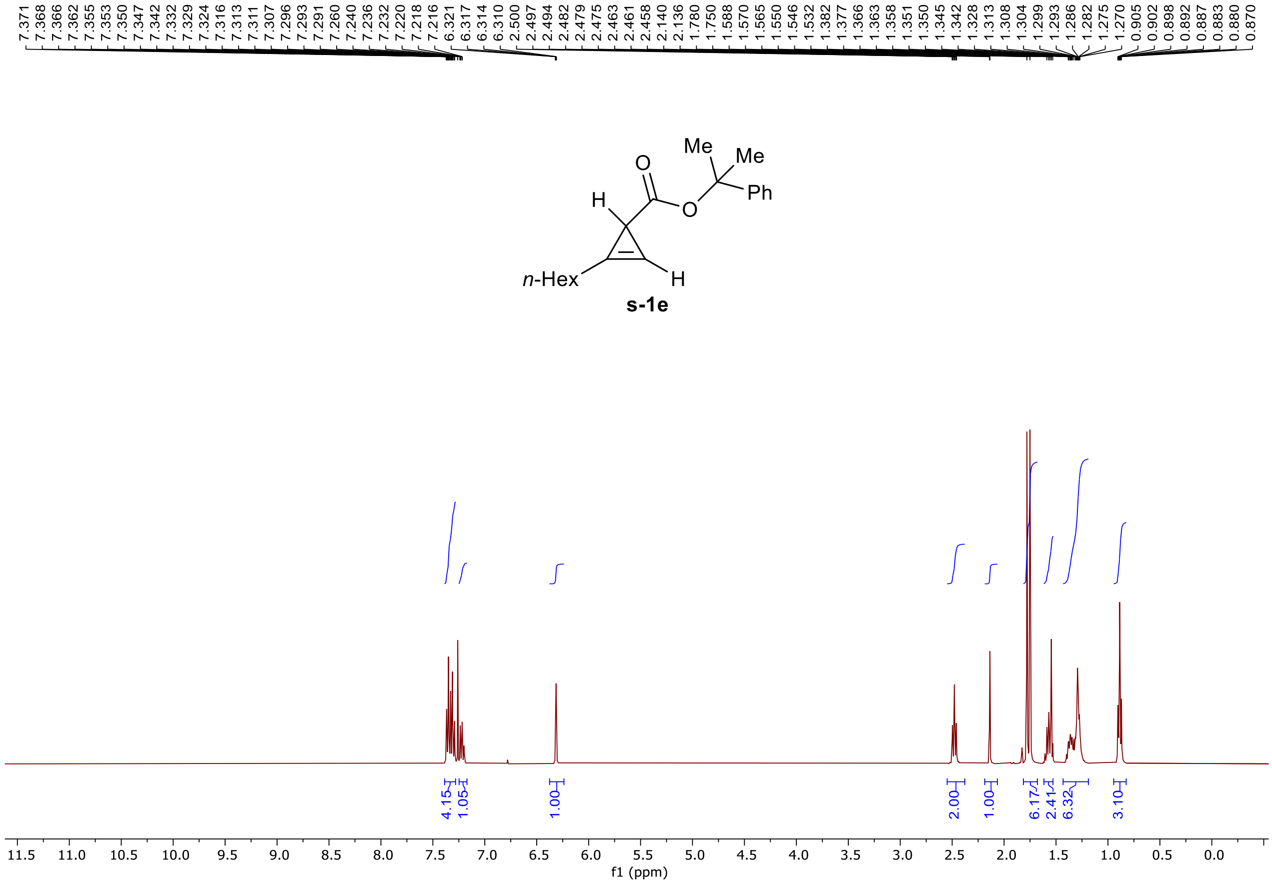

^{13}C NMR (101 MHz, $CDCl_3$) of s-1e

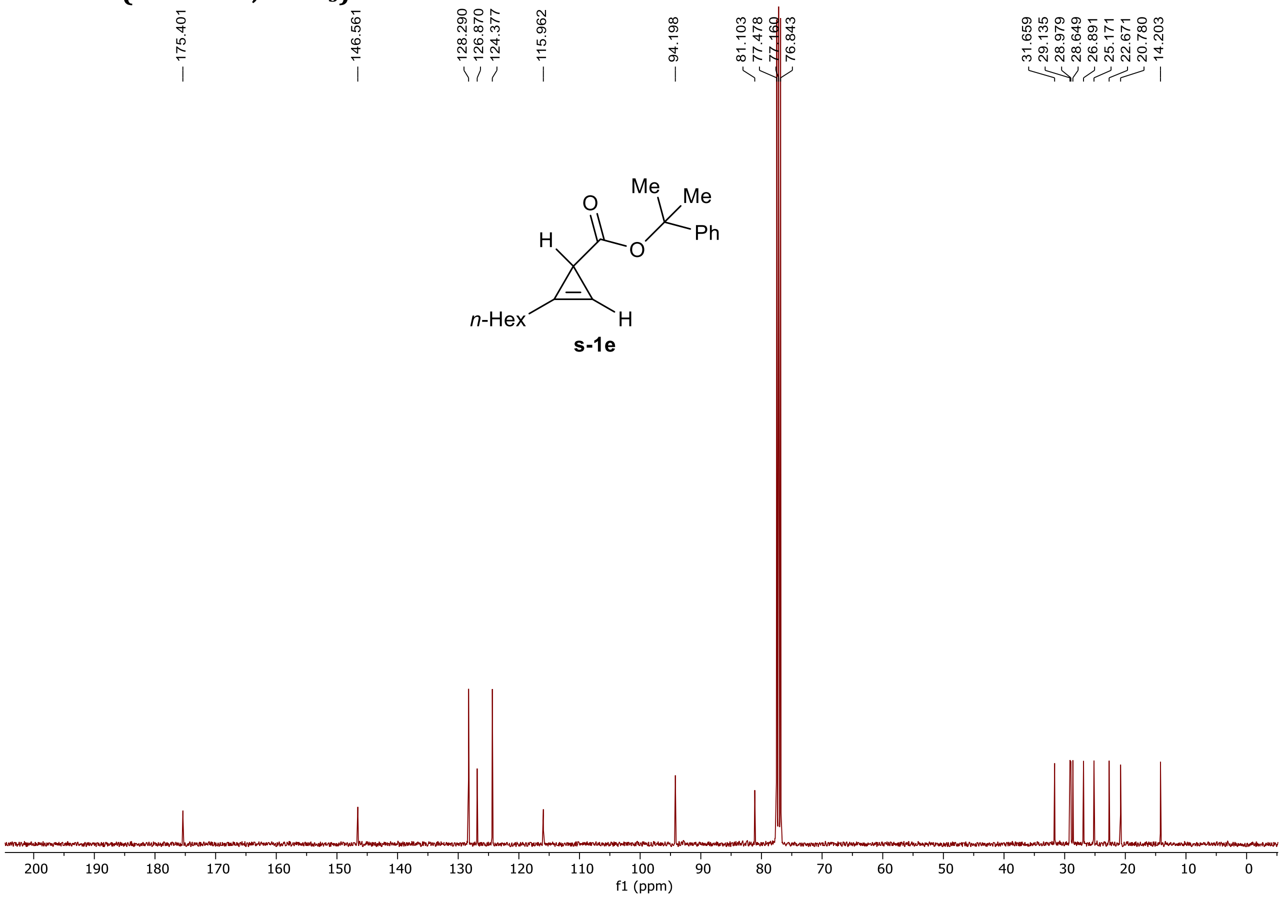

^{1}H NMR (400 MHz, $CDCl_3$) of s-1f

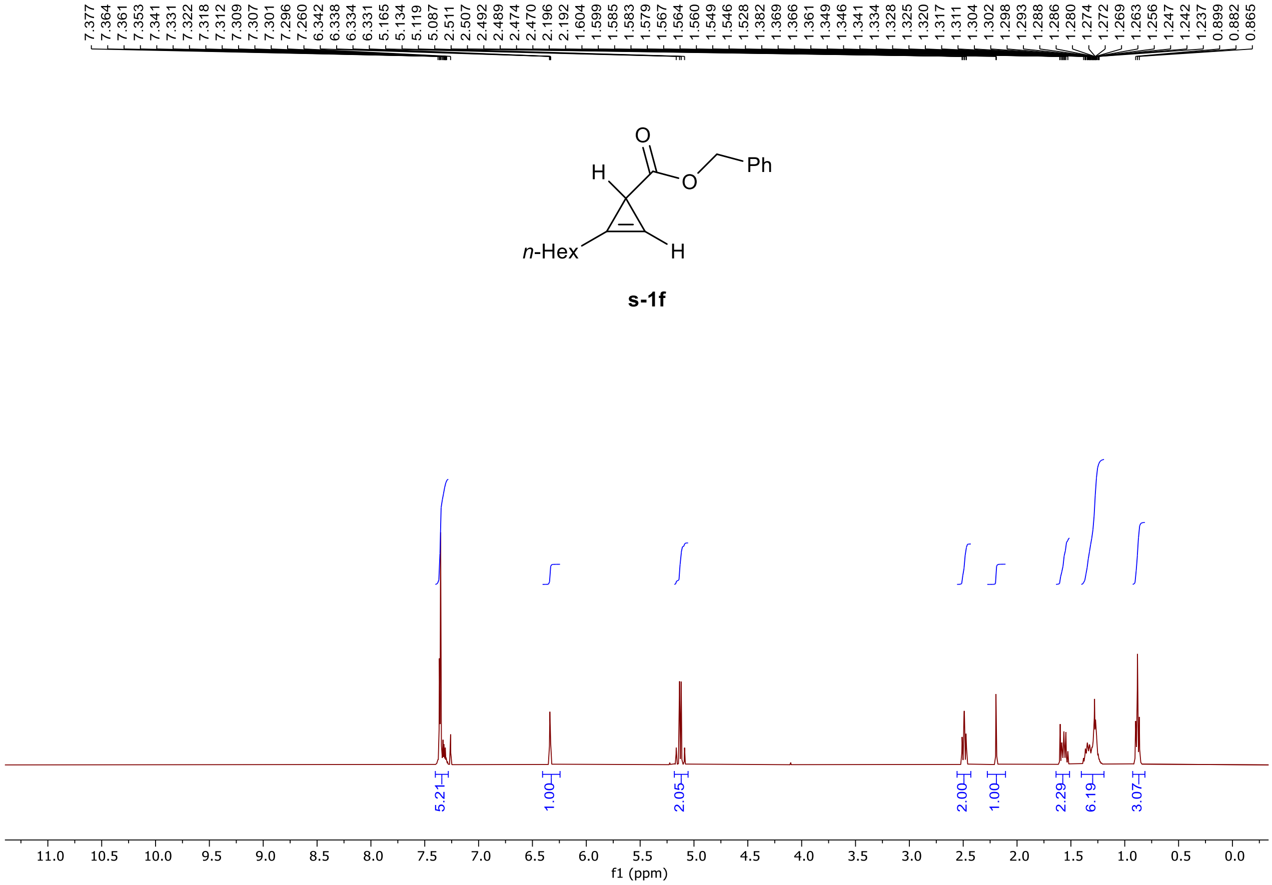

^{13}C NMR (101 MHz, $CDCl_3$) of s-1f

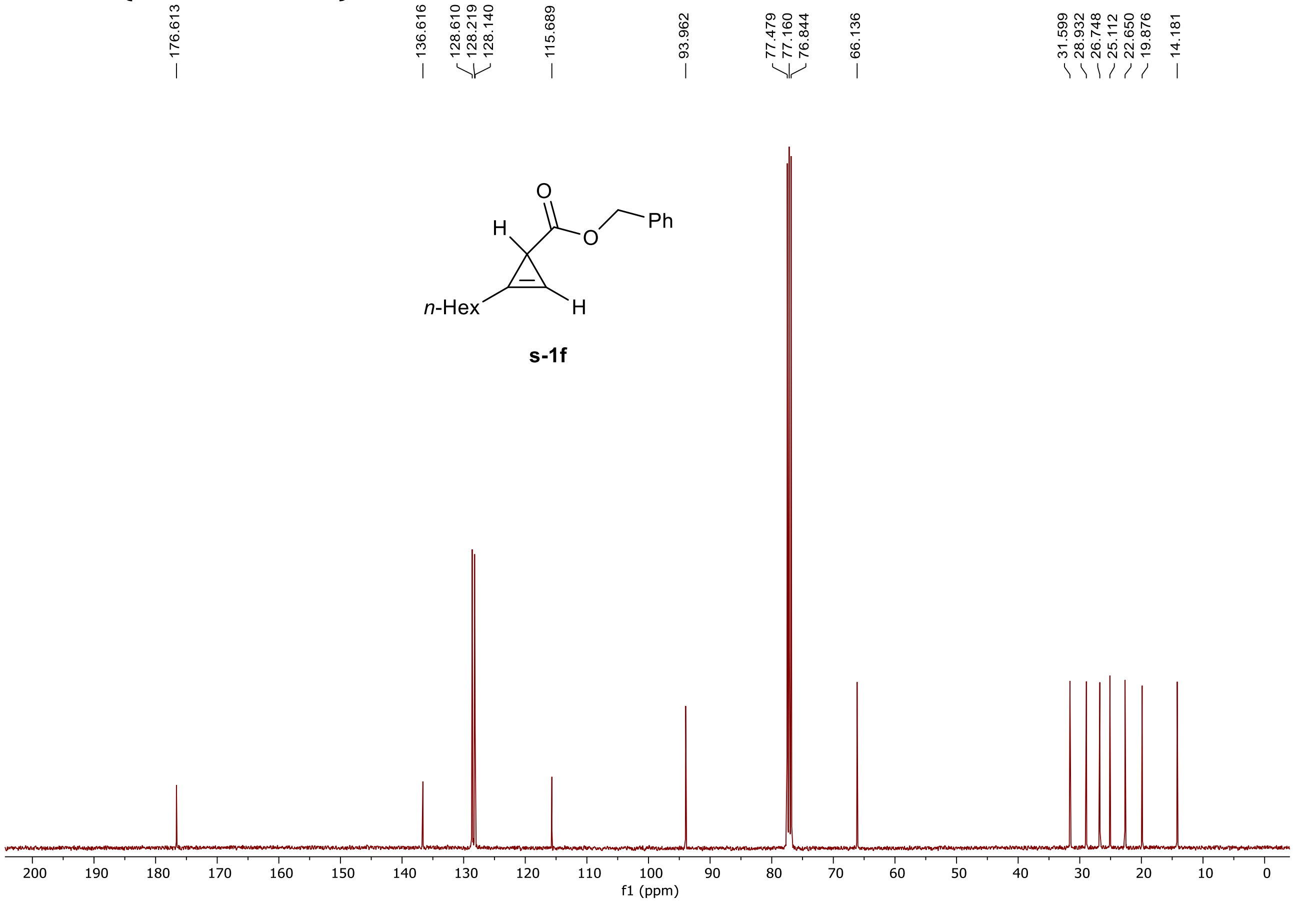

^{1}H NMR (400 MHz, $CDCl_3$) of s-1g

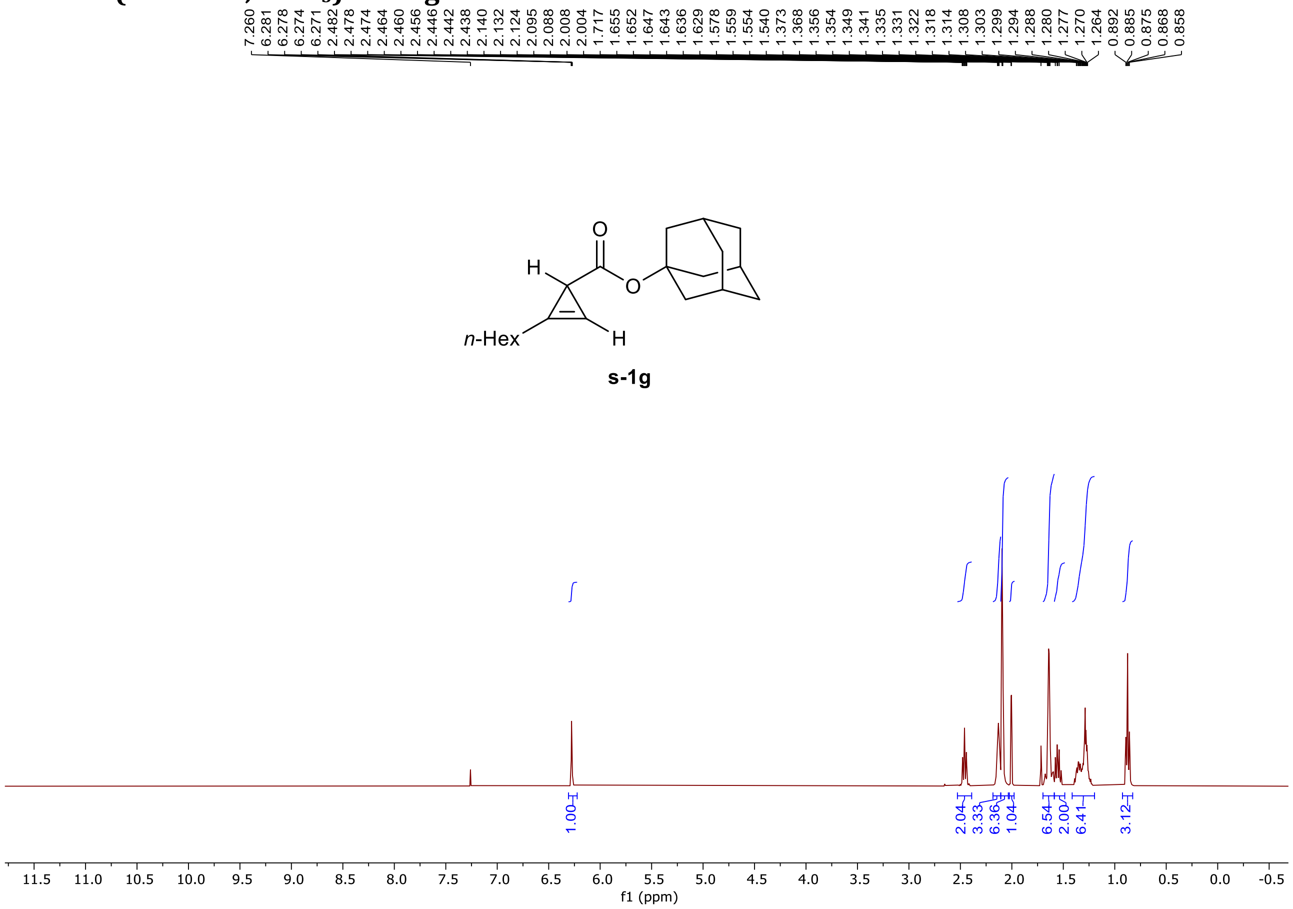

^{13}C NMR (101 MHz, $CDCl_3$) of s-1g

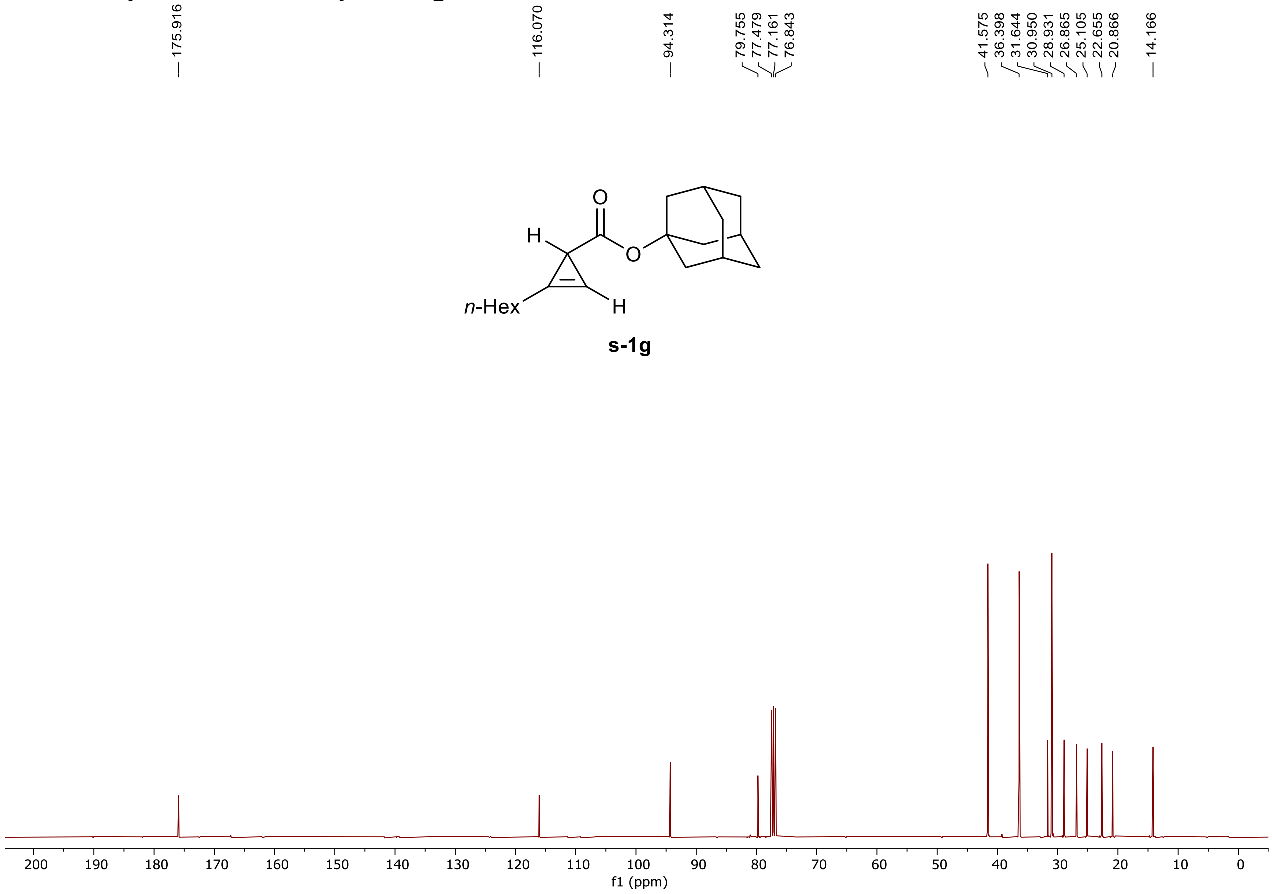

^{1}H NMR (400 MHz, $CDCl_3$) of s-1h

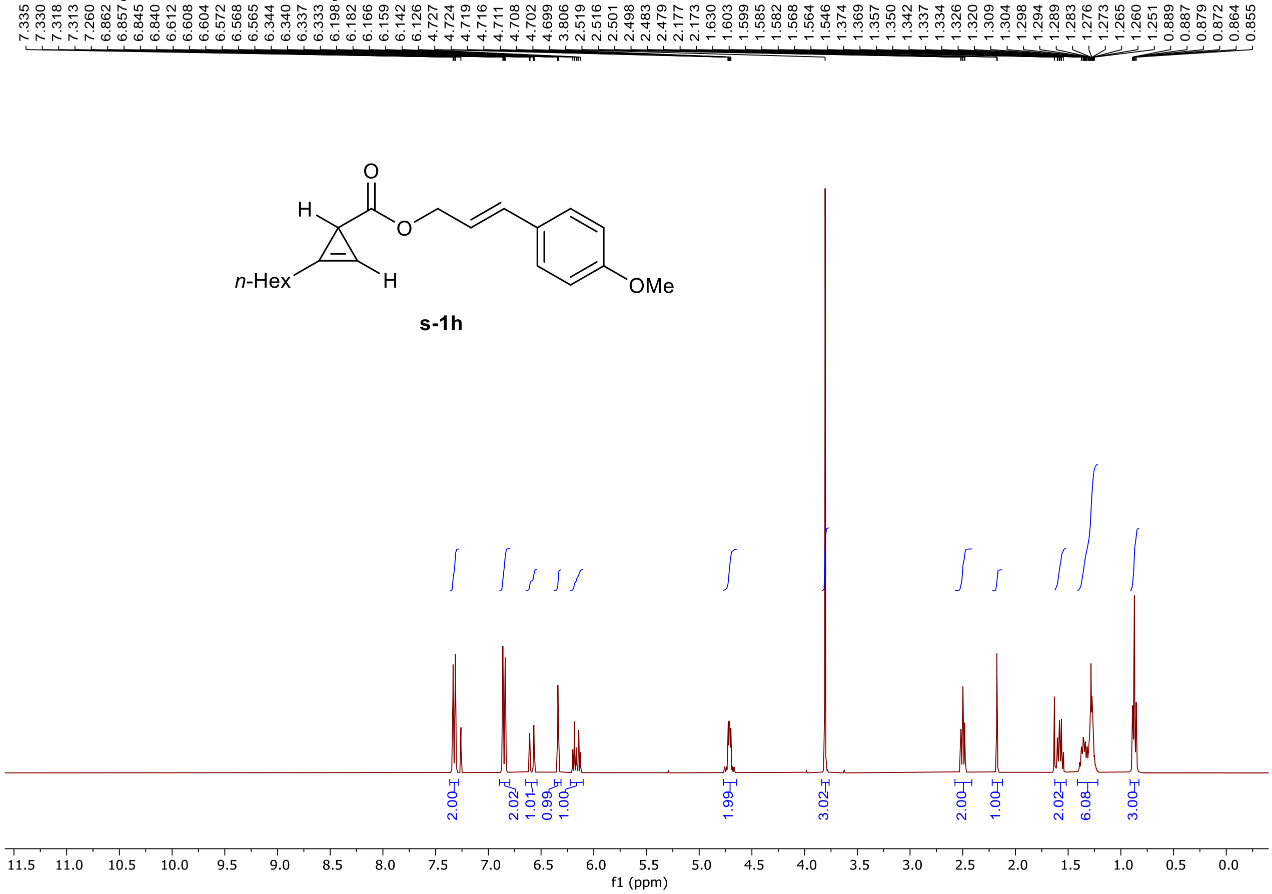

^{13}C NMR (101 MHz, $CDCl_3$) of s-1h

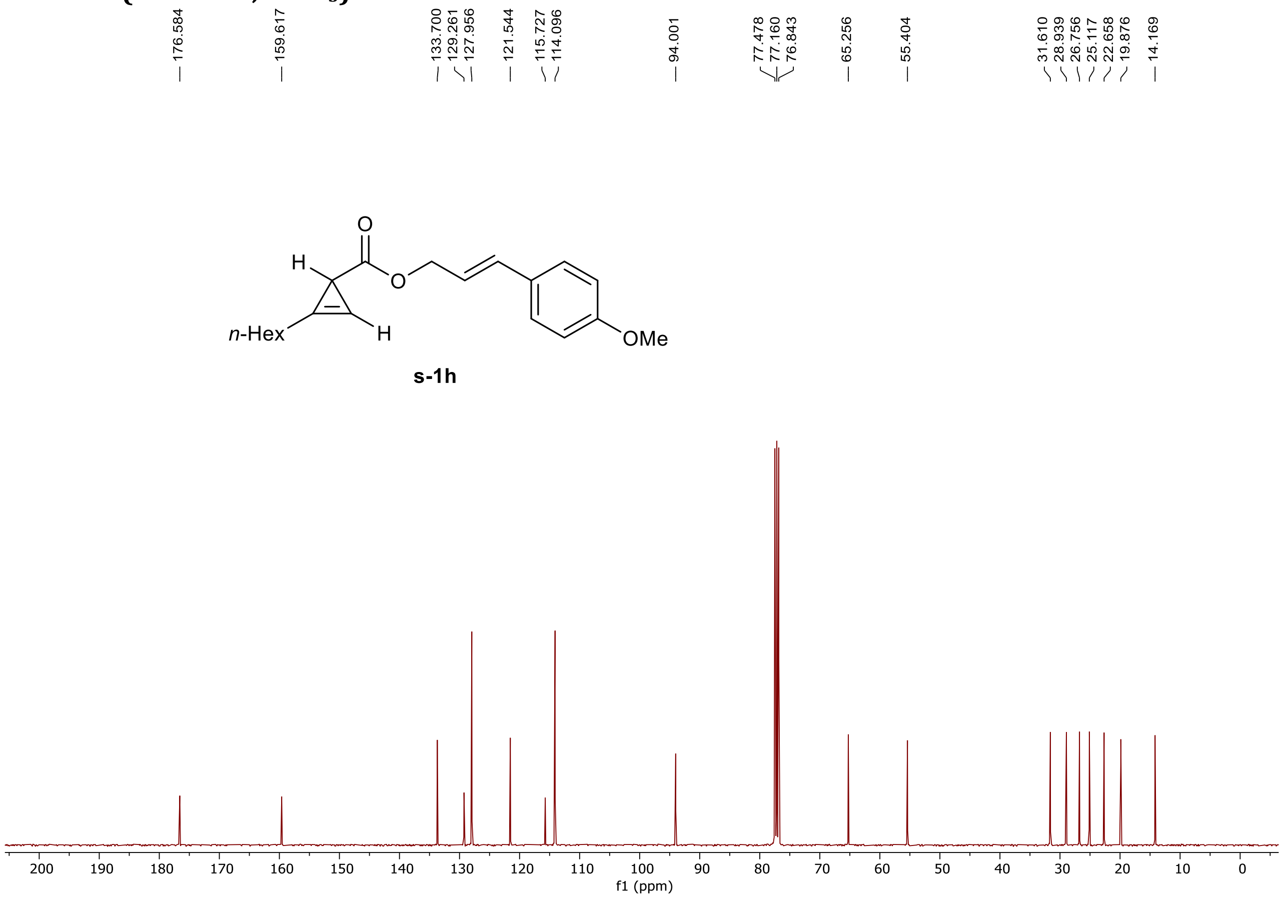

s-1h

^{1}H NMR (400 MHz, $CDCl_3$) of s-1i

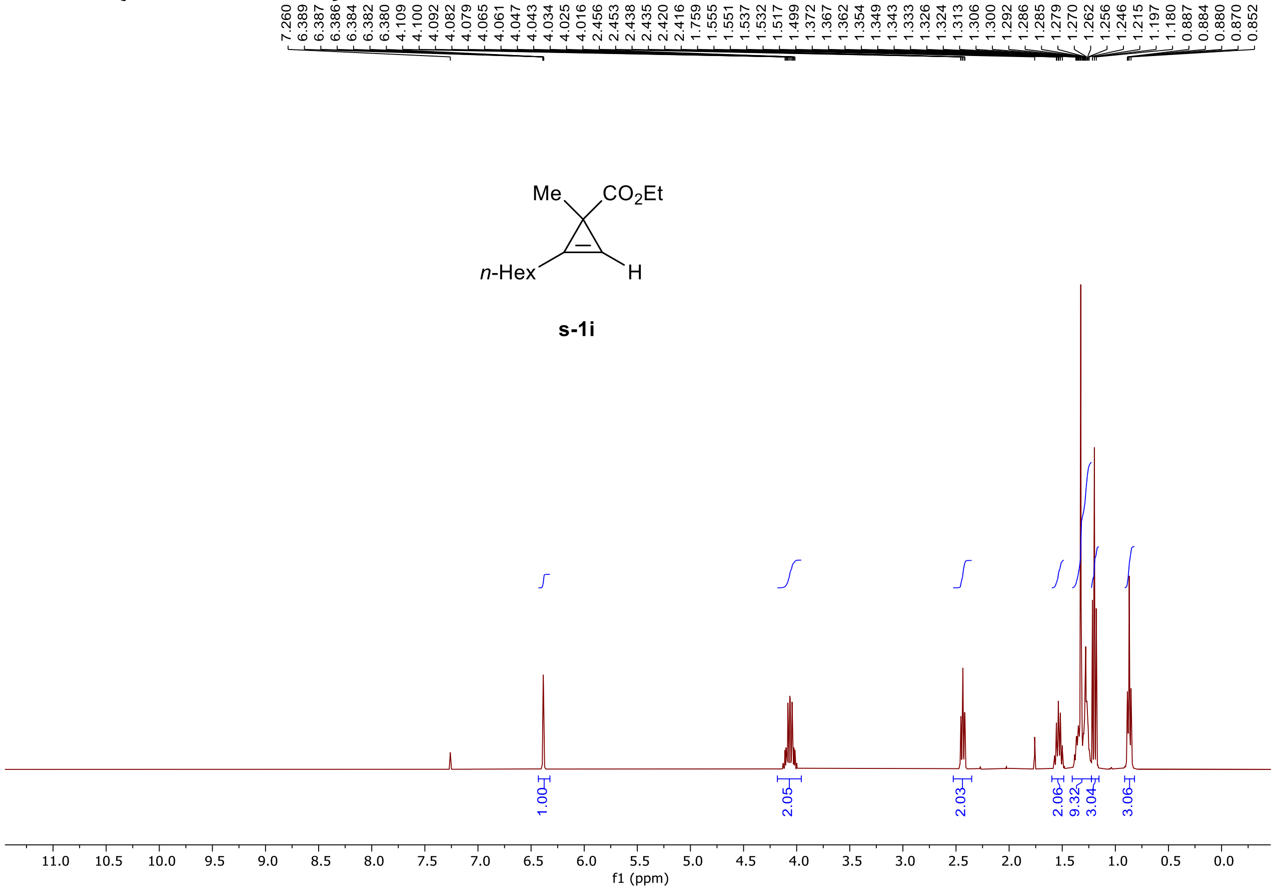

s-1i

^{13}C NMR (101 MHz, $CDCl_3$) of s-1i

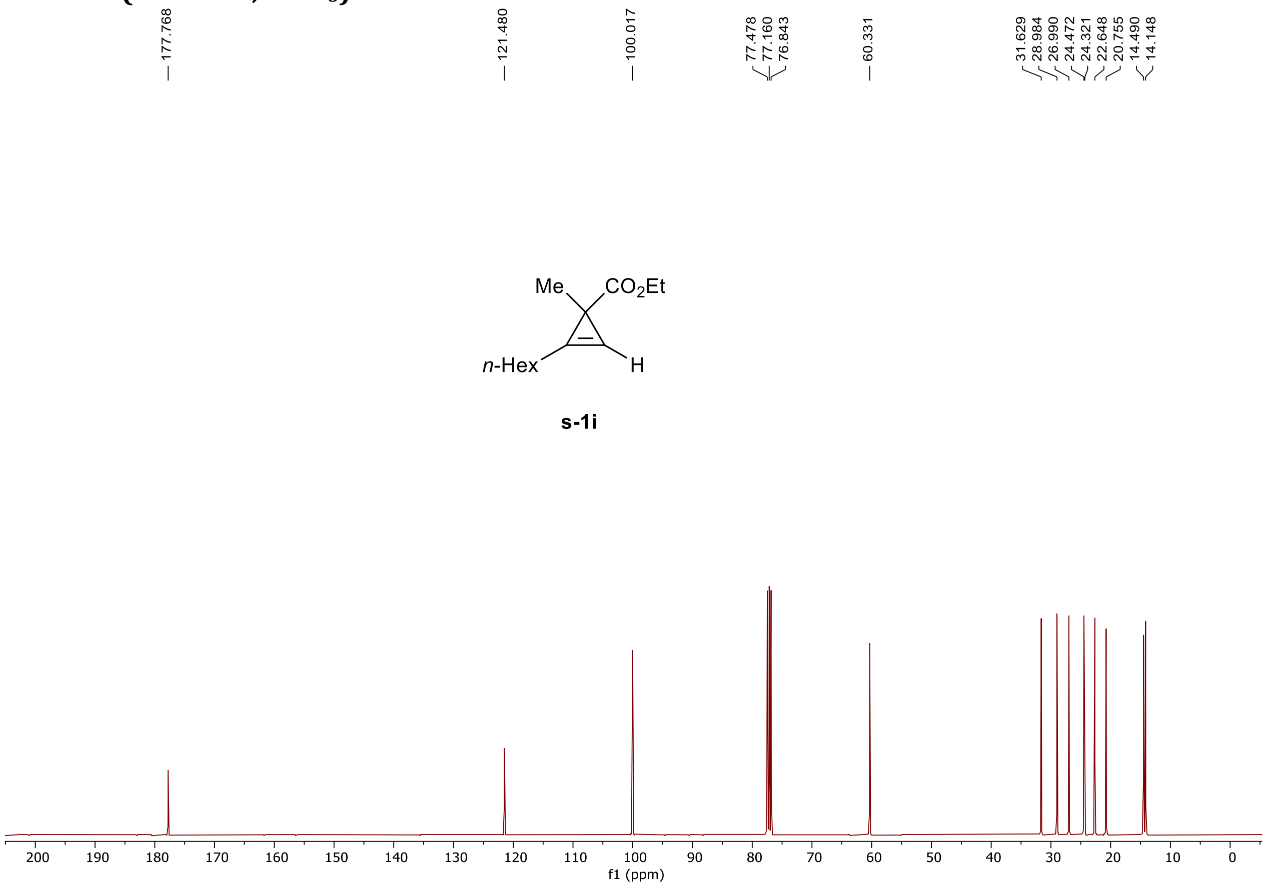

^{1}H NMR (400 MHz, $CDCl_3$) of s-1j

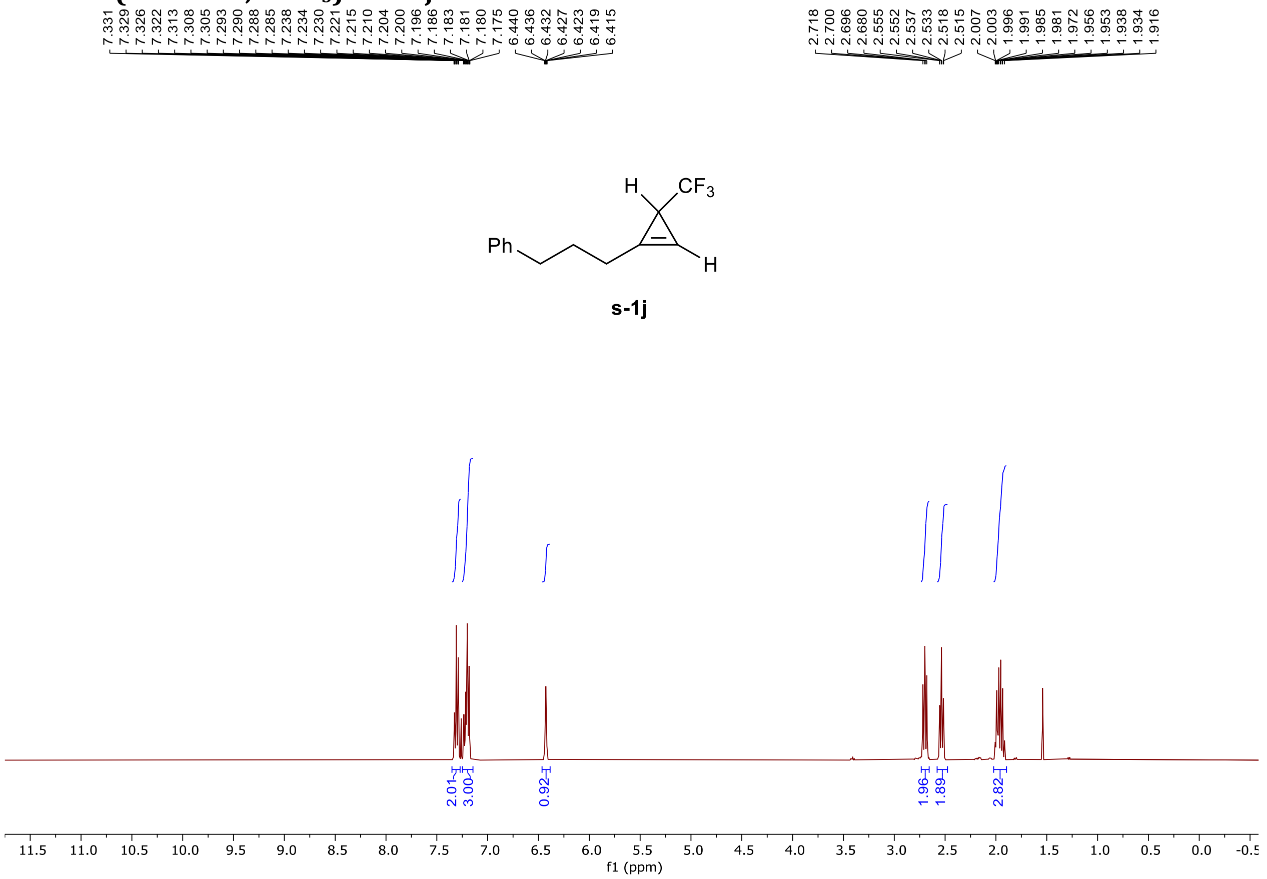

^{13}C NMR (101 MHz, $CDCl_3$) of s-1j

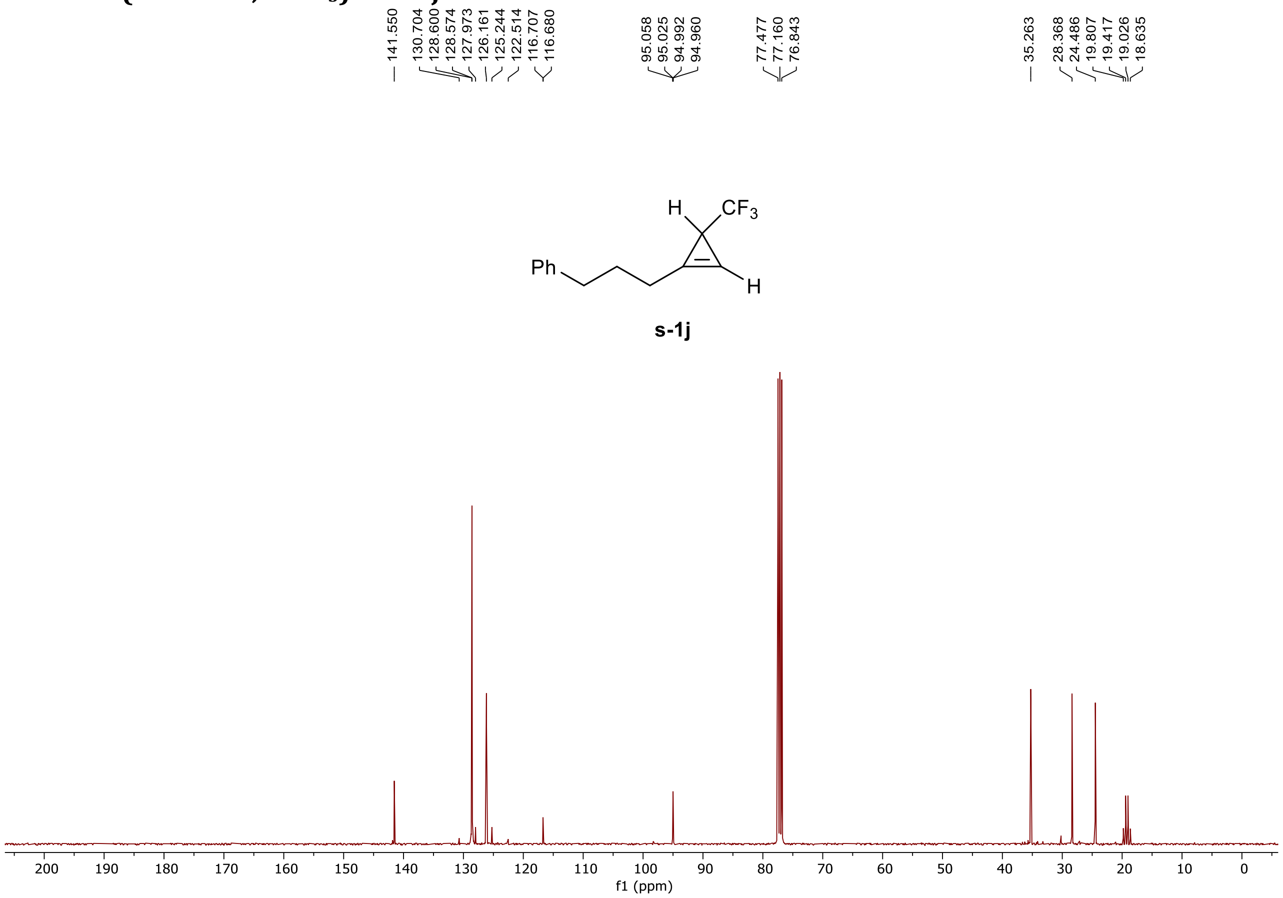

^{19}F NMR (377 MHz, $CDCl_3$) of s-1j

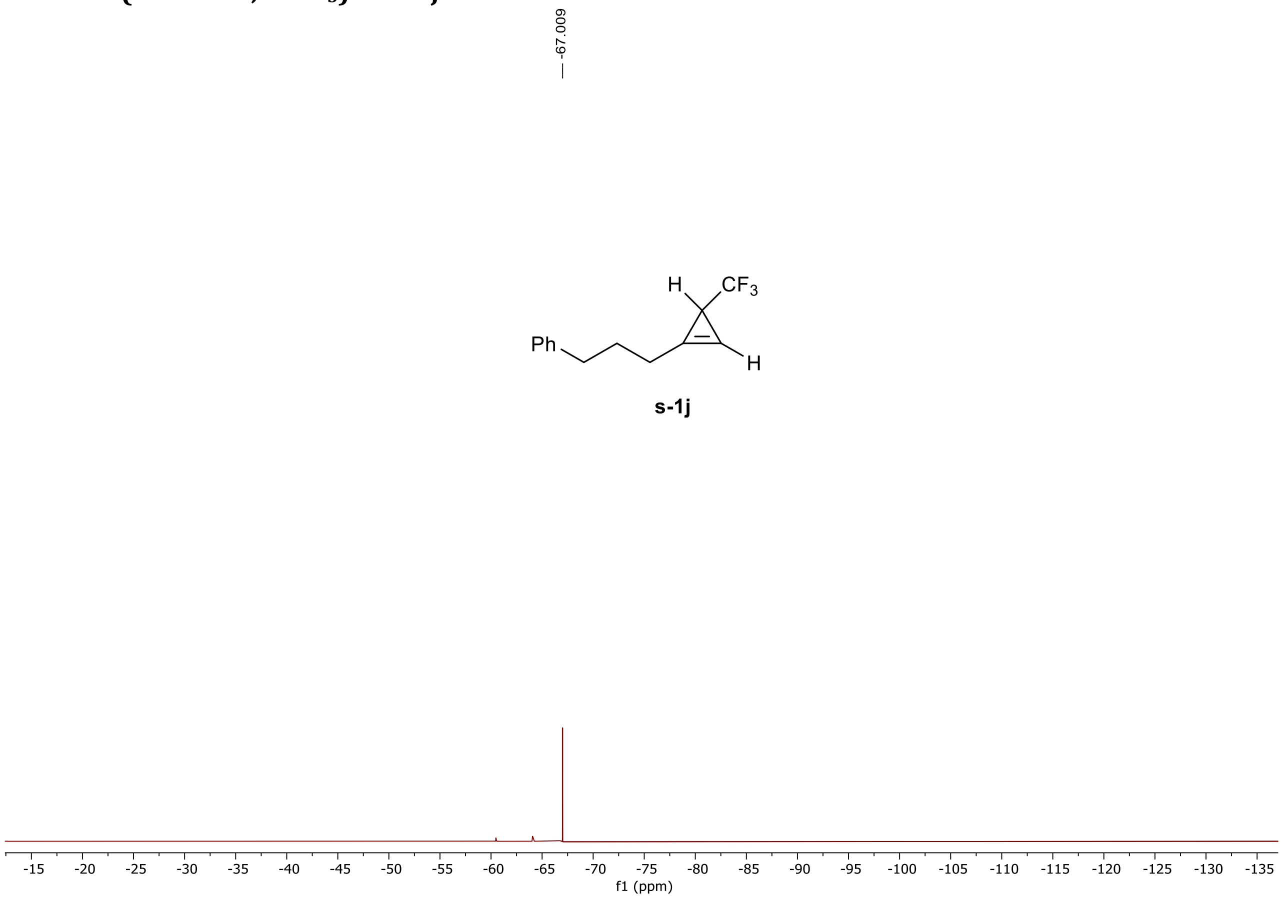

^{1}H NMR (400 MHz, $CDCl_3$) of s-1k

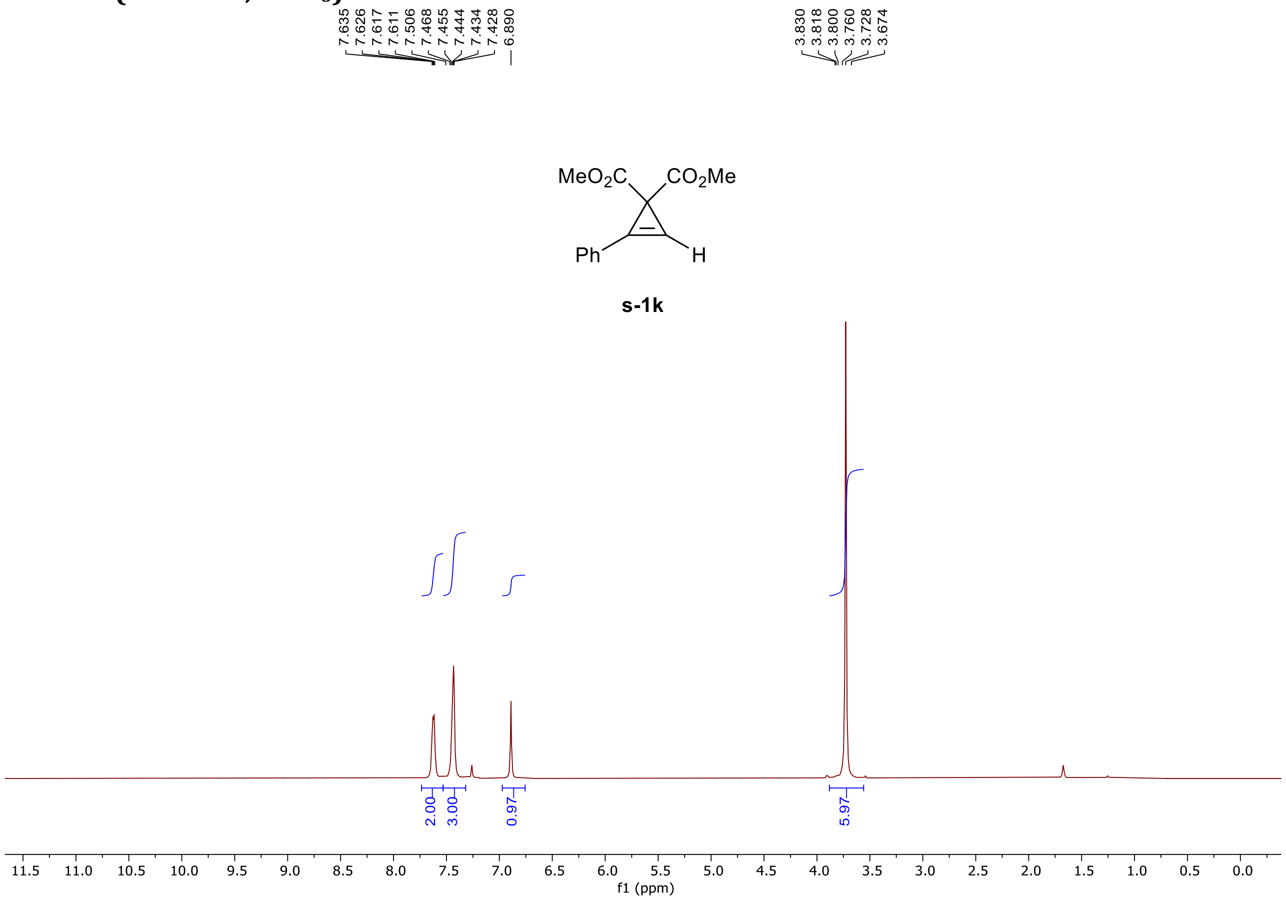

^{13}C NMR (101 MHz, $CDCl_3$) of s-1k

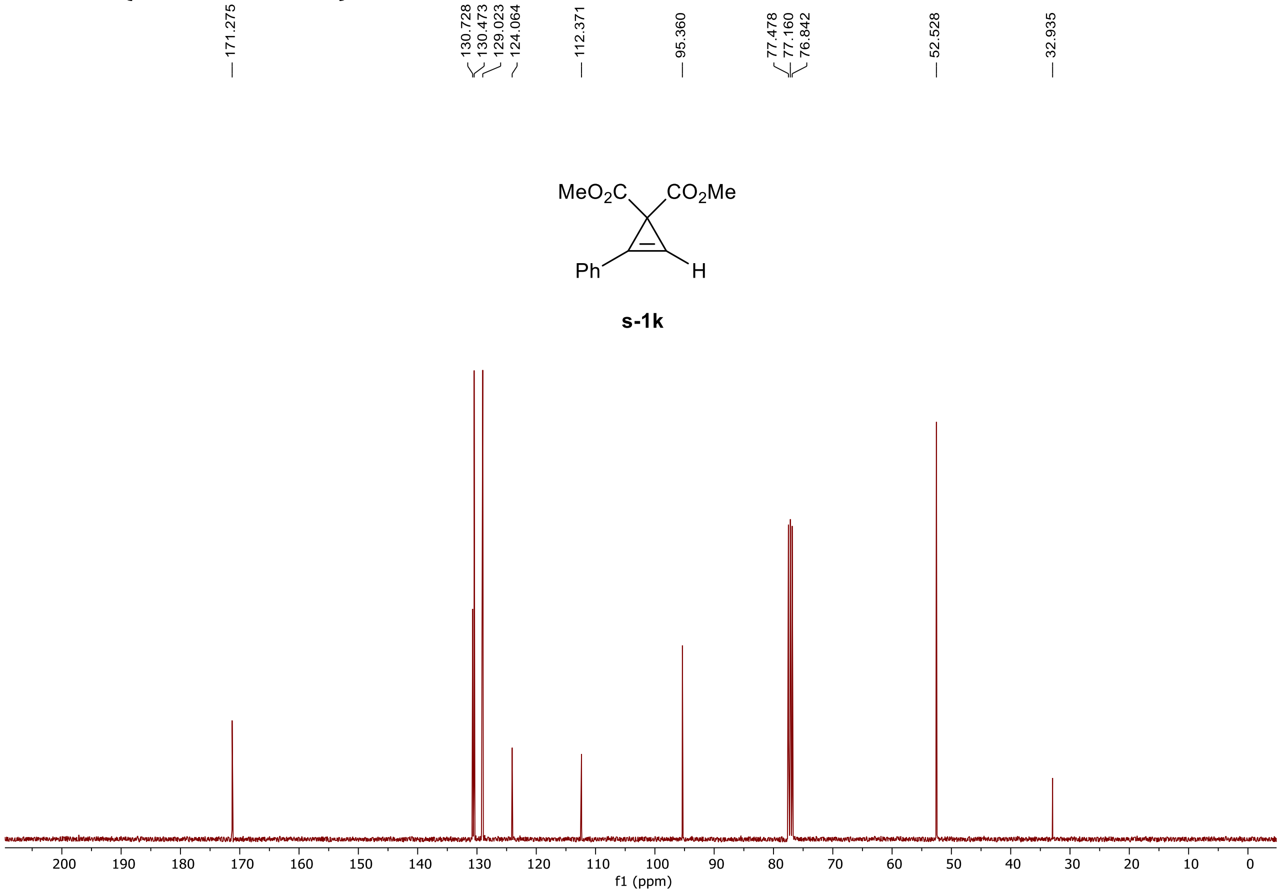

^{1}H NMR (400 MHz, $CDCl_3$) of s-1l

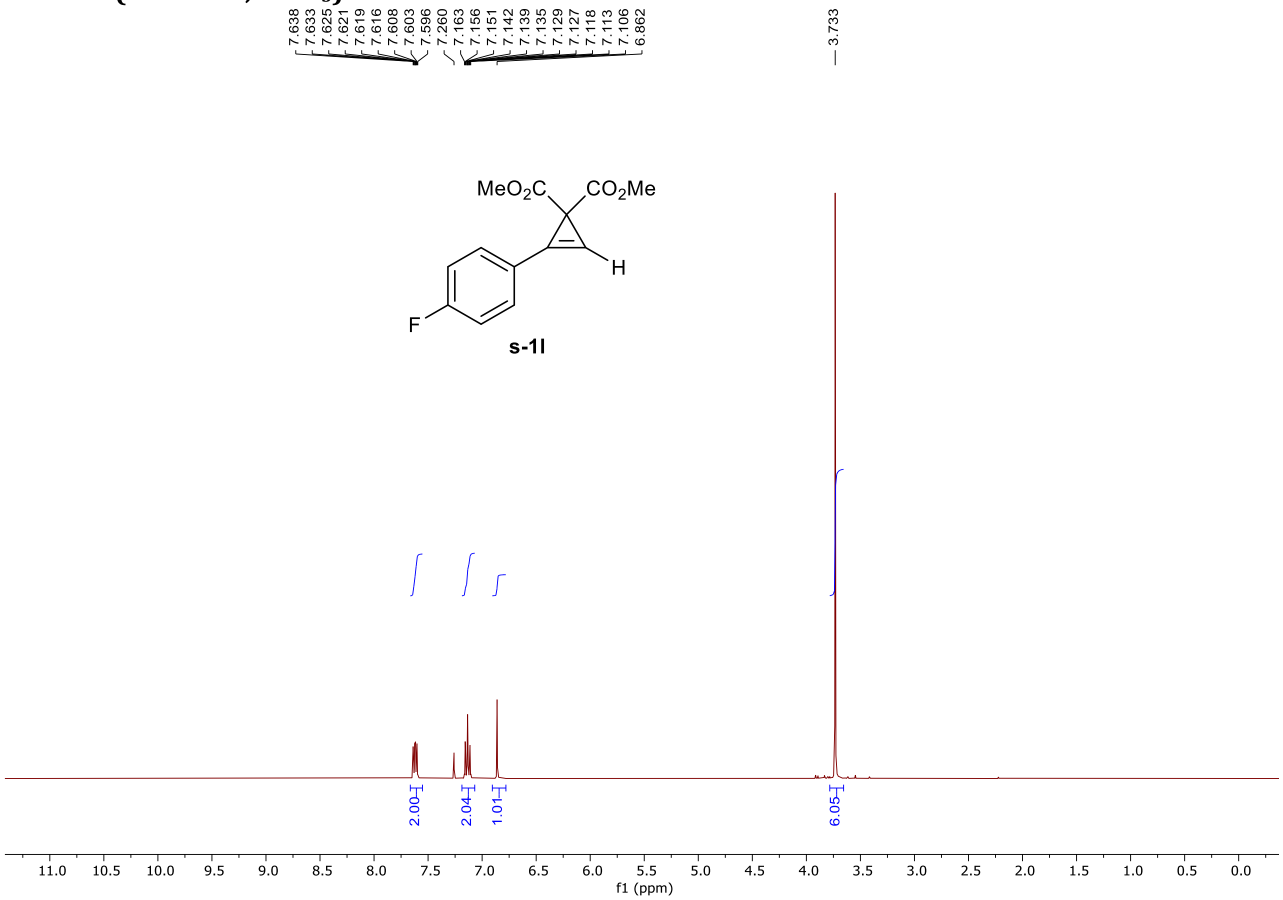

^{13}C NMR (101 MHz, $CDCl_3$) of s-1l

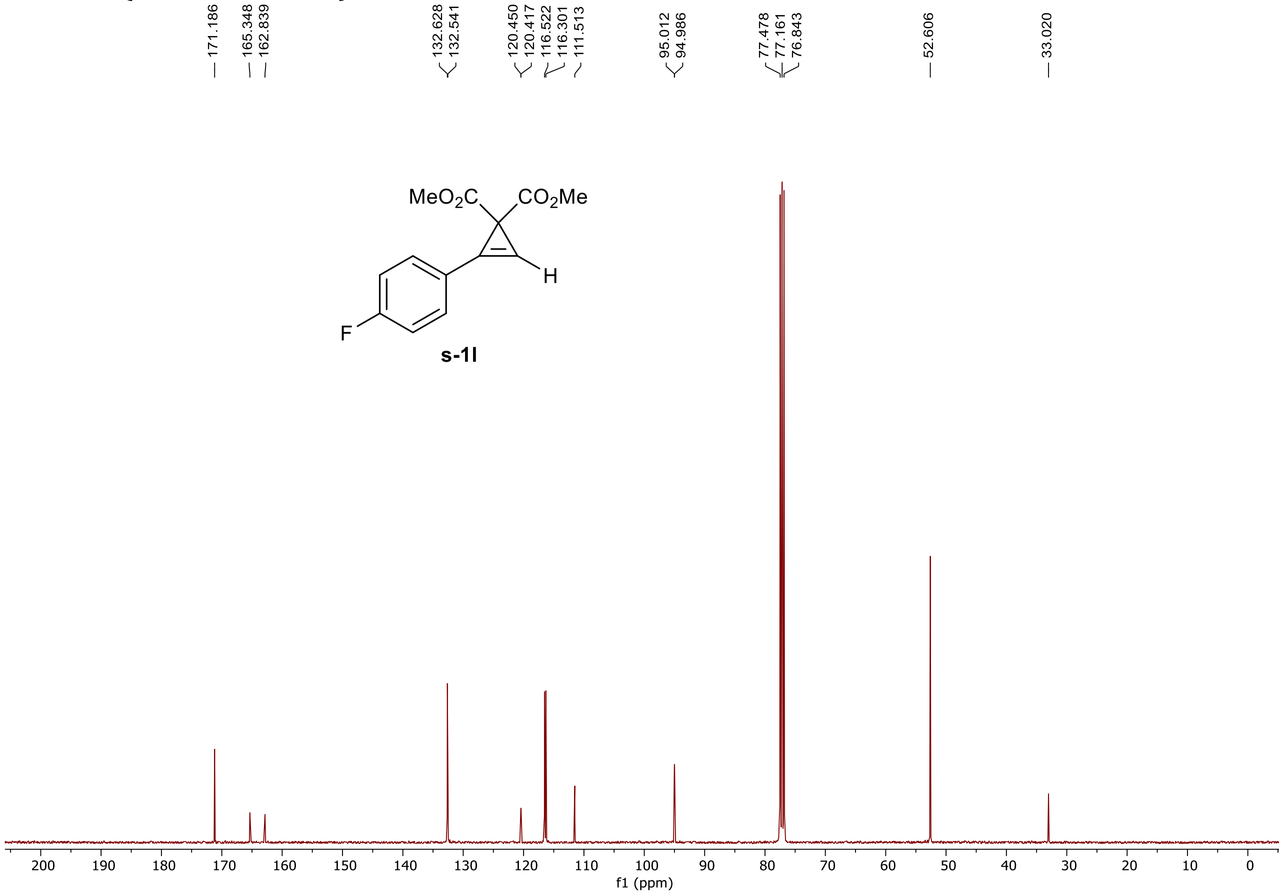

^{19}F NMR (377 MHz, $CDCl_3$) of s-1l

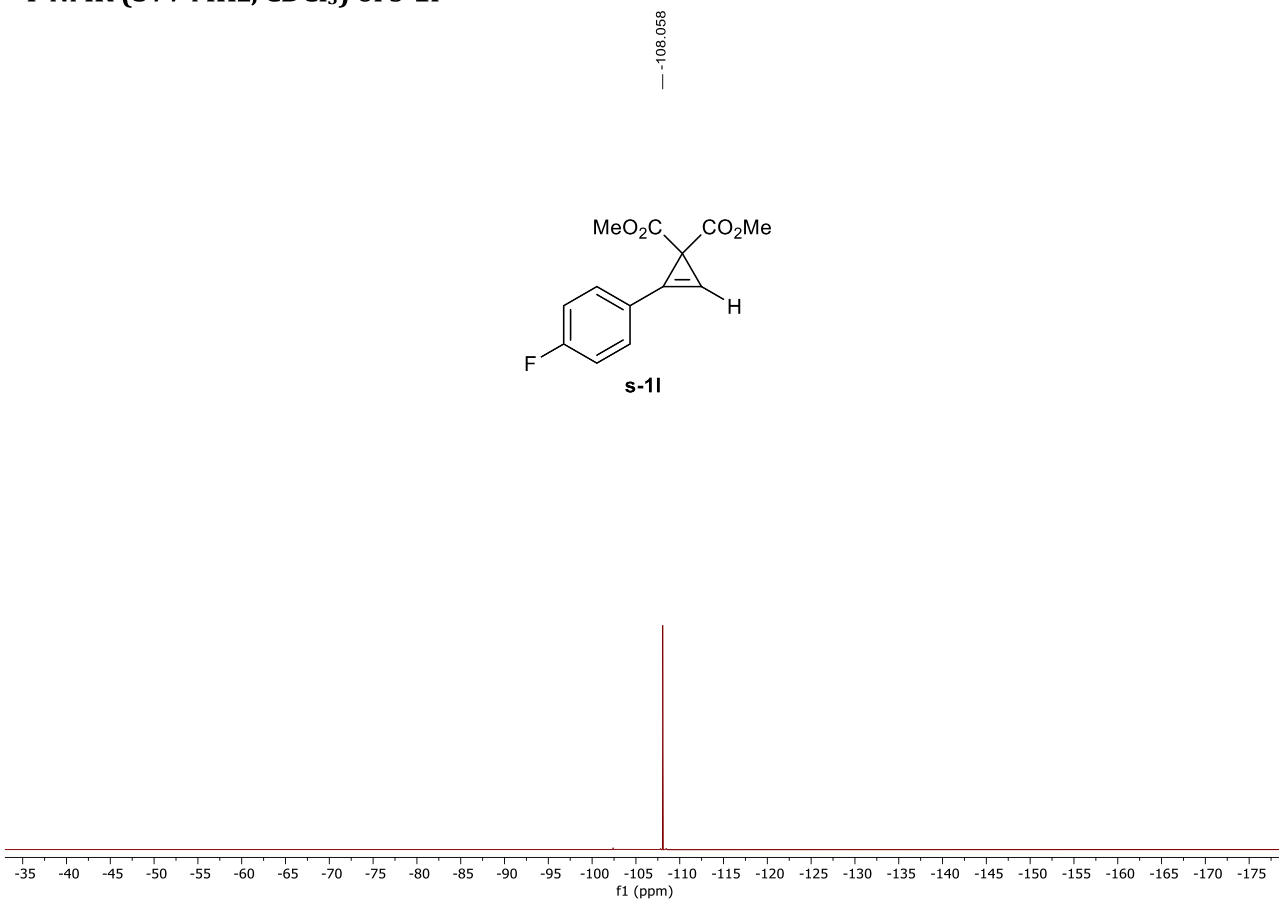

^{1}H NMR (400 MHz, $CDCl_3$) of s-1m

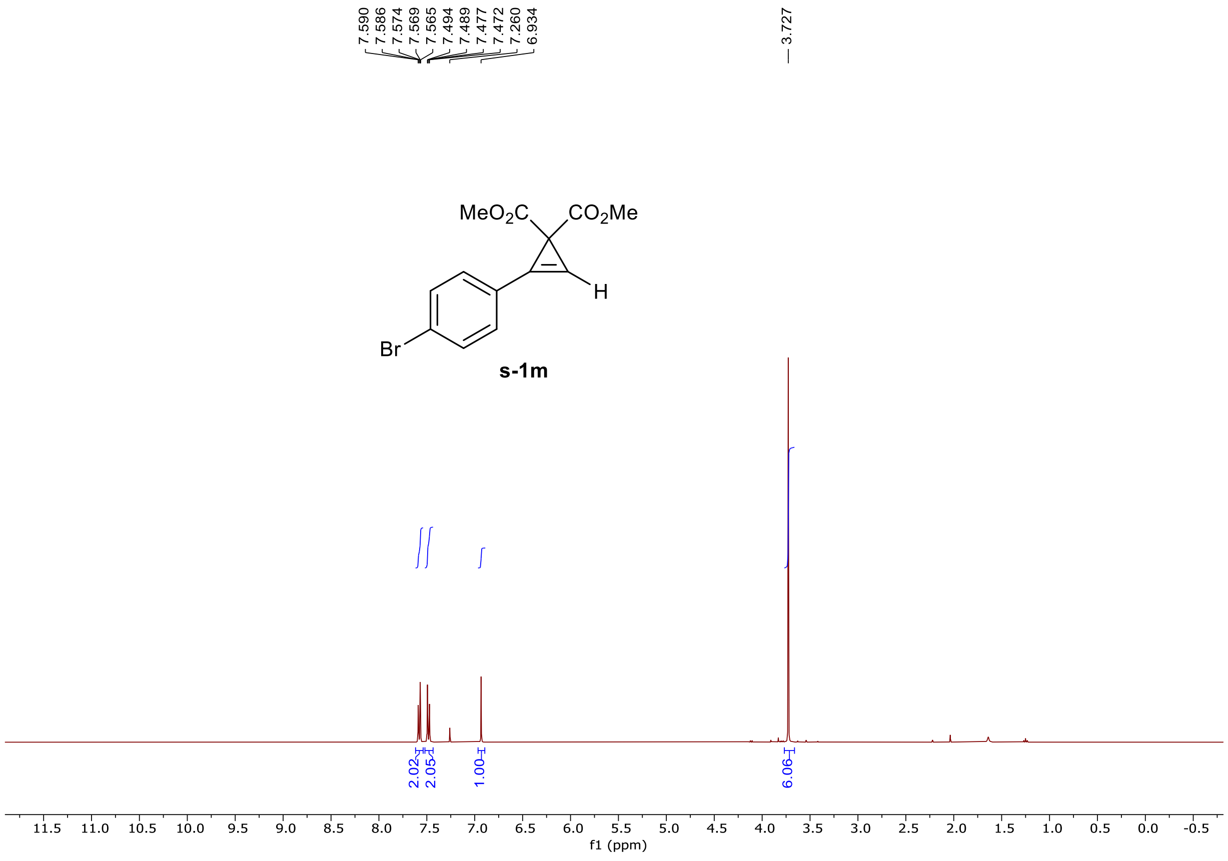

^{13}C NMR (101 MHz, $CDCl_3$) of s-1m

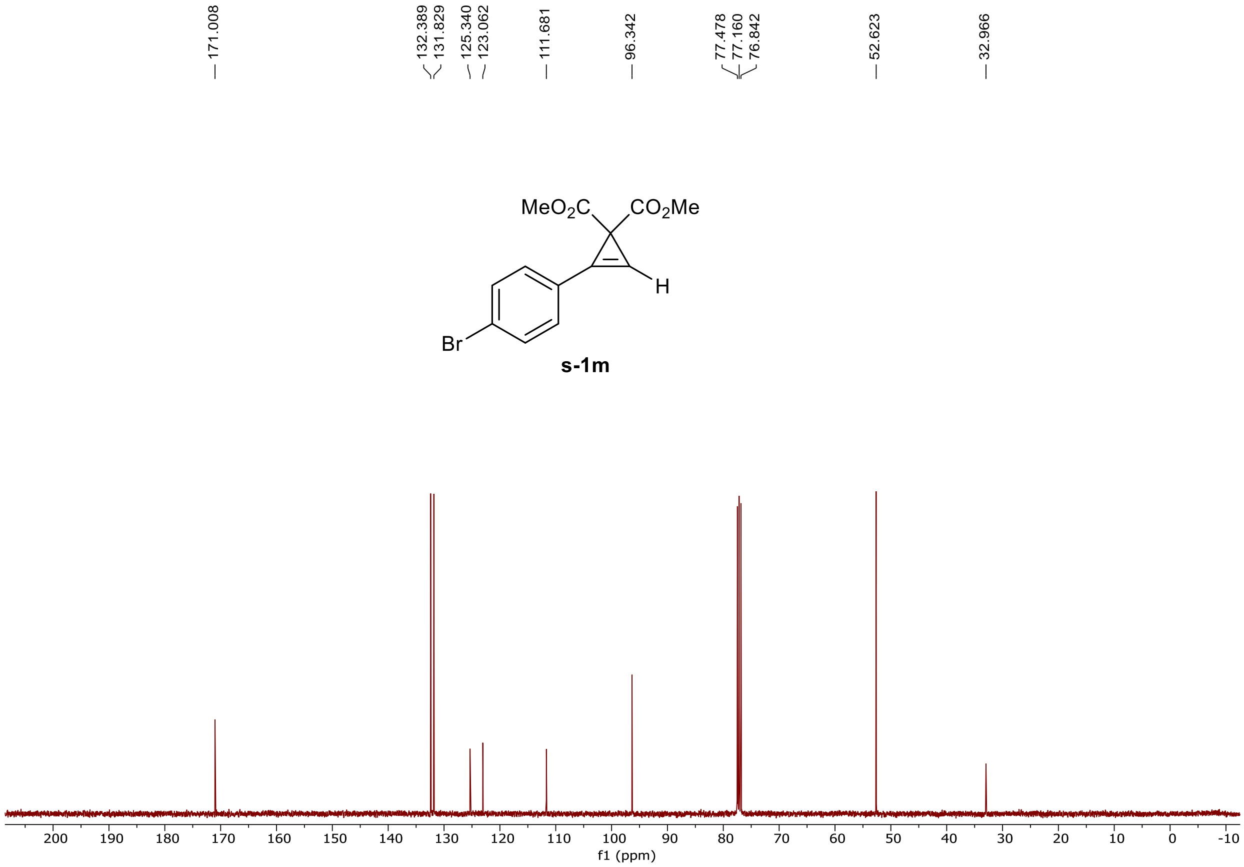

^{1}H NMR (400 MHz, $CDCl_3$) of s-1n

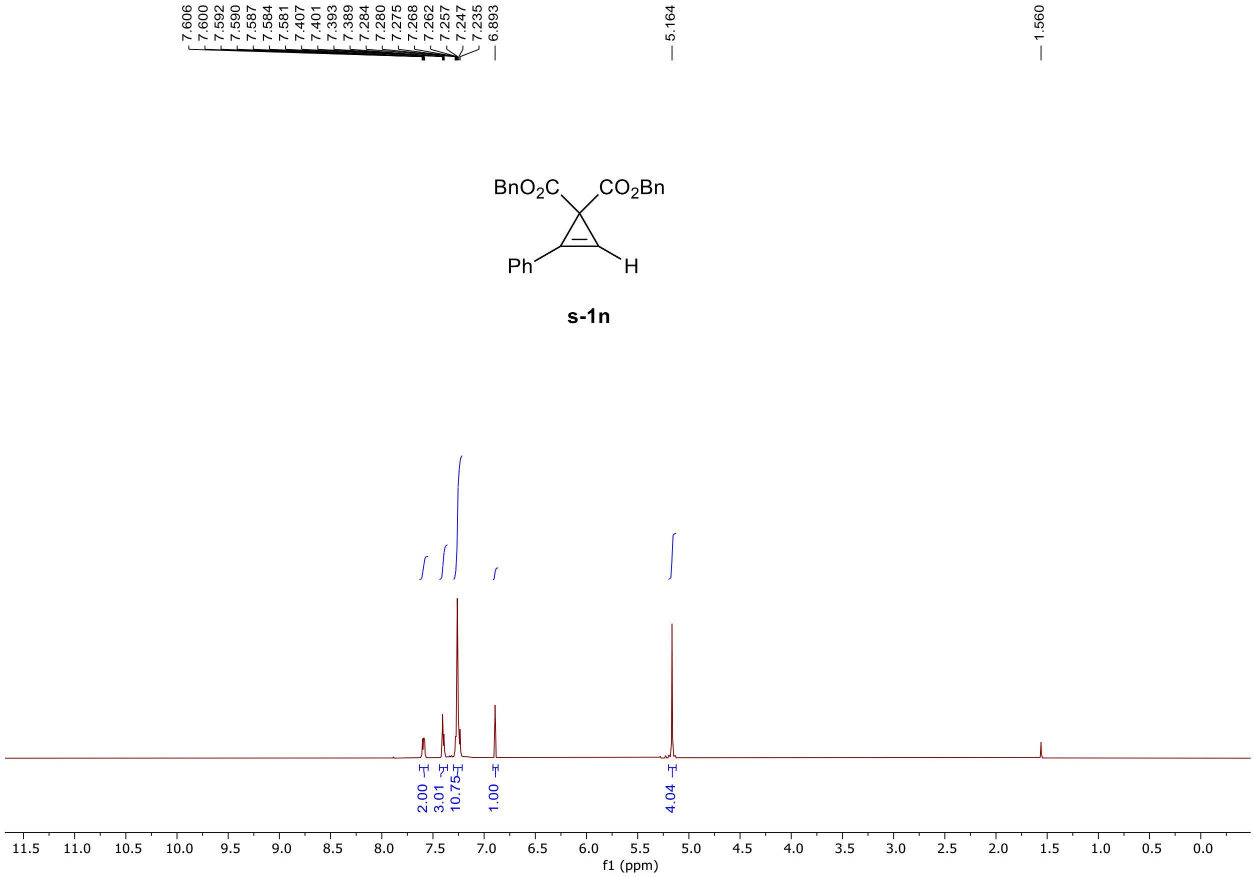

^{13}C NMR (101 MHz, $CDCl_3$) of s-1n

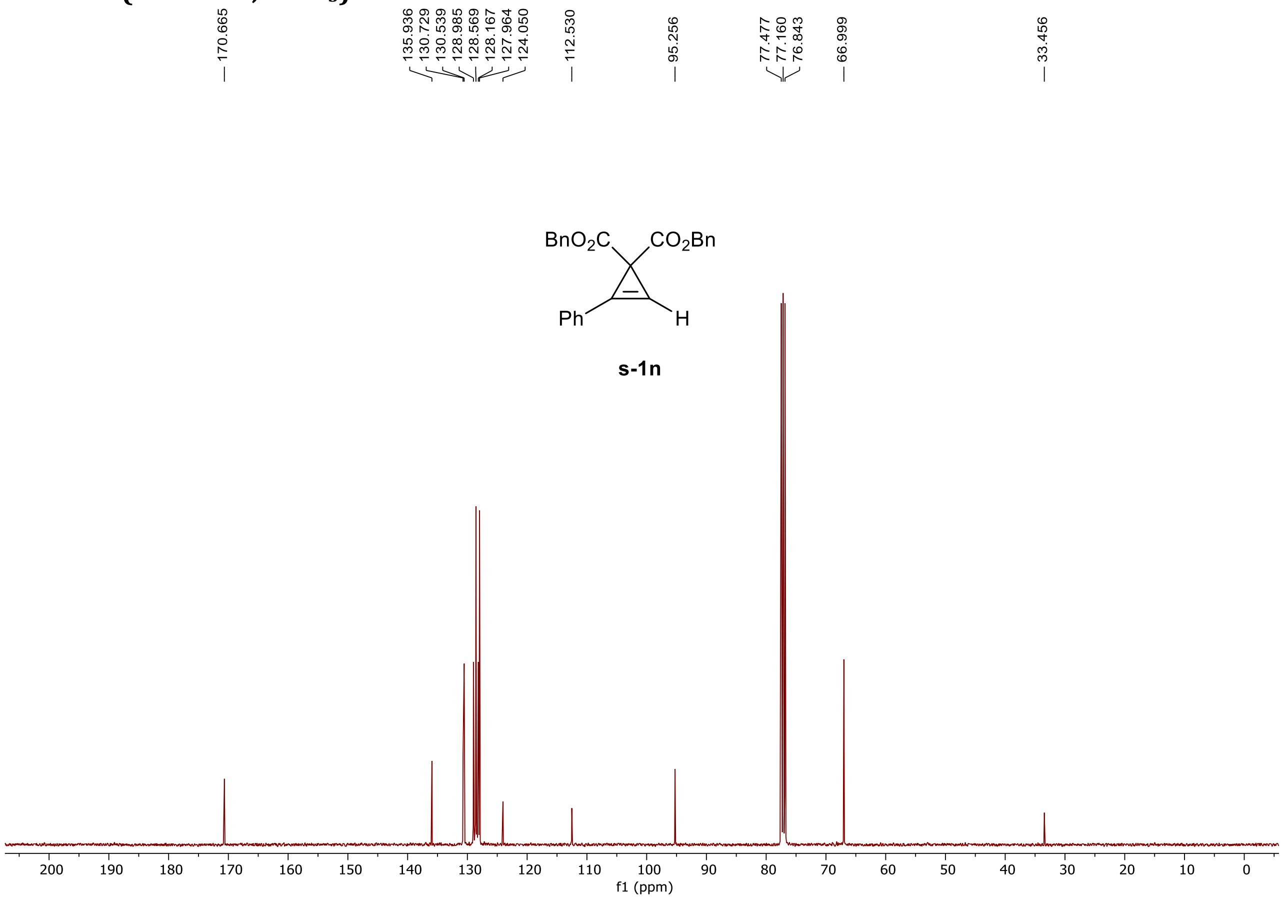

^{1}H NMR (400 MHz, $CDCl_3$) of s-1o

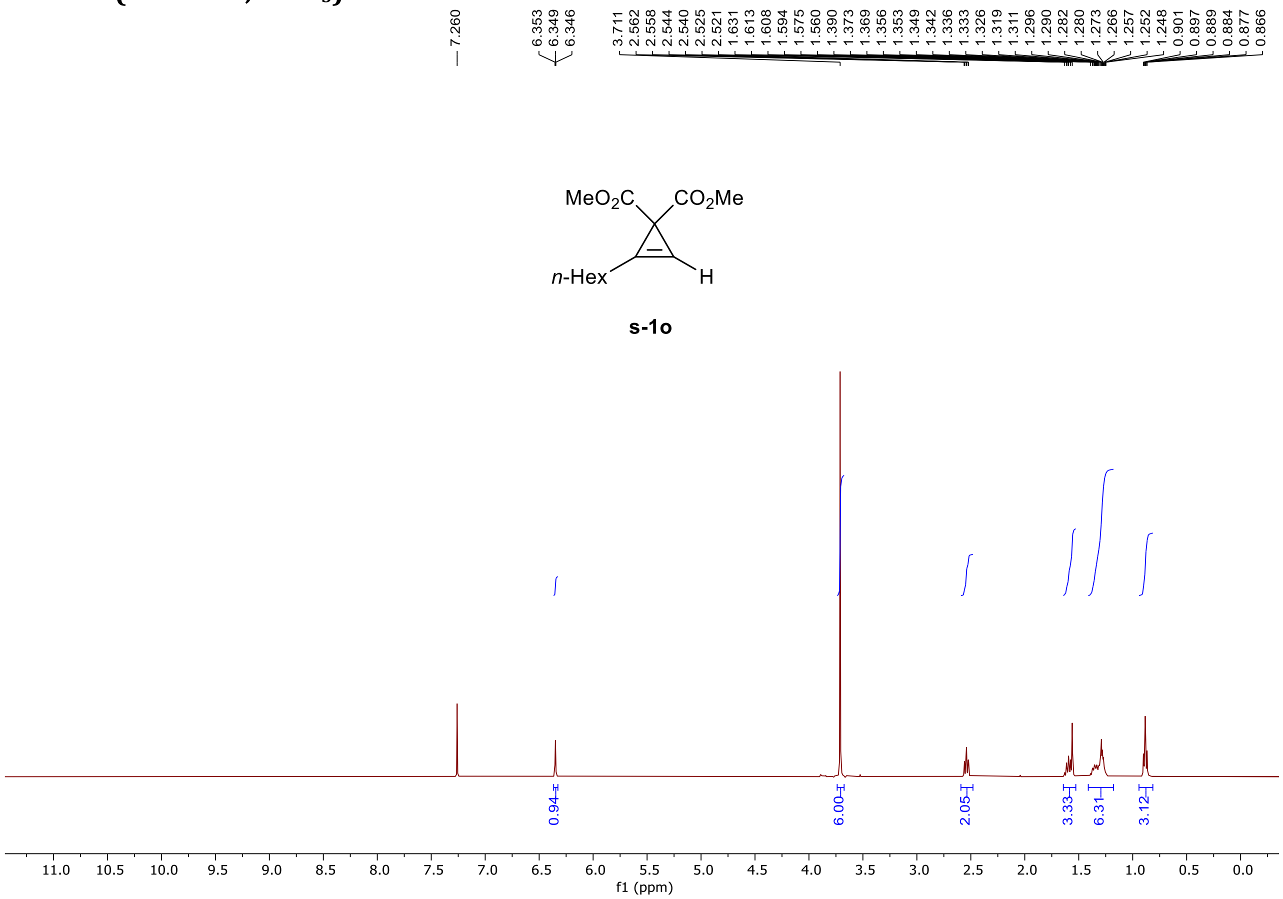

^{1}H NMR (400 MHz, Acetone-d_6) of hypervalent iodine precursor I1

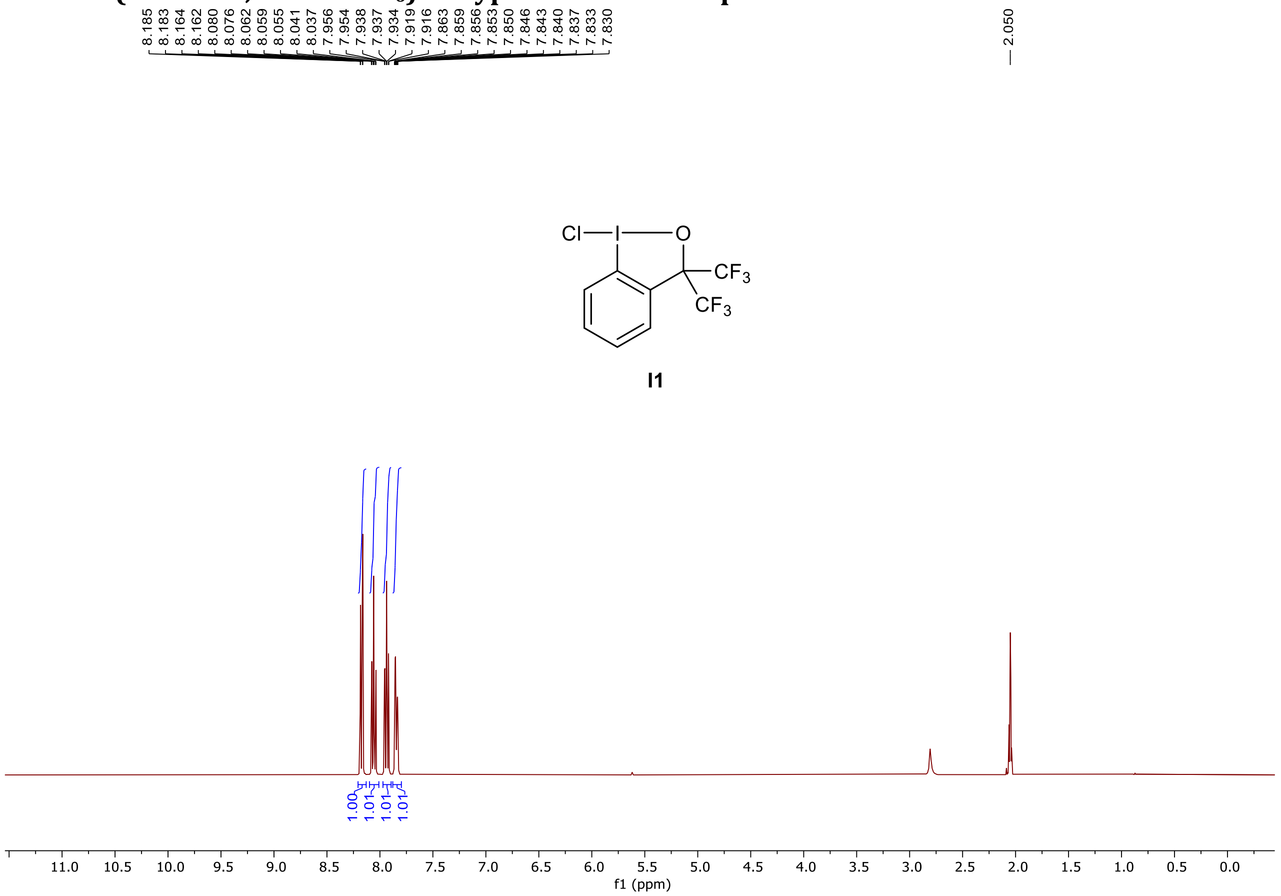

^{13}C NMR (101 MHz, Acetone-d_6) of hypervalent iodine precursor I1

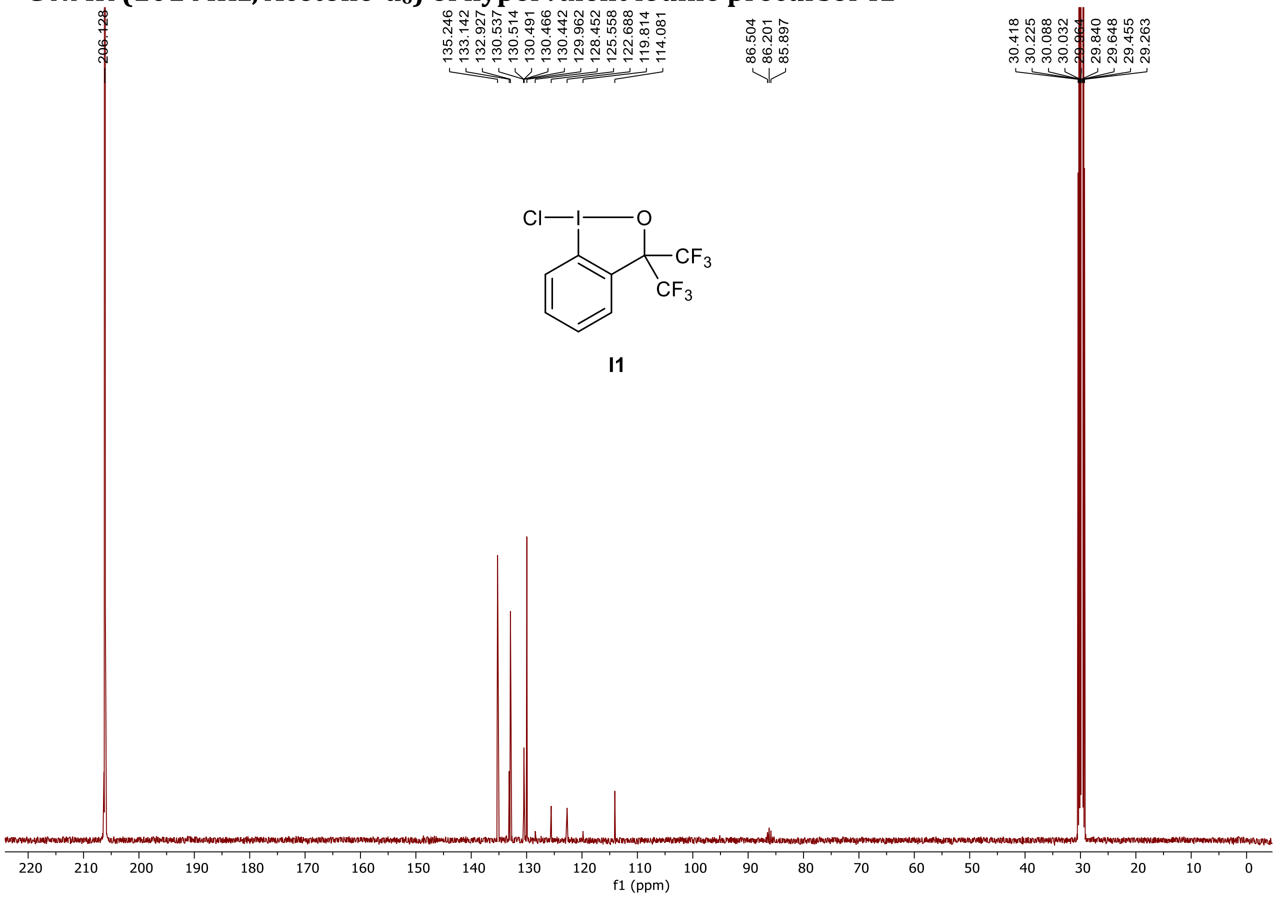

^{19}F NMR (377 MHz, Acetone-d$_6$) of hypervalent iodine precursor I1

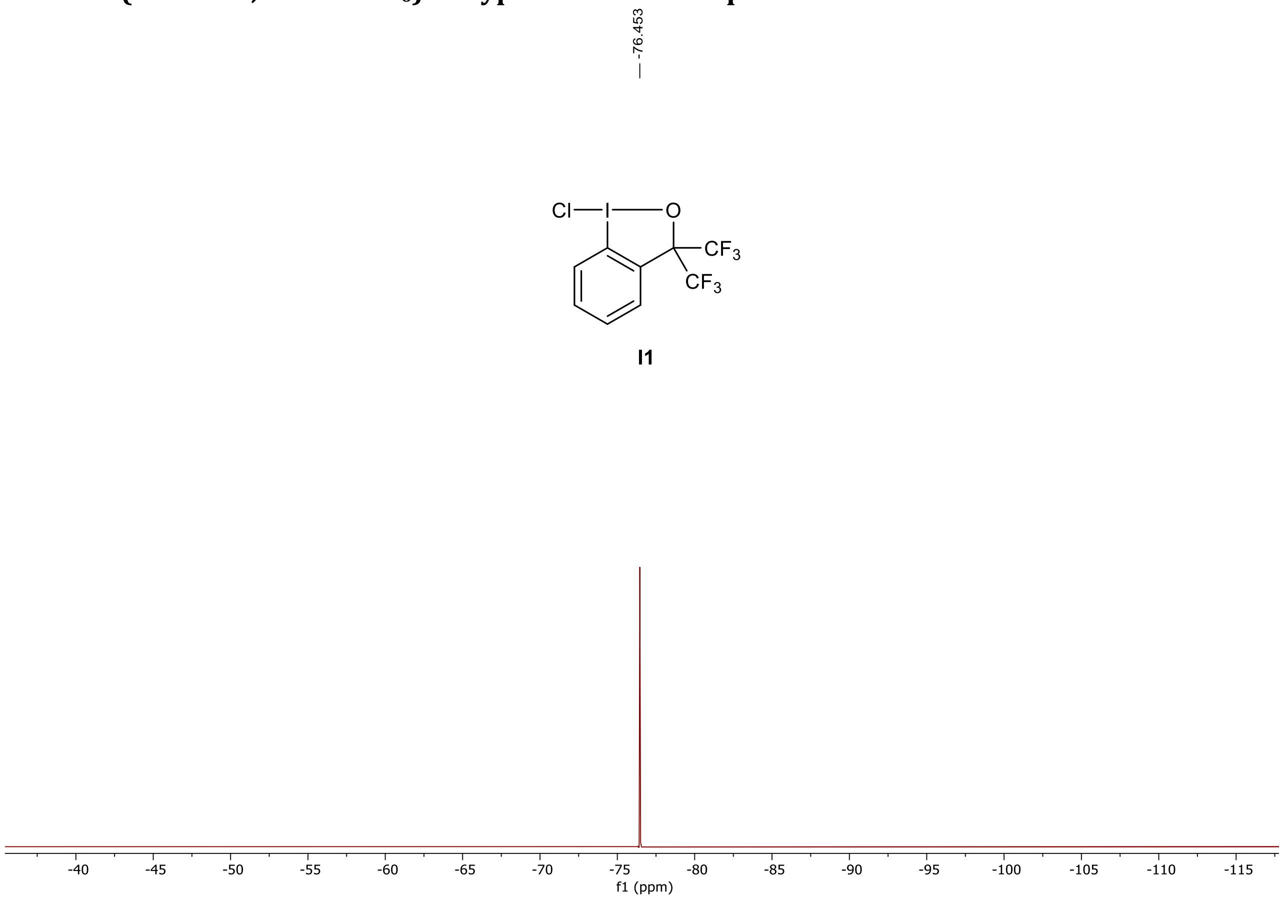

^{1}H NMR (400 MHz, $CDCl_3$) of hypervalent iodine precursor I2

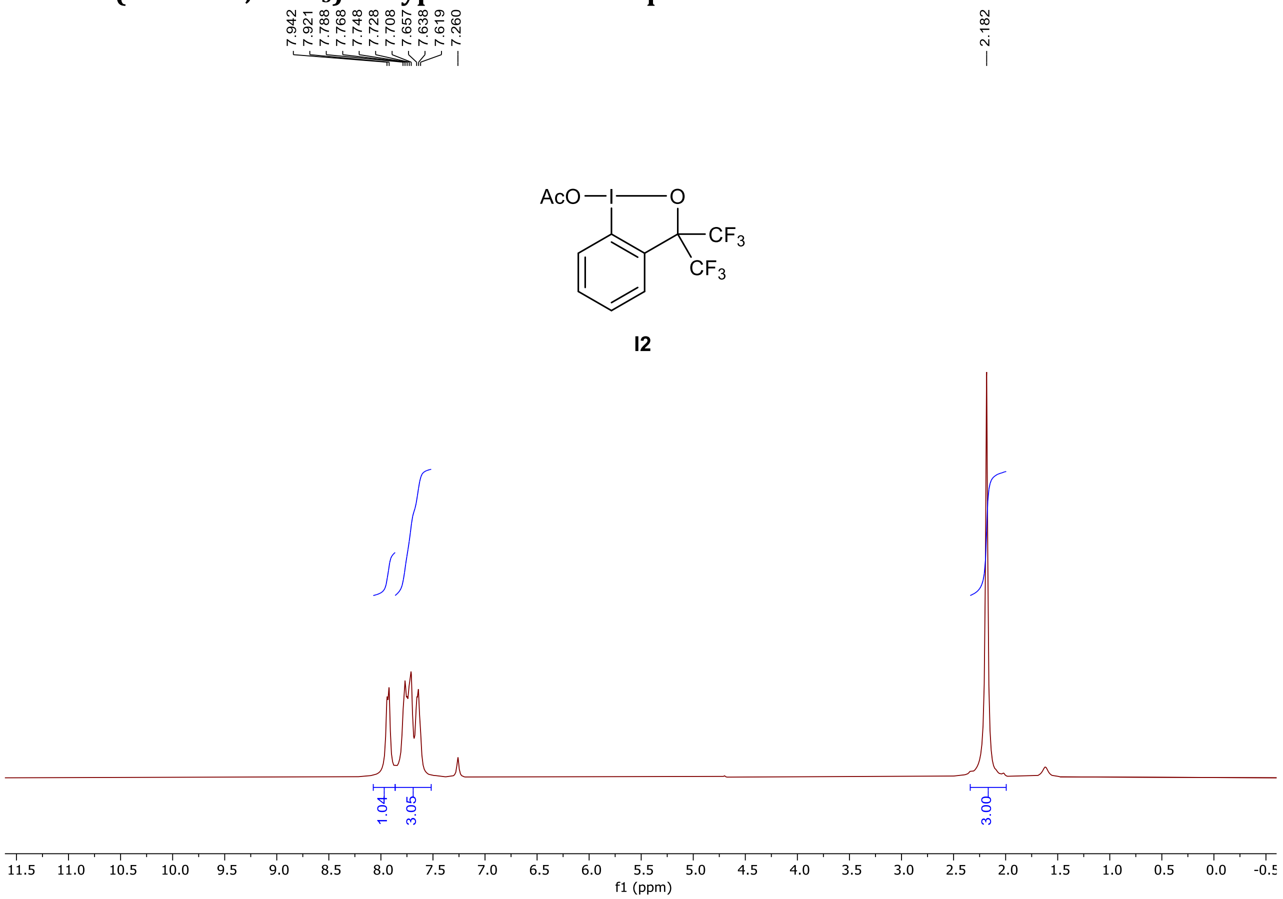

^{13}C NMR (101 MHz, $CDCl_3$) of hypervalent iodine precursor I2

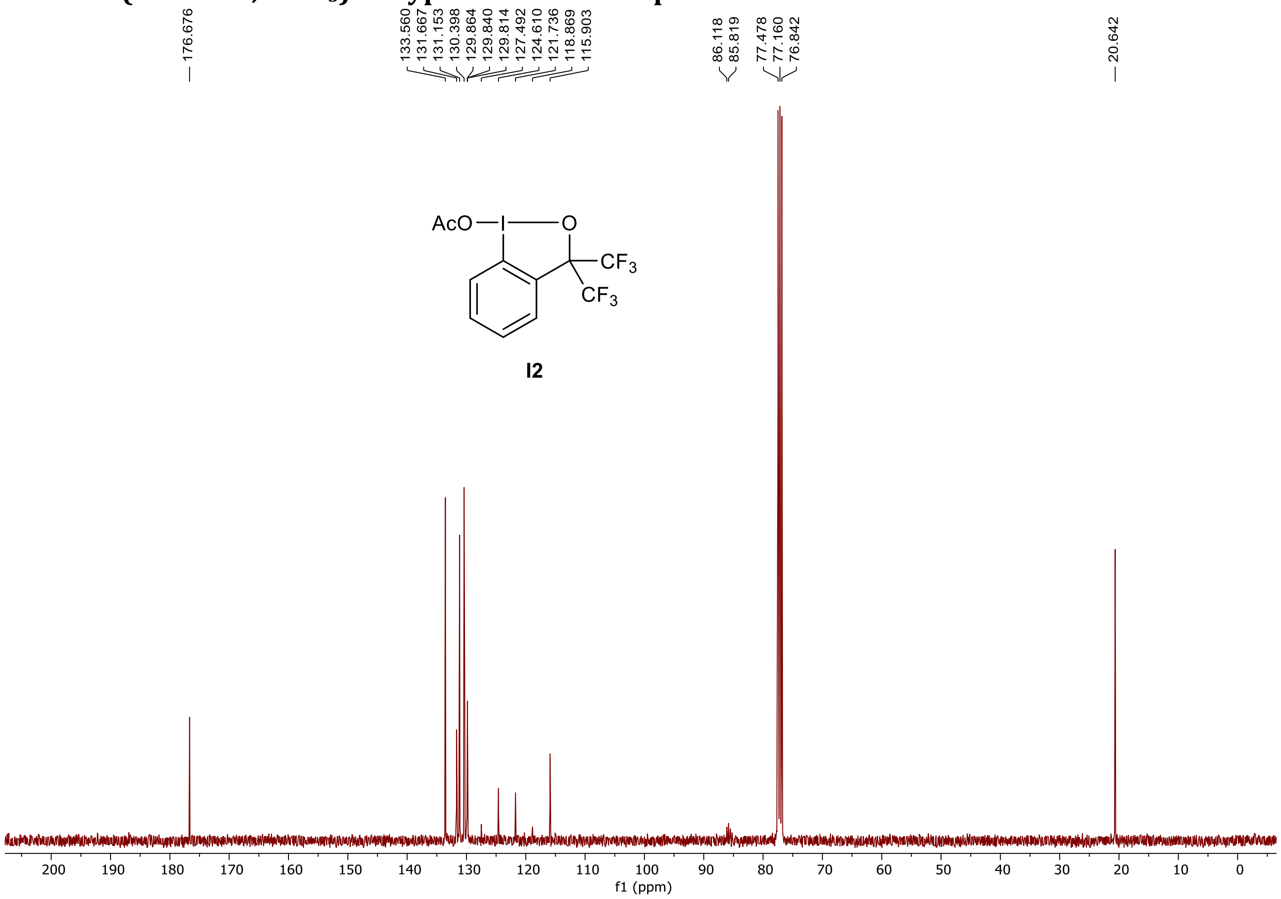

^{19}F NMR (377 MHz, $CDCl_3$) of hypervalent iodine precursor I2

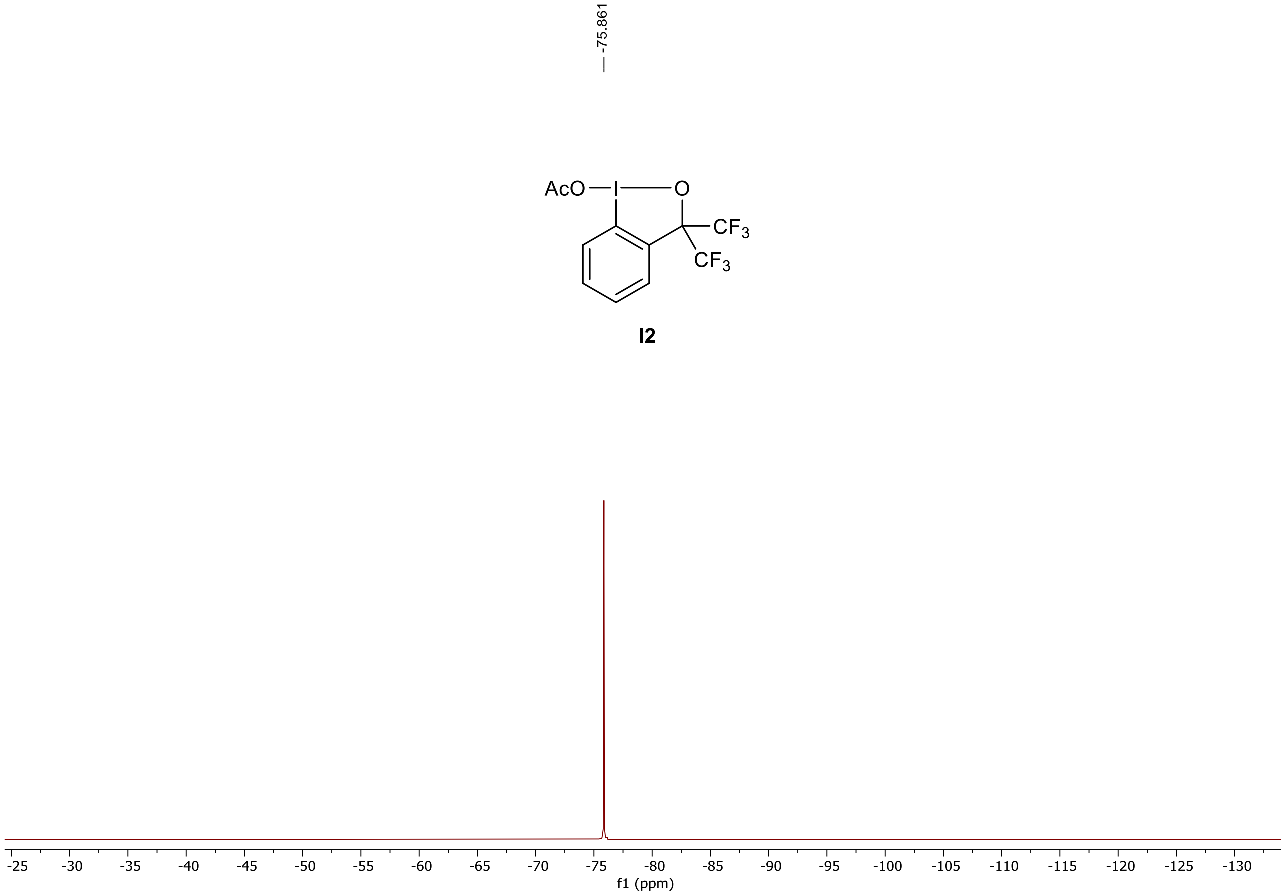

¹H NMR (400 MHz, $CDCl_3$) of 1a

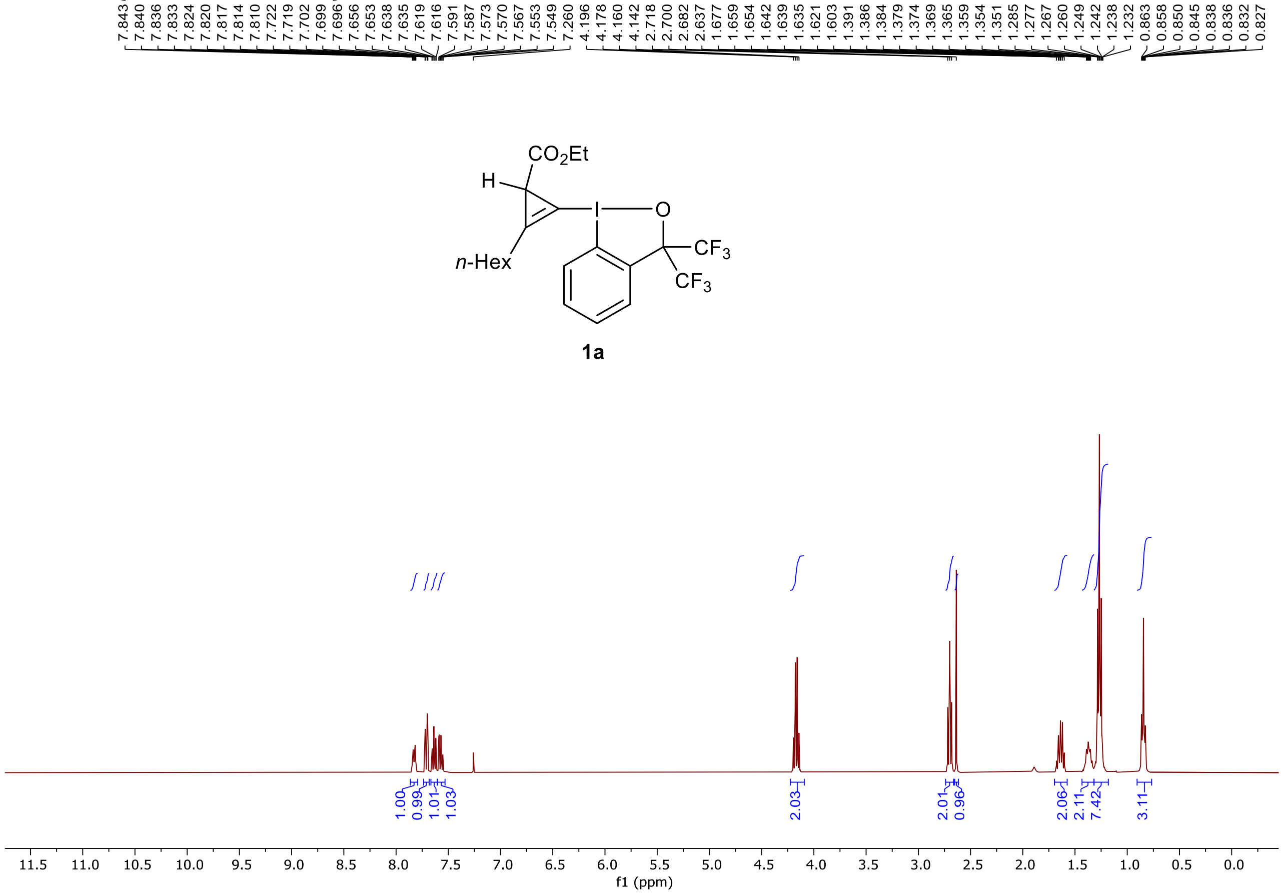

¹³C NMR (101 MHz, $CDCl_3$) of 1a

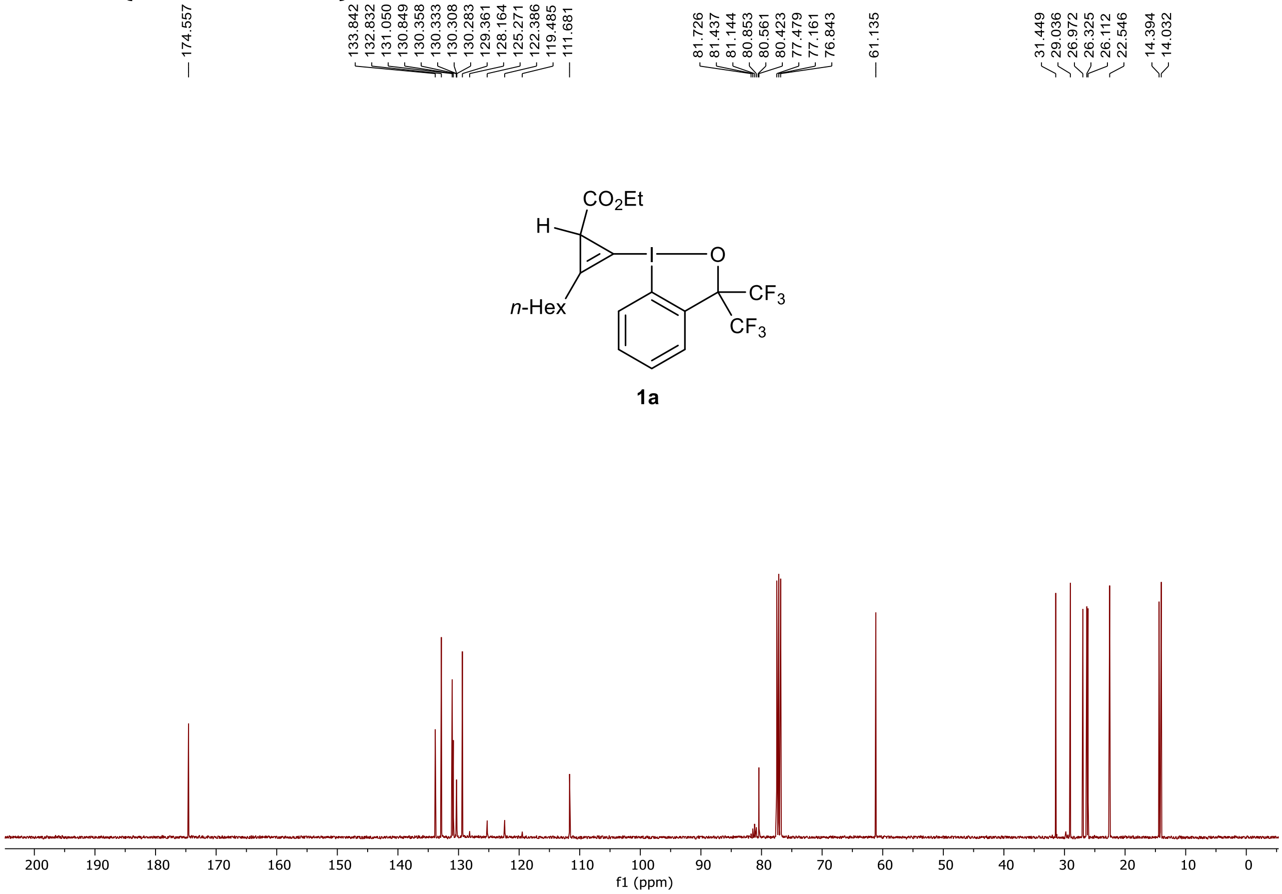

^{19}F NMR (377 MHz, $CDCl_3$) of 1a

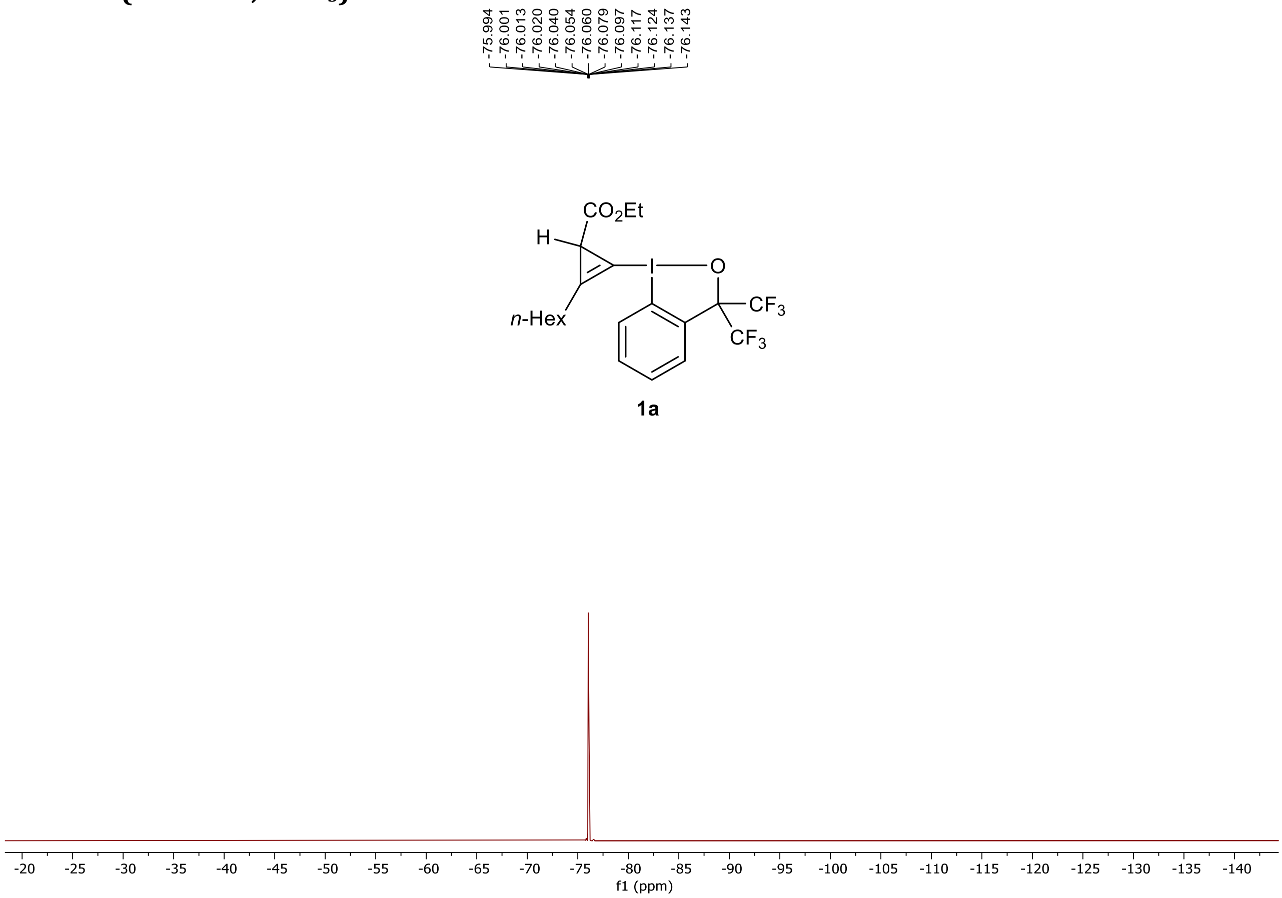

^{1}H NMR (400 MHz, $CDCl_3$) of 1b

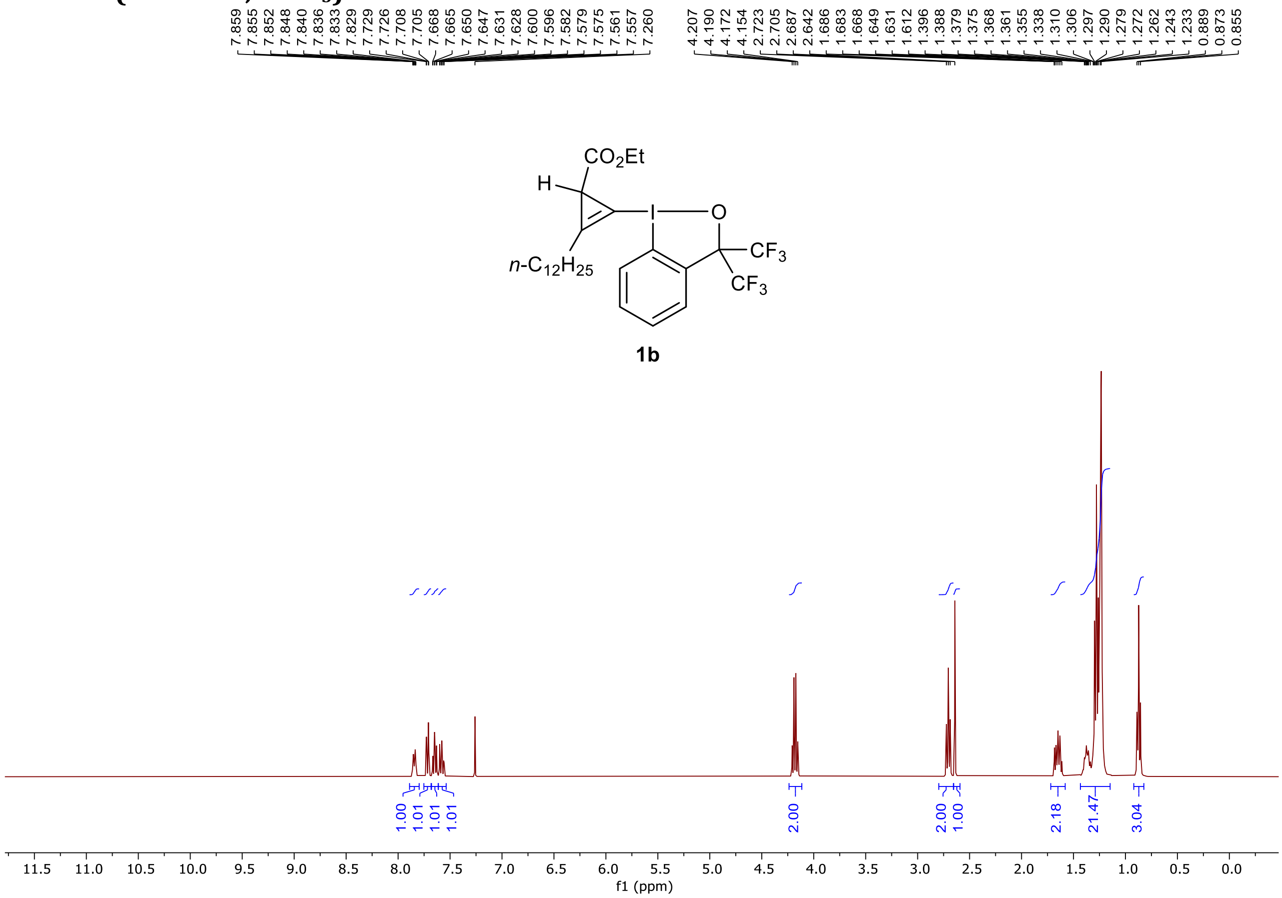

^{13}C NMR (101 MHz, $CDCl_3$) of 1b

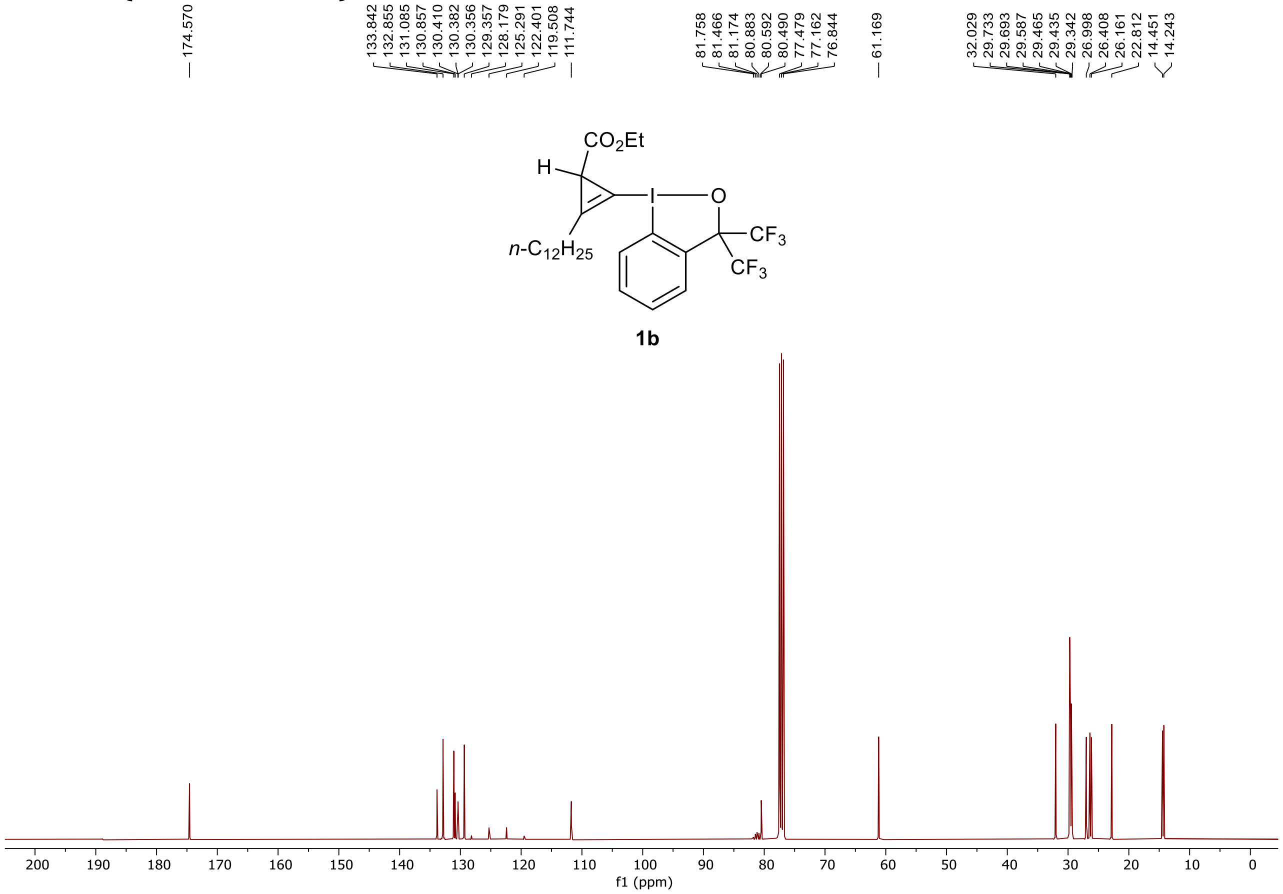

^{19}F NMR (377 MHz, $CDCl_3$) of 1b

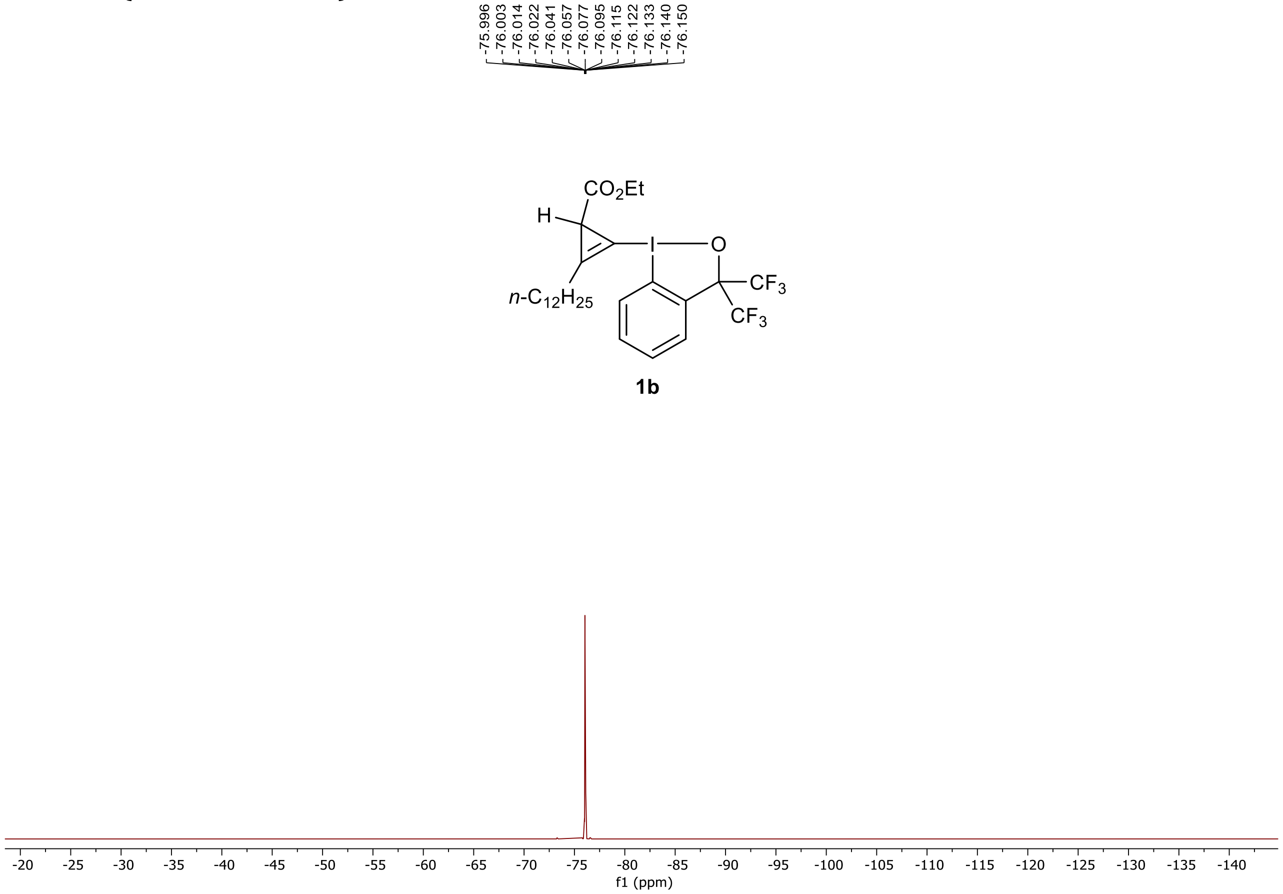

^{1}H NMR (400 MHz, $CDCl_3$) of 1c

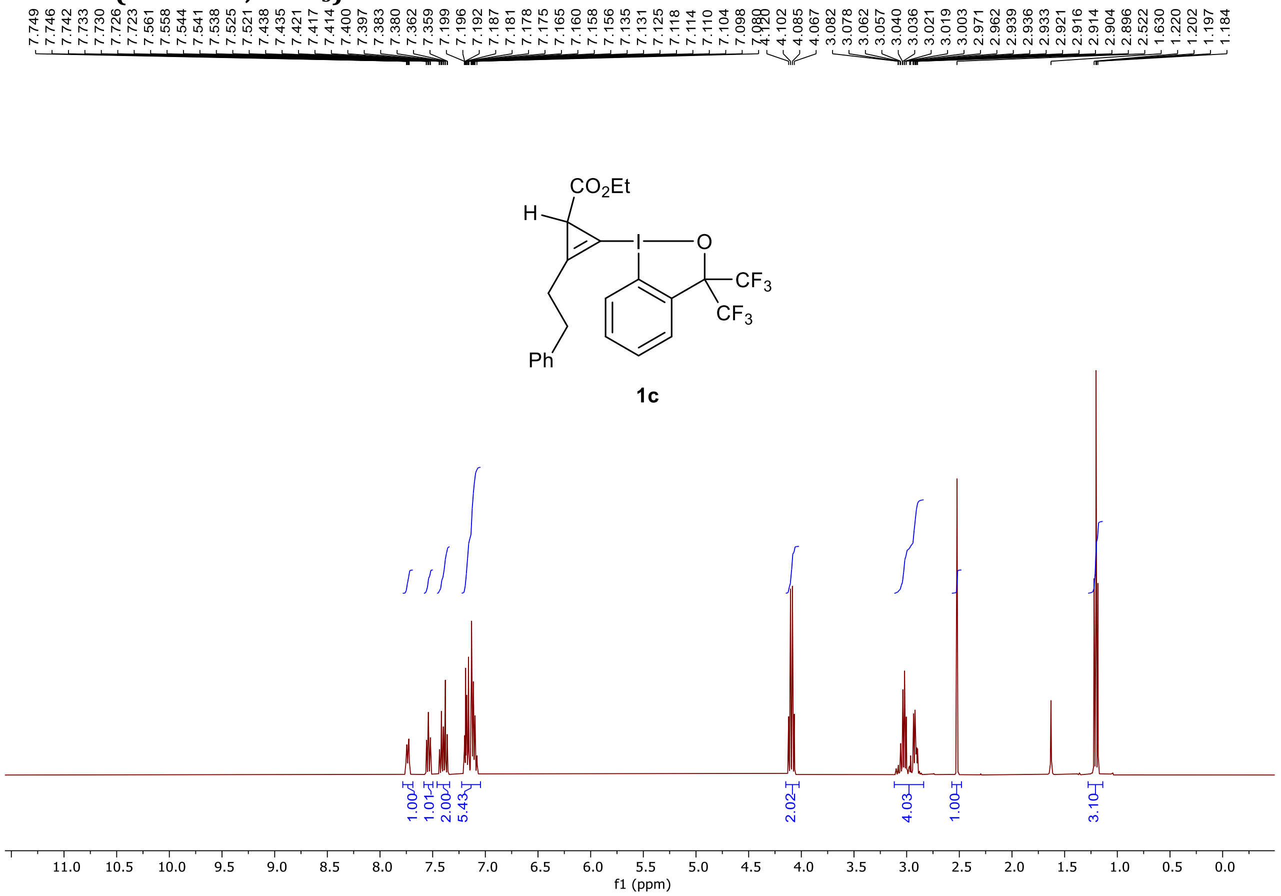

^{13}C NMR (101 MHz, $CDCl_3$) of 1c

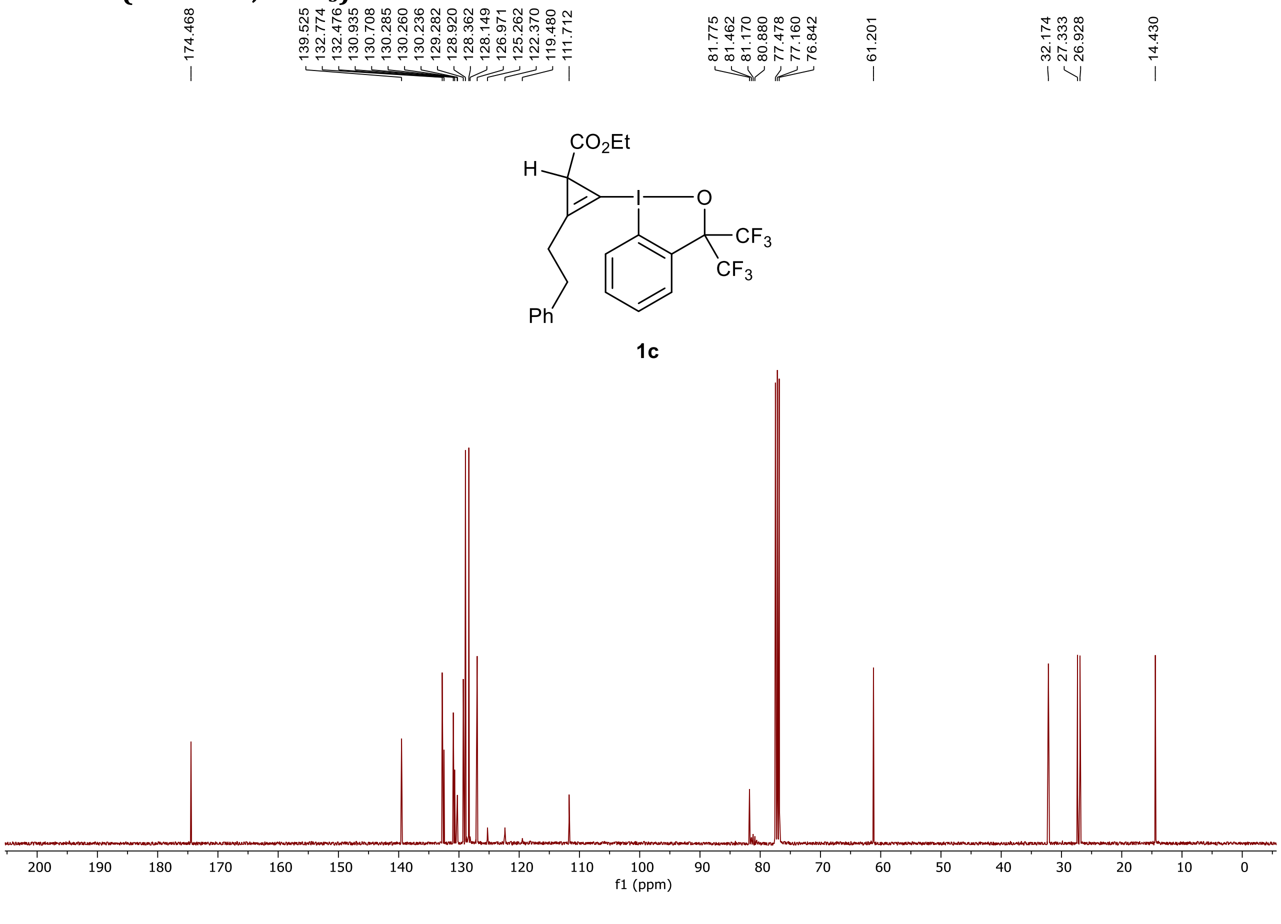

^{19}F NMR (377 MHz, $CDCl_3$) of 1c

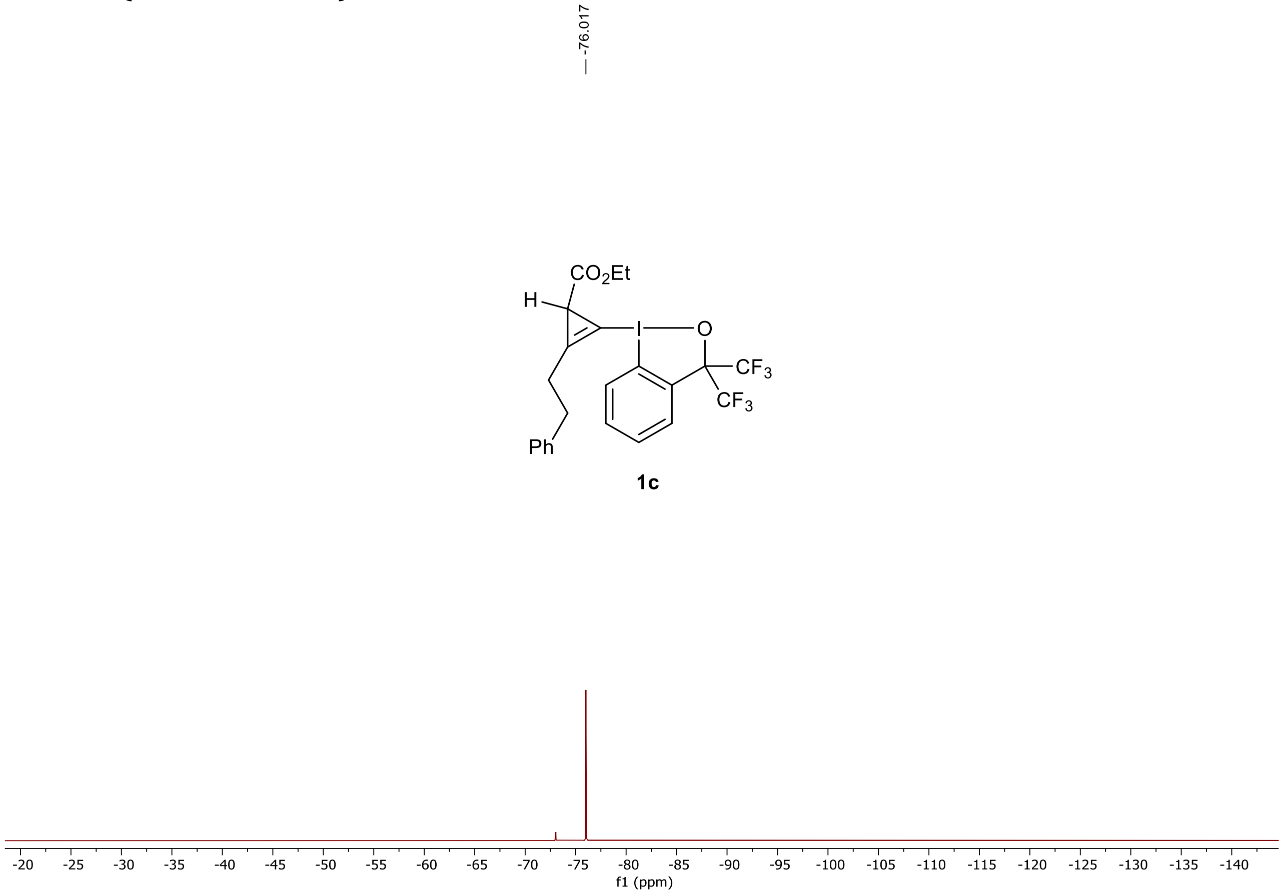

^{1}H NMR (400 MHz, $CDCl_3$) of 1d

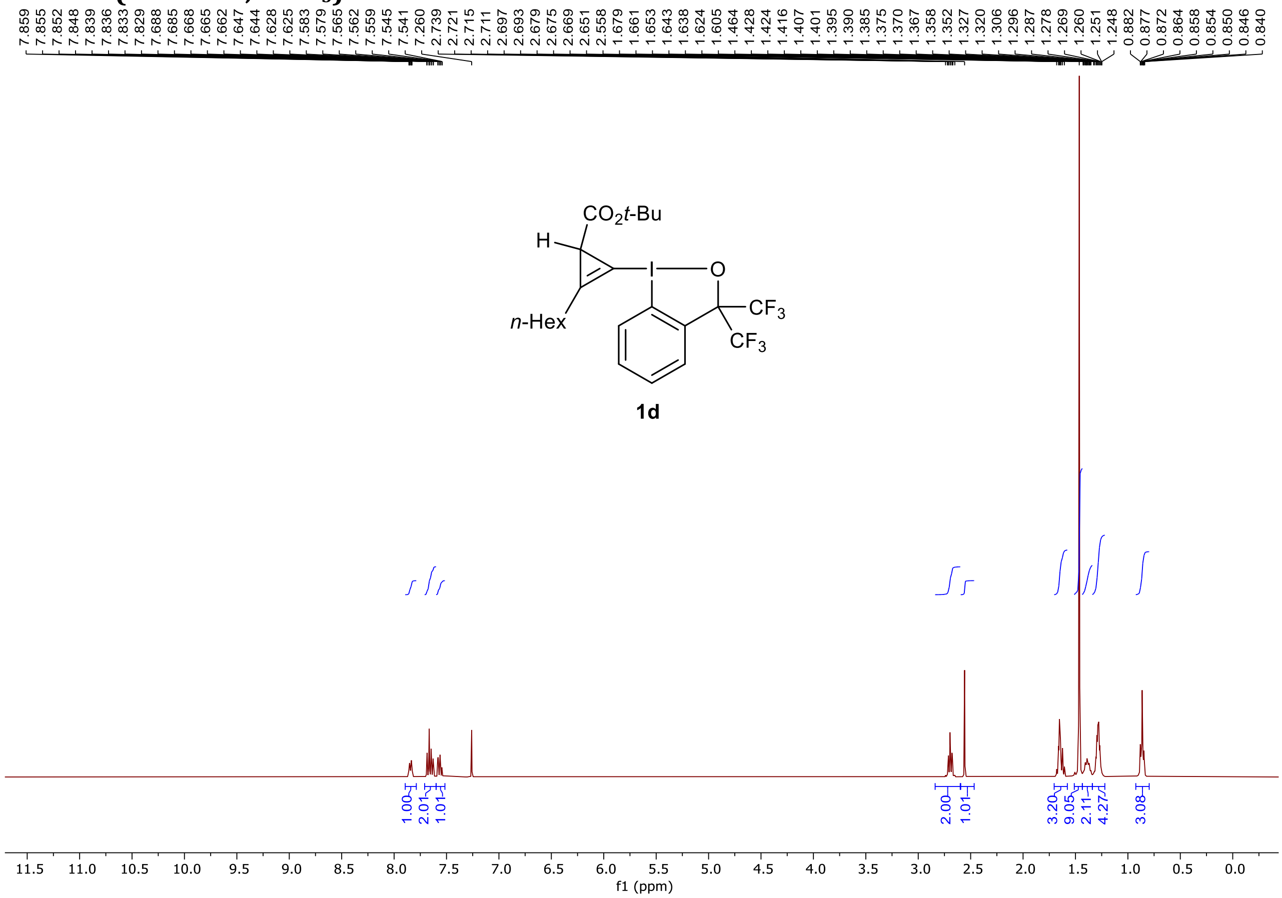

^{13}C NMR (101 MHz, $CDCl_3$) of 1d

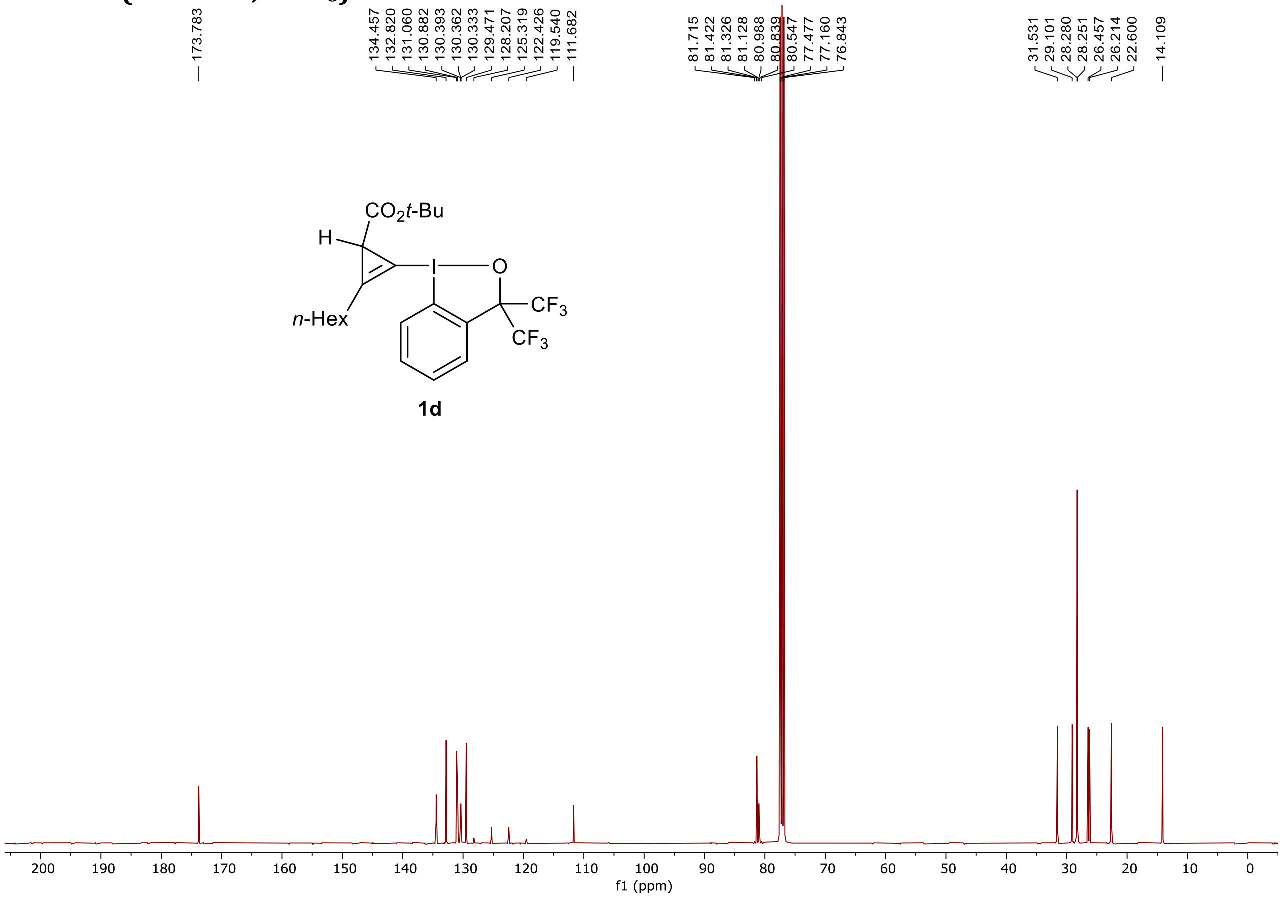

^{19}F NMR (377 MHz, $CDCl_3$) of 1d

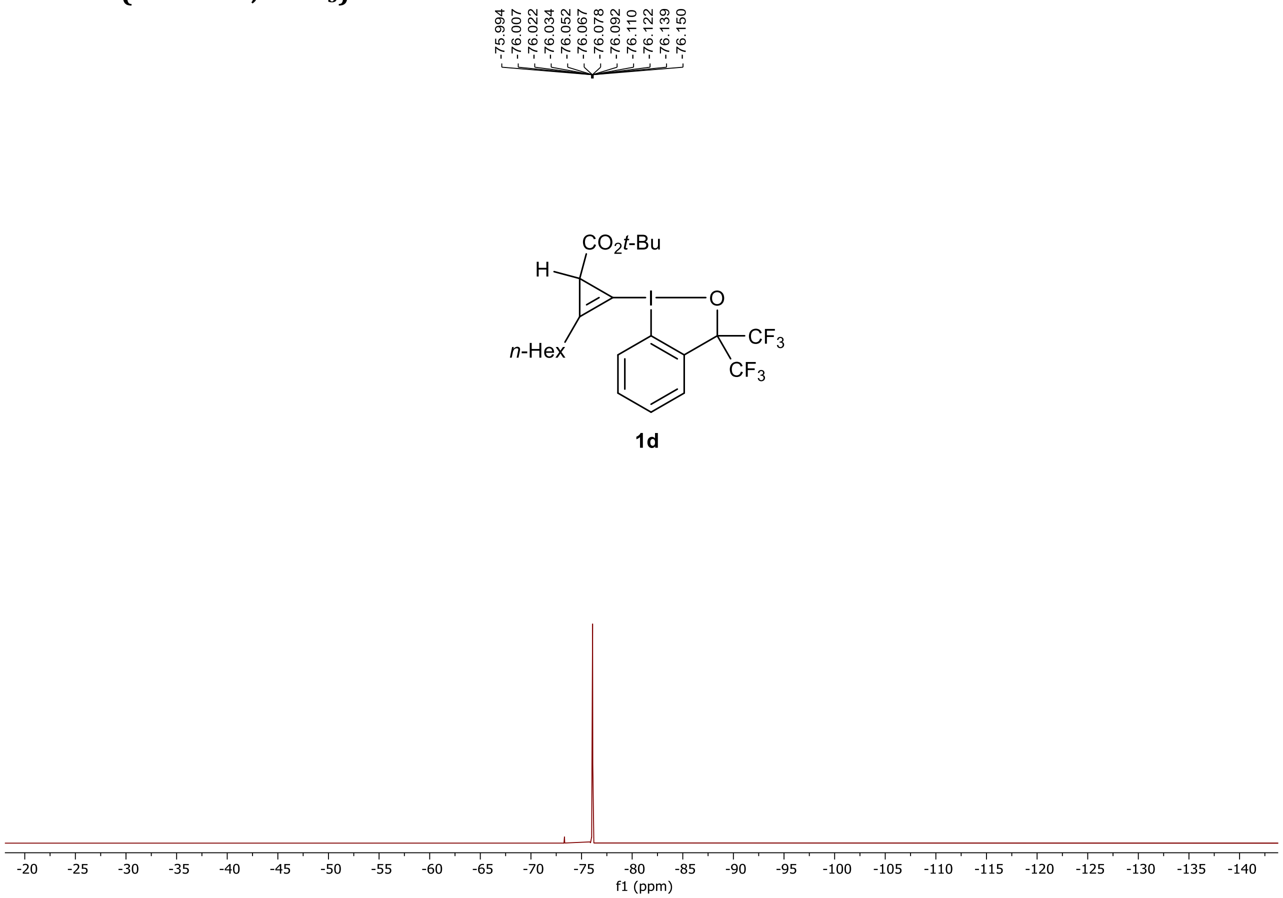

^{1}H NMR (400 MHz, $CDCl_3$) of 1e

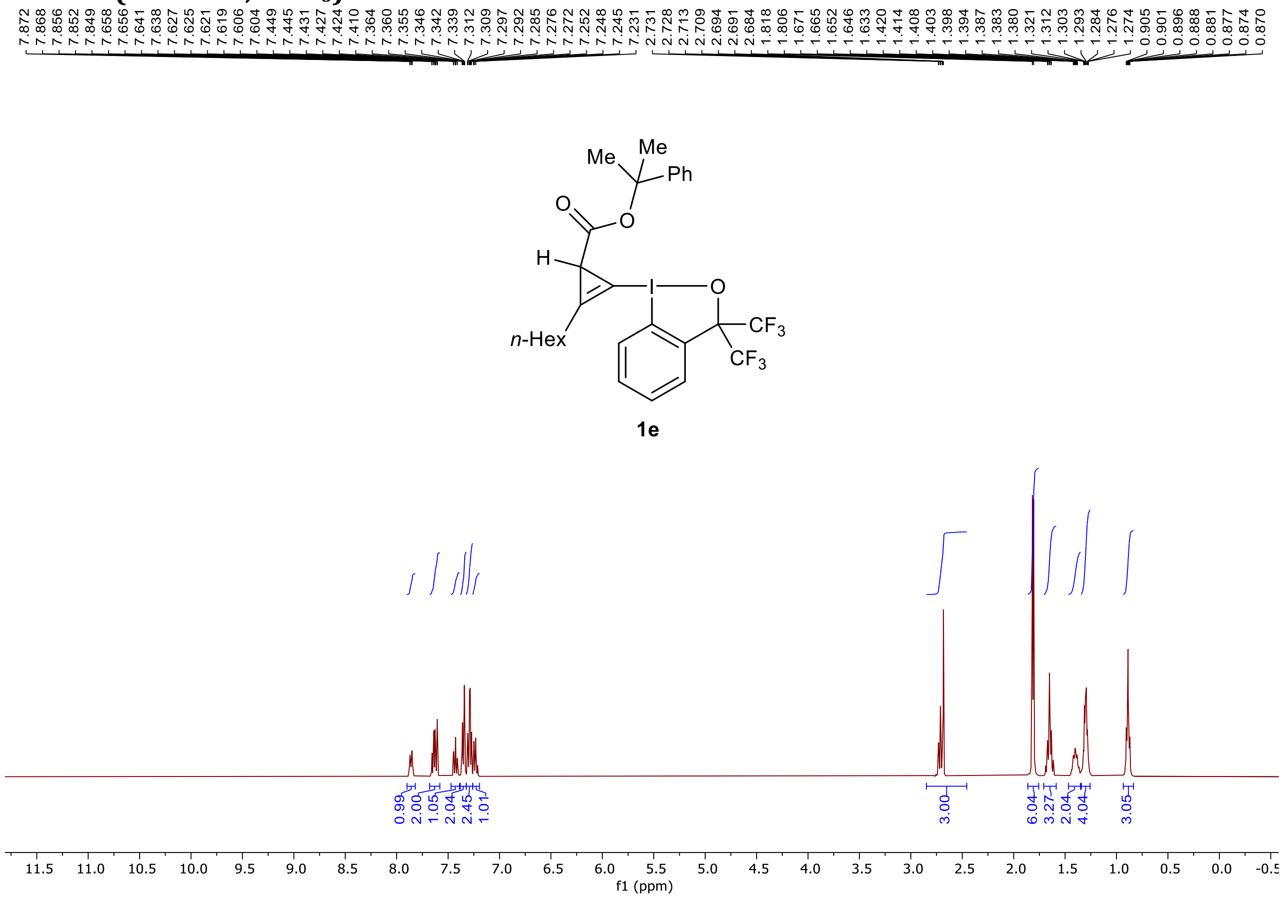

^{13}C NMR (101 MHz, $CDCl_3$) of 1e

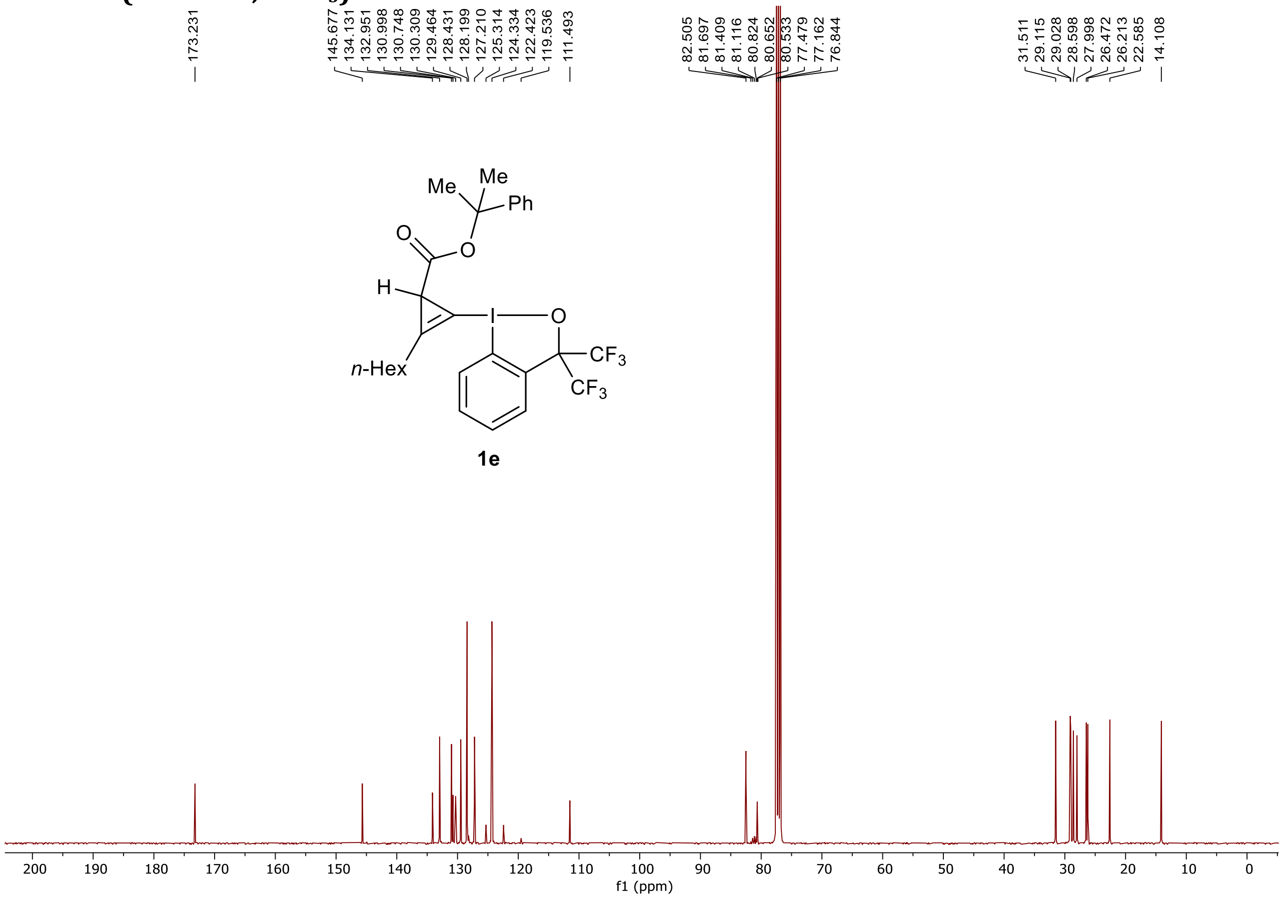

^{19}F NMR (377 MHz, $CDCl_3$) of 1e

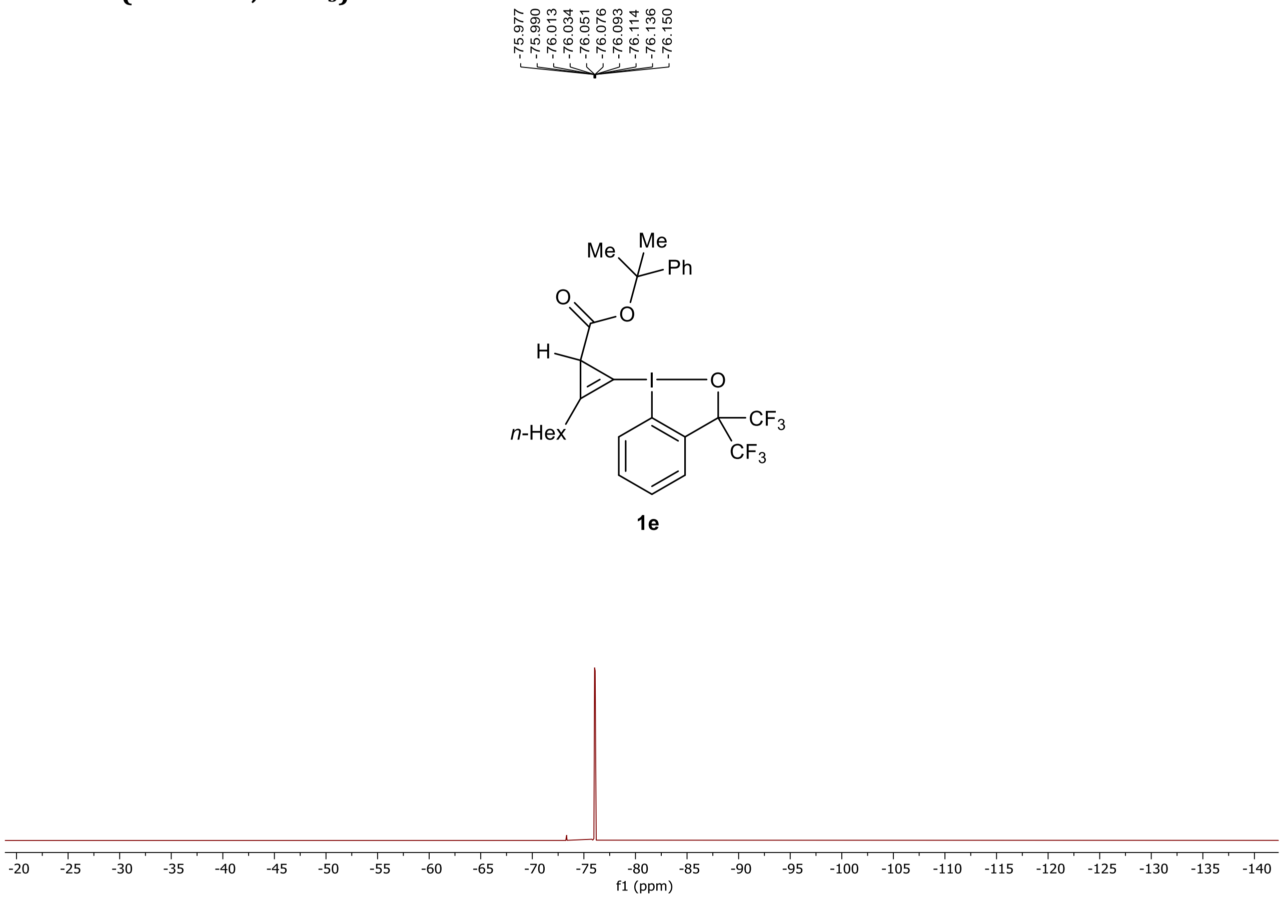

^{1}H NMR (400 MHz, $CDCl_3$) of 1f

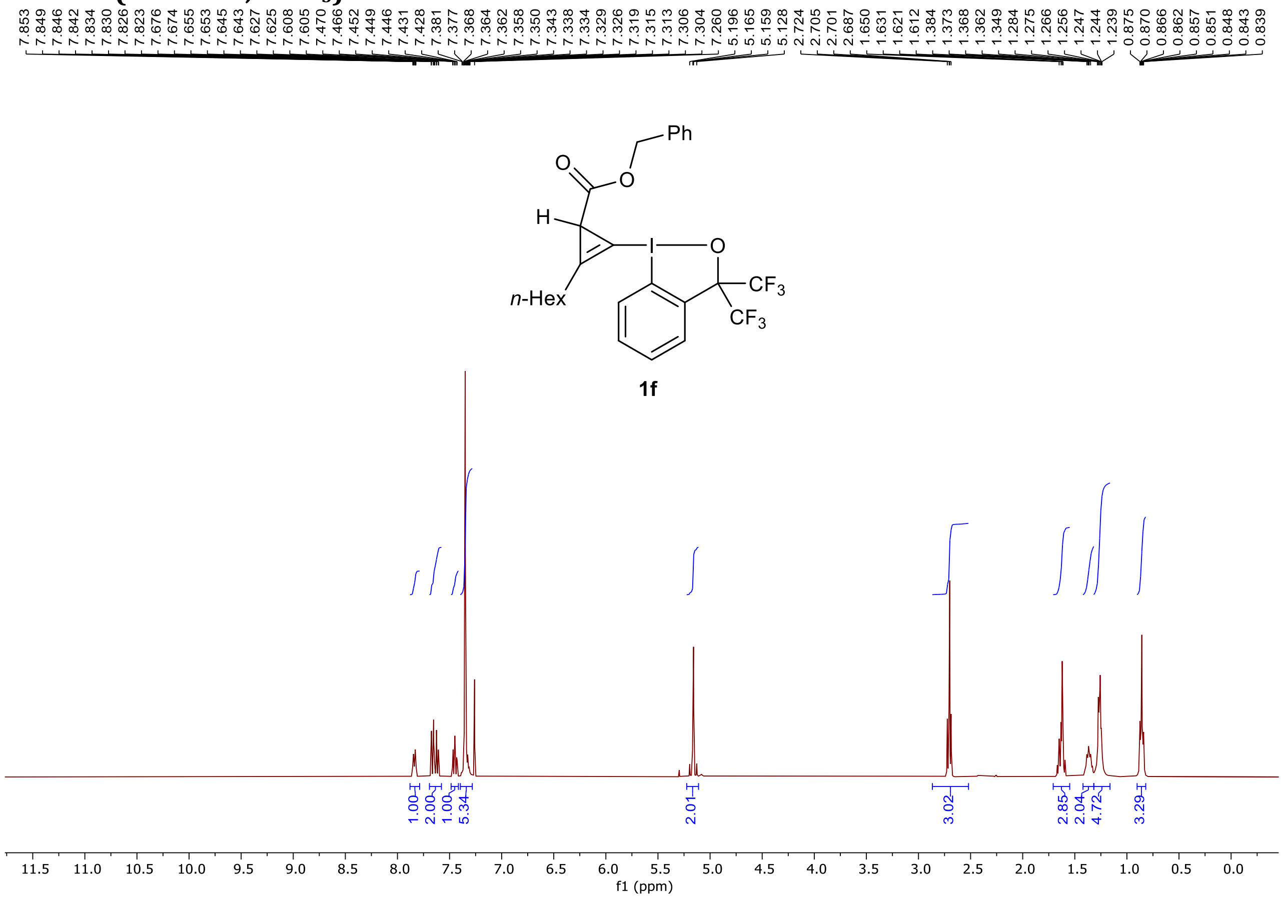

^{13}C NMR (101 MHz, $CDCl_3$) of 1f

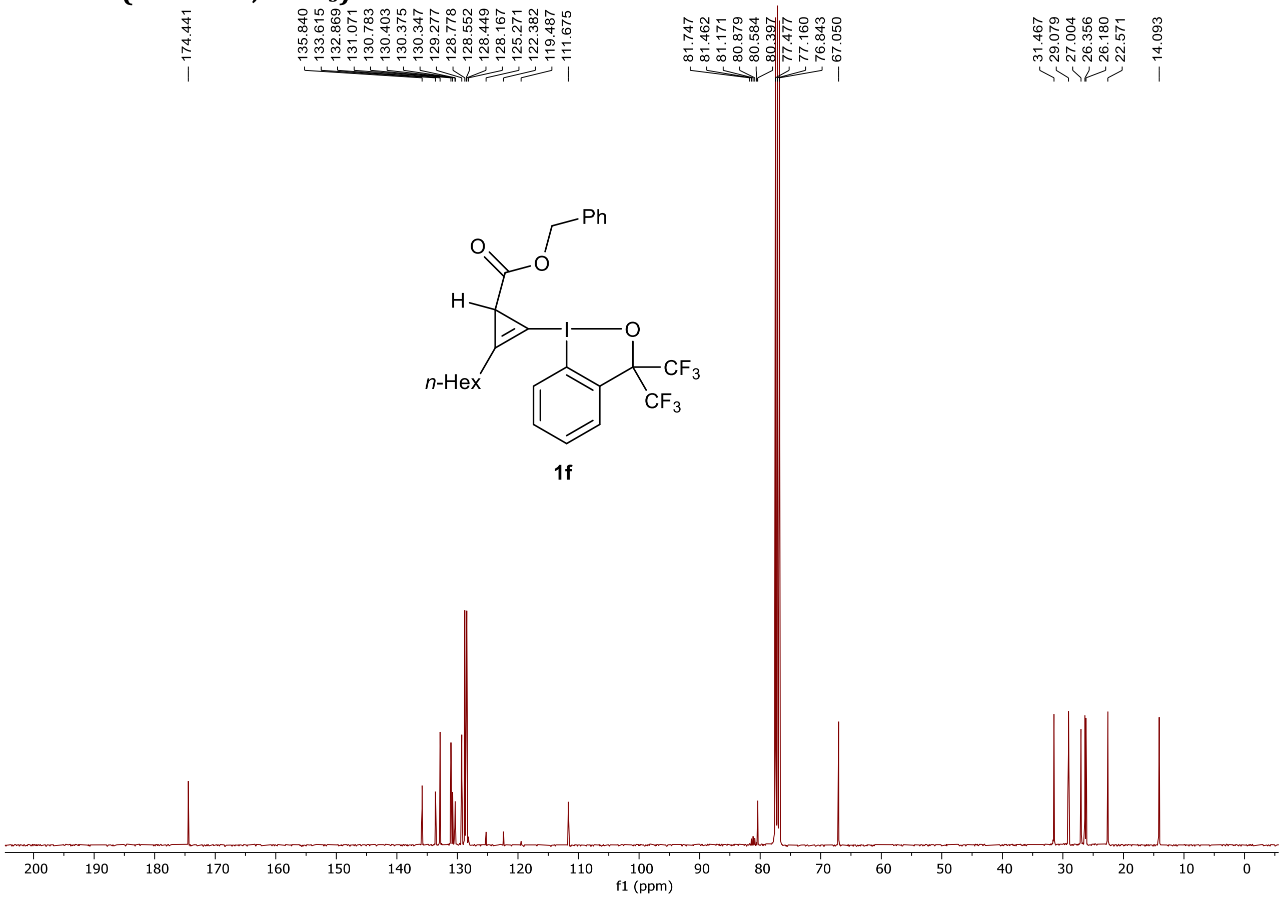

^{19}F NMR (377 MHz, $CDCl_3$) of 1f

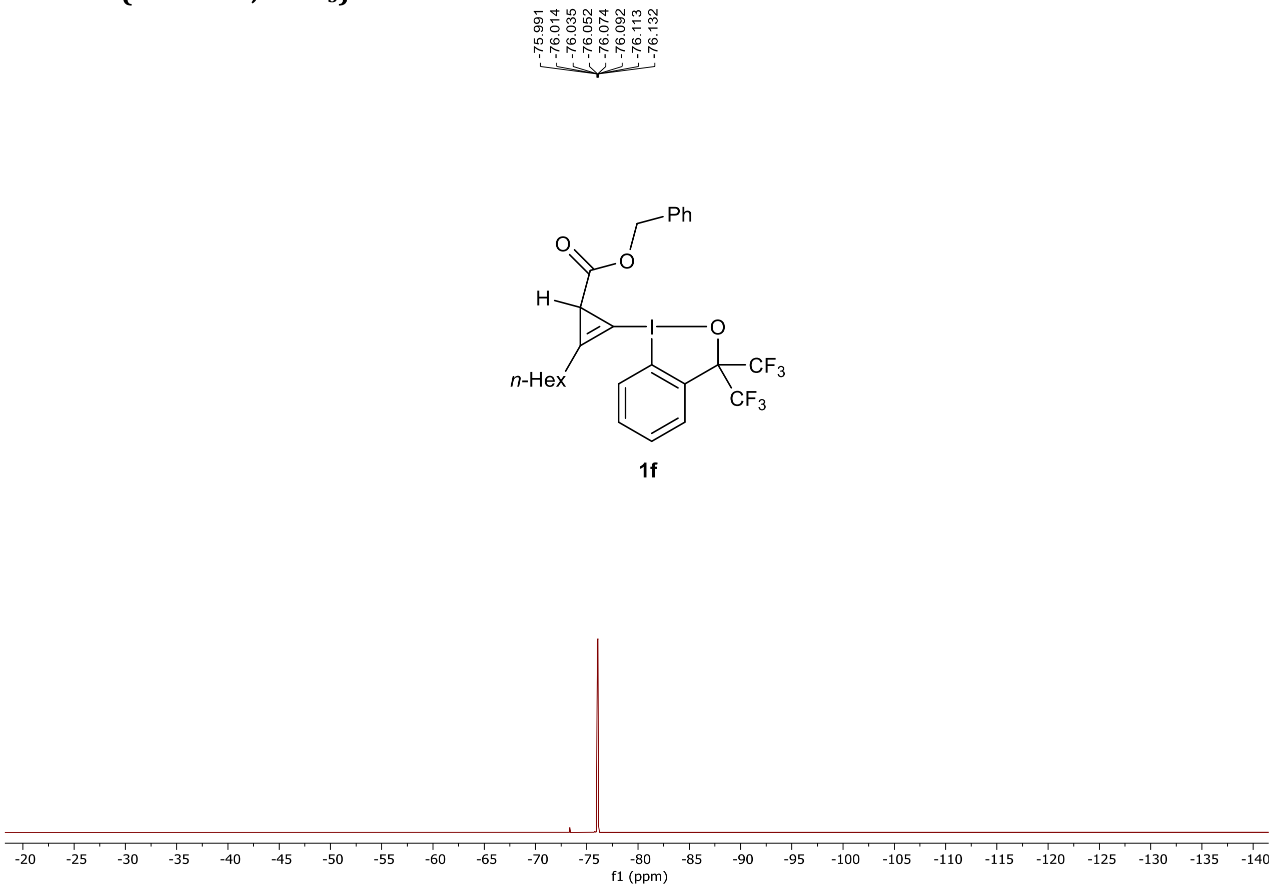

^{1}H NMR (400 MHz, $CDCl_3$) of 1g

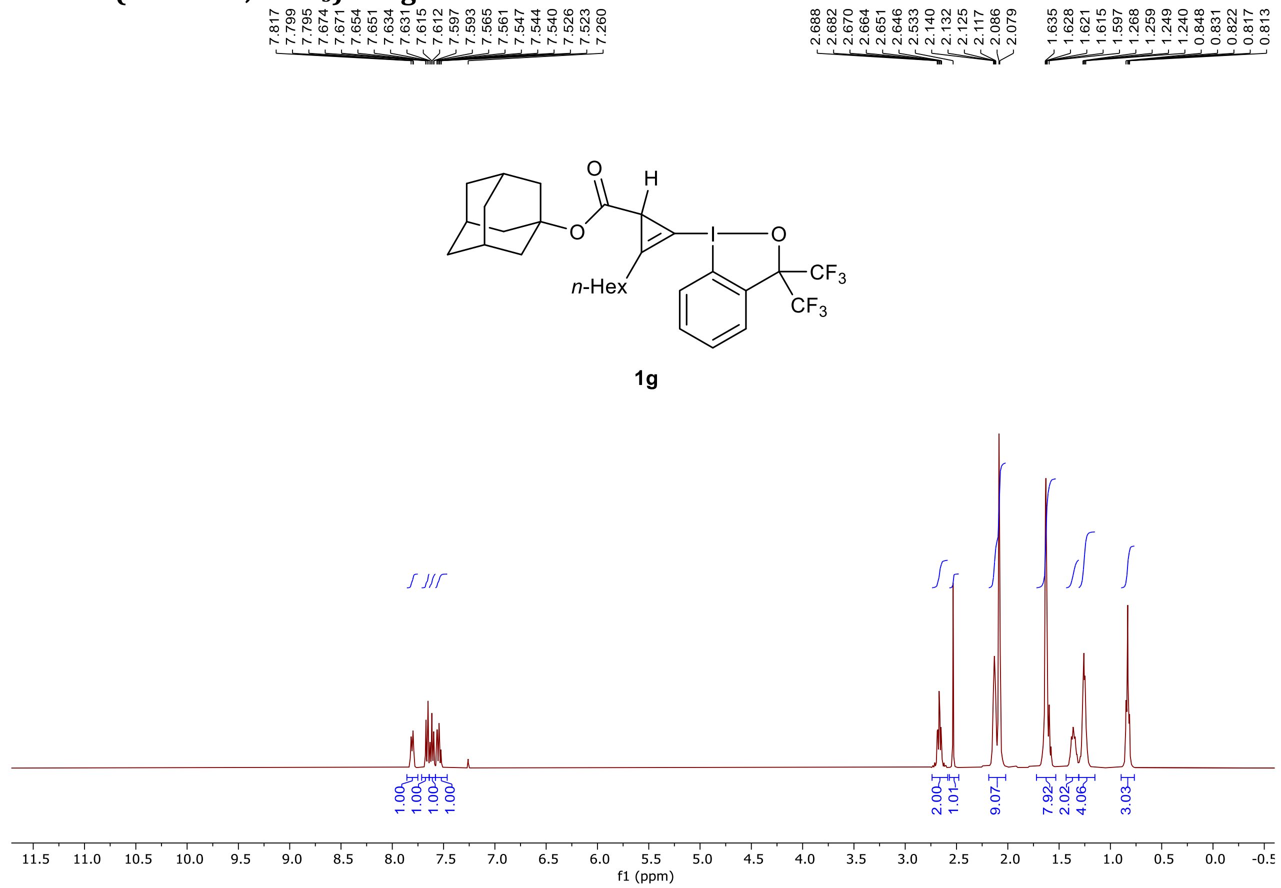

^{13}C NMR (101 MHz, $CDCl_3$) of 1g

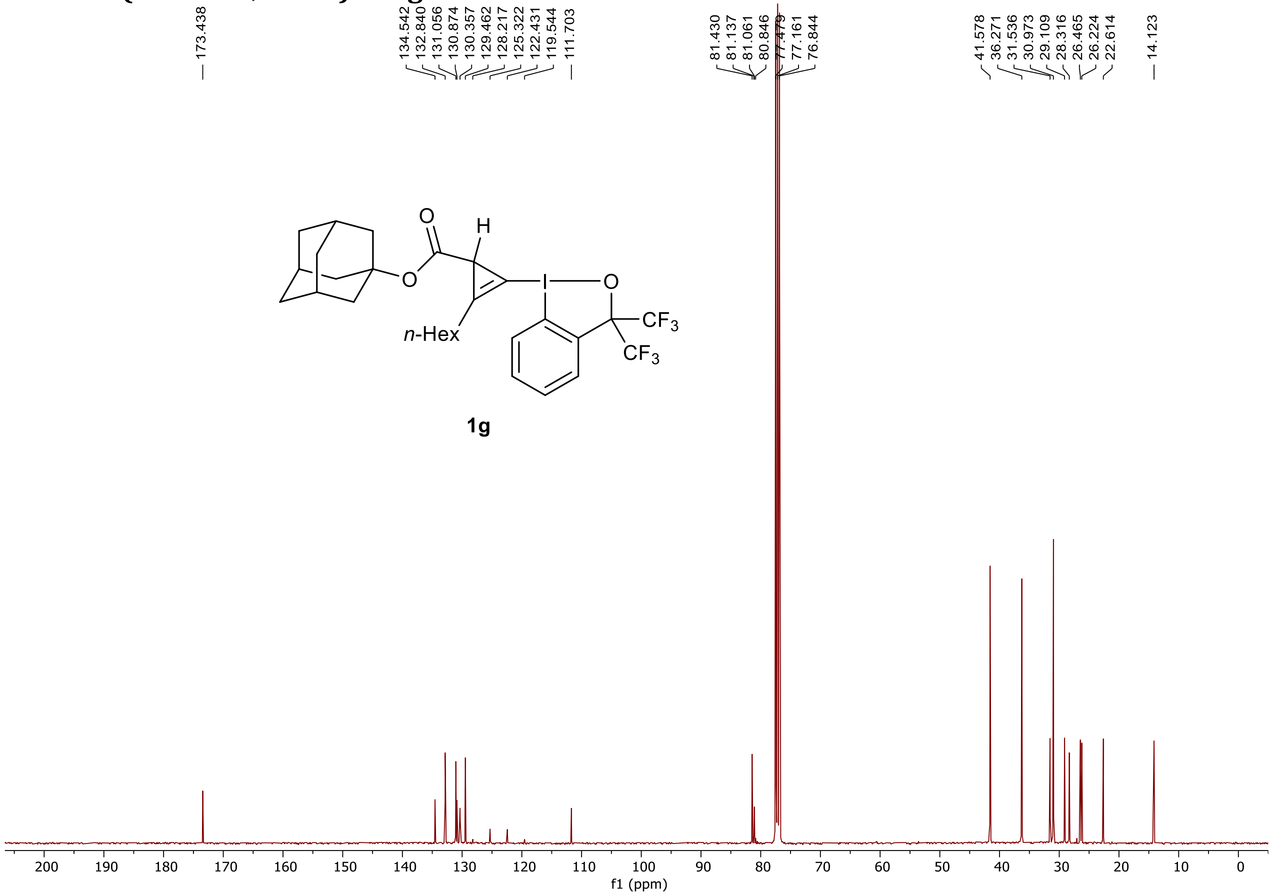

^{19}F NMR (377 MHz, CDCl$_3$) of 1g

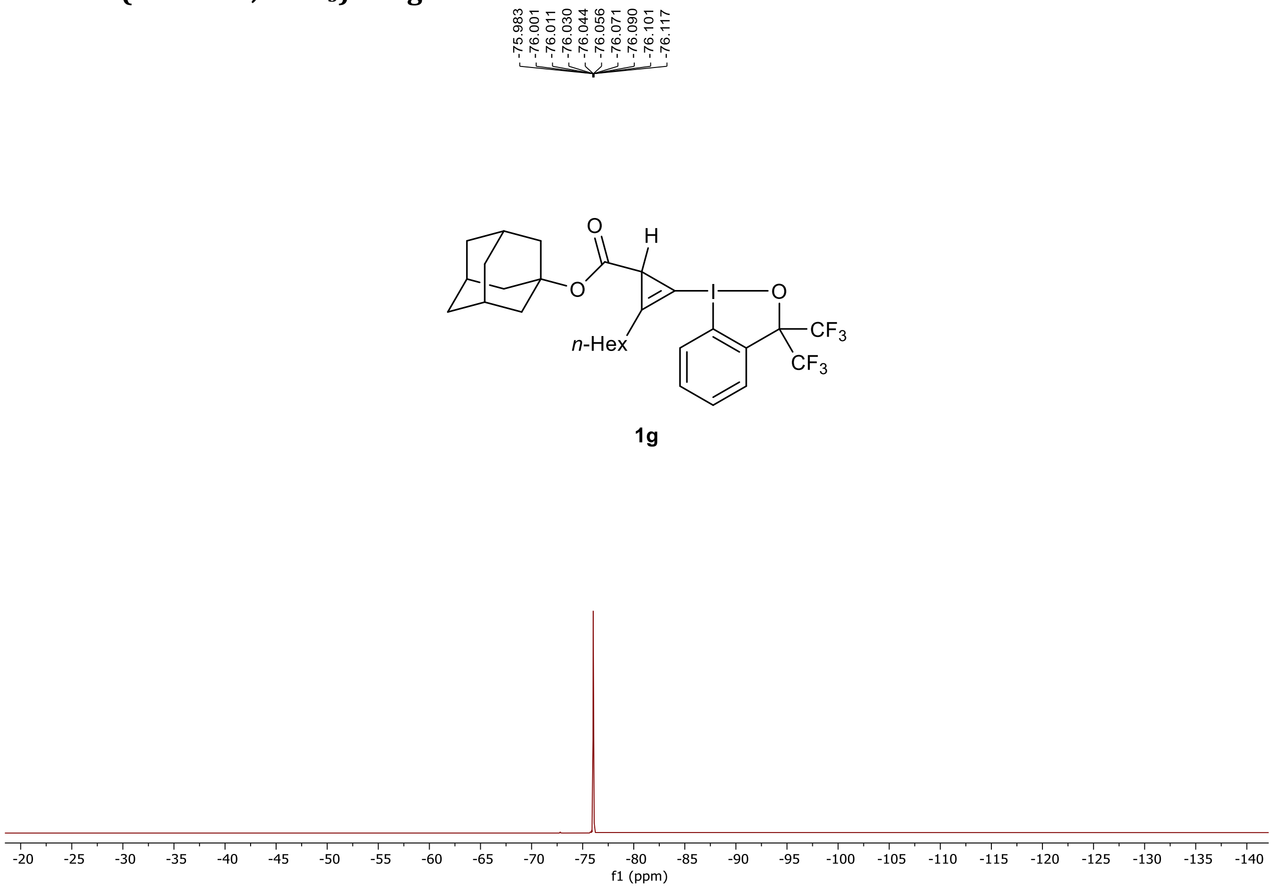

^{1}H NMR (400 MHz, CDCl$_3$) of 1h

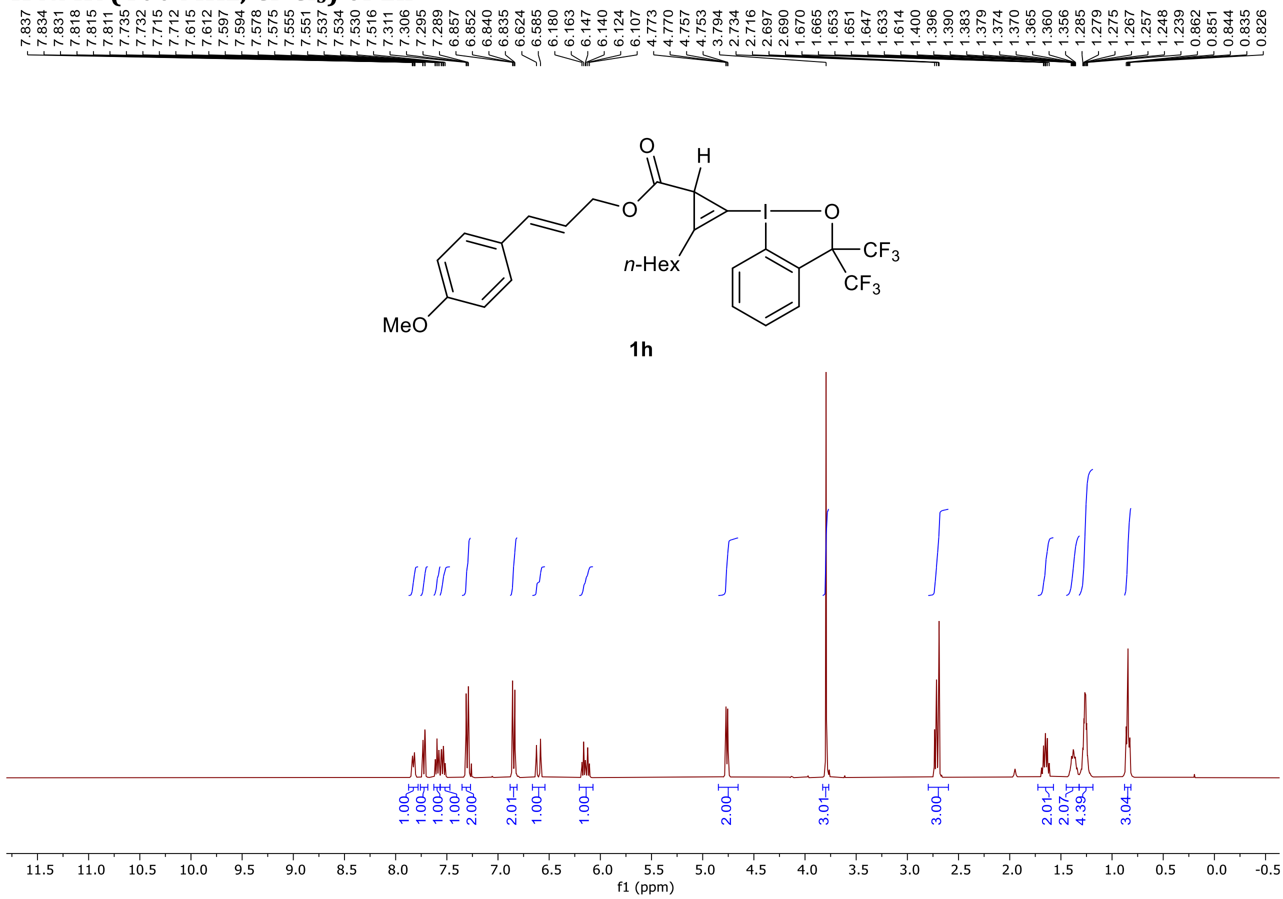

^{13}C NMR (101 MHz, $CDCl_3$) of 1h

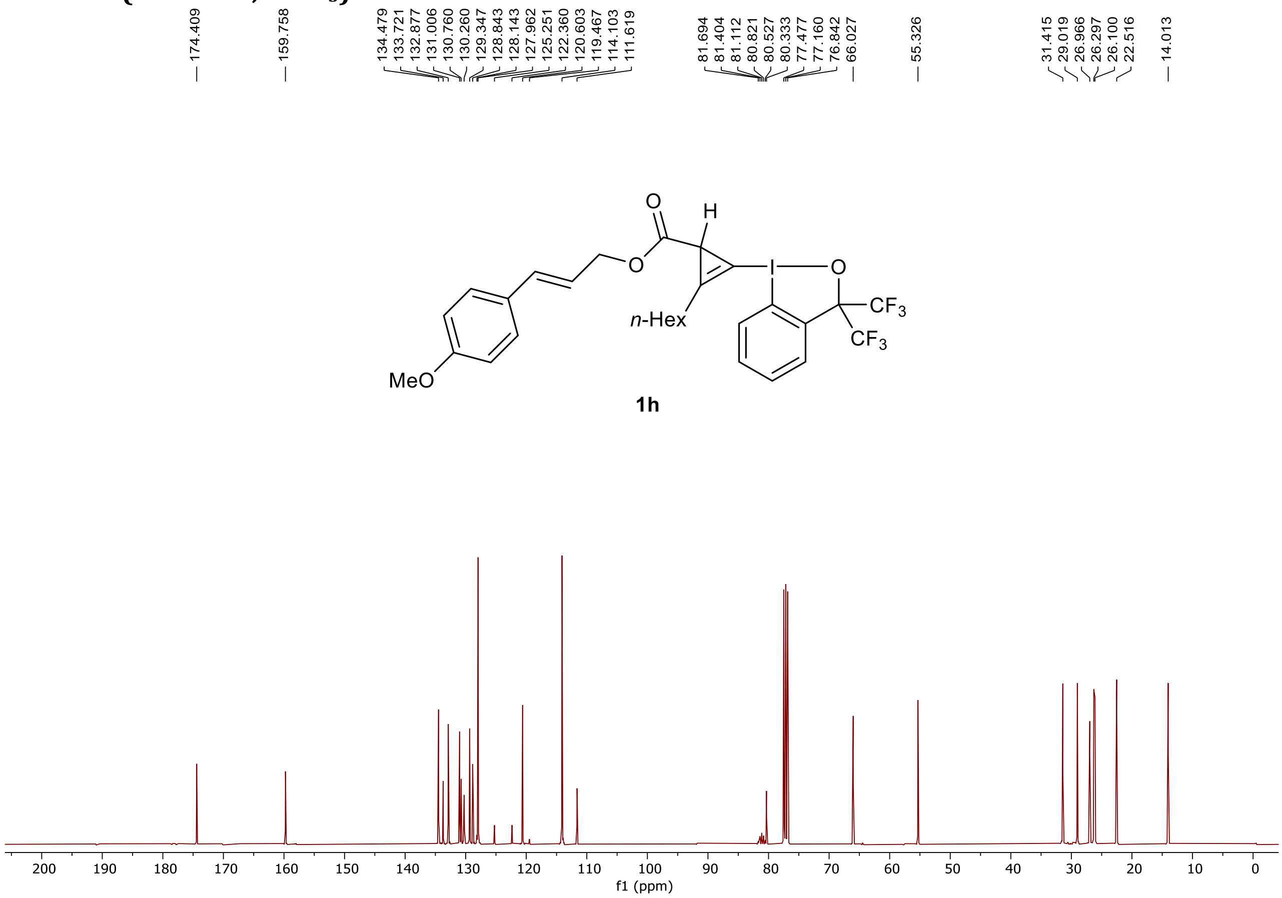

^{19}F NMR (377 MHz, $CDCl_3$) of 1h

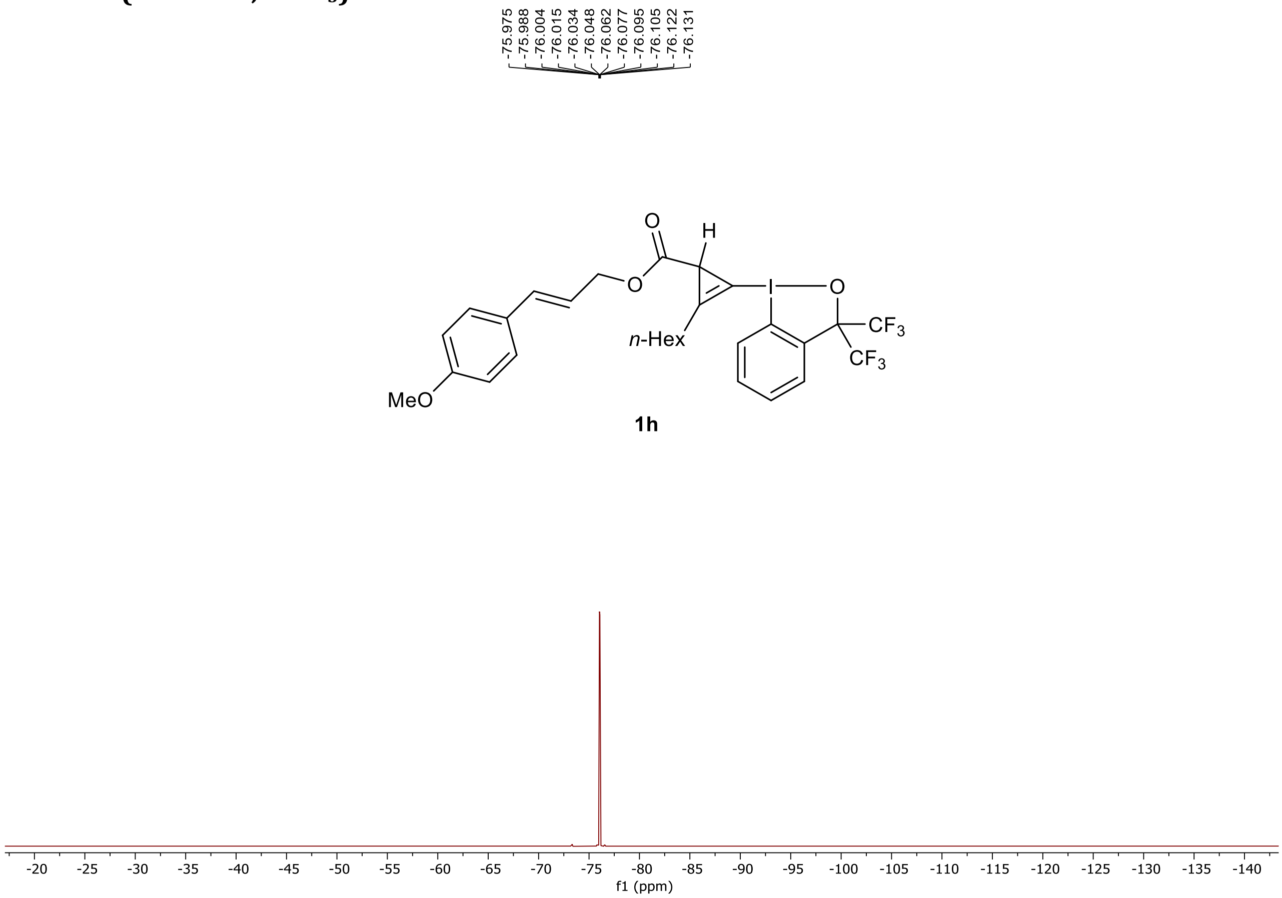

^{1}H NMR (400 MHz, $CDCl_3$) of 1i

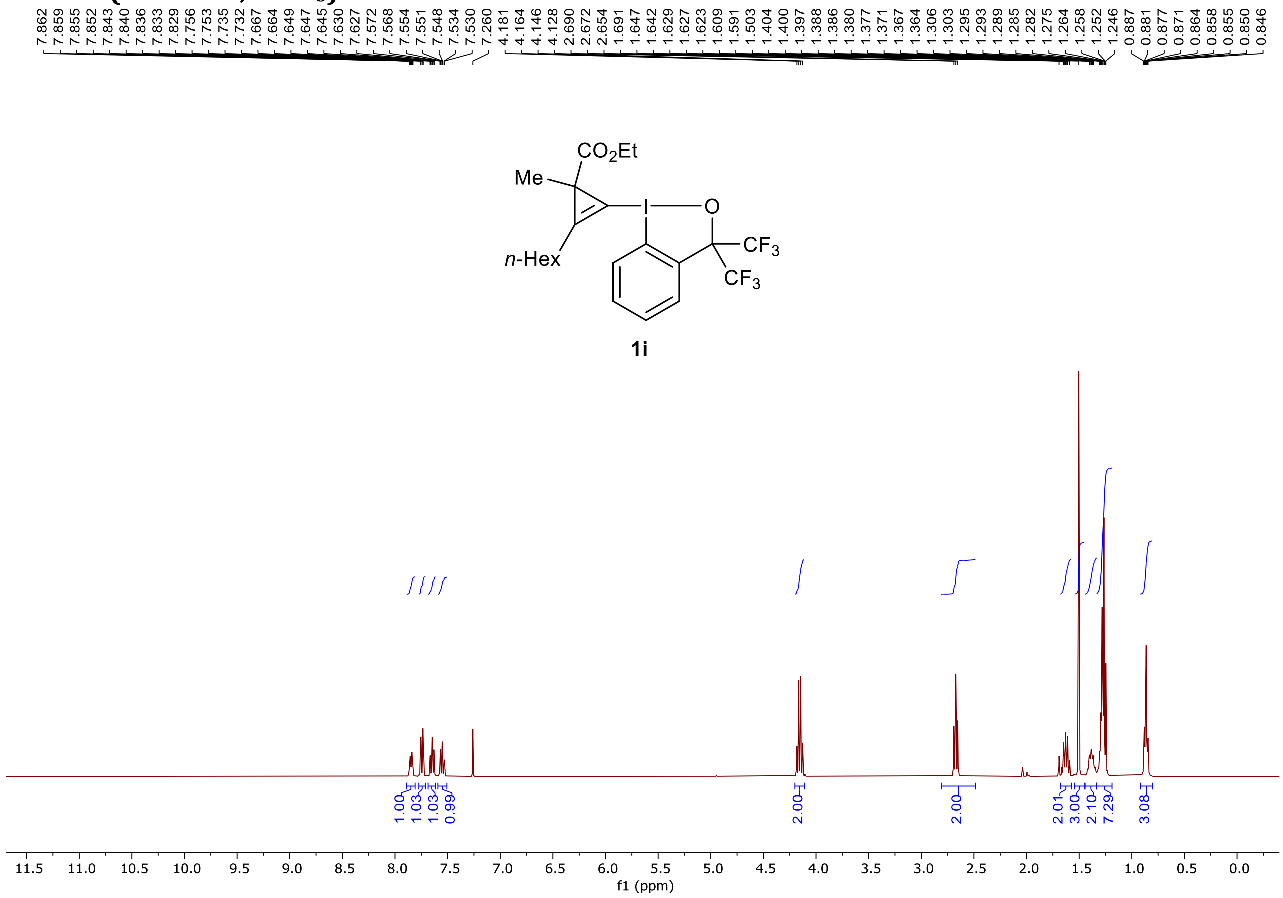

^{13}C NMR (101 MHz, $CDCl_3$) of 1i

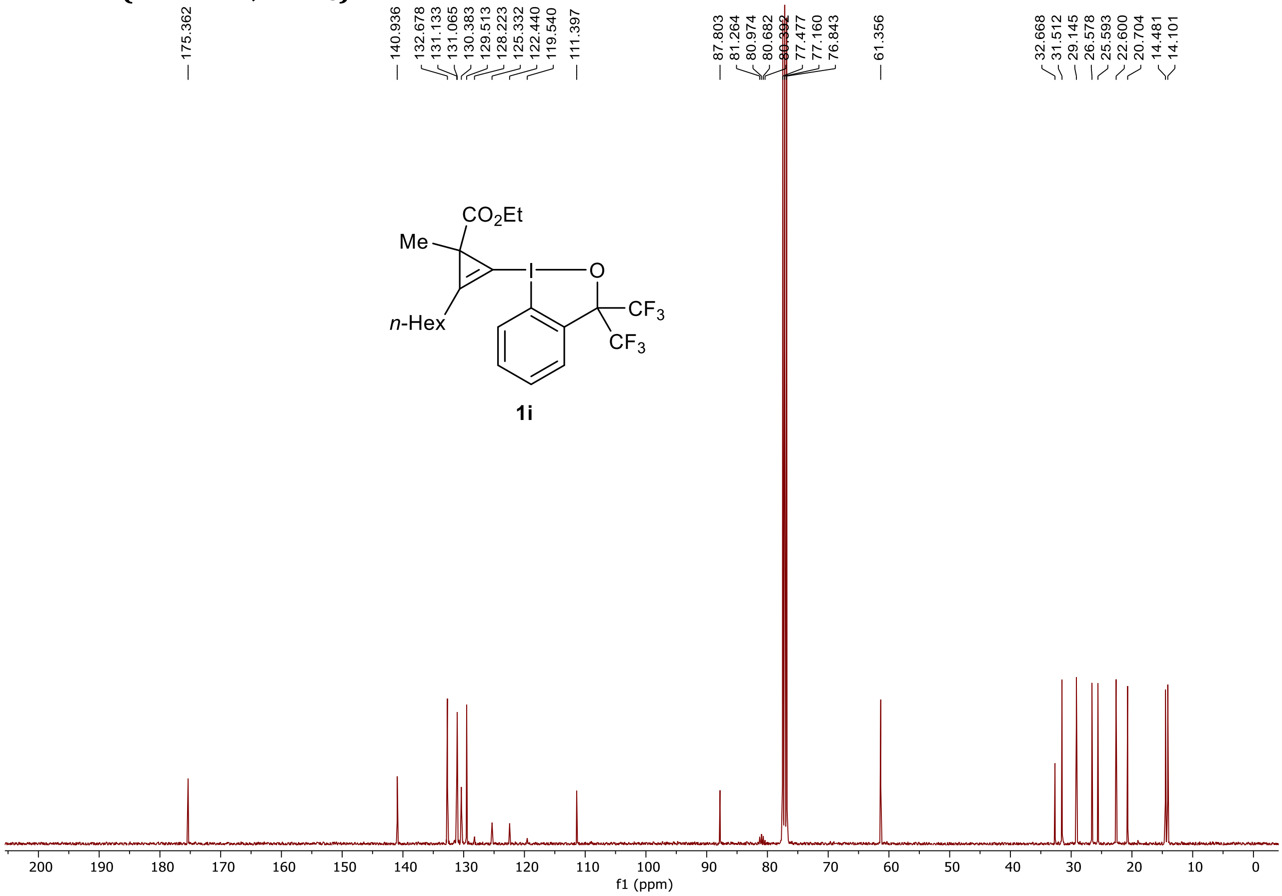

^{19}F NMR (377 MHz, $CDCl_3$) of 1i

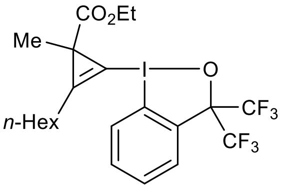

1i

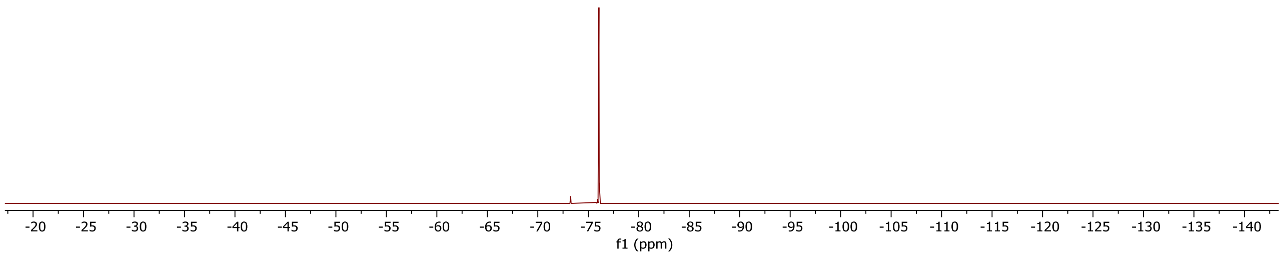

^{1}H NMR (400 MHz, $CDCl_3$) of 1j

7.894 7.890 7.887 7.883 7.875 7.871 7.867 7.864 7.697 7.694 7.679 7.676 7.660 7.657 7.591 7.587 7.573 7.569 7.566 7.552 7.548 7.402 7.399 7.381 7.379 7.318 7.315 7.311 7.302 7.297 7.294 7.282 7.278 7.274 7.260 7.237 7.233 7.230 7.221 7.215 7.209 7.197 7.170 7.166 7.160 7.153 7.149 7.146 2.775 2.757 2.749 2.739 2.730 2.712 2.476 2.465 2.454 2.442 2.070 2.051 2.033 2.014 1.996 1.672

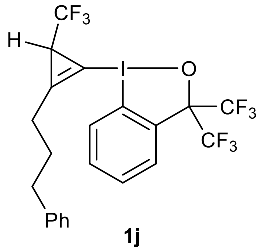

1j

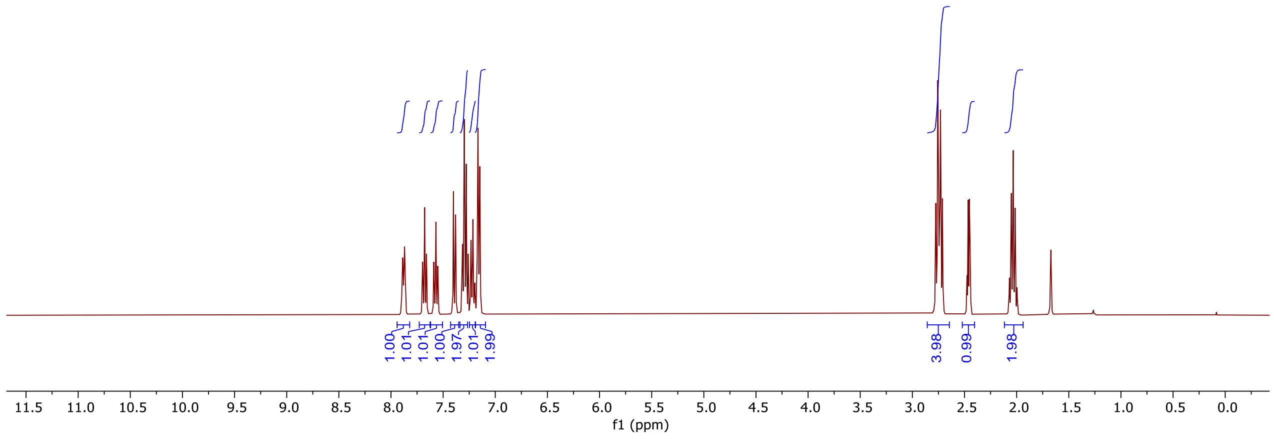

^{13}C NMR (101 MHz, $CDCl_3$) of 1j

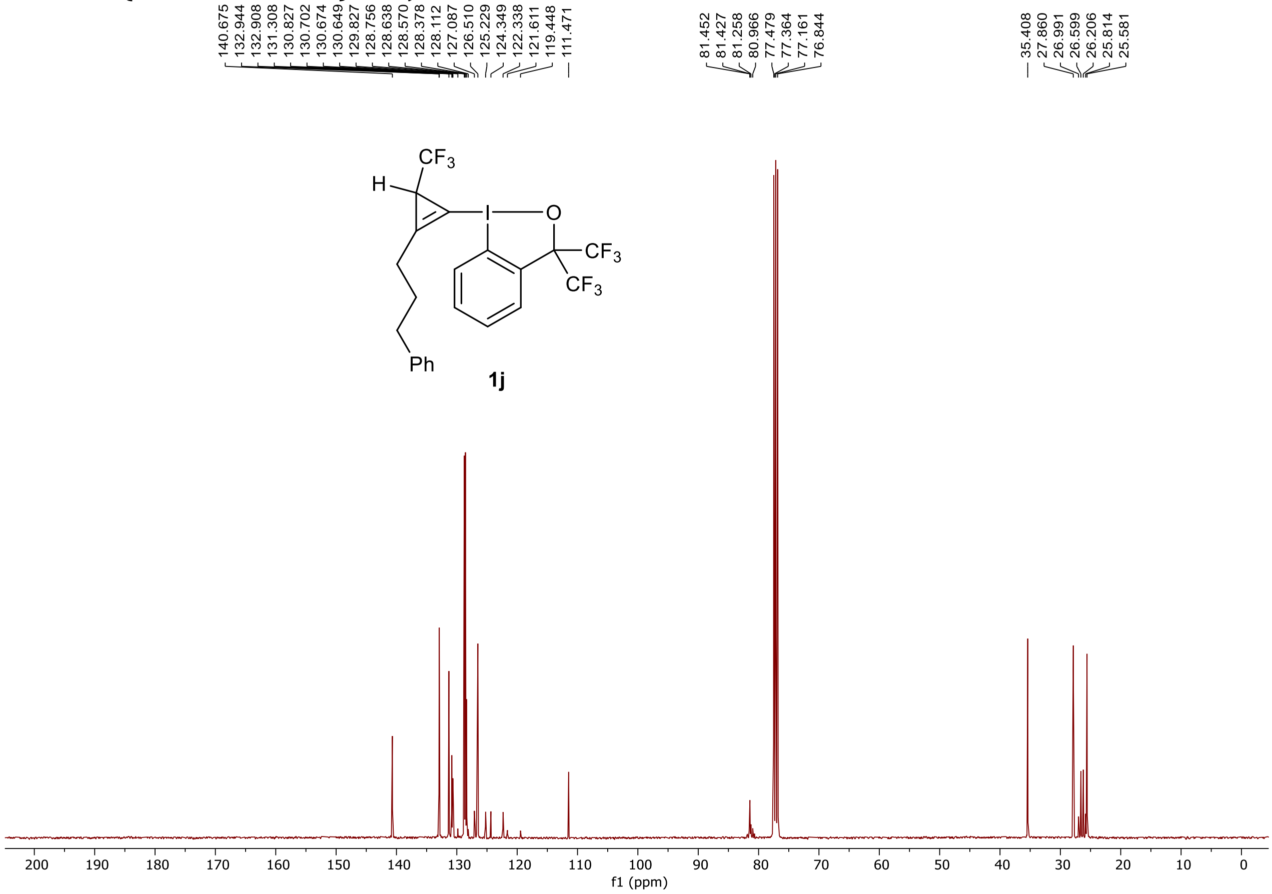

^{19}F NMR (377 MHz, $CDCl_3$) of 1j

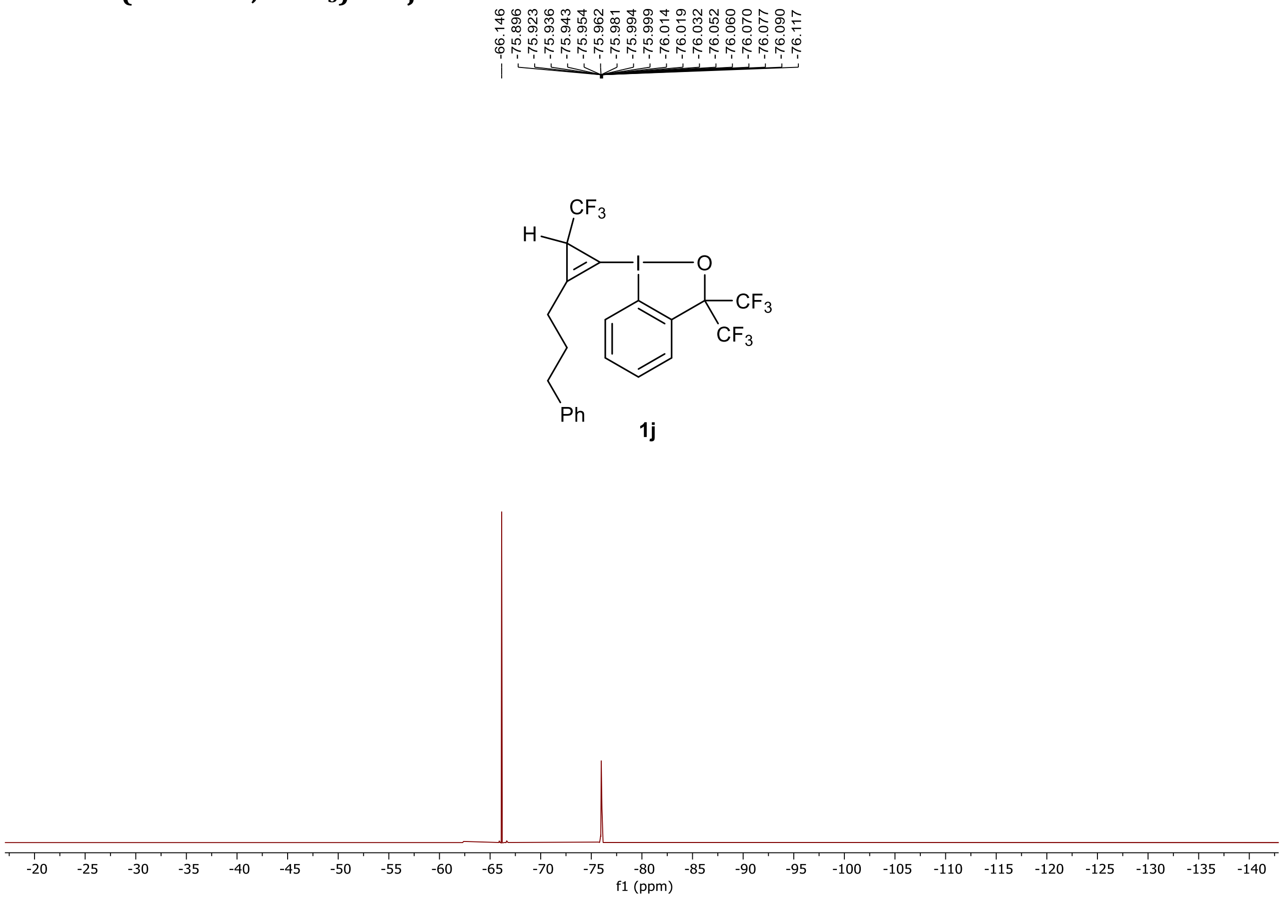

^{1}H NMR (400 MHz, $CDCl_3$) of 1k

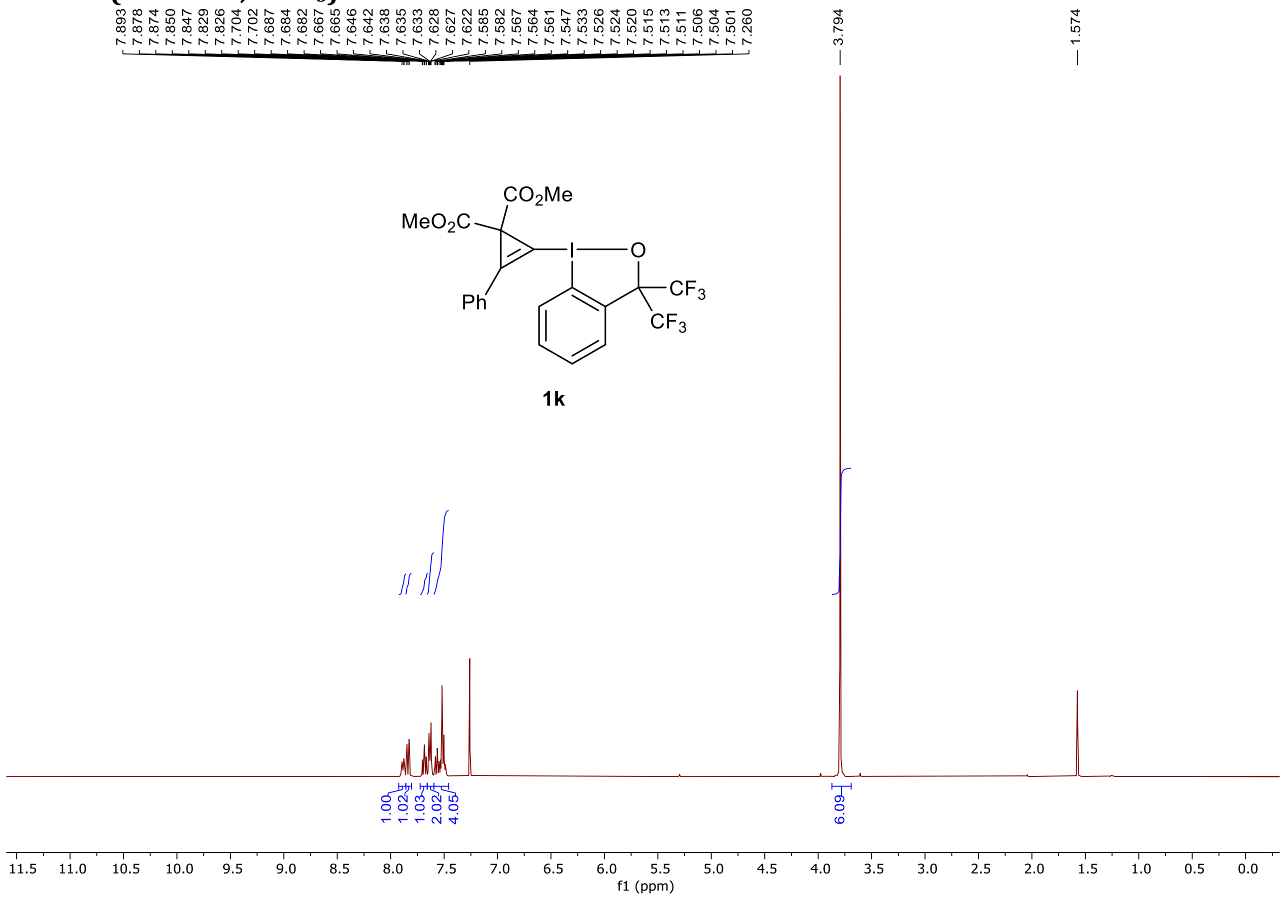

^{13}C NMR (101 MHz, $CDCl_3$) of 1k

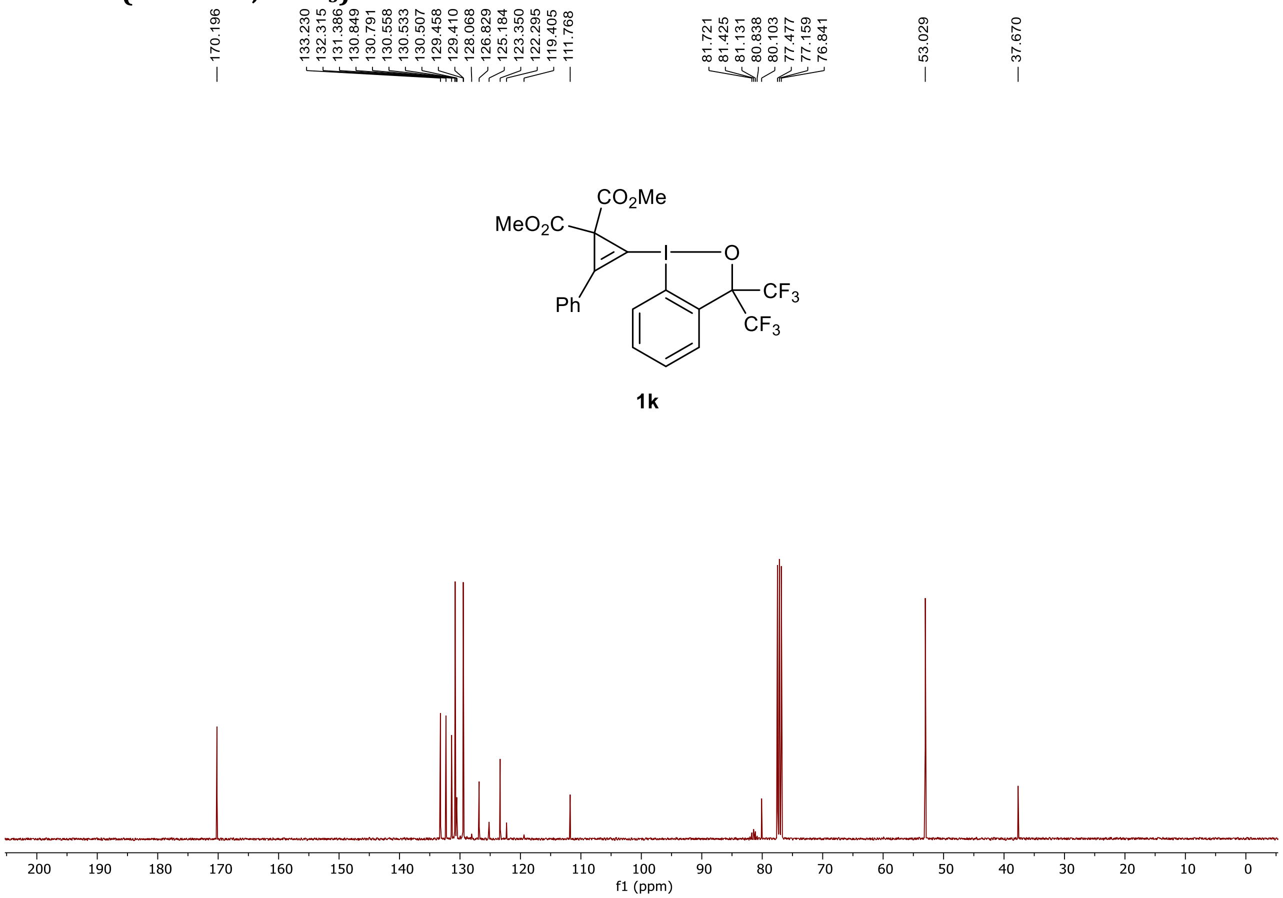

^{19}F NMR (377 MHz, $CDCl_3$) of 1k

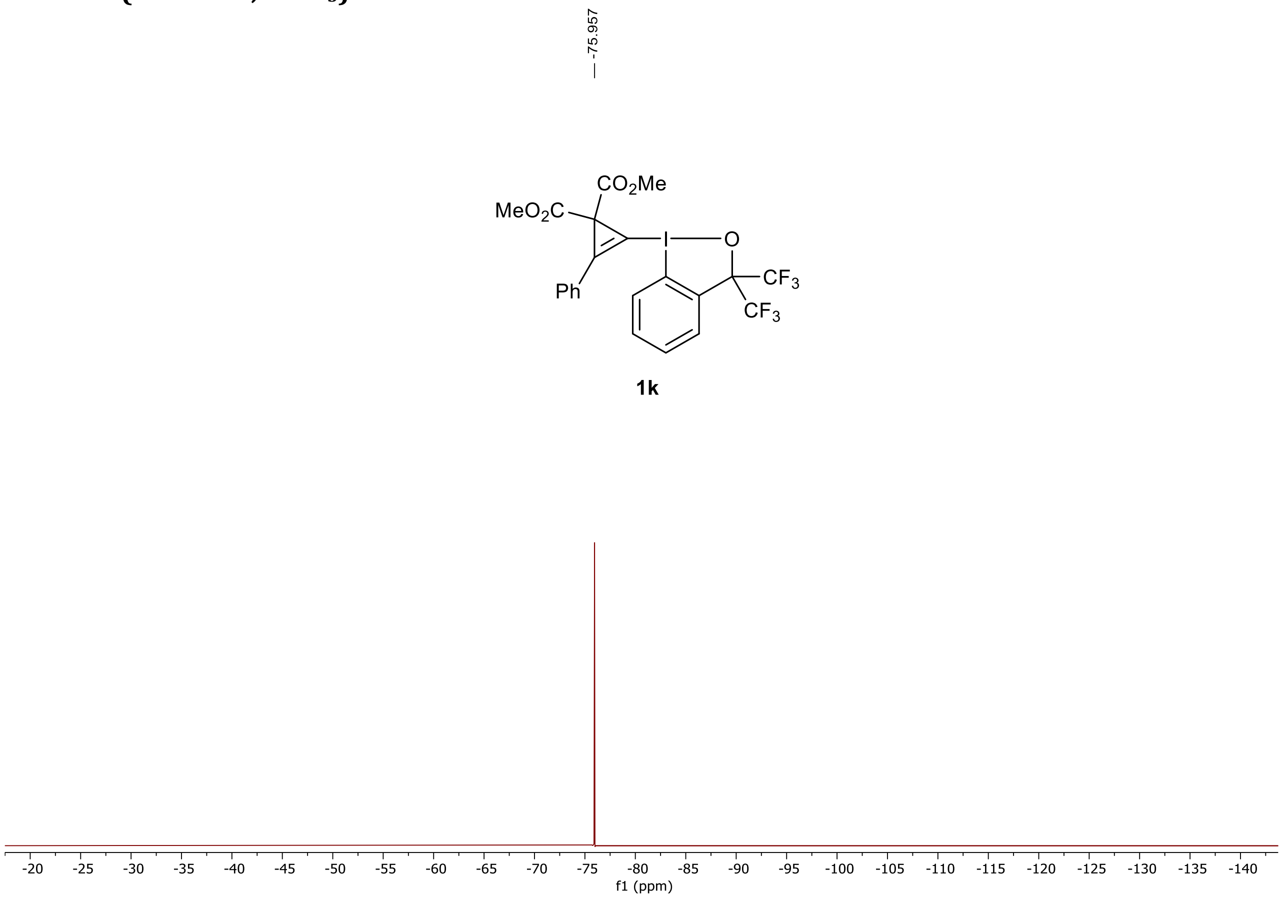

^{1}H NMR (400 MHz, $CDCl_3$) of 1l

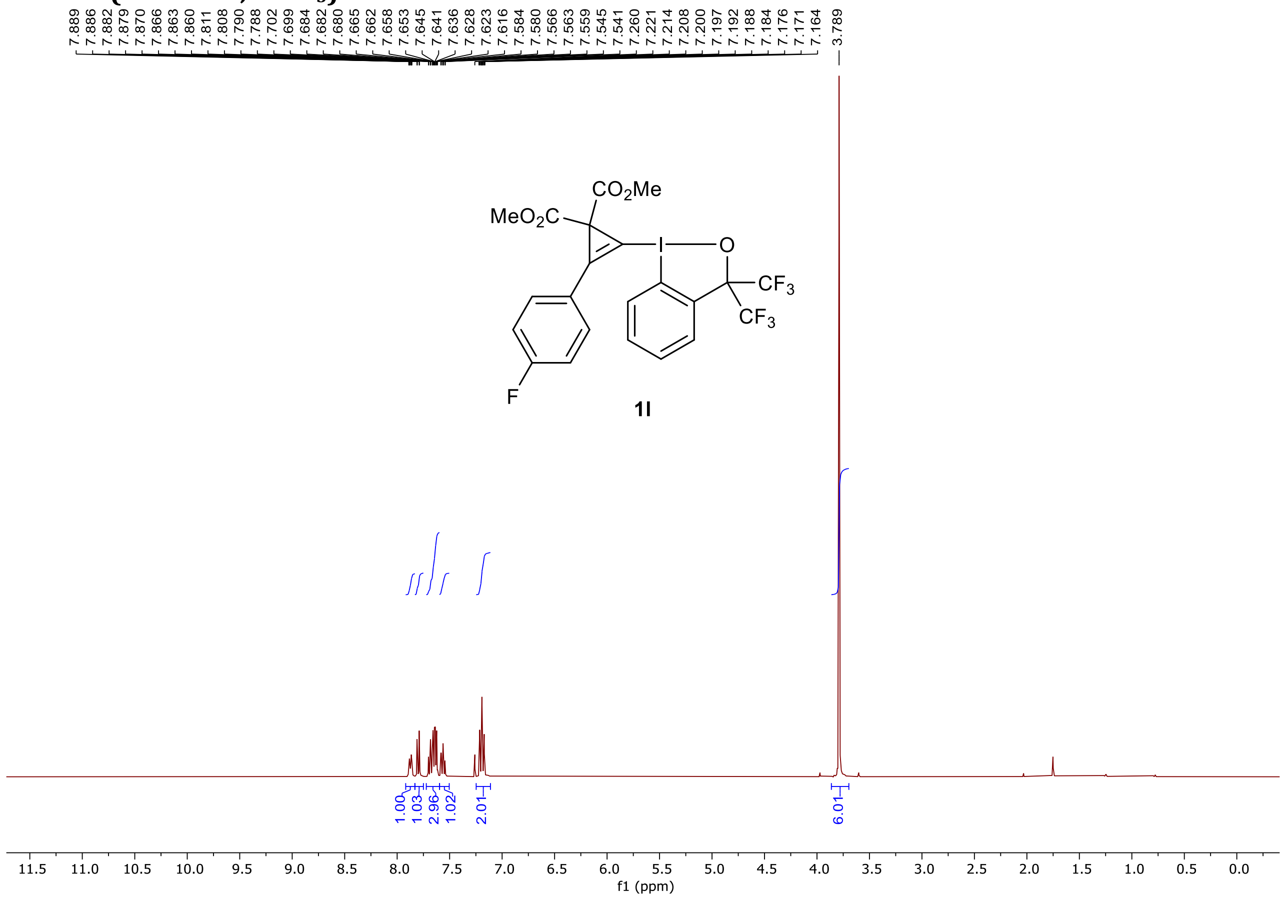

^{13}C NMR (101 MHz, $CDCl_3$) of 1l

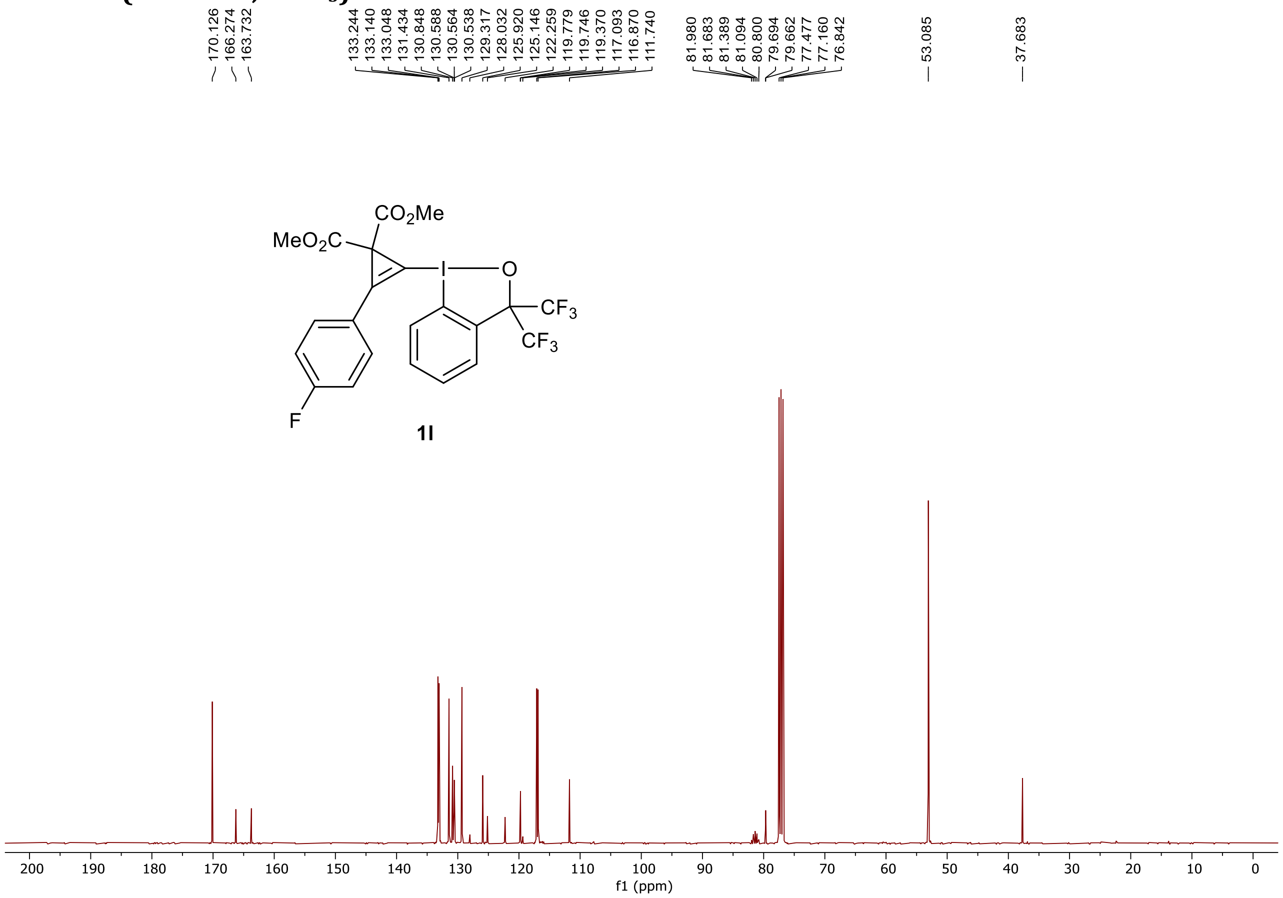

^{19}F NMR (377 MHz, $CDCl_3$) of 1l

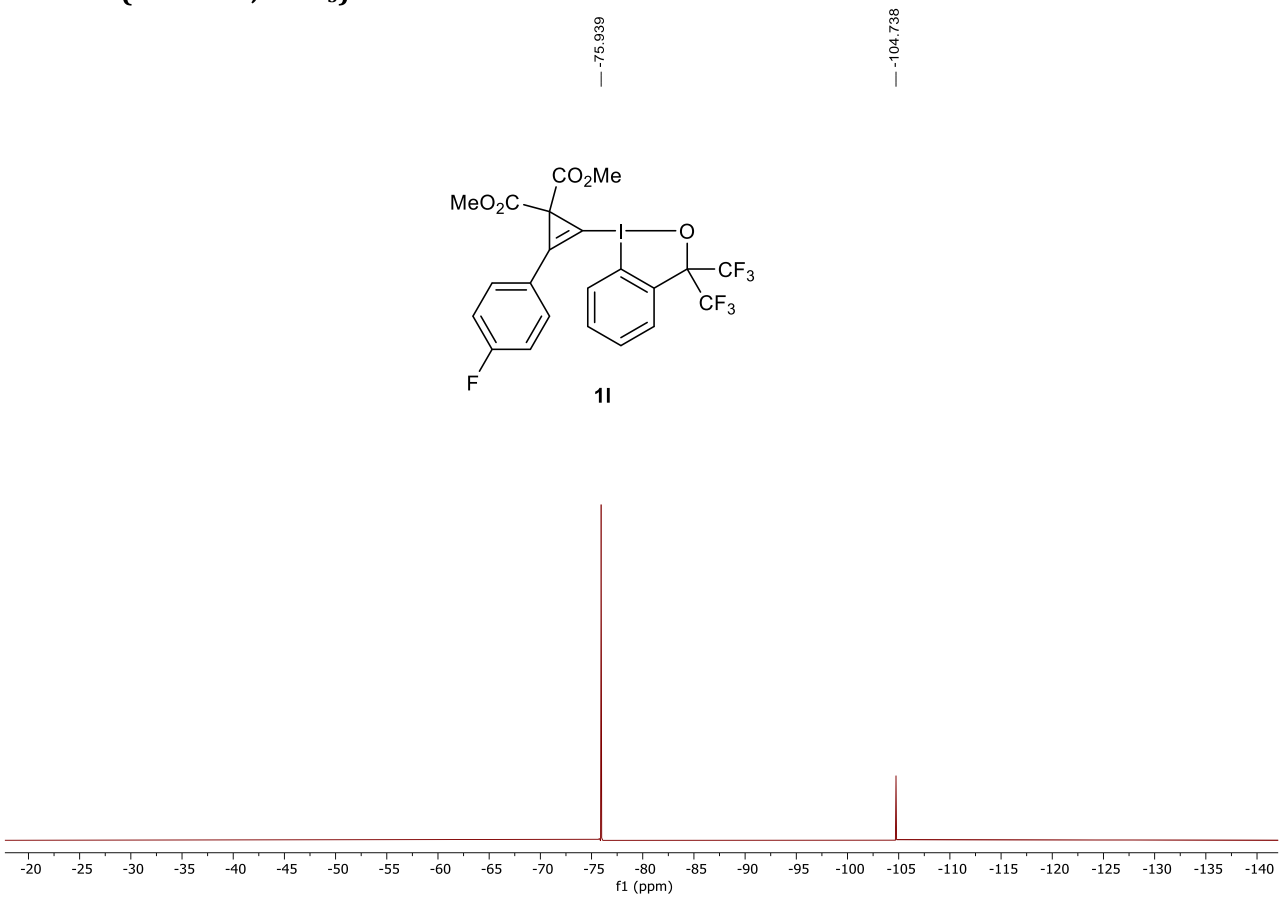

^1H NMR (400 MHz, $CDCl_3$) of 1m

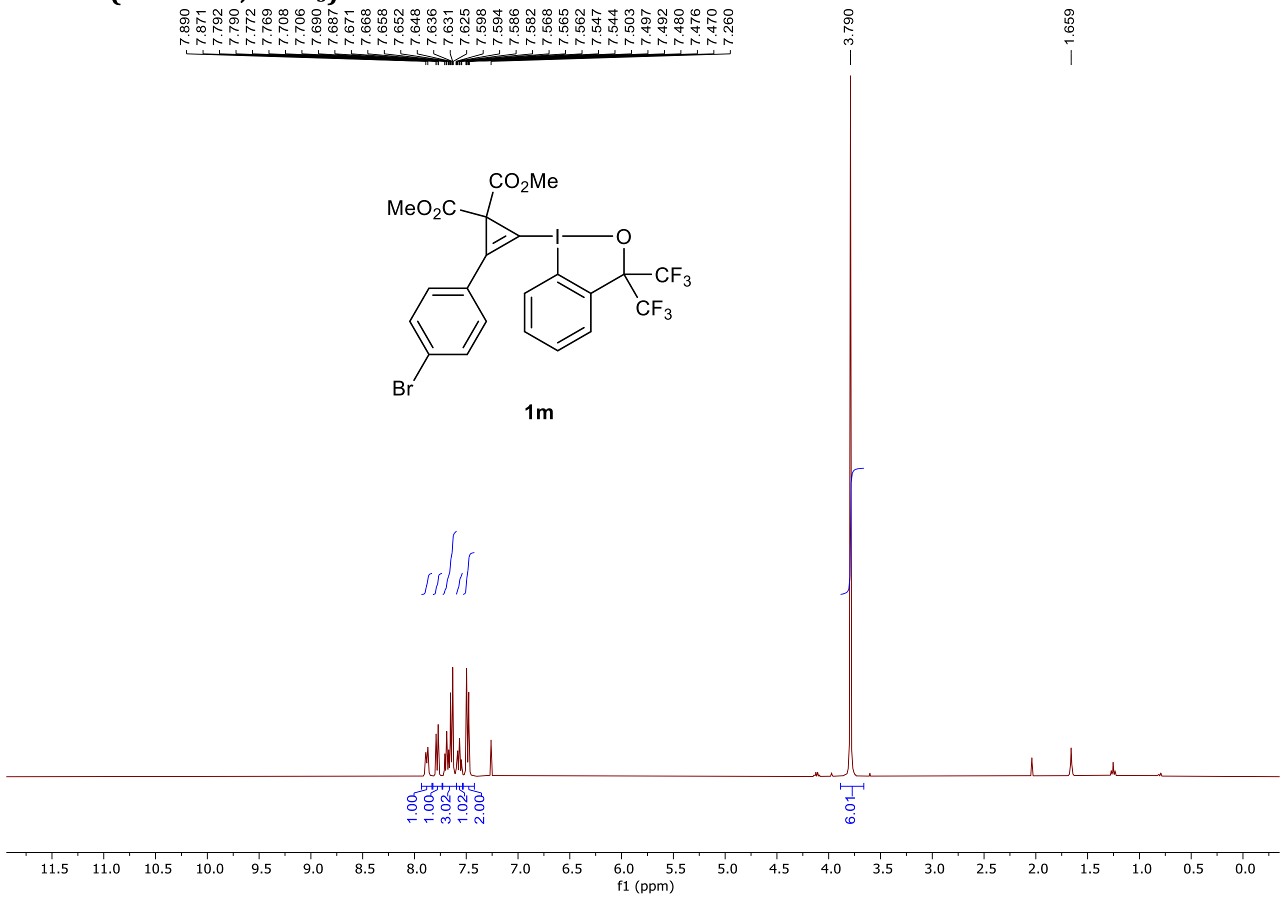

^{13}C NMR (101 MHz, $CDCl_3$) of 1m

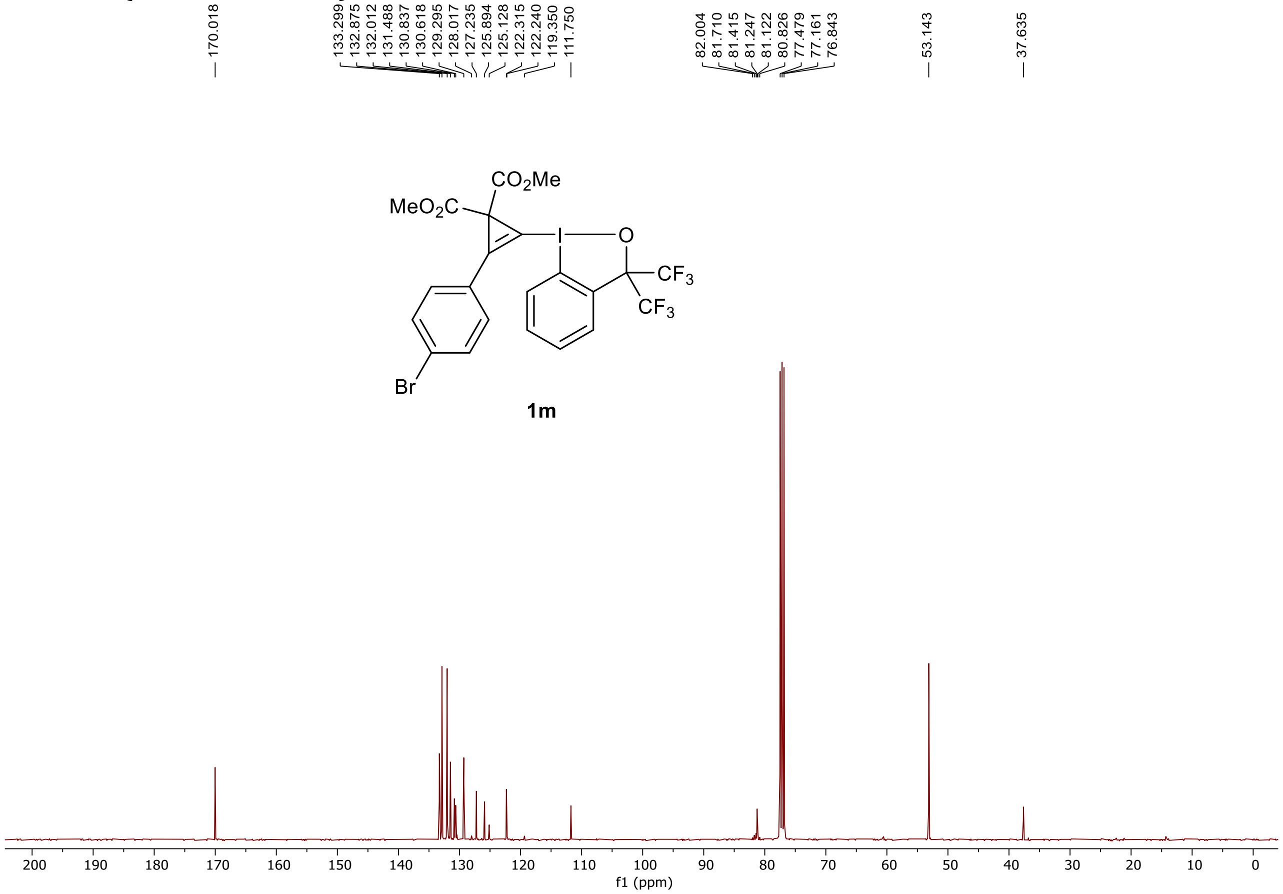

^{19}F NMR (377 MHz, $CDCl_3$) of 1m

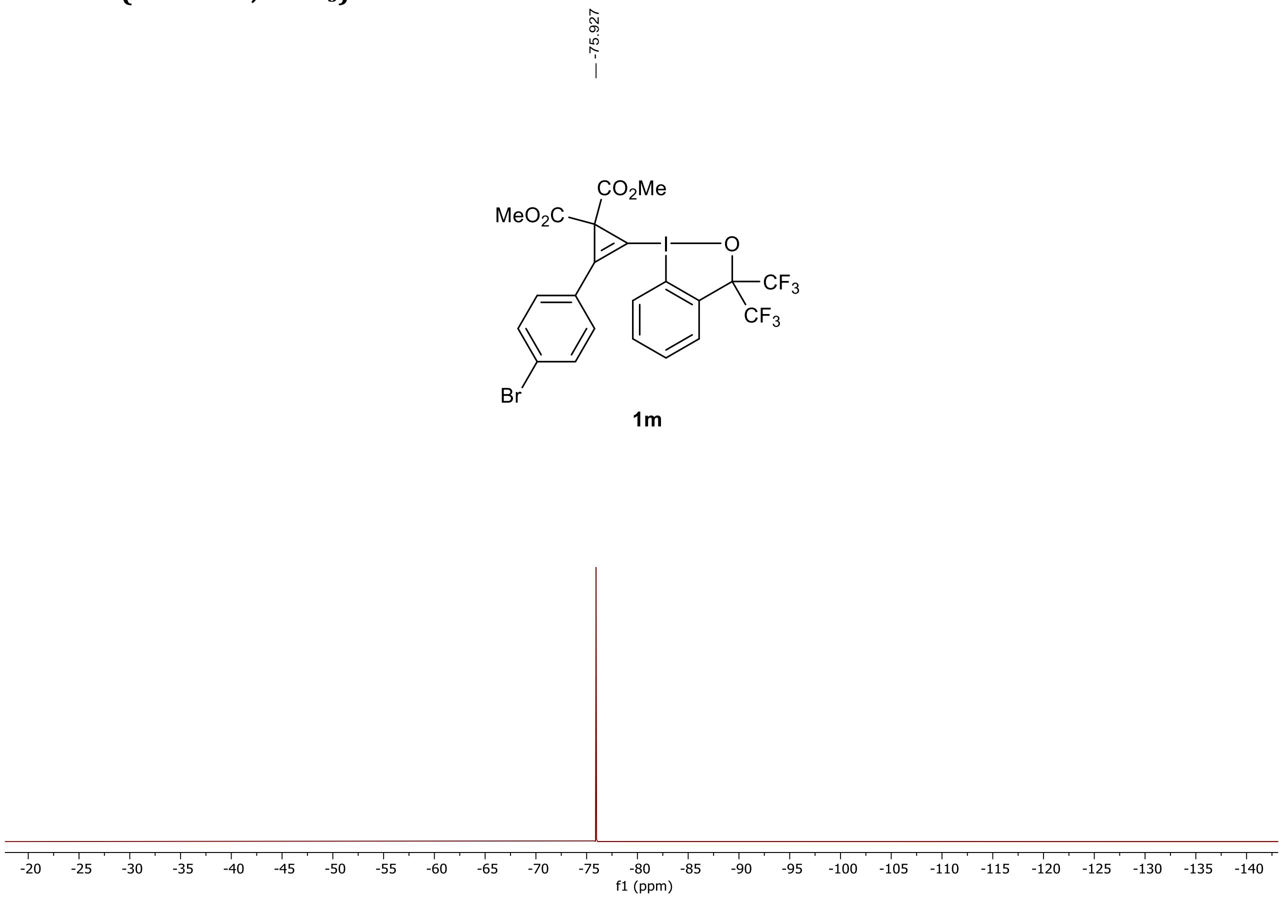

^{1}H NMR (400 MHz, $CDCl_3$) of 1n

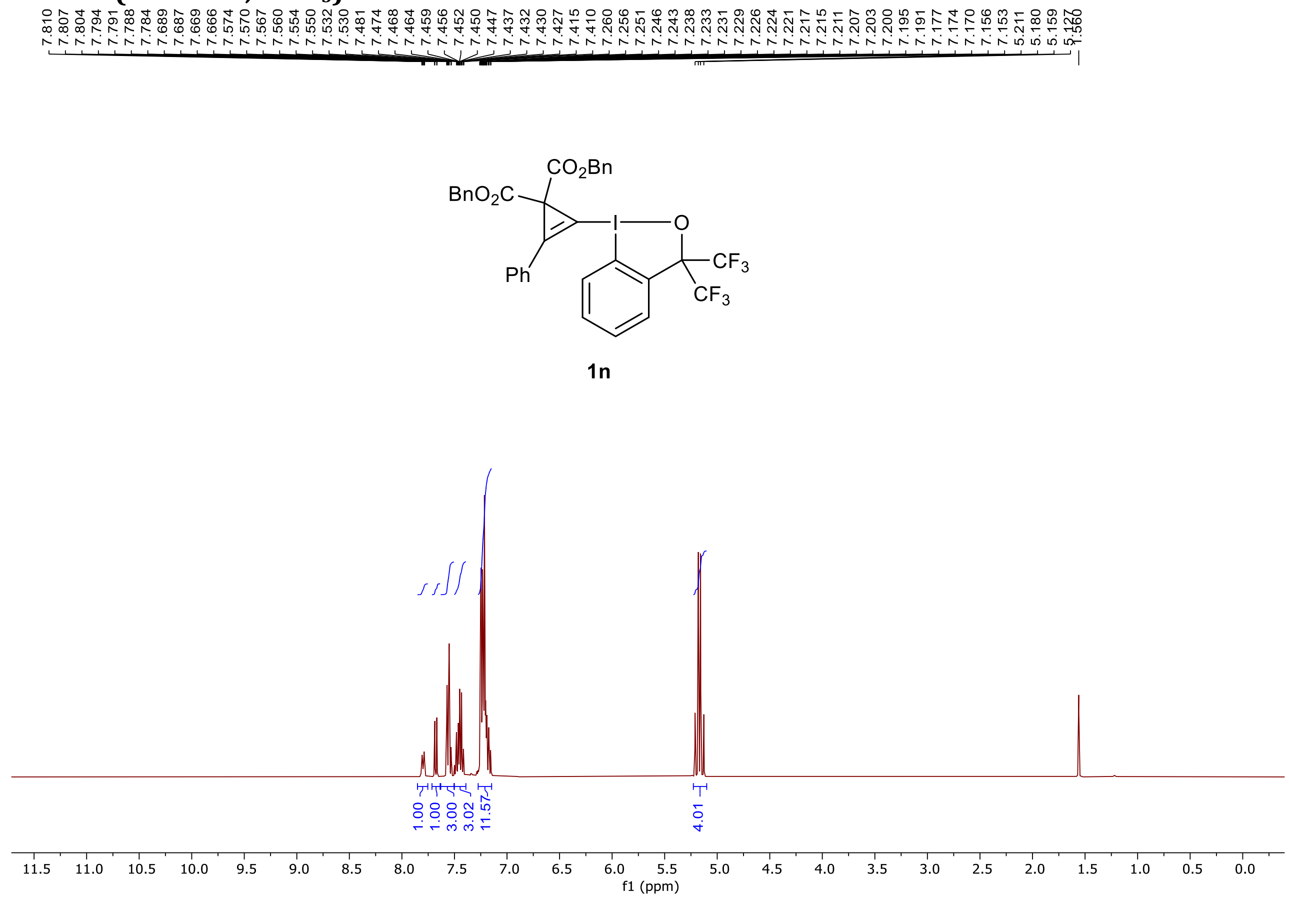

^{13}C NMR (101 MHz, $CDCl_3$) of 1n

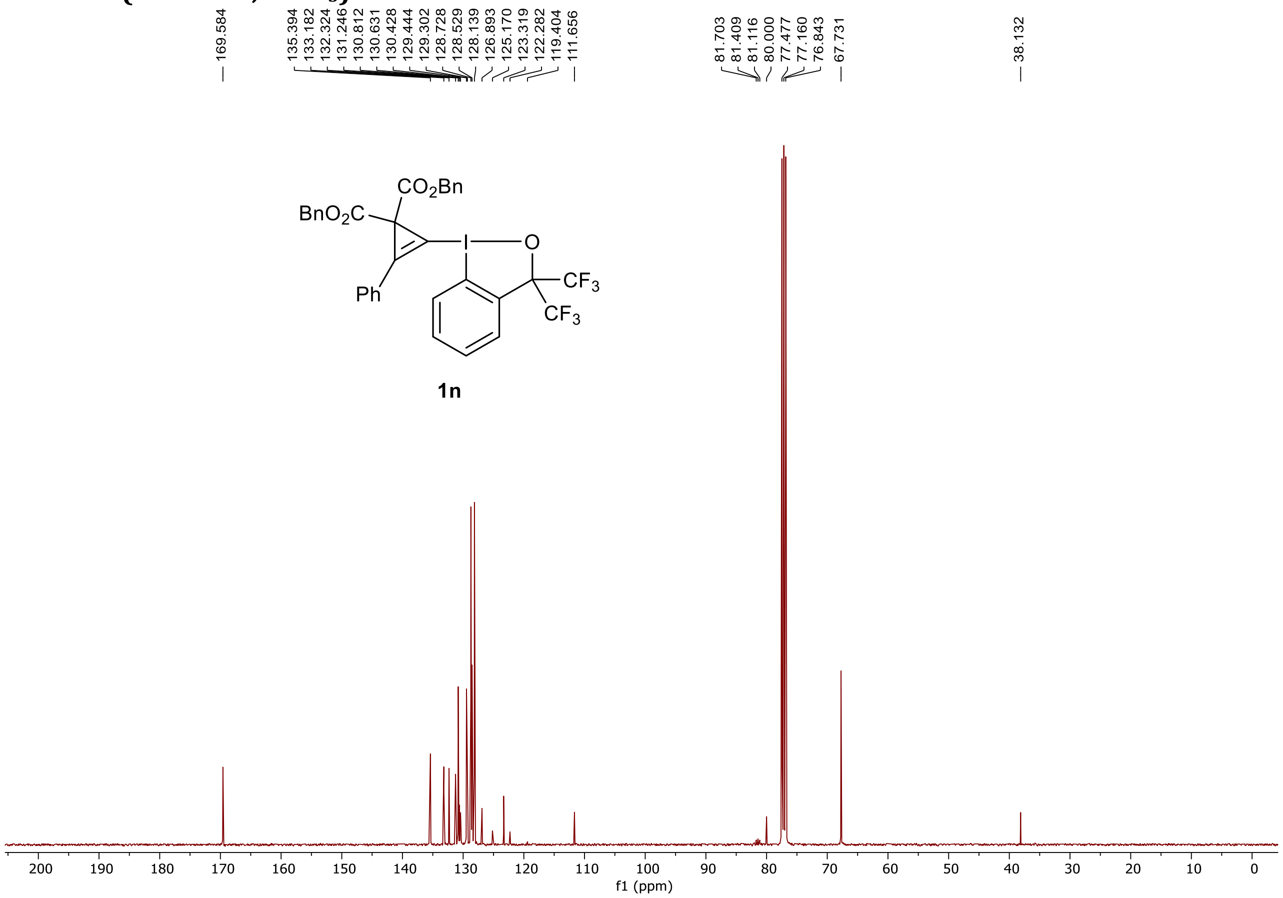

^{19}F NMR (377 MHz, $CDCl_3$) of 1n

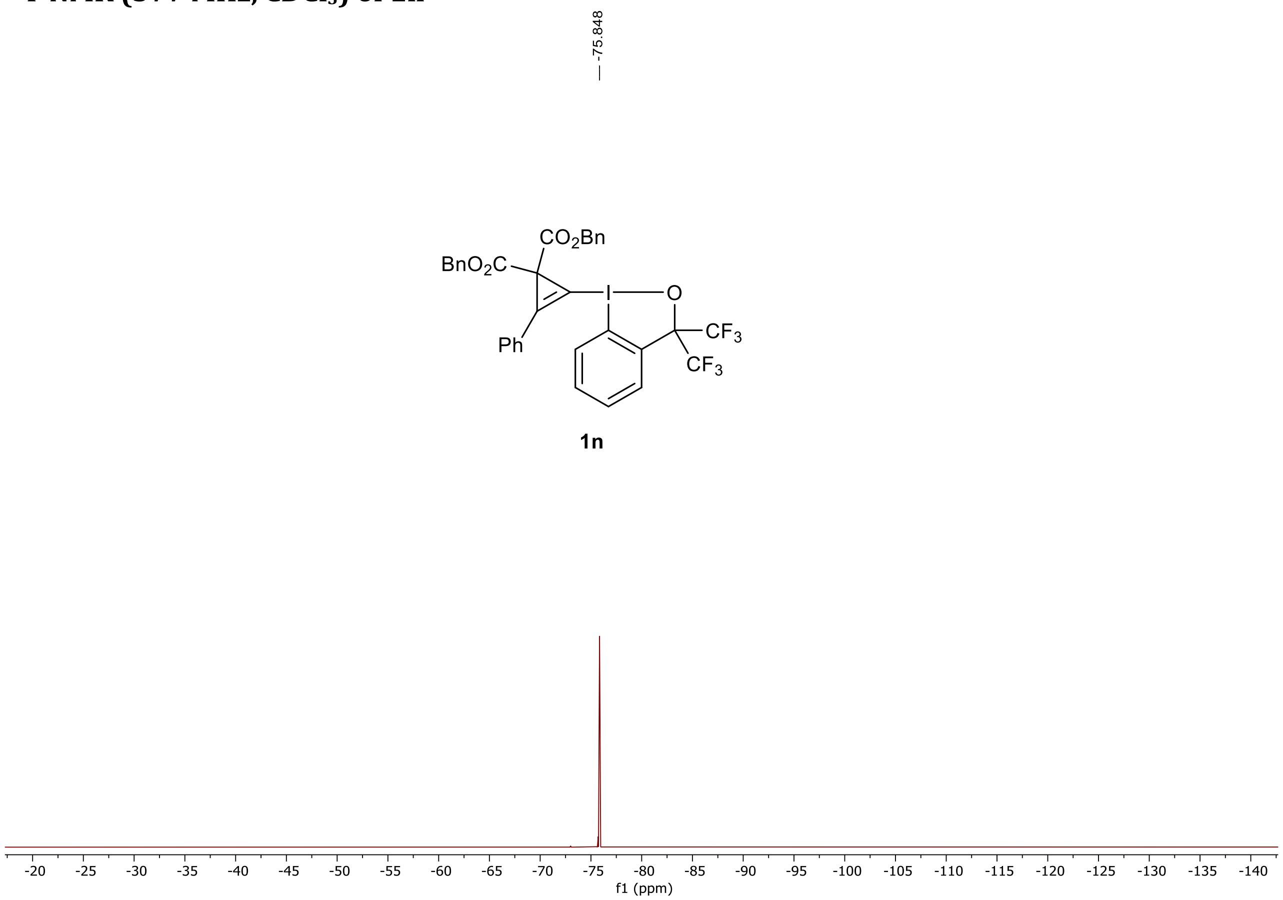

^{1}H NMR (400 MHz, $CDCl_3$) of 1o

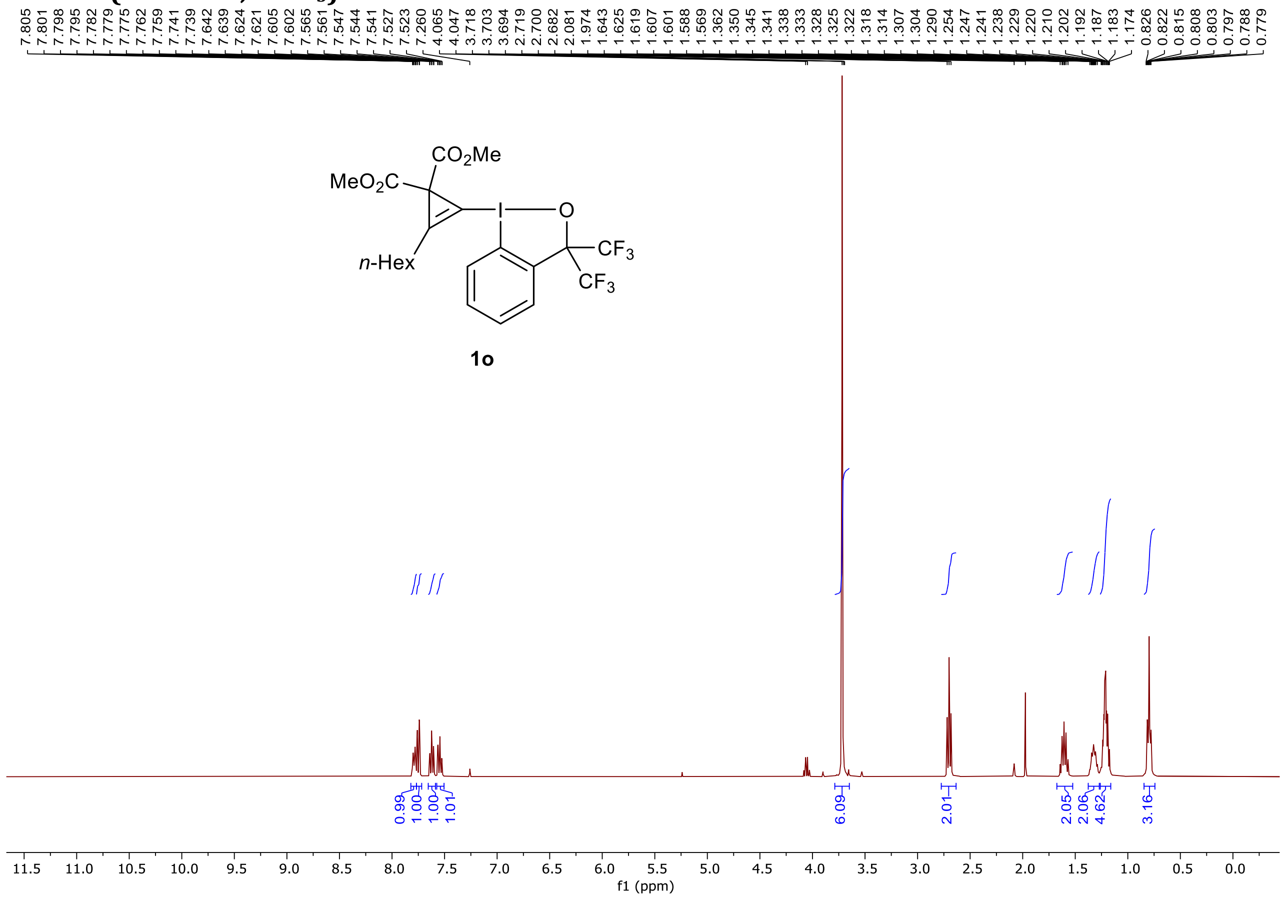

^{13}C NMR (101 MHz, $CDCl_3$) of 1o

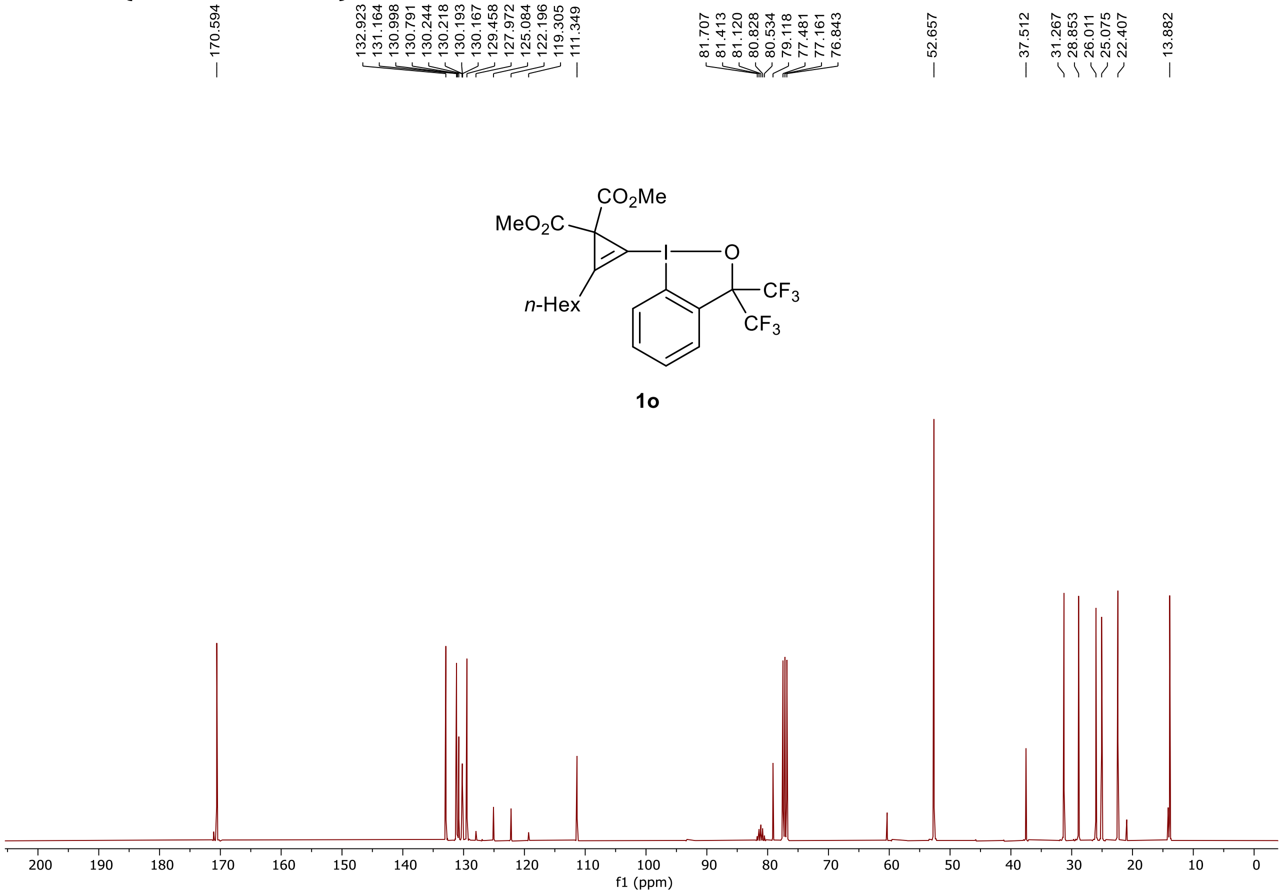

^{19}F NMR (377 MHz, $CDCl_3$) of 1o

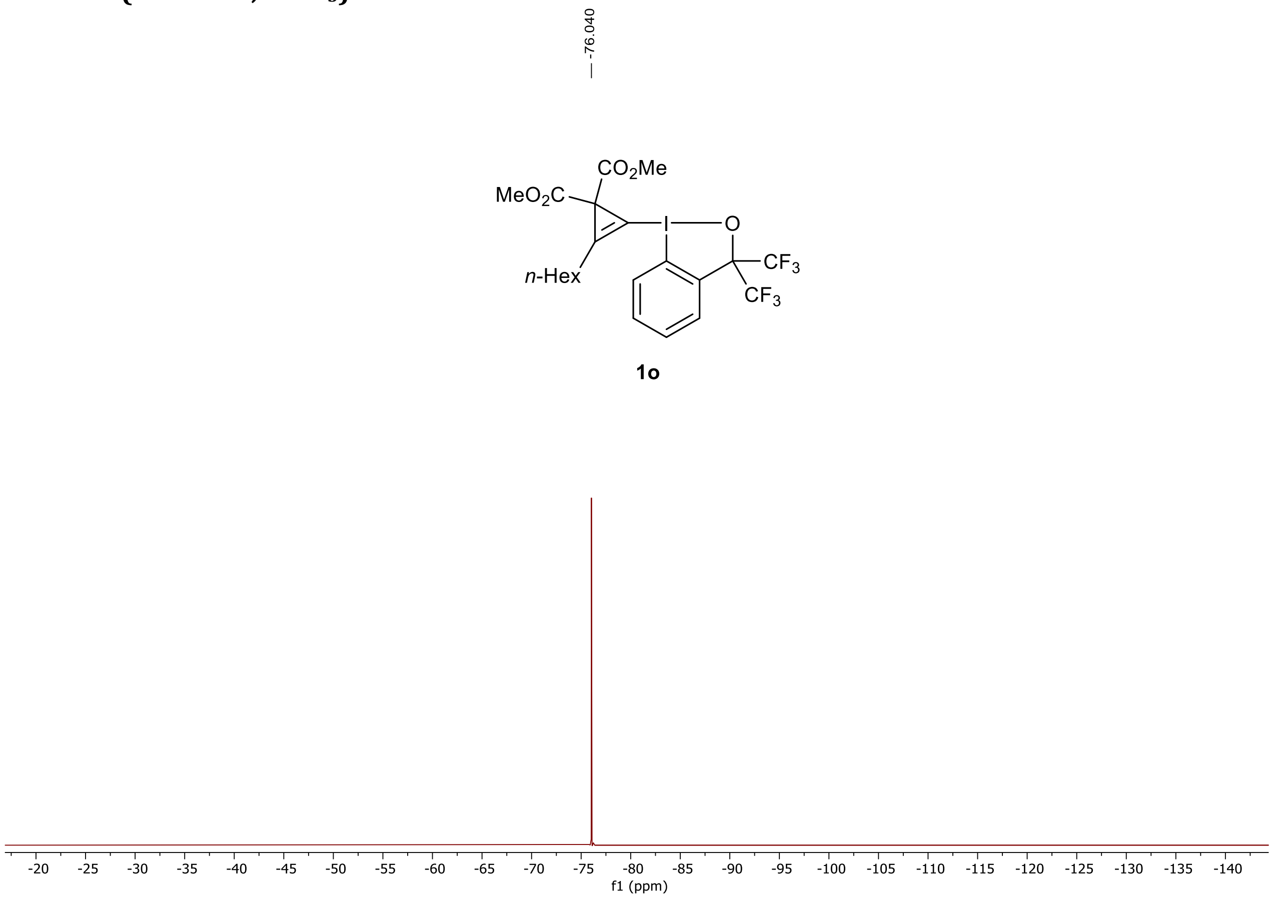

^{1}H NMR (400 MHz, $CDCl_3$) of 1a-1

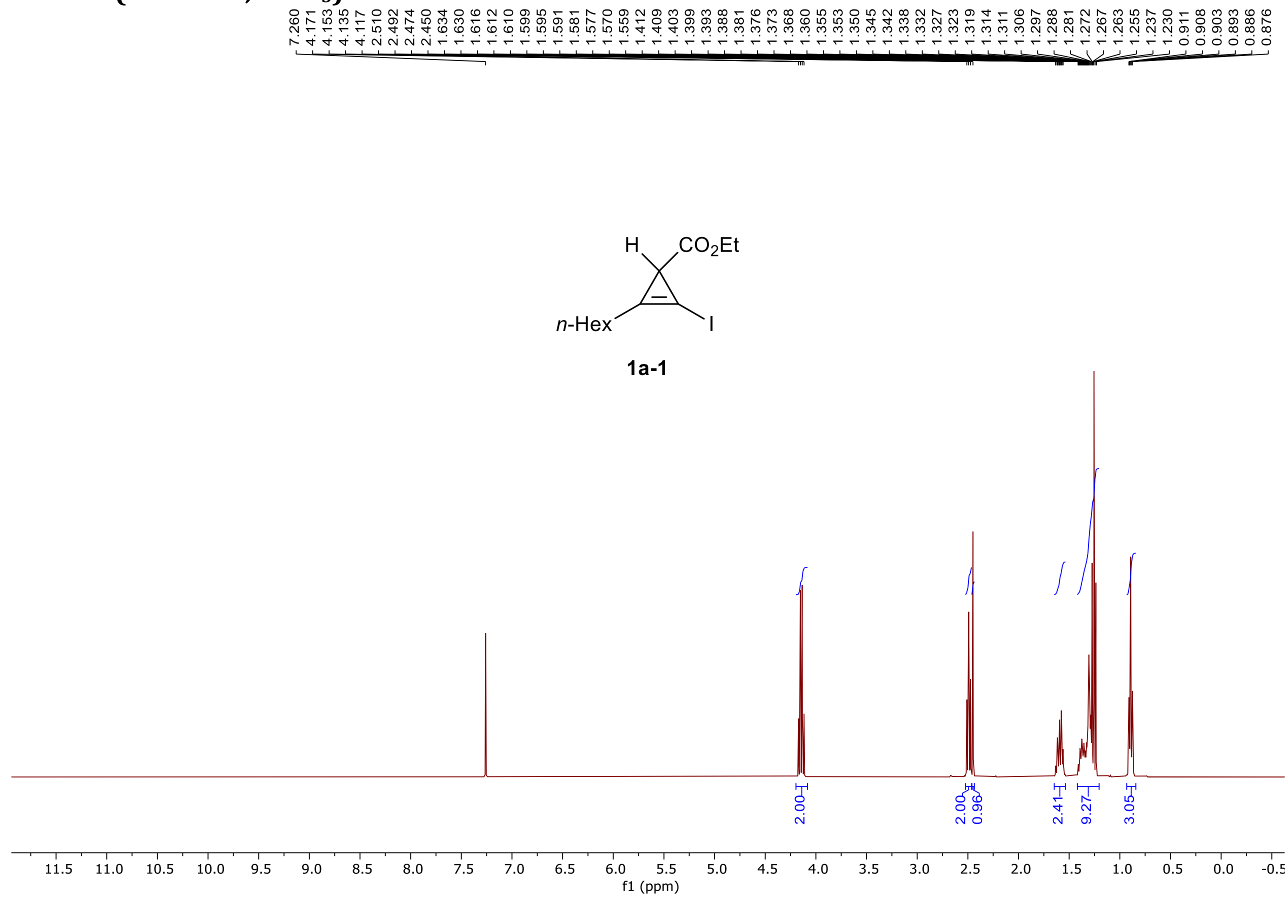

^{13}C NMR (101 MHz, $CDCl_3$) of 1a-1

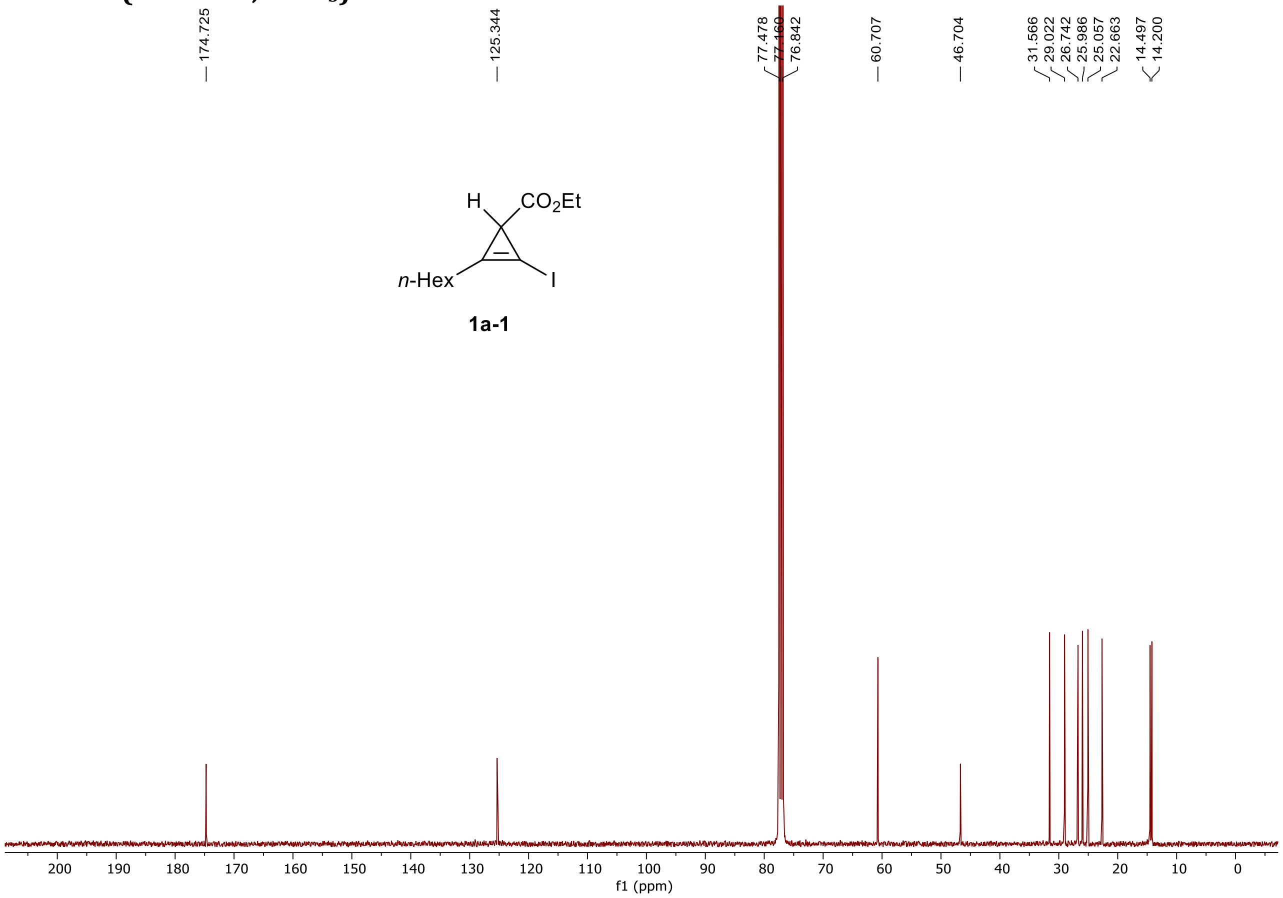

^{1}H NMR (400 MHz, $CDCl_3$) of 2a-4

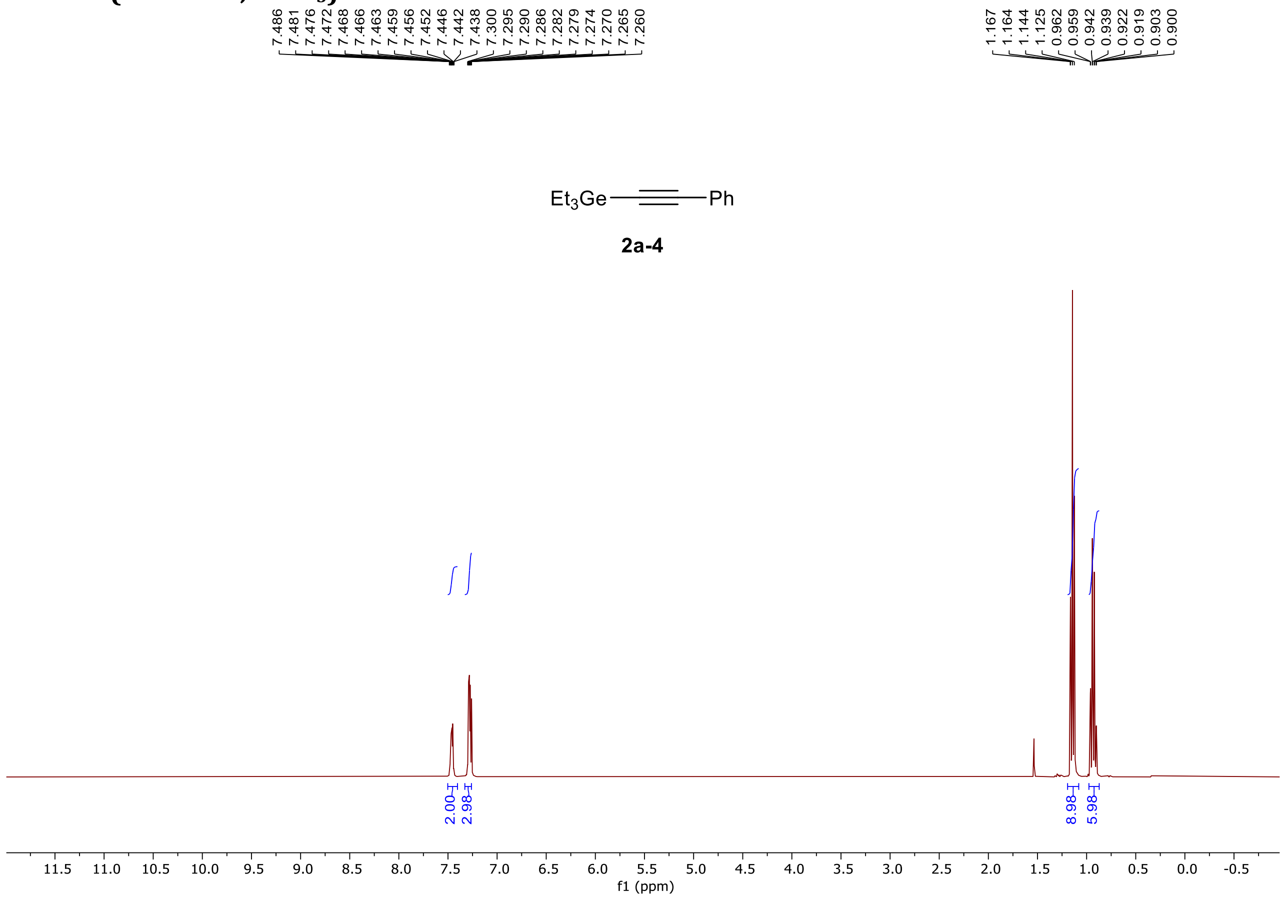

^{13}C NMR (101 MHz, $CDCl_3$) of 2a-4

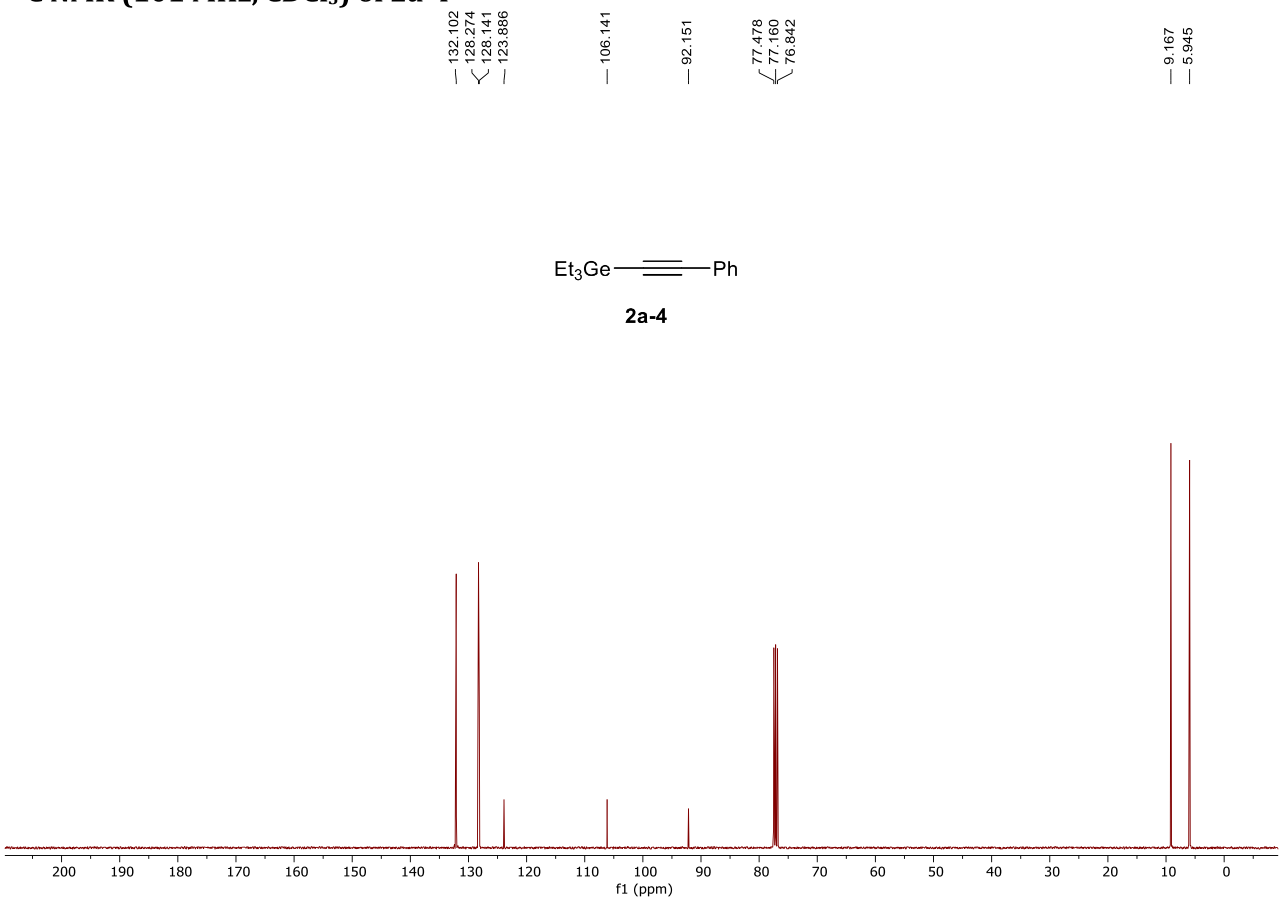

^{1}H NMR (400 MHz, $CDCl_3$) of 2c

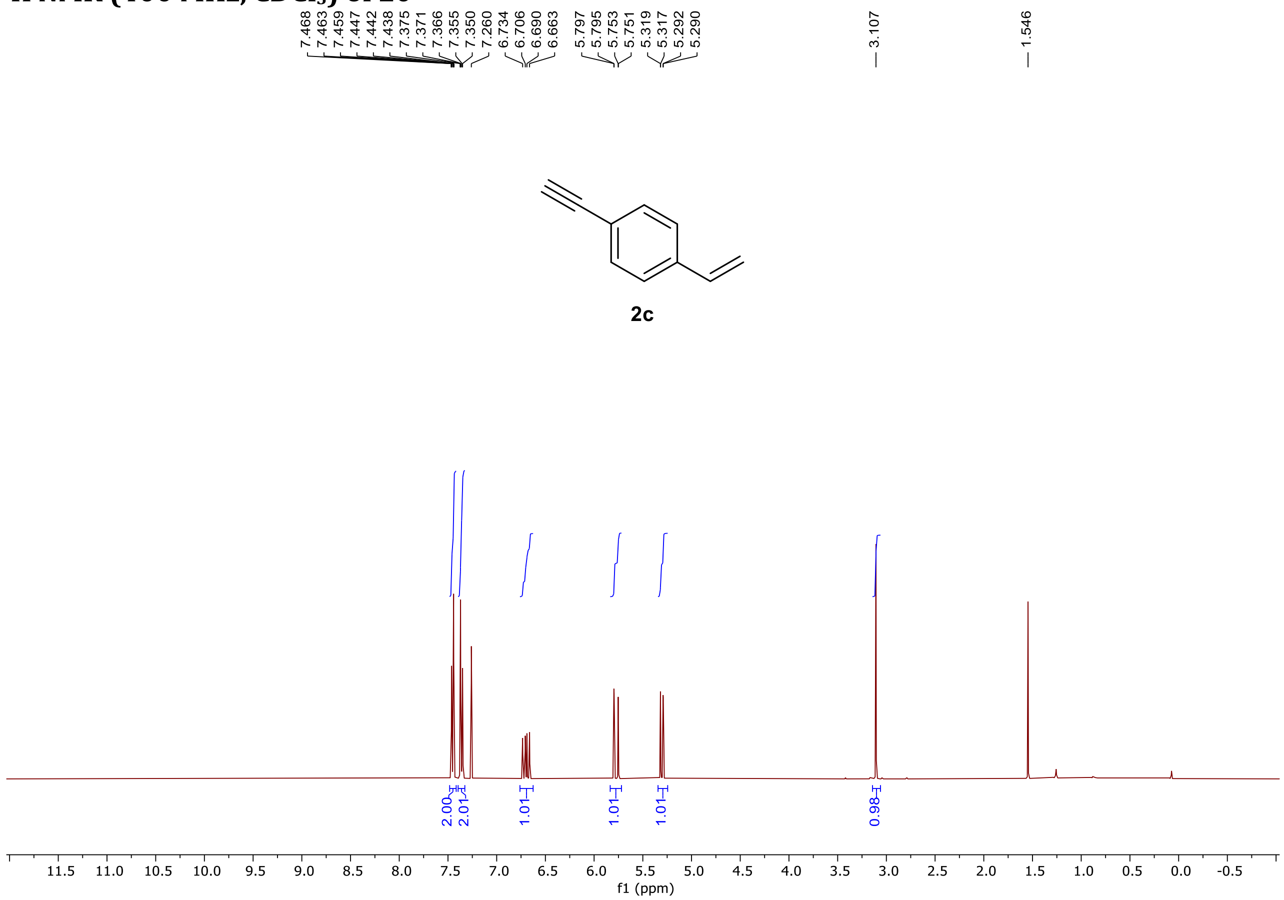

^{13}C NMR (101 MHz, $CDCl_3$) of 2c

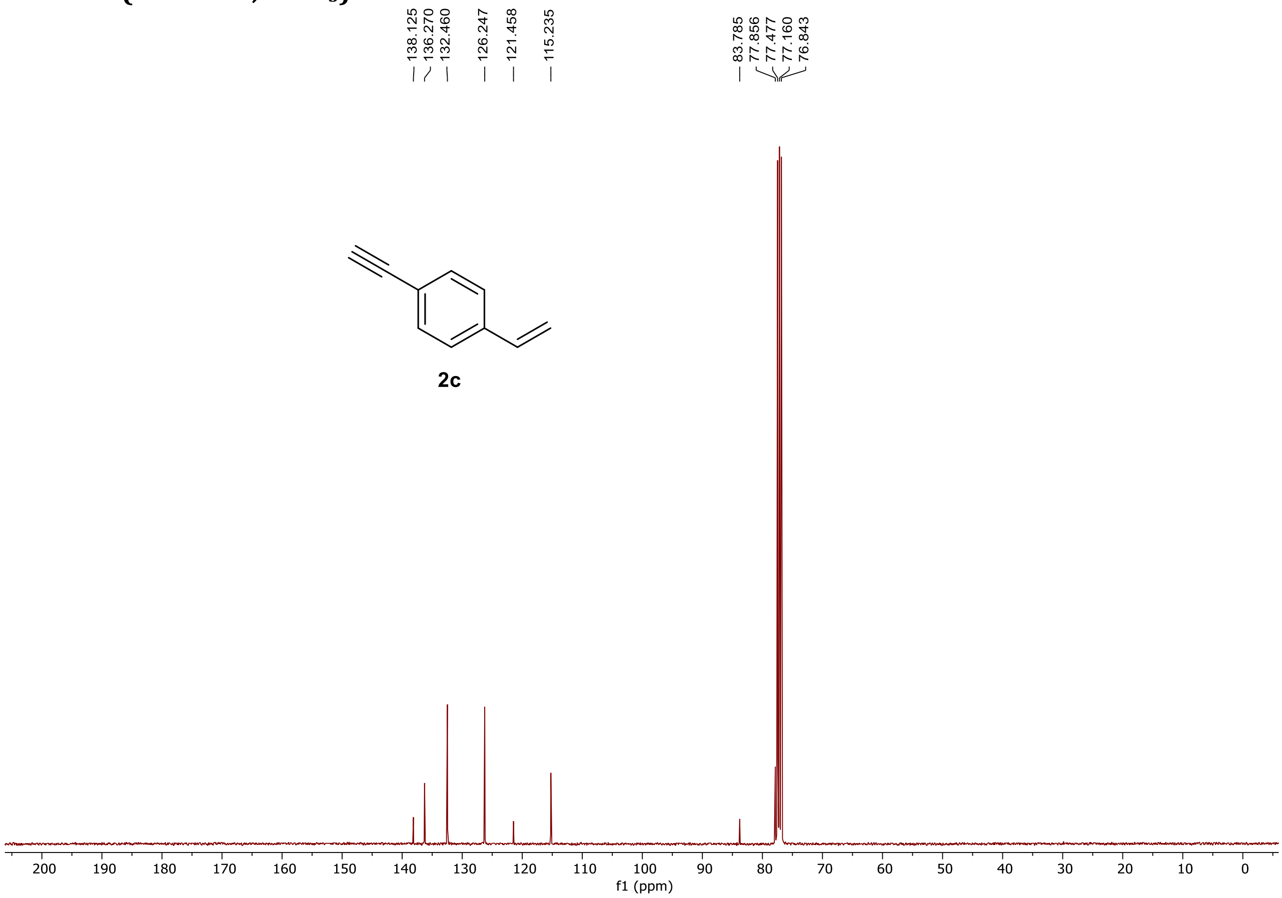

^{1}H NMR (400 MHz, $CDCl_3$) of 2o

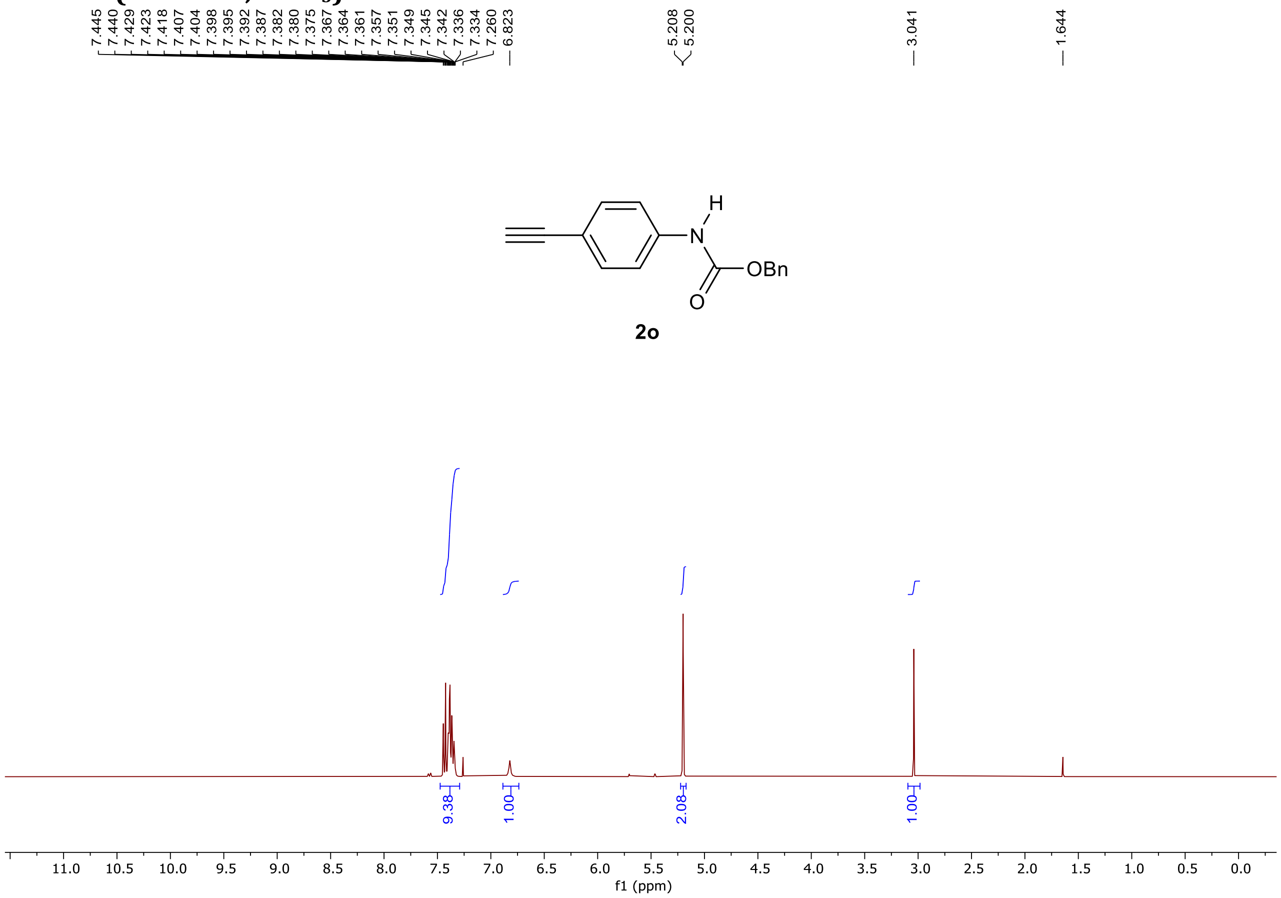

^{13}C NMR (101 MHz, $CDCl_3$) of 2o

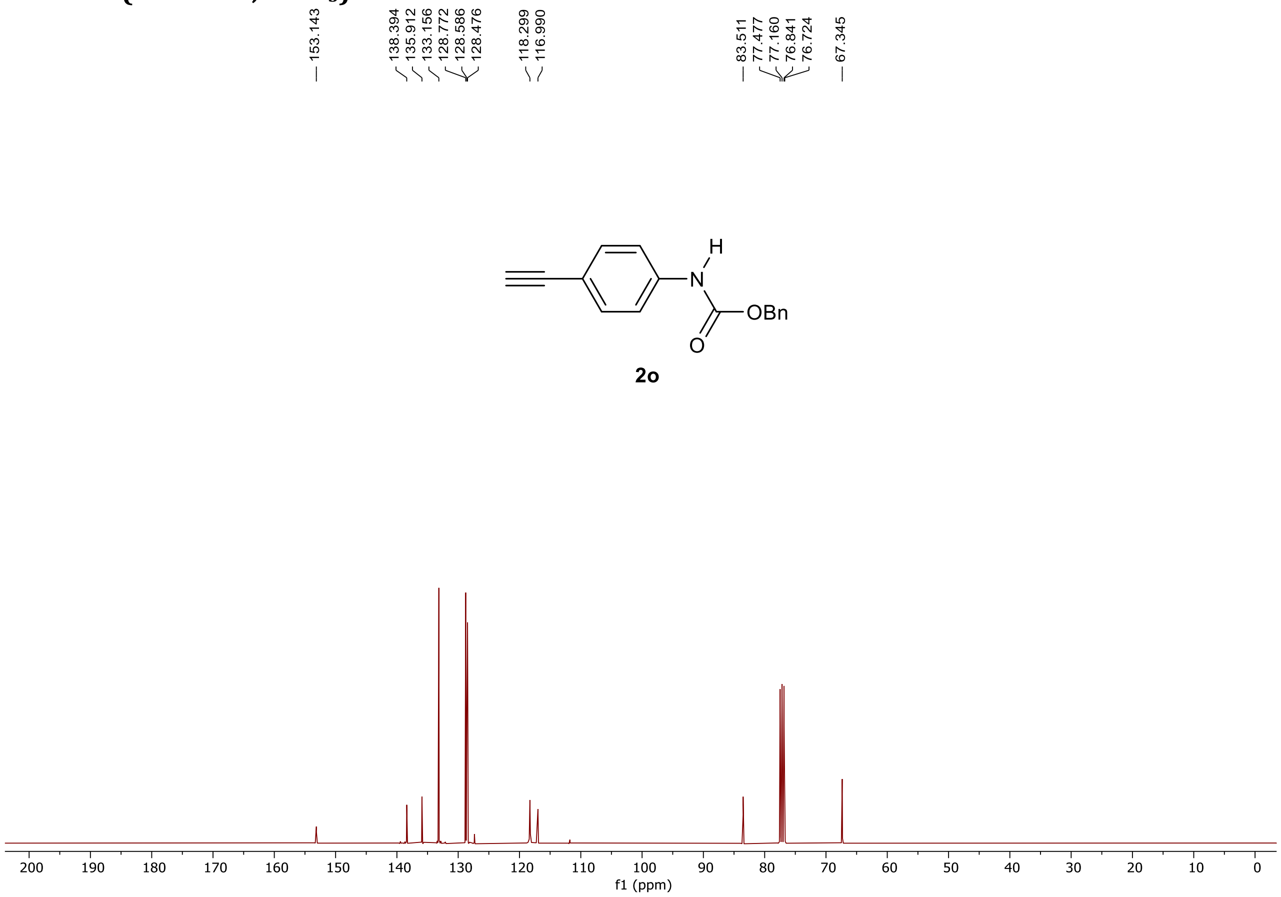

^{1}H NMR (400 MHz, $CDCl_3$) of 2r

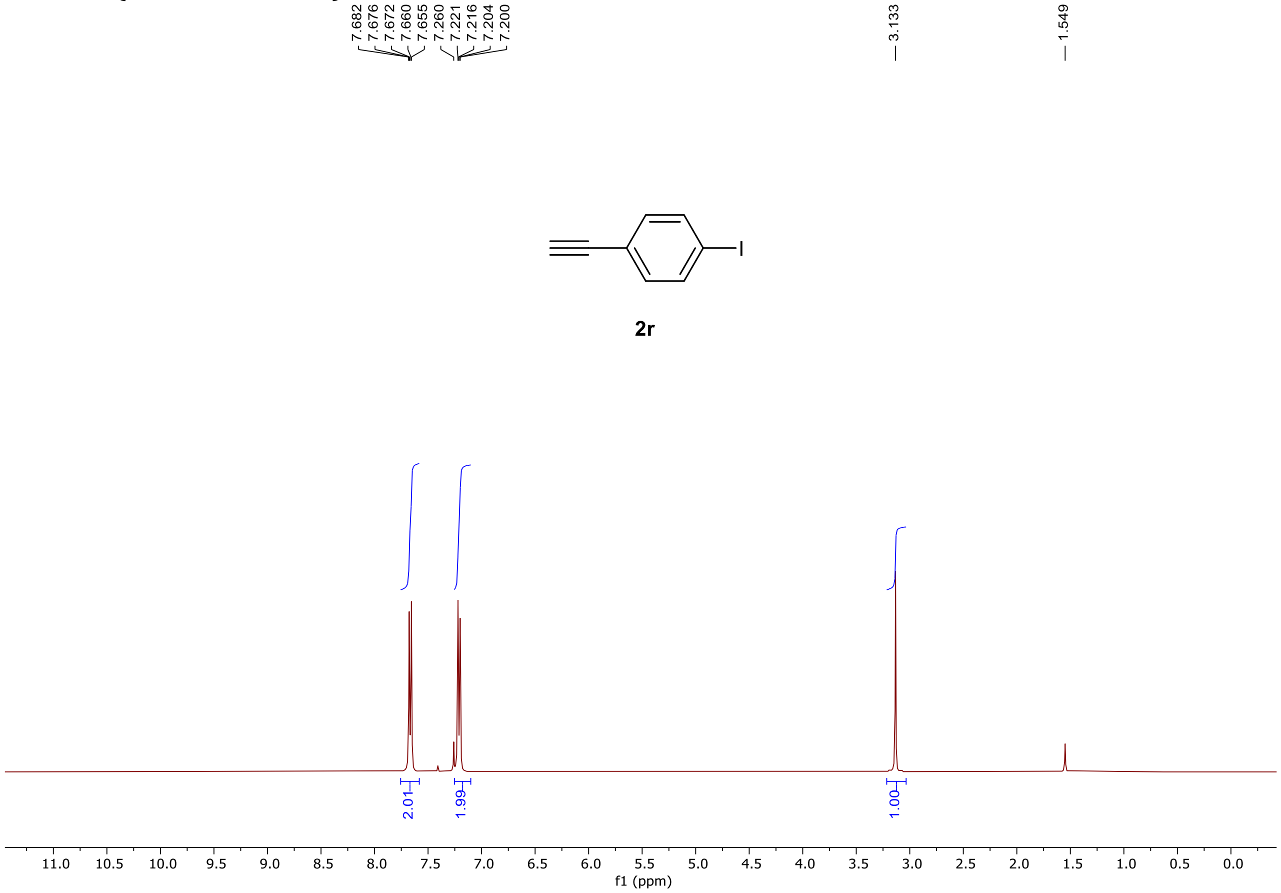

^{13}C NMR (101 MHz, $CDCl_3$) of 2r

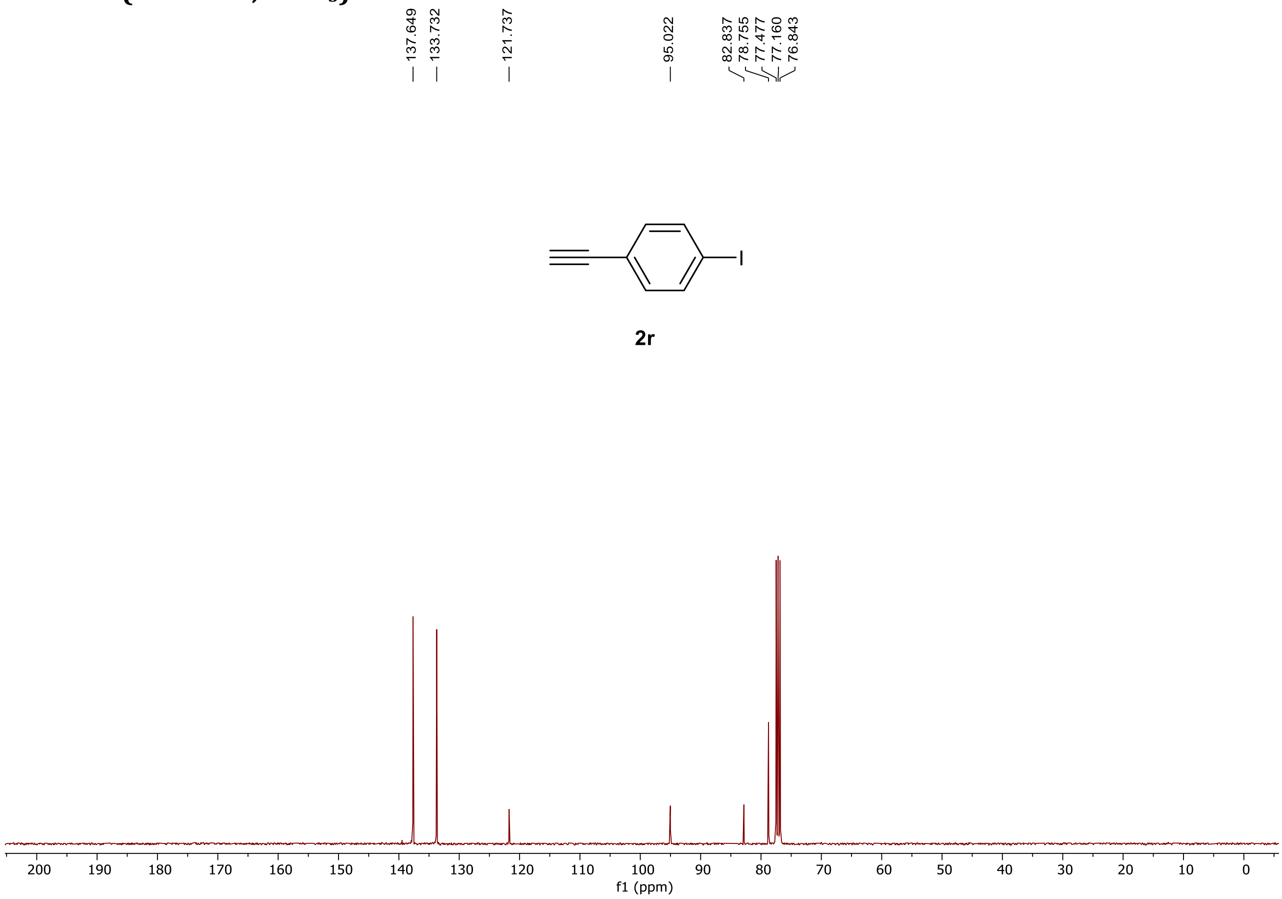

^{1}H NMR (400 MHz, $CDCl_3$) of 2s

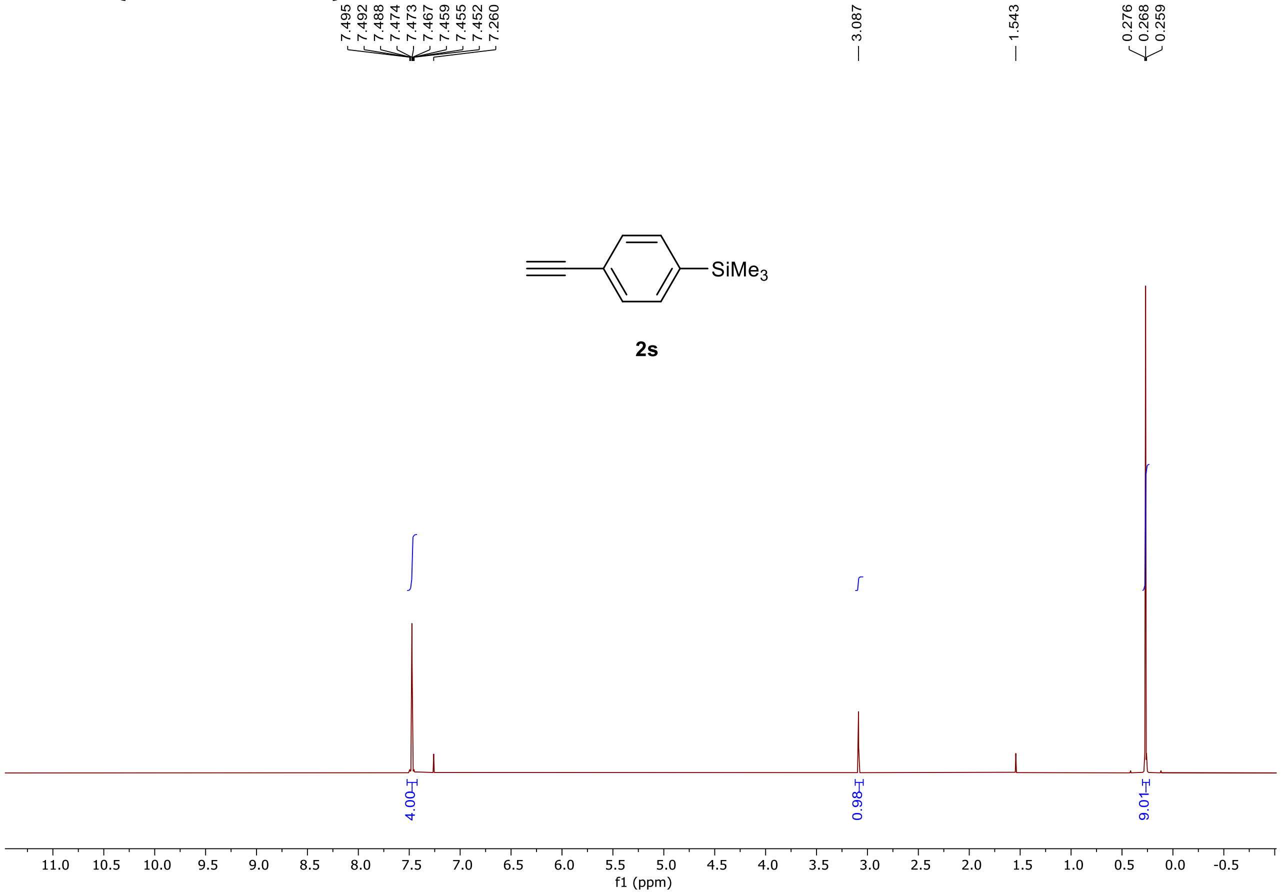

¹³C NMR (101 MHz, $CDCl_3$) of 2s

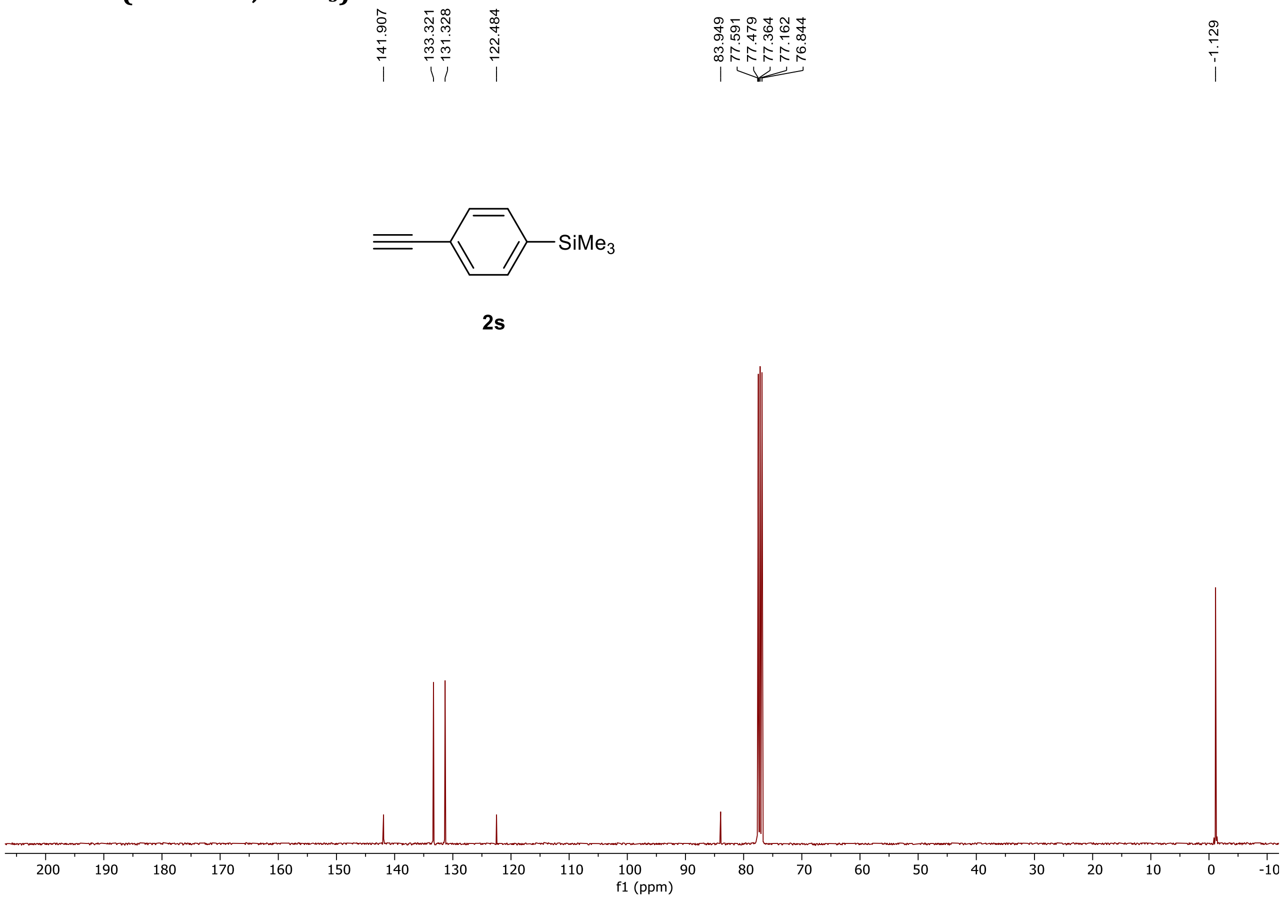

¹H NMR (400 MHz, $CDCl_3$) of 2t

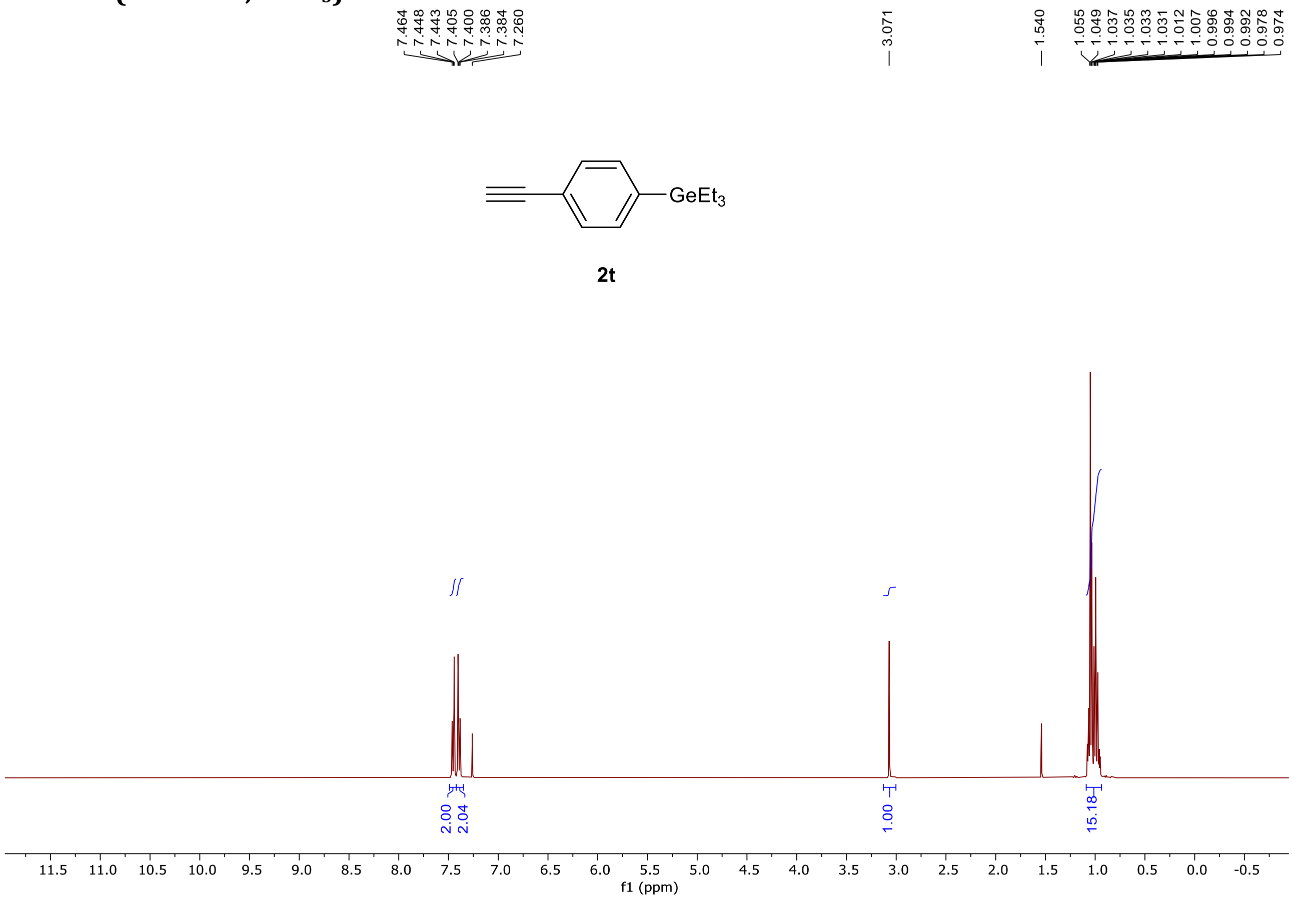

^{13}C NMR (101 MHz, $CDCl_3$) of 2t

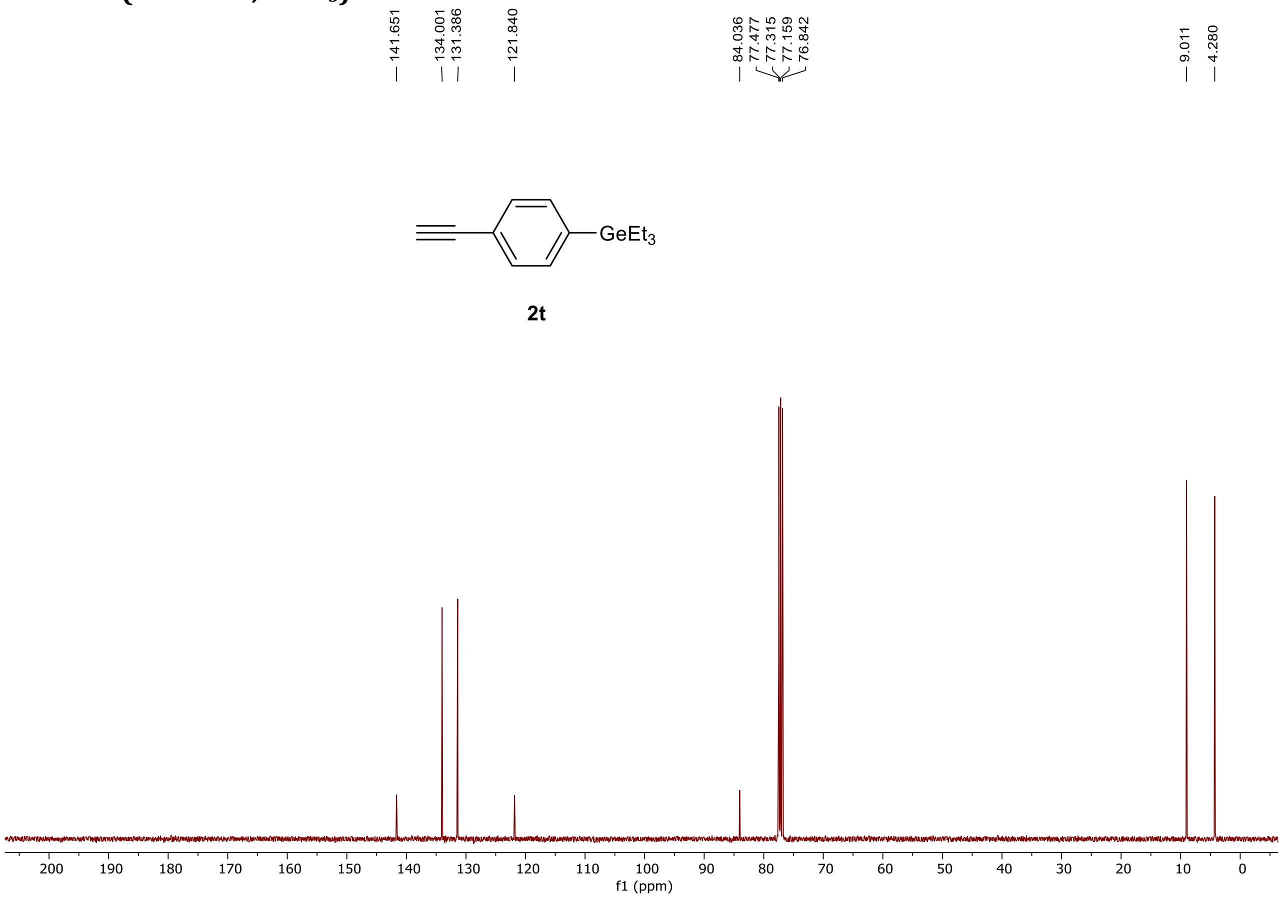

^{1}H NMR (400 MHz, $CDCl_3$) of 2u

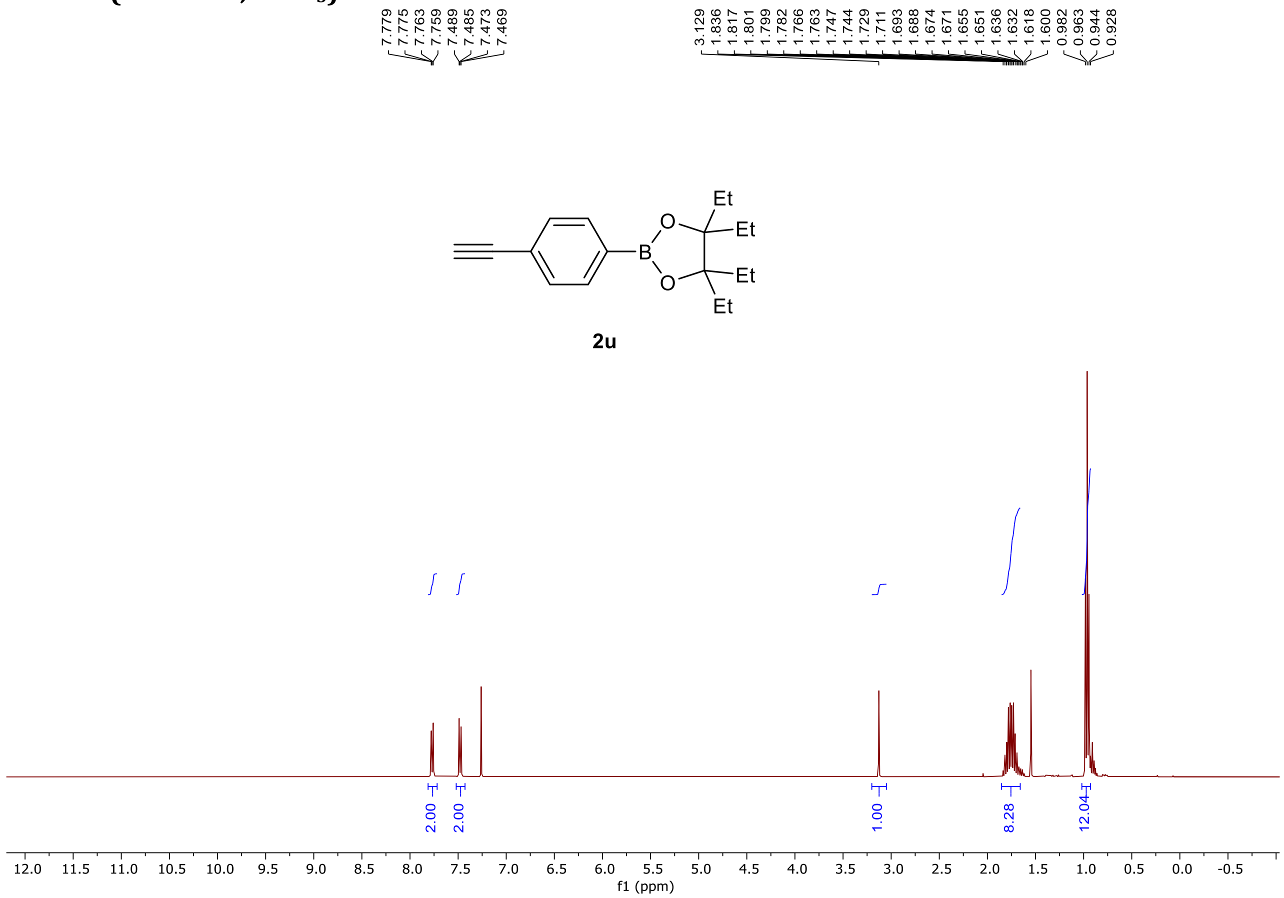

^{13}C NMR (101 MHz, $CDCl_3$) of 2u

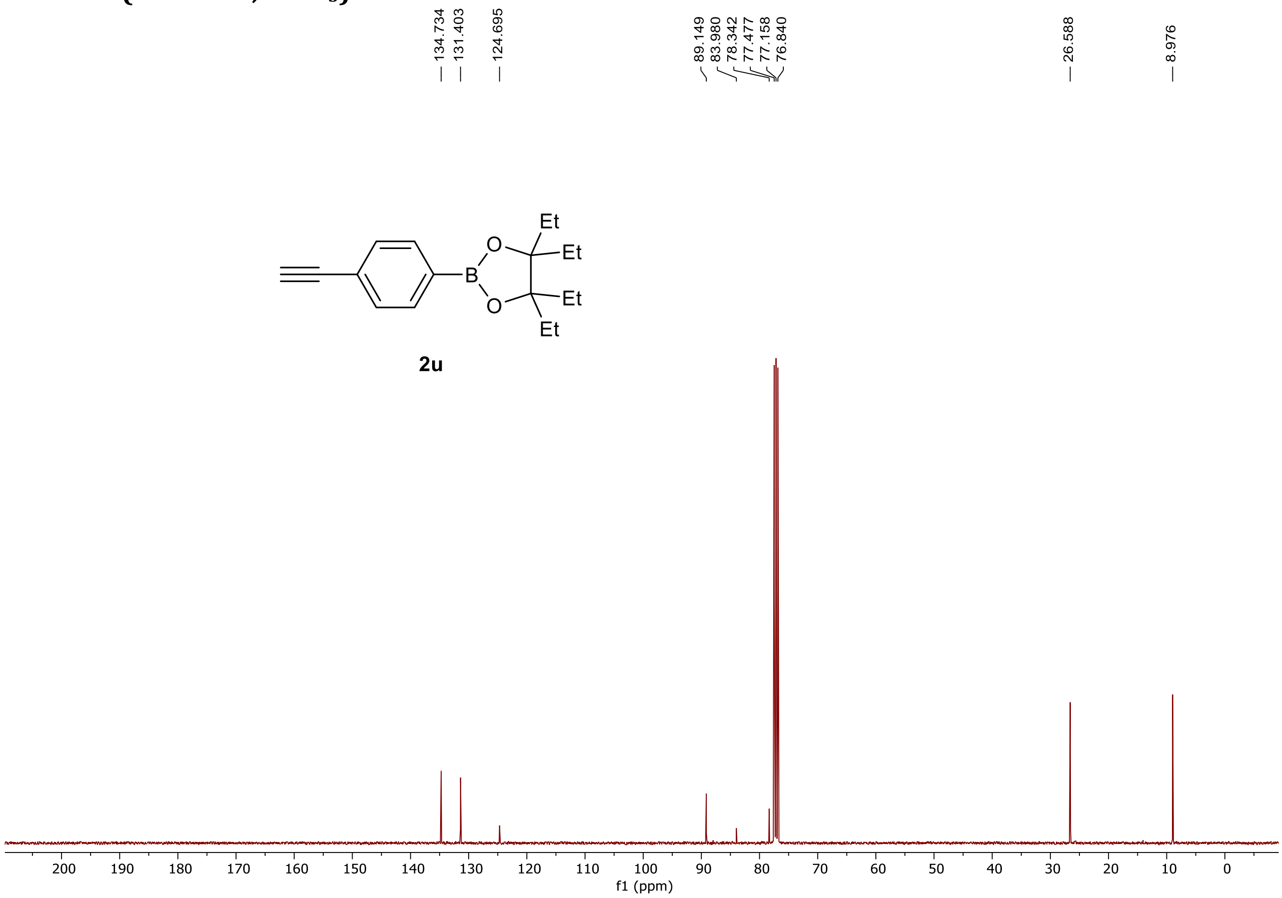

^{11}B NMR (128 MHz, $CDCl_3$) of 2u

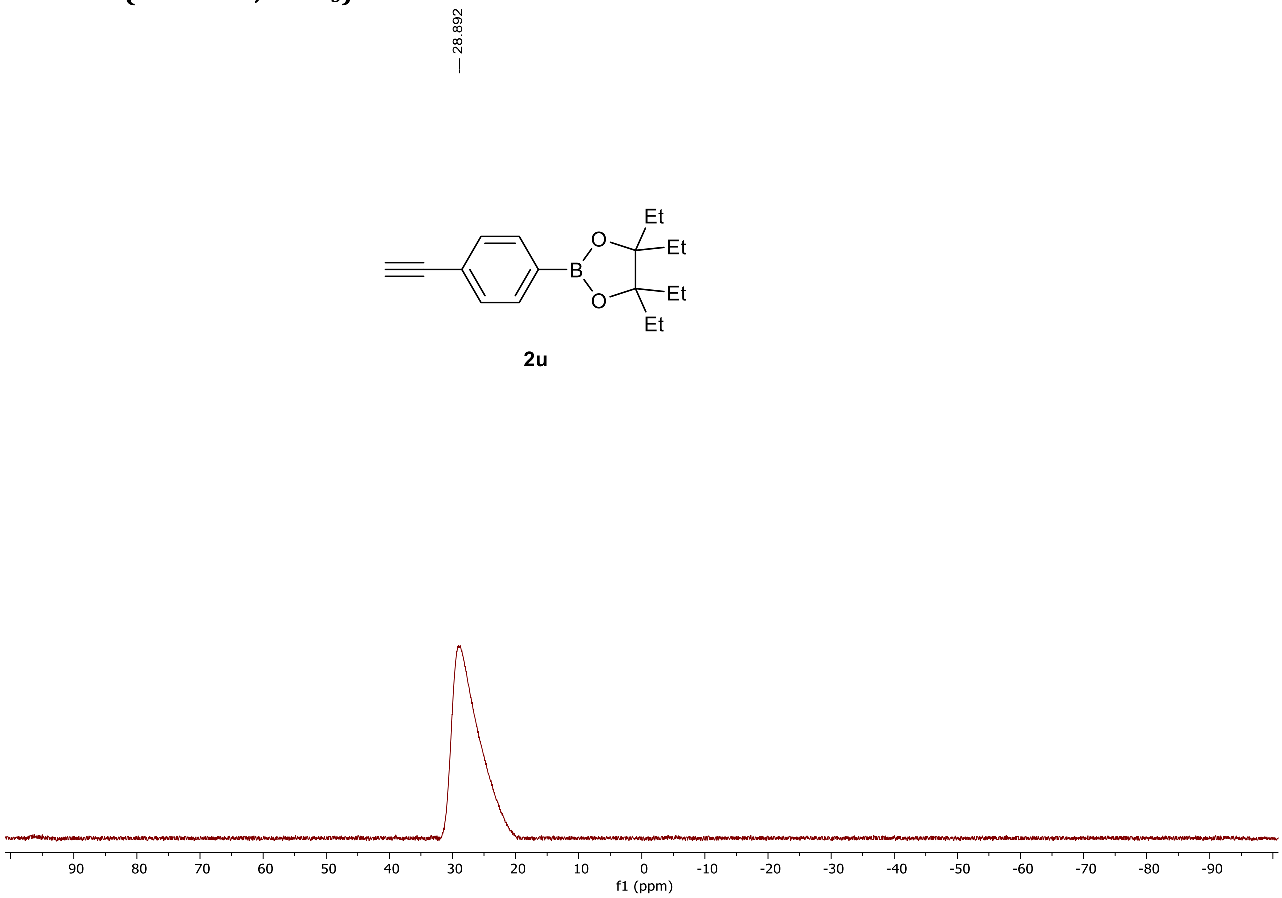

^{1}H NMR (400 MHz, $CDCl_3$) of 2ax

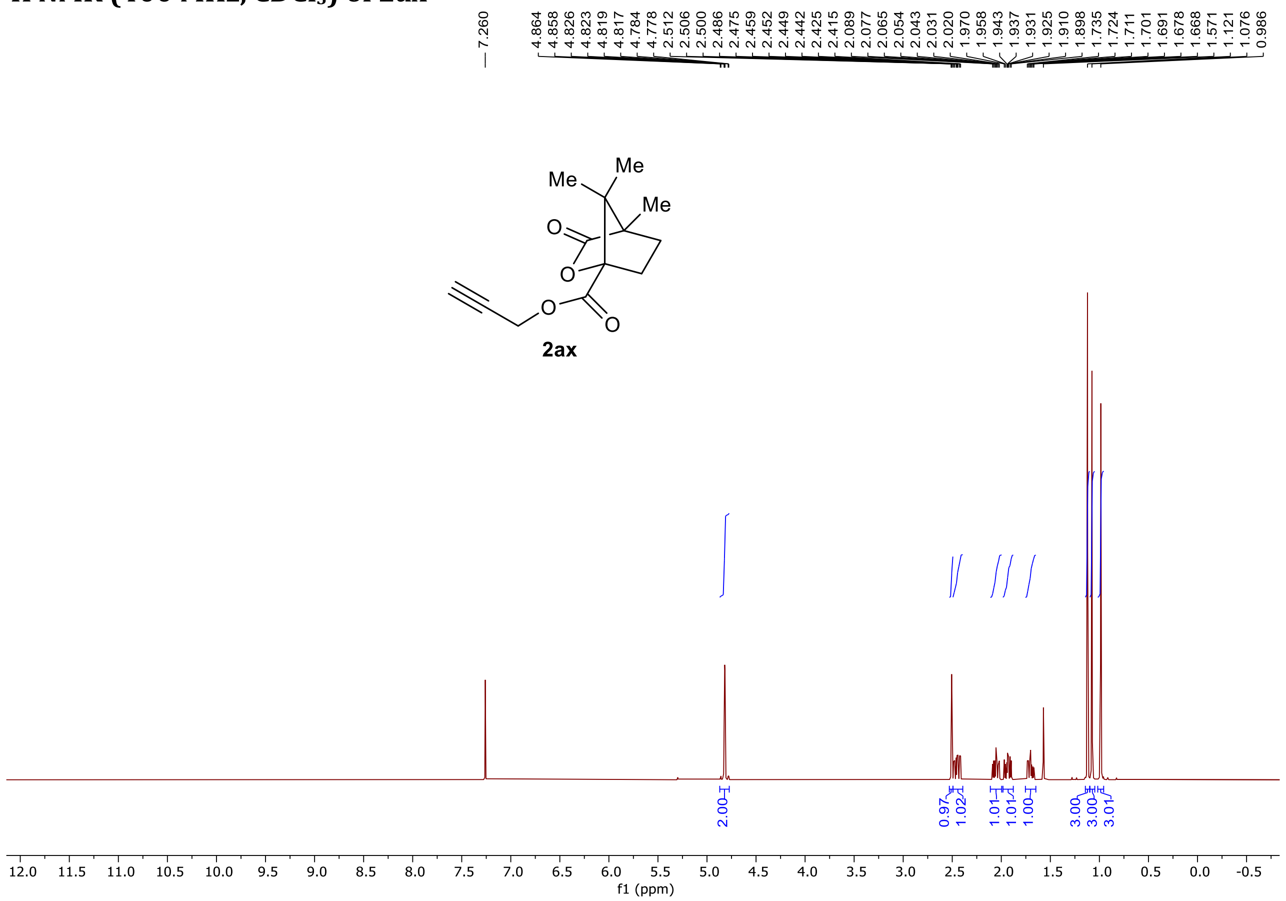

^{13}C NMR (101 MHz, $CDCl_3$) of 2ax

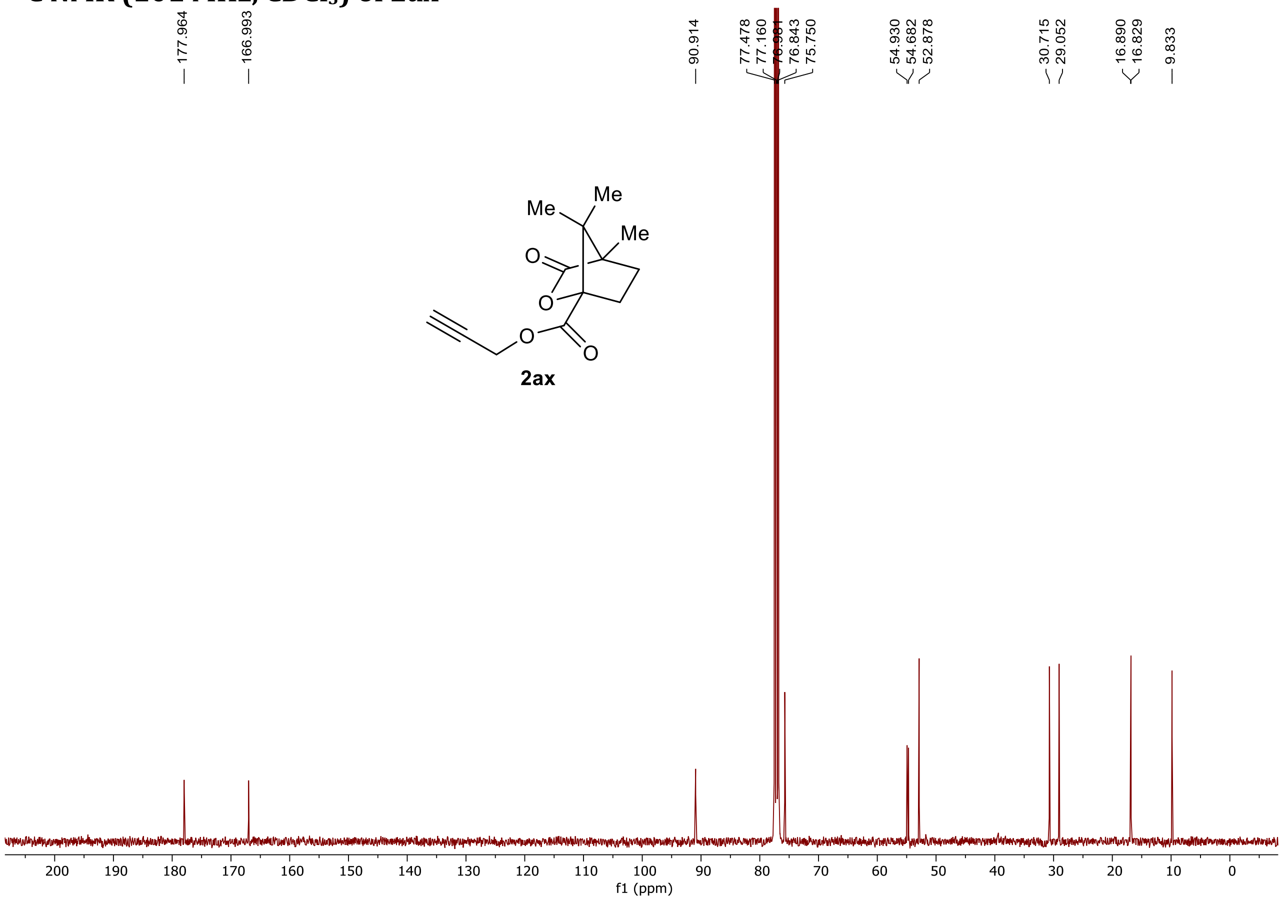

^{1}H NMR (400 MHz, $CDCl_3$) of 2ay

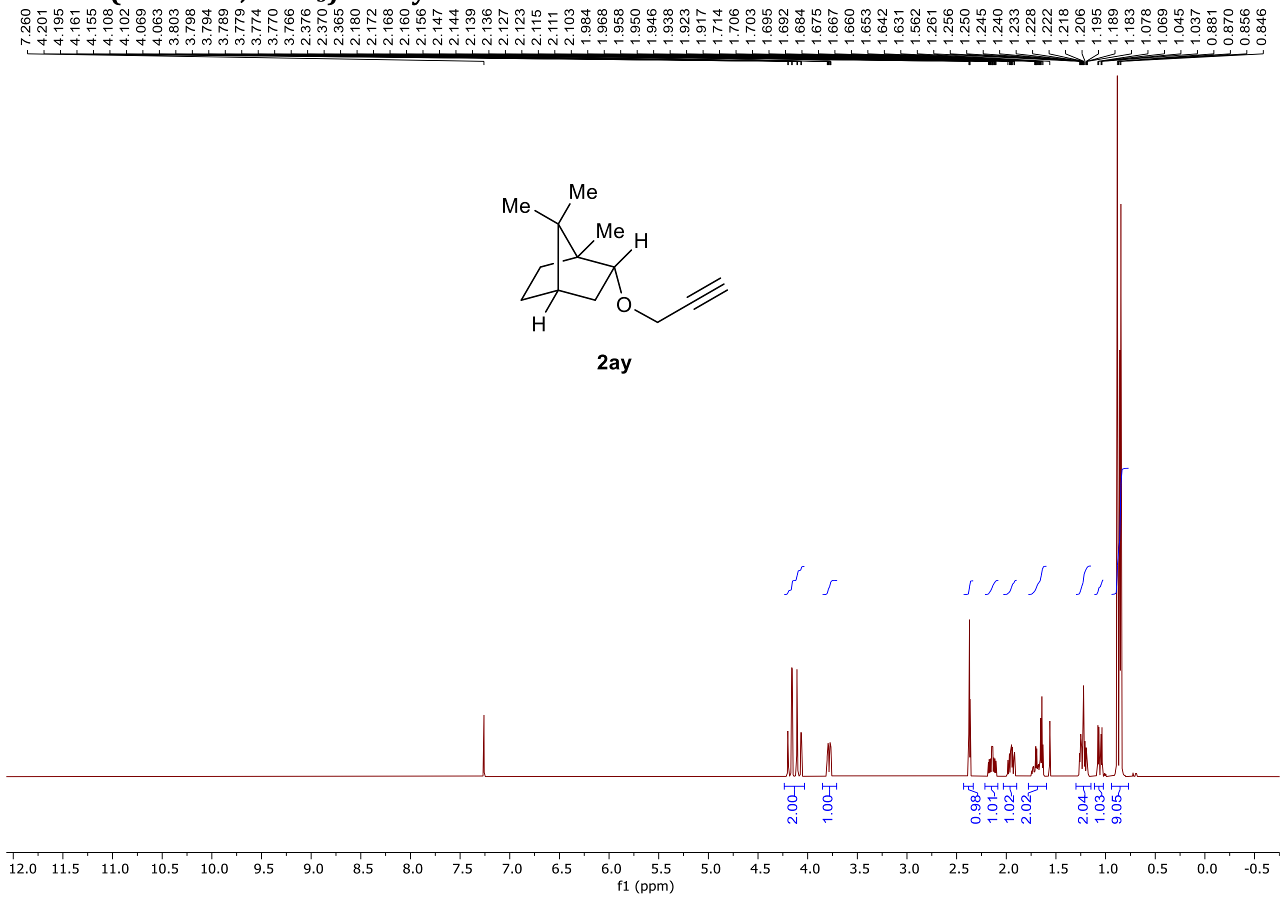

^{13}C NMR (101 MHz, $CDCl_3$) of 2ay

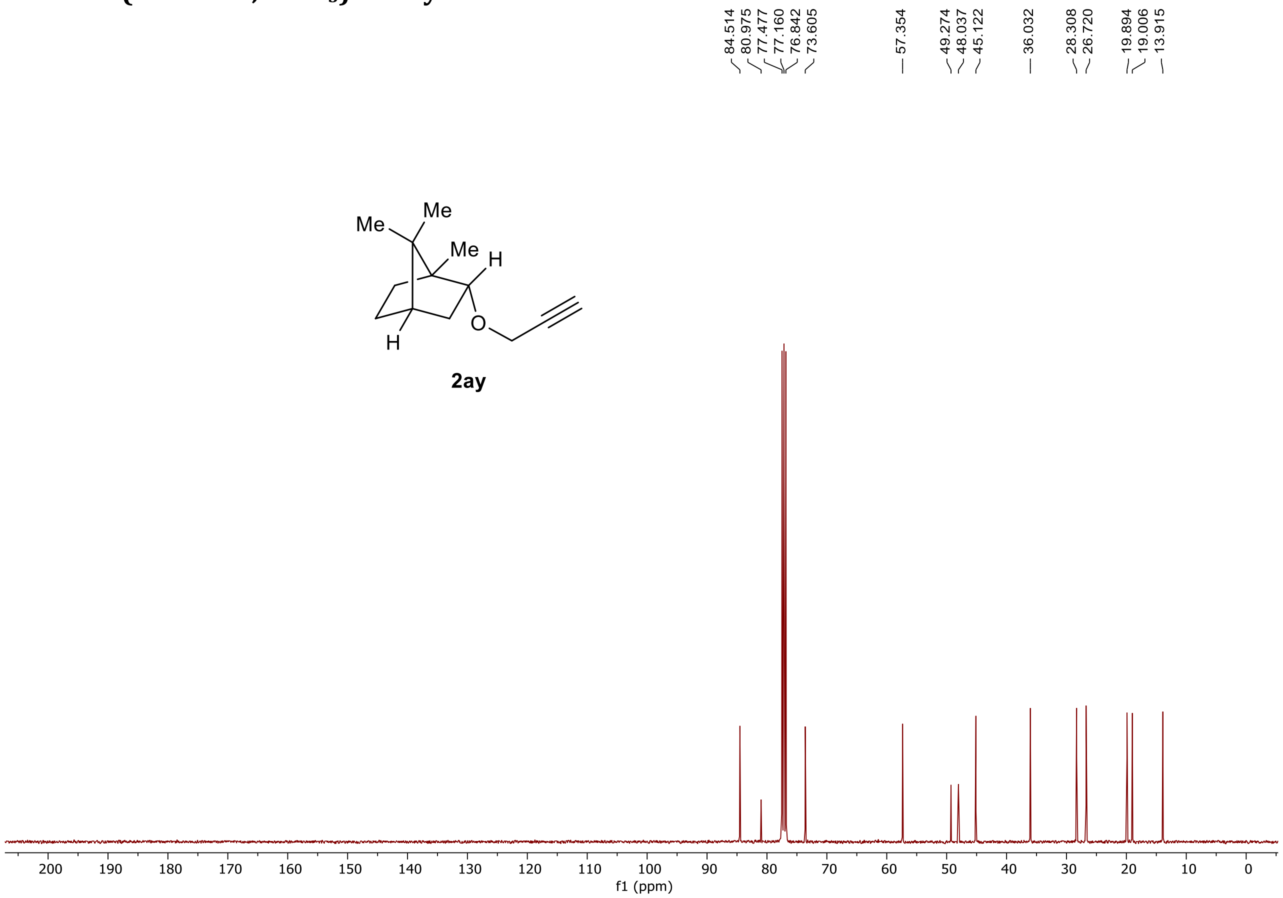

^1H NMR (400 MHz, $CDCl_3$, 60 °C) of 2az

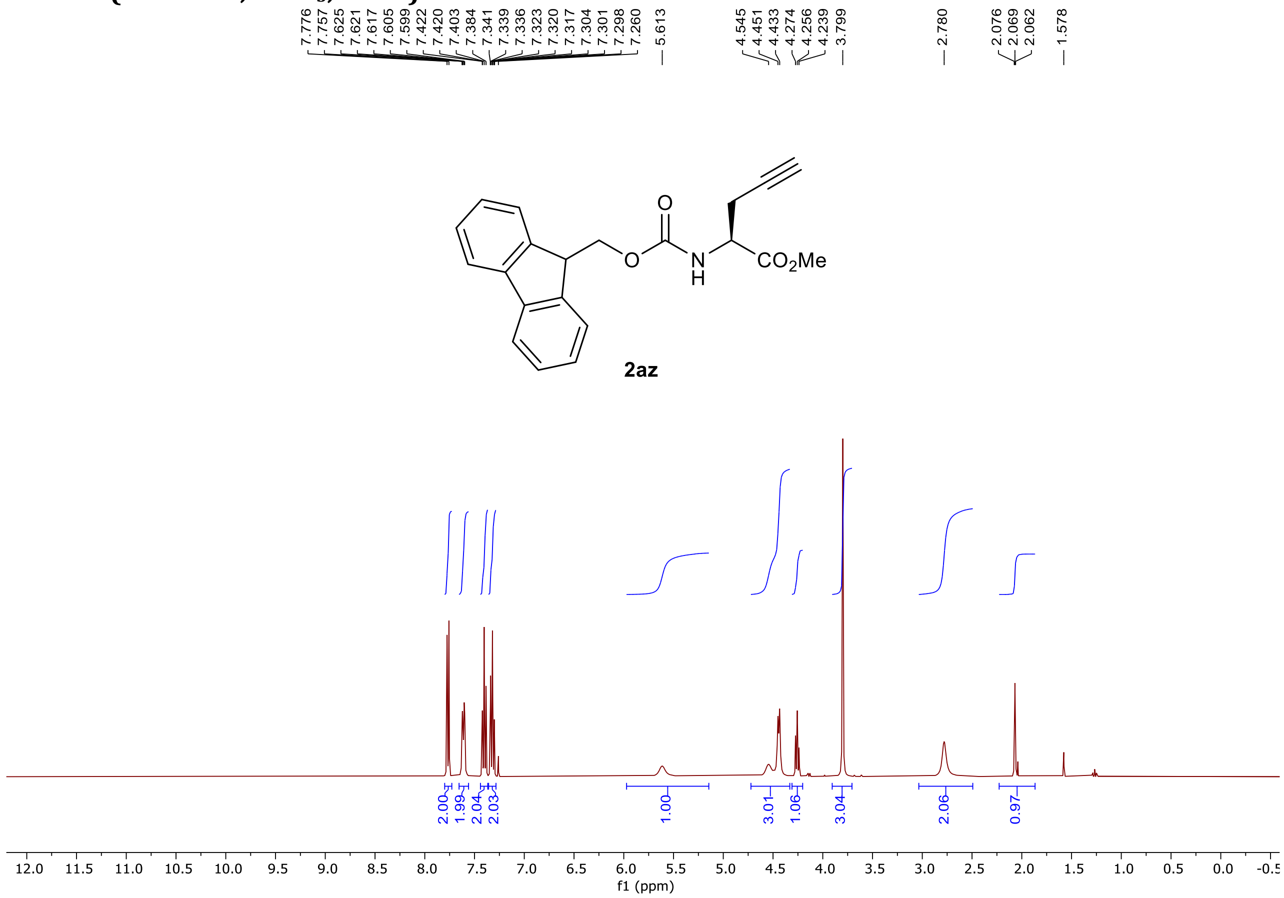

^{13}C NMR (101 MHz, $CDCl_3$, 60 °C) of 2az

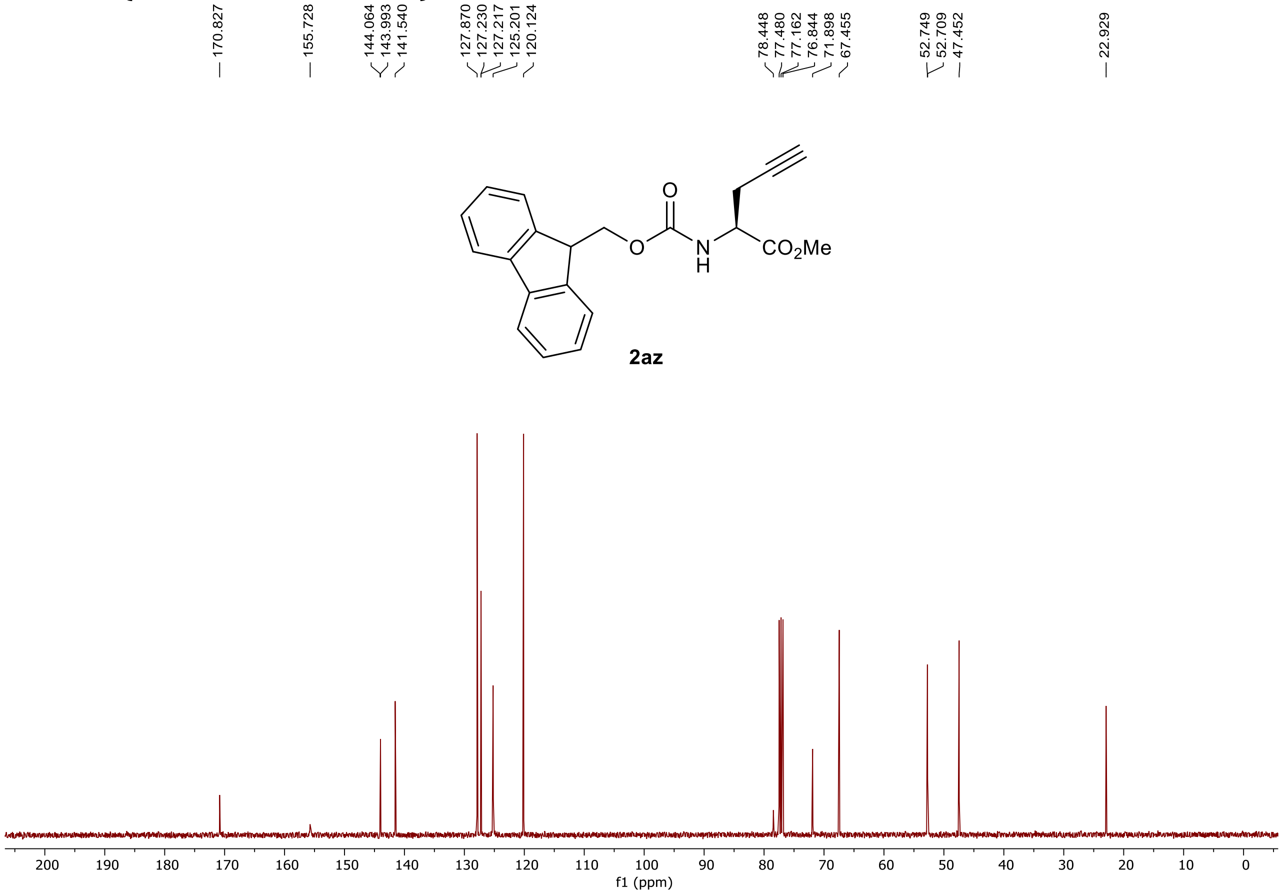

^{1}H NMR (400 MHz, $CDCl_3$, 60 °C) of 2ba

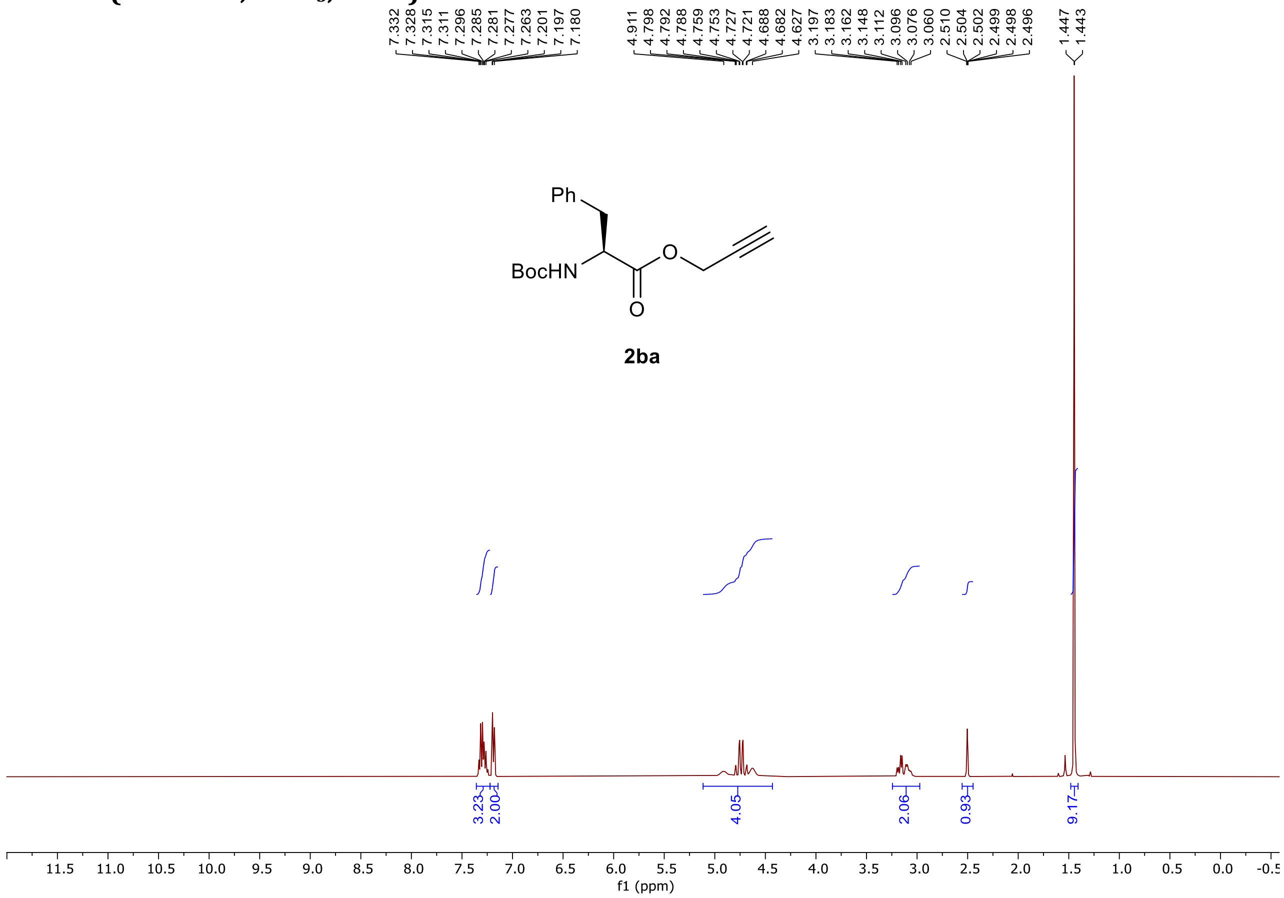

^{13}C NMR (101 MHz, $CDCl_3$, 60 °C) of 2ba

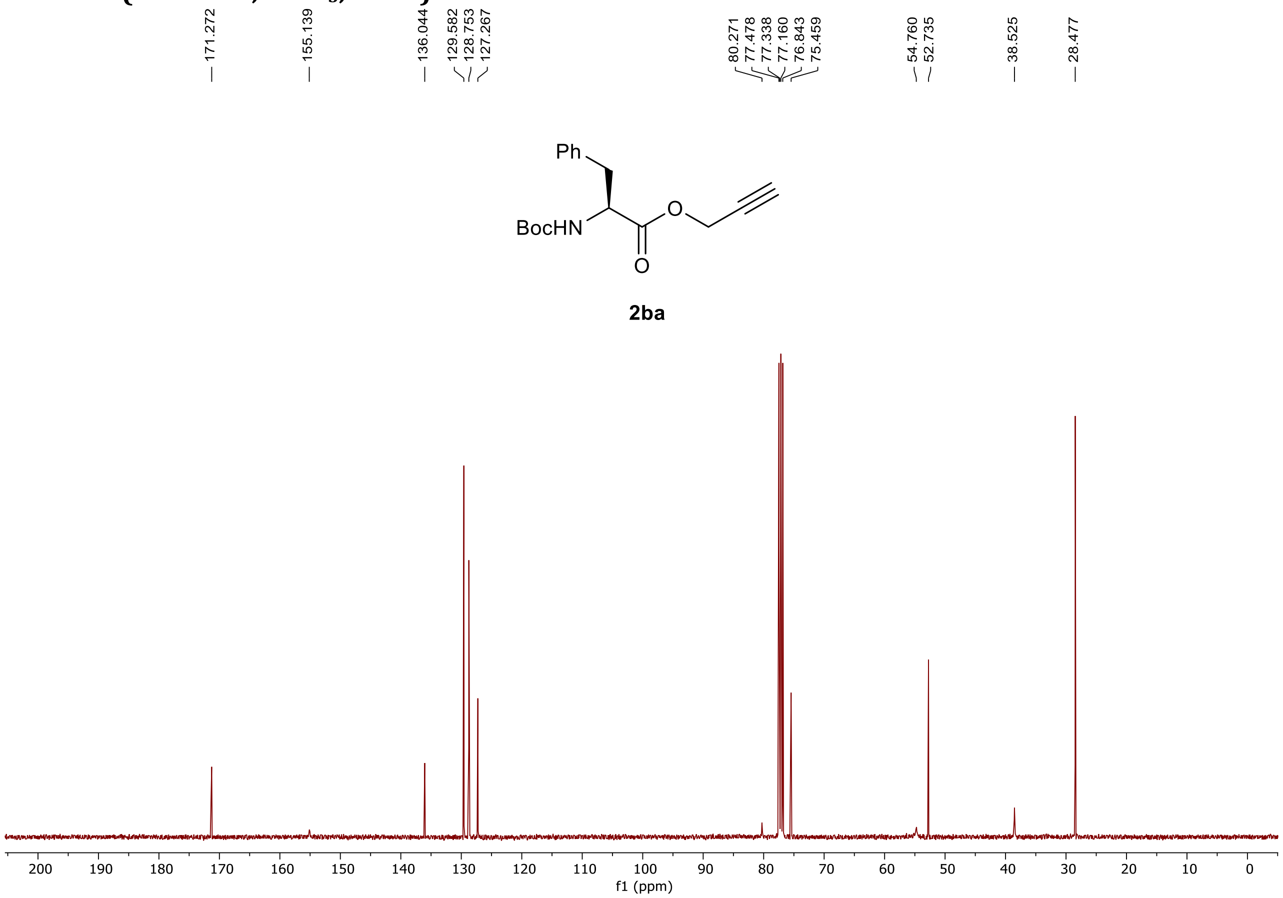

^{1}H NMR (400 MHz, $CDCl_3$) of 2bb

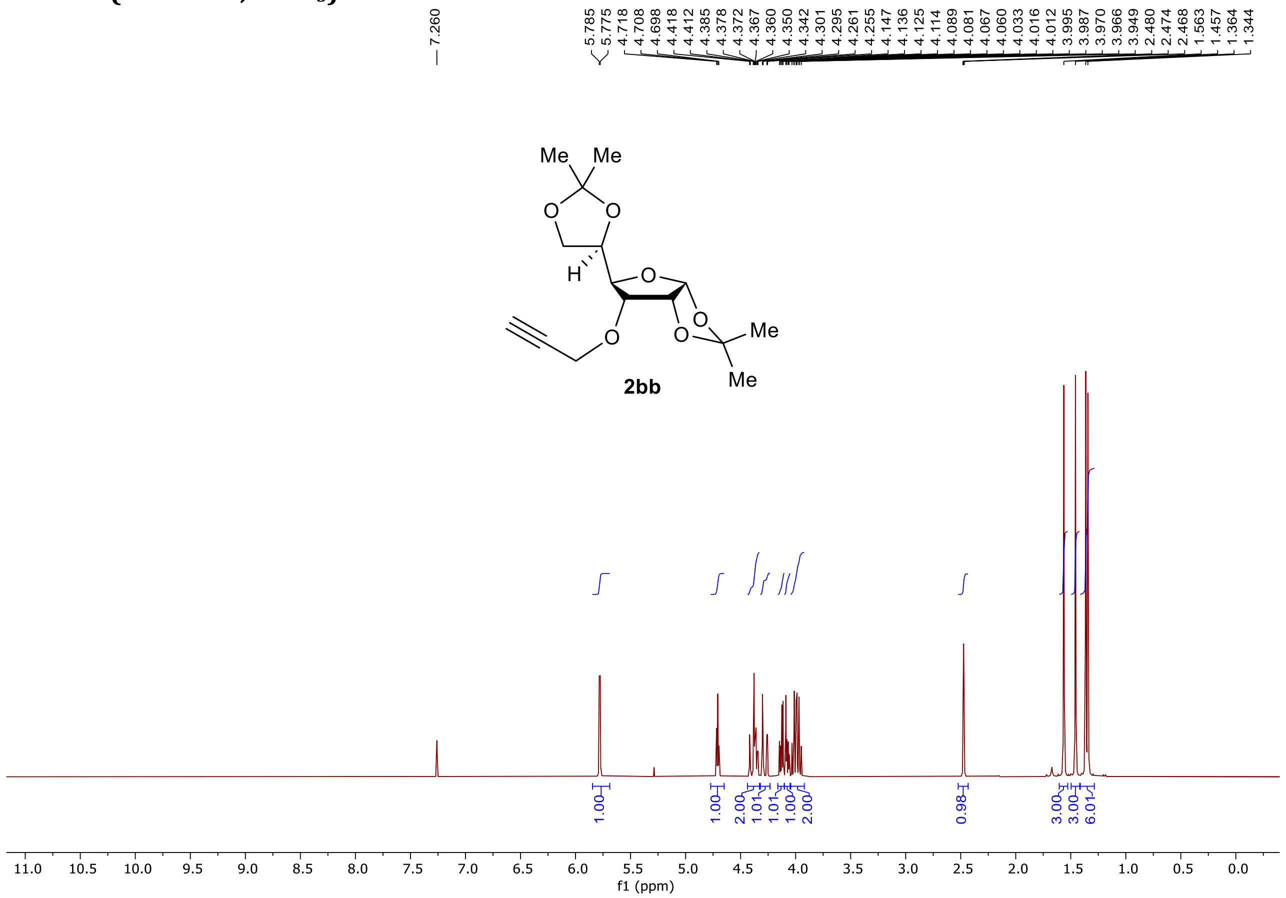

^{13}C NMR (101 MHz, $CDCl_3$) of 2bb

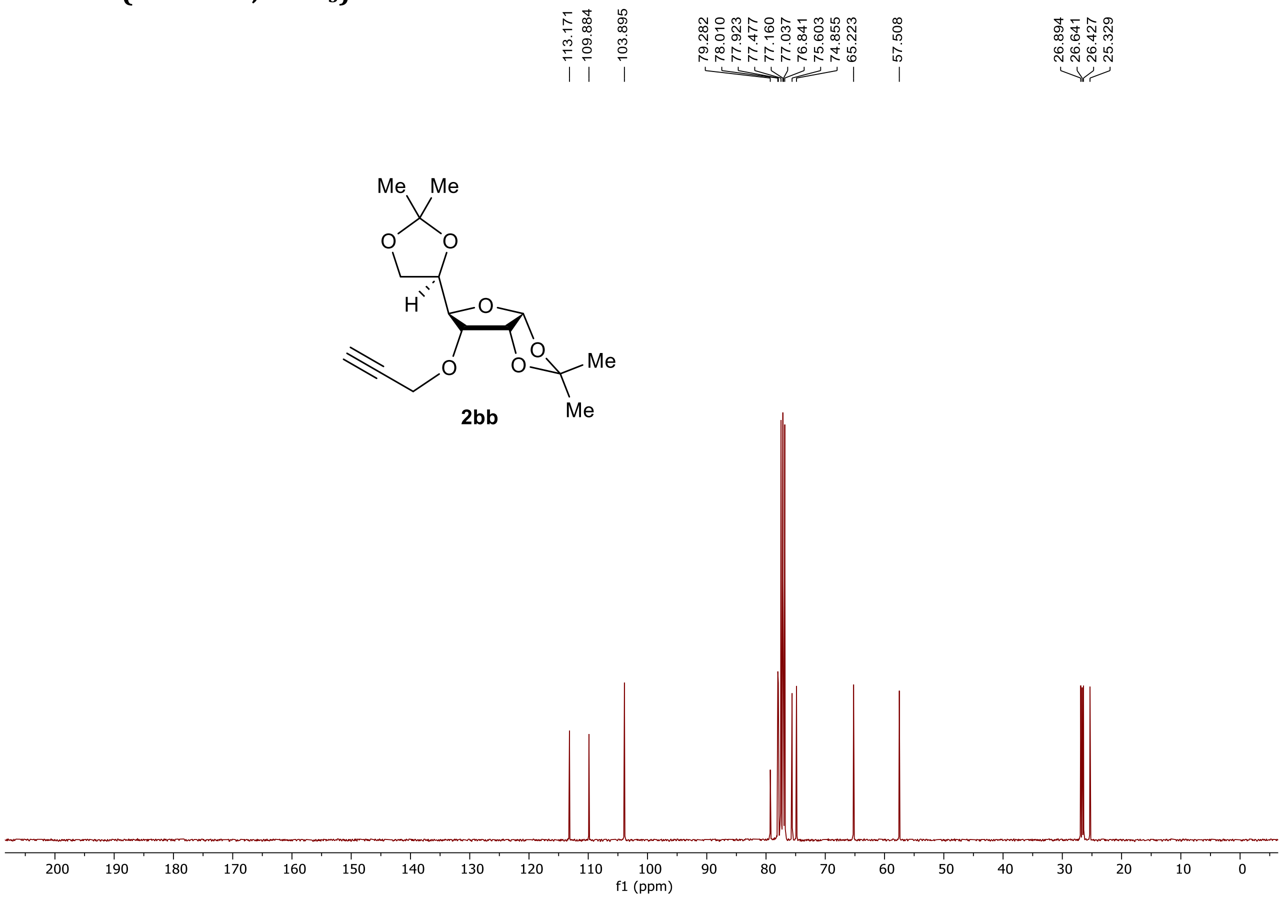

1H NMR (400 MHz, CDCl3) of 2bc

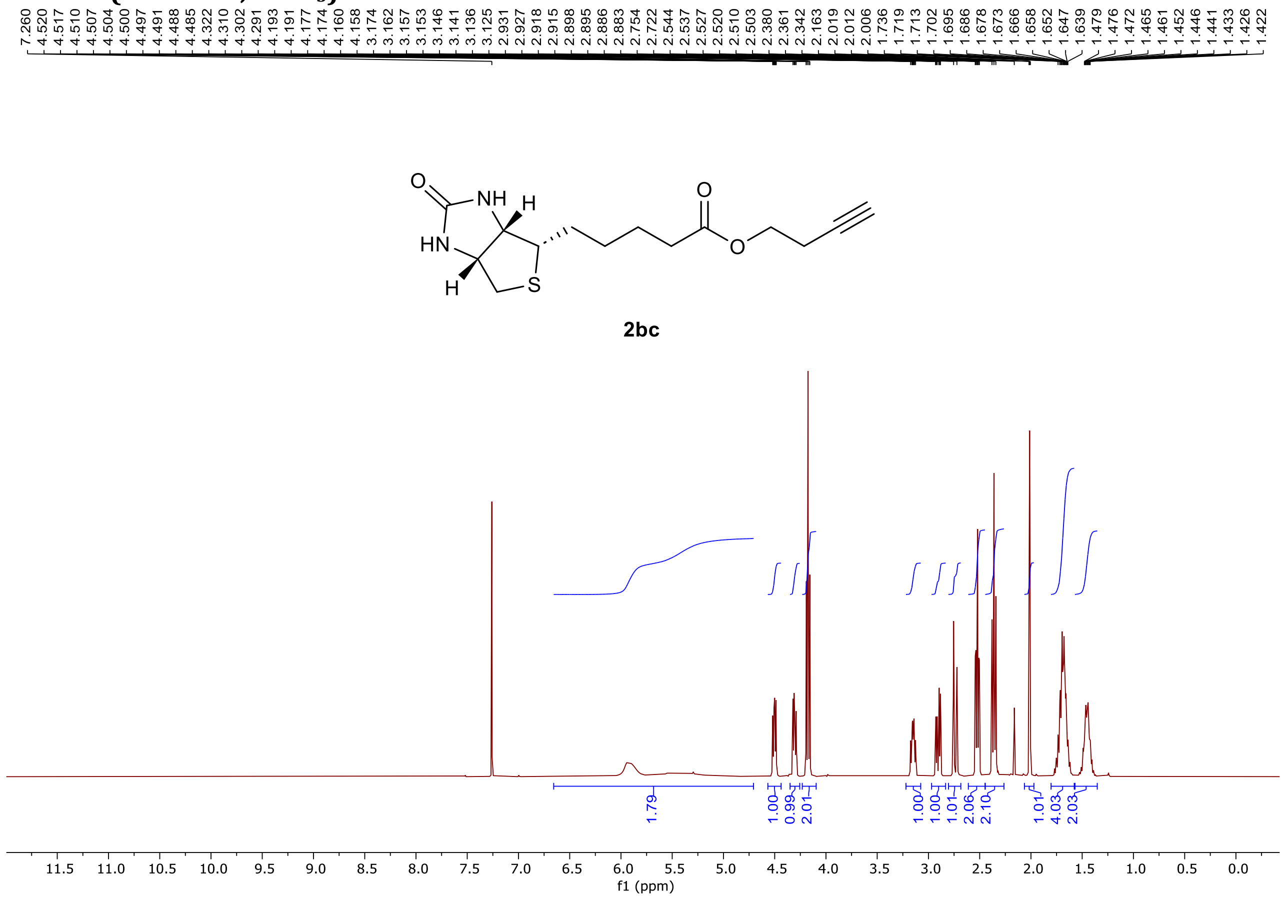

13C NMR (101 MHz, CDCl3) of 2bc

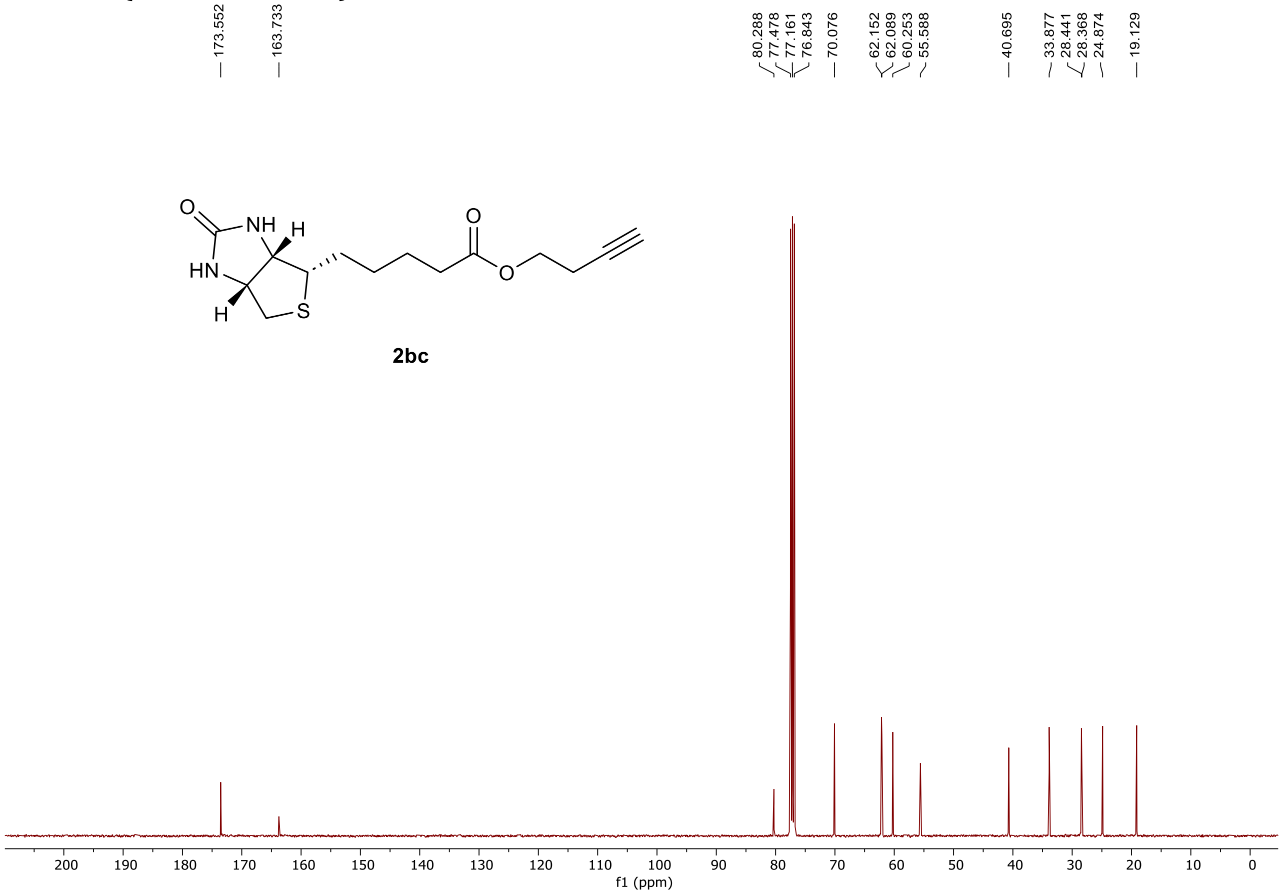

^{1}H NMR (400 MHz, $CDCl_3$) of 2bd

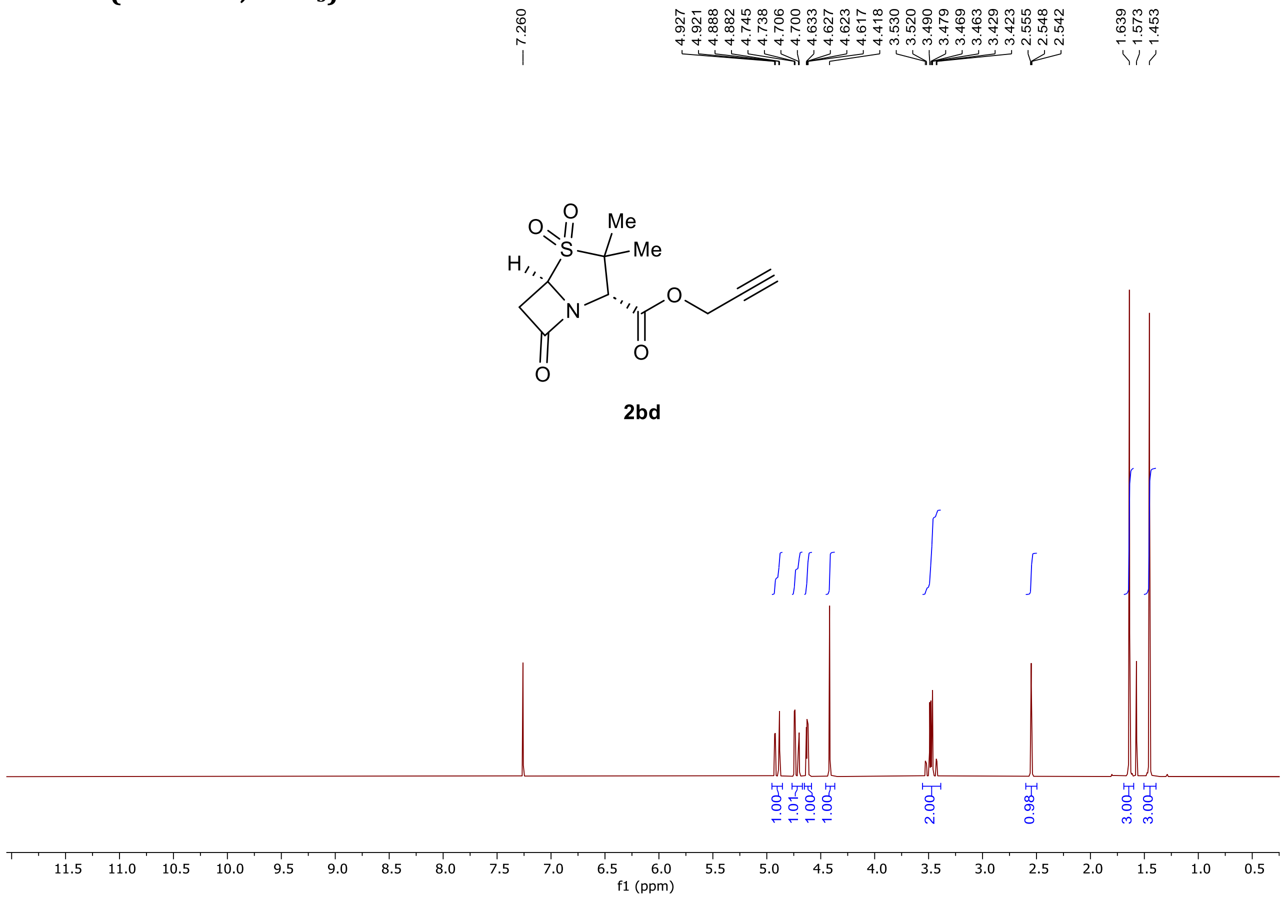

^{13}C NMR (101 MHz, $CDCl_3$) of 2bd

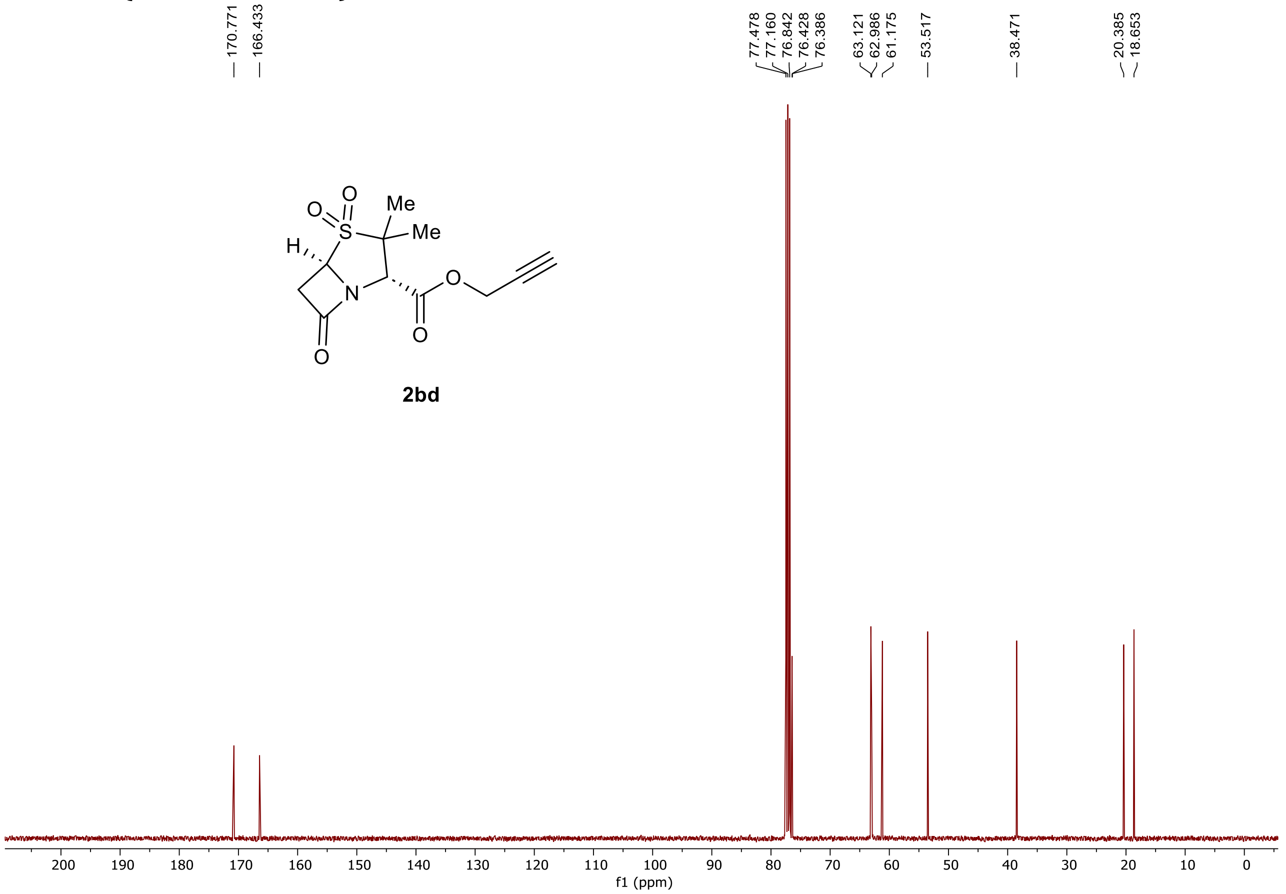

1H NMR (400 MHz, CDCl3) of 2be

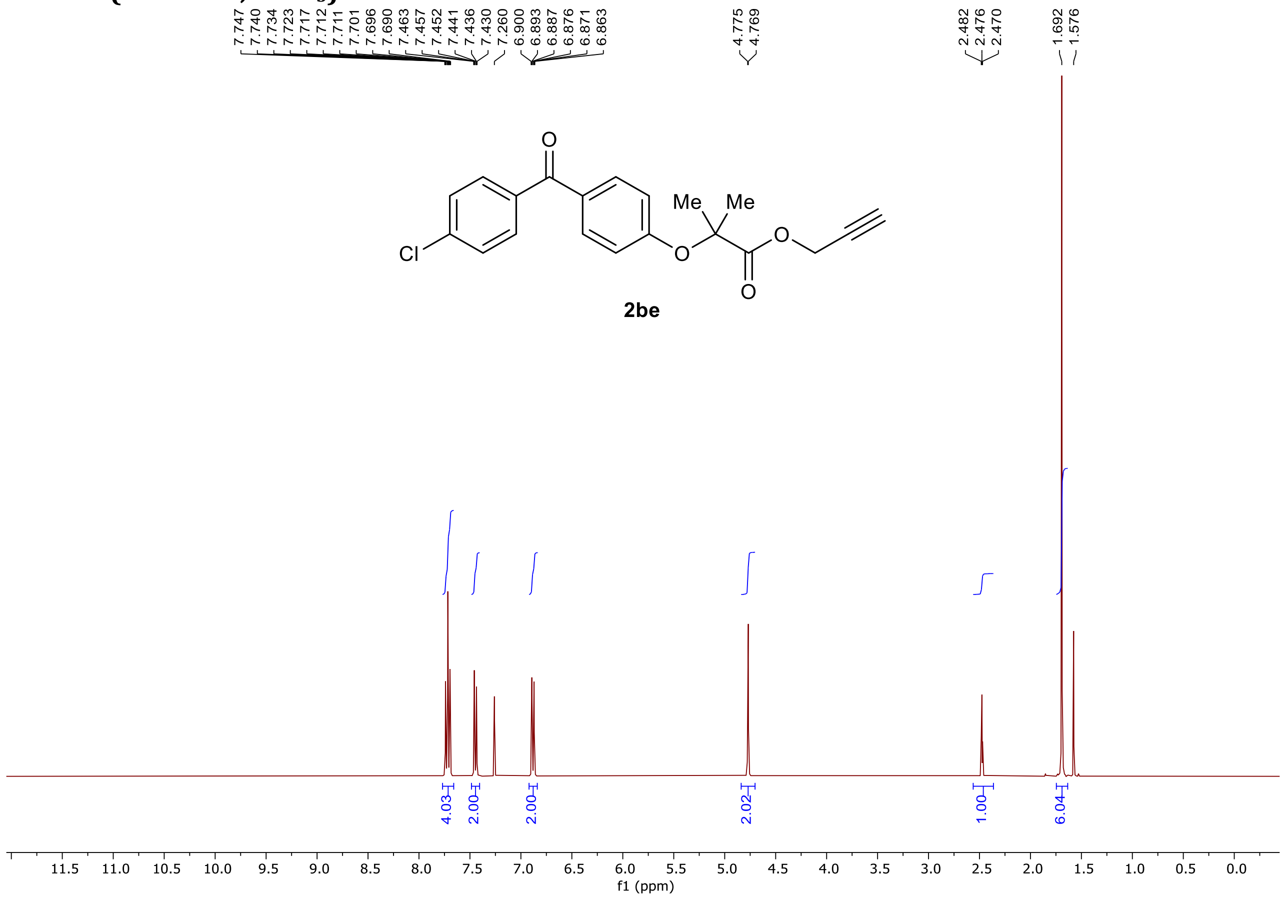

13C NMR (101 MHz, CDCl3) of 2be

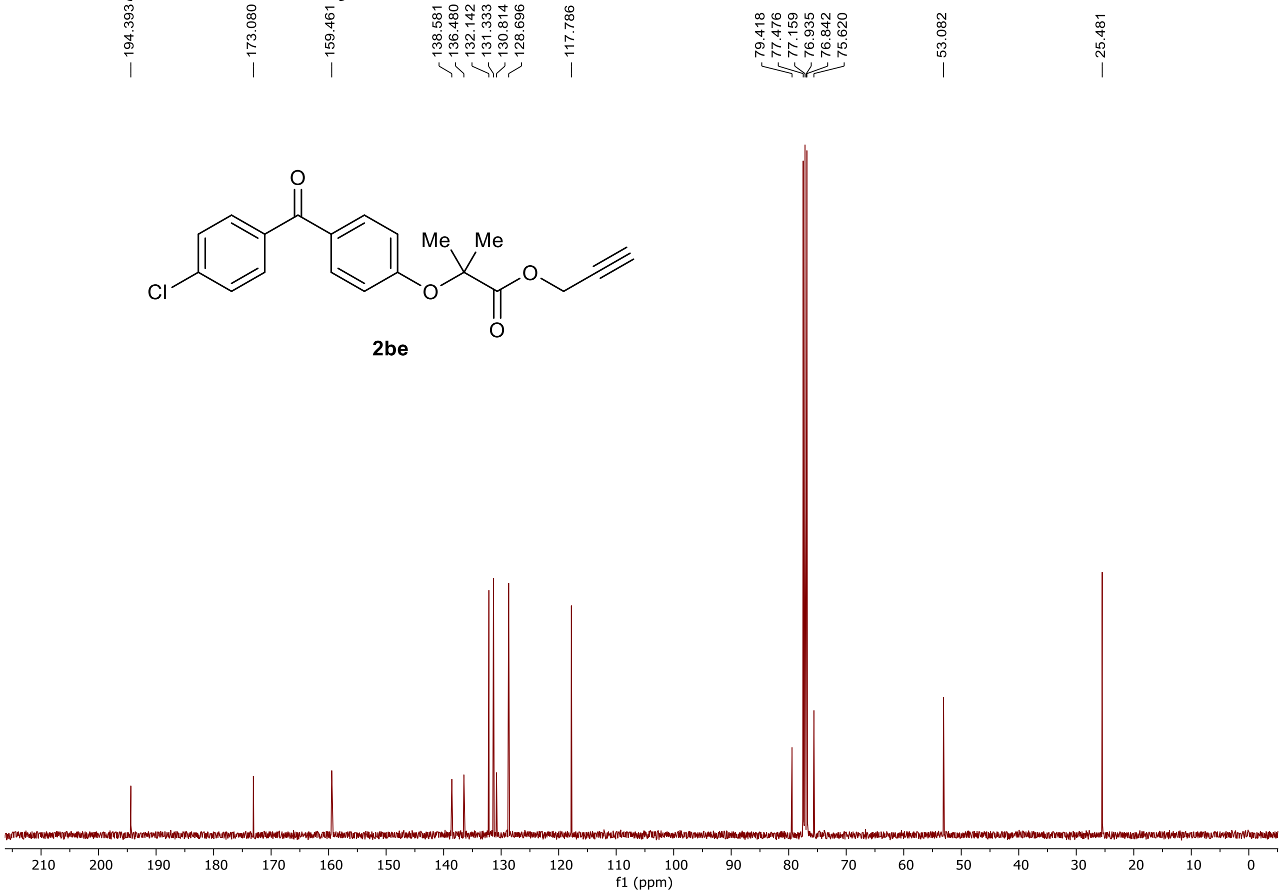

^{1}H NMR (400 MHz, $CDCl_3$) of 2bf

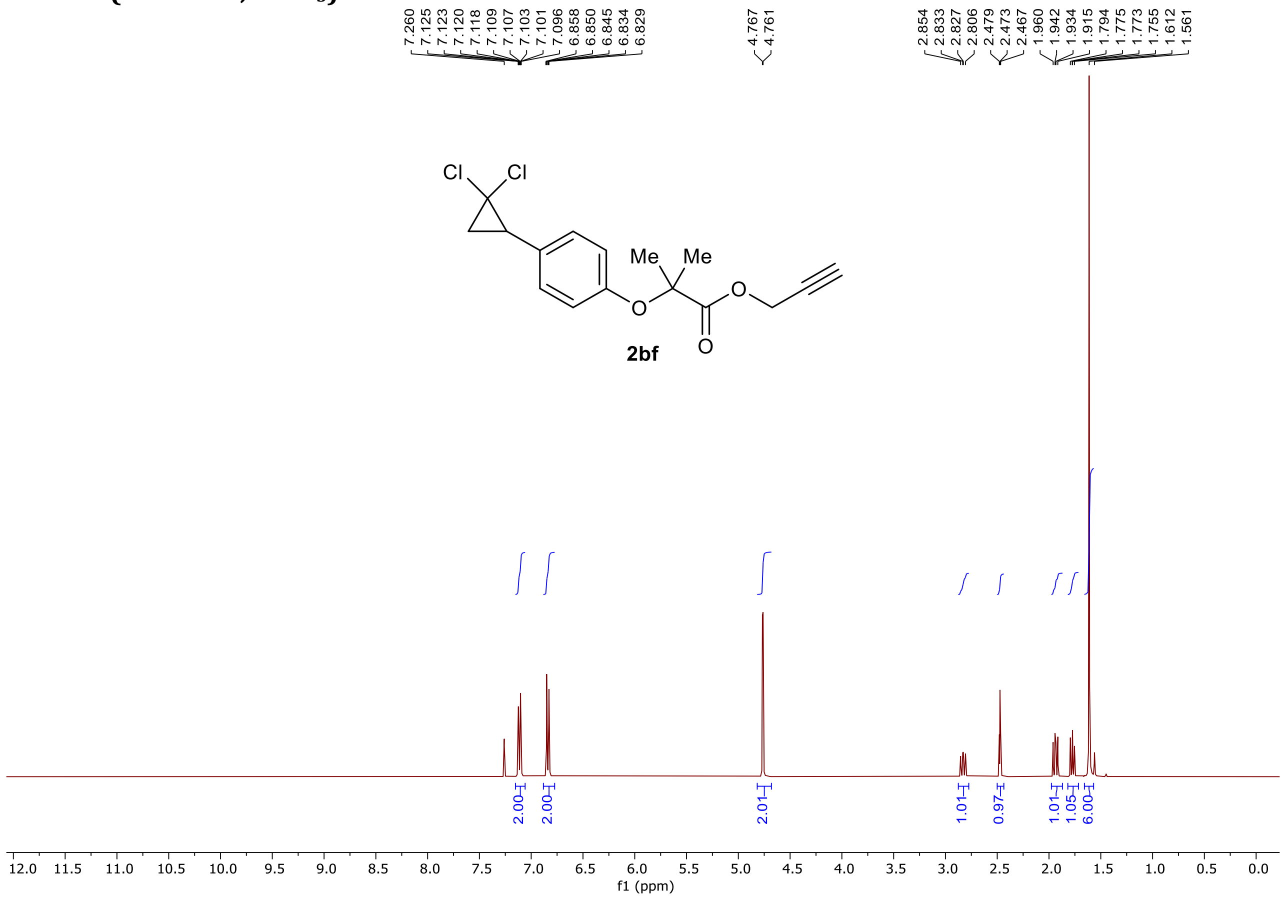

^{13}C NMR (101 MHz, $CDCl_3$) of 2bf

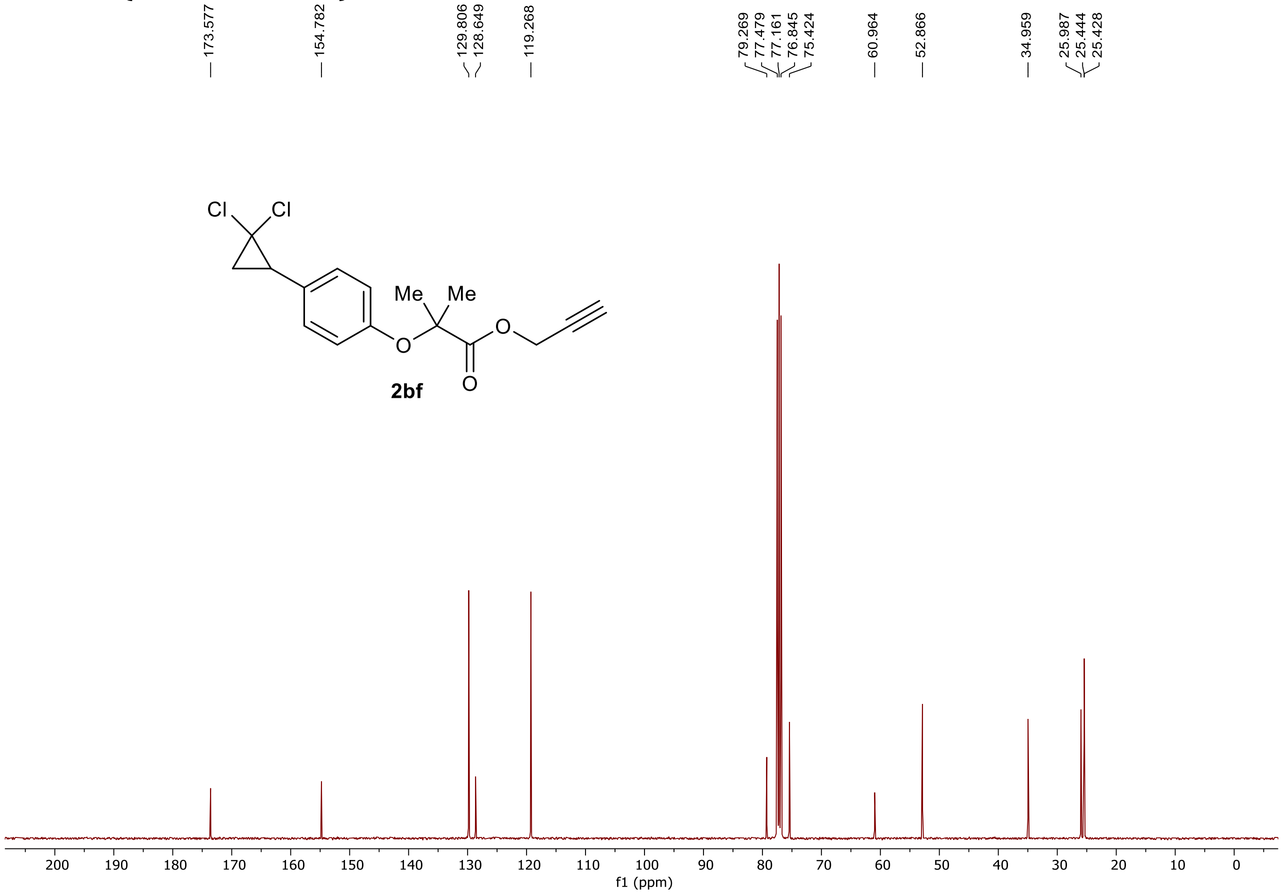

^{1}H NMR (400 MHz, $CDCl_3$) of 2bg

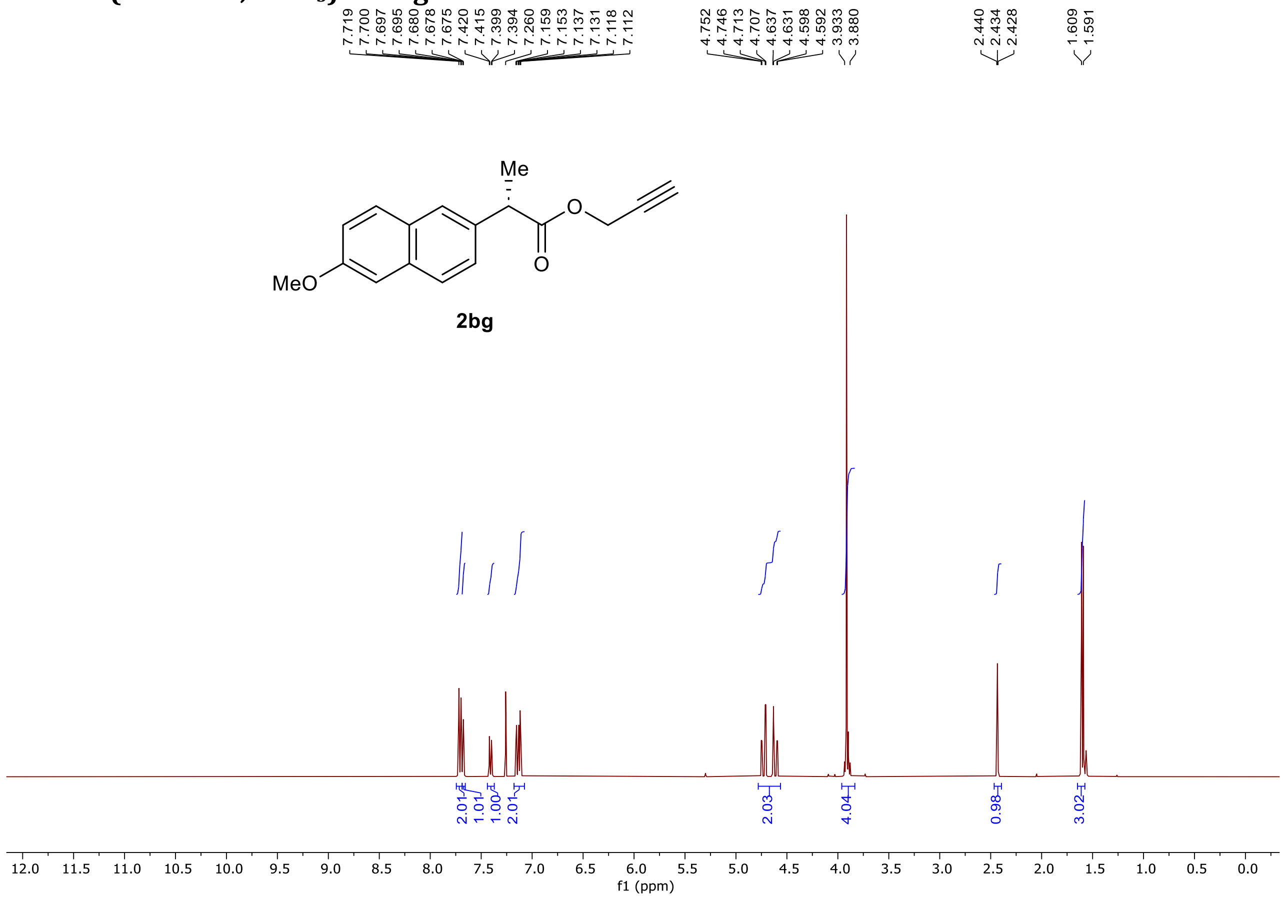

^{13}C NMR (101 MHz, $CDCl_3$) of 2bg

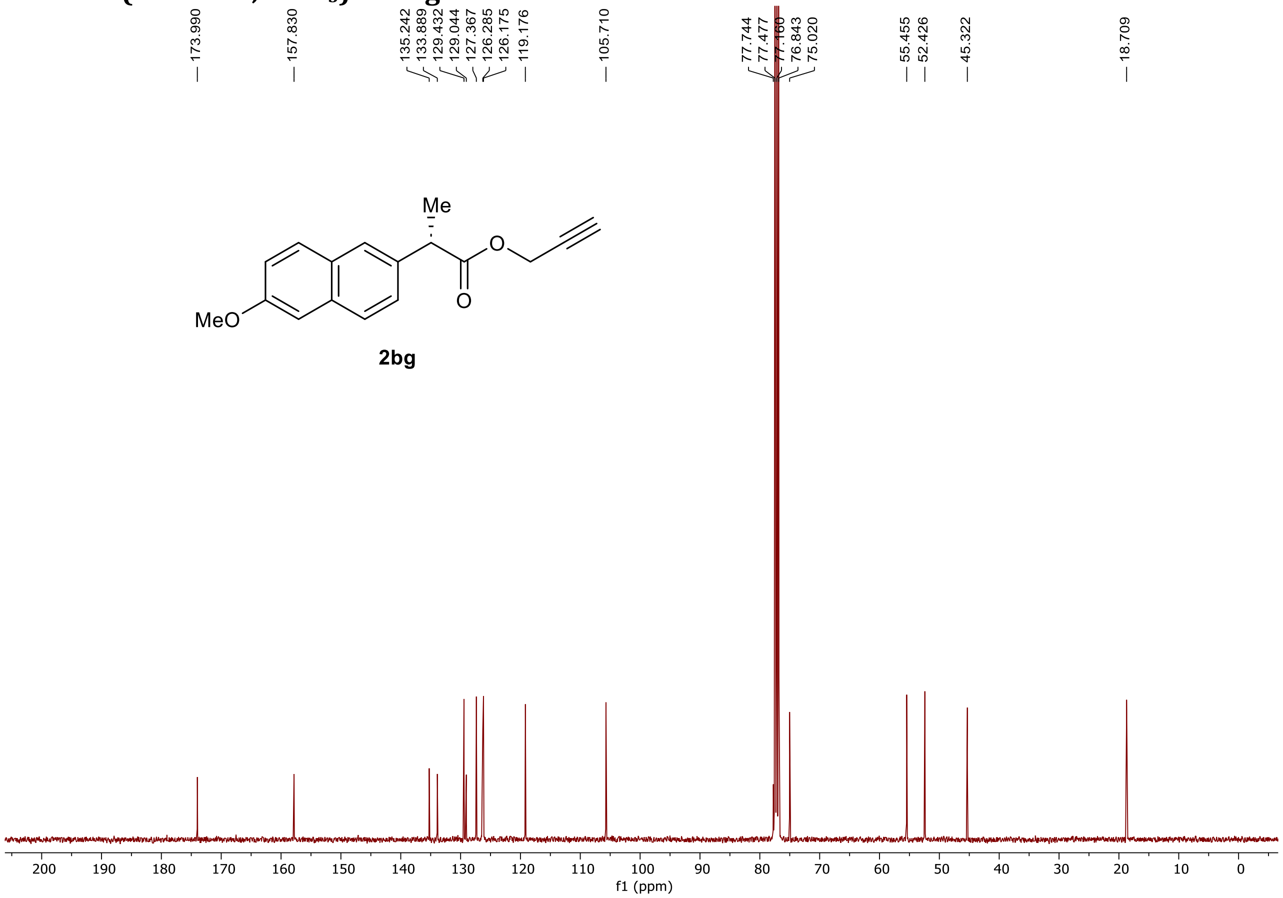

^{1}H NMR (400 MHz, $CDCl_3$) of 2bh

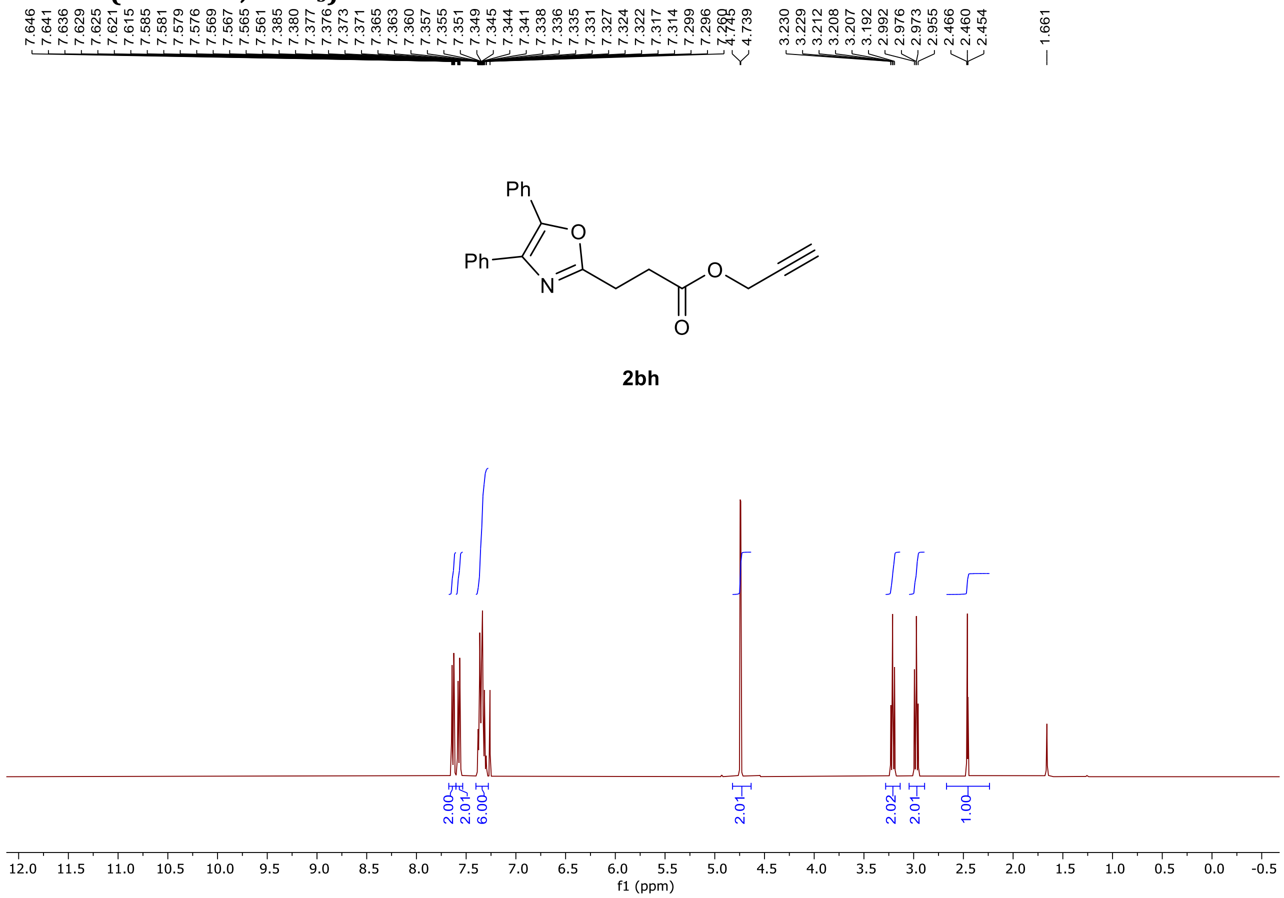

^{13}C NMR (101 MHz, $CDCl_3$) of 2bh

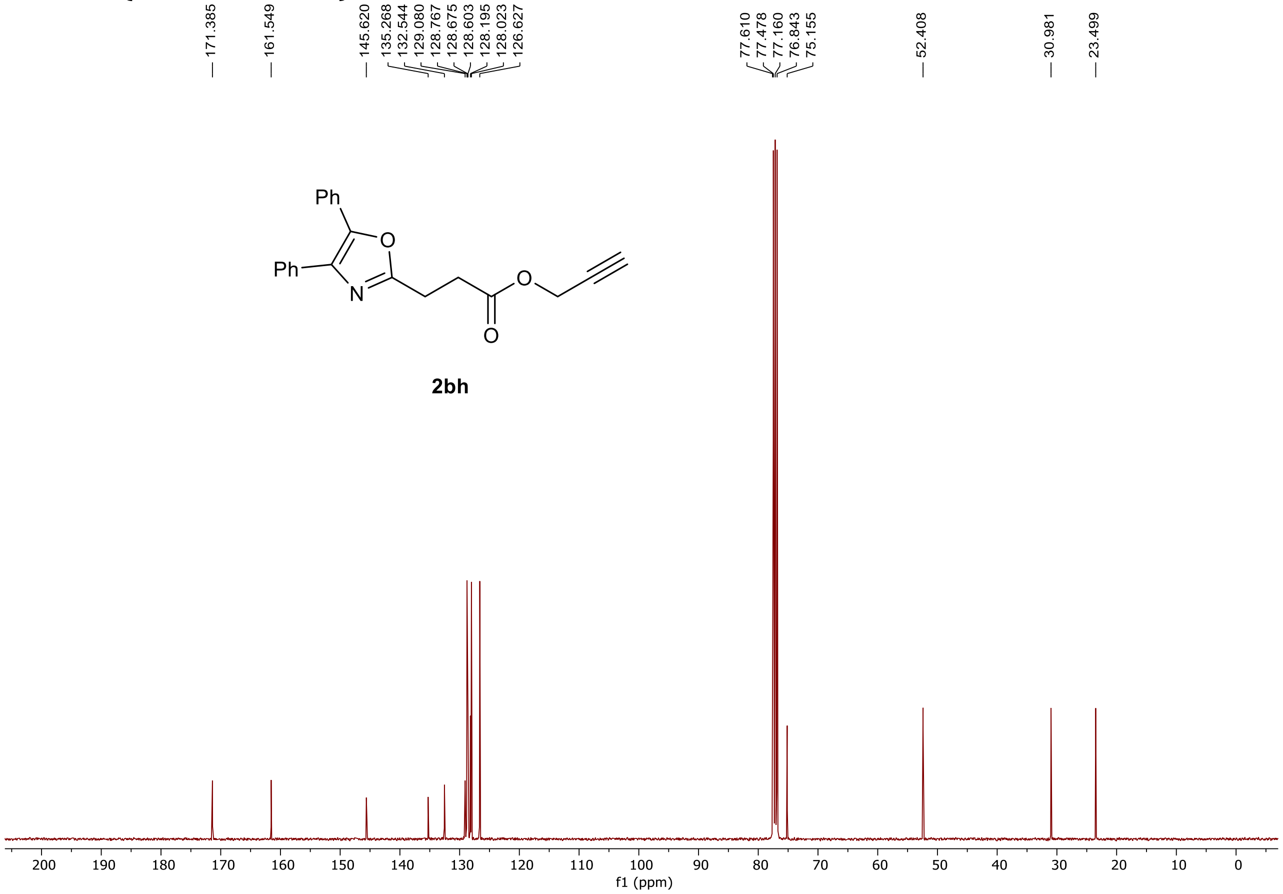

^{1}H NMR (400 MHz, $CDCl_3$) of 2bi

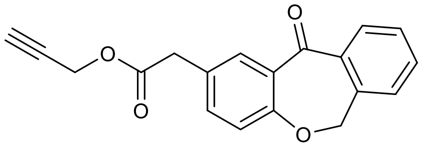

2bi

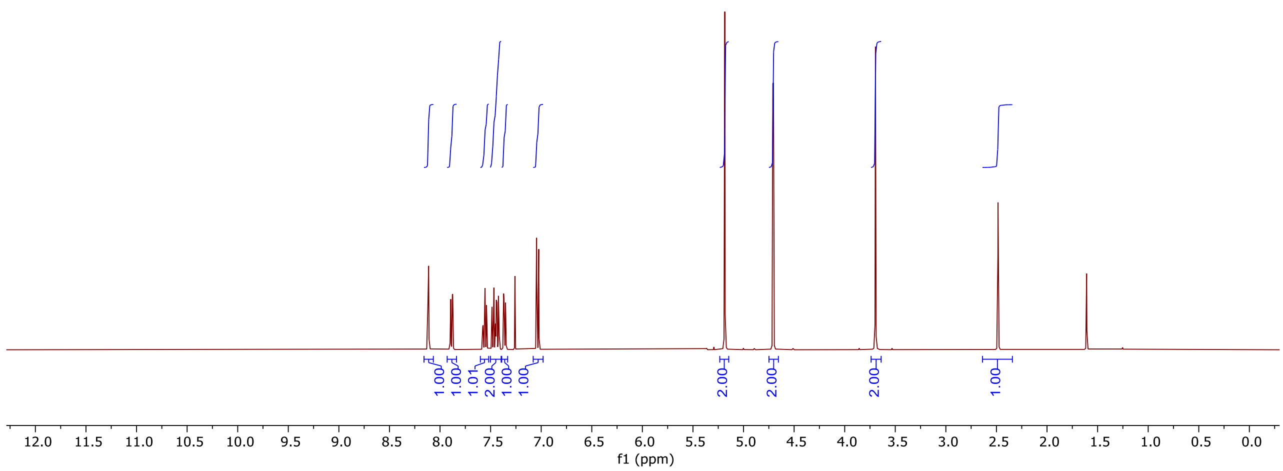

^{13}C NMR (101 MHz, $CDCl_3$) of 2bi

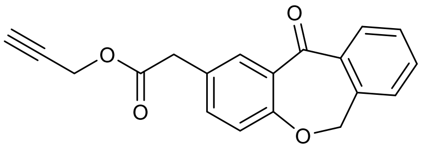

2bi

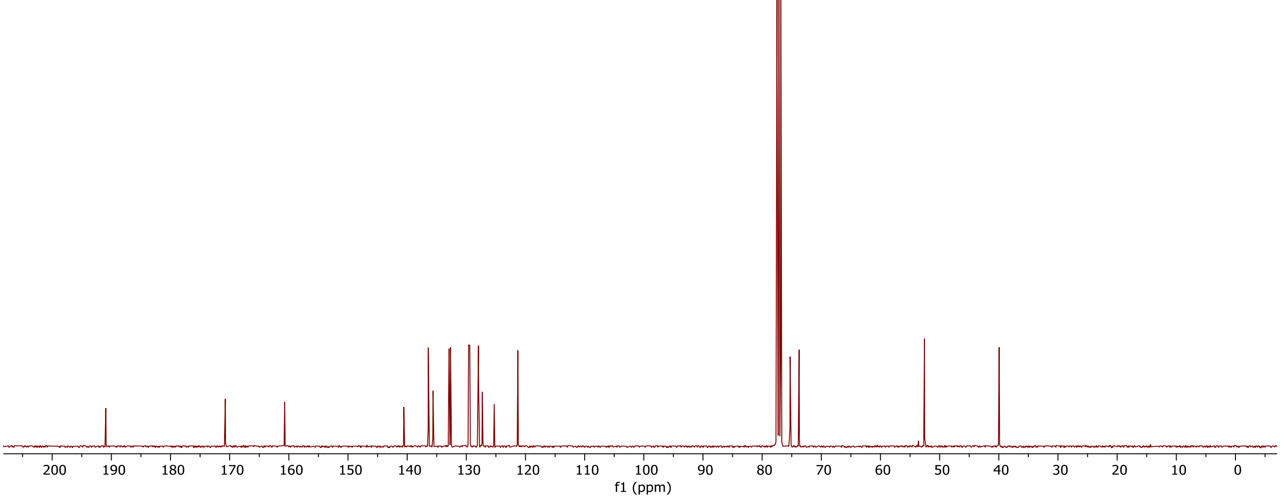

^{1}H NMR (400 MHz, $CDCl_3$) of 2bj

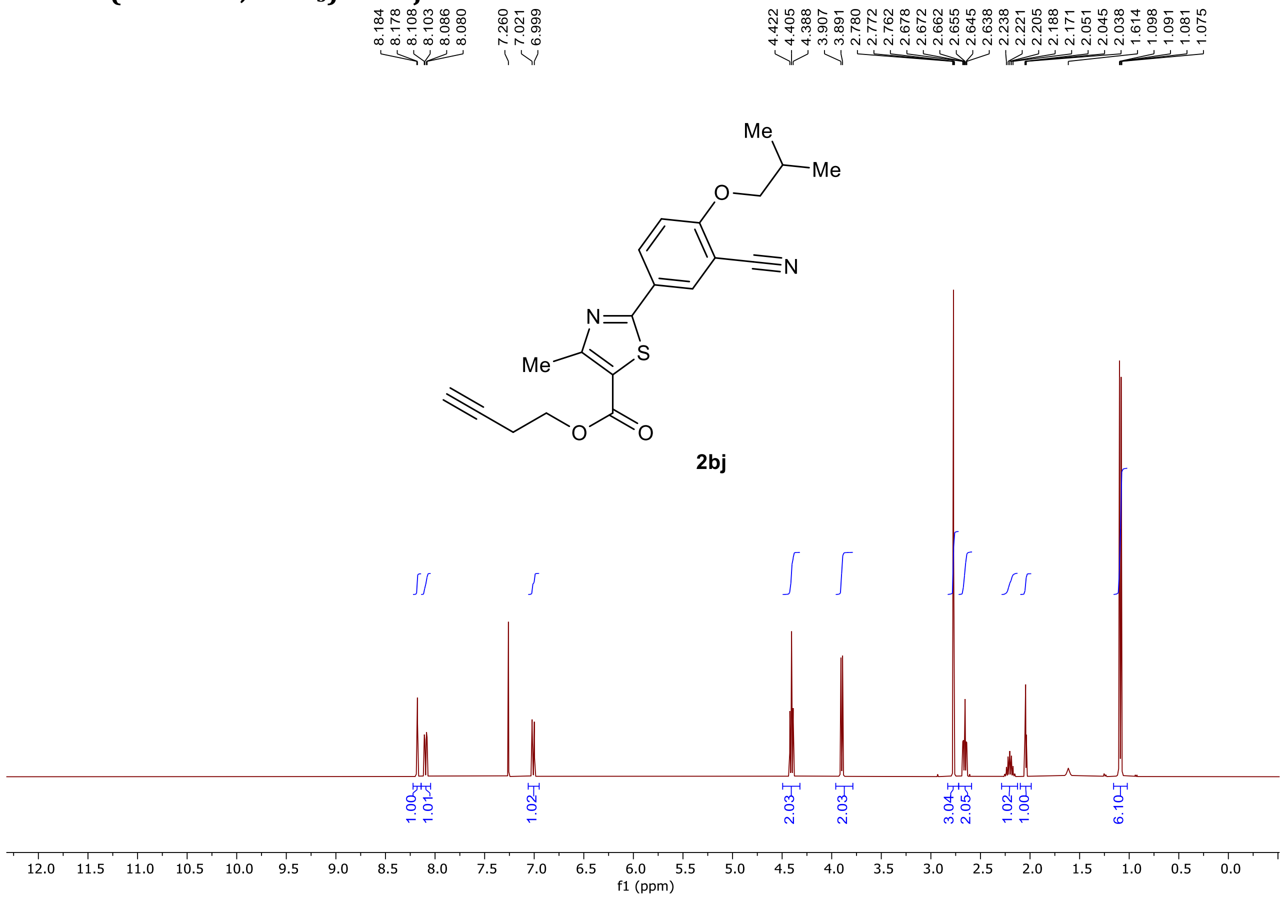

^{13}C NMR (101 MHz, $CDCl_3$) of 2bj

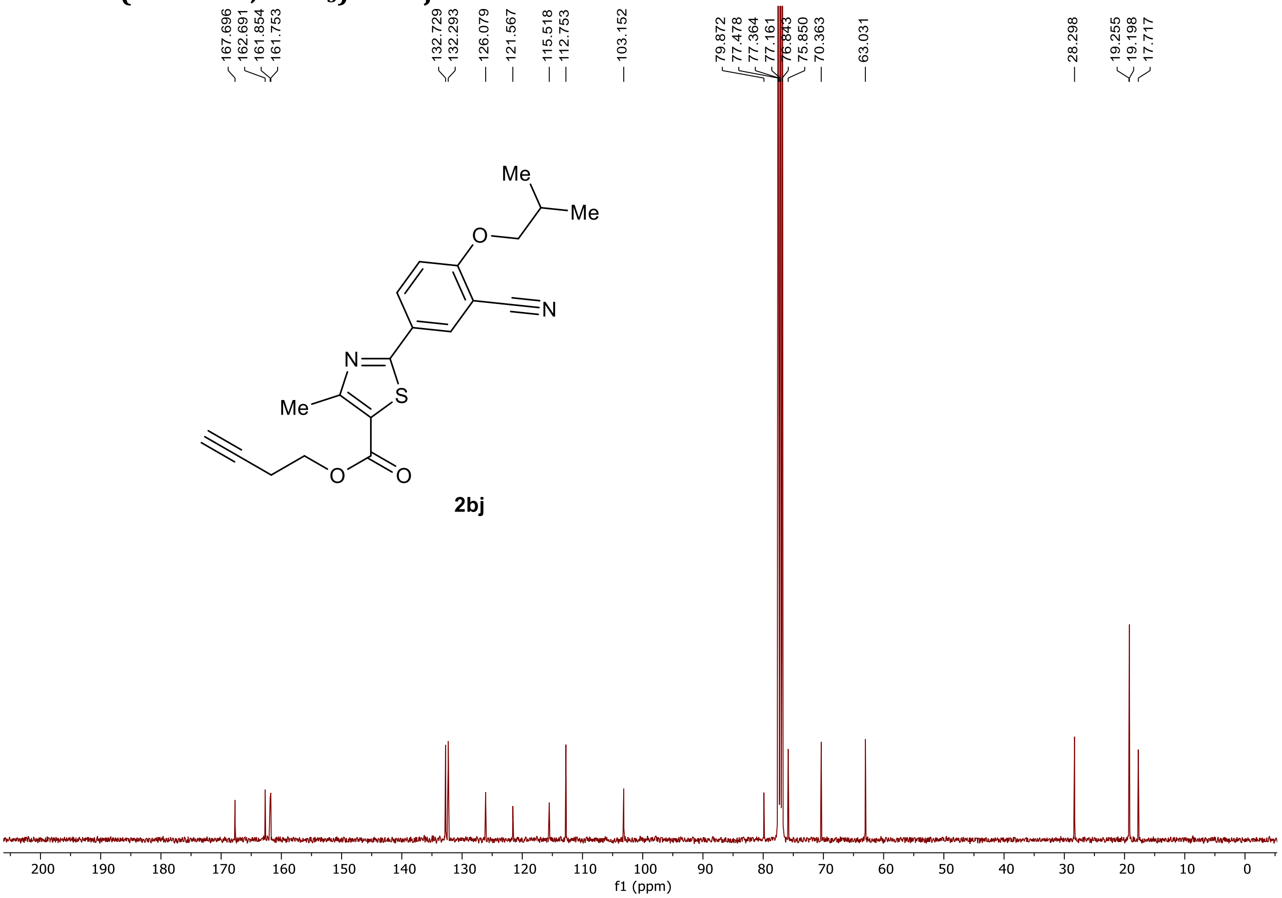

^{1}H NMR (400 MHz, $CDCl_3$) of 2bk

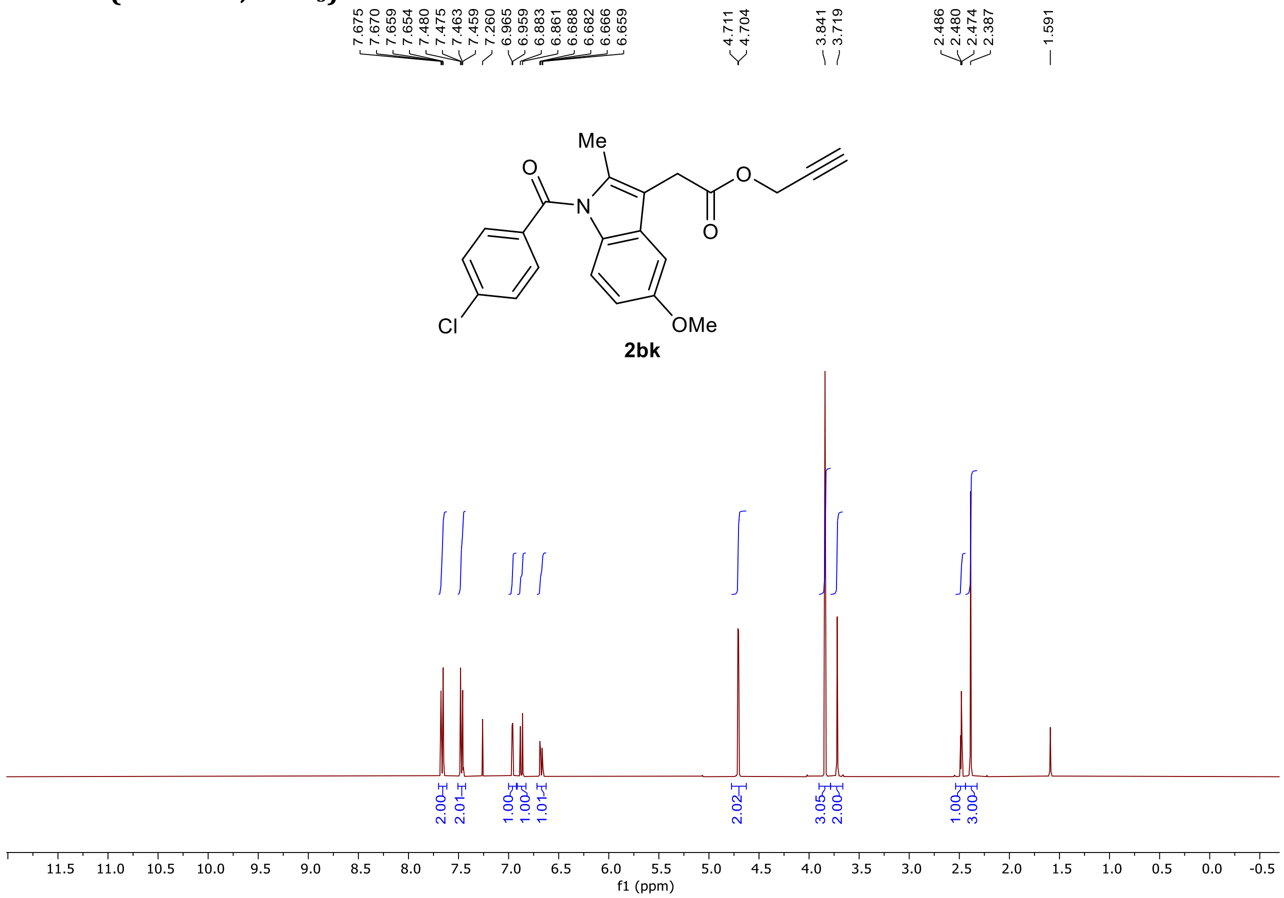

^{13}C NMR (101 MHz, $CDCl_3$) of 2bk

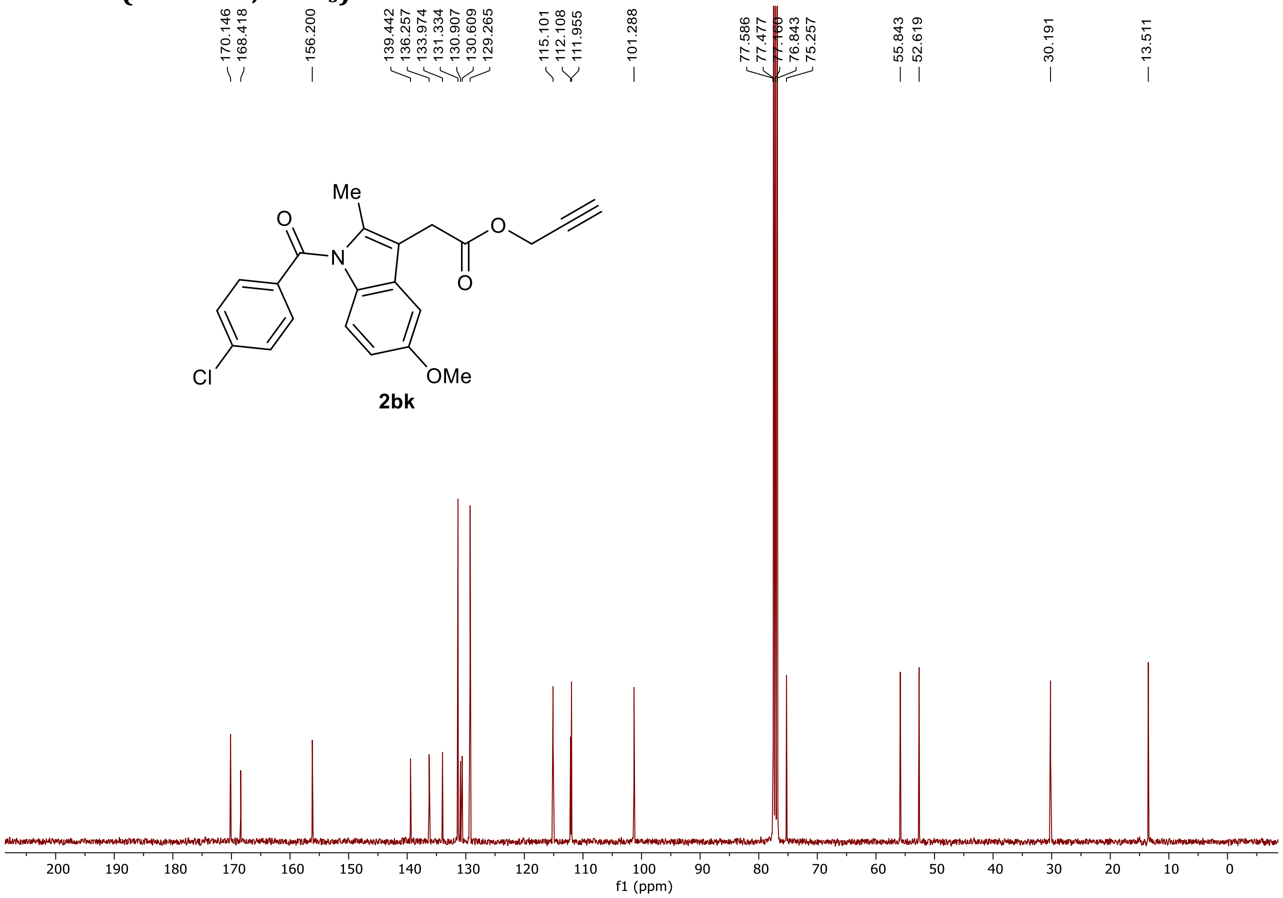

^{1}H NMR (400 MHz, $CDCl_3$) of 2bn

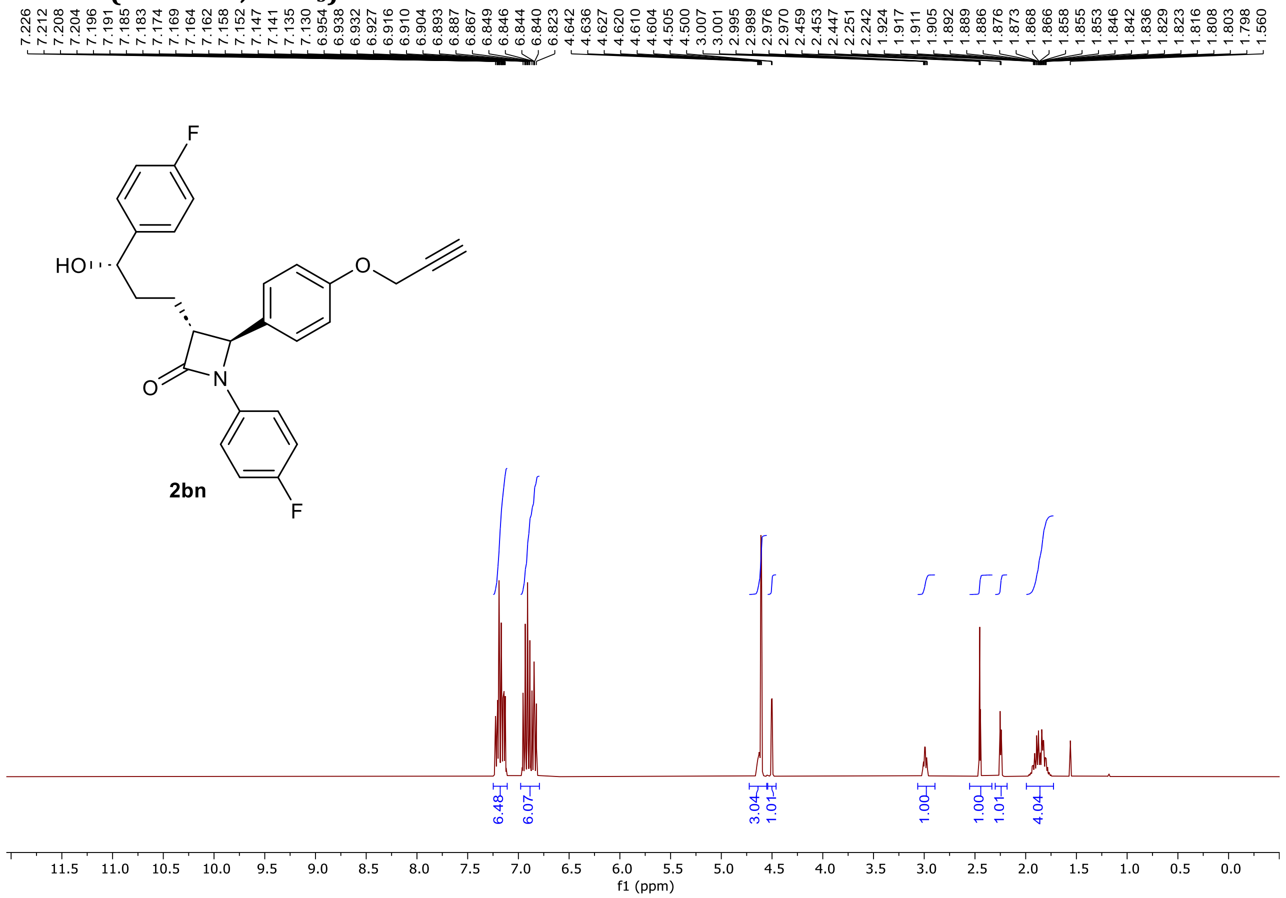

^{13}C NMR (101 MHz, $CDCl_3$) of 2bn

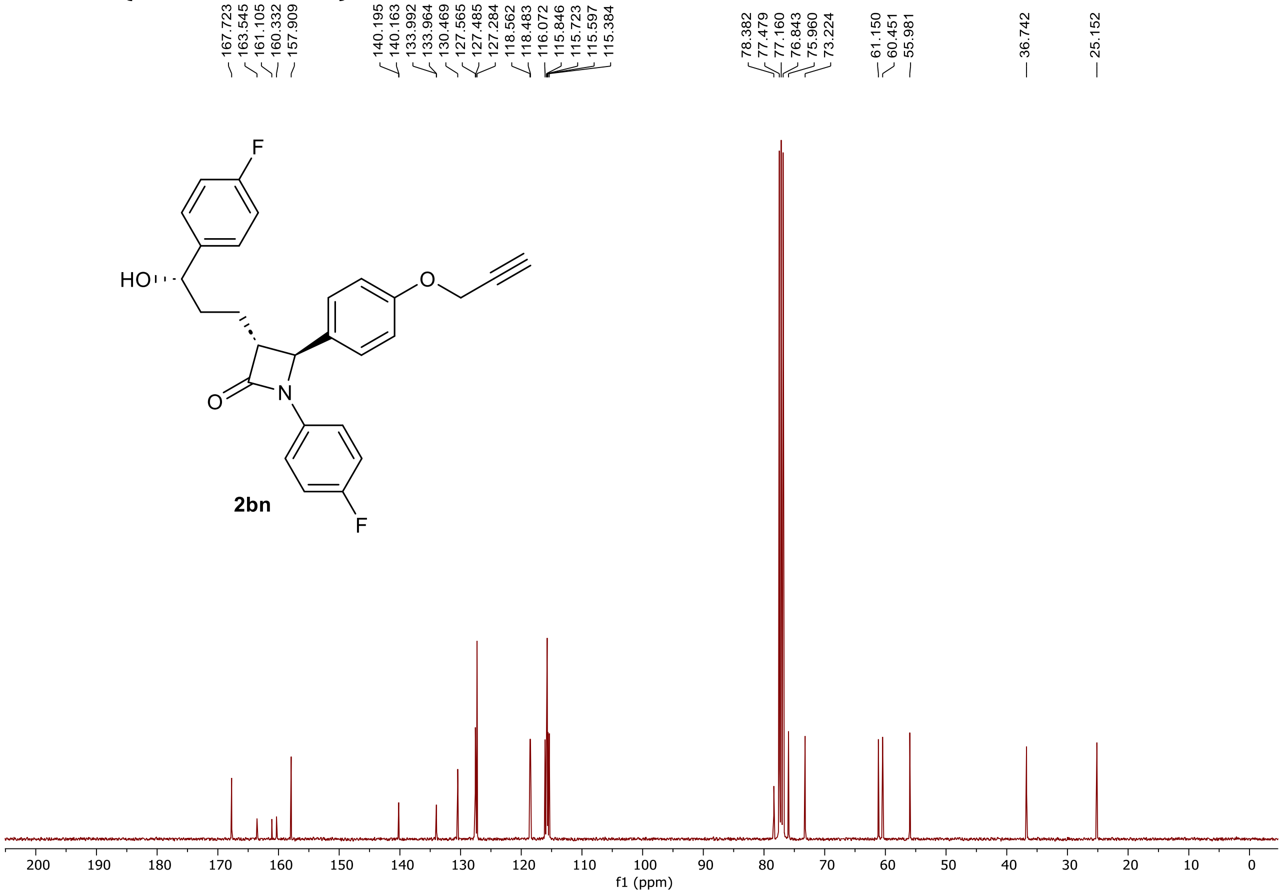

^{19}F NMR (377 MHz, $CDCl_3$) of 2bn

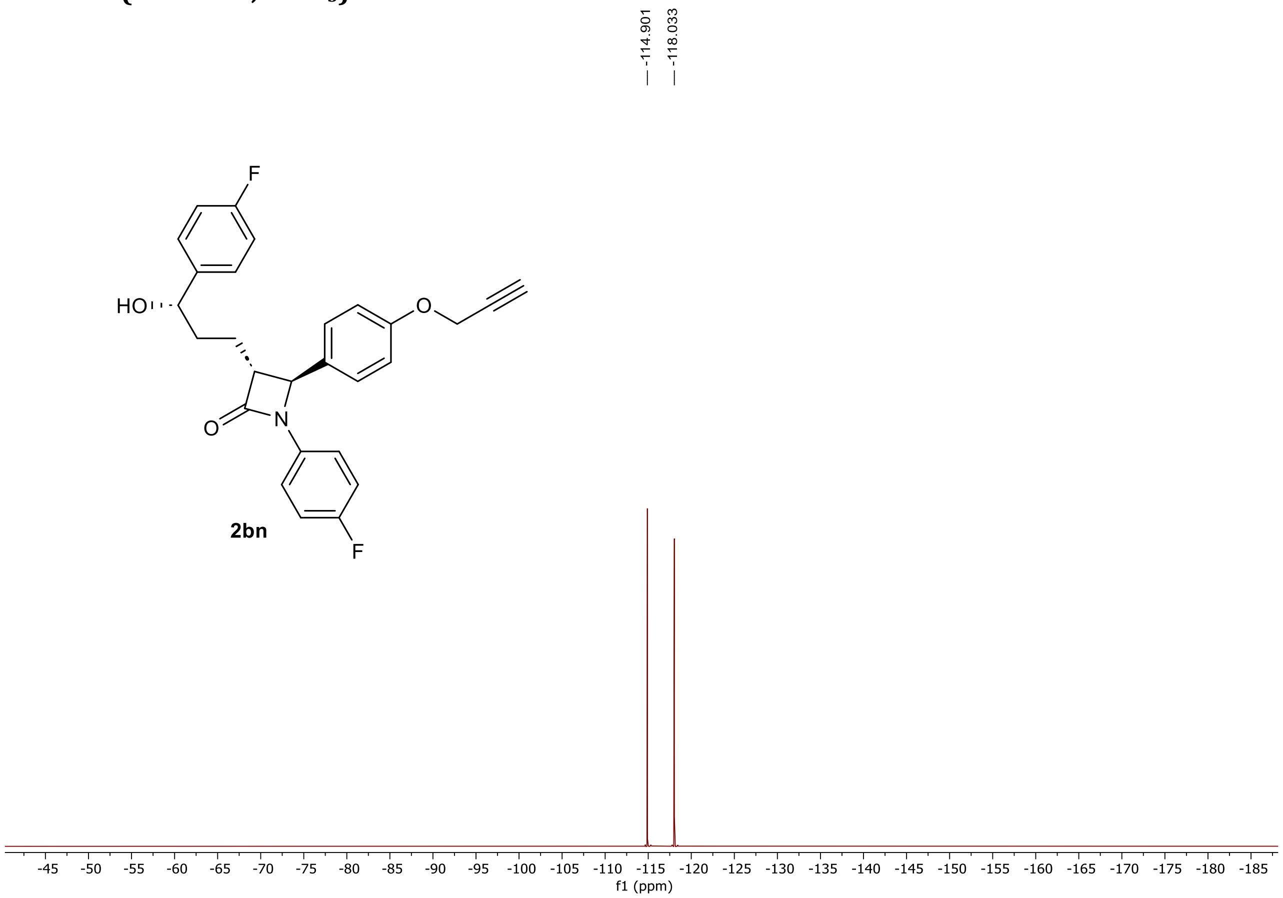

^{1}H NMR (400 MHz, $CDCl_3$) of 2bo

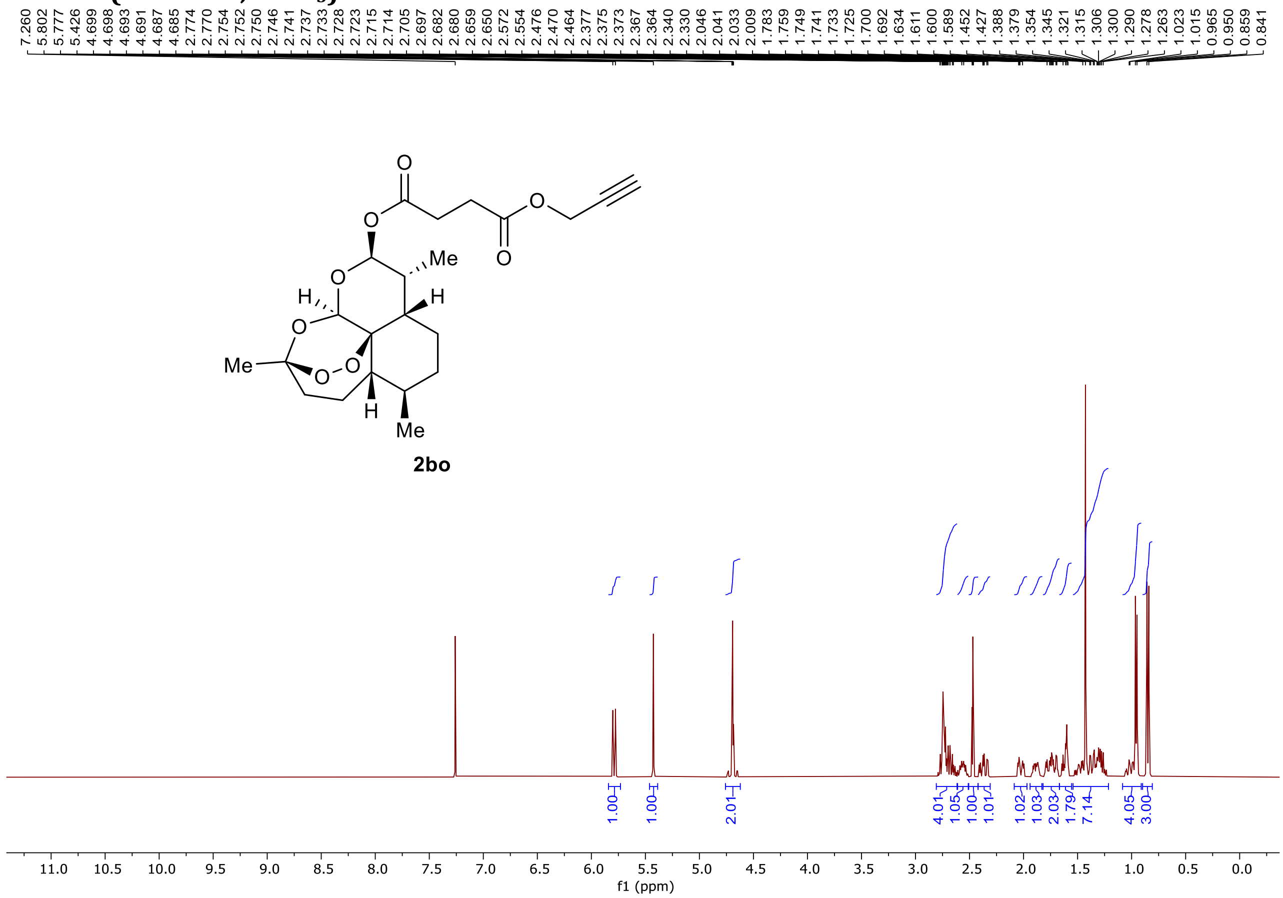

^{13}C NMR (101 MHz, CDCl$_3$) of 2bo

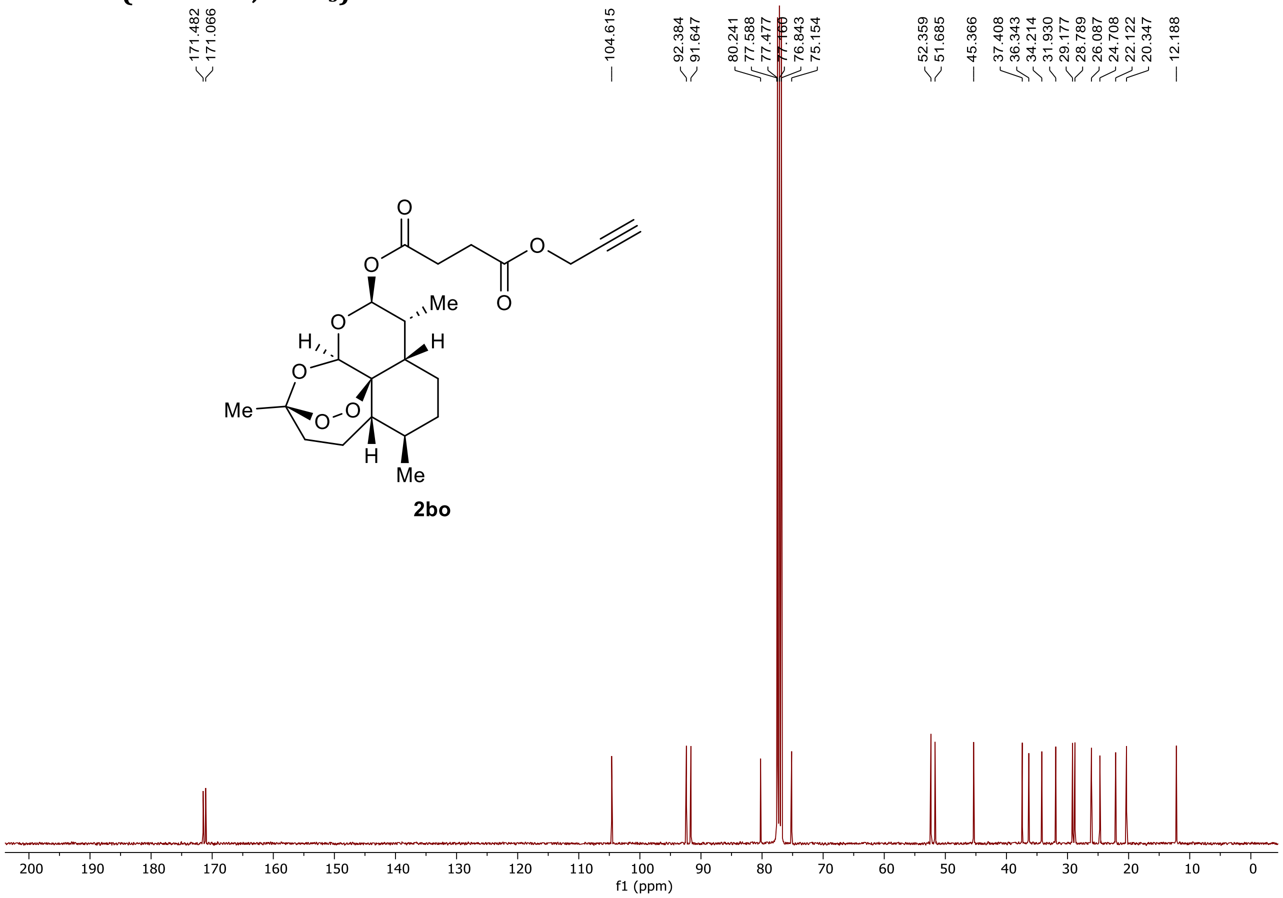

^{1}H NMR (400 MHz, CDCl$_3$) of 2bp

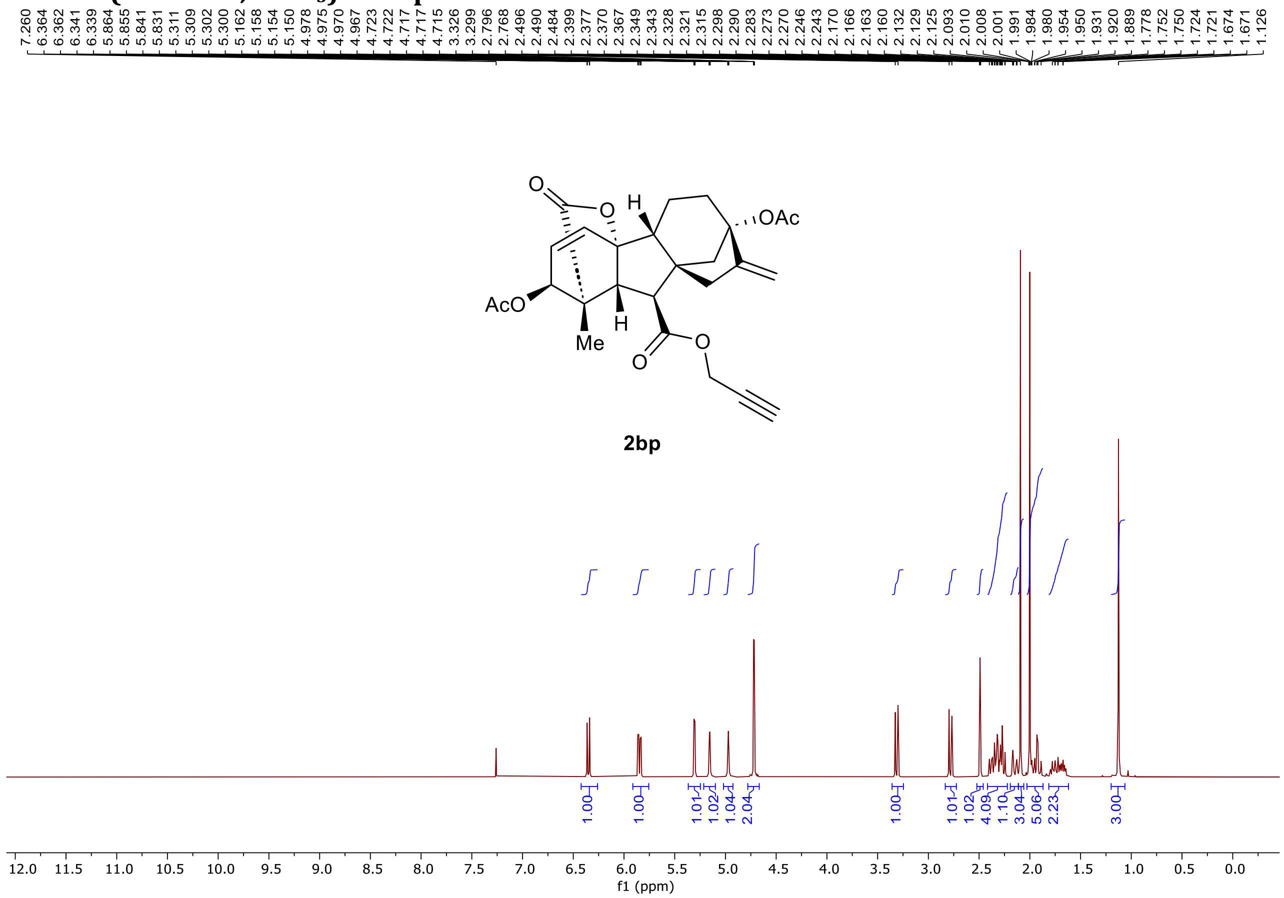

^{13}C NMR (101 MHz, CDCl$_3$) of 2bp

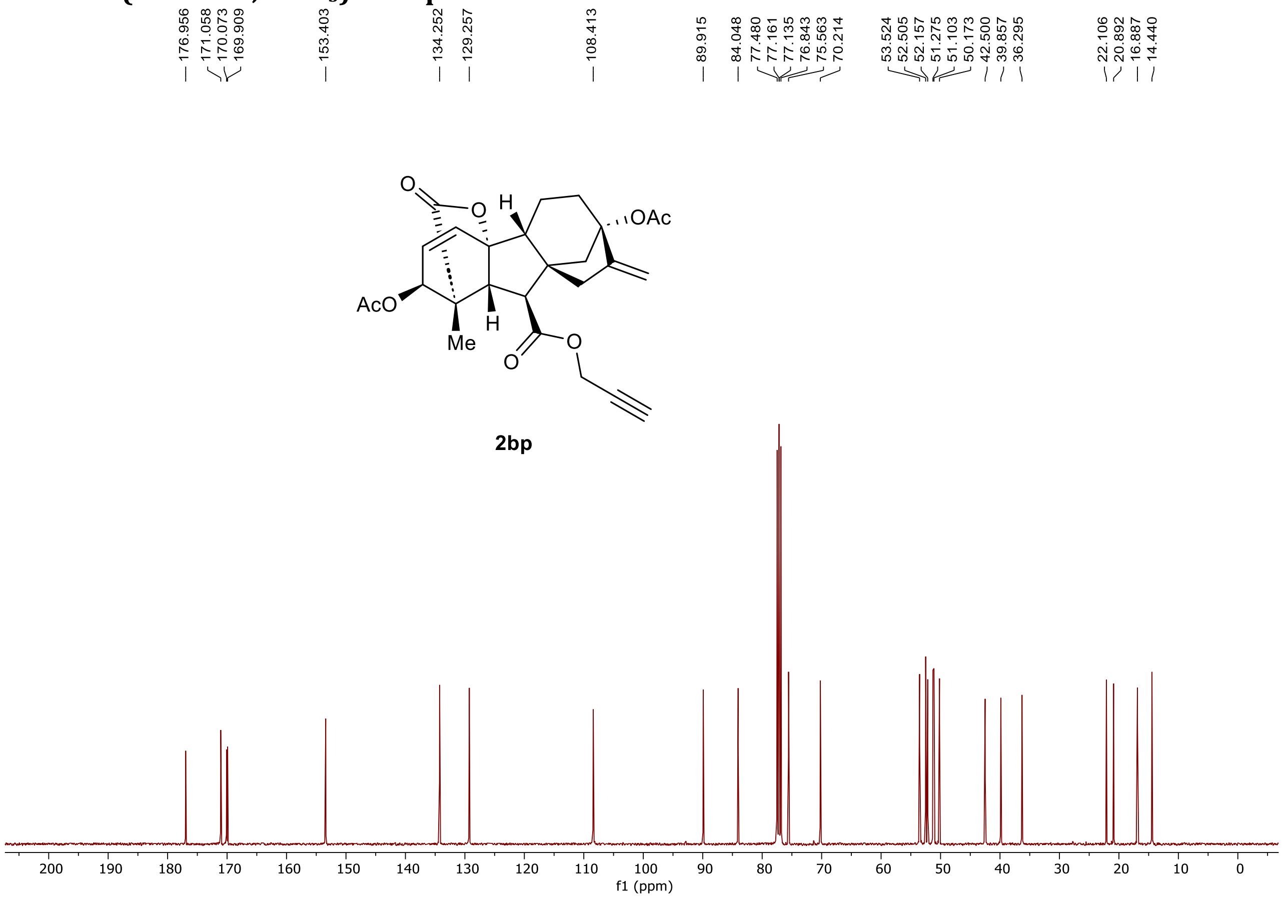

^{1}H NMR (400 MHz, CDCl$_3$) of 4-methyl-*N*-phenyl-*N*-(prop-2-yn-1-yl)benzenesulfonamide

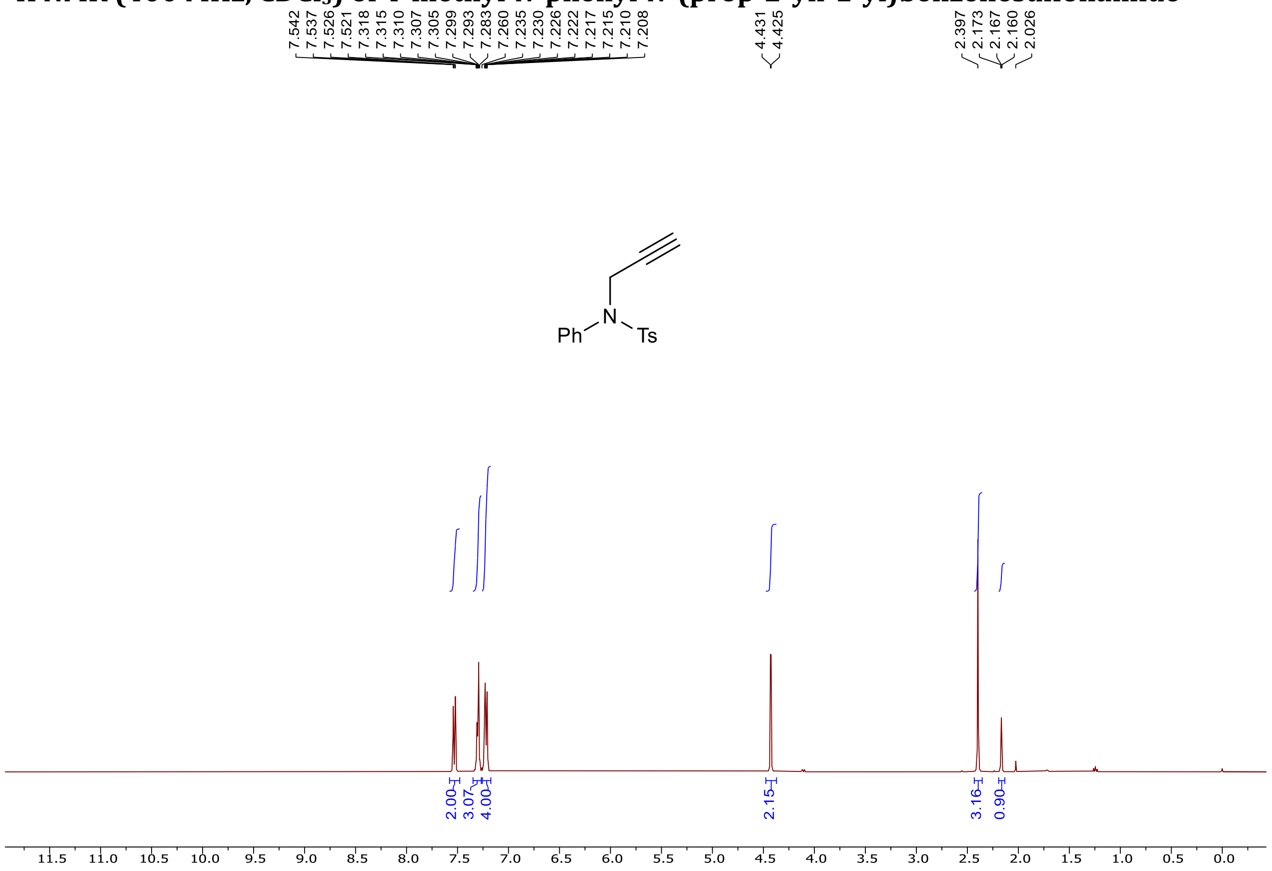

^{13}C NMR (101 MHz, $CDCl_3$) of 4-methyl-*N*-phenyl-*N*-(prop-2-yn-1-yl)benzenesulfonamide

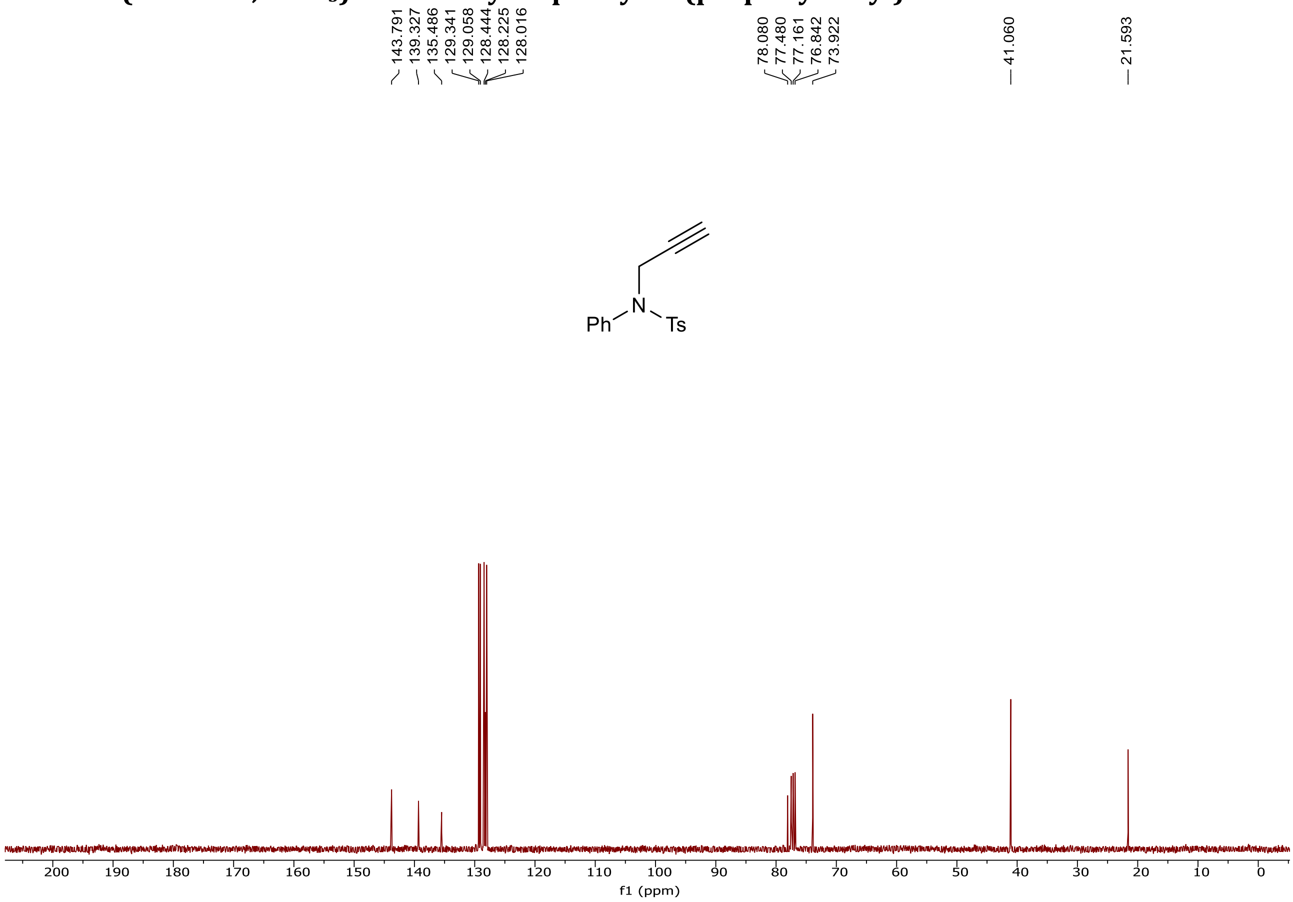

^{1}H NMR (400 MHz, $CDCl_3$) of 4-methyl-*N*-phenyl-*N*-(propa-1,2-dien-1-yl)benzenesulfonamide

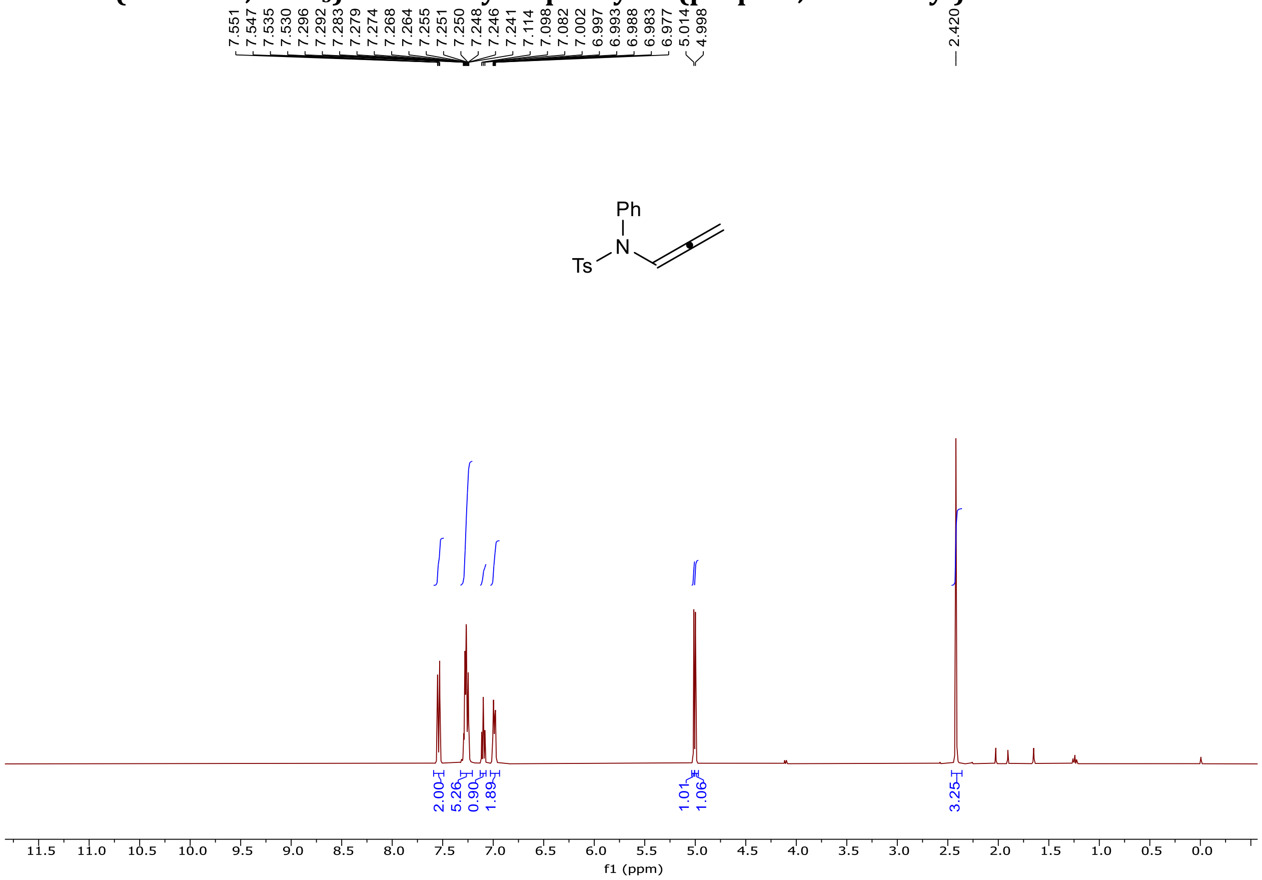

^{13}C NMR (101 MHz, $CDCl_3$) of 4-methyl-*N*-phenyl-*N*-(propa-1,2-dien-1-yl)benzenesulfonamide

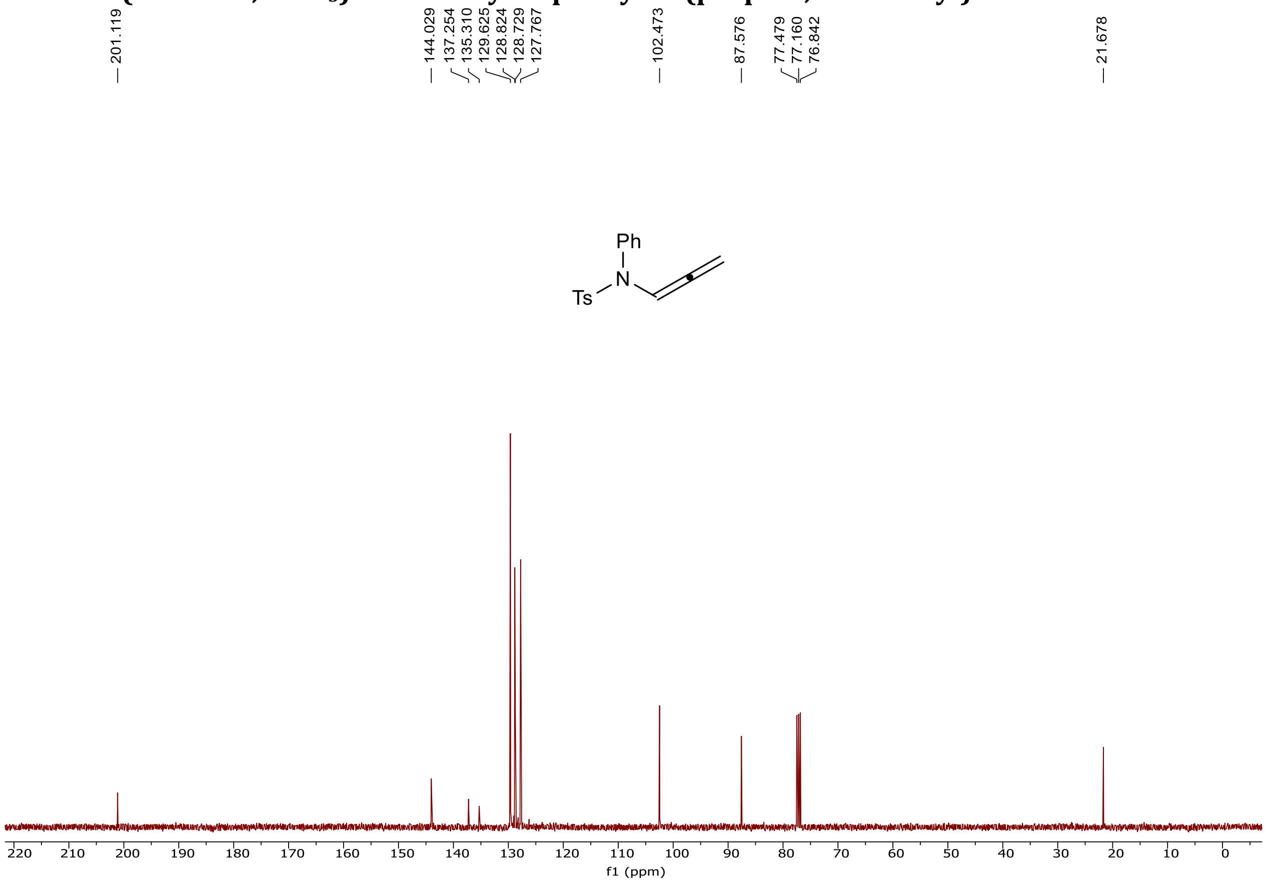

^{1}H NMR (400 MHz, $CDCl_3$) of 3a

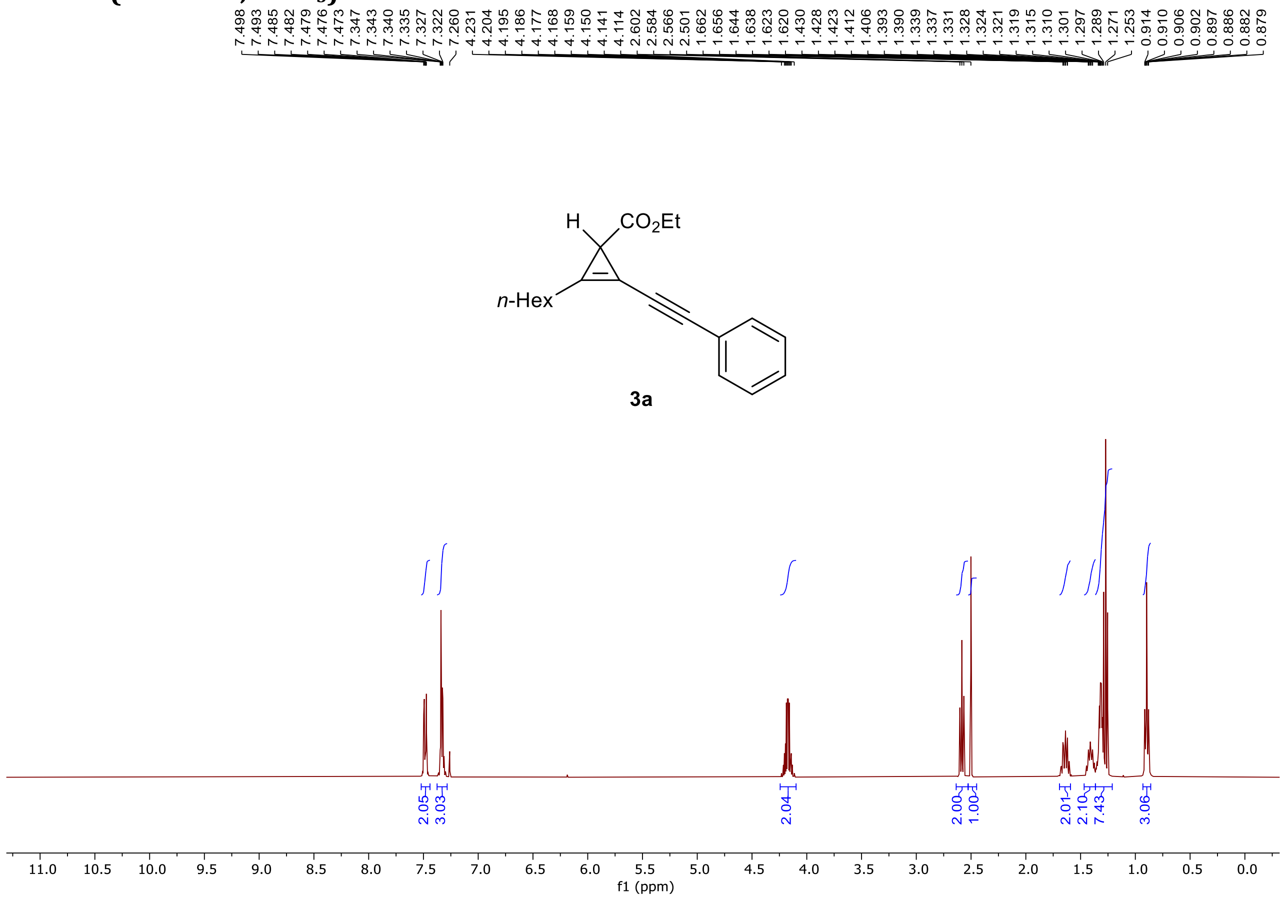

^{13}C NMR (101 MHz, $CDCl_3$) of 3a

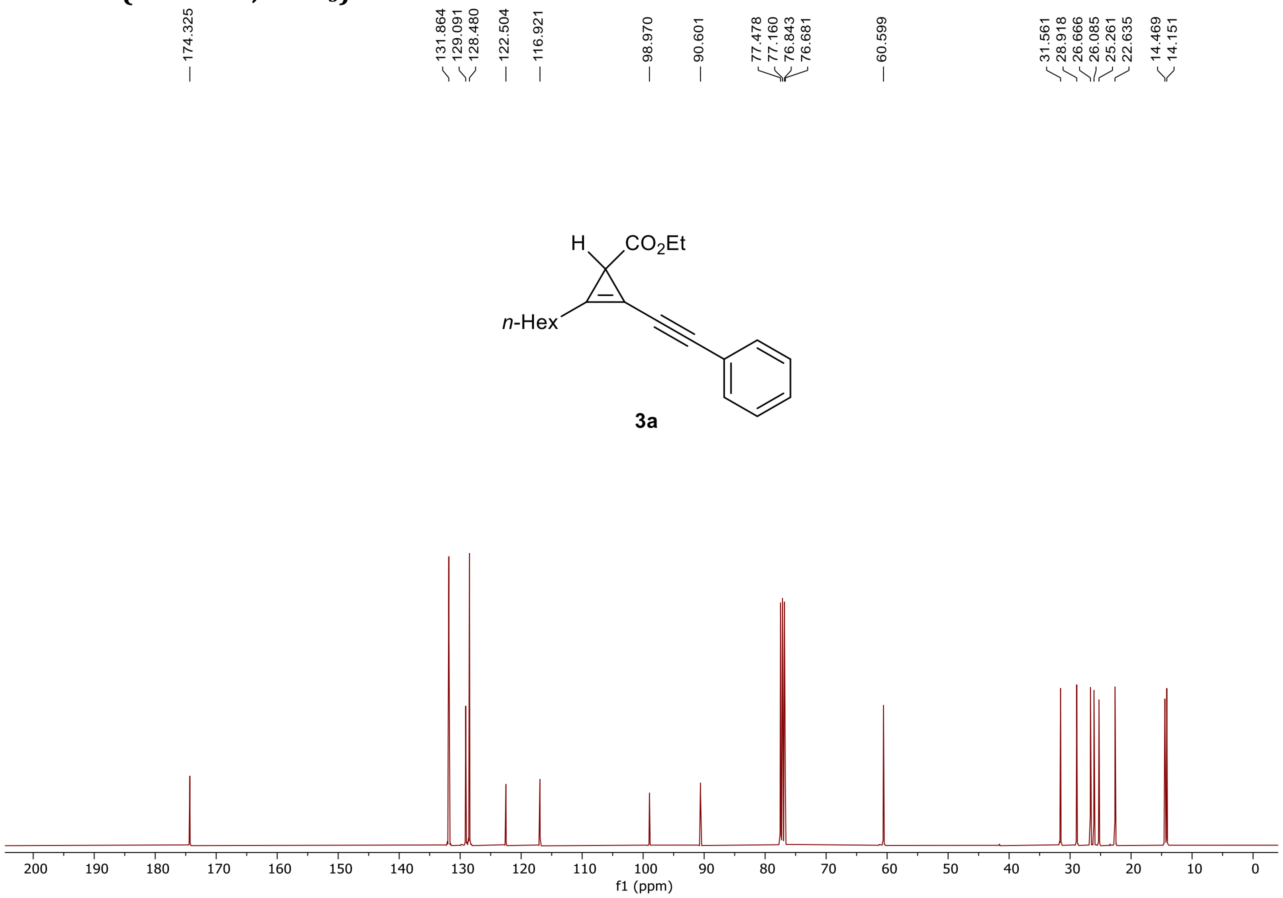

^{1}H NMR (400 MHz, $CDCl_3$) of 3b

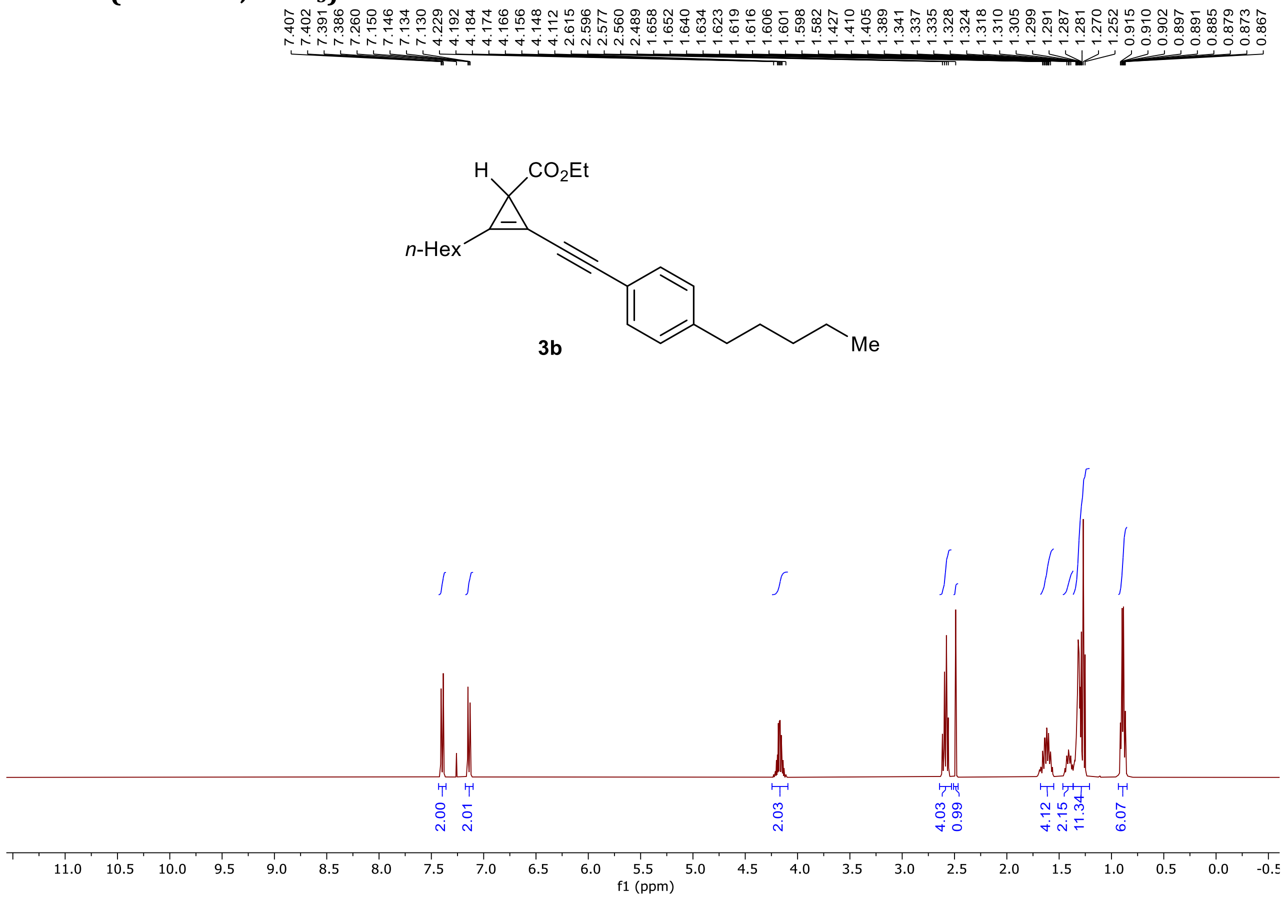

^{13}C NMR (101 MHz, $CDCl_3$) of 3b

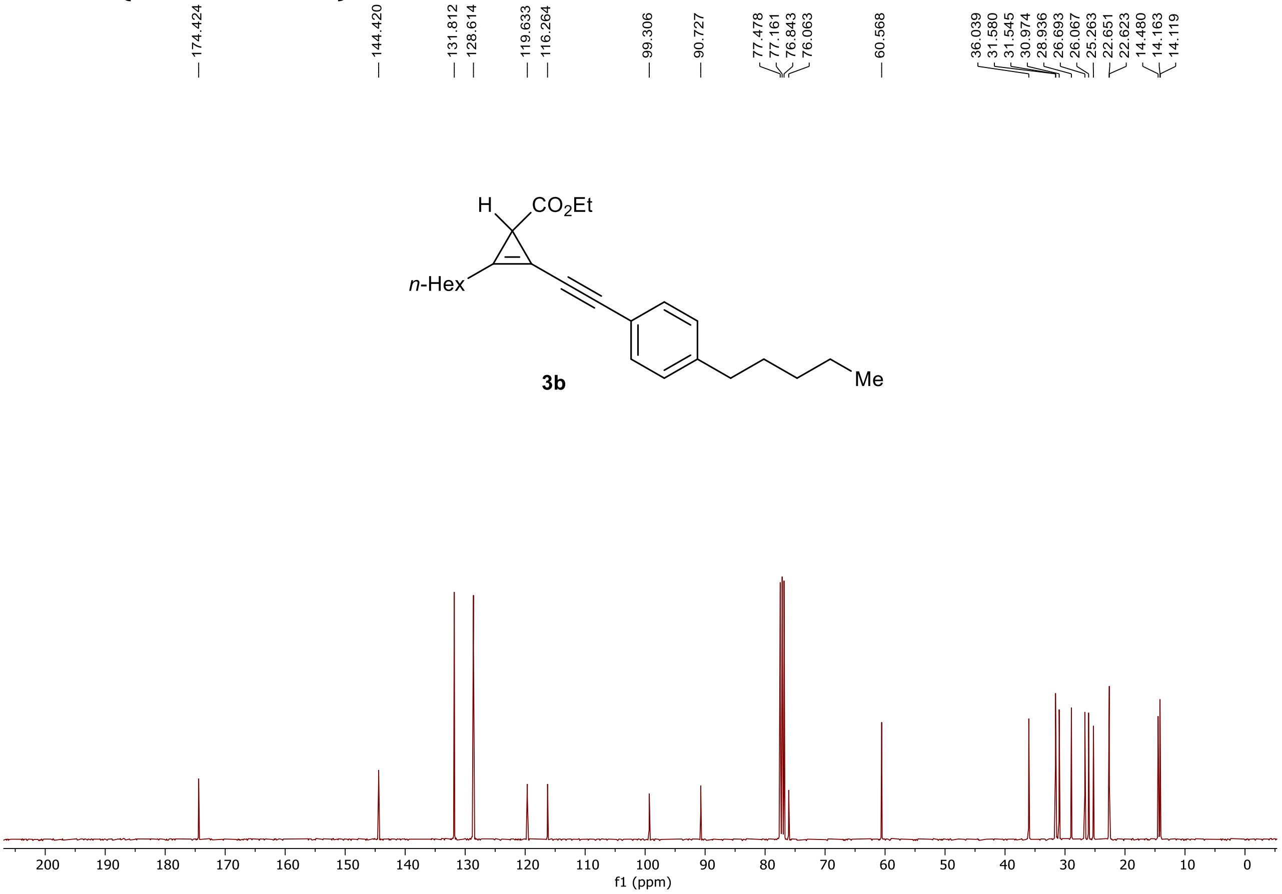

^{1}H NMR (400 MHz, $CDCl_3$) of 3c

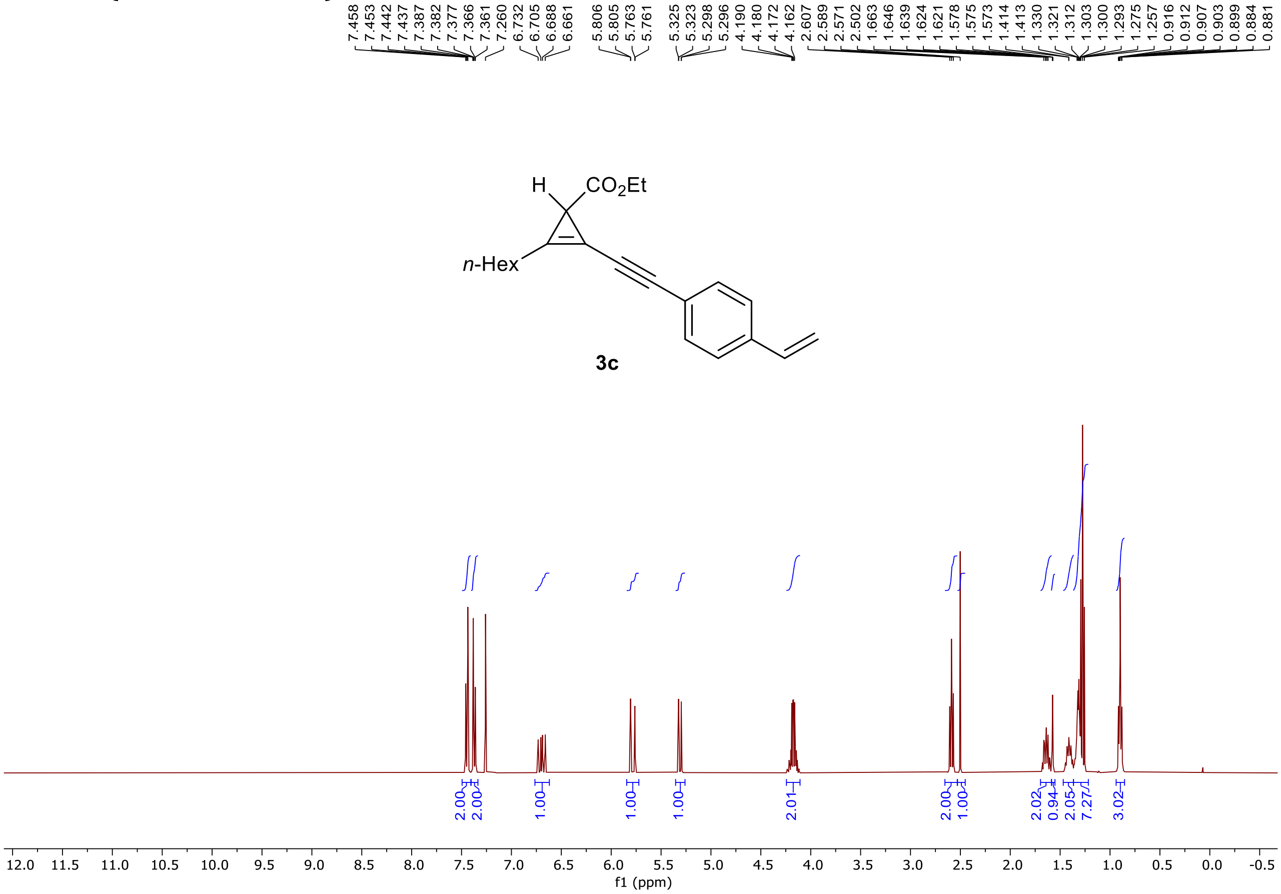

^{13}C NMR (101 MHz, $CDCl_3$) of 3c

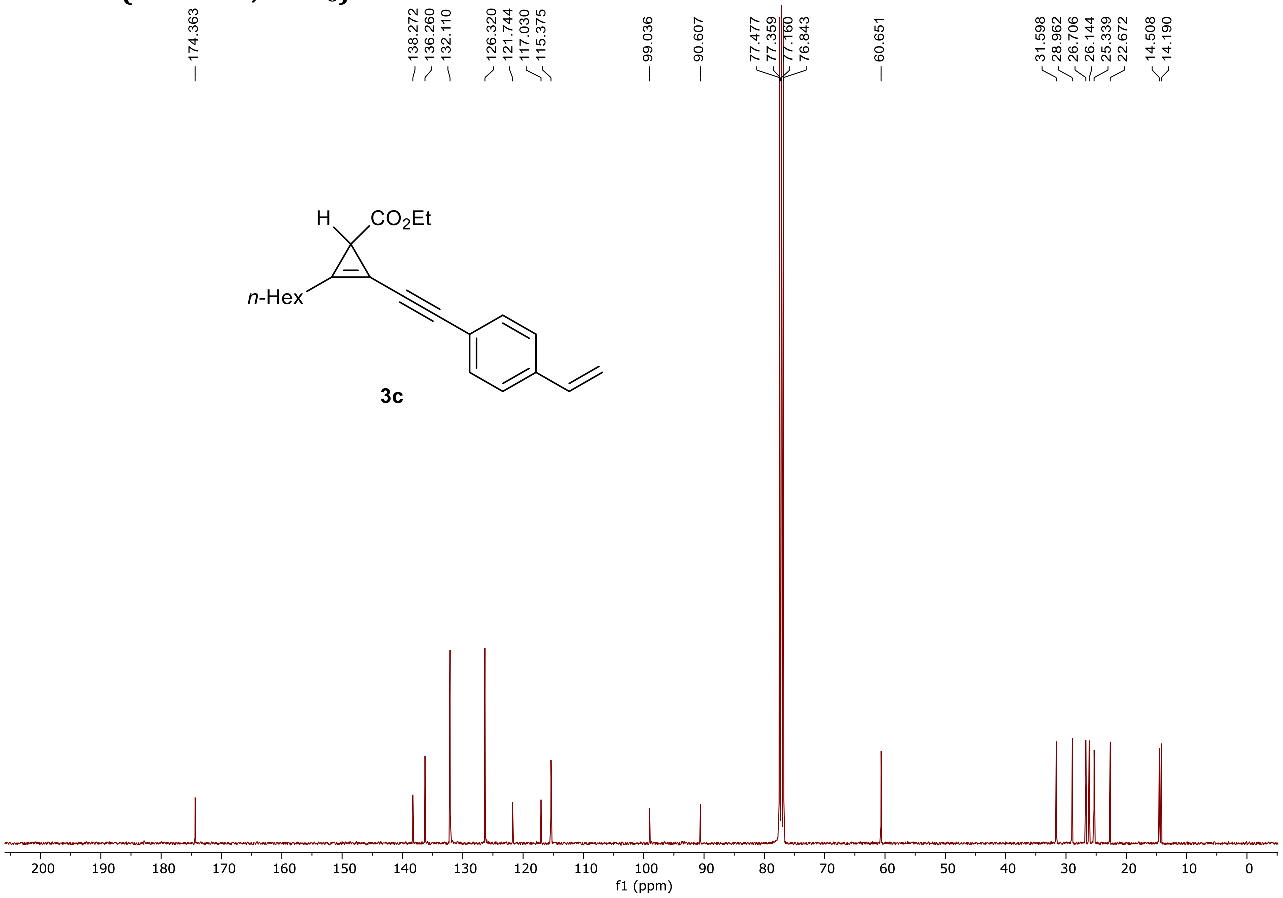

^{1}H NMR (400 MHz, $CDCl_3$) of 3d

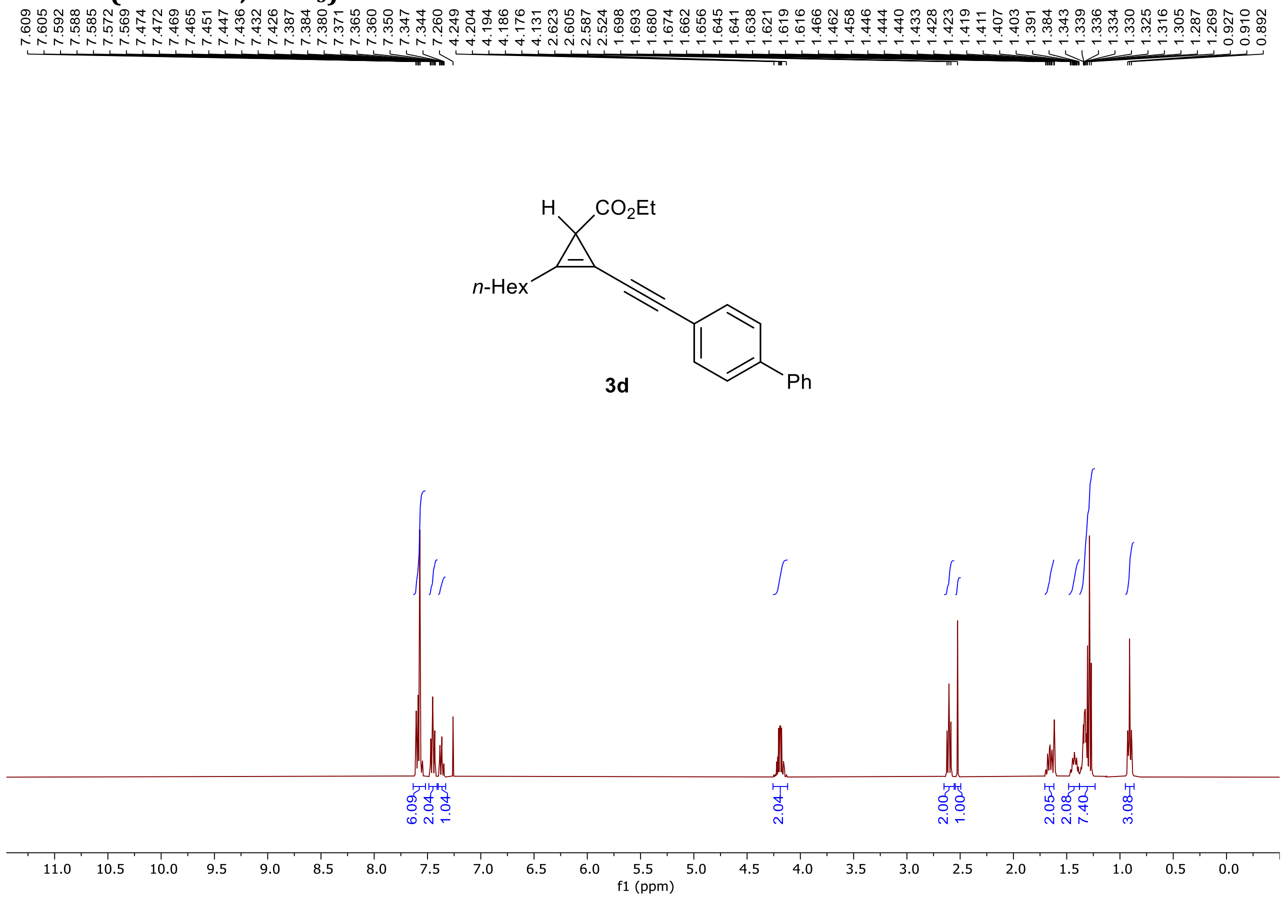

^{13}C NMR (101 MHz, $CDCl_3$) of 3d

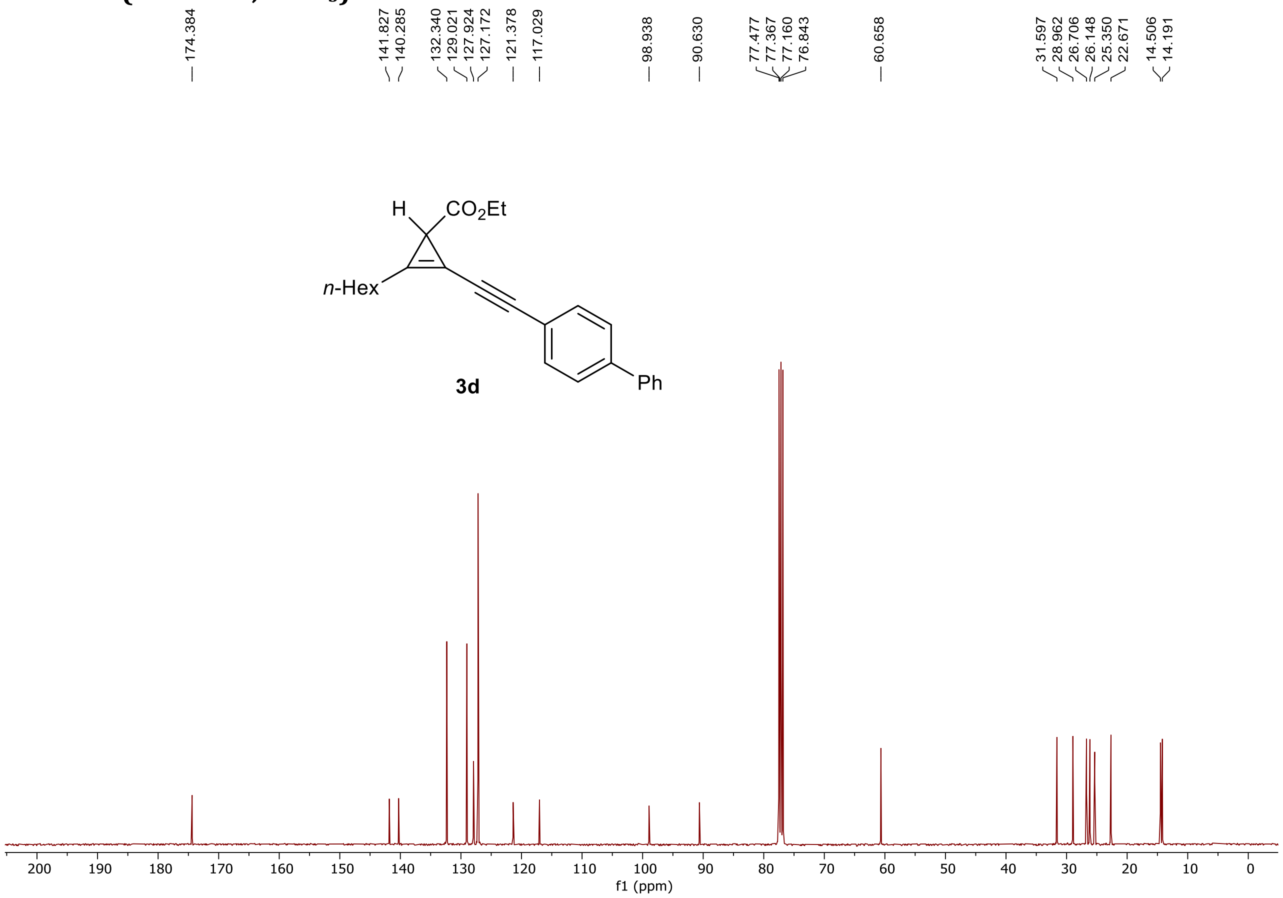

^{1}H NMR (400 MHz, $CDCl_3$) of 3e

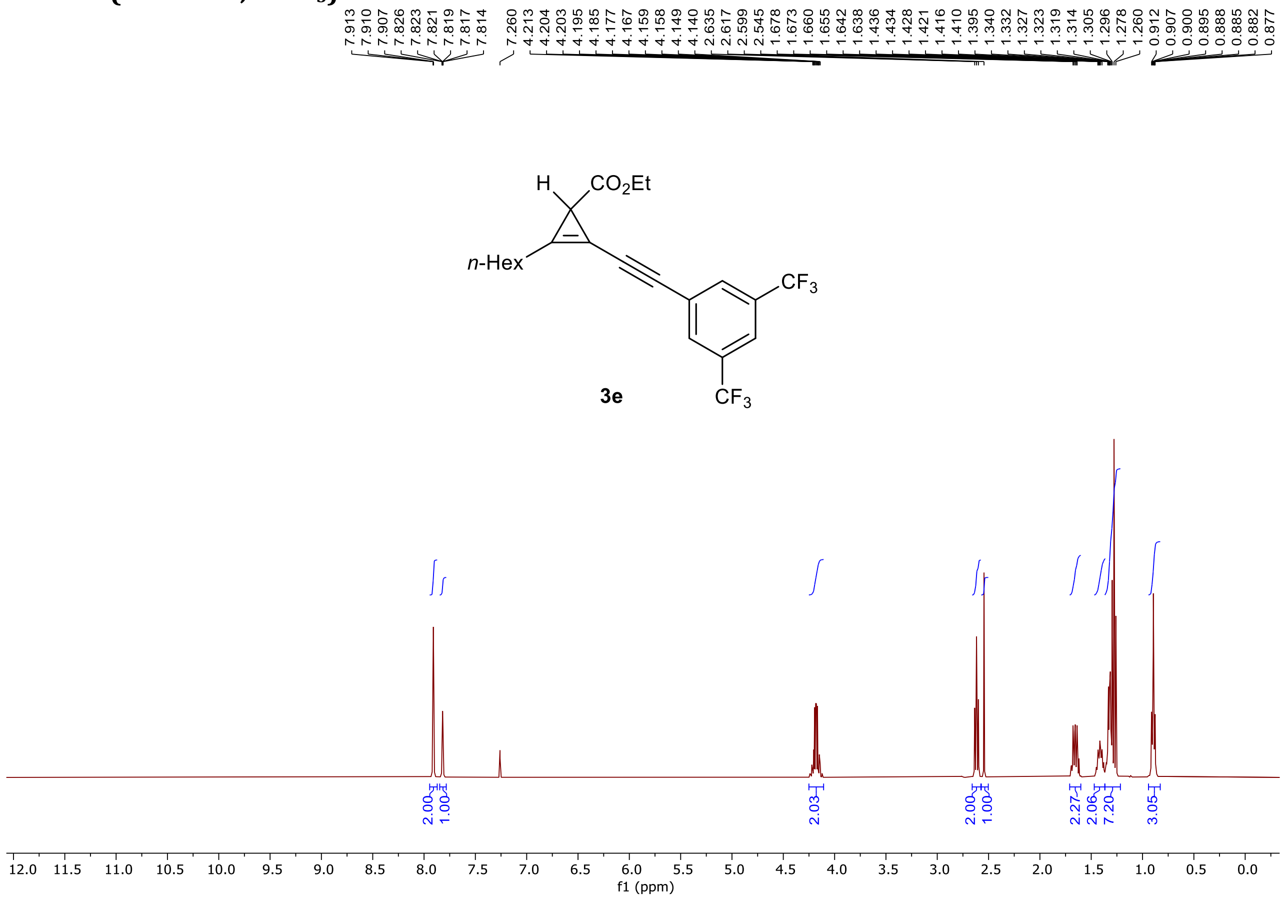

^{13}C NMR (101 MHz, CDCl$_3$) of 3e

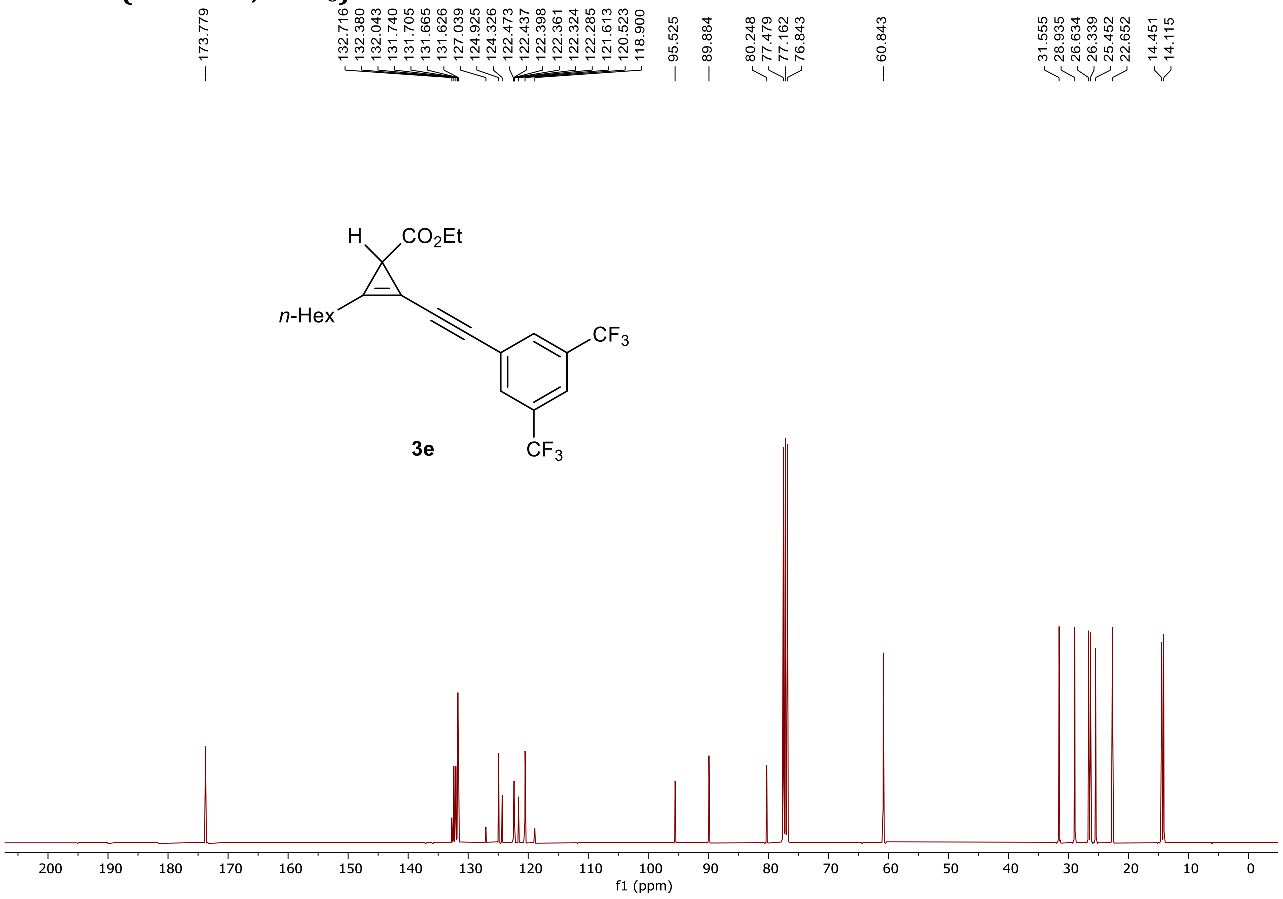

^{19}F NMR (377 MHz, CDCl$_3$) of 3e

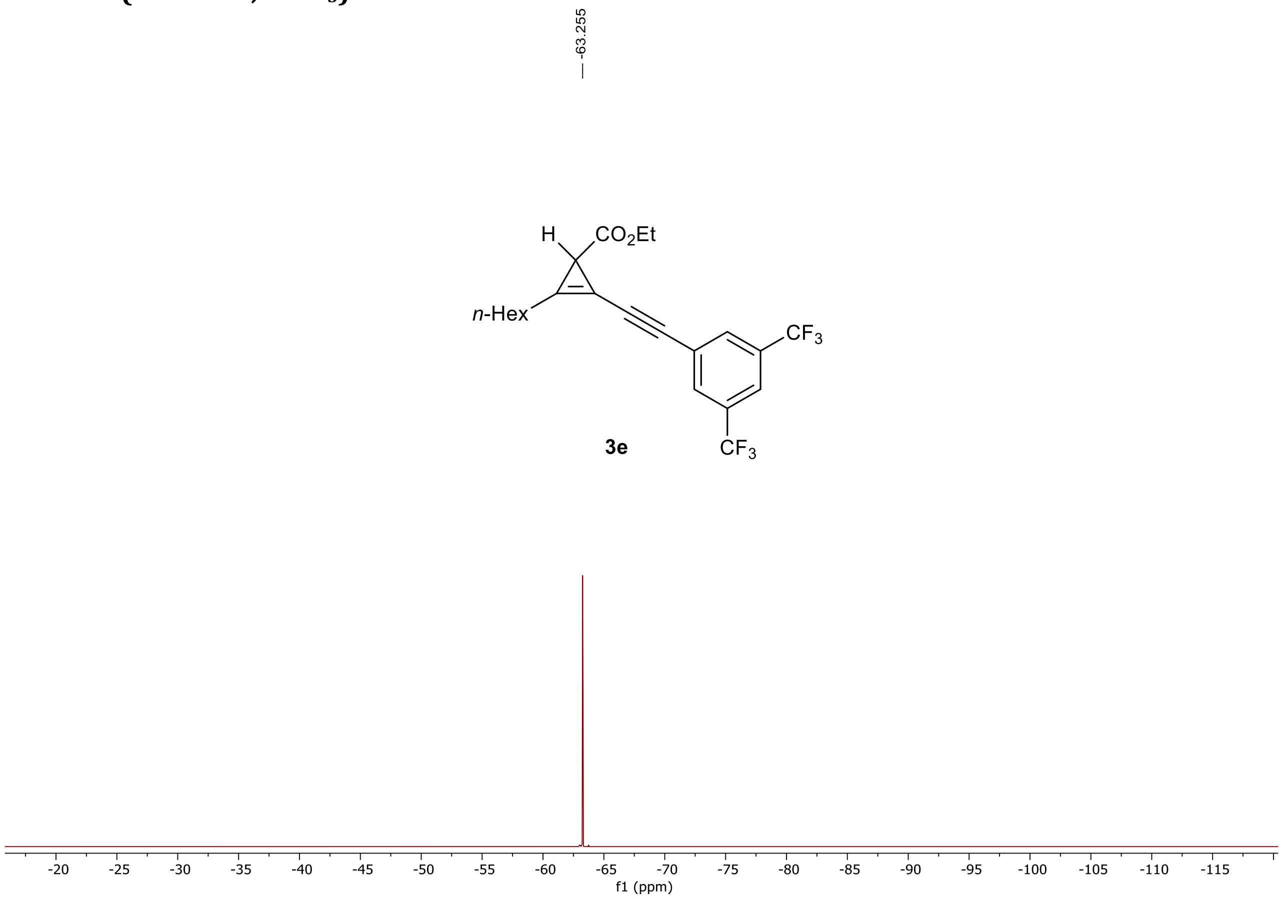

¹H NMR (400 MHz, $CDCl_3$) of 3f

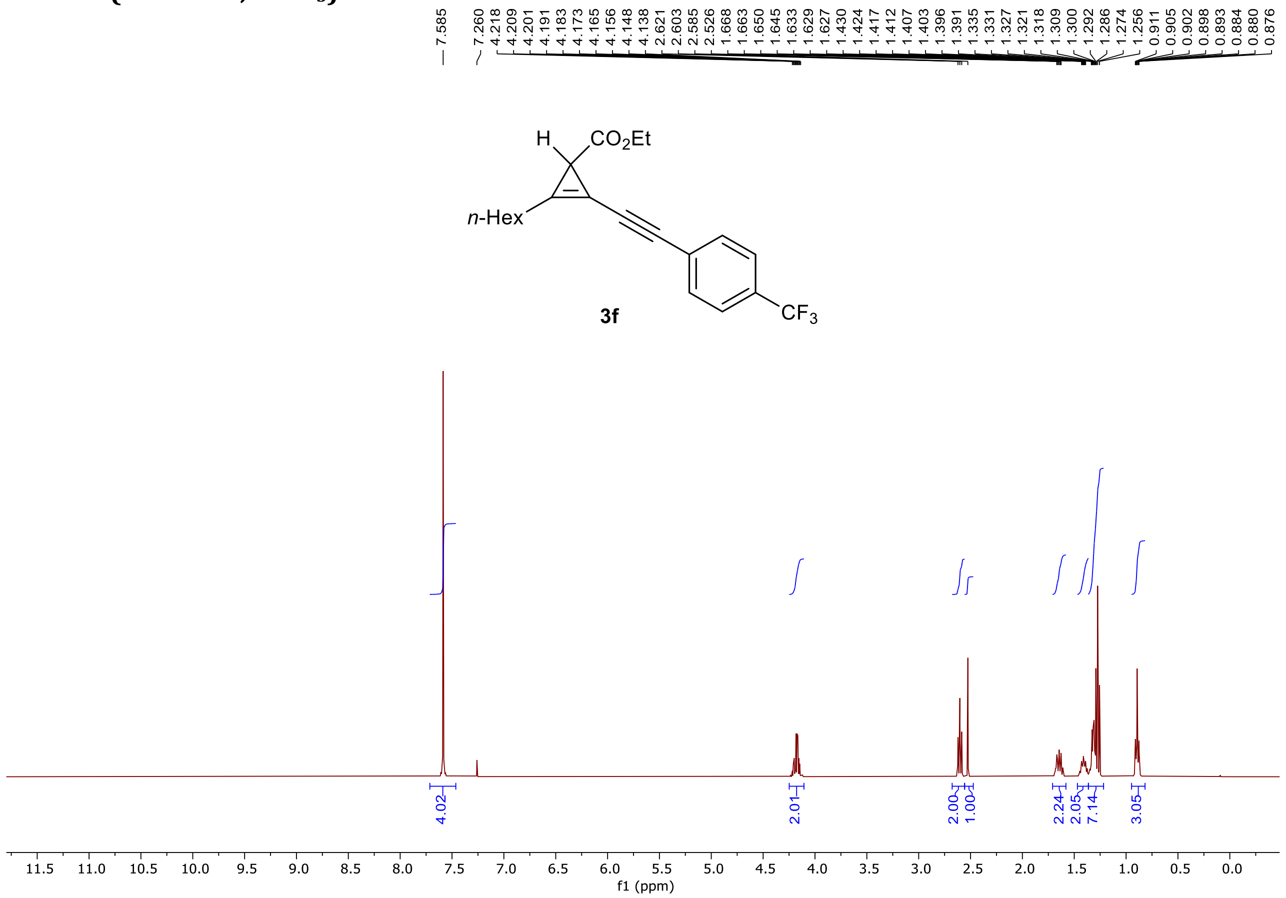

¹³C NMR (101 MHz, $CDCl_3$) of 3f

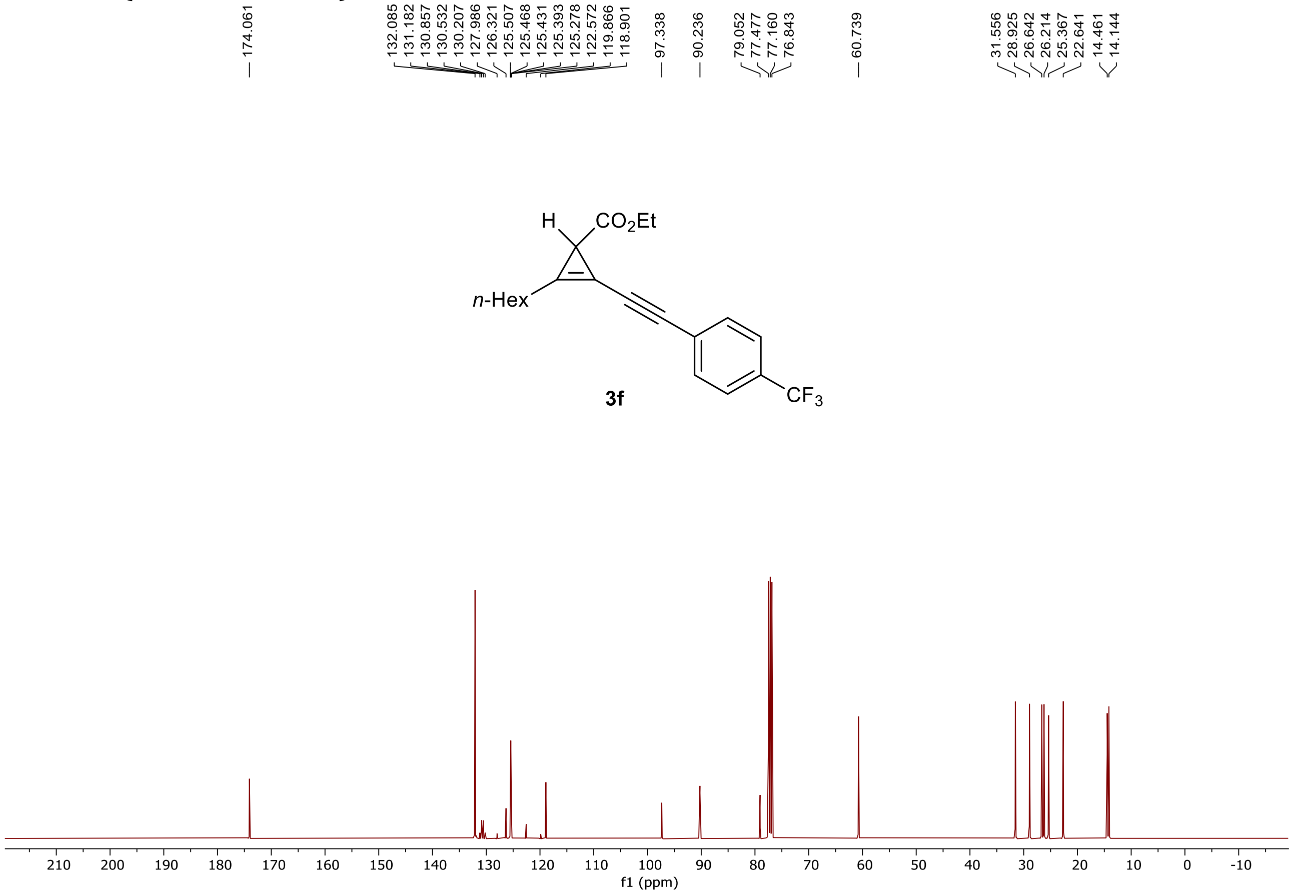

^{19}F NMR (377 MHz, $CDCl_3$) of 3f

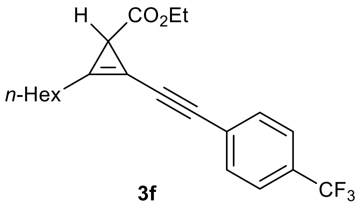

3f

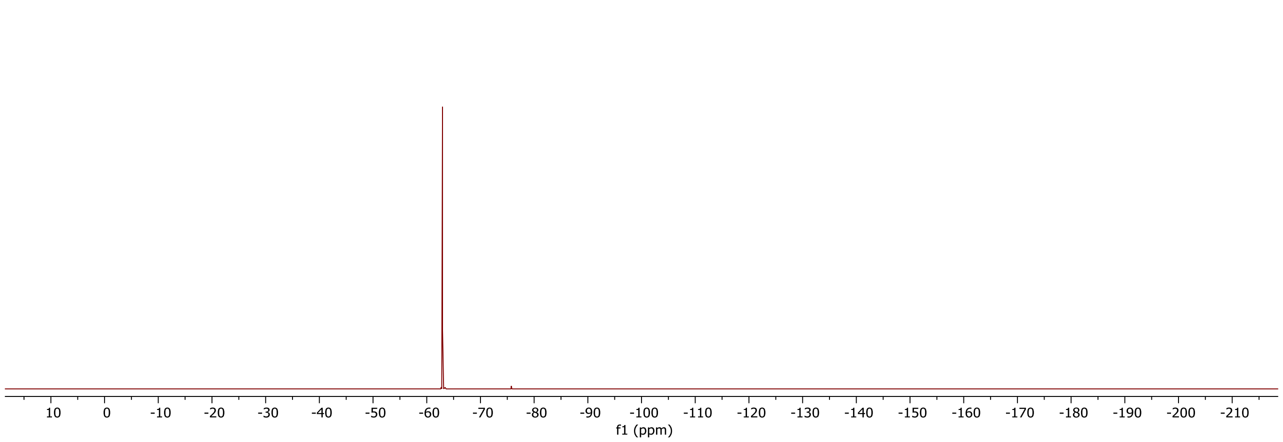

^{1}H NMR (400 MHz, $CDCl_3$) of 3g

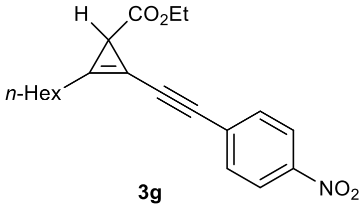

3g

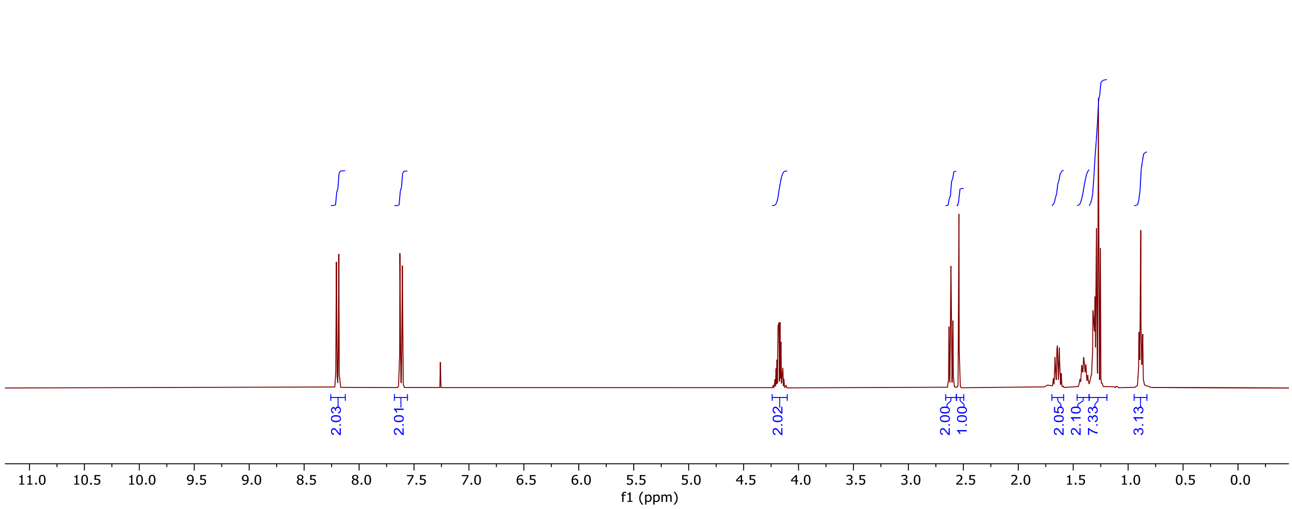

^{13}C NMR (101 MHz, $CDCl_3$) of 3g

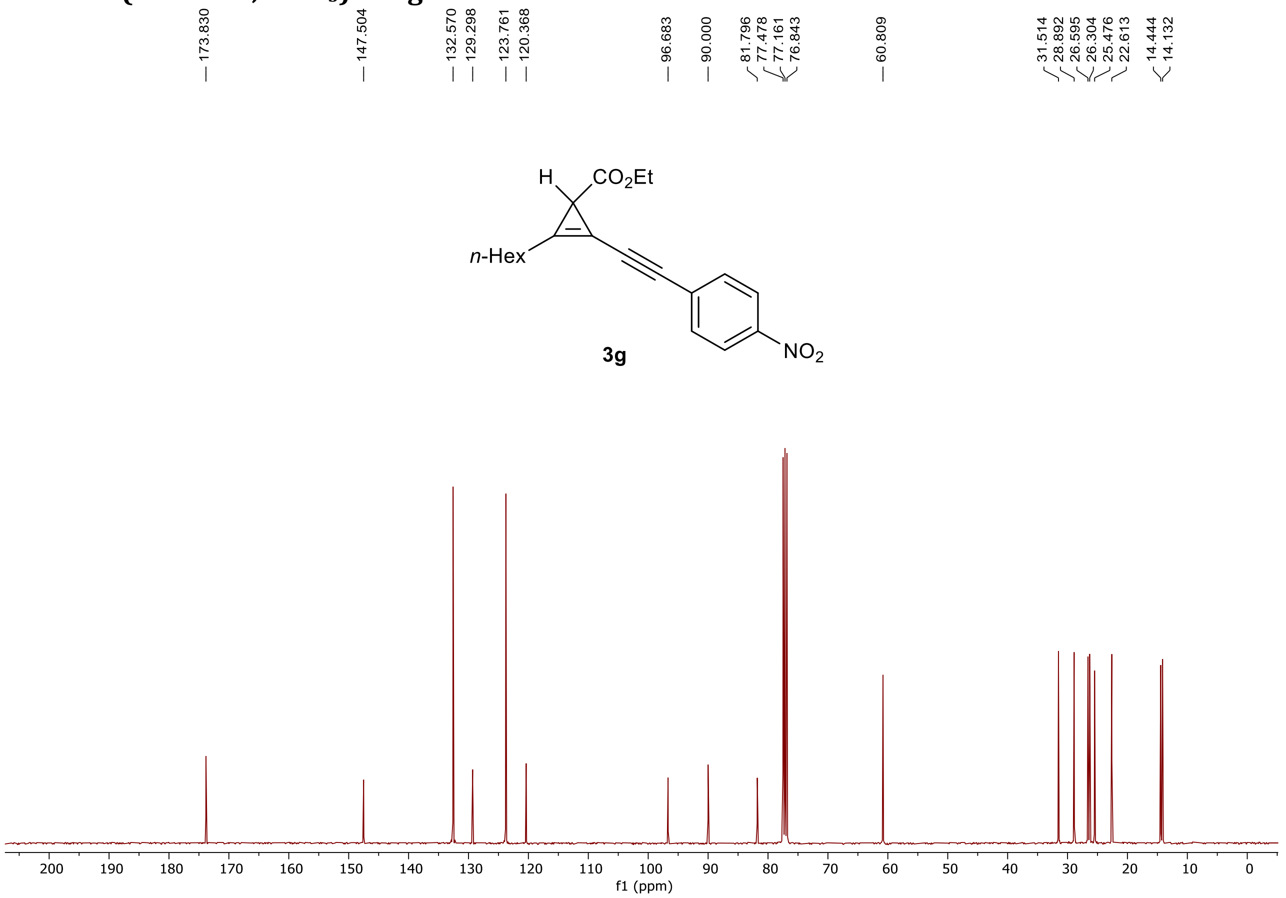

^{1}H NMR (400 MHz, $CDCl_3$) of 3h

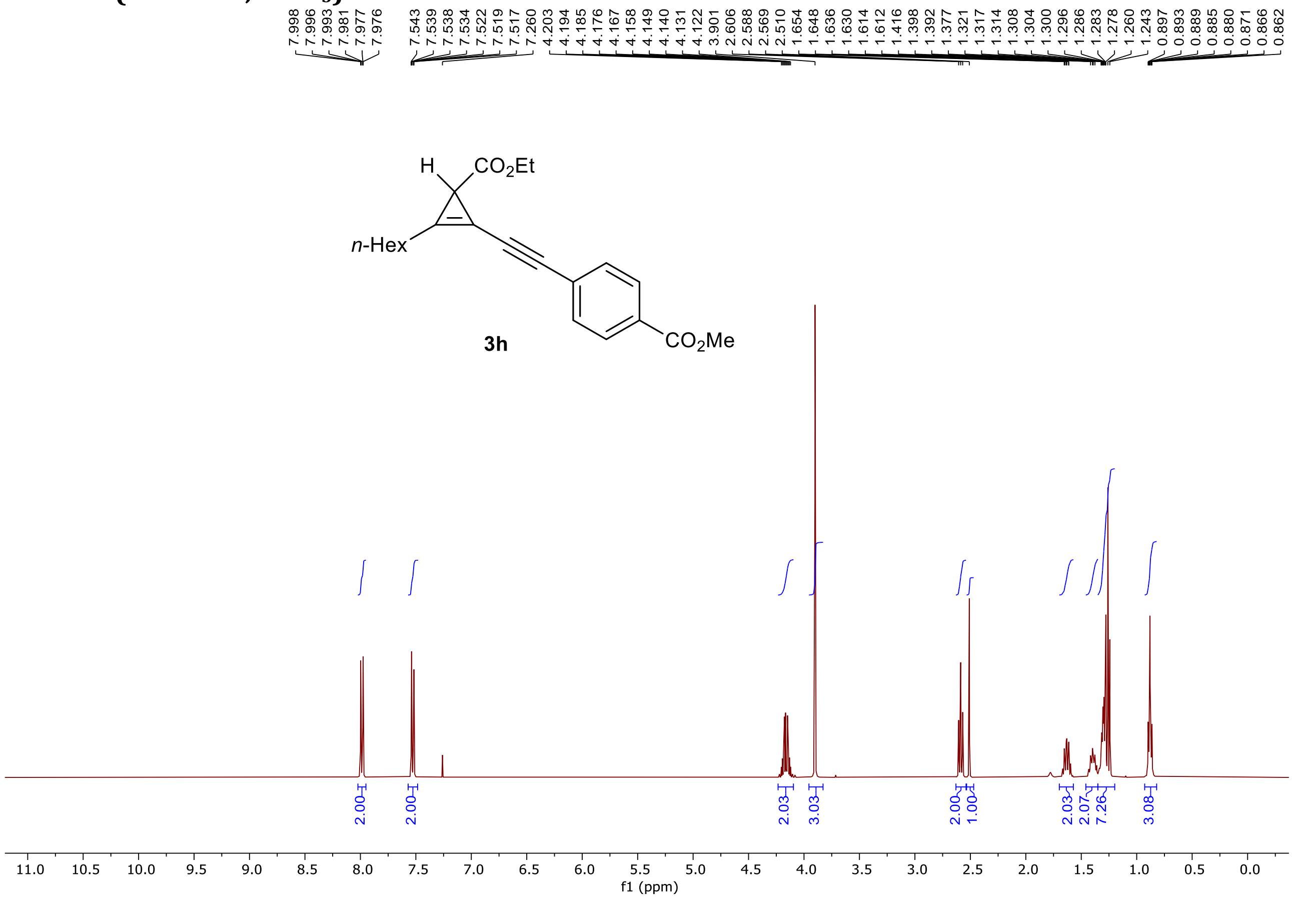

^{13}C NMR (101 MHz, $CDCl_3$) of 3h

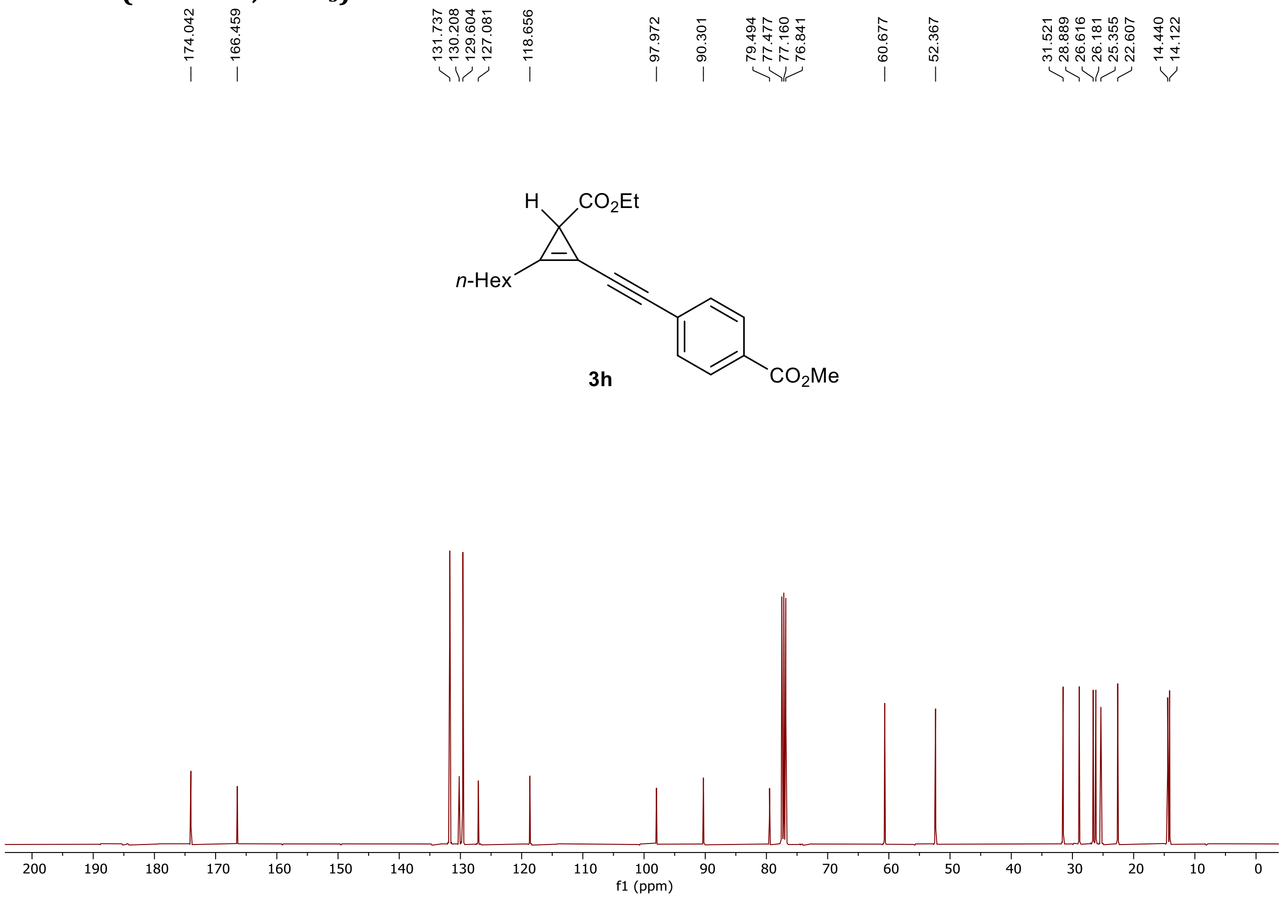

^{1}H NMR (400 MHz, $CDCl_3$) of 3i

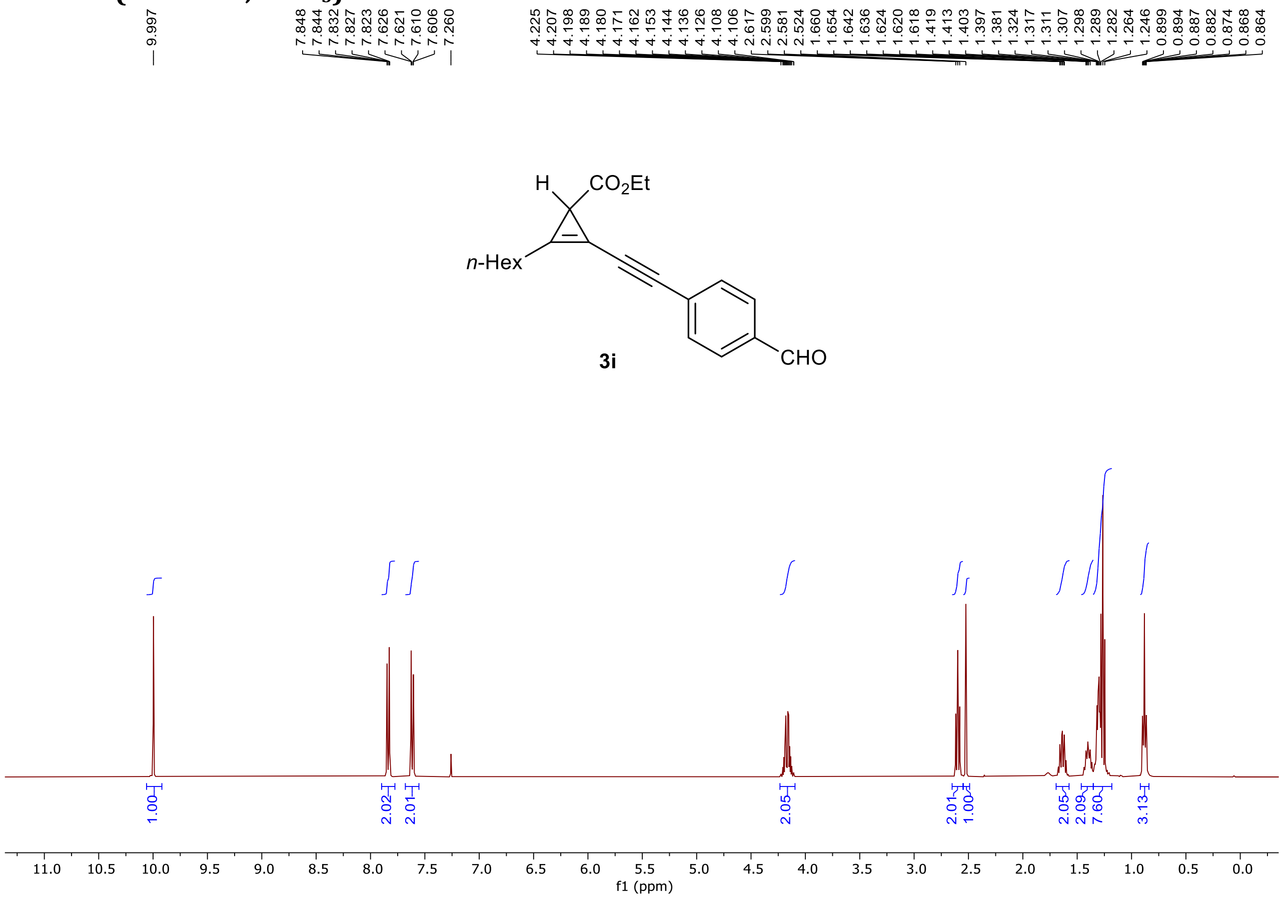

^{13}C NMR (101 MHz, $CDCl_3$) of 3i

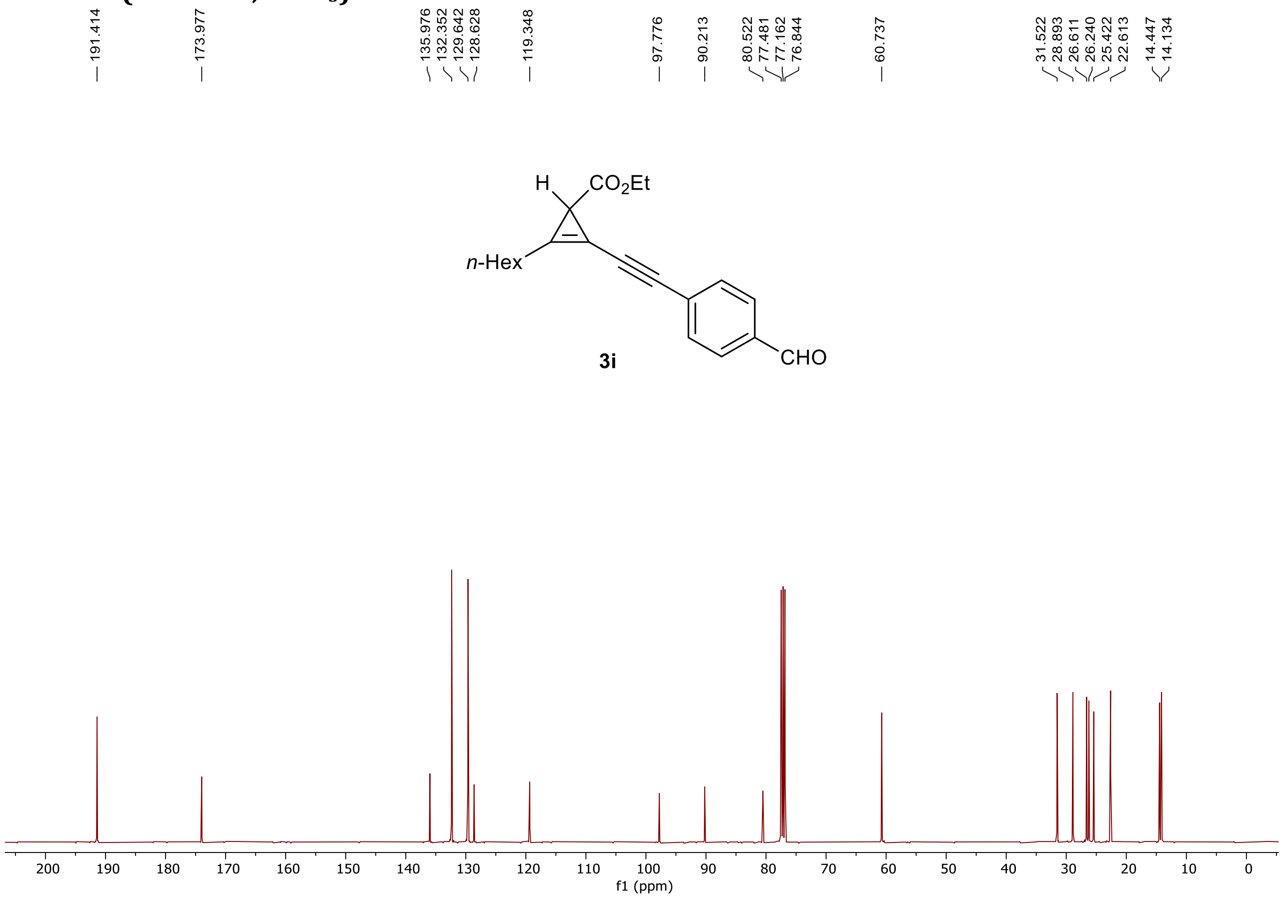

^{1}H NMR (400 MHz, $CDCl_3$) of 3j

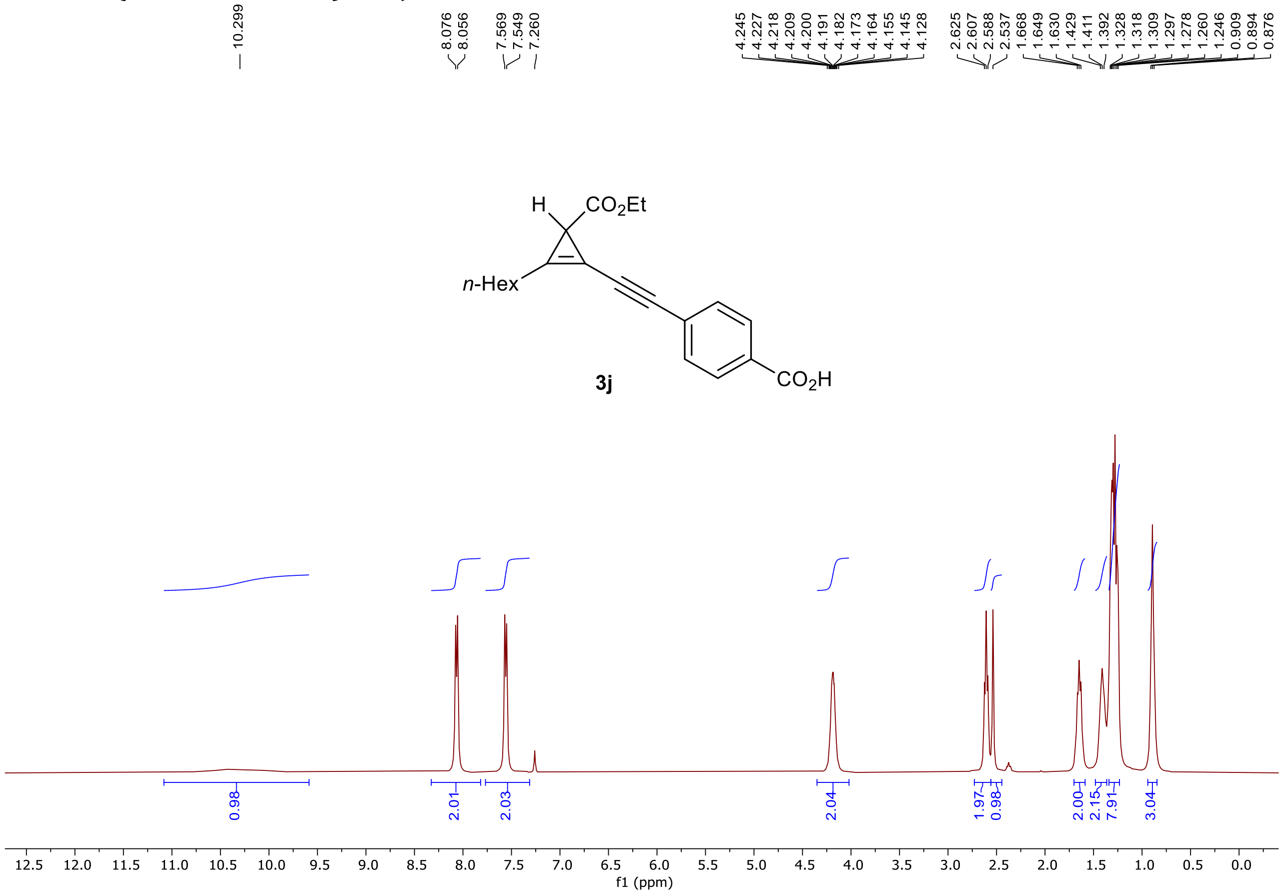

^{13}C NMR (101 MHz, $CDCl_3$) of 3j

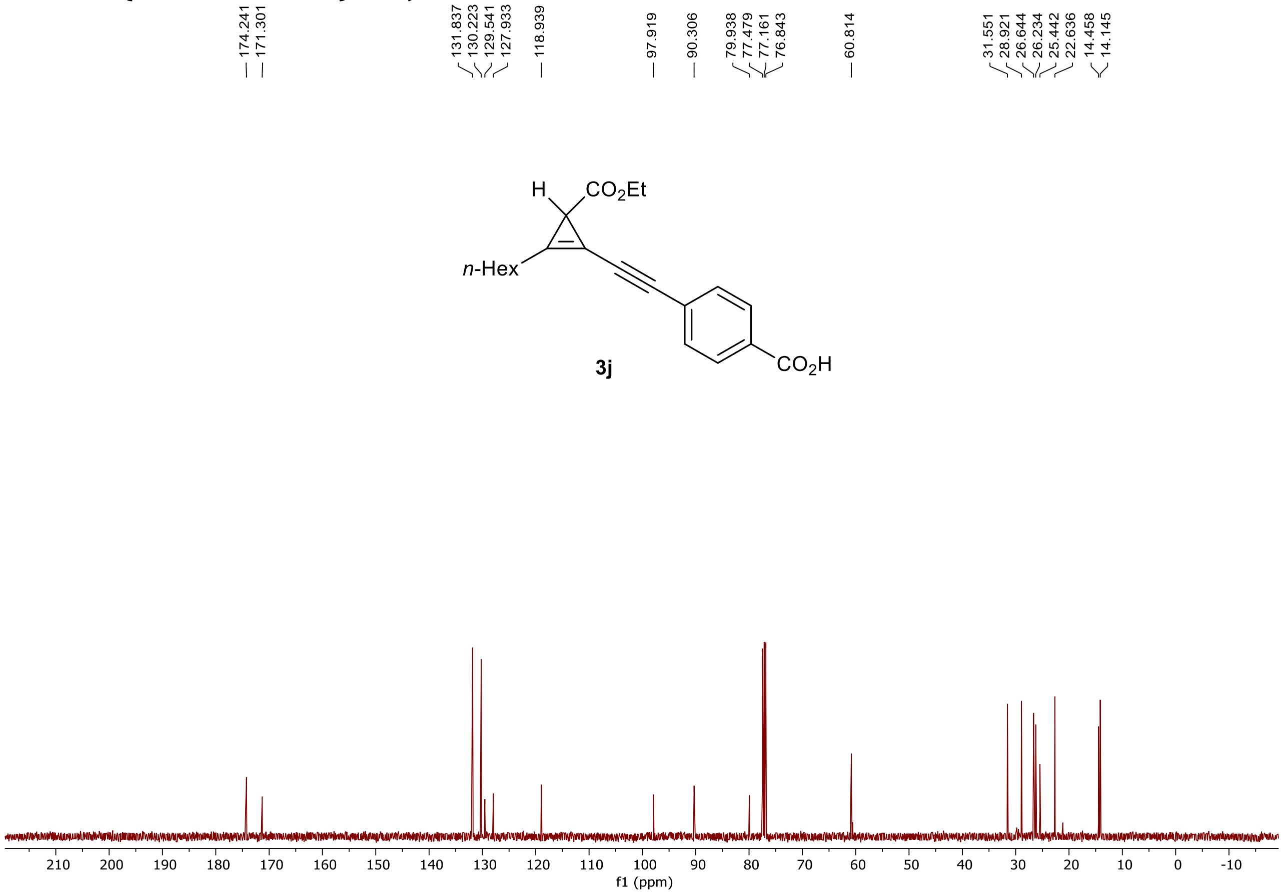

^{1}H NMR (400 MHz, $CDCl_3$) of 3k

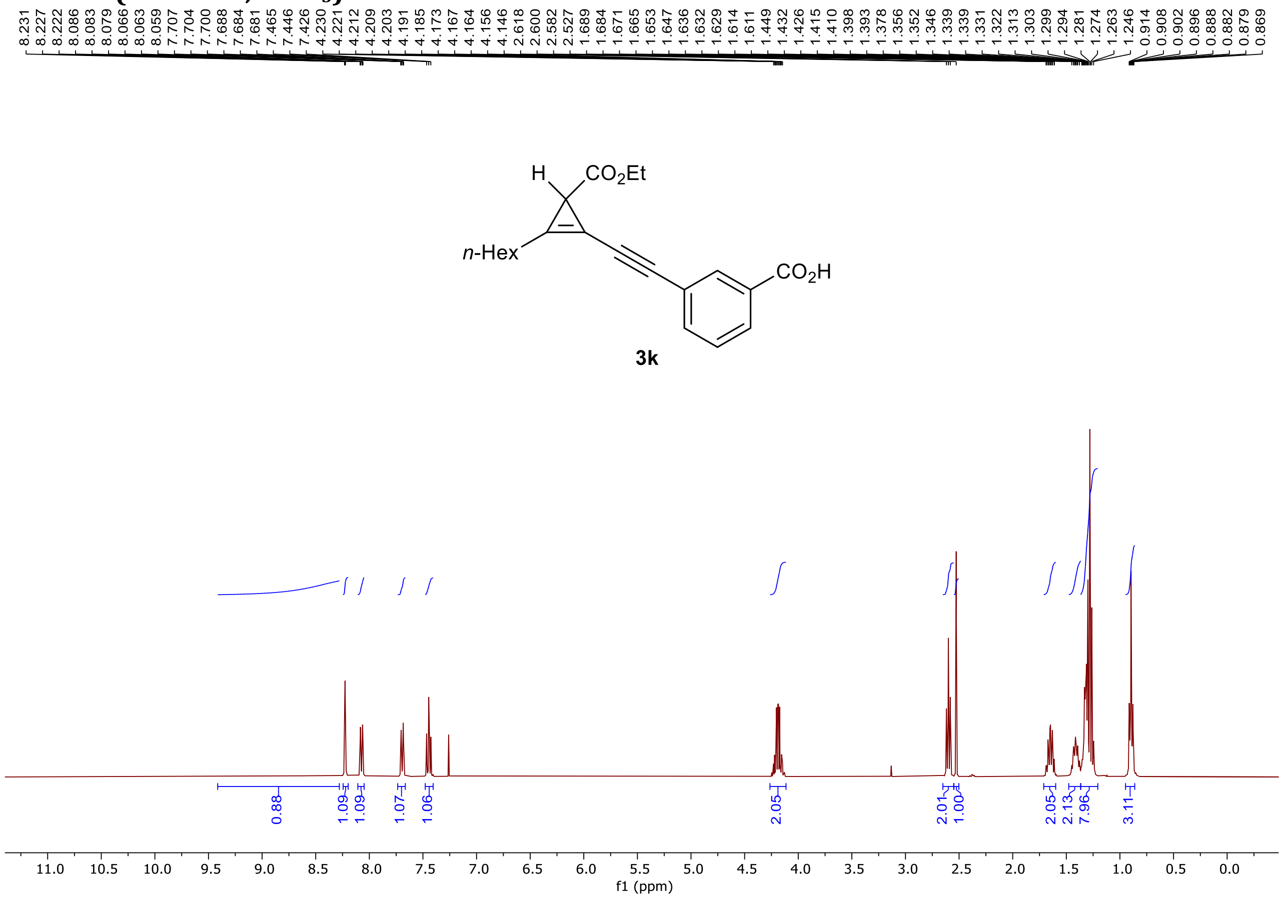

^{13}C NMR (101 MHz, $CDCl_3$) of 3k

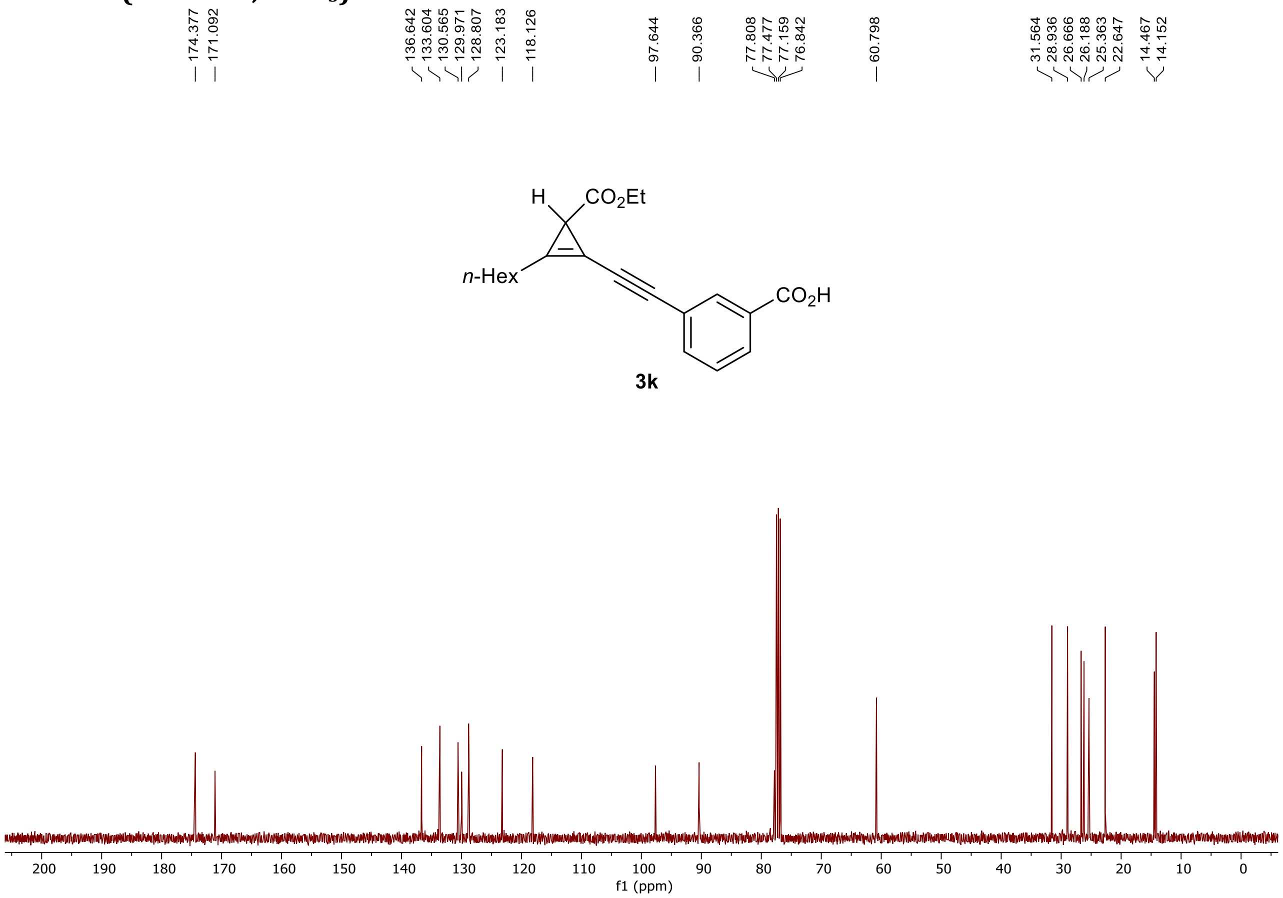

^{1}H NMR (400 MHz, $CDCl_3$) of 3l

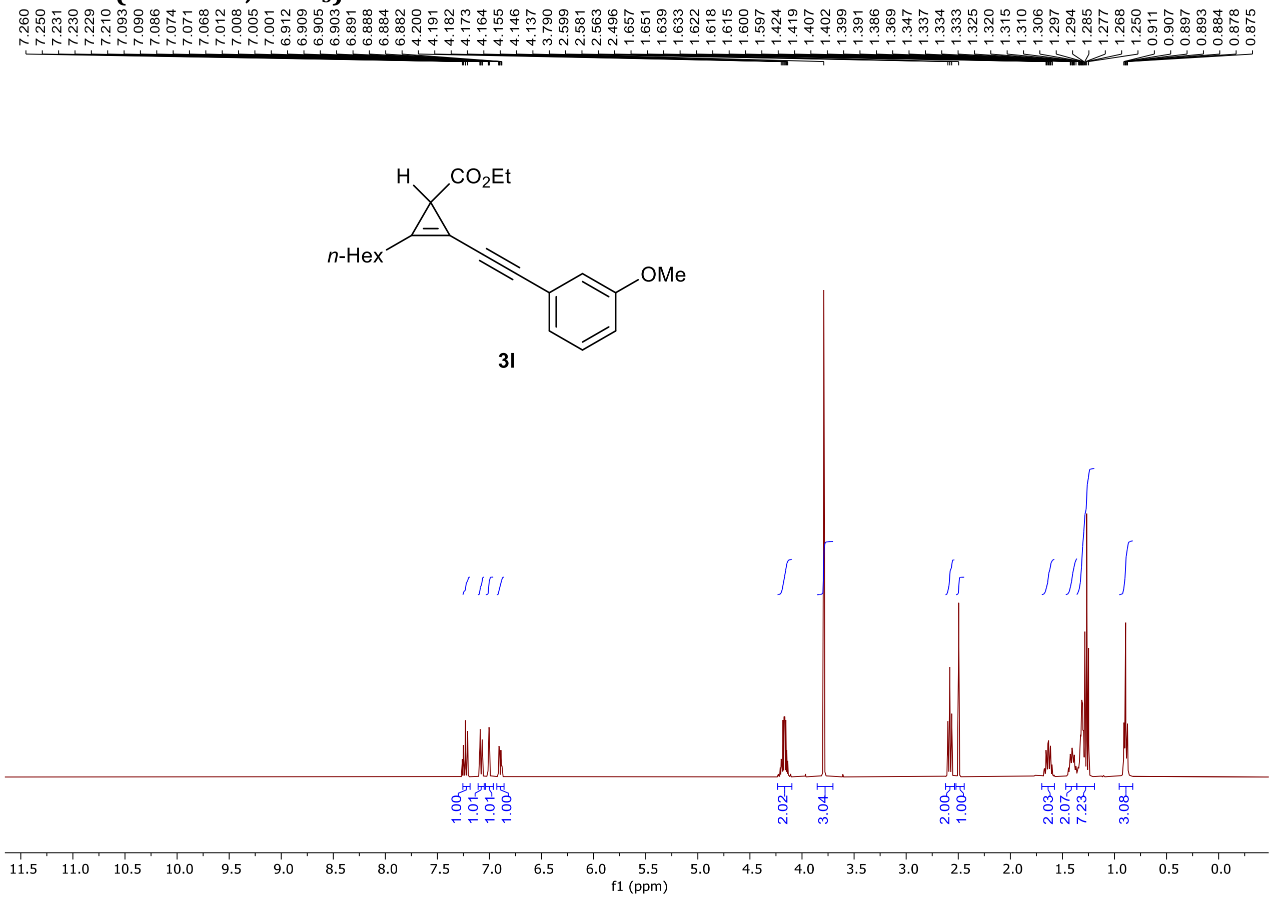

^{13}C NMR (101 MHz, $CDCl_3$) of 3l

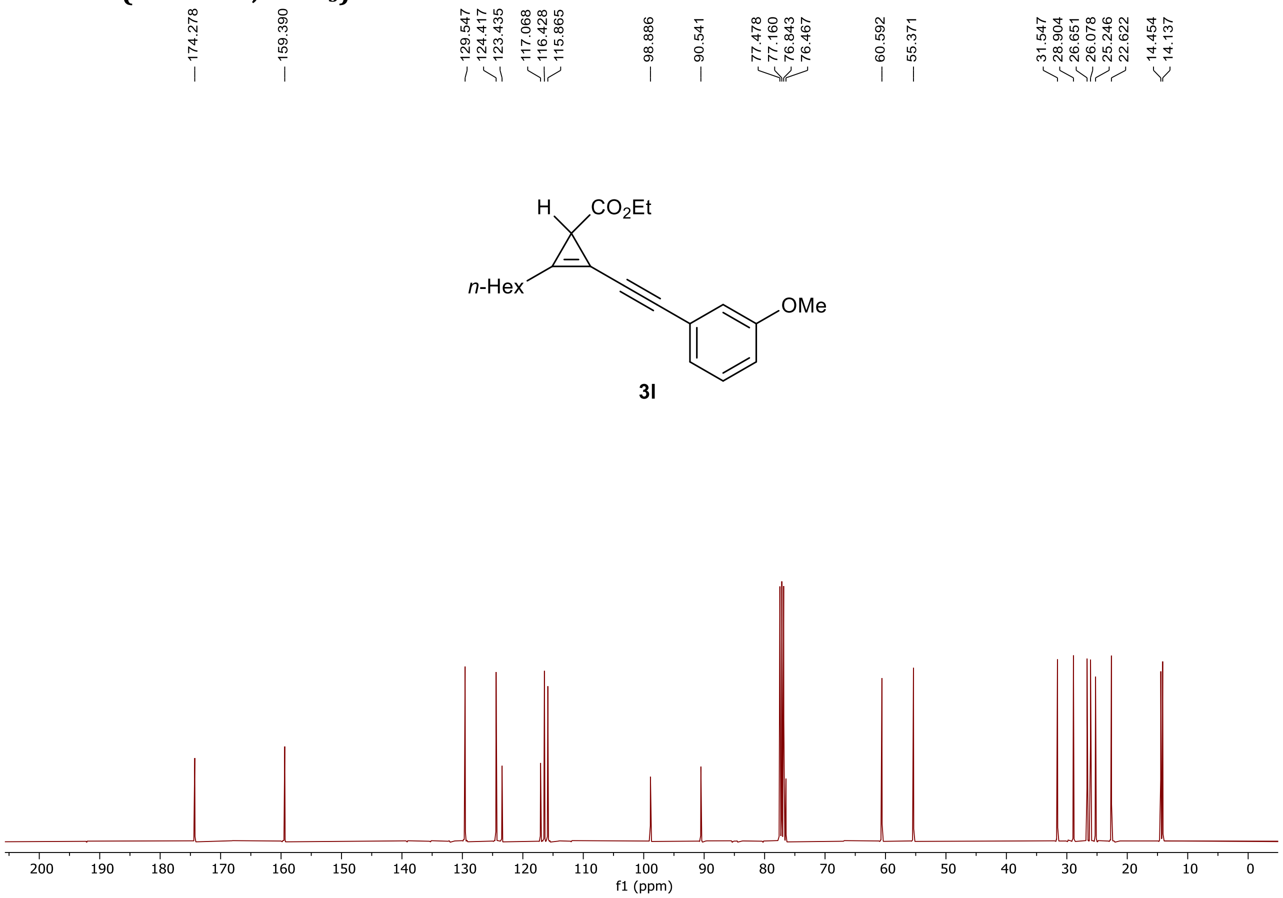

^{1}H NMR (400 MHz, $CDCl_3$) of 3m

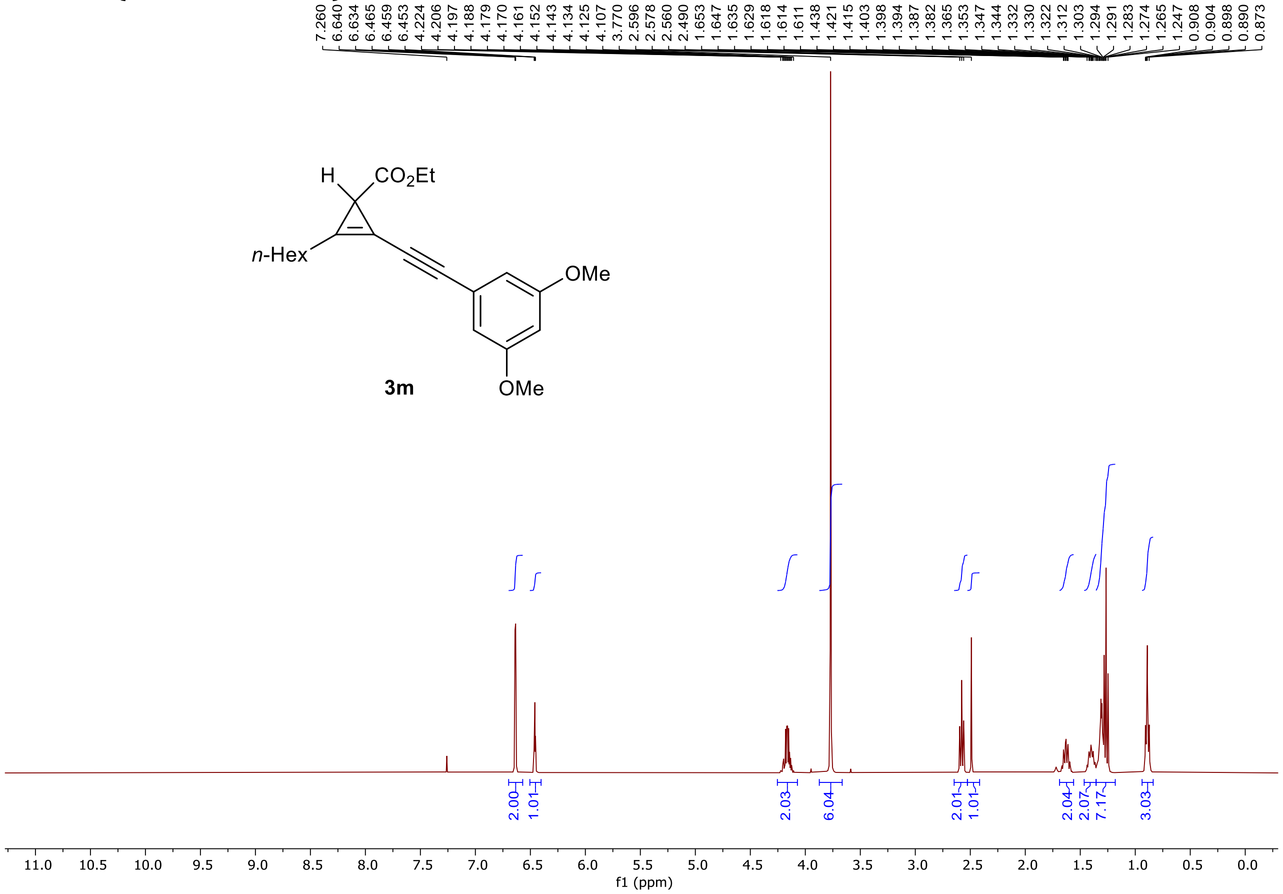

^{13}C NMR (101 MHz, $CDCl_3$) of 3m

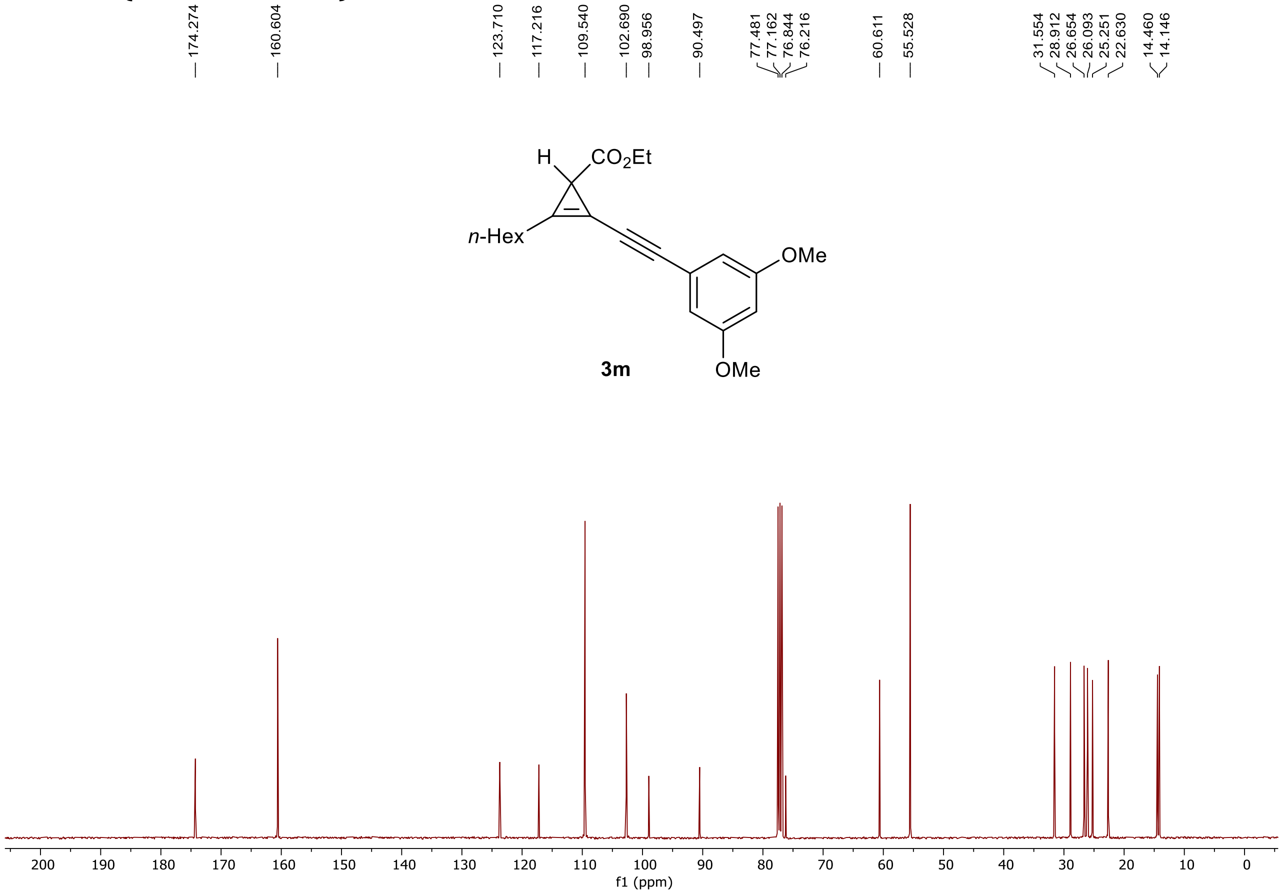

^{1}H NMR (400 MHz, $CDCl_3$) of 3n

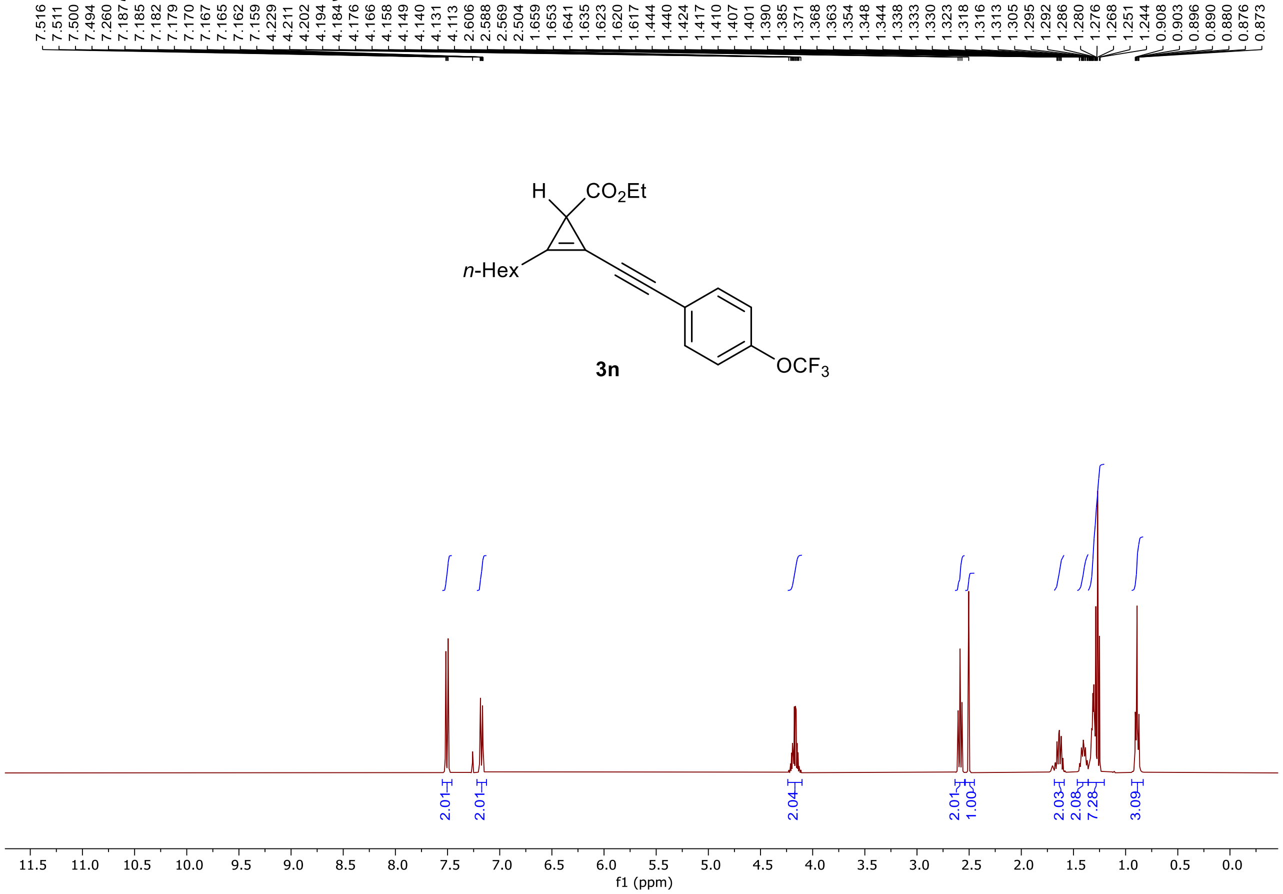

^{13}C NMR (101 MHz, $CDCl_3$) of 3n

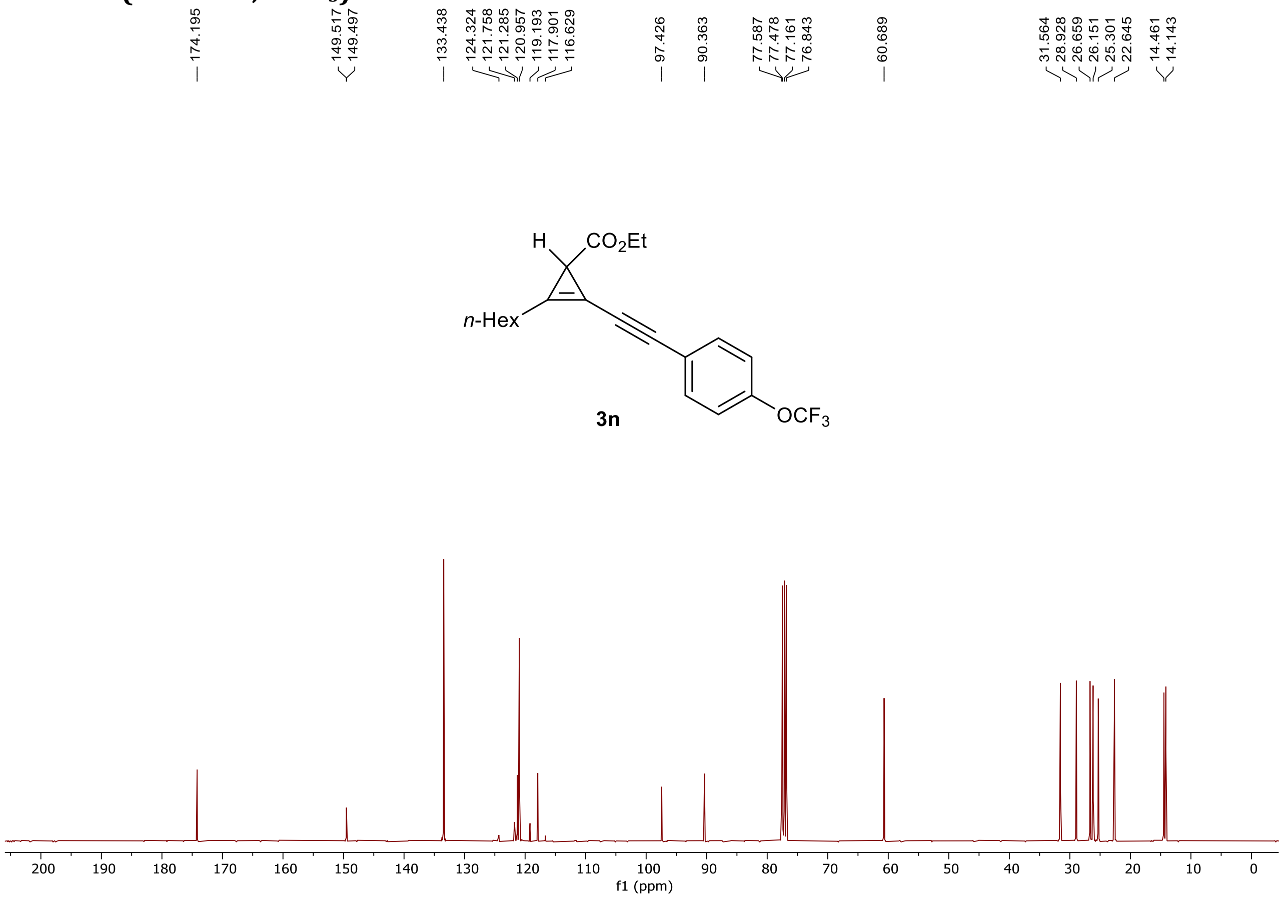

^{19}F NMR (377 MHz, $CDCl_3$) of 3n

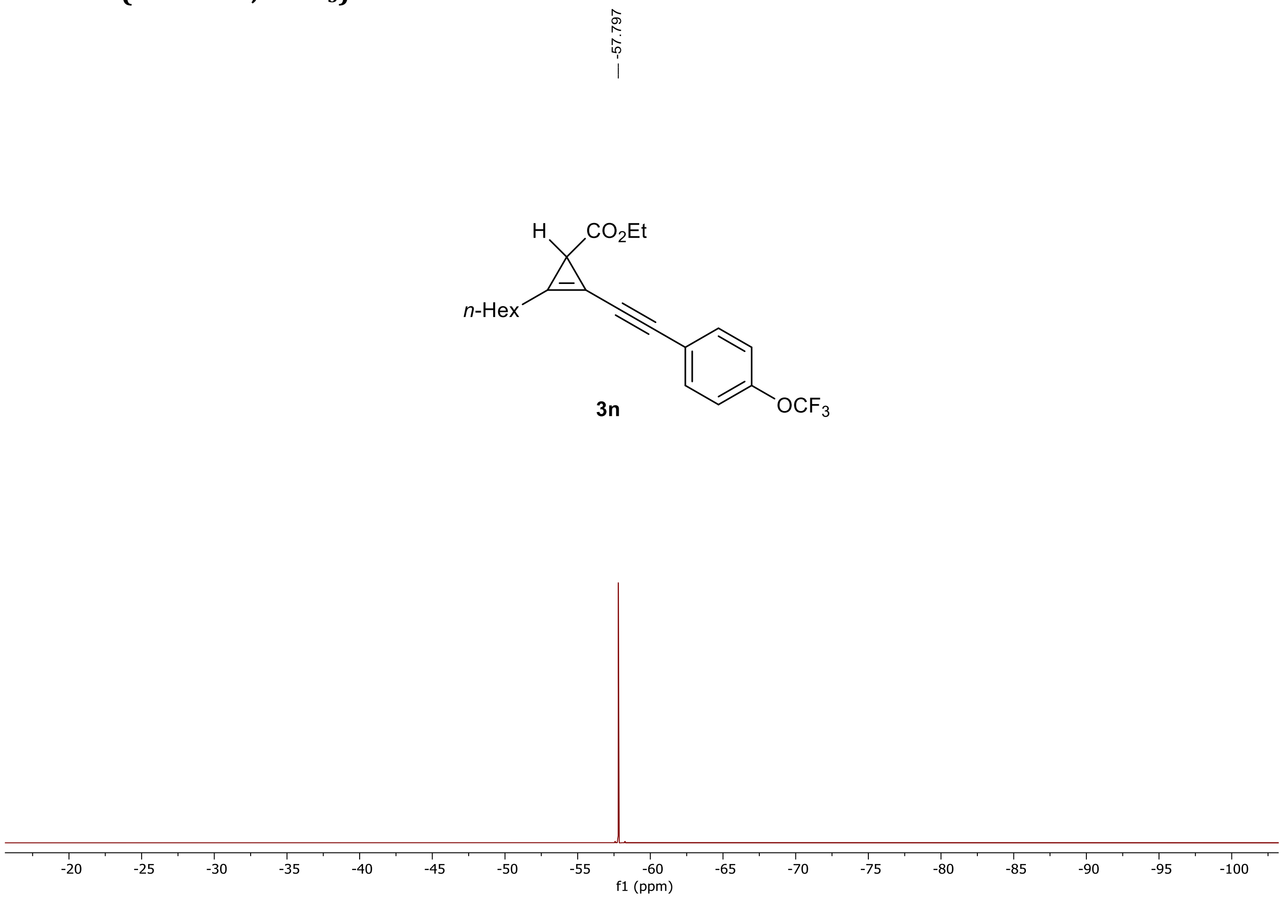

^{1}H NMR (400 MHz, $CDCl_3$) of 3o

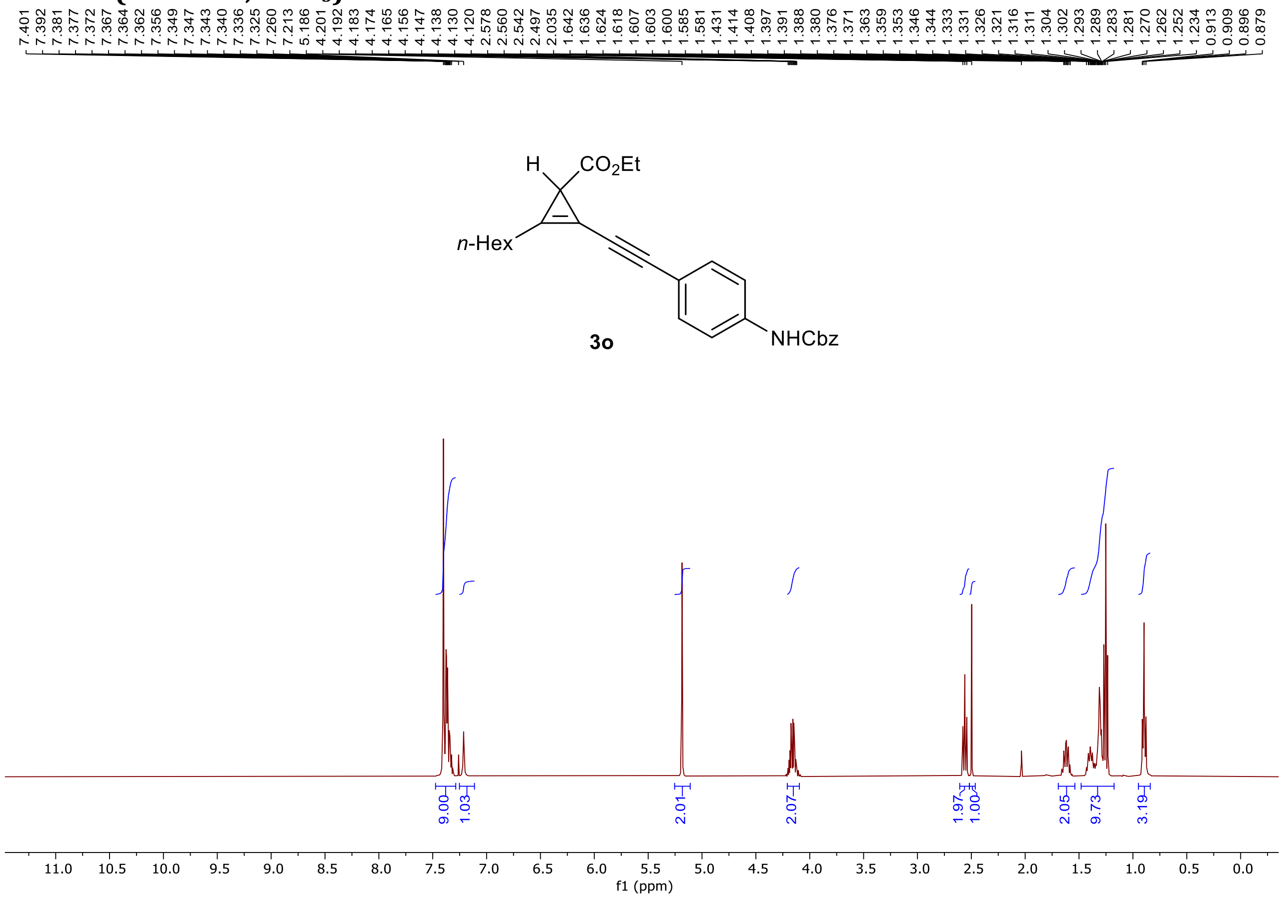

^{13}C NMR (101 MHz, $CDCl_3$) of 3o

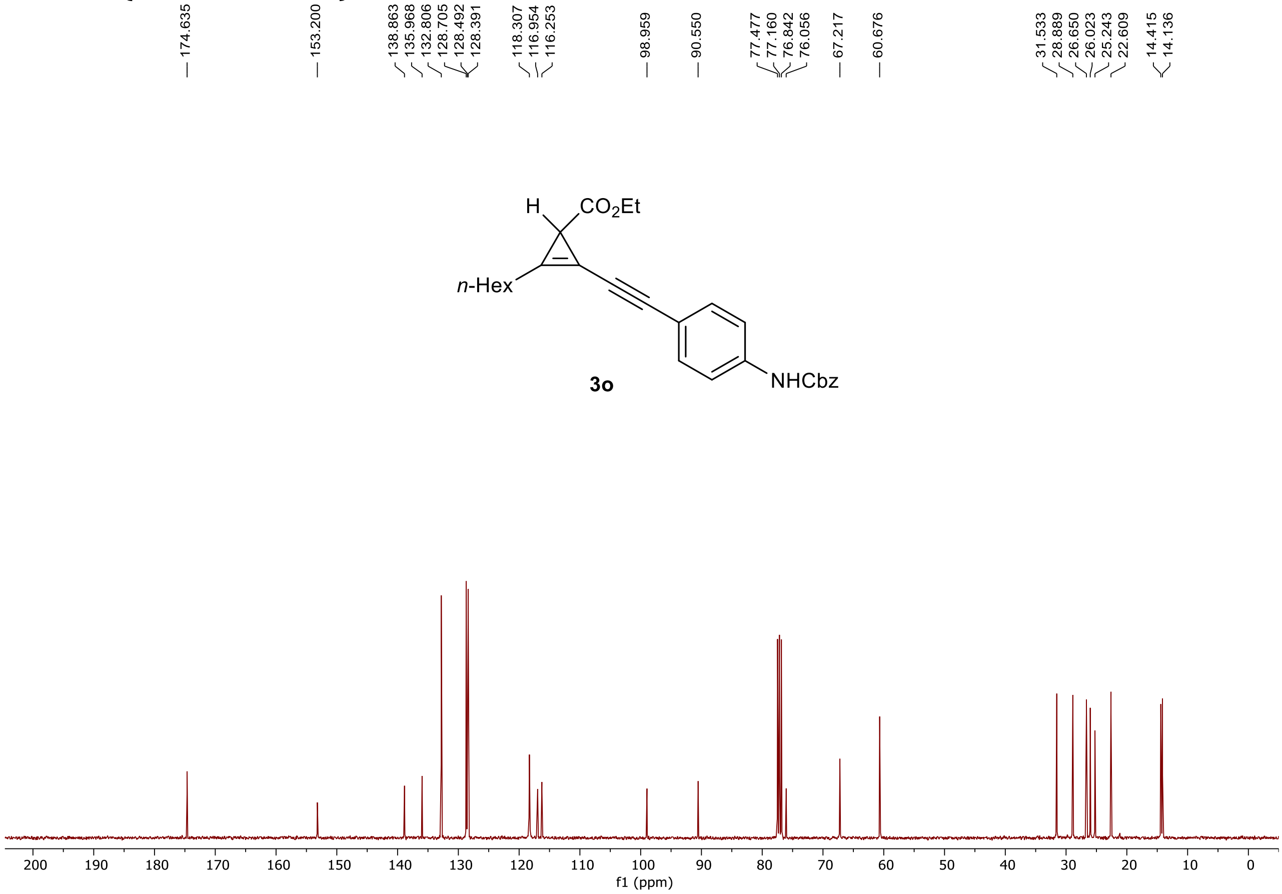

^{1}H NMR (400 MHz, $CDCl_3$) of 3p

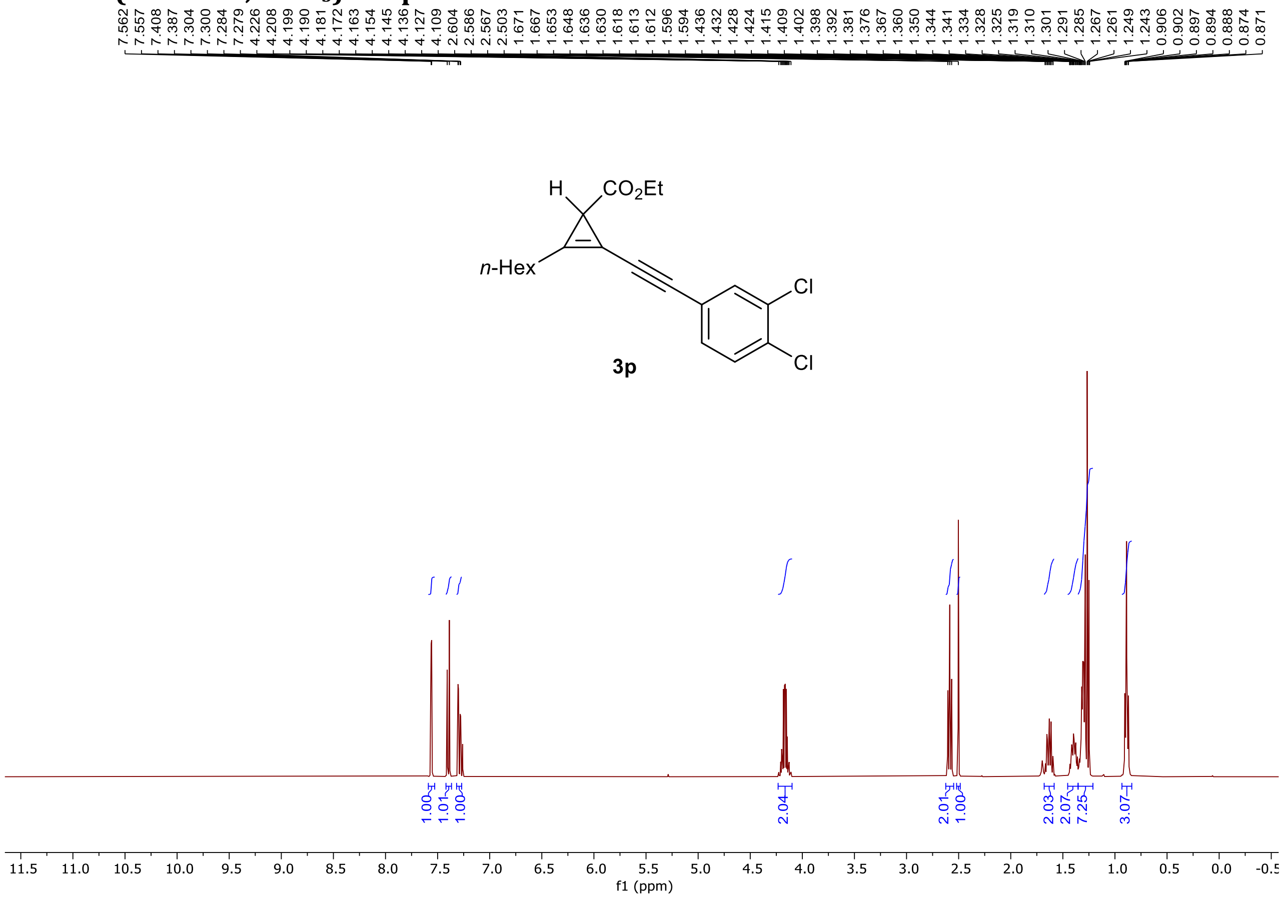

^{13}C NMR (101 MHz, $CDCl_3$) of 3p

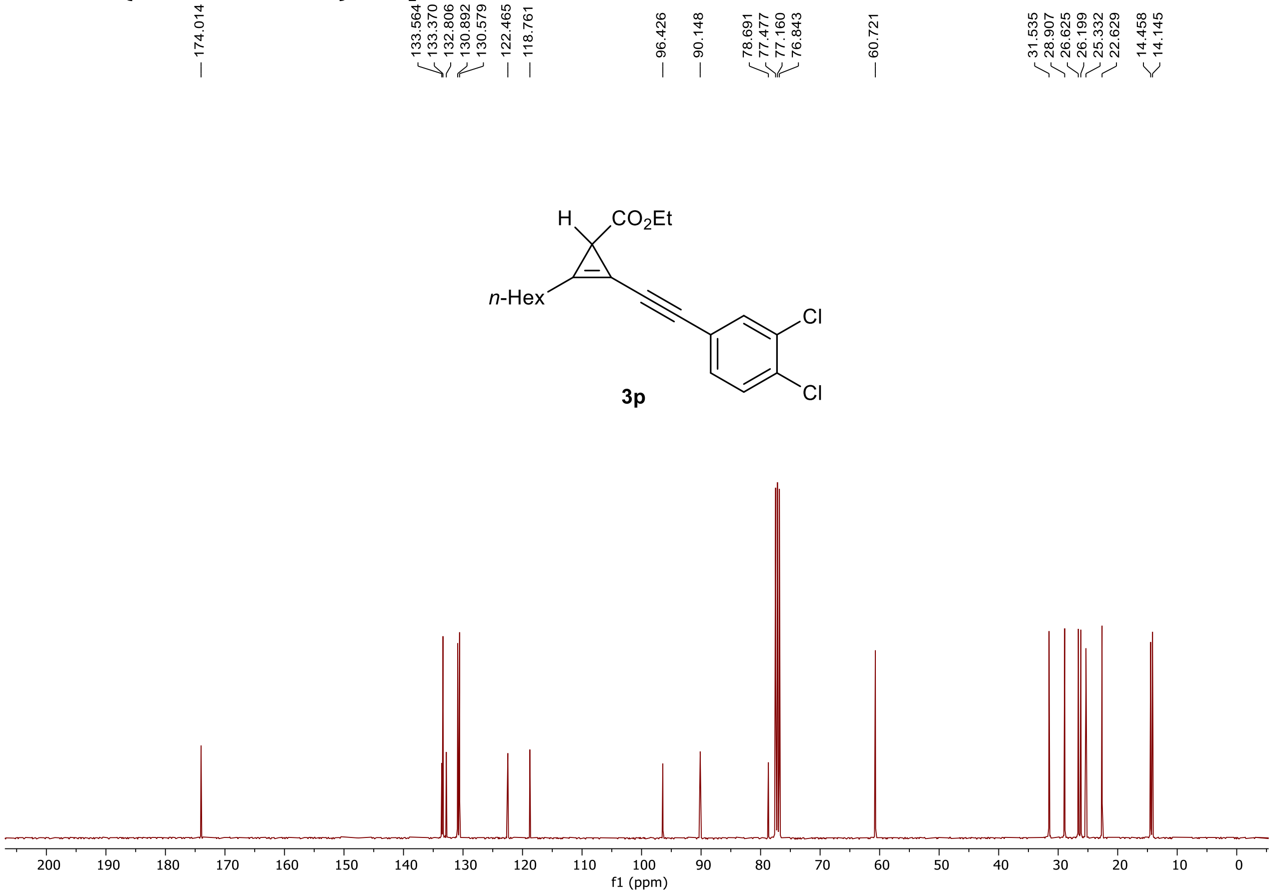

^{1}H NMR (400 MHz, $CDCl_3$) of 3q

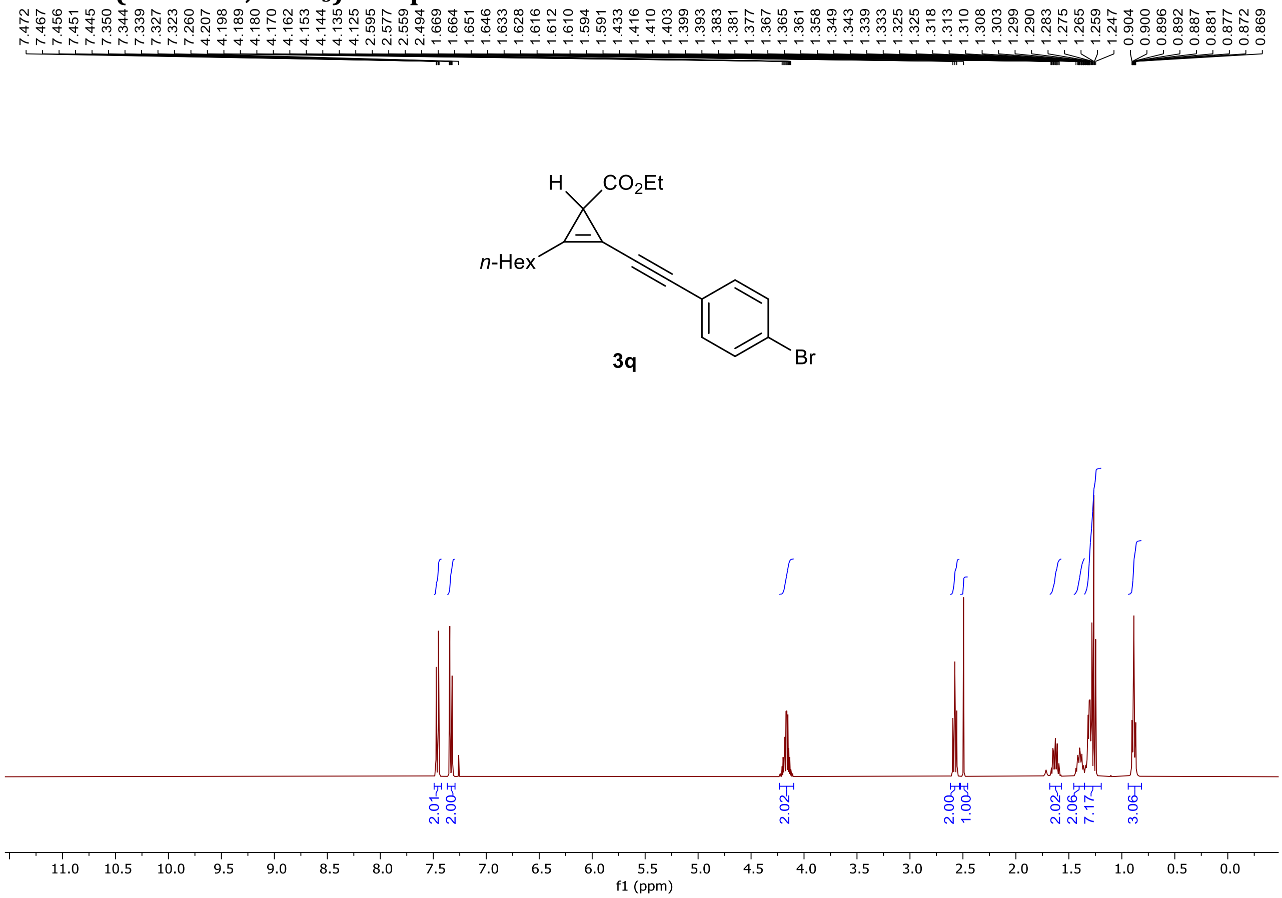

^{13}C NMR (101 MHz, $CDCl_3$) of 3q

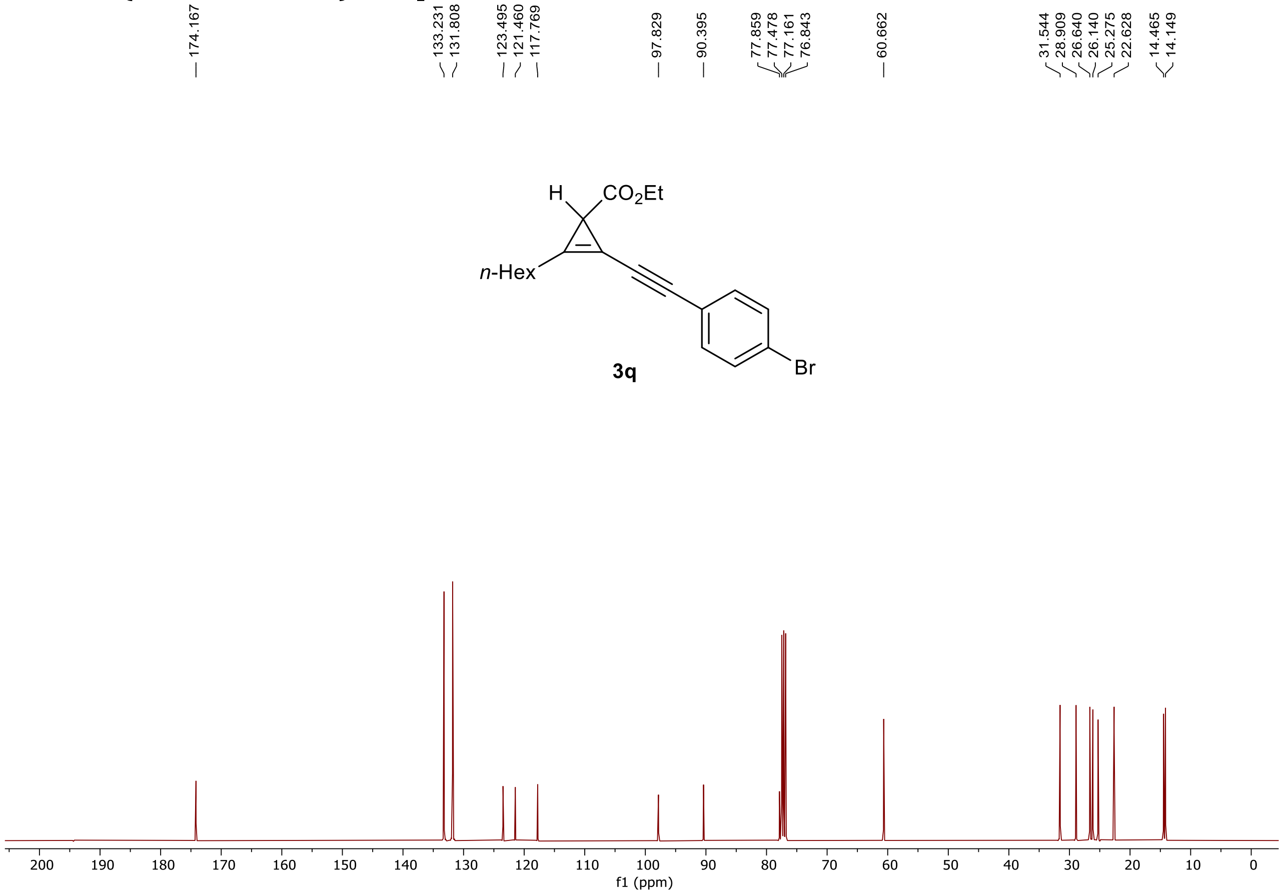

¹H NMR (400 MHz, CDCl$_3$) of 3r

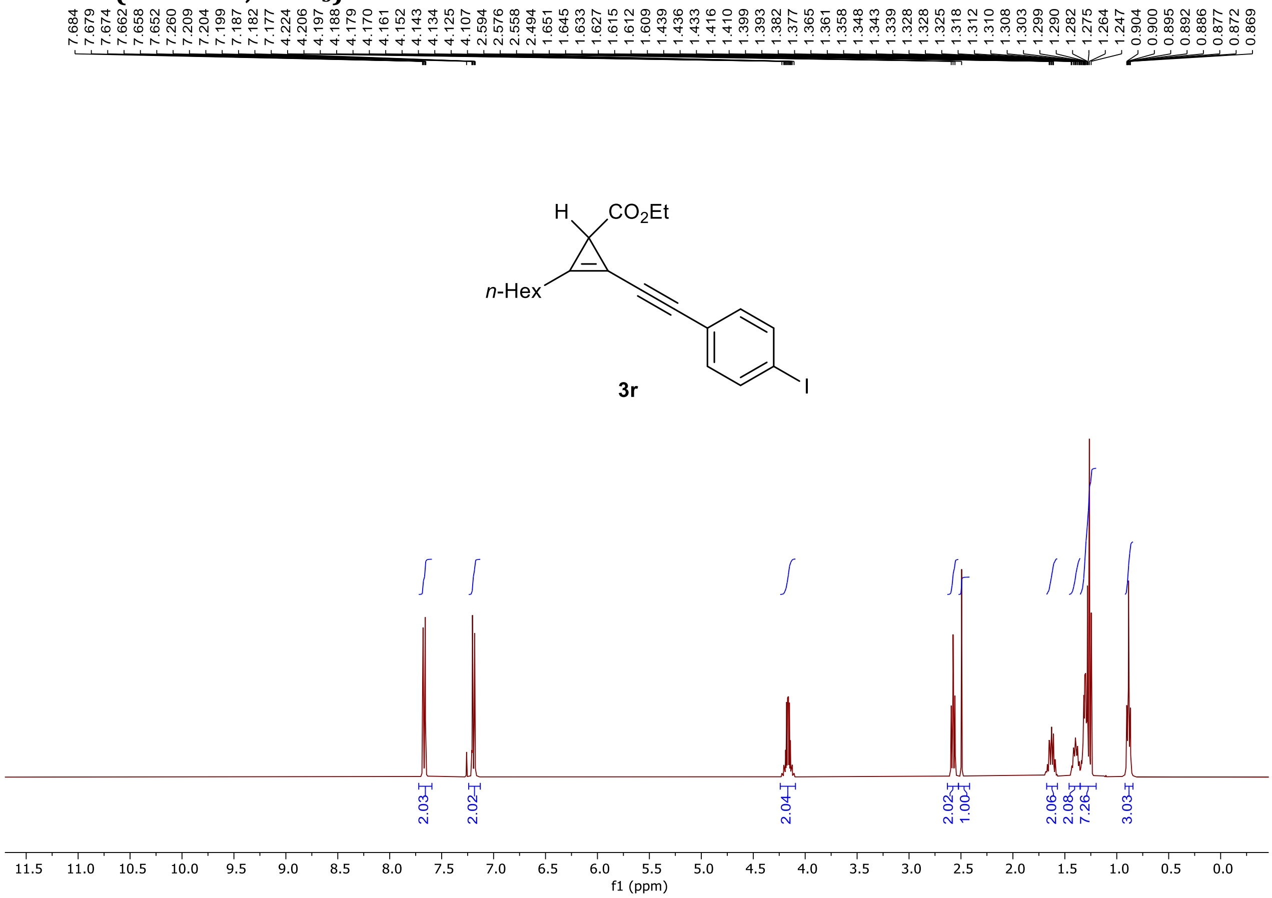

¹³C NMR (101 MHz, CDCl$_3$) of 3r

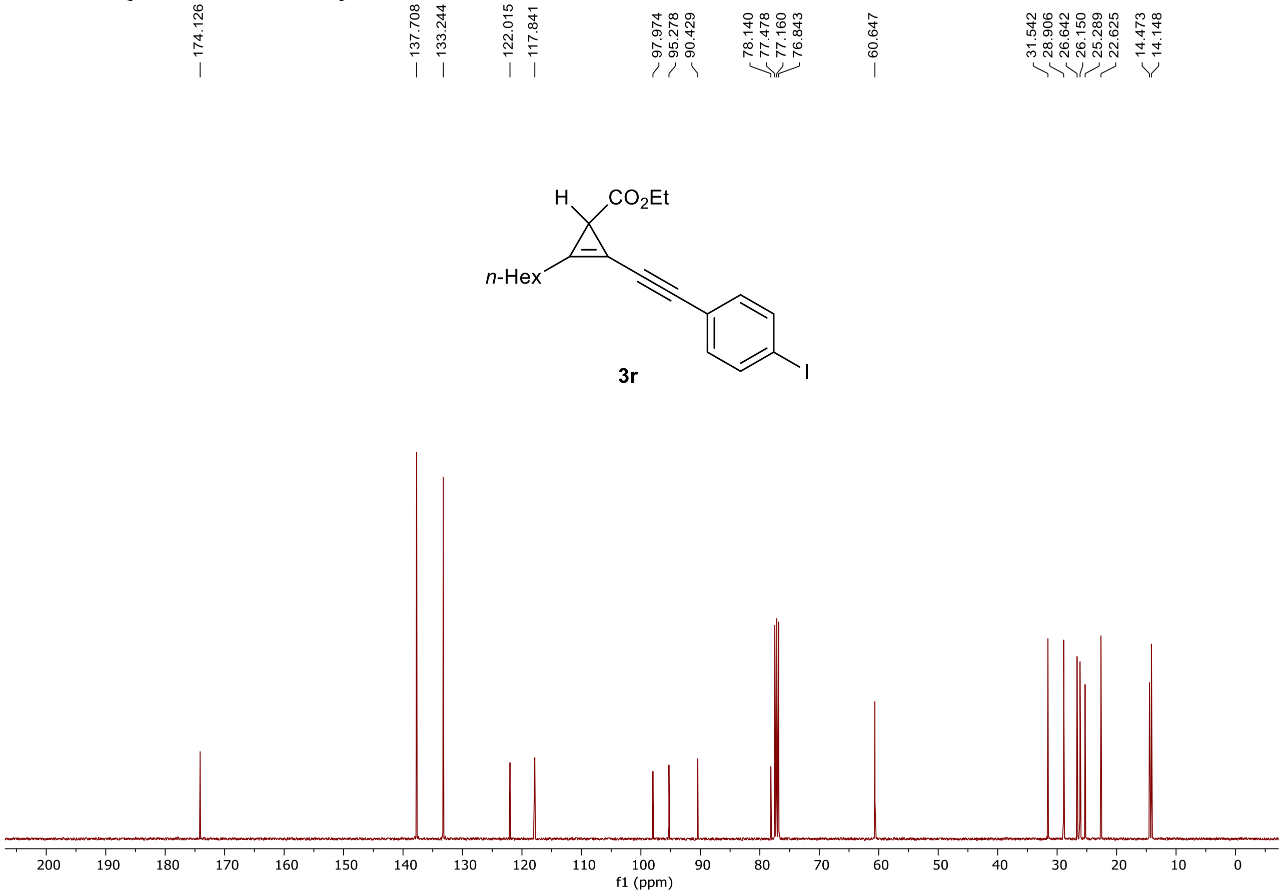

^{1}H NMR (400 MHz, $CDCl_3$) of 3s

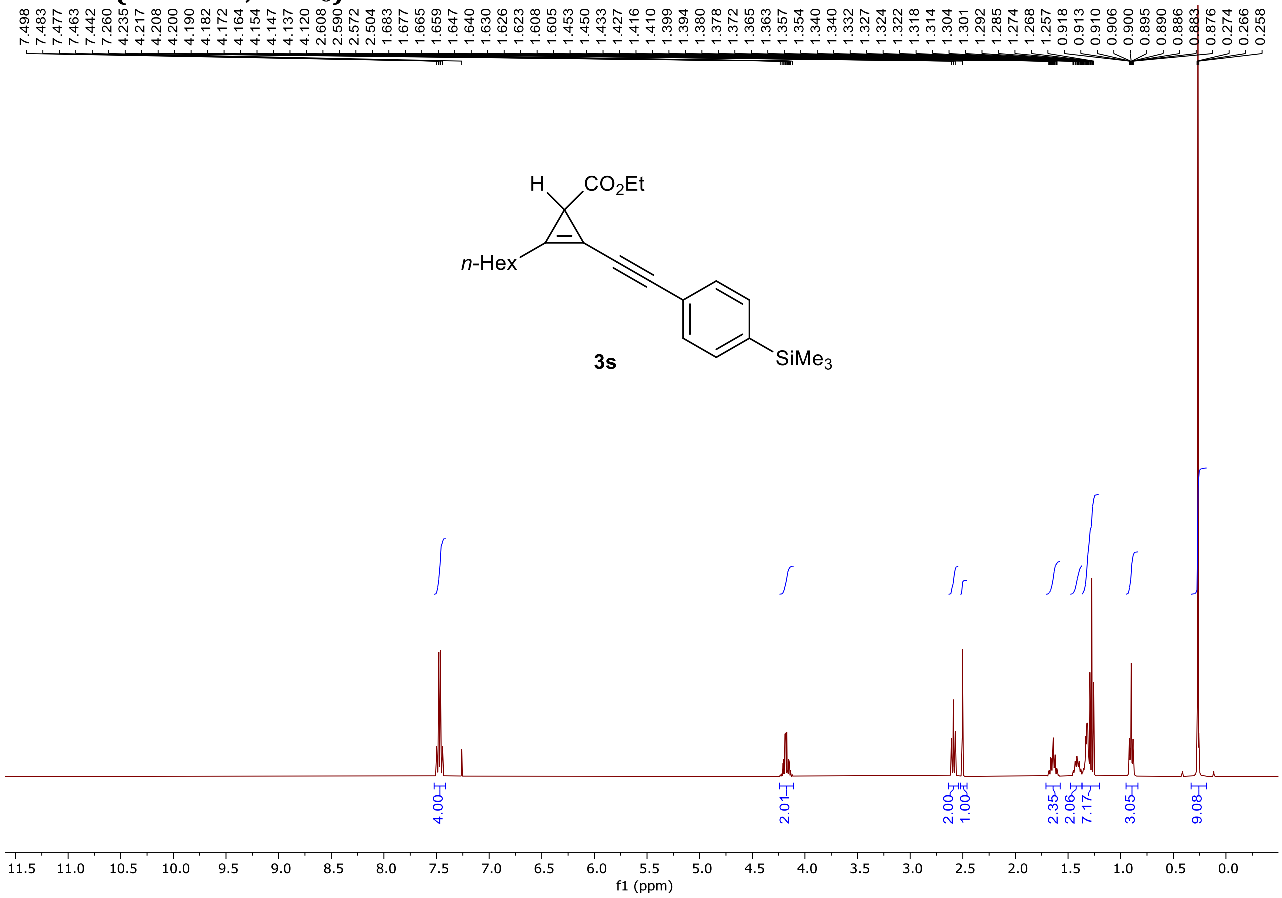

^{13}C NMR (101 MHz, $CDCl_3$) of 3s

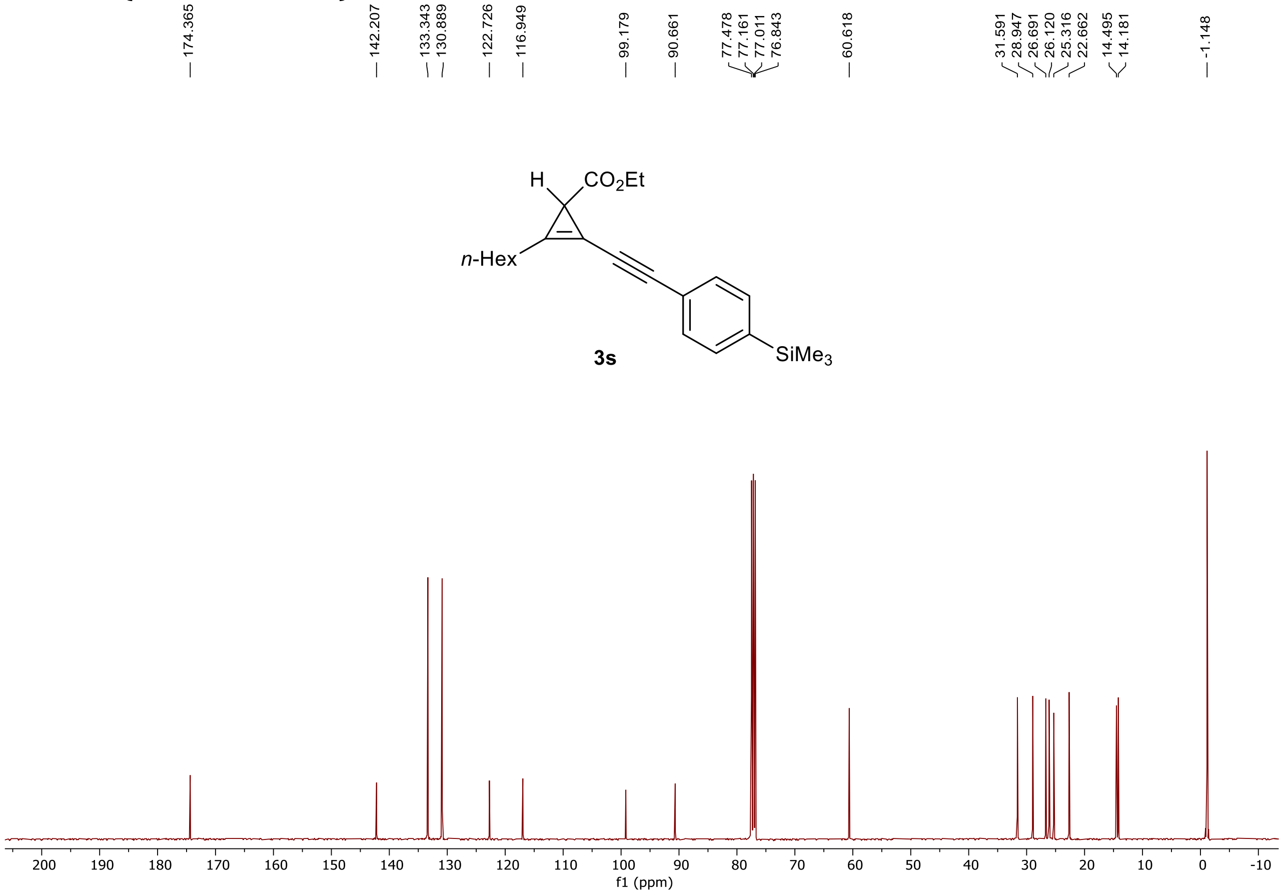

^{1}H NMR (400 MHz, $CDCl_3$) of 3t

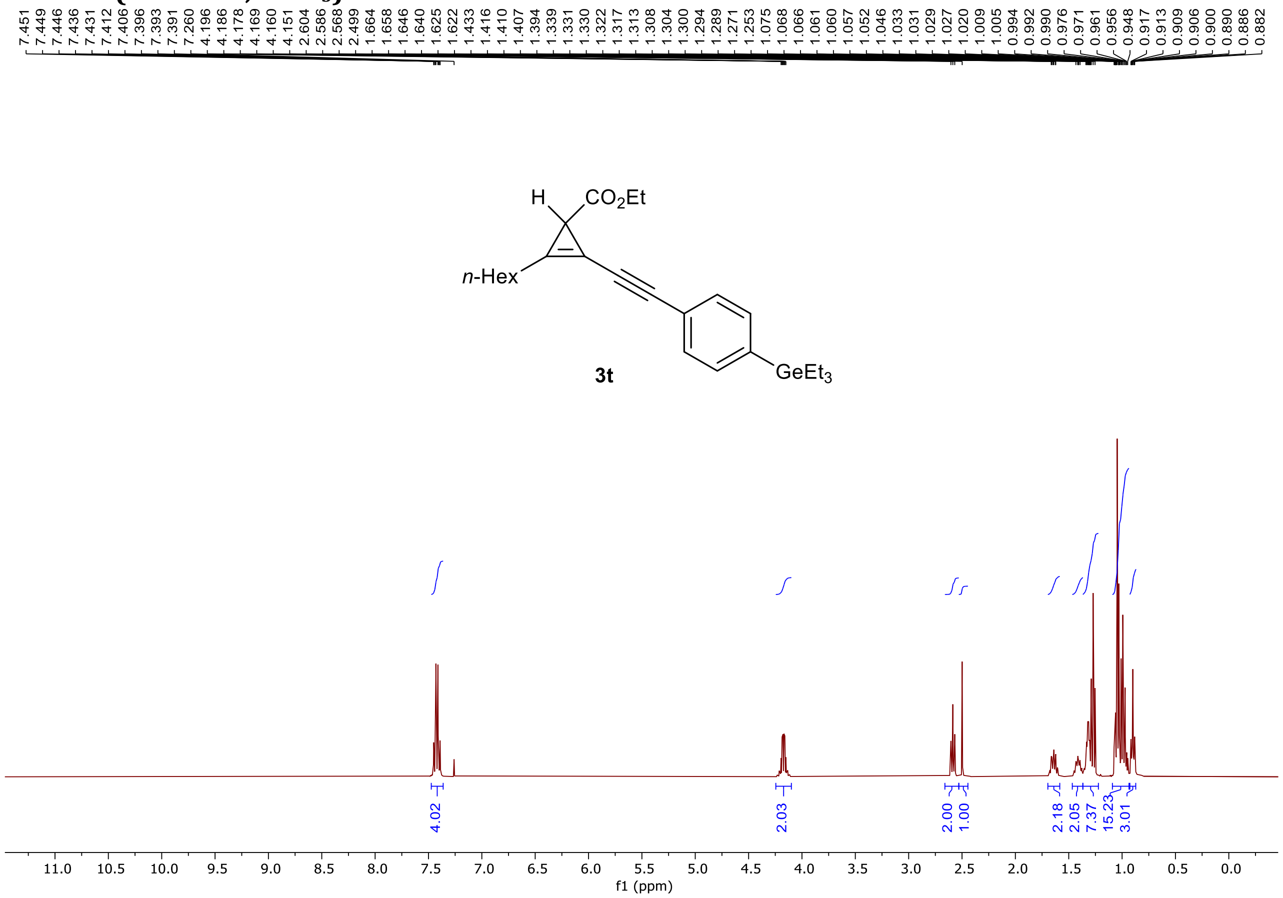

^{13}C NMR (101 MHz, $CDCl_3$) of 3t

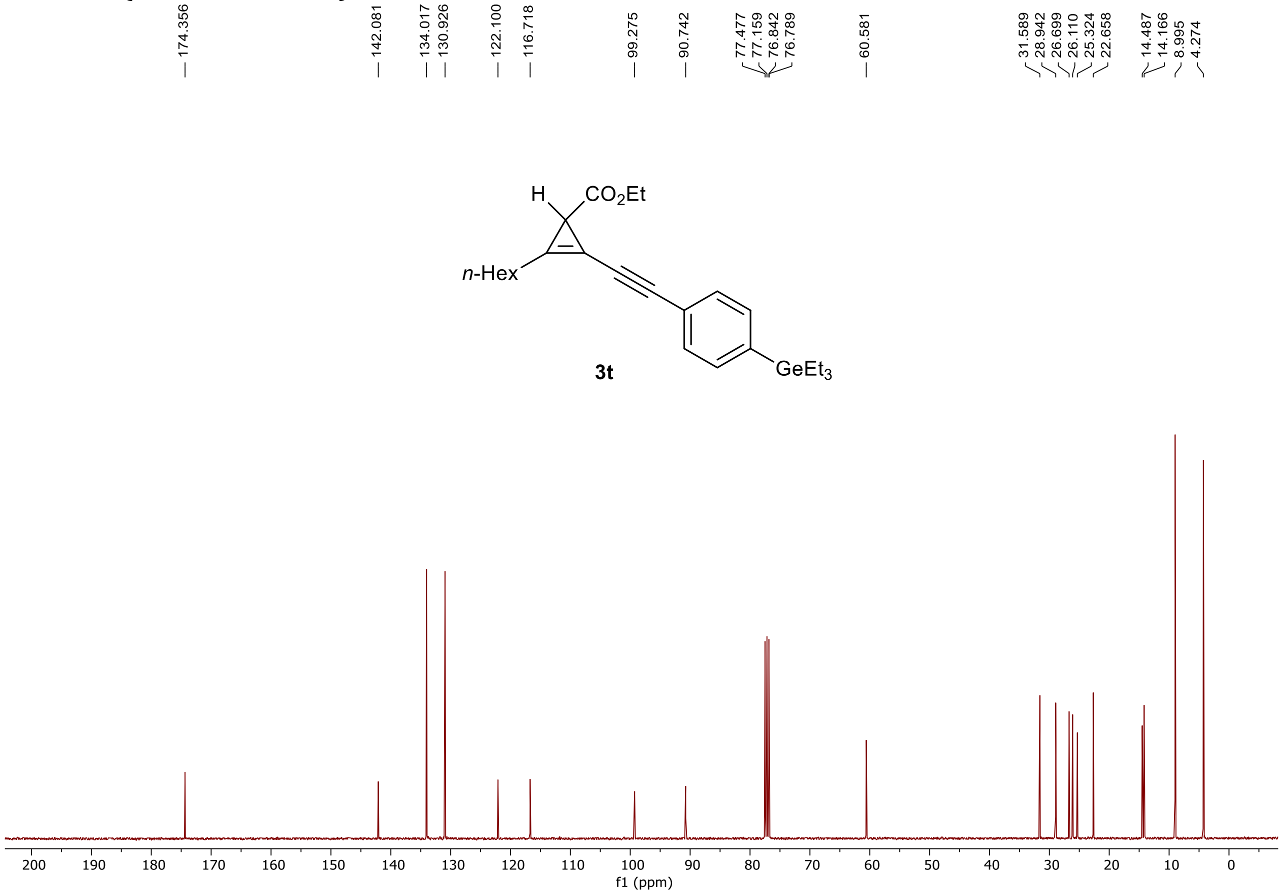

^{1}H NMR (400 MHz, $CDCl_3$) of 3u

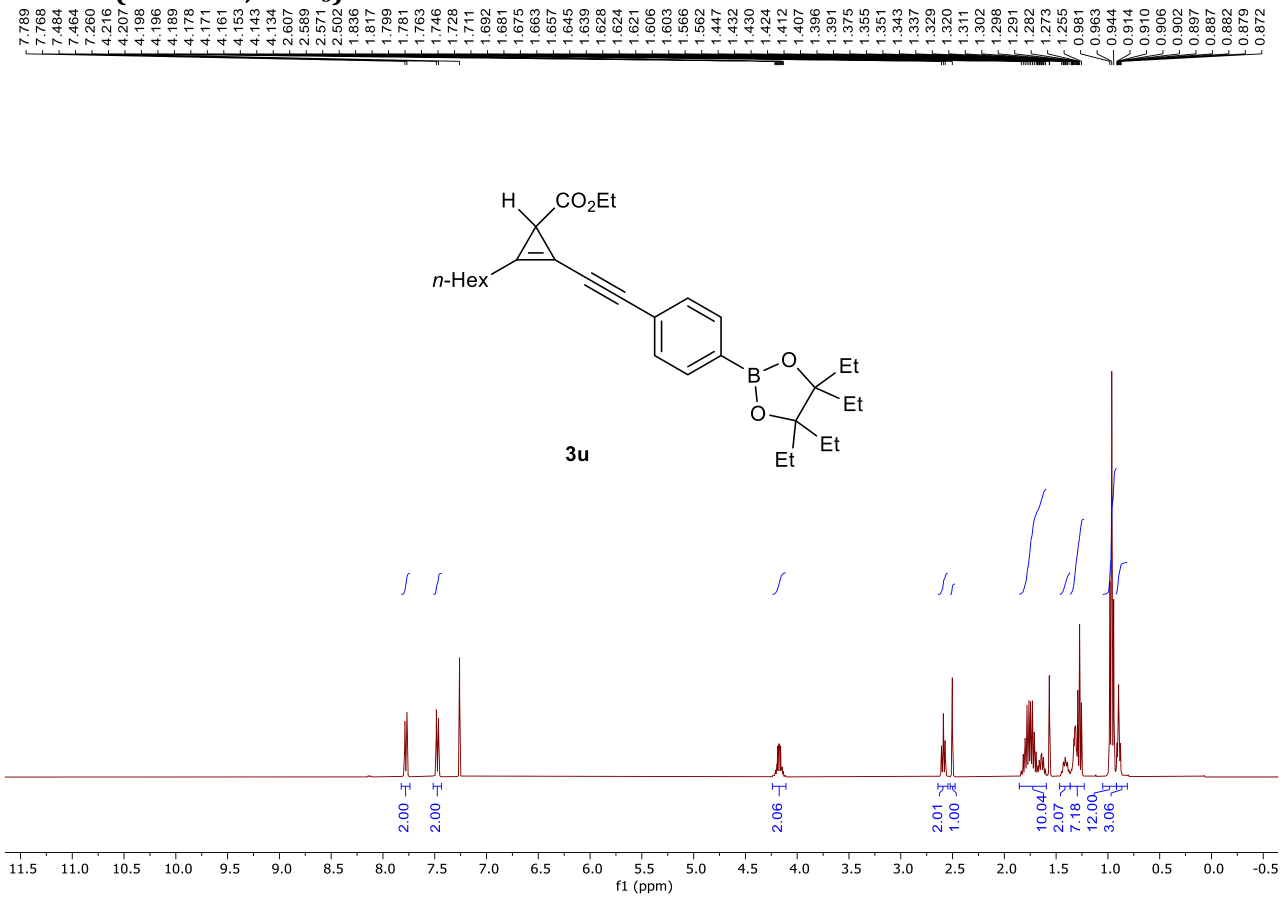

^{13}C NMR (101 MHz, $CDCl_3$) of 3u

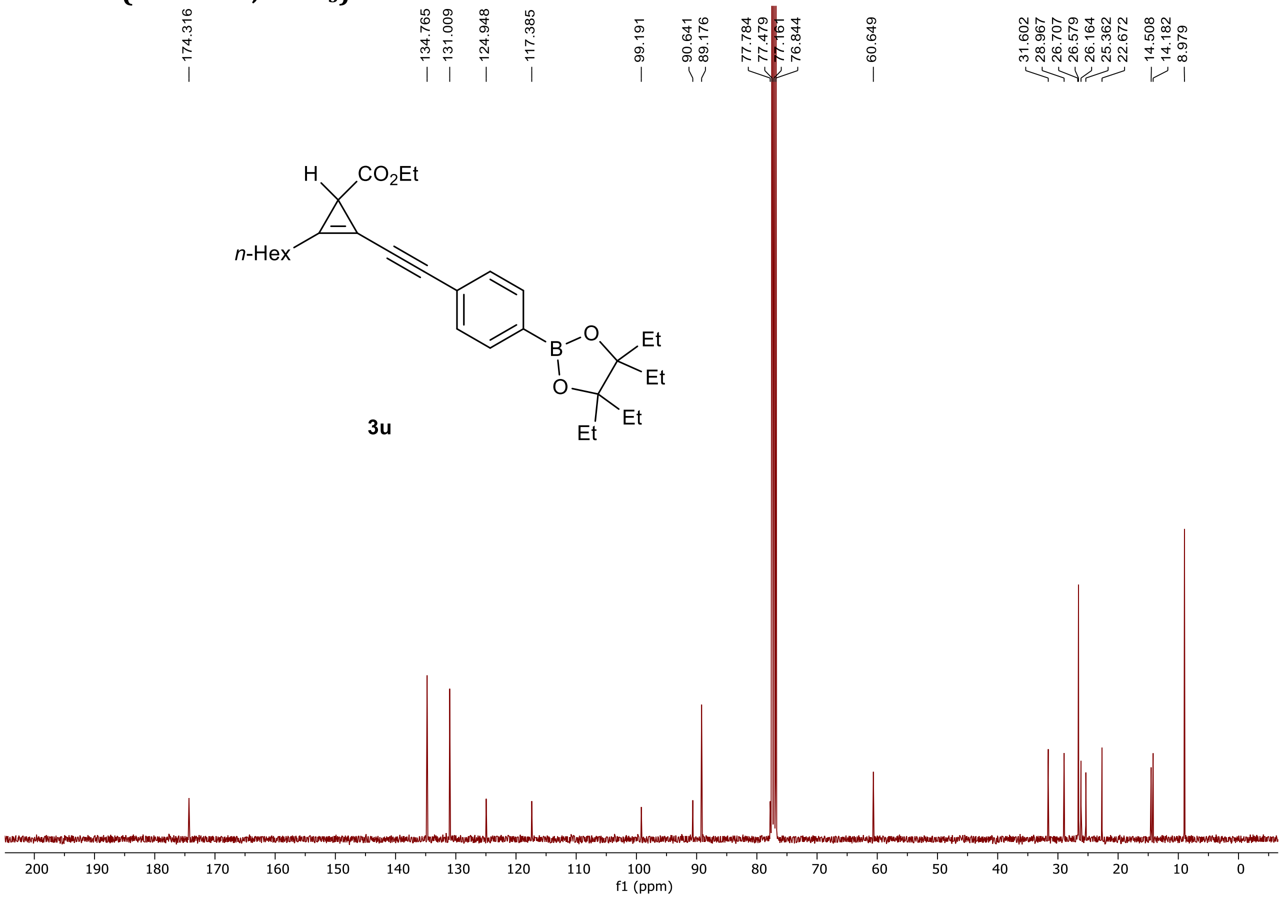

^{11}B NMR (128 MHz, CDCl3) of 3u

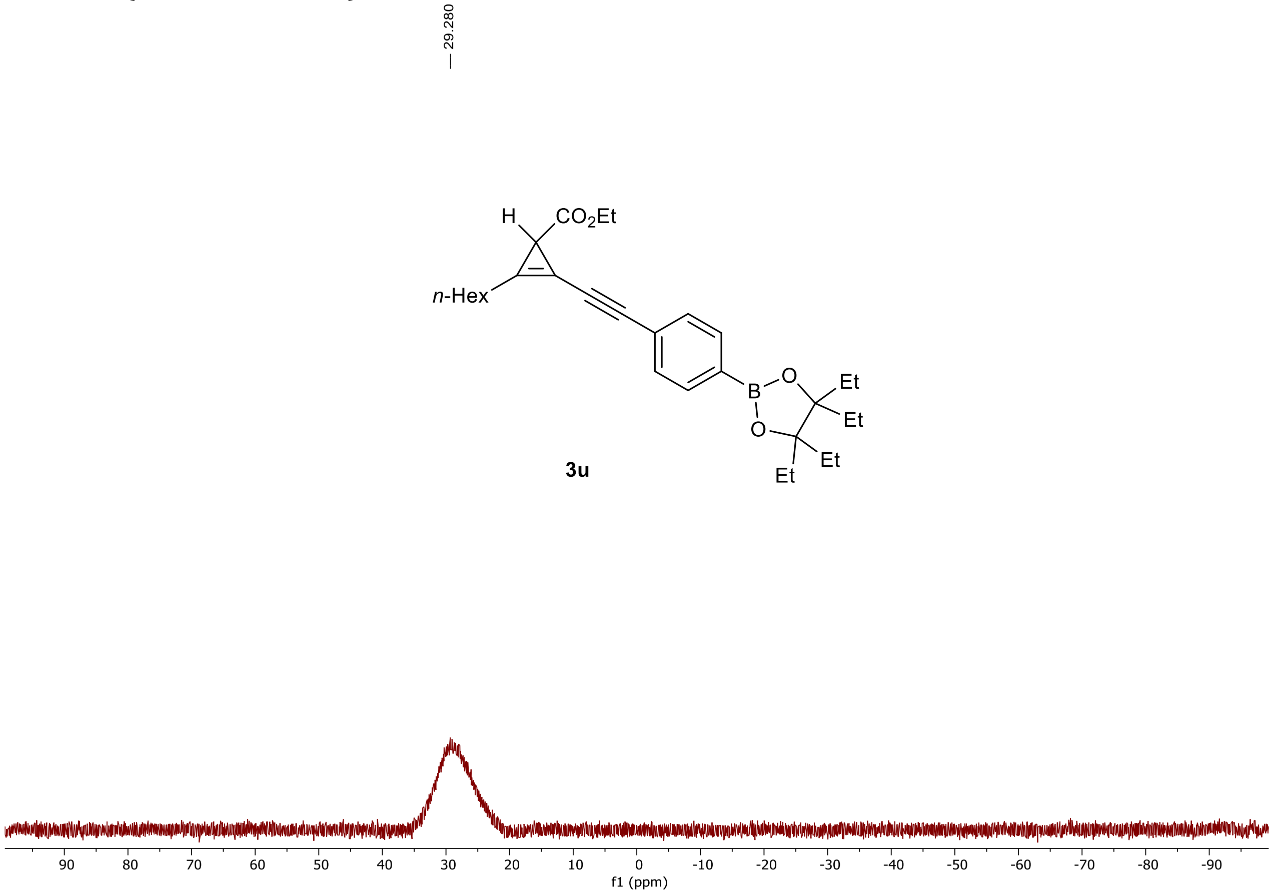

^{1}H NMR (400 MHz, $CDCl_3$) of 3v

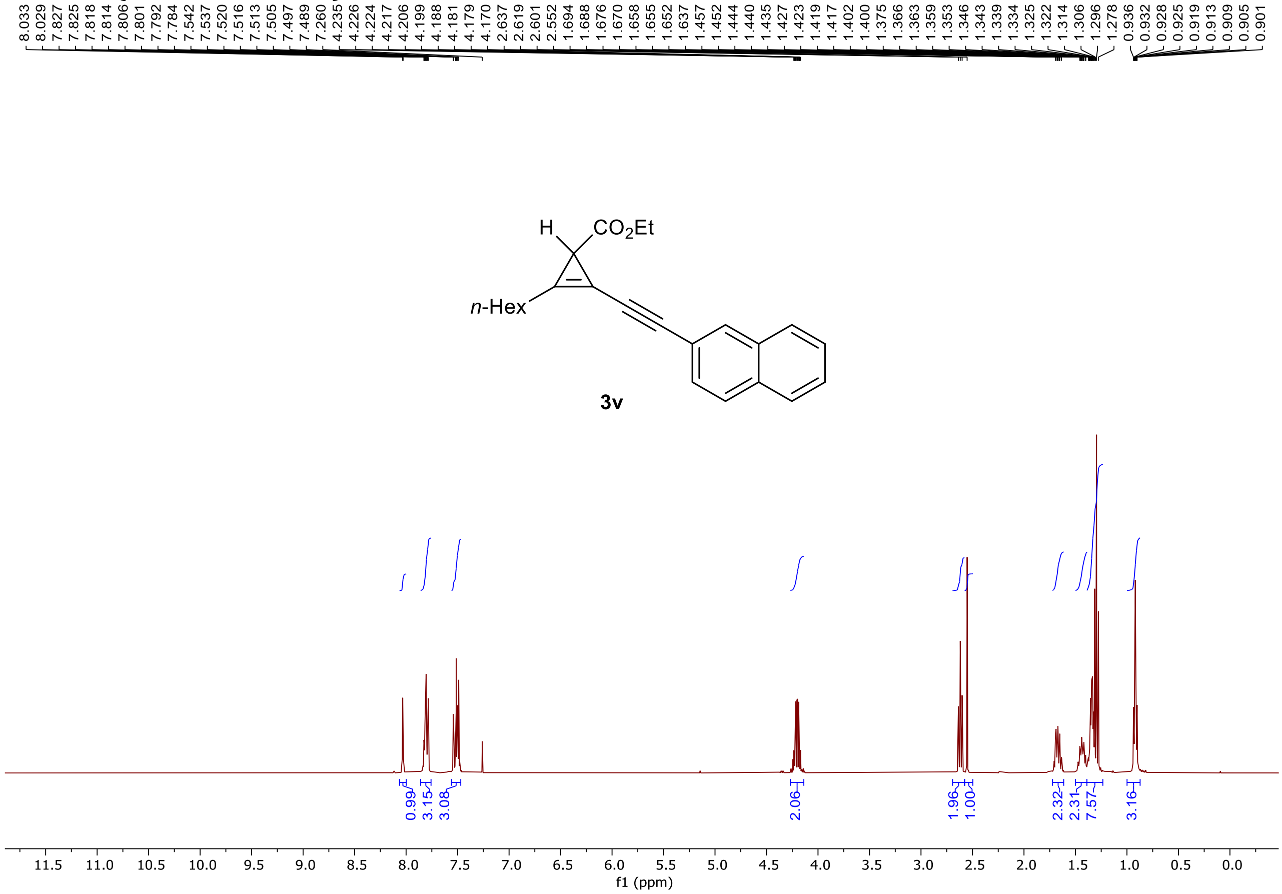

^{13}C NMR (101 MHz, $CDCl_3$) of 3v

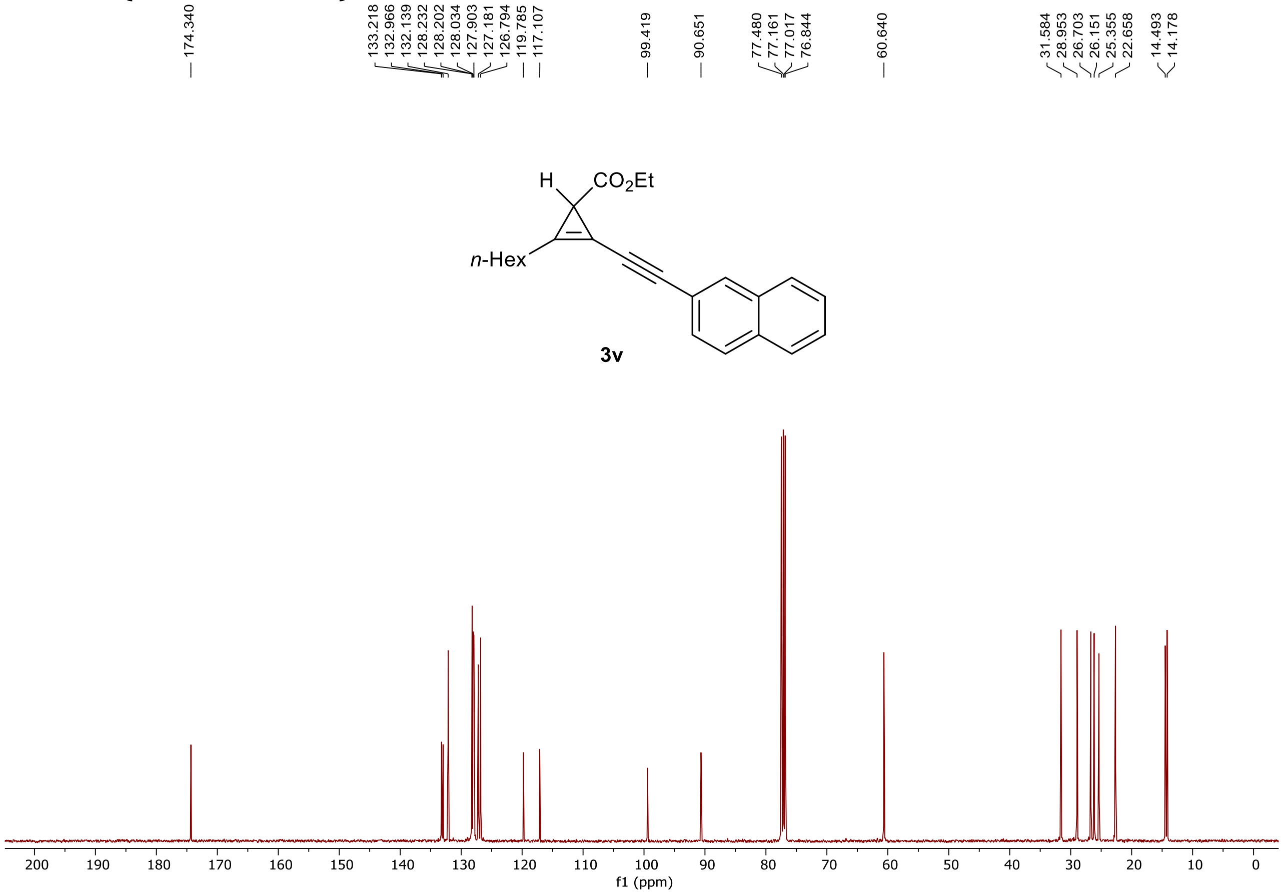

^{1}H NMR (400 MHz, $CDCl_3$) of 3w

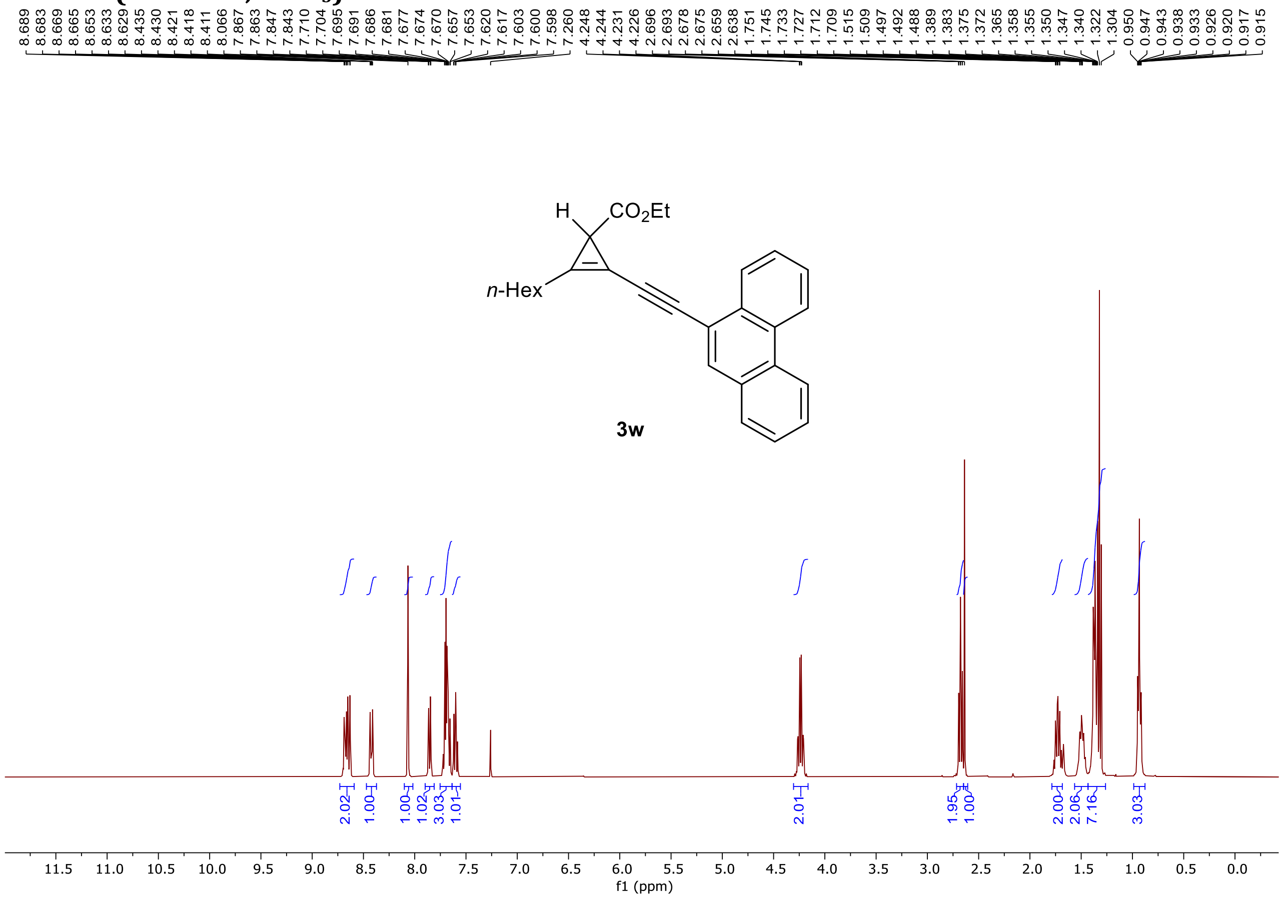

^{13}C NMR (101 MHz, $CDCl_3$) of 3w

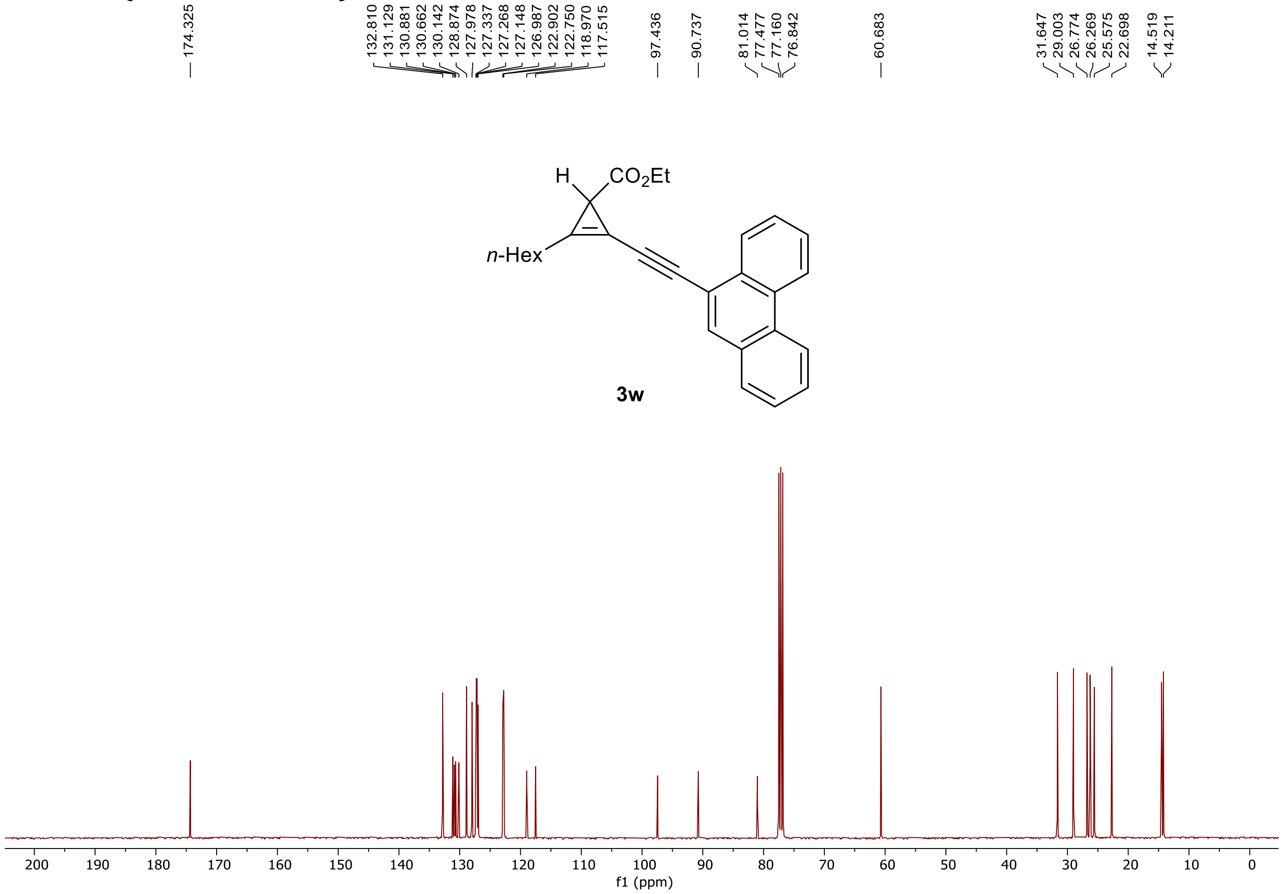

^{1}H NMR (400 MHz, $CDCl_3$) of 3x

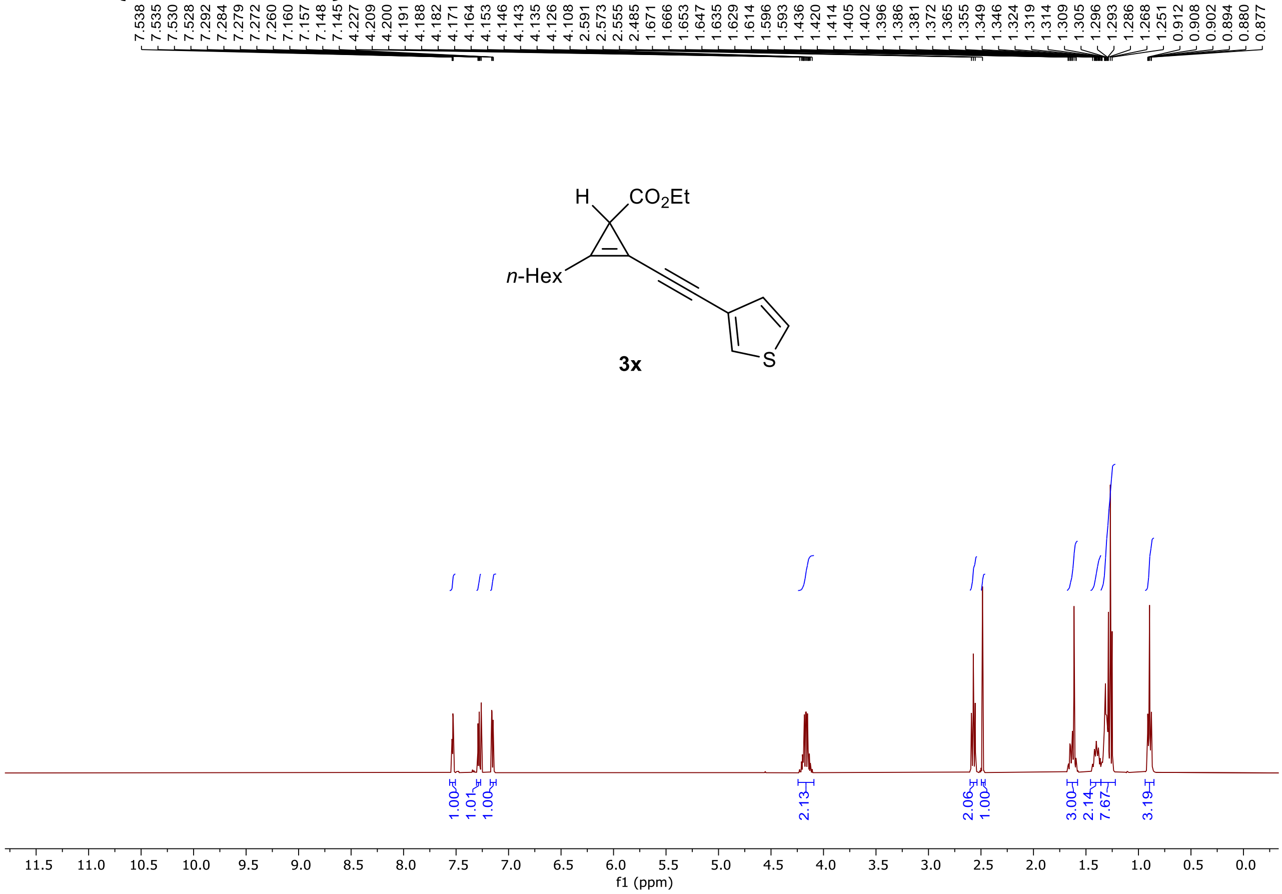

^{13}C NMR (101 MHz, $CDCl_3$) of 3x

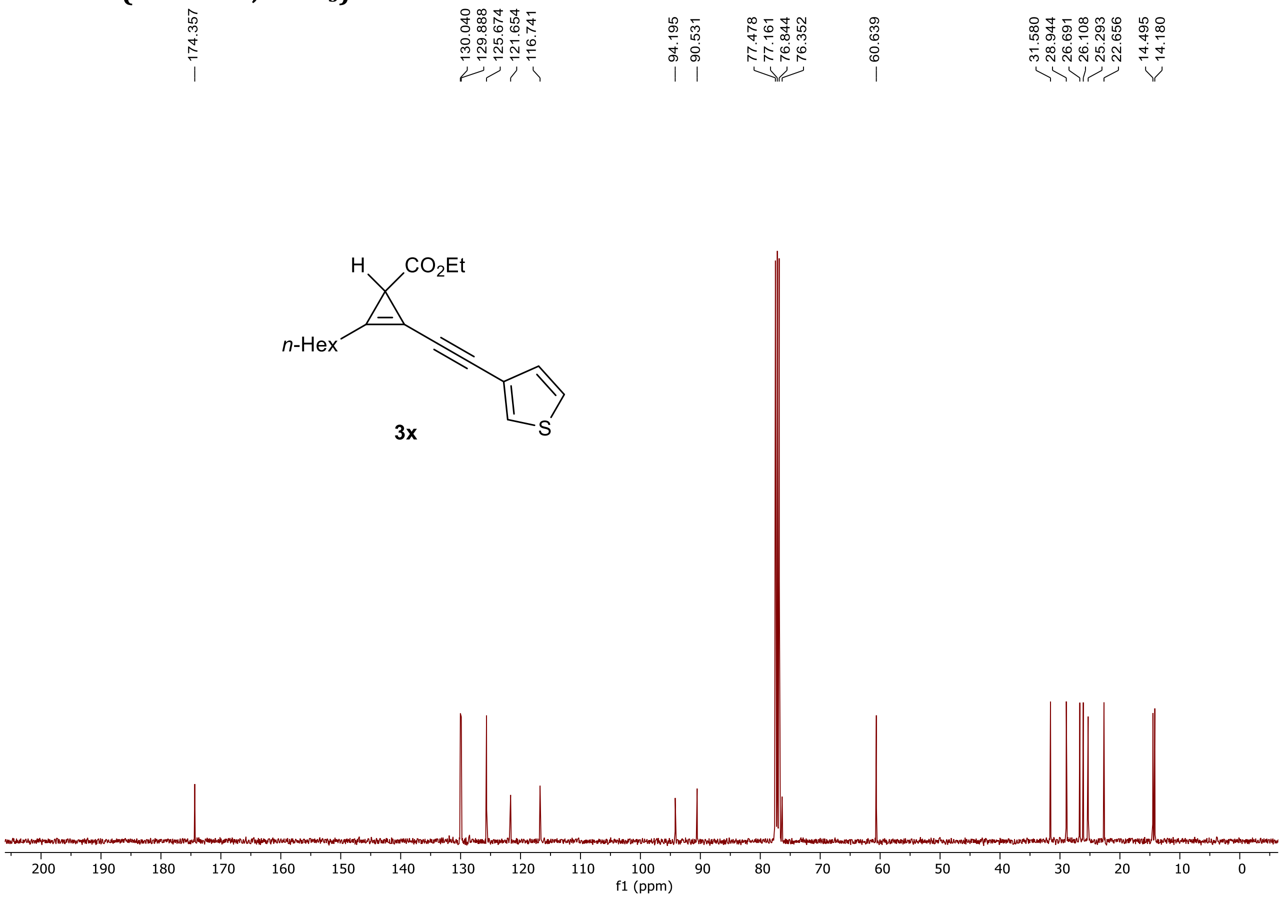

^{1}H NMR (400 MHz, $CDCl_3$) of 3y

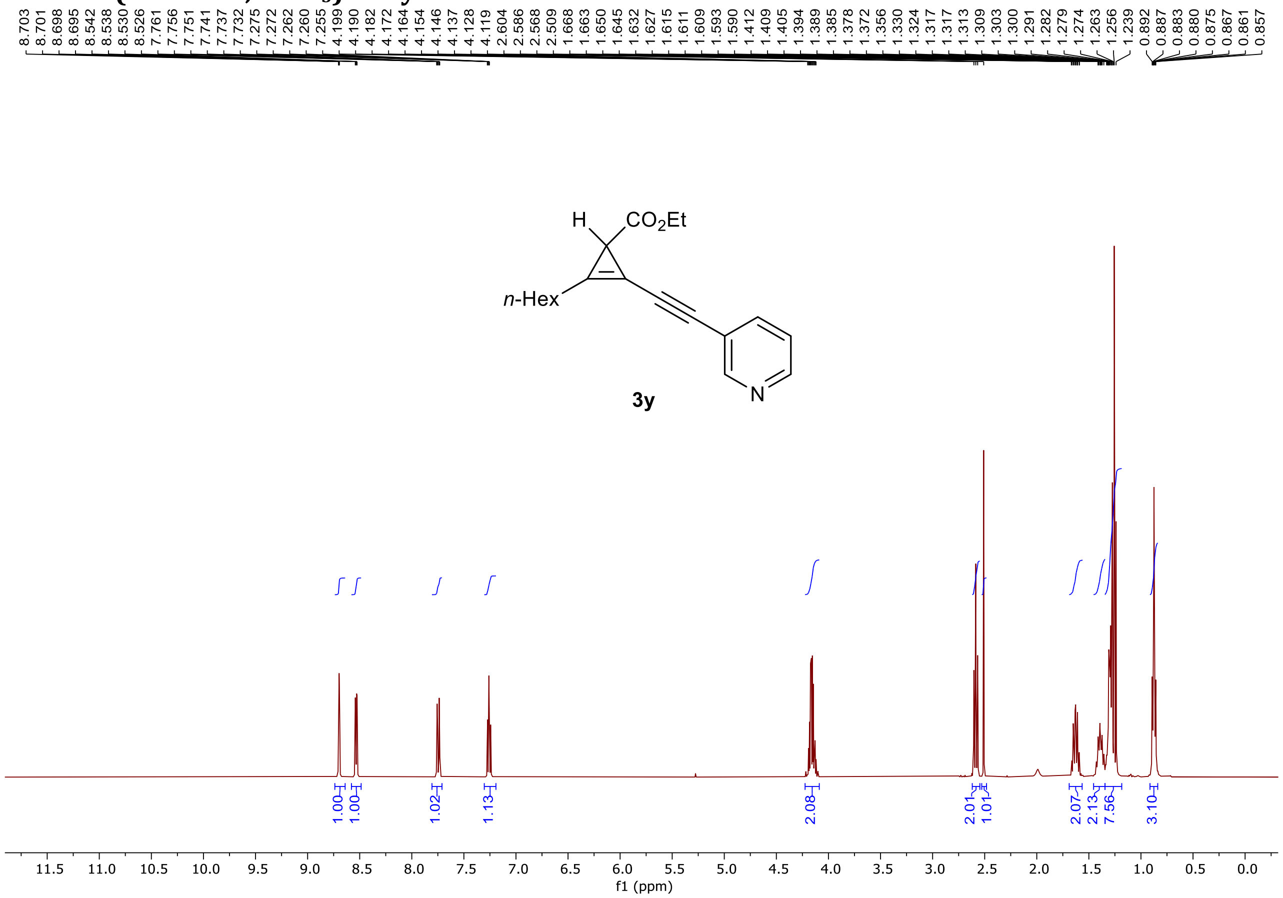

^{13}C NMR (101 MHz, $CDCl_3$) of 3y

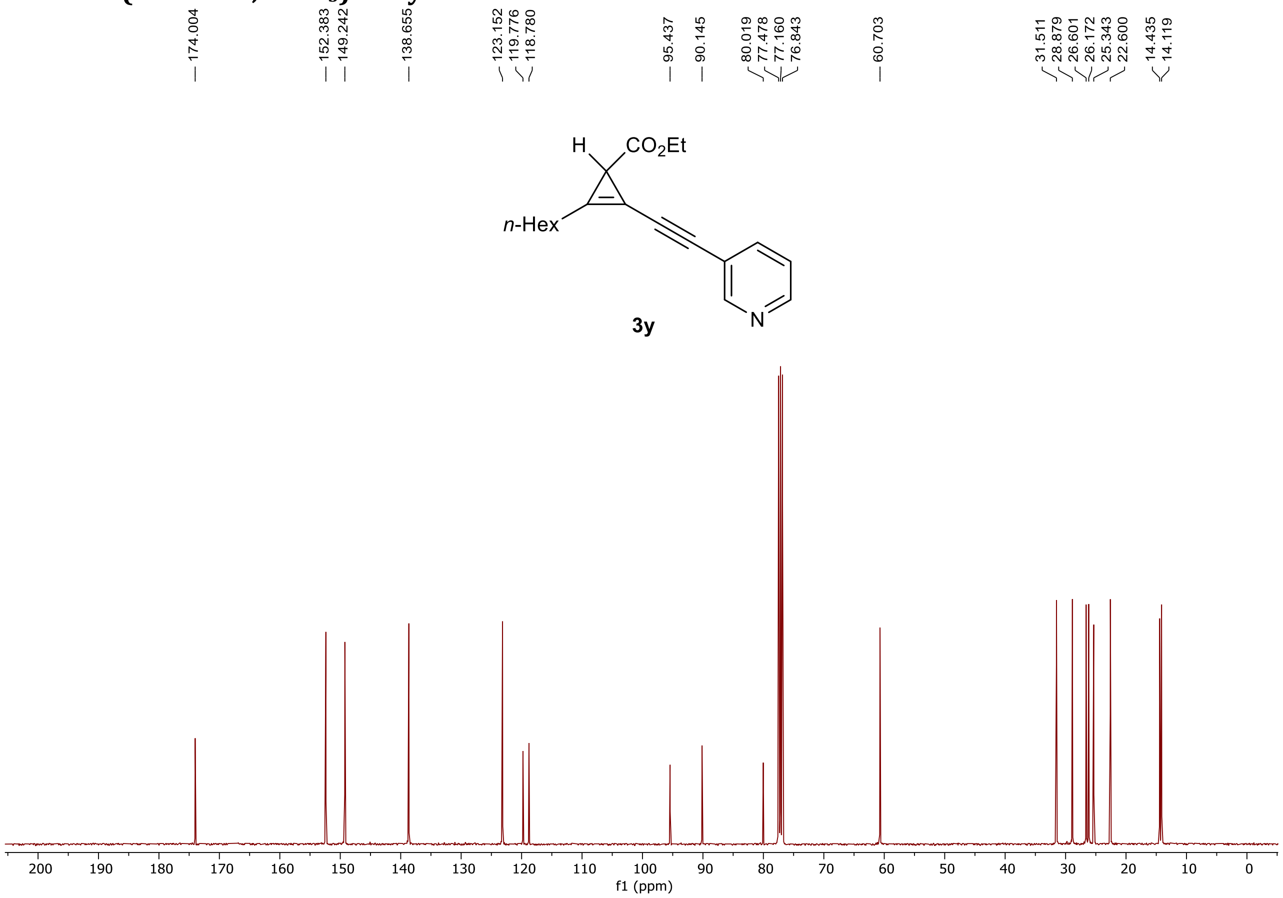

^{1}H NMR (400 MHz, $CDCl_3$) of 3z

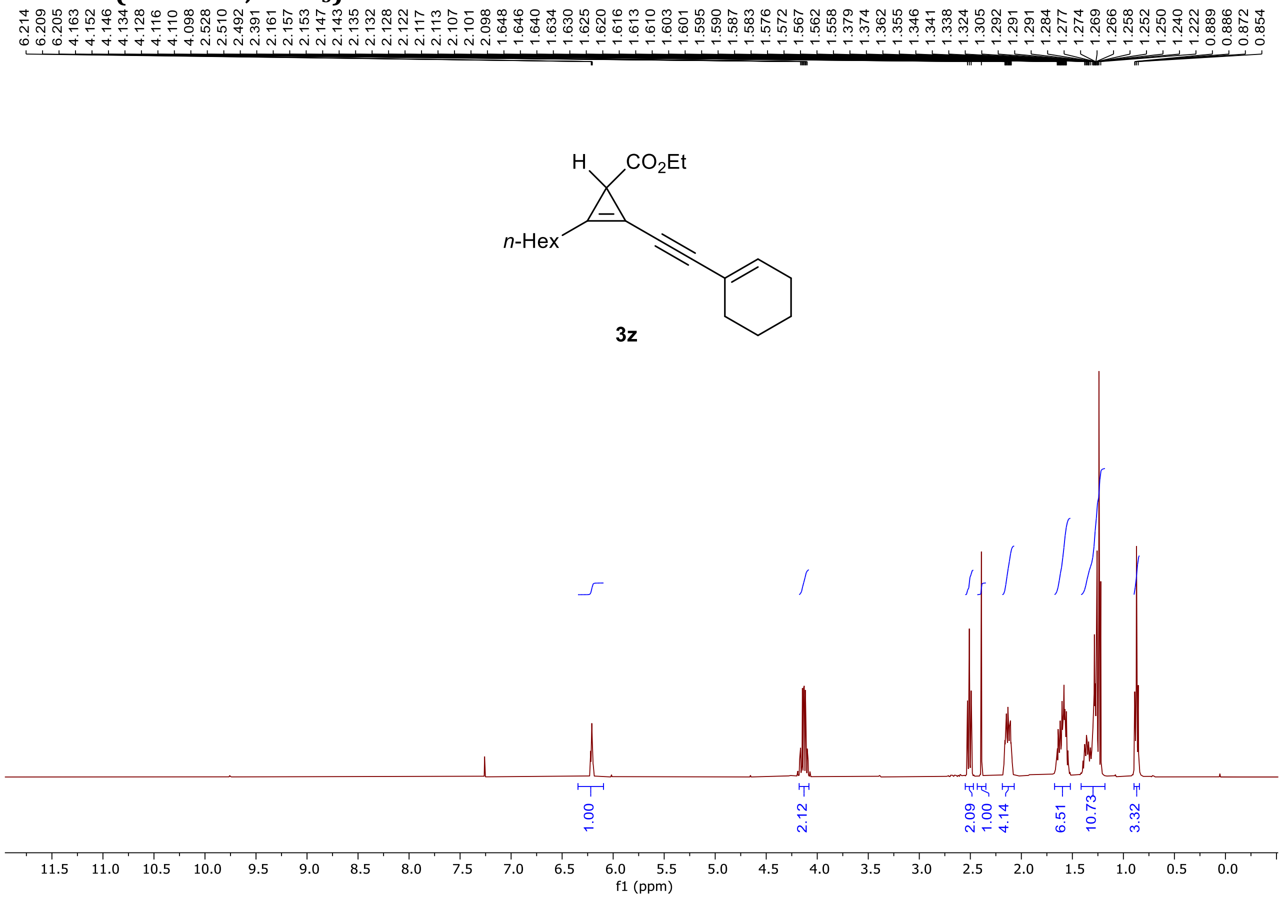

^{13}C NMR (101 MHz, $CDCl_3$) of 3z

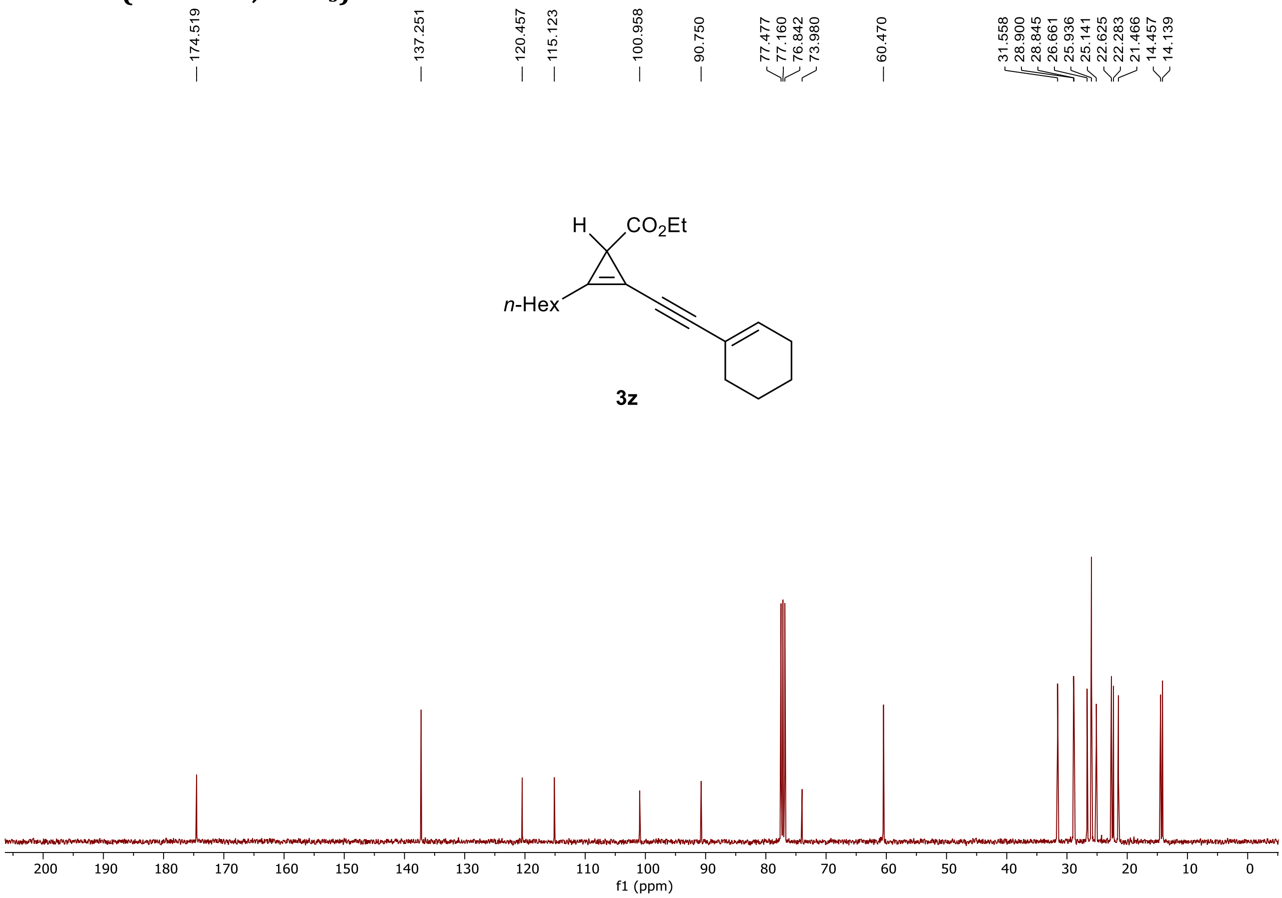

^{1}H NMR (400 MHz, $CDCl_3$) of 3aa

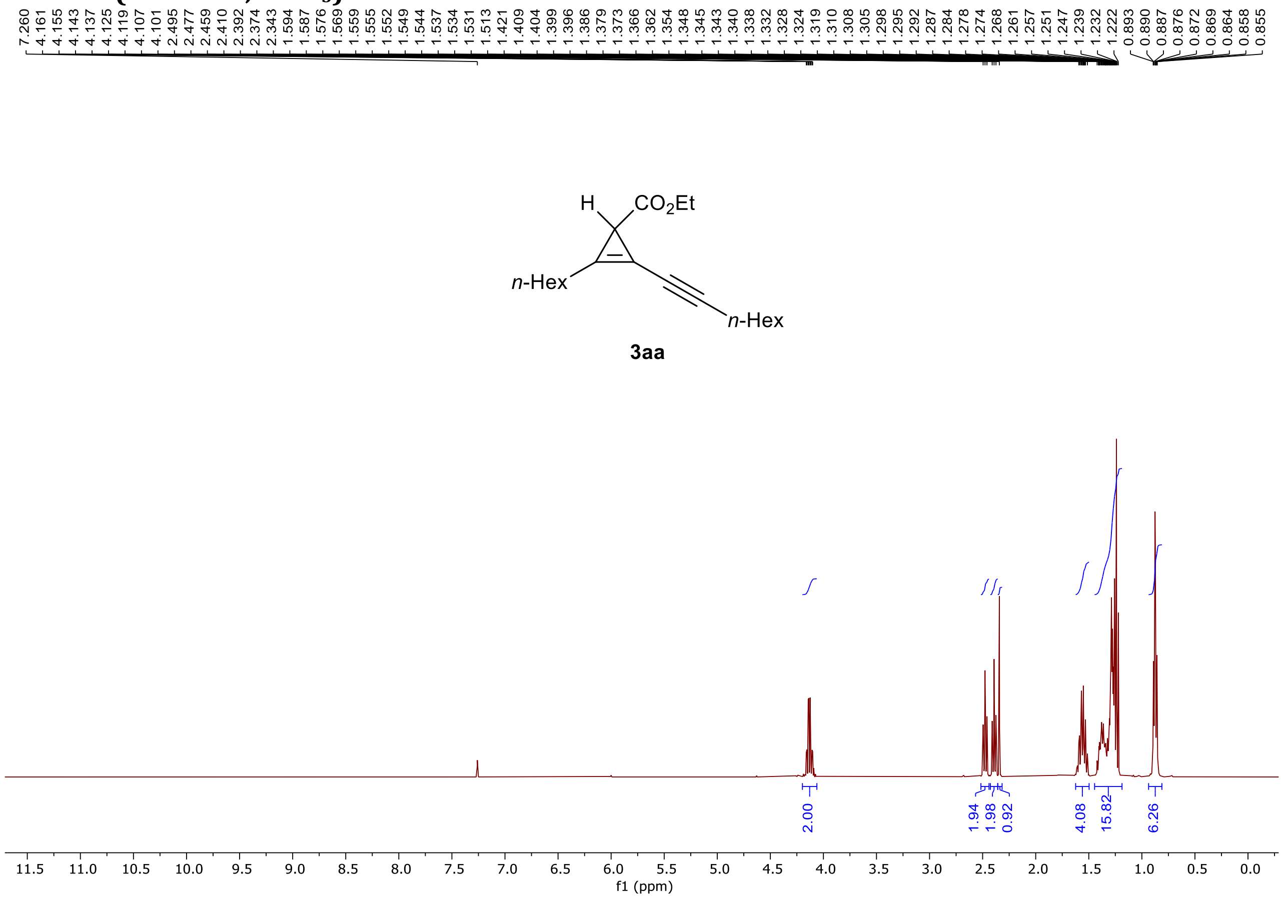

^{13}C NMR (101 MHz, $CDCl_3$) of 3aa

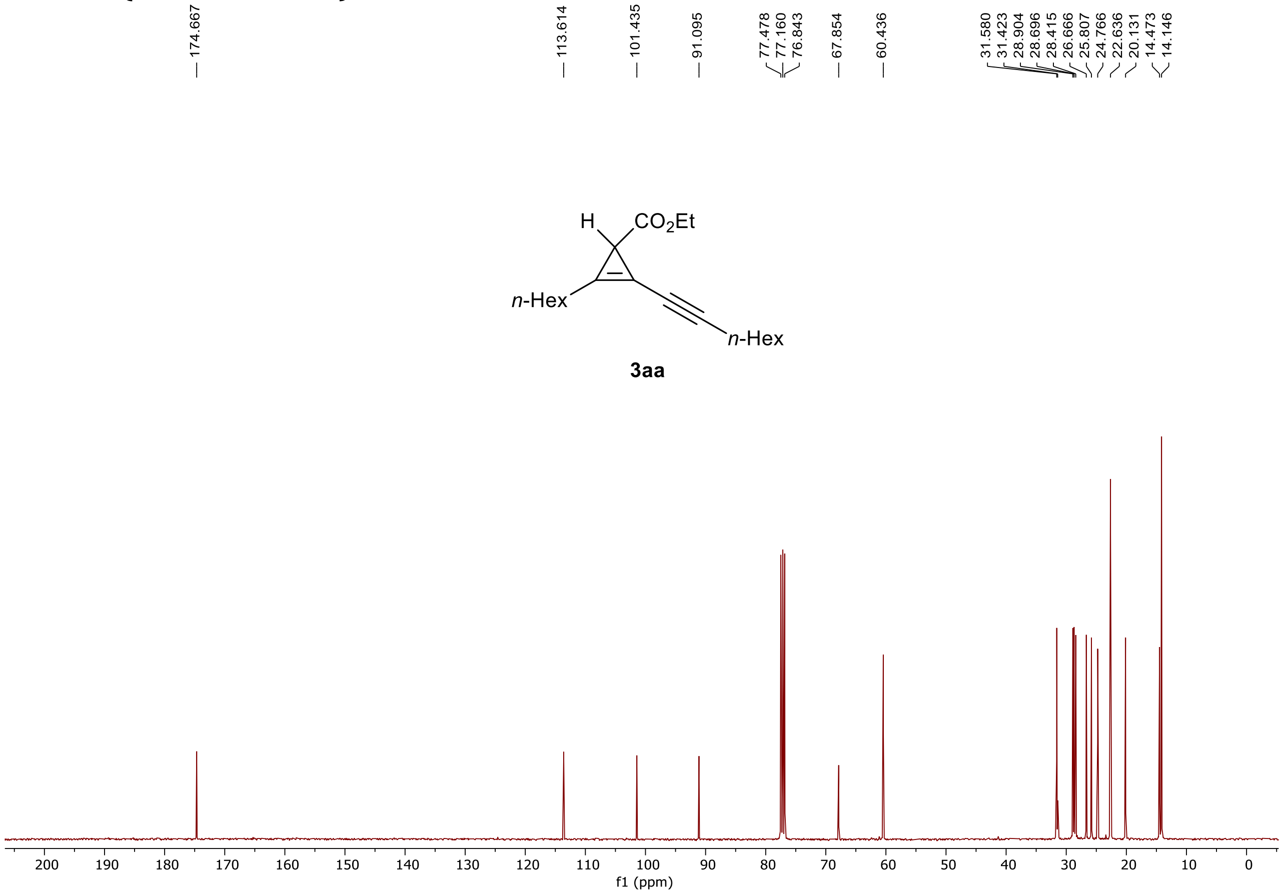

^{1}H NMR (400 MHz, $CDCl_3$) of 3ab

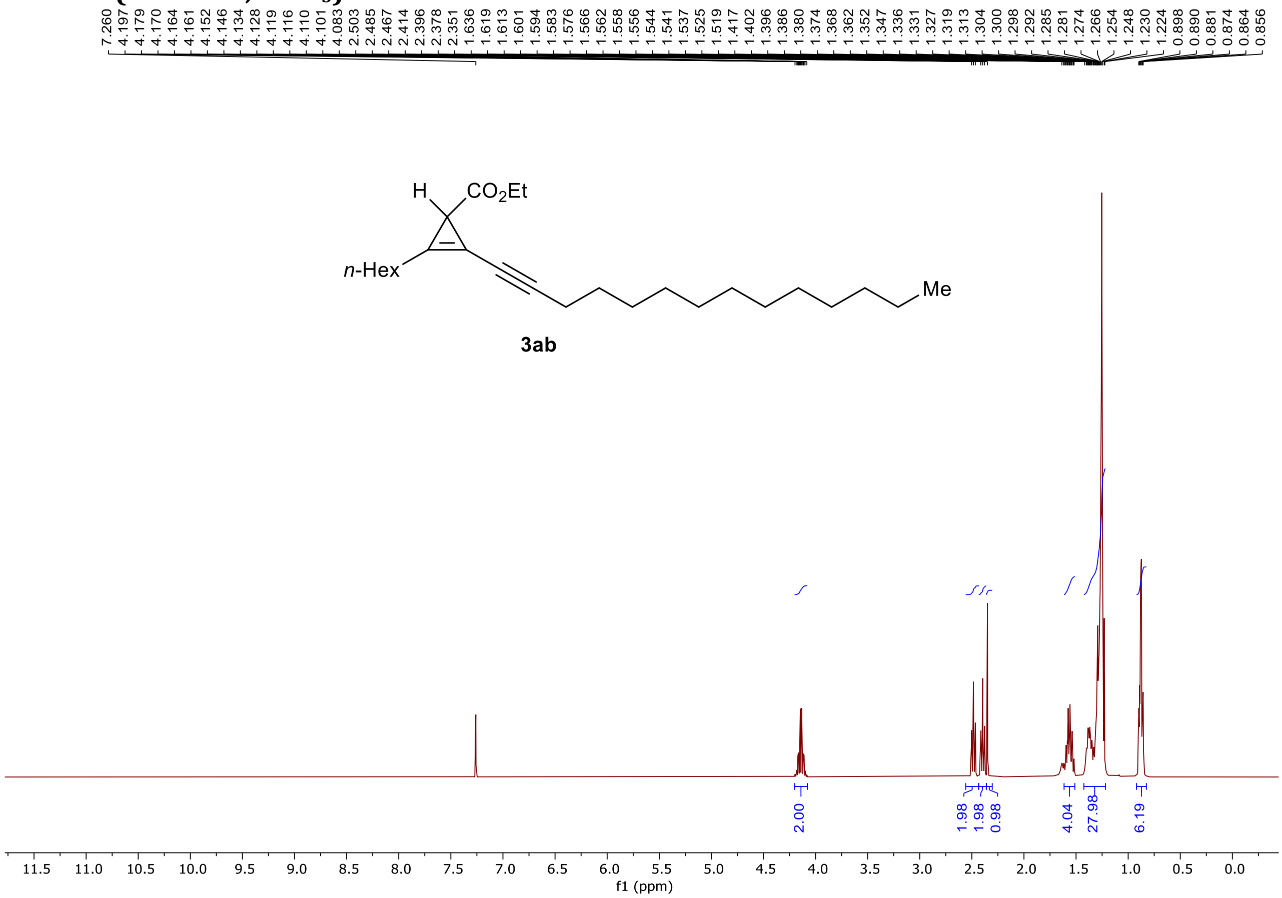

^{13}C NMR (101 MHz, $CDCl_3$) of 3ab

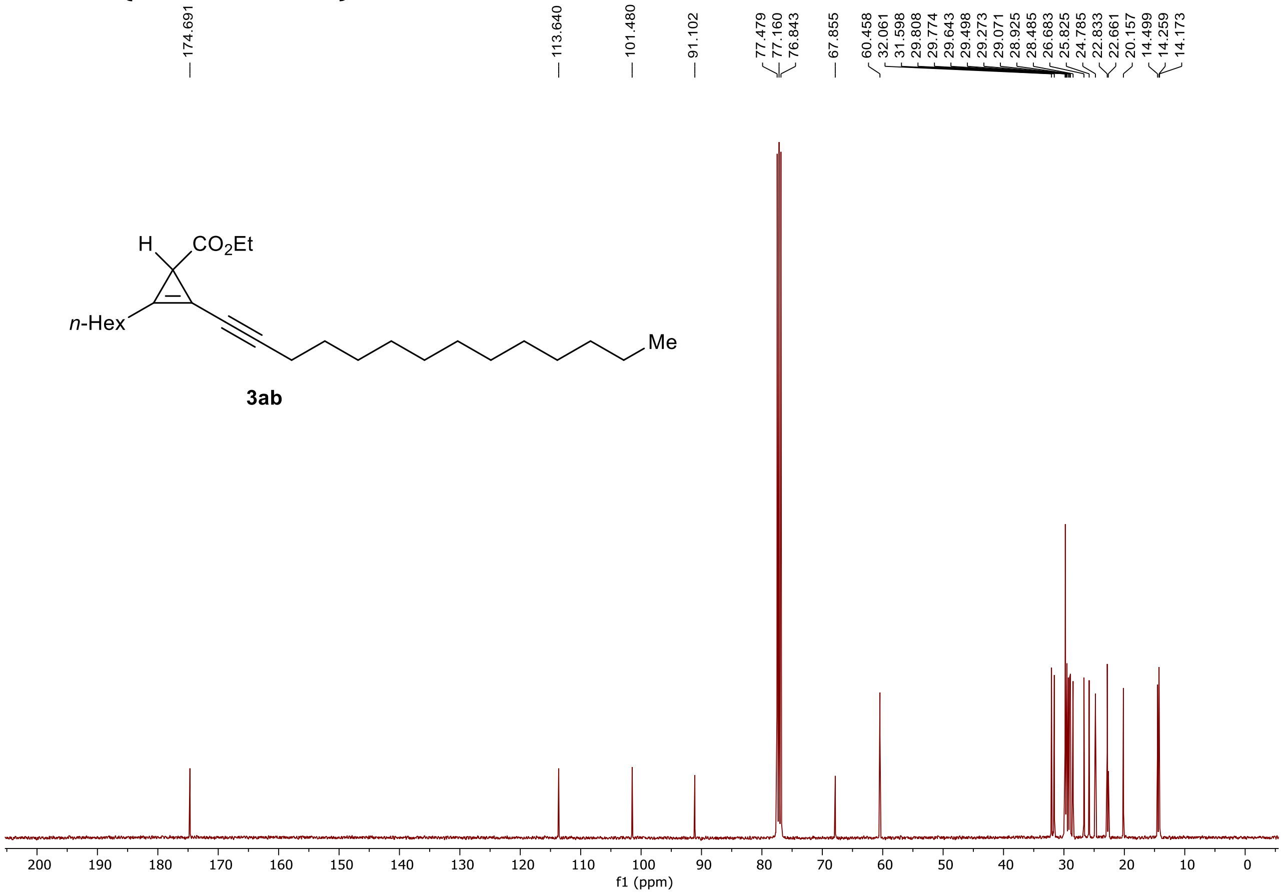

^{1}H NMR (400 MHz, $CDCl_3$) of 3ac

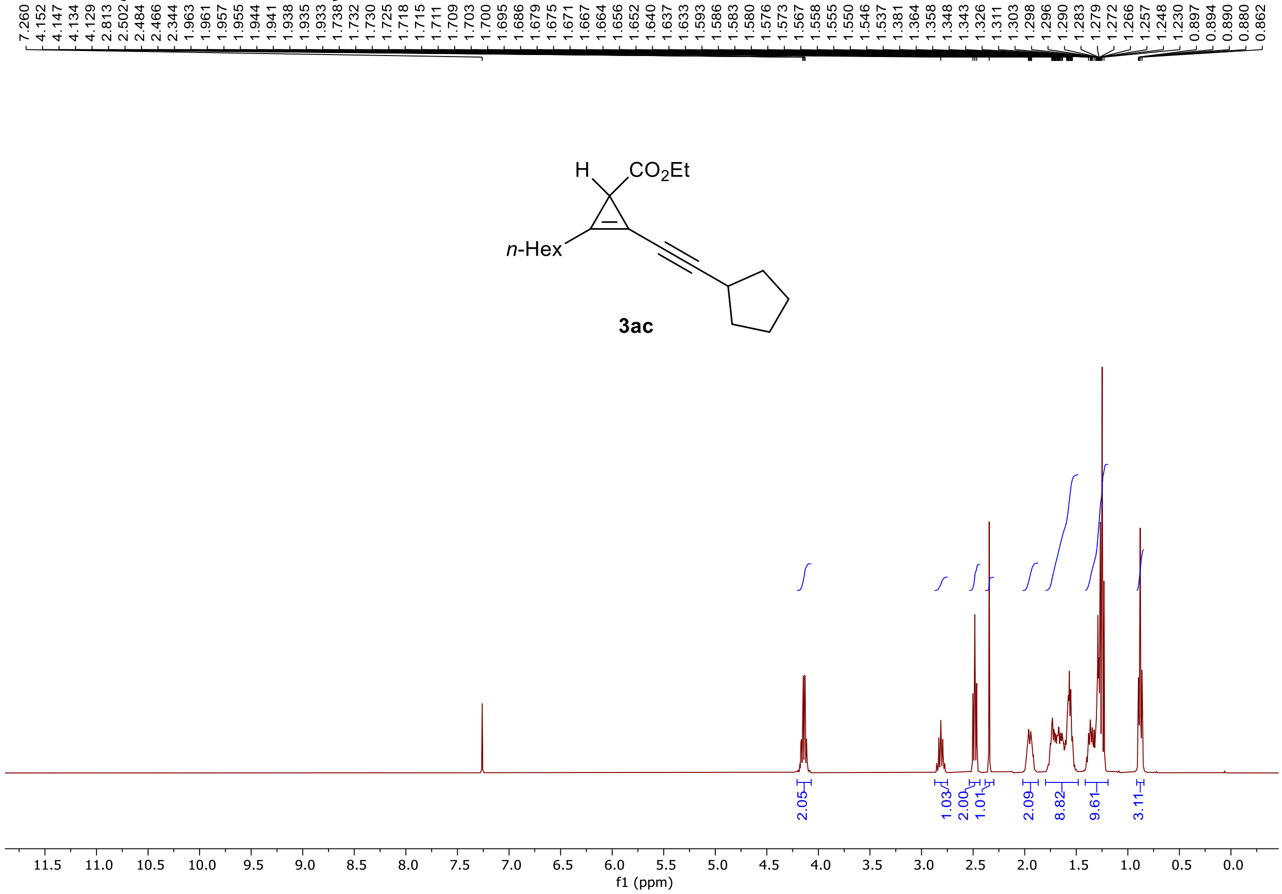

^{13}C NMR (101 MHz, $CDCl_3$) of 3ac

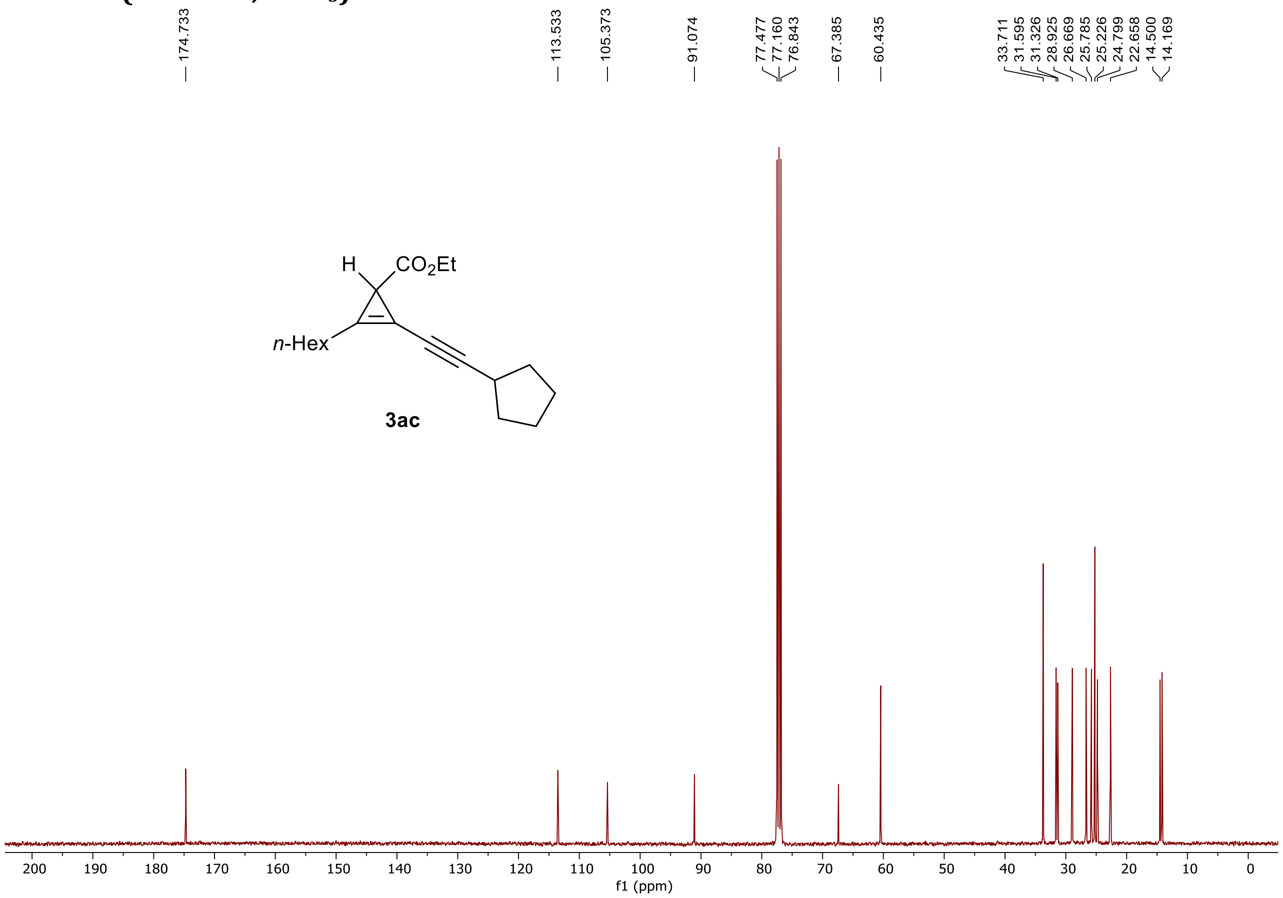

^{1}H NMR (400 MHz, $CDCl_3$) of 3ad

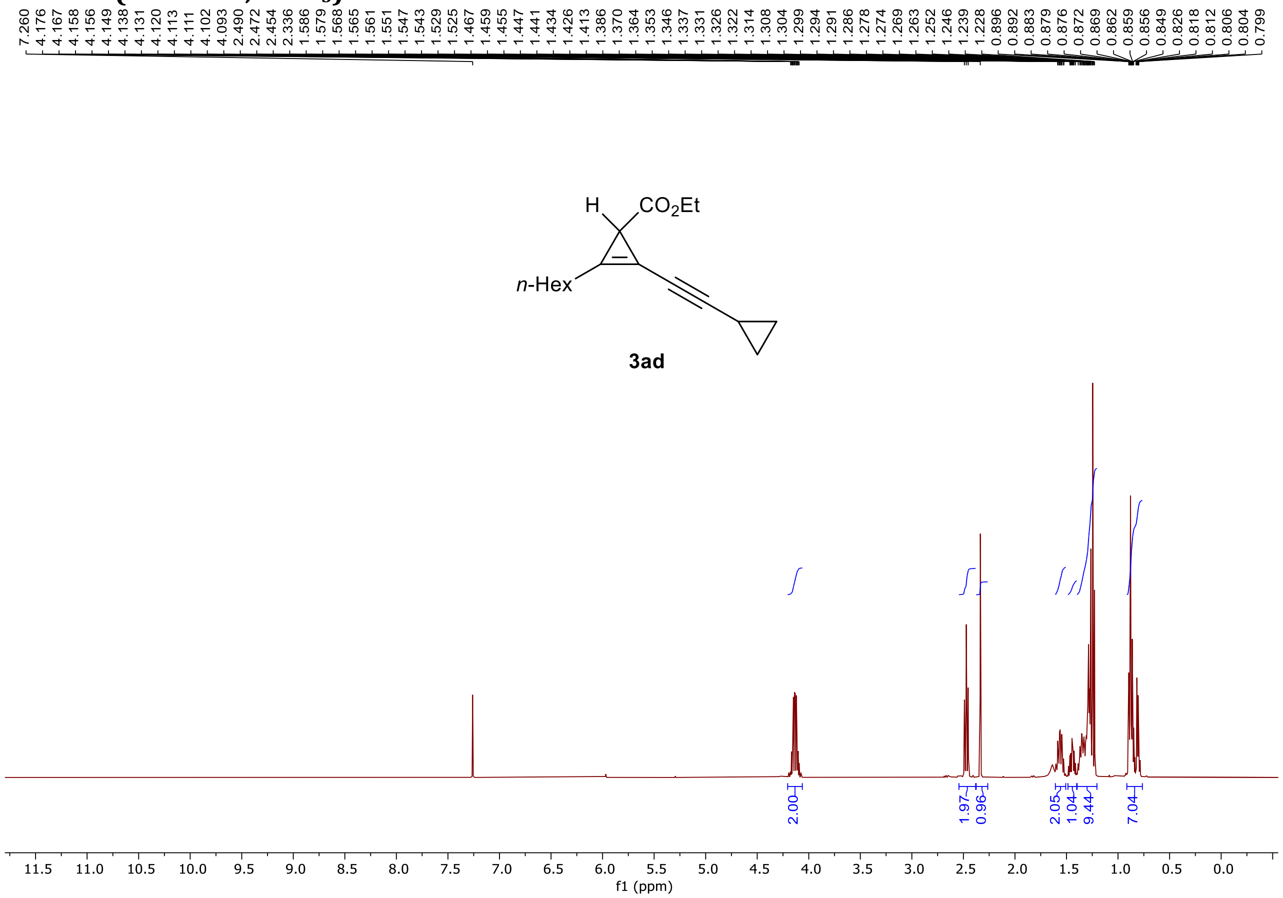

^{13}C NMR (101 MHz, $CDCl_3$) of 3ad

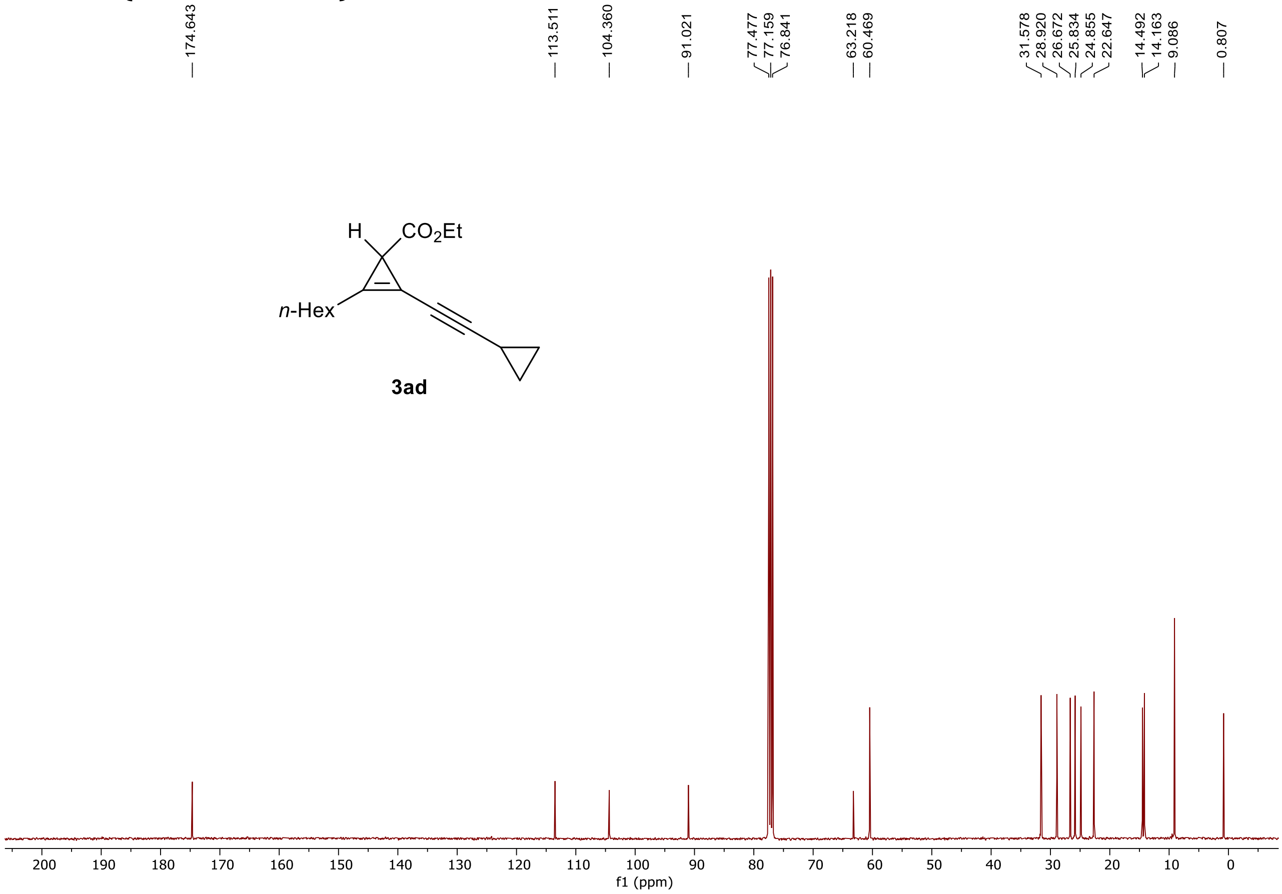

^{1}H NMR (400 MHz, $CDCl_3$) of 3ae

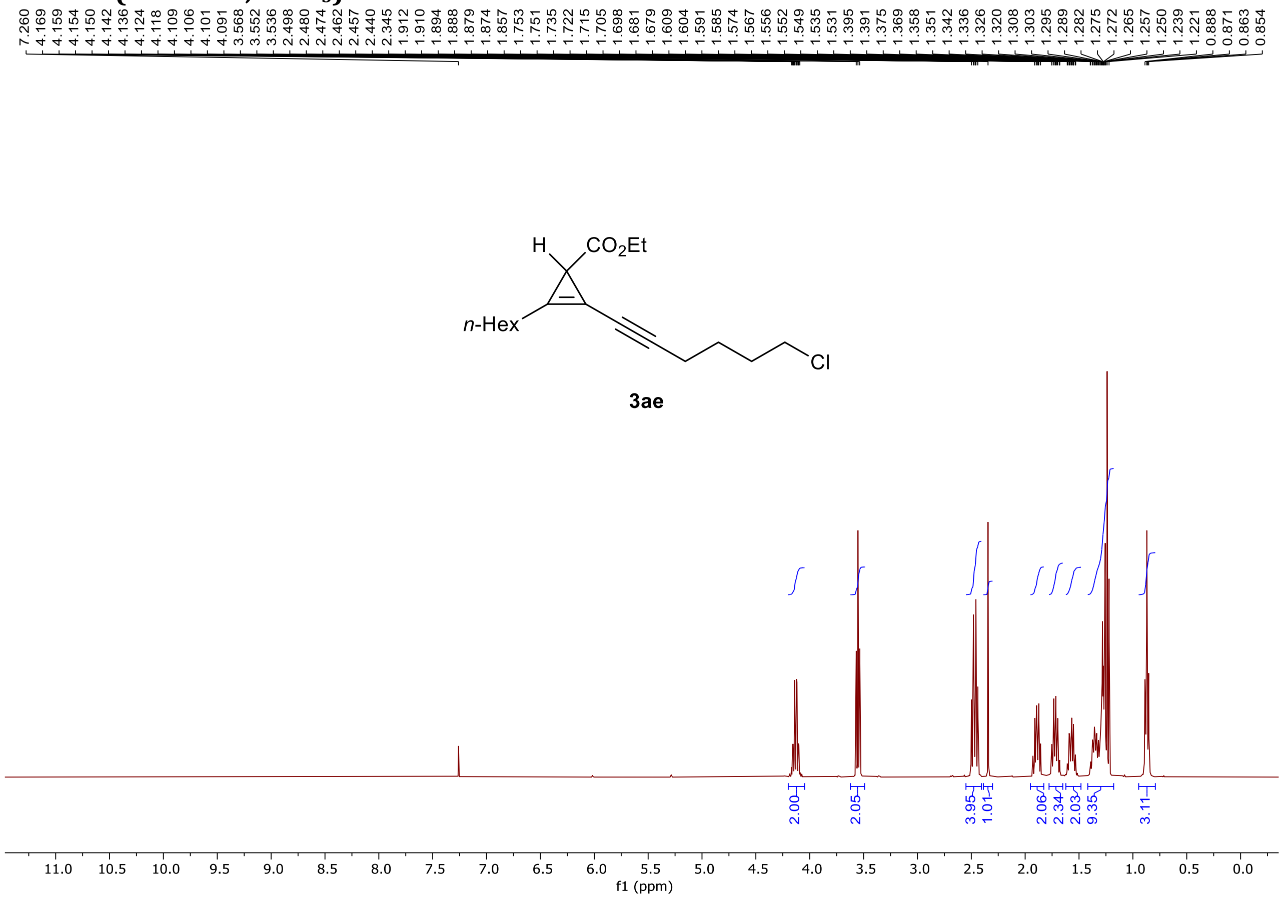

^{13}C NMR (101 MHz, $CDCl_3$) of 3ae

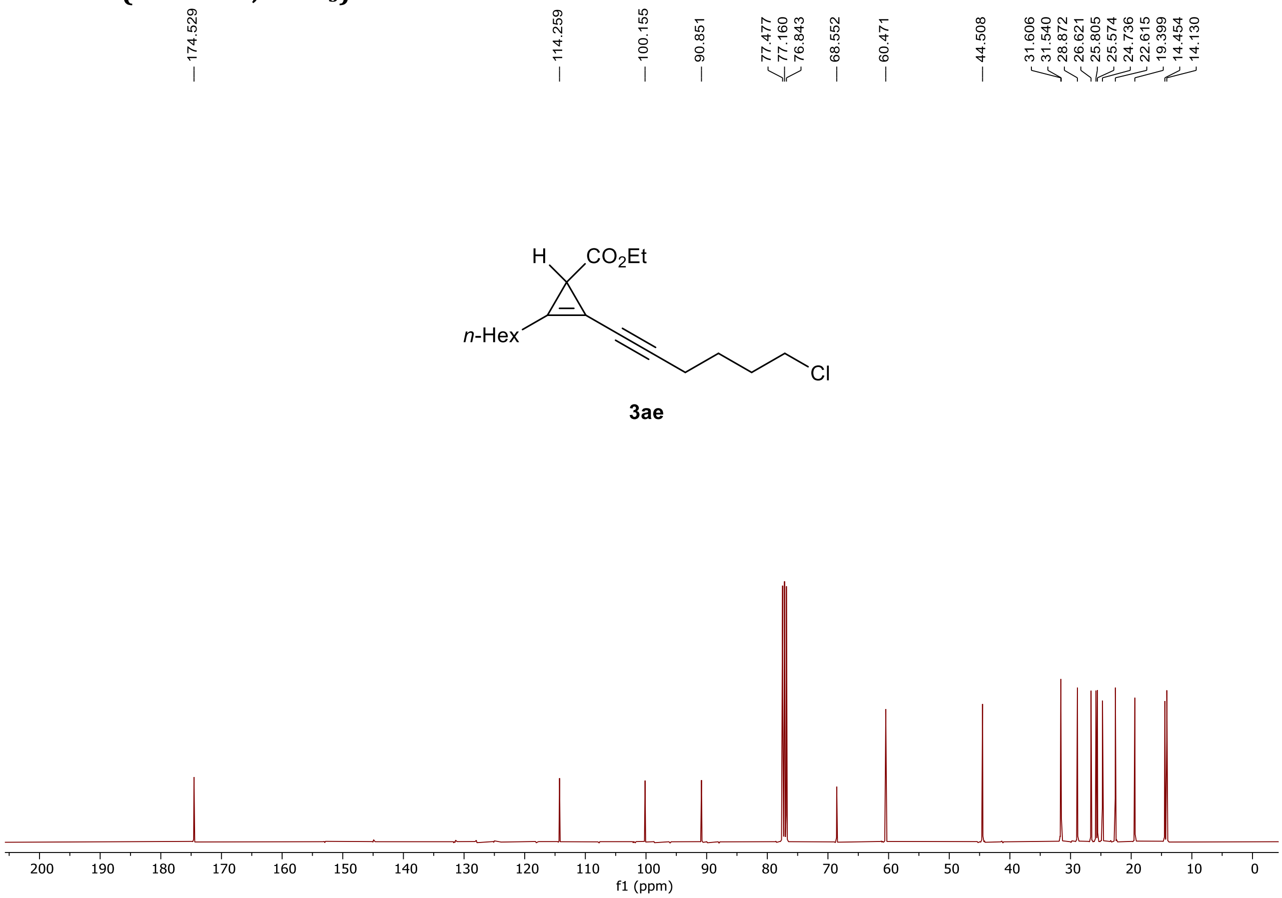

^{1}H NMR (400 MHz, $CDCl_3$) of 3af

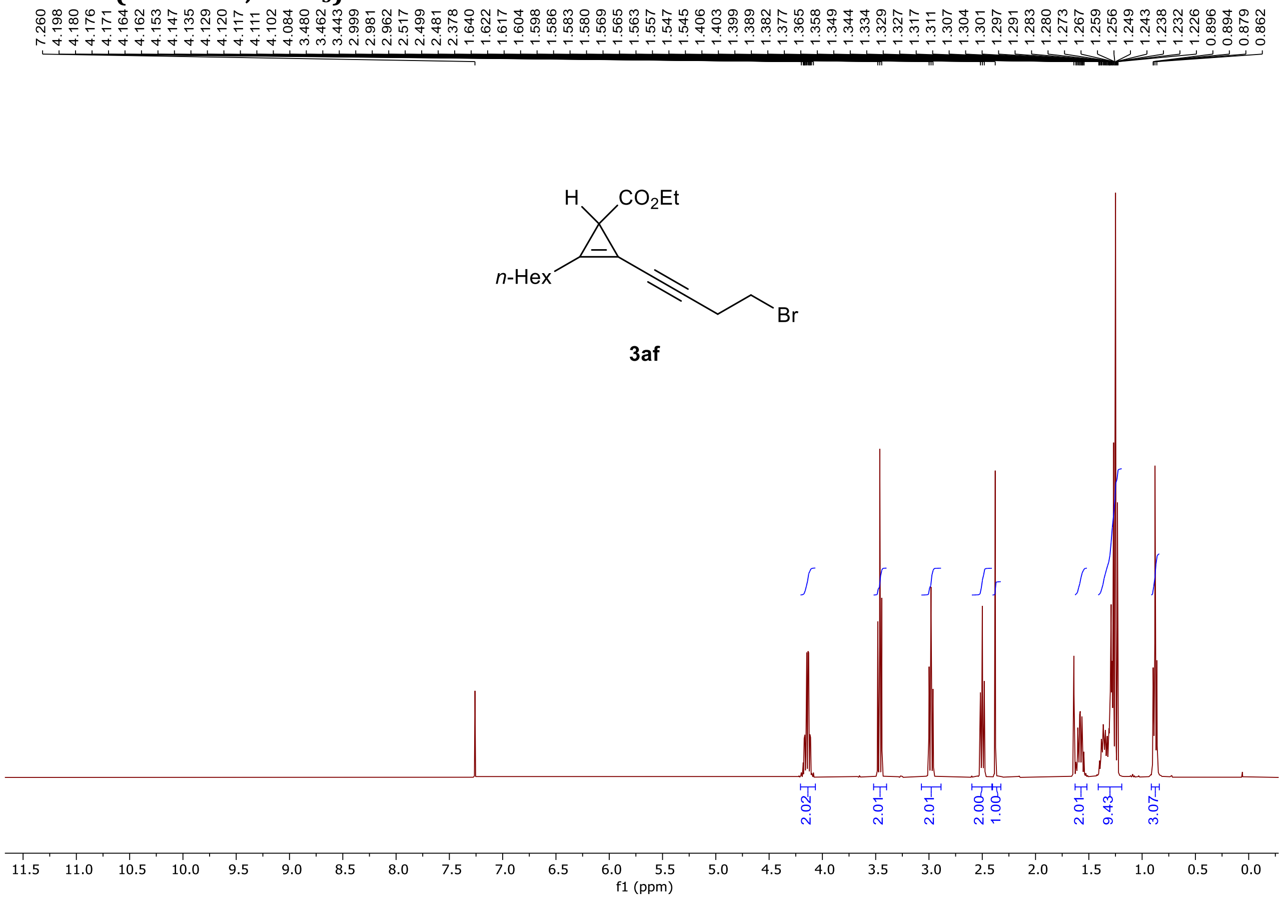

^{13}C NMR (101 MHz, $CDCl_3$) of 3af

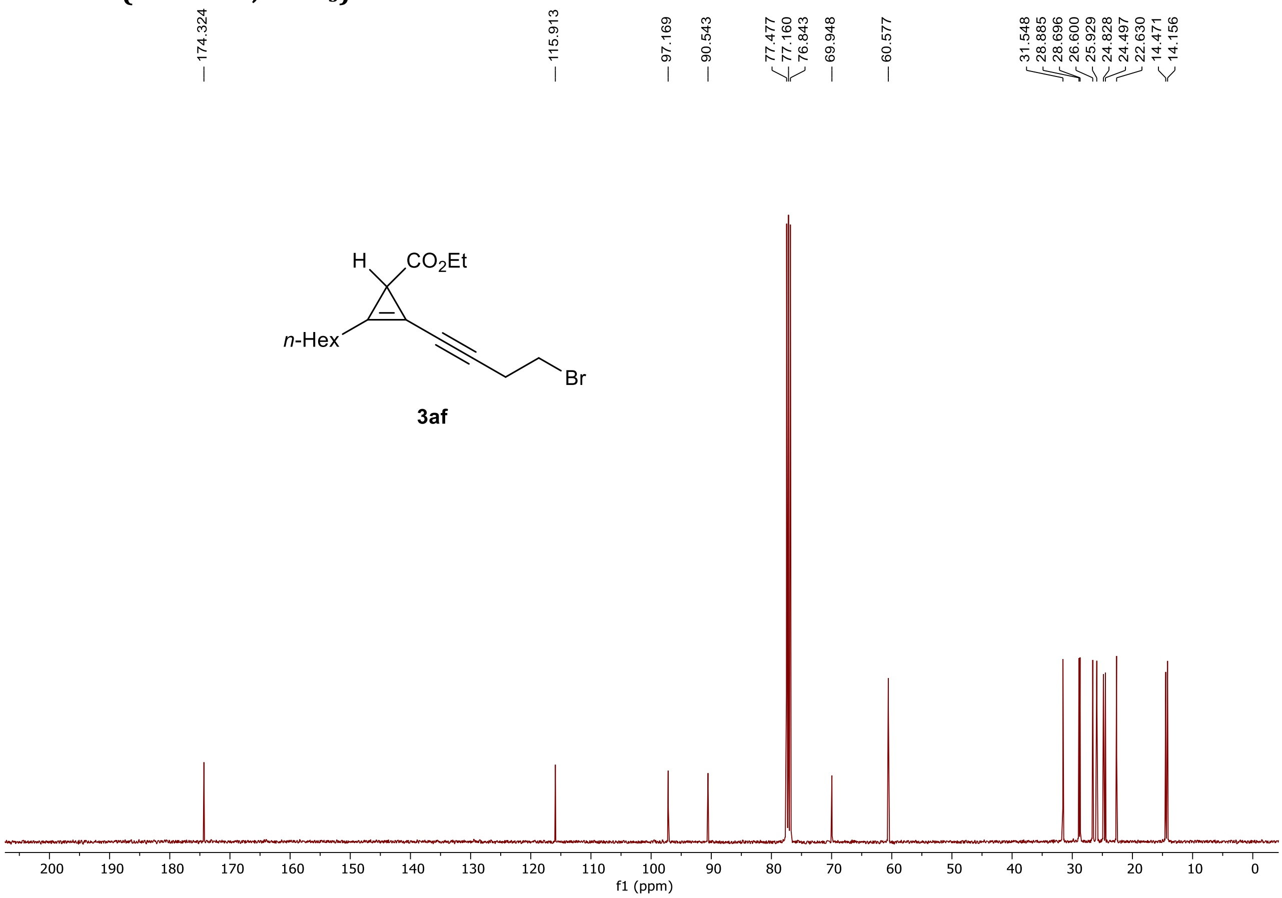

^{1}H NMR (400 MHz, $CDCl_3$) of 3ag

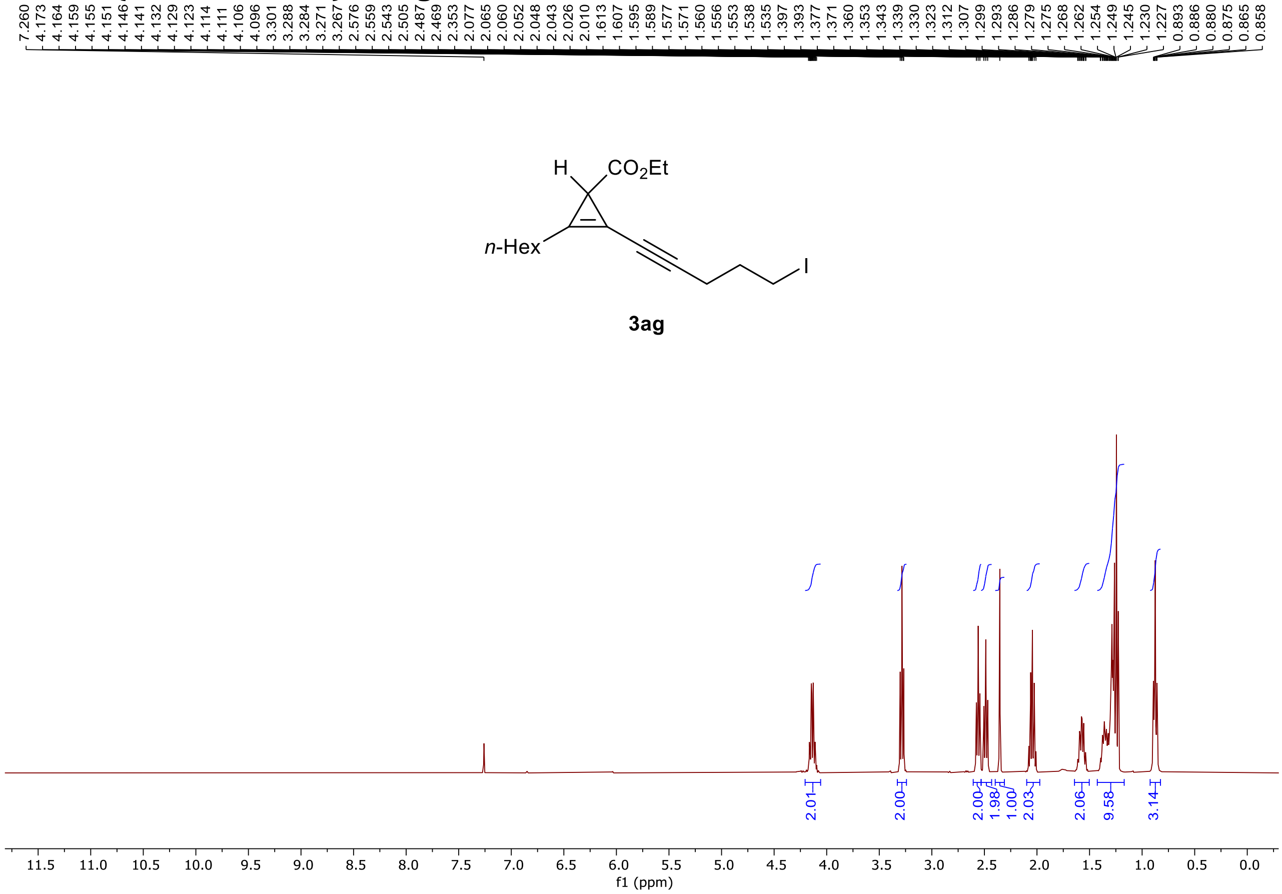

^{13}C NMR (101 MHz, $CDCl_3$) of 3ag

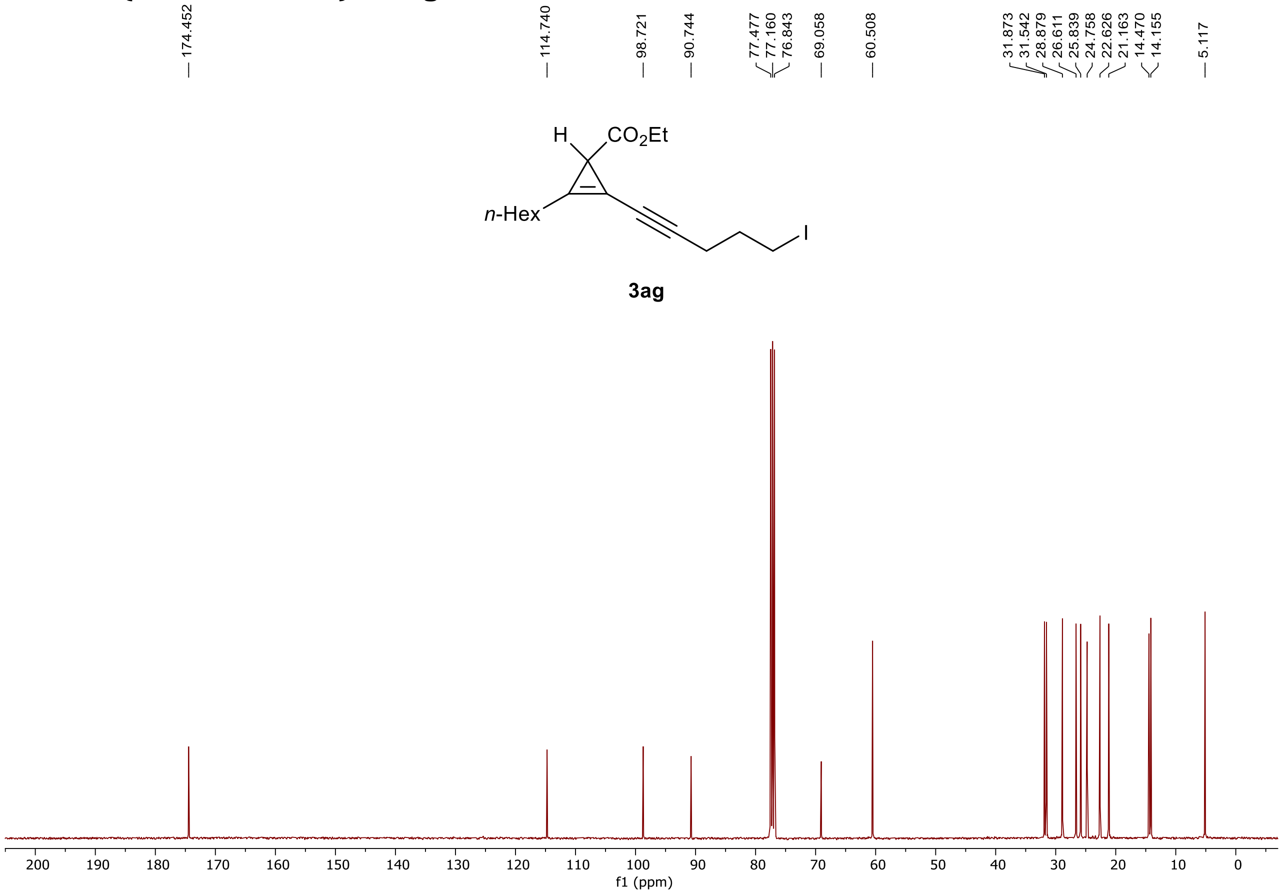

^{1}H NMR (400 MHz, $CDCl_3$) of 3ah

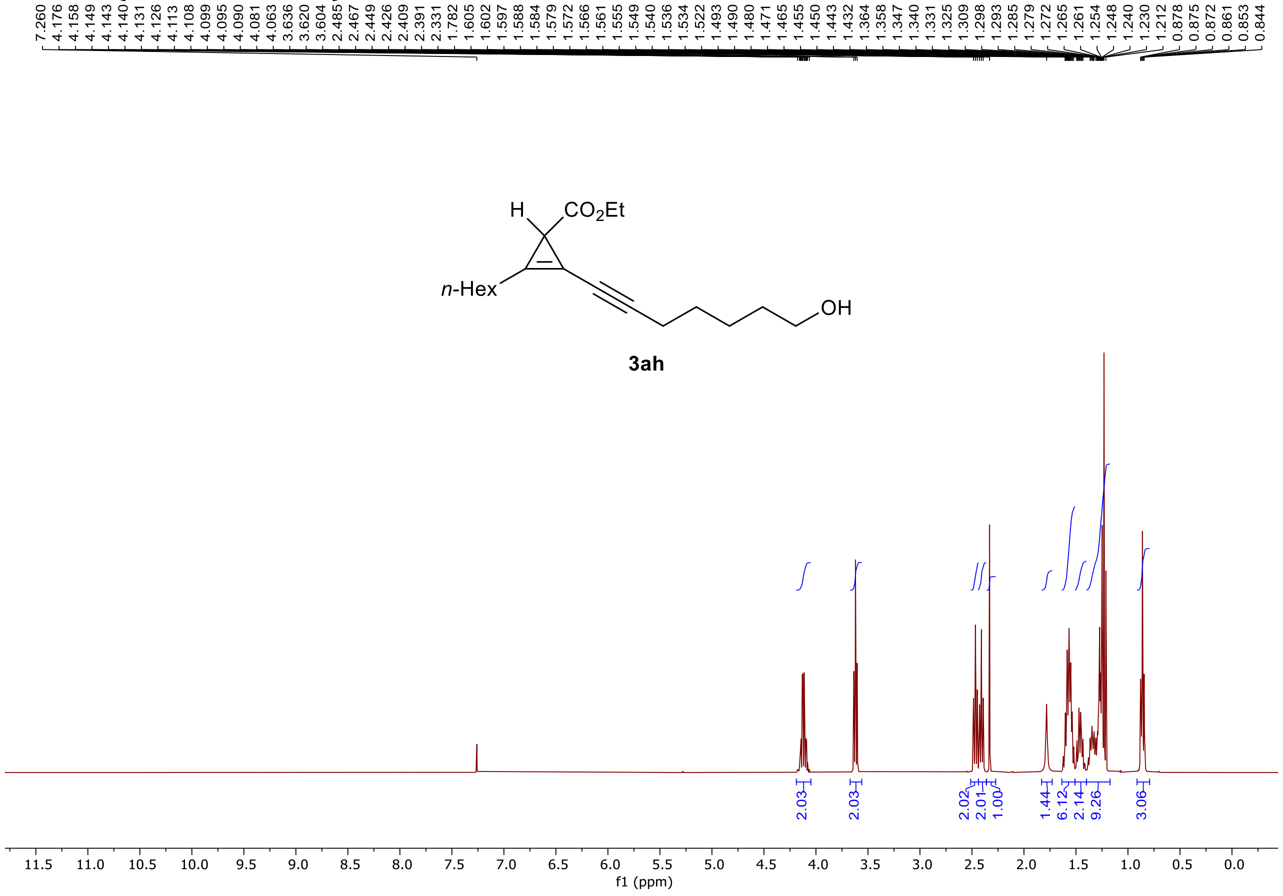

^{13}C NMR (101 MHz, $CDCl_3$) of 3ah

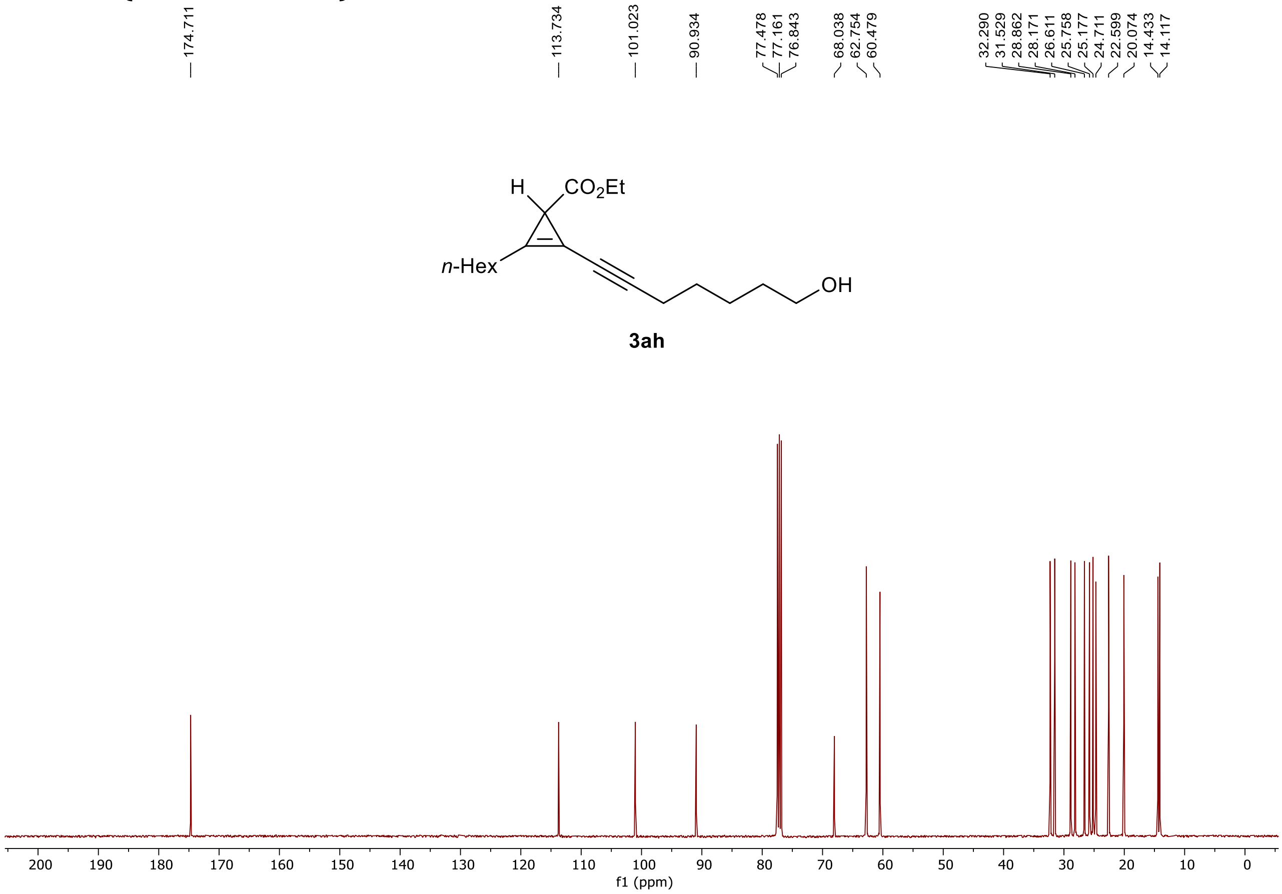

^{1}H NMR (400 MHz, $CDCl_3$) of 3ai

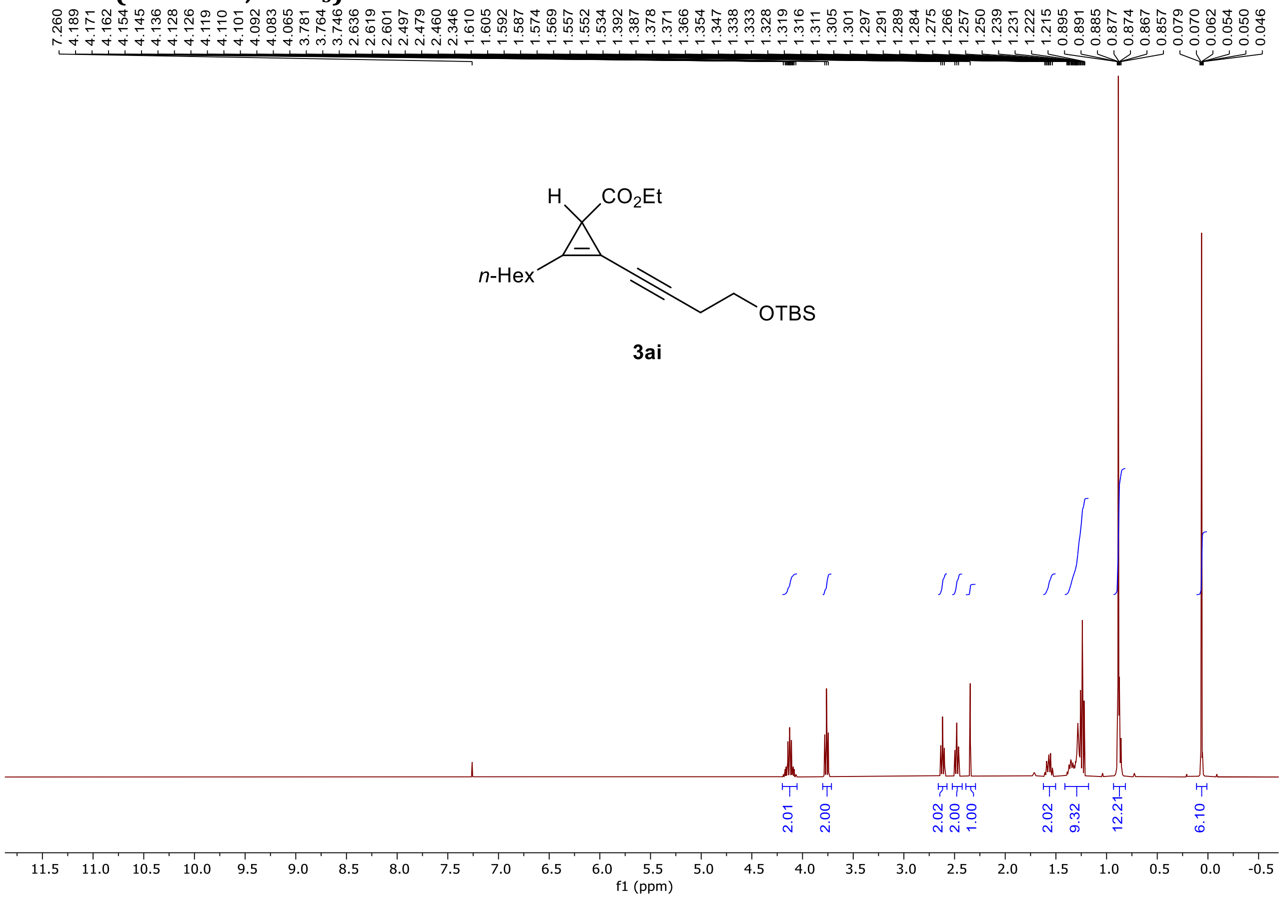

^{13}C NMR (101 MHz, $CDCl_3$) of 3ai

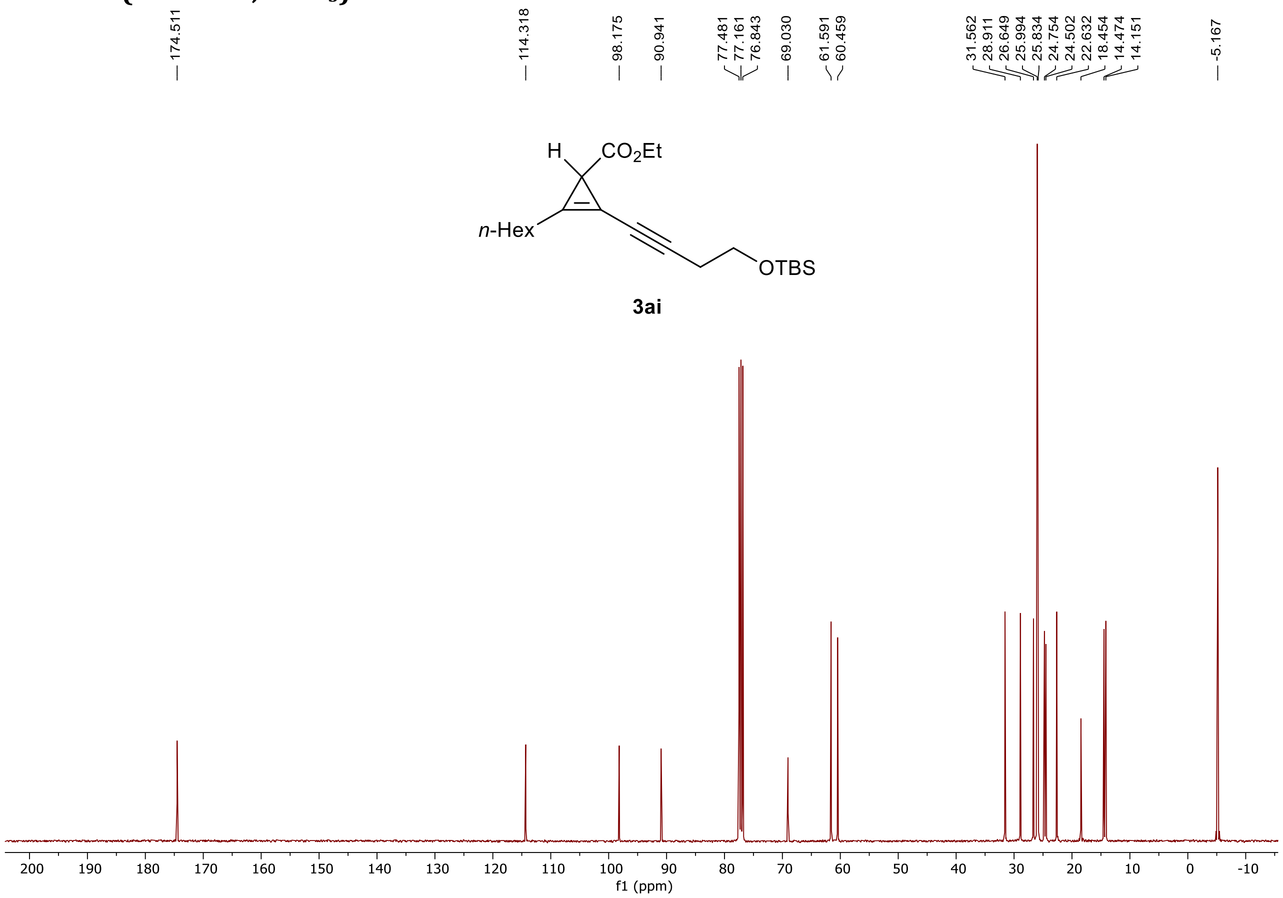

^{1}H NMR (400 MHz, $CDCl_3$) of 3aj

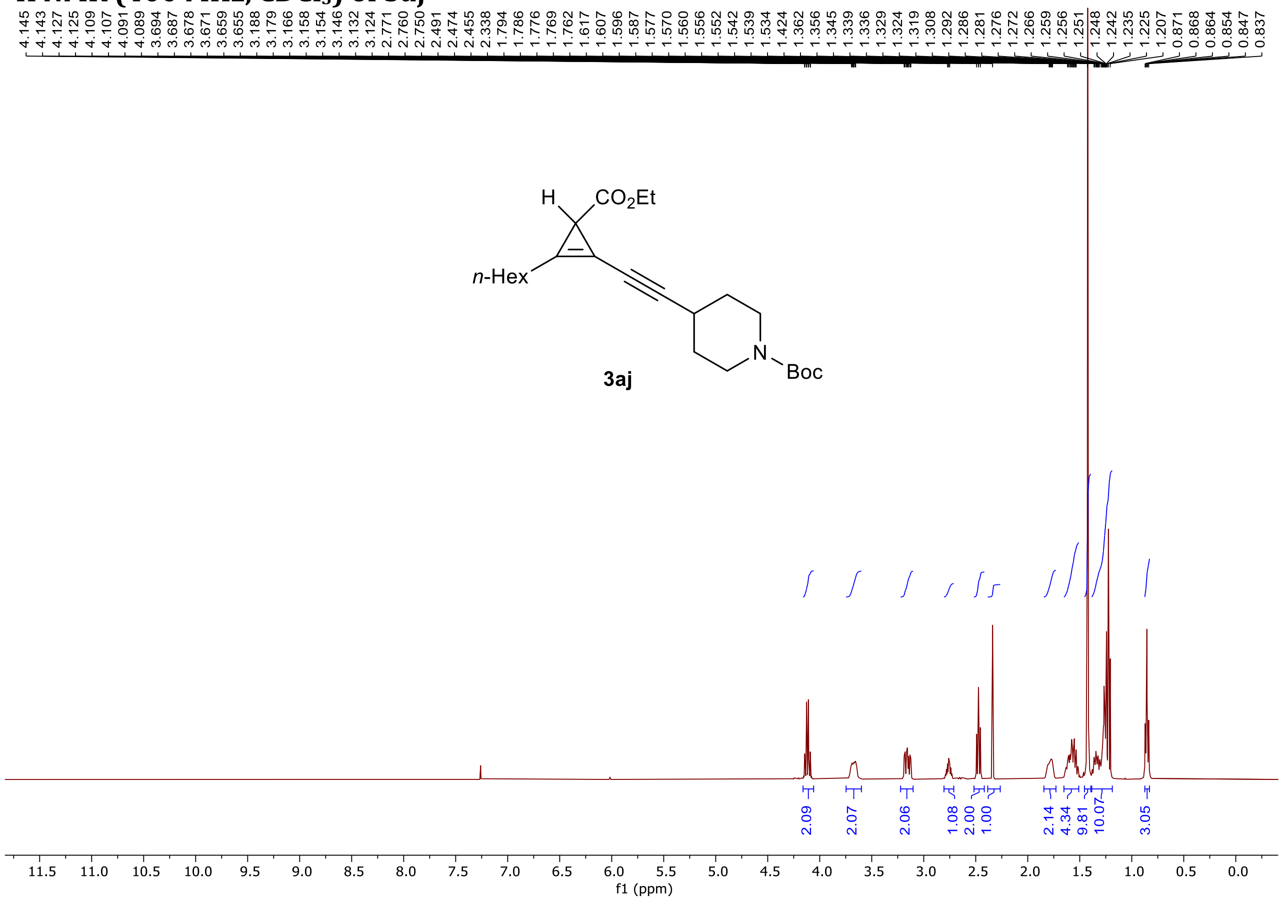

^{13}C NMR (101 MHz, $CDCl_3$) of 3aj

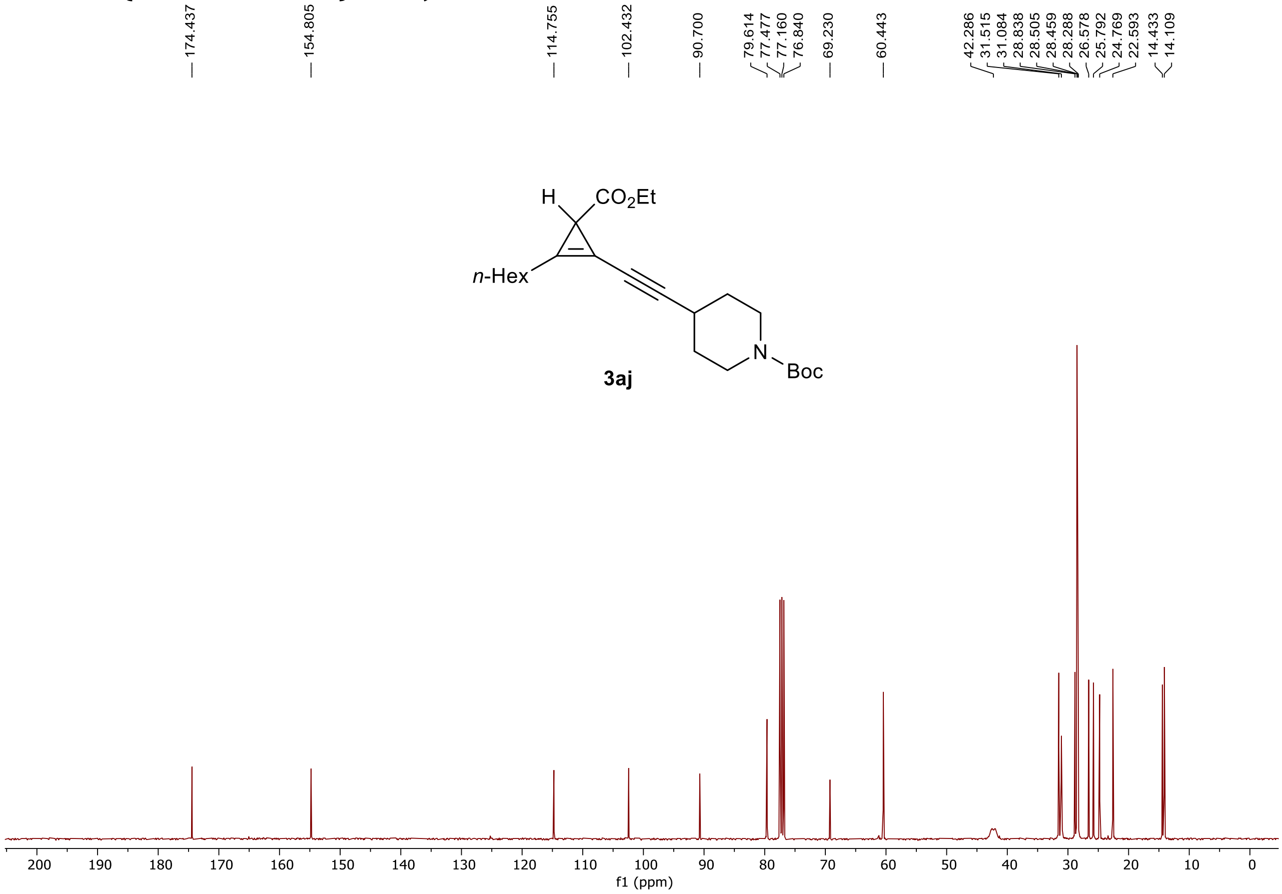

^{1}H NMR (400 MHz, $CDCl_3$) of 3ak

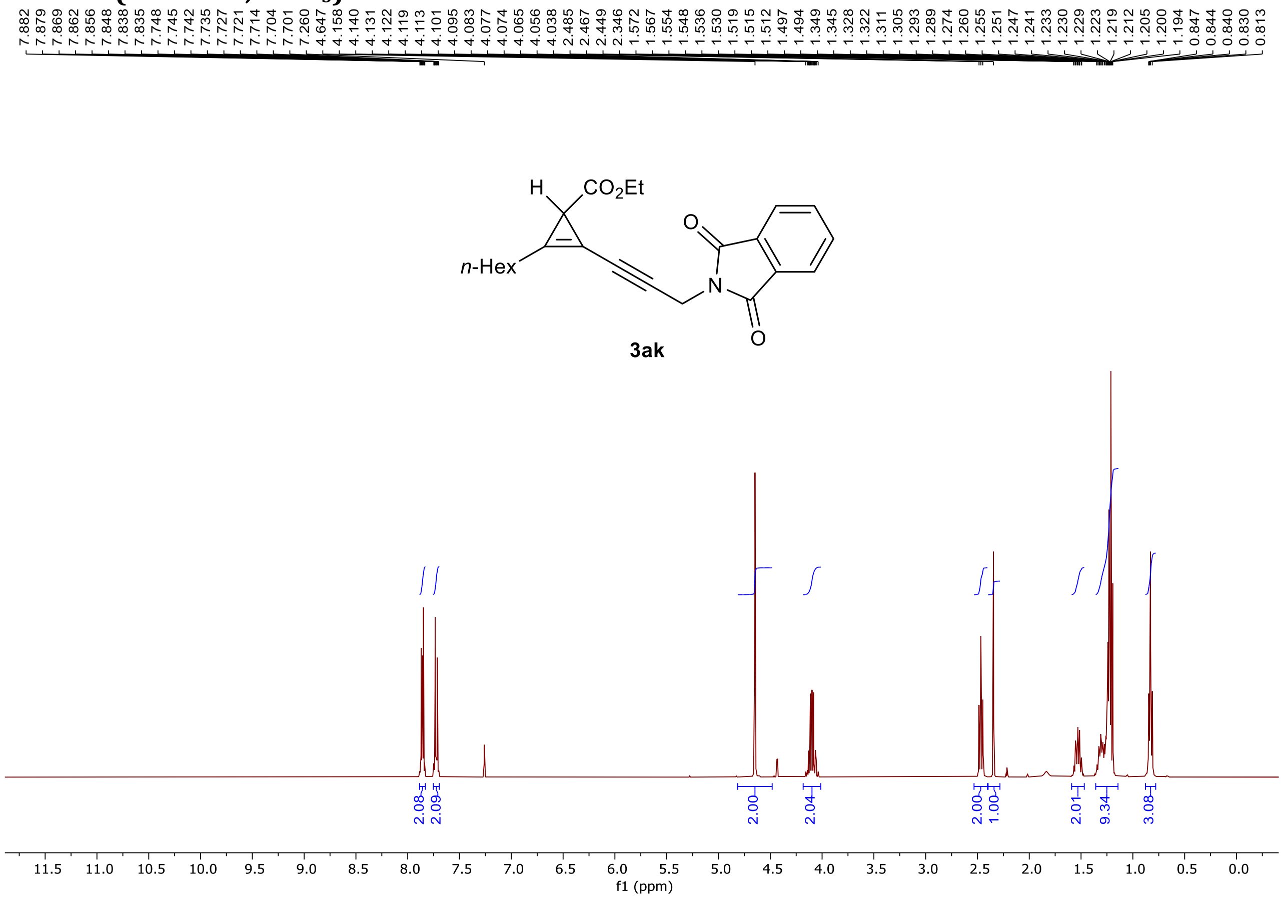

^{13}C NMR (101 MHz, $CDCl_3$) of 3ak

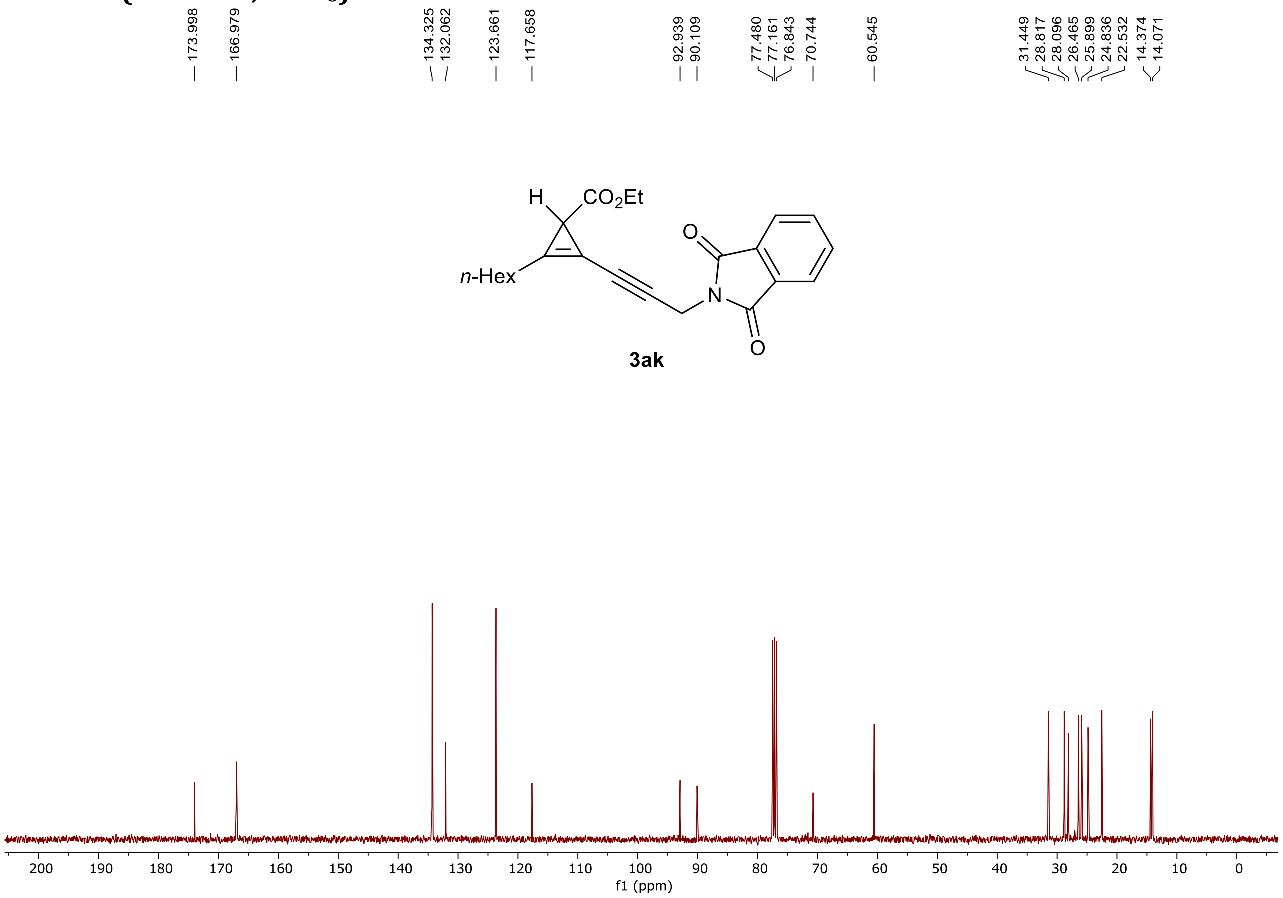

^{1}H NMR (400 MHz, $CDCl_3$) of 3al

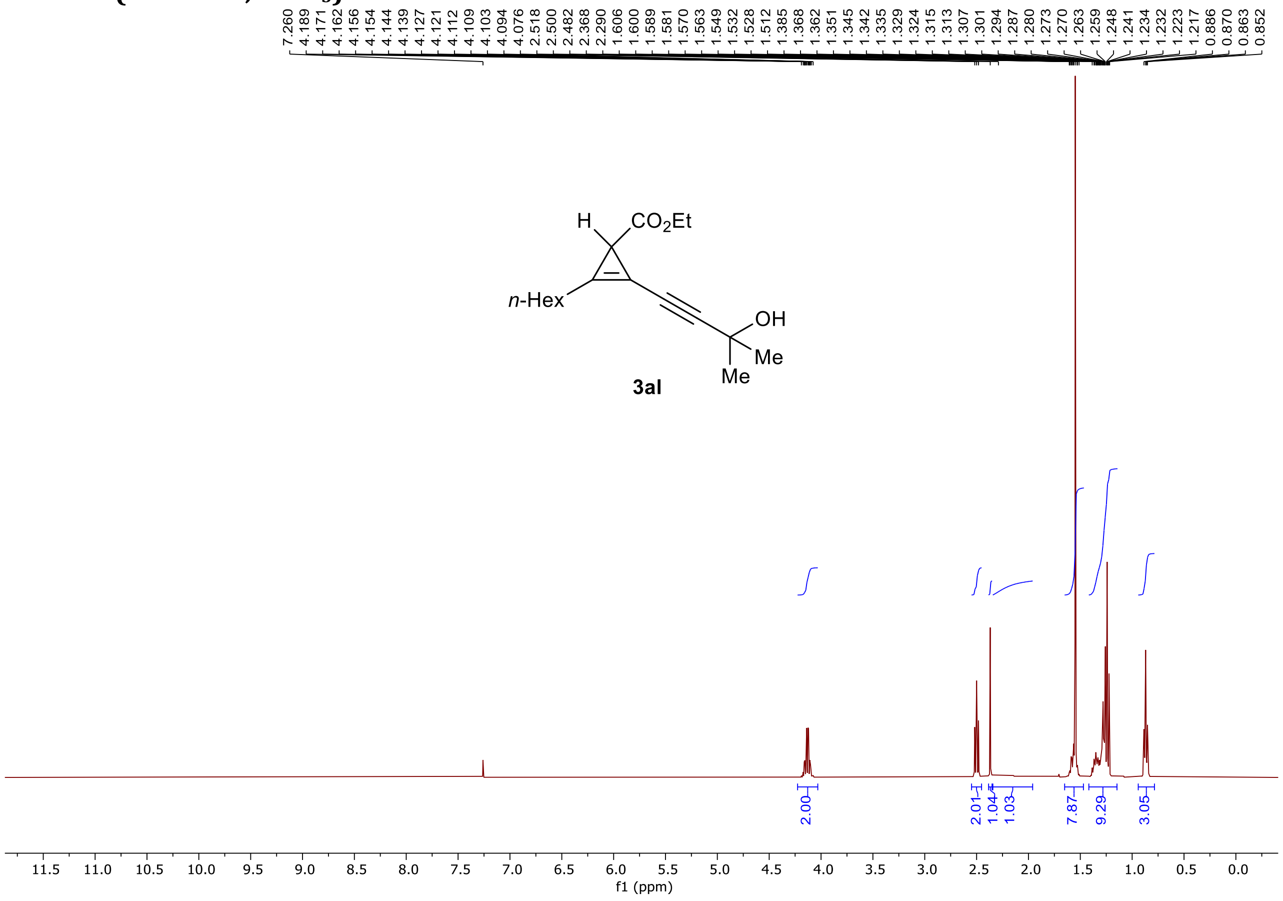

^{13}C NMR (101 MHz, $CDCl_3$) of 3al

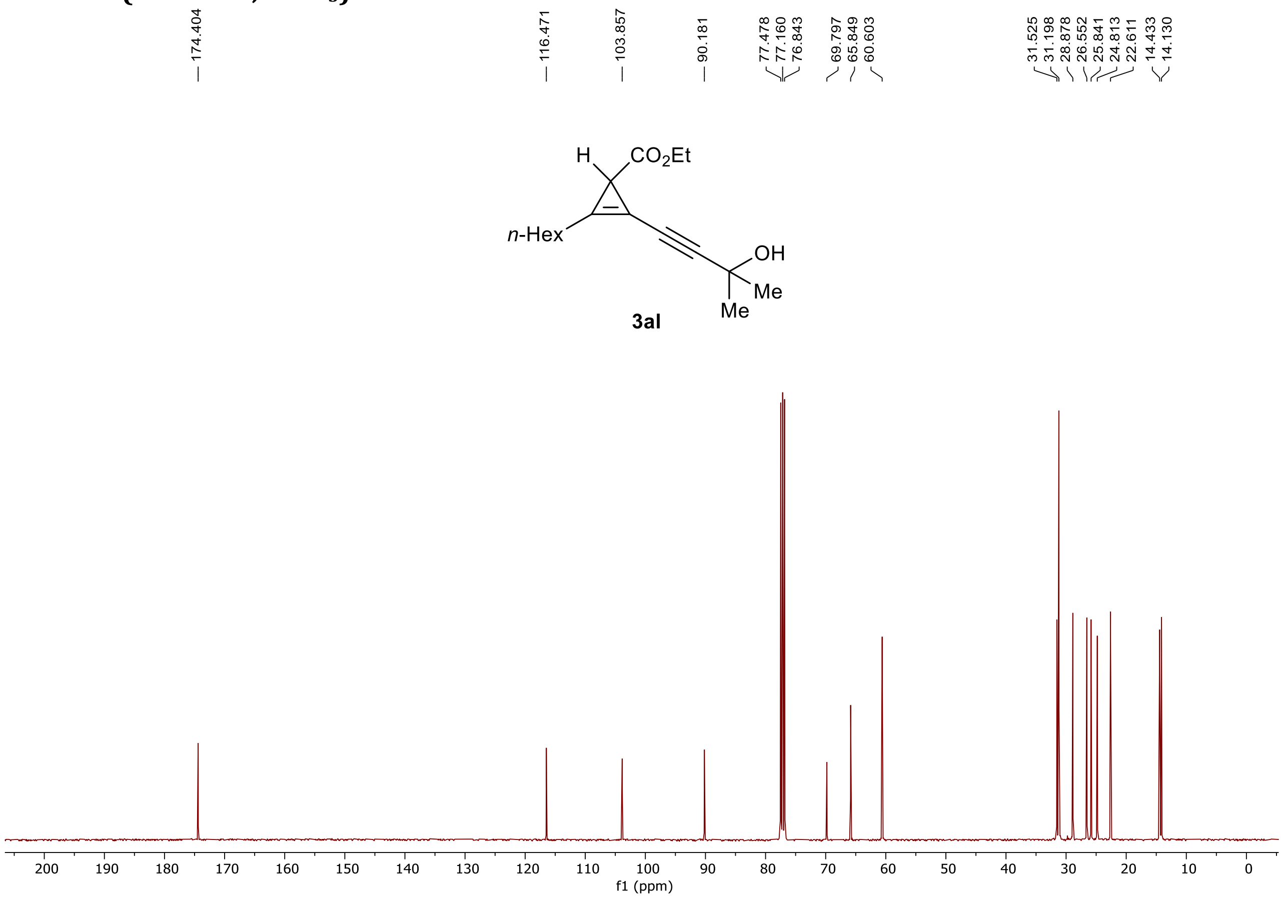

^{1}H NMR (400 MHz, $CDCl_3$) of 3am

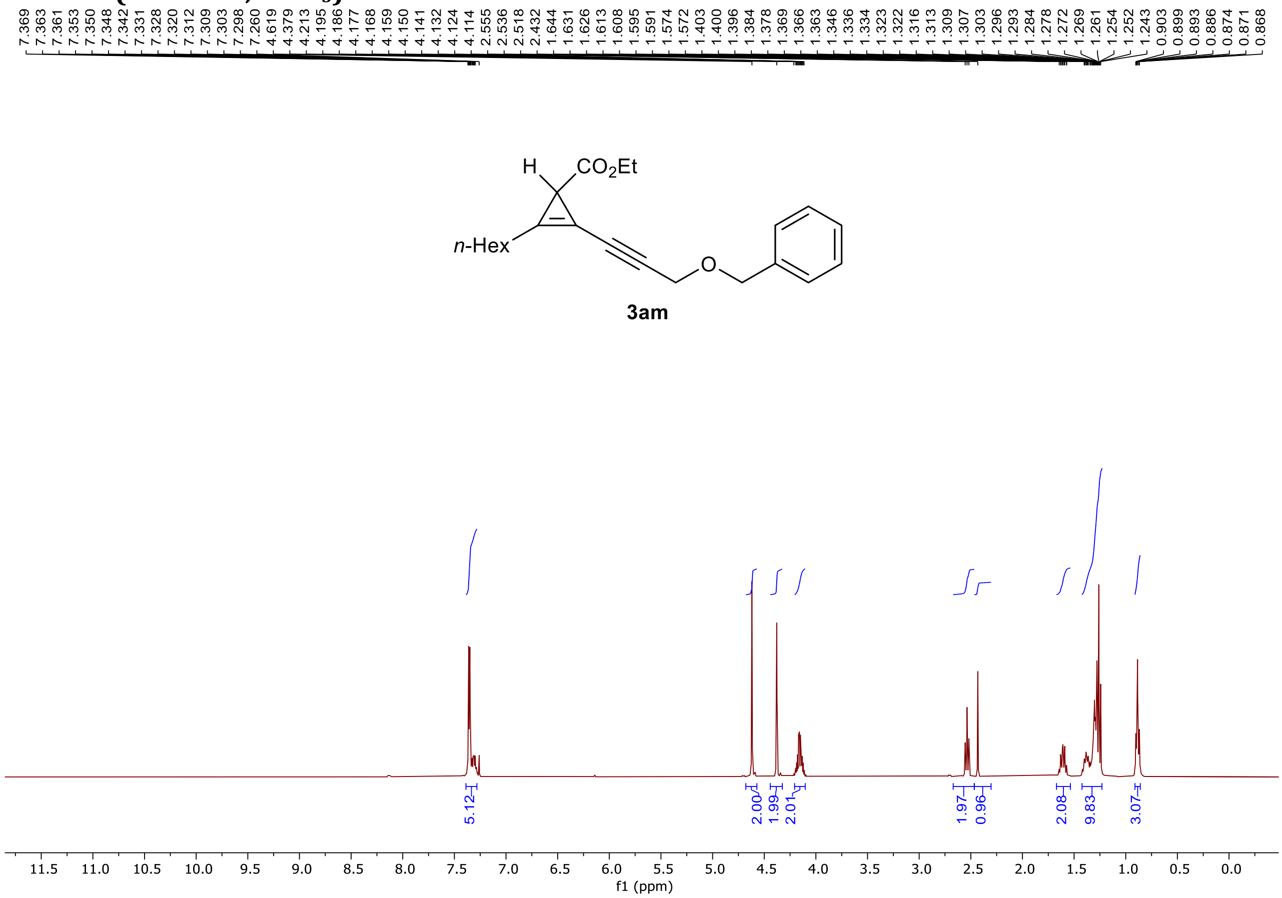

^{13}C NMR (101 MHz, $CDCl_3$) of 3am

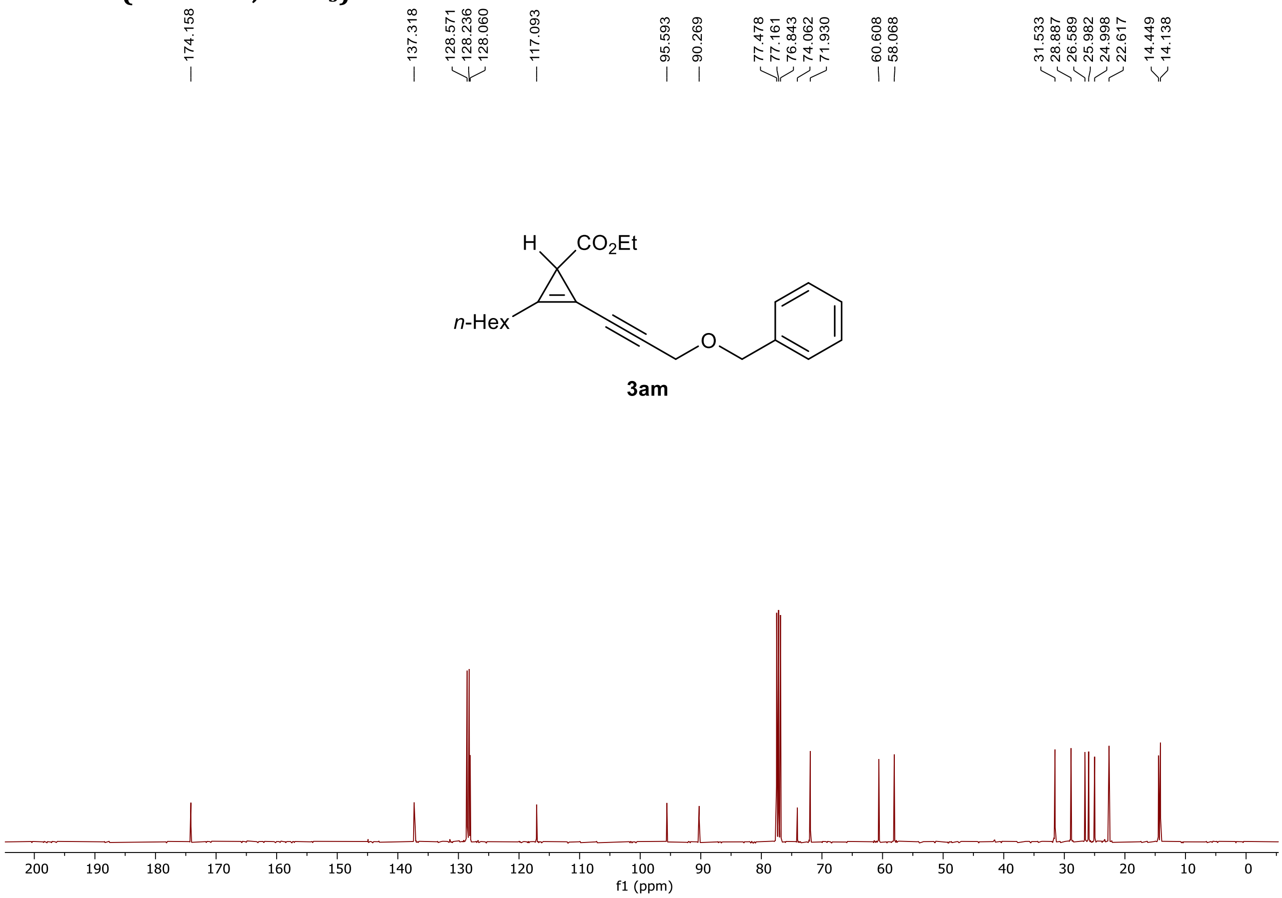

^{1}H NMR (400 MHz, $CDCl_3$) of 3an

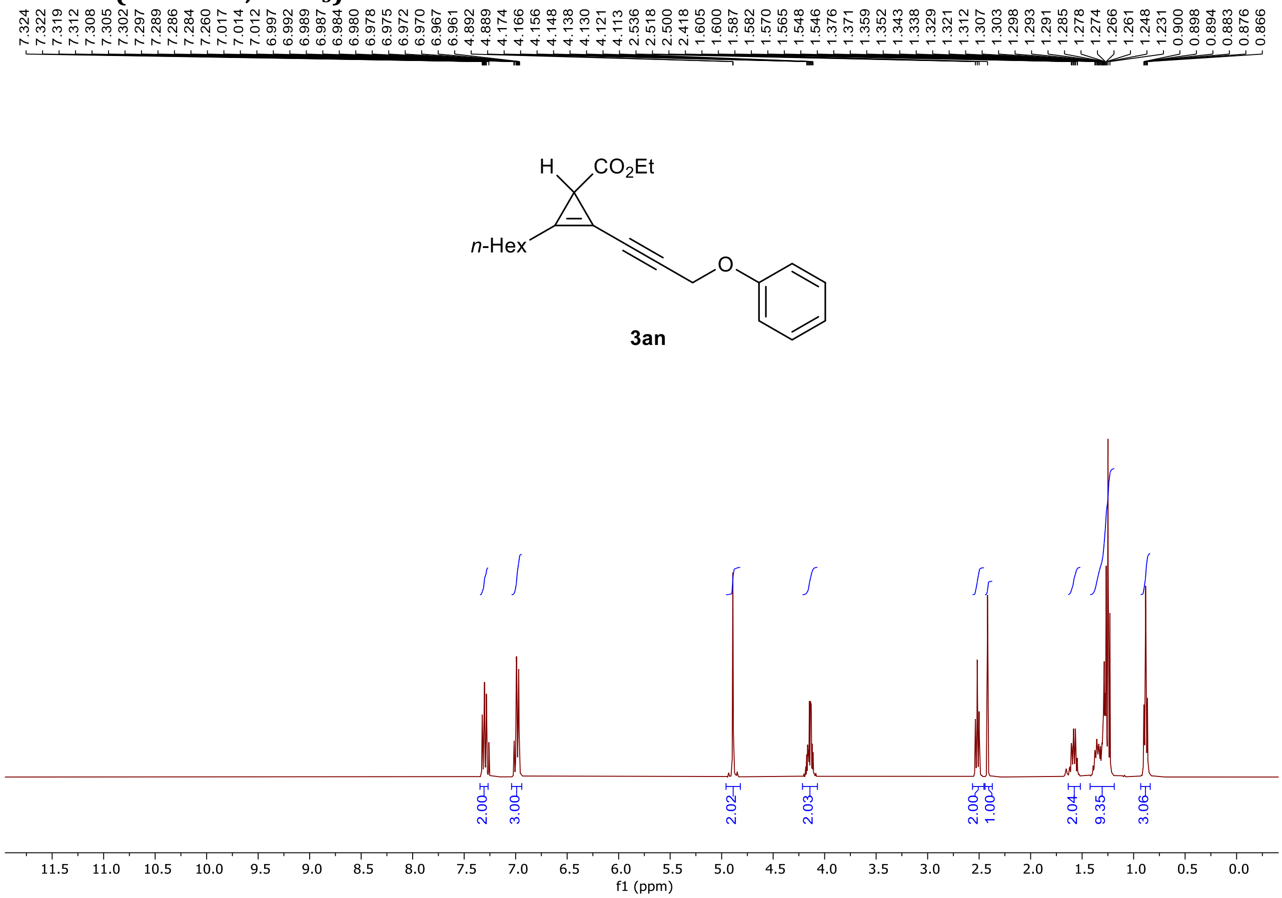

^{13}C NMR (101 MHz, $CDCl_3$) of 3an

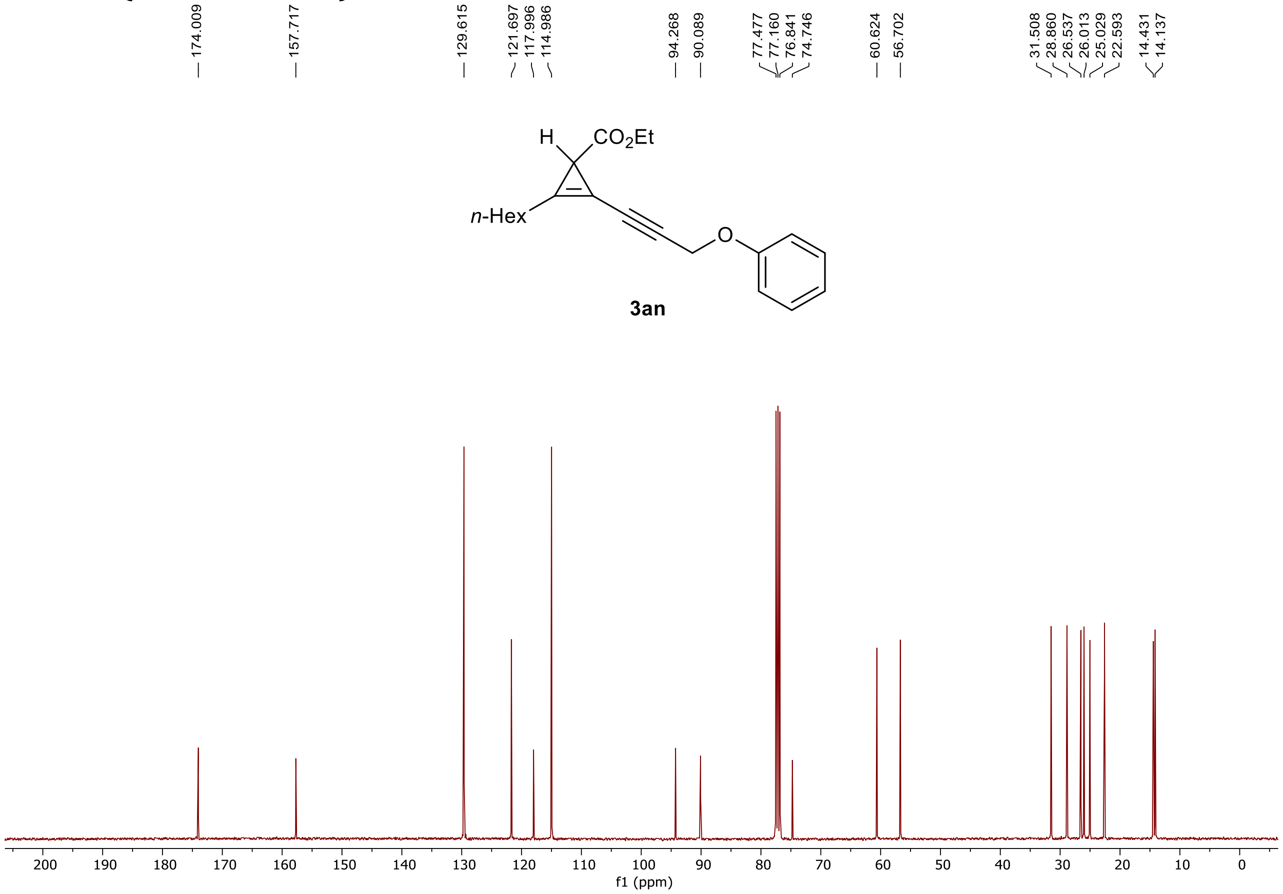

^{1}H NMR (400 MHz, $CDCl_3$) of 3ao

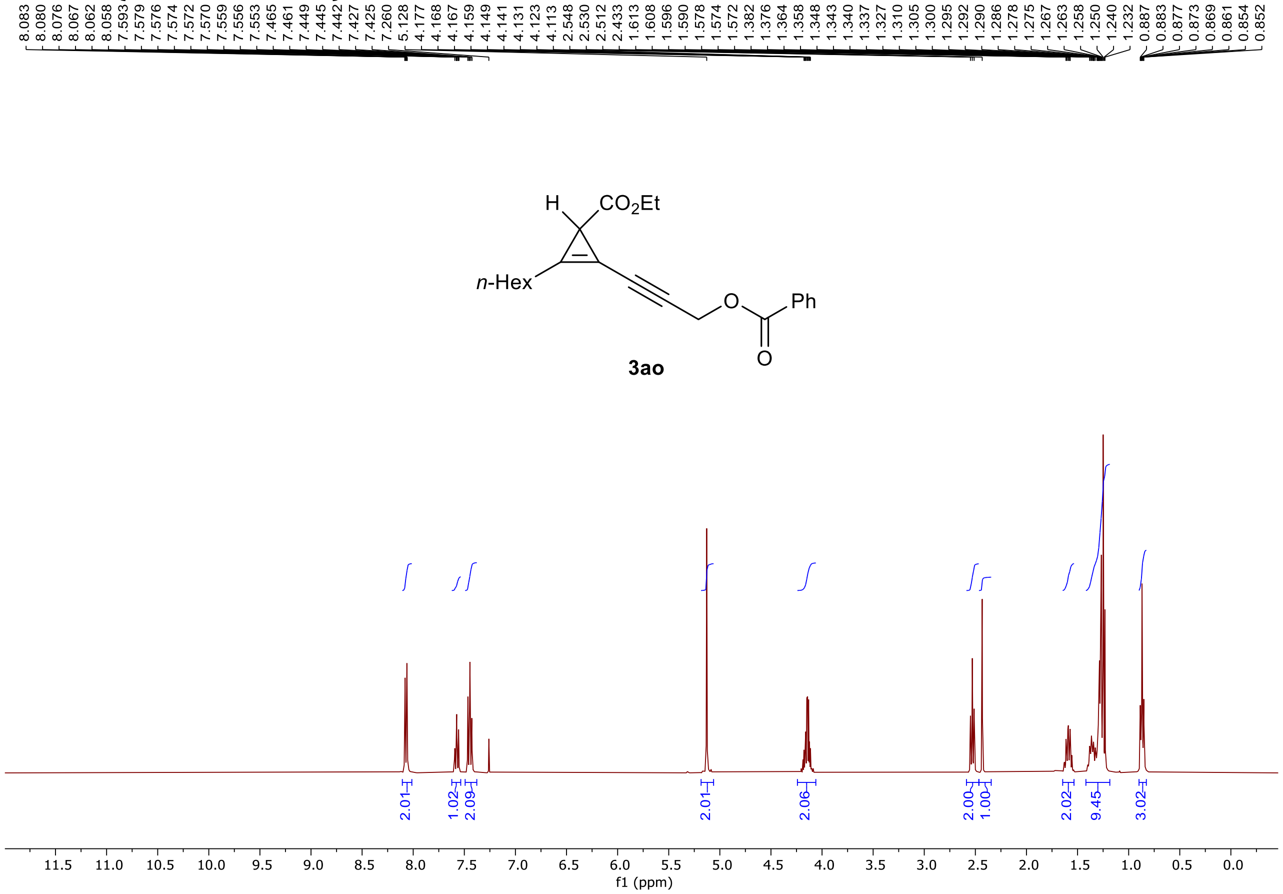

^{13}C NMR (101 MHz, $CDCl_3$) of 3ao

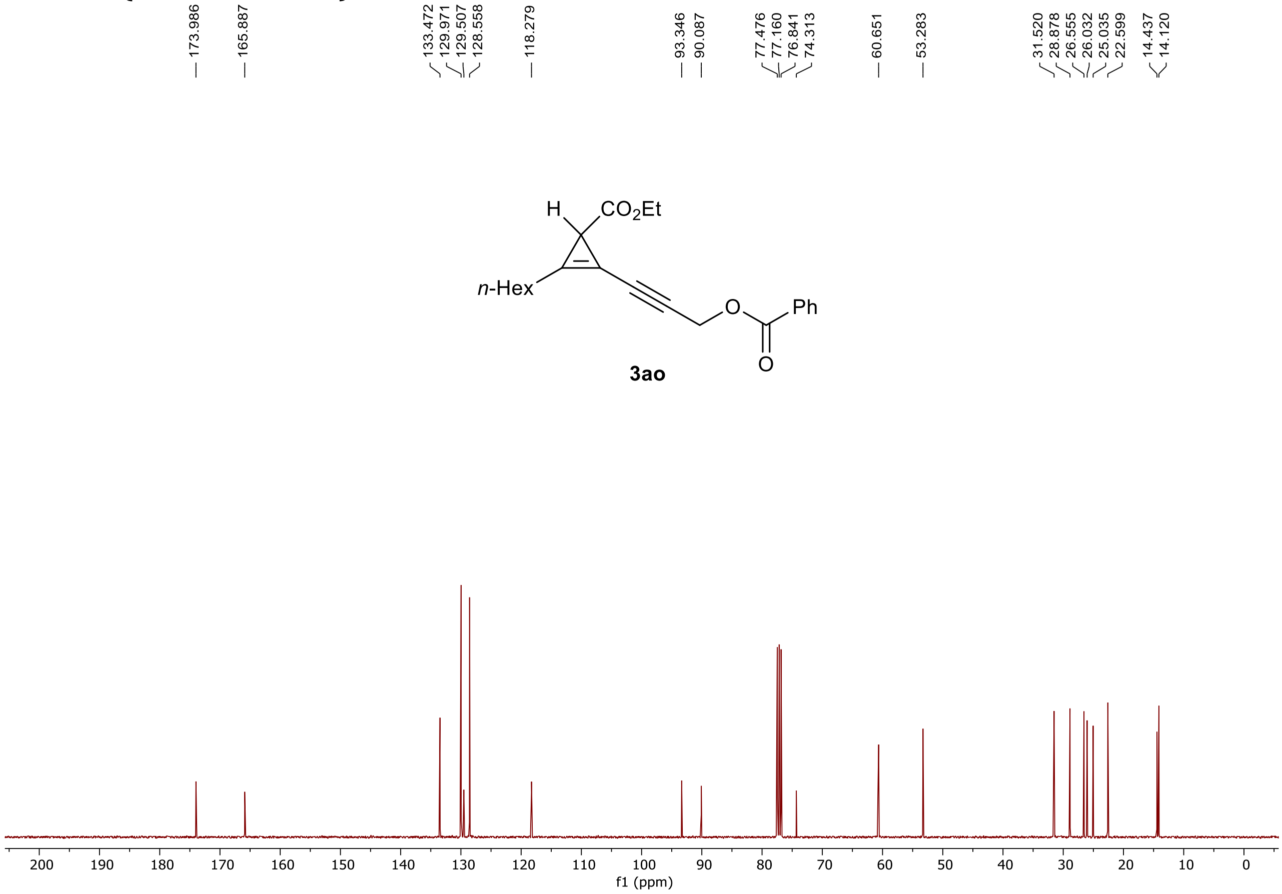

^{1}H NMR (400 MHz, $CDCl_3$) of 3ap

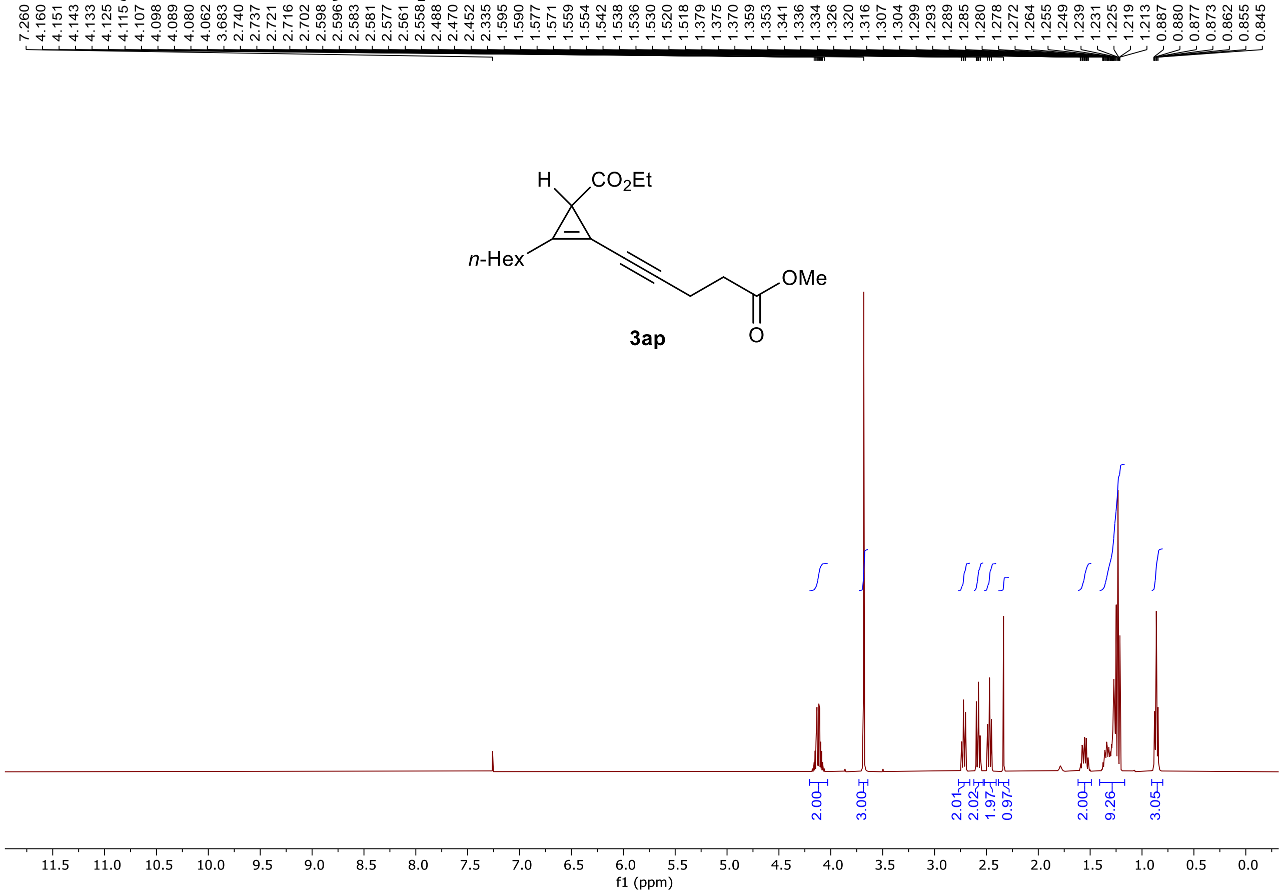

^{13}C NMR (101 MHz, $CDCl_3$) of 3ap

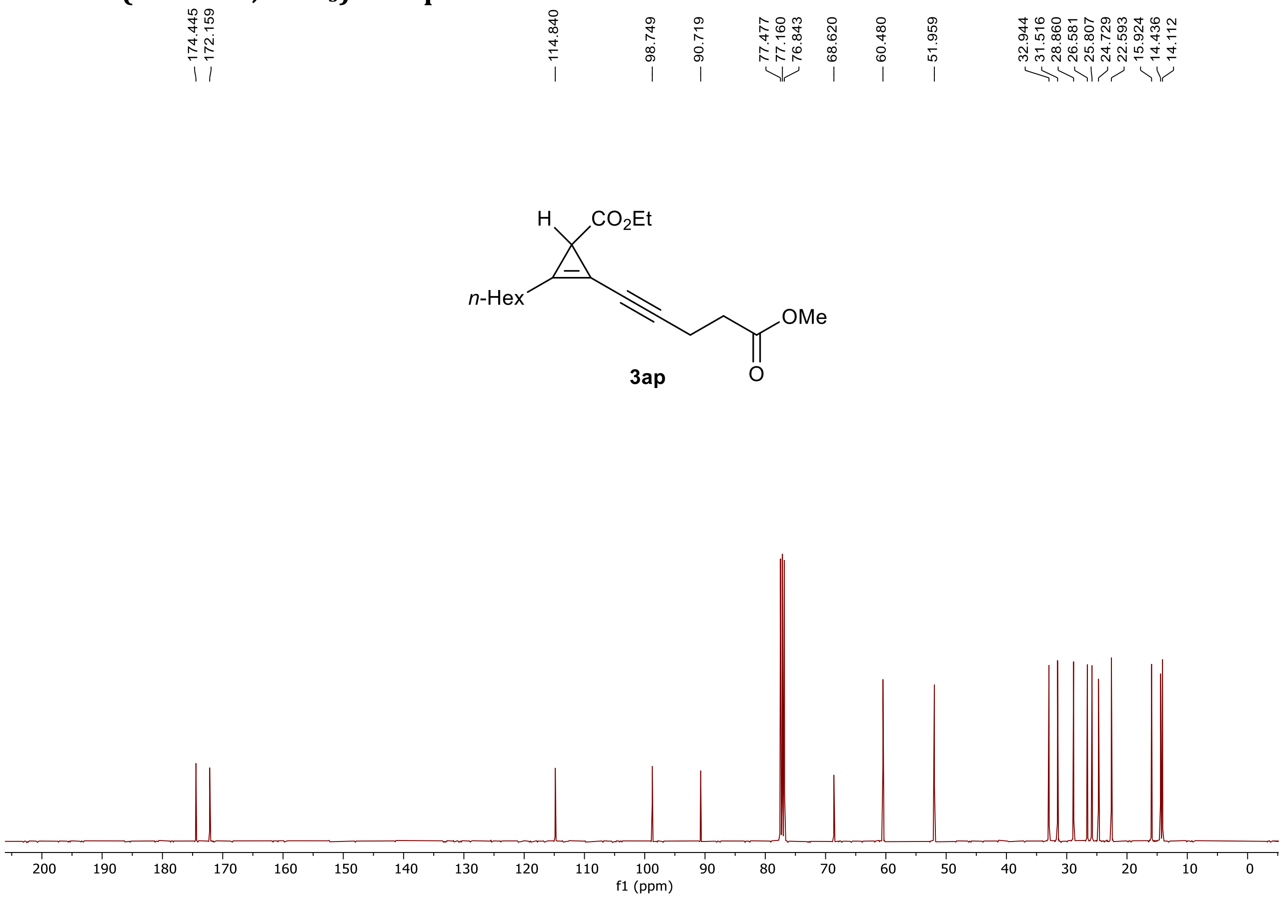

3ap

^{1}H NMR (400 MHz, $CDCl_3$) of 3aq

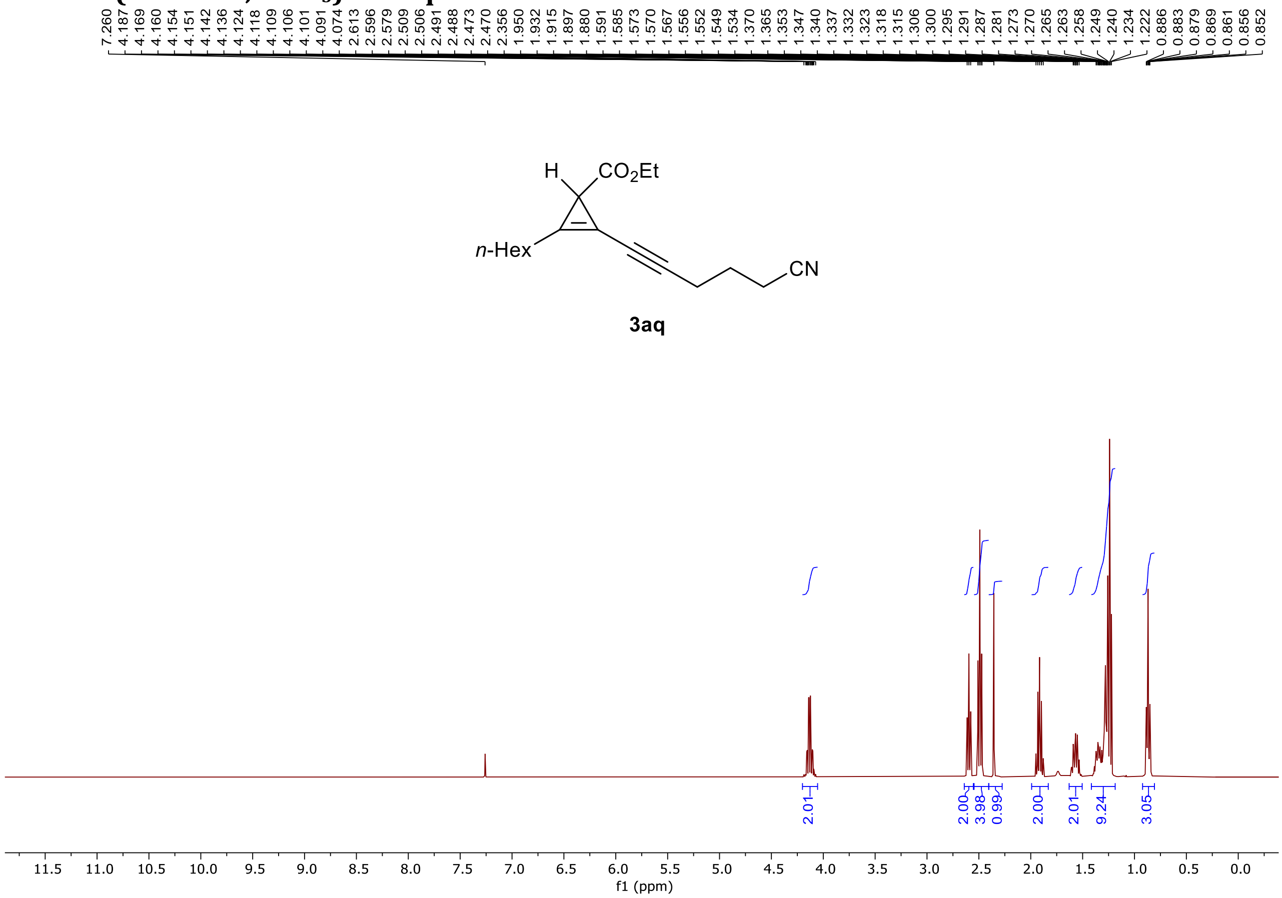

3aq

^{13}C NMR (101 MHz, $CDCl_3$) of 3aq

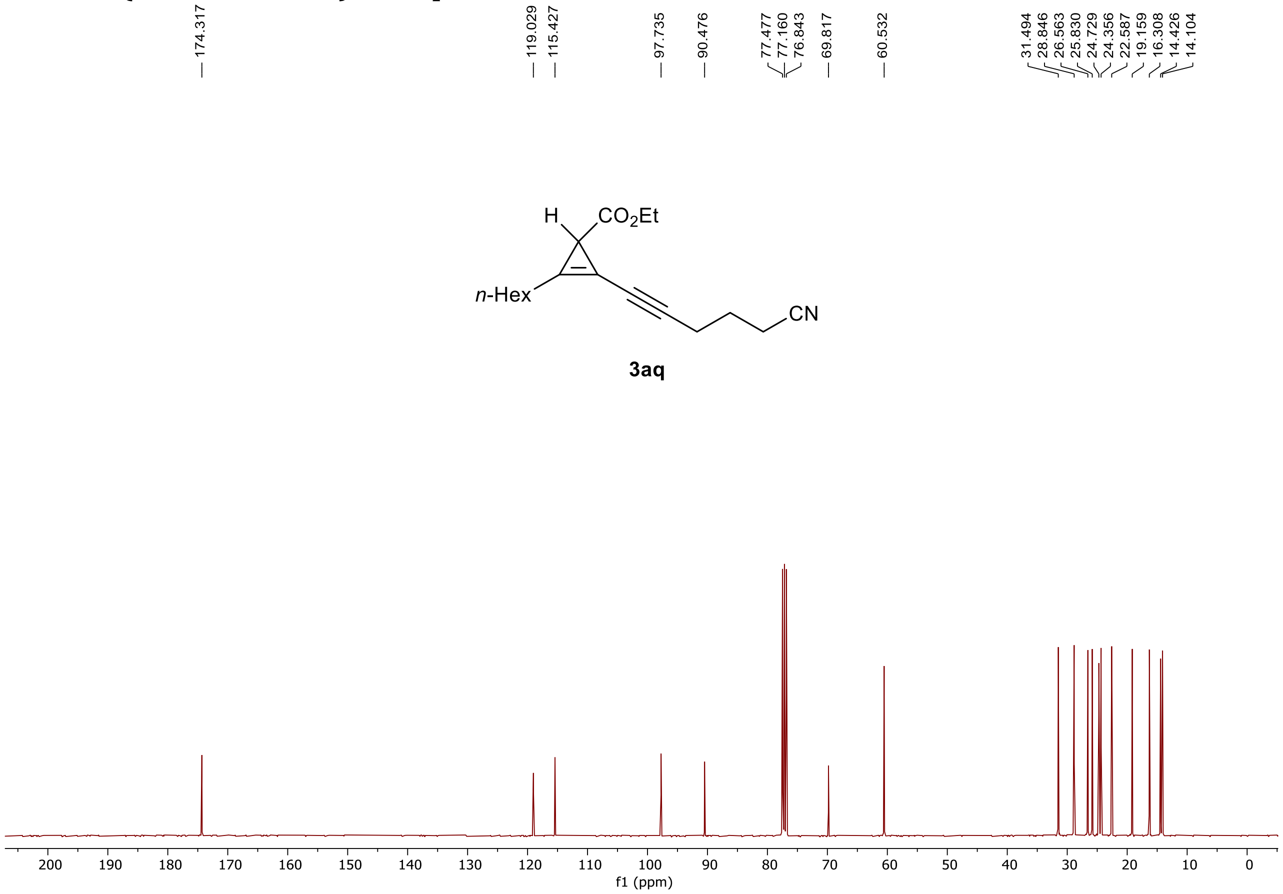

^{1}H NMR (400 MHz, $CDCl_3$) of 3ar

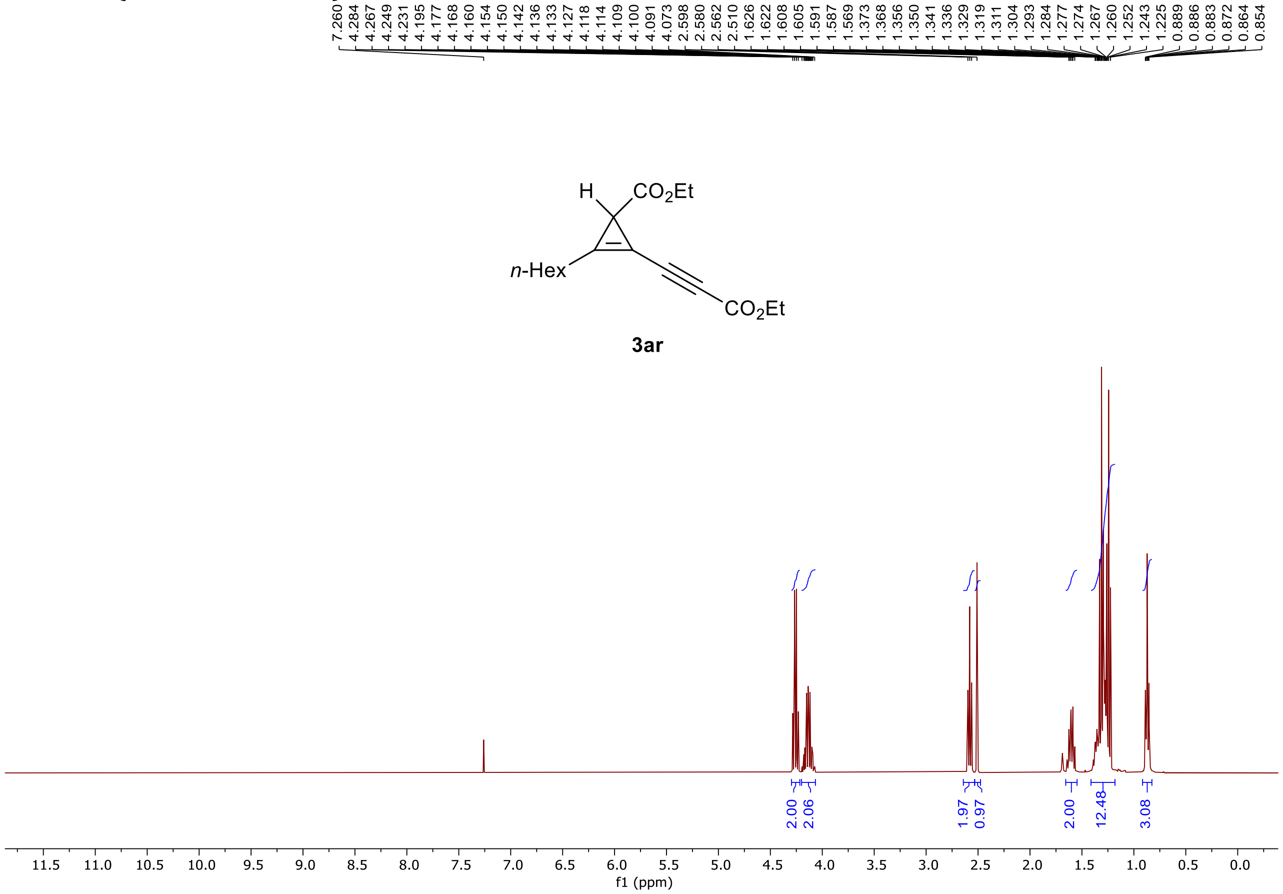

^{13}C NMR (101 MHz, $CDCl_3$) of 3ar

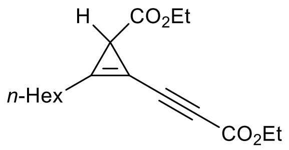

3ar

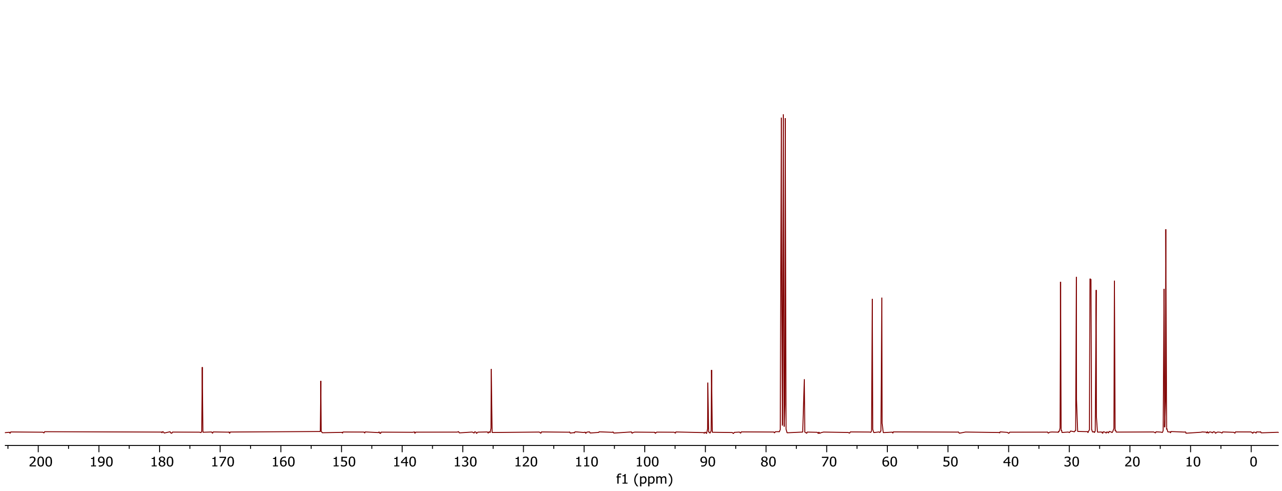

^{1}H NMR (400 MHz, $CDCl_3$) of 3as

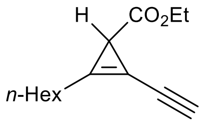

3as

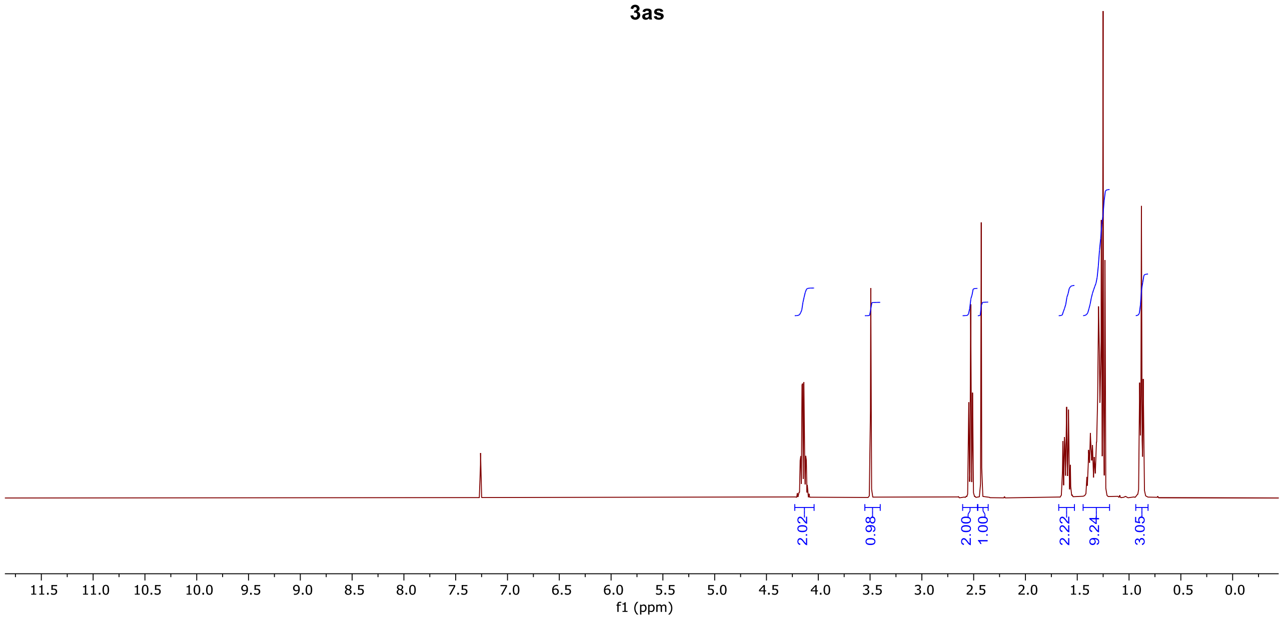

^{13}C NMR (101 MHz, $CDCl_3$) of 3as

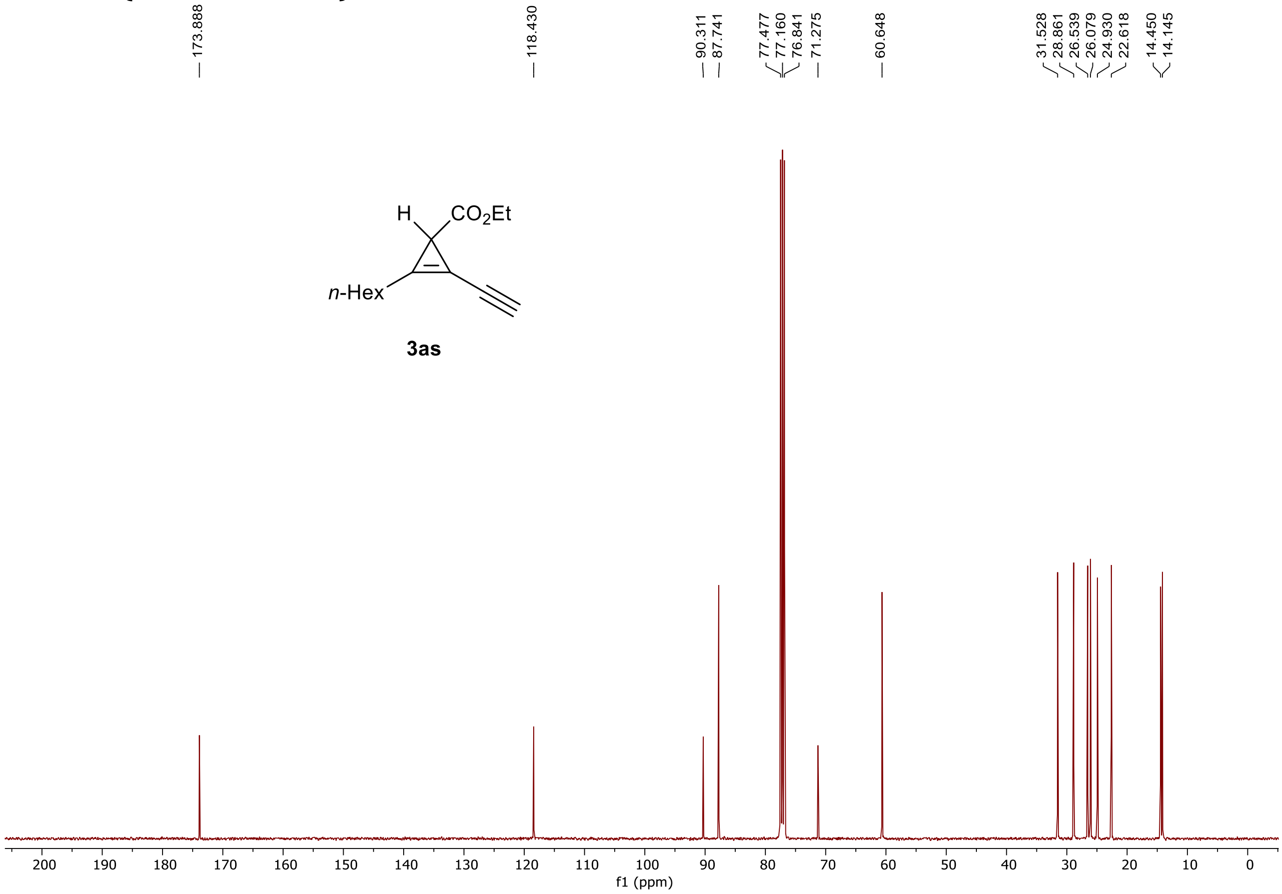

^{1}H NMR (400 MHz, $CDCl_3$) of 3at

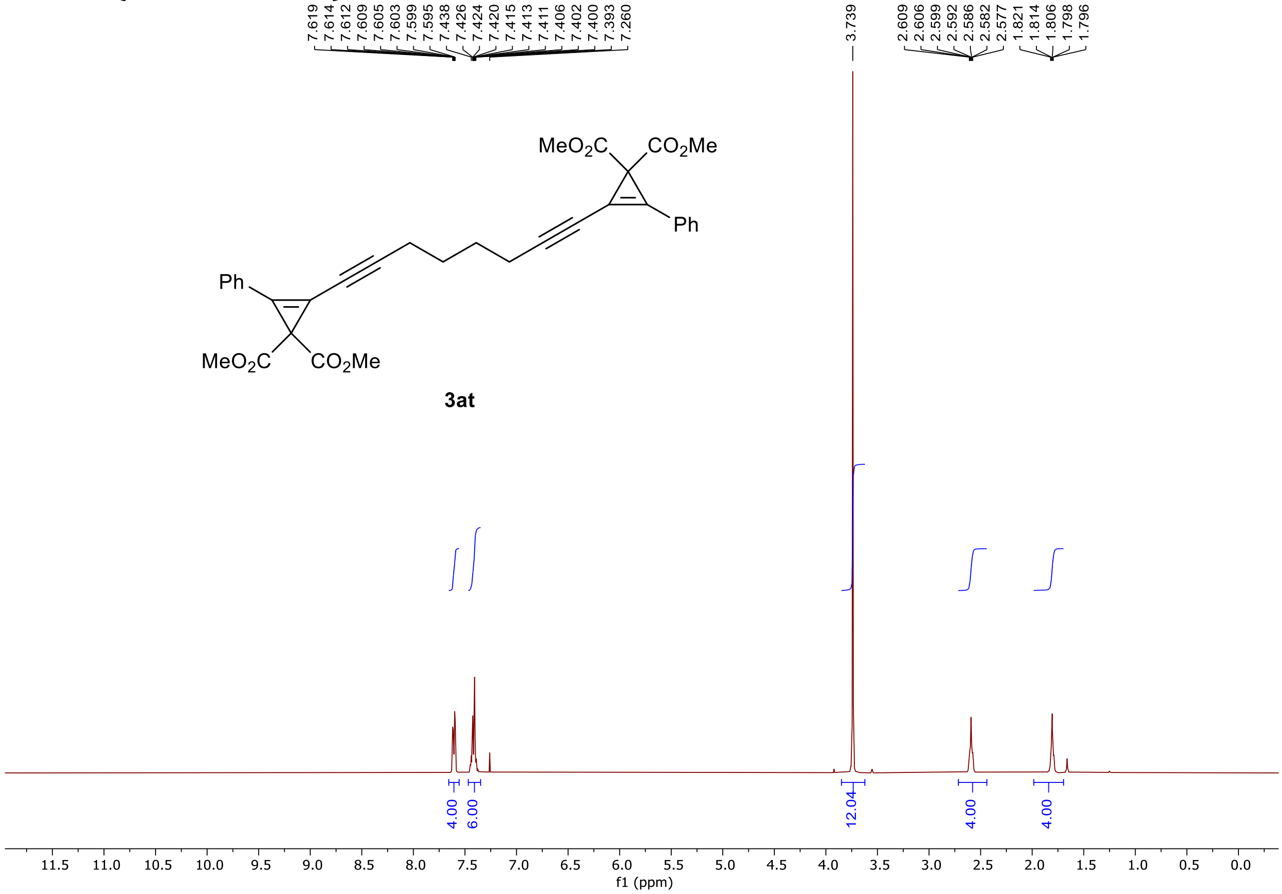

^{13}C NMR (101 MHz, CDCl$_3$) of 3at

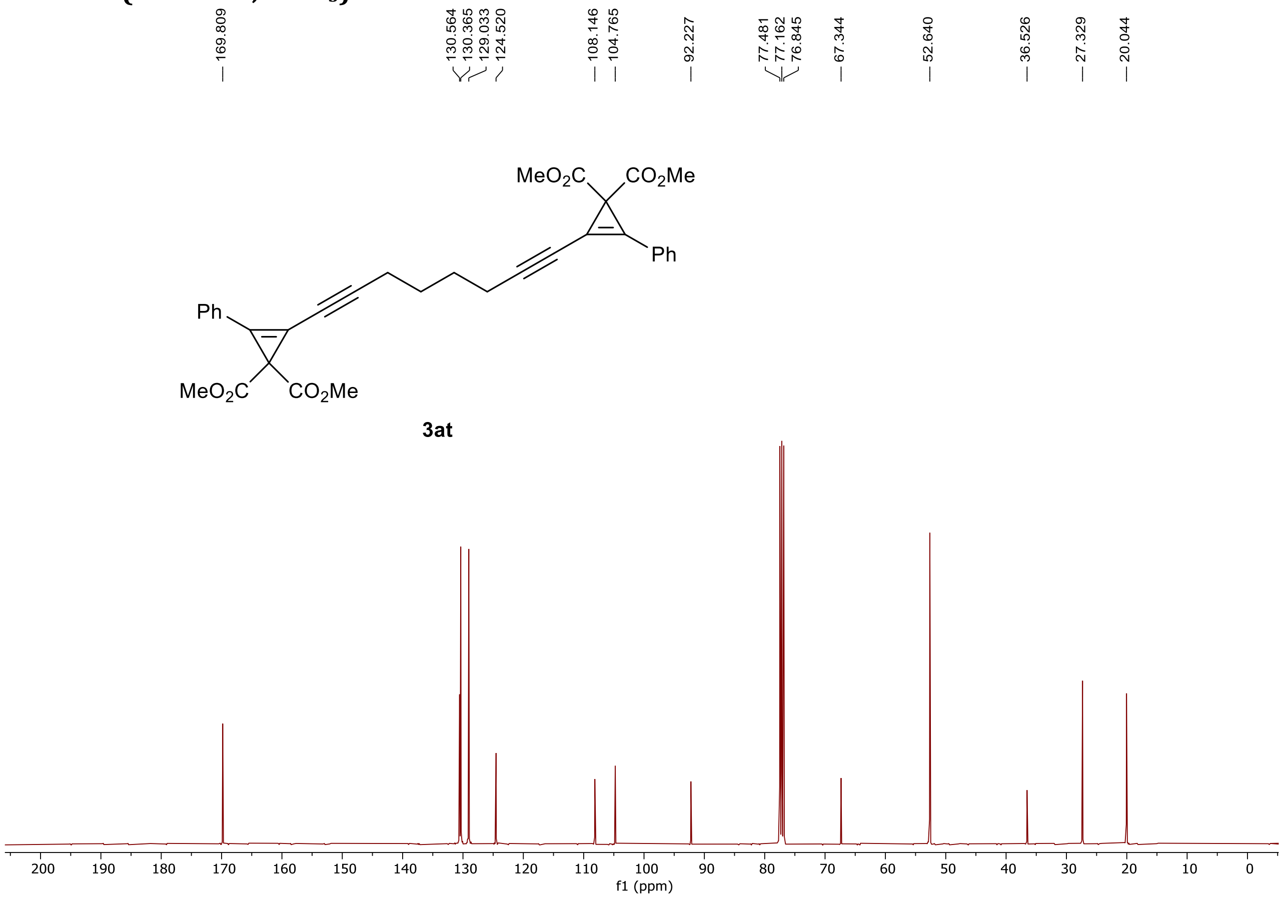

^{1}H NMR (400 MHz, CDCl$_3$) of 3au

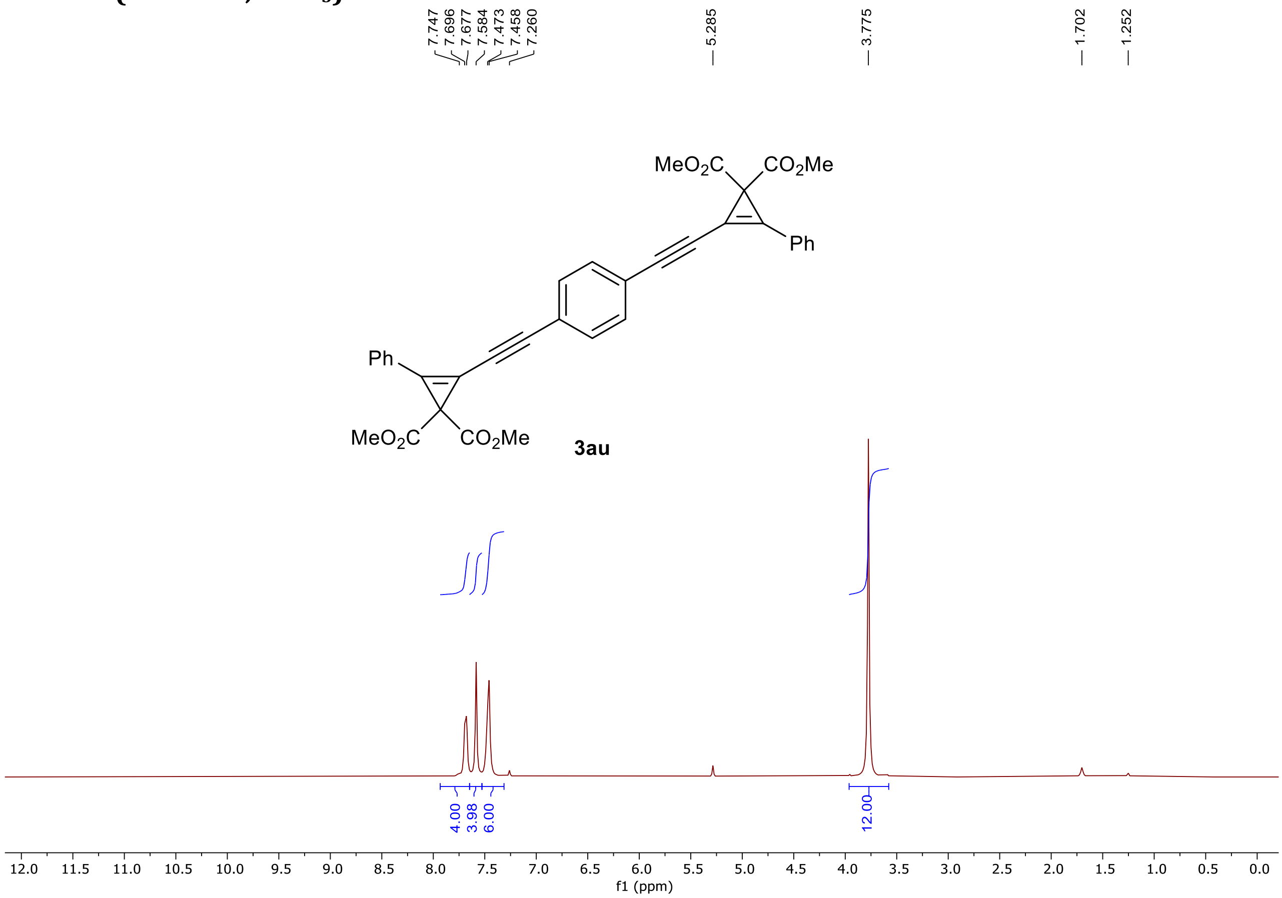

^{13}C NMR (101 MHz, $CDCl_3$) of 3au

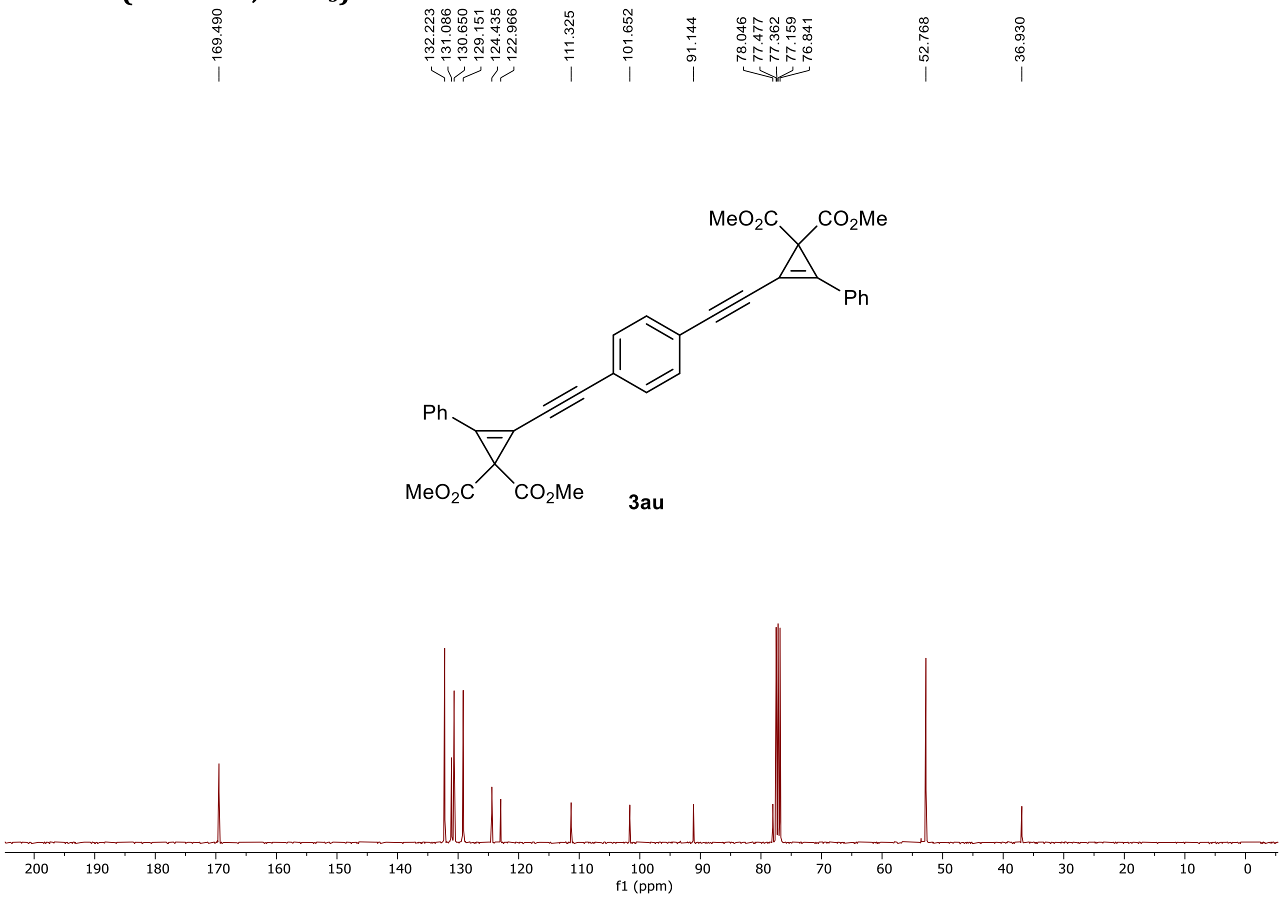

^{1}H NMR (400 MHz, $CDCl_3$) of 3av

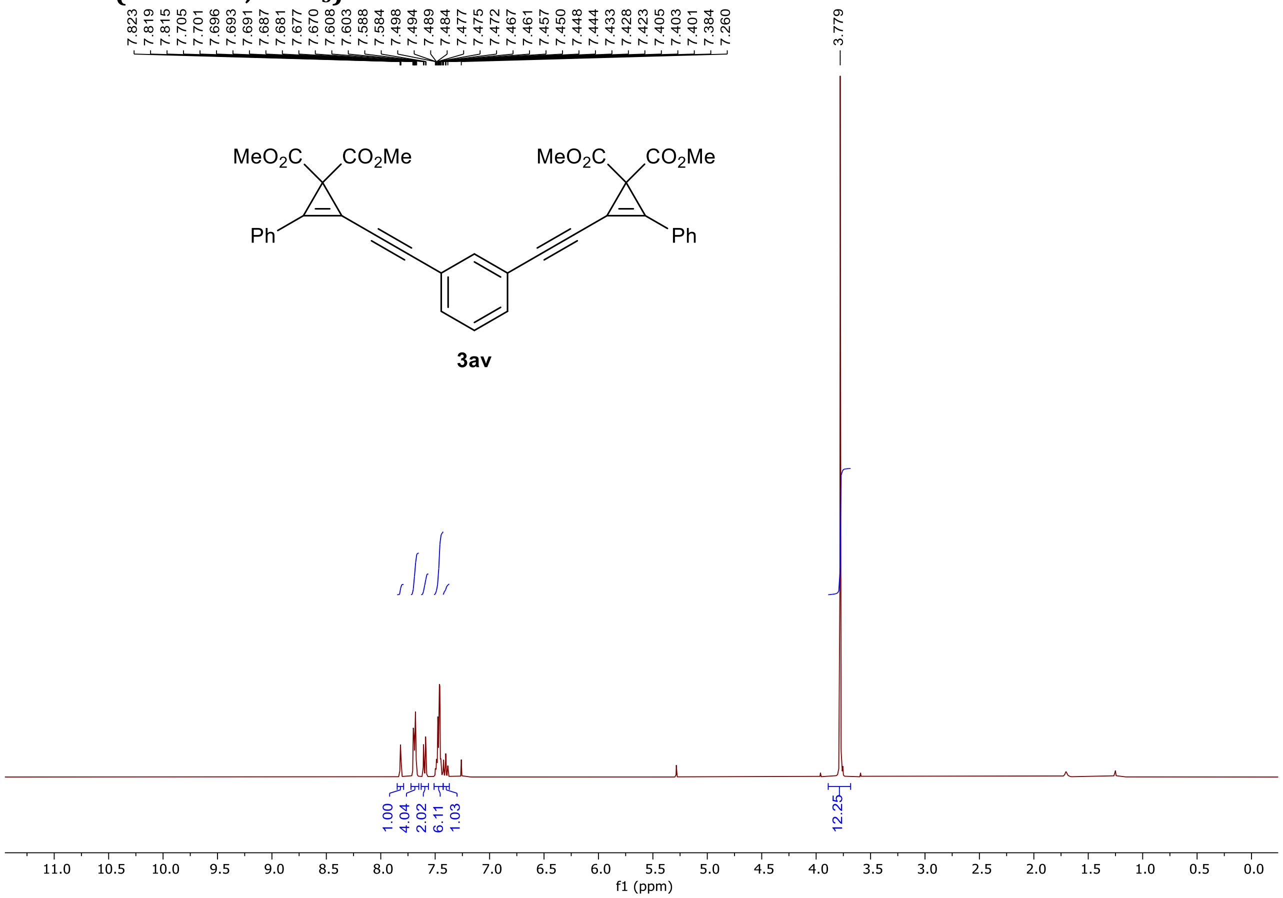

^{13}C NMR (101 MHz, $CDCl_3$) of 3av

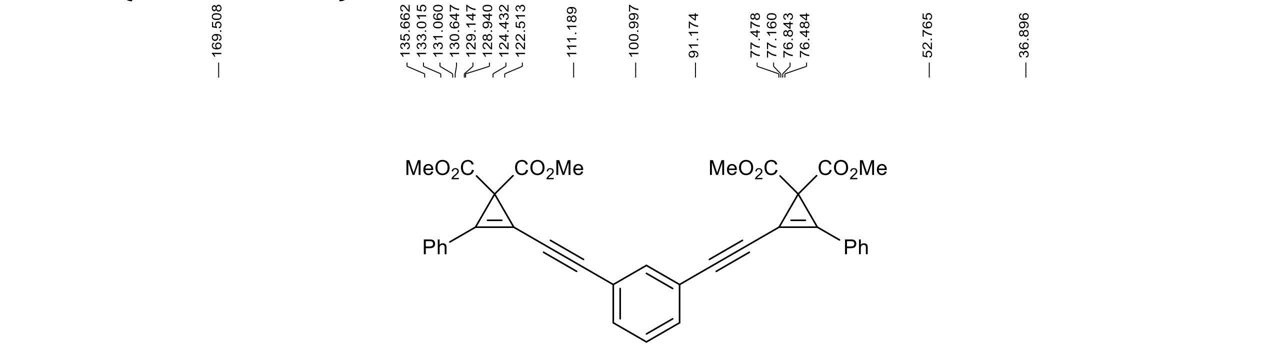

3av

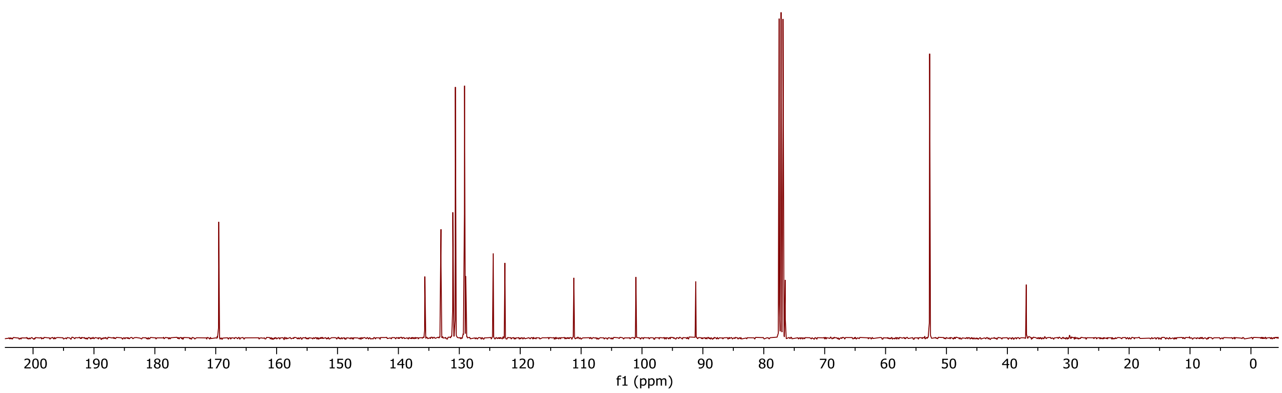

^{1}H NMR (400 MHz, $CDCl_3$) of 3aw

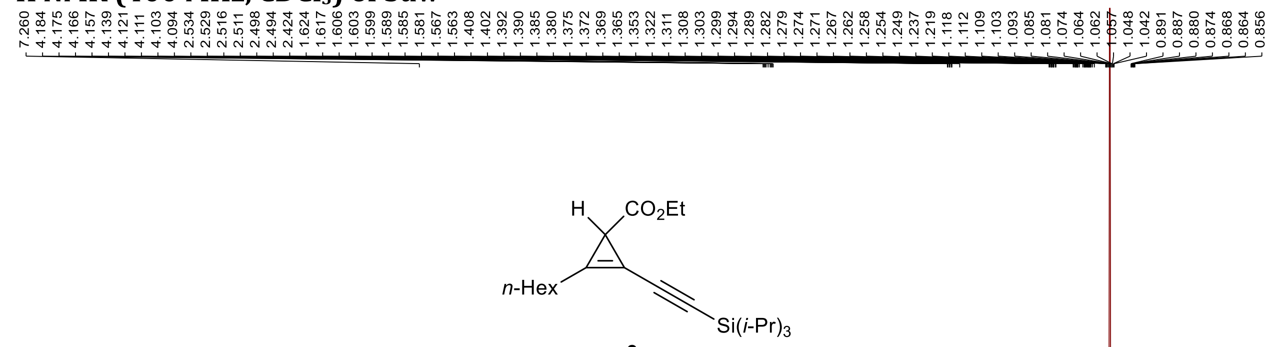

3aw

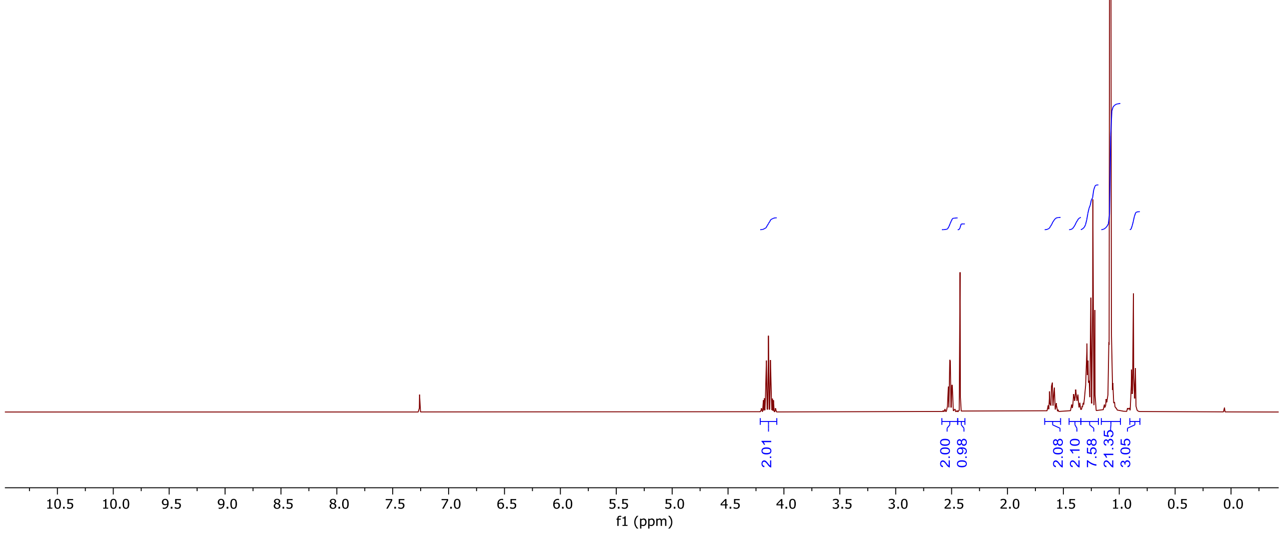

^{13}C NMR (101 MHz, $CDCl_3$) of 3aw

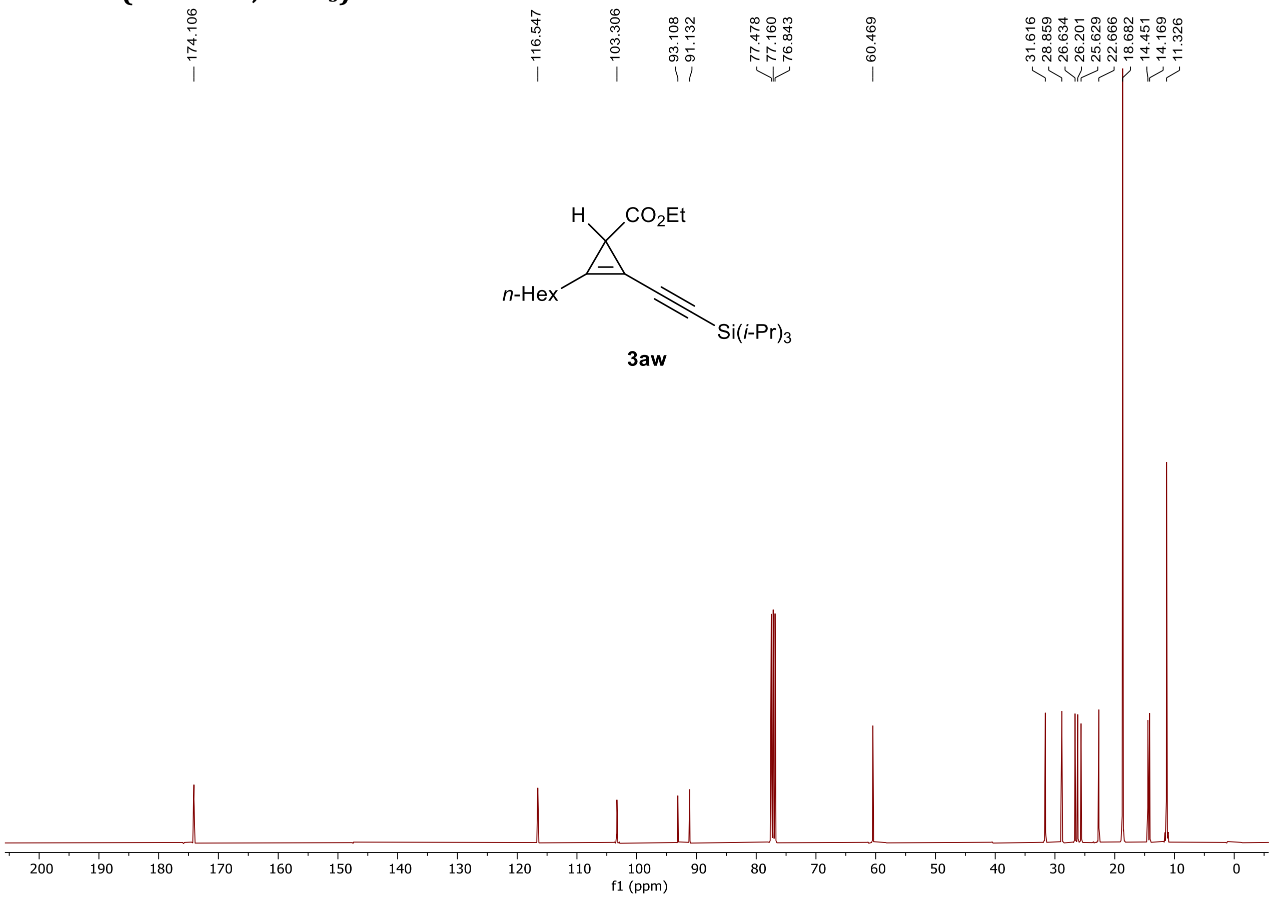

^{1}H NMR (400 MHz, $CDCl_3$) of 3ax

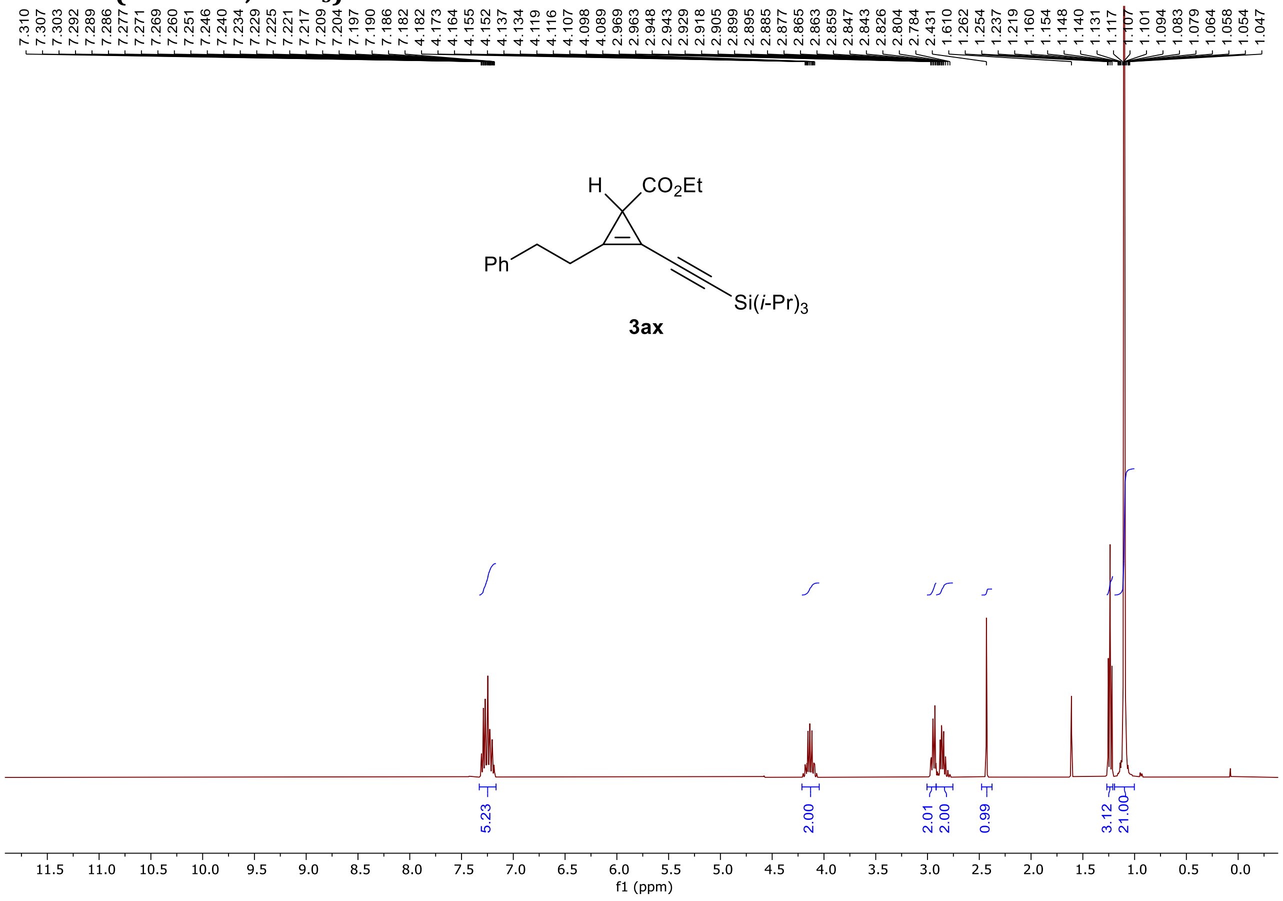

^{13}C NMR (101 MHz, $CDCl_3$) of 3ax

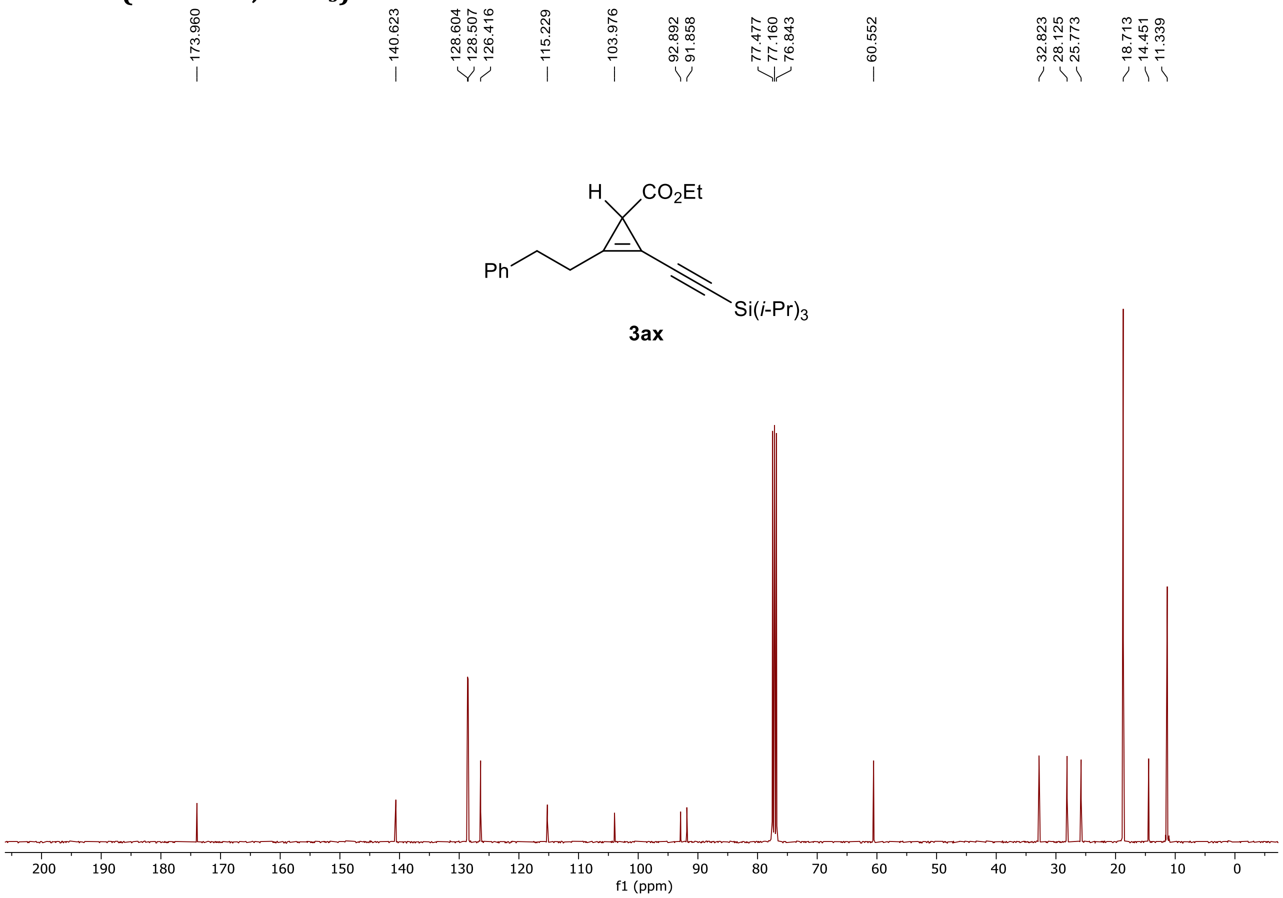

^{1}H NMR (400 MHz, $CDCl_3$) of 3ay

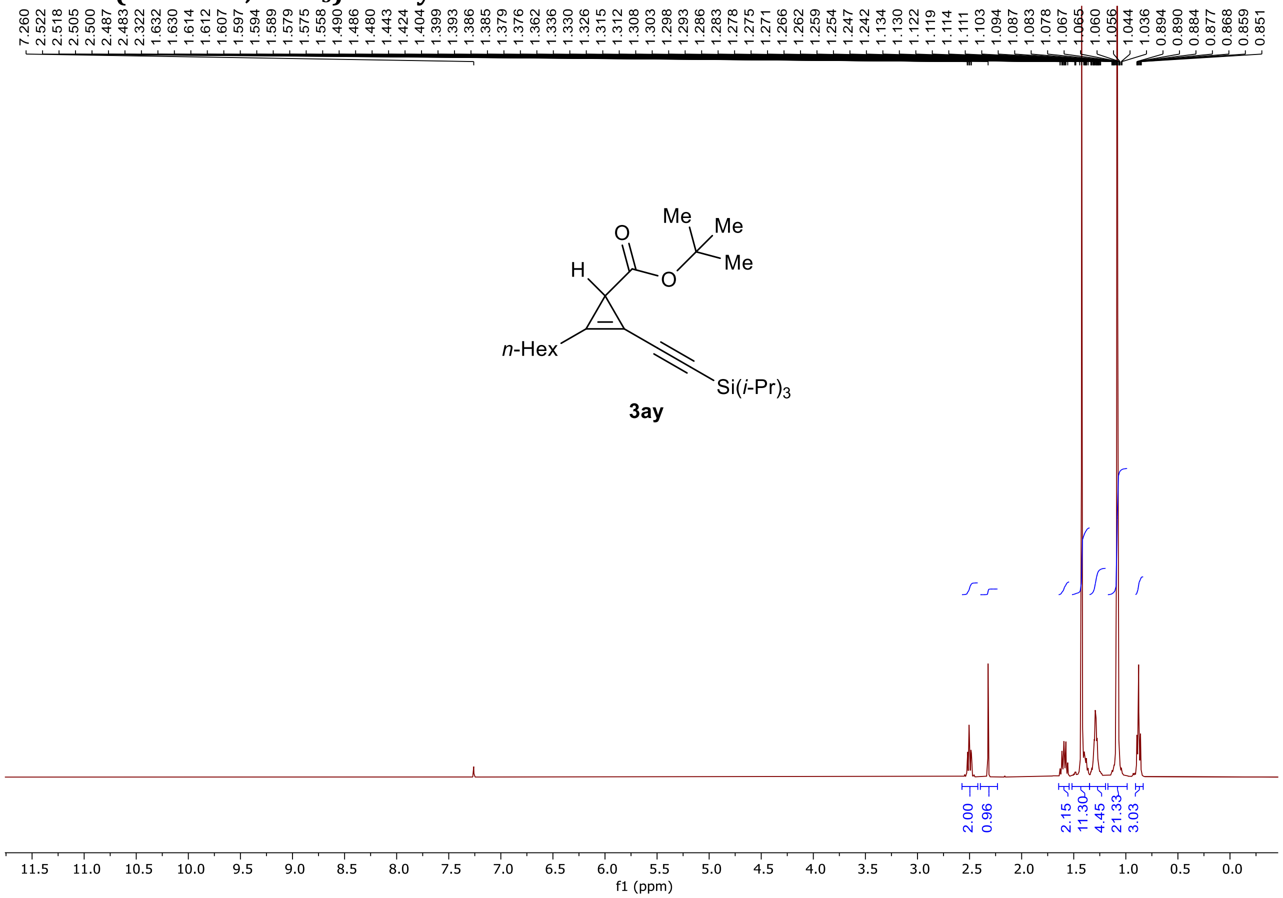

^{13}C NMR (101 MHz, $CDCl_3$) of 3ay

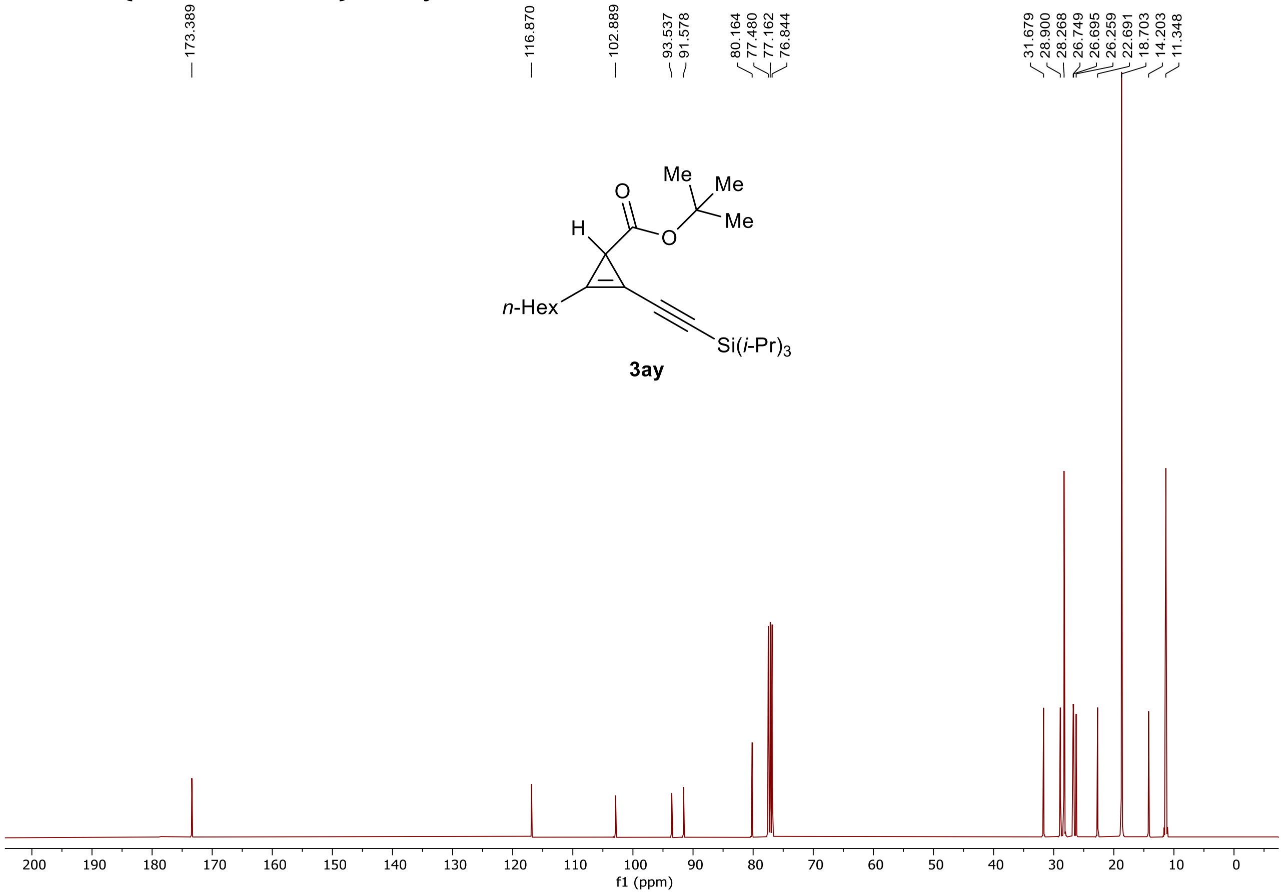

^{1}H NMR (400 MHz, $CDCl_3$) of 3az

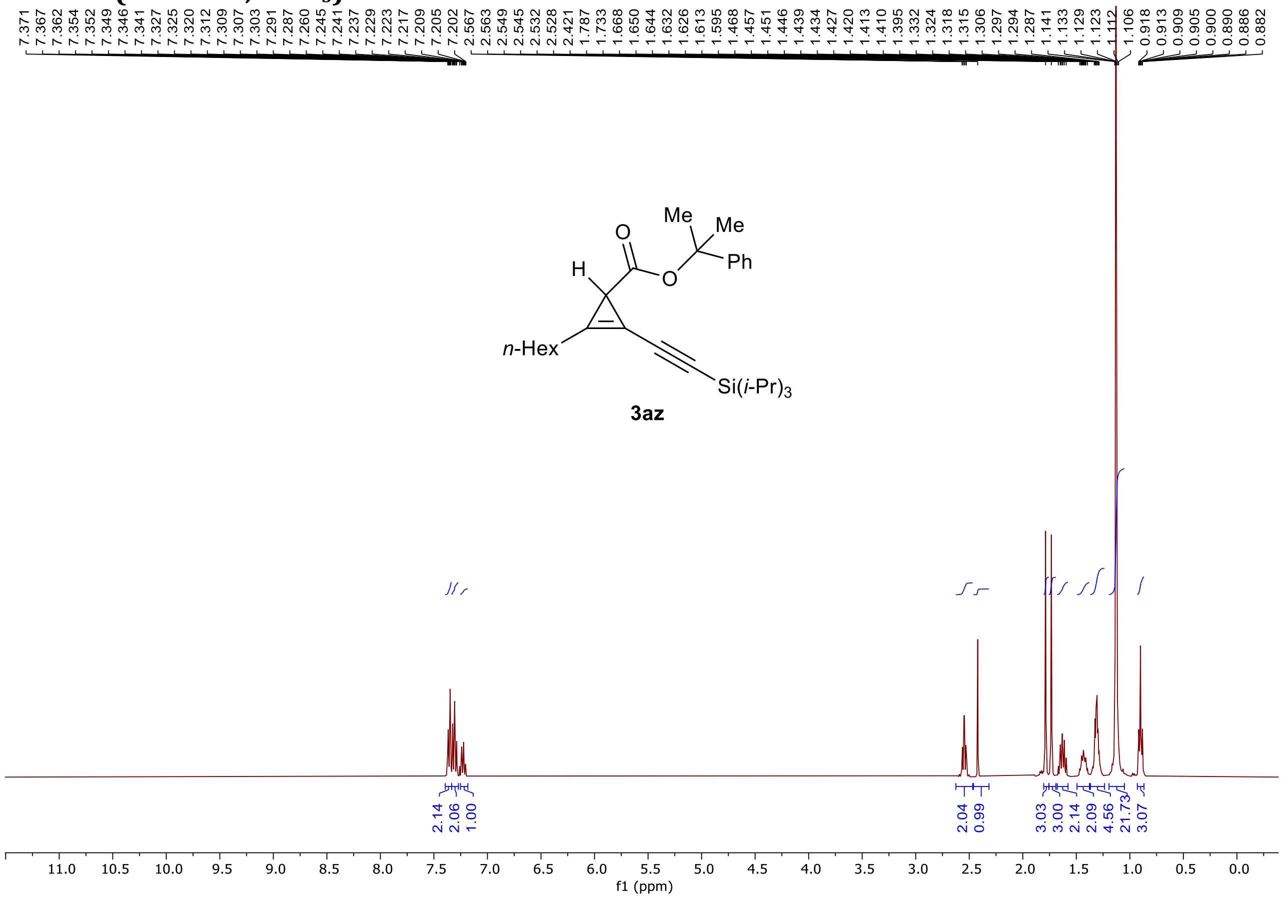

^{13}C NMR (101 MHz, $CDCl_3$) of 3az

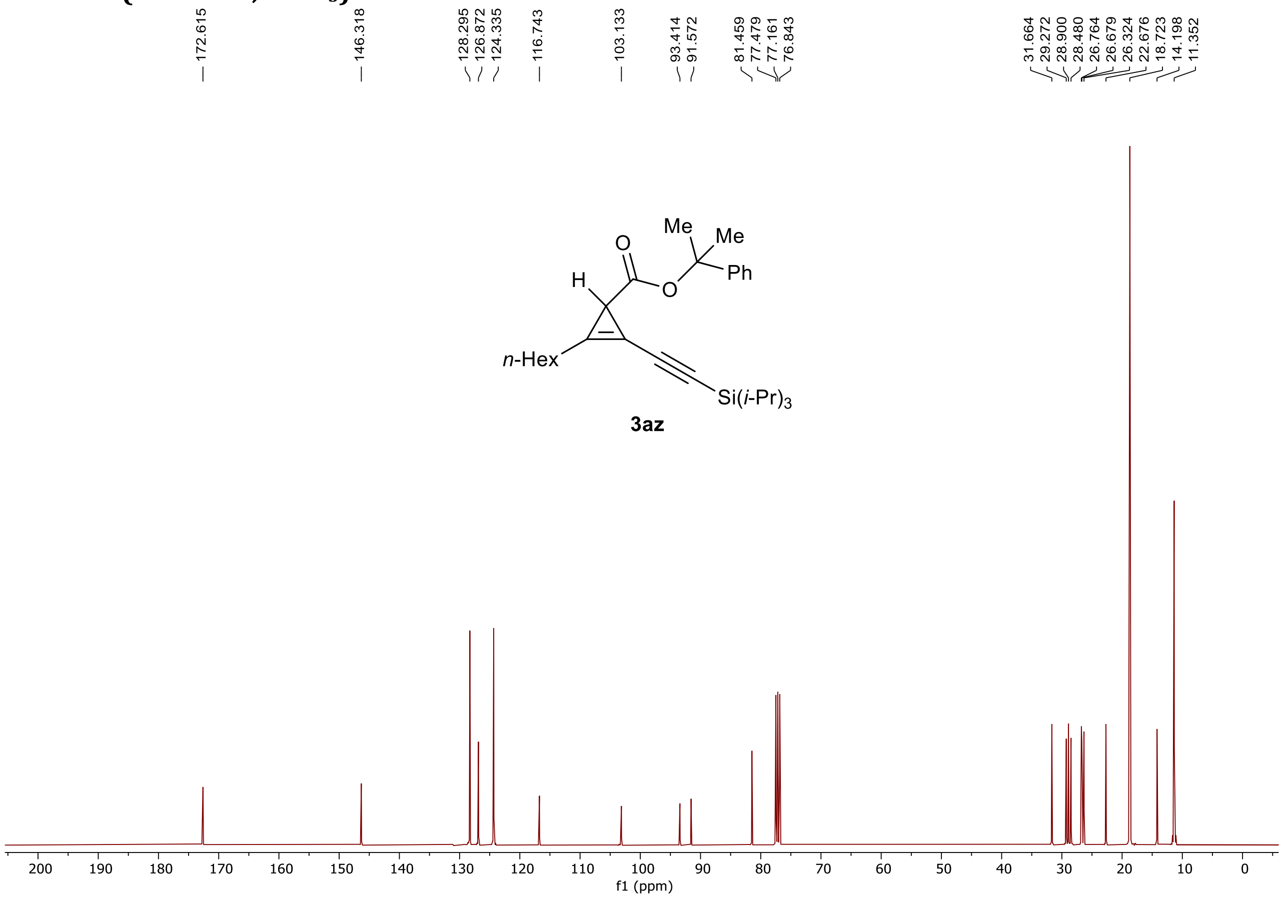

^{1}H NMR (400 MHz, $CDCl_3$) of 3ba

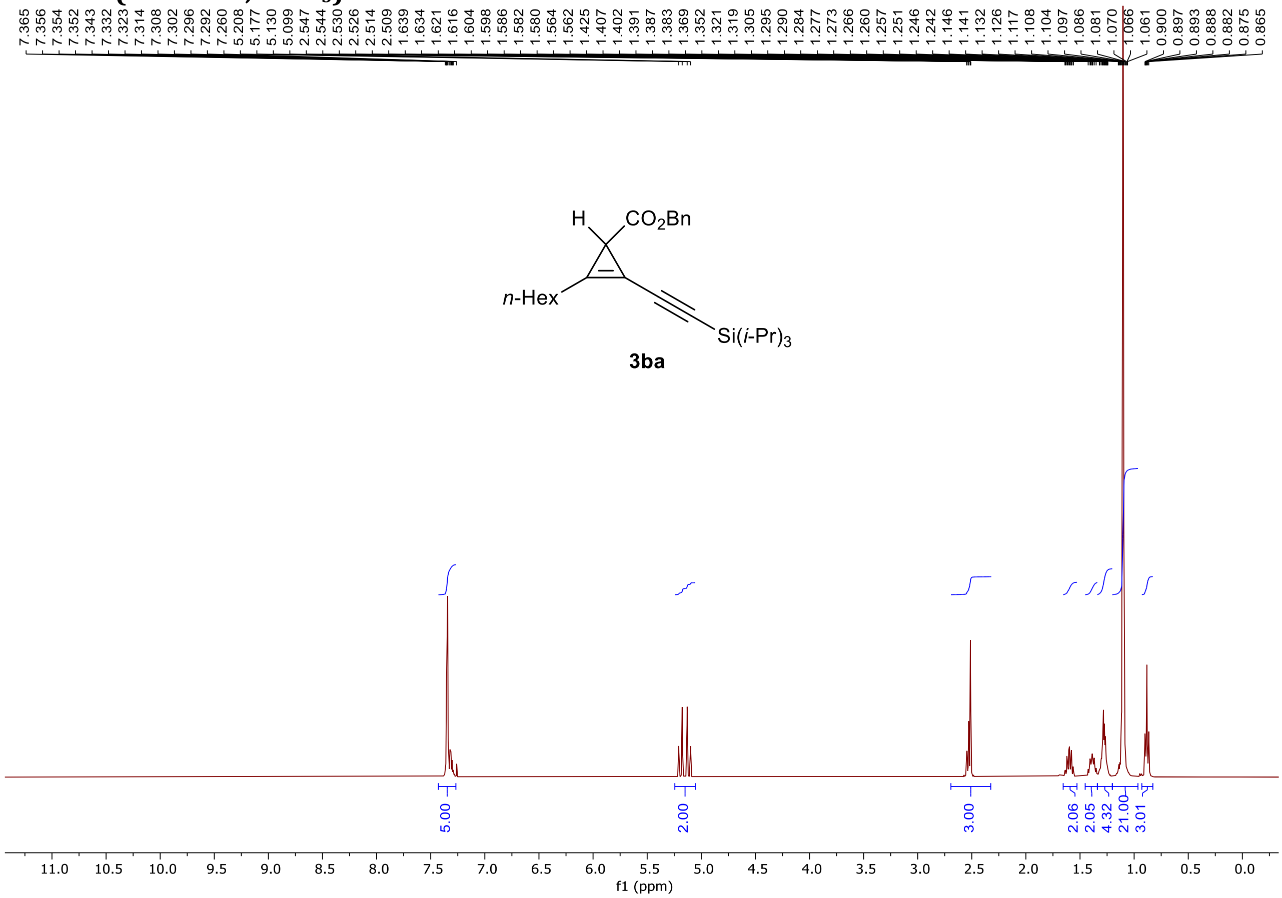

^{13}C NMR (101 MHz, $CDCl_3$) of 3ba

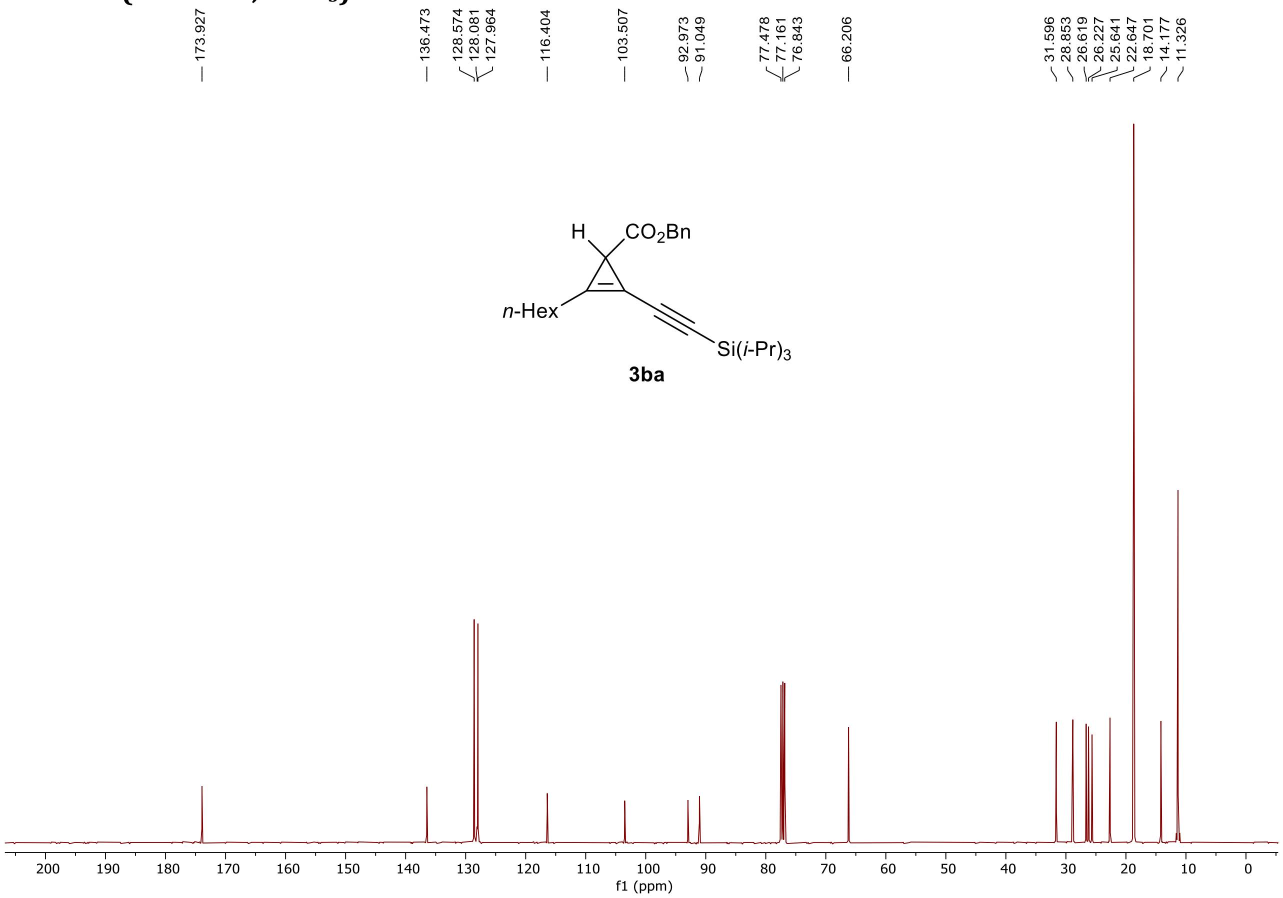

^{1}H NMR (400 MHz, $CDCl_3$) of 3bb

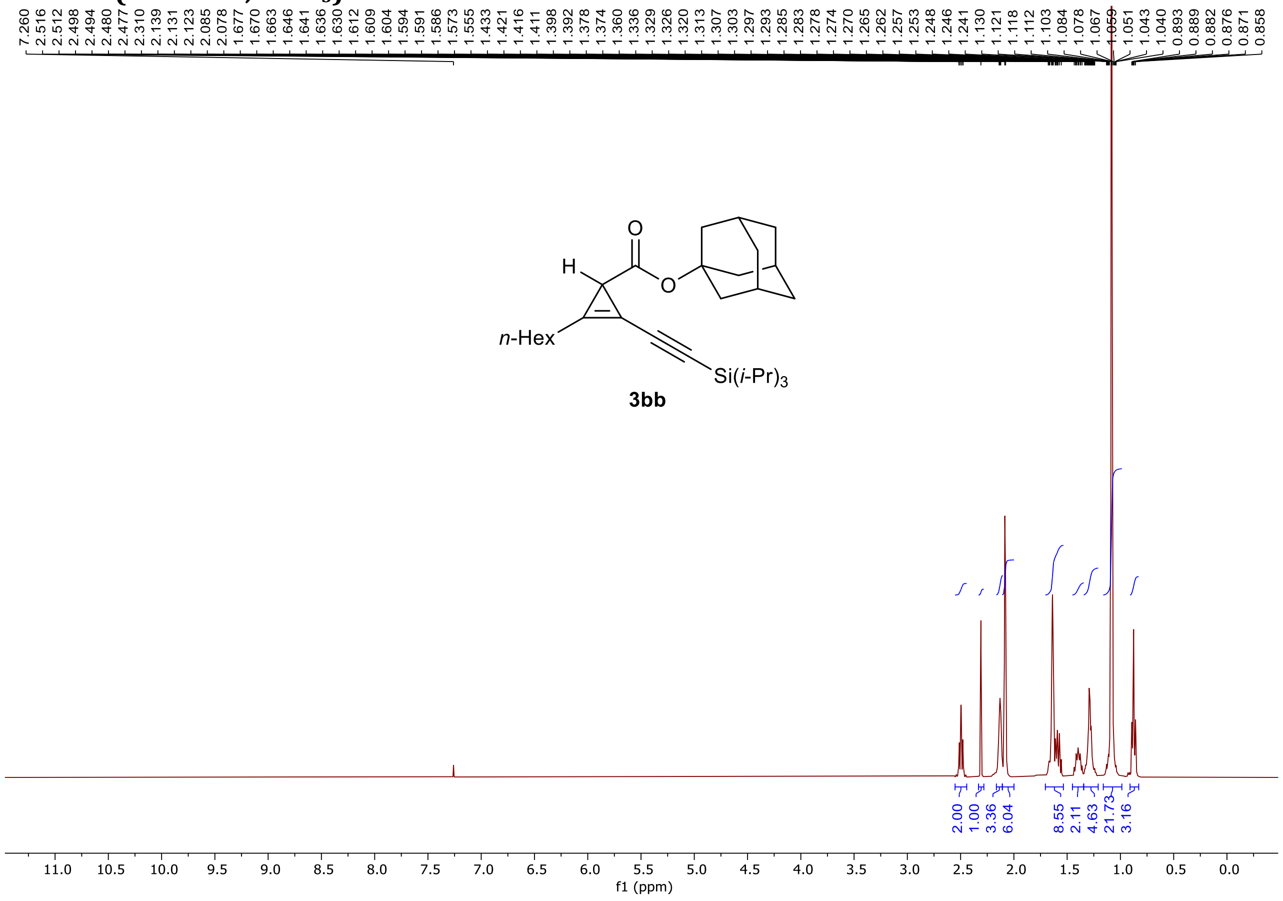

^{13}C NMR (101 MHz, $CDCl_3$) of 3bb

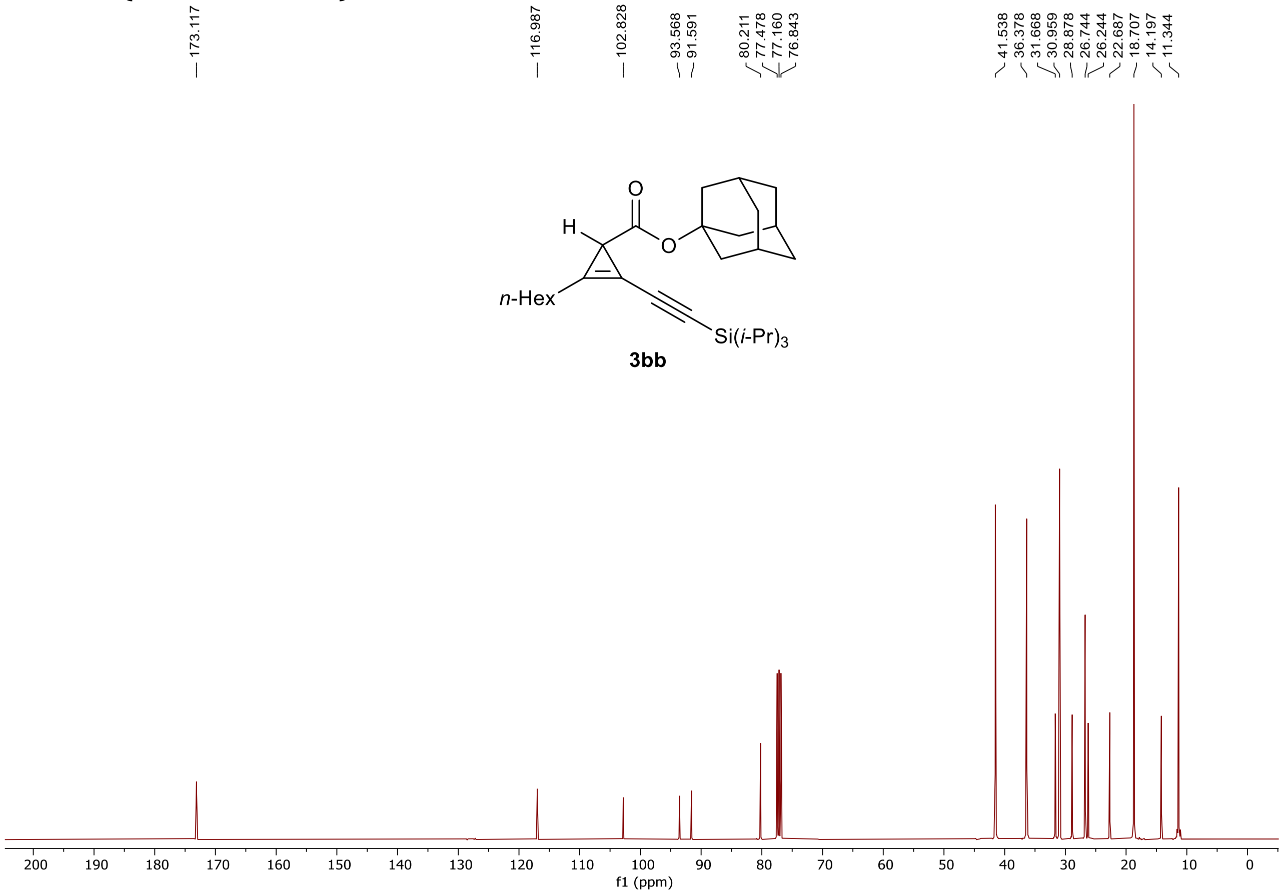

^{1}H NMR (400 MHz, $CDCl_3$) of 3bc

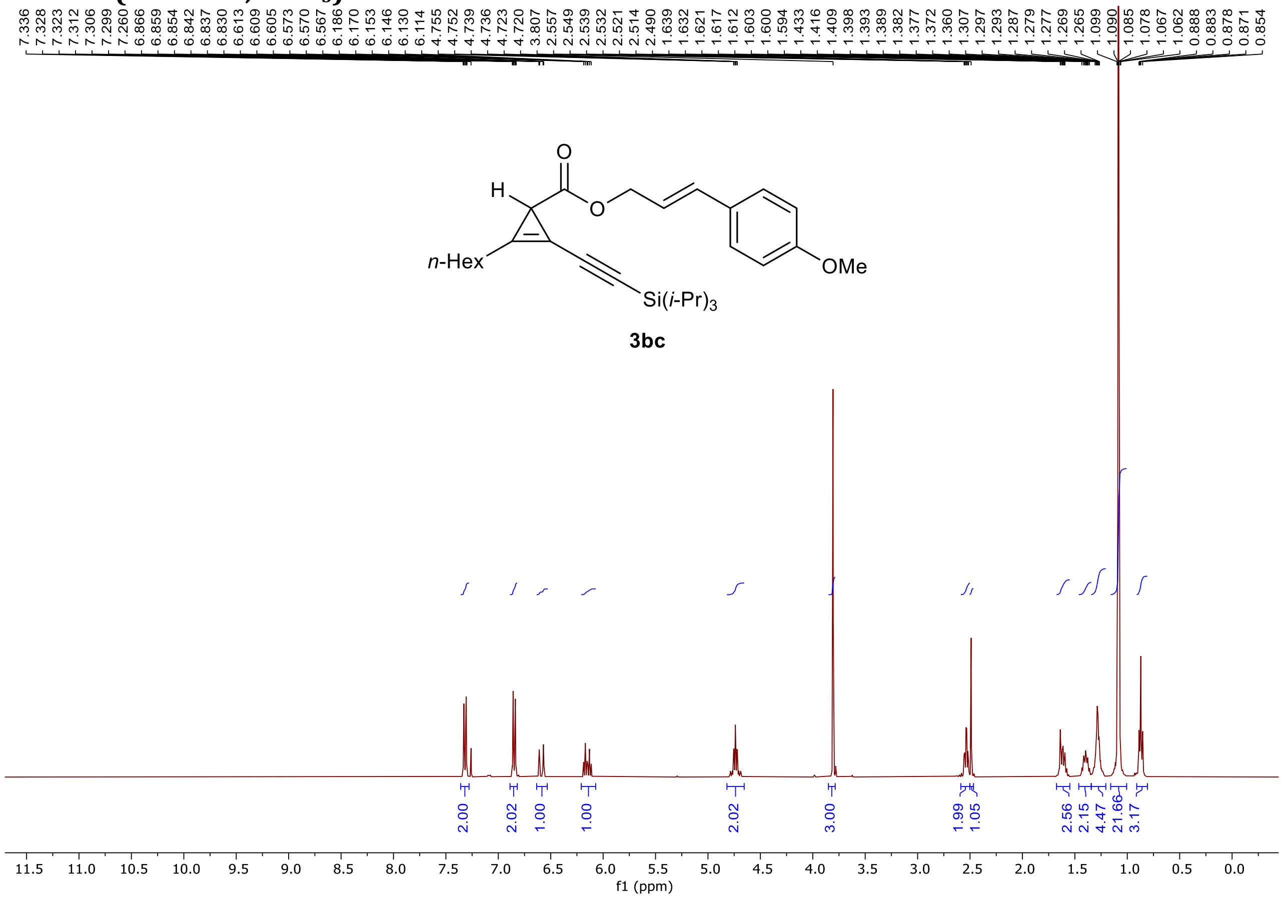

^{13}C NMR (101 MHz, $CDCl_3$) of 3bc

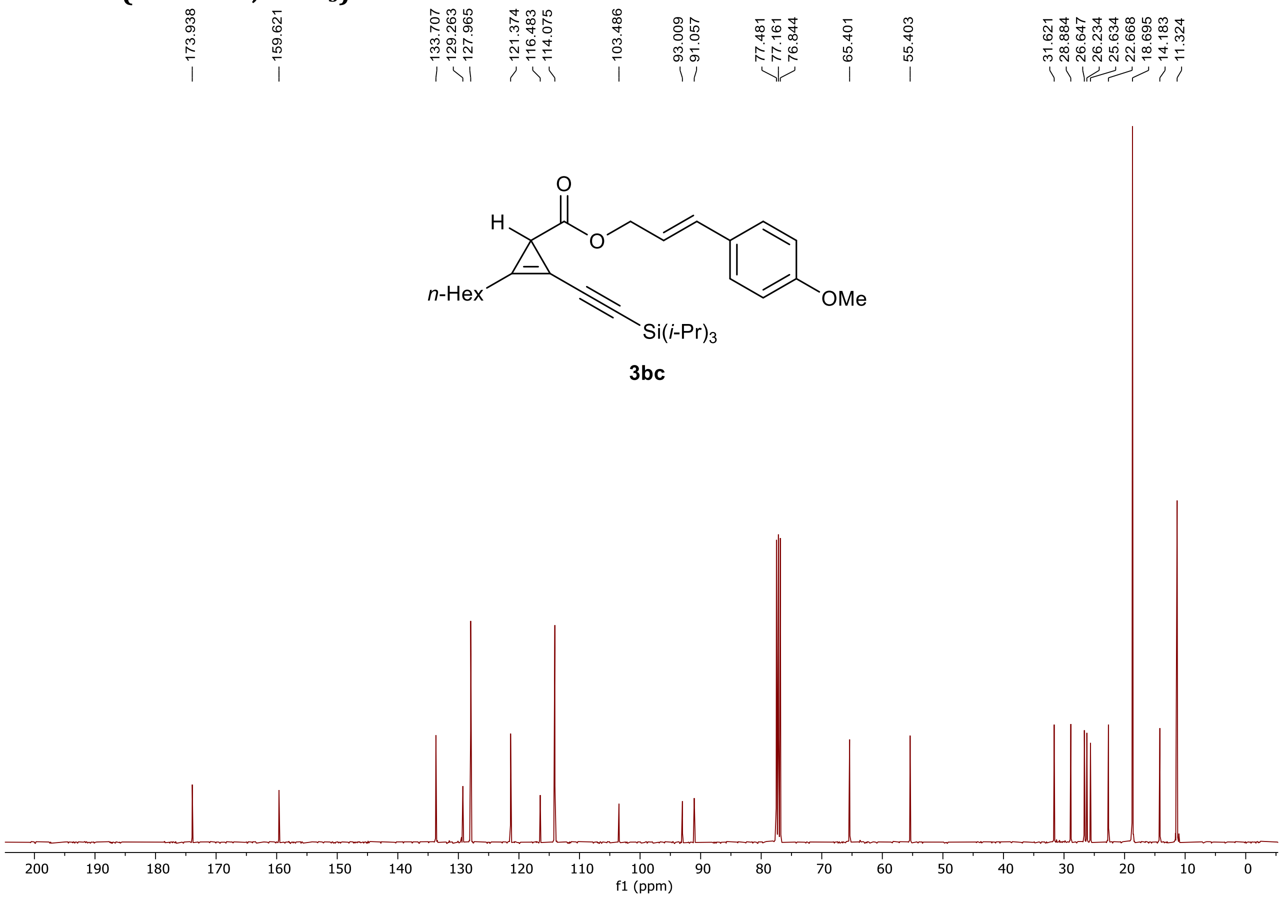

^{1}H NMR (400 MHz, $CDCl_3$) of 3bd

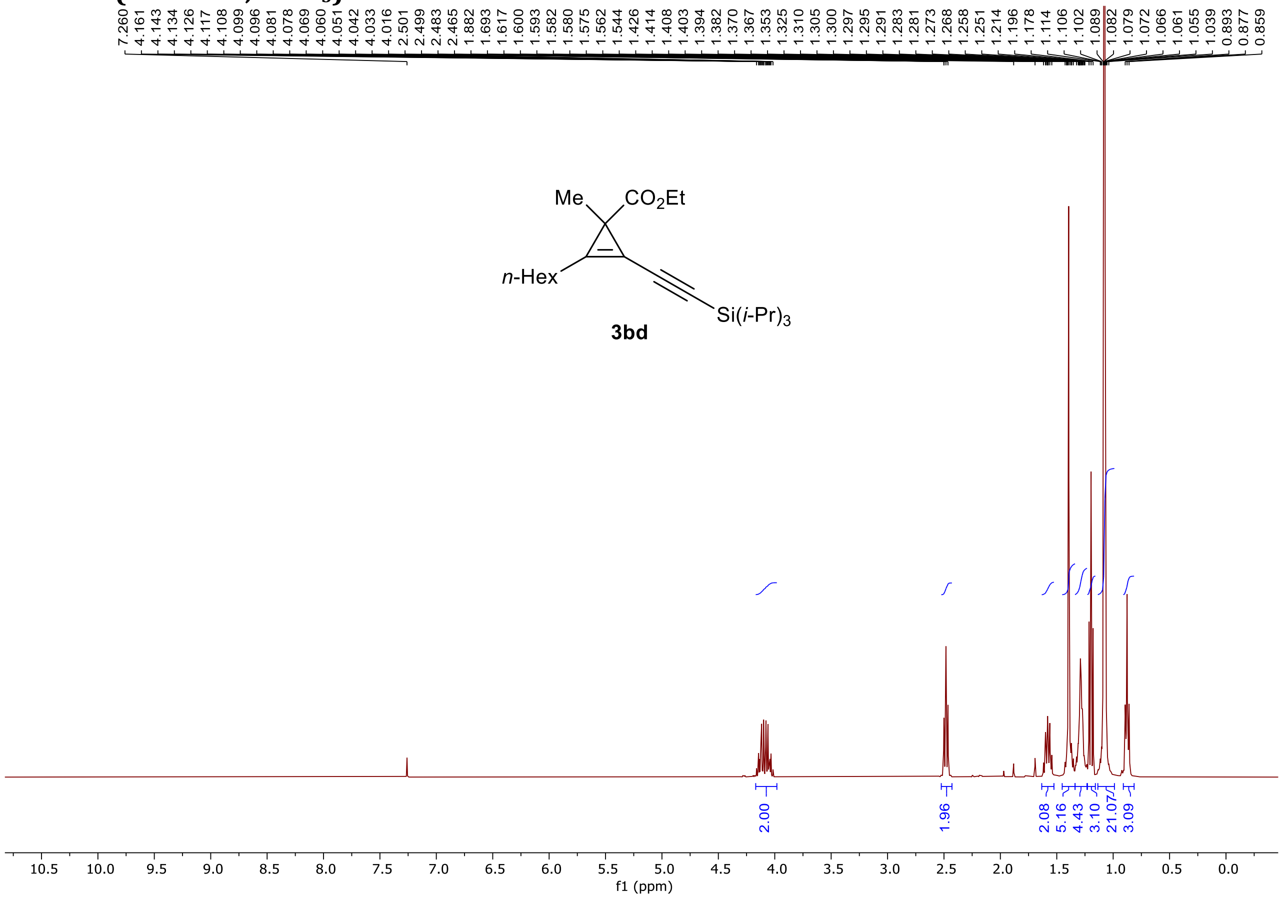

^{13}C NMR (101 MHz, $CDCl_3$) of 3bd

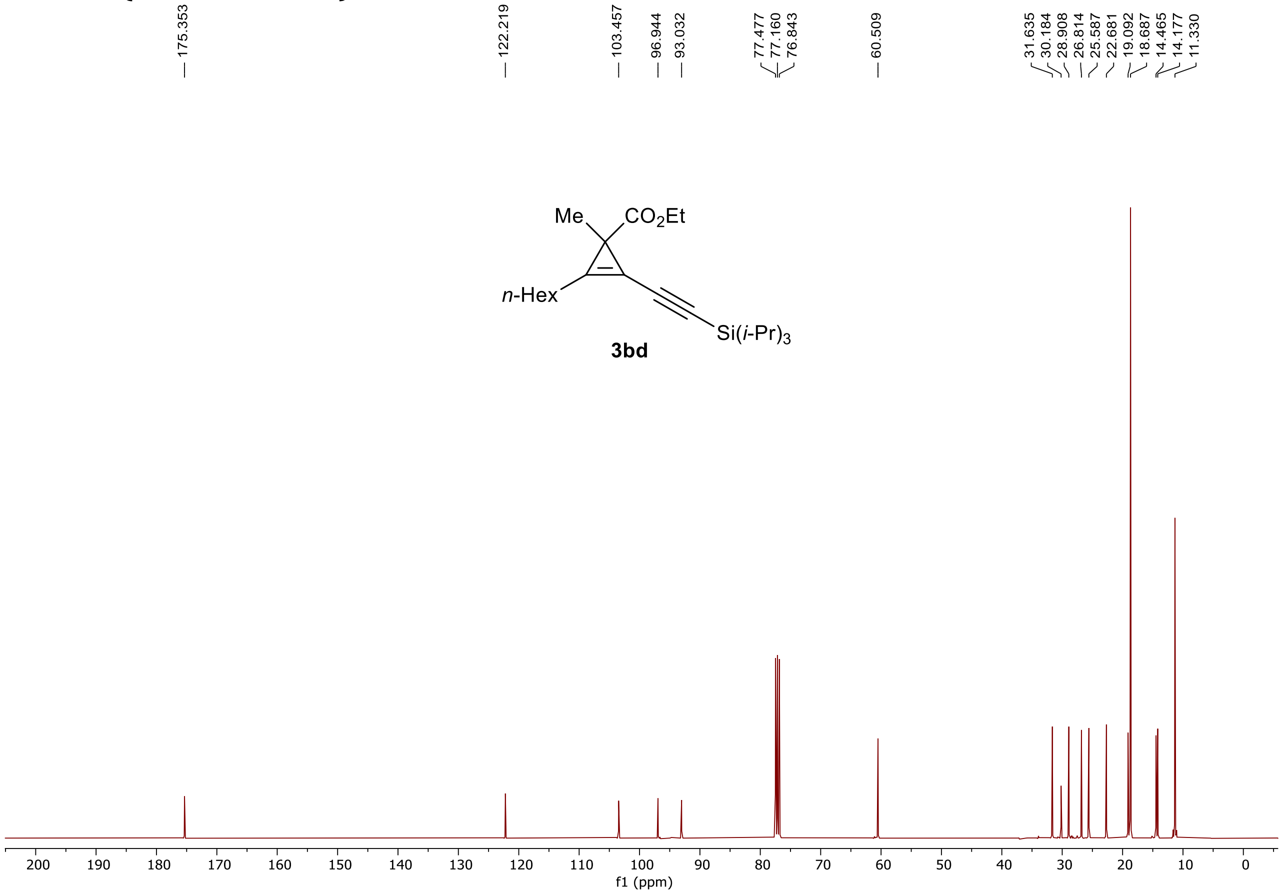

^{1}H NMR (400 MHz, $CDCl_3$) of 3be

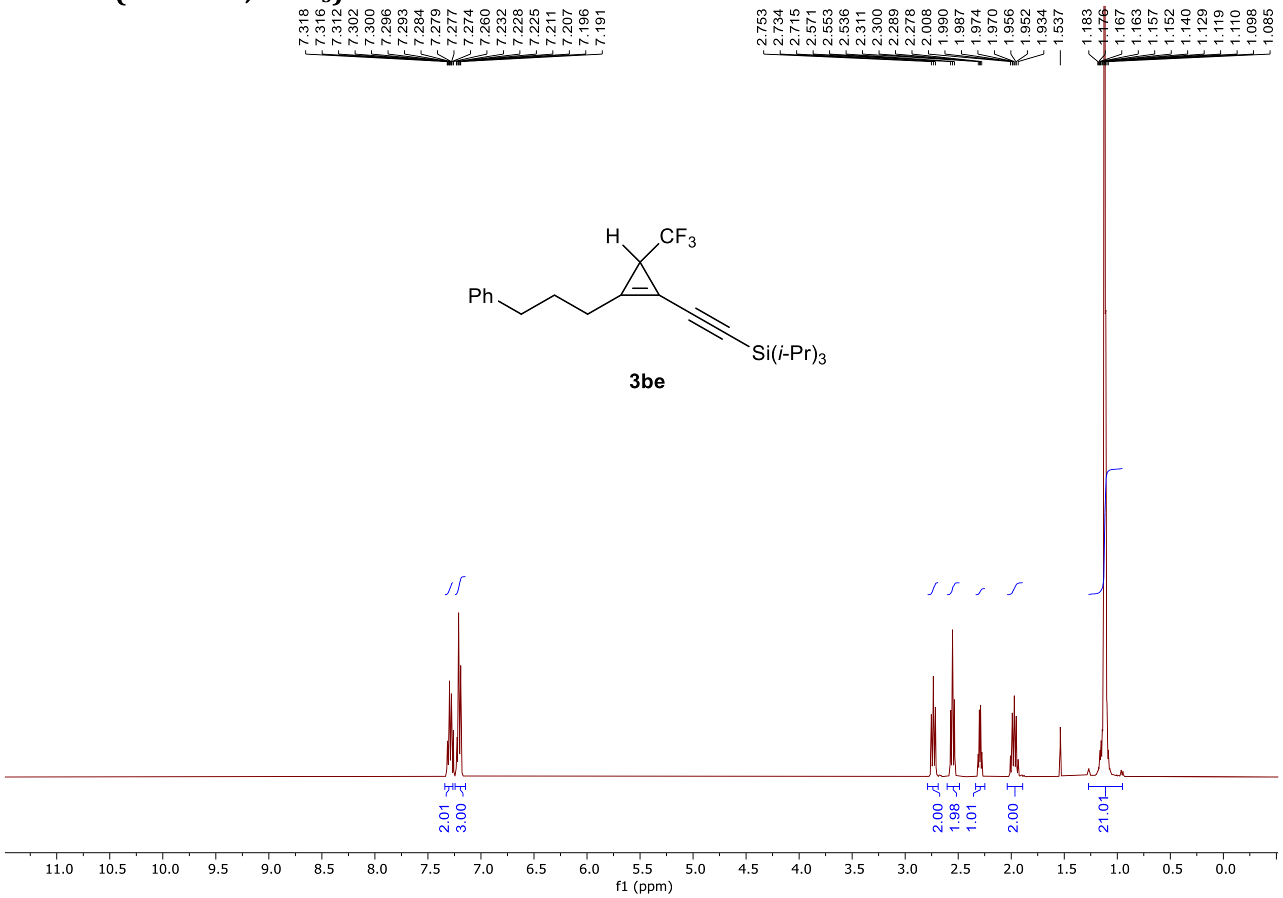

^{13}C NMR (101 MHz, $CDCl_3$) of 3be

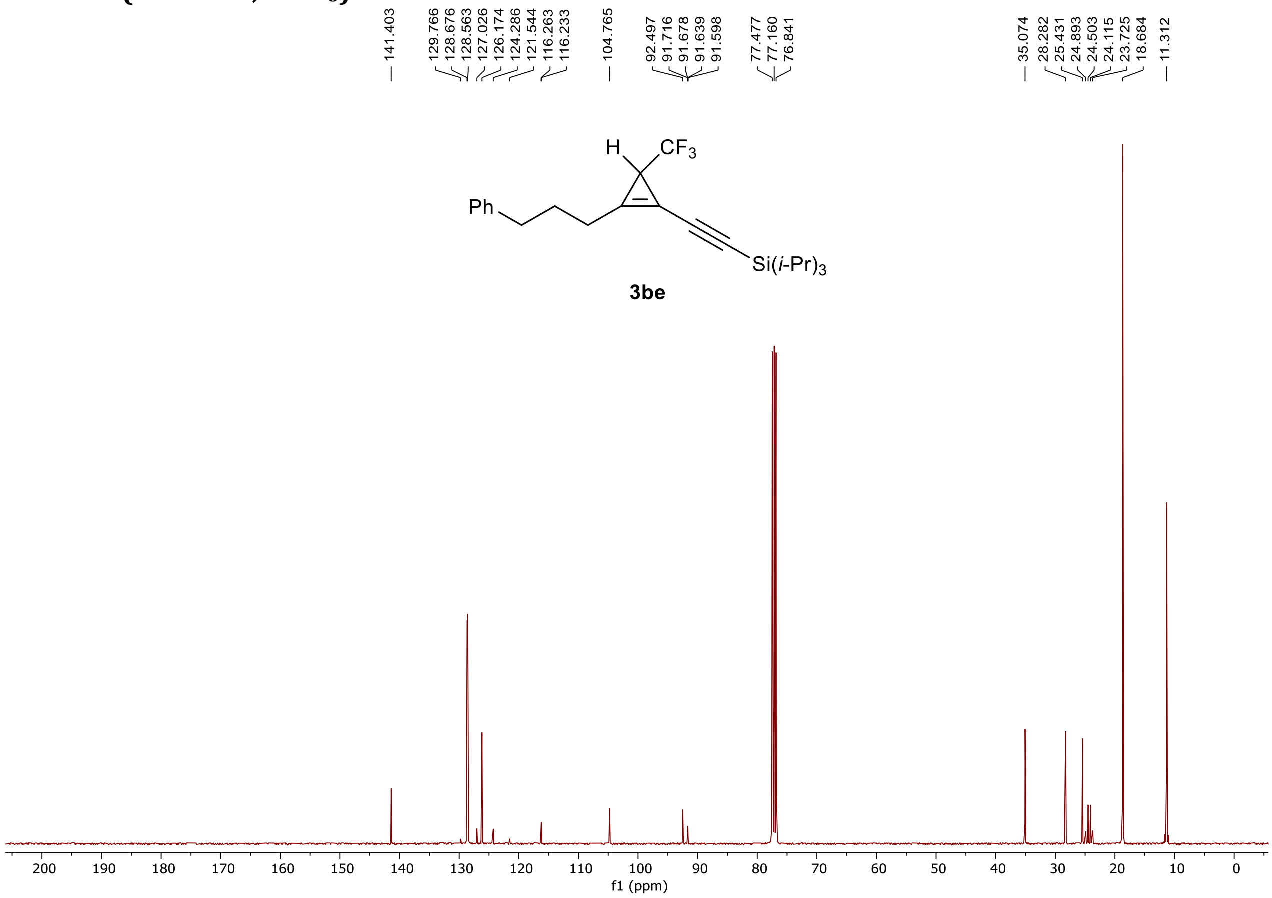

^{19}F NMR (377 MHz, $CDCl_3$) of 3be

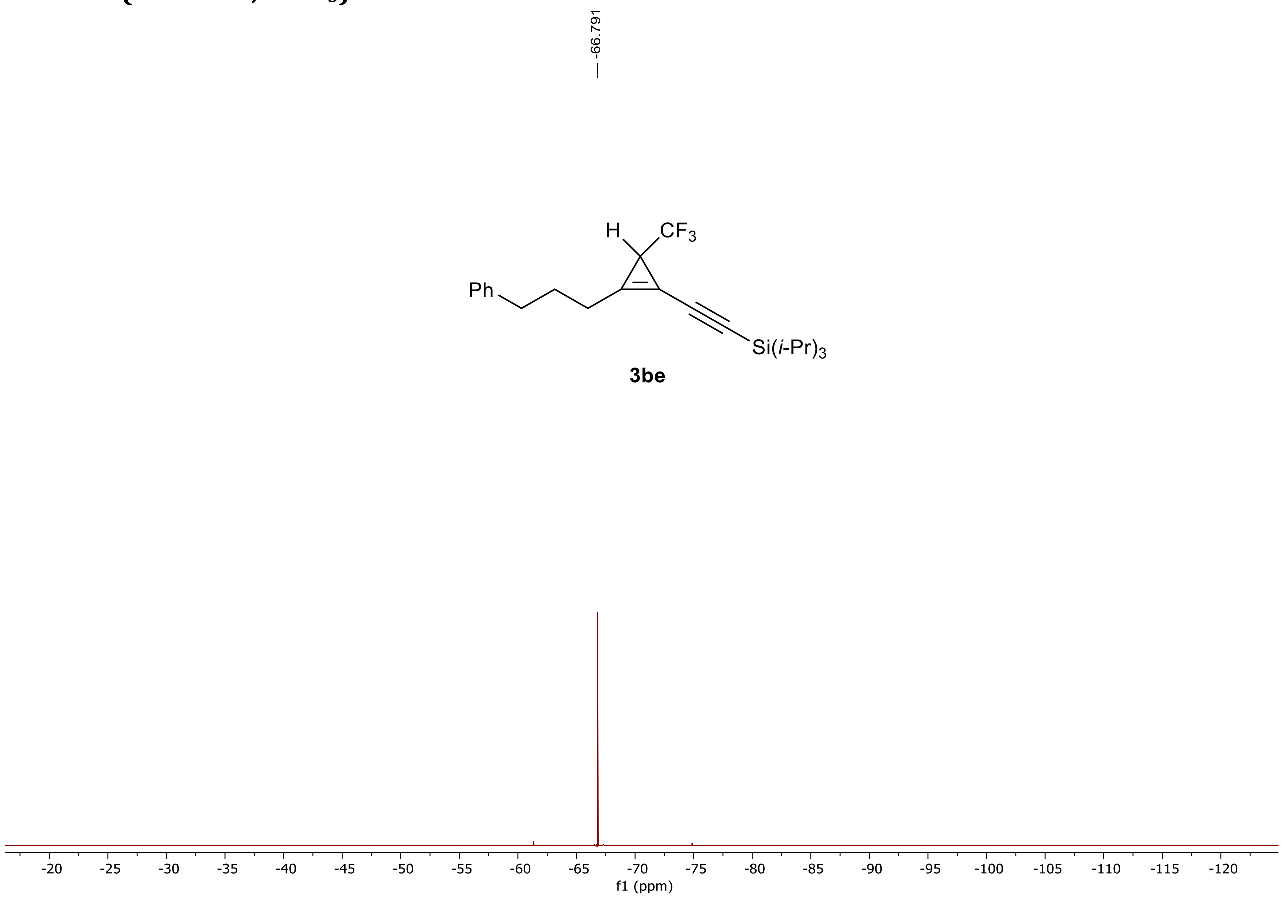

^{1}H NMR (400 MHz, $CDCl_3$) of 3bf

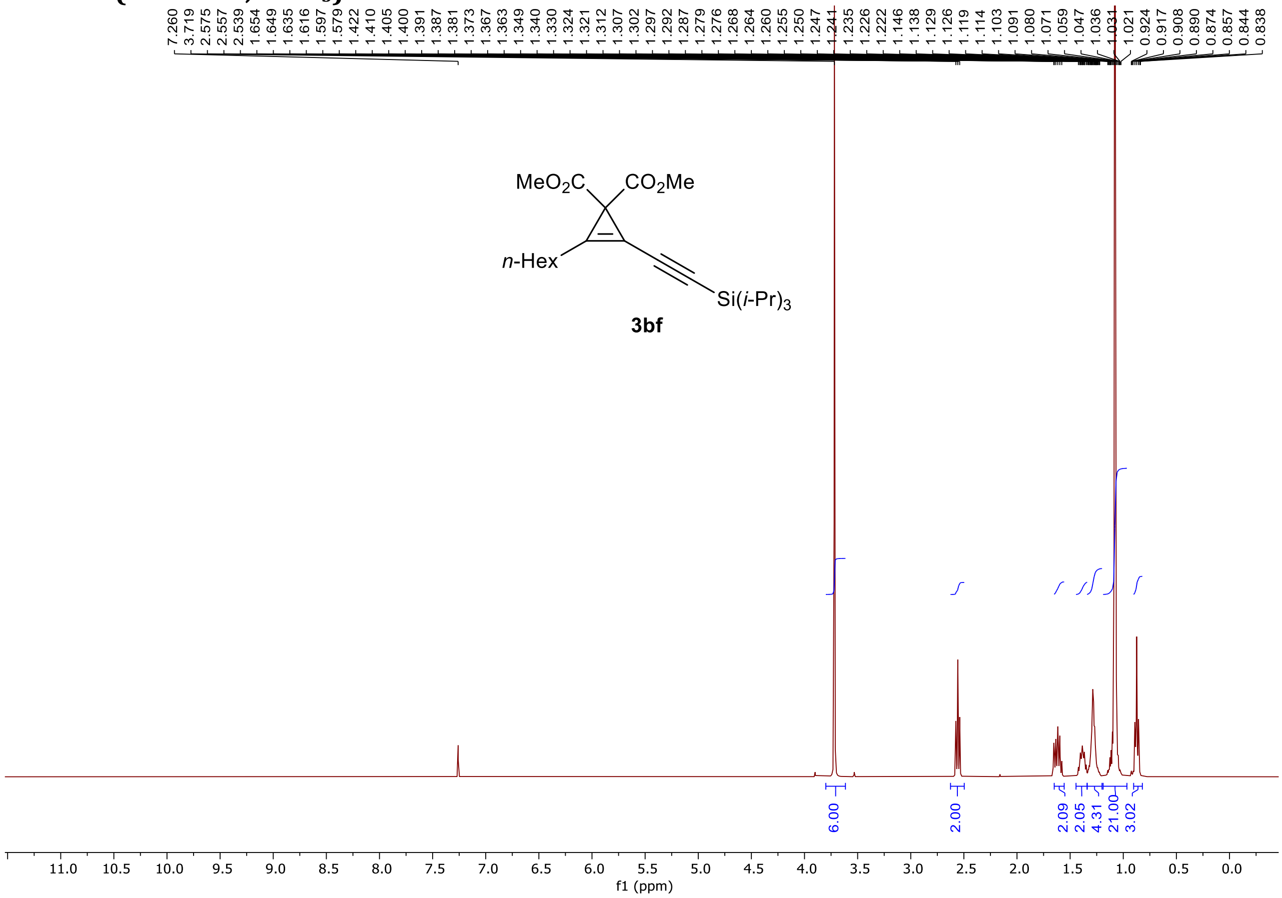

^{13}C NMR (101 MHz, $CDCl_3$) of 3bf

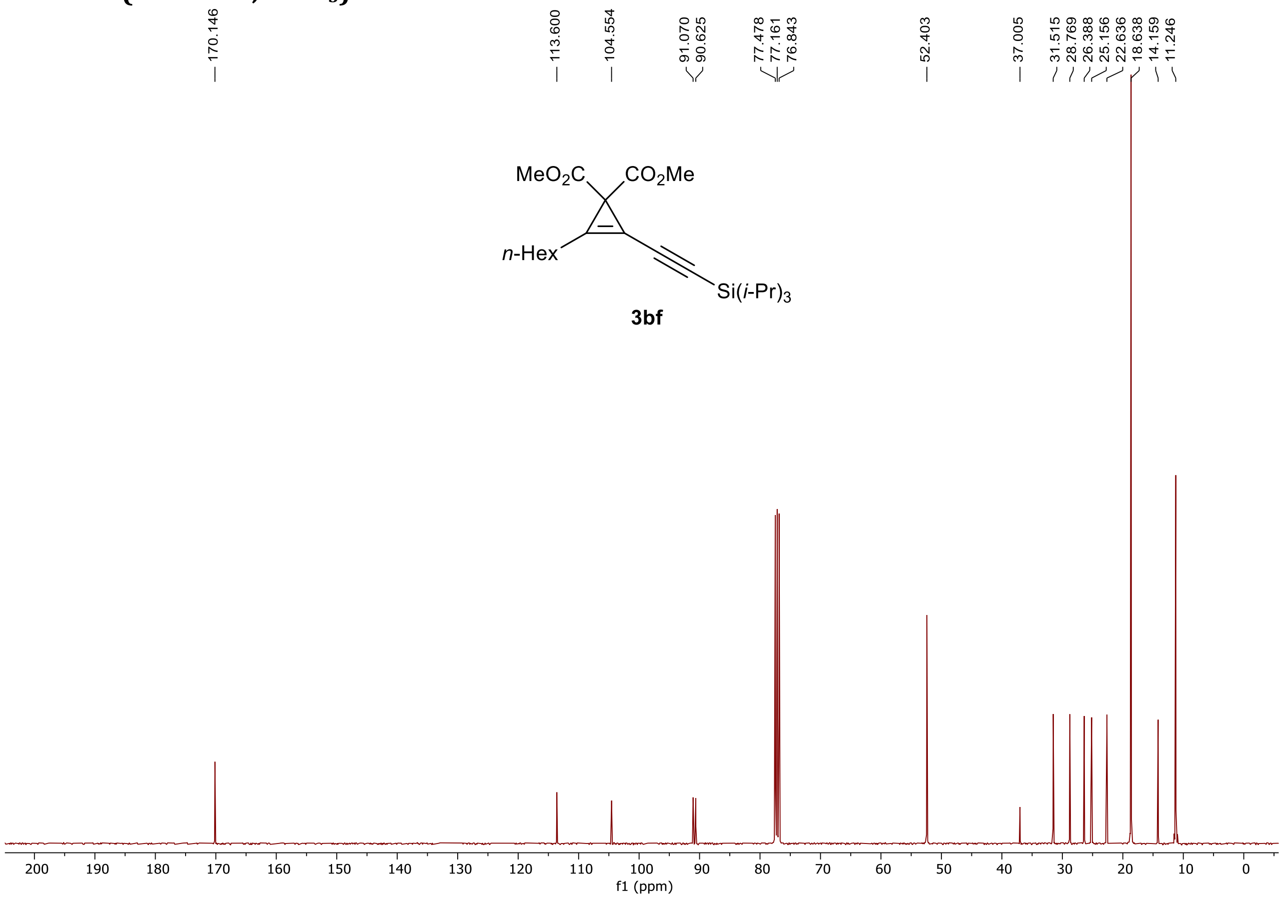

^{1}H NMR (400 MHz, $CDCl_3$) of 3bg

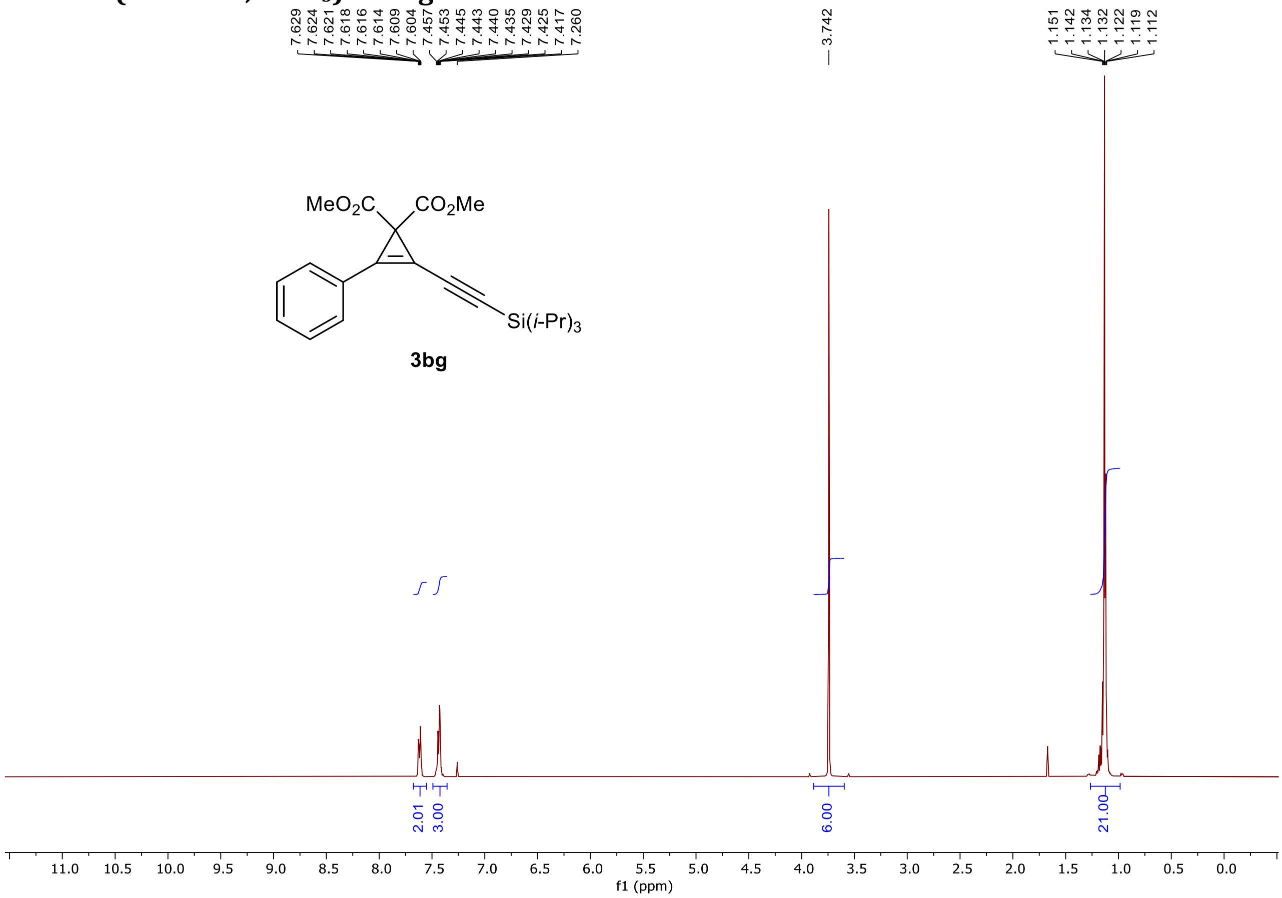

^{13}C NMR (101 MHz, $CDCl_3$) of 3bg

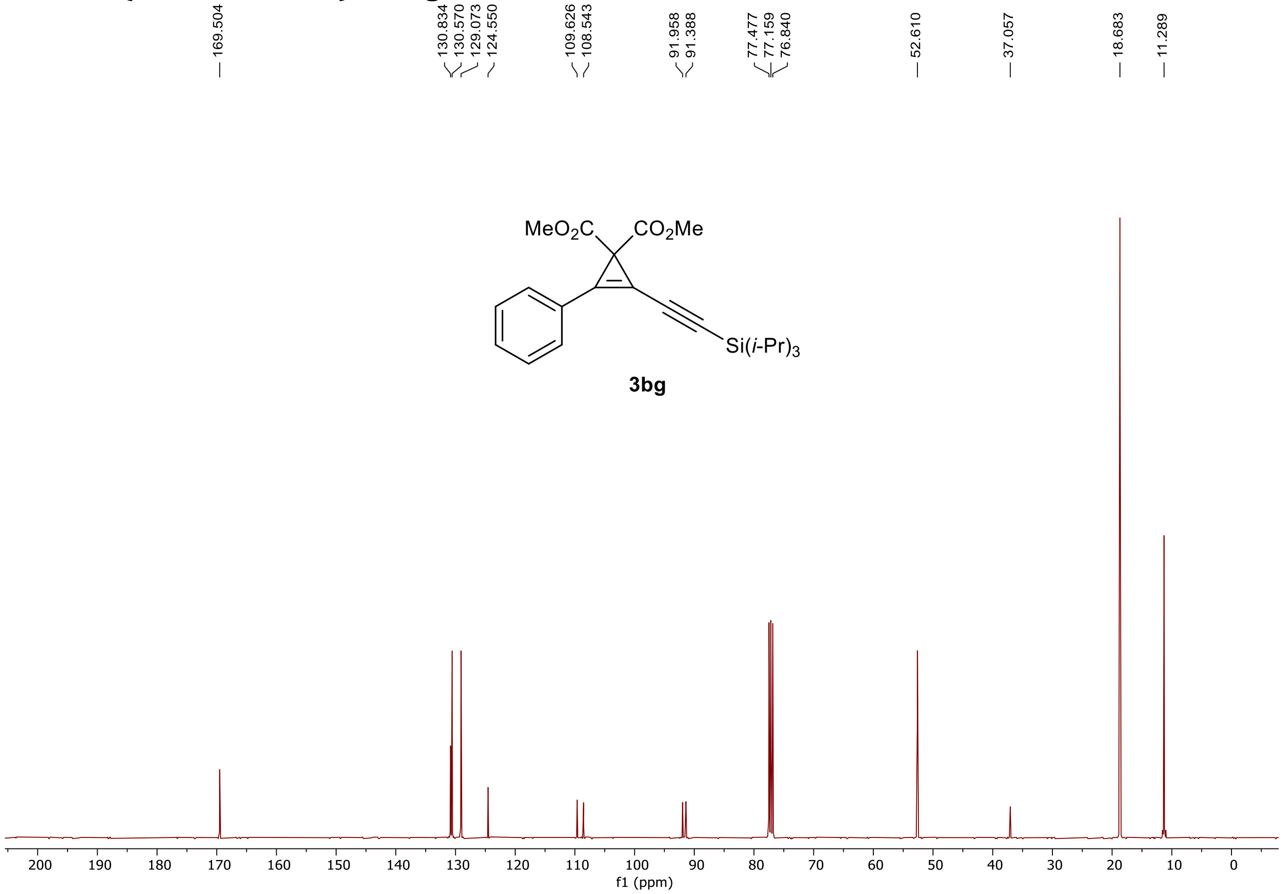

^1H NMR (400 MHz, $CDCl_3$) of 3bh

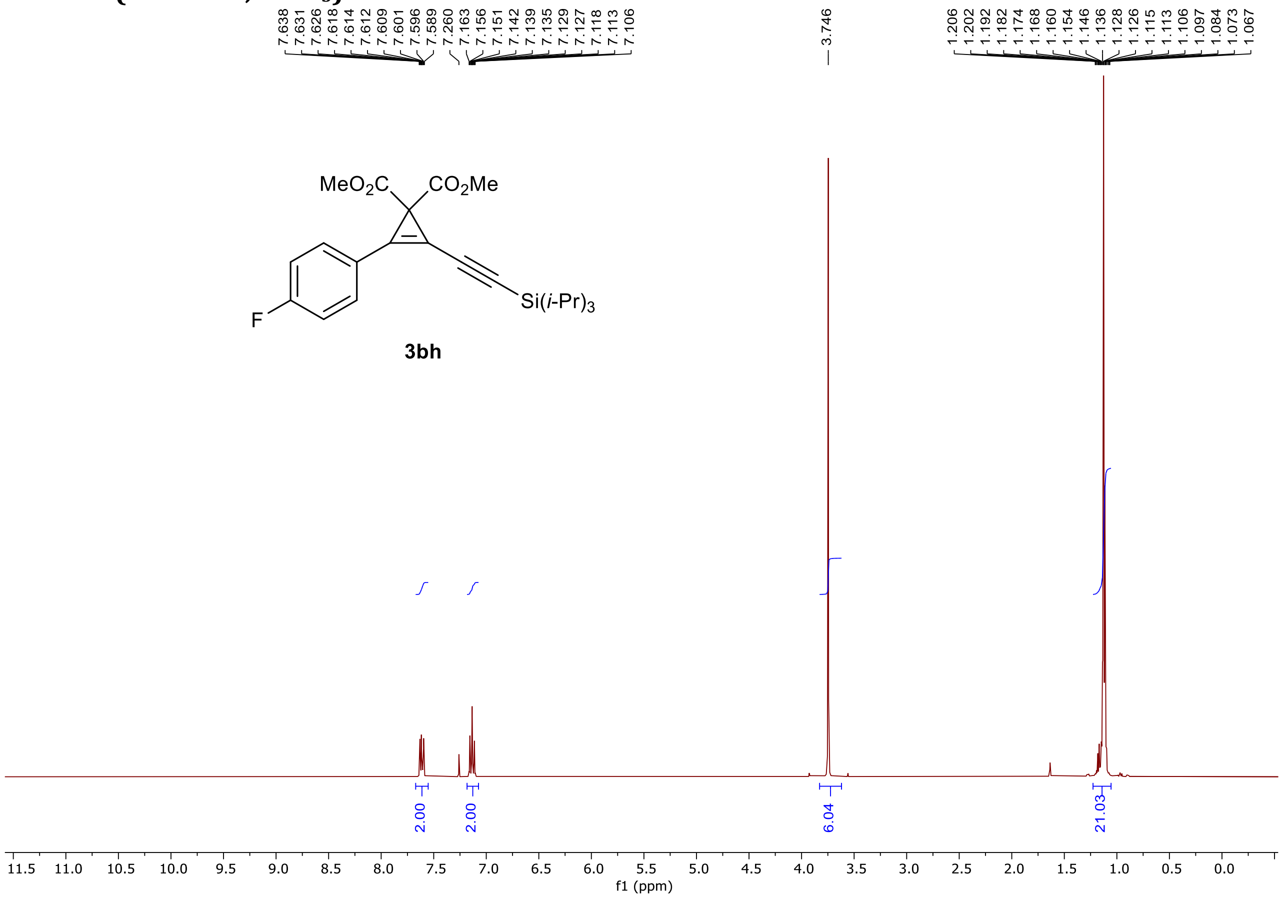

^{13}C NMR (101 MHz, $CDCl_3$) of 3bh

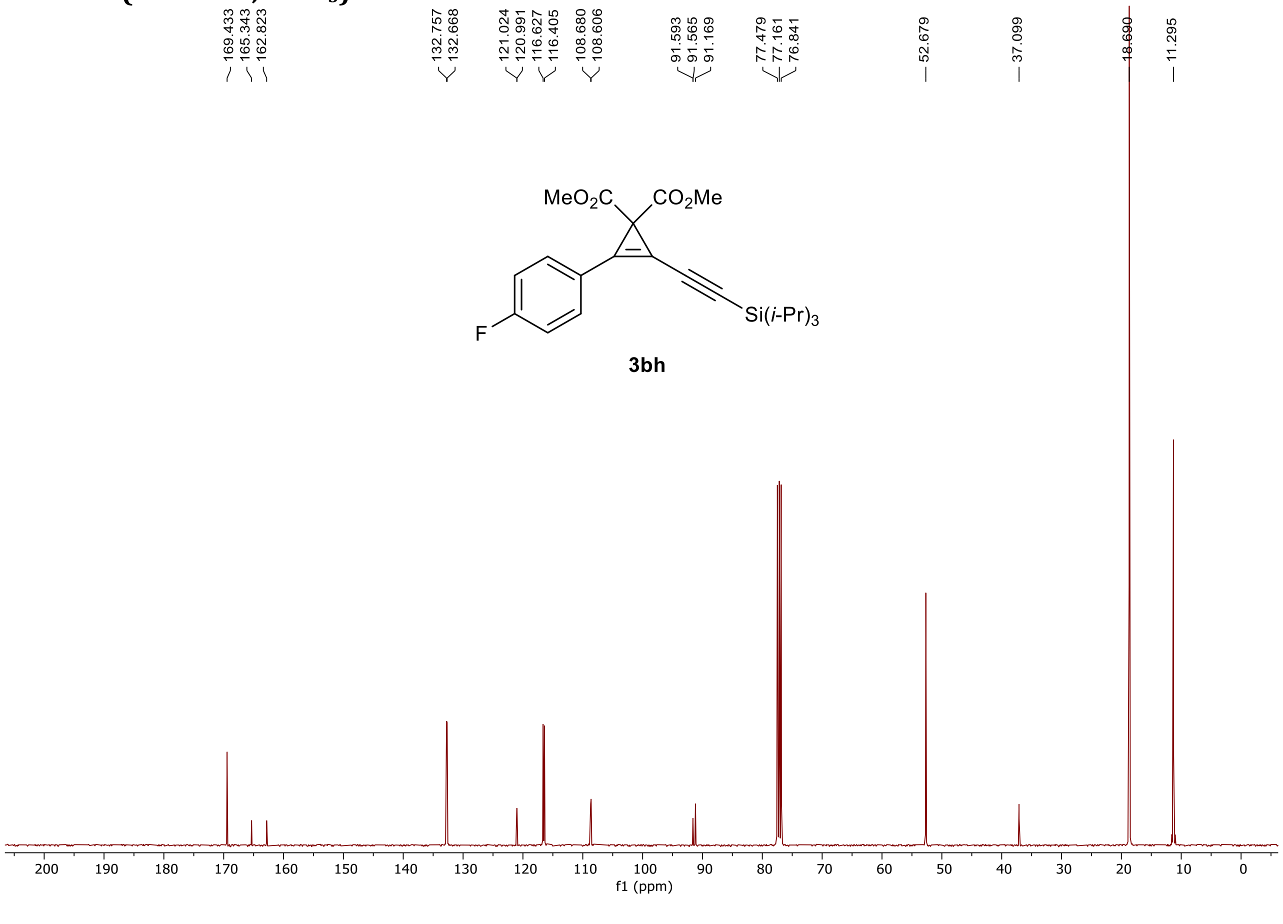

^{19}F NMR (377 MHz, $CDCl_3$) of 3bh

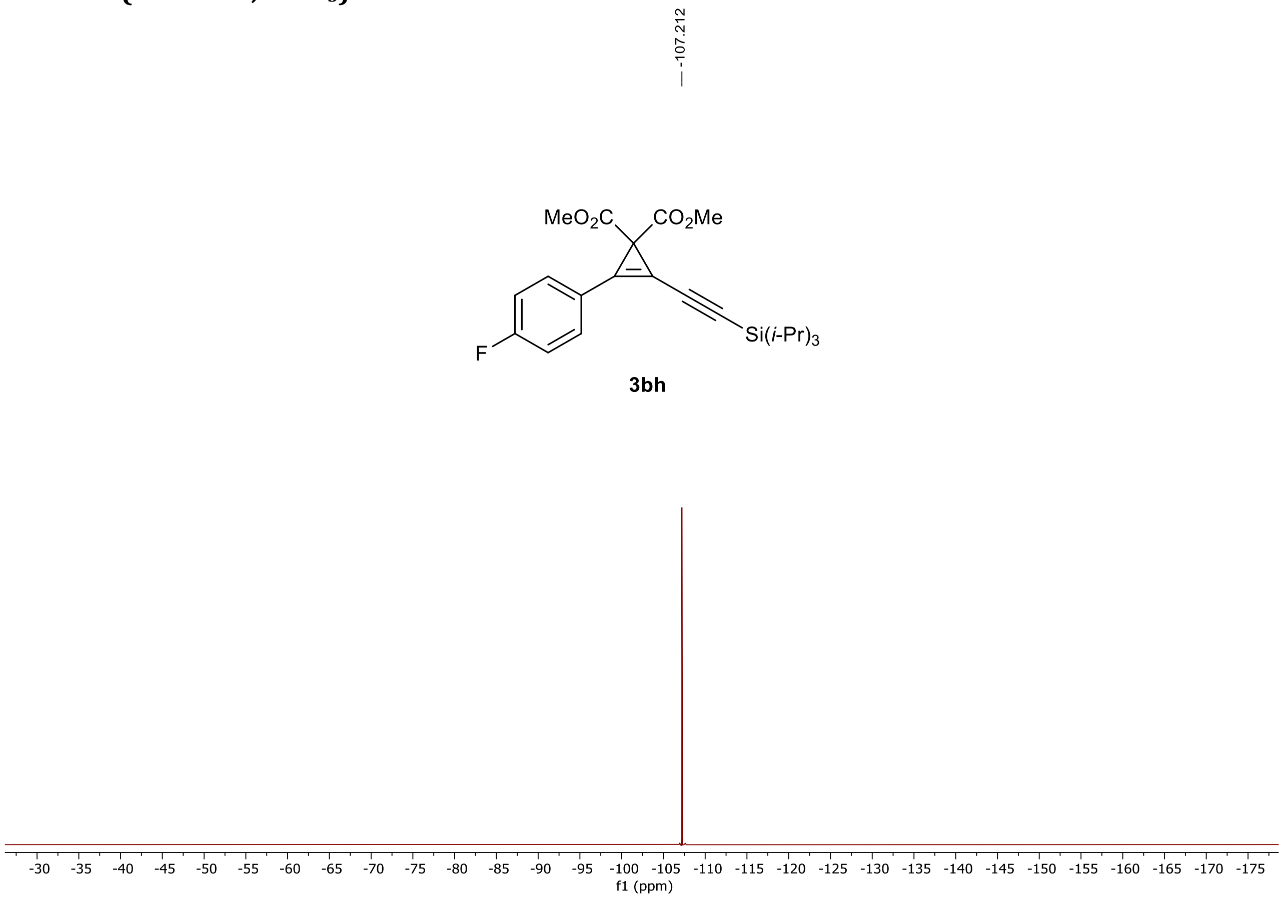

^{1}H NMR (400 MHz, CD_3CN) of 3bi

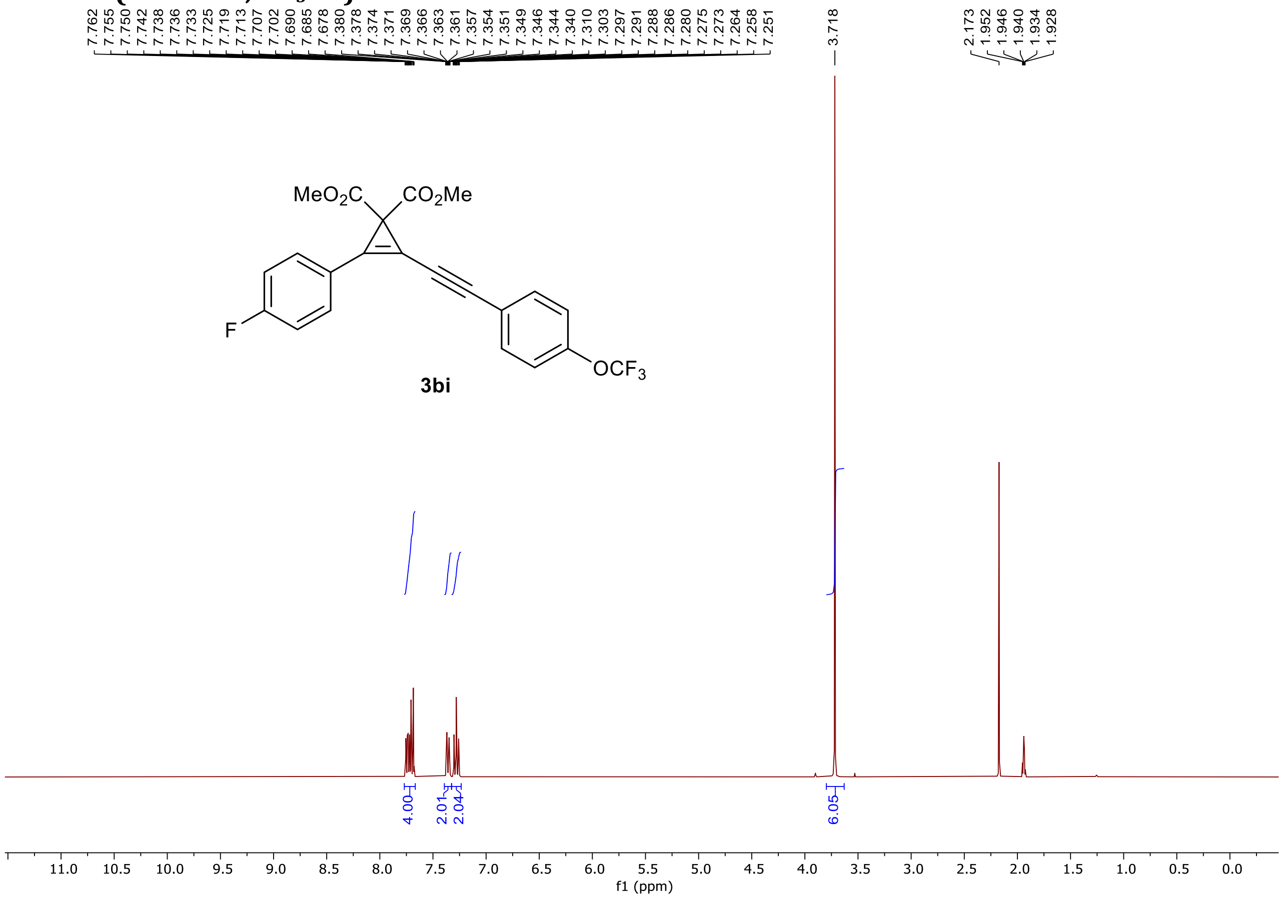

^{13}C NMR (101 MHz, CD_3CN) of 3bi

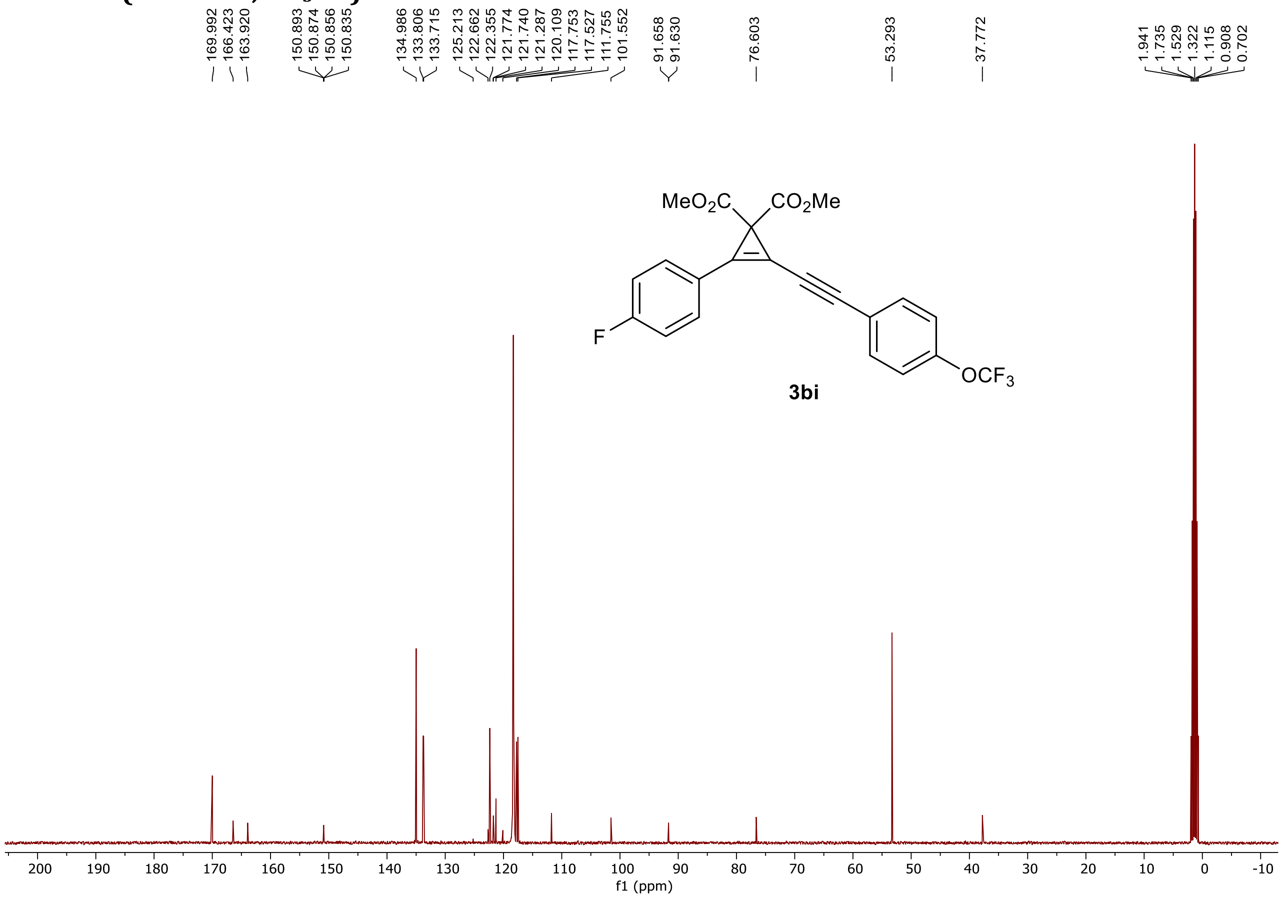

^{19}F NMR (377 MHz, CD_3CN) of 3bi

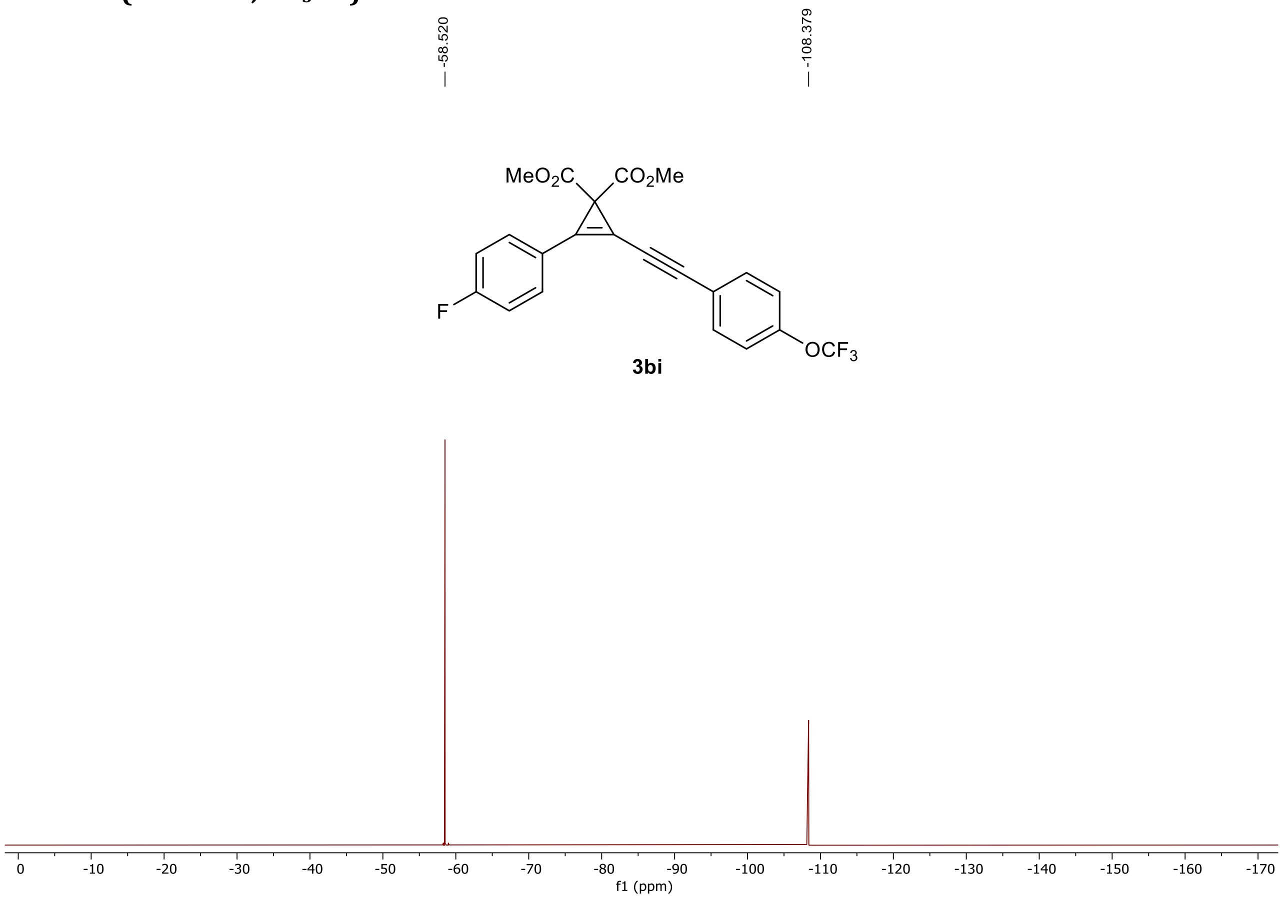

^{1}H NMR (400 MHz, $CDCl_3$) of 3bj

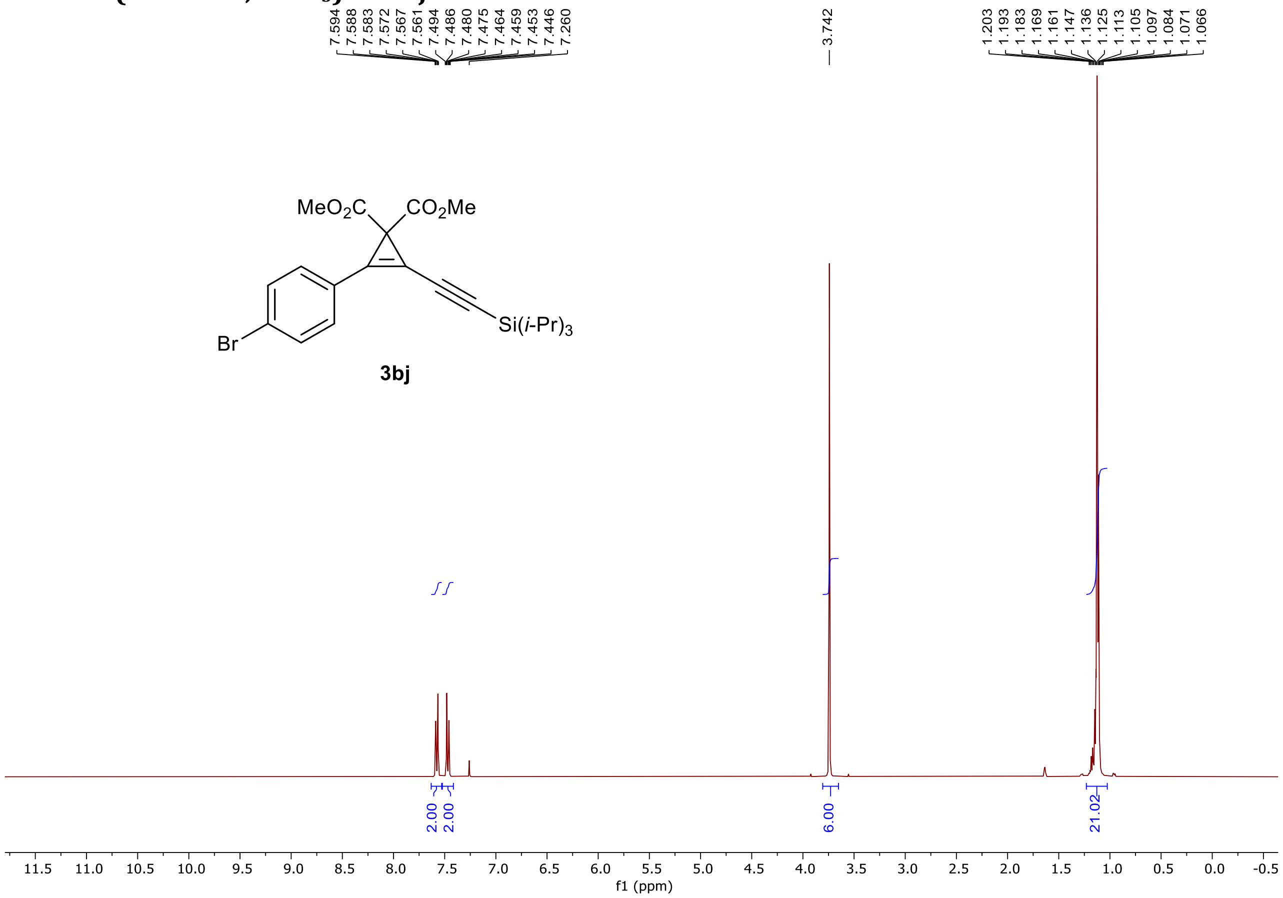

^{13}C NMR (101 MHz, $CDCl_3$) of 3bj

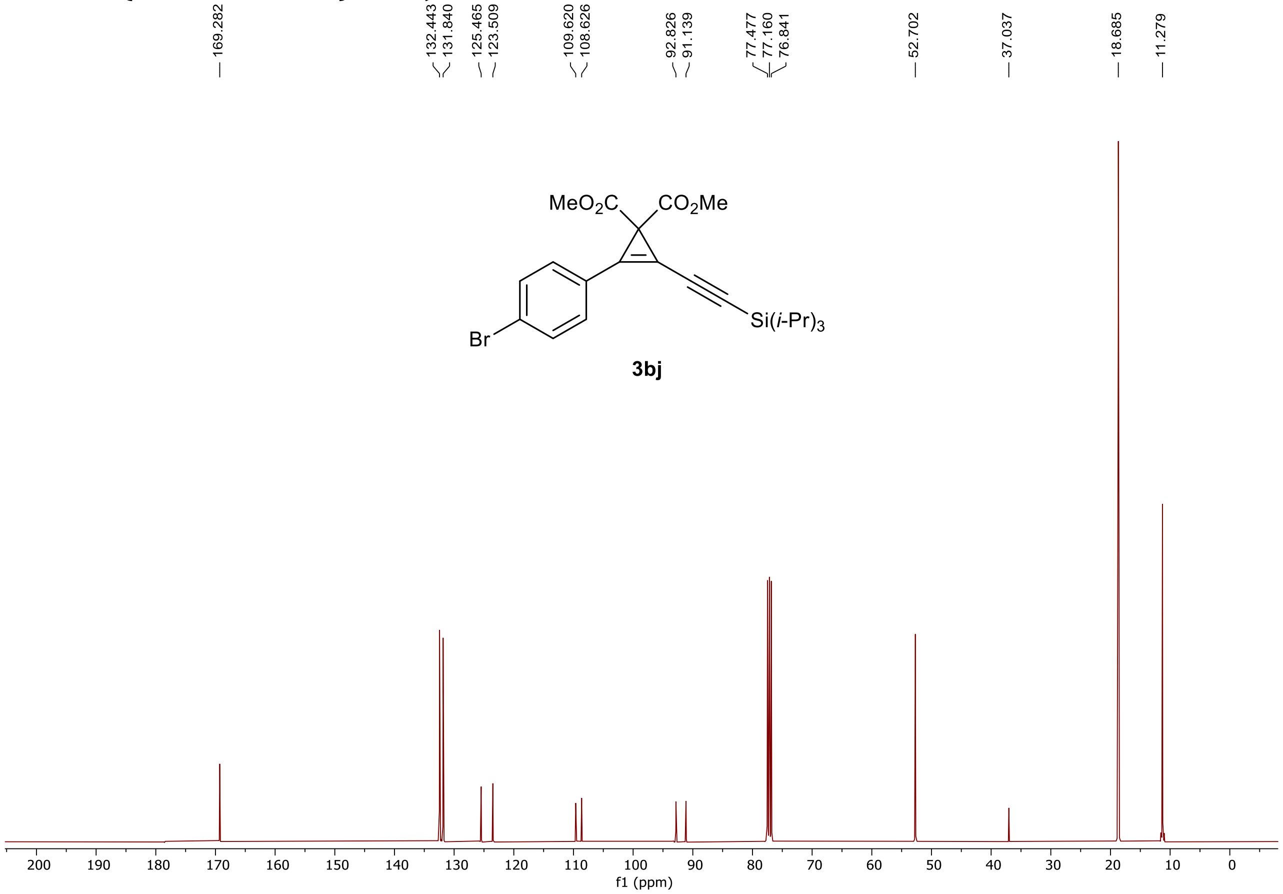

^{1}H NMR (400 MHz, $CDCl_3$) of 3bk

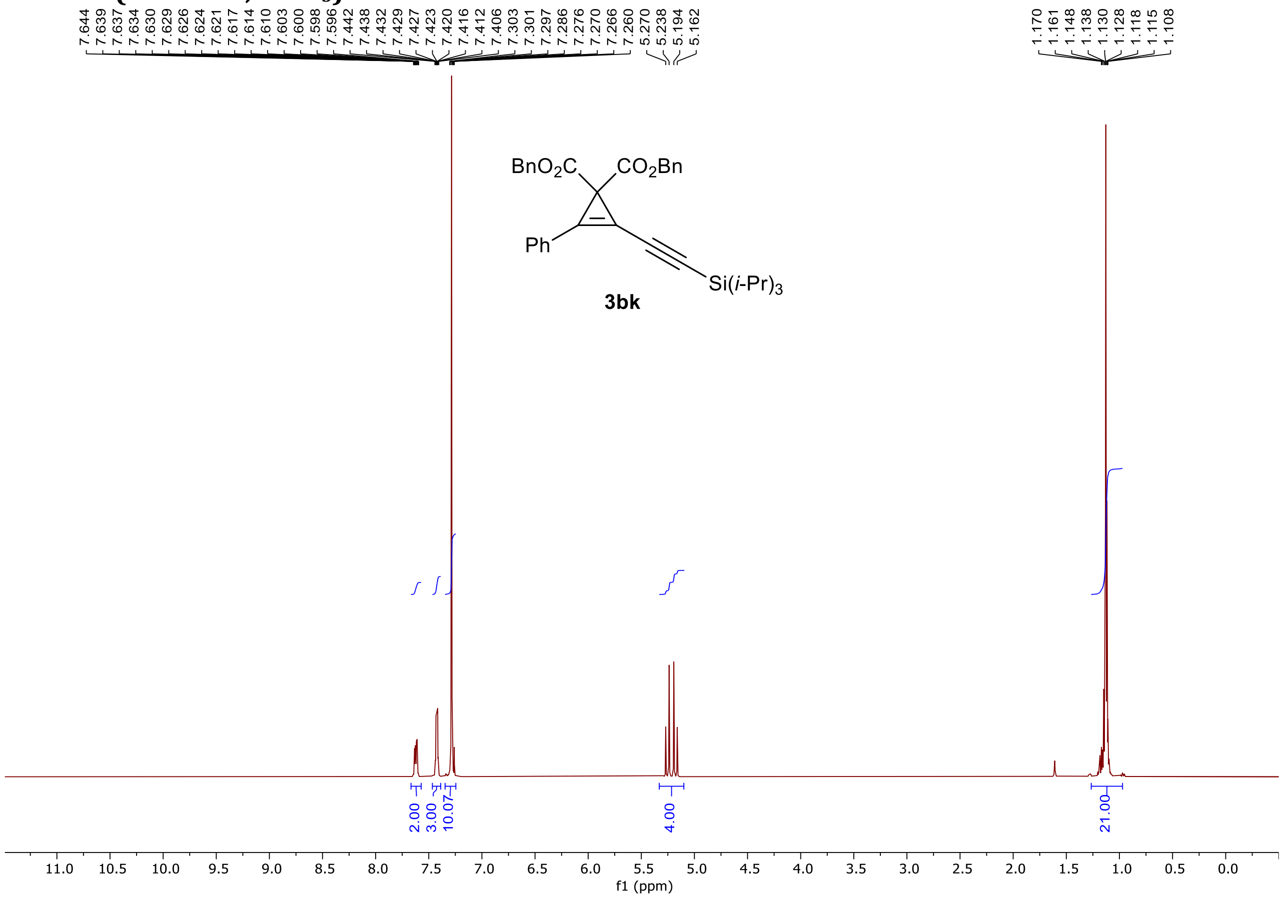

^{13}C NMR (101 MHz, $CDCl_3$) of 3bk

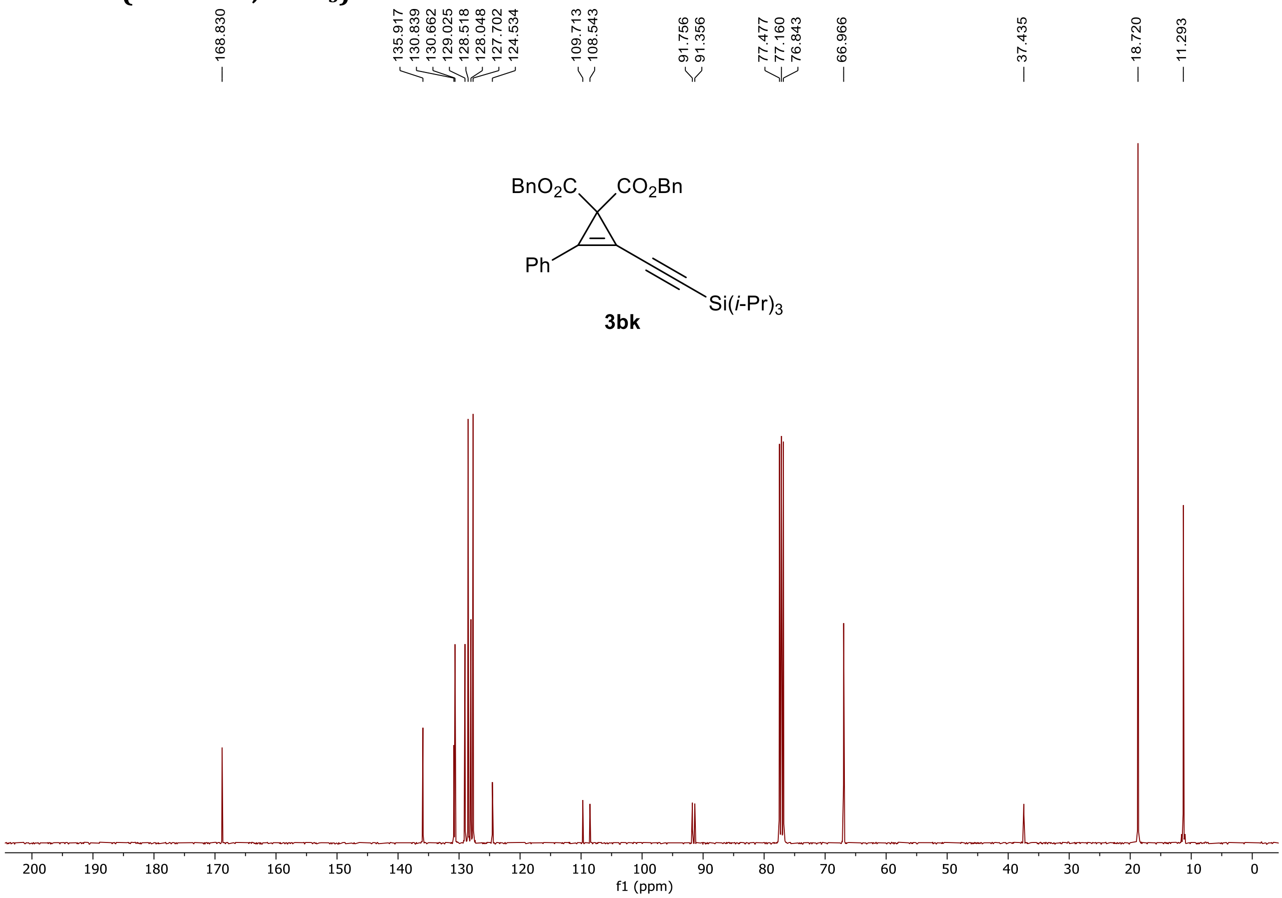

^{1}H NMR (400 MHz, $CDCl_3$) of 3bl

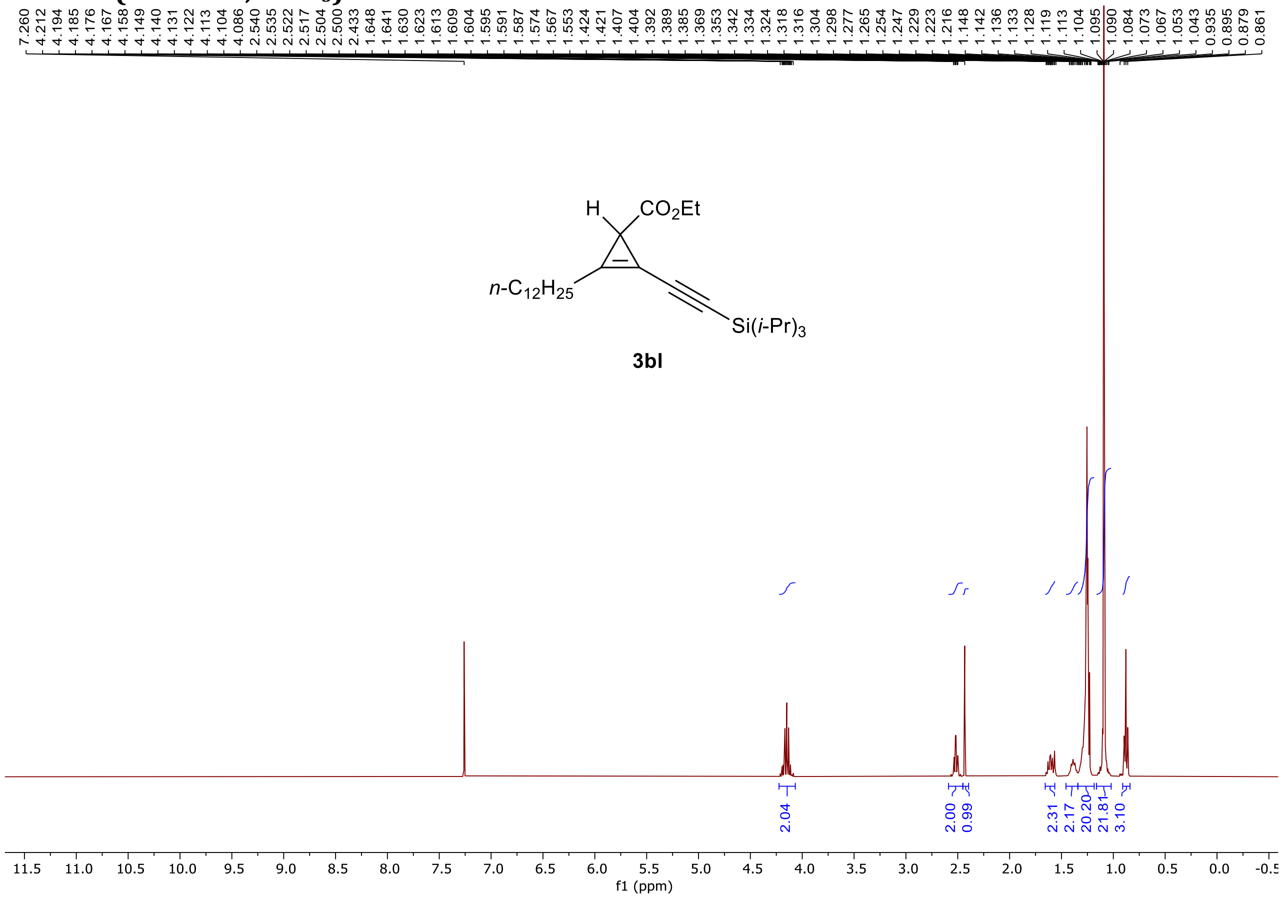

^{13}C NMR (101 MHz, $CDCl_3$) of 3bl

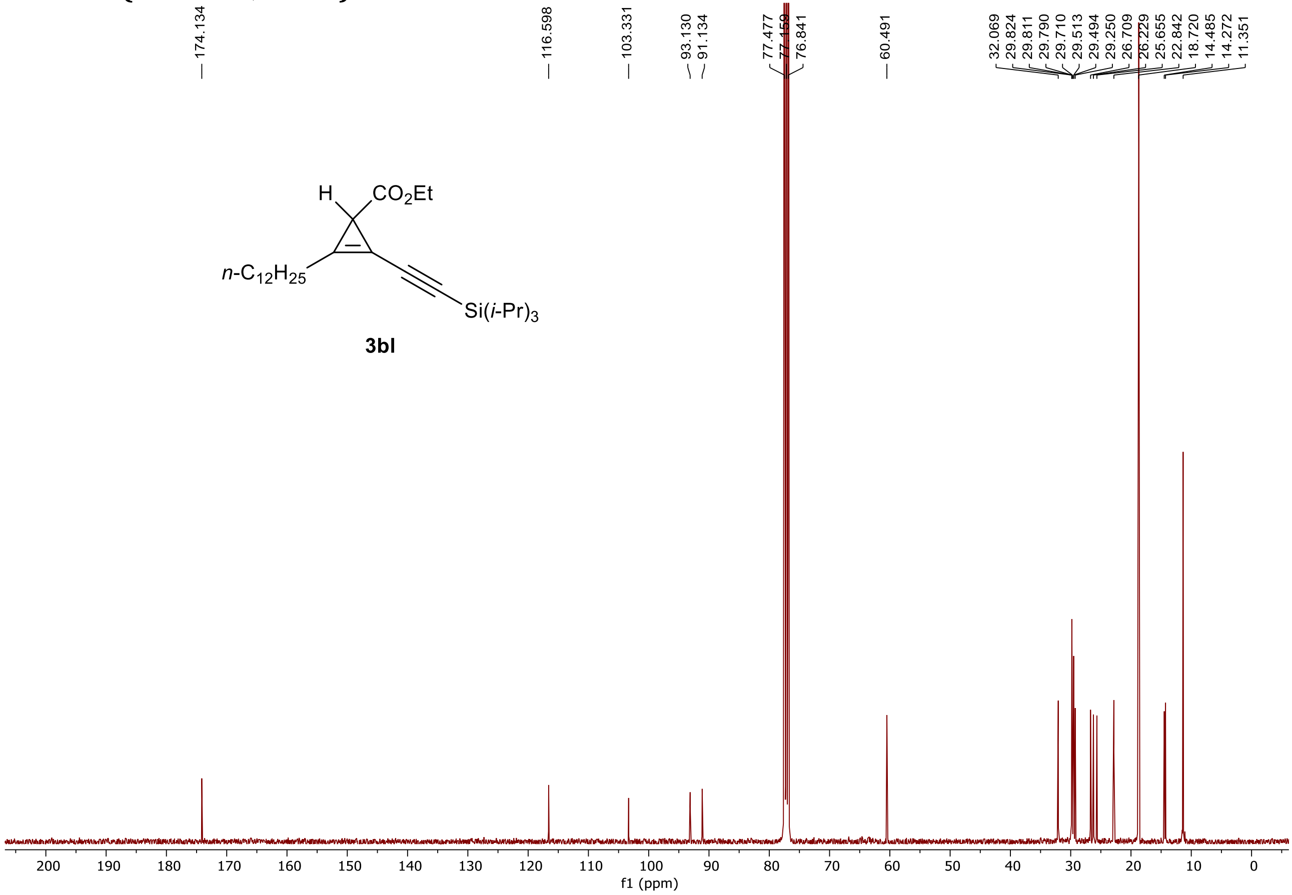

^{1}H NMR (400 MHz, $CDCl_3$) of 3bm

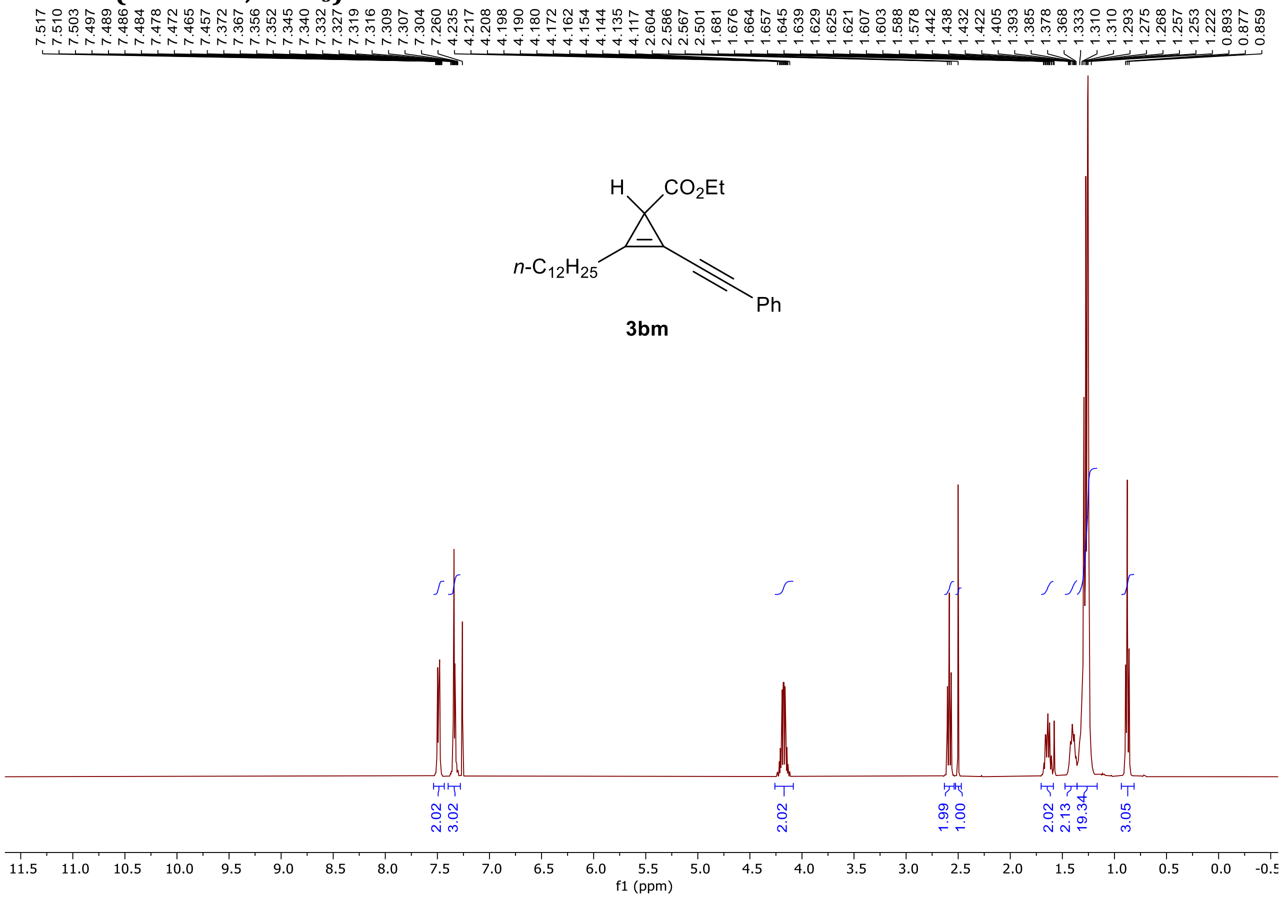

^{13}C NMR (101 MHz, $CDCl_3$) of 3bm

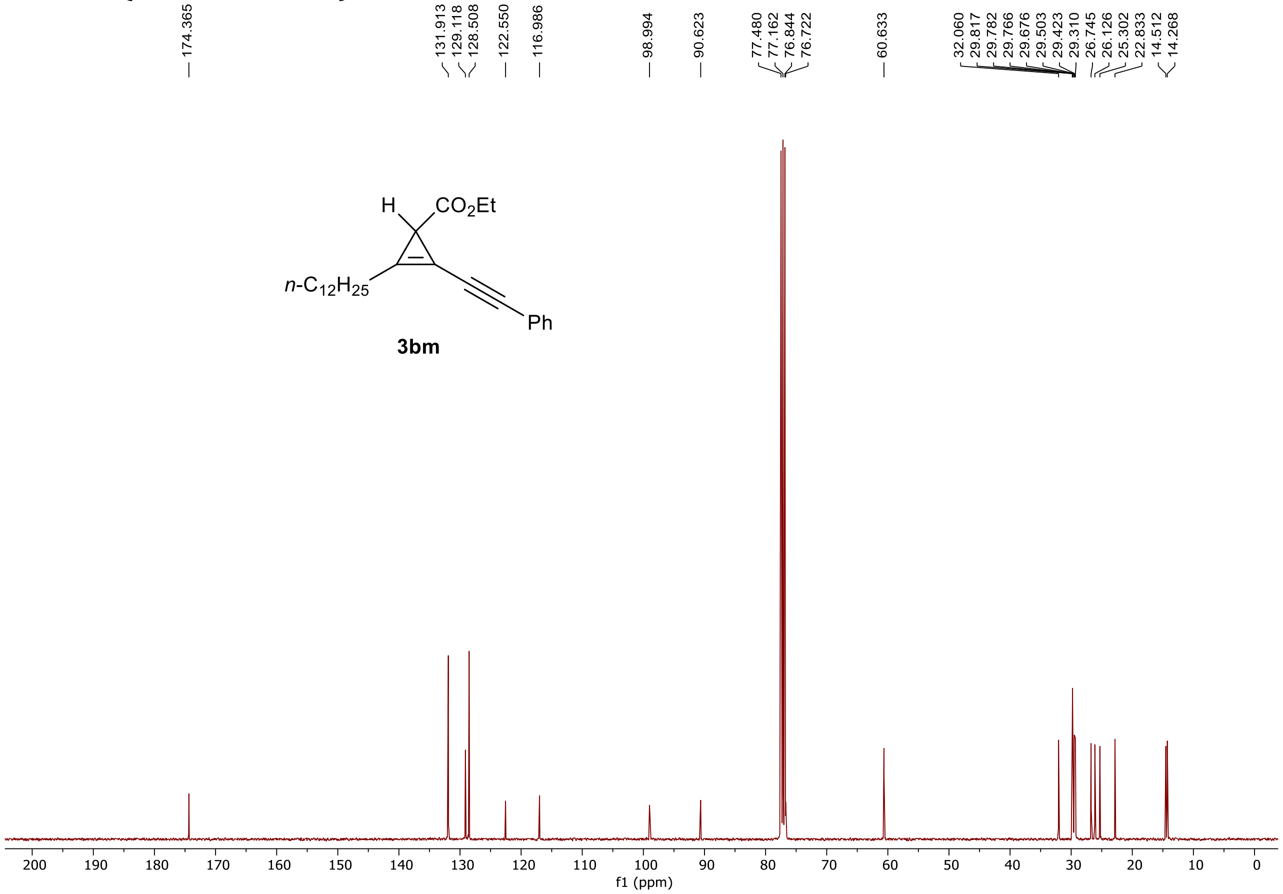

^{1}H NMR (400 MHz, $CDCl_3$) of 3bn

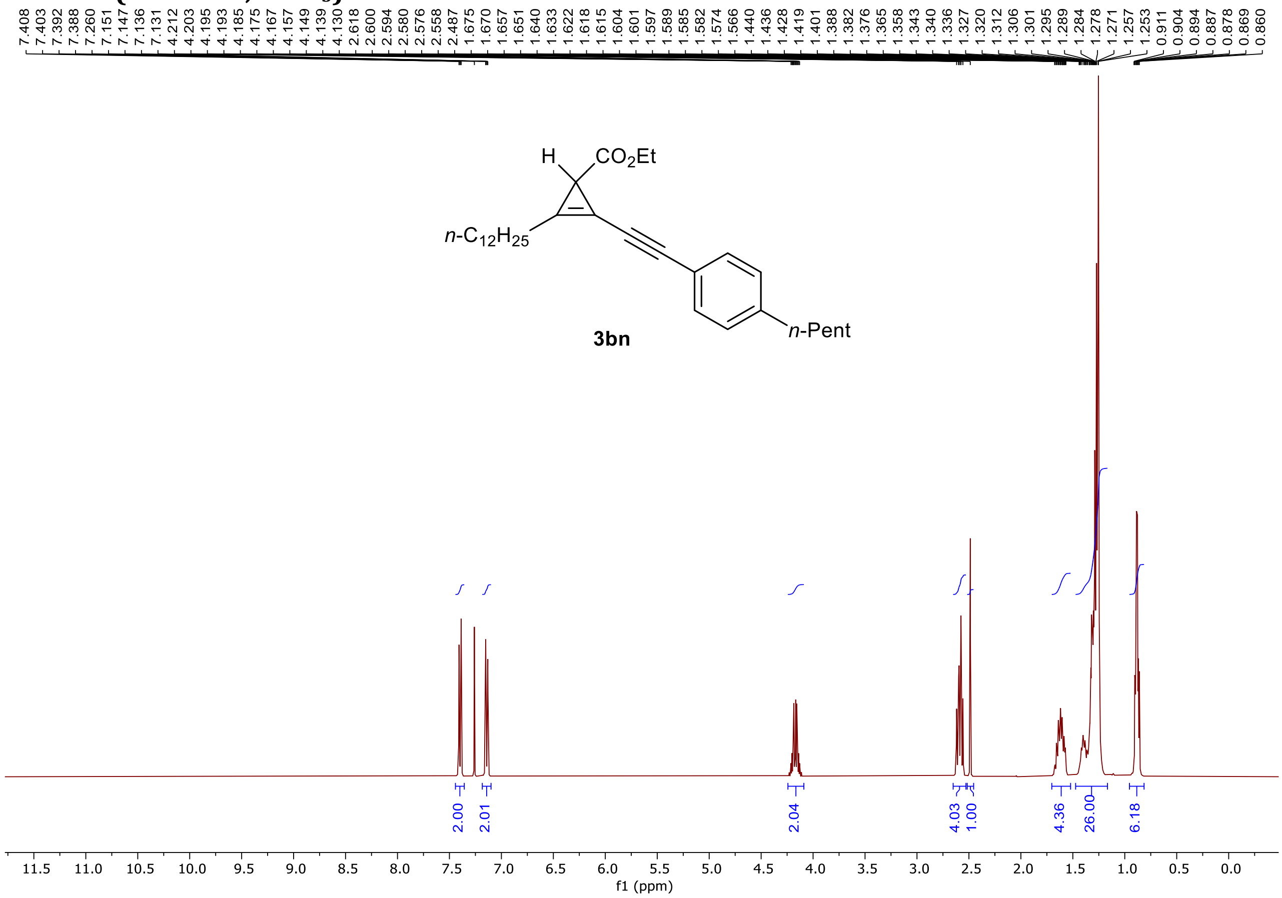

^{13}C NMR (101 MHz, $CDCl_3$) of 3bn

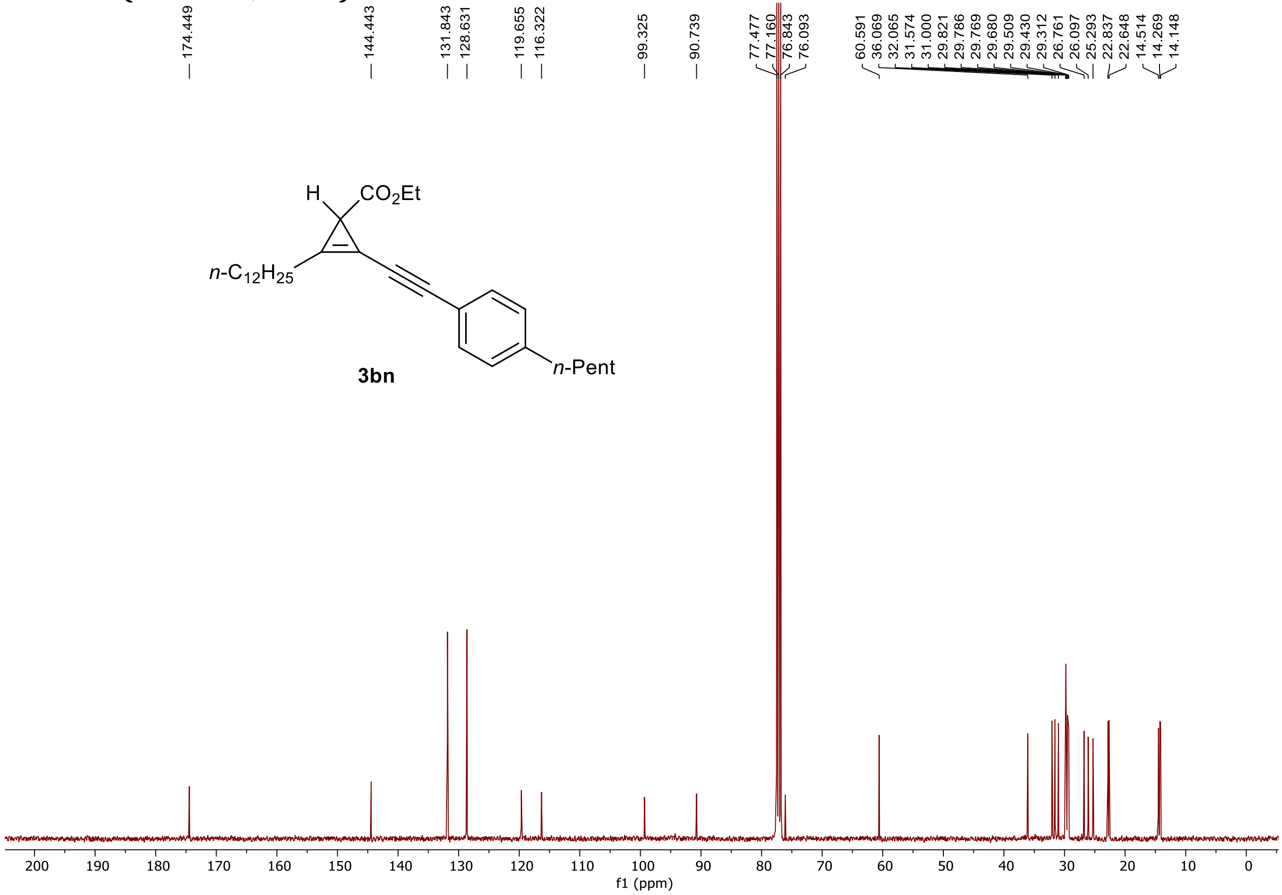

^{1}H NMR (400 MHz, $CDCl_3$) of 3bo

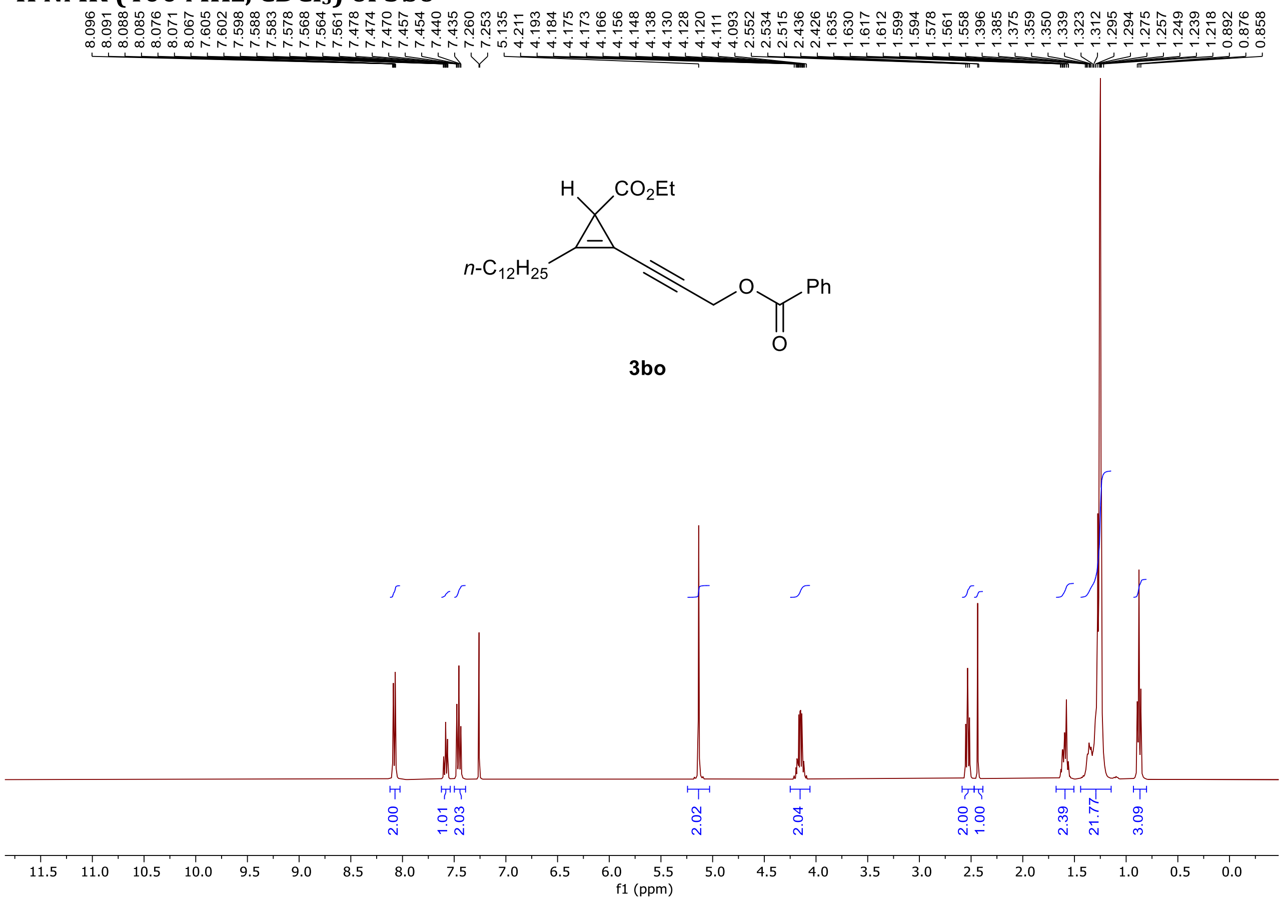

^{13}C NMR (101 MHz, $CDCl_3$) of 3bo

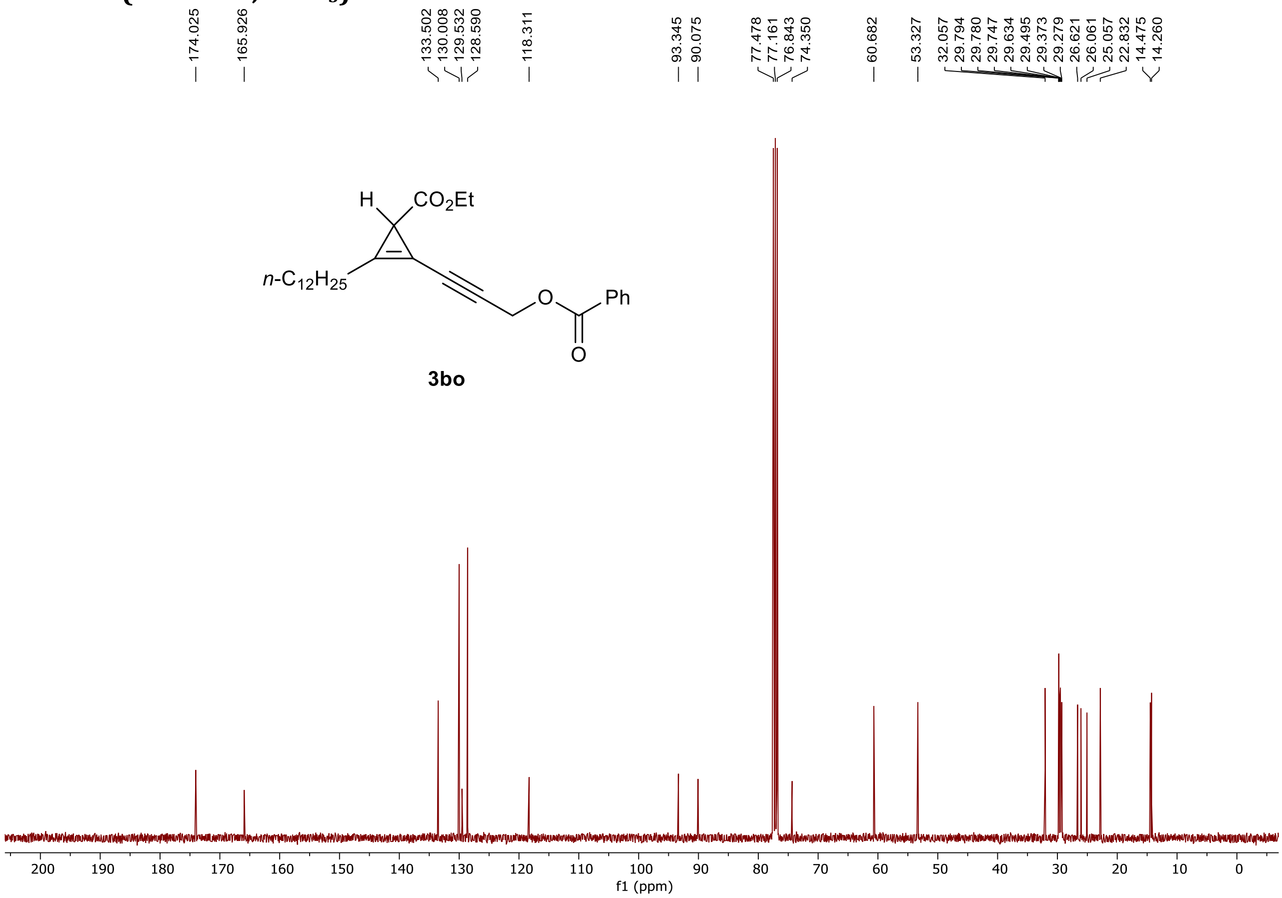

^{1}H NMR (400 MHz, $CDCl_3$) of 3bp

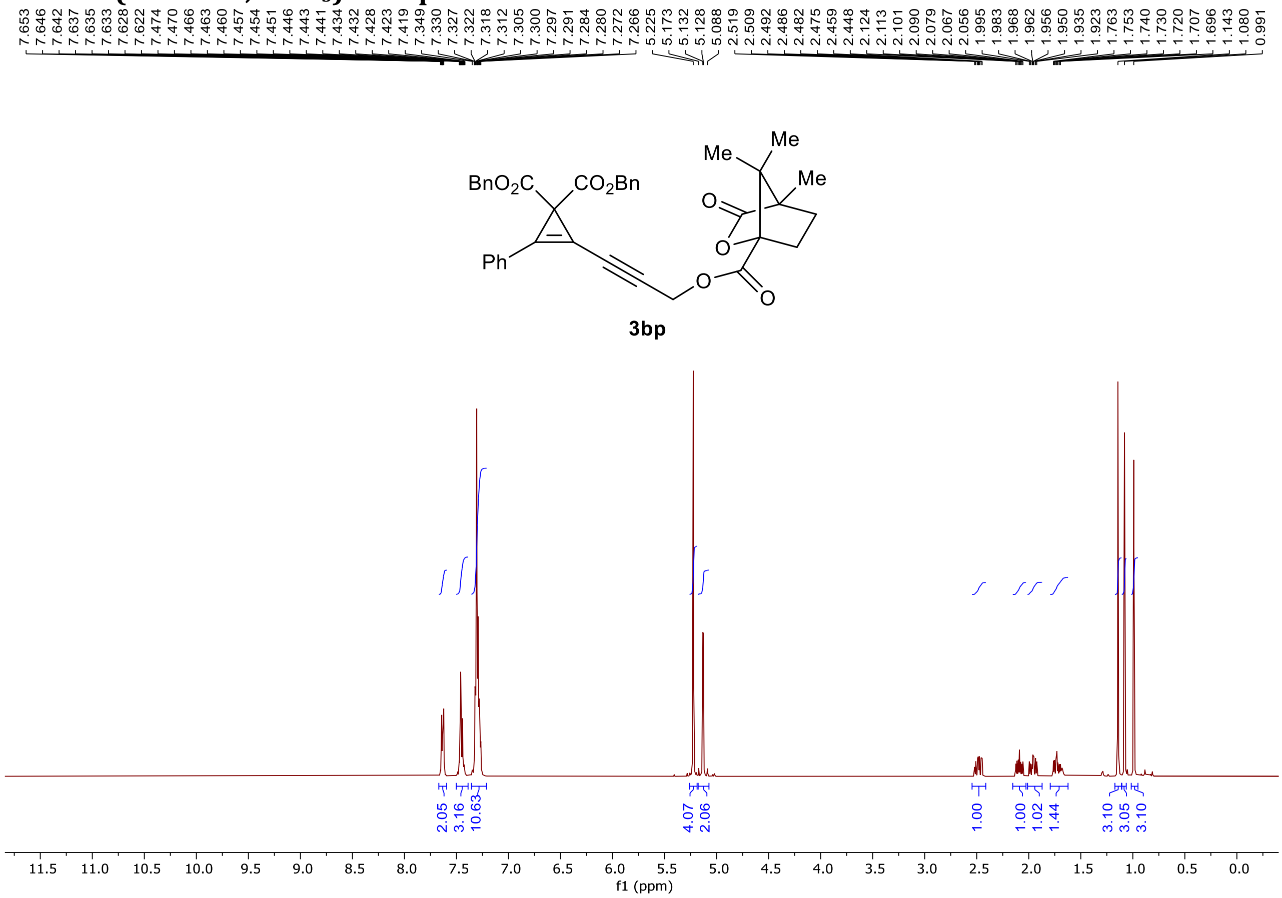

^{13}C NMR (101 MHz, $CDCl_3$) of 3bp

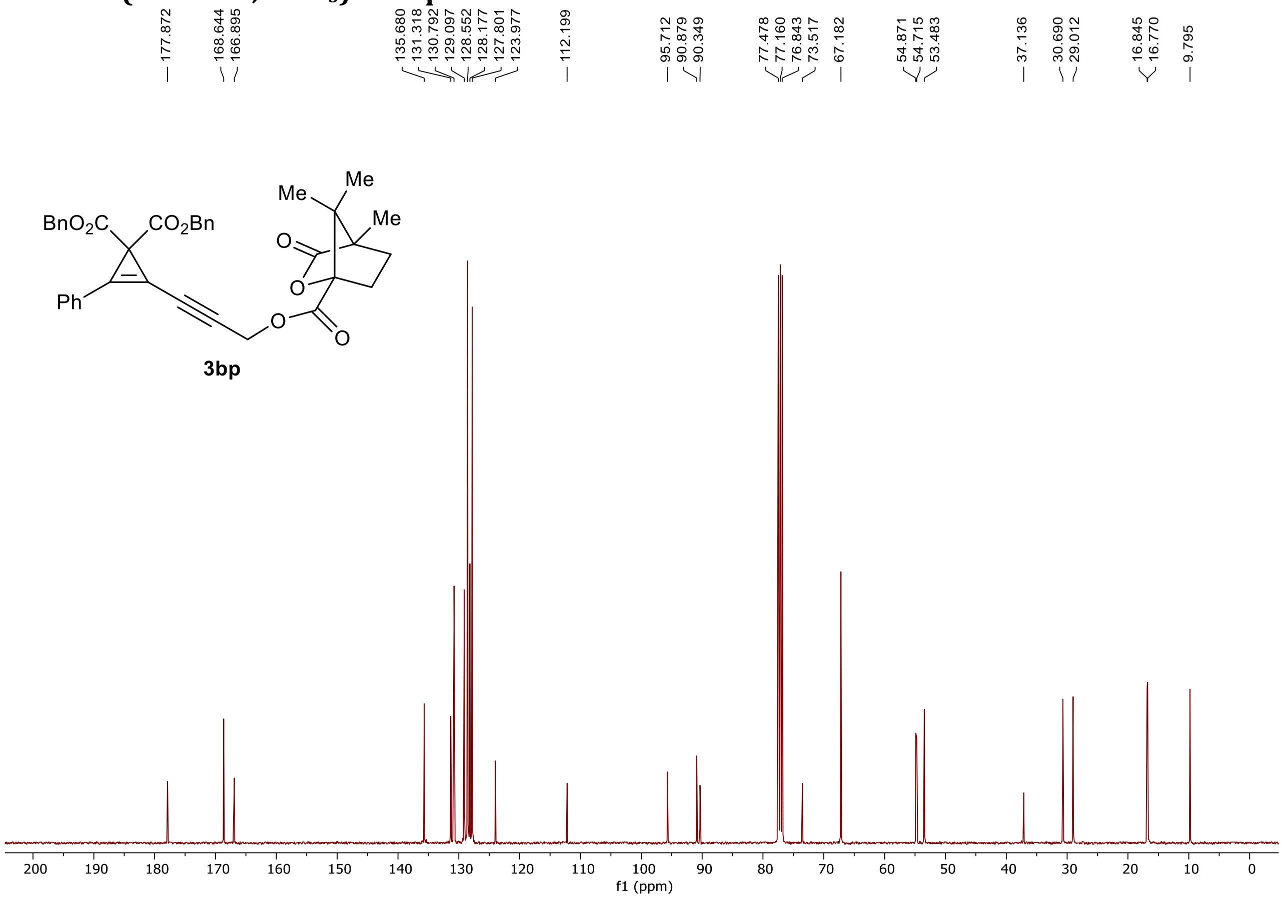

^{1}H NMR (400 MHz, $CDCl_3$) of 3bq

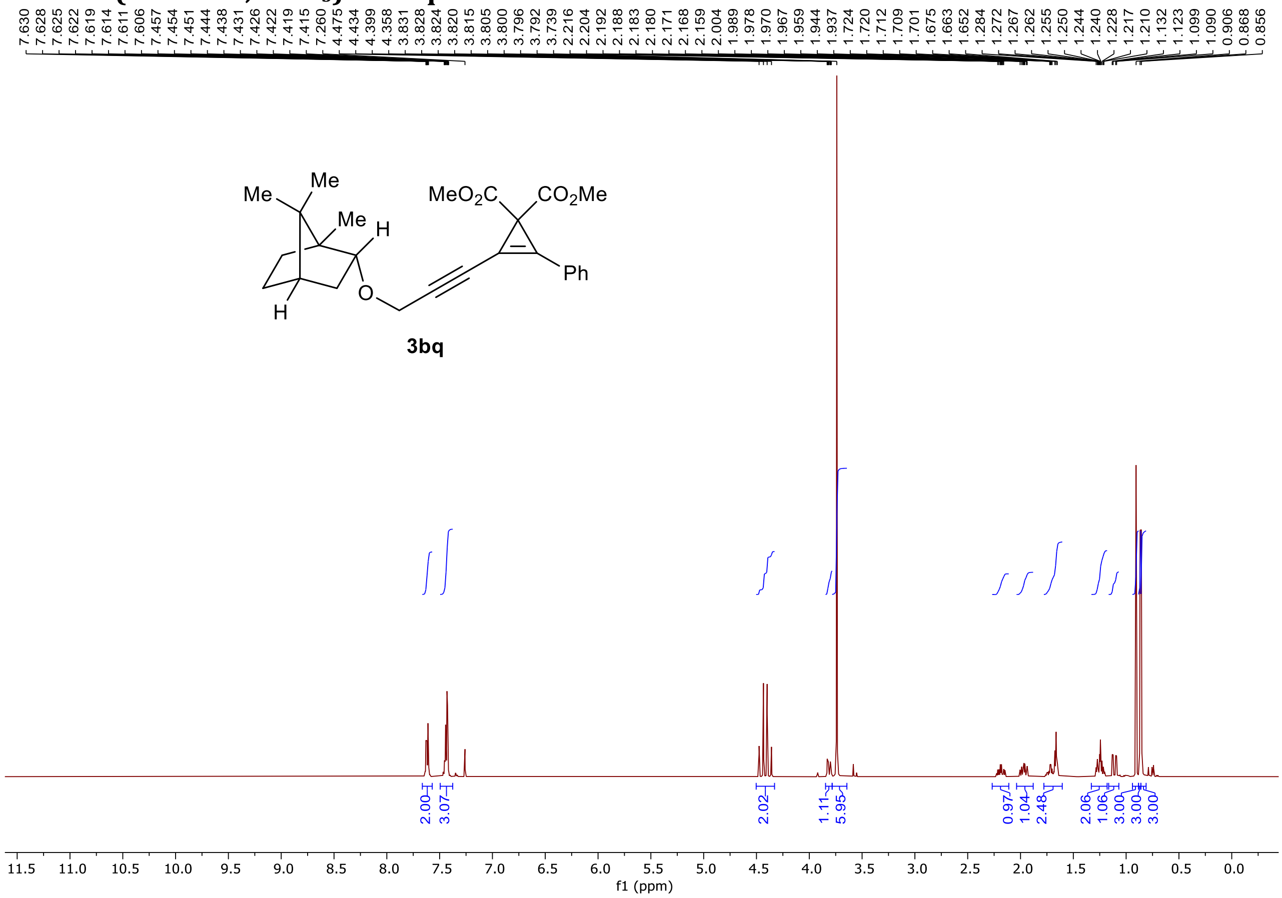

^{13}C NMR (101 MHz, $CDCl_3$) of 3bq

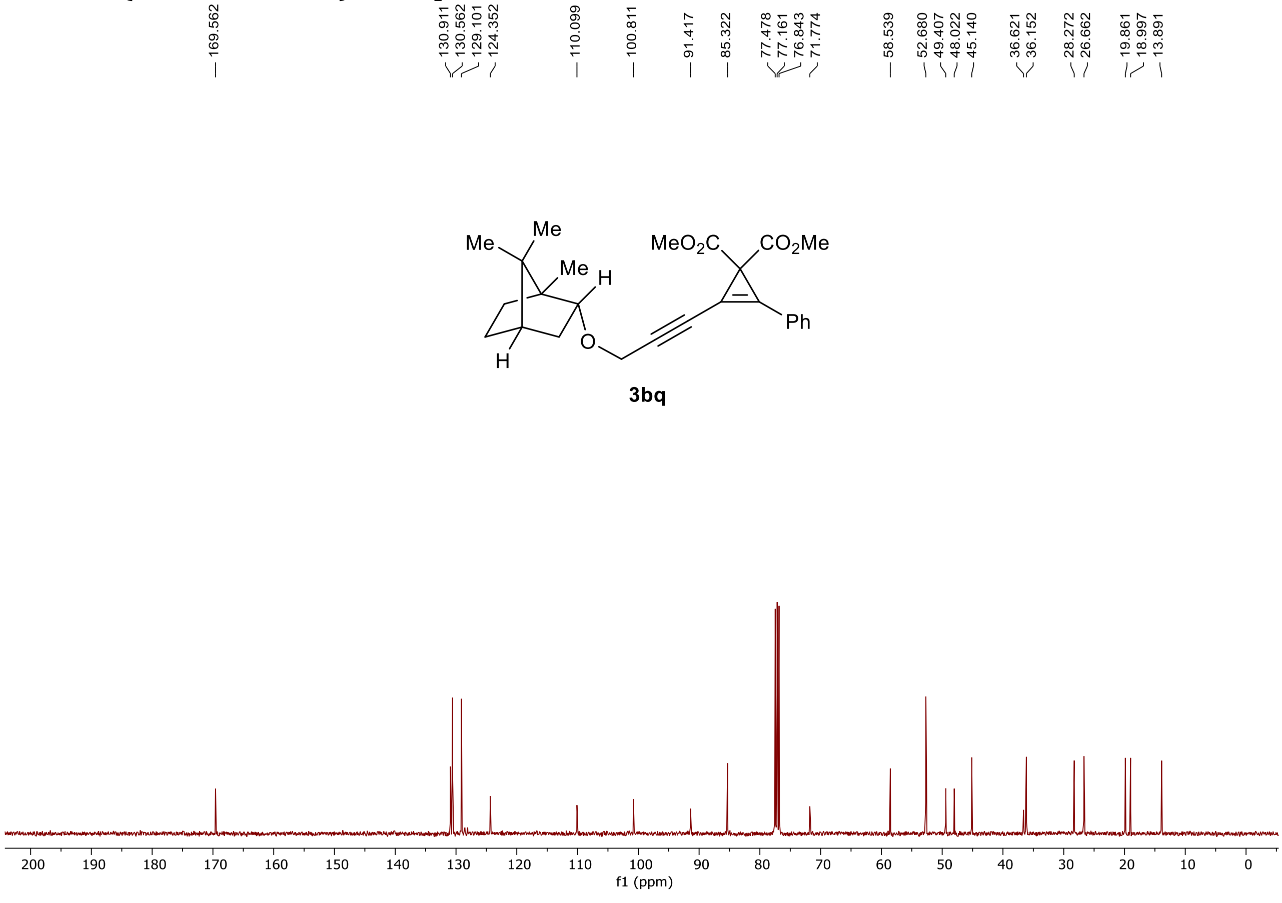

^{1}H NMR (400 MHz, $CDCl_3$, 60 °C) of 3br

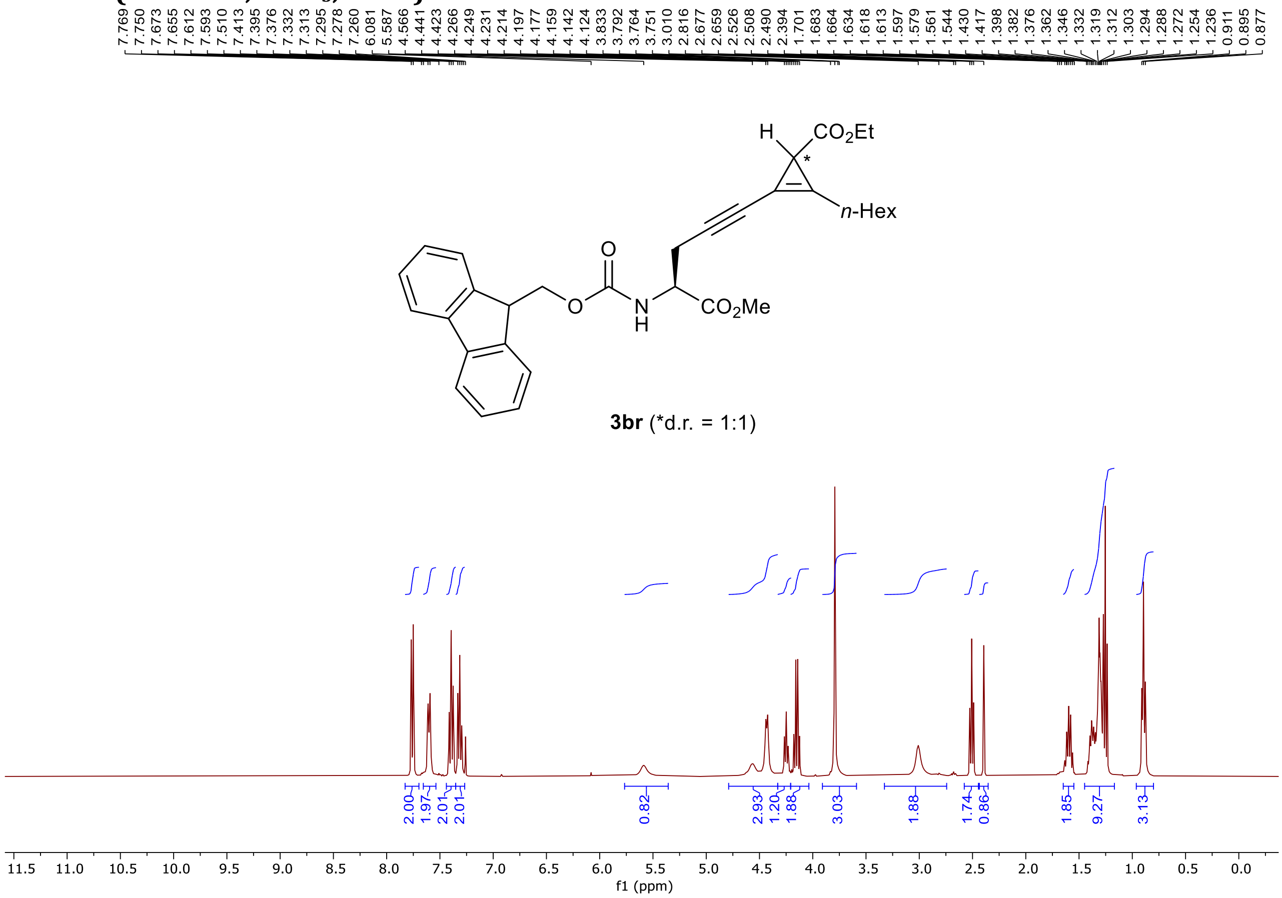

^{13}C NMR (101 MHz, $CDCl_3$, 60 °C) of 3br

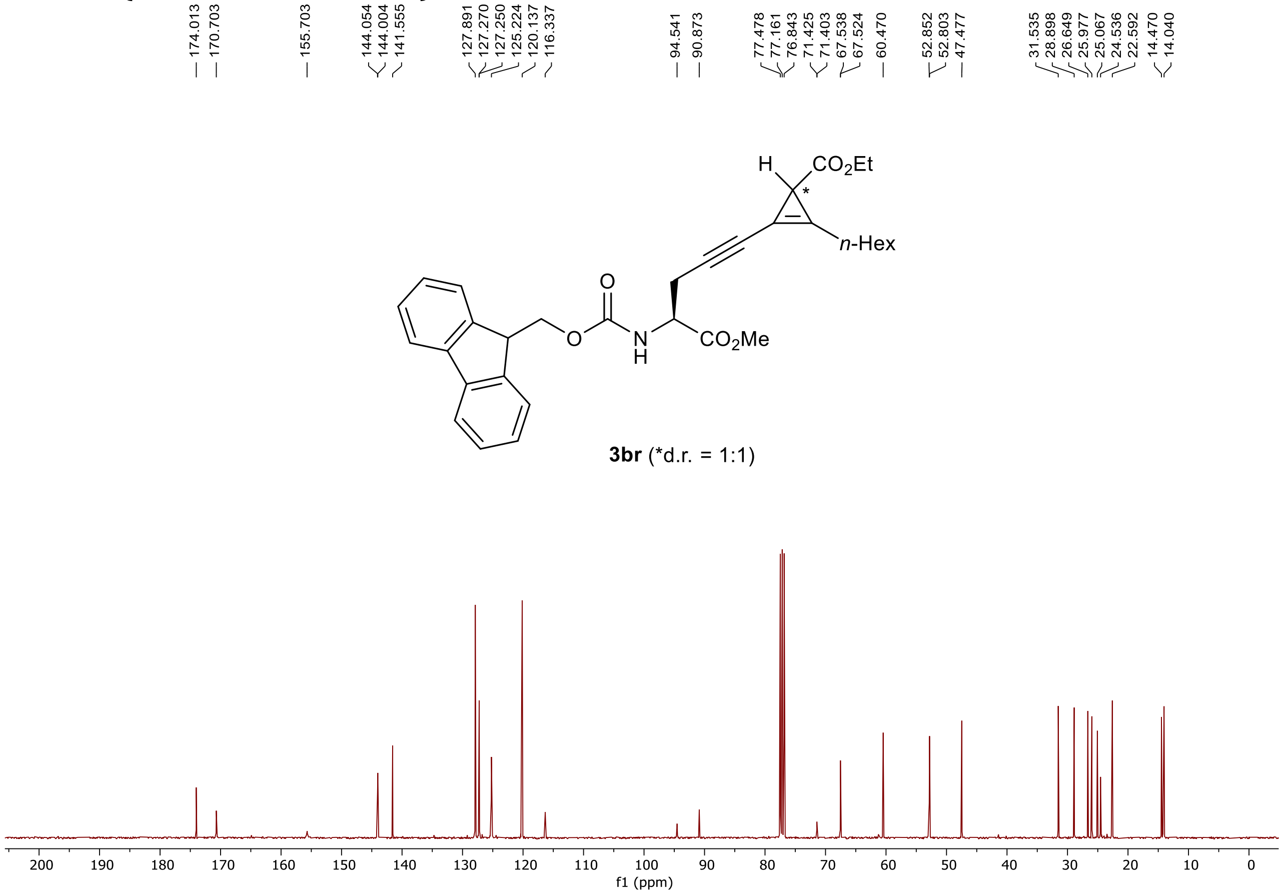

^{1}H NMR (400 MHz, $CDCl_3$, 60 °C) of 3bs

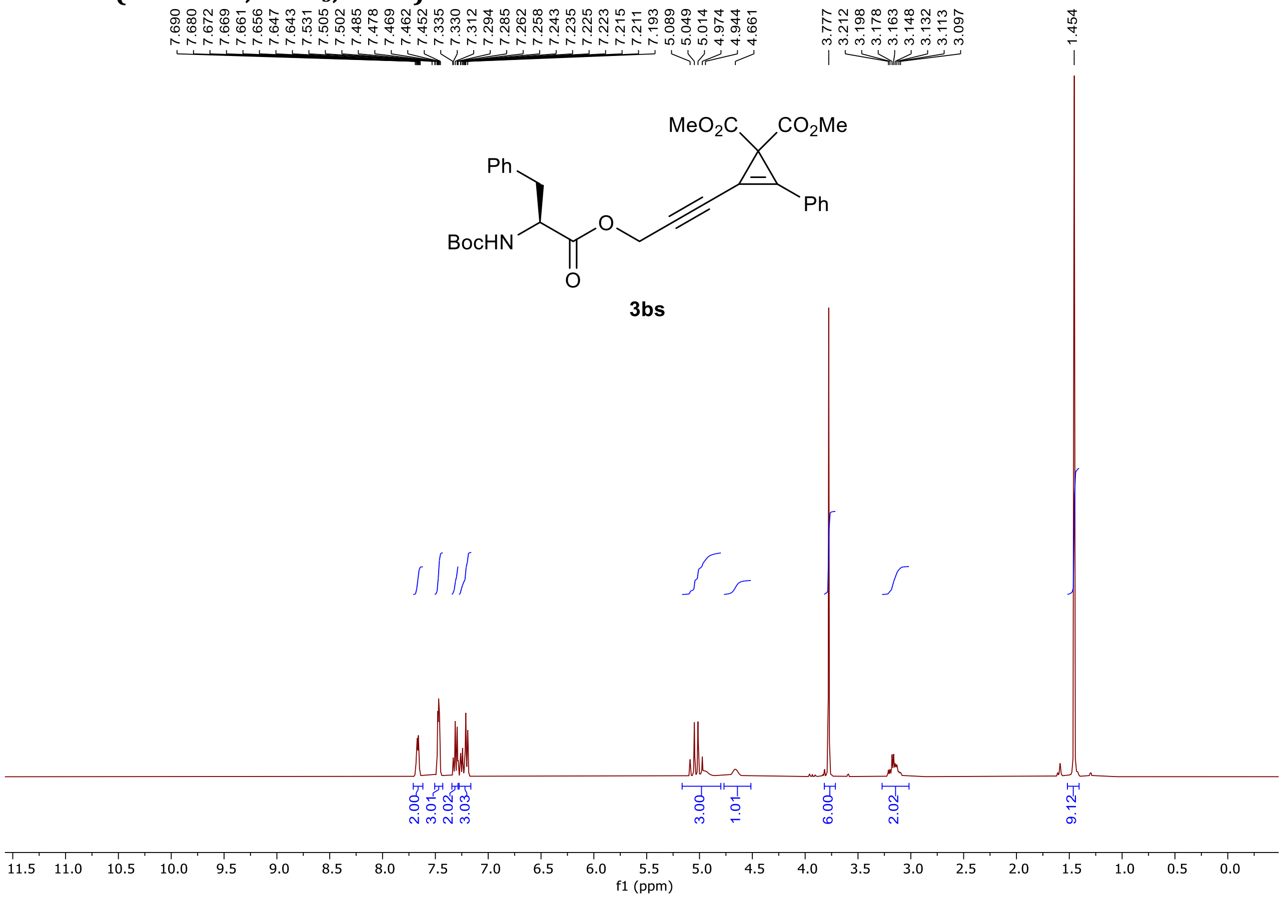

^{13}C NMR (101 MHz, $CDCl_3$, 60 °C) of 3bs

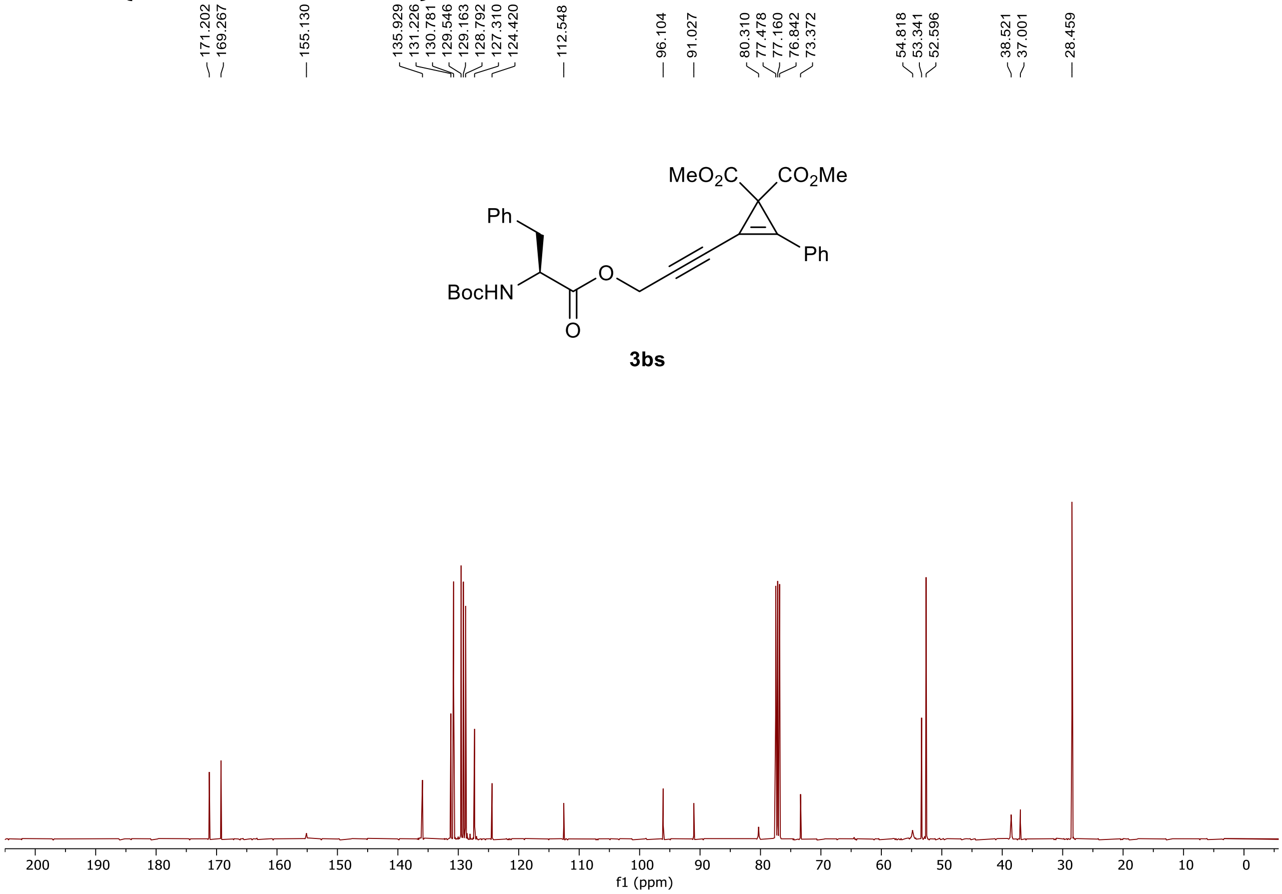

^{1}H NMR (400 MHz, $CDCl_3$) of 3bt

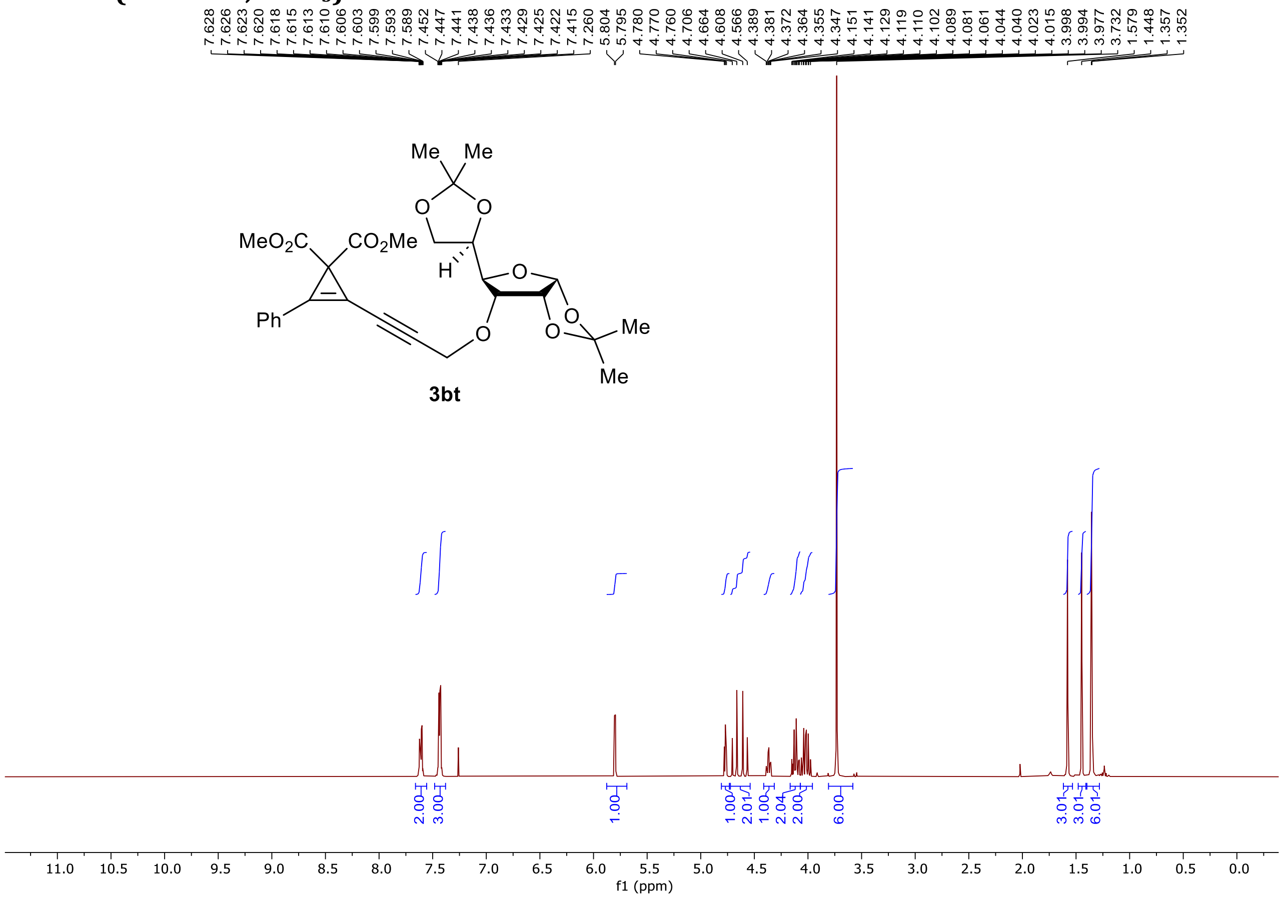

^{13}C NMR (101 MHz, $CDCl_3$) of 3bt

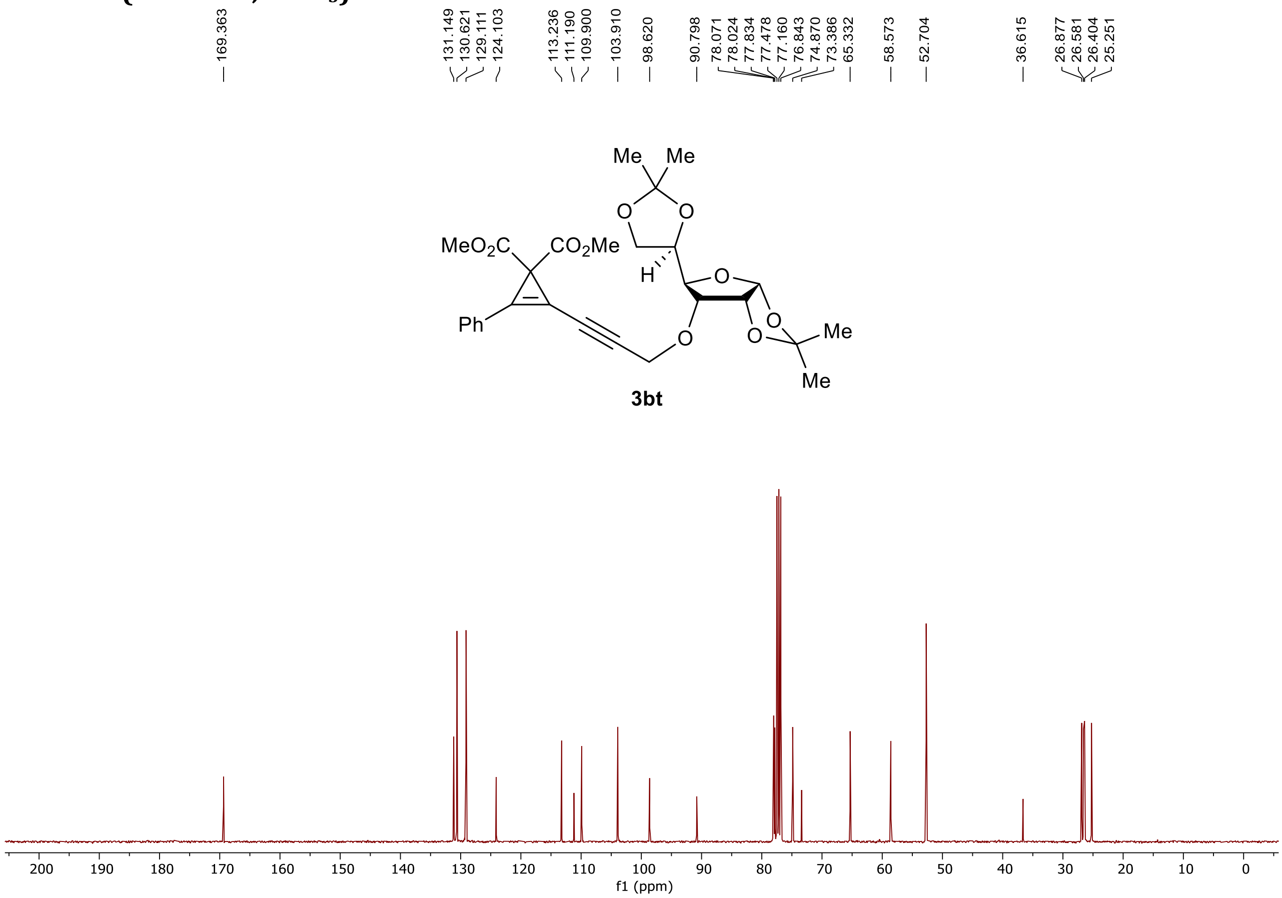

^{1}H NMR (400 MHz, $CDCl_3$) of 3bu

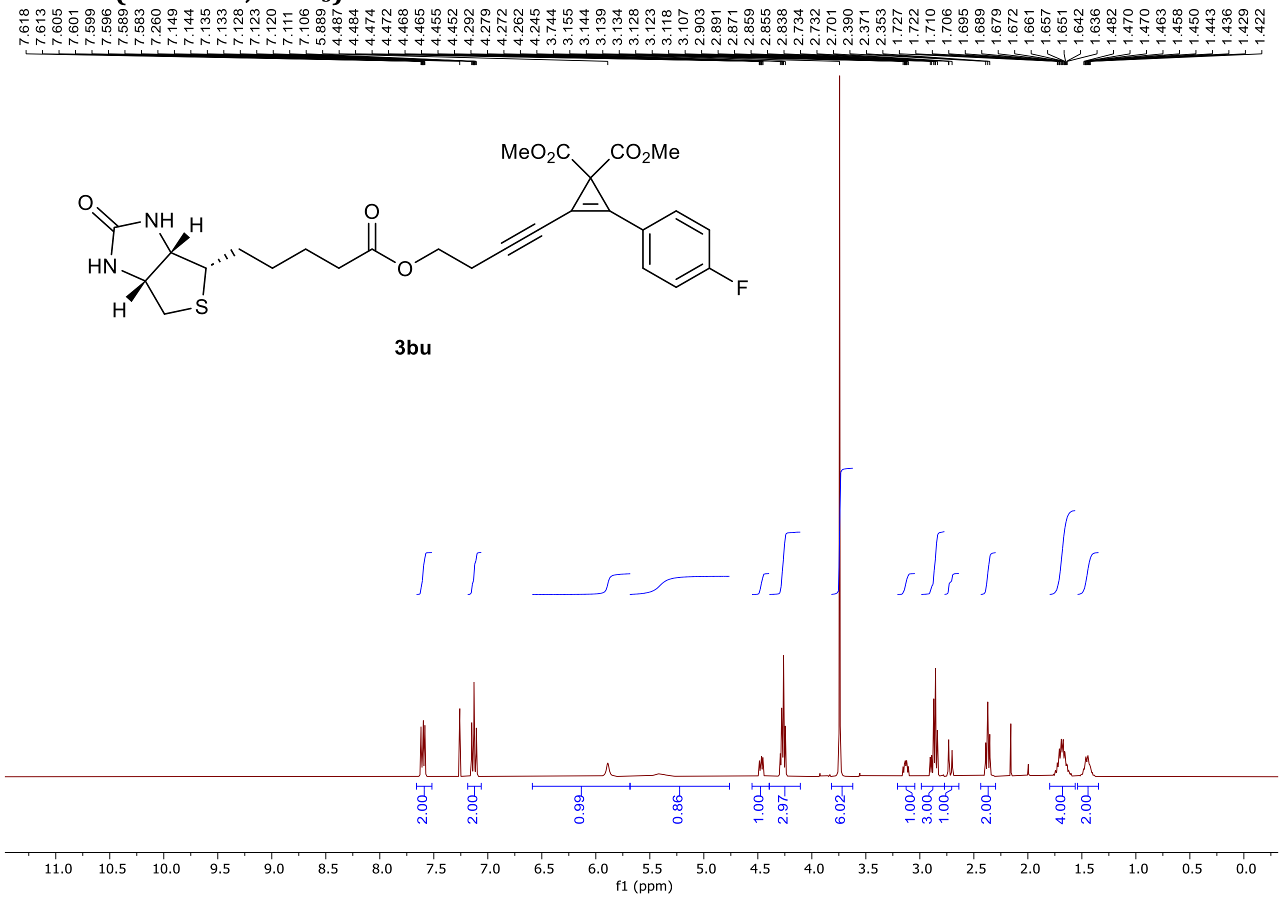

^{13}C NMR (101 MHz, $CDCl_3$) of 3bu

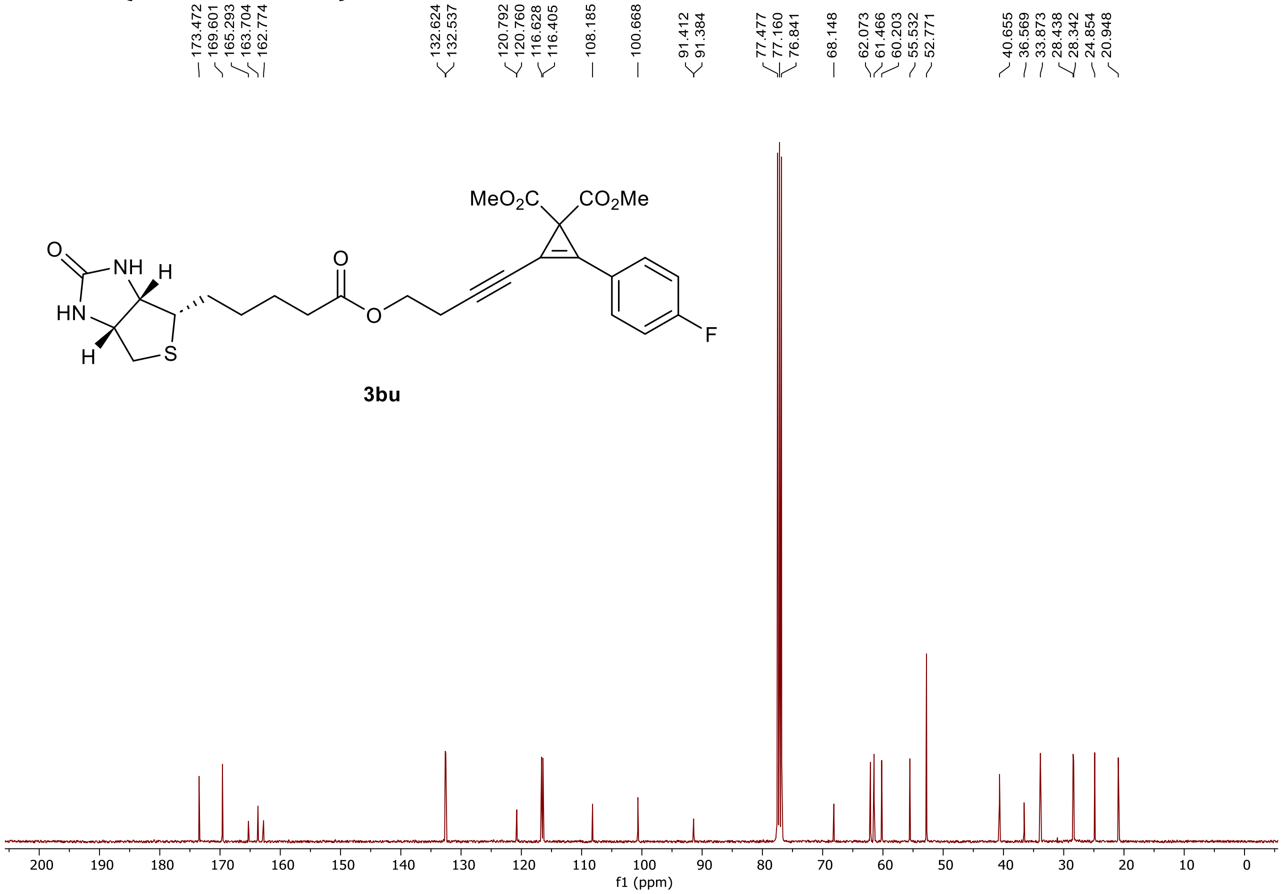

^{19}F NMR (377 MHz, $CDCl_3$) of 3bu

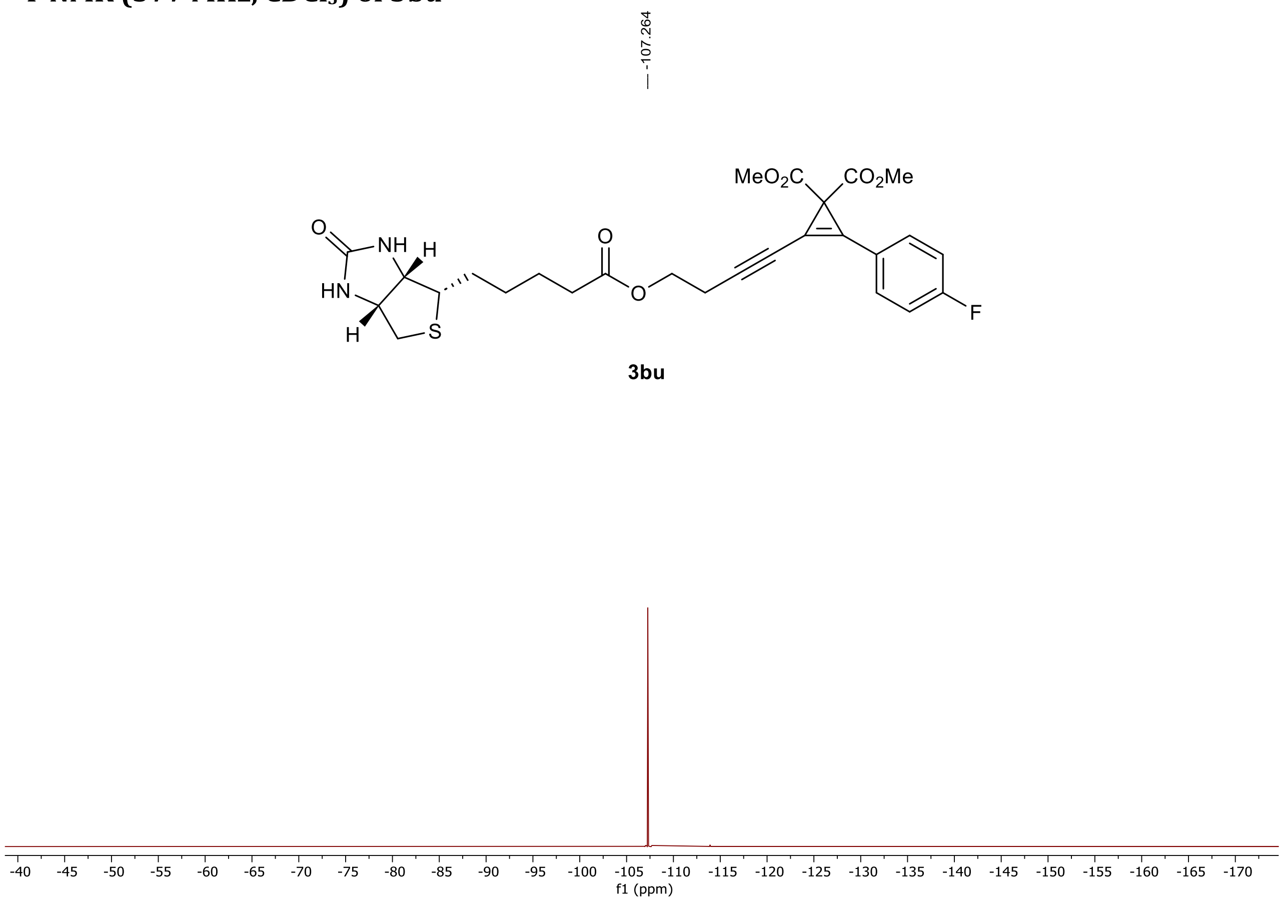

^{1}H NMR (400 MHz, $CDCl_3$) of 3bv

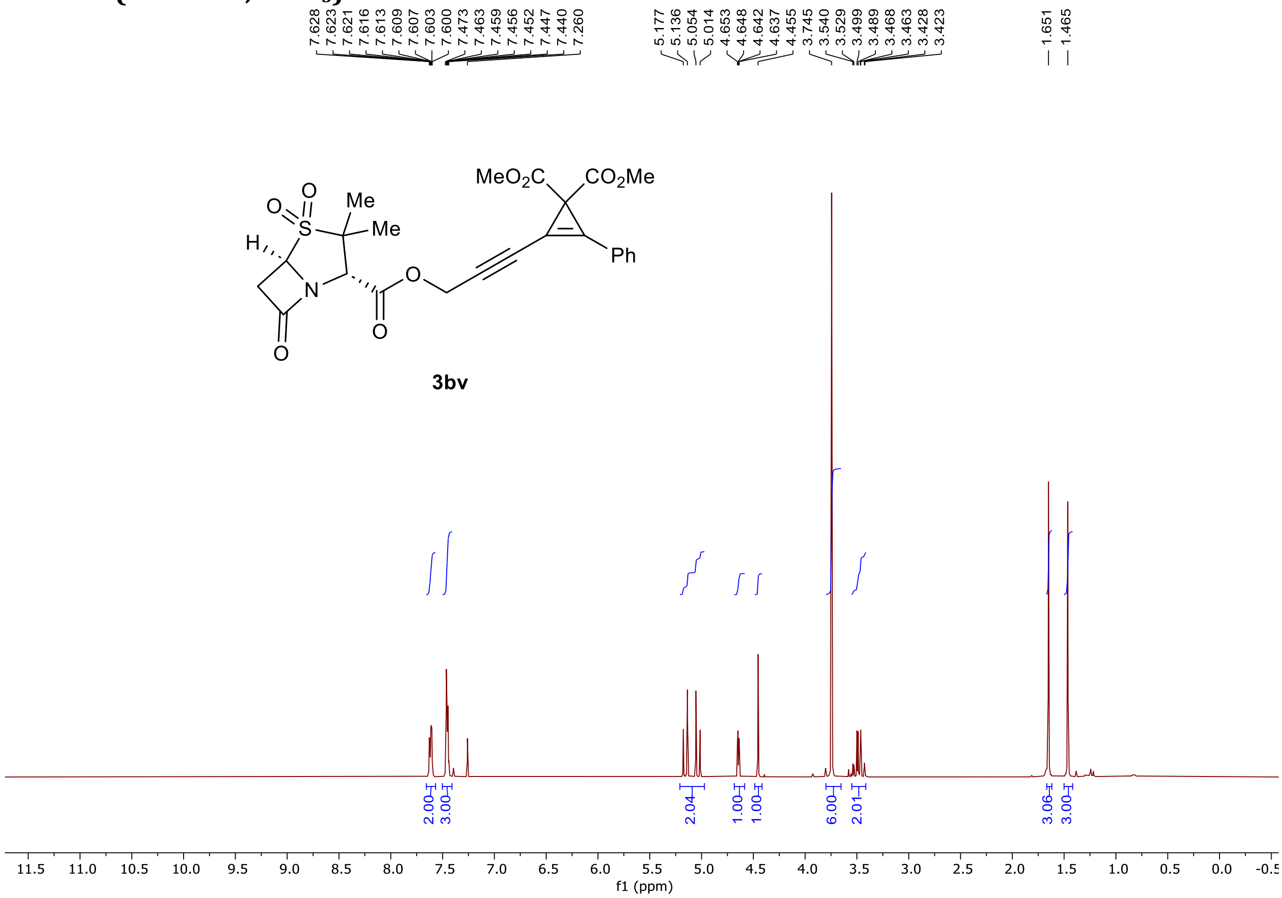

^{13}C NMR (101 MHz, $CDCl_3$) of 3bv

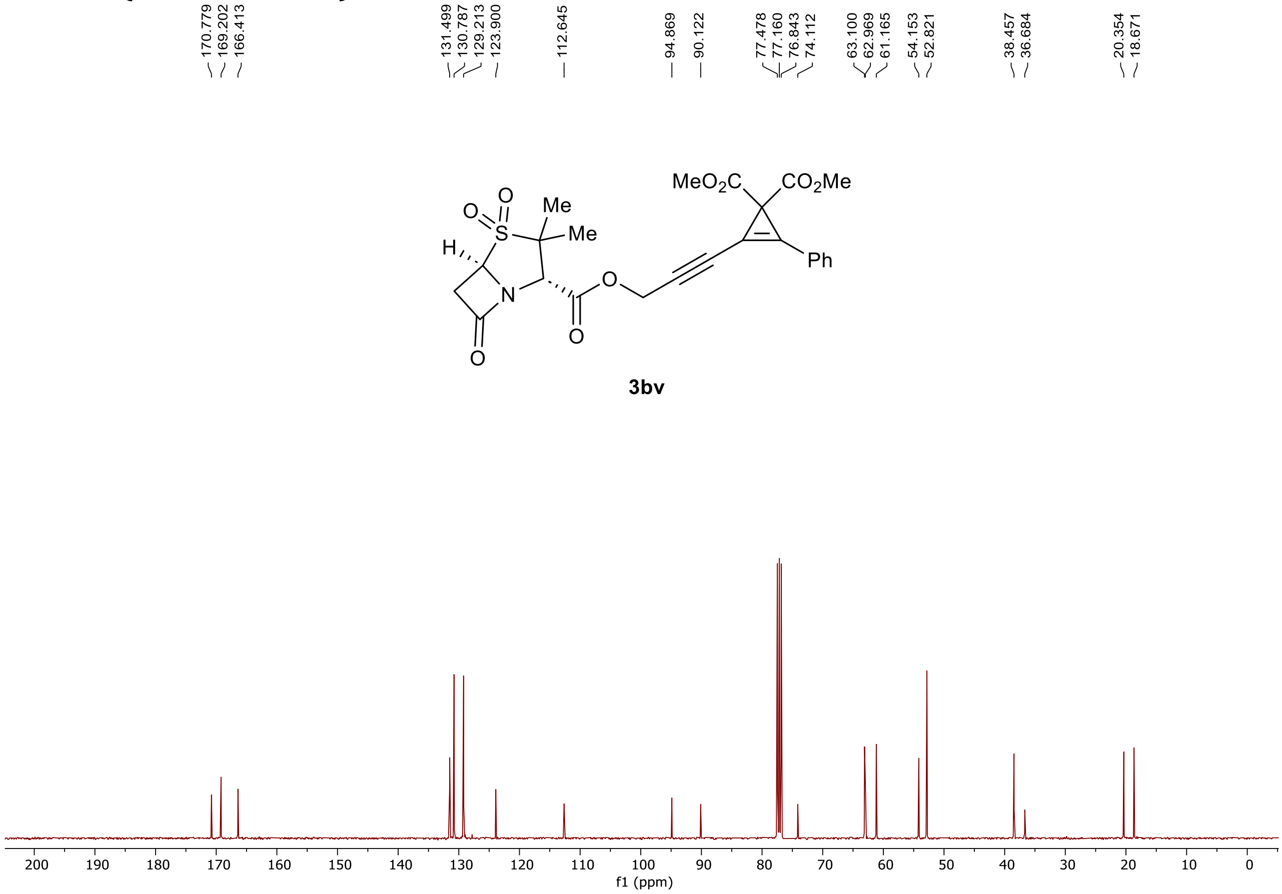

^{1}H NMR (400 MHz, $CDCl_3$) of 3bw

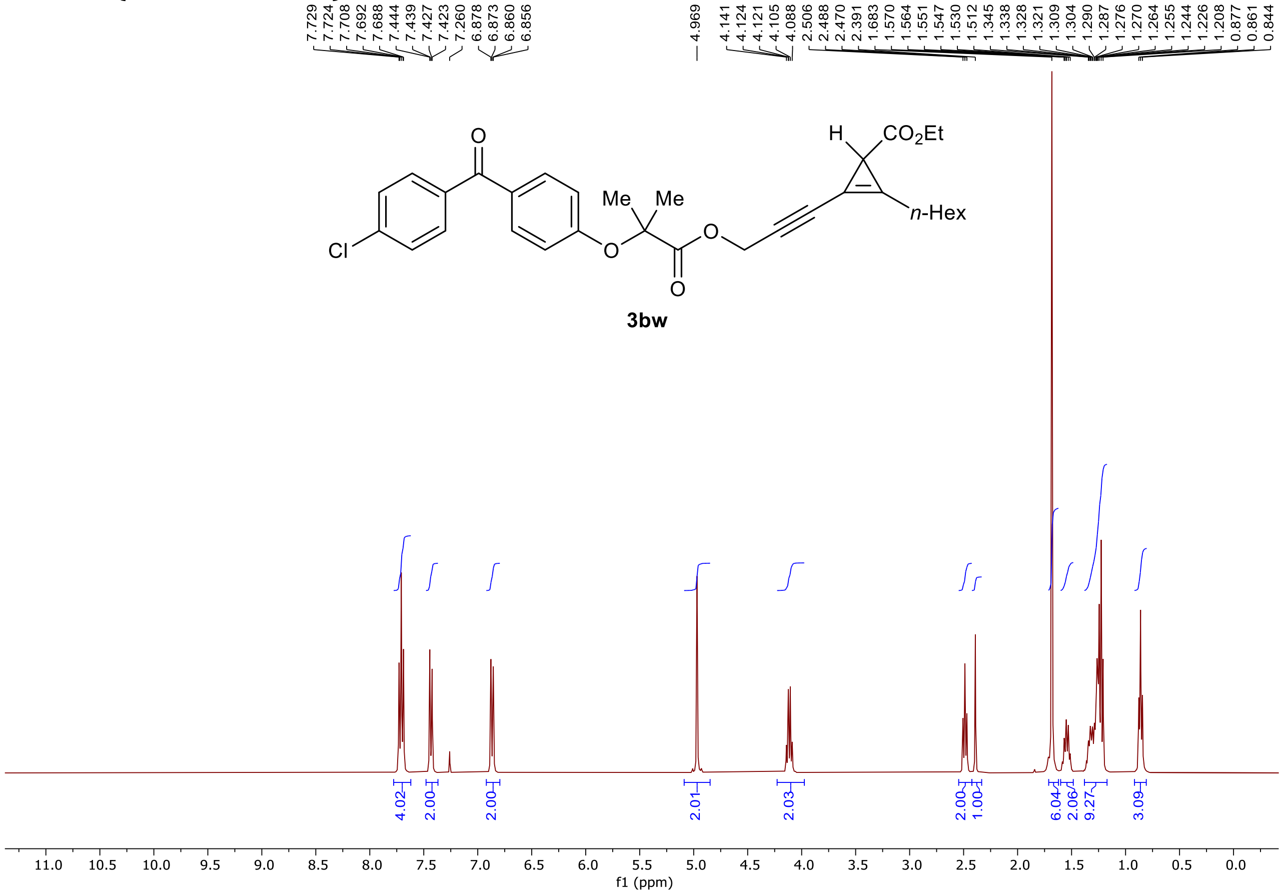

^{13}C NMR (101 MHz, $CDCl_3$) of 3bw

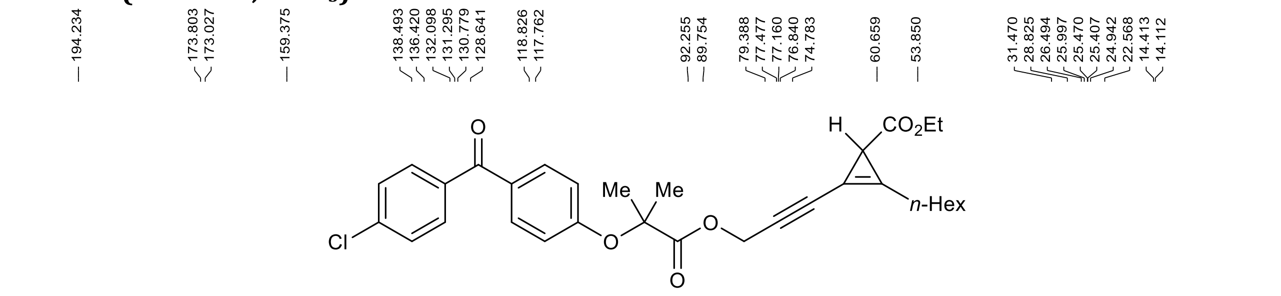

3bw

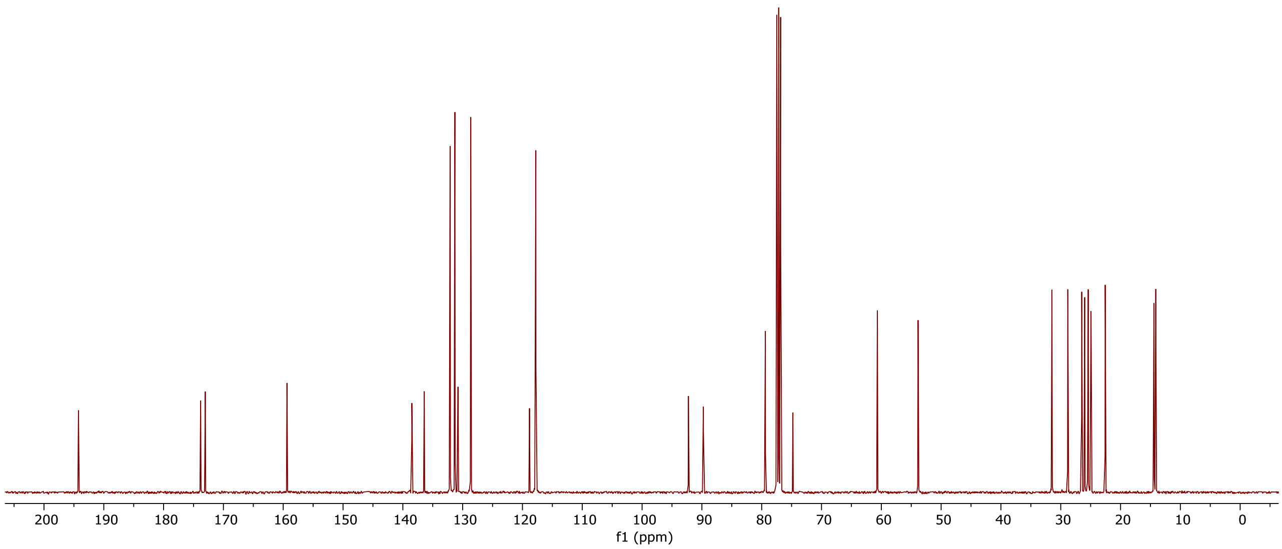

^{1}H NMR (400 MHz, $CDCl_3$) of 3bx

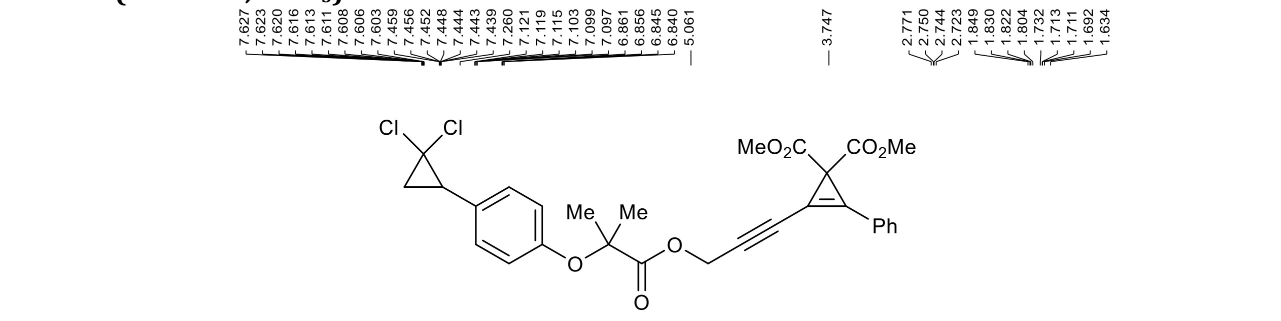

3bx

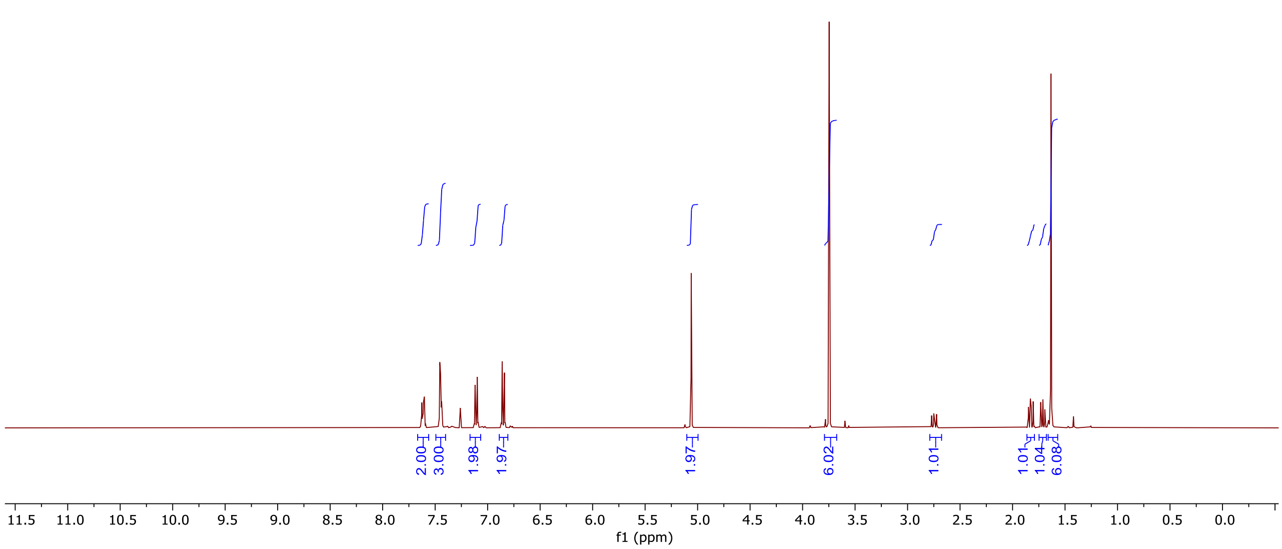

^{13}C NMR (101 MHz, $CDCl_3$) of 3bx

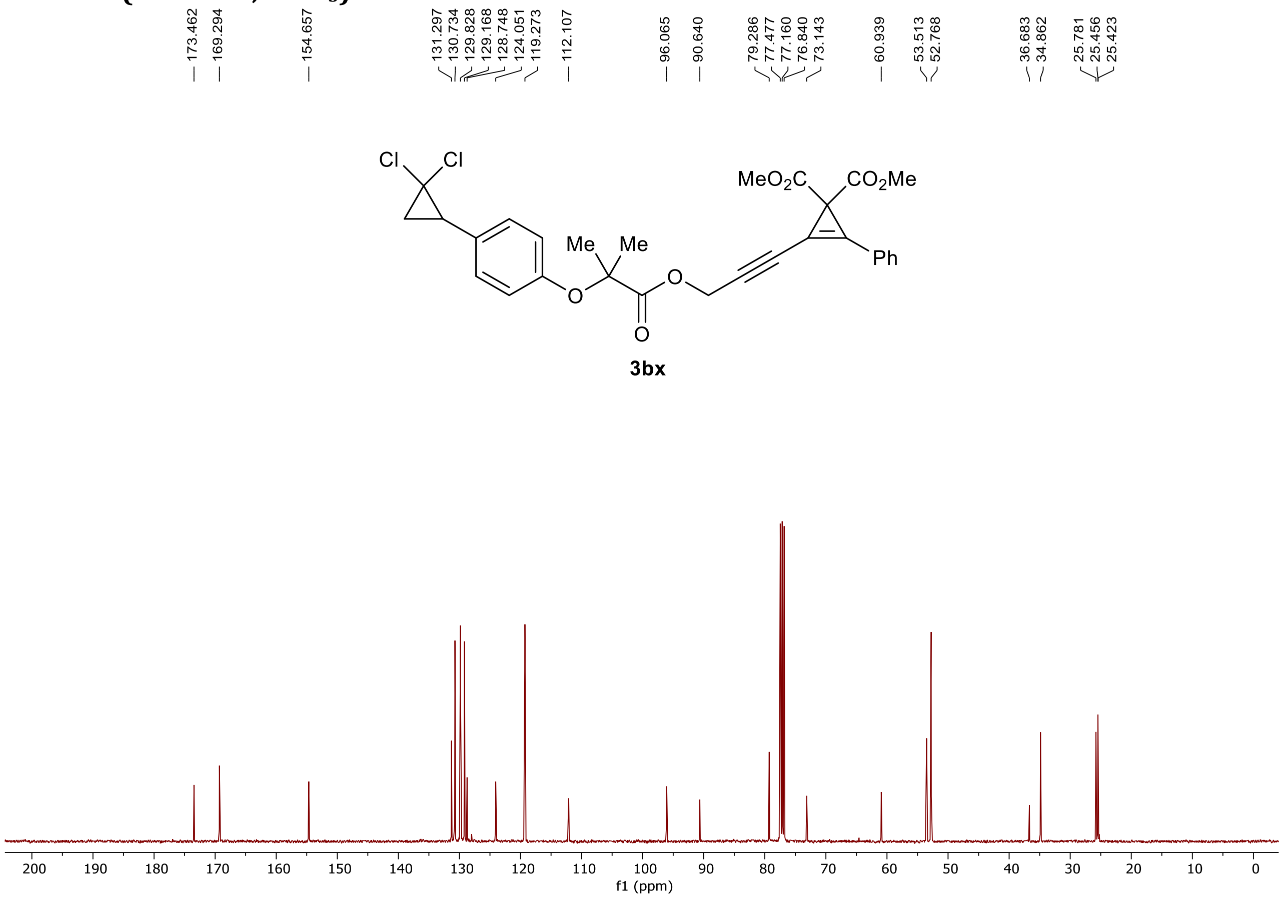

^{1}H NMR (400 MHz, $CDCl_3$) of 3by

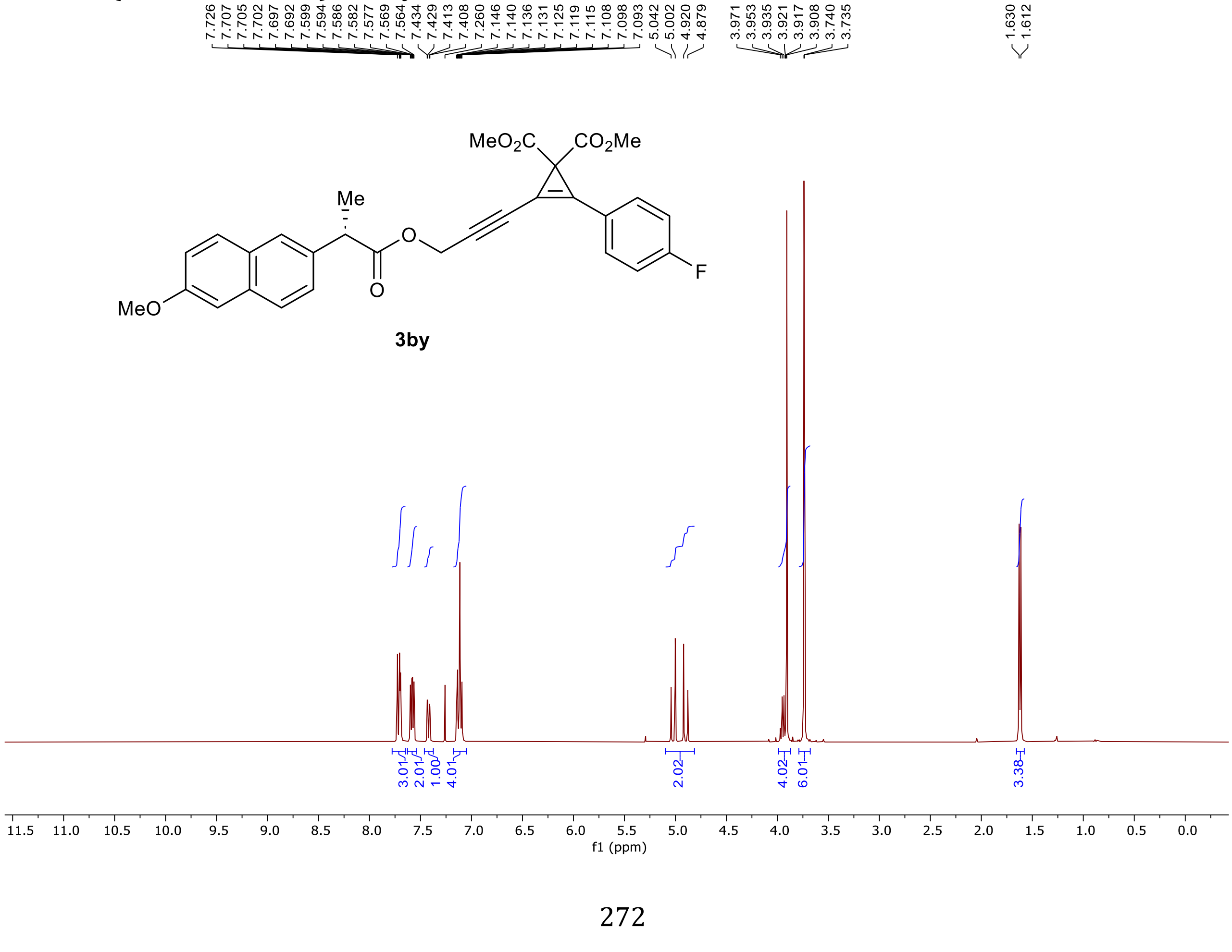

^{13}C NMR (101 MHz, $CDCl_3$) of 3by

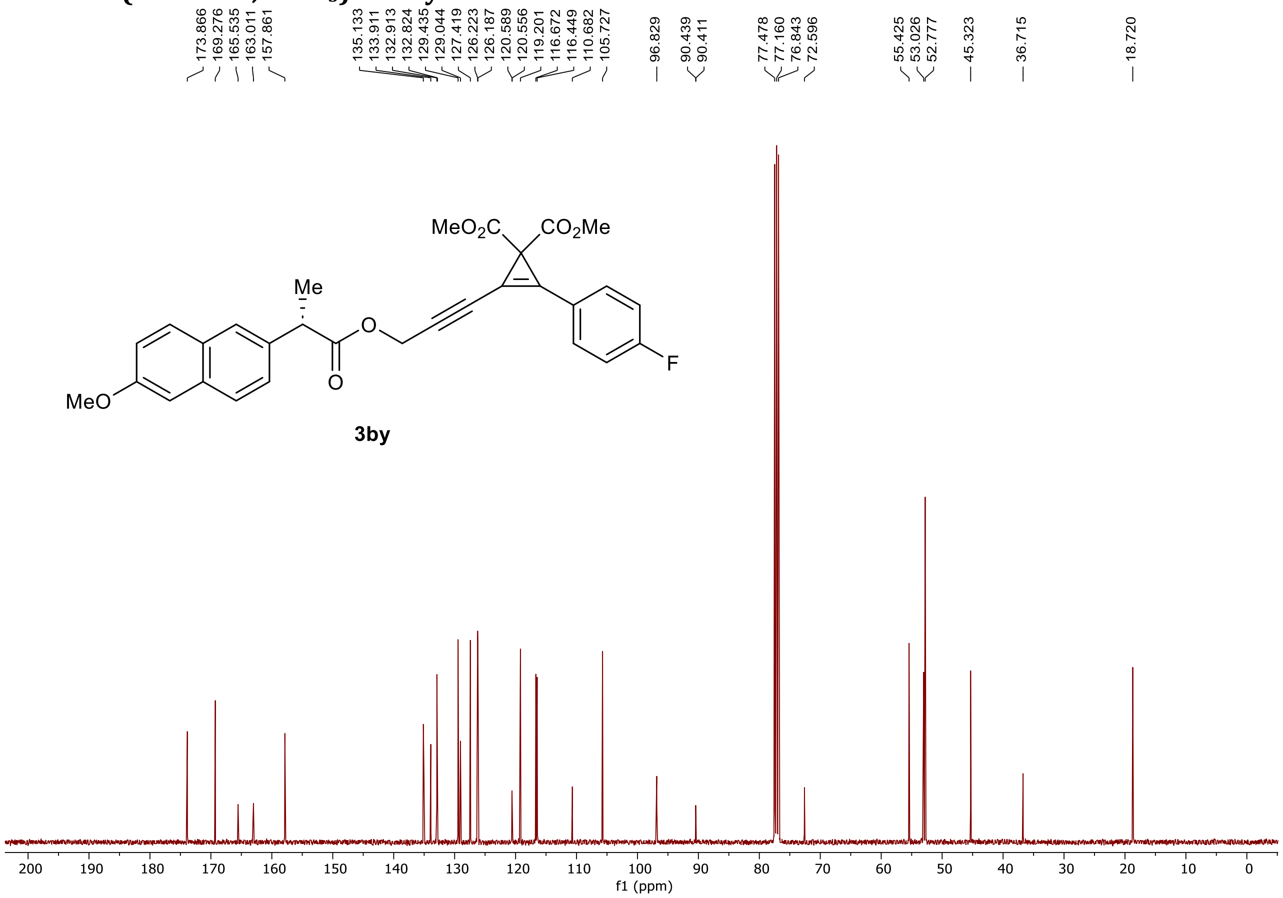

^{19}F NMR (377 MHz, $CDCl_3$) of 3by

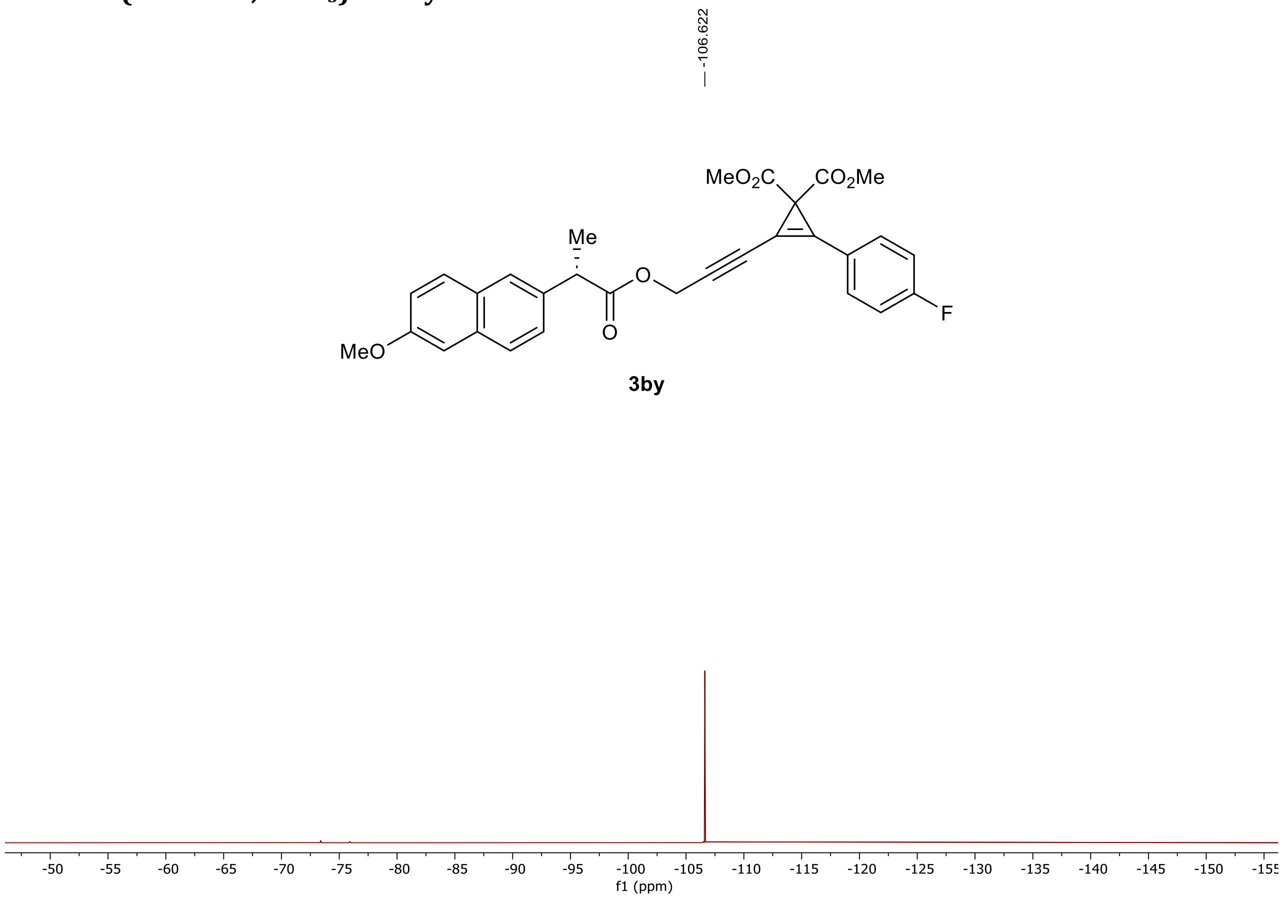

^{1}H NMR (400 MHz, $CDCl_3$) of 3bz

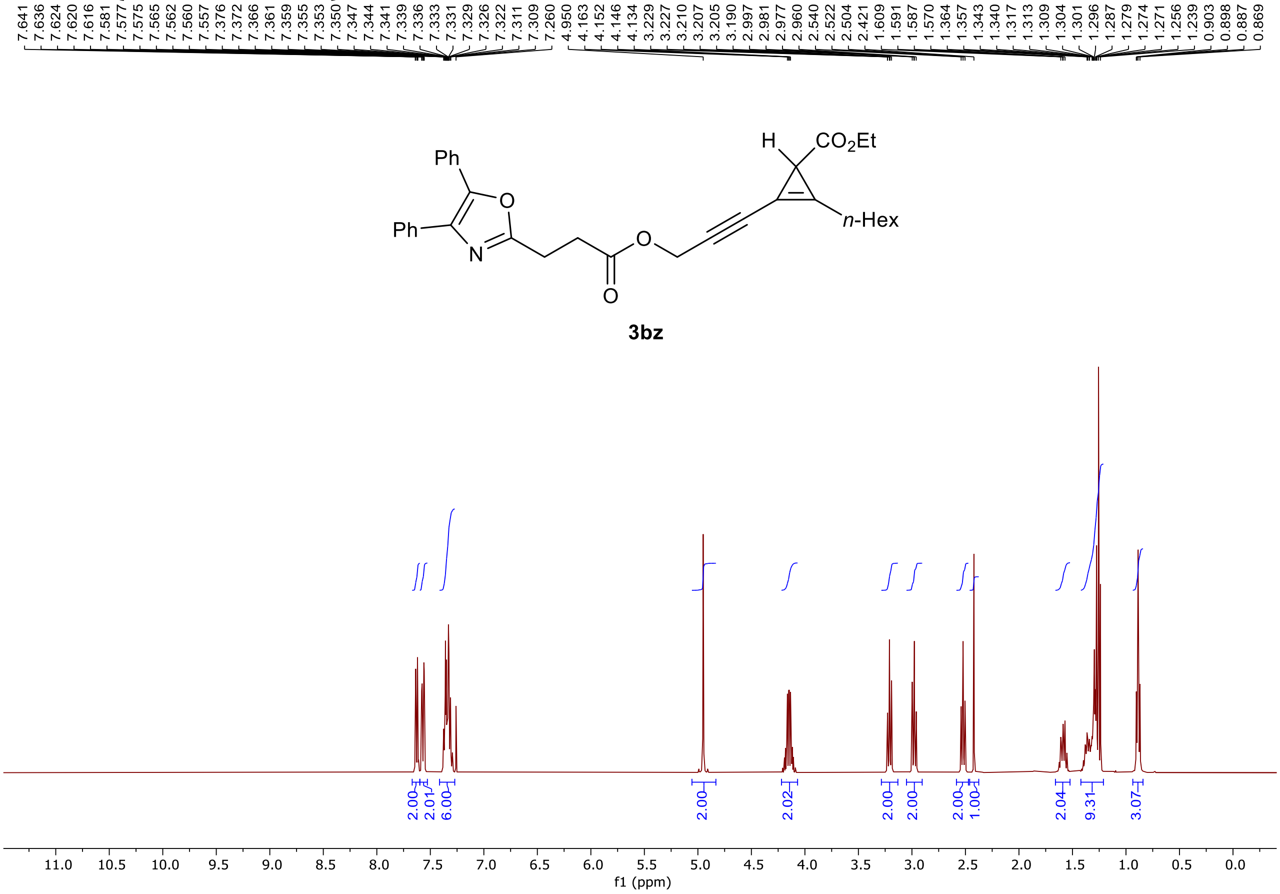

^{13}C NMR (101 MHz, $CDCl_3$) of 3bz

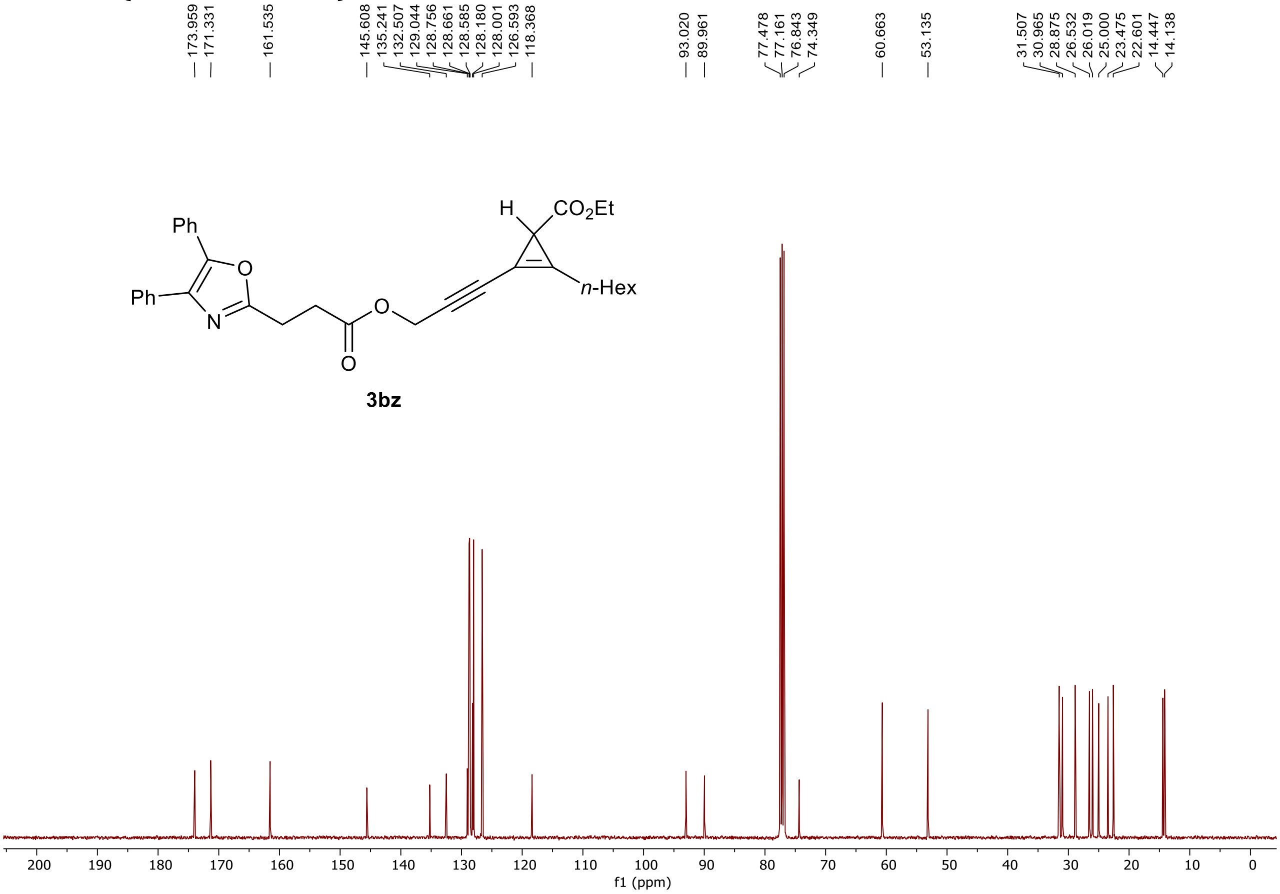

^{1}H NMR (400 MHz, $CDCl_3$) of 3ca

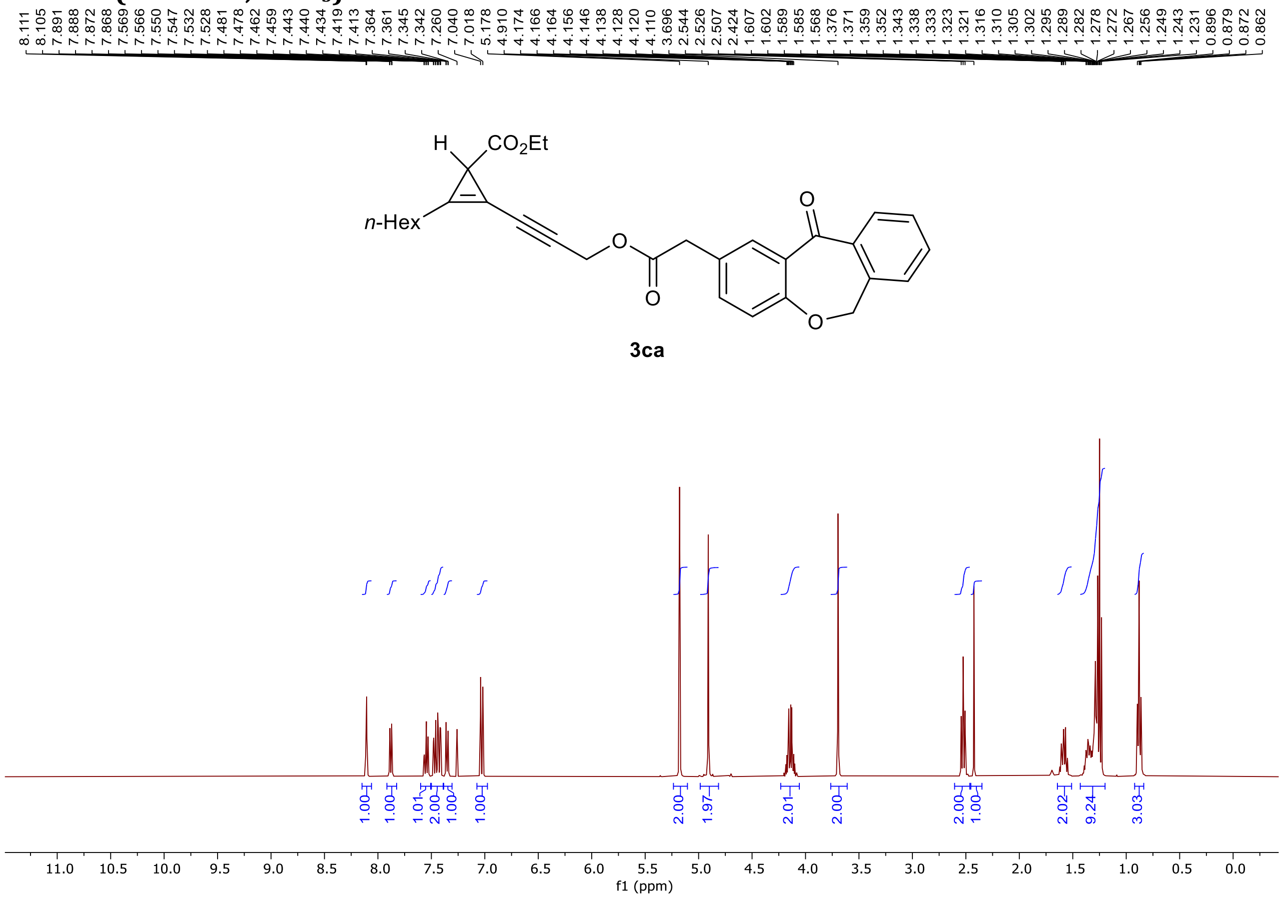

^{13}C NMR (101 MHz, $CDCl_3$) of 3ca

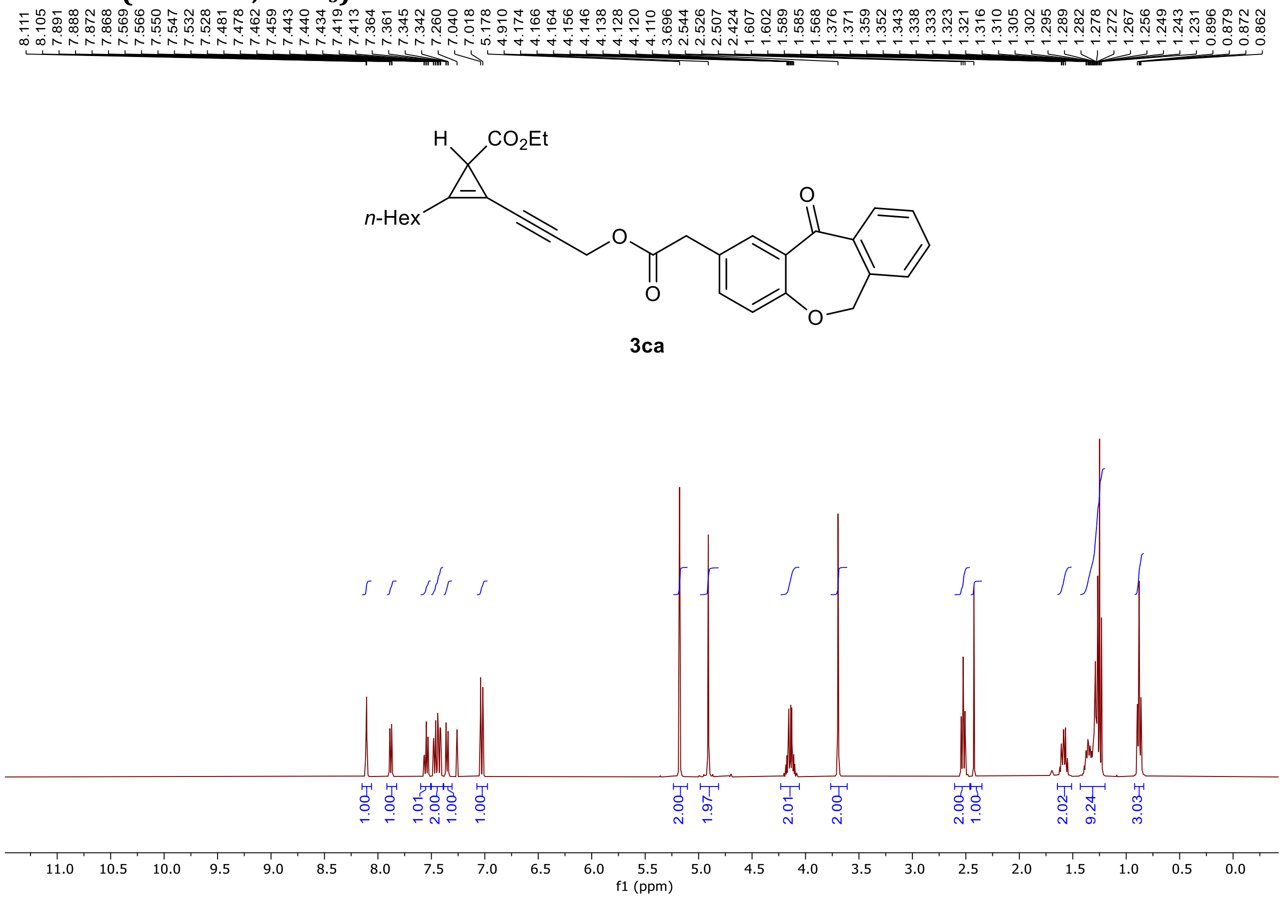

^{1}H NMR (400 MHz, $CDCl_3$) of 3cb

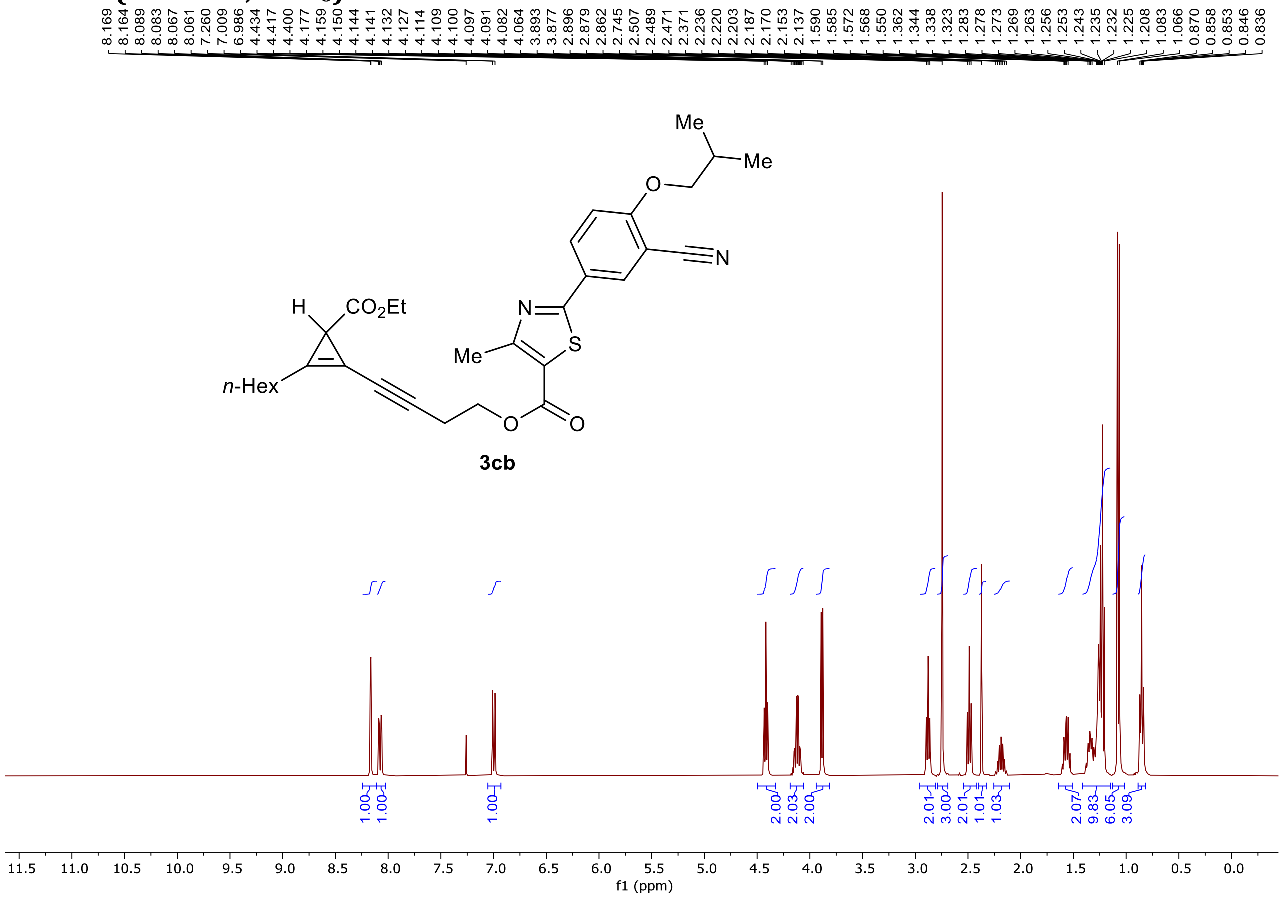

^{13}C NMR (101 MHz, $CDCl_3$) of 3cb

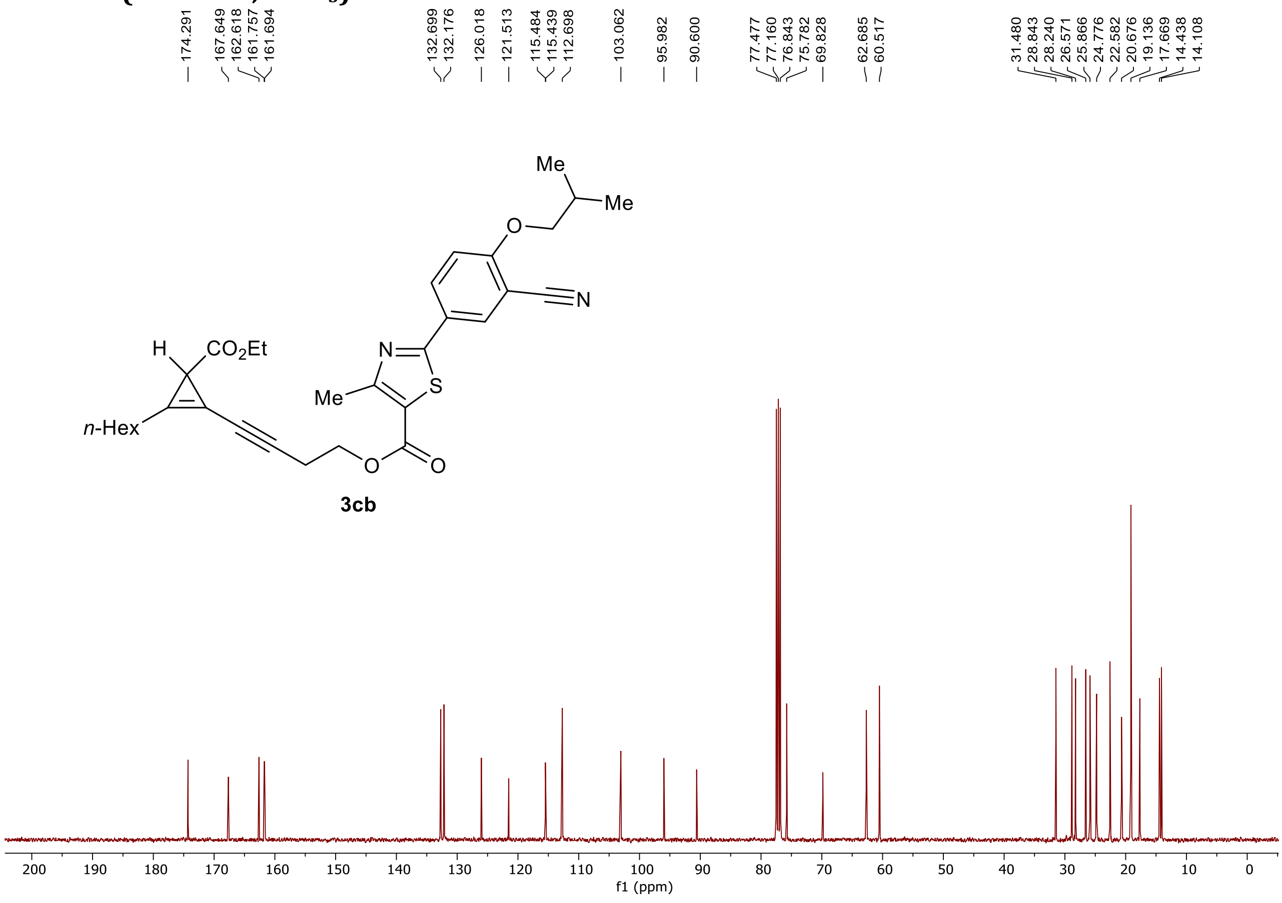

^{1}H NMR (400 MHz, $CDCl_3$) of 3cc

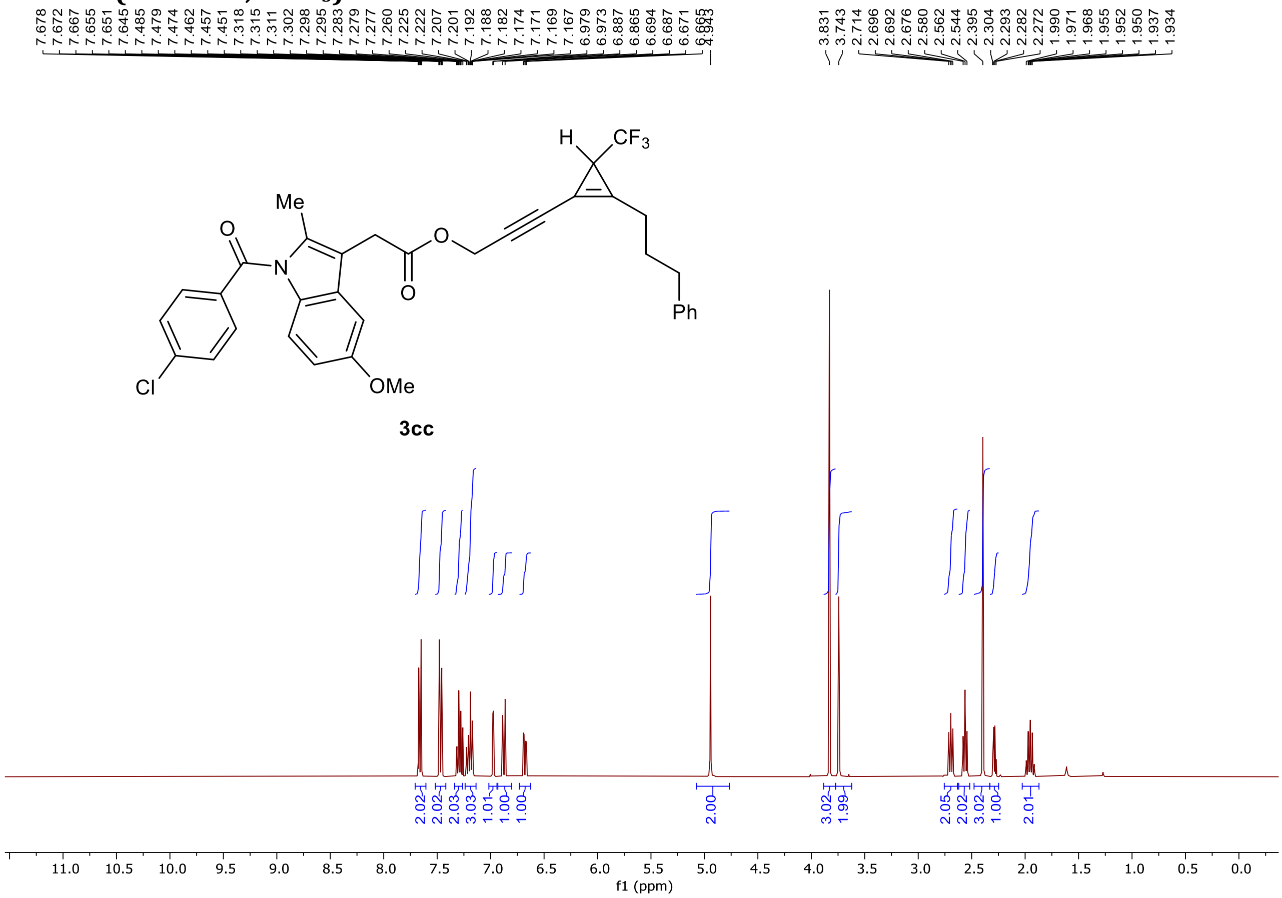

^{13}C NMR (101 MHz, $CDCl_3$) of 3cc

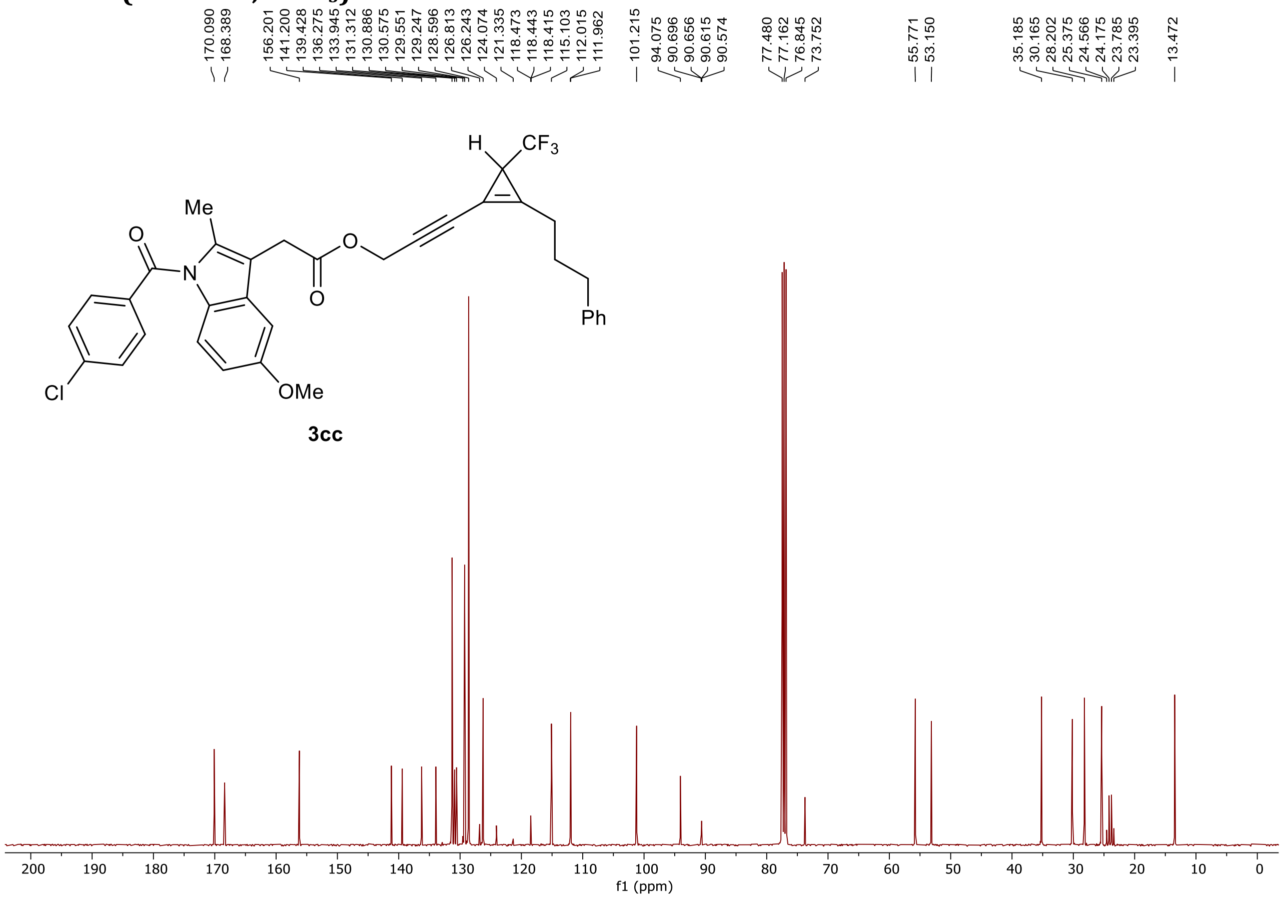

^{19}F NMR (377 MHz, $CDCl_3$) of 3cc

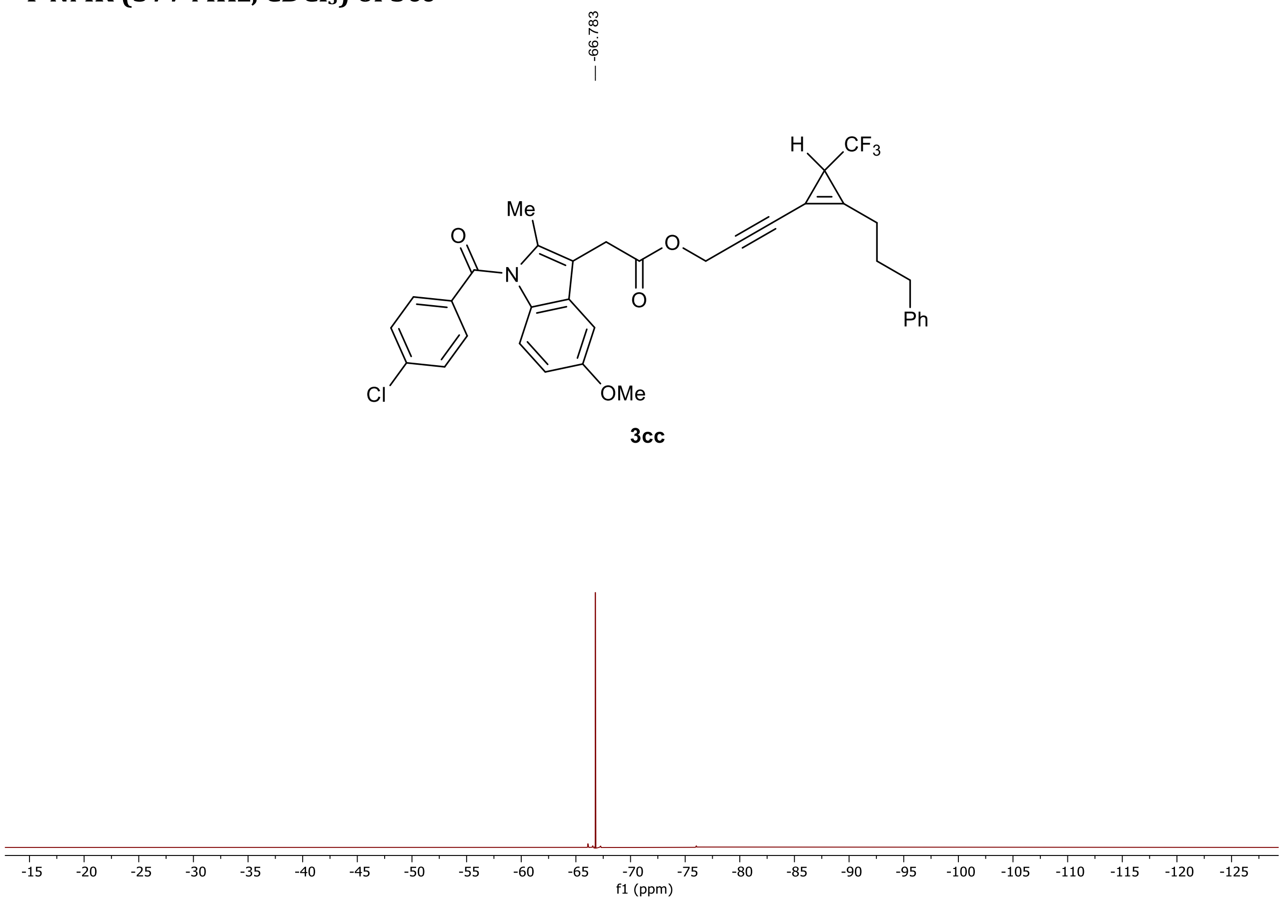

^{1}H NMR (400 MHz, $CDCl_3$) of 3cd

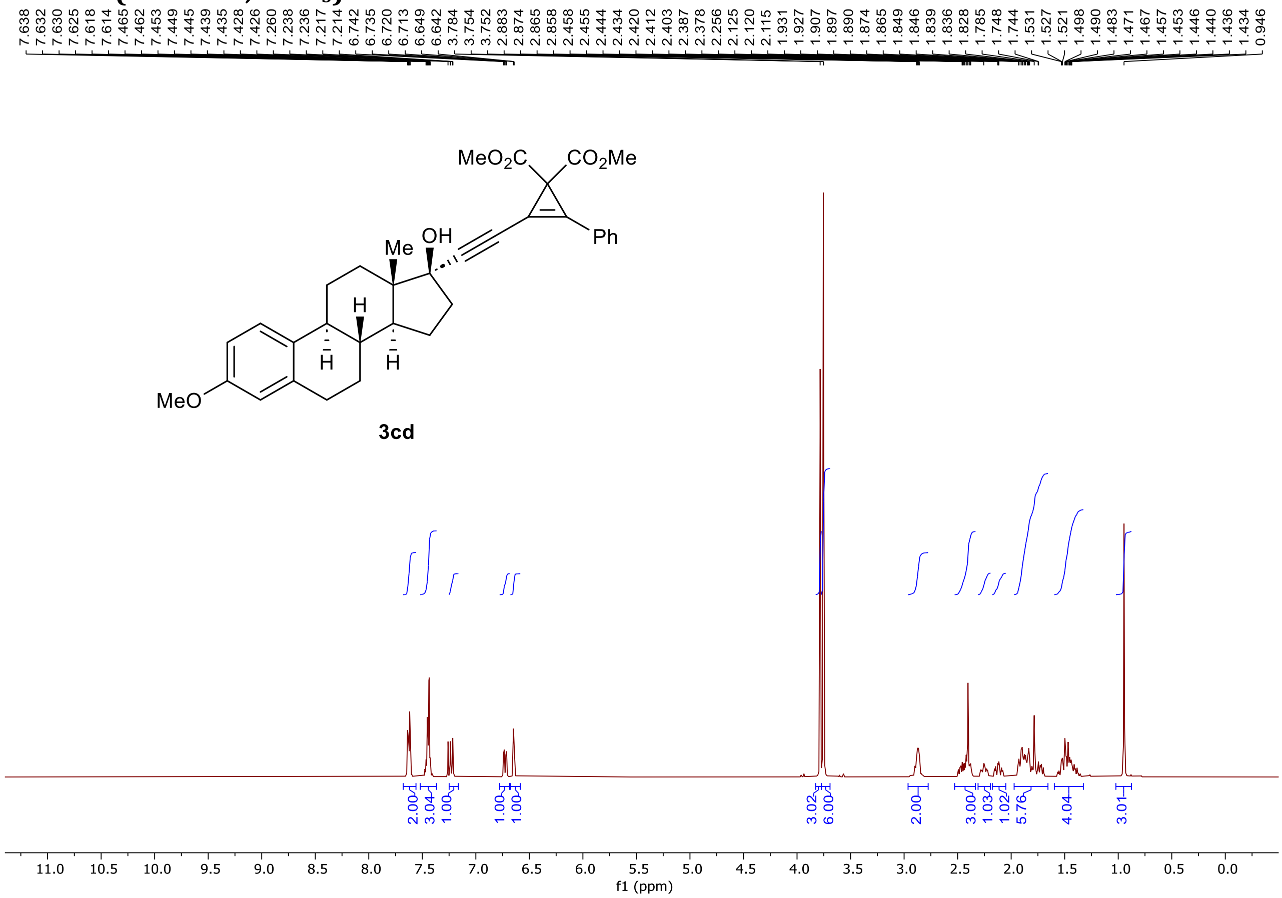

^{13}C NMR (101 MHz, $CDCl_3$) of 3cd

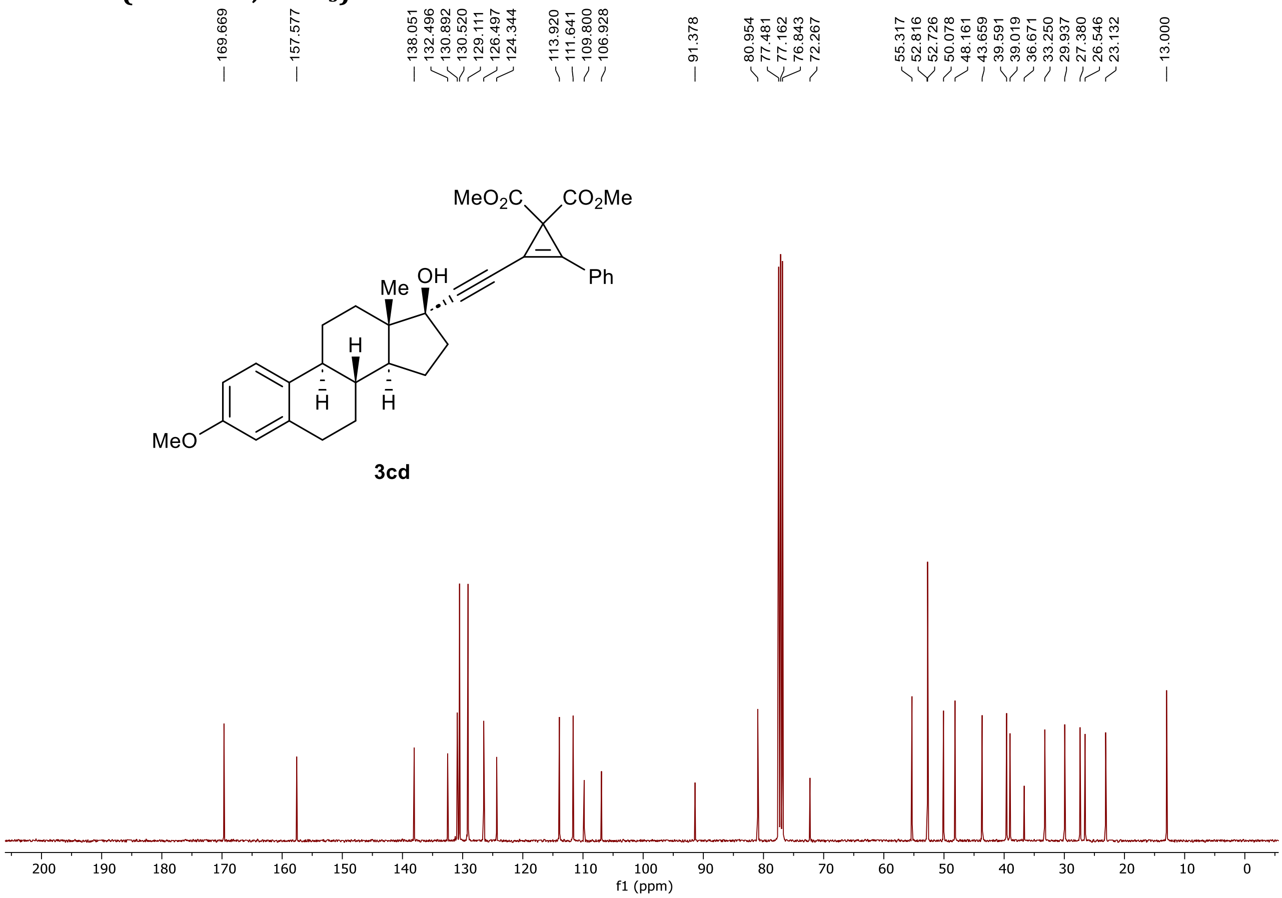

^{1}H NMR (400 MHz, $CDCl_3$) of 3ce

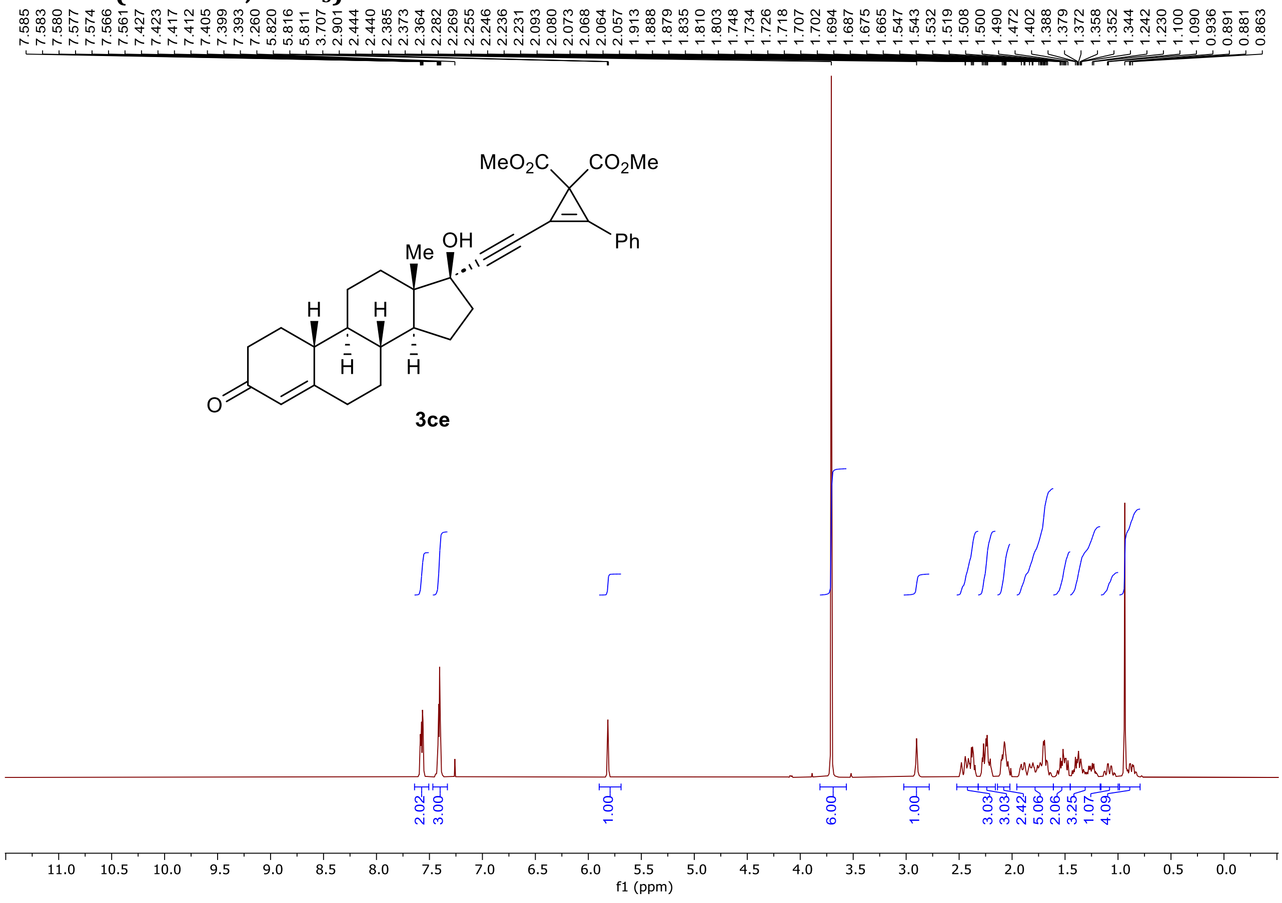

^{13}C NMR (101 MHz, $CDCl_3$) of 3ce

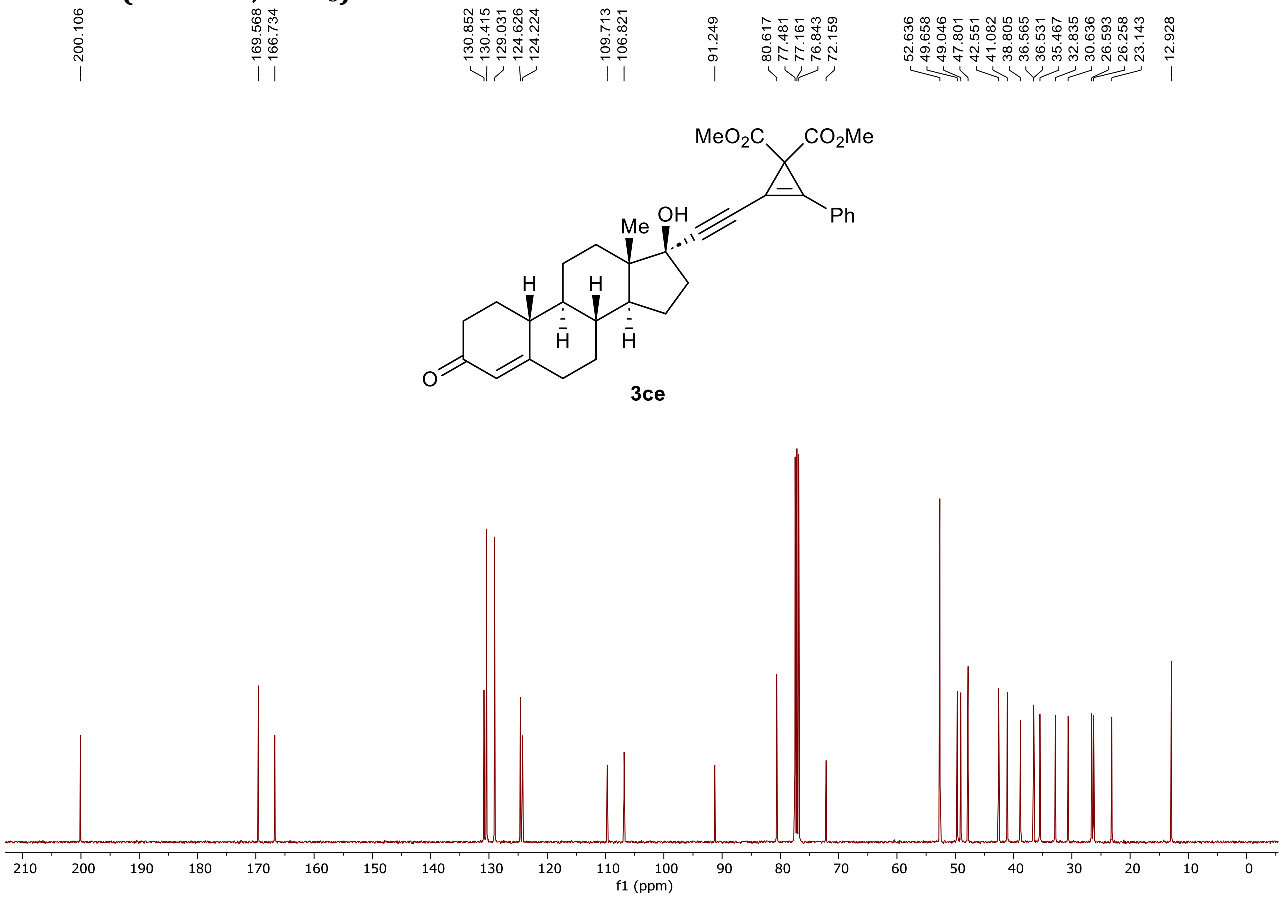

^{1}H NMR (400 MHz, $CDCl_3$) of 3cf

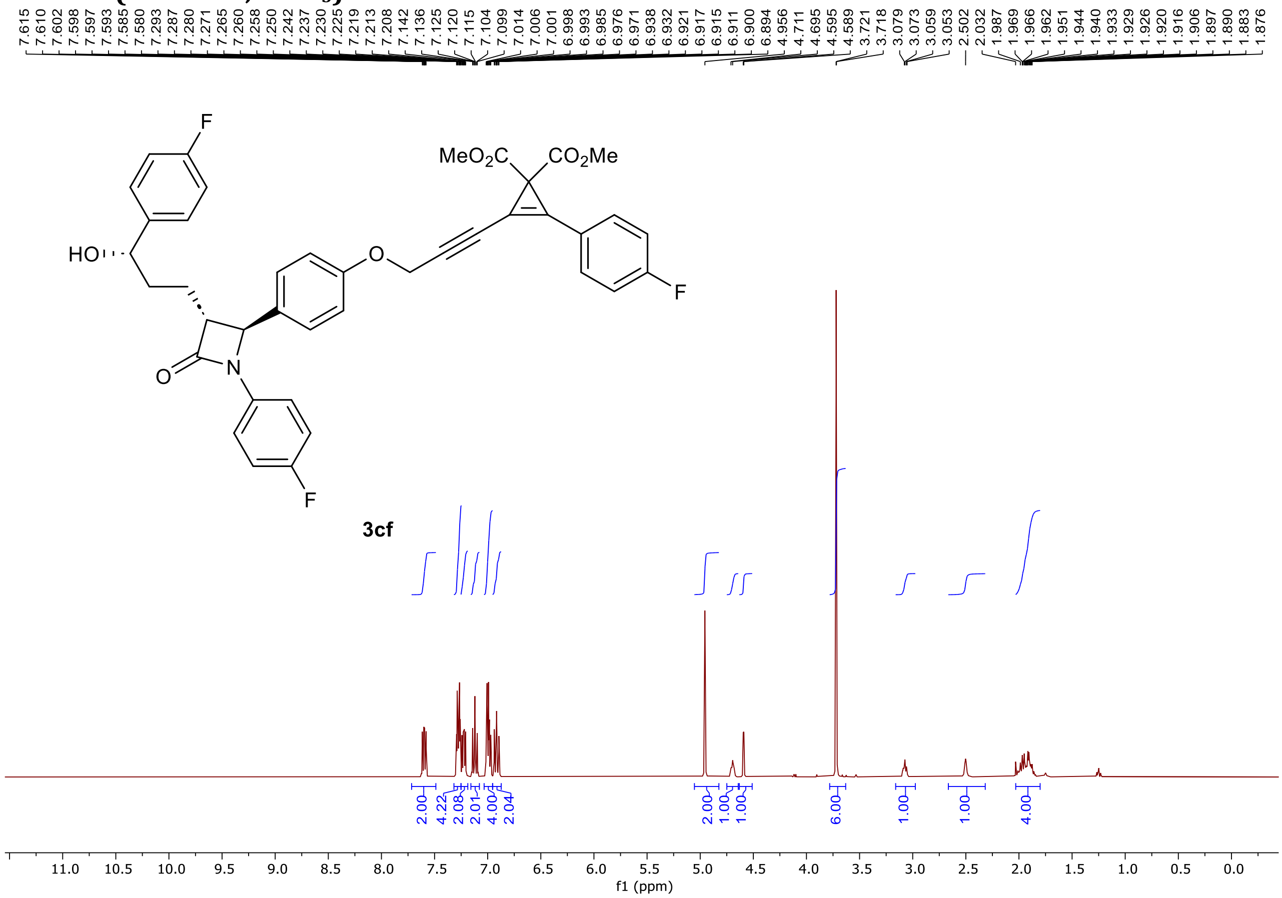

^{13}C NMR (101 MHz, $CDCl_3$) of 3cf

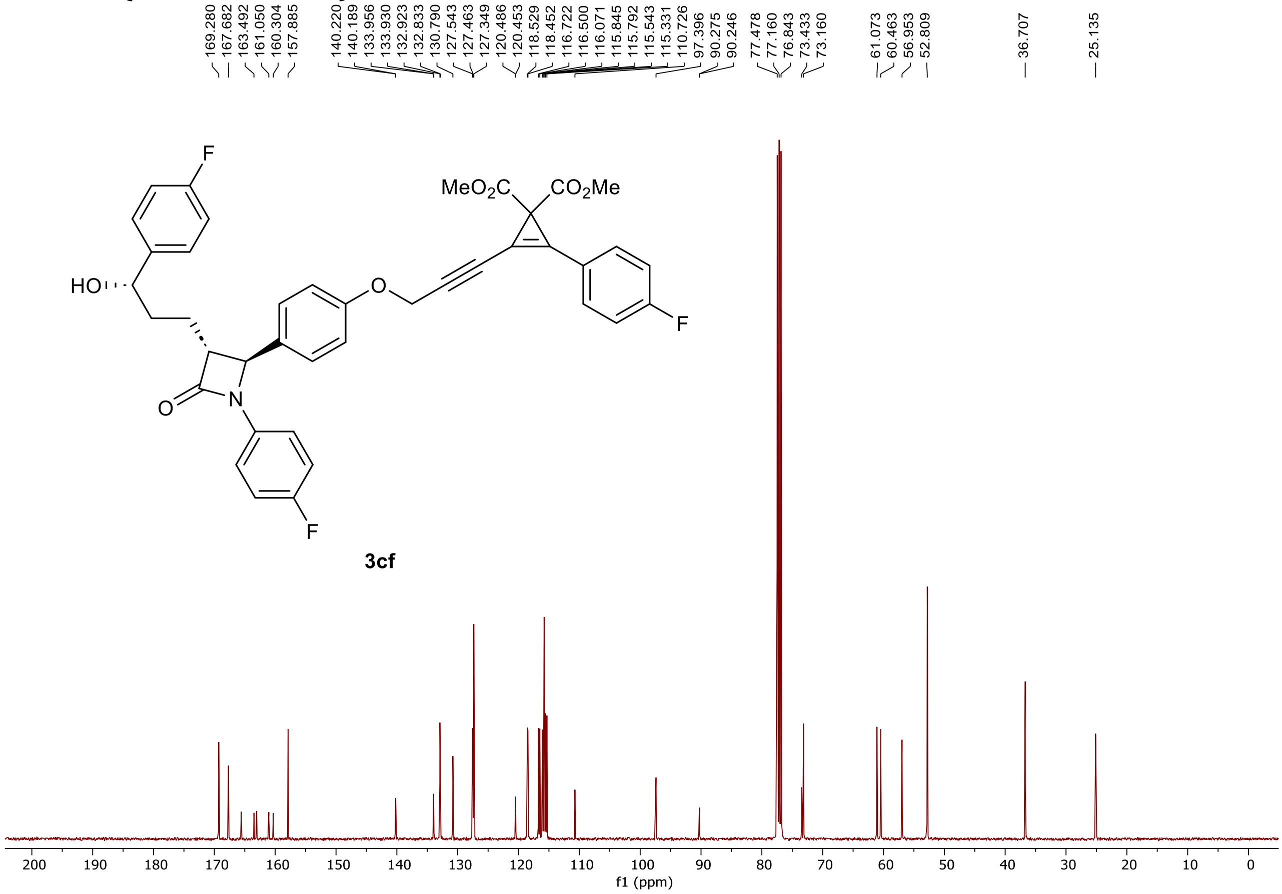

^{19}F NMR (377 MHz, $CDCl_3$) of 3cf

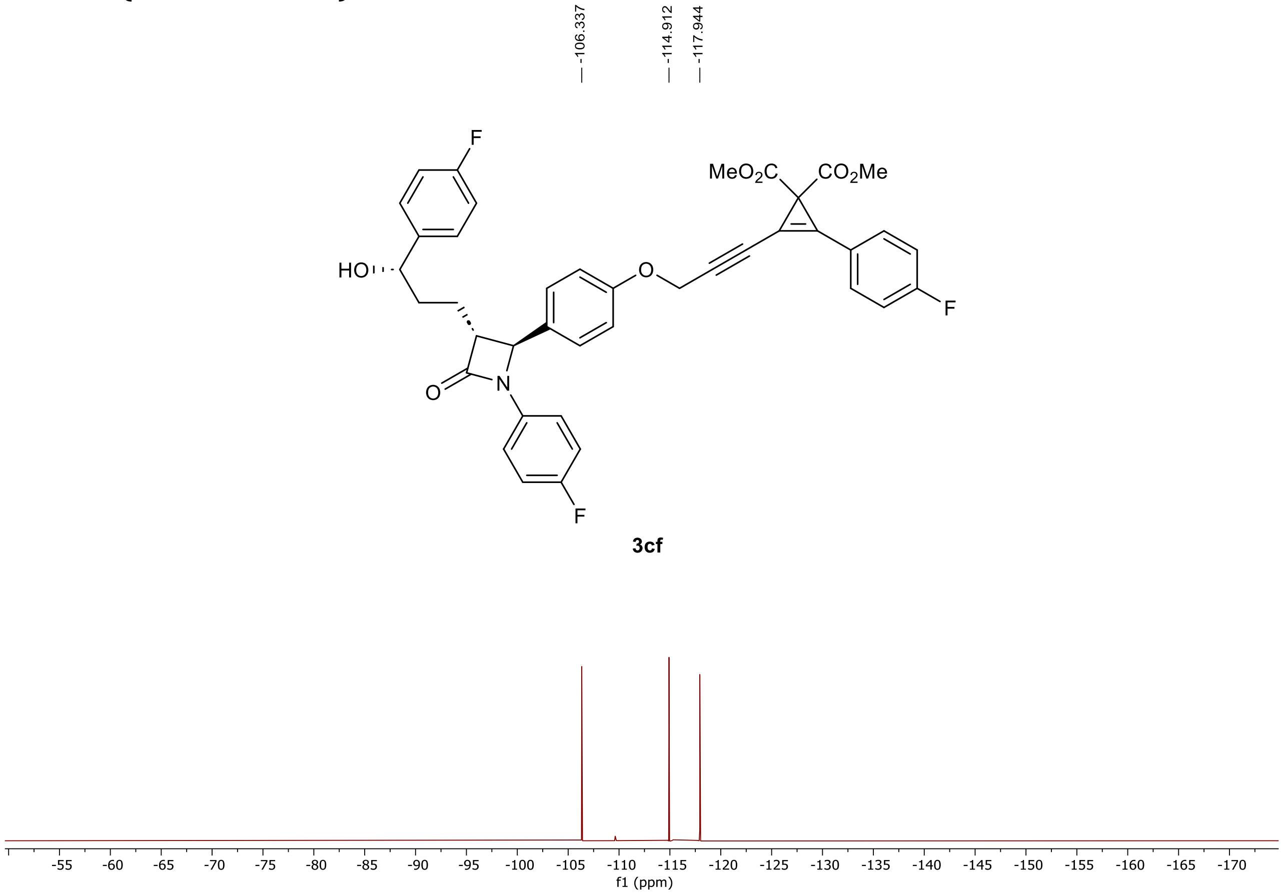

^{1}H NMR (400 MHz, $CDCl_3$) of 3cg

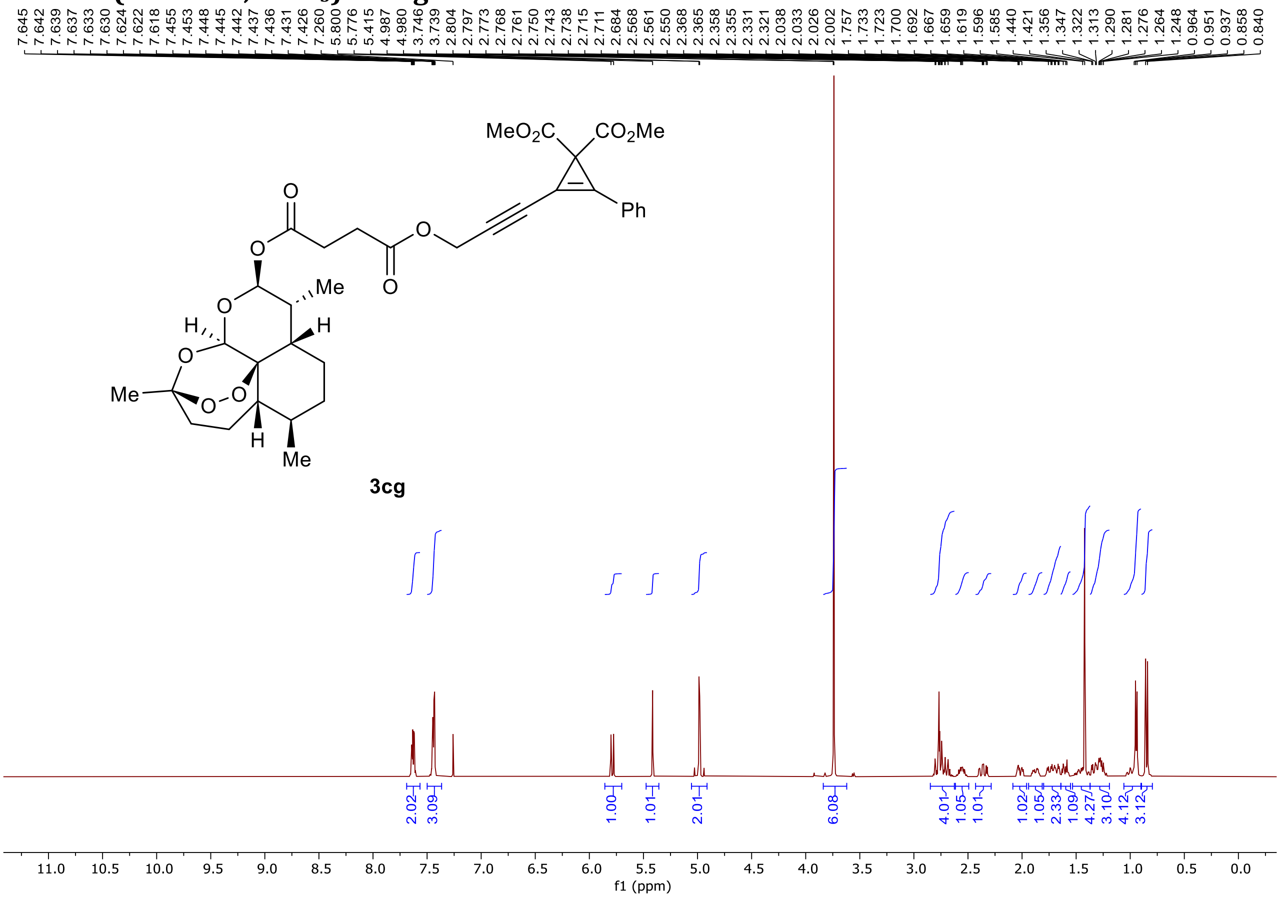

^{13}C NMR (101 MHz, $CDCl_3$) of 3cg

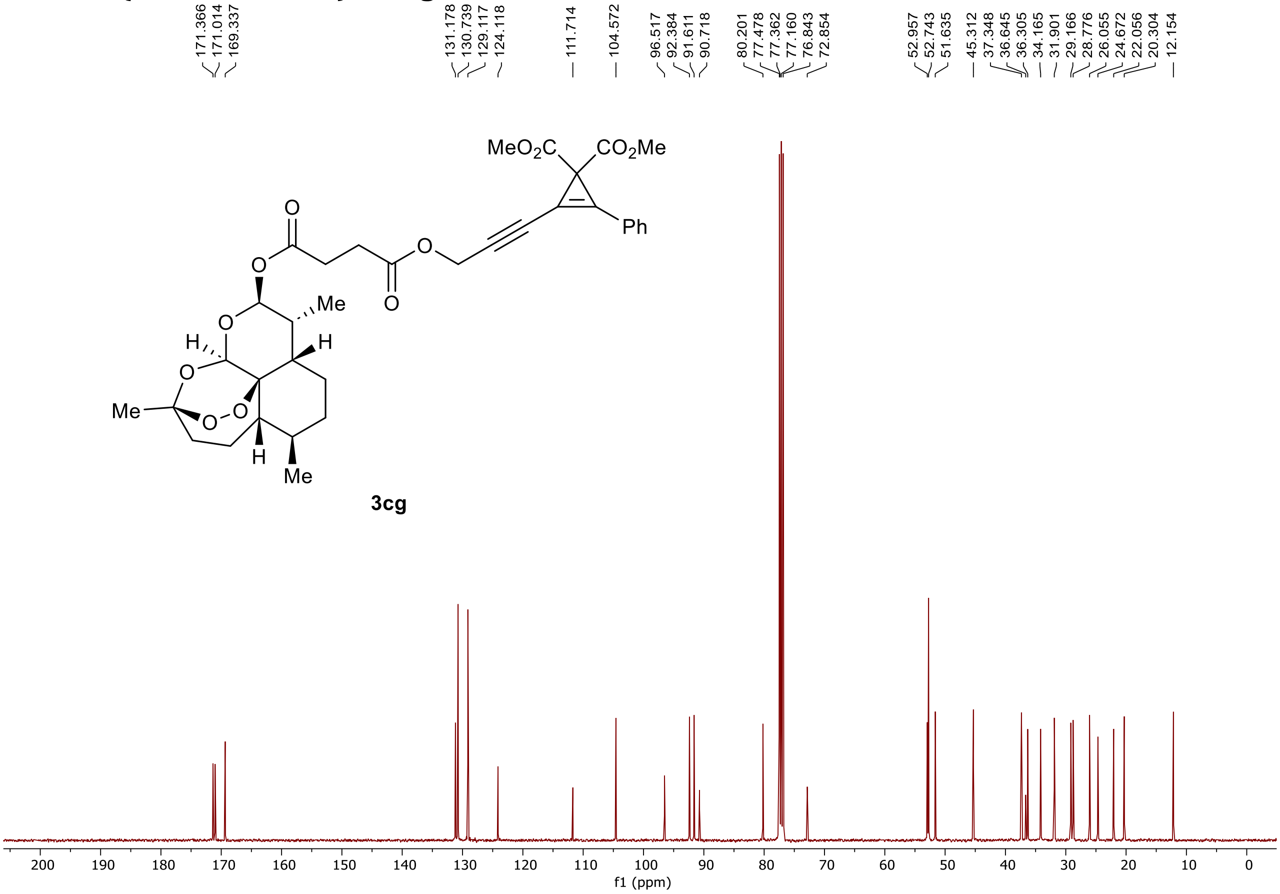

^{1}H NMR (400 MHz, $CDCl_3$) of 3ch

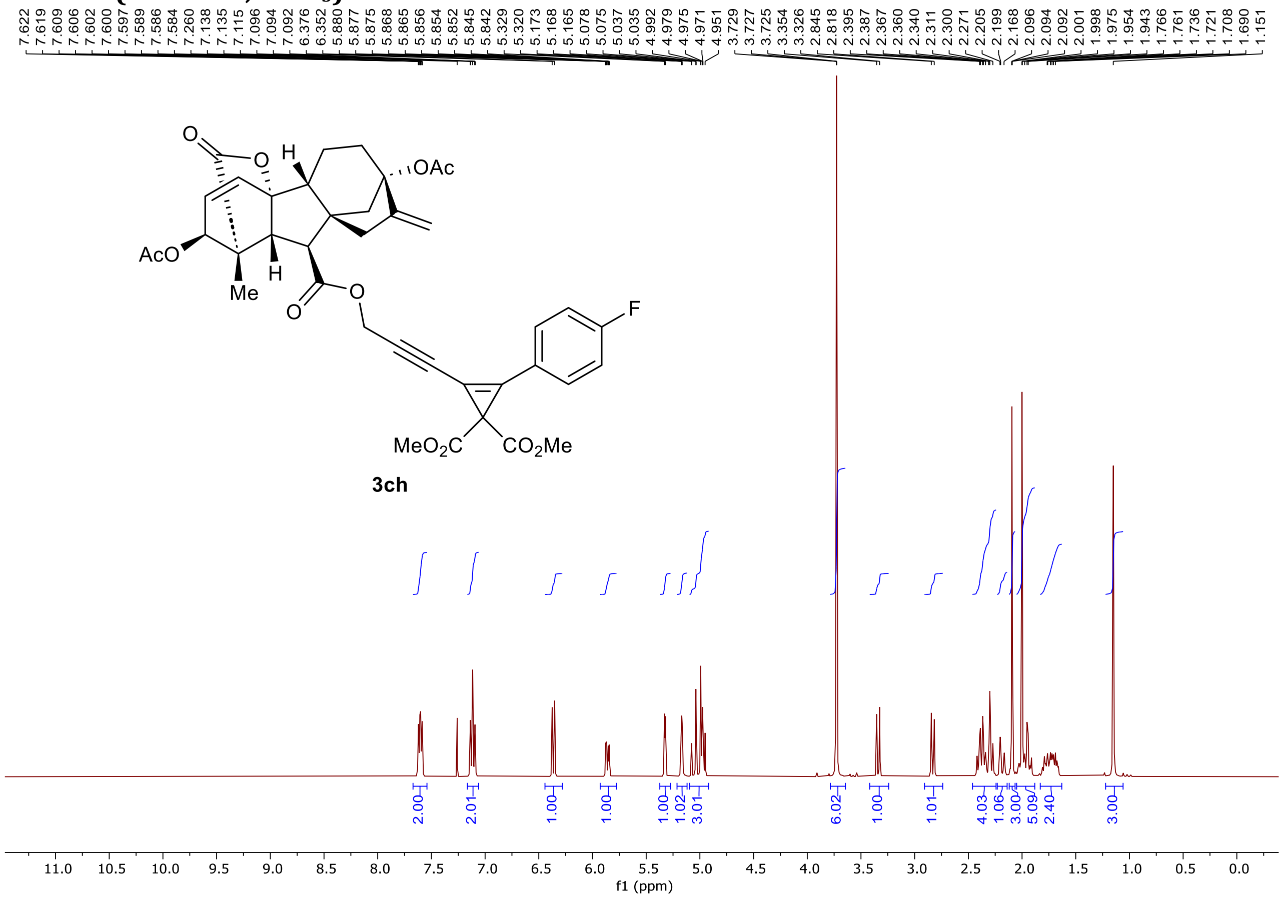

^{13}C NMR (101 MHz, $CDCl_3$) of 3ch

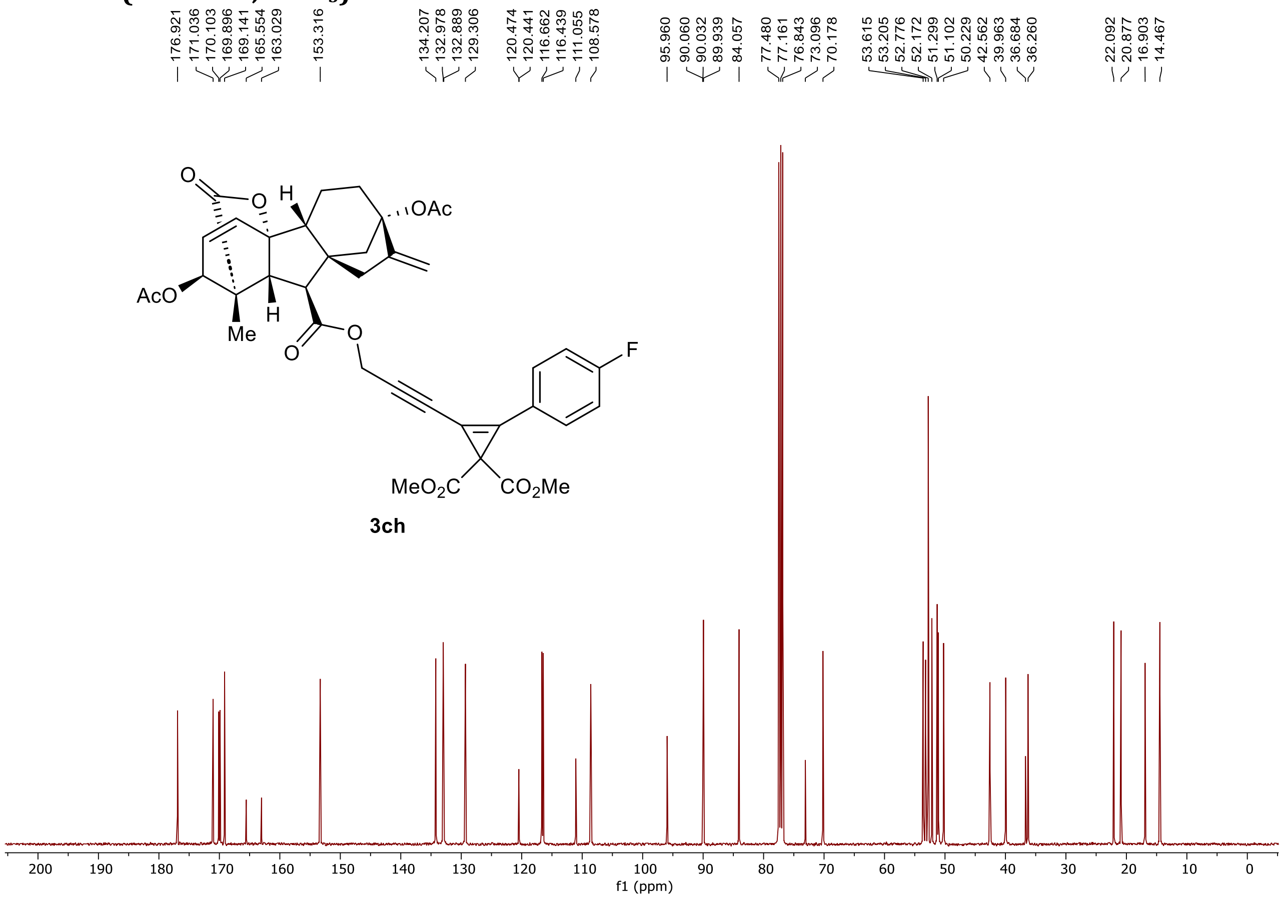

^{19}F NMR (377 MHz, $CDCl_3$) of 3ch

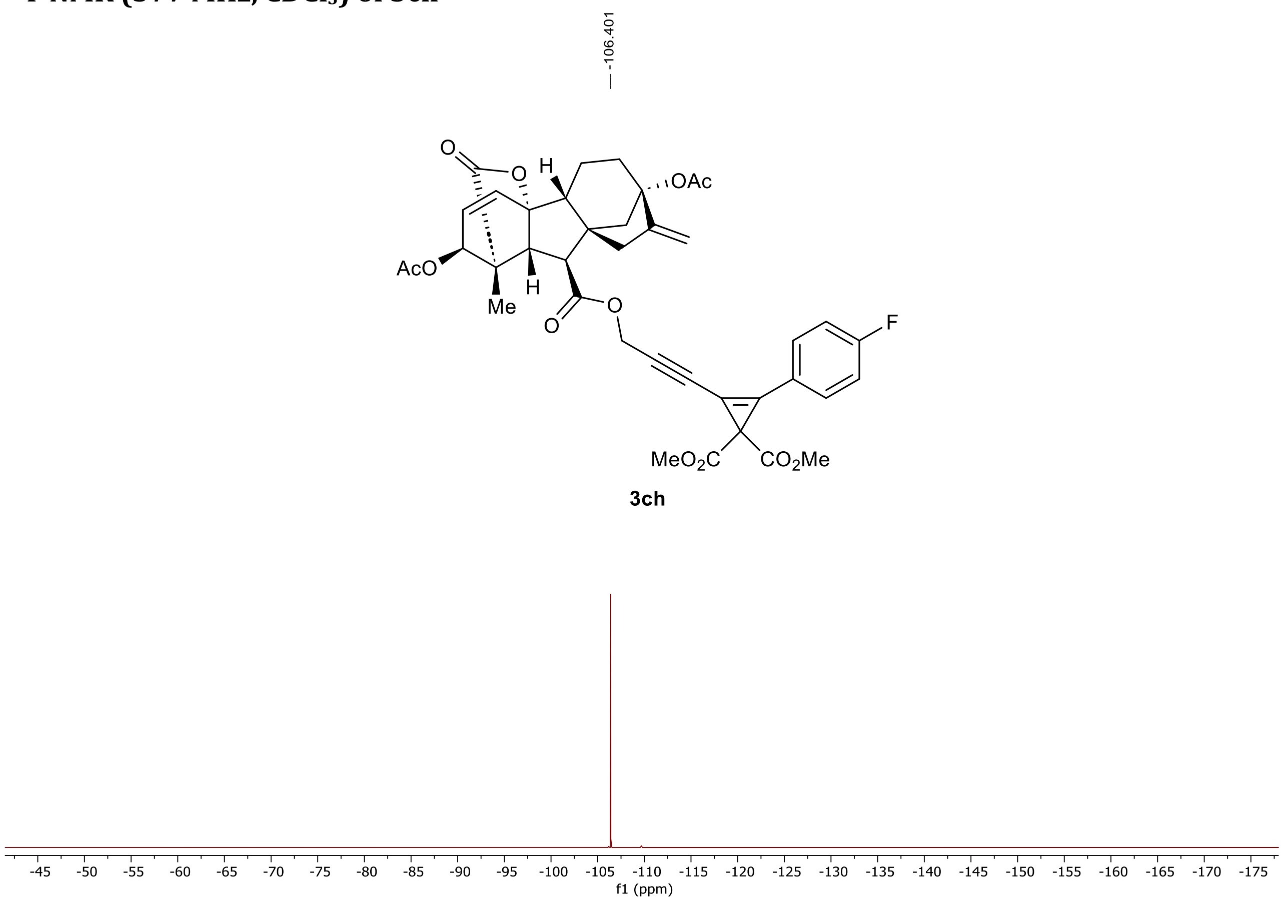

^{1}H NMR (400 MHz, $CDCl_3$) of 6a

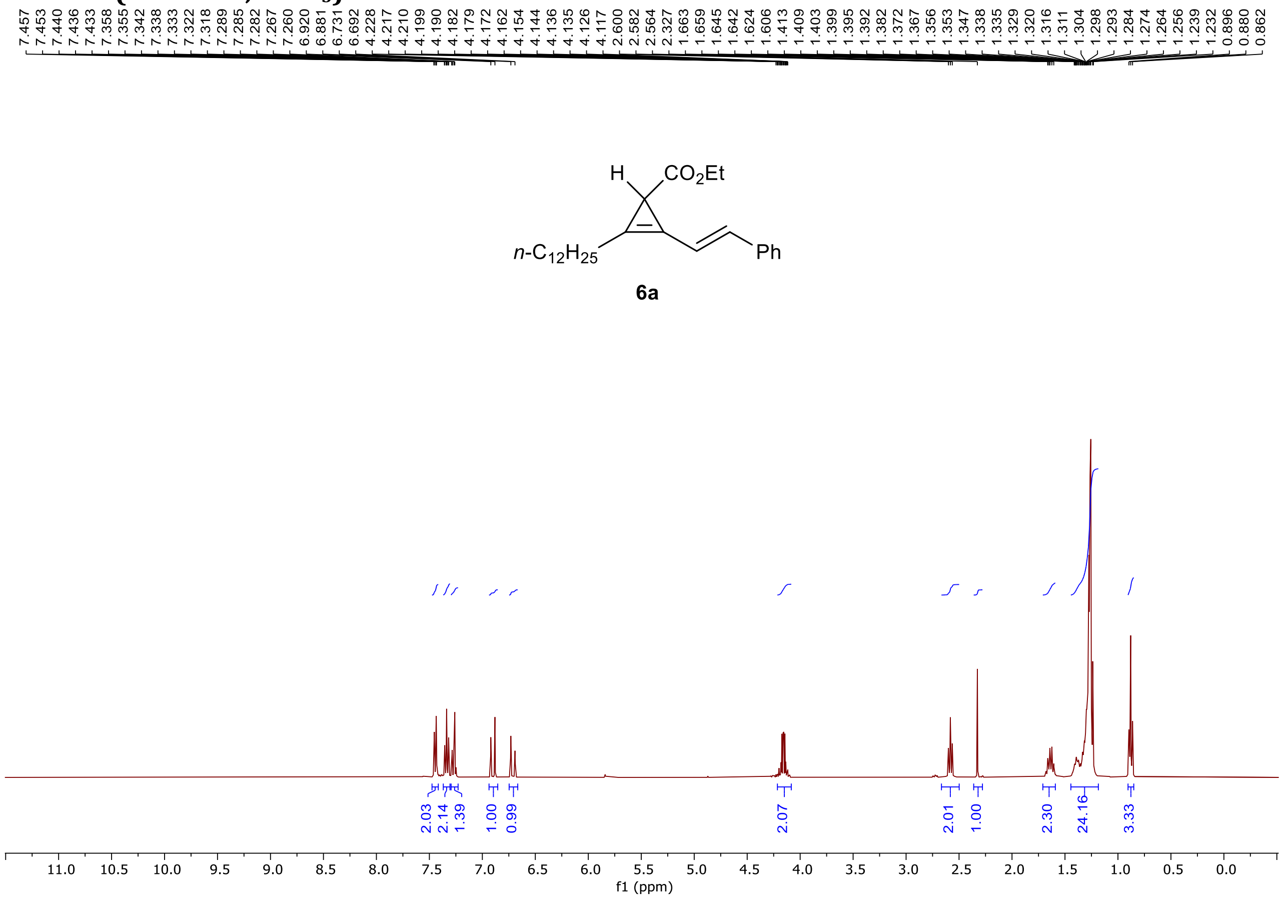

^{13}C NMR (101 MHz, CDCl$_3$) of 6a

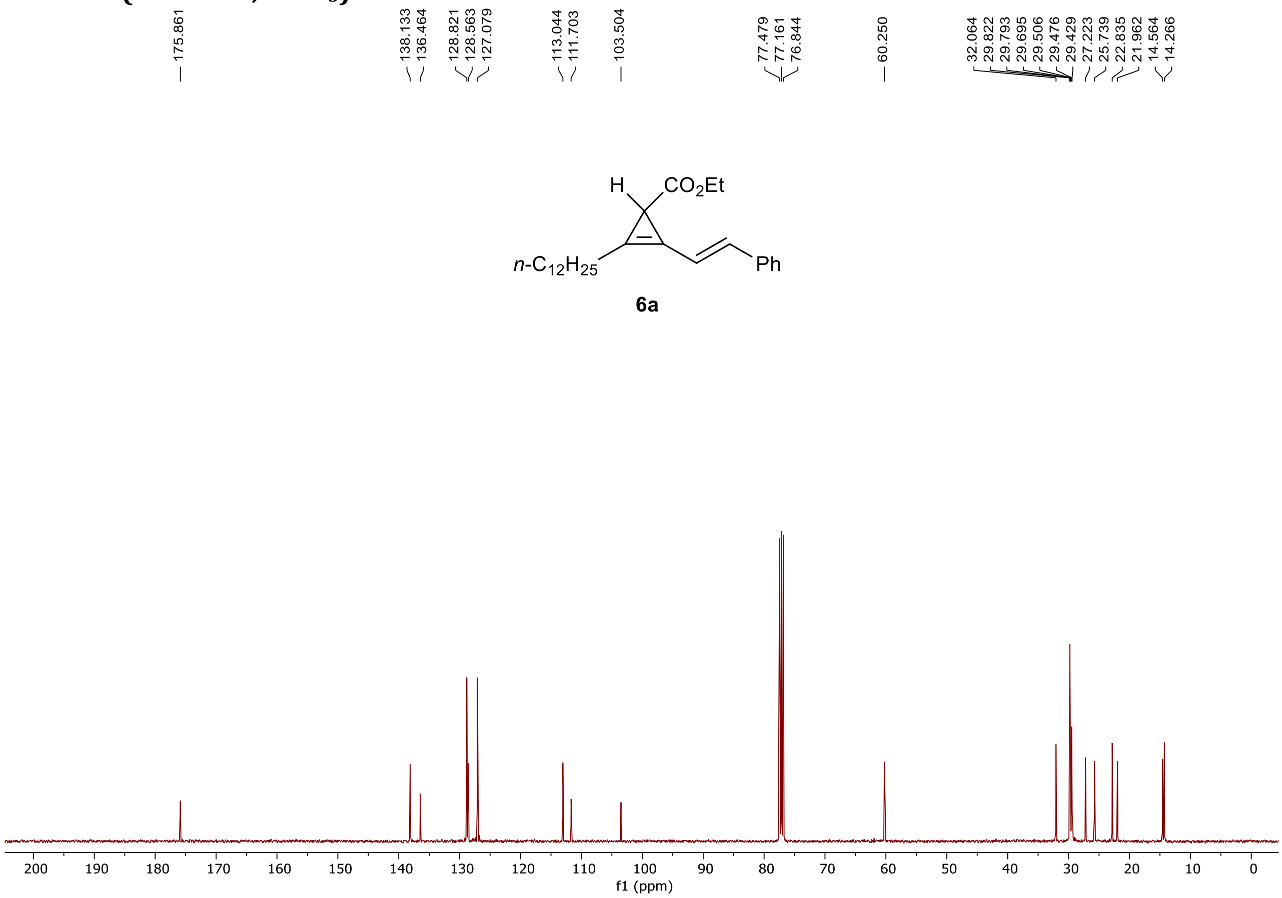

^{1}H NMR (400 MHz, CDCl$_3$) of 6b

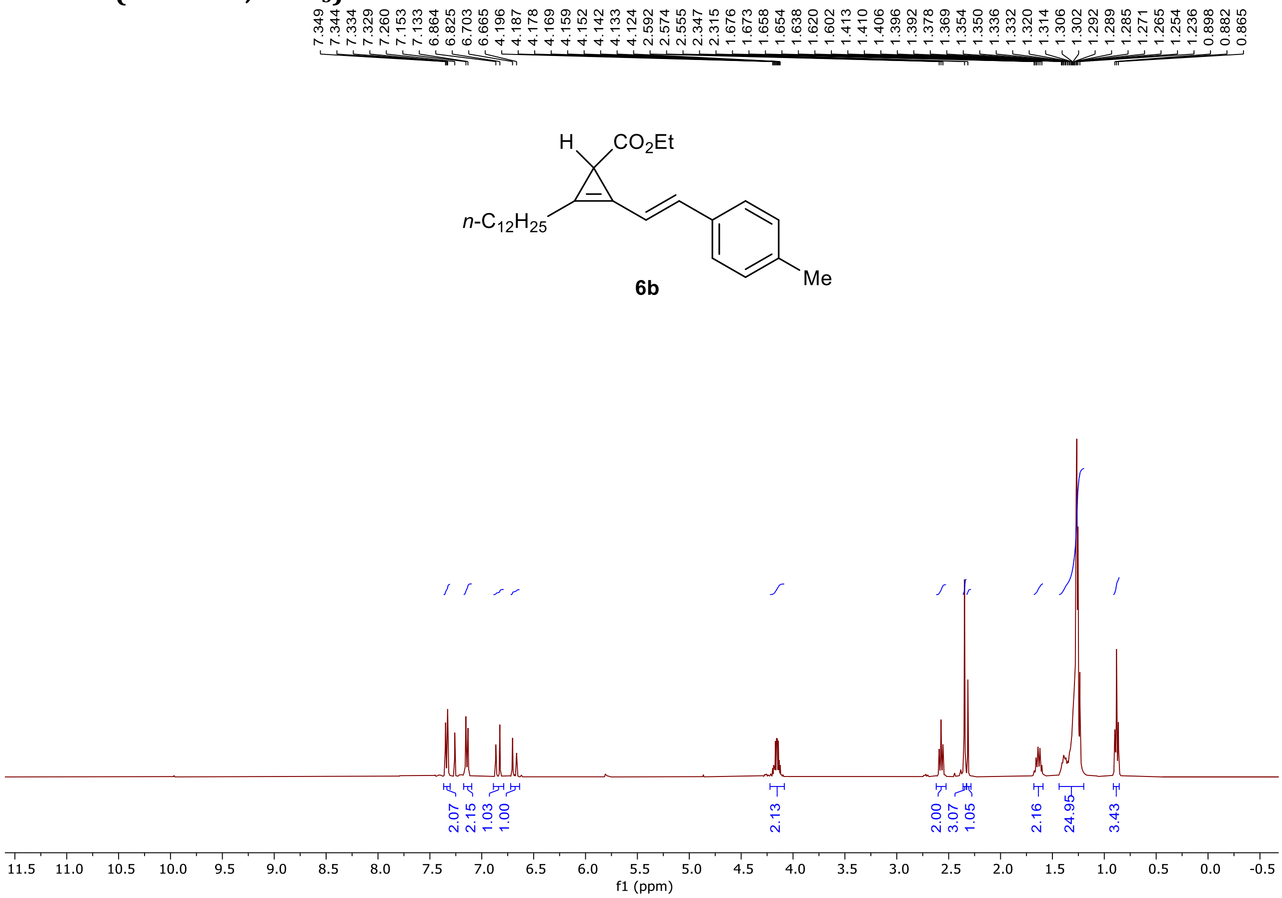

^{13}C NMR (101 MHz, $CDCl_3$) of 6b

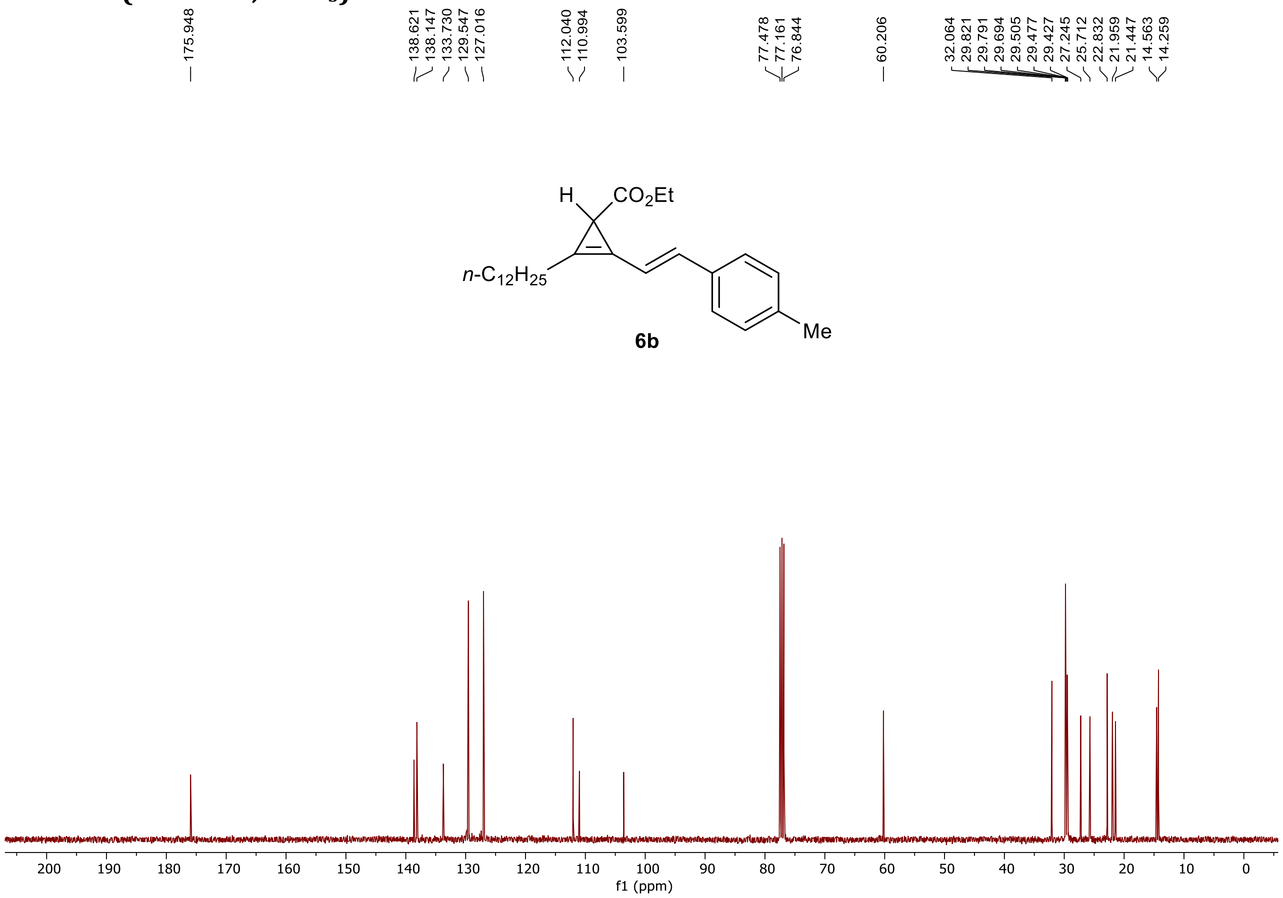

^{1}H NMR (400 MHz, $CDCl_3$) of 6c

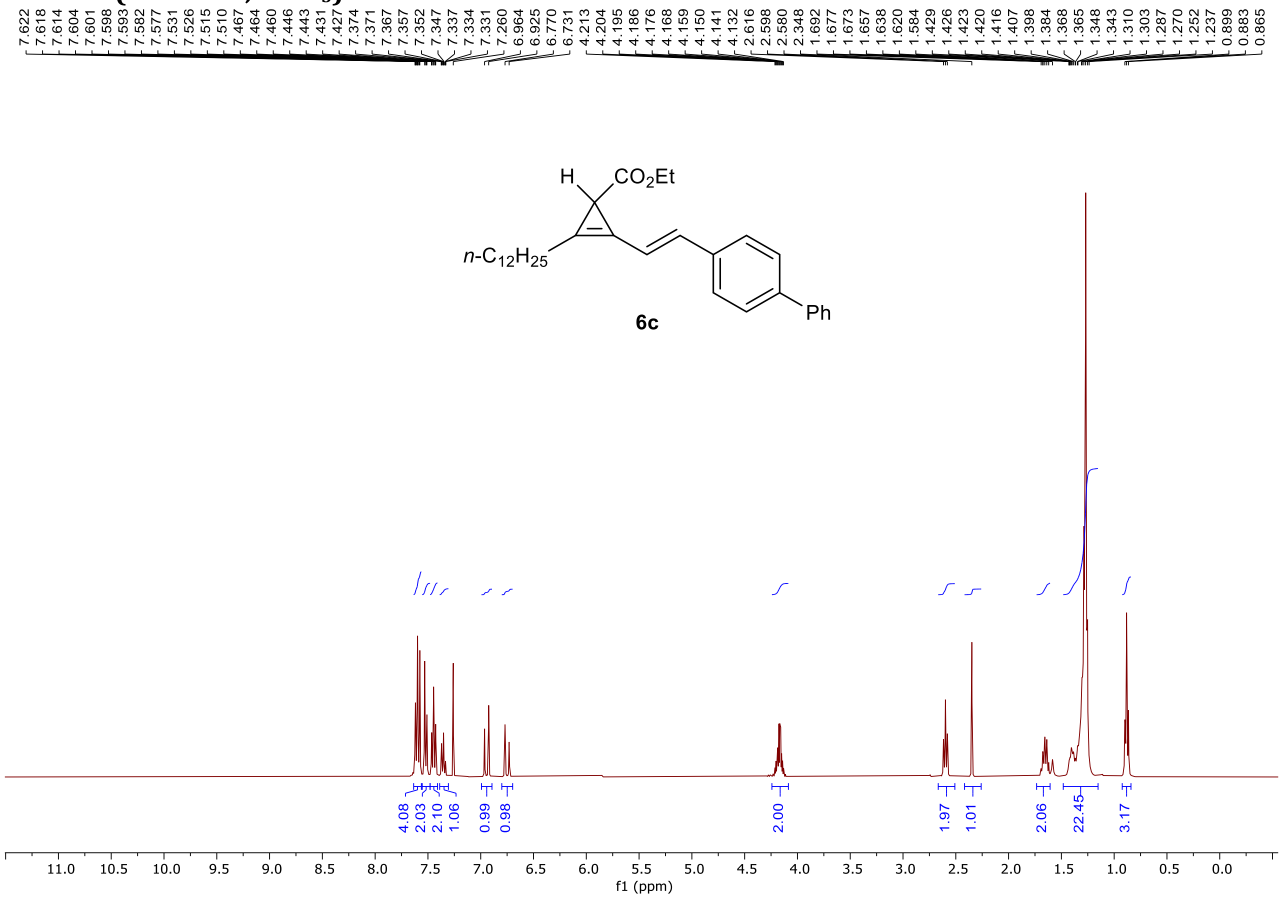

^{13}C NMR (101 MHz, $CDCl_3$) of 6c

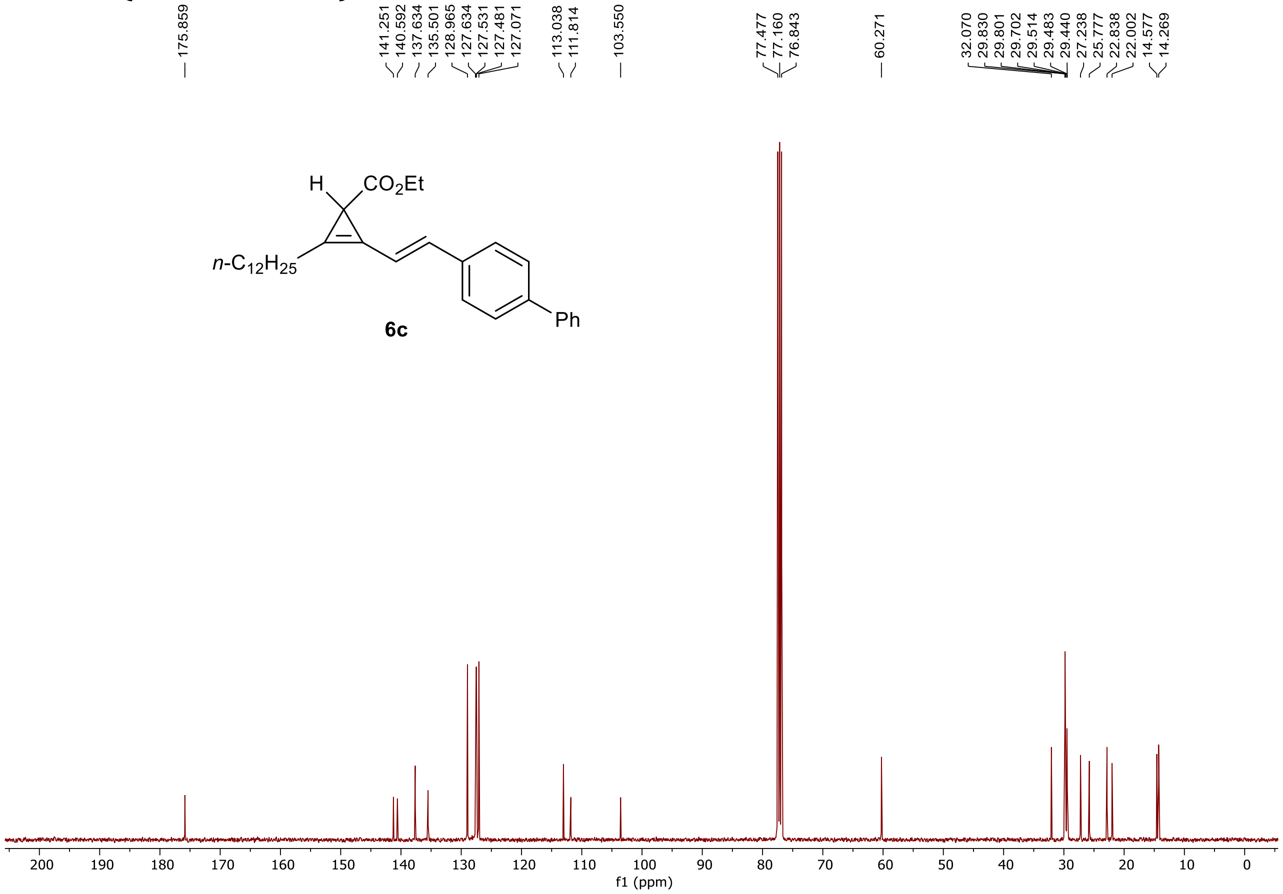

^{1}H NMR (400 MHz, $CDCl_3$) of 6d

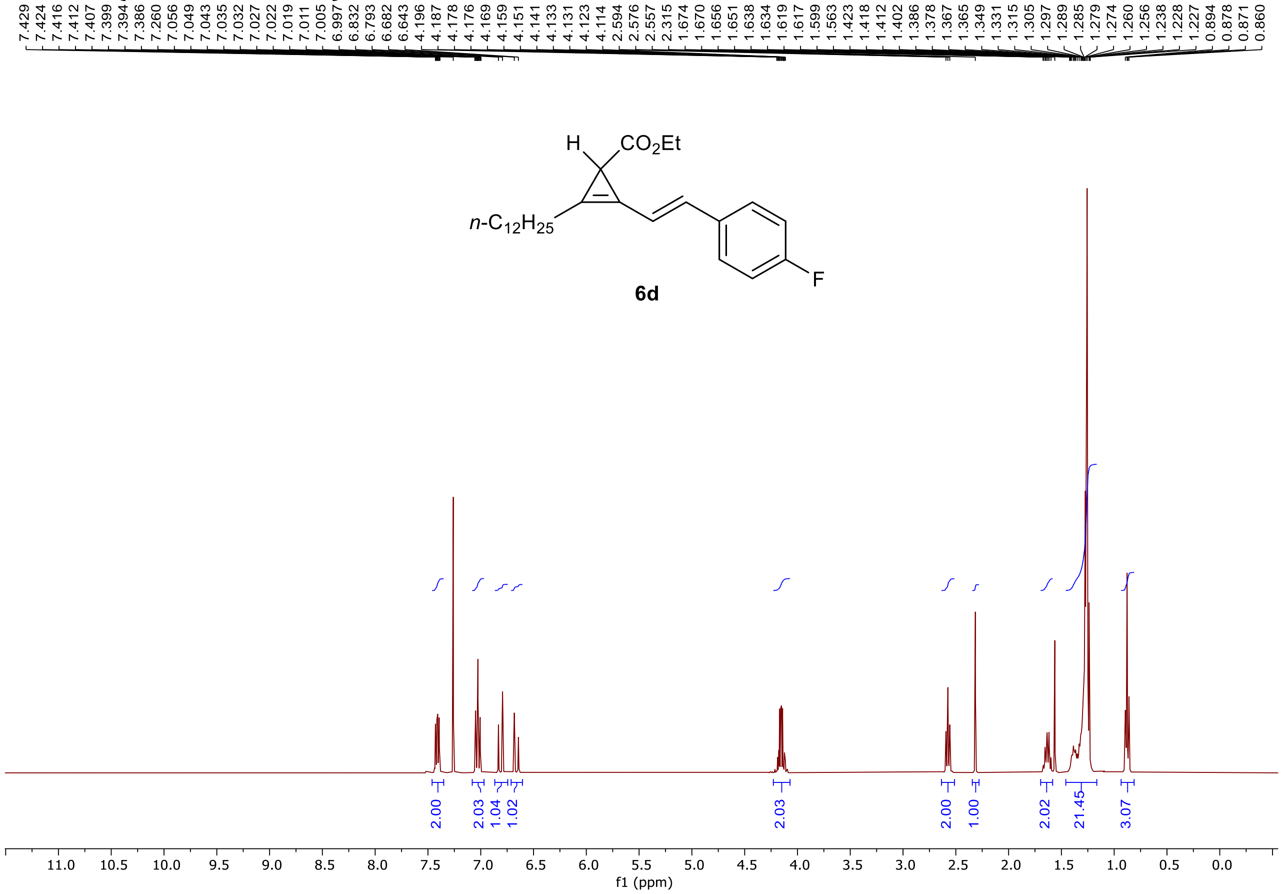

^{13}C NMR (101 MHz, $CDCl_3$) of 6d

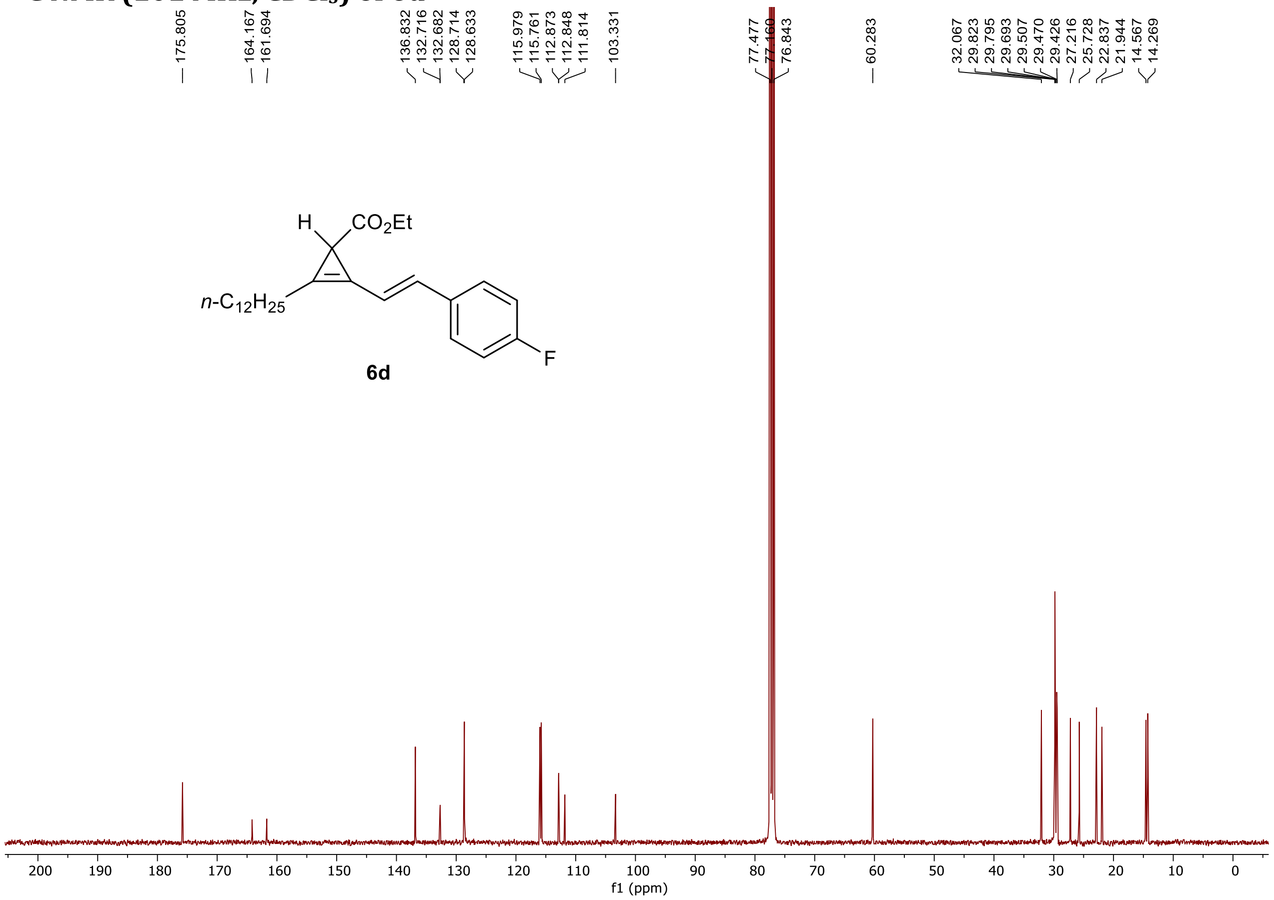

^{19}F NMR (377 MHz, $CDCl_3$) of 6d

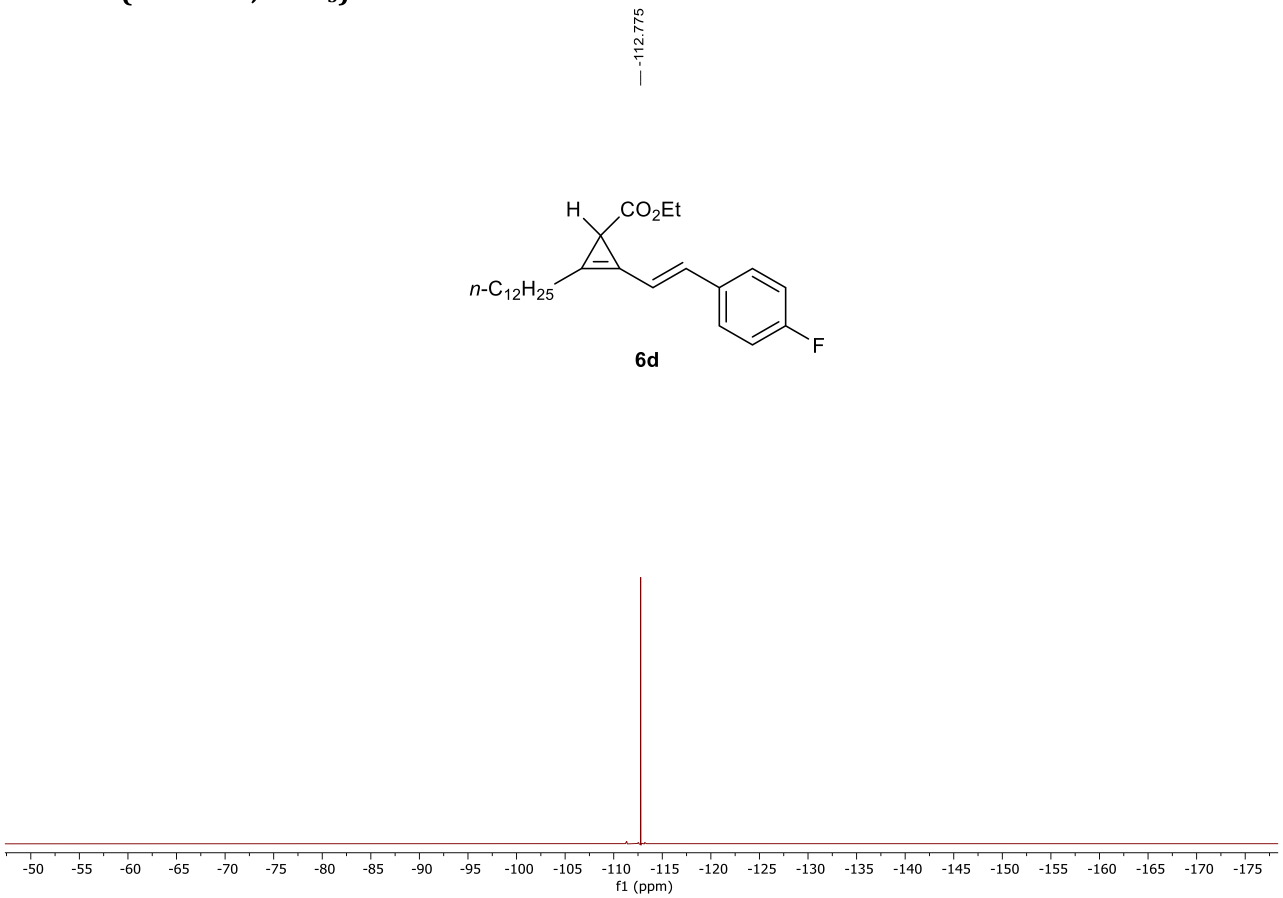

^{1}H NMR (400 MHz, $CDCl_3$) of 6e

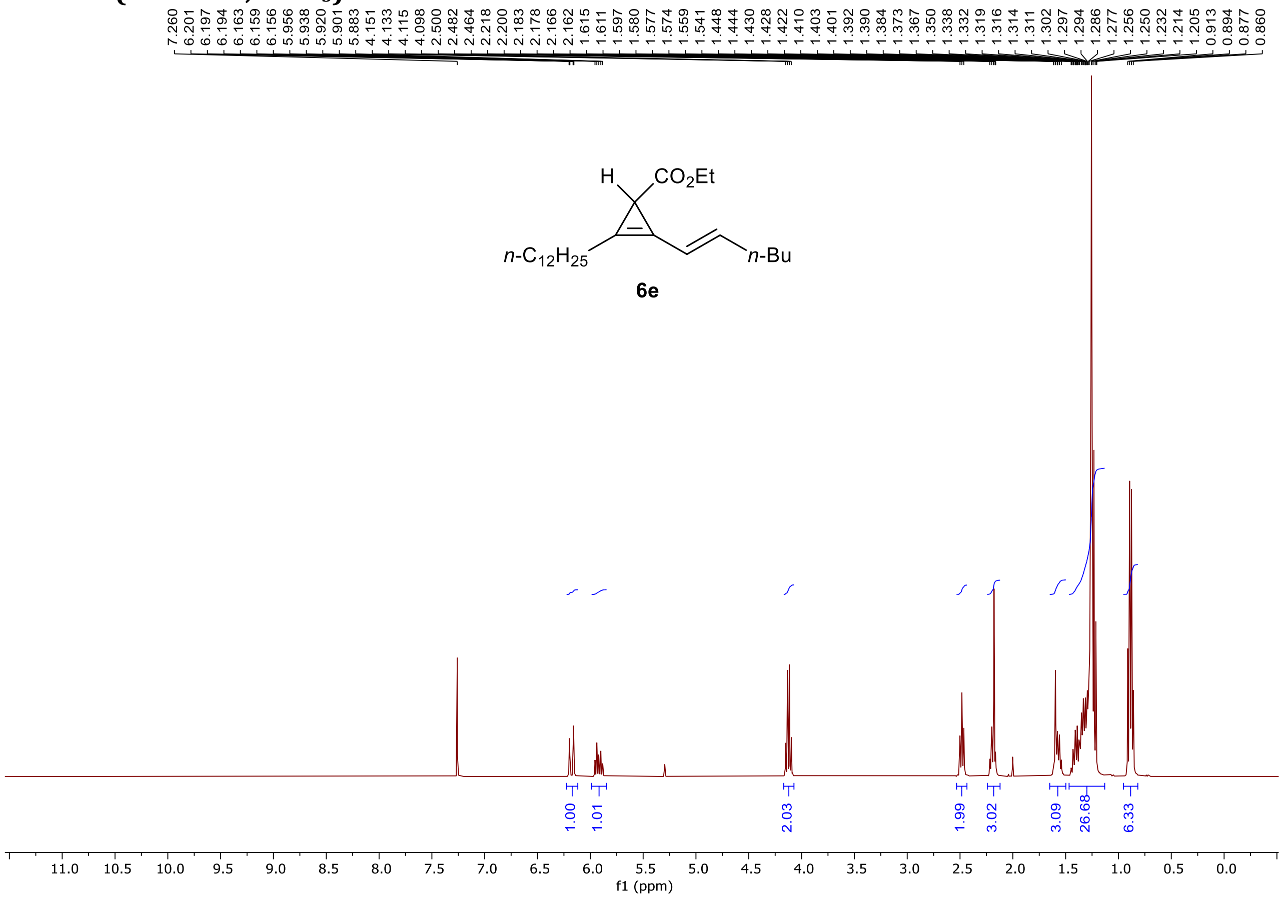

^{13}C NMR (101 MHz, $CDCl_3$) of 6e

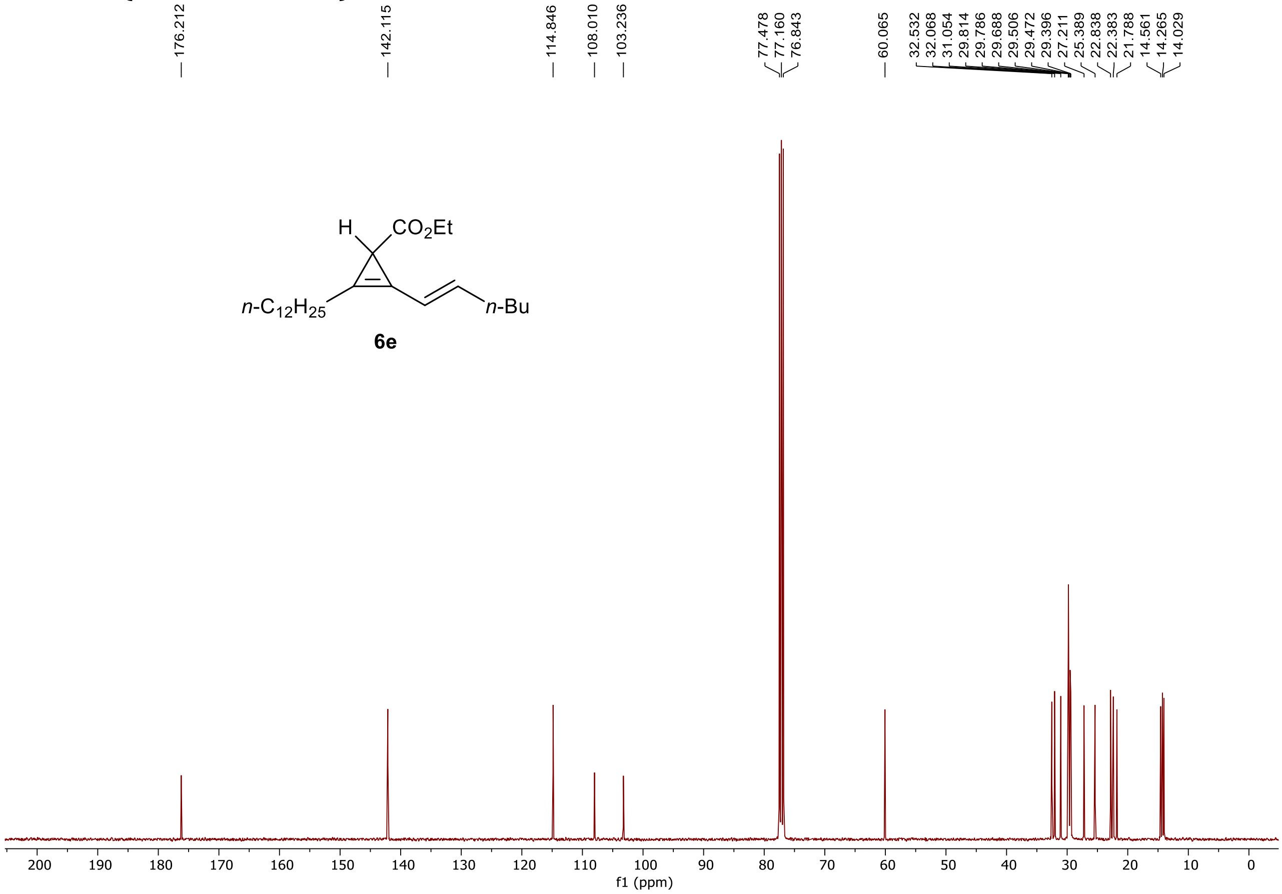

^{1}H NMR (400 MHz, $CDCl_3$) of 6f

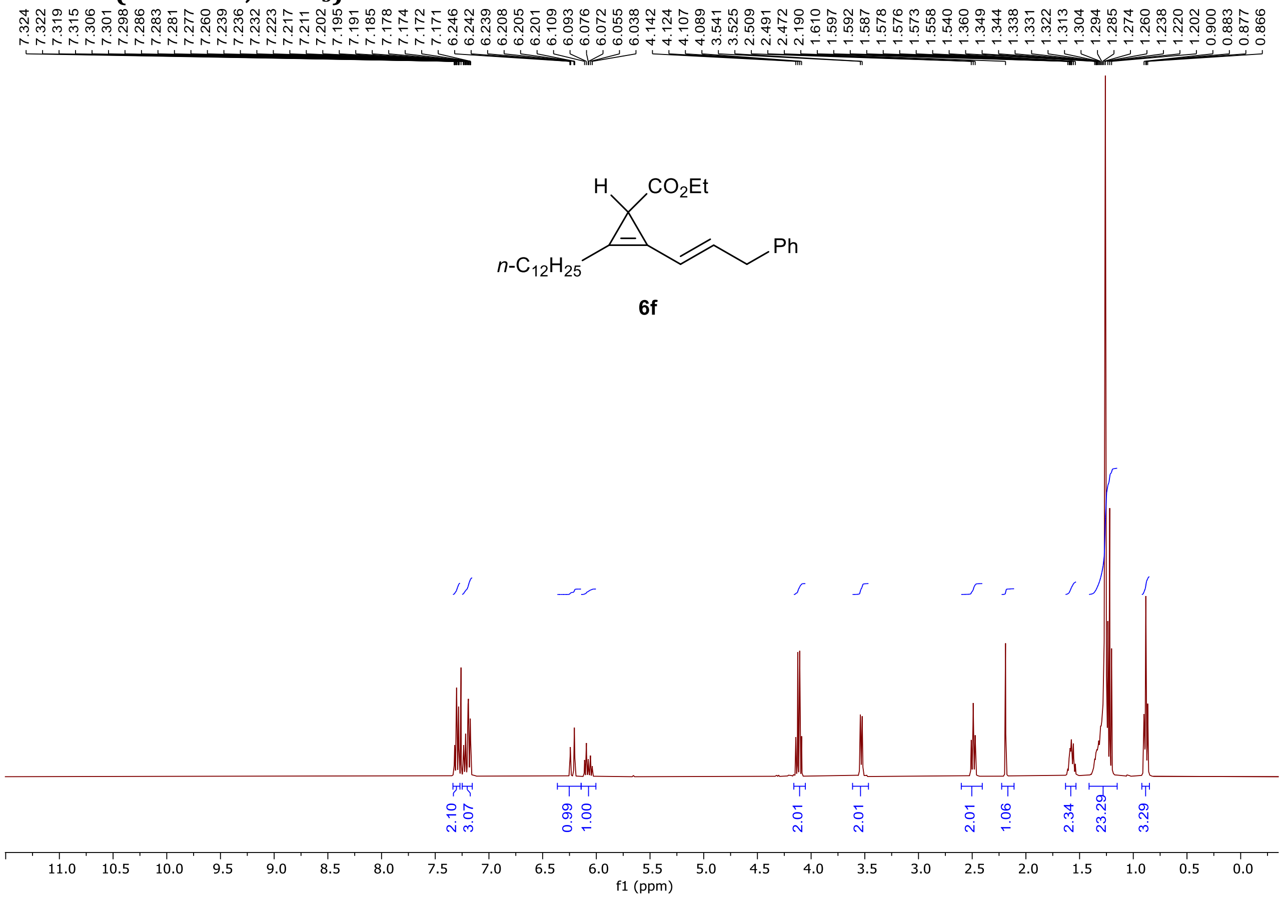

^{13}C NMR (101 MHz, $CDCl_3$) of 6f

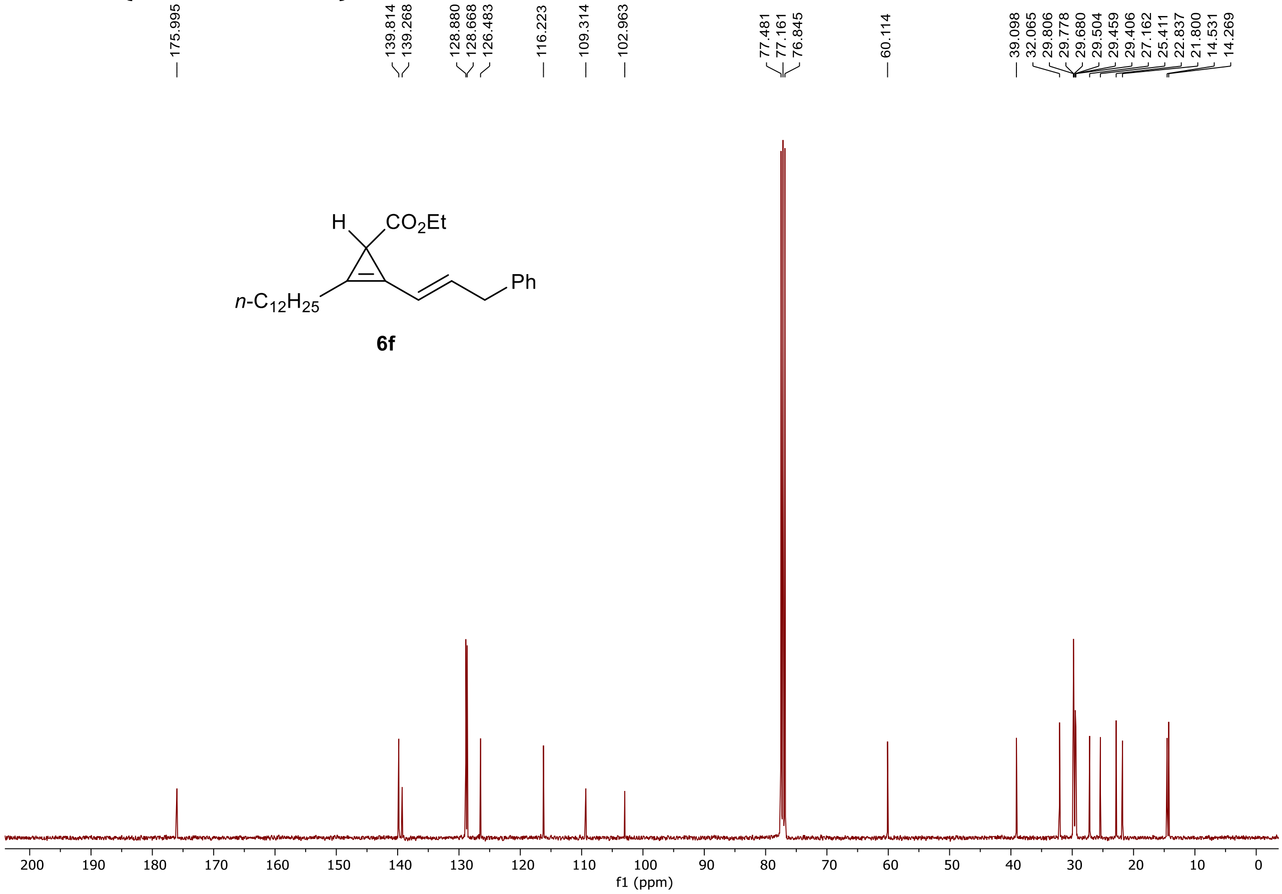

^1H NMR (400 MHz, $CDCl_3$) of 6g

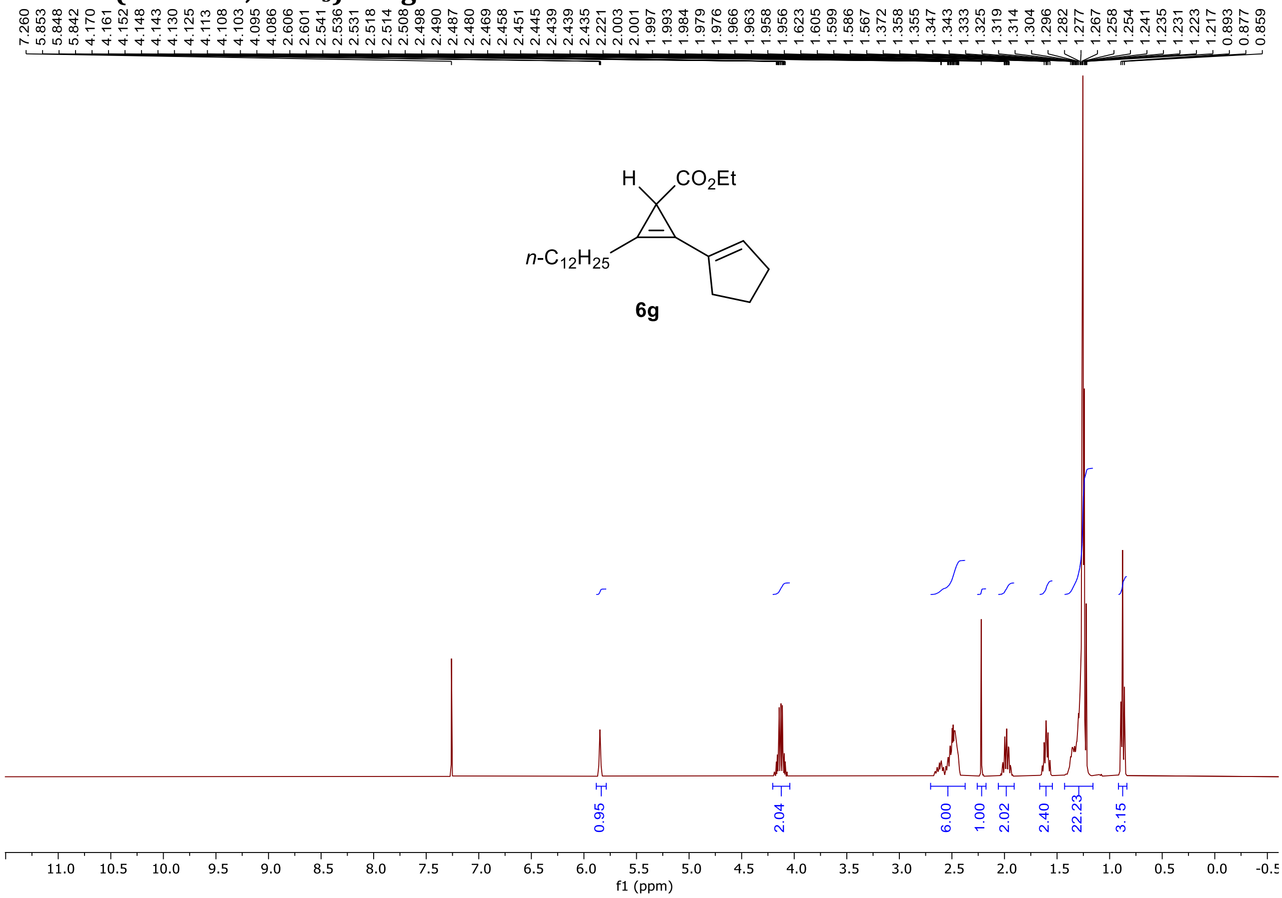

^{13}C NMR (101 MHz, $CDCl_3$) of 6g

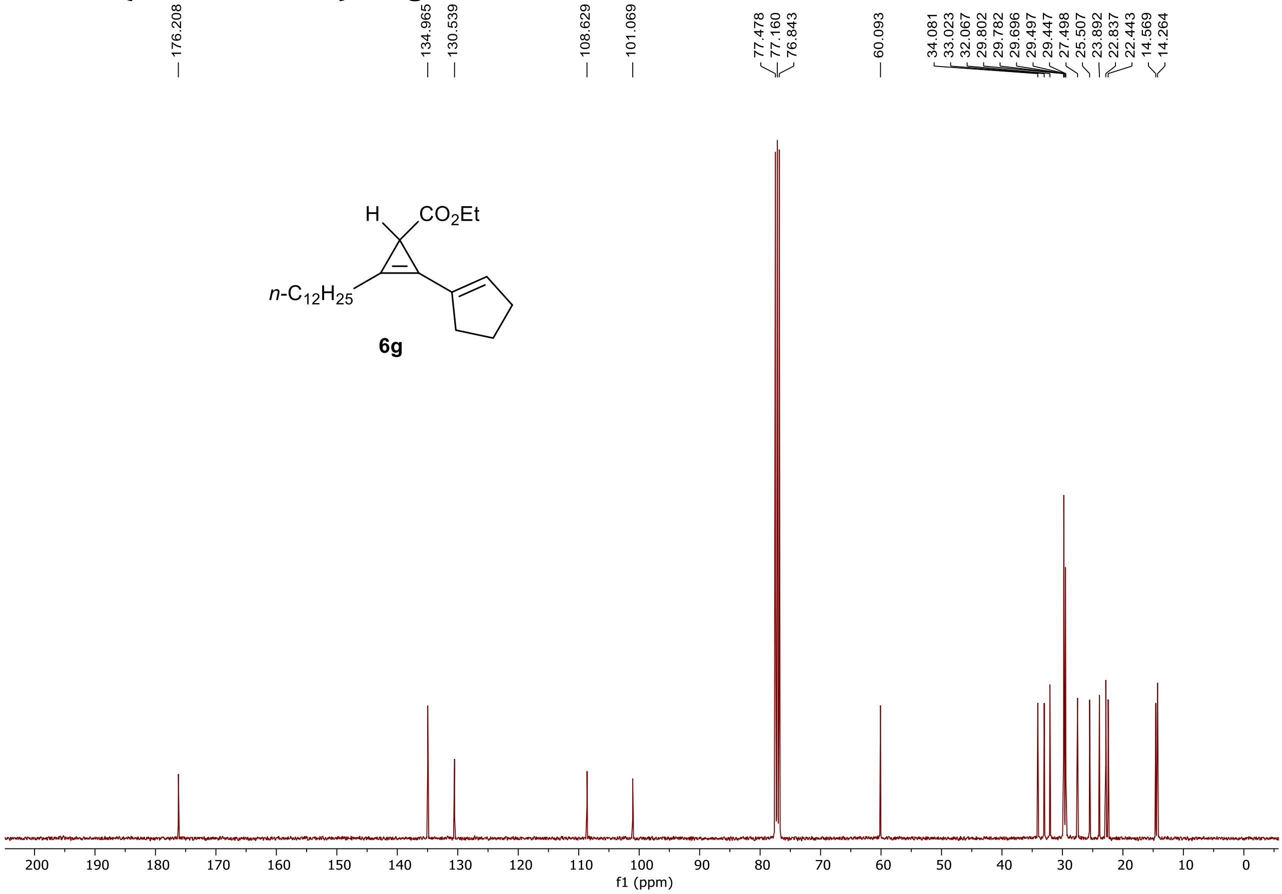

^{1}H NMR (400 MHz, $CDCl_3$) of 6h

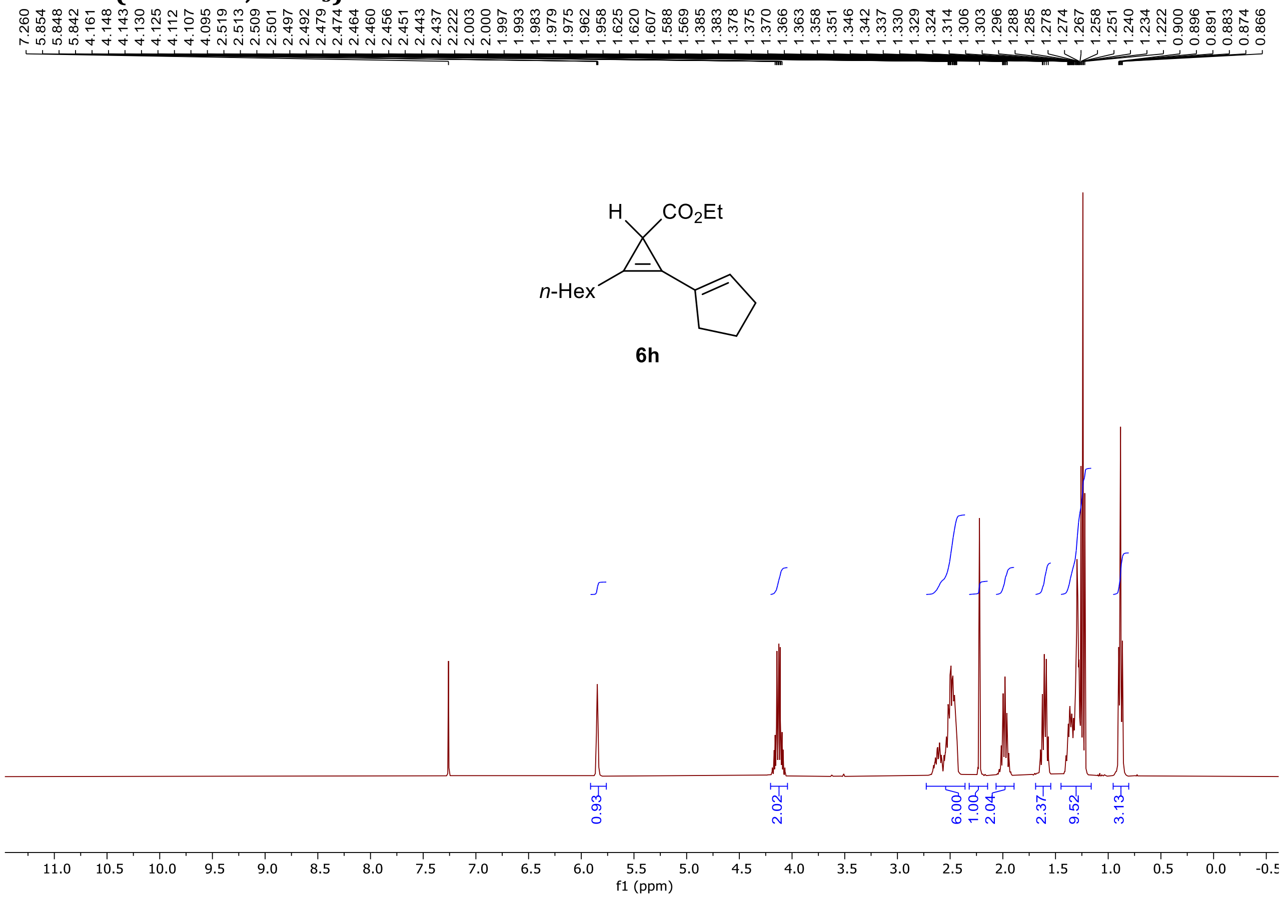

^{13}C NMR (101 MHz, $CDCl_3$) of 6h

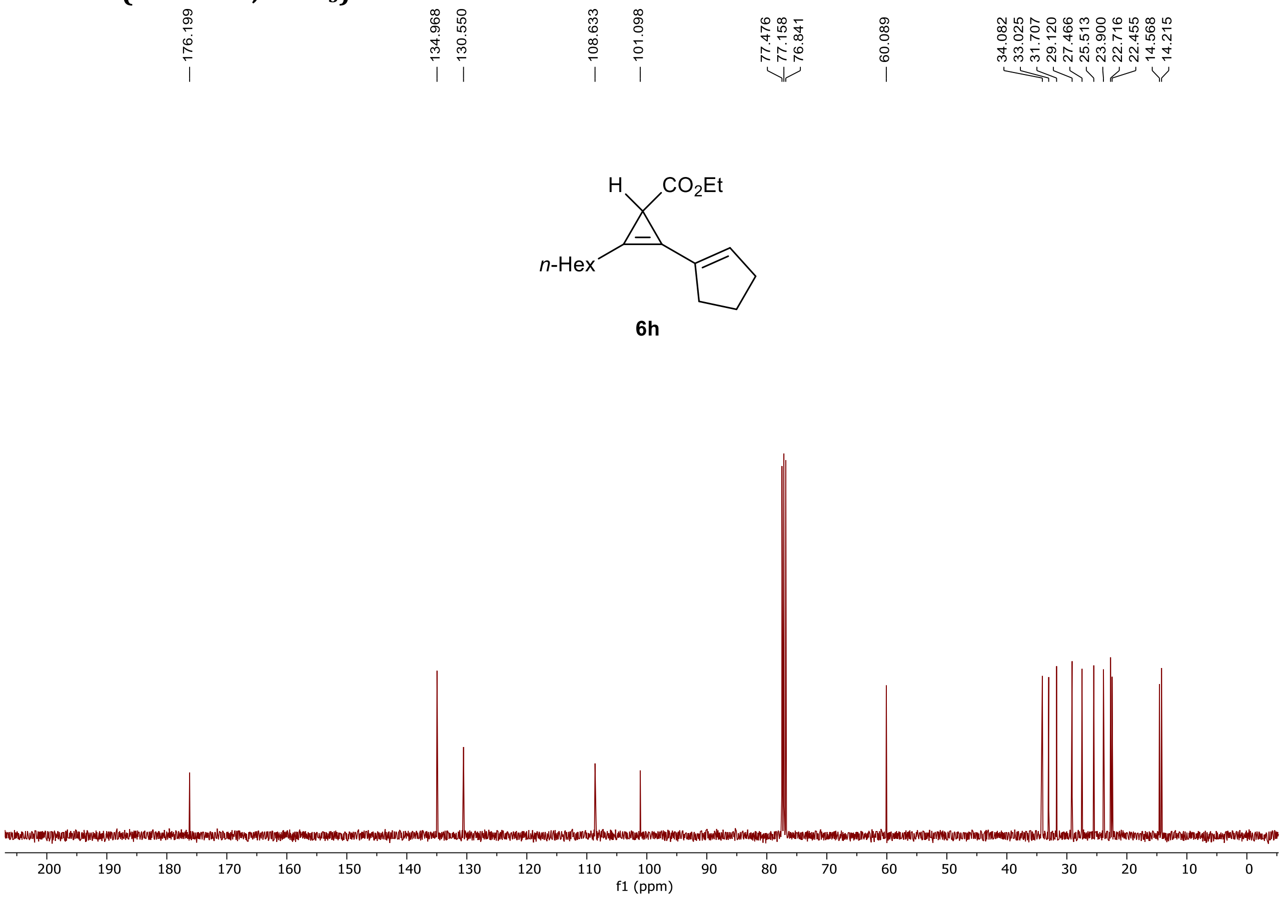

^{1}H NMR (400 MHz, $CDCl_3$) of 6i

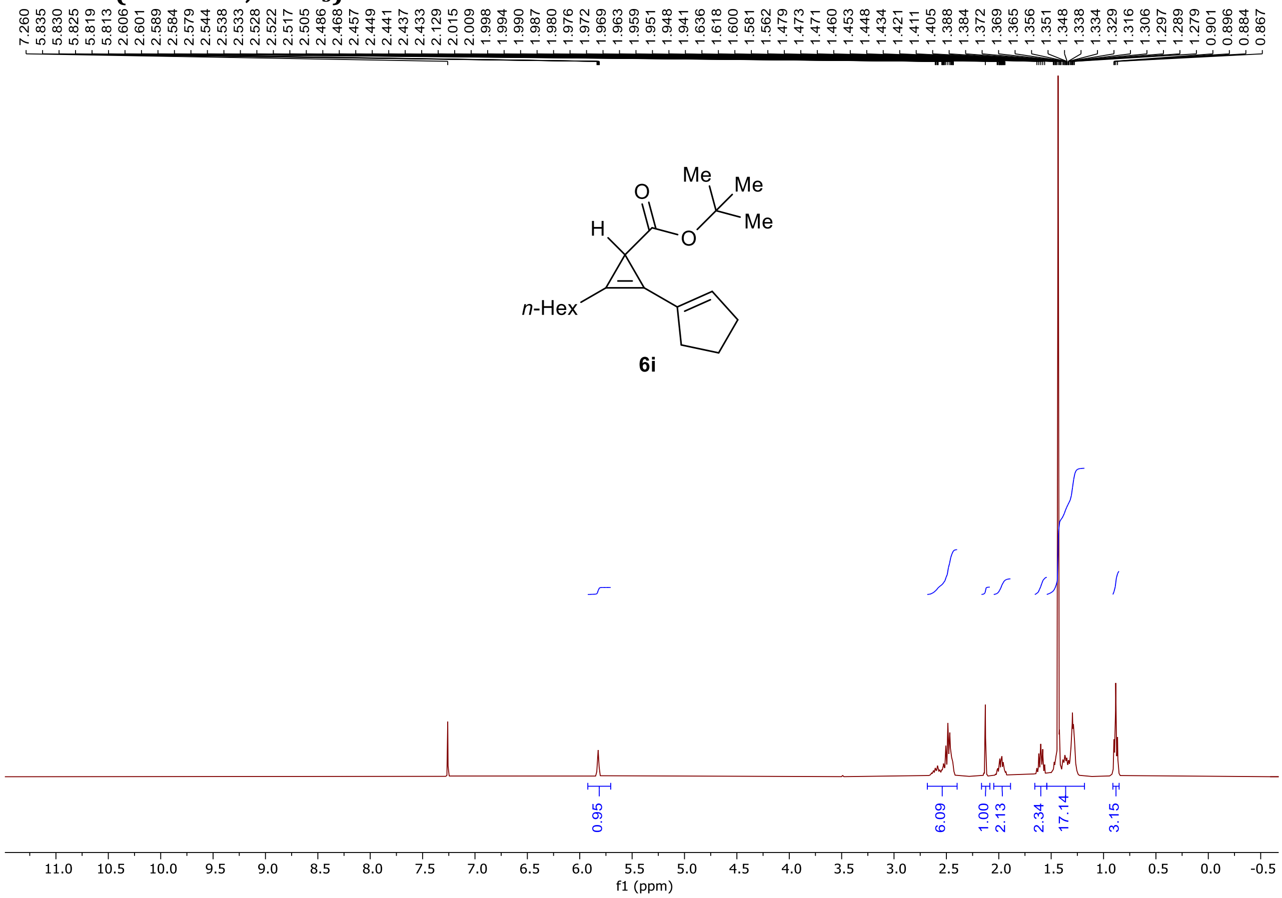

^{13}C NMR (101 MHz, $CDCl_3$) of 6i

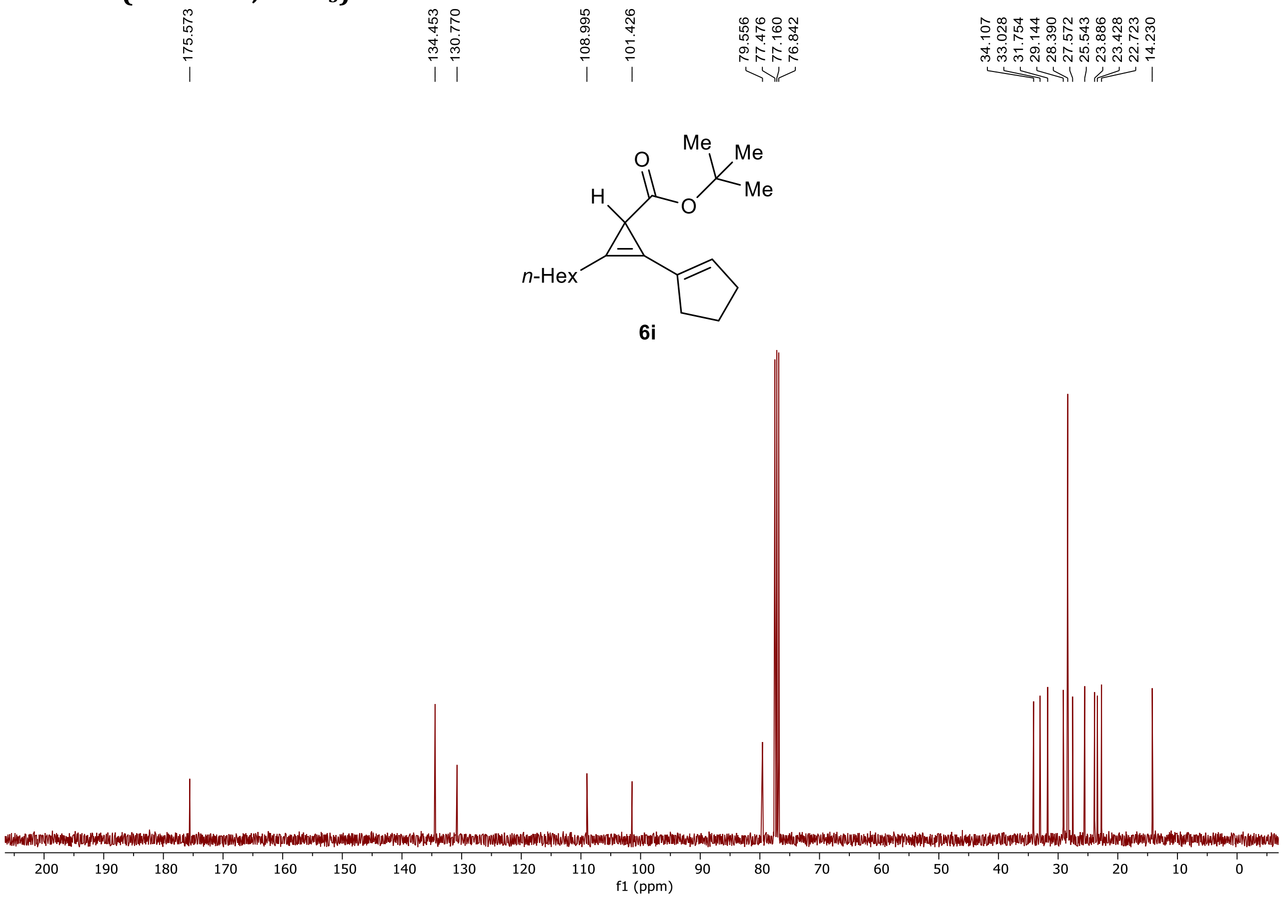

^{1}H NMR (400 MHz, $CDCl_3$) of 6j

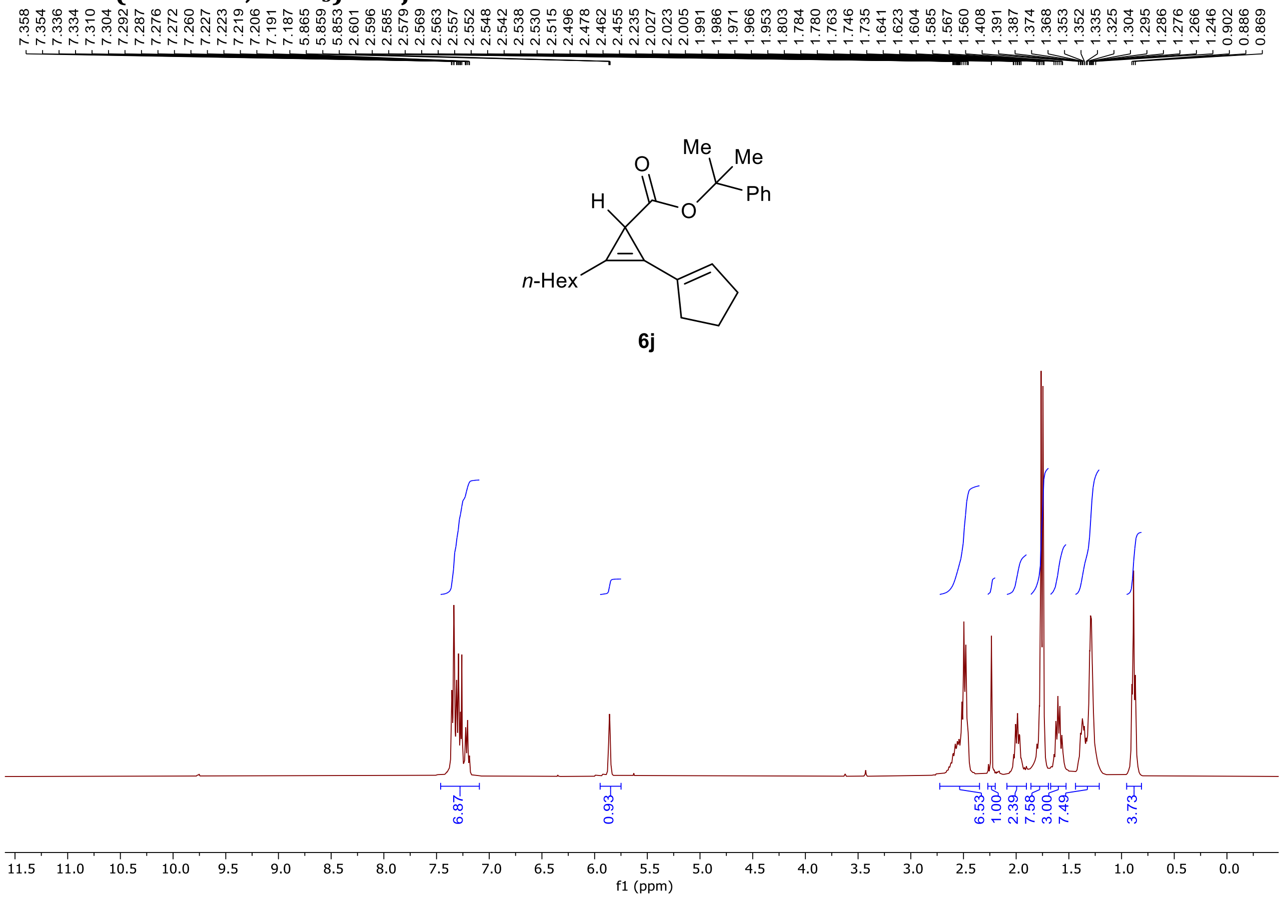

^{13}C NMR (101 MHz, $CDCl_3$) of 6j

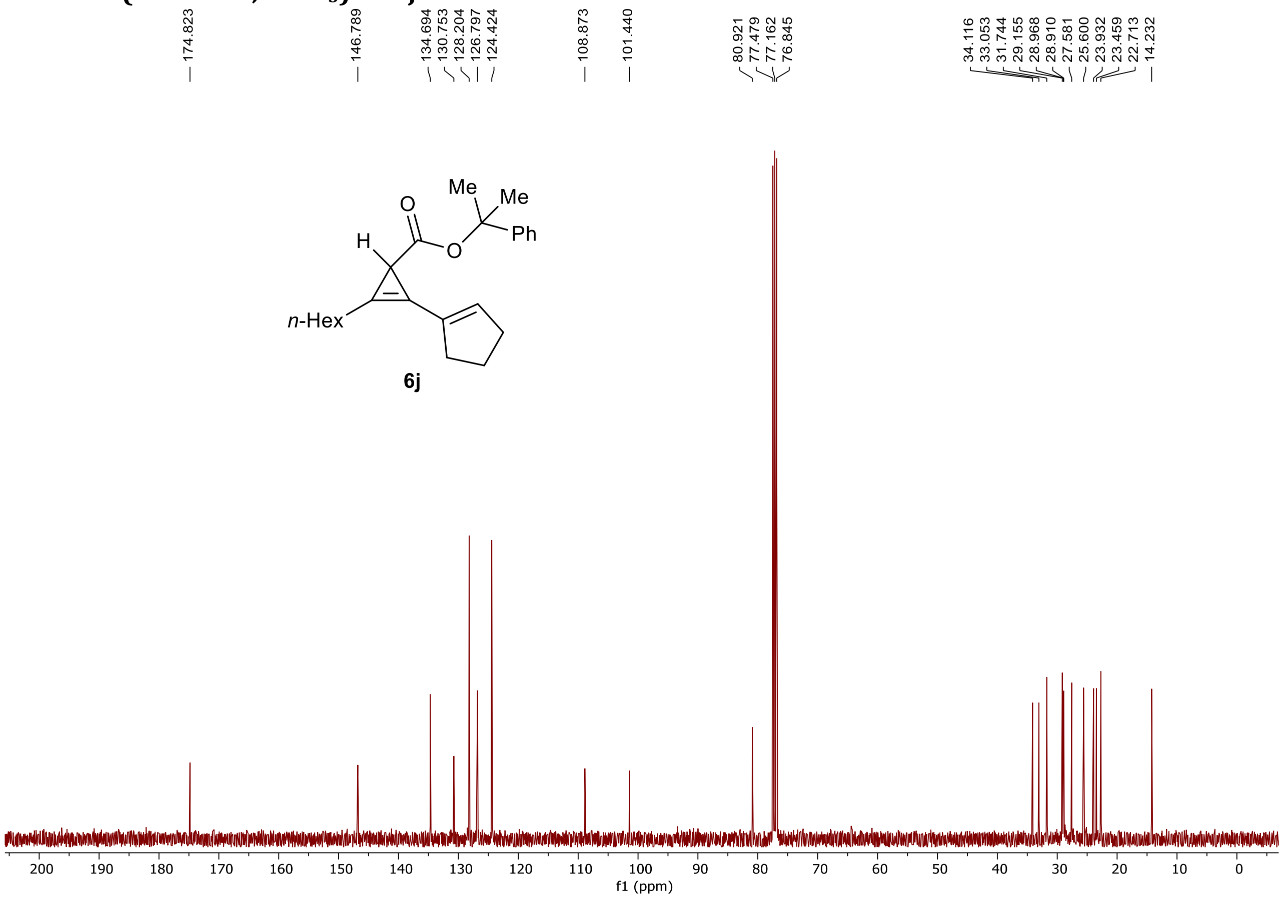

^{1}H NMR (400 MHz, $CDCl_3$) of 6k

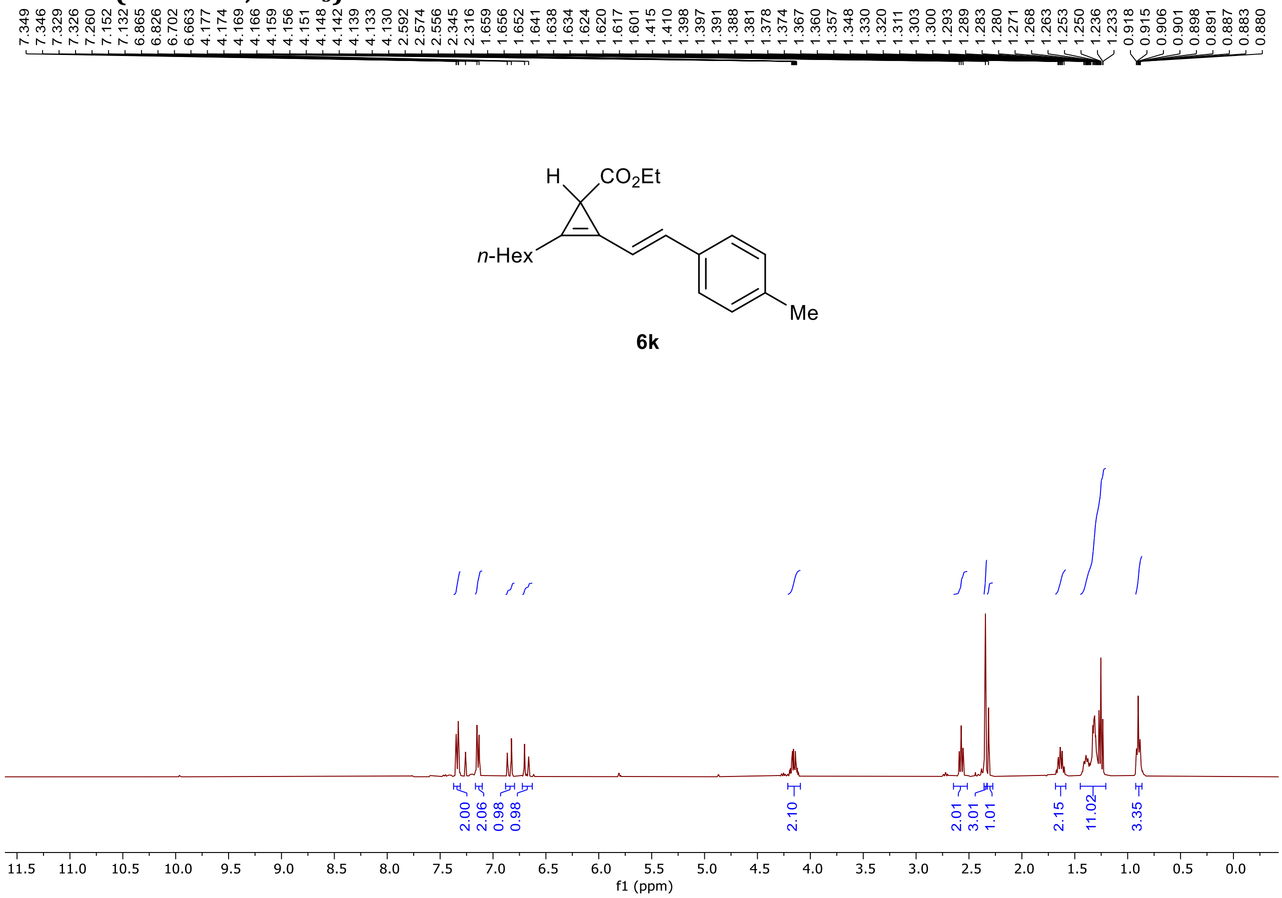

^{13}C NMR (101 MHz, $CDCl_3$) of 6k

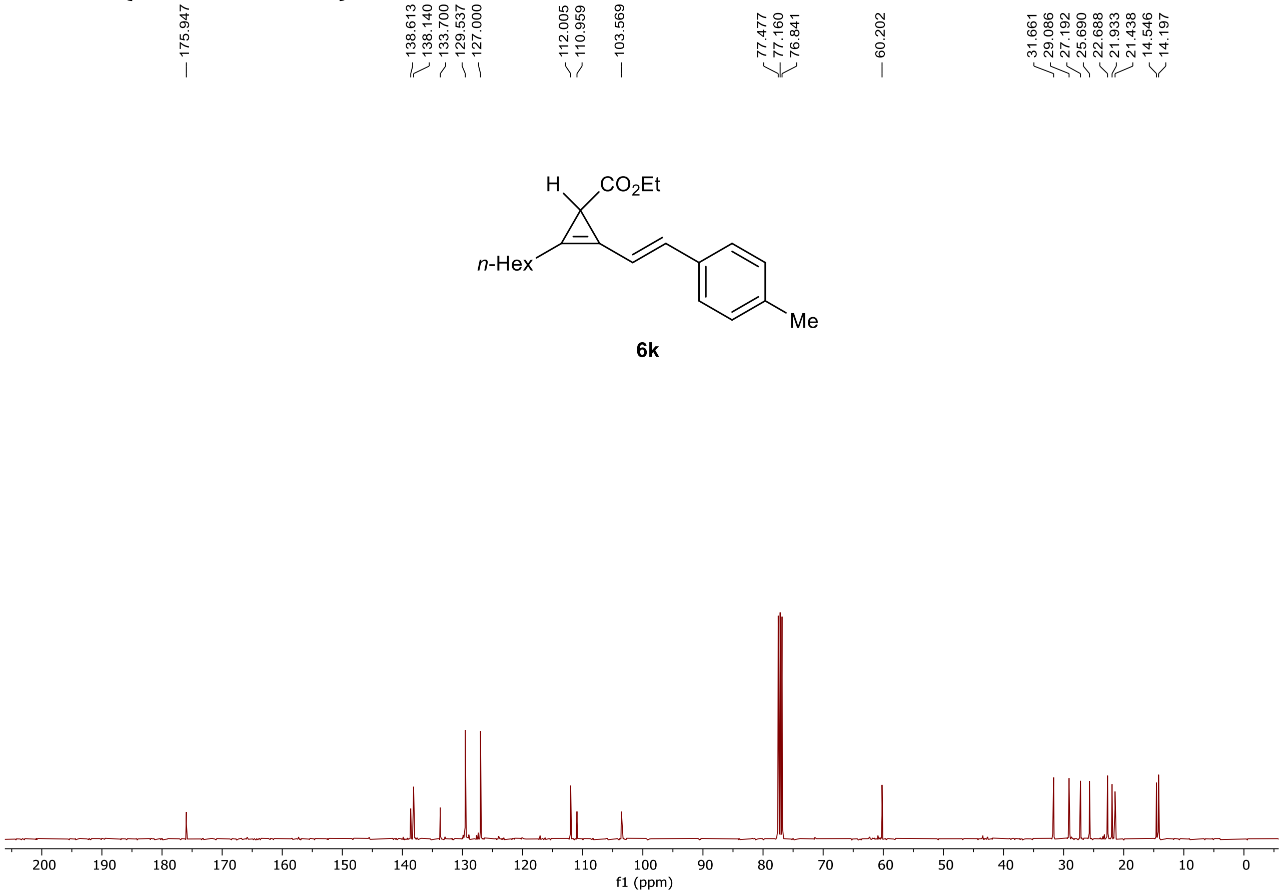

^{1}H NMR (400 MHz, $CDCl_3$) of 6l

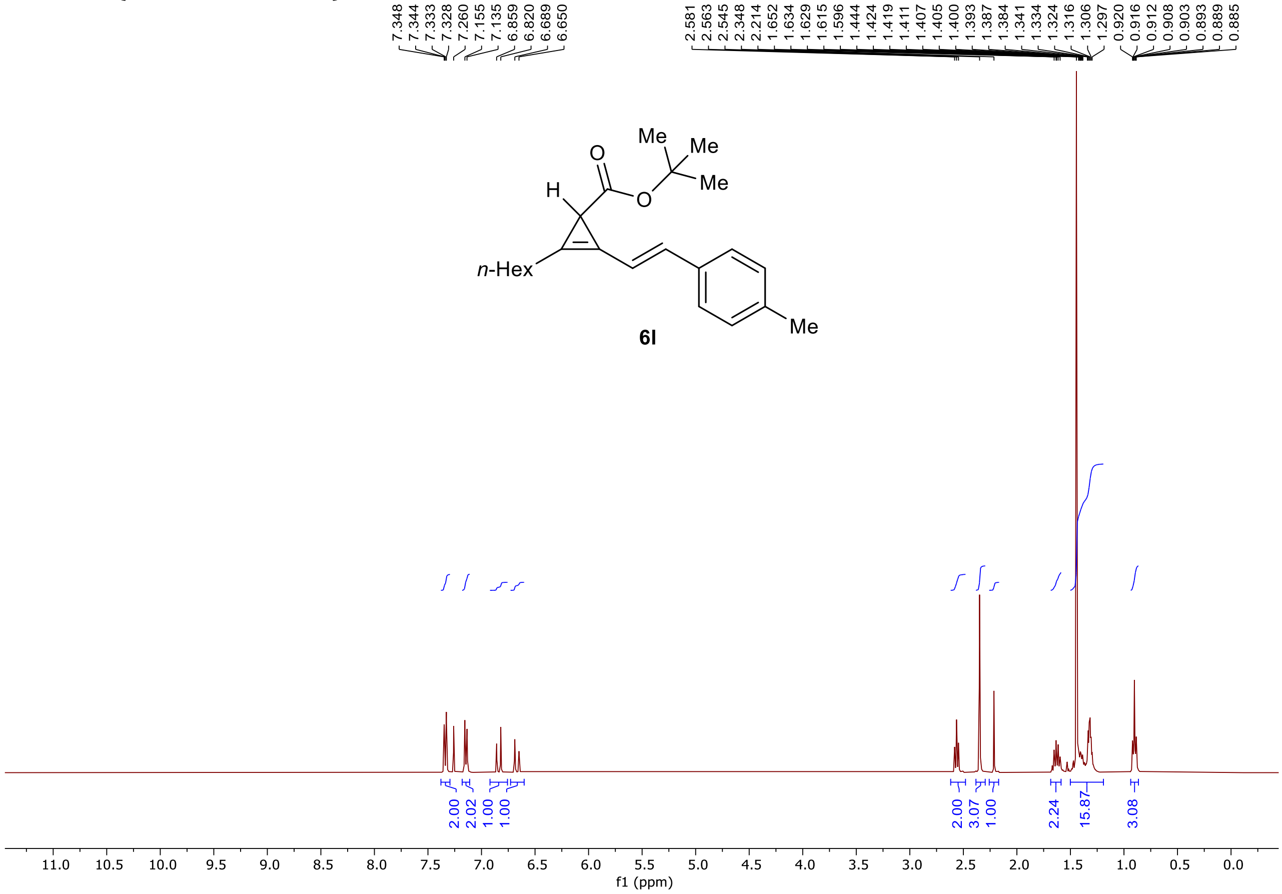

^{13}C NMR (101 MHz, $CDCl_3$) of 6l

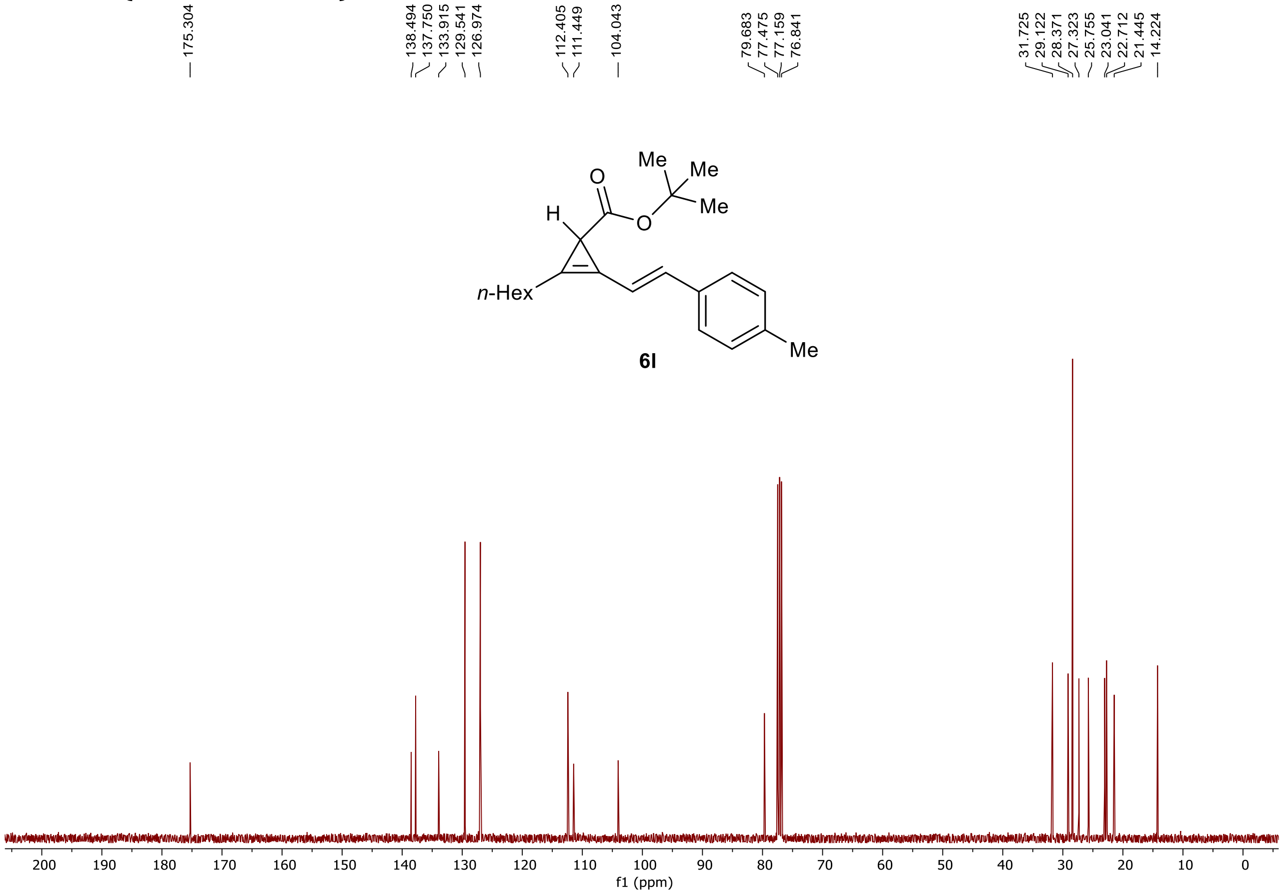

^{1}H NMR (400 MHz, $CDCl_3$) of 6m

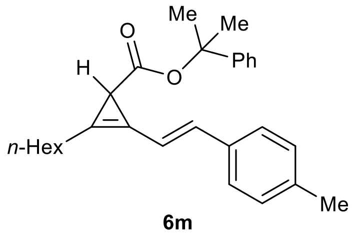

6m

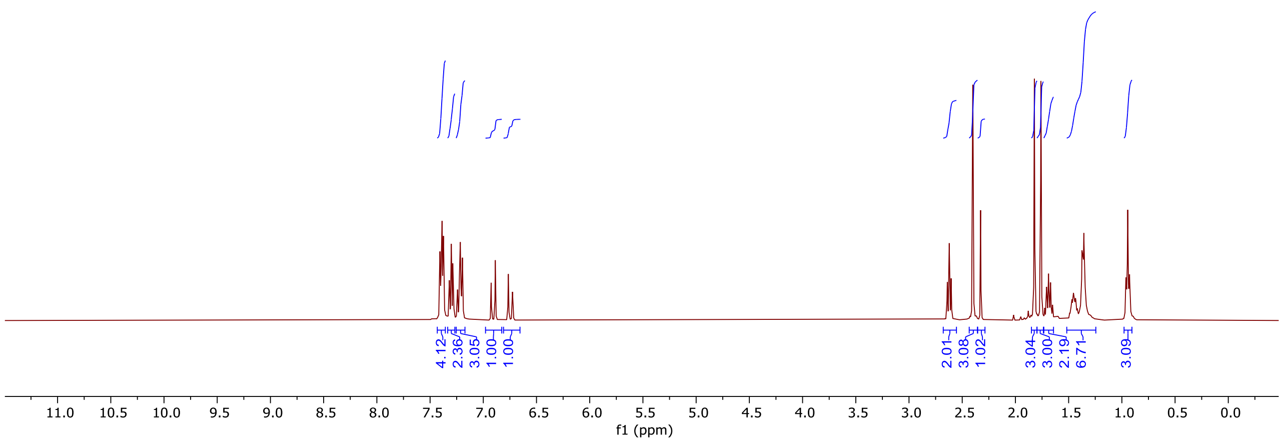

^{13}C NMR (101 MHz, $CDCl_3$) of 6m

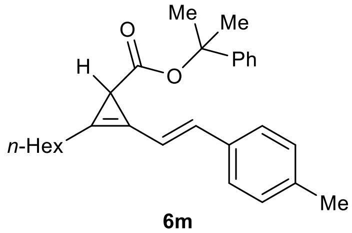

6m

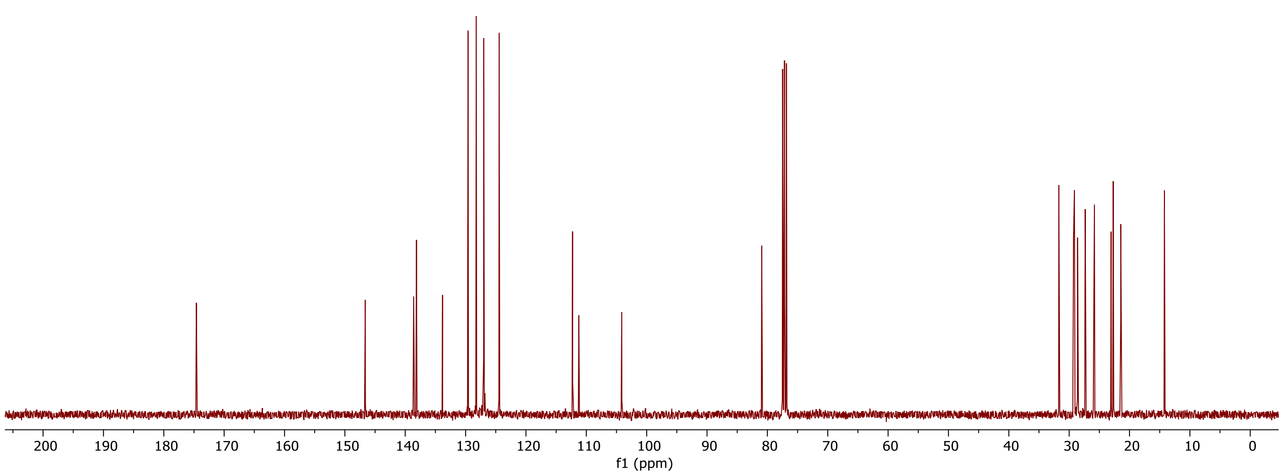

^{1}H NMR (400 MHz, $CDCl_3$) of 6n

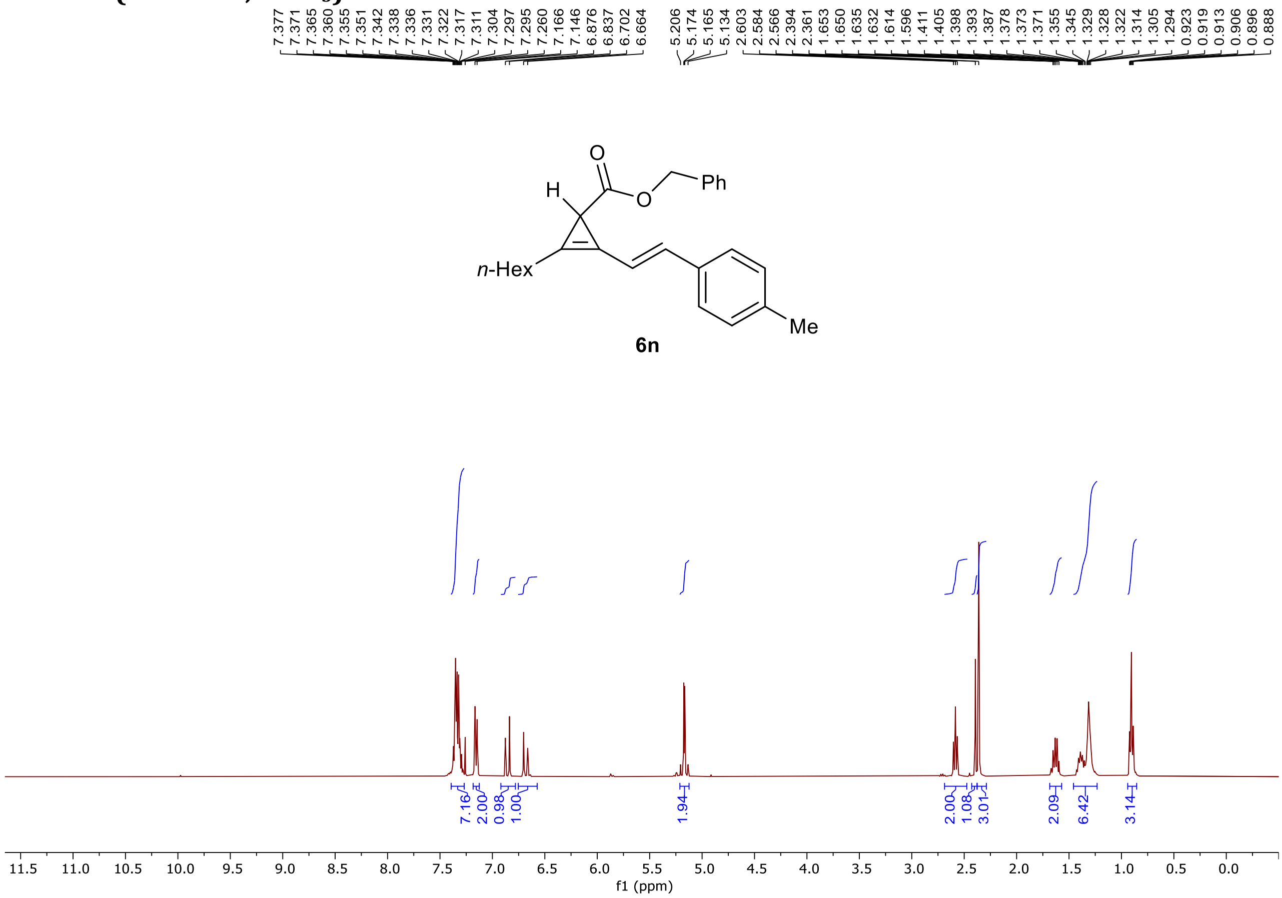

^{13}C NMR (101 MHz, $CDCl_3$) of 6n

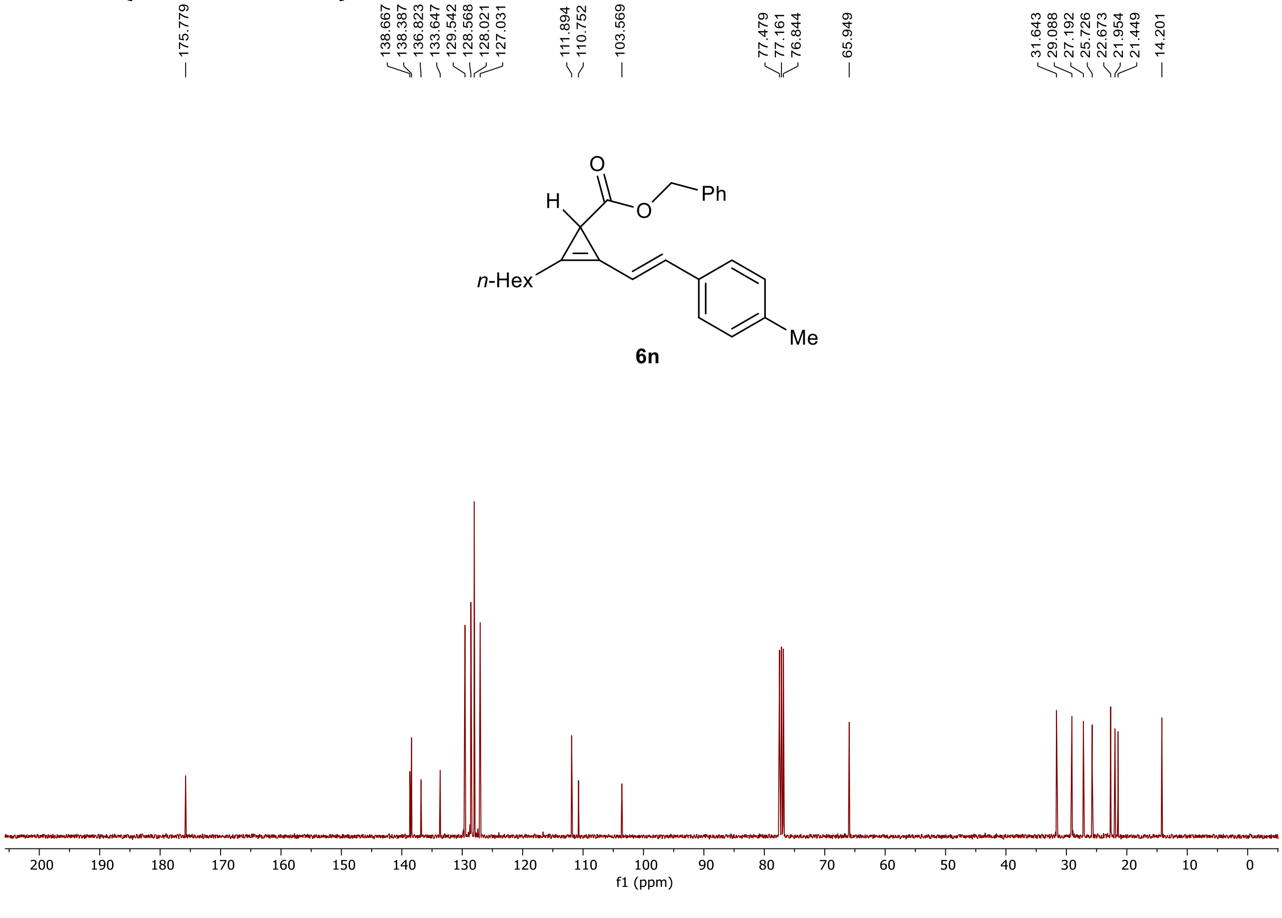

^{1}H NMR (400 MHz, $CDCl_3$) of 6o

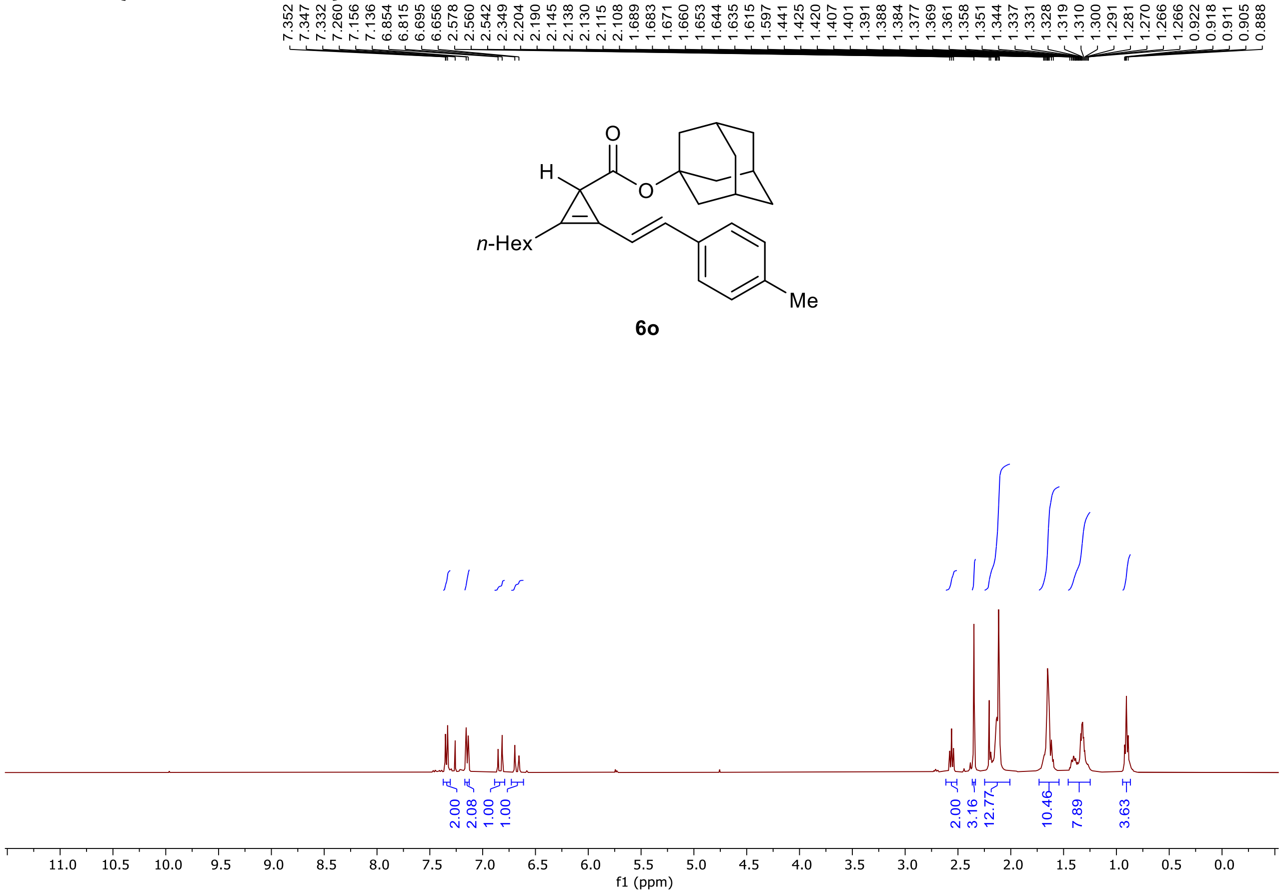

^{13}C NMR (101 MHz, $CDCl_3$) of 6o

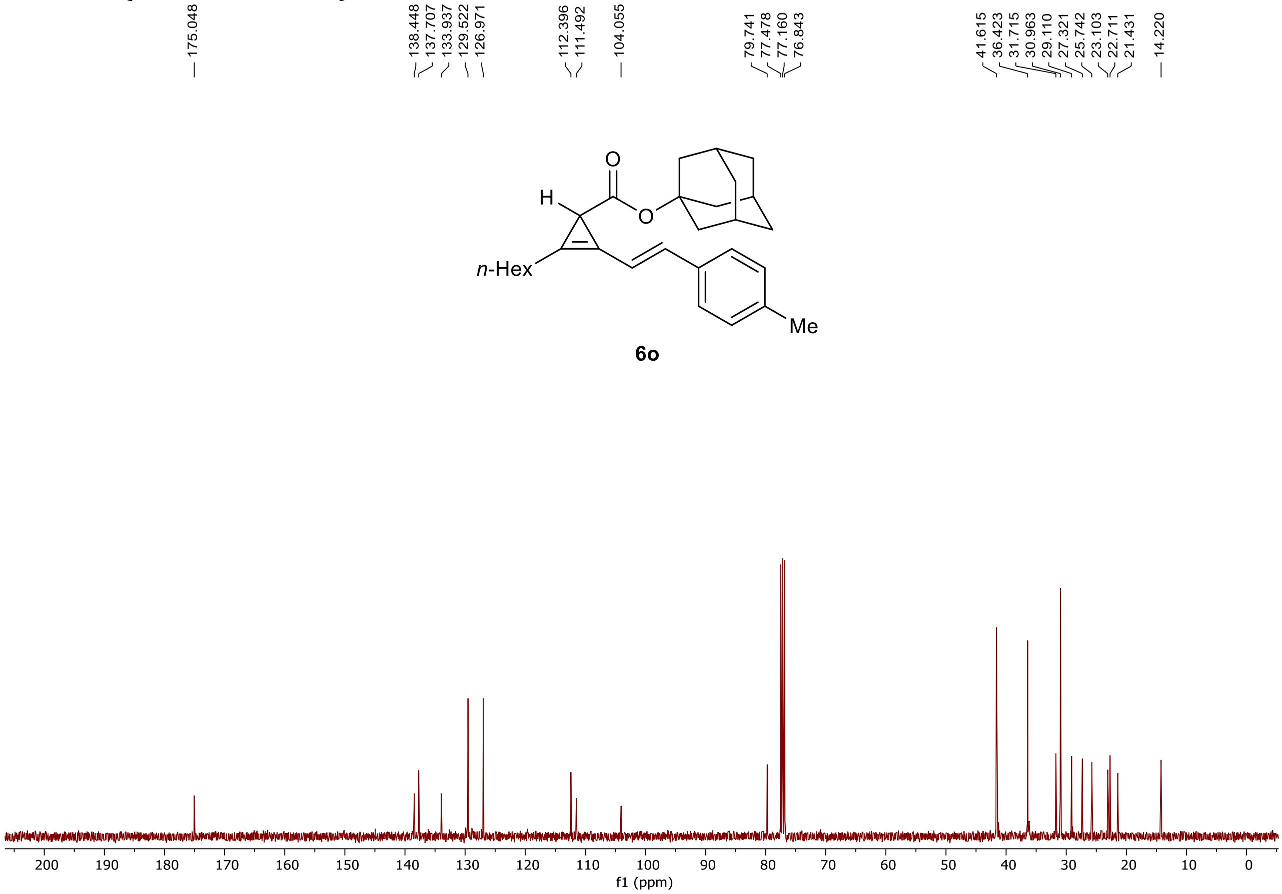

^{1}H NMR (400 MHz, $CDCl_3$) of 7

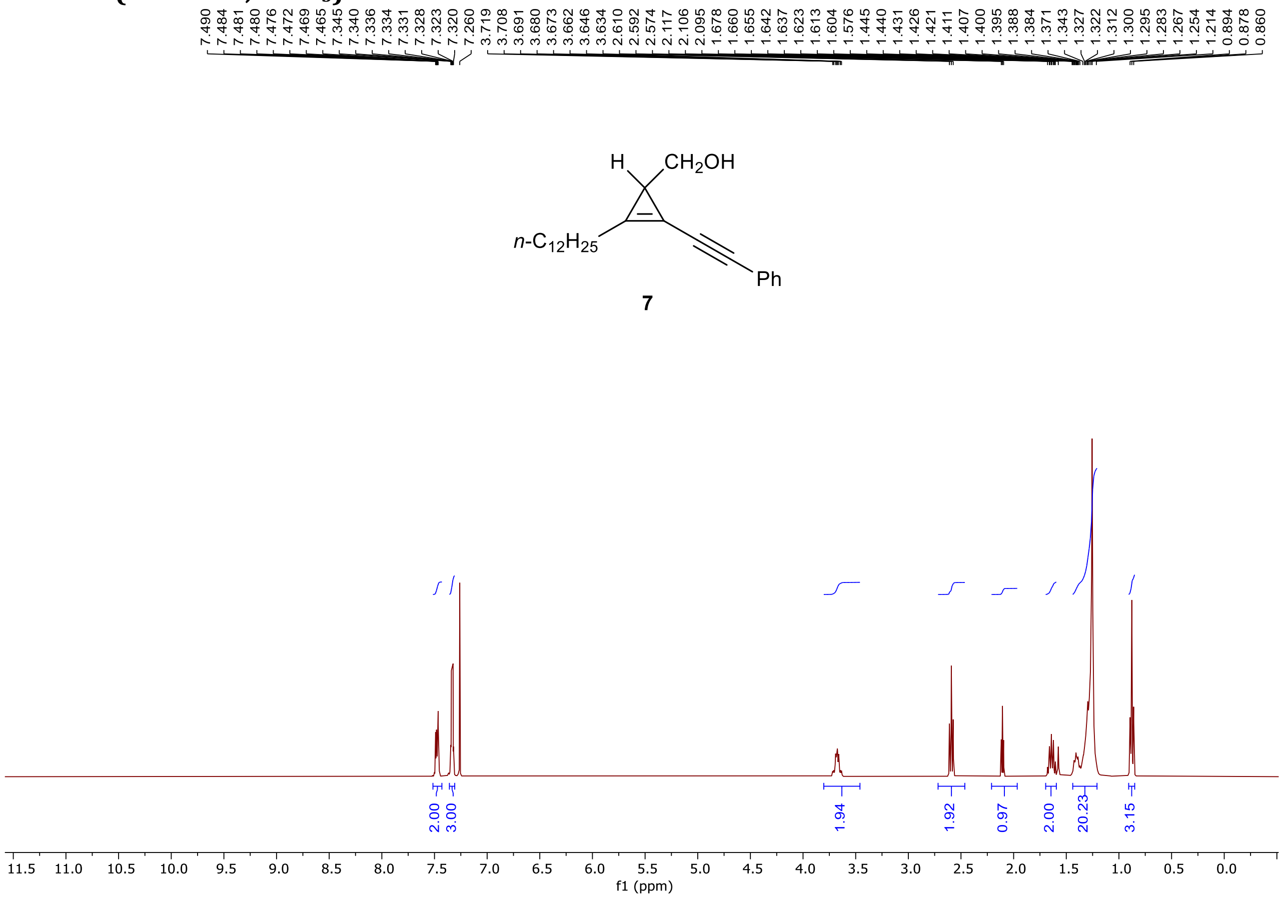

^{13}C NMR (101 MHz, $CDCl_3$) of 7

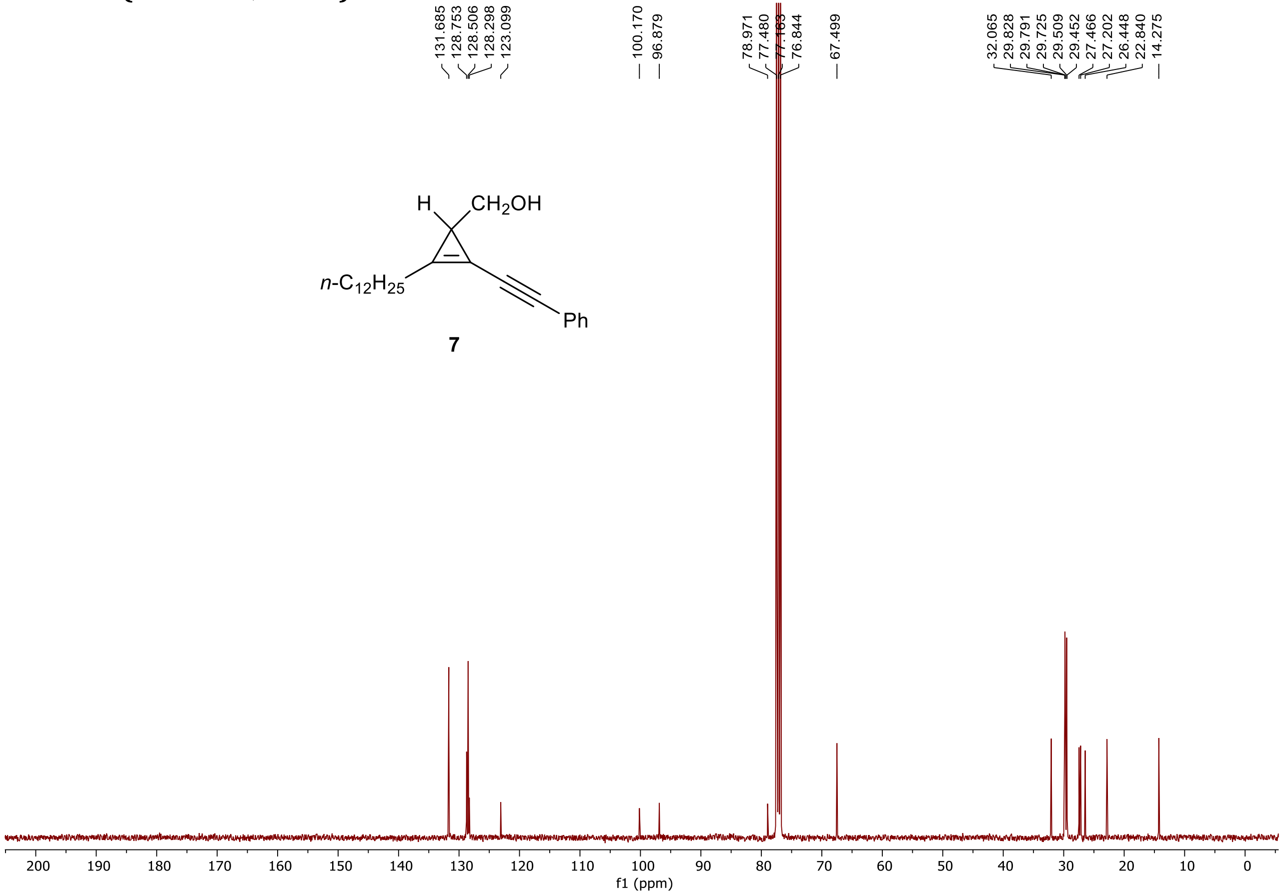

^{1}H NMR (400 MHz, $CDCl_3$) of 8

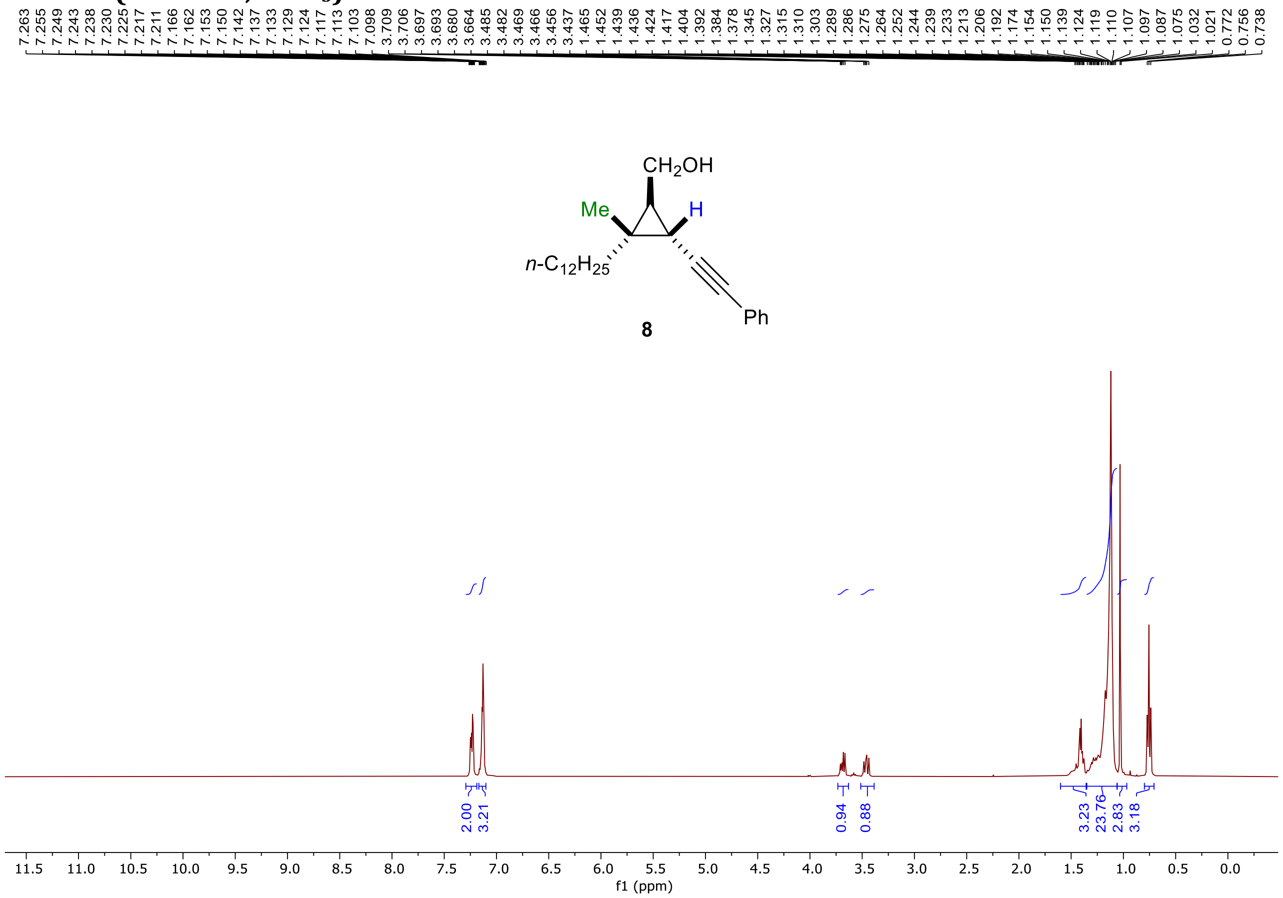

^{13}C NMR (101 MHz, $CDCl_3$) of 8

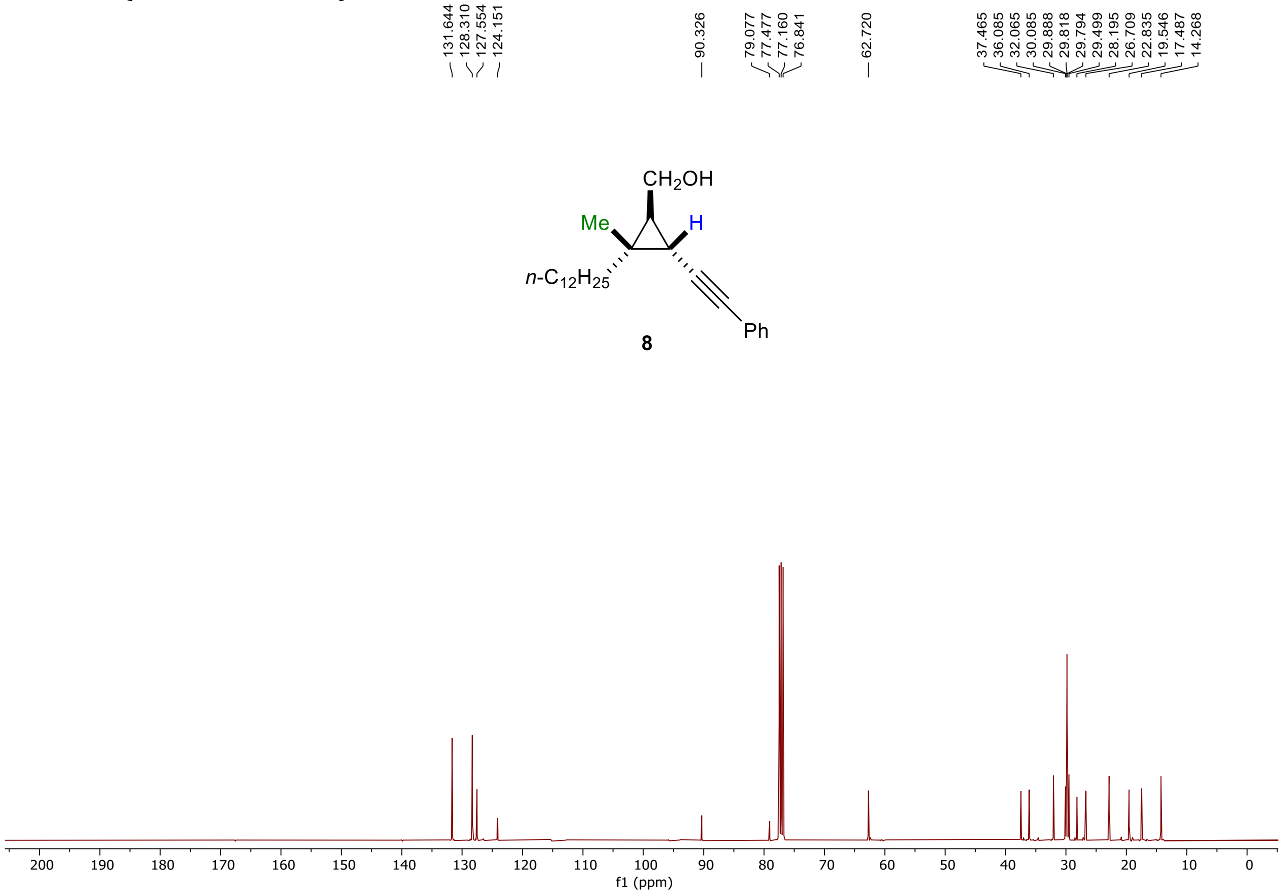

^{1}H NMR (400 MHz, $CDCl_3$) of 9

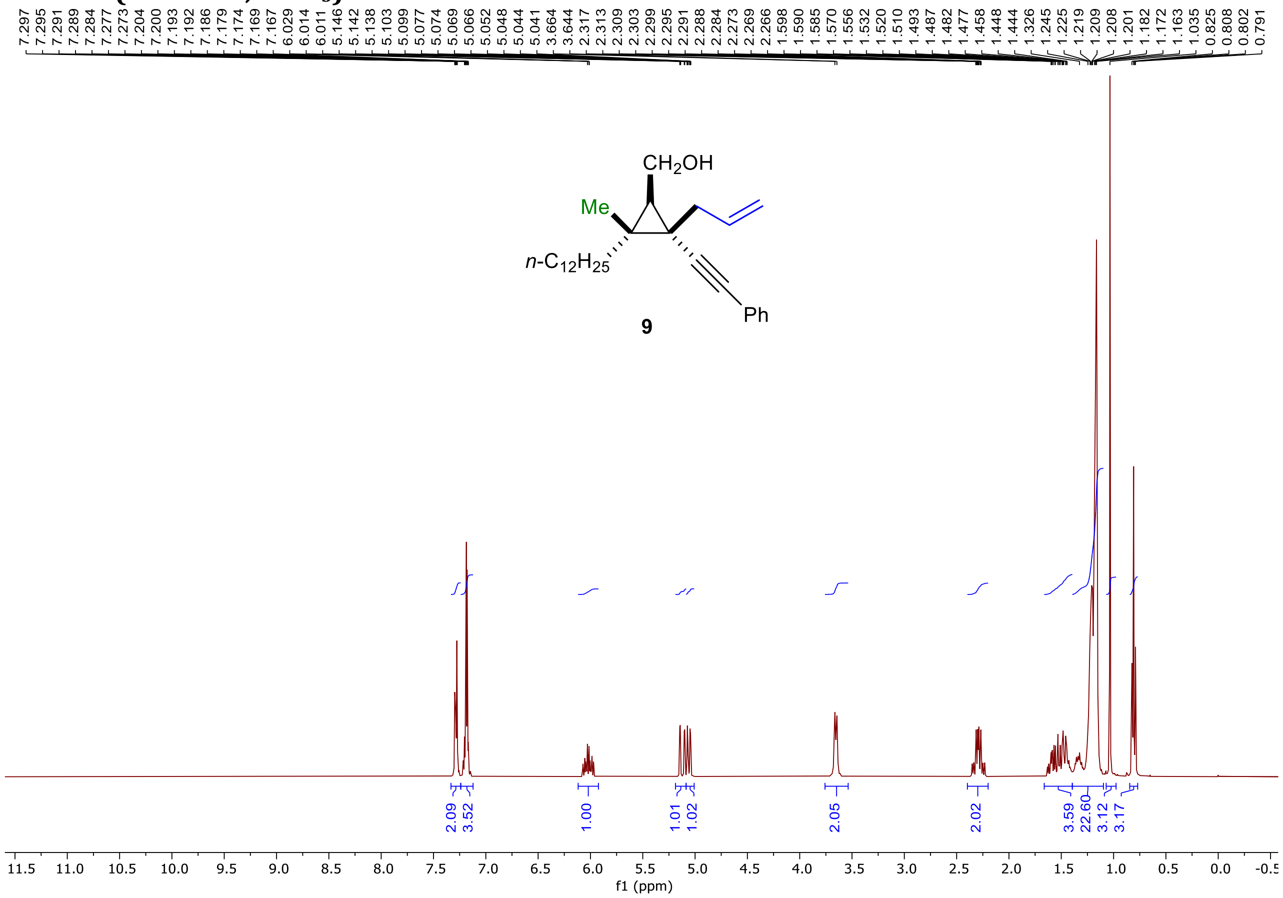

^{13}C NMR (101 MHz, $CDCl_3$) of 9

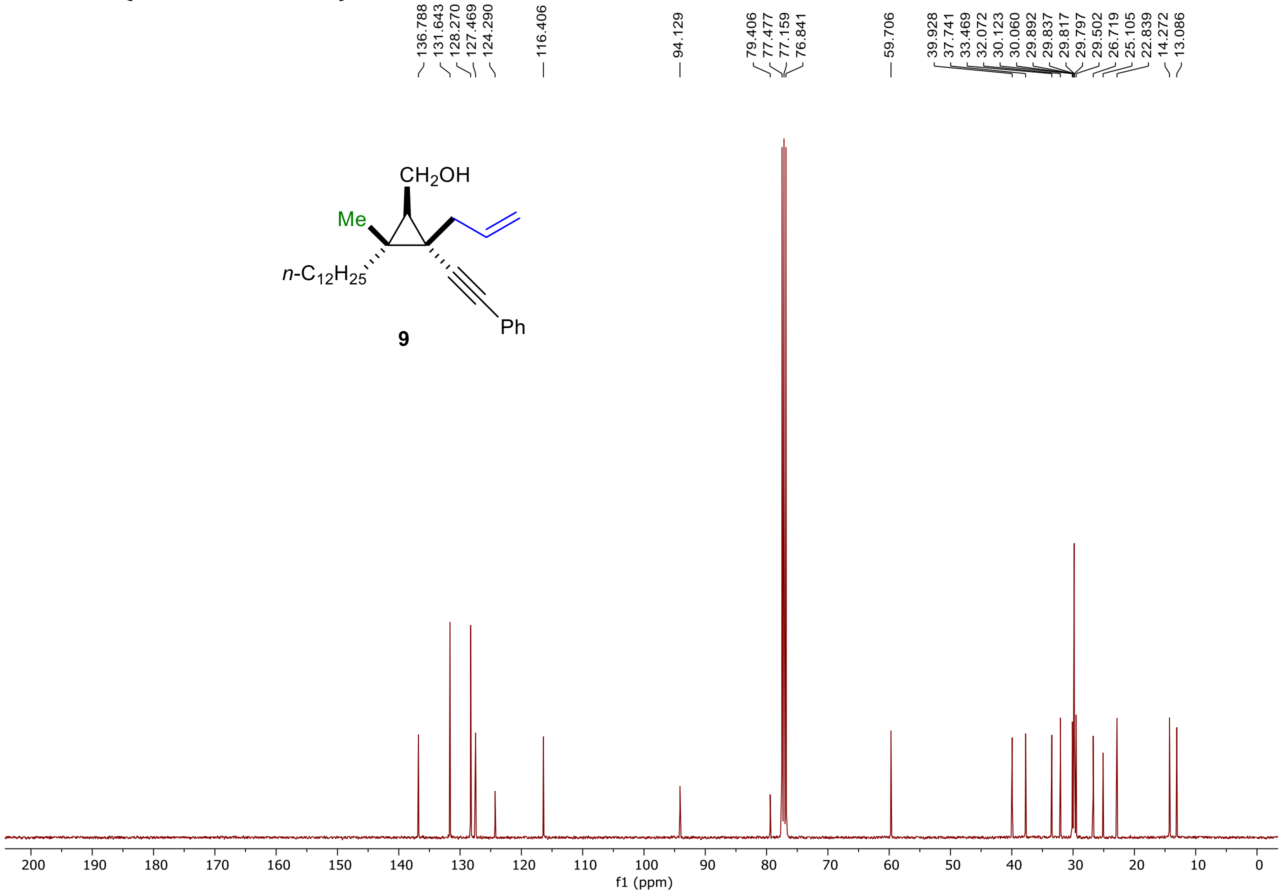

^{1}H NMR (400 MHz, $CDCl_3$) of 10

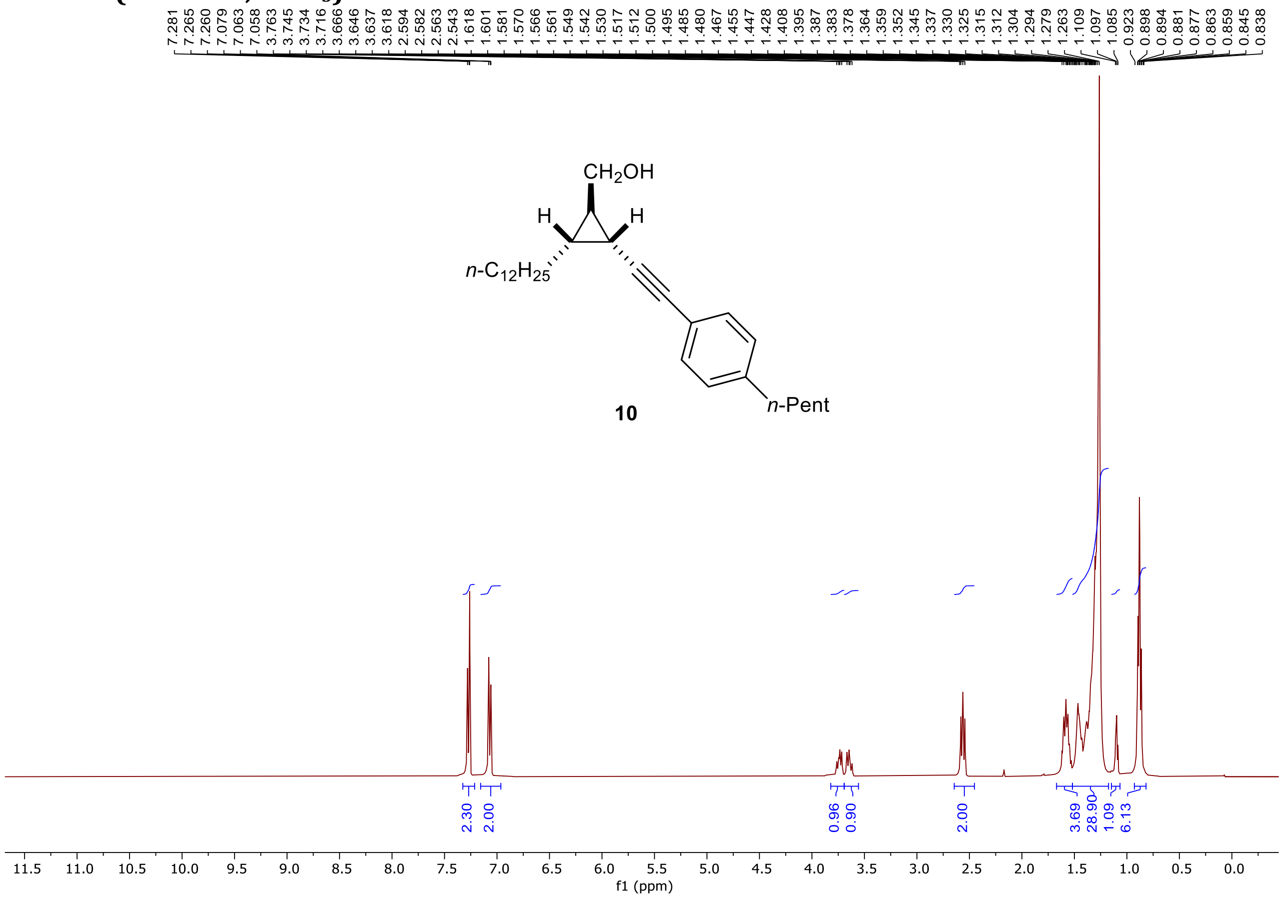

^{13}C NMR (101 MHz, $CDCl_3$) of 10

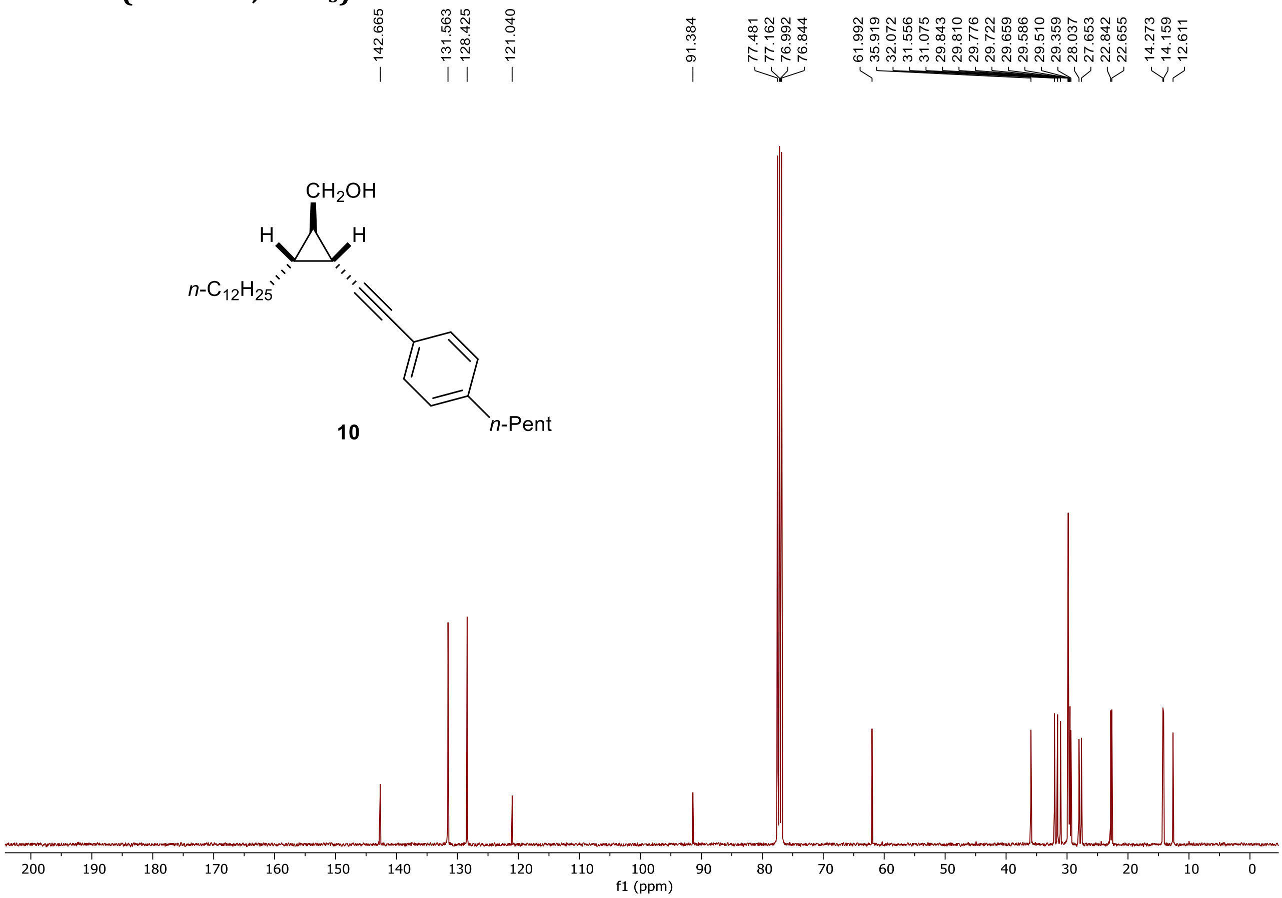

^{1}H NMR (400 MHz, $CDCl_3$) of 11

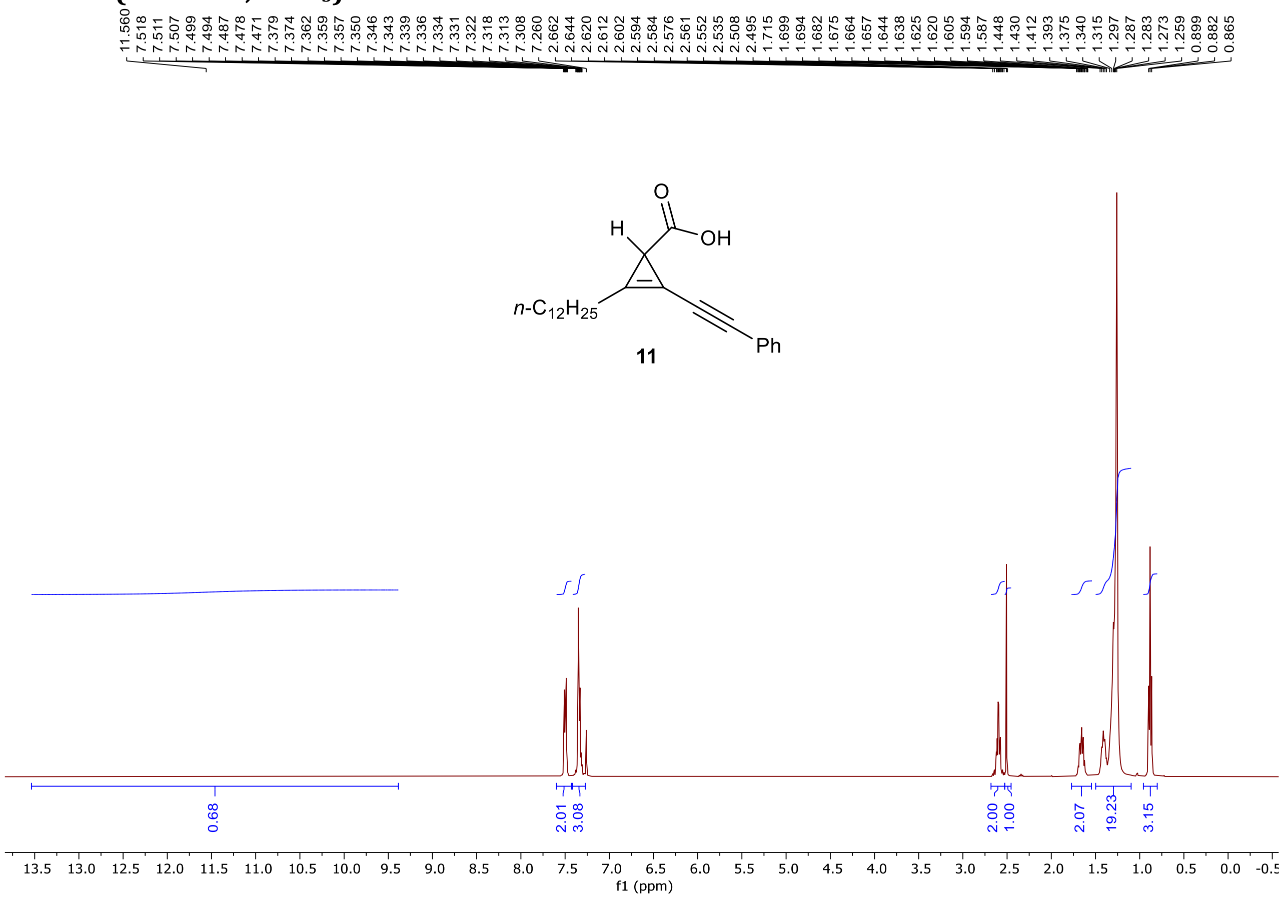

^{13}C NMR (101 MHz, $CDCl_3$) of 11

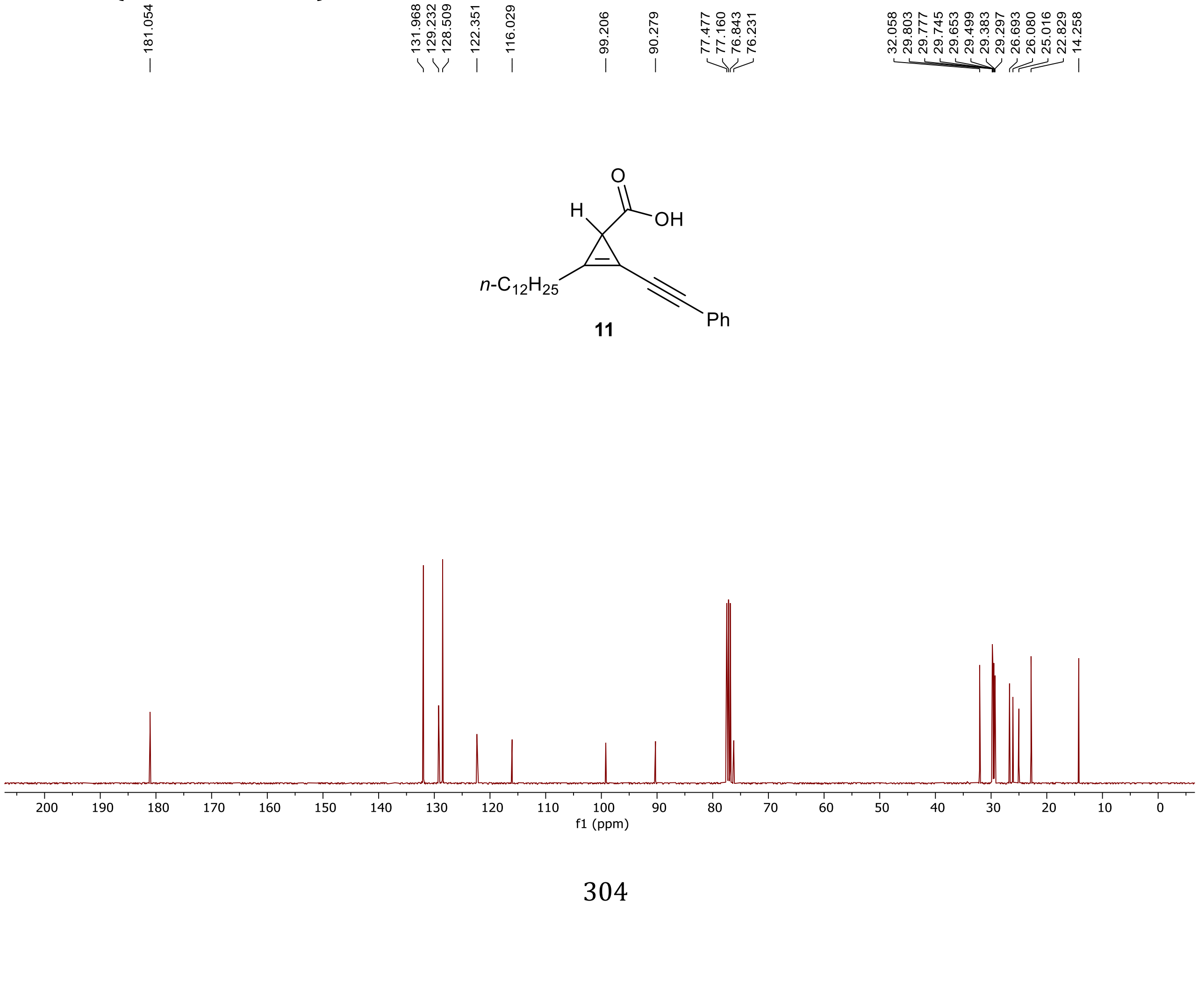

^{1}H NMR (400 MHz, $CDCl_3$) of 12

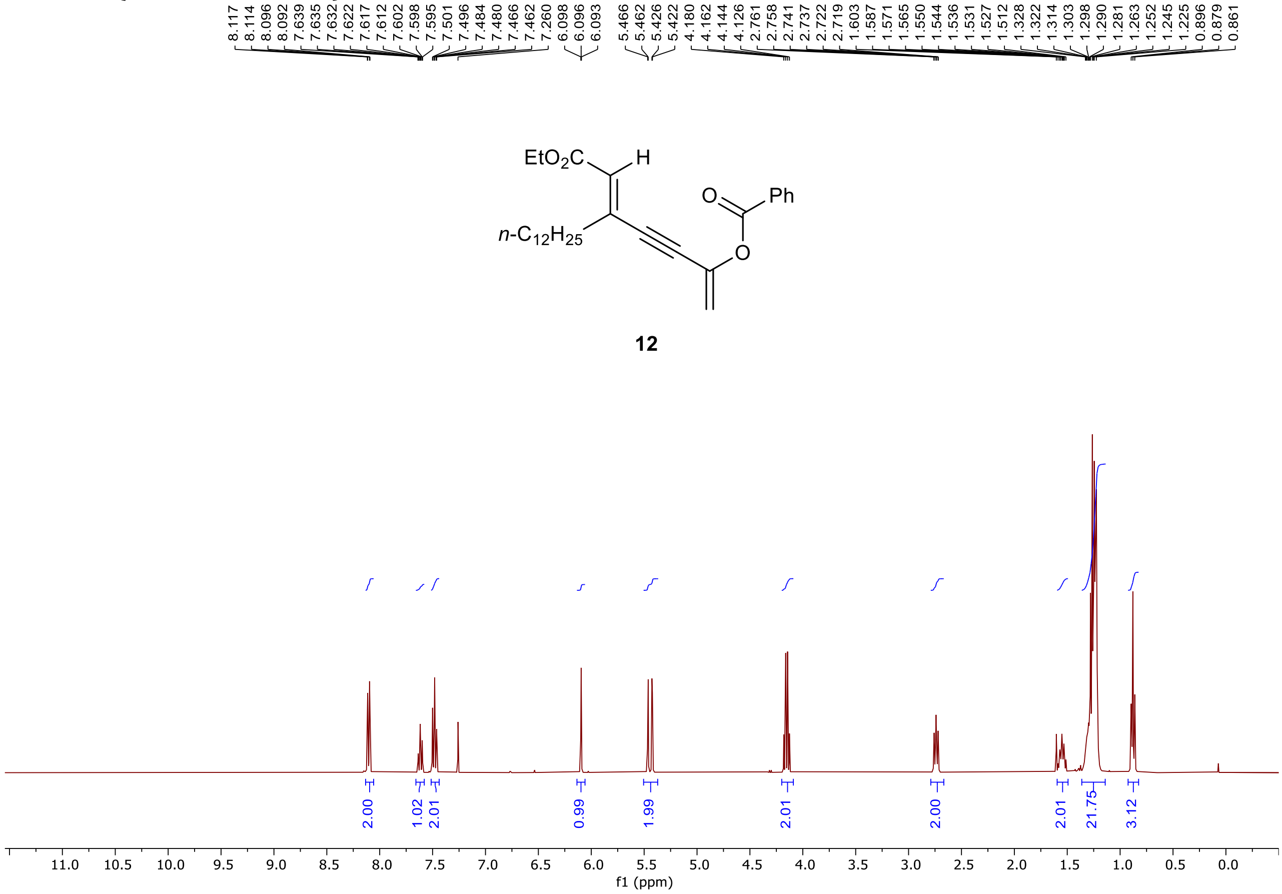

^{13}C NMR (101 MHz, $CDCl_3$) of 12

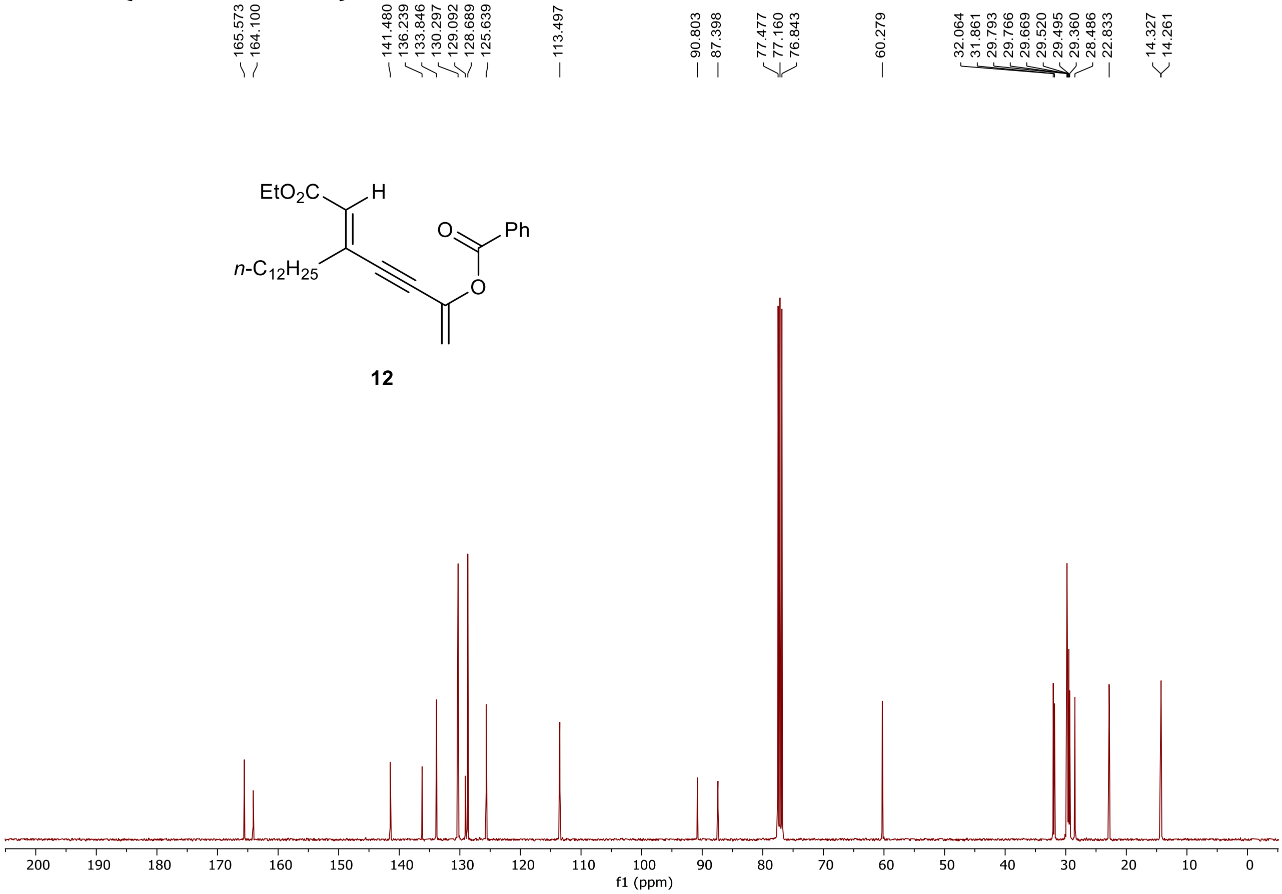

^{1}H NMR (400 MHz, $CDCl_3$) of 13

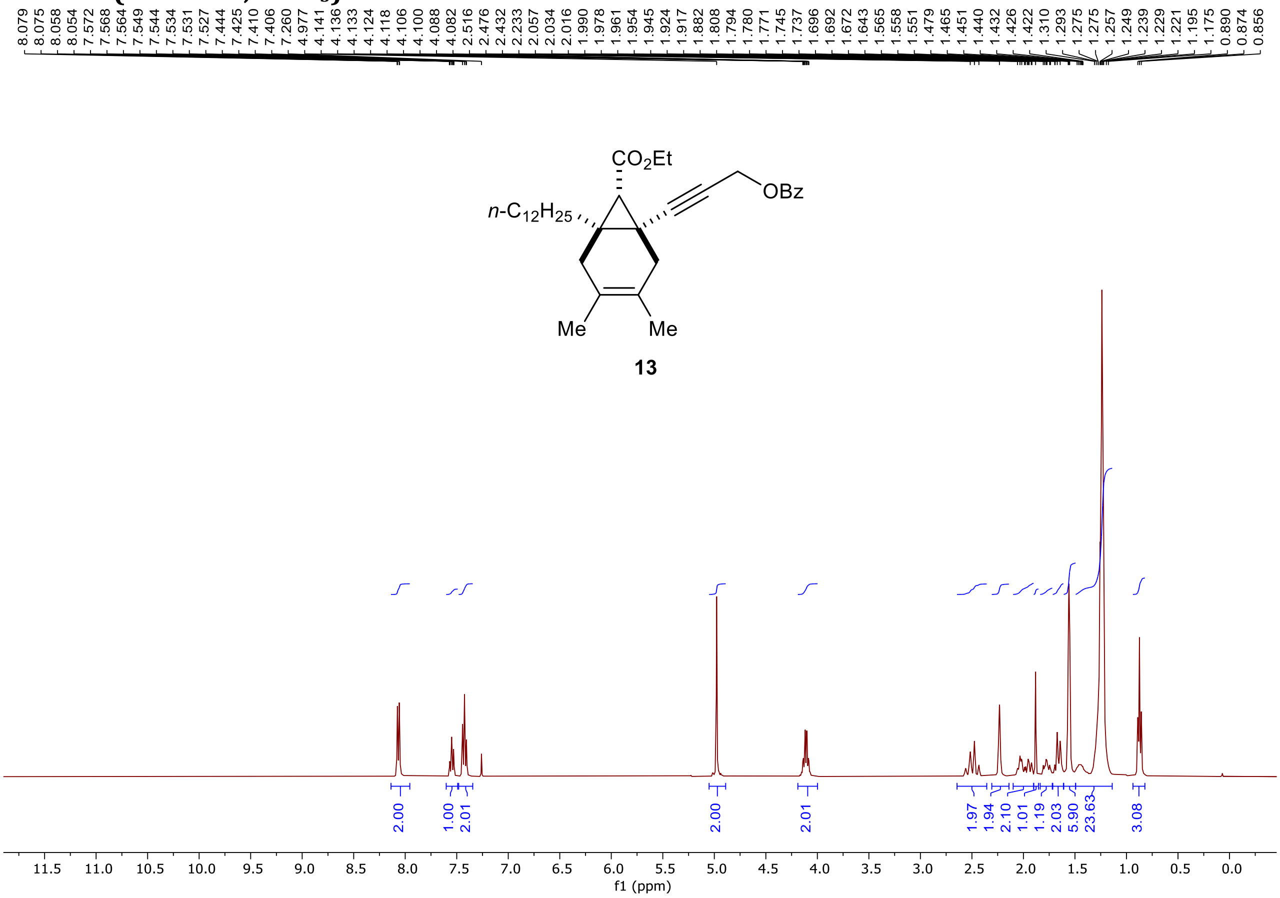

^{13}C NMR (101 MHz, $CDCl_3$) of 13

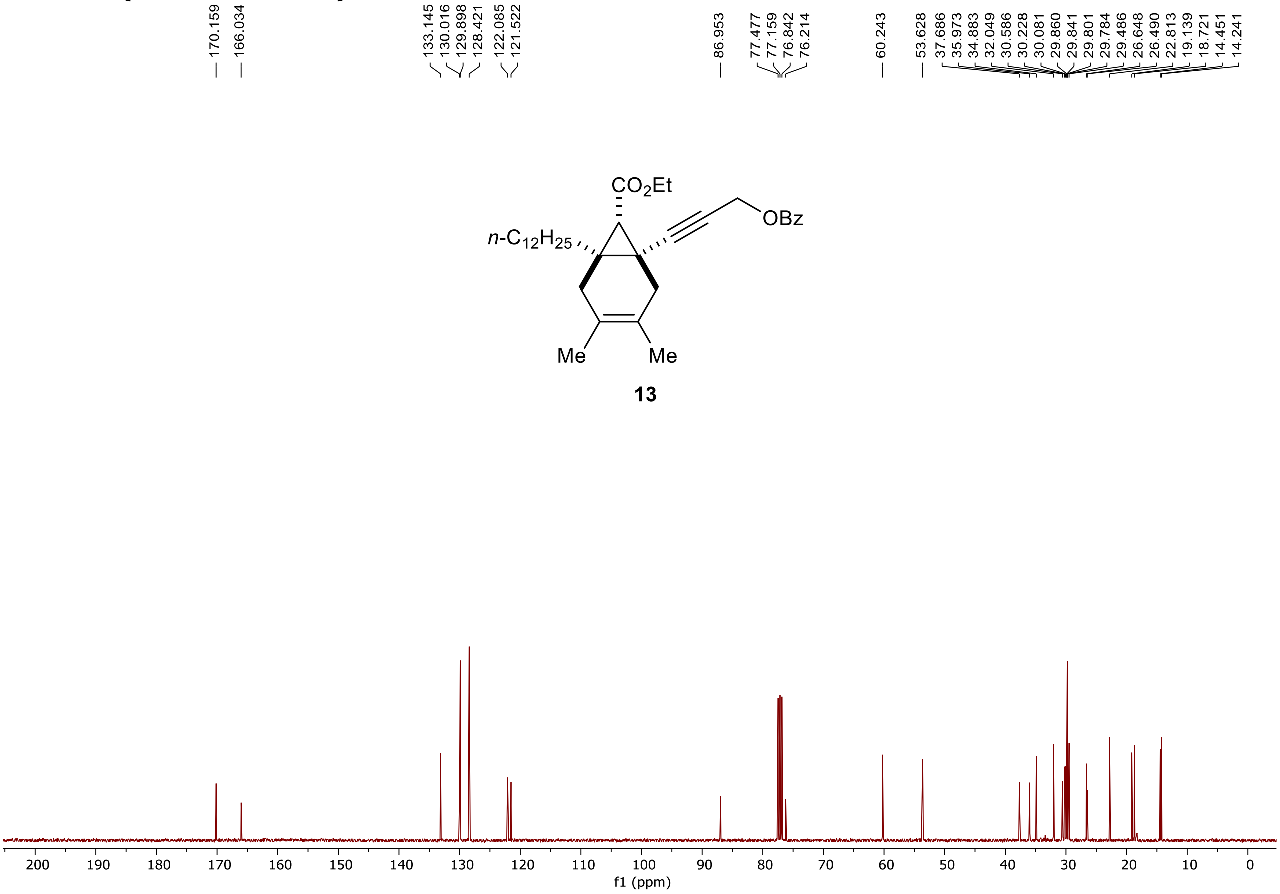

^{1}H NMR (400 MHz, $CDCl_3$) of 14

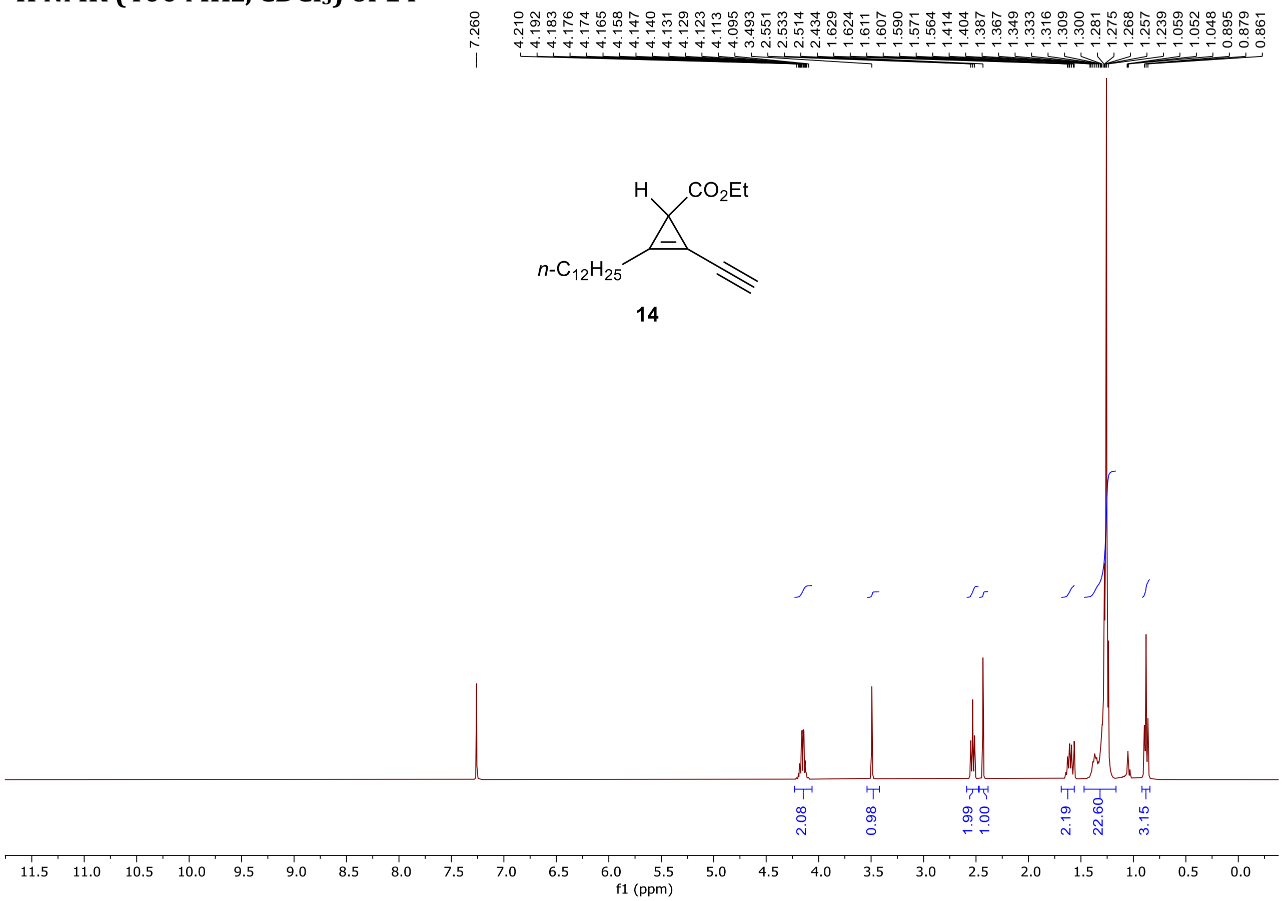

^{13}C NMR (101 MHz, $CDCl_3$) of 14

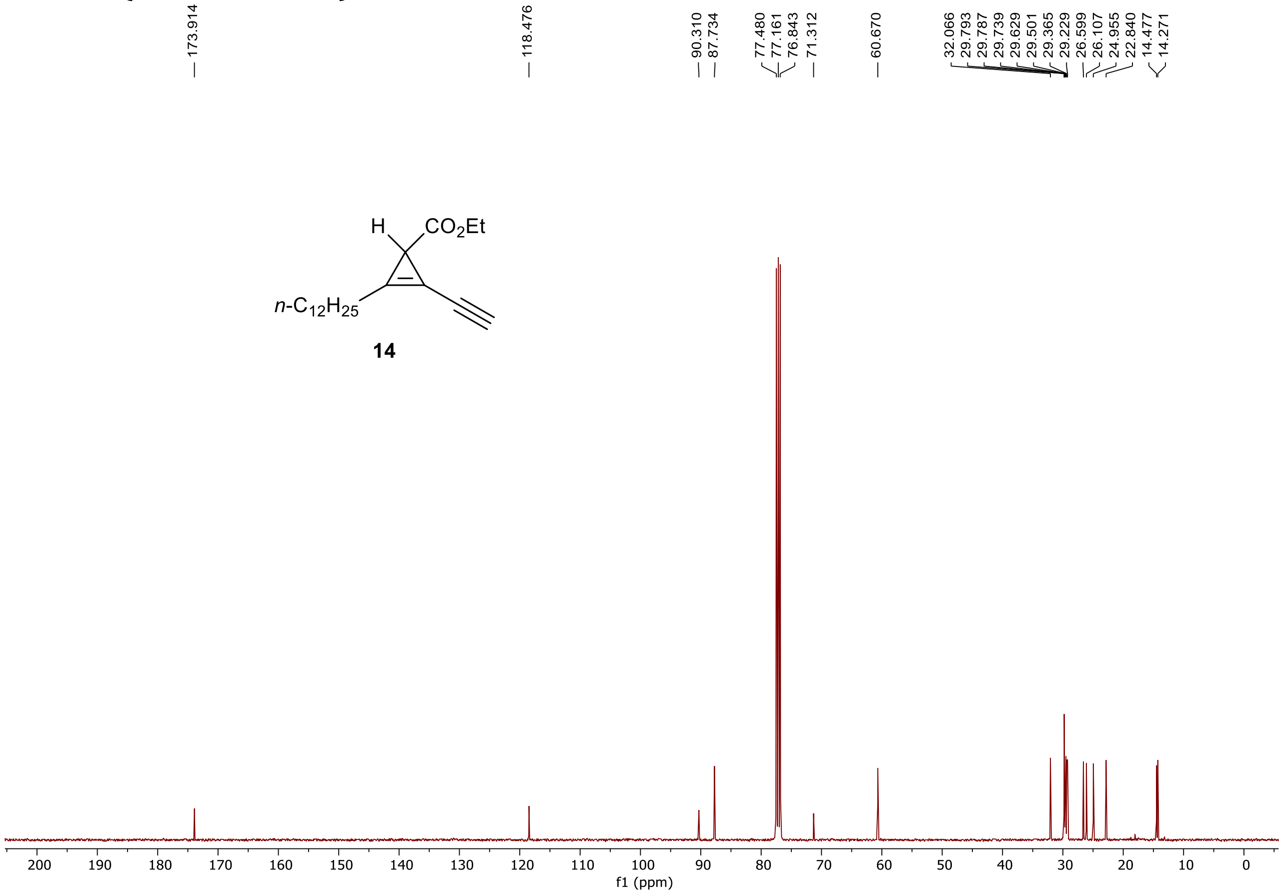

^{1}H NMR (400 MHz, $CDCl_3$) of 15

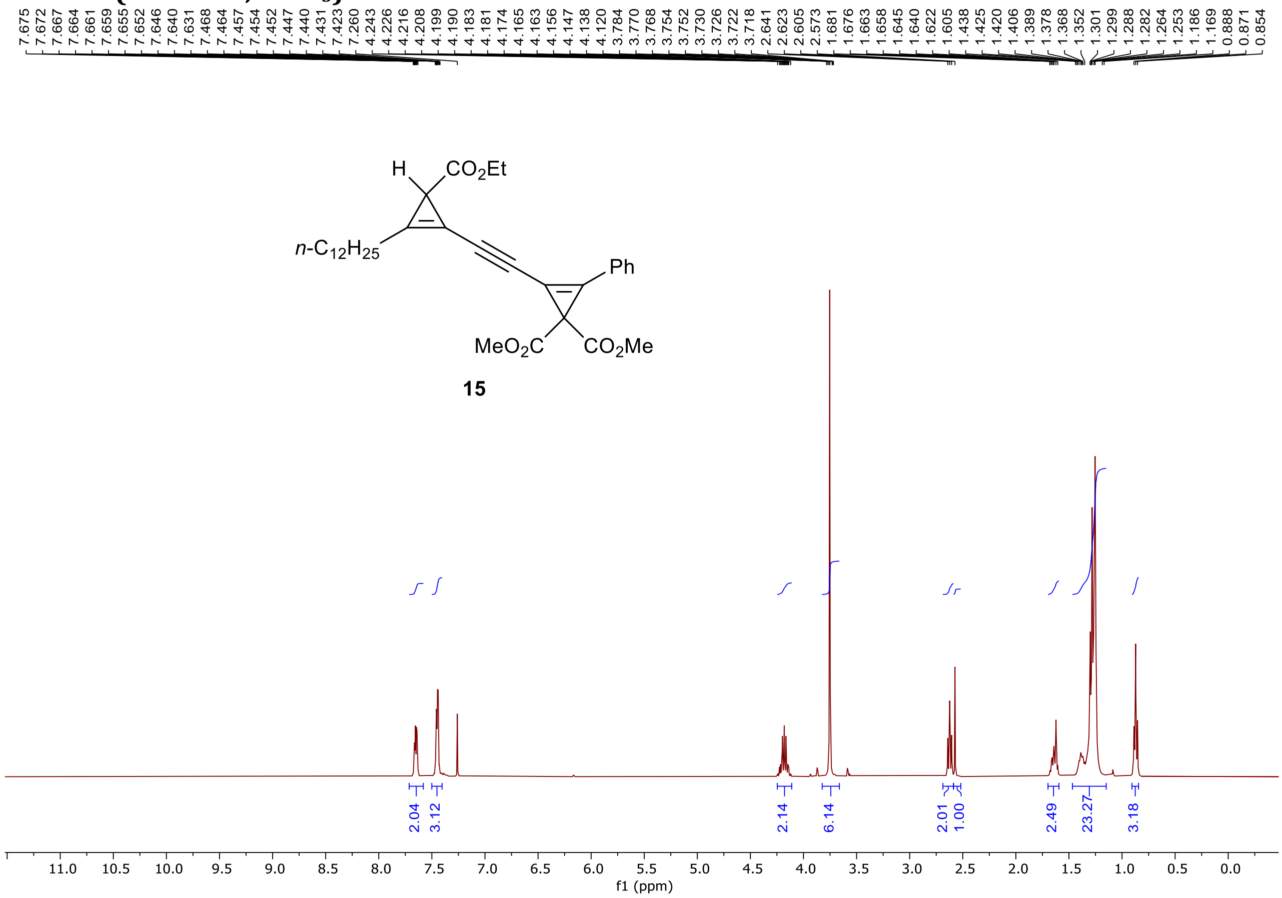

^{13}C NMR (101 MHz, $CDCl_3$) of 15

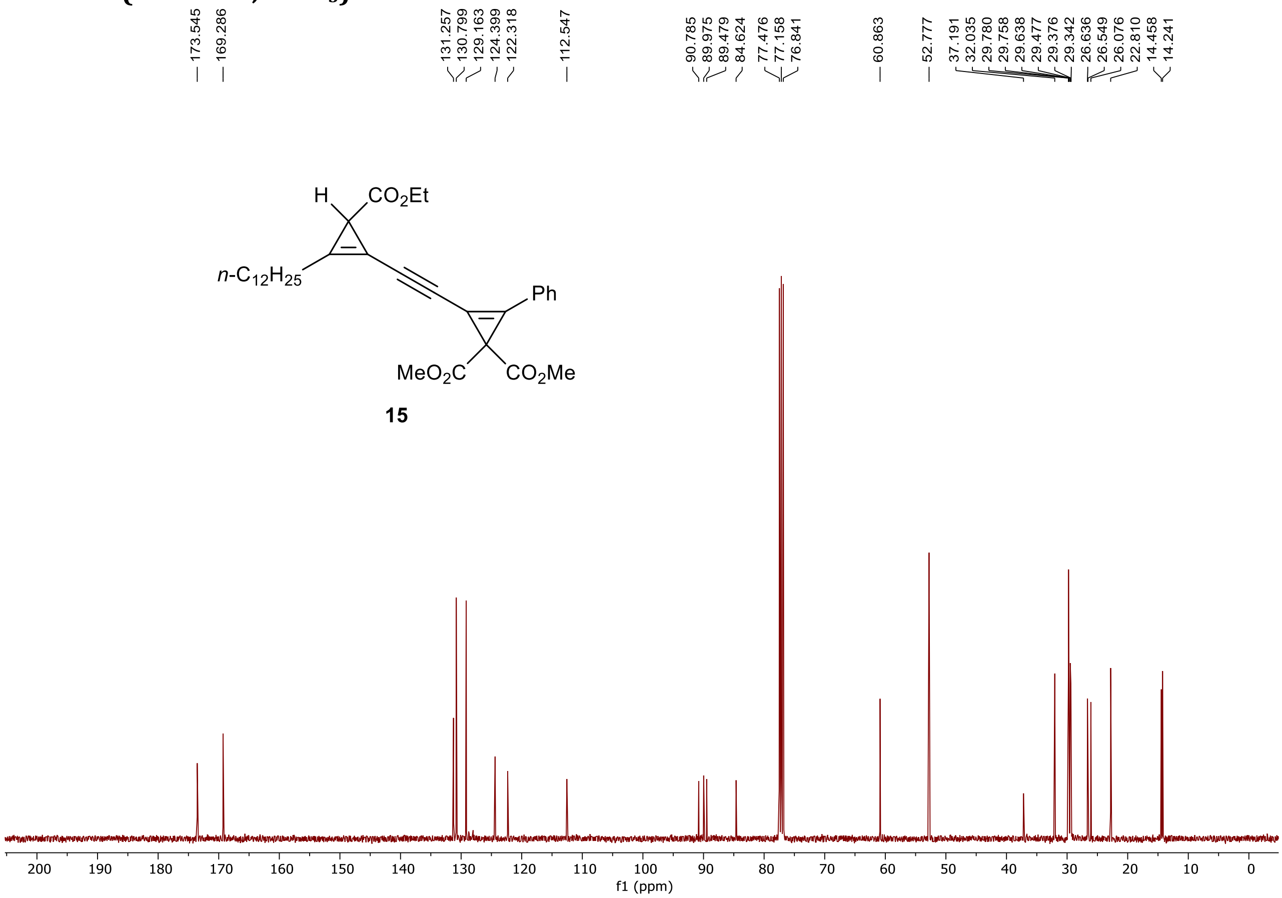

^{1}H NMR (400 MHz, $CDCl_3$) of 16

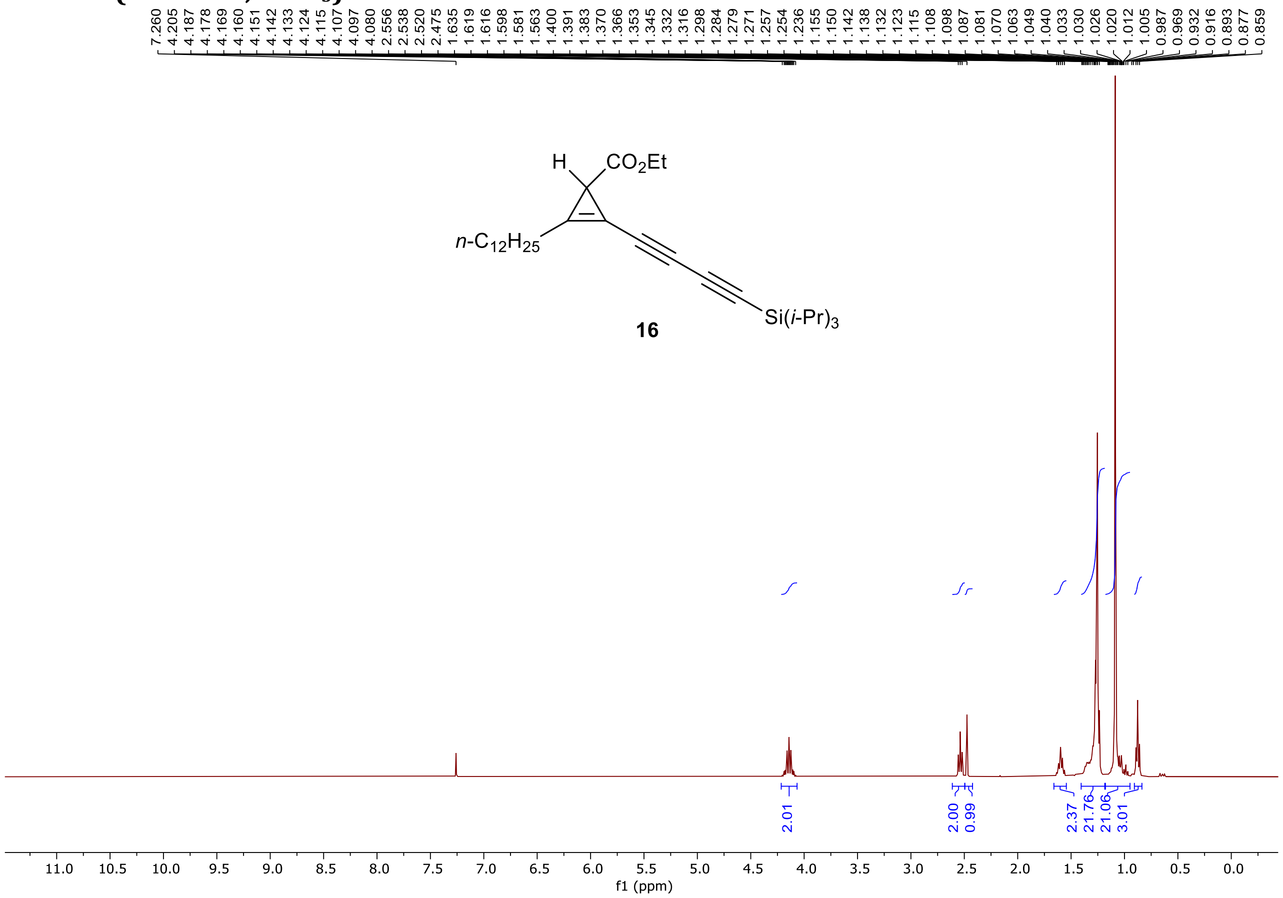

^{13}C NMR (101 MHz, $CDCl_3$) of 16

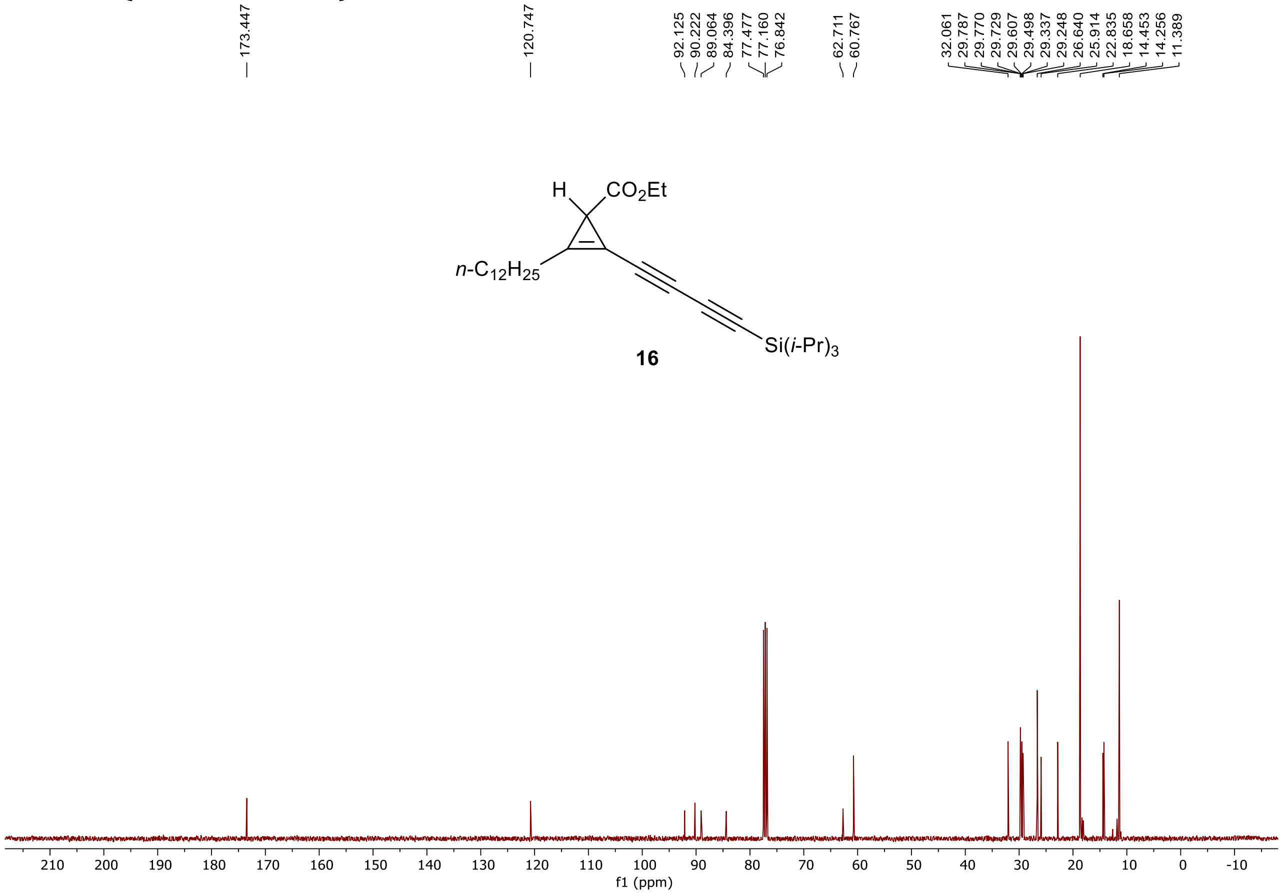

^{1}H NMR (400 MHz, $CDCl_3$) of 17

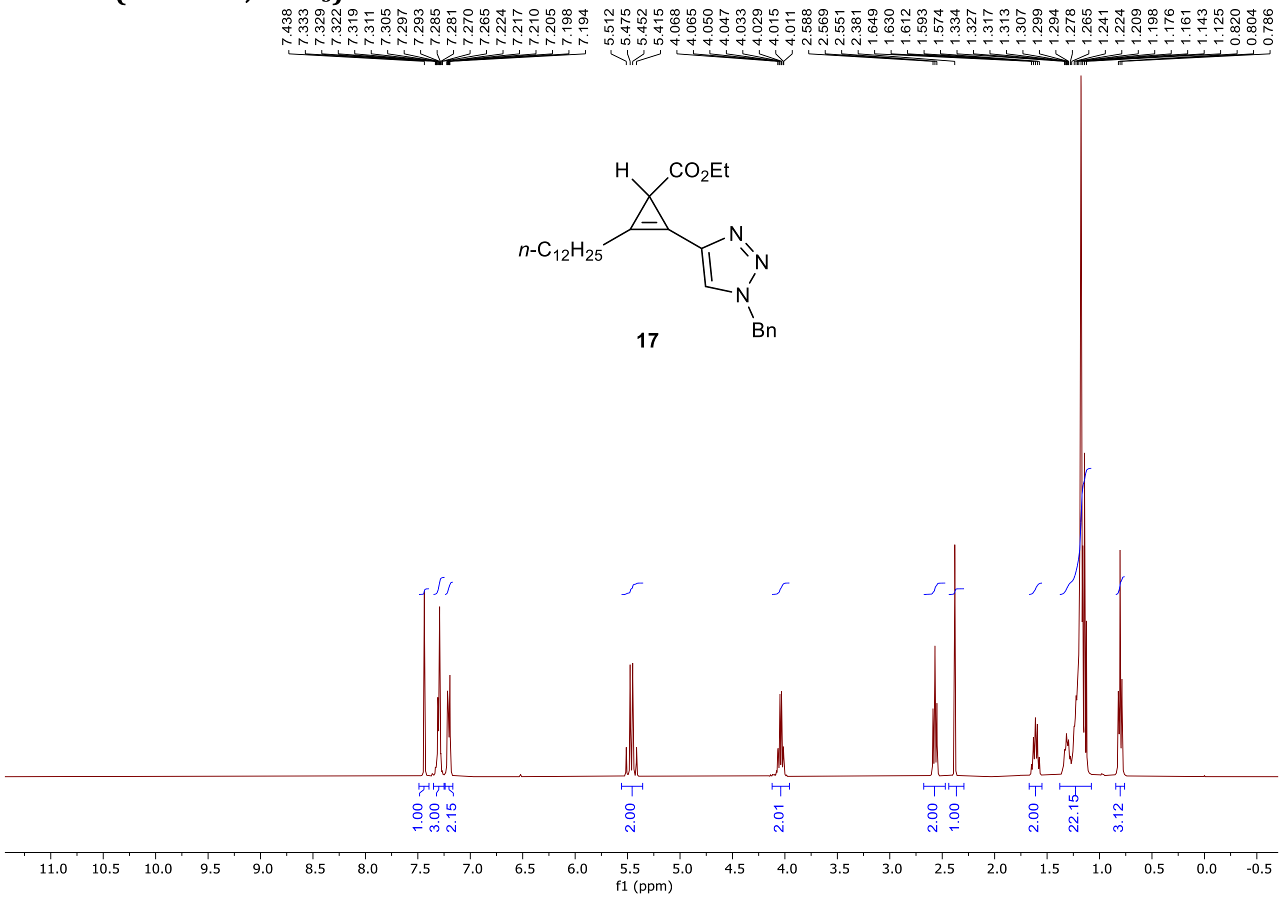

^{13}C NMR (101 MHz, $CDCl_3$) of 17

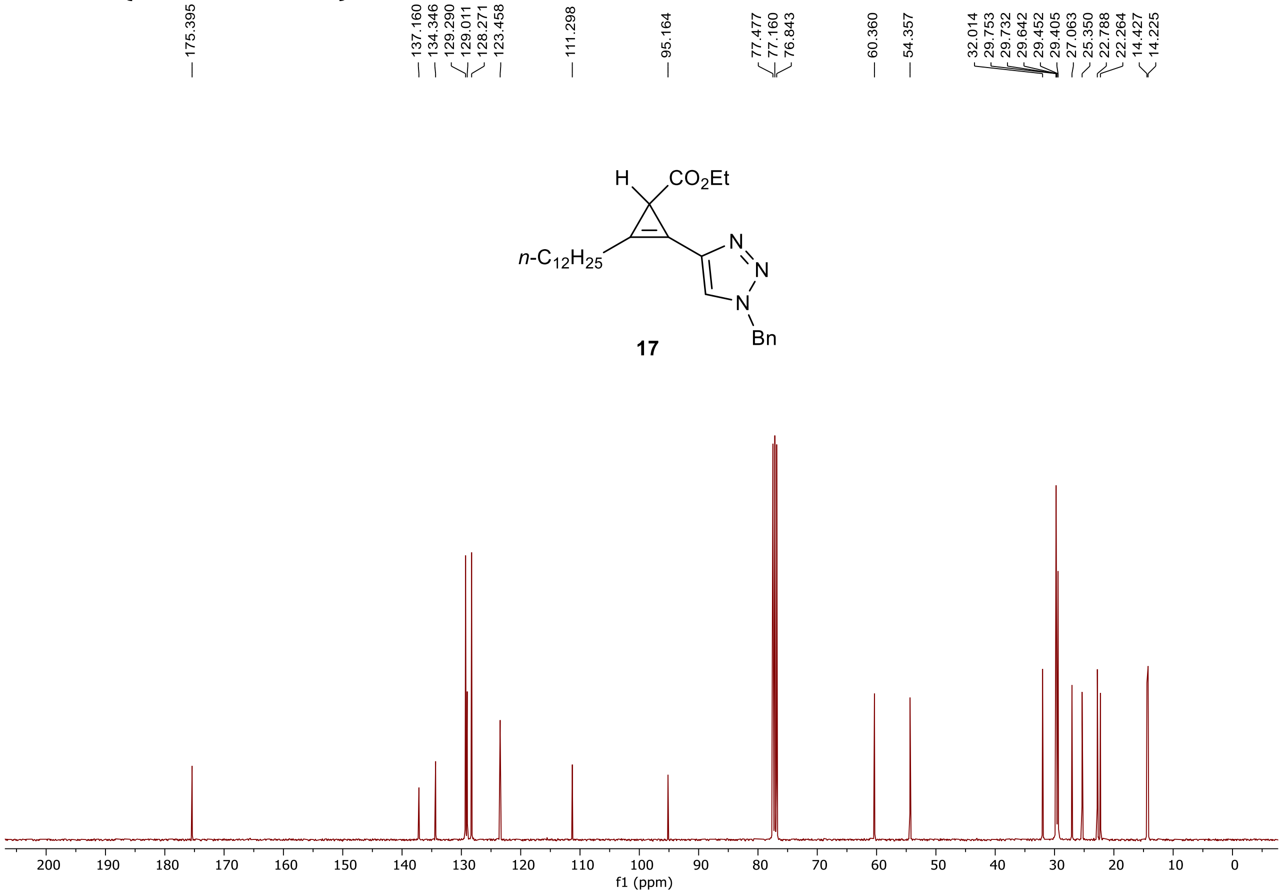

^1H NMR (400 MHz, CD_2Cl_2) of 21

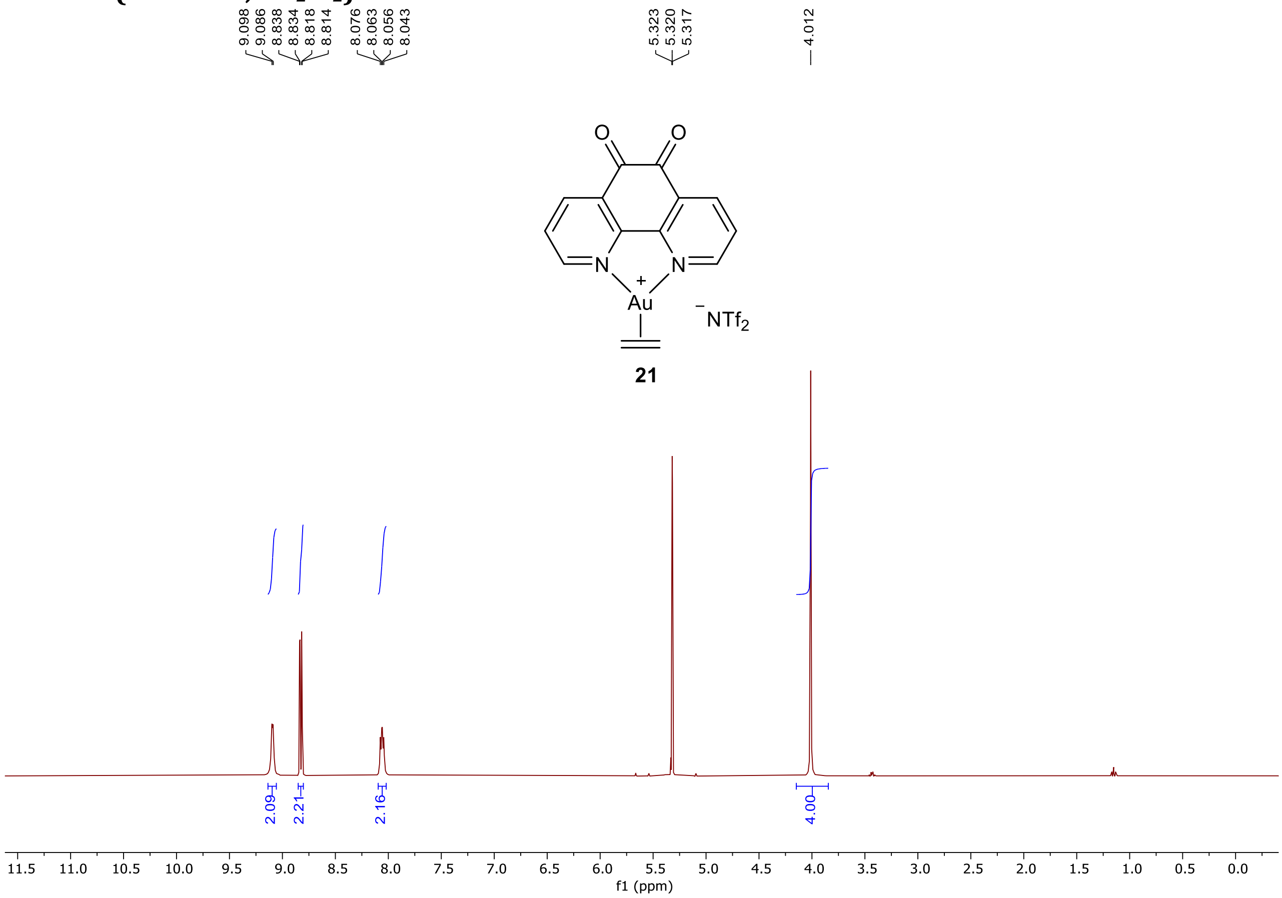

^{13}C NMR (101 MHz, CD_2Cl_2) of 21

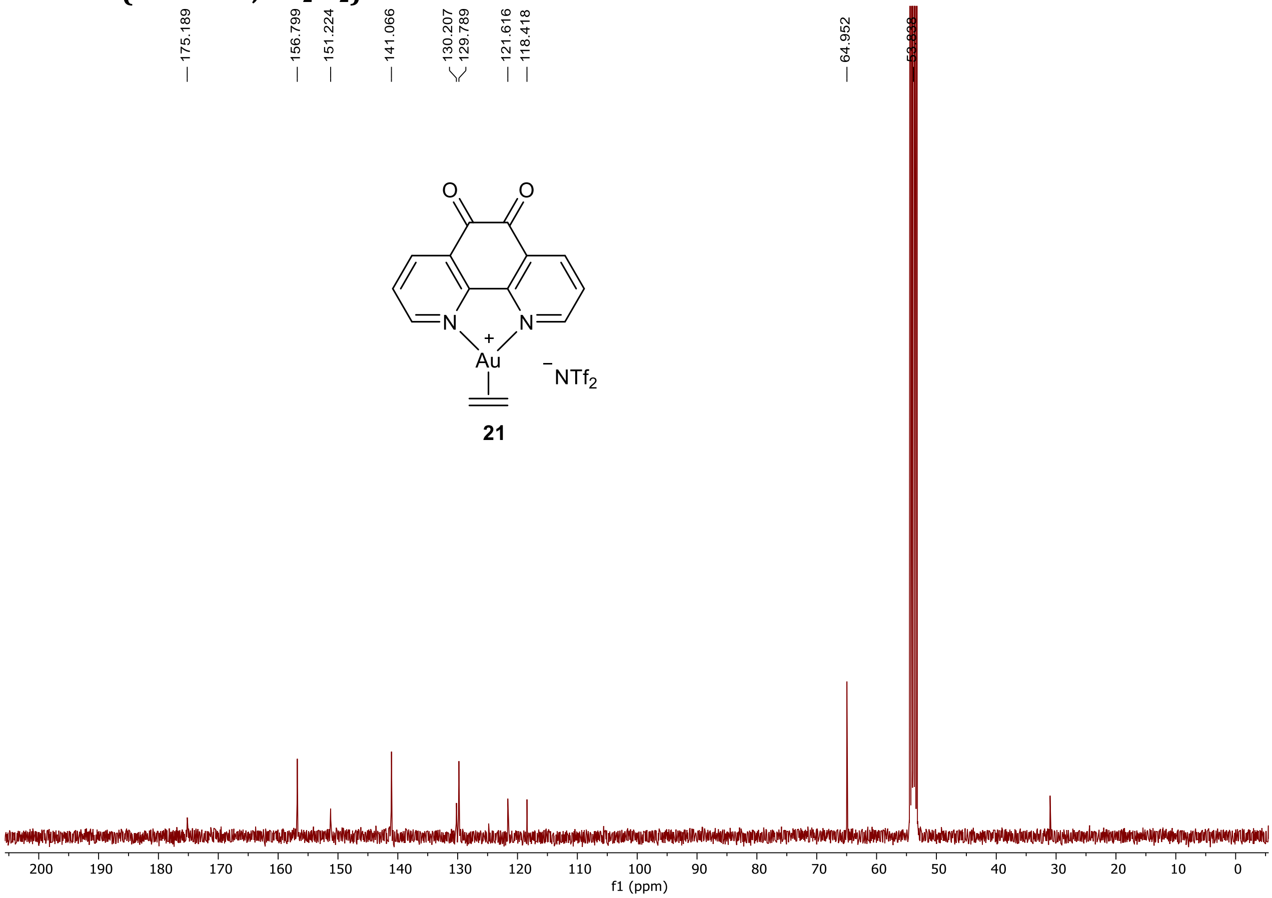

^{19}F NMR (376 MHz, CD_2Cl_2) of 21

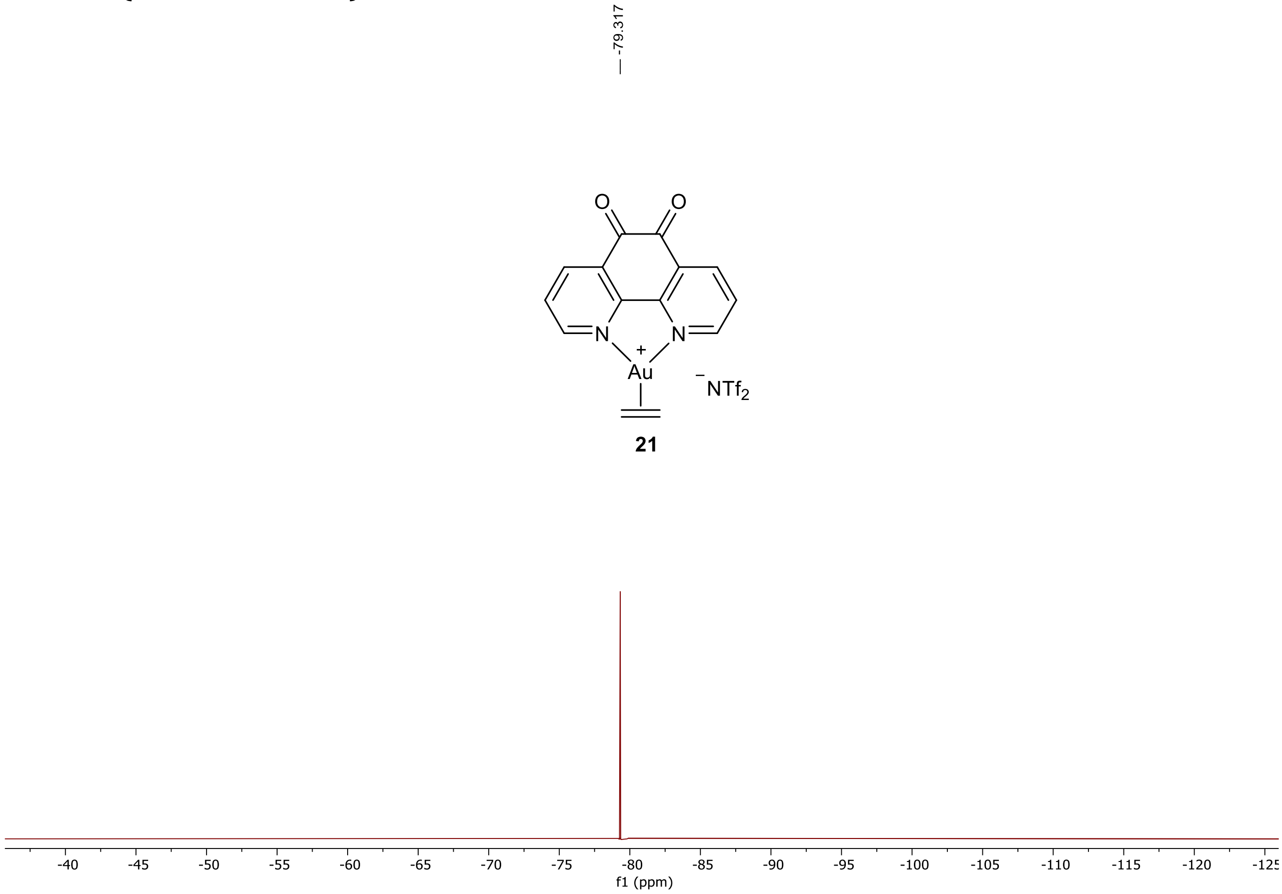

^{1}H NMR (400 MHz, CD_3CN) of 23

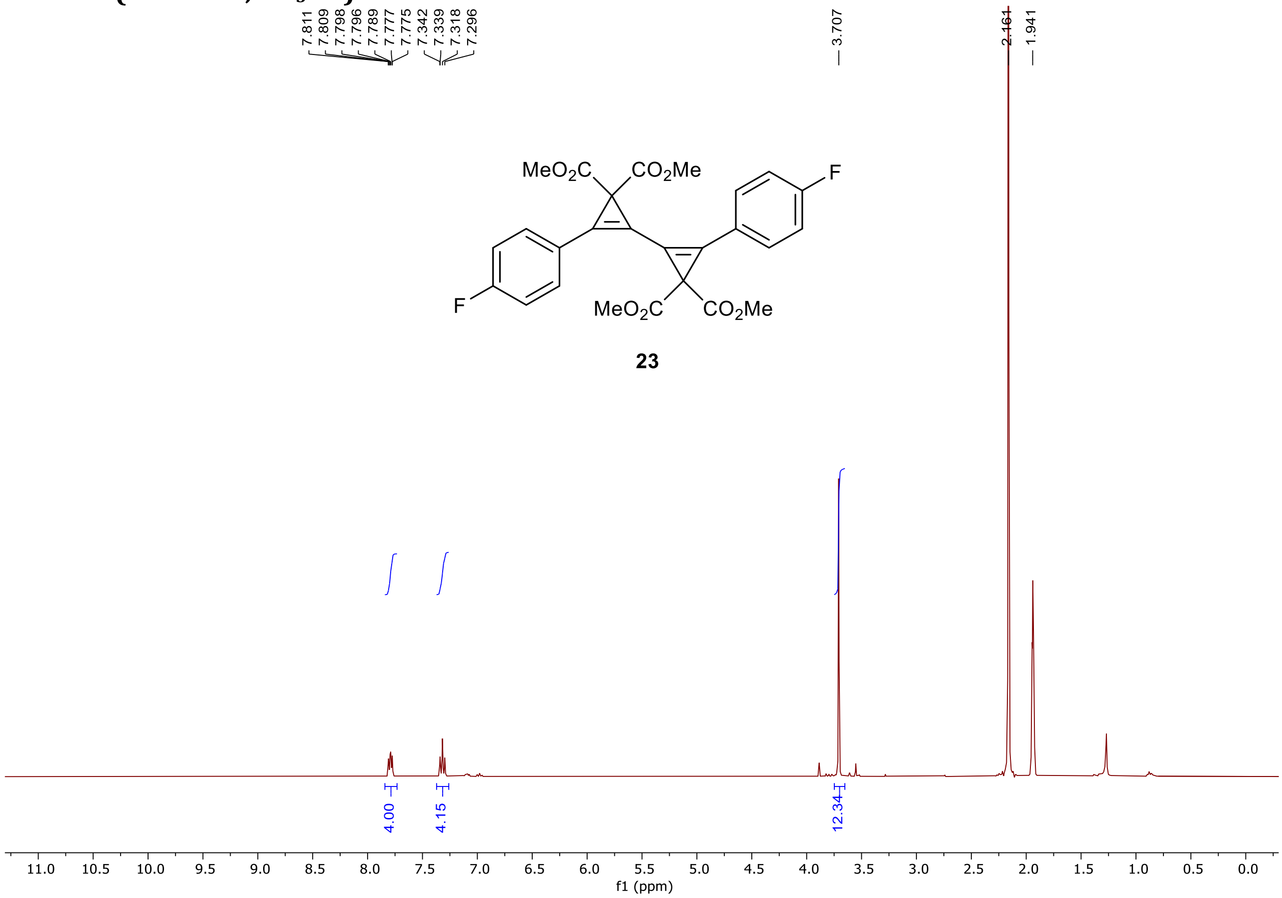

13C NMR (101 MHz, CD3CN) of 23

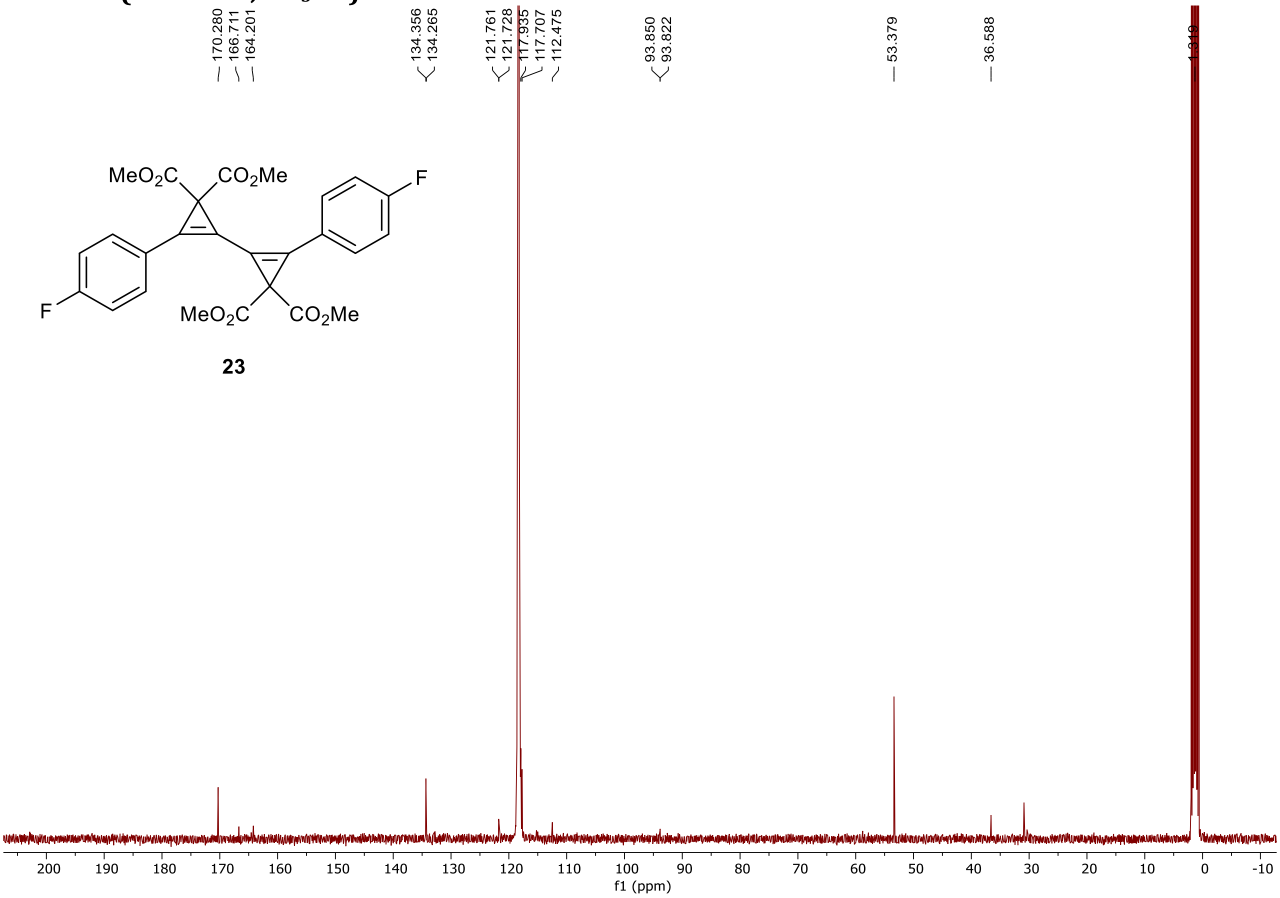

19F NMR (376 MHz, CD3CN) of 23

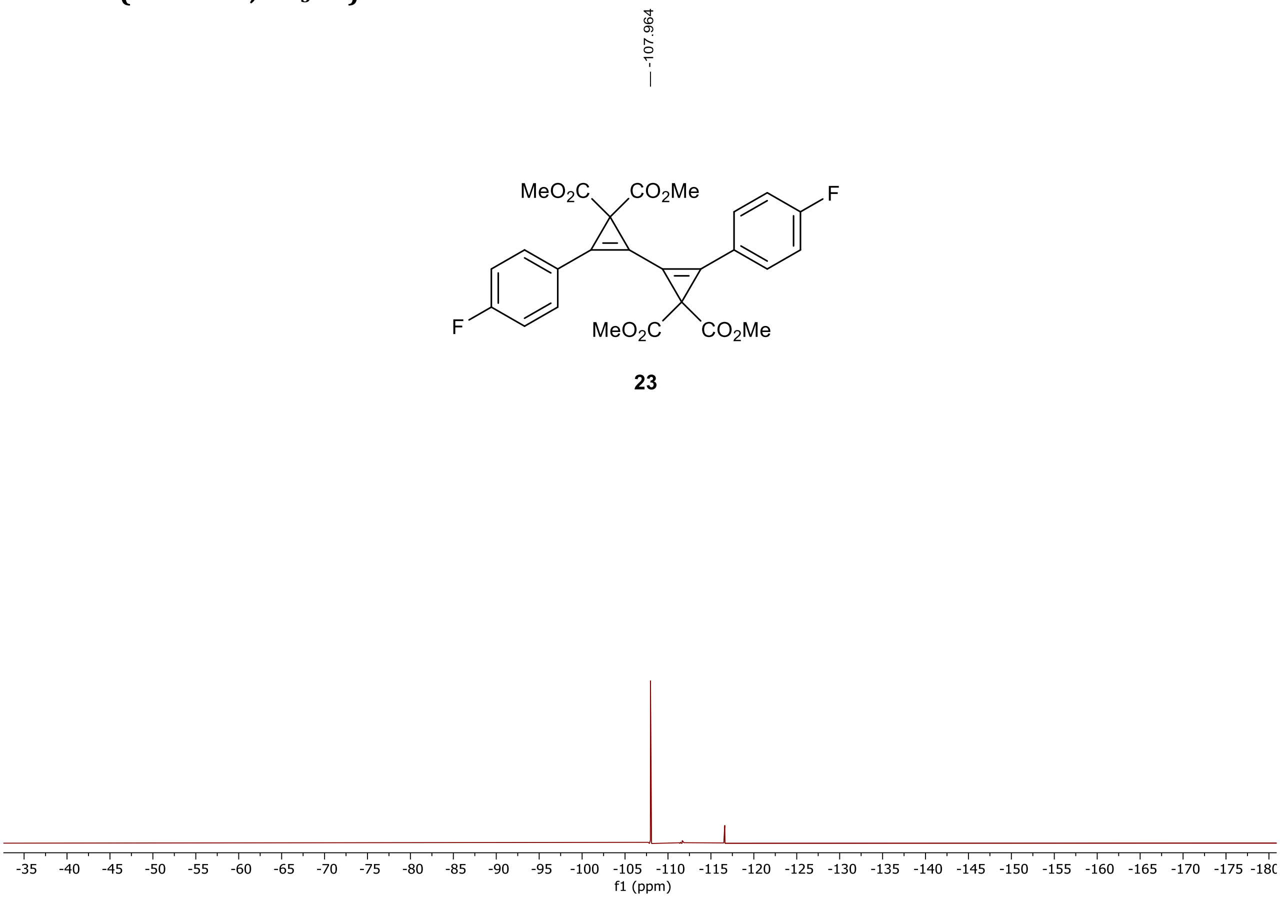

11. References

[1] Song, C., Ju, L., Wang, M., Liu, P., Zhang, Y., Wang, J. & Xu, Z. From cyclopropenes to tetrasubstituted furans: tandem isomerization/alkenylation sequence with Cu/Pd relay catalysis. *Chem. Eur. J.* **19**, 3584 – 3589 (2013).
[2] Dalling, A. G., Yamauchi, T., McCreanor, N. G., Cox, L. & Bower, J. F. Carbonylative C–C bond activation of electron-poor cyclopropanes: rhodium-catalyzed (3+1+2) cycloadditions of cyclopropylamides. *Angew. Chem. Int. Ed*. **58**, 221–225 (2019).
[3] Hari, D. P. & Waser, J. Enantioselective copper-catalyzed oxy-alkynylation of diazo compounds. *J. Am. Chem. Soc*. **139**, 8420–8423 (2017).
[4] Roy, S. R., Didier, D., Kleiner, A. & Marek, I. Diastereodivergent combined carbometalation/zinc homologation/C–C fragmentation reaction as an efficient tool to prepare acyclic allylic quaternary carbon stereocenters. *Chem. Sci.* **7**, 5989–5994 (2016).
[5] Abu-Elfotoh, A.-M., Nguyen, D. P. T., Chanthamath, S., Phomkeona, K., Shibatomi, K. & Iwasa, S. Water-soluble chiral ruthenium(II) phenyloxazoline complex: reusable and highly enantioselective catalyst for intramolecular cyclopropanation reactions. *Adv. Synth. Catal*. **354**, 3435 – 3439 (2012).
[6] Tarwade, V., Liu, X., Yan, N. & Fox, J. M. Directed carbozincation reactions of cyclopropene derivatives. *J. Am. Chem. Soc*. **131**, 5382–5383 (2009).
[7] Morandi, B. & Carreira, E. M. Rhodium-catalyzed cyclopropenation of alkynes: Synthesis of trifluoromethyl-substituted cyclopropenes. *Angew. Chem. Int. Ed.* **49**, 4294 –4296 (2010).
[8] Muriel, B. & Waser, J. Azide radical initiated ring opening of cyclopropenes leading to alkenyl nitriles and polycyclic aromatic compounds. *Angew. Chem. Int. Ed.* **60**, 4075–4079 (2021).
[9] Zhang, F. & Fox, J. M. Synthesis of cyclopropene α-amino acids via enantioselective desymmetrization. *Org. Lett.* **14**, 2965–2968 (2006).
[10] Wang, Y., Fordyce, E. A. F., Chen, F. Y. & Lam, H. W. Stereoselective synthesis of tri- and tetrasubstituted alkenes by iron-catalyzed carbometalation ring-opening reactions of cyclopropenes. *Angew. Chem. Int. Ed*. **47**, 7350–7353 (2008).
[11] Caramenti, P., Nandi, R. K. & Waser, J. Metal-free oxidative cross coupling of indoles with electron-rich (hetero)arenes. *Chem. Eur. J.* **24**, 10049-10053 (2018).
[12] Tsuzuki, S., Sakamoto, R. & Maruoka, K. Practical synthesis of α,β-alkynyl ketones by oxidative alkynylation of aldehydes with hypervalent alkynyliodine reagents. *Chem. Lett.* **49**, 633–636 (2020).
[13] Maity, A., Hyun, S.-M. & Powers, D. C. Oxidase catalysis via aerobically generated hypervalent iodine intermediates. *Nat. Chem.* **10**, 200–204 (2018).
[14] Ramirez, N. P. & Waser, J. Copper (I)-BOX catalyzed asymmetric 3-component reaction for the synthesis of trifluoromethylated propargylic ethers and anilines. *Angew. Chem. Int. Ed*. **62**, e202305776 (2023).
[15] Nguyen, T. N. & May, J. A. Branched amine synthesis via aziridine or azetidine opening with organotrifluoroborates by cooperative Brønsted/Lewis ccid catalysis: An acid-dependent divergent mechanism. *Org. Lett.* **20**, 3618–3621 (2018).
[16] Mundal, D. A., Lutz, K. E. & Thomson, R. J. A direct synthesis of allenes by a traceless Petasis reaction. *J. Am. Chem. Soc*. **134**, 5782–5785 (2012).
[17] Rzonsowska, M., Woźniak, B., Dudziec, B., Pyziak, J., Kownacki, I. & Marciniec, B. A simple catalytic route for alkynylgermanes. *Eur. J. Inorg. Chem.* **2016**, 339-346 (2016).
[18] Dahiya, A. & Schoenebeck, F. Direct C–H dehydrogenative germylation of terminal alkynes with hydrogermanes. *Org. Lett.* **24**, 2728–2732 (2022).
[19] Mandal, S., Mandal, S. & Geetharani, K. Zinc-catalysed hydroboration of terminal and internal alkynes. *Chem. Eur. J.* **14**, 4553-4556 (2019).
[20] Dasgupta, A., Stefkova, K., Babaahmadi, R., Yates, B. F., Buurma, N. J., Ariafard, A., Richards, E. & Melen, R. L. Site-selective $C_{sp}3$–C_{sp}/$C_{sp}3$–$C_{sp}2$ cross-coupling reactions using frustrated lewis pairs. *J. Am. Chem. Soc.* **143**, 4451–4464 (2021).
[21] Ishizaki, M., Zyo, M., Kasama, Y., Niimi, Y., Hoshino, O., Nishitani, K. & Hara, H. Investigation of the intermolecular Pauson-Khand reaction of various 1-alkynes with cyclic exo-methylene compounds. *Heterocycles* **60**, 2259 – 2271 (2003).
[22] Ueda, H., Yamaguchi, M., Kameya, H., Sugimoto, K. & Tokuyama, H. Autotandem catalysis: Synthesis of pyrroles by gold-catalyzed cascade reaction. *Org. Lett.* **16**, 4948–4951 (2014).
[23] Montoro-Garcia, C. Mayoral, M. J., Chamorro, R. & González-Rodríguez, D. How large can we build a cyclic assembly? Impact of ring size on chelate cooperativity in noncovalent macrocyclizations. *Angew. Chem. Int. Ed*. **56**, 15649-15653 (2017).

[24] Ming, X.-X., Wu, S., Tian, Z.-Y., Song, J.-W. & Zhang, C.-P. Pd/Cu-catalyzed vinylation of terminal alkynes with (2-bromoethyl)diphenylsulfonium triflate. *Org. Lett.* **23**, 6795–6800 (2021).
[25] Wang, Y., Wang, D., Xu, C., Wang, R., Han, J. & Feng, S. Click polymerization: Synthesis of novel σ-π conjugated organosilicon polymers. *J. Organomet. Chem.* **18**, 3000–3005 (2011).
[26] Wu, J., Watson, M. D., Tchebotareva, N., Wang, Z. & Müllen, K. Oligomers of hexa-peri-hexabenzocoronenes as "super-oligophenylenes": Synthesis, electronic properties, and self-assembly. *J. Org. Chem.* **69**, 8194-8204 (2004).
[27] Langle, S., David-Quillot, F., Balland, A., Abarbri, M. & Duchêne, A. General access to para-substituted styrenes. *J. Organomet. Chem.* **671**, 113-119 (2003).
[28] Oka, N., Yamada, T., Sajiki, H., Akai, S. & Ikawa, T. Aryl boronic esters are stable on silica gel and reactive under Suzuki–Miyaura coupling conditions. *Org. Lett.* **24**, 3510–3514 (2022).
[29] Reddyrajula, R. & Dalimba, U. The bioisosteric modification of pyrazinamide derivatives led to potent antitubercular agents: Synthesis via click approach and molecular docking of pyrazine-1,2,3-triazoles. *Bioorganic Med. Chem. Lett.* **30**, 126846 (2020).
[30] Farran, D. Slawin, A. M. Z., Kirsch, P. & O'Hagan, D. Diastereoselective synthesis of 2,3,4,5,6-pentafluoroheptanes. *J. Org. Chem.* **74**, 7168–7171 (2009).
[31] Almansa, C., Moyano, A., Pericàs, M. A. & Serratosa, F. *Synthesis* **20**, 707-709 (1988).
[32] Yamamoto, Y., Shi, Y., Masui, T., Saito, D., Inoue, T., Sato, H., Dohi, C., Muneta, E., Shang, R. & Nakamoto, M. Synthesis and characterization of hypervalent pentacoordinate carbon compounds bearing a 7-6-7-ring skeleton. *Chem. Eur. J.* **29**, e202203162 (2023).
[33] Roldan, R. J., Pajarillo, A. O., Greenberg, J. D., Karlinsey, J. E., Cafiero, M., Frawley, E. R. & Peterson, L. W. Propargylglycine-based antimicrobial compounds are targets of TolC-dependent efflux systems in Escherichia coli. *Bioorg. Med. Chem. Lett.* **30**, 126875 (2020).
[34] Sogawa, H., Shiotsuki, M. & Sanda, F. α-Propargyl amino acid-derived optically active novel substituted polyacetylenes: Synthesis, secondary structures, and responsiveness to ions. *J. Polym. Sci., Part A: Polym. Chem.* **50**, 2008–2018 (2012).
[35] Kalbarczyk, K. P. & Diver, S. T. Enyne metathesis/Brønsted acid-promoted heterocyclization. *J. Org. Chem.* **74**, 2193–2196 (2009).
[36] Brandolese, A., Monica, F. D., Pericàs, M. À. & Kleij, A. W. Catalytic ring-opening copolymerization of fatty acid epoxides: Access to functional biopolyesters. *Macromolecules* **55**, 2566–2573 (2022).
[37] Bew, S. P. & Hiatt-Gipson, G. D. Synthesis of *C*-propargylic esters of *N*-protected amino acids and peptides. *J. Org. Chem.* **75**, 3897–3899 (2010).
[38] Xu, Y., Hong, Y. J., Tantillo, D. J. & Brown, M. K. Intramolecular chirality transfer [2 + 2] cycloadditions of allenoates and alkenes. *Org. Lett.* **19**, 3703–3706 (2017).
[39] Rochet, P., Vatèle, J.-M. & Goré, J. An efficient synthesis of enantiopure 1-alkoxy-1,2-propadienes from propargyl bromide. *Synthesis* **26**, 795–799 (1994).
[40] Teske, N. S., Voigt, J. & Shastri, V. P. Clickable Degradable aliphatic polyesters via copolymerization with alkyne epoxy esters: Synthesis and postfunctionalization with organic dyes. *J. Am. Chem. Soc.* **136**, 10527–10533 (2014).
[41] Schmidt, K., Jung, M., Keilitz, R., Schnurr, B. & Gust, R. Acetylenehexacarbonyldicobalt complexes, a novel class of antitumor drugs. *Inorg. Chim. Acta* **306**, 6–16 (2000).
[42] Li, H., Guo, L., Feng, X., Huo, L., Zhu, S. & Chu, L. Sequential C–O decarboxylative vinylation/C–H arylation of cyclic oxalates via a nickel-catalyzed multicomponent radical cascade. *Chem. Sci.* **11**, 4904–4910 (2020).
[43] Zhao, X., Zhu, S., Qing, F.-L. & Chu, L. Reductive hydrobenzylation of terminal alkynes via photoredox and nickel dual catalysis. *Chem. Commun.* **57**, 9414–9417 (2021).
[44] Das, K. K., Ghosha, A. K. & Hajra, A. Late-stage ortho-C–H alkenylation of 2-arylindazoles in aqueous medium by Manganese(i)-catalysis. *RSC Adv.* **12**, 19412-19416 (2022).
[45] Bokhtia, R. M., Panda, S. S., Girgis, A. S., Samir, N., Said, M. F., Abdelnaser, A., Nasr, S., Bekheit, M. S., Dawood, A. S., Sharma, H., Wade, M., Sharma, S. K. & Ghanim, A. M. New NSAID conjugates as potent and selective COX-2 inhibitors: synthesis, molecular modeling and biological investigation. *Molecules* **28**, 1945 (2023).
[46] Li, J., Yang, F., Ma, Y.-T., & Ji, K. Gold(III)-catalyzed intermolecular oxidation-cyclization of ynones: Access to 4-substituted chroman-3-ones. *Adv. Synth. Catal.* **361**, 2148-2153 (2019).
[47] Çapcı, A., Lorion, M. M., Wang, H., Simon, N., Leidenberger, M., Borges Silva, M. C., Moreira, D. R. M., Zhu, Y., Meng, Y., Chen, J. Y., Lee, Y. M., Friedrich, O., Kappes, B., Wang, J., Ackermann, L. & Tsogoeva, S. B. Artemisinin–(iso)quinoline hybrids by C–H activation and click chemistry: combating multidrug-resistant malaria. *Angew. Chem. Int. Ed.* **58**, 13066–13079 (2019).
[48] Liu, E.-C. & Topczewski, J. J. Enantioselective copper catalyzed alkyne–azide cycloaddition by dynamic kinetic resolution. *J. Am. Chem. Soc.* **141**, 5135–5138 (2019).

[49] García, L., Sendra, J., Miralles, N., Reyes, E., Carbó, J. J., Vicario, J. L. & Fernández, E. Transition-metal-free stereoselective borylation of allenamides. *Chem. Eur. J.* **24**, 14059–14063 (2018).
[50] Ballesteros, A., Morán-Poladura, P. & González, J. M. Gold(i) operational in synergistic catalysis for the intermolecular α-addition reaction of aldehydes across allenamides. *Chem. Commun.* **52**, 2905-2908 (2016).
[51] Pisella, G., Gagnebin, A. & Waser, J. Copper-catalyzed oxyvinylation of diazo compounds. *Org. Lett.* **22**, 3884–3889 (2020).
[52] Yang, Y., Antoni, P., Zimmer, M., Sekine, K., Mulks, F., Hu, L., Zhang, L., Rudolph, M., Rominger, F. & Hashmi, A. S. K. Dual gold/silver catalysis involving alkynylgold(III) intermediates formed by oxidative addition and C,H-activation for the direct alkynylation of cyclopropenes. *Angew. Chem. Int. Ed.* **58**, 5129–5133 (2019).
[53] Banerjee, S., Senthilkumar, B. & Patil, N. T. Gold-catalyzed 1,2-oxyalkynylation of *N*-allenamides with ethylnylbenziodoxolones. *Org. Lett*. **21**, 180–184 (2019).
[54] Li, C., Zhang, H., Feng, J., Zhang, Y. & Wang, J. Rh(I)-catalyzed carbonylative carbocyclization of tethered ene– and yne–cyclopropenes. *Org. Lett.* **12**, 3082–3085 (2010).
[55] Cohen, Y., Augustin, A. U., Levy, L., Jones, P. G., Werz, D. B. & Marek, I. Regio- and Diastereoselective copper-catalyzed carbomagnesiation for the synthesis of penta- and hexa-substituted cyclopropanes. *Angew. Chem. Int. Ed*. **60**, 11804–11808 (2021).
[56] Lou, Y., Horikawa, M., Kloster, R. A., Hawryluk, N. A. & Corey, E. J. A new chiral Rh(II) catalyst for enantioselective [2+1]-cycloaddition. Mechanistic implications and applications. *J. Am. Chem. Soc.* **126**, 8916–8918 (2004).
[57] Liao, L.-a., Zhang, F., Yan, N., Golen, J. & Fox, J. M. An efficient and general method for resolving cyclopropene carboxylic acids. *Tetrahedron* **60**, 1803–1816 (2004).
[58] Zhang, Z.-Q., Zheng, M.-M., Xue, X.-S., Marek, I., Zhang, F.-G. & Ma, J.-A. Catalytic enantioselective cyclopropenation of internal alkynes: access to difluoromethylated three-membered carbocycles. *Angew. Chem. Int. Ed*. **58**, 18191 –18196 (2019).
[59] Liu, Y.-L., Zhu, X.-L., Huang, Y., Qing, F.-L. & Xu, X.-H. Radical coupling of arylthiodifluoroacetic acids and ethynylbenziodoxolone (EBX) reagents to access arylthiodifluoromethylated alkynes. *J. Fluorine Chem*. **242**, 109715 (2021).
[60] Li, X., Xie, X., Sun, N. & Liu, Y. Gold-catalyzed Cadiot–Chodkiewicz-type cross-coupling of terminal alkynes with alkynyl hypervalent iodine reagents: Highly selective synthesis of unsymmetrical 1,3-diynes. *Angew. Chem. Int. Ed.* **56**, 6994 –6998 (2017).
[61] Shao, C., Wang, X., Xu, J., Zhao, J., Zhang, Q. & Hu, Y. Carboxylic acid-promoted copper(I)-catalyzed azide-alkyne cycloaddition. *J. Org. Chem*. **75**, 7002–7005 (2010).
[62] Theulier, C. A., García-Rodeja, Y., Saffon-Merceron, N., Miqueu, K., Bouhadir, G. & Bourissou, D. 1,1-Phosphaboration of CRC and CQC bonds at gold. *Chem. Commun*. **57**, 347–350 (2021).
[63] Harper, M. J., Arthur, C. J., Crosby, J., Emmett, E. J., Falconer, R. A., Fensham-Smith, A. J., Gates, P. G., Leman, T., McGrady, J. E., Bower, J. F. & Russell, C. A. Oxidative addition, transmetalation, and reductive elimination at a 2,2-bipyridyl-ligated gold center. *J. Am. Chem. Soc.* **140**, 4440–4445 (2018).
[64] Dias, H. V. R., Fianchini, M., Cundari, T. R. & Campana, C. F. Synthesis and characterization of the gold(I) tris(ethylene) complex $[Au(C_2H_4)_3][SbF_6]$. *Angew. Chem. Int. Ed*. **47**, 556–559 (2008).
[65] Perdew, J. P., Burke, K. & Ernzerhof, M. Generalized gradient approximation made simple. *Phys. Rev. Lett.* **77**, 3865–3868 (1996).
[66] Adamo, C. & Barone, V. Toward reliable density functional methods without adjustable parameters: The PBE0 model. *J. Chem. Phys.* **110**, 6158–6170 (1998).
[67] Grimme, S., Ehrlich, S. & Goerigk, L. Effect of the damping function in dispersion corrected density functional theory. *J. Comput. Chem.* **32**, 1456–1465 (2011).
[68] Becke, A. D. & Johnson, E. R. A density-functional model of the dispersion interaction. *J. Chem. Phys.* **123**, 154101 (2005).
[69] Johnson, E. R. & Becke, A. D. A post-Hartree–Fock model of intermolecular interactions. *J. Chem. Phys.* **123**, 024101 (2005).
[70] Johnson, E. R. & Becke, A. D. A post-Hartree-Fock model of intermolecular interactions: Inclusion of higher-order corrections. *J. Chem. Phys.* **124**, 174104 (2006).
[71] Weigend, F. & Ahlrichs, R. Balanced Basis Sets of Split Valence, Triple Zeta Valence and Quadruple Zeta Valence Quality for H to Rn: Design and Assessment of Accuracy. *Phys. Chem. Chem. Phys.* **7**, 3297–3305 (2005).
[72] Marenich, A. V., Cramer, C. J. & Truhlar, D. G. Universal Solvation Model Based on Solute Electron Density and on a Continuum Model of the Solvent Defined by the Bulk Dielectric Constant and Atomic Surface Tensions. *J. Phys. Chem. B* **113**, 6378–6396 (2009).

[73] Gaussian 16, Revision C.01, Frisch, M. J.; Trucks, G. W.; Schlegel, H. B.; Scuseria, G. E.; Robb, M. A.; Cheeseman, J. R.; Scalmani, G.; Barone, V.; Petersson, G. A.; Nakatsuji, H.; Li, X.; Caricato, M.; Marenich, A. V.; Bloino, J.; Janesko, B. G.; Gomperts, R.; Mennucci, B.; Hratchian, H. P.; Ortiz, J. V.; Izmaylov, A. F.; Sonnenberg, J. L.; Williams-Young, D.; Ding, F.; Lipparini, F.; Egidi, F.; Goings, J.; Peng, B.; Petrone, A.; Henderson, T.; Ranasinghe, D.; Zakrzewski, V. G.; Gao, J.; Rega, N.; Zheng, G.; Liang, W.; Hada, M.; Ehara, M.; Toyota, K.; Fukuda, R.; Hasegawa, J.; Ishida, M.; Nakajima, T.; Honda, Y.; Kitao, O.; Nakai, H.; Vreven, T.; Throssell, K.; Montgomery, J. A., Jr.; Peralta, J. E.; Ogliaro, F.; Bearpark, M. J.; Heyd, J. J.; Brothers, E. N.; Kudin, K. N.; Staroverov, V. N.; Keith, T. A.; Kobayashi, R.; Normand, J.; Raghavachari, K.; Rendell, A. P.; Burant, J. C.; Iyengar, S. S.; Tomasi, J.; Cossi, M.; Millam, J. M.; Klene, M.; Adamo, C.; Cammi, R.; Ochterski, J. W.; Martin, R. L.; Morokuma, K.; Farkas, O.; Foresman, J. B.; Fox, D. J. Gaussian, Inc., Wallingford CT, 2016.
[74] Becke, A. D. Density-functional thermochemistry. III. The role of exact exchange. *J. Chem. Phys.* **98**, 5648–5652 (1993).
[75] Perdew, J. P. Electronic Structure of Solids. in *Electronic Structure of Solids* 11 (Akademie Verlag, 1991).
[76] Perdew, J. P., Burke, K. & Wang, Y. Generalized gradient approximation for the exchange-correlation hole of a many-electron system. *Phys. Rev. B* **54**, 16533–16539 (1996).
[77] Martin, R. L., Hay, P. J. & Pratt, L. R. Hydrolysis of Ferric Ion in Water and Conformational Equilibrium. *J. Phys. Chem. A* **102**, 3565–3573 (1998).
